# Molecular Typing of ST239-MRSA-III From Diverse Geographic Locations and the Evolution of the SCC*mec* III Element During Its Intercontinental Spread

**DOI:** 10.3389/fmicb.2018.01436

**Published:** 2018-07-06

**Authors:** Stefan Monecke, Peter Slickers, Darius Gawlik, Elke Müller, Annett Reissig, Antje Ruppelt-Lorz, Patrick E. Akpaka, Dirk Bandt, Michele Bes, Samar S. Boswihi, David C. Coleman, Geoffrey W. Coombs, Olivia S. Dorneanu, Vladimir V. Gostev, Margaret Ip, Bushra Jamil, Lutz Jatzwauk, Marco Narvaez, Rashida Roberts, Abiola Senok, Anna C. Shore, Sergey V. Sidorenko, Leila Skakni, Ali M. Somily, Muhammad Ali Syed, Alexander Thürmer, Edet E. Udo, Teodora Vremerǎ, Jeannete Zurita, Ralf Ehricht

**Affiliations:** ^1^Abbott (Alere Technologies GmbH), Jena, Germany; ^2^InfectoGnostics Research Campus Jena, Jena, Germany; ^3^Medical Faculty “Carl Gustav Carus”, Institute for Medical Microbiology and Hygiene, Technische Universität Dresden, Dresden, Germany; ^4^Department of Paraclinical Sciences, The University of the West Indies, St. Augustine, Trinidad and Tobago; ^5^Instituts für Labordiagnostik, Mikrobiologie und Krankenhaushygiene, Oberlausitz-Kliniken, Bautzen, Germany; ^6^Centre National de Référence des Staphylocoques, Institut des Agents Infectieux, Hospices Civils de Lyon, Lyon, France; ^7^Microbiology Department, Faculty of Medicine, Kuwait University, Kuwait City, Kuwait; ^8^Microbiology Research Unit, Dublin Dental University Hospital, University of Dublin, Trinity College Dublin, Dublin, Ireland; ^9^School of Veterinary and Life Sciences, Murdoch University, Murdoch, WA, Australia; ^10^Microbiology Unit, Department of Preventive and Interdisciplinary Medicine, University of Medicine & Pharmacy “Grigore T Popa”, Iaşi, Romania; ^11^Pediatric Research and Clinical Center for Infectious Diseases, Saint Petersburg, Russia; ^12^Department of Microbiology, Faculty of Medicine, The Chinese University of Hong Kong, Shatin, Hong Kong; ^13^Department of Biosciences, COMSATS Institute of Information Technology, Islamabad, Pakistan; ^14^Department of Biogenetics, National University of Medical Sciences, Rawalpindi, Pakistan; ^15^Department of Hospital Infection Control, Dresden University Hospital, Dresden, Germany; ^16^Department of Basic Medical Sciences, College of Medicine, Mohammed Bin Rashid University of Medicine and Health Sciences, Dubai, United Arab Emirates; ^17^Molecular Pathology Laboratory, King Fahad Medical City, Riyadh, Saudi Arabia; ^18^Department of Pathology and Laboratory Medicine, College of Medicine, King Saud University and King Saud University Medical City, Riyadh, Saudi Arabia; ^19^Department of Microbiology, University of Haripur, Haripur, Pakistan; ^20^Facultad de Medicina, Pontificia Universidad Catolica del Ecuador, Quito, Ecuador; ^21^Zurita & Zurita Laboratorios, Unidad de Investigaciones en Biomedicina, Quito, Ecuador

**Keywords:** *Staphylococcus aureus*, MRSA, ST239-MRSA-III, SCCmec element, molecular epidemiology

## Abstract

ST239-MRSA-III is probably the oldest truly pandemic MRSA strain, circulating in many countries since the 1970s. It is still frequently isolated in some parts of the world although it has been replaced by other MRSA strains in, e.g., most of Europe. Previous genotyping work (Harris et al., 2010; Castillo-Ramírez et al., 2012) suggested a split in geographically defined clades. In the present study, a collection of 184 ST239-MRSA-III isolates, mainly from countries not covered by the previous studies were characterized using two DNA microarrays (i) targeting an extensive range of typing markers, virulence and resistance genes and (ii) a SCC*mec* subtyping array. Thirty additional isolates underwent whole-genome sequencing (WGS) and, together with published WGS data for 215 ST239-MRSA-III isolates, were analyzed using *in-silico* analysis for comparison with the microarray data and with special regard to variation within SCC*mec* elements. This permitted the assignment of isolates and sequences to 39 different SCC*mec* III subtypes, and to three major and several minor clades. One clade, characterized by the integration of a transposon into *nsaB* and by the loss of *fnbB* and *splE* was detected among isolates from Turkey, Romania and other Eastern European countries, Russia, Pakistan, and (mainly Northern) China. Another clade, harboring *sasX/sesI* is widespread in South-East Asia including China/Hong Kong, and surprisingly also in Trinidad & Tobago. A third, related, but *sasX/sesI*-negative clade occurs not only in Latin America but also in Russia and in the Middle East from where it apparently originated and from where it also was transferred to Ireland. Minor clades exist or existed in Western Europe and Greece, in Portugal, in Australia and New Zealand as well as in the Middle East. Isolates from countries where this strain is not epidemic (such as Germany) frequently are associated with foreign travel and/or hospitalization abroad. The wide dissemination of this strain and the fact that it was able to cause a hospital-borne pandemic that lasted nearly 50 years emphasizes the need for stringent infection prevention and control and admission screening.

## Introduction

*Staphylococcus aureus* is a bacterial species that colonizes the skin and mucous membranes of a high percentage of the human population (van Belkum et al., 2009) and several animal species. It can cause localized infections, such as skin and soft tissue infections (SSTIs), bone, joint and implant infections or pneumonia as well as sepsis and toxicoses including toxic shock syndrome. Resistance toward antibiotics in *S. aureus* is a highly relevant issue. Methicillin resistance and resistance to most beta-lactams is due to the production of modified penicillin-binding proteins encoded by *mec* genes. The *mecA/mecC* genes are located on large, complex and potentially mobile staphylococcal cassette chromosome (SCC*mec*) elements (while *mecB* was observed on a plasmid; Becker et al., 2018). SCC*mec* elements additionally encode regulatory elements and, variably, genes encoding resistance to other antimicrobials, such as aminoglycosides, macrolides, tetracyclines, fusidic acid and to heavy metals (Oliveira et al., 2000; Ito et al., 2001). The comparatively older SCC*mec* types I, II, and III are typically restricted to MRSA strains involved in healthcare-associated infections (HCA-MRSA).

The HCA-MRSA, sequence type (ST) 239 MRSA, as designated by multilocus sequence typing (MLST), is of special interest. As ST240 and ST241 are single locus variants of ST239 (which differ only by mutations in MLST marker genes *pta* or *yqil*), these STs are here discussed together as clonal complex (CC) 239. CC239 harboring SCC*mec* type III have been designated various names in different geographic regions including “Wiener Epidemiestamm” (Vienna Epidemic Strain), the Hungarian Clone, UK-EMRSA−1,−4,−7,−9, or−11, Irish Phenotype III, Irish AR01,−09,−44, and−23, the Brazilian Clone, Australian Epidemic MRSA−2 and−3 as well as Canadian MRSA−3 or−6. CC239-MRSA-III is probably the oldest truly pandemic MRSA strain. In contrast to other early MRSA strains, it is still common and widespread, at least in some parts of the world.

CC239-MRSA-III has been reported in many European countries including the UK (Edgeworth et al., 2007), Ireland (Humphreys et al., 1990; Shore et al., 2005), Spain (Cuevas et al., 2007), Portugal (Smyth et al., 2010), Italy (Campanile et al., 2009), Malta (Scicluna et al., 2010), Croatia (Budimir et al., 2009), Germany (Witte et al., 1997; Albrecht et al., 2011), Austria (Krziwanek et al., 2008), the Czech Republic (Melter et al., 2003), Hungary (Conceicao et al., 2007), and Greece (Aires de Sousa et al., 2003a). It is also frequently observed in Romania (Cirlan et al., 2005; Chen et al., 2010; Monecke et al., 2014a) and Russia (Afanas'ev et al., 2010; Baranovich et al., 2010; Yamamoto et al., 2012; Gostev et al., 2017). CC239-MRSA-III is common to abundant in Mediterranean and Middle Eastern countries such as Greece (Aires de Sousa et al., 2003a), Turkey (Alp et al., 2009; Tekeli et al., 2016), Egypt (El-baz et al., 2017), Morocco, Tunisia, Algeria (Abdulgader et al., 2015), Iran (Fatholahzadeh et al., 2009), Saudi Arabia (Cirlan et al., 2005; Monecke et al., 2012c; Senok et al., 2016), Abu Dhabi (Weber et al., 2010), and Kuwait (Boswihi et al., 2016). The strain's presence has been reported in China (Chen et al., 2014a,b) including Hong Kong (Ip et al., 2005), Taiwan (Aires de Sousa et al., 2003b; Takano et al., 2007), Singapore (Hsu et al., 2007), Malaysia (Ghaznavi-Rad et al., 2010), Mongolia (Orth et al., 2006), Pakistan (Shabir et al., 2010; Zafar et al., 2011; Arfat, 2013; Jamil et al., 2017), India (D'Souza et al., 2010; Neetu and Murugan, 2016), South Korea (Cha et al., 2005; Peck et al., 2009), Laos (Yeap et al., 2017), and Thailand (Smyth et al., 2010). There are reports from several African countries including Ghana, Kenya, Niger, Nigeria, Senegal, and South Africa (Jansen van Rensburg et al., 2011; Abdulgader et al., 2015). In Eastern Australia and New Zealand it was a common cause of HCA infection, and it has been reported in association with large MRSA outbreaks (Coombs et al., 2004; Howden et al., 2010). In the western hemisphere, CC239-MRSA-III has been reported from the United States (Schaefler et al., 1981), Brazil (Vivoni et al., 2006), Paraguay (Mayor et al., 2007), Ecuador (Zurita et al., 2016), and Trinidad & Tobago where it recently was still the predominant MRSA strain (Akpaka et al., 2007; Monecke et al., 2012b, 2014b).

In terms of genetic structure, ST239 is an interesting clone, with six of the seven MLST housekeeping genes being identical to ST8 (Robinson and Enright, 2004a,b). However ST239 as well as ST240 and ST241 harbor a very different *arcC*-allele (*arcC*-2, rather than *arcC*-3). The difference in the *arcC*-allele and the presence of some other features that differentiate ST239 from canonical ST8 (including the presence of the collagen adhesion gene *cna*, capsule type 5 and RIDOM *spa* types t030 or t037), indicate the integration of a large fragment of genomic DNA of clonal complex (CC) 30 origin into a CC8 chromosome (Robinson and Enright, 2004b; Holden et al., 2009). The fragment consists of ~635,000 base pairs, which is ~20% of a *S. aureus* genome. The mechanism of integration is yet not known.

Another interesting recent observation has been the discovery of *sasX/sesI* in CC239-MRSA, a virulence factor thought to have a key role in nasal colonization, pathogenesis of lung disease, and abscess formation (Li et al., 2012). The *sasX* gene is located on a 127 kb lysogenic prophage phiSPbeta (Li et al., 2012) and it encodes the surface-anchored protein X, an LPxTG motif surface-anchored protein, and does not have orthologues in any of the other sequenced *S. aureus* genomes. A highly similar gene, *sesI*, is present in the *S. epidermidis* phiSPbeta region and has also been identified in other coagulase-negative staphylococci such as *S. capitis* (GenBank JGYJ) and *S. cohnii* (GenBank LATU and LATV).

With the rise of Next Generation Sequencing (NGS) technologies, Harris and Castillo-Ramírez (Harris et al., 2010; Castillo-Ramírez et al., 2012) sequenced a large collection of CC239-MRSA-III from very diverse geographic origins. They suggested a phylogenetic framework in which isolates of CC239-MRSA-III clustered in several major “clades” and a couple of isolated branches. The clades are largely associated with geographic background and thus were referred to as “European”, “Latin American”, “Turkish”, and “Asian” clades.

In the present study, a collection of CC239-MRSA-III isolates was characterized, primarily using previously published DNA microarray technology targeting typing markers, virulence and resistance genes and SCC*mec* subtypes (Monecke et al., 2008b, 2011, 2016). Published whole-genome sequence data, in particular those by Harris and Castillo-Ramírez (Harris et al., 2010; Castillo-Ramírez et al., 2012), were re-analyzed with regard to the presence or absence of the marker genes as used experimentally for array hybridization and with regard to variation within SCC*mec* elements. A comparison between our strain collection, mainly from countries that were not covered by the Harris and Castillo-Ramírez' work, to published genome sequences was then performed in order to see if and how they fit into their proposed phylogenetic framework and to look for epidemiological connections and suitable marker genes.

## Materials and methods

### Isolates

In total, 214 clinical or screening isolates were included in the present study. These isolates originated from hospitals in Ireland, Germany, Romania, Kuwait, Saudi Arabia, Russia, Pakistan, China/Hong Kong, Australia, Trinidad&Tobago, and Ecuador as well as some reference strains (see Supplemental Table 1 and below). Some of the isolates were a convenience sample from previous studies (Monecke et al., 2008a, 2011, 2012b,c, 2014a,b, 2016; Albrecht et al., 2011; Boswihi et al., 2016; Senok et al., 2016; Zurita et al., 2016; Gostev et al., 2017; Jamil et al., 2017). Others came from ongoing routine diagnostics, outbreak investigations or typing tasks performed by the authors and have not been published previously. Only one isolate per patient was included. Isolates were stored frozen using cryobank tubes (Microbank, Pro-Lab Diagnostics, Richmond Hill, Canada) at −80°C. Isolates were routinely cultured on Columbia blood agar plates, and DNA preparation was performed as previously described (Monecke et al., 2008b, 2011).

### DNA microarrays for SCC*mec* typing and subtyping

Two microarrays were used in the study, and were applied to 184 of the isolates investigated herein (another 30 were directly subjected to sequencing; see below). Both arrays have previously been described including probe and primer sequences, details of DNA extraction, labeling, amplification, hybridization protocols as well as data analysis and interpretation. The first microarray (Monecke et al., 2008b, 2011) detects genes associated with antibiotic resistance and virulence as well as a multitude of genes that can be used for typing purposes, such as genes related to *agr* or capsule types, *set/ssl* genes and genes coding for adhesion factors. In addition to the detection of individual genes, the array also allows the assignment of isolates to MLST CCs, to known epidemic strains and to SCC*mec* types. The second microarray (Monecke et al., 2016) was designed to subtype SCC*mec* elements. It also includes probes for *sasX/sesI* and for heavy metal resistance genes. Furthermore, it included a set of probes termed “SCCterm” followed by a number (see Table 1) which were designed to recognize intergenic regions alternative to *dcs*, between *orfX* and the first codon on the SCC*mec* element.

**Table 1:** SCC*mec*-associated genes and other relevant markers that were targeted by probes used for this study.

**Gene/Marker**	**Gene product/function**	**Comments**	**Reference sequence**	**Reference for probes and primers**
*aacA-aphD*	Bifunctional enzyme Aac/Aph, gentamicin and tobramycin resistance	–	GU565967.1: 24980…26419	Monecke et al., 2008b, 2011
*aadD*	Aminoglycoside adenyltransferase, tobramycin resistance	–	BA000017.4: 40819…41580	Monecke et al., 2008b, 2011
*aadE*	Streptomycin aminoglycoside 6-adenyltransferase	Part of a gene cluster comprising of *aadE, sat, aphA3*	AB300568.1: 1…909	Probe: AB300568.1 [643:669] Primer: AB300568.1 [714:733:r]
*ant9*	Streptomycin 3′-adenylyltransferase	–	AJ810120.1: 14339…15121	Probe: AJ810120.1 [14651:14678] Primer: AJ810120.1 [14681:14700:r]
*aphA3*	3′5′-aminoglycoside phosphotransferase, neo- and kanamycin resistance	Part of a gene cluster comprising of *sat, aphA3* and variably *aadE*	U51474.1: 957…1751	Monecke et al., 2008b, 2011
*arcA*-SCC	Arginine deiminase	Part of ACME I and ACME II clusters, that occurs alone or in combination with SCC*mec* elements	CP000255.1: 73113…74348	Monecke et al., 2008b, 2011
*arcB*-SCC	Ornithine carbamoyltransferase	Part of ACME I and ACME II clusters, that occurs alone or in combination with SCC*mec* elements	CP000255.1: 69839…70837	Monecke et al., 2008b, 2011
*arcC*-SCC	Carbamate kinase	Part of ACME I and ACME II clusters, that occurs alone or in combination with SCC*mec* elements	CP000255.1: 68890…69819	Monecke et al., 2008b, 2011
*arcD*-SCC	Arginine/ornithine antiporter	Part of ACME I and ACME II clusters, that occurs alone or in combination with SCC*mec* elements	CP000255.1: 71606…73027	Monecke et al., 2008b, 2011
*arsC*-SCC	Arsenate reductase	Probe was designed for SCC-borne allele(s) of that gene, as for instance in JCSC6943, JCSC6945	AB505628.1: 31467…31868	Monecke et al., 2016
*blaZ*	Penicillinase	Plasmid-borne penicillinase. This does NOT include the allele from SCC*mec* XI which is of no relevance for the present study.	M15526.1: 140…985	Monecke et al., 2008b, 2011
*ble*	Bleomycin resistance protein	–	AF181950.1: 4163…4567	Probe: AF181950.1 [4246:4270] Primer: AF181950.1 [4276:4296:r]
*cadD*_(R35)_	Cadmium transport protein D	Probe was designed for SCC-borne allele(s) of that gene, as for instance in strain R35, GenBank L10909.1 or strain 85/2082, GenBank AB037671.1.	L10909.1: 5577…6194	Monecke et al., 2016
*cadX*	Putative regulator of cadmium efflux	Can be part of SCC*mec* elements (as in JCSC6943; GenBank AB505628.1) but Table 3 refers to the plasmid-borne variant	BX571858.1: 8646…8993	Probes: BX571858.1 [8797:8827] and [8676:8705] Primers: BX571858.1 [8855:8874:r] and [8762:8783:r]
*cat*	Chloramphenicol acetyltransferase	Located on different plasmids	X02529.1: 2267…2914 AY355285.1: 1000…1647 U35036.1: 1167…1814	Monecke et al., 2008b, 2011
*ccrA-3*	Cassette chromosome recombinase A, type 3	Cassette chromosome recombinase A allele found in SCC*mec* III elements	AB037671.1: 5430…6776	Monecke et al., 2008b, 2011
*ccrA-4*	Cassette chromosome recombinase A, type 4	Cassette chromosome recombinase A allele found in SCC*mec* VI and SCC*mec* VIII elements	AF411935.3: 7849…9210	Monecke et al., 2008b, 2011
*ccrAA*	Cassette chromosome recombinase AA	Gene for a hypothetical protein accompanying *ccrC*. The allele in CC239 is poorly (ambiguously) recognized by the probe herein, but sequence analysis shows it presence. Hence, it is indicated in **Tables 2a,b** in brackets.	AB121219.1: 14264…15907 AM292304.1: 5654…7273	Monecke et al., 2008b, 2011
*ccrB-3*	Cassette chromosome recombinase B, type 3	Cassette chromosome recombinase B allele found in SCC*mec* III elements	AB037671.1: 6797…8425	Monecke et al., 2008b, 2011
*ccrB-4*	Cassette chromosome recombinase B, type 4	Cassette chromosome recombinase B allele found in SCC*mec* VI and SCC*mec* VIII elements	AE015929.1: 58592…60220	Monecke et al., 2008b, 2011
*ccrC*	Cassette chromosome recombinase C	Cassette chromosome recombinase C allele found in in SCC*mec* V, SCC*mec* VT and SCC*mec* VII elements. Sequence analysis (but not array hybridization) allows split into two alleles.	*ccrC*_(TSGH17)_: AB512767.1: 25157…26836 *ccrC*_(PM1)_: AB462393.1: 7514…9190	Monecke et al., 2008b, 2011
*chp*	Chemotaxis-inhibiting protein (CHIPS)	Situated on a *hlb*-integrating phage	BX571856.1: 2126786…2127235	Monecke et al., 2008b, 2011
*cstB*-SCC1 (Q2G1R6)	CsoR-like sulfur transferase-regulated genes B/metallo-beta-lactamase superfamily protein. Pseudogene containing two stop codons	Subtyping SCC*mec* II (usually present, but absent in Irish SCC*mec* II variants A to E) and III (usually present, but absent in CMFT492 GenBank HF569112.1). Also present in SCC*mec* VIII and irregular elements such as *Staphylococcus fleurettii* GenBank AB546266	BA000017.4: 50957…52291	Monecke et al., 2016
*cstB*-SCC2 (Q2G1R6)	CsoR-like sulfur transferase-regulated genes B/metallo-beta-lactamase superfamily protein.	Present in SCC*mec* IVa (truncated) and SCC*mec* X, variably present in SCC*mec* I and VT	CP000046.1: 53428…54756	Monecke et al., 2016
*czrC*	Cadmium and zinc resistance gene C, heavy metal translocating P-type ATPase	Frequently associated with SCC*mec* elements from livestock MRSA	AE015929.1: 64066…66000	Monecke et al., 2016
*dfrA*	Dihydrofolate reductase mediating trimethoprim resistance	–	AF051916.1: 2823…3308	Monecke et al., 2008b, 2011
*dfrG*	Dihydrofolate reductase mediating trimethoprim resistance	–	AB205645.1: 1013…1510	Probes: AB205645.1 [1039:1068] and [1142:1167] Primers: AB205645.1[1082:1098:r] and [1172:1189:r]
D1GU38	Putative protein	Subtyping SCC*mec* III, identification of SCC*mec* VT, SCC*mec* ZH47, SCC*mec* VII because of an association with (additional/second) *ccrC* copies	FN433596.1: 34888…35751	Monecke et al., 2016
D1GU55	Putative membrane protein	Subtyping SCC*mec* III, additional marker for SCC*mec* VII	FN433596.1: 52909…53208	Monecke et al., 2016
*erm*(A)	rRNA adenine N-6-methyltransferase, erythromycin/clindamycin resistance	–	D86934.2: 35074…35805	Monecke et al., 2008b, 2011
*erm*(C)	rRNA adenine N-6-methyltransferase, erythromycin/clindamycin resistance	–	V01278.1: 2004…2738	Monecke et al., 2008b, 2011
*fnbA*	Fibronectin-binding protein A	–	CP000046.1: 2569440…2572496	Monecke et al., 2008b, 2011
*fnbB*	Fibronectin-binding protein B	–	CP000046.1: 2565936…2568758	Monecke et al., 2008b, 2011
*hla*	Haemolysin alpha, sphingomyelin phosphodiesterase	–	X13404.1: 752…1744	Monecke et al., 2008b, 2011
*mecA*	Modified penicillin-binding protein (PBP2a)	Modified penicillin-binding protein (PBP2a) causing oxacillin/methicillin resistance and thus defining MRSA	BA000017.4: 44992…46998	Monecke et al., 2008b, 2011
*mecI*	Methicillin-resistance regulatory protein	Present in SCC*mec* II (although absent from Irish SCC*mec* II variants C and E), SCC*mec* III, SCC*mec* VIII	BA000017.4: 48855…49226	Monecke et al., 2008b, 2011
*mecR1*	Methicillin resistance operon repressor 1; Un-truncated sequence in SCC*mec* II, III, VIII. Truncated *mecR1* is present in SCC*mec* I, IV, V, VI, VII. Absent from SCC*me*c V, IX, X.	Two probes are utilized. Both yield signals in SCC*mec* II, SCC*mec* III, SCC*mec* VIII while only the Δ *mecR1* probe gives a signal for the truncated *mecR1* of SCC*mec* I, IV, V, VI, VII. Some “European Clade” strains related to 85/2082, GenBank AB037671.1 have *another* truncation resulting in a signal for the probe associated with untruncated *mecR1* but an absence of a signal for the Δ *mecR1* probe.	BA000017.4: 47098…48855	Monecke et al., 2008b, 2011
*merA*	Mercury reductase	Part of a mercury resistance operon that is plasmid-borne, although the plasmid can be integrated into SCC*mec* elements (for instance, in strains 85/2082, GenBank AB037671.1 or TW20, GenBank FN433596.1)	AB037671.1: 38289…39932	Monecke et al., 2008b, 2011
*merB*	Alkylmercury lyase	Part of a mercury resistance operon that is plasmid-borne, although the plasmid can be integrated into SCC*mec* elements (for instance, in strain TW20, GenBank FN433596.1)	AB037671.1: 37557…38207	Monecke et al., 2008b, 2011
*mupA*	Mupirocin resistance protein, alternative isoleucyl-tRNA synthase	-	X75439.1:…477…3551	Monecke et al., 2008b, 2011
*mvaS*-SCC	Truncated 3-hydroxy-3-methylglutaryl CoA synthase	Subtyping SCC*mec* I, II, IV, V	BA000033.2: 37179…37531	Monecke et al., 2016
PSM*-mec*	Phenol soluble modulin from SCC*mec*	Present in SCC*mec* II (although absent from Irish SCC*mec* II variants C and E), SCC*mec* III, SCC*mec* VIII	BA000017.4: 49311…49379	(Monecke et al., 2016)
Q4LAG7	Putative protein located within SCC*mec* type V/SCC*fus* elements	Identification of SCC*mec* V/VT elements and of SCC*fus* elements	AM990992.1: 50512…50940 (V/VT) BX571857.1: 55452…55880 (fus)	Monecke et al., 2016
Q8CU82	Putative protein	Present in some SCC*mec*/*fus* composite elements such as e.g., CMFT120, GenBank HF569094.1 and CMFT2, GenBank HF569101.1	AE015929.1: 32604…32786	Monecke et al., 2016
Q933A2	Putative ADP-ribosyltransferase	Subtyping SCC*mec* III and SCC*mec* IX	FN433596.1: 101805…102377	Monecke et al., 2016
Q93IB7	LytTR domain DNA-binding regulator	Subtyping SCC*mec* III (present in, e.g., TW20 GenBank FN433596.1, but absent in, e.g., Bmb9393 GenBank CP005288.1) and IV (usually absent, but present in, e.g., CMFT503 GenBank HF569113.1)	FN433596.1: 67873…68115	Monecke et al., 2016
Q9S0M4	Putative protein	Subtyping SCC*mec* I, SCC*mec*/ACME composites and SCC*mec* from WA40	JQ746621.1: 10406…11456	Monecke et al., 2016
Q9XB68*-dcs*	Located at the terminus of SCC*mec* directly next to *orfX*	This locus comprises the downstream constant segment (*dcs*) that in turn comprises a copy of the SCC direct repeat DR_SCC (AGAAGCTTATCATAAGTAA).	*dcs*: CP000046.1: 34192…34371 Q9XB68: CP000046.1: 34372…35667	Monecke et al., 2008b, 2011
*qacA*	Quaternary ammonium compound resistance protein A	–	AF535086.1: 29…1573	Monecke et al., 2008b, 2011
*qacC*	Quaternary ammonium compound resistance protein C	–	AY121858.1: 261…584	Monecke et al., 2008b, 2011
*sak*	Staphylokinase	Situated on a *hlb*-integrating phage	AF424783.1: 38467…38958	Monecke et al., 2008b, 2011
*sat*	Streptothricin-acetyltransferase	Part of a gene cluster comprising of *sat, aphA3* and variably *aadE*	CP000028.1: 8575…9105	Monecke et al., 2008b, 2011
*sasX/sesI*	Surface-anchored protein X	Located on 127 kb prophage phiSPbeta	FN433596.1: 2307955…2308569	Probes: FN433596.1 [2308472:2308498:r] and [2308232:2308256:r] Primers: FN433596.1 [2308444:2308466] and [2308203:2308220]
SCC terminus 01	SCC integration site alternate to *dcs*	–	GU235983.1: 488…808	Monecke et al., 2016
SCC terminus 02	SCC integration site alternate to *dcs*	–	FN433596.1: 34140…34456	Monecke et al., 2016
SCC terminus 03	SCC integration site alternate to *dcs*	–	FR753166.1: 481…568	Monecke et al., 2016
SCC terminus 05	SCC integration site alternate to *dcs*	–	AB425427.1: 606…1027	Monecke et al., 2016
SCC terminus 07	SCC integration site alternate to *dcs*	–	GU122149.1: 119…222	Monecke et al., 2016
*scn*	Staphylococcal complement inhibitor (SCIN)	Situated on a *hlb*-integrating phage	AF424783.1: 41170…41520	Monecke et al., 2008b, 2011
*sea*	Enterotoxin A	Situated on a *hlb*-integrating phage	M18970.1: 1…774	Monecke et al., 2008b, 2011
*sea*_(N315)_*/sep*	Enterotoxin A allele from strain N315 (GenBank BA000018.3), also known as Enterotoxin P	Situated on a *hlb*-integrating phage	BA000018.3: 2011380…2012153	Monecke et al., 2008b, 2011
*sek*	Enterotoxin K	–	CP000046.1: 904800…905528	Monecke et al., 2008b, 2011
*seq*	Enterotoxin Q	–	CP000046.1: 905552…906280	Monecke et al., 2008b, 2011
*splA*	Serine protease A	–	AF271715.1: 1001…1708	Monecke et al., 2008b, 2011
*splB*	Serine protease B	–	AF271715.1: 1833…2555	Monecke et al., 2008b, 2011
*splE*	Serine protease E	–	AF271715.1: 4330…5046	Monecke et al., 2008b, 2011
*tet*(K)	Tetracycline efflux protein variant K	–	U38656.1: 436…1815	Monecke et al., 2008b, 2011
*tet*(M)	Ribosomal protection tetracycline resistance protein M	–	M21136.1: 458…2377	Monecke et al., 2008b, 2011
*tst1*	Toxic shock syndrome toxin 1	–	BA000017.4: 2137509…2138213	Monecke et al., 2008b, 2011
*ugpQ*	Glycerophosphoryl diester phosphodiesterase	Accompanies *mecA* in all *S. aureus* SCC*mec* sequences except SCC*mec* IV A from CN1 GenBank CP003979.1	BA000018.3: 43717…44460	Monecke et al., 2008b, 2011
*xylR/mecR2*	Methicillin resistance operon repressor 2, Homolog of xylose repressor	Located next to *mec* operon downstream of *mecI* (not present if *mecI* is truncated). Present in SCC*mec* II (although absent from Irish SCC*mec* II variants C and E), SCC*mec* III, SCC*mec* VIII	BA000018.3: 49738…50882	Monecke et al., 2008b, 2011

All markers of relevance for SCC*mec* subtyping are listed in the Supplemental Table 1. Markers that were detected among isolates in the present study are listed, and presented in more detail, in Table 1.

### DNA microarrays for *mecA* subtyping

A third assay was used to identify and categorize alleles and variants of the *mecA/C* gene as previously described (Monecke et al., 2012a). While there are a multitude of *mecA* variants (also named *mecA*1) in staphylococcal species other than *S*. *aureus*, only four different *mecA/C* alleles are of relevance in *S. aureus*/MRSA that also differ in amino acid sequences encoded, i.e., (named with respect to representative GenBank entries), *mecA*_(CP000046)_ (as in the CC8-MRSA-I strain COL), *mecA*_(BA000018)_ (as in the CC5-MRSA-II strain N315), *mecA*_(GQ902038)_ (as in the CC398-MRSA-VT strain UMCG-M4) and *mecC* (GenBank NG_047955.1). This array was applied to 30 isolates representing at least one isolate per SCC*mec* subtype.

### SCC*mec* subtypes and nomenclature

The guidelines of the International Working Group (IWG-SCC, 2009) were used for the assignment of Roman numerals to SCC*mec* types defined by the class of *mec* gene complex and type of cassette chromosome recombinase (*ccr*) genes. As proposed by Shore and Coleman (2013) we named elements lacking *ccr* recombinase genes “pseudoSCC*mec* elements.” Composite elements are indicated by listing relevant components in square brackets. Heavy metal resistance genotypes were described by adding chemical symbols rather than individual gene designations e.g., SCC [*mec* III+Cd/Hg+*ccrC*]).

Genes *aadD, aacA-aphD, ant9, ble*, and *erm*(A) as well as *tet* genes were not included in the analysis of the SCC*mec* III subtypes. Although these genes may be situated on SCC*mec* elements, they also may be found on plasmids or other mobile genetic elements at various locations [see below for *erm*(A) and *ant9*]. Neither array hybridization nor those NGS technologies that yield a high number of short contigs can provide reliable information on the actual localizations of genes, or whether a plasmid [such as p*T181*/*tet*(K)] was free or integrated into the genome. Consequently, we did not differentiate between SCC*mec* III and IIIA (Vandenesch et al., 2003).

In contrast, the mercury resistance operon was included into the analysis of the SCC*mec* III subtypes. In CC239 this operon is part of a composite SCC element, although it can indeed be found outside of SCC elements (e.g., GenBank: AB179623.1).

All previously sequenced variants were tagged with the designation of one reference strain in which they have been sequenced [e.g., the particular variant of SCC*mec* III from the strain TW20 is indicated SCC [*mec* III+Cd/Hg+ccrC] _(TW20)_]. If we were not able to identify a reference sequence to a given SCC hybridization pattern, we added “unknown” followed by the clonal complex(es) and, if there were several similar such elements, by chronologically assigned numbers [as in SCC [*mec* III+Cd+*ccrC*] _(Unknown, ST239−3)_].

### Sequencing

The genomes of 30 CC239 isolates from Perth/Australia have been sequenced with Illumina MiSEQ. Sequencing libraries were prepared with the Nextera kit (Illumina).

### Genome assembly

Sequencing reads of 30 CC239 isolates from Perth/Australia as well as several read sets downloaded from NCBI Short read Archive (https://www.ncbi.nlm.nih.gov/sra/; see **Table 4** and Supplemental Table 1) were assembled with SPAdes version 3.10.1 (Bankevich et al., 2012). No attempts were made to close gaps between contigs. Contigs shorter than 500 nt were excluded from further analysis.

### Bioinformatics, virtual hybridizations, and probe mapping

To date, several thousand either partially or fully assembled genomes of *S. aureus* isolates are available in NCBI GenBank. Fully assembled genomes comprise one or several sequences representing complete replicons (the bacterial chromosome and a variable number of plasmids). Partially assembled sequences consist of a set of contigs. The contigs usually end in repeats and the sequencing reads do not comprise enough information to link contigs unambiguously. The number of contigs varies between about 10 and several hundred depending on the sequencing method, read length, fragment size, coverage depth and assembling strategy and settings. Partially assembled contigs are available in NCBI Genbank (http://www.ncbi.nlm.nih.gov/Traces/wgs/) with special accession numbers assigned which start with four letters followed by eight digits. The entire set of contigs is referred to by a accession number which has all digits set to zero (e.g., AICH00000000.1). For the sake of conciseness, we will refer to these four-letter codes as an unambiguous identifier of a specific genome here. To genomes which we have assembled from raw sequencing reads obtained from the Short Read Archive (https://www.ncbi.nlm.nih.gov/sra/), we refer to henceforth by the BioSample accession number (e.g., SAMEA1029552).

A total of 215 genome sequences of CC239 available in the NCBI database (**Table 4** and Supplemental Table 1) as well as genome sequences of 30 previously unpublished Australian study isolates were subjected to an *in silico* analysis or “virtual hybridization” that allowed a direct comparison to array hybridization experiments (Tables 2a,b, 3 and Supplemental Table 1). Hybridization patterns were generated from complete or from partially assembled genomic sequences.

**Table 2a:** SCC*mec*-associated patterns observed in, or derived from sequences of, CC239-MRSA assigned to the “Eurasian” (**EA**), “European” (**EU**), “Australian/NZ” (**AU/NZ**) Clades, to the “unassigned Middle Eastern Strain“ (**ME**), or to the atypical cluster of “South-East Asian Clade” isolates (S2, DEN907; **SEA**).

**SCC*mec* subtype**	**Reference sequence**	***mecA* allele**	***mec* complex**	**Heavy metal resistance genes**	**Other payload**	**Recombinase genes**	**SCC termini**	***nsaB* status**	**Clade**
**SCC [*****mec*** **III**+**Cd**+***ccrC*****]**_(T0131)_	T0131, CP002643.1	*mecA*_(BA000018)_	*mecA, ugpQ, mecR1*, Δ *mecR1, mecI*, PSM-*mec, xylR/mecR2, cstB-SCC1, mvaS-SCC*	*cadD*_(R35)_	D1GU38, Q933A2	*ccrA+B-3, ccr(AA)+C*_(PM1)_	*dcs*, SCCterm02	Insertion	**EA**
**SCC [*****mec*** **III**+**As/Cd**+***ccrC*****]**_(Unknown, ST239−1)_	N/A	*mecA*_(BA000018)_	*mecA, ugpQ, mecR1*, Δ *mecR1, mecI*, PSM-*mec, xylR/mecR2, cstB-SCC1, mvaS-SCC*	*cadD*_(R35)_, *arsC*-SCC	D1GU38, Q933A2	*ccrA+B-3, ccr(AA)+C*	*dcs*, SCCterm02	N/A	**EA**
**SCC [*****mec*** **III**+**Cd**+***ccrC*****]**_(CN79)_	CN79, ANCJ	*mecA*_(BA000018)_	*mecA, ugpQ, mecR1*, Δ *mecR1, mecI*, PSM*-mec, xylR/mecR*2*, cstB*-SCC1*, mvaS*-SCC	*cadD*_(R35)_	D1GU38, D1GU55, Q933A2	*ccrA+B-3, ccr(AA)+C*_(PM1)_	*dcs*, SCCterm02	Insertion	**EA**
SCC [*mec* III+Cd+*ccrC*] _(IU17)_	IU17, CTXB	*mecA*_(BA000018)_	*mecA, ugpQ, mecR1*, Δ *mecR1, mecI*, PSM*-mec, xylR/mecR*2*, cstB*-SCC1*, mvaS*-SCC	*cadD*_(R35)_	D1GU38, D1GU55, Q933A2	*ccrA+B-3, ccr(AA)+C*_(PM1)_	SCCterm02	Insertion	**EA**
**SCC [*****mec*** **III**+**As/Cd**+***ccrC*****]**_(Unknown, ST239−2)_	N/A	*mecA*_(BA000018)_	*mecA, ugpQ, mecR1*, Δ *mecR1, mecI, PSM-mec, xylR/mecR2, cstB-SCC1, mvaS-SCC*	*cadD*_(R35)_, *arsC*-SCC	D1GU38, D1GU55, Q933A2	*ccrA+B-3, ccr(AA)+C*	*dcs*, SCCterm02	N/A	**EA**
**SCC [*****mec*** **III**+**Cd**+***ccrC*****]**_(Unknown, ST239−1)_	N/A	*mecA*_(BA000018)_	*mecA, ugpQ, mecR1*, Δ *mecR1, mecI*, PSM*-mec, xylR/mecR*2*, cstB*-SCC1*, mvaS*-SCC	*cadD*_(R35)_	Q933A2, Q93IB7	*ccrA+B-3, ccr(AA)+C*	SCCterm01	N/A	**ME**
**SCC [*****mec*** **III**+**Cd**+***ccrC*****]**_(Unknown, ST239−2)_	N/A	*mecA*_(BA000018)_	*mecA, ugpQ, mecR1*, Δ *mecR1, mecI*, PSM*-mec, xylR/mecR*2*, cstB*-SCC1*, mvaS*-SCC	*cadD*_(R35)_	D1GU38, D1GU55, Q4LAG7, Q933A2, Q9S0M4	*ccrA+B-3, ccr(AA)+C*	*dcs*, SCCterm02, SCCterm05	N/A	**EA**
**SCC [*****mec*** **III**+**Cd**+***ccrC*****]**_(Unknown, ST239−3)_	N/A	*mecA*_(BA000018)_	*mecA, ugpQ, mecR1*, Δ *mecR1, mecI*, PSM*-mec, xylR/mecR*2*, cstB*-SCC1*, mvaS*-SCC	*cadD*_(R35)_	D1GU55, Q4LAG7, Q933A2, Q9S0M4	*ccrA+B-3, ccr(AA)+C*	*dcs*, SCCterm05	N/A	**EA**
**SCC [*****mec*** **III**+ **ACME II**+**Cd**+***ccrC*****]**_(Unknown, ST239)_	N/A	*mecA*_(BA000018)_	*mecA, ugpQ, mecR1*, Δ *mecR1, mecI*, PSM*-mec, xylR/mecR*2*, cstB*-SCC1*, mvaS*-SCC	*cadD*_(R35)_	D1GU38, D1GU55, Q933A2, Q9S0M4, *arc*-genes	*ccrA+B-3, ccr(AA)+C*	*dcs*, SCCterm02, SCCterm03, SCCterm05	N/A	**EA**
SCC [*mec* III+Cd+*ccrC*] _(TUR1)_	TUR1, SAMEA 1029552	*mecA*_(CP000046)_	*mecA, ugpQ, mecR1*, Δ *mecR1, mecI*, PSM-*mec, xylR/mecR2, cstB-SCC1, mvaS-SCC*	*cadD*_(R35)_	D1GU38, D1GU55, Q933A2, Q93IB7	*ccrA+B-3, ccr(AA)+C*_(PM1)_	SCCterm01, SCCterm02	Insertion	**EA**
**SCC [*****mec*** **III**+**Cd/Hg**+***ccrC*****]**_(SK1585)_	SK1585, KL662257.1	*mecA*_(CP000046)_	*mecA, ugpQ, mecR1*, Δ *mecR1, mecI*, PSM-*mec, xylR/mecR2, cstB-SCC1, mvaS*-SCC	*mer*-operon*, cadD*_(R35)_	D1GU38, D1GU55, Q933A2, Q93IB7	*ccrA+B-3, ccr(AA)+C*_(PM1)_	SCCterm01, SCCterm02	Wild type	**EU**
SCC [*mec* III (Δ *mec*R1-)+Cd/Hg+*ccrC*] _(BK2421)_	BK2421, SAMEA 1029510	*mecA*_(CP000046)_	*mecA, ugpQ, mecR*1*, mecI*, PSM*-mec, xylR/mecR*2*, cstB*-SCC1	*mer*-operon, *cadD*_(R35)_	D1GU38, D1GU55, Q933A2, Q93IB7	*ccrA+B-3, ccr(AA)+C*_(PM1)_	SCCterm01, SCCterm02	Wild type	**EU**
**SCC [*****mec*** **III (**Δ ***mec*****R1-)**+**Cd/Hg**+***ccrC*****]**_(85/2082)_	85/2082, AB037671.1	*mecA*_(CP000046)_	*mecA, ugpQ, mecR*1*, mecI*, PSM*-mec, xylR/mecR*2*, cstB*-SCC1*, mvaS*-SCC	*mer*-operon, *cadD*_(R35)_	D1GU38, D1GU55, Q933A2, Q93IB7	*ccrA+B-3, ccr(AA)+C*_(PM1)_	SCCterm01, SCCterm02	Wild type	**EU**
**SCC [*****mec*** **III**+**Cd]**_(JKD6008)_	JKD6008, CP002120.1	*mecA*_(BA000018)_	*mecA, ugpQ, mecR1*, Δ *mecR1, mecI*, PSM*-mec, xylR/mecR*2*, cstB*-SCC1*, mvaS*-SCC	*cadD*_(R35)_	Q933A2	*ccrA+B-3*	*dcs*	Wild type	**AU/NZ**
**SCC [*****mec*** **III**+ **ACME II**+**Cd]**_(Unknown, ST239−1)_	N/A	*mecA*_(BA000018)_	*mecA, ugpQ, mecR1*, Δ *mecR1, mecI*, PSM*-mec, xylR/mecR*2*, cstB*-SCC1*, mvaS*-SCC	*cadD*_(R35)_	Q933A2, Q9S0M4, *arc*-genes	*ccrA+B-3*	*dcs*, SCCterm03, SCCterm05	N/A	**AU/NZ**
SCC [*mec* III+Cd] _(S2)_	S2, SAMEA 1029563	*mecA*_(CP000046)_	*mecA, ugpQ, mecR1*, Δ *mecR1, mecI*, PSM*-mec, xylR/mecR*2*, cstB*-SCC1	*cadD*_(R35)_	Q933A2	*ccrA+B-3*	*dcs*	N/A	**SEA**
	M116, GenBank CTXQ	*mecA*_(BA000018)_	*mecA, ugpQ, mecR1*, Δ *mecR1, mecI*, PSM*-mec, xylR/mecR*2*, cstB*-SCC1	*cadD*_(R35)_	Q933A2	*ccrA+B-3*	*dcs*		**SEA**
SCC [*mec* III+Cd/Hg] _(DEN907)_	DEN907, SAMEA 1029548	*mecA*_(CP000046)_	*mecA, ugpQ, mecR1*, Δ *mecR1, mecI*, PSM*-mec, xylR/mecR*2*, cstB*-SCC1	*mer*-operon, *cadD*_(R35)_	Q933A2	*ccrA+B-3*	*dcs*	Wild type	**SEA**
SCCmec III _(KM1381)_, *for comparison*	KM1381, AM904732.1	*mecA*_(BA000018)_	*mecA, ugpQ, mecR1*, Δ *mecR1, mecI*, PSM-*mec, xylR/mecR2, cstB-SCC1, mvaS-SCC*	N/A	Q933A2	*ccrA+B-3*	*dcs*	N/A	***pseudint***.

*The predicted pattern for S. pseudintermedius, KM1381 is provided for comparison. Bold font of element designations indicates those elements that were found in study isolates*.

**Table 2b:** SCC*mec*-associated patterns observed in, or derived from sequences of, CC239-MRSA assigned to the “South-East Asian” (**SEA**), “South American/Middle Eastern” (**SA/ME**) and “Portuguese” (**POR**) clades.

**SCC*mec* subtype**	**Reference sequence**	***mecA* allele**	***mec* complex**	**Heavy metal resistance genes**	**Other payload**	**Recombinase genes**	**SCC termini**	***nsaB* status**	**Clade**
**SCC [*****mec*** **III**+**Cd/Hg**+***ccrC*****]**_(TW20)_	TW20, FN433596.1	*mecA*_(CP000046)_	*mecA, ugpQ, mecR1*, Δ *mecR1, mecI*, PSM*-mec, xylR/mecR*2*, cstB*-SCC1*, mvaS*-SCC	*mer*-operon, *cadD*_(R35)_	D1GU38, Q933A2, Q93IB7	*ccrA+B-3, ccr(AA)+C*_(TSGH17)_	SCCterm01, SCCterm02	Wild type	**SEA, SA/ME**
	NMR05, LWAL	*mecA*_(BA000018)_	*mecA, ugpQ, mecR1*, Δ *mecR1, mecI*, PSM*-mec, xylR/mecR*2*, cstB*-SCC1*, mvaS*-SCC	*mer*-operon, *cadD*_(R35)_	D1GU38, Q933A2, Q93IB7	*ccrA+B-3, ccr(AA)+C*_(TSGH17)_	SCCterm01, SCCterm02	Wild type	**SEA, SA/ME**
**SCC [*****mec*** **III**+**Hg**+***ccrC*****]**_(AR23)_	N/A	*mecA*_(CP000046)_	*mecA, ugpQ, mecR1*, Δ*mecR1, mecI*, PSM*-mec, xylR/mecR*2*, cstB*-SCC1*, mvaS*-SCC	*mer*-operon	D1GU38, Q933A2, Q93IB7	*ccrA+B-3, ccr(AA)+C*	SCCterm01, SCCterm02	N/A	**SA/ME**
**SCC [*****mec*** **III**+**Hg]**_(Unknown, ST239)_	N/A	*mecA*_(CP000046)_	*mecA, ugpQ, mecR1*, ΔΔ *mecR1, mecI*, PSM*-mec, xylR/mecR*2*, cstB*-SCC1*, mvaS*-SCC	*mer*-operon	D1GU38, Q933A2, Q93IB7	*ccrA+B-3*	SCCterm01, SCCterm02	N/A	**SEA**
**SCC [*****mec*** **III**+**Cd/Hg]**_(Unknown, ST239−1)_	N/A	*mecA*_(CP000046)_	*mecA, ugpQ, mecR1*, Δ*mecR1, mecI*, PSM*-mec, xylR/mecR*2*, cstB*-SCC1*, mvaS*-SCC	*mer*-operon, *cadD*_(R35)_	D1GU38, Q933A2, Q93IB7	*ccrA+B-3*	SCCterm01, SCCterm02	N/A	**SEA**
**SCC [*****mec*** **III**+**Cd**+***ccrC*****]**_(S85)_	S85, SAMEA 1029529	*mecA*_(CP000046)_	*mecA, ugpQ, mecR1*, Δ *mecR1, mecI*, PSM*-mec, xylR/mecR*2*, cstB*-SCC1*, mvaS*-SCC	*cadD*_(R35)_	D1GU38, Q933A2, Q93IB7	*ccrA+B-3, ccr(AA)+C*_(TSGH17)_	SCCterm01, SCCterm02	Wild type	**SEA, SA/ME**
**SCC [*****mec*** **III**+**Cd/Hg**+***ccrC*****]**_(Bmb9393)_	Bmb9393, CP005288.1	*mecA*_(CP000046)_	*mecA, ugpQ, mecR1*, Δ *mecR1, mecI*, PSM*-mec, xylR/mecR*2*, cstB*-SCC1*, mvaS*-SCC	*mer*-operon, *cadD*_(R35)_	D1GU38, Q933A2	*ccrA+B-3, ccr(AA)+C*_(TSGH17)_	SCCterm02	Wild type	**SEA, SA/ME**
	URU110, SAMEA 1029540	*mecA*_(BA000018)_	*mecA, ugpQ, mecR1*, Δ *mecR1, mecI*, PSM*-mec, xylR/mecR*2*, cstB*-SCC1*, mvaS*-SCC	*mer*-operon, *cadD*_(R35)_	D1GU38, Q933A2	*ccrA+B-3, ccr(AA)+C*_(TSGH17)_	SCCterm02	Wild type	**SA/ME**
**SCC [*****mec*** **III**+**Cd**+***ccrC*****]**_(PPUKM−775−2009)_	PPUKM-775-2009, AMRE	*mecA*_(CP000046)_	*mecA, ugpQ, mecR1*, Δ *mecR1, mecI*, PSM*-mec, xylR/mecR*2*, cstB*-SCC1	*cadD*_(R35)_	D1GU38, Q933A2	*ccrA+B-3, ccr(AA)+C*_(TSGH17)_	SCCterm02	Wild type	**SEA**
**SCC [*****mec*** **III**+**Cd/Hg]**_(Unknown, ST239−2)_	N/A	*mecA*_(CP000046)_	*mecA, ugpQ, mecR1*, Δ *mecR1, mecI*, PSM*-mec, xylR/mecR*2*, cstB*-SCC1*, mvaS*-SCC	*mer*-operon, *cadD*_(R35)_	D1GU38, Q933A2	*ccrA+B-3*	SCCterm02	N/A	**SEA**
SCC [*mec* III+Cd/Hg] _(BRA2)_	BRA2, SAMEA 1029514	*mecA*_(CP000046)_	*mecA, ugpQ, mecR1*, Δ *mecR1, mecI*, PSM*-mec, xylR/mecR*2*, cstB*-SCC1*, mvaS*-SCC	*mer*-operon, *cadD*_(R35)_	Q933A2	*ccrA+B-3*	SCCterm02	Wild type	**SA/ME**
**SCC [*****mec*** **III**+**Cd/Hg**+***ccrC*****]**_(Z172)_	Z172, CP006838.1	*mecA*_(CP000046)_	*mecA, ugpQ, mecR1*, Δ *mecR1, mecI*, PSM*-mec, xylR/mecR*2*, cstB*-SCC1*, mvaS*-SCC	*mer*-operon, *cadD*_(R35)_	D1GU38, Q933A2	*ccrA+B-3, ccr(AA)+C*_(TSGH17)_	SCCterm02	Wild type	**SEA**
**SCC [*****mec*** **III**+**Cd/Hg**+***ccrC*****]**_(Unknown ST239)_	N/A	*mecA*_(CP000046)_	*mecA, ugpQ, mecR1*, Δ *mecR1, mecI*, PSM*-mec, xylR/mecR*2*, cstB*-SCC1	*mer*-operon, *cadD*_(R35)_	D1GU38, Q933A2	*ccrA+B-3, ccr(AA)+C*	SCCterm02	N/A	**SEA**
**SCC [*****mec*** **III**+**Cd**+***ccrC*****]**_(XN108)_	XN108, CP007447.1	*mecA*_(CP000046)_	*mecA, ugpQ, mecR1*, Δ *mecR1, mecI*, PSM*-mec, xylR/mecR*2*, cstB*-SCC1*, mvaS*-SCC	*cadD*_(R35)_	D1GU38, Q933A2	*ccrA+B-3, ccr(AA)+C*_(TSGH17)_	SCCterm02	Wild type	**SEA, SA/ME**
	DS_014, FQPU	*mecA*_(BA000018)_	*mecA, ugpQ, mecR1*, Δ *mecR1, mecI*, PSM*-mec, xylR/mecR*2*, cstB*-SCC1*, mvaS*-SCC	*cadD*_(R35)_	D1GU38, Q933A2	*ccrA+B-3, ccr(AA)+C*_(TSGH17)_	SCCterm02	Wild type	**SA/ME**
**SCC [*****mec*** **III**+**Cd]**_(HSA10/ATCC33592)_	ATCC33592, JXZH	*mecA*_(CP000046)_	*mecA, ugpQ, mecR1*, Δ *mecR1, mecI*, PSM*-mec, xylR/mecR*2*, cstB*-SCC1*, mvaS*-SCC	*cadD*_(R35)_	Q933A2, Q93IB7	*ccrA+B-3*	SCCterm01	Wild type	**POR**
	P32, SAMEA 862589	*mecA*_(BA000018)_	*mecA, ugpQ, mecR1*, Δ *mecR1, mecI*, PSM*-mec, xylR/mecR*2*, cstB*-SCC1*, mvaS*-SCC	*cadD*_(R35)_	Q933A2, Q93IB7	*ccrA+B-3*	SCCterm01	Wild type	**SEA**
**SCC [*****mec*** **III**+**Cd/Hg**+***ccrC*****]**_(M418/UK−9)_	M418, CTXS	*mecA*_(CP000046)_	*mecA, ugpQ, mecR1*, Δ *mecR1, mecI*, PSM*-mec, xylR/mecR*2*, cstB*-SCC1*, mvaS*-SCC	*mer*-operon, *cadD*_(R35)_	D1GU38, Q933A2, Q9S0M4	*ccrA+B-3, ccr(AA)+C*	SCCterm02, SCCterm05	Wild type	**SEA, SA/ME**
**SCC [*****mec*** **III**+**Cd**+***ccrC*****]**_(NMR08)_	CP023560.1	*mecA*_(CP000046)_	*mecA, ugpQ, mecR1*, Δ *mecR1, mecI*, PSM*-mec, xylR/mecR*2*, cstB*-SCC1*, mvaS*-SCC	*cadD*_(R35)_	D1GU38, Q933A2, Q9S0M4	*ccrA+B-3, ccr(AA)+C*_(TSGH17)_	SCCterm02, SCCterm05	Wild type	**SEA**
**SCC [*****mec*** **III**+**Cd/Hg]**_(Unknown, ST239−3)_	N/A	*mecA*_(CP000046)_	*mecA, ugpQ, mecR1*, Δ *mecR1, mecI*, PSM*-mec, xylR/mecR*2*, cstB*-SCC1*, mvaS*-SCC	*mer*-operon, *cadD*_(R35)_	Q933A2, Q9S0M4	*ccrA+B-3*	SCCterm05	N/A	**SA/ME**
**SCC [*****mec*** **III**+**speG**+**Cd/*****czrC**+**ccrAB4C*****]**_(Unknown, ST239)_	N/A	*mecA*_(CP000046)_	*mecA, ugpQ, mecR1*, Δ *mecR1, mecI*, PSM*-mec, xylR/mecR*2*, cstB*-SCC1*, mvaS*-SCC	*czrC, cadD*_(R35)_	D1GU38, Q933A2, *speG*	*ccrA+B-3, ccr(AA)+C ccr*(A)*/B-4*,	SCCterm02, SCCterm07	N/A	**SEA**
**SCC [*****mec*** **III**+**speG**+**Cd/Hg/*****czrC**+**ccrAB4C*****]**_(Unknown, ST239)_	N/A	*mecA*_(CP000046)_	*mecA, ugpQ, mecR1*, Δ *mecR1, mecI*, PSM*-mec, xylR/mecR*2*, cstB*-SCC1*, mvaS*-SCC	*mer*-operon, *czrC, cadD*_(R35)_	D1GU38, Q933A2, *speG*	*ccrA+B-3, ccr(AA)/C, ccr*(A)*/B-4*	SCCterm02, SCCterm07	N/A	**SEA**
**SCC [*****mec*** **III**+ **ACME II**+**Cd/Hg]**_(Unknown, ST239)_	N/A	*mecA*_(CP000046)_	*mecA, ugpQ, mecR1*, Δ *mecR1, mecI*, PSM*-mec, xylR/mecR*2*, cstB*-SCC1*, mvaS*-SCC	*mer*-operon, *cadD*_(R35)_	Q933A2, Q93IB7, Q9S0M4, *arc*-genes	*ccrA+B-3*	SCCterm05	N/A	**SEA**
**SCC [*****mec*** **III**+ **ACME II**+**Cd]**_(Unknown, ST239−2)_	N/A	*mecA*_(CP000046)_	*mecA, ugpQ, mecR1*, Δ *mecR1, mecI*, PSM*-mec, xylR/mecR*2*, cstB*-SCC1*, mvaS*-SCC	*cadD*_(R35)_	Q933A2, Q93IB7, *arc*-genes	*ccrA+B-3*	SCCterm01, SCCterm03	N/A	**SEA**
**PseudoSCC*****mec*** **[class A**+**Cd/Hg**+***ccrC*****]**_(UCIM6015)_	UCIM6015, JBMO	*mecA*_(CP000046)_	*mecA, ugpQ, mecR1*, Δ *mecR1, mecI*, PSM*-mec, xylR/mecR*2*, cstB*-SCC1*, mvaS*-SCC	*mer*-operon, *cadD*_(R35)_	D1GU38, Q93IB7	*ccr(AA)+C*_(TSGH17)_	SCCterm01, SCCterm02	Wild type	**SEA**
PseudoSCC*mec* [class A+Cd/Hg+*ccrC*] _(UP1073)_	UP1073, MIHO	*mecA*_(CP000046)_	*mecA, ugpQ, mecR1*, Δ *mecR1, mecI*, PSM*-mec, xylR/mecR*2*, cstB*-SCC1*, mvaS*-SCC	*mer*-operon, *cadD*_(R35)_	–	–	SCCterm02	Wild type	**SA/ME**

*Bold font of element designations indicates those elements that were found in study isolates*.

**Table 3:** Clades and Strains and their genotypic characterization, as identified in this study or when analyzing published genome sequences.

**Clade**	**Strain/isolate name, reference strain or description**	**Reference sequence**	**PS**	**SI**	**SCCmec element**	***cadX***	***blaZ+R+I***	***erm*(A)**	***erm*(C)**	***aacA-aphD***	***aadD***	***aadE***	***ant9***	***aphA3+sat***	***ble***	***dfrA***	***dfrG***	***mupA***	***tet*(K)**	***tet*(M)**	***cat***	***qacA***	***qacC***	***sek+seq***	***sesX/sesI***	***Hlb*-integrating phage**	**Other notable features**
**Eurasian**	TUR1	SAMEA1029552	2	0	SCC [*mec* III+Cd+*ccrC*] _(TUR1)_	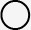	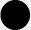	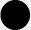	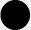	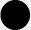	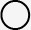	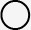	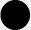	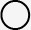	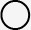	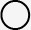	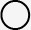	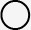	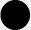	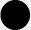	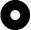	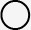	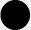	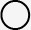	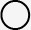	*sea+sak+scn*	***splE*** **and** ***fnbB*** **absent**
	**T0131**	CP002643.1	4	1	SCC [*mec* III+Cd+*ccrC*] _(T0131)_	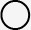	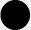	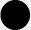	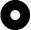	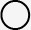	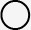	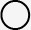	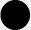	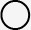	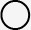	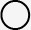	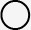	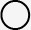	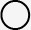	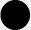	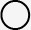	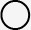	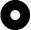	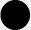	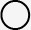	*sea+sak+scn*	***splE*** **and** ***fnbB*** **absent**
	***arsC*****-SCC-pos. strains from Romania**	–	0	2	SCC [*mec* III+As/Cd+*ccrC*] _(Unknown, ST239−1)_ SCC [*mec* III+As/Cd+*ccrC*] _(Unknown, ST239−2)_	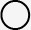	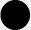	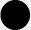	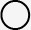	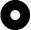	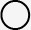	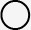	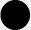	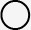	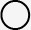	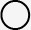	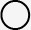	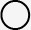	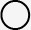	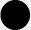	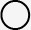	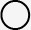	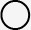	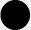	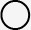	*sea+ sak+ scn*	***splE*** **and** ***fnbB*** **absent**
	**16K/CN79**	GenBank ANCJ, BABZ	60	28	SCC [*mec* III+Cd+*ccrC*] _(CN79)_	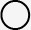	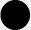	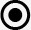	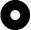	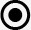	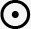	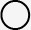	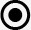	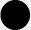	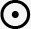	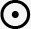	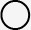	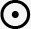	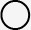	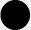	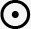	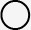	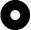	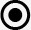	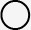	*sea+sak+scn* (comm.); *sea*_(N315)_*+ sak + chp + scn* (rare, Saint Petersburg only); none (rare)	***splE*** **and** ***fnbB*** **absent**
	IU17	GenBank CTXB	2	0	SCC [*mec* III+Cd+*ccrC*] _(IU17)_	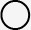	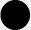	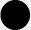	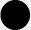	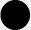	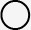	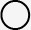	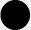	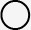	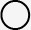	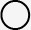	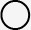	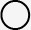	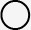	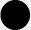	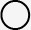	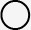	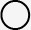	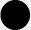	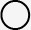	*sea+sak+scn*	***splE and fnbB absent***
	**Sporadic strains from Saxony**	–	0	2	SCC [*mec* III+Cd+*ccrC*] _(Unknown, ST239−2)_ SCC [*mec* III+Cd+*ccrC*] _(Unknown, ST239−3)_	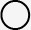	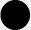	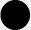	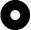	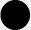	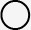	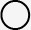	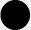	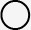	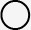	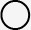	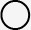	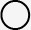	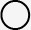	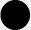	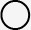	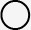	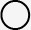	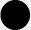	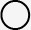	*sea*	***splE*** **and** ***fnbB*** **absent**
	**Kuwaiti ACME II-pos. Strain**	–	0	2	SCC [*mec* III+ ACME II+Cd+*ccrC*] _(Unknown, ST239)_	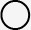	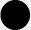	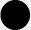	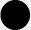	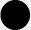	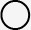	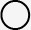	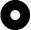	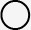	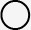	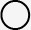	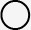	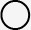	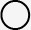	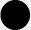	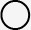	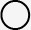	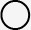	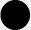	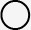	*sea*	***splE*** **and** ***fnbB*** **absent**. ACME II present
**European**	**“Greek Epidemic Strain”**	SAMEA1029513, SAMEA1029532	3	7	SCC [*mec* III+Cd/Hg+*ccrC*] _(SK1585)_	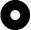	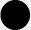	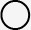	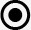	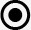	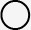	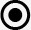	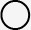	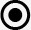	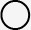	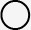	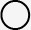	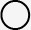	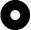	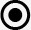	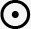	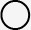	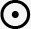	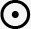	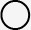	*sea+sak+scn*	*fnbA* commonly absent
	**Irish AR01/UK EMRSA-01**	SAMEA1029537, AB037671.1 (SCC*mec* only)	2	5	SCC [*mec* III (Delta *mecR*1-)+ Cd/Hg+*ccrC*] _(85/2082)_	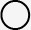	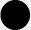	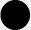	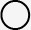	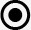	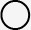	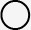	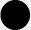	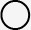	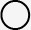	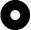	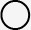	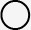	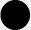	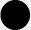	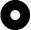	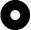	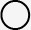	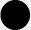	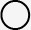	*sea+sak+scn*	*bbp* absent from UK1 and Irish isolates
	BK2421	SAMEA1029510	1	0	SCC [*mec* III (Delta *mecR*1-)+ Cd/Hg+*ccrC*] _(BK2421)_	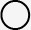	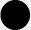	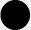	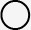	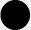	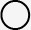	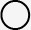	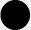	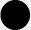	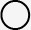	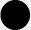	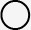	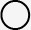	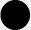	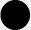	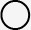	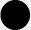	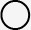	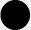	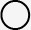	*sea+sak+scn*	-
**Australian/NZ**	**JKD6008**	CP002120.1	2	29	SCC [*mec* III+Cd] _(JKD6008)_	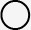	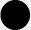	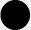	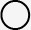	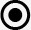	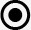	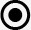	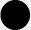	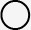	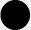	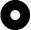	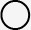	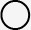	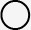	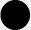	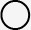	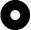	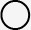	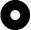	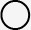	*sea+sak+scn* (var.); or none	*splA+B+E* variable
	**ACME-pos. variant of JKD6008**	–	0	3	SCC [*mec* III+ ACME II+Cd_]__(Unknown, ST239−1)_	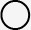	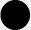	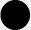	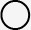	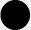	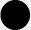	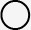	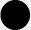	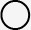	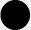	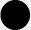	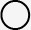	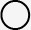	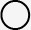	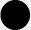	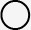	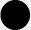	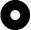	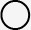	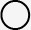	*sea+sak+scn*	ACME II present
**South-East Asian**	**TW20 (Irish AR44)**	GenBank FN433596.1	37	36	SCC [*mec* III+Cd/Hg+*ccrC*] _(TW20)_	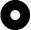	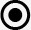	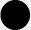	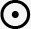	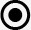	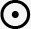	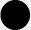	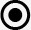	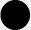	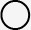	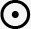	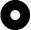	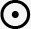	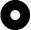	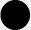	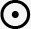	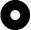	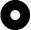	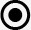	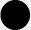	*sea+sak+scn* (comm.); *sak+chp+scn* (var.); *sak+scn* (rare) none (rare)	*splE* absent from Mideastern isolates and Syrian sequence M592, SAMEA862550
	**Strain with a TW20-derived pseudoelement**	GenBank JBMO	1	1	PseudoSCC*mec* [class A+Cd/Hg+*ccrC*] _(UCIM6015)_	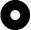	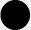	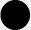	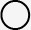	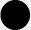	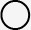	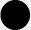	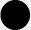	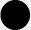	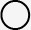	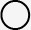	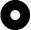	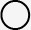	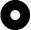	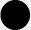	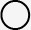	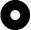	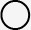	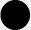	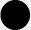	*sea+sak+scn* or none	–
	**Z172**	GenBank CP006838.1	1	1	SCC [*mec* III+Cd/Hg+*ccrC*] _(Z172)_	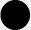	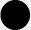	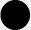	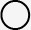	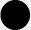	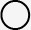	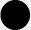	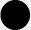	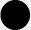	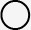	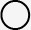	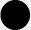	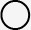	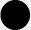	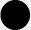	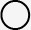	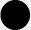	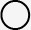	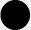	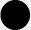	*sea+sak+scn* (var.); or none	*splE* variable
	**mvaS-deletion variant of Z1721**		0	1	SCC [*mec* III+Cd/Hg+*ccrC*] _(UnknownST239)_	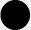	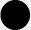	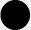	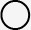	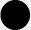	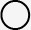	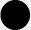	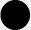	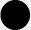	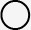	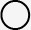	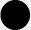	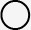	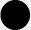	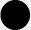	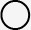	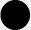	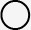	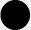	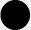	*sea+sak+scn*	–
	**XN108**	GenBank CP007447.1	4	9	SCC [*mec* III+Cd+*ccrC*] _(XN108)_	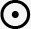	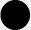	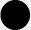	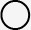	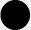	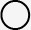	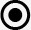	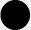	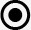	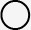	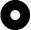	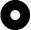	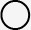	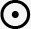	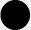	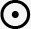	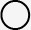	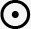	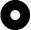	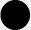	*sea+sak+scn* (var.); *sak+chp+scn* (rare); none (rare)	–
	**S85**	SAMEA1029529	2	3	SCC [*mec* III+Cd+*ccrC*] _(S85)_	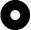	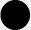	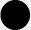	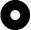	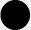	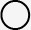	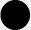	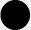	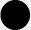	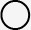	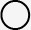	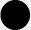	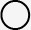	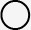	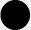	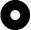	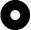	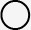	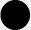	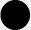	*sak+chp+scn*	–
	**PPUKM-775-2009-**	GenBank AMRE	1	1	SCC [*mec* III+Cd+*ccrC*] _(PPUKM−775−2009)_	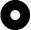	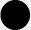	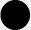	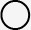	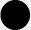	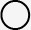	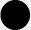	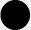	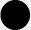	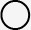	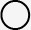	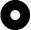	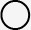	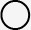	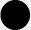	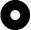	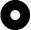	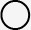	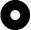	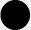	*sea+sak+scn* or *sak+chp+scn*	–
	***sasX*****-pos**. ***mer***+**/*****ccrC*** **neg. isolates from Kuwait**	–	0	2	SCC [*mec* III+Hg] _(Unknown, ST239)_	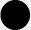	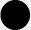	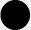	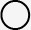	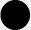	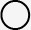	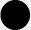	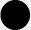	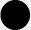	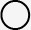	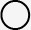	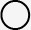	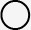	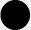	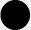	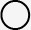	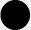	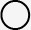	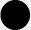	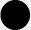	*sea+sak+scn* or none	–
	***sasX*****-pos. strain with a Bmb9393-like SCC element**	GenBank CP013957.1	18	17	SCC [*mec* III+Cd/Hg+*ccrC*] _(Bmb9393)_	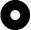	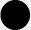	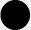	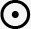	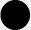	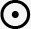	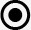	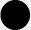	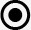	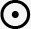	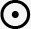	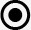	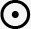	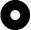	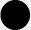	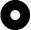	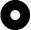	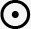	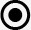	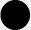	*sea+sak+scn* (var.); *sak+chp+scn* (rare); *sak+scn* (rare) none (rare)	–
	M418	GenBank CTXS	1	0	SCC [*mec* III+Cd/Hg+*ccrC*] _(M418/UK−9)_	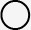	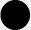	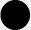	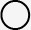	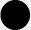	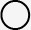	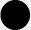	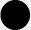	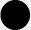	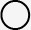	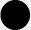	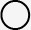	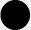	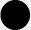	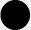	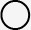	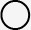	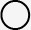	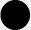	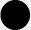	*sea+sak+scn*	–
	**NMR08**	GenBank CP023560.1	2	1	SCC [*mec* III+Cd+*ccrC*] _(NMR08)_	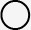	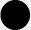	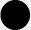	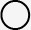	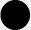	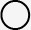	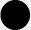	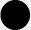	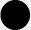	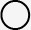	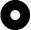	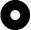	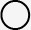	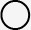	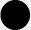	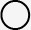	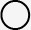	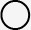	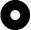	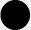	*sea+sak+scn*	–
	***sasX*****-pos. ACME-pos. strain**	–	0	1	SCC [*mec* III+ ACME II+Cd/Hg] _(Unknown, ST239)_	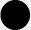	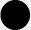	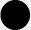	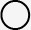	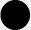	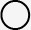	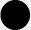	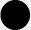	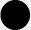	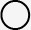	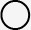	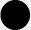	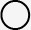	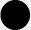	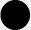	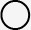	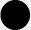	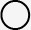	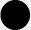	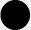	*sea+sak+scn*	ACME II present
	***sasX*****-pos*****/czrC*****-pos. strains**	–	0	5	SCC [*mec* III+speG+Cd/Hg/*czrC* +cc*rAB*4*C*] _(Unknown, ST239)_ SCC [*mec* III+*speG*+Cd/czrC+c*crAB*4*C*] _(Unknown, ST239)_	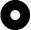	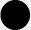	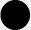	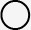	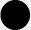	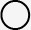	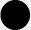	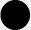	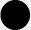	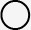	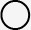	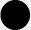	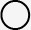	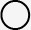	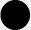	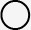	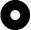	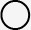	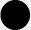	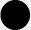	none	–
	***sasX*****-pos. sporadic strains from Kuwait and Hong Kong**		0	5	SCC [*mec* III+Cd/Hg] _(Unknown, ST239−1)_ SCC [*mec* III+Cd/Hg] _(Unknown, ST239−2)_	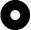	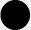	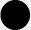	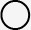	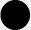	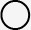	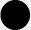	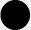	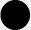	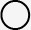	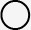	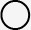	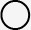	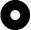	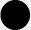	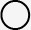	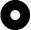	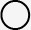	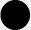	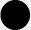	*sea+sak+chp+scn sea+sak+scn* or none	*ssl01* to *ssl05* absent in HK isolate
	P32	GenBank CTXV	1	0	SCC [*mec* III+Cd] _(HSA10/ATCC33592)_	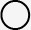	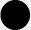	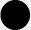	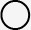	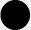	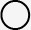	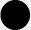	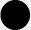	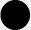	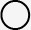	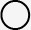	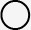	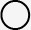	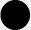	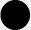	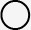	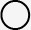	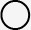	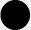	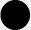	none	*sdrD* absent
	**ACME-pos. strain similar to P32**			1	SCC [*mec* III+ACME II+Cd] _(Unknown, ST239−2)_	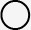	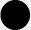	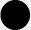	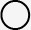	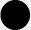	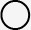	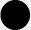	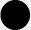	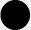	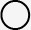	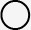	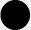	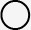	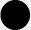	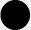	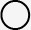	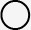	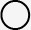	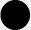	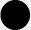	*sak+scn*	–
***hla-*****negative South-East Asian**	DEN907	SAMEA1029548	1	0	SCC [*mec* III+Cd/Hg] _(DEN907)_	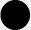	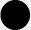	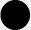	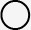	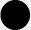	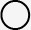	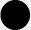	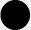	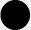	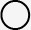	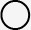	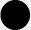	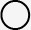	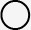	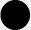	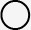	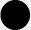	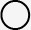	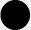	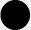	*sea+sak+scn*	***hla*** **absent**
	S2/M116	SAMEA1029563 GenBank CTXQ	12	0	SCC [*mec* III+Cd] _(S2)_	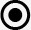	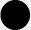	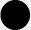	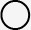	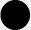	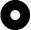	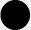	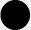	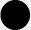	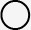	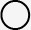	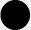	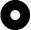	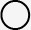	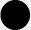	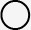	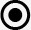	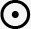	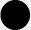	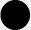	*sea+sak+scn*	***hla*** **absent**
**Portuguese**	**Portuguese & ATCC 33592 strain**	GenBank JXZH	8	5	SCC [*mec* III+Cd] _(HSA10/ATCC33592)_	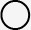	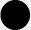	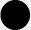	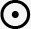	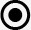	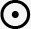	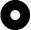	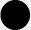	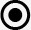	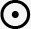	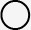	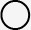	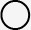	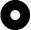	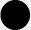	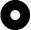	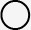	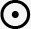	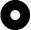	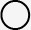	*sea+sak+scn* (rare); *sak+chp+scn* (rare); *sak+scn (var)* or none (var)	*spl* genes variably absent, *bbp* and *sdrD* sporadically absent
**South American/ Middle Eastern**	**Bmb9393/UK EMRSA-11**	GenBank CP005288.1	34	4	SCC [*mec* III+Cd/Hg+*ccrC*] _(Bmb9393)_	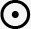	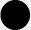	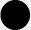	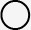	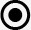	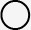	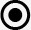	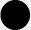	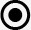	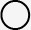	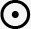	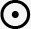	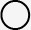	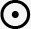	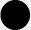	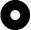	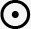	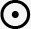	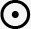	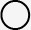		
	BRA2-Strain	SAMEA1029514	6	0	SCC [*mec* III+Cd/Hg] _(BRA2)_	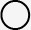	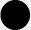	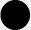	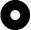	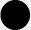	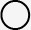	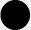	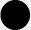	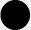	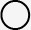	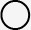	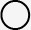	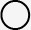	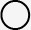	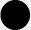	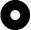	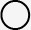	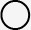	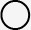	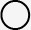	*sak+chp+scn* (comm.) or none	
	Strain with a BRA2-derived pseudoelement	GenBank MIHO	1	0	PseudoSCC*mec* [class A+Cd/Hg] _(UP1073)_	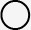	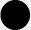	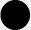	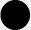	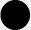	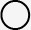	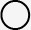	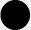	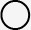	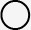	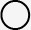	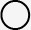	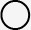	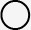	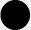	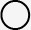	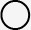	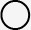	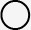	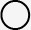	*sak+chp+scn*	
	**MRSA_PR1**	GenBank ANPO	2	3	SCC [*mec* III+Cd+ccrC] _(XN108)_	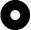	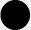	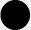	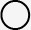	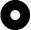	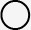	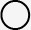	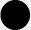	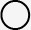	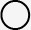	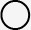	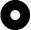	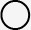	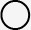	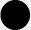	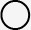	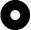	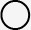	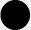	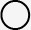	*sea+sak+scn* (var.); *sak+chp+scn* (var.)	*bbp* absent in MRSA_PR1 but present in others
	***sasX-*****negative strain with a S85-like SCC element**	–	0	2	SCC [*mec* III+Cd+*ccrC*] _(S85)_	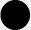	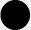	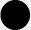	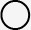	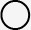	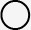	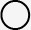	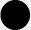	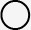	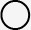	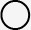	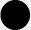	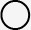	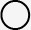	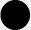	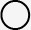	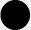	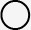	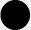	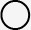	*sak+chp+scn*	*splE* absent
	**UK EMRSA-09**	–	0	1	SCC [*mec* III+Cd/Hg+ccrC] _(UK−EMRSA−9)_	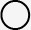	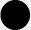	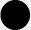	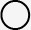	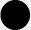	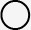	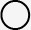	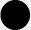	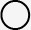	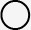	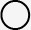	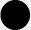	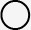	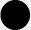	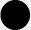	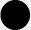	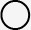	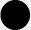	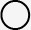	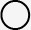	*sak+scn*	
	**Isolate from Togo**	–	0	1	SCC [*mec* III+Cd/Hg] _(Unknown, ST239−3)_	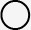	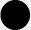	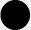	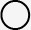	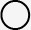	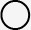	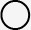	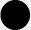	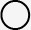	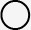	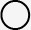	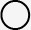	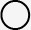	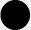	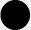	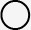	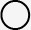	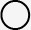	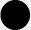	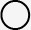	*sea+sak+scn*	
	**Irish AR09/ UK EMRSA-04 and**−**07**	GenBank AEEK	7	20	SCC [*mec* III+Cd/Hg+*ccrC*] _(TW20)_	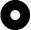	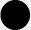	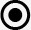	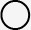	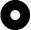	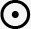	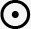	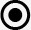	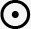	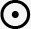	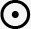	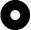	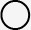	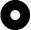	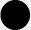	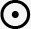	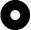	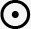	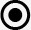	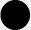	*sea+sak+scn* (var); *sak+chp+scn* (var.) *sea*_(N315)_*+sak+chp+ scn* (rare, Algeria only); none (rare)	*splE* variably absent, *ssl01* to *ssl06* absent in HK isolates
	**Irish AR23**	–	0	1	SCC [*mec* III+Hg+*ccrC*] _(AR23)_	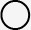	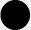	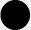	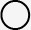	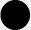	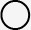	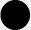	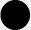	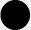	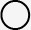	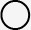	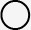	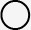	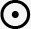	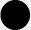	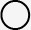	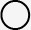	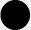	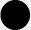	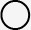	*sea+sak+scn*	
**Related to South American/ Middle Eastern**	**Krasnoyarsk** ***tst1***+ **Strain**	GenBank BBKC	1	4	SCC [*mec* III+Cd/Hg+*ccrC*] _(Bmb9393)_ with *mecA*_(BA000018)_	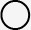	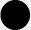	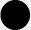	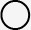	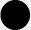	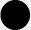	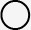	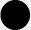	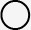	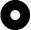	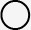	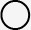	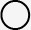	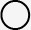	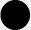	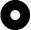	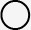	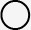	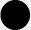	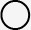	*sak+scn*	***tst1*** **positive**
	URU110	SAMEA1029540	1	0	SCC [*mec* III+Cd/Hg+*ccrC*] _(Bmb9393)_ with *mecA*_(BA000018)_	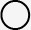	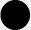	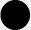	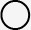	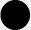	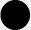	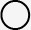	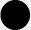	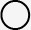	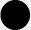	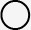	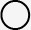	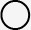	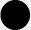	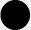	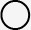	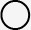	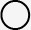	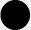	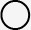	*sea+sak+scn*	
	DS_014	GenBank FQPU	1	0	SCC [*mec* III+Cd+*ccrC*] _(XN108)_ with *mecA*_(BA000018)_	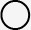	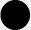	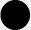	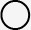	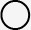	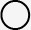	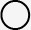	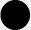	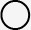	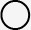	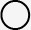	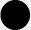	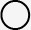	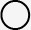	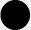	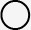	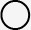	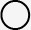	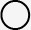	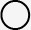	*sak+chp+scn*	
**Unassigned**	**Unassigned Middle Eastern Strain**	–	0	4	SCC [*mec* III+Cd+*ccrC*] _(Unknown, ST239−1)_	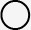	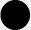	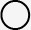	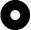	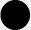	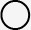	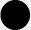	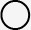	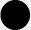	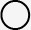	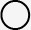	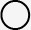	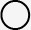	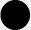	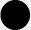	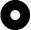	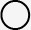	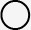	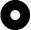	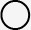	*sea+sak+scn*	***hla*** **absent**

*Bold font of element designations indicates those elements that were found in study isolates. Column “**PS**” indicates the number of (non-duplicate) previously ***p***ublished ***s***equences analyzed, the Column “**SI**” indicates the number of ***s***tudy ***i***solates. Symbols/abbreviations for frequencies of variable genes: 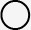, always absent; 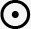, rare, i.e., present in < 20% of analyzed sequences and/or characterized isolates; 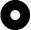, variable, i.e., present in 20–80%; 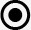, common, i.e., present in more than 80%; 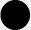, always present*.

Probe sequences were mapped on contigs using the program blastn (Camacho et al., 2009) from the NCBI blast+ suite and all sites were identified that matched the probe sequences with less than four mismatches. A signal value between 0 and 1 was assigned to each probe based on the actual number of mismatches derived from, and mimicking the normalized signals from a real hybridization experiment. A probe without mismatches was assigned signal intensity of 0.9; with 1 mismatch, a signal of 0.6; with two mismatches, a signal of 0.3; with three mismatches, a signal of 0.1. Probes with four—or more—mismatches were set as 0. These numerical values were then analyzed exactly as data from real hybridization experiments (Monecke et al., 2008b, 2011).

This approach has been developed and optimized based on real experiments performed with fully assembled strains (such as MSSA476, GenBank BX571857; N315, GenBank BA000018; COL, GenBank CP000046; MRSA252, GenBank BX571856; see also Monecke et al., 2016). For three strains (ATCC33592, UK-EMRSA-4, isolate Russia_0085) full genome sequences were available and we have done real as well as virtual hybridization experiments that were analyzed in parallel.

### Bioinformatics, analysis of insertions

Some CC239 genomes comprise a site-specific insertion of a mobile element into the chromosomale genes *nsaB* (locus tag SATW20_27600) or *yeeE* (locus tag SAT0131_RS10920). Two query sequences with a size of 80 nt were used to evaluate assembled genomes for the presents of uninterrupted *nasB* and *yeeE* genes. The two query sequences were choosen to span the insertion sites (for *nasB*, FN433596.1[2933542:2933621:r] and for *yeeE*, CP002643.1[2151962:2152041:r]). These query sequences were mapped on all full genome sequences with blastn. If they did not match for their full length, we assumed that the target gene was interrupted.

## Results

### Subtypes of SCC*mec* III in CC239-MRSA-III

Thirty-nine different variants of SCC*mec* III or SCC*mec* III-derived composite SCC elements or pseudoSCC-elements were observed in the 425 CC239-MRSA-III isolates and sequences. A description of these variants is provided in Tables 2a,b. Full profiles for individual isolates and sequences are shown in Supplemental Table 1.

Alleles of *mecA* in CC239-MRSA-III were assigned to two alleles matching the CC8-MRSA-I strain COL, CP000046 (among study isolates tested or sequenced, n = 21) and the CC5-MRSA-II strain N315, BA000018 (among study isolates, n = 38; one sequence not unambiguously assigned). When analyzing the binding sites of the probes used, the difference between the alleles is an “A” or, respectively, a “G” in position 737 (of the TW20 *mecA* sequence). Among the sequences and isolates investigated, *mecA* alleles largely correlate with the SCC*mec* subtypes and strains within CC239, i.e., a single *mecA* allele was found in association with each SCC*mec* subtype. However, there were five exceptions, *i.e*., SCC*mec* subtypes in which both *mecA* alleles were detected. This included the more common subtypes and strains (SCC [*mec* III+Cd/Hg+ccrC] _(TW20)_, SCC [*mec* III+Cd/Hg+ccrC] _(Bmb9393)_, SCC [*mec* III+Cd] _(S2)_, SCC [*mec* III+Cd+*ccrC*] _(XN108)_, SCC [*mec* III+Cd] _(HSA10/ATCC33592)_). This might suggest that the *mecA* sequence, and its allele assignment, is not a reliable phylogenetic marker but subject to random mutation (or to sequencing errors).

All SCC*mec* III elements from CC239 include a cadmium resistance operon for which *cadD*_(R35)_ was used as a marker.

Fifteen SCC*mec* III elements in CC239 (“Eurasian” strains with *ccrC* being located elsewhere not included) were composite elements that additionally harbor the recombinase gene *ccrC*. Sequence analysis identified two different *ccrC* alleles (Tables 1, 2a,b) that could not be differentiated with the current set of probes. Genes accompanying *ccrC* are *ccrAA* (although the present allele yields usually only ambiguous signals with the probes used herein) and D1GU38. Nineteen SCC*mec* III elements in CC239 also include the mercury resistance operon; this is often but not always linked to *ccrC*.

Composite elements that include ACME II (that is, *arc* genes present but *opp* genes absent), an arsenic resistance operon, genes *speG* and *czrC* (zinc/cadmium resistance) were occasionally found (see Tables 2a,b). No isolates were identified that harbored composite elements involving ACME I (*arc* and *opp* genes), ACME III (*opp* genes only) or SCC*fus* (fusidic acid resistance, *fusC*).

The presence of *dcs* and a SCC terminus sequence or multiple SCC terminus sequences suggests the presence of composite elements. Sequence analyses has shown that SCC terminus sequences are not necessarily situated “terminally” toward *orfX* but can be found within a composite element demarking it components. For example in TW20, SCCterm02 (GenBank FN433596.1; positions 34,140 to 34,456) is situated toward *orfX* (positions 33,660 to 34,139) while SCCterm01 can be found between the region including the mercury resistance operon and the SCC*mec* element (positions 67,191 to 67,511). As described below, SCCterm02 can also be associated with integration of a transposon into the *nsaB* gene, *i.e.*, at a distant position from *orfX*.

### Strains and clades of CC239-MRSA-III

Harris and Castillo-Ramírez (Harris et al., 2010; Castillo-Ramírez et al., 2012) divided CC239-MRSA-III into several clades based on SNP analysis. We re-analyzed published sequences from this project, other previously published sequences as well as our own sequences and array hybridization patterns regarding presence of SCC*mec* III subtypes (see above), the gene *sasX/sesI* (see below), the enterotoxin genes *sek* and *seq* (representative for *S. aureus* pathogenicity island 3), certain resistance markers and regarding other conspicuous features such as *spa* types or deletions of individual genes.

Individual results as well as clade/strain assignments for each isolate and sequence are listed in the Supplemental Table 1. A list of strains and clades as well as of their genotypic features is provided in Table 3. Strain definitions are mainly based on SCC*mec* subtypes plus other notable features.

#### The “Eurasian Clade” and the insertion into the *nsaB* gene

Harris and Castillo-Ramírez describe a distinct and rather homogenous “Turkish Clade” (Harris et al., 2010; Castillo-Ramírez et al., 2012). We found that this clade was not restricted to Turkey. It also included isolates and sequences (including T0131, CN79, 16K, 3HK and others) that originate from Eastern Europe and the Balkans, Russia, Pakistan as well as from China (mainly Northern China, but also Hong Kong). Thus we suggest renaming this clade “Eurasian Clade.”

Isolates and sequences assigned to this clade are characterized by the presence of *mecA*_(BA000018)_ and *dcs*. Harris' strains TUR1 and TUR9 differ in this regard being an intermediate to the “European Clade” or a product of a horizontal gene transfer of another variant of a SCC*mec* III element.

Furthermore, “Eurasian Clade” strains are characterized by integration of a IS431 based transposon—carrying several genes that appear to origin from the SCC*mec* III element—into the *nsaB* gene which is located 140,000 nt away from the SCC element (T0131, GenBank CP002643: position 2,779,896 to 2,803,726). This transposon consists of *ccrC, ccrAA*, SCCterm02 and additional genes such as *erm*(A)*, ant9, hsdR*2-WIS, D1GU60, A9UFT0, Q93IA1, A5INT3, Q9KX75, Q0P7G0, Q93IE0, Q3T2M7, Q4LAG3, D2N370, D1GU38, Q2FKL3, transposase genes and IS431 sequences as well as *aacA-aphD* (absent from T0131, but present in 16K; Yamamoto et al., 2012). This disruption of the *nsaB* gene was described first in isolates from Romania (Chen et al., 2010) and Russia (Yamamoto et al., 2012) but it can be detected in all “Eurasian Clade” sequences. A insertion into *nsaB* is also present in TUR1, SAMEA1029552 and TUR9, SAMEA985415 but due to fragmentation of the sequences into a high number of contigs, the gene content of their insertion cannot be reliably determined.

All other sequences and clades of CC239-MRSA-III present with an un-truncated, wildtype *nsaB* gene. This includes, despite their similarity to the “Eurasian Clade”, JKD6008 and related strains from Australia/New Zealand (see below). However, *erm*(A) and *ant9* are not restricted to the insertion into *nsaB*. These two genes can frequently be found in CC239-MRSA strains without the *nsaB* insertion, where they are present in different localizations. In the previously published genome sequence Bmb9393, a transposon carrying these two genes disrupts *radC* (SABB_05268) while in TW20, this transposon is present twice, once integrated into *radC* and once co-localized with the SCC*mec* III element. In JKD6008, there are also two copies of this transposon, one within *radC* (SAA6008_01621) and one disrupting *ywqG* (SAA6008_00825). Therefore the detection of *erm*(A) and *ant9* cannot be used as a surrogate marker for the identification of the “Eurasian Clade” but the insertion into *nsaB* can.

Other features of the “Eurasian Clade” include a predominance of RIDOM *spa* type t030 (with all isolates previously assigned to *spa* type t030 belonging to this clade; Gostev et al., 2017) or t632 (Moscow and Saint Petersburg), the uniform absence of the mercury resistance operon, the adhesion factor gene *fnbB* and the protease gene *splE* while *splA* and *splB* are present.

#### The “European Clade”

The basal “European Clade” (Harris et al., 2010) consists of isolates and sequences that share the SCC [*mec* III+Cd/Hg+*ccrC*] _(SK1585)_ element and variants thereof from which some genes are fully (*mvaS*) or partially (*mecR1*) deleted. This particular SCC*mec* element is a composite SCC*mec* III/heavy metal resistance element that was first observed in a strain isolated in Australia as early as in 1973 (see Nimmo et al., 2015 and section Discussions).

The “European Clade” includes a cluster of homogenous sequences and isolates from, or with epidemiological connection to, Greece. These include Harris' Greek sequences (Harris et al., 2010), isolates from Saxony/Germany epidemiologically linked to Greece (see below and Albrecht et al., 2011) and the Greek reference strain from the Harmony collection (Greece 1_3680). It also includes two isolates from Morocco.

Some “European Clade” strains have a characteristic deletion of 166 nt (in 85/2082, GenBank AB037671.1; corresponding the region in TW20 of FN433596.1 [79030 to 79195]) in the *mecR1* gene that results in the paradoxical observation that the probe associated with *mecR1* yields a signal while the one for Δ*mecR1* does not. These include genome sequences from Australia and the US (ANS46, LHH1, BK2421) as well as epidemic strains British UK-MRSA-01 and Irish AR01.

#### The “South-East Asian Clade” and the *sasX/sesI* gene

The sequences assigned by Harris and Castillo-Ramírez (Harris et al., 2010; Castillo-Ramírez et al., 2012) to the (South-East) “Asian Clade” all contain the *sasX/sesI* gene which was absent from all sequences not assigned to this clade. Consequently, *sasX/sesI* was used in the present study as an identifying marker for this clade. In all *sasX/sesI*-positive CC239 sequences analyzed, the *sasX/sesI* prophage is localized in the same position within the genome, splitting *yeeE* (FN433596.1; positions 2180899 to 2181684 downstream of the phage insertion, and 2308889 to 2309177 upstream). This, and the observation that the entire cluster appeared in previous sequencing studies to be monophyletic (Harris et al., 2010; Castillo-Ramírez et al., 2012) suggest that the “South-East Asian Clade” is one distinct lineage resulting from one single acquisition of the *sasX/sesI* prophage. Differences affecting mobile genetic elements (including SCC*mec*) and the presence of the alpha haemolysin gene *hla* (which is absent from several sequences, mainly from the Middle East and Thailand) could then be considered secondary.

SCC*mec* elements in the “South-East Asian Clade” are complex composite elements consisting of SCC*mec* III [usually, but not always, with *mecA*_(CP000046)_], *ccrC, cadD* as well as of SCCterm01 and/or 02 sequences. The mercury resistance operon is nearly always present, and the few exceptions might be regarded as secondary deletions.

Isolates and sequences originate from South-East Asia, including Hong Kong and Southern China, India, Australia, the Middle East, Western Europe and, surprisingly, Trinidad & Tobago. The clade comprises also TW20 (NCTC13626), AUS-EMRSA-3 and Harmony Collection Finland E24_98541.

#### The “South American/Middle Eastern Clade”

Furthermore, there is a large clade encompassing a wide variety of isolates and sequences and that show identical or very similar SCC*mec* types as the “South-East Asian Clade” (Table 2b) but that are *sasX/sesI*-negative. This includes the “South American Clade” sequences (Harris et al., 2010; Castillo-Ramírez et al., 2012) with three different SCC*mec* subtypes (SCC [*mec* III+Cd/Hg+*ccrC*] _(Bmb9393)_, SCC [*mec* III+Cd/Hg] _(BRA2)_ without *ccrC* and associated genes and pseudoSCC*mec* [class A+Cd/Hg] _(UP1073)_ without any *ccr* genes). However, there are also isolates and sequences that originate mainly from the Middle East, but also from Europe and Russia, and that have identical or similar SCC*mec* types. Hence, we referred to this clade herein as the “South American/Middle Eastern Clade.” This clade includes Bmb9393, ATCC BAA-39, NCTC13131, UK-EMRSA-4, UK-EMRSA-7, UK-EMRSA-9 and UK-EMRSA-11, Irish AR09 and AR23 and the unique *tst1*-positive strain from Krasnoyarsk, Russia.

#### Other clades

Furthermore, there are additional geographically restricted clades and strains that do not fit into the larger clades as defined by Harris and Castillo-Ramírez (Harris et al., 2010; Castillo-Ramírez et al., 2012).

One group of sequences and isolates could be named the “Australian/New Zealand Clade” consisting of a number of isolates from Australia and New Zealand with JKD6009 and JKD6008 being representative genome sequences. Isolates lacked *sasX/sesI*, the *mer* operon and *ccrC*. They carried *mecA*_(BA000018)_. Similar to the “Eurasian Clade” strains, they harbored *dcs* but they differed in the absence of the secondary SCC-like gene cluster inserted into *nsaB*.

Another clade comprises a very homogenous cluster of Portuguese genome sequences from Harris' work and a similar strain, ATCC 33592, from New York City. The isolates and sequences lack *sasX/sesI, dcs, ccrC*, the mercury resistance operon and an integration into *nsaB*, but harbor SCCterm01.

We also observed a cluster of isolates from the Middle East, Libya and Russia that did not match any published sequences. These isolates carried *mecA*_(BA000018)_ and *ccrC* but lacked the mercury resistance operon, the *nsaB* integration, *sasX/sesI* and *hla*.

### Isolates by geographic origin

An overview on geographic origins by clade and strain is provided in Table 4. In the following paragraph a short summary by countries and different sampling sites is given.

**Table 4:** Clades and strains of CC239-MRSA-III, geographic origin of isolates and sequences, GenBank and BioSample accession numbers.

**Clade**	**Strain/Isolate**	**Origin of study isolates**	**Origin of previously published sequences**	**Fully assembled genome sequences (GenBank)**	**WGS (Whole Genome Shotgun) genomes (Prefixes)**	**BioSample accession numbers of self-assembled genomes**
**Eurasian**	TUR1	–	Turkey	–	–	SAMEA1029552,−985415
	**T0131**	Romania (1)	Turkey, China	CP002643.1	CTYE, CTYI, CTYV	–
	***arsC*****-SCC-pos. strains from Romania**	Romania (2)	–	–	–	–
	**16K/CN79**	Germany (from Turkey; 1), Germany (from Makedonia; 1), Germany (1), Romania (7); Russia (Moscow; 5); Russia (Saint Pietersburg; 4); Russia (Chelyabinsk; 2); Russia (Kurgan; 2); Pakistan (5), China (Hong Kong; 2)	Czech Republic, Hungary, Romania, Russia, Turkey, Pakistan, China		ANCJ, BABZ, AZMY, AZMX, CTWB, CTWC, CTWD, CTWE, CTWF, CTWG, CTWH, CTWI, CTWJ, CTWK, CTWL, CTWM, CTWN, CTWO, CTWP, CTWQ, CTWT, CTWX, CTXD, CTXF, CTXJ, CTXK, CTYB, CTYF, CTYH, CTYJ, CTYK, CTYL, CTYM, CTYN, CTYO, CTYP, CTYR, CTYS, CTYT, CTYU, CTYW, CTYX, CTYY, CTYZ, CTZC, CTZF, CTZG, CTZI, CTZJ, CTZK, CTZM, CTZO, CTZP, JTJX	SAMEA1029551, −1029557, −1029566, −1029567
	IU17		Turkey	–	CTXB, CTXE	–
	**Sporadic strains from Saxony**	Germany (from Middle East; 1), Germany (1)	–	–	–	–
	**Kuwaiti ACME II-pos. Strain**	Kuwait (2)	–	–	–	–
**European**	**“Greek Epidemic Strain”**	Germany (from Greece, 4), Greece (Harmony collection; 1), Morocco (2)	Greece, USA (R35)	KL662257.1 (SCC*mec* only)	–	SAMEA1029513, −1029532, −1029546, −1029564
	**UK-01/Irish AR01 Epidemic Strain**	Ireland (4), UK (NCTC11939; 1)	Australia, USA	AB037671.1 (SCC*mec* only)	–	SAMEA1029537,−1029523
	BK2421	–	USA	–	–	SAMEA1029510
**Australian/NZ**	**JKD6008-Strain**	Australia (31)	Australia, New Zealand	CP002120.1	ABSA	–
	**ACME-pos. variant of JKD6008-Strain**	Australia (3)	–	–	–	–
**South-East Asian**	**TW20 (Irish AR44)**	Ireland (2), Kuwait (3), Saudi Arabia (3), China (Hong Kong; 9), Australia (6), Uganda (1), Trinidad&Tobago (13)	UK, Germany, Denmark, Syria, Sri Lanka, India, Thailand, Malaysia, China (Nanjing), China (Hong Kong), USA	FN433596.1, CP015447.1	AZJQ, CTWW, CTWZ, CTXH, CTXM, CTXN, CTXT, CTXU, CTXZ, CTYD, FFPZ, FGZX, FQJD, FQJP, JBEK, JBGY, JBNV, JBOE, JBPU, JBRH, JBSL, JVZJ, JVZK, JVZL, LWAL	SAMEA1029512, −1029516, −1029519, −1029524, −1029528, −1029538, −1029541, −1029560, SAMN04903357
	**Strain with a TW20-derived pseudoelement**	China (Hong Kong; 1)	N/A	–	JBMO	–
	**Z172**	Russia (Kurgan, 1)	Taiwan	CP006838.1	–	–
	***mvaS*****-deletion variant of Z1721**	Australia (1)	–	–	–	–
	**XN108**	Germany (from India; 1), Saudi Arabia (1), India (3), China (Hong Kong; 3), Trinidad&Tobago (1)	India, China (Chongqing), Thailand, Malaysia	CP007447.1	FQFW, JQNU, LWBY	
	**S85**	Saudi Arabia (3)	Thailand	–	CTWU	SAMEA1029529
	**PPUKM-775-2009**	China (Hong Kong; 1)	Malaysia	–	AMRE	
	***sasX*****-pos**. ***mer***+**/*****ccrC*** **neg. isolates from Kuwait**	Kuwait (2)	–	–	–	–
	***sasX*****-pos. strain with a Bmb9393-like SCC element (AUS-EMRSA-3)**	Finland (Harmony collection; 1), Kuwait (1), Saudi Arabia (1), India (1), Pakistan (1), China (Hong Kong; 7), Australia (5), Trinidad&Tobago (1)	Lebanon, India, Hong Kong, Korea, Thailand, Malaysia	CP013957.1	AHZL, AICJ, AMRB, AMRC, AVPR, AZMZ, CTWV, CTXL, CVRW, FGBR, FQJI, FQJW, LWAK, MLQB, NESW	SAMEA1029556,−1029568
	M418	–	India	–	CTXS	–
	**NMR08**	India (1), China (Hong Kong; 1)	India	CP023560.1	LWAM	
	***sasX*****-pos. ACME-pos. strain**	Australia (1)	–	–	–	–
	***sasX*****-pos*****/czrC*****-pos. strains**	Trinidad&Tobago (5)	–	–	–	–
	***sasX*****-pos. sporadic strains from Kuwait and Hongkong**	Kuwait (4), China (Hong Kong; 1)	–	–	–	–
	P32	–	Poland	–	CTXV	–
	**ACME-pos. strain similar to P32**	Australia (1)	–	–	–	–
***hla-*****negative South-East Asian**	DEN907	–	Denmark	–	–	SAMEA1029548
	S2/M116	–	Thailand, Vietnam	–	CTXQ	SAMEA1029511, −1029515, −1029522, −1029525, −1029535, −1029536, −1029547, −1029553, −1029558, −1029563, −1029565
**Portuguese**	**Portuguese & ATCC 33592 strain**	Russia (Chelyabinsk;1), Russia (Kurgan; 3) USA (ATCC 33592)	Portugal, USA	–	JXZH	SAMEA1029520, −1029533, −1029543, −1029545, −1029561, −1029562, −1029542
**South American/ Middle Eastern**	**Bmb9393/UK EMRSA-11**	UK (UK EMRSA-11; 1), Germany (from Middle East; 1), Ecuador (2)	UK, Germany, Denmark, France, Spain, Portugal, Czech Republic, Lithuania, Brazil, Uruguay, Argentina	CP005288.1, CP009681.1, CP012011.1, CP012012.1, CP012013.1, CP012015.1, CP012018.1	AHVC, AICH, CTXC, CTXG, CTXI, CTXO, CTXP, CTXW, CTXX, CTXY, CTYA, CTYC, CTYG, LDPC, LGWS	SAMEA1029508, −1029509,−1029517, −1029527, −1029534, −1029544, −1029549, −1029550, −1029554, −1029559, −1029569, −985416
	BRA2-Strain	–	Brazil, Chile, Peru	–	LGWM, LGWY, LGWZ	SAMEA1029514, −1029526, −1029539
	Strain with a BRA2-derived pseudoelement	–	Peru	–	MIHO	–
	**MRSA_PR1**	Saudi Arabia (3)	Malaysia, Lithuania		ANPO, CTXR	–
	***sasX-*****negative strain with a S85-like SCC element**	Saudi Arabia (2)	–	–	–	–
	**UK EMRSA-09**	UK (UK EMRSA-09)	–	–	–	–
	**Isolate from Togo**	Togo (1)	–	–	–	–
	**Irish AR09/ UK EMRSA-04 and**−**07**	UK (UK EMRSA-04 and−07), Ireland (3), Russia (Krasnoyarsk; 1), Saudi Arabia (9), Kuwait (1), Algeria (1), China (Hong Kong; 1), Australia (1)	UK, Denmark, Hungary, Egypt		AEEK, CTWR, CTWS, FFVG	SAMEA1029518, −1029531, −3529263
	**Irish AR23**	Ireland (1)	–	–	–	–
**Related to South American/ Middle Eastern**	**Krasnoyarsk** ***tst1***+ **Strain**	Russia (Krasnoyarsk; 4)	Russia	–	BBKC	–
	URU110	–	Uruguay	–		SAMEA1029540
	DS_014	–	Thailand	–	FQPU	–
**Unassigned**	**Unassigned Middle Eastern Strain**	Germany (from Libya, 1), Saudi Arabia (1), Kuwait (1), Russia (Moscow; 1)	–	–	–	–

*Bold font indicates that isolates belonging to those strains have been found in the study*.

#### African countries

Although CC239-MRSA-III has been observed in several African contries (Jansen van Rensburg et al., 2011; Abdulgader et al., 2015), there are insufficient data on epidemiological trends or molecular epidemiology.

Six isolates from five different African countries were included in the study. One isolate from Algeria was a “South American/Middle Eastern Clade” strain carrying SCC*mec* III+Cd/Hg+*ccrC*_(TW20)_, and was identical to Irish AR09. One isolate from a Libyan patient (who was brought to North-Eastern Germany for humanitarian aid) belonged to the unassigned “Middle Eastern” strain. Two isolates from Morocco matched the “European Clade”, being most similar to the “Greek Strain” but differed from the other isolates in the presence of *sek/seq* and the absence of *cadX, erm*(C) and *tet*(M). One isolate from Togo matched the “South American/Middle Eastern Clade” but harbored an unique SCC*mec* subtype [designated SCC*mec* III+Cd/Hg _(Unknown, ST239−3)_ in Table 2b]. This isolate might be a derivative of UK EMRSA-9 that has lost *ccrC* and accompanying genes. One isolate from Uganda (Monecke et al., 2013a) was *sasX/sesI*-positive and belonged to the “South-East Asian Clade.”

#### Australia

CC239-MRSA-III have been present in Australia for decades (Dubin et al., 1991; Coombs et al., 2004; Howden et al., 2010; Nimmo et al., 2015) with distinct variants (Aus-2 EMRSA and Aus-3 EMRSA) being distinguished based on mercury susceptibility or resistance, respectively (Coombs et al., 2006).

Eighteen Australian isolates were genotyped by microarray, and Ilumina NGS sequences of an additional 30 isolates were analyzed. The majority of isolates (*n* = 31) were very similar to JKD6008, GenBank CP002120.1, and JKD6009, GenBank ABSA from New Zealand and Australia, respectively, forming a distinct “Australian/New Zealand (NZ) Clade” (correlating to the mercury susceptible “Aus-2 EMRSA”). Three isolates harbored an ACME II cluster as well as Q9S0M4 and yielded additional signals for SCCterm03 and 05. The isolates might be regarded as direct derivatives of the JKD6008/6009 strain.

Thirteen isolates belonged to the “South-East Asian Clade” possibly indicating foreign importation which, given the geographic links between Australia and Asia, seems likely. Interestingly, two of these isolates also harbored the ACME II cluster. One isolate matched the Middle Eastern/Irish AR09 strain. One previously published Australian CC239 genome sequence, ANS46 (SAMEA1029537) belonged to the “European Clade,” UK-01/AR01.

The SCC*mec* element of the “Greek Strain,” SCC [*mec* III+Cd/Hg+*ccrC*] _(SK1585)_, was also previously observed in Australia, in the chimeric strain, ST2249-MRSA-III, SK1585 (GenBank AYLT and KL662257.1) (Nimmo et al., 2015).

#### China/Hong Kong

CC239-MRSA-III has been epidemic in China for decades. In Hong Kong, a presence of the “South-East Asian Clade” was reported while in Northern China, “Eurasian Clade” strains emerge and spread (Ip et al., 2005; Chen et al., 2010, 2014a; Wang et al., 2014).

Twenty-seven isolates originated from Hong Kong. The majority (*n* = 23) belonged to the “South-East Asian Clade” with the most common strains carrying SCC [*mec* III+Cd/Hg+*ccrC*] _(TW20)_ or SCC [*mec* III+Cd/Hg+*ccrC*] _(Bmb9393)_ (nine and seven isolates, respectively). Another isolate had a SCC [*mec* III+Cd/Hg+*ccrC*] _(TW20)_-derived pseudoSCC*mec* element. Two isolates belonged to the “Eurasian Clade”, likely to indicate influx from mainland China (Wang et al., 2014) and two isolates were assigned to the “South American/Middle Eastern Clade.”

#### Ecuador

In Ecuador, 2005-2013, CC239-MRSA-III was the second most common MRSA strain (Zurita et al., 2016) and contrarily to other Latin American countries it is still common there (Arias et al., 2017).

Two isolates were assigned to the “South American/Middle Eastern Clade” being *sasX/sesI* negative while carrying SCC [*mec* III+Cd/Hg+*ccrC*] _(Bmb9393)._

#### Germany/Saxony

CC239-MRSA-III is not an epidemic strain in the German state of Saxony, or at least it has not been since 2000 and many cases are related to foreign travel (Albrecht et al., 2011).

Eleven isolates were included into the study. Nine of them were obtained from patients with known travel history (including admission to foreign healthcare facilities), with nosocomial contact to travelers, or from immigrant patients.

Five isolates were assigned to the “Eurasian Clade.” Two of them were obtained from Macedonian and Turkish nationals, respectively, the latter with history of hospitalization in Turkey after trauma. A third patient with an “Eurasian Clade” isolate appeared to have a Middle Eastern background while for two remaining cases, no history of immigration or travel was known. The “Greek Strain” CC239-MRSA-[III+Cd/Hg+*ccrC*] _(SK1585)_ was found in four outbreak isolates, with an index patient who was repatriated from Greece after trauma and emergency care (Albrecht et al., 2011). One patient with a “South American/Middle Eastern Clade” strain had a Middle Eastern background indeed. Finally, one isolate from a patient of Indian background belonged to the “South-East Asian Clade” and matched the SCC*mec* element of XN108.

#### India

CC239-MRSA-III appear to be common and widespread in India; and although other strains emerged meanwhile, it is, at least regionally, still a dominant MRSA strain (D'Souza et al., 2010; Abimanyu et al., 2012; Neetu and Murugan, 2016).

In addition to one isolate from an Indian patient in Saxony (see above), five isolates with an Indian background were tested. All belonged to the “South-East Asian Clade”; three matched genome sequence of XN108 and its SCC*mec* subtype, and one the Indian genome sequences of NMR07/08. A fifth isolate was *sasX/sesI* positive but had a Bmb-9393-like SCC*mec* element.

#### Ireland

CC239-MRSA-III predominated in Irish hospitals in the mid-to-late 1980s [locally known as phenotype III and antibiogram-resistogram (AR) types 01 and 09] but has since only been recovered sporadically or as part of localized outbreaks, represented by AR15 and AR23 isolates recovered in 1992/93 and AR44 recovered in 2002 (Carroll et al., 1989; Rossney et al., 1994; Shore et al., 2005). The 10 Irish ST239-MRSA-III isolates investigated clustered into three clades and four strains.

Firstly, AR01/AR15 isolates with a distinct truncation of *mecR1* matched Harris' “Basal/European Clade,” being identical to sequences of Ans46 and LHH1 (from the US and Australia) as well as to UK-EMRSA-1.

Secondly, AR09/Phenotype III isolates harbored SCC [*mec* III+Cd/Hg+*ccrC*] _(TW20)_, lacked *sasX/sesI* and matched Middle Eastern isolates. This fits to the observation that this strain was first brought to Ireland with an oil worker who was repatriated from Iraq in 1985 with a subsequent major outbreak (Humphreys et al., 1990).

AR23 could be considered a variant of the AR09 strain that was *cadD*-negative.

Thirdly, AR44 harbored SCC [*mec* III+Cd/Hg+*ccrC*] _(TW20)_ and were *sasX/sesI*-positive. It has been suggested previously that this strain was imported from Singapore (Rossney, 2003), and indeed that this variant predominates in South-East Asia. This outbreak was contained and did not spread beyond one unit (Shore et al., 2005).

#### Kuwait

In Kuwait, CC239-MRSA-III accounted for more than 50% of typed MRSA isolates collected in a period from 1992 to 2010 (Boswihi et al., 2016). Currently, it is still present although it appears to be replaced by community-acquired MRSA strains (Udo and Al-Sweih, 2017).

Fourteen Kuwaiti isolates belonged to nine different strains and were assigned to the “Eurasian,” “South American/Middle Eastern,” and the “South-East Asian” clades. Two isolates of the “Eurasian Clade” harbored ACME II elements thus differing from all other isolates of that clade. One was identical to the Irish AR09 outbreak strain that was reported to originate from the Middle East (see above and Humphreys et al., 1990). Three “South-East Asian Clade” isolates were essentially identical to TW20 and one had a Bmb9393-like SCC*mec* element. Six others represented sporadic variants that were characterized by a loss of *ccrC* although the usually accompanying D1GU38 was present. One isolate belonged to an unassigned strain that was found mainly in the Middle East.

#### Pakistan

There are few studies on genotyping of MRSA from Pakistan indicating a presence of CC239-MRSA-III in hospitals (Shabir et al., 2010; Zafar et al., 2011; Arfat, 2013; Jamil et al., 2017) but its absence in the community.

Five Pakistani isolates from Rawalpindi (as well as the one previously published genome sequence from Pakistan, NCTR #32S, GenBank JTJX) belonged to the “Eurasian Clade”, CN79/16K-Strain. One isolate was assigned to “South-East Asian Clade” strain with a BMB939-like SCC*mec* element.

#### Romania

CC239-MRSA-III matching the “Eurasian Clade” has been reported to be common in Romania after 2000 (Cirlan et al., 2005; Chen et al., 2010; Monecke et al., 2014a).

All 10 Romanian isolates included, as well as previously published sequences, clustered into the “Eurasian Clade”, lacking *sasX/sesI* but carrying *mecA*_(BA000018)_, *dcs* as well as SCCterm02 (indicating the secondary SCC-like gene cluster inserted into *nsaB*). Seven matched genome sequences 16K and CN79, and one T0131. Two isolates had unsequenced composite SCC*mec* elements including the arsenic resistance gene *arsC* that might be regarded as variants of 16K/CN79- and T0131-like elements, respectively.

#### Russia

After 2000, CC239-MRSA-III was found to be common in different regions across Russia, not only in hospitals but also in the community (Afanas'ev et al., 2010; Baranovich et al., 2010; Yamamoto et al., 2012; Khokhlova et al., 2015; Gostev et al., 2017). Previous reports (Afanas'ev et al., 2010; Baranovich et al., 2010; Yamamoto et al., 2012; Gostev et al., 2017) and sequences (16 K) indicated a presence of the “Eurasian Clade” as well as of other variants (Gostev et al., 2017). Besides, there was a notable emergence of a distinct *tst1*-positive variant in the city of Krasnoyarsk (Khokhlova et al., 2015).

The majority of Russian isolates (13/24), from Moscow, Saint Petersburg, Kurgan and Chelyabinsk, matched the “Eurasian Clade” and genome sequences CN79 and 16 K. However, three isolates from Saint Petersburg differed from the others in the presence of the *sea*_(N315)_/*sep* allele. Four isolates from Krasnoyarsk represent a local epidemic strain harboring *tst1* and SCC [*mec* III+Cd/Hg+*ccrC*] _(Bmb9393)._ Isolates were identical (although one isolate lacked presumably plasmid-borne *cat*, encoding chloramphenicol resistance) to the genome sequence of MRSA-OC3, GenBank BBKC, SAMD00019145 which also originated from this town. The four isolates from Kurgan and Chelyabinsk were essentially identical to ATCC 33592 (representing a clade previously known from Portugal and the USA). One isolate from Kurgan was identical to the Taiwanese genome sequence Z172 (SAMN02370325). One isolate from Krasnoyarsk belonged to the “South American/Middle Eastern Clade” and one isolate from Moscow matched the unassigned strain that was otherwise found in the Middle East.

#### Saudi Arabia

Although CC239-MRSA-III is known to be present in the Middle Eastern/Gulf region for decades (Humphreys et al., 1990), molecular data confirming a presence of CC239-MRSA-III in the Kingdom of Saudi Arabia have been published only in recent years (Cirlan et al., 2005; Al-Obeid et al., 2010; Monecke et al., 2012c; Senok et al., 2016), and differences in carriage of *ccrC, merA/B* and aminoglycoside resistance genes indicated a simultaneous existence of different variants of this strain (Monecke et al., 2012c).

Twenty-three isolates from two different hospitals in Riyadh were characterized. Fourteen were assigned to the “South American/Middle Eastern Clade”, nine had SCC [*mec* III+Cd/Hg+*ccrC*] _(TW20)_ thus matching Irish AR09 (see above and Humphreys et al., 1990). However, the 14 contemporary Saudi Arabian isolates lacked *splE*. As isolates were obtained from two hospitals in one city this might indicate a recent outbreak situation. The differences in SCC*mec* subtypes (absence of the *mer* operon resulting in SCC [*mec* III+Cd+*ccrC*] _(S85)_ and of *mer*, SCCterm01 and Q93IB7 resulting in SCC [*mec* III+Cd+*ccrC*] _(XN108)_) would then only be secondary to the loss of *splE*.

Another eight isolates belonged to the “South-East Asian Clade” (being also all *splE*-negative) and one belonged to the unassigned “Middle Eastern Strain.”

#### Trinidad & Tobago

CC239-MRSA-III has been the dominant MRSA clone in Trinidad & Tobago at least from 2000/01 (Akpaka et al., 2007) until 2012/2013 (Monecke et al., 2012b, 2014b).

Twenty-one isolates from Trinidad & Tobago where characterized. All were *sasX/sesI*-positive. This observation places CC239 from Trinidad & Tobago into the “South-East Asian Clade,” rather than any other Latin American strains. The majority (*n* = 14) carried SCC [*mec* III+Cd/Hg+*ccrC*] _(TW20)_. Four isolates harbored composite SCC elements that included *czrC, speG* and additional recombinase genes *ccrA/B*-4. Based on the overall patterns, the isolates could be regarded as derivatives of SCC [*mec* III+Cd/Hg+*ccrC*] _(Bmb9393)_.

## Discussion

### Evolution of the SCC*mec* III element

CC239 is closely linked to SCC*mec* III. Only a few other lineages carry this type of SCC*mec* element. These include CC5 (Kwon et al., 2006; Shittu et al., 2009; Monecke et al., 2013b), CC398 (Nemati et al., 2008) and *Staphylococcus pseudintermedius* (KM1381, GenBank AM904732.1; KM241, GenBank AM904731). Virtually all CC239-MRSA isolates harbor one of the many different subtypes and composite elements of SCC*mec* III.

A relatively simple SCC*mec* III element, i.e., harboring *ccrAB3* and a class A *mec* complex without additional *ccr* genes, heavy metal resistance markers, integrated transposons etc. has only been observed in *S. pseudintermedius* (KM1381, GenBank AM904732.1). However, it cannot safely be assumed that SCC*mec* III was initially transmitted from *S. intermedius/pseudintermedius* as, to the best of our knowledge, the earliest observation of methicillin resistance in “*S. intermedius*” was reported in 1984 (Roy et al., 1984). The most similar SCC*mec* III element in *S. aureus* can be found in the Sanger sequenced “Eurasian Clade” strain T0131 (CP002643) where it is only supplemented by the integration of a cadmium resistance operon (for which *cadD*_(R35)_ is used as marker herein). The secondary set of SCC markers—*ccrC, ccrAA*, SCCterm02, D1GU38, *erm*(A) and *ant9*—are integrated elsewhere in the genomes of this lineage, distant from SCC*mec* and *orfX*. This is not only an interesting oddity, but raises the very practical question whether other SCC and SCC*mec* elements exist at alternative chromosomal sites away from *orfX*. If they do, this would have major consequences for rapid molecular MRSA tests as these assays target the integration of SCC*mec* into *orfX*.

Another relatively simple SCC*mec* element can be found in JKD6008 (CP002120.1) that, however, harbors additional resistance genes *cadD*_(R35)_*, aadD* and *ble*. Hybridization patterns consistent with this element were observed in most “Australian/NZ Clade” isolates and in a livestock-associated CC5-MRSA-III isolate (see Monecke et al., 2013b and Supplemental Table 1). KM1381, JKD6008 as well as all “Australian/NZ Clade” and “Eurasian Clade” isolates and sequences (except TUR1 and TUR9; Harris et al., 2010) have *dcs* rather than other SCC terminal sequences and *mecA*_(BA000018)_.

TUR1/TUR9 and strains of the “European” (85/2082, AB037671.1), “South-East Asian” (TW20, FN433596.1; XN108, CP007447; Z172, CP006838.1) and “South American/Middle Eastern” (Bmb9393, CP005288.1) clades harbor more complex SCC*mec* elements that include the cadmium resistance operon, *ccrC, ccrAA* and D1GU38 as well as (often, but not always) *erm*(A) and *ant9, tet*(K) and the mercury resistance operon. These isolates do not have *dcs* and a vast majority of isolates carry *mecA*_(CP000046)._ “European Clade” sequences share the *ccrC* allele (*ccrC*_(PM1)_) with the “Eurasian Clade” while “South-East Asian” and “South American/Middle Eastern” clades have, if present, a different *ccr*C allele [*ccrC*_(TSGH17)_].

The presence of the *mer* genes raises the question for the benefit of mercury resistance in *S. aureus*/MRSA. One possible explanation could be the past medical use of mercury (e.g., for the treatment of syphilis, in topical agents such as merbromin, or in dental restorative materials such as amalgam) that could pose a selective pressure also on staphylococci colonizing the patients in question, regardless of whether they belonged to *S. aureus* or to other, coagulase-negative staphylococcal species. This could mean that SCC*mer* elements predated SCC*mec* in the same way as mercury use predated the clinical use of antibiotics. If SCC*mec* elements evolved indeed already after the introduction of penicillin (Harkins et al., 2017), there may have been a couple of decades of time for the evolution and selection of composite SCC*mec*/SCC*mer* elements.

The composite SCC [*mec* III+Cd/Hg+*ccrC*] _(SK1585)_ element (SK1585, KL662257.1) existed at least already in the very early 1970s (as it was found in a strain epidemic in Australia from 1973 on; see below) and it appears to be ancestral to many SCC elements in CC239-MRSA. All SCC elements in “European,” “South-East Asian,” and “South American/Middle Eastern” clades could easily be described as variants of this particular element that have either acquired additional genes (ACME II, *speG, czrC, ccrA/B4*) or lost some or several genes. These latter genes have either no known function, are redundant (in case of the transposon with *erm*(A) and *ant9* as a second copy is present elsewhere in the genome) or may no longer be of major advantage anymore because the compounds that provide a selective pressure are no longer frequently used (as in the case of mercury).

When mapping the presence of subtypes of SCC*mec* III on the phylogenetic trees as proposed by Harris and Castillo-Ramírez (Harris et al., 2010; Castillo-Ramírez et al., 2012), it becomes clear that identical subtypes can be observed in different clades (e.g., SCC [*mec* III+Cd/Hg+*ccrC*] _(TW20)_, SCC [*mec* III+Cd/Hg+*ccrC*] _(Bmb939)_). There might be two different, mutually non-exclusive explanations. Firstly, these elements are subject to horizontal transfer so that SCC*mec* elements may be lost, acquired and exchanged after differentiation into different clades. Secondly, many of the SCC*mec* III subtypes differ in losses or acquisitions of accessory, purposeless or redundant genes (see above), and such events may have occurred several times. For instance, SCC [*mec* III+Cd/Hg+*ccrC*] _(TW20)_ and SCC [*mec* III+Cd/Hg+*ccrC*] _(Bmb9393)_ differ only in absence of the redundant transposon carrying *erm*(A) and *ant9*, Q93IB7 and SCCterm01 from the latter. It seems to be possible that this loss (or other similar losses) may have happened multiple times, independently from each other, to different lineages harboring SCC [*mec* III+Cd/Hg+*ccrC*] _(TW20)_. Among a cluster of “South American/Middle Eastern Clade” isolates from Riyadh with a characteristic *splE* deletion, we observed three SCC*mec* subtypes suggesting that the losses of the mercury operon, SCCterm01 and Q93IB7 are secondary only to the loss of this gene and that they may spontaneously change SCC [*mec* III+Cd/Hg+*ccrC*] _(TW20)_ to SCC [*mec* III+Cd+*ccrC*] _(S85)_ and SCC [*mec* III+Cd+*ccrC*] _(XN108)_.

An example for multiple acquisitions of one gene cluster is the observation of ACME II in dissimilar SCC elements of rather unrelated Australian and Kuwaiti strains. Likewise, repeated and independent acquisitions of *arc* genes have already been observed in Singapore (Hsu et al., 2015).

### Evolution and spread of the CC239-MRSA-III strain

Based on accumulation of SNPs and mutation rates, previous work (Harris et al., 2010; Castillo-Ramírez et al., 2012) estimated the emergence of CC239 to have occurred in the mid- or late 1960s. The preservation of CC239-MRSA isolated in 1971 in a Norwegian strain collection (Smyth et al., 2010) also hints to an emergence and early spread of this strain in the late 1960s to 1970.

When analyzing gene content, one needs to assume two major recombination events to have occurred. One event was a horizontal gene transfer of a large segment of CC30 DNA into a CC8 genome (Robinson and Enright, 2004b; Holden et al., 2009). The other was a transfer of a SCC*mec* III element either before that “CC8/CC30 hybridization” into the CC30 ancestral strain, or afterwards, into the CC239 chimeric strain. It is not clear which gene transfer happened first. We are not aware of a CC30-MRSA-III strain that may have posed as a donor for the CC30 core genomic DNA and the SCC*mec* III element. CC239-MSSA strains have been identified (Strain 21178, GenBank AGRN and Luedicke et al., 2010), but they might be secondary deletion variants that lost SCC*mec* III rather than methicillin-susceptible ancestors to CC239-MRSA-III.

Our own observations also indicate that at least the “Greek Strain” and its SCC [*mec* III+Cd/Hg+*ccrC*] _(SK1585)_ must have existed already in the early 1970s. There are reports of CC239 from Australia from this time and another strain, ST2249-MRSA-III was present in Melbourne, Australia, from 1973-1979 (predating the oldest Australian isolates of CC239 by 3 years). ST2249-MRSA-III is a chimeric strain (Nimmo et al., 2015) that combined features of CC45, CC30, and CC8 parental strains. The CC30- and CC8-like parts of its genome can be seen as one continuous segment originating from a CC239 parental strain, also including the SCC [*mec* III+Cd/Hg+*ccrC*] _(SK1585)_ element that is characteristic for the “Greek Strain”. This allows two assumptions. Firstly, an importation of the “Greek Strain” of CC239 (or of the ST2249 chimera after the hybridization event) from Greece to Melbourne appears not improbable given a large Greek community in this city. Secondly, if the recombination that gave raise to ST2249-MRSA-III happened in 1973 or earlier, the “Greek Strain” CC239 and its SCC*mec* III element must have existed already some time before allowing for its emergence and spread as far as Australia. As discussed above, the SCC [*mec* III+Cd/Hg+*ccrC*] _(SK1585)_ element could conveniently be regarded ancestral to many SCC*mec* elements in “European,” “South-East Asian,” and “South American/Middle Eastern” clades assuming that these elements emerged by serial or multiple deletions, and, occasionally, by acquisitions of genes. The other “European Clade” strains characterized by a distinct *mecR1* deletion may have evolved in the early 1980s and spread in a rather limited way, i.e., in Ireland, UK, Australia, New Zealand and the USA during that decade (Ito et al., 2001; Shore et al., 2005; Harris et al., 2010). “European Clade” strains have been in recent years still of some relevance in Greece (and in travelers returning from there), but otherwise they have been replaced by other MRSA strains.

Then there is the “Eurasian Clade” (or Harris' and Castillo-Ramírez' “Turkish Clade”; Harris et al., 2010; Castillo-Ramírez et al., 2012). A comparatively low number of distinct strains within this clade might indicate a rather recent emergence, and earliest sequences identified (Harris et al., 2010) originate from Eastern Europe and Turkey, from the mid-/late 1990s. Genotyping data indicate relatedness to the “European Clade” (Harris et al., 2010; Castillo-Ramírez et al., 2012) but the “Eurasian Clade” and the “Australian/NZ Clade” differ from others by harboring less complex composite SCC elements with *dcs* and *mecA*_(BA000018)_. Whether this indicates an independent, second acquisition of SCC*mec* III cannot yet be determined. The absence of *splE* and *fnbB* from all isolates and sequences indicate a monophyletic, clonal origin of the entire clade. TUR1 and TUR9 differ from other strains suggesting yet another horizontal gene transfer (possibly of a SCC*mec* III element from a “European” strain into an “Eurasian” strain with interrupted *nsaB*). The “Eurasian Clade” can be found in Turkey where it is frequently isolated and widespread (Tekeli et al., 2016). Furthermore it occurs in Eastern Europe including Macedonia (from where one study patient came from, see paragraph on Saxony/Germany) and, especially, Romania. In Hungary, it was common in the 1990s but it is declining since then, being replaced by other strains (Conceicao et al., 2007). It is also present in Russia, Pakistan and China. Recent reports from China indicate an emergence of the “Eurasian Clade” (with *spa* t030) at the expense of other CC239 strains (that is, of the “South-East Asian Clade”) following a North South gradient. Its distribution within China suggests import from Central Asia and/or a spill-over across the Russian border (Chen et al., 2014b). It appears to replicate faster than the “South-East Asian Clade” strains (Shang et al., 2016) and this advantage appears to outweigh in direct competition whatever advantage the presence of *sasX/sesI* may confer to the “South-East Asian” strains.

As mentioned, we found isolates matching Harris' and Castillo-Ramírez' “South American Clade” also in Russia and the Middle East. This raises the question where it emerged and to where it spread secondarily. Castillo-Ramírez estimated “*the introduction into South America to have occurred approximately…in 1992 (late 1989, 1993)*” (Castillo-Ramírez et al., 2012). Since the Irish AR09/Phenotype III outbreak strain was brought to Ireland from Iraq in 1985, and since it was described to be similar to a strain sampled in Baghdad as early as 1984 (Humphreys et al., 1990) we assume that this clade evolved earlier, possibly in the Middle East from where it may have spread to India, Russia and Europe. Again, travel from India to the Middle East and back as well as from the Middle East to Europe might have played a role. Strains of this clade may have come to Latin America from Europe or directly from the Middle East, and it became common and widespread in several Latin American countries (Harris et al., 2010; Castillo-Ramírez et al., 2012). Recent evidence, however, shows that this clade is declining or disappearing, except possibly in Ecuador and Peru (Arias et al., 2017). Many sequences of the “South American/Middle Eastern Clade” originated from Brazil (Harris et al., 2010; Castillo-Ramírez et al., 2012). While it is tempting to assume a link between Portugal and Brazil, a majority of Portuguese sequences clearly belong to a separate, geographically restricted, clade; and the few sequences that match the “South American/Middle Eastern Clade” might be re-imported by travelers (Harris et al., 2010; Castillo-Ramírez et al., 2012). While it is receding in Latin America and in India (D'Souza et al., 2010) and while it largely disappeared from Ireland, this clade still appears to be endemic in the Middle East and in Russia. One notable strain carrying *tst1* has been endemic in the Russian town of Krasnoyarsk for several years (first observed in 2008; Iwao et al., 2012). The presence of the *tst1* gene in CC239 is rather unique although this or a similar strain has also been described from Iran (Havaei et al., 2013).

The “South-East Asian Clade” most likely evolved from a “South American/Middle Eastern Clade” strain (or from a common ancestor of both clades) by acquisition of a prophage carrying *sasX/sesI*. Providing that this gene was acquired only once, which is the most parsimonious assumption, it might be assumed that this happened between the split of the related “South-East Asian,” “Portuguese,” and “South American/Middle Eastern” lineages and the proliferation of different strains within “South-East Asian Clade”, i.e., between ca. 1969 and 1985 based on Castillo-Ramírez' data (Castillo-Ramírez et al., 2012). The oldest published genome sequences originate from 1997 (CUHK_HK1997), 1998 (CHI59), 2001 (DEN907), and 2003 (TW20). The “South-East Asian Clade” spread in South-East Asia, including India, Thailand, Malaysia, Singapore, and China. Although it is still present in Hong Kong and in Southern Mainland China, in Northern China “Eurasian Clade” strains predominate nowadays (see above and Chen et al., 2014b; Shang et al., 2016). The “South-East Asian Clade” was also occasionally introduced to Europe, most likely by travelers (DEN907, TW20, AR44, P32, Finland E24_98541 from the Harmony collection), without becoming endemic there, and it also has been identified in Canada and the USA. The presence of the “South-East Asian Clade” and particularly of strains that appear to originate from India and South-East Asia in Kuwait and Saudi Arabia may easily be attributed to the large number of Indian and South-East Asian workers in the Gulf States (Birks et al., 1988). An interesting observation is the presence of this clade, rather than of the “South American” one, on the Caribbean islands of Trinidad & Tobago. A possible explanation is the Indian/South Asian descent of a high proportion of inhabitants of Trinidad & Tobago (ca. 38% of the total population, or 1.4 million people; http://www.tt.undp.org/content/dam/trinidad_tobago/docs/DemocraticGovernance/Publications/TandT_Demographic_Report_2011.pdf). Another 35% are of African descent, but no sufficient subtyping data for African CC239-MRSA are available. Possibly, an importation of MRSA by visits to ancestral lands might have played a greater role in the case of Trinidad & Tobago than just the mere geographic proximity to Latin America.

Some of Harris' strains from Thailand, one from Vietnam and one from Denmark are placed into the “South-East Asian Clade” by presence of *sasX/sesI* and by sequence analysis (Harris et al., 2010; Castillo-Ramírez et al., 2012). However, they differ from other strains of that clade in lacking *hla* and in harboring *dcs* instead of other SCC terminal sequences. The latter could indicate that their SCC*mec* elements rather originated from a horizontal gene transfer, maybe from the Australian/NZ lineage (see Table 2a).

Finally, there are some isolates and sequences that do not fit into the major clades. This includes the “Portuguese Clade” and the “Australian/New Zealand Clade.” The former is, according to sequence analysis (Harris et al., 2010; Castillo-Ramírez et al., 2012), related to the “South-East Asian” and “South American/Middle Eastern” clades. The latter was not represented by SNP-based studies and its SCC*mec* element might be more related to the one in non-CC239 strains (including *S. pseudintermedius* KM1381) than to the SCC*mec* elements in other CC239. We identified a cluster of Middle Eastern isolates (including one from Libya and one from Russia) that might constitute yet another clade. Finally there are strains such as DS_014, UR110 and P32 that could be assigned to the major clades but that still differ from them in particular features (such as *mecA* alleles). They may have evolved by further horizontal gene transfers. They also could be representatives of separate lineages or clades of CC239 that may be restricted to certain geographic regions poorly, or not at all, covered by previous typing and sequencing work. It might be expected that there are even more such unrecognized clades because CC239 was common in Western Europe and the USA before modern typing and sequencing technologies emerged, and because it is now common in countries were such technologies are not extensively applied.

Regarding typing technologies, NGS methods and DNA array hybridization profiling allow assignment to clades and strains. Arrays are currently cheaper and more convenient in a clinical setting. NGS can achieve a higher resolution although the definition of a “breakpoint for identity or non-identity” (i.e., how many differences between related isolates rule out direct transmission) still poses a challenge. This is quite a relevant issue for practical purposes. Traditionally, a “group of isolates that can be distinguished from other isolates of the same genus and species by phenotypic characteristics or genotypic characteristics or both” were regarded as a strain or clone (Tenover et al., 1995; Dijkshoorn et al., 2000). However, recent typing technologies achieve a level of resolution that is sufficiently informative to differentiate dozens of variants within one “strain” such as CC239-MRSA-III (as seen in the tables herein). Therefore, defining “strains” may still be useful for epidemiological purposes, but it is somewhat awkward and prone to subjectivity. Both approaches, short read NGS methods and microarray hybridization profiling, have difficulties with gene duplications and translocations if potentially mobile genes are flanked by repetitive and multi-copy sequences. Practically, this means that both technologies are useful for typing, but for the reconstruction of phylogenetic relationships, conventional sequencing still is unsurpassed.

On a very practical level, the definition of clades or variants can be useful for infection control purposes. For this pandemic strain it was possible to define such clades and to link molecular identifiers to geographic origins. Analyses of markers discussed herein, regardless whether by array hybridization, multiplex PCR, or by genome sequencing, can help assigning clinical isolates to these clades or variants and thus help to identify the provenance of an isolate and to discern imported from locally acquired cases. This is relevant as this strain was able to cause large hospital-born outbreaks upon importation with travelers or repatriated patients, as for instance the Irish experience (Humphreys et al., 1990; Shore et al., 2005), the TW20 outbreak in London (Holden et al., 2009), our own observations of the “Greek Strain” in Saxony or the spread of the “South American/Middle Eastern” clade in Latin America or of the “Eurasian Clade” in China showed. Based on European and North American experience, it is tempting to assume that CC239-MRSA-III has been side-lined by other clones or has even become extinct. Given the increasing scale of global travel and migration, there is still a possibility of re-importation and secondary spread. One should keep in mind that this strain still frequently detected in hospitals serving literally more than half of the world's population, i.e., China, India, South-East Asia, Turkey and the Middle East, Romania, Russia and parts of Latin America.

In conclusion, CC239-MRSA-III is a truly pandemic strain that, for nearly half a century, traveled around the world, infecting and even killing thousands of patients. This pandemic does not originate from elusive animals hosts in jungles and savannahs but from professionals working in the cleanest and most hygienic environments possible, that is, hospitals and operating theaters. Typing techniques allow following these movements, and even pinpointing individual index patients from whom this strain was brought into certain countries. However, understanding of a pandemic does not automatically results in an ability to prevent it. The very fact that an exclusively hospital-borne pandemic can spread that far and can last that long emphasizes an urgent need for improved hand hygiene, mandatory screening of staff and admitted patients, and decolonization procedures, a prudent use of antimicrobial agents and in general far more effective infection prevention and control measures.

## Author contributions

SM designed the study, supervised and analyzed experiments, and wrote the manuscript. PS designed the primers and probes for the arrays used herein and analyzed genome sequences as well as experimental data. DG, EM, AR, AR-L, and RR performed experiments. SB and VG performed experiments and obtained isolates. PA, DB, MB, OD, MI, BJ, LJ, MN, AS, SS, LS, AMS, MS, AT, EU, TV, and JZ obtained isolates and provided clinical/epidemiological data. DC, GC, and ACS obtained isolates, provided clinical/epidemiological data and revised the manuscript. RE designed the study, supervised experiments, and revised the manuscript.

### Conflict of interest statement

SM, PS, DG, EM, AR, and RE are employees of Abbott (Alere Technologies GmbH), the company that manufactures the microarrays used herein. The remaining authors declare that the research was conducted in the absence of any commercial or financial relationships that could be construed as a potential conflict of interest.

## Supplementary material

Supplementary Table 1 Full hybridization profiles for isolates characterized by microarray, and predicted hybridization profiles for analyzed sequences.
