# Supplementary material for: Molecular Typing of ST239-MRSA-III From Diverse Geographic Locations and the Evolution of the SCCmec III Element During Its Intercontinental Spread
Source: Front Microbiol. 2018 Jul 6;9:1436. doi: 10.3389/fmicb.2018.01436 (PMC6066798; doi:10.3389/fmicb.2018.01436)
Supplement: Supplementary Table 1 — Full hybridization profiles for isolates characterized by microarray, and predicted hybridization profiles for analyzed sequences. [file Table_1.PDF]

STRAIN / ISOLATE	Gen Bank	Bio sample Accession Number	Year of isolation	RIDOM spa	Virtual or real hybridisation	SCC*mec* type	METHICILLIN RESISTANCE AND SCCmec TYPING							
							ugpQ	mecA				delta_mecR	mecR	mecI
							ugpQ	mecA	mecA CP000046.1 SaurCOL	mecA BA000018 SaurN315	mecA GQ902038 Saur_haem pseud	Delta mecR1	mecR1	mecI
							Glycerophosphoryl diester phospho-diesterase.	Modified penicillin binding protein (PBP2a)				Truncated methicillin resistance operon repressor 1. Truncated mecR1 is present in SCCmec I, IV, V, VI, VII; complete absence of mecR1 from SCCmec	Methicillin resistance operon repressor 1. Un-truncated sequence in SCCmec II, SCCmec III, SCCmec VIII	Methicillin-resistance regulatory protein. Present in SCCmec II (although absent from Irish SCCmec II variants C and E), SCCmec III, SCCmec VIII
**>"Eurasian Clade": TUR1**														
TUR1 (Turkey)		SAMEA1029552	1996	t030	Virtual hybr. (prev. publ.)	SCC [mec III+Cd+ccrC] (TUR1)	POS	POS	POS	NEG	NEG	POS	POS	POS
TUR9 (Turkey)		SAMEA985415	1995	t030	Virtual hybr. (prev. publ.)	SCC [mec III+Cd+ccrC] (TUR1)	POS	POS	POS	NEG	NEG	POS	POS	POS
**>"Eurasian Clade" : T0131-Strain**														
T0131 (China)	CP002643.1	SAMN02603905	2006	t030	Virtual hybr. (prev. publ.)	SCC [mec III+Cd+ccrC] (T0131)	POS	POS	NEG	POS	NEG	POS	POS	POS
MU4 (Turkey)	CTYE	SAMEA862564	2008		Virtual hybr. (prev. publ.)	SCC [mec III+Cd+ccrC] (T0131)	POS	POS	NEG	POS	NEG	POS	POS	POS
DEU11 (Turkey)	CTYI	SAMEA862554	2009		Virtual hybr. (prev. publ.)	SCC [mec III+Cd+ccrC] (T0131)	POS	POS	NEG	POS	NEG	POS	POS	POS
DEU16 (Turkey)	CTYV	SAMEA862557	2008		Virtual hybr. (prev. publ.)	SCC [mec III+Cd+ccrC] (T0131)	POS	POS	NEG	POS	NEG	POS	POS	POS
Romania_Iasi_MRSA-07_54			2009		Array hybr.	SCC [mec III+Cd+ccrC] (T0131)	POS	POS	NEG	POS	NEG	POS	POS	POS
**>"Eurasian Clade": arsC-SCC-pos. strains from Romania**														
Romania_Iasi_MRSA-05_46			2009		Array hybr.	SCC [mec III+As/Cd+ccrC] (Unknown, ST239-1)	POS	POS	NEG	POS	NEG	POS	POS	POS
Romania_Iasi_MRSA-06_47			2009		Array hybr.	SCC [mec III+As/Cd+ccrC] (Unknown, ST239-2)	POS	POS	NEG	POS	NEG	POS	POS	POS
**>"Eurasian Clade": 16K/CN79**														
CN79 (China)	ANCJ	SAMN02470786	2006	t030	Virtual hybr. (prev. publ.)	SCC [mec III+Cd+ccrC] (CN79)	POS	POS	NEG	POS	NEG	POS	POS	POS
CUHK_BJ2002 (China)	AZMY	SAMN02442047	2002		Virtual hybr. (prev. publ.)	SCC [mec III+Cd+ccrC] (CN79)	POS	POS	NEG	POS	NEG	POS	POS	POS
CUHK_BJ2007 (China)	AZMX	SAMN02442048	2007		Virtual hybr. (prev. publ.)	SCC [mec III+Cd+ccrC] (CN79)	POS	POS	NEG	POS	NEG	POS	POS	POS
3HK (Czech Republic)		SAMEA1029566	2000	t030	Virtual hybr. (prev. publ.)	SCC [mec III+Cd+ccrC] (CN79)	POS	POS	NEG	POS	NEG	POS	POS	POS
HU109 (Hungary)		SAMEA1029567	1996	t984	Virtual hybr. (prev. publ.)	SCC [mec III+Cd+ccrC] (CN79)	POS	POS	NEG	POS	NEG	POS	POS	POS
HUR18 (Hungary)		SAMEA1029551	1997	t984	Virtual hybr. (prev. publ.)	SCC [mec III+Cd+ccrC] (CN79)	POS	POS	NEG	POS	NEG	POS	POS	POS
H482 (Romania)	CTXK	SAMEA862595	2007		Virtual hybr. (prev. publ.)	SCC [mec III+Cd+ccrC] (CN79)	POS	POS	NEG	POS	NEG	POS	POS	POS
16K (Russia)	BABZ	SAMD00036546	N/A	t030	Virtual hybr. (prev. publ.)	SCC [mec III+Cd+ccrC] (CN79)	POS	POS	NEG	POS	NEG	POS	POS	POS
DEU10 (Turkey)	CTYJ	SAMEA862583			Virtual hybr. (prev. publ.)	SCC [mec III+Cd+ccrC] (CN79)	POS	POS	NEG	POS	NEG	POS	POS	POS
DEU12 (Turkey)	CTYN	SAMEA862553			Virtual hybr. (prev. publ.)	SCC [mec III+Cd+ccrC] (CN79)	POS	POS	NEG	POS	NEG	POS	POS	POS
DEU14 (Turkey)	CTYU	SAMEA862555			Virtual hybr. (prev. publ.)	SCC [mec III+Cd+ccrC] (CN79)	POS	POS	NEG	POS	NEG	POS	POS	POS
DEU15 (Turkey)	CTYT	SAMEA862558			Virtual hybr. (prev. publ.)	SCC [mec III+Cd+ccrC] (CN79)	POS	POS	NEG	POS	NEG	POS	POS	POS
DEU17 (Turkey)	CTYX	SAMEA862559			Virtual hybr. (prev. publ.)	SCC [mec III+Cd+ccrC] (CN79)	POS	POS	NEG	POS	NEG	POS	POS	POS
DEU19 (Turkey)	CTYZ	SAMEA862561			Virtual hybr. (prev. publ.)	SCC [mec III+Cd+ccrC] (CN79)	POS	POS	NEG	POS	NEG	POS	POS	POS
DEU2 (Turkey)	CTYM	SAMEA862577			Virtual hybr. (prev. publ.)	SCC [mec III+Cd+ccrC] (CN79)	POS	POS	NEG	POS	NEG	POS	POS	POS
DEU20 (Turkey)	CTYY	SAMEA862560			Virtual hybr. (prev. publ.)	SCC [mec III+Cd+ccrC] (CN79)	POS	POS	NEG	POS	NEG	POS	POS	POS
DEU23 (Turkey)	CTYS	SAMEA862541	2008		Virtual hybr. (prev. publ.)	SCC [mec III+Cd+ccrC] (CN79)	POS	POS	NEG	POS	NEG	POS	POS	POS
DEU3 (Turkey)	CTYL	SAMEA862574			Virtual hybr. (prev. publ.)	SCC [mec III+Cd+ccrC] (CN79)	POS	POS	NEG	POS	NEG	POS	POS	POS
DEU5 (Turkey)	CTYO	SAMEA862580			Virtual hybr. (prev. publ.)	SCC [mec III+Cd+ccrC] (CN79)	POS	POS	NEG	POS	NEG	POS	POS	POS
DEU6 (Turkey)	CTYW	SAMEA862581			Virtual hybr. (prev. publ.)	SCC [mec III+Cd+ccrC] (CN79)	POS	POS	NEG	POS	NEG	POS	POS	POS
DEU8 (Turkey)	CTYR	SAMEA862579			Virtual hybr. (prev. publ.)	SCC [mec III+Cd+ccrC] (CN79)	POS	POS	NEG	POS	NEG	POS	POS	POS
HU11 (Turkey)	CTZF	SAMEA862634	2007		Virtual hybr. (prev. publ.)	SCC [mec III+Cd+ccrC] (CN79)	POS	POS	NEG	POS	NEG	POS	POS	POS
HU13 (Turkey)	CTWP	SAMEA862636			Virtual hybr. (prev. publ.)	SCC [mec III+Cd+ccrC] (CN79)	POS	POS	NEG	POS	NEG	POS	POS	POS
HU14 (Turkey)	CTWN	SAMEA862637			Virtual hybr. (prev. publ.)	SCC [mec III+Cd+ccrC] (CN79)	POS	POS	NEG	POS	NEG	POS	POS	POS
HU15 (Turkey)	CTWK	SAMEA862630			Virtual hybr. (prev. publ.)	SCC [mec III+Cd+ccrC] (CN79)	POS	POS	NEG	POS	NEG	POS	POS	POS
HU16 (Turkey)	CTWJ	SAMEA862631			Virtual hybr. (prev. publ.)	SCC [mec III+Cd+ccrC] (CN79)	POS	POS	NEG	POS	NEG	POS	POS	POS
HU17 (Turkey)	CTWI	SAMEA862632			Virtual hybr. (prev. publ.)	SCC [mec III+Cd+ccrC] (CN79)	POS	POS	NEG	POS	NEG	POS	POS	POS
HU21 (Turkey)	CTWC	SAMEA862639			Virtual hybr. (prev. publ.)	SCC [mec III+Cd+ccrC] (CN79)	POS	POS	NEG	POS	NEG	POS	POS	POS
HU23 (Turkey)	CTWH	SAMEA862643	2009		Virtual hybr. (prev. publ.)	SCC [mec III+Cd+ccrC] (CN79)	POS	POS	NEG	POS	NEG	POS	POS	POS
HU26 (Turkey)	CTWB	SAMEA862644			Virtual hybr. (prev. publ.)	SCC [mec III+Cd+ccrC] (CN79)	POS	POS	NEG	POS	NEG	POS	POS	POS
HU4 (Turkey)	CTZI	SAMEA862618			Virtual hybr. (prev. publ.)	SCC [mec III+Cd+ccrC] (CN79)	POS	POS	NEG	POS	NEG	POS	POS	POS
HU5 (Turkey)	CTZJ	SAMEA862625			Virtual hybr. (prev. publ.)	SCC [mec III+Cd+ccrC] (CN79)	POS	POS	NEG	POS	NEG	POS	POS	POS
HU6 (Turkey)	CTZO	SAMEA862624			Virtual hybr. (prev. publ.)	SCC [mec III+Cd+ccrC] (CN79)	POS	POS	NEG	POS	NEG	POS	POS	POS
HU7 (Turkey)	CTZK	SAMEA862623			Virtual hybr. (prev. publ.)	SCC [mec III+Cd+ccrC] (CN79)	POS	POS	NEG	POS	NEG	POS	POS	POS
HU8 (Turkey)	CTZP	SAMEA862622			Virtual hybr. (prev. publ.)	SCC [mec III+Cd+ccrC] (CN79)	POS	POS	NEG	POS	NEG	POS	POS	POS
HU9 (Turkey)	CTZM	SAMEA862627			Virtual hybr. (prev. publ.)	SCC [mec III+Cd+ccrC] (CN79)	POS	POS	NEG	POS	NEG	POS	POS	POS
IU1 (Turkey)	CTWE	SAMEA862647	2006		Virtual hybr. (prev. publ.)	SCC [mec III+Cd+ccrC] (CN79)	POS	POS	NEG	POS	NEG	POS	POS	POS
IU10 (Turkey)	CTXF	SAMEA862652			Virtual hybr. (prev. publ.)	SCC [mec III+Cd+ccrC] (CN79)	POS	POS	NEG	POS	NEG	POS	POS	POS
IU11 (Turkey)	CTWM	SAMEA862653			Virtual hybr. (prev. publ.)	SCC [mec III+Cd+ccrC] (CN79)	POS	POS	NEG	POS	NEG	POS	POS	POS
IU12 (Turkey)	CTWD	SAMEA862658			Virtual hybr. (prev. publ.)	SCC [mec III+Cd+ccrC] (CN79)	POS	POS	NEG	POS	NEG	POS	POS	POS
IU13 (Turkey)	CTWO	SAMEA862659			Virtual hybr. (prev. publ.)	SCC [mec III+Cd+ccrC] (CN79)	POS	POS	NEG	POS	NEG	POS	POS	POS
IU15 (Turkey)	CTXD	SAMEA862657			Virtual hybr. (prev. publ.)	SCC [mec III+Cd+ccrC] (CN79)	POS	POS	NEG	POS	NEG	POS	POS	POS
IU18 (Turkey)	CTWT	SAMEA862534			Virtual hybr. (prev. publ.)	SCC [mec III+Cd+ccrC] (CN79)	POS	POS	NEG	POS	NEG	POS	POS	POS
IU19 (Turkey)	CTXJ	SAMEA862533			Virtual hybr. (prev. publ.)	SCC [mec III+Cd+ccrC] (CN79)	POS	POS	NEG	POS	NEG	POS	POS	POS
IU2 (Turkey)	CTWG	SAMEA862646			Virtual hybr. (prev. publ.)	SCC [mec III+Cd+ccrC] (CN79)	POS	POS	NEG	POS	NEG	POS	POS	POS
IU4 (Turkey)	CTWF	SAMEA862648			Virtual hybr. (prev. publ.)	SCC [mec III+Cd+ccrC] (CN79)	POS	POS	NEG	POS	NEG	POS	POS	POS
IU6 (Turkey)	CTWL	SAMEA862651			Virtual hybr. (prev. publ.)	SCC [mec III+Cd+ccrC] (CN79)	POS	POS	NEG	POS	NEG	POS	POS	POS
IU7 (Turkey)	CTWX	SAMEA862650			Virtual hybr. (prev. publ.)	SCC [mec III+Cd+ccrC] (CN79)	POS	POS	NEG	POS	NEG	POS	POS	POS
IU9 (Turkey)	CTWQ	SAMEA862617			Virtual hybr. (prev. publ.)	SCC [mec III+Cd+ccrC] (CN79)	POS	POS	NEG	POS	NEG	POS	POS	POS
MU1 (Turkey)	CTZC	SAMEA862569	2008		Virtual hybr. (prev. publ.)	SCC [mec III+Cd+ccrC] (CN79)	POS	POS	NEG	POS	NEG	POS	POS	POS
MU10 (Turkey)	CTYP	SAMEA862600			Virtual hybr. (prev. publ.)	SCC [mec III+Cd+ccrC] (CN79)	POS	POS	NEG	POS	NEG	POS	POS	POS
MU20 (Turkey)	CTZG	SAMEA862620			Virtual hybr. (prev. publ.)	SCC [mec III+Cd+ccrC] (CN79)	POS	POS	NEG	POS	NEG	POS	POS	POS
Mu3 (Turkey)	CTYB	SAMEA862565			Virtual hybr. (prev. publ.)	SCC [mec III+Cd+ccrC] (CN79)	POS	POS	NEG	POS	NEG	POS	POS	POS
MU5 (Turkey)	CTYF	SAMEA862604			Virtual hybr. (prev. publ.)	SCC [mec III+Cd+ccrC] (CN79)	POS	POS	NEG	POS	NEG	POS	POS	POS
MU6 (Turkey)	CTYK	SAMEA862605			Virtual hybr. (prev. publ.)	SCC [mec III+Cd+ccrC] (CN79)	POS	POS	NEG	POS	NEG	POS	POS	POS
MU7 (Turkey)	CTYH	SAMEA862602			Virtual hybr. (prev. publ.)	SCC [mec III+Cd+ccrC] (CN79)	POS	POS	NEG	POS	NEG	POS	POS	POS
TUR27 (Turkey)		SAMEA1029557	1996	t030	Virtual hybr. (prev. publ.)	SCC [mec III+Cd+ccrC] (CN79)	POS	POS	NEG	POS	NEG	POS	POS	POS
NCTR 32S	JTJX	SAMN03161113	N/A		Virtual hybr. (prev. publ.)	SCC [mec III+Cd+ccrC] (CN79)	POS	POS	NEG	POS	NEG	POS	POS	POS
SA02_A4 = Russia-06_0085_Moscow		SAMN05230108	2011	t632	Virtual hybr. (prev. publ.)	SCC [mec III+Cd+ccrC] (CN79)	POS	POS	NEG	POS	NEG	POS	POS	POS
					Array hybr.		POS	POS				POS	POS	POS
Dresden_08V35987 (Turkey)			2008		Array hybr.	SCC [mec III+Cd+ccrC] (CN79)	POS	POS				POS	POS	POS
Dresden_17ANR577152			2017		Array hybr.	SCC [mec III+Cd+ccrC] (CN79)	POS	POS				POS	POS	POS
Dresden_17ANR580374 (Makedonia)			2017		Array hybr.	SCC [mec III+Cd+ccrC] (CN79)	POS	POS				POS	POS	POS
Hong Kong_69-II			2009-2010		Array hybr.	SCC [mec III+Cd+ccrC] (CN79)	POS	POS				POS	POS	POS
Hong Kong_93			2009-2010		Array hybr.	SCC [mec III+Cd+ccrC] (CN79)	POS	POS				POS	POS	POS
Rawalpindi_Kidney Center_03_SK1			2017		Array hybr.	SCC [mec III+Cd+ccrC] (CN79)	POS	POS				POS	POS	POS
Rawalpindi_Kidney Center_08			2017		Array hybr.	SCC [mec III+Cd+ccrC] (CN79)	POS	POS				POS	POS	POS
Rawalpindi_Kidney Center_10			2017		Array hybr.	SCC [mec III+Cd+ccrC] (CN79)	POS	POS				POS	POS	POS
Rawalpindi_Kidney Center_19			2017		Array hybr.	SCC [mec III+Cd+ccrC] (CN79)	POS	POS				POS	POS	POS
Rawalpindi_Kidney Center_50_SK1			2017		Array hybr.	SCC [mec III+Cd+ccrC] (CN79)	POS	POS				POS	POS	POS
Romania_Iasi_BC-49_430			2012		Array hybr.	SCC [mec III+Cd+ccrC] (CN79)	POS	POS	NEG	POS	NEG	POS	POS	POS
Romania_Iasi_MRSA_284			2011		Array hybr.	SCC [mec III+Cd+ccrC] (CN79)	POS	POS				POS	POS	POS
Romania_Iasi_MRSA-32_318			2011		Array hybr.	SCC [mec III+Cd+ccrC] (CN79)	POS	POS				POS	POS	POS
Romania_Iasi_MRSA-54_430			2008-2012 (?)		Array hybr.	SCC [mec III+Cd+ccrC] (CN79)	POS	POS				POS	POS	POS
Romania_Iasi_SSTI-01_2			2008-2012 (?)		Array hybr.	SCC [mec III+Cd+ccrC] (CN79)	POS	POS				POS	POS	POS
Romania_Iasi_SSTI-10_101			2009		Array hybr.	SCC [mec III+Cd+ccrC] (CN79)	POS	POS				POS	POS	POS
Romania_Iasi_SSTI-11_106			2009		Array hybr.	SCC [mec III+Cd+ccrC] (CN79)	POS	POS				POS	POS	POS
Russia-01_0001_SaintPetersburg			2011	t632	Array hybr.	SCC [mec III+Cd+ccrC] (CN79)	POS	POS	NEG	POS	NEG	POS	POS	POS
Russia-02_0004_SaintPetersburg			2011	t632	Array hybr.	SCC [mec III+Cd+ccrC] (CN79)	POS	POS				POS	POS	POS
Russia-03_0057_SaintPetersburg			2011	t632	Array hybr.	SCC [mec III+Cd+ccrC] (CN79)	POS	POS				POS	POS	POS
Russia-04_0076_Moscow			2011	t632	Array hybr.	SCC [mec III+Cd+ccrC] (CN79)	POS	POS				POS	POS	POS
Russia-05_0078_Moscow			2011	t632	Array hybr.	SCC [mec III+Cd+ccrC] (CN79)	POS	POS				POS	POS	POS
Russia-08_0135_Moscow			2011	t030	Array hybr.	SCC [mec III+Cd+ccrC] (CN79)	POS	POS				POS	POS	POS
Russia-09_0150_Kurgan			2011	t030	Array hybr.	SCC [mec III+Cd+ccrC] (CN79)	POS	POS				POS	POS	POS
Russia-10_0162_Kurgan			2011	t030	Array hybr.	SCC [mec III+Cd+ccrC] (CN79)	POS	POS				POS	POS	POS
Russia-14_0184_Moscow			2007	t030	Array hybr.	SCC [mec III+Cd+ccrC] (CN79)	POS	POS				POS	POS	POS
Russia-15_0232_SaintPetersburg			2012	t4238	Array hybr.	SCC [mec III+Cd+ccrC] (CN79)	POS	POS				POS	POS	POS
Russia-22_0391_Chelyabinsk			2012	t030	Array hybr.	SCC [mec III+Cd+ccrC] (CN79)	POS	POS				POS	POS	POS
Russia-23_0392_Chelyabinsk			2012	t030	Array hybr.	SCC [mec III+Cd+ccrC] (CN79)	POS	POS				POS	POS	POS
**>"Eurasian Clade" : IU17**														
IU17 (Turkey)	CTXB	SAMEA862629	2014		Virtual hybr. (prev. publ.)	SCC [mec III+Cd+ccrC] (IU17)	POS	POS	NEG	POS	NEG	POS	POS	POS
IU20 (Turkey)	CTXE	SAMEA862563	2014		Virtual hybr. (prev. publ.)	SCC [mec III+Cd+ccrC] (IU17)	POS	POS	NEG	POS	NEG	POS	POS	POS

STRAIN / ISOLATE	Gen Bank	Bio sample Accession Number	Year of isolation	RIDOM spa	Virtual or real hybridisation	SCC*mec* type	METHICILLIN RESISTANCE AND SCCmec TYPING							
							ugpQ	mecA				delta_mecR	mecR	mecI
							ugpQ	mecA	mecA CP000046.1 SaurCOL	mecA BA000018 SaurN315	mecA GQ902038 Saur_haem pseud	Delta mecR1	mecR1	mecI
							Glycerophosphoryl diester phospho-diesterase.	Modified penicillin binding protein (PBP2a)				Truncated methicillin resistance operon repressor 1. Truncated mecR1 is present in SCCmec I, IV, V, VI, VII; complete absence of mecR1 from SCCmec	Methicillin resistance operon repressor 1. Un-truncated sequence in SCCmec II, SCCmec III, SCCmec VIII	Methicillin-resistance regulatory protein. Present in SCCmec II (although absent from Irish SCCmec II variants C and E), SCCmec III, SCCmec VIII
**>"Eurasian Clade": Sporadic strains from Saxony**														
Dresden_16ANR581769		279651	2016		Array hybr.	SCC [mec III+Cd+ccrC] (Unknown, ST239-2)	POS	POS	NEG	POS	NEG	POS	POS	POS
Dresden_17ANR578769		297337	2017		Array hybr.	SCC [mec III+Cd+ccrC] (Unknown, ST239-3)	POS	POS	NEG	POS	NEG	POS	POS	POS
**>"Eurasian Clade": Kuwaiti ACME II-pos. Strain**														
Kuwait_306		304527	2010		Array hybr.	SCC [mec III+ACME2+Cd+ccrC] (Unknown, ST239)	POS	POS	NEG	POS	NEG	POS	POS	POS
Kuwait_301		306221	2010		Array hybr.	SCC [mec III+ACME2+Cd+ccrC] (Unknown, ST239)	POS	POS				POS	POS	POS
**>"European Clade": "Greek Epidemic Strain"**														
GRE18 (Greece)		SAMEA1029546	1998	t037	Virtual hybr. (prev. publ.)	SCC [mec III+Cd/Hg+ccrC] (SK1585)	POS	POS	POS	NEG	NEG	POS	POS	POS
GRE317 (Greece)		SAMEA1029564	1999	t138	Virtual hybr. (prev. publ.)	SCC [mec III+Cd/Hg+ccrC] (SK1585)	POS	POS	POS	NEG	NEG	POS	POS	POS
GRE4 (Greece)		SAMEA1029513	1998	t037	Virtual hybr. (prev. publ.)	SCC [mec III+Cd/Hg+ccrC] (SK1585)	POS	POS	POS	NEG	NEG	POS	POS	POS
R35 (USA)		SAMEA1029532	1987	t037	Virtual hybr. (prev. publ.)	SCC [mec III+Cd/Hg+ccrC] (SK1585)	POS	POS	POS	NEG	NEG	POS	POS	POS
Casablanca HT20060548			2005		Array hybr.	SCC [mec III+Cd/Hg+ccrC] (SK1585)	POS	POS				POS	POS	POS
Casablanca HT20060550			2005		Array hybr.	SCC [mec III+Cd/Hg+ccrC] (SK1585)	POS	POS				POS	POS	POS
Dresden_01V34572 (Greece)			2001	t037	Array hybr.	SCC [mec III+Cd/Hg+ccrC] (SK1585)	POS	POS	POS	NEG	NEG	POS	POS	POS
Dresden_01V36469			2001		Array hybr.	SCC [mec III+Cd/Hg+ccrC] (SK1585)	POS	POS				POS	POS	POS
Dresden 01V39123			2001		Array hybr.	SCC [mec III+Cd/Hg+ccrC] (SK1585)	POS	POS				POS	POS	POS
Dresden 01V39143			2001		Array hybr.	SCC [mec III+Cd/Hg+ccrC] (SK1585)	POS	POS				POS	POS	POS
Greece 1 3680 Harmony			1994		Array hybr.	SCC [mec III+Cd/Hg+ccrC] (SK1585)	POS	POS				POS	POS	POS
**>"European Clade": UK-01/Irish AR01 Epidemic Strain**														
ANS46 (Australia)		SAMEA1029537	1982	t037	Virtual hybr. (prev. publ.)	SCC [mec III (Delta mecR1-)+Cd/Hg+ccrC] (85/2082)	POS	POS	POS	NEG	NEG	NEG	POS	POS
LHH1 (USA)		SAMEA1029523	1994	t037	Virtual hybr. (prev. publ.)	SCC [mec III (Delta mecR1-)+Cd/Hg+ccrC] (85/2082)	POS	POS	POS	NEG	NEG	NEG	POS	POS
Dublin-DSH_AR01_0-1085			1989	t037	Array hybr.	SCC [mec III (Delta mecR1-)+Cd/Hg+ccrC] (85/2082)	POS	POS				NEG	POS	POS
Dublin-DSH_AR01_0-1118			1989	t037	Array hybr.	SCC [mec III (Delta mecR1-)+Cd/Hg+ccrC] (85/2082)	POS	POS				NEG	POS	POS
Dublin-DSH_AR15_0098			1993	t037	Array hybr.	SCC [mec III (Delta mecR1-)+Cd/Hg+ccrC] (85/2082)	POS	POS				NEG	POS	POS
Dublin-DSH_AR15_0104			1993	t037	Array hybr.	SCC [mec III (Delta mecR1-)+Cd/Hg+ccrC] (85/2082)	POS	POS				NEG	POS	POS
UK_NCTC11939_UK-1_Harmony			"Early 1980s"		Array hybr.	SCC [mec III (Delta mecR1-)+Cd/Hg+ccrC] (85/2082)	POS	POS				NEG	POS	POS
**>"European Clade": BK2421**														
BK2421 (USA)		SAMEA1029510	1996	t037	Virtual hybr. (prev. publ.)	SCC [mec III (Delta mecR1-)+Cd/Hg+ccrC] (BK2421)	POS	POS	POS	NEG	NEG	NEG	POS	POS
**>"Aussi/NZ Clade", JKD6008 Strain**														
JKD6009 (Australia)	ABSA	SAMN02470553	N/A	t037	Virtual hybr. (prev. publ.)	SCC [mec III+Cd] (JKD6008)	POS	POS	NEG	POS	NEG	POS	POS	POS
JKD6008 (New Zealand)	CP002120.1	SAMN02603619	2003	t037	Virtual hybr. (prev. publ.)	SCC [mec III+Cd] (JKD6008)	POS	POS	NEG	POS	NEG	POS	POS	POS
Perth_2015-S20209			2015		Virtual hybr. (study isol.)	SCC [mec III+Cd] (JKD6008)	POS	POS	NEG	POS	NEG	POS	POS	POS
Perth_2015-S20305			2015		Virtual hybr. (study isol.)	SCC [mec III+Cd] (JKD6008)	POS	POS	NEG	POS	NEG	POS	POS	POS
Perth_2015-S20603			2015		Virtual hybr. (study isol.)	SCC [mec III+Cd] (JKD6008)	POS	POS	NEG	POS	NEG	POS	POS	POS
Perth_2015-S20607			2015		Virtual hybr. (study isol.)	SCC [mec III+Cd] (JKD6008)	POS	POS	NEG	POS	NEG	POS	POS	POS
Perth_2015-S20608			2015		Virtual hybr. (study isol.)	SCC [mec III+Cd] (JKD6008)	POS	POS	NEG	POS	NEG	POS	POS	POS
Perth_2015-S20610			2015		Virtual hybr. (study isol.)	SCC [mec III+Cd] (JKD6008)	POS	POS	NEG	POS	NEG	POS	POS	POS
Perth_2015-S20613			2015		Virtual hybr. (study isol.)	SCC [mec III+Cd] (JKD6008)	POS	POS	NEG	POS	NEG	POS	POS	POS
Perth_2015-S20617			2015		Virtual hybr. (study isol.)	SCC [mec III+Cd] (JKD6008)	POS	POS	NEG	POS	NEG	POS	POS	POS
Perth_2015-S20622			2015		Virtual hybr. (study isol.)	SCC [mec III+Cd] (JKD6008)	POS	POS	NEG	POS	NEG	POS	POS	POS
Perth_2015-S23619			2015		Virtual hybr. (study isol.)	SCC [mec III+Cd] (JKD6008)	POS	POS	NEG	POS	NEG	POS	POS	POS
Perth_2015-S41210			2015		Virtual hybr. (study isol.)	SCC [mec III+Cd] (JKD6008)	POS	POS	NEG	POS	NEG	POS	POS	POS
Perth_2015-S51001			2015		Virtual hybr. (study isol.)	SCC [mec III+Cd] (JKD6008)	POS	POS	NEG	POS	NEG	POS	POS	POS
Perth_2015-S51003			2015		Virtual hybr. (study isol.)	SCC [mec III+Cd] (JKD6008)	POS	POS	NEG	POS	NEG	POS	POS	POS
Perth_2015-S51006			2015		Virtual hybr. (study isol.)	SCC [mec III+Cd] (JKD6008)	POS	POS	NEG	POS	NEG	POS	POS	POS
Perth_2015-S51007			2015		Virtual hybr. (study isol.)	SCC [mec III+Cd] (JKD6008)	POS	POS	NEG	POS	NEG	POS	POS	POS
Perth_2015-S51008			2015		Virtual hybr. (study isol.)	SCC [mec III+Cd] (JKD6008)	POS	POS	NEG	POS	NEG	POS	POS	POS
Perth_2015-S51014			2015		Virtual hybr. (study isol.)	SCC [mec III+Cd] (JKD6008)	POS	POS	NEG	POS	NEG	POS	POS	POS
Perth_2015-S51408			2015		Virtual hybr. (study isol.)	SCC [mec III+Cd] (JKD6008)	POS	POS	NEG	POS	NEG	POS	POS	POS
Perth_2016-S20614			2016		Virtual hybr. (study isol.)	SCC [mec III+Cd] (JKD6008)	POS	POS	NEG	POS	NEG	POS	POS	POS
Perth_2016-S23614			2016		Virtual hybr. (study isol.)	SCC [mec III+Cd] (JKD6008)	POS	POS	NEG	POS	NEG	POS	POS	POS
Perth_2016-S42009			2016		Virtual hybr. (study isol.)	SCC [mec III+Cd] (JKD6008)	POS	POS	NEG	POS	NEG	POS	POS	POS
Perth_2016-S51001			2016		Virtual hybr. (study isol.)	SCC [mec III+Cd] (JKD6008)	POS	POS	NEG	POS	NEG	POS	POS	POS
Perth_2016-S51403			2016		Virtual hybr. (study isol.)	SCC [mec III+Cd] (JKD6008)	POS	POS	NEG	POS	NEG	POS	POS	POS
Perth_07 RPA 20			2007		Array hybr.	SCC [mec III+Cd] (JKD6008)	POS	POS	NEG	POS	NEG	POS	POS	POS
Perth_08-19088			2008		Array hybr.	SCC [mec III+Cd] (JKD6008)	POS	POS				POS	POS	POS
Perth_01-15351			2001		Array hybr.	SCC [mec III+Cd] (JKD6008)	POS	POS				POS	POS	POS
Perth_01-15357			2001		Array hybr.	SCC [mec III+Cd] (JKD6008)	POS	POS				POS	POS	POS
Perth_01-15419			2001		Array hybr.	SCC [mec III+Cd] (JKD6008)	POS	POS	NEG	POS	NEG	POS	POS	POS
Perth_01-16309			2001		Array hybr.	SCC [mec III+Cd] (JKD6008)	POS	POS				POS	POS	POS
Perth_03 SNP 95			2003		Array hybr.	SCC [mec III+Cd] (JKD6008)	POS	POS				POS	POS	POS
Perth_2003_AH_47			2003		Array hybr.	SCC [mec III+Cd] (JKD6008)	POS	POS				POS	POS	POS
**>"Australian/NZ Clade": ACME-pos. variant of JKD6008-Strain**														
Perth_2015-S20623			2015		Virtual hybr. (study isol.)	SCC [mec III+ACME2+Cd] (Unknown, ST239-1)	POS	POS	NEG	POS	NEG	POS	POS	POS
Perth_2015-S20826			2015		Virtual hybr. (study isol.)	SCC [mec III+ACME2+Cd] (Unknown, ST239-1)	POS	POS	NEG	POS	NEG	POS	POS	POS
Perth_05 NH 56			2005		Array hybr.	SCC [mec III+ACME2+Cd] (Unknown, ST239-1)	POS	POS	NEG	POS	NEG	POS	POS	POS
**>CC5-MRSA-III LA MRSA for comparison**														
Delbrück 38 isolate from domestic turkey				t002		SCC [mec III+Cd] (JKD6008)	POS	POS				POS	POS	POS
**> Staph. pseudintermedius KM1381 for comparison**														
KM1381	AM904732.1				Virtual hybr. (prev. publ.)		POS	POS	NEG	POS	NEG	POS	POS	POS
**>"South-East Asian Clade": TW20 (Irish AR44)**														
M92 (Canada)	CP015447.1	SAMN04903357			Virtual hybr. (prev. publ.)	SCC [mec III+Cd/Hg+ccrC] (TW20)	POS	POS	POS	NEG	NEG	POS	POS	POS
CHI59 (China)		SAMEA1029524	1998	t037	Virtual hybr. (prev. publ.)	SCC [mec III+Cd/Hg+ccrC] (TW20)	POS	POS	POS	NEG	NEG	POS	POS	POS
CHI61 (China)		SAMEA1029538	1998	t037	Virtual hybr. (prev. publ.)	SCC [mec III+Cd/Hg+ccrC] (TW20)	POS	POS	NEG	POS	NEG	POS	POS	POS
CUHK_HK1997 (China)	AZJQ	SAMN02438465			Virtual hybr. (prev. publ.)	SCC [mec III+Cd/Hg+ccrC] (TW20)	POS	POS	POS	NEG	NEG	POS	POS	POS
M996 (China)	CTXU	SAMEA862551			Virtual hybr. (prev. publ.)	SCC [mec III+Cd/Hg+ccrC] (TW20)	POS	POS	NEG	POS	NEG	POS	POS	POS
H211 (Denmark)	CTWW	SAMEA862528			Virtual hybr. (prev. publ.)	SCC [mec III+Cd/Hg+ccrC] (TW20)	POS	POS	POS	NEG	NEG	POS	POS	POS
H216 (Denmark)	CTXZ	SAMEA862587	2006		Virtual hybr. (prev. publ.)	SCC [mec III+Cd/Hg+ccrC] (TW20)	POS	POS	POS	NEG	NEG	POS	POS	POS
D71 (Germany)	CTXH	SAMEA862562			Virtual hybr. (prev. publ.)	SCC [mec III+Cd/Hg+ccrC] (TW20)	POS	POS	POS	NEG	NEG	POS	POS	POS
NMR05 (India)	LWAL	SAMN04621060	2014		Virtual hybr. (prev. publ.)	SCC [mec III+Cd/Hg+ccrC] (TW20)	POS	POS	NEG	POS	NEG	POS	POS	POS
MAL11 (Malaysia)	CTXM	SAMEA862543			Virtual hybr. (prev. publ.)	SCC [mec III+Cd/Hg+ccrC] (TW20)	POS	POS	POS	NEG	NEG	POS	POS	POS
MAL9 (Malaysia)	CTWZ	SAMEA862607			Virtual hybr. (prev. publ.)	SCC [mec III+Cd/Hg+ccrC] (TW20)	POS	POS	POS	NEG	NEG	POS	POS	POS
Na21 (Sri Lanka)	CTXN	SAMEA862544			Virtual hybr. (prev. publ.)	SCC [mec III+Cd/Hg+ccrC] (TW20)	POS	POS	NEG	POS	NEG	POS	POS	POS
M592 (Syria)	CTXT	SAMEA862550			Virtual hybr. (prev. publ.)	SCC [mec III+Cd/Hg+ccrC] (TW20)	POS	POS	NEG	POS	NEG	POS	POS	POS
H202 (Thailand)	CTYD	SAMEA862584			Virtual hybr. (prev. publ.)	SCC [mec III+Cd/Hg+ccrC] (TW20)	POS	POS	POS	NEG	NEG	POS	POS	POS
S102 (Thailand)		SAMEA1029519	2007		Virtual hybr. (prev. publ.)	SCC [mec III+Cd/Hg+ccrC] (TW20)	POS	POS	POS	NEG	NEG	POS	POS	POS
S130 (Thailand)		SAMEA1029516	2007		Virtual hybr. (prev. publ.)	SCC [mec III+Cd/Hg+ccrC] (TW20)	POS	POS	POS	NEG	NEG	POS	POS	POS
S40 (Thailand)		SAMEA1029528	2006		Virtual hybr. (prev. publ.)	SCC [mec III+Cd/Hg+ccrC] (TW20)	POS	POS	POS	NEG	NEG	POS	POS	POS
S71 (Thailand)		SAMEA1029541	2007		Virtual hybr. (prev. publ.)	SCC [mec III+Cd/Hg+ccrC] (TW20)	POS	POS	POS	NEG	NEG	POS	POS	POS
S87 (Thailand)		SAMEA1029512	2007		Virtual hybr. (prev. publ.)	SCC [mec III+Cd/Hg+ccrC] (TW20)	POS	POS	POS	NEG	NEG	POS	POS	POS
S93 (Thailand)		SAMEA1029560	2007		Virtual hybr. (prev. publ.)	SCC [mec III+Cd/Hg+ccrC] (TW20)	POS	POS	POS	NEG	NEG	POS	POS	POS
US_002 (Thailand)	FQJD	SAMEA3448943			Virtual hybr. (prev. publ.)	SCC [mec III+Cd/Hg+ccrC] (TW20)	POS	POS	POS	NEG	NEG	POS	POS	POS
US_017 (Thailand)	FQJP	SAMEA3448955			Virtual hybr. (prev. publ.)	SCC [mec III+Cd/Hg+ccrC] (TW20)	POS	POS	POS	NEG	NEG	POS	POS	POS
WAMC6102 (Thailand)	JBGY	SAMN02381744			Virtual hybr. (prev. publ.)	SCC [mec III+Cd/Hg+ccrC] (TW20)	POS	POS	POS	NEG	NEG	POS	POS	POS
NCTC_13626_TW20 (UK)	FN433596.1	SAMEA2272282		t037	Virtual hybr. (prev. publ.)	SCC [mec III+Cd/Hg+ccrC] (TW20)	POS	POS	POS	NEG	NEG	POS	POS	POS
BSAC3021 (UK)	FFPZ	SAMEA1464569			Virtual hybr. (prev. publ.)	SCC [mec III+Cd/Hg+ccrC] (TW20)	POS	POS	POS	NEG	NEG	POS	POS	POS
BSAC3046 (UK)	FGZX	SAMEA1464296			Virtual hybr. (prev. publ.)	SCC [mec III+Cd/Hg+ccrC] (TW20)	POS	POS	POS	NEG	NEG	POS	POS	POS
1193_SAUR (USA)	JVZL	SAMN03197153			Virtual hybr. (prev. publ.)	SCC [mec III+Cd/Hg+ccrC] (TW20)	POS	POS	POS	NEG	NEG	POS	POS	POS
1194_SAUR (USA)	JVZK	SAMN03197154			Virtual hybr. (prev. publ.)	SCC [mec III+Cd/Hg+ccrC] (TW20)	POS	POS	POS	NEG	NEG	POS	POS	POS

STRAIN / ISOLATE	Gen Bank	Bio sample Accession Number	Year of isolation	RIDOM spa	Virtual or real hybridisation	SCC*mec* type	METHICILLIN RESISTANCE AND SCCmec TYPING							
							ugpQ	mecA				delta_mecR	mecR	mecI
							ugpQ	mecA	mecA CP000046.1 SaurCOL	mecA BA000018 SaurN315	mecA GQ902038 Saur_haem pseud	Delta mecR1	mecR1	mecI
							Glycerophosphoryl diester phospho-diesterase.	Modified penicillin binding protein (PBP2a)				Truncated methicillin resistance operon repressor 1. Truncated mecR1 is present in SCCmec I, IV, V, VI, VII; complete absence of mecR1 from SCCmec	Methicillin resistance operon repressor 1. Un-truncated sequence in SCCmec II, SCCmec III, SCCmec VIII	Methicillin-resistance regulatory protein. Present in SCCmec II (although absent from Irish SCCmec II variants C and E), SCCmec III, SCCmec VIII
>"South-East Asian Clade": TW20 (Irish AR44), continued														
1195_SAUR (USA)	JVZJ	SAMN03197155	N/A		Virtual hybr. (prev. publ.)	SCC [mec III+Cd/Hg+ccrC] (TW20)	POS	POS	POS	NEG	NEG	POS	POS	POS
FVRH6002 (USA)	JBSL	SAMN02381511	N/A		Virtual hybr. (prev. publ.)	SCC [mec III+Cd/Hg+ccrC] (TW20)	POS	POS	POS	NEG	NEG	POS	POS	POS
KINW6048 (USA)	JBEK	SAMN02381844			Virtual hybr. (prev. publ.)	SCC [mec III+Cd/Hg+ccrC] (TW20)	POS	POS	POS	NEG	NEG	POS	POS	POS
LAMC0011 (USA)	JBRH	SAMN02381541			Virtual hybr. (prev. publ.)	SCC [mec III+Cd/Hg+ccrC] (TW20)	POS	POS	POS	NEG	NEG	POS	POS	POS
SJOS6053 (USA)	JBOE	SAMN02381426			Virtual hybr. (prev. publ.)	SCC [mec III+Cd/Hg+ccrC] (TW20)	POS	POS	POS	NEG	NEG	POS	POS	POS
SJOS6072 (USA)	JBNV	SAMN02381435	N/A		Virtual hybr. (prev. publ.)	SCC [mec III+Cd/Hg+ccrC] (TW20)	POS	POS	POS	NEG	NEG	POS	POS	POS
UCIM6042 (USA)	JBPU	SAMN02381592			Virtual hybr. (prev. publ.)	SCC [mec III+Cd/Hg+ccrC] (TW20)	POS	POS	POS	NEG	NEG	POS	POS	POS
Perth_2015-S31902			2015		Virtual hybr. (study isol.)	(SCC [mec III+Cd/Hg+ccrC] (TW20))	POS	POS	NEG	NEG	NEG	NEG	NEG	NEG
Perth_2015-S31904			2015		Virtual hybr. (study isol.)	SCC [mec III+Cd/Hg+ccrC] (TW20)	POS	POS	POS	NEG	NEG	POS	POS	POS
Perth_2015-S31913			2015		Virtual hybr. (study isol.)	SCC [mec III+Cd/Hg+ccrC] (TW20)	POS	POS	POS	NEG	NEG	POS	POS	POS
Perth_2015-S31918			2015		Virtual hybr. (study isol.)	(SCC [mec III+Cd/Hg+ccrC] (TW20))	POS	POS	POS	NEG	NEG	NEG	NEG	NEG
Dublin-DSH_AR44_E1183			2002	t037	Array hybr.	SCC [mec III+Cd/Hg+ccrC] (TW20)	POS	POS				POS	POS	POS
Dublin-DSH_Unfamiliar-3_E1520			2003	t037	Array hybr.	SCC [mec III+Cd/Hg+ccrC] (TW20)	POS	POS				POS	POS	POS
Hong Kong_042			2009-2010	t037	Array hybr.	SCC [mec III+Cd/Hg+ccrC] (TW20)	POS	POS				POS	POS	POS
Hong Kong_101			2009-2010		Array hybr.	SCC [mec III+Cd/Hg+ccrC] (TW20)	POS	POS				POS	POS	POS
Hong Kong_111			2009-2010		Array hybr.	SCC [mec III+Cd/Hg+ccrC] (TW20)	POS	POS				POS	POS	POS
Hong Kong_113			2009-2010		Array hybr.	SCC [mec III+Cd/Hg+ccrC] (TW20)	POS	POS				POS	POS	POS
Hong Kong_115			2009-2010		Array hybr.	SCC [mec III+Cd/Hg+ccrC] (TW20)	POS	POS				POS	POS	POS
Hong Kong_118			2009-2010		Array hybr.	SCC [mec III+Cd/Hg+ccrC] (TW20)	POS	POS				POS	POS	POS
Hong Kong_125			2009-2010		Array hybr.	SCC [mec III+Cd/Hg+ccrC] (TW20)	POS	POS				POS	POS	POS
Hong Kong_129			2009-2010		Array hybr.	SCC [mec III+Cd/Hg+ccrC] (TW20)	POS	POS				POS	POS	POS
Hong Kong_133			2009-2010		Array hybr.	SCC [mec III+Cd/Hg+ccrC] (TW20)	POS	POS				POS	POS	POS
Kuwait_009			1999		Array hybr.	SCC [mec III+Cd/Hg+ccrC] (TW20)	POS	POS				POS	POS	POS
Kuwait_103			1996		Array hybr.	SCC [mec III+Cd/Hg+ccrC] (TW20)	POS	POS				POS	POS	POS
Kuwait_107			1996		Array hybr.	SCC [mec III+Cd/Hg+ccrC] (TW20)	POS	POS				POS	POS	POS
Perth_03 AH 88			2003		Array hybr.	SCC [mec III+Cd/Hg+ccrC] (TW20)	POS	POS				POS	POS	POS
Perth_08-16905			2008		Array hybr.	SCC [mec III+Cd/Hg+ccrC] (TW20)	POS	POS				POS	POS	POS
Riyadh_Alfaisal-10_SICU_13_B5672_1117353			2014		Array hybr.	SCC [mec III+Cd/Hg+ccrC] (TW20)	POS	POS				POS	POS	POS
Riyadh_SSTI_17_3508602			2011-2012		Array hybr.	SCC [mec III+Cd/Hg+ccrC] (TW20)	POS	POS				POS	POS	POS
Riyadh-2822088-R			2010-2011		Array hybr.	SCC [mec III+Cd/Hg+ccrC] (TW20)	POS	POS	POS	NEG	NEG	POS	POS	POS
Trinidad&Tobago_2012_195 (619/12)			2012		Array hybr.	SCC [mec III+Cd/Hg+ccrC] (TW20)	POS	POS				POS	POS	POS
Trinidad&Tobago_2012_370 (1112-52177)			2012		Array hybr.	SCC [mec III+Cd/Hg+ccrC] (TW20)	POS	POS				POS	POS	POS
Trinidad&Tobago_2012_378 (4139)			2012		Array hybr.	SCC [mec III+Cd/Hg+ccrC] (TW20)	POS	POS				POS	POS	POS
Trinidad&Tobago_2013_PA21			2013		Array hybr.	SCC [mec III+Cd/Hg+ccrC] (TW20)	POS	POS				POS	POS	POS
Trinidad&Tobago_2013_PA22			2013		Array hybr.	SCC [mec III+Cd/Hg+ccrC] (TW20)	POS	POS				POS	POS	POS
Trinidad&Tobago_2912_340 (8523/12)			2012		Array hybr.	SCC [mec III+Cd/Hg+ccrC] (TW20)	POS	POS				POS	POS	POS
Trinidad&Tobago_MRSA2010_105			2010		Array hybr.	SCC [mec III+Cd/Hg+ccrC] (TW20)	POS	POS				POS	POS	POS
Trinidad&Tobago_MRSA2010_108			2010		Array hybr.	SCC [mec III+Cd/Hg+ccrC] (TW20)	POS	POS				POS	POS	POS
Trinidad&Tobago_MRSA2010_33			2010		Array hybr.	SCC [mec III+Cd/Hg+ccrC] (TW20)	POS	POS				POS	POS	POS
Trinidad&Tobago_MRSA2010_79			2010		Array hybr.	SCC [mec III+Cd/Hg+ccrC] (TW20)	POS	POS				POS	POS	POS
Trinidad&Tobago_MRSA2010_88			2010		Array hybr.	SCC [mec III+Cd/Hg+ccrC] (TW20)	POS	POS				POS	POS	POS
Trinidad&Tobago_MRSA2010_97			2010		Array hybr.	SCC [mec III+Cd/Hg+ccrC] (TW20)	POS	POS				POS	POS	POS
Trinidad&Tobago_SSTI 2012_031			2010		Array hybr.	SCC [mec III+Cd/Hg+ccrC] (TW20)	POS	POS				POS	POS	POS
Uganda-29_17148_250811			2011		Array hybr.	SCC [mec III+Cd/Hg+ccrC] (TW20)	POS	POS				POS	POS	POS
>"South-East Asian Clade": Strain with a TW20-derived pseudoelement														
UCIM6015	JBMO	SAMN02381468	N/A		Virtual hybr. (prev. publ.)	PseudoSCCmec [class A+Cd/Hg+ccrC] (UCIM6015)	POS	POS	POS	NEG	NEG	POS	POS	POS
Hong Kong_121			2009-2010		Array hybr.	PseudoSCCmec [class A+Cd/Hg+ccrC] (UCIM6015)	POS	POS				POS	POS	POS
>"South-East Asian Clade": Z172														
Z172 (Taiwan)	CP006838.1	SAMN02370325	2010	t037	Virtual hybr. (prev. publ.)	SCC [mec III+Cd/Hg+ccrC] (Z172)	POS	POS	POS	NEG	NEG	POS	POS	POS
Russia-19_0342_Kurgan			2012		Array hybr.	SCC [mec III+Cd/Hg+ccrC] (Z172)	POS	POS				POS	POS	POS
>"South-East Asian Clade": mvaS-deletion variant of Z1721														
Perth_01-15392			2001		Array hybr.	SCC [mec III+Cd/Hg+ccrC] (Unknown ST239)	POS	POS	POS	NEG	NEG	POS	POS	POS
>"South-East Asian Clade": XN108														
XN108 (China)	CP007447.1	SAMN02693431	2004		Virtual hybr. (prev. publ.)	SCC [mec III+Cd+ccrC] (XN108)	POS	POS	POS	NEG	NEG	POS	POS	POS
NMR09 (India)	LWBY				Virtual hybr. (prev. publ.)	SCC [mec III+Cd+ccrC] (XN108)	POS	POS	POS	NEG	NEG	POS	POS	POS
346 (Malaysia)	JQNU	SAMN02978821	2013		Virtual hybr. (prev. publ.)	SCC [mec III+Cd+ccrC] (XN108)	POS	POS	POS	NEG	NEG	POS	POS	POS
DS_009 (Thailand)	FQFW	SAMEA3448838			Virtual hybr. (prev. publ.)	SCC [mec III+Cd+ccrC] (XN108)	POS	POS	POS	NEG	NEG	POS	POS	POS
18ANR581005 (Ind)			2018		Array hybr.	SCC [mec III+Cd+ccrC] (XN108)	POS	POS				POS	POS	POS
Bengalaru_ST20141405			2012		Array hybr.	SCC [mec III+Cd+ccrC] (XN108)	POS	POS				POS	POS	POS
Bengalaru_ST20141406			2012		Array hybr.	SCC [mec III+Cd+ccrC] (XN108)	POS	POS				POS	POS	POS
Bengalaru_ST20141417			2012		Array hybr.	SCC [mec III+Cd+ccrC] (XN108)	POS	POS				POS	POS	POS
Hong Kong_107			2009-2010		Array hybr.	SCC [mec III+Cd+ccrC] (XN108)	POS	POS				POS	POS	POS
Hong Kong_12			2009-2010		Array hybr.	SCC [mec III+Cd+ccrC] (XN108)	POS	POS				POS	POS	POS
Hong Kong_132			2009-2010		Array hybr.	SCC [mec III+Cd+ccrC] (XN108)	POS	POS				POS	POS	POS
Riyadh-3028763-R			2010-2011		Array hybr.	SCC [mec III+Cd+ccrC] (XN108)	POS	POS				POS	POS	POS
Trinidad&Tobago_MRSA2010_94			2010		Array hybr.	SCC [mec III+Cd+ccrC] (XN108)	POS	POS				POS	POS	POS
>"South-East Asian Clade": S85														
M705 (Thailand)	CTWU	SAMEA862613	2007		Virtual hybr. (prev. publ.)	SCC [mec III+Cd+ccrC] (S85)	POS	POS	POS	NEG	NEG	POS	POS	POS
S85 (Thailand)		SAMEA1029529	2007		Virtual hybr. (prev. publ.)	SCC [mec III+Cd+ccrC] (S85)	POS	POS	POS	NEG	NEG	POS	POS	POS
Riyadh_Alfaisal-04_281014_S5076			2014		Array hybr.	SCC [mec III+Cd+ccrC] (S85)	POS	POS	POS	NEG	NEG	POS	POS	POS
Riyadh_Alfaisal-26_S15145_1104163			2014		Array hybr.	SCC [mec III+Cd+ccrC] (S85)	POS	POS				POS	POS	POS
Riyadh-2817437-W			2010-2011		Array hybr.	SCC [mec III+Cd+ccrC] (S85)	POS	POS				POS	POS	POS
>"South-East Asian Clade": PPUKM-775-2009														
PPUKM-775-2009 (Malaysia)	AMRE	SAMN02471404	2009	incomplete	Virtual hybr. (prev. publ.)	SCC [mec III+Cd+ccrC] (PPUKM-775-2009)	POS	POS	POS	NEG	NEG	POS	POS	POS
Hong Kong_138					Array hybr.	SCC [mec III+Cd+ccrC] (PPUKM-775-2009)	POS	POS				POS	POS	POS
>"South-East Asian Clade": sasX-pos. mer+/ccrC neg. isolates from Kuwait														
Kuwait_001			2002		Array hybr.	SCC [mec III+Hg] (Unknown, ST239)	POS	POS	POS	NEG	NEG	POS	POS	POS
Kuwait_337			2010		Array hybr.	SCC [mec III+Hg] (Unknown, ST239)	POS	POS				POS	POS	POS
>"South-East Asian Clade": sasX-pos. strain with a Bmb9393-like SCC element (AUS-EMRSA-3)														
CUHK_HK2007 (China)	AZMZ	SAMN02442046	2007		Virtual hybr. (prev. publ.)	SCC [mec III+Cd/Hg+ccrC] (Bmb9393)	POS	POS	POS	NEG	NEG	POS	POS	POS
NMR02 (India)	LWAK	SAMN04620659			Virtual hybr. (prev. publ.)	SCC [mec III+Cd/Hg+ccrC] (Bmb9393)	POS	POS	POS	NEG	NEG	POS	POS	POS
VB1490 (India)	MLQB	SAMN05908416	2015		Virtual hybr. (prev. publ.)	SCC [mec III+Cd/Hg+ccrC] (Bmb9393)	POS	POS	POS	NEG	NEG	POS	POS	POS
V521 (Korea)	CP013957.1	SAMN04219577			Virtual hybr. (prev. publ.)	SCC [mec III+Cd/Hg+ccrC] (Bmb9393)	POS	POS	POS	NEG	NEG	POS	POS	POS
HST-077 (Lebanon)	AVPR	SAMN02744534	2011		Virtual hybr. (prev. publ.)	SCC [mec III+Cd/Hg+ccrC] (Bmb9393)	POS	POS	POS	NEG	NEG	POS	POS	POS
MAL1 (Malaysia)	CTXL	SAMEA862552			Virtual hybr. (prev. publ.)	SCC [mec III+Cd/Hg+ccrC] (Bmb9393)	POS	POS	POS	NEG	NEG	POS	POS	POS
MAL3 (Malaysia)	CTWV	SAMEA862609	1996		Virtual hybr. (prev. publ.)	SCC [mec III+Cd/Hg+ccrC] (Bmb9393)	POS	POS	POS	NEG	NEG	POS	POS	POS
PPUKM-261-2009 (Malaysia)	AMRB	SAMN02471406			Virtual hybr. (prev. publ.)	SCC [mec III+Cd/Hg+ccrC] (Bmb9393)	POS	POS	POS	NEG	NEG	POS	POS	POS
PPUKM-332-2009 (Malaysia)	AMRC	SAMN02471407		incomplete	Virtual hybr. (prev. publ.)	SCC [mec III+Cd/Hg+ccrC] (Bmb9393)	POS	POS	POS	NEG	NEG	POS	POS	POS
S38 (Thailand)		SAMEA1029568	2006		Virtual hybr. (prev. publ.)	SCC [mec III+Cd/Hg+ccrC] (Bmb9393)	POS	POS	POS	NEG	NEG	POS	POS	POS
S7 (Thailand)		SAMEA1029556	2006		Virtual hybr. (prev. publ.)	SCC [mec III+Cd/Hg+ccrC] (Bmb9393)	POS	POS	POS	NEG	NEG	POS	POS	POS
US_008 (Thailand)	FQJI	SAMEA3448948			Virtual hybr. (prev. publ.)	SCC [mec III+Cd/Hg+ccrC] (Bmb9393)	POS	POS	POS	NEG	NEG	POS	POS	POS
US_030 (Thailand)	FQJW	SAMEA3448965			Virtual hybr. (prev. publ.)	SCC [mec III+Cd/Hg+ccrC] (Bmb9393)	POS	POS	POS	NEG	NEG	POS	POS	POS
M170 (Tsunami-related)	CVRW	SAMEA862547	2005		Virtual hybr. (prev. publ.)	SCC [mec III+Cd/Hg+ccrC] (Bmb9393)	POS	POS	POS	NEG	NEG	POS	POS	POS
BSAC697 (UK)	FGBR	SAMEA1464583	2003		Virtual hybr. (prev. publ.)	SCC [mec III+Cd/Hg+ccrC] (Bmb9393)	POS	POS	POS	NEG	NEG	POS	POS	POS
IS-189	AICJ	SAMN00138230		incomplete	Virtual hybr. (prev. publ.)	SCC [mec III+Cd/Hg+ccrC] (Bmb9393)	POS	POS	POS	NEG	NEG	POS	POS	POS
MRGR3	AHZL	SAMN02470733		t037	Virtual hybr. (prev. publ.)	SCC [mec III+Cd/Hg+ccrC] (Bmb9393)	POS	POS	POS	NEG	NEG	POS	POS	POS
smg07	NESW	SAMN06842170			Virtual hybr. (prev. publ.)	SCC [mec III+Cd/Hg+ccrC] (Bmb9393)	POS	POS	POS	NEG	NEG	POS	POS	POS
AH1-AUS-EMRSA-3					Array hybr.	SCC [mec III+Cd/Hg+ccrC] (Bmb9393)	POS	POS	POS	NEG	NEG	POS	POS	POS
Bengalaru_ST20141403			2014		Array hybr.	SCC [mec III+Cd/Hg+ccrC] (Bmb9393)	POS	POS				POS	POS	POS
Finland E24_98541_Harmony			1998		Array hybr.	SCC [mec III+Cd/Hg+ccrC] (Bmb9393)	POS	POS				POS	POS	POS
Hong Kong_10			2009-2010	t037	Array hybr.	SCC [mec III+Cd/Hg+ccrC] (Bmb9393)	POS	POS				POS	POS	POS
Hong Kong_100			2009-2010		Array hybr.	SCC [mec III+Cd/Hg+ccrC] (Bmb9393)	POS	POS				POS	POS	POS
Hong Kong_199			2009-2010		Array hybr.	SCC [mec III+Cd/Hg+ccrC] (Bmb9393)	POS	POS				POS	POS	POS
Hong Kong_34			2009-2010	t037	Array hybr.	SCC [mec III+Cd/Hg+ccrC] (Bmb9393)	POS	POS				POS	POS	POS
Hong Kong_88			2009-2010		Array hybr.	SCC [mec III+Cd/Hg+ccrC] (Bmb9393)	POS	POS				POS	POS	POS
Hong Kong_92			2009-2010		Array hybr.	SCC [mec III+Cd/Hg+ccrC] (Bmb9393)	POS	POS				POS	POS	POS
Hong Kong_94			2009-2010		Array hybr.	SCC [mec III+Cd/Hg+ccrC] (Bmb9393)	POS	POS				POS	POS	POS
Kuwait_257			2010		Array hybr.	SCC [mec III+Cd/Hg+ccrC] (Bmb9393)	POS	POS				POS	POS	POS
Perth_01-15330			2001		Array hybr.	SCC [mec III+Cd/Hg+ccrC] (Bmb9393)	POS	POS				POS	POS	POS
Perth_01-15349			2001		Array hybr.	SCC [mec III+Cd/Hg+ccrC] (Bmb9393)	POS	POS				POS	POS	POS
Perth_01-16176			2001		Array hybr.	SCC [mec III+Cd/Hg+ccrC] (Bmb9393)	POS	POS				POS	POS	POS
Perth_WBG8379					Array hybr.	SCC [mec III+Cd/Hg+ccrC] (Bmb9393)	POS	POS				POS	POS	POS
Rawalpindi_Kidney Center_56			2017		Array hybr.	SCC [mec III+Cd/Hg+ccrC] (Bmb9393)	POS	POS				POS	POS	POS
Riyadh_SSTI_05_3464614			2011-2012		Array hybr.	SCC [mec III+Cd/Hg+ccrC] (Bmb9393)	POS	POS				POS	POS	POS
Trinidad&Tobago_2012_157			2012		Array hybr.	SCC [mec III+Cd/Hg+ccrC] (Bmb9393)	POS	POS				POS	POS	POS

STRAIN / ISOLATE	Gen Bank	Bio sample Accession Number	Year of isolation	RIDOM spa	Virtual or real hybridisation	SCC*mec* type	METHICILLIN RESISTANCE AND SCCmec TYPING							
							ugpQ	mecA				delta_mecR	mecR	mecI
							ugpQ	mecA	mecA CP000046.1 SaurCOL	mecA BA000018 SaurN315	mecA GQ902038 Saur_haem pseud	Delta mecR1	mecR1	mecI
							Glycerophosphoryl diester phospho-diesterase.	Modified penicillin binding protein (PBP2a)				Truncated methicillin resistance operon repressor 1. Truncated mecR1 is present in SCCmec I, IV, V, VI, VII; complete absence of [illegible]	Methicillin resistance operon repressor 1. Un-truncated sequence in SCCmec II, SCCmec III, SCCmec VIII	Methicillin-resistance regulatory protein. Present in SCCmec II (although absent from Irish SCCmec II variants C and E), SCCmec III, [illegible]
>"South-East Asian Clade": M418														
M418	CTXS	SAMEA862549	2006		Virtual hybr. (prev. publ.)	SCC [mec III+Cd/Hg+ccrC] (M418/UK-9)	POS	POS	POS	NEG	NEG	POS	POS	POS
>"South-East Asian Clade": NMR08														
NMR07 (India)	LWAM	SAMN04621061	2014		Virtual hybr. (prev. publ.)	SCC [mec III+Cd+ccrC] (NMR08)	POS	POS	POS	NEG	NEG	POS	POS	POS
NMR08 (India)	CP023560.1	SAMN04621062	2013		Virtual hybr. (prev. publ.)	SCC [mec III+Cd+ccrC] (NMR08)	POS	POS	POS	NEG	NEG	POS	POS	POS
Bengalaru_ST20121978			2006-2012		Array hybr.	SCC [mec III+Cd+ccrC] (NMR08)	POS	POS				POS	POS	POS
Hong Kong_89			2009-2010		Array hybr.	SCC [mec III+Cd+ccrC] (NMR08)	POS	POS	POS	NEG	NEG	POS	POS	POS
>"South-East Asian Clade": sasX-pos. ACME-pos. Strain														
Perth_2005_AH_5			2005		Array hybr.	SCC [mec III+ACME2+Cd/Hg] (Unknown, ST239)	POS	POS	POS	NEG	NEG	POS	POS	POS
>"South-East Asian Clade": sasX-pos/czrC-pos. strains														
Trinidad&Tobago_MRSA2010_103			2010		Array hybr.	SCC [mec III+speG+Cd/czrC+ccrAB4C] (Unknown, ST239)	POS	POS				POS	POS	POS
Trinidad&Tobago_2013_PA25			2013		Array hybr.	SCC [mec III+speG+Cd/Hg/czrC+ccrAB4C] (Unknown, ST239)	POS	POS	POS	NEG	NEG	POS	POS	POS
Trinidad&Tobago_MRSA2010_110			2010		Array hybr.	SCC [mec III+speG+Cd/Hg/czrC+ccrAB4C] (Unknown, ST239)	POS	POS				POS	POS	POS
Trinidad&Tobago_SSTI 2012_111			2012		Array hybr.	SCC [mec III+speG+Cd/Hg/czrC+ccrAB4C] (Unknown, ST239)	POS	POS				POS	POS	POS
Trinidad&Tobago_MRSA2010_158			2010		Array hybr.	SCC [mec III+speG+Cd/czrC+ccrAB4C] (Unknown, ST239)	POS	POS	POS	NEG	NEG	POS	POS	POS
>"South-East Asian Clade": sasX-pos. sporadic strains from Kuwait and Hongkong														
Kuwait_122			1998		Array hybr.	SCC [mec III+Cd/Hg] (Unknown, ST239-1)	POS	POS	POS	NEG	NEG	POS	POS	POS
Kuwait_002			2005		Array hybr.	SCC [mec III+Cd/Hg] (Unknown, ST239-1)	POS	POS				POS	POS	POS
Kuwait_196			2010		Array hybr.	SCC [mec III+Cd/Hg] (Unknown, ST239-2)	POS	POS	POS	NEG	NEG	POS	POS	POS
Kuwait_199			2010		Array hybr.	SCC [mec III+Cd/Hg] (Unknown, ST239-2)	POS	POS				POS	POS	POS
Hong Kong_136			2009-2010		Array hybr.	SCC [mec III+Cd/Hg] (Unknown, ST239-1)	POS	POS				POS	POS	POS
>"South-East Asian Clade": P32														
P32 (Poland)	CTXV	SAMEA862589	1996		Virtual hybr. (prev. publ.)	SCC [mec III+Cd] (HSA10/ATCC33592)	POS	POS	NEG	POS	NEG	POS	POS	POS
>"South-East Asian Clade": ACME-pos. strain similar to P32														
Perth_2015-S32313			2015		Virtual hybr. (study isol.)	SCC [mec III+ACME2+Cd] (Unknown, ST239-2)	POS	POS	POS	NEG	NEG	POS	POS	POS
>"hla-negative South-East Asian Clade": DEN907														
DEN907 (Denmark)		SAMEA1029548	2001	t037	Virtual hybr. (prev. publ.)	SCC [mec III+Cd/Hg] (DEN907)	POS	POS	POS	NEG	NEG	POS	POS	POS
>"hla-negative South-East Asian Clade": S2/M116														
S26 (Thailand)		SAMEA1029511	2006		Virtual hybr. (prev. publ.)	SCC [mec III+Cd] (S2)	POS	POS	POS	NEG	NEG	POS	POS	POS
S42 (Thailand)		SAMEA1029515	2006		Virtual hybr. (prev. publ.)	SCC [mec III+Cd] (S2)	POS	POS	POS	NEG	NEG	POS	POS	POS
S97 (Thailand)		SAMEA1029522	2007		Virtual hybr. (prev. publ.)	SCC [mec III+Cd] (S2)	POS	POS	POS	NEG	NEG	POS	POS	POS
S78 (Thailand)		SAMEA1029525	2007		Virtual hybr. (prev. publ.)	SCC [mec III+Cd] (S2)	POS	POS	POS	NEG	NEG	POS	POS	POS
S25 (Thailand)		SAMEA1029535	2006		Virtual hybr. (prev. publ.)	SCC [mec III+Cd] (S2)	POS	POS	POS	NEG	NEG	POS	POS	POS
S21 (Thailand)		SAMEA1029536	2006		Virtual hybr. (prev. publ.)	SCC [mec III+Cd] (S2)	POS	POS	POS	NEG	NEG	POS	POS	POS
S106 (Thailand)		SAMEA1029547	2007		Virtual hybr. (prev. publ.)	SCC [mec III+Cd] (S2)	POS	POS	POS	NEG	NEG	POS	POS	POS
S24 (Thailand)		SAMEA1029553	2006		Virtual hybr. (prev. publ.)	SCC [mec III+Cd] (S2)	POS	POS	POS	NEG	NEG	POS	POS	POS
S39 (Thailand)		SAMEA1029558	2006		Virtual hybr. (prev. publ.)	SCC [mec III+Cd] (S2)	POS	POS	POS	NEG	NEG	POS	POS	POS
S2 (Thailand)		SAMEA1029563	2006		Virtual hybr. (prev. publ.)	SCC [mec III+Cd] (S2)	POS	POS	POS	NEG	NEG	POS	POS	POS
S81 (Thailand)		SAMEA1029565	2007		Virtual hybr. (prev. publ.)	SCC [mec III+Cd] (S2)	POS	POS	POS	NEG	NEG	POS	POS	POS
M116 (Vietnam)	CTXQ	SAMEA862545			Virtual hybr. (prev. publ.)	SCC [mec III+Cd] (S2)	POS	POS	NEG	POS	NEG	POS	POS	POS
>"Portuguese Clade": Portuguese & ATCC 33592 strain														
FFP103 (Portugal)		SAMEA1029533	1990	t421	Virtual hybr. (prev. publ.)	SCC [mec III+Cd] (HSA10/ATCC33592)	POS	POS	POS	NEG	NEG	POS	POS	POS
HDG2 (Portugal)		SAMEA1029561	1992	t421	Virtual hybr. (prev. publ.)	SCC [mec III+Cd] (HSA10/ATCC33592)	POS	POS	POS	NEG	NEG	POS	POS	POS
HSA10 (Portugal)		SAMEA1029520	1992	t421	Virtual hybr. (prev. publ.)	SCC [mec III+Cd] (HSA10/ATCC33592)	POS	POS	POS	NEG	NEG	POS	POS	POS
HSA11 (Portugal)		SAMEA1029542	1992	t388	Virtual hybr. (prev. publ.)	SCC [mec III+Cd] (HSA10/ATCC33592)	POS	POS	POS	NEG	NEG	POS	POS	POS
ICP5011 (Portugal)		SAMEA1029545	1993	t421	Virtual hybr. (prev. publ.)	SCC [mec III+Cd] (HSA10/ATCC33592)	POS	POS	POS	NEG	NEG	POS	POS	POS
ICP5014 (Portugal)		SAMEA1029543	1993	t421	Virtual hybr. (prev. publ.)	SCC [mec III+Cd] (HSA10/ATCC33592)	POS	POS	POS	NEG	NEG	POS	POS	POS
ICP5062 (Portugal)		SAMEA1029562	1993	t421	Virtual hybr. (prev. publ.)	SCC [mec III+Cd] (HSA10/ATCC33592)	POS	POS	POS	NEG	NEG	POS	POS	POS
Strain 1063_ATCC 33592 FDAARGOS_3 (USA)	JXZH	SAMN03255443	1981		Virtual hybr. (prev. publ.)	SCC [mec III+Cd] (HSA10/ATCC33592)	POS	POS	POS	NEG	NEG	POS	POS	POS
					Array hybr.		POS	POS				POS	POS	POS
Russia-07_0110_Kurgan			2011	t138	Array hybr.	SCC [mec III+Cd] (HSA10/ATCC33592)	POS	POS				POS	POS	POS
Russia-11_0164_Kurgan			2011	t037	Array hybr.	SCC [mec III+Cd] (HSA10/ATCC33592)	POS	POS				POS	POS	POS
Russia-20_0367_Kurgan			2012	t037	Array hybr.	SCC [mec III+Cd] (HSA10/ATCC33592)	POS	POS				POS	POS	POS
Russia-21_0390_Chelyabinsk			2012	t037	Array hybr.	SCC [mec III+Cd] (HSA10/ATCC33592)	POS	POS				POS	POS	POS
>"South American/ Middle Eastern Clade": Bmb9393/UK EMRSA-11														
AGT1 (Argentina)		SAMEA1029527	1997	t037	Virtual hybr. (prev. publ.)	SCC [mec III+Cd/Hg+ccrC] (Bmb9393)	POS	POS	POS	NEG	NEG	POS	POS	POS
AGT120 (Argentina)		SAMEA1029554	1998	t037	Virtual hybr. (prev. publ.)	SCC [mec III+Cd/Hg+ccrC] (Bmb9393)	POS	POS	POS	NEG	NEG	POS	POS	POS
AGT67 (Argentina)		SAMEA985416	1997	t037	Virtual hybr. (prev. publ.)	SCC [mec III+Cd/Hg+ccrC] (Bmb9393)	POS	POS	POS	NEG	NEG	POS	POS	POS
AGT9 (Argentina)		SAMEA1029509	1997	t037	Virtual hybr. (prev. publ.)	SCC [mec III+Cd/Hg+ccrC] (Bmb9393)	POS	POS	POS	NEG	NEG	POS	POS	POS
RA3 (Argentina)	CTXW	SAMEA862588			Virtual hybr. (prev. publ.)	SCC [mec III+Cd/Hg+ccrC] (Bmb9393)	POS	POS	POS	NEG	NEG	POS	POS	POS
RA6 (Argentina)	CTXX	SAMEA862591	1996		Virtual hybr. (prev. publ.)	SCC [mec III+Cd/Hg+ccrC] (Bmb9393)	POS	POS	POS	NEG	NEG	POS	POS	POS
RA7 (Argentina)	CTXC	SAMEA862616			Virtual hybr. (prev. publ.)	SCC [mec III+Cd/Hg+ccrC] (Bmb9393)	POS	POS	POS	NEG	NEG	POS	POS	POS
Be62 (Brazil)	CP012013.1	SAMN03765155			Virtual hybr. (prev. publ.)	SCC [mec III+Cd/Hg+ccrC] (Bmb9393)	POS	POS	POS	NEG	NEG	POS	POS	POS
Bmb9393 (Brazil)	CP005288.1	SAMN02603524		t138	Virtual hybr. (prev. publ.)	SCC [mec III+Cd/Hg+ccrC] (Bmb9393)	POS	POS	POS	NEG	NEG	POS	POS	POS
BRA36 (Brazil)		SAMEA1029559	1997	t037	Virtual hybr. (prev. publ.)	SCC [mec III+Cd/Hg+ccrC] (Bmb9393)	POS	POS	POS	NEG	NEG	POS	POS	POS
BZ48 (Brazil)		SAMEA1029508	1997	t037	Virtual hybr. (prev. publ.)	SCC [mec III+Cd/Hg+ccrC] (Bmb9393)	POS	POS	POS	NEG	NEG	POS	POS	POS
Gv51 (Brazil)	CP012015.1	SAMN03835466			Virtual hybr. (prev. publ.)	SCC [mec III+Cd/Hg+ccrC] (Bmb9393)	POS	POS	POS	NEG	NEG	POS	POS	POS
Gv69 (Brazil)	CP009681.1	SAMN03144721			Virtual hybr. (prev. publ.)	SCC [mec III+Cd/Hg+ccrC] (Bmb9393)	POS	POS	POS	NEG	NEG	POS	POS	POS
Gv88 (Brazil)	CP012018.1	SAMN03835488	1997		Virtual hybr. (prev. publ.)	SCC [mec III+Cd/Hg+ccrC] (Bmb9393)	POS	POS	POS	NEG	NEG	POS	POS	POS
HC1335 (Brazil)	CP012012.1	SAMN03835494			Virtual hybr. (prev. publ.)	SCC [mec III+Cd/Hg+ccrC] (Bmb9393)	POS	POS	POS	NEG	NEG	POS	POS	POS
HC1340 (Brazil)	CP012011.1	SAMN03835492			Virtual hybr. (prev. publ.)	SCC [mec III+Cd/Hg+ccrC] (Bmb9393)	POS	POS	POS	NEG	NEG	POS	POS	POS
HC556 (Brazil)	LDPC	SAMN03737803			Virtual hybr. (prev. publ.)	SCC [mec III+Cd/Hg+ccrC] (Bmb9393)	POS	POS	POS	NEG	NEG	POS	POS	POS
HU25 (Brazil)		SAMEA1029569	1993	t138	Virtual hybr. (prev. publ.)	SCC [mec III+Cd/Hg+ccrC] (Bmb9393)	POS	POS	POS	NEG	NEG	POS	POS	POS
UB563 (Brazil)	LGWS	SAMN03940750			Virtual hybr. (prev. publ.)	SCC [mec III+Cd/Hg+ccrC] (Bmb9393)	POS	POS	POS	NEG	NEG	POS	POS	POS
2A8 (Czech Republic)		SAMEA1029549	2001	t037	Virtual hybr. (prev. publ.)	SCC [mec III+Cd/Hg+ccrC] (Bmb9393)	POS	POS	POS	NEG	NEG	POS	POS	POS
M1229 (Denmark)	CTXO	SAMEA862546			Virtual hybr. (prev. publ.)	SCC [mec III+Cd/Hg+ccrC] (Bmb9393)	POS	POS	POS	NEG	NEG	POS	POS	POS
FRICAR (France)	CTXG	SAMEA862529			Virtual hybr. (prev. publ.)	SCC [mec III+Cd/Hg+ccrC] (Bmb9393)	POS	POS	POS	NEG	NEG	POS	POS	POS
D90 (Germany)	CTXY	SAMEA862585	1990		Virtual hybr. (prev. publ.)	SCC [mec III+Cd/Hg+ccrC] (Bmb9393)	POS	POS	POS	NEG	NEG	POS	POS	POS
LIT68 (Lithuania)	CTYC	SAMEA862586			Virtual hybr. (prev. publ.)	SCC [mec III+Cd/Hg+ccrC] (Bmb9393)	POS	POS	POS	NEG	NEG	POS	POS	POS
LIT76 (Lithuania)	CTYA	SAMEA862593			Virtual hybr. (prev. publ.)	SCC [mec III+Cd/Hg+ccrC] (Bmb9393)	POS	POS	POS	NEG	NEG	POS	POS	POS
HGSA142 (Portugal)		SAMEA1029517	2003	t037	Virtual hybr. (prev. publ.)	SCC [mec III+Cd/Hg+ccrC] (Bmb9393)	POS	POS	POS	NEG	NEG	POS	POS	POS
HGSA9 (Portugal)		SAMEA1029534	1997	NT	Virtual hybr. (prev. publ.)	SCC [mec III+Cd/Hg+ccrC] (Bmb9393)	POS	POS	POS	NEG	NEG	POS	POS	POS
HSJ216 (Portugal)		SAMEA1029550	1997	t037	Virtual hybr. (prev. publ.)	SCC [mec III+Cd/Hg+ccrC] (Bmb9393)	POS	POS	POS	NEG	NEG	POS	POS	POS
M278 (Portugal)	CTXP	SAMEA862548			Virtual hybr. (prev. publ.)	SCC [mec III+Cd/Hg+ccrC] (Bmb9393)	POS	POS	POS	NEG	NEG	POS	POS	POS
ES26 (Spain)	CTXI	SAMEA862530			Virtual hybr. (prev. publ.)	SCC [mec III+Cd/Hg+ccrC] (Bmb9393)	POS	POS	POS	NEG	NEG	POS	POS	POS
UK102 (UK)	CTYG	SAMEA862590			Virtual hybr. (prev. publ.)	SCC [mec III+Cd/Hg+ccrC] (Bmb9393)	POS	POS	POS	NEG	NEG	POS	POS	POS
URU34 (Uruguay)		SAMEA1029544	1997	t037	Virtual hybr. (prev. publ.)	SCC [mec III+Cd/Hg+ccrC] (Bmb9393)	POS	POS	POS	NEG	NEG	POS	POS	POS
IS-125	AHVC	SAMN00138228		incomplete	Virtual hybr. (prev. publ.)	SCC [mec III+Cd/Hg+ccrC] (Bmb9393)	POS	POS	POS	NEG	NEG	POS	POS	POS
IS-157	AICH	SAMN02436496		incomplete	Virtual hybr. (prev. publ.)	SCC [mec III+Cd/Hg+ccrC] (Bmb9393)	POS	POS	POS	NEG	NEG	POS	POS	POS
Ecuador_1			2009		Array hybr.	SCC [mec III+Cd/Hg+ccrC] (Bmb9393)	POS	POS				POS	POS	POS
Ecuador_2			2010		Array hybr.	SCC [mec III+Cd/Hg+ccrC] (Bmb9393)	POS	POS				POS	POS	POS
Dresden_15ANR574263			2015		Array hybr.	SCC [mec III+Cd/Hg+ccrC] (Bmb9393)	POS	POS	POS	NEG	NEG	POS	POS	POS
UK-EMRSA-11					Array hybr.	SCC [mec III+Cd/Hg+ccrC] (Bmb9393)	POS	POS				POS	POS	POS
>"South American/ Middle Eastern Clade": BRA2-Strain														
BRA2 (Brazil)		SAMEA1029514	1997	t037	Virtual hybr. (prev. publ.)	SCC [mec III+Cd/Hg] (BRA2)	POS	POS	POS	NEG	NEG	POS	POS	POS
CHL1 (Chile)		SAMEA1029526	1997	t037	Virtual hybr. (prev. publ.)	SCC [mec III+Cd/Hg] (BRA2)	POS	POS	POS	NEG	NEG	POS	POS	POS
CHL151 (Chile)		SAMEA1029539	1998	t037	Virtual hybr. (prev. publ.)	SCC [mec III+Cd/Hg] (BRA2)	POS	POS	POS	NEG	NEG	POS	POS	POS
UCL378 (Chile)	LGWM	SAMN03940744			Virtual hybr. (prev. publ.)	SCC [mec III+Cd/Hg] (BRA2)	POS	POS	POS	NEG	NEG	POS	POS	POS
UP72 (Peru)	LGWY	SAMN03940725			Virtual hybr. (prev. publ.)	SCC [mec III+Cd/Hg] (BRA2)	POS	POS	POS	NEG	NEG	POS	POS	POS
UP81 (Peru)	LGWZ	SAMN03940726			Virtual hybr. (prev. publ.)	SCC [mec III+Cd/Hg] (BRA2)	POS	POS	POS	NEG	NEG	POS	POS	POS
>"South American/ Middle Eastern Clade": Strain with a BRA2-derived pseudoelement														
UP1073 (Peru)	MIHO	SAMN05721754	2013		Virtual hybr. (prev. publ.)	PseudoSCCmec [class A+Cd/Hg] (UP1073)	POS	POS	POS	NEG	NEG	POS	POS	POS

STRAIN / ISOLATE	Gen Bank	Bio sample Accession Number	Year of isolation	RIDOM spa	Virtual or real hybridisation	SCC*mec* type	METHICILLIN RESISTANCE AND SCCmec TYPING							
							ugpQ	mecA				delta_mecR	mecR	mecI
							ugpQ	mecA	mecA CP000046.1 SaurCOL	mecA BA000018 SaurN315	mecA GQ902038 Saur_haem pseud	Delta mecR1	mecR1	mecI
							Glycerophosphoryl diester phospho-diesterase.	Modified penicillin binding protein (PBP2a)				Truncated methicillin resistance operon repressor 1. Truncated mecR1 is present in SCCmec I, IV, V, VI, VII; complete absence of mecR1 from SCCmec	Methicillin resistance operon repressor 1. Un-truncated sequence in SCCmec II, SCCmec III, SCCmec VIII	Methicillin-resistance regulatory protein. Present in SCCmec II (although absent from Irish SCCmec II variants C and E), SCCmec III, SCCmec VIII
**>"South American/ Middle Eastern Clade": MRSA_PR1**														
LIT89 (Lithuania)	CTXR	SAMEA862592	1996		Virtual hybr. (prev. publ.)	SCC [mec III+Cd+ccrC] (XN108)	POS	POS	POS	NEG	NEG	POS	POS	POS
MRSA_PR1 (Malaysia)	ANPO	SAMN03196741	2009		Virtual hybr. (prev. publ.)	SCC [mec III+Cd+ccrC] (XN108)	POS	POS	POS	NEG	NEG	POS	POS	POS
Riyadh-2888905-R			2010-2011		Array hybr.	SCC [mec III+Cd+ccrC] (XN108)	POS	POS				POS	POS	POS
Riyadh-2888915-BC			2010-2011		Array hybr.	SCC [mec III+Cd+ccrC] (XN108)	POS	POS				POS	POS	POS
Riyadh-2793706-R			2010-2011		Array hybr.	SCC [mec III+Cd+ccrC] (XN108)	POS	POS				POS	POS	POS
**>"South American/ Middle Eastern Clade": sasX-negative strain with a S85-like SCC element**														
Riyadh_SSTI_18_3502925			2011-2012		Array hybr.	SCC [mec III+Cd+ccrC] (S85)	POS	POS	POS	NEG	NEG	POS	POS	POS
Riyadh-2822825-W			2010-2011		Array hybr.	SCC [mec III+Cd+ccrC] (S85)	POS	POS				POS	POS	POS
**>"South American/ Middle Eastern Clade": UK EMRSA-09**														
UK-EMRSA-9					Array hybr.	SCC [mec III+Cd/Hg+ccrC] (M418/UK-9)	POS	POS	POS	NEG	NEG	POS	POS	POS
**>"South American/ Middle Eastern Clade": Isolate from Togo**														
Lome_HT20020815			2002		Array hybr.	SCC [mec III+Cd/Hg] (Unknown, ST239-3)	POS	POS	POS	NEG	NEG	POS	POS	POS
**>"South American/ Middle Eastern Clade": Irish AR09/ UK EMRSA-04 and -07**														
NA32 (Denmark)	CTWR	SAMEA862615	2006		Virtual hybr. (prev. publ.)	SCC [mec III+Cd/Hg+ccrC] (TW20)	POS	POS	NEG	POS	NEG	POS	POS	POS
H24 (Egypt)	CTWS	SAMEA862527			Virtual hybr. (prev. publ.)	SCC [mec III+Cd/Hg+ccrC] (TW20)	POS	POS	POS	NEG	NEG	POS	POS	POS
ATCC BAA-39 (=HUSA304) (Hungary)	AEEK	SAMN00117207	1993	incomplete	Virtual hybr. (prev. publ.)	SCC [mec III+Cd/Hg+ccrC] (TW20)	POS	POS	POS	NEG	NEG	POS	POS	POS
HUSA304 (Hungary)		SAMEA1029531	1993	t1053	Virtual hybr. (prev. publ.)	SCC [mec III+Cd/Hg+ccrC] (TW20)	POS	POS	POS	NEG	NEG	POS	POS	POS
HU106 (Hungary)		SAMEA1029518	1996	t538	Virtual hybr. (prev. publ.)	SCC [mec III+Cd/Hg+ccrC] (TW20)	POS	POS	POS	NEG	NEG	POS	POS	POS
BSAC27 (UK)	FFVG	SAMEA1317461			Virtual hybr. (prev. publ.)	SCC [mec III+Cd/Hg+ccrC] (TW20)	POS	POS	POS	NEG	NEG	POS	POS	POS
NCTC13131, UK-EMRSA-4 (UK)		SAMEA3529263		t037	Virtual hybr. (prev. publ.)	SCC [mec III+Cd/Hg+ccrC] (TW20)	POS	POS	POS	NEG	NEG	POS	POS	POS
					Array hybr.	SCC [mec III+Cd/Hg+ccrC] (TW20)	POS	POS				POS	POS	POS
Algiers_HT20040080			2003		Array hybr.	SCC [mec III+Cd/Hg+ccrC] (TW20)	POS	POS				POS	POS	POS
Dublin-DSH_AR09_0.0066			1989	t037	Array hybr.	SCC [mec III+Cd/Hg+ccrC] (TW20)	POS	POS				POS	POS	POS
Dublin-DSH_AR09_0-0065			1989	t037	Array hybr.	SCC [mec III+Cd/Hg+ccrC] (TW20)	POS	POS				POS	POS	POS
Dublin-DSH_Phenotype-III_84			1985-1987	t037	Array hybr.	SCC [mec III+Cd/Hg+ccrC] (TW20)	POS	POS				POS	POS	POS
Hong Kong_130			2009-2010		Array hybr.	SCC [mec III+Cd/Hg+ccrC] (TW20)	POS	POS				POS	POS	POS
Kuwait_018			2001		Array hybr.	SCC [mec III+Cd/Hg+ccrC] (TW20)	POS	POS				POS	POS	POS
Perth_08-17726			2008		Array hybr.	SCC [mec III+Cd/Hg+ccrC] (TW20)	POS	POS				POS	POS	POS
Riyadh_Alfaisal/KKKSUH_86_MRS-14-279			2014		Array hybr.	SCC [mec III+Cd/Hg+ccrC] (TW20)	POS	POS				POS	POS	POS
Riyadh_Alfaisal-04_Z3861_831588			2014		Array hybr.	SCC [mec III+Cd/Hg+ccrC] (TW20)	POS	POS				POS	POS	POS
Riyadh_Alfaisal-30_S15724_1108013			2014		Array hybr.	SCC [mec III+Cd/Hg+ccrC] (TW20)	POS	POS				POS	POS	POS
Riyadh_Alfaisal-6_22A_13_B3992_397721			2014		Array hybr.	SCC [mec III+Cd/Hg+ccrC] (TW20)	POS	POS				POS	POS	POS
Riyadh_SSTI_52_3615482			2011-2012		Array hybr.	SCC [mec III+Cd/Hg+ccrC] (TW20)	POS	POS				POS	POS	POS
Riyadh-2817276-2			2010-2011		Array hybr.	SCC [mec III+Cd/Hg+ccrC] (TW20)	POS	POS				POS	POS	POS
Riyadh-2891670-W			2010-2011		Array hybr.	SCC [mec III+Cd/Hg+ccrC] (TW20)	POS	POS				POS	POS	POS
Riyadh-3006920-W			2010-2011		Array hybr.	SCC [mec III+Cd/Hg+ccrC] (TW20)	POS	POS				POS	POS	POS
Riyadh-R2567782			2010-2011		Array hybr.	SCC [mec III+Cd/Hg+ccrC] (TW20)	POS	POS				POS	POS	POS
Russia-18_0252_Krasnoyarsk_SK2			2012		Array hybr.	SCC [mec III+Cd/Hg+ccrC] (TW20)	POS	POS				POS	POS	POS
UK-EMRSA-7					Array hybr.	SCC [mec III+Cd/Hg+ccrC] (TW20)	POS	POS				POS	POS	POS
**>"South American/ Middle Eastern Clade": Irish AR23**														
Dublin-DSH_AR23_0073			1992	t037	Array hybr.	SCC [mec III+Hg+ccrC] (Unknown ST239, AR23)	POS	POS	POS	NEG	NEG	POS	POS	POS
**>Related to "South American/ Middle Eastern Clade": Krasnoyarsk tst1+ Strain**														
MRSA-OC3 (Russia)	BBKC	SAMD00019145	2007-2009		Virtual hybr. (prev. publ.)	SCC [mec III+Cd/Hg+ccrC] (Bmb9393)	POS	POS	NEG	POS	NEG	POS	POS	POS
Russia-12_0176_Krasnoyarsk			2009		Array hybr.	SCC [mec III+Cd/Hg+ccrC] (Bmb9393)	POS	POS				POS	POS	POS
Russia-13_0180_Krasnoyarsk			2009		Array hybr.	SCC [mec III+Cd/Hg+ccrC] (Bmb9393)	POS	POS				POS	POS	POS
Russia-16_0249_Krasnoyarsk			2012		Array hybr.	SCC [mec III+Cd/Hg+ccrC] (Bmb9393)	POS	POS				POS	POS	POS
Russia-17_0250_Krasnoyarsk			2012		Array hybr.	SCC [mec III+Cd/Hg+ccrC] (Bmb9393)	POS	POS				POS	POS	POS
**>Related to "South American/ Middle Eastern Clade": URU110**														
URU110 (Uruguay)		SAMEA1029540	1998	t037	Virtual hybr. (prev. publ.)	SCC [mec III+Cd/Hg+ccrC] (Bmb9393)	POS	POS	NEG	POS	NEG	POS	POS	POS
**>Related to "South American/ Middle Eastern Clade": DS_014**														
DS_014 (Thailand)	FQPU	SAMEA3448843	2014		Virtual hybr. (prev. publ.)	SCC [mec III+Cd+ccrC] (XN108)	POS	POS	NEG	POS	NEG	POS	POS	POS
**>Unassigned Middle Eastern Strain**														
Frankfurt_Oder_0490031797 (Libya)			2015		Array hybr.	SCC [mec III+Cd+ccrC] (Unknown, ST239-1)	POS	POS				POS	POS	POS
Kuwait_192			2005		Array hybr.	SCC [mec III+Cd+ccrC] (Unknown, ST239-1)	POS	POS				POS	POS	POS
Riyadh-9			2010-2011		Array hybr.	SCC [mec III+Cd+ccrC] (Unknown, ST239-1)	POS	POS	NEG	POS	NEG	POS	POS	POS
Russia-24_0407_Moscow			2013	t037	Array hybr.	SCC [mec III+Cd+ccrC] (Unknown, ST239-1)	POS	POS	NEG	POS	NEG	POS	POS	POS

STRAIN / ISOLATE	METHICILLIN RESISTANCE AND SCCmec TYPING																	
	fudoh-PSM	cstB-SCC1 (ex Q2G1R6 - sCCmec	xylR/mecR2	mecC	blaZ (SCCmec XI)	plsSCC (COL)	mvaS-SCC	cstB-SCC2 (Q2G1R6)	Q9S0M4	kdpA-SCC	kdpB-SCC	kdpC-SCC	kdpD-SCC	kdpE-SCC	Q93IB7	D1GU38		Q933A2
	fudoh-PSM	cstB-SCC1 (Q2G1R6)	xylR/mecR2	mecC	blaZ (SCCmec XI)	pls-SCC (COL)	mvaS-SCC	cstB-SCC2 (Q2G1R6)	Q9S0M4	kdpA-SCC	kdpB-SCC	kdpC-SCC	kdpD-SCC	kdpE-SCC	Q93IB7	D1GU38	D1GU38 (TW20)	Q933A2
	Phenol soluble modulin from SCCmec	CsoR-like sulfur transferase-regulated genes B/metallo-beta-lactamase superfamily protein. Pseudogene containing two stop	Methicillin resistance operon repressor 2, Homolog of xylose repressor. Located next to mec operon downstream of mecI (not present if mecI is truncated)	Alternate gene encoding a modified penicillin binding protein . Present in, and characteristic for, SCCmec XI	Beta-lactamase from SCCmec XI	Plasmin-sensitive surface protein, prevents bacterial adhesion in vitro, located in SCC, close to mec operon. Subtyping SCCmec I	Truncated 3-hydroxy-3-methylglutaryl CoA synthase. Subtyping SCCmec I, II, IV, V	CsoR-like sulfur transferase-regulated genes B/metallo-beta-lactamase superfamily protein. Present in SCCmec IVa (truncated) and	Putative protein. Subtyping SCCmec I, SCCmec/ACME composites and SCCmec from WA40	Potassium-translocating ATPase A, chain 2	Potassium-transporting ATPase B, chain 1	Potassium-translocating ATPase C, chain 2	Sensor kinase protein	KDP operon transcriptional regulatory protein	LytTR domain DNA-binding regulator. Subtyping SCCmec III (HF569113.1)	Putative protein. Subtyping SCCmec III, identification of SCCmec VT, SCCmec ZH47, SCCmec VII because of an association with (additional/second)	Putative protein	Putative ADP-ribosyltransferase. Subtyping SCCmec III and SCCmec IX
>"Eurasian Clade": TUR1																		
TUR1 (Turkey)	POS	POS	POS	NEG	NEG	NEG	POS	NEG	NEG	NEG	NEG	NEG	NEG	NEG	POS	POS	POS	POS
TUR9 (Turkey)	POS	POS	POS	NEG	NEG	NEG	POS	NEG	NEG	NEG	NEG	NEG	NEG	NEG	POS	POS	POS	POS
>"Eurasian Clade" : T013																		
T0131 (China)	POS	POS	POS	NEG	NEG	NEG	POS	NEG	NEG	NEG	NEG	NEG	NEG	NEG	NEG	POS	POS	POS
MU4 (Turkey)	POS	POS	POS	NEG	NEG	NEG	POS	NEG	NEG	NEG	NEG	NEG	NEG	NEG	NEG	POS	POS	POS
DEU11 (Turkey)	POS	POS	POS	NEG	NEG	NEG	POS	NEG	NEG	NEG	NEG	NEG	NEG	NEG	NEG	POS	POS	POS
DEU16 (Turkey)	POS	POS	POS	NEG	NEG	NEG	POS	NEG	NEG	NEG	NEG	NEG	NEG	NEG	NEG	POS	POS	POS
Romania_Iasi_MRSA-07_54	POS	POS	POS	neg	NEG	NEG	POS	NEG	NEG	NEG	NEG	NEG	NEG	NEG	NEG	POS	POS	POS
>"Eurasian Clade": arsC-																		
Romania_Iasi_MRSA-05_46	POS	POS	POS	neg	NEG	NEG	POS	NEG	NEG	NEG	NEG	NEG	NEG	NEG	NEG	POS	POS	POS
Romania_Iasi_MRSA-06_47	POS	POS	POS	neg	NEG	NEG	POS	NEG	NEG	NEG	NEG	NEG	NEG	NEG	NEG	POS	POS	POS
>"Eurasian Clade": 16K/																		
CN79 (China)	POS	POS	POS	NEG	NEG	NEG	POS	NEG	NEG	NEG	NEG	NEG	NEG	NEG	NEG	POS	POS	POS
CUHK_BJ2002 (China)	POS	POS	POS	NEG	NEG	NEG	POS	NEG	NEG	NEG	NEG	NEG	NEG	NEG	NEG	POS	POS	POS
CUHK_BJ2007 (China)	POS	POS	POS	NEG	NEG	NEG	POS	NEG	NEG	NEG	NEG	NEG	NEG	NEG	NEG	POS	POS	POS
3HK (Czech Republic)	POS	POS	POS	NEG	NEG	NEG	POS	NEG	NEG	NEG	NEG	NEG	NEG	NEG	NEG	POS	POS	POS
HU109 (Hungary)	POS	POS	POS	NEG	NEG	NEG	POS	NEG	NEG	NEG	NEG	NEG	NEG	NEG	NEG	POS	POS	POS
HUR18 (Hungary)	POS	POS	POS	NEG	NEG	NEG	POS	NEG	NEG	NEG	NEG	NEG	NEG	NEG	NEG	POS	POS	POS
H482 (Romania)	POS	POS	POS	NEG	NEG	NEG	POS	NEG	NEG	NEG	NEG	NEG	NEG	NEG	NEG	POS	POS	POS
16K (Russia)	POS	POS	POS	NEG	NEG	NEG	POS	NEG	NEG	NEG	NEG	NEG	NEG	NEG	NEG	POS	POS	POS
DEU10 (Turkey)	POS	POS	POS	NEG	NEG	NEG	POS	NEG	NEG	NEG	NEG	NEG	NEG	NEG	NEG	POS	POS	POS
DEU12 (Turkey)	POS	POS	POS	NEG	NEG	NEG	POS	NEG	NEG	NEG	NEG	NEG	NEG	NEG	NEG	POS	POS	POS
DEU14 (Turkey)	POS	POS	POS	NEG	NEG	NEG	POS	NEG	NEG	NEG	NEG	NEG	NEG	NEG	NEG	POS	POS	POS
DEU15 (Turkey)	POS	POS	POS	NEG	NEG	NEG	POS	NEG	NEG	NEG	NEG	NEG	NEG	NEG	NEG	POS	POS	POS
DEU17 (Turkey)	POS	POS	POS	NEG	NEG	NEG	POS	NEG	NEG	NEG	NEG	NEG	NEG	NEG	NEG	POS	POS	POS
DEU19 (Turkey)	POS	POS	POS	NEG	NEG	NEG	POS	NEG	NEG	NEG	NEG	NEG	NEG	NEG	NEG	POS	POS	POS
DEU2 (Turkey)	POS	POS	POS	NEG	NEG	NEG	POS	NEG	NEG	NEG	NEG	NEG	NEG	NEG	NEG	POS	POS	POS
DEU20 (Turkey)	POS	POS	POS	NEG	NEG	NEG	POS	NEG	NEG	NEG	NEG	NEG	NEG	NEG	NEG	POS	POS	POS
DEU23 (Turkey)	POS	POS	POS	NEG	NEG	NEG	POS	NEG	NEG	NEG	NEG	NEG	NEG	NEG	NEG	POS	POS	POS
DEU3 (Turkey)	POS	POS	POS	NEG	NEG	NEG	POS	NEG	NEG	NEG	NEG	NEG	NEG	NEG	NEG	POS	POS	POS
DEU5 (Turkey)	POS	POS	POS	NEG	NEG	NEG	POS	NEG	NEG	NEG	NEG	NEG	NEG	NEG	NEG	POS	POS	POS
DEU6 (Turkey)	POS	POS	POS	NEG	NEG	NEG	POS	NEG	NEG	NEG	NEG	NEG	NEG	NEG	NEG	POS	POS	POS
DEU8 (Turkey)	POS	POS	POS	NEG	NEG	NEG	POS	NEG	NEG	NEG	NEG	NEG	NEG	NEG	NEG	POS	POS	POS
HU11 (Turkey)	POS	POS	POS	NEG	NEG	NEG	POS	NEG	NEG	NEG	NEG	NEG	NEG	NEG	NEG	POS	POS	POS
HU13 (Turkey)	POS	POS	POS	NEG	NEG	NEG	POS	NEG	NEG	NEG	NEG	NEG	NEG	NEG	NEG	POS	POS	POS
HU14 (Turkey)	POS	POS	POS	NEG	NEG	NEG	POS	NEG	NEG	NEG	NEG	NEG	NEG	NEG	NEG	POS	POS	POS
HU15 (Turkey)	POS	POS	POS	NEG	NEG	NEG	POS	NEG	NEG	NEG	NEG	NEG	NEG	NEG	NEG	POS	POS	POS
HU16 (Turkey)	POS	POS	POS	NEG	NEG	NEG	POS	NEG	NEG	NEG	NEG	NEG	NEG	NEG	NEG	POS	POS	POS
HU17 (Turkey)	POS	POS	POS	NEG	NEG	NEG	POS	NEG	NEG	NEG	NEG	NEG	NEG	NEG	NEG	POS	POS	POS
HU21 (Turkey)	POS	POS	POS	NEG	NEG	NEG	POS	NEG	NEG	NEG	NEG	NEG	NEG	NEG	NEG	POS	POS	POS
HU23 (Turkey)	POS	POS	POS	NEG	NEG	NEG	POS	NEG	NEG	NEG	NEG	NEG	NEG	NEG	NEG	POS	POS	POS
HU26 (Turkey)	POS	POS	POS	NEG	NEG	NEG	POS	NEG	NEG	NEG	NEG	NEG	NEG	NEG	NEG	POS	POS	POS
HU4 (Turkey)	POS	POS	POS	NEG	NEG	NEG	POS	NEG	NEG	NEG	NEG	NEG	NEG	NEG	NEG	POS	POS	POS
HU5 (Turkey)	POS	POS	POS	NEG	NEG	NEG	POS	NEG	NEG	NEG	NEG	NEG	NEG	NEG	NEG	POS	POS	POS
HU6 (Turkey)	POS	POS	POS	NEG	NEG	NEG	POS	NEG	NEG	NEG	NEG	NEG	NEG	NEG	NEG	POS	POS	POS
HU7 (Turkey)	POS	POS	POS	NEG	NEG	NEG	POS	NEG	NEG	NEG	NEG	NEG	NEG	NEG	NEG	POS	POS	POS
HU8 (Turkey)	POS	POS	POS	NEG	NEG	NEG	POS	NEG	NEG	NEG	NEG	NEG	NEG	NEG	NEG	POS	POS	POS
HU9 (Turkey)	POS	POS	POS	NEG	NEG	NEG	POS	NEG	NEG	NEG	NEG	NEG	NEG	NEG	NEG	POS	POS	POS
IU1 (Turkey)	POS	POS	POS	NEG	NEG	NEG	POS	NEG	NEG	NEG	NEG	NEG	NEG	NEG	NEG	POS	POS	POS
IU10 (Turkey)	POS	POS	POS	NEG	NEG	NEG	POS	NEG	NEG	NEG	NEG	NEG	NEG	NEG	NEG	POS	POS	POS
IU11 (Turkey)	POS	POS	POS	NEG	NEG	NEG	POS	NEG	NEG	NEG	NEG	NEG	NEG	NEG	NEG	POS	POS	POS
IU12 (Turkey)	POS	POS	POS	NEG	NEG	NEG	POS	NEG	NEG	NEG	NEG	NEG	NEG	NEG	NEG	POS	POS	POS
IU13 (Turkey)	POS	POS	POS	NEG	NEG	NEG	POS	NEG	NEG	NEG	NEG	NEG	NEG	NEG	NEG	POS	POS	POS
IU15 (Turkey)	POS	POS	POS	NEG	NEG	NEG	POS	NEG	NEG	NEG	NEG	NEG	NEG	NEG	NEG	POS	POS	POS
IU18 (Turkey)	POS	POS	POS	NEG	NEG	NEG	POS	NEG	NEG	NEG	NEG	NEG	NEG	NEG	NEG	POS	POS	POS
IU19 (Turkey)	POS	POS	POS	NEG	NEG	NEG	POS	NEG	NEG	NEG	NEG	NEG	NEG	NEG	NEG	POS	POS	POS
IU2 (Turkey)	POS	POS	POS	NEG	NEG	NEG	POS	NEG	NEG	NEG	NEG	NEG	NEG	NEG	NEG	POS	POS	POS
IU4 (Turkey)	POS	POS	POS	NEG	NEG	NEG	POS	NEG	NEG	NEG	NEG	NEG	NEG	NEG	NEG	POS	POS	POS
IU6 (Turkey)	POS	POS	POS	NEG	NEG	NEG	POS	NEG	NEG	NEG	NEG	NEG	NEG	NEG	NEG	POS	POS	POS
IU7 (Turkey)	POS	POS	POS	NEG	NEG	NEG	POS	NEG	NEG	NEG	NEG	NEG	NEG	NEG	NEG	POS	POS	POS
IU9 (Turkey)	POS	POS	POS	NEG	NEG	NEG	POS	NEG	NEG	NEG	NEG	NEG	NEG	NEG	NEG	POS	POS	POS
MU1 (Turkey)	POS	POS	POS	NEG	NEG	NEG	POS	NEG	NEG	NEG	NEG	NEG	NEG	NEG	NEG	POS	POS	POS
MU10 (Turkey)	POS	POS	POS	NEG	NEG	NEG	POS	NEG	NEG	NEG	NEG	NEG	NEG	NEG	NEG	POS	POS	POS
MU20 (Turkey)	POS	POS	POS	NEG	NEG	NEG	POS	NEG	NEG	NEG	NEG	NEG	NEG	NEG	NEG	POS	POS	POS
Mu3 (Turkey)	POS	POS	POS	NEG	NEG	NEG	POS	NEG	NEG	NEG	NEG	NEG	NEG	NEG	NEG	POS	POS	POS
MU5 (Turkey)	POS	POS	POS	NEG	NEG	NEG	POS	NEG	NEG	NEG	NEG	NEG	NEG	NEG	NEG	POS	POS	POS
MU6 (Turkey)	POS	POS	POS	NEG	NEG	NEG	POS	NEG	NEG	NEG	NEG	NEG	NEG	NEG	NEG	POS	POS	POS
MU7 (Turkey)	POS	POS	POS	NEG	NEG	NEG	POS	NEG	NEG	NEG	NEG	NEG	NEG	NEG	NEG	POS	POS	POS
TUR27 (Turkey)	POS	POS	POS	NEG	NEG	NEG	POS	NEG	NEG	NEG	NEG	NEG	NEG	NEG	NEG	POS	POS	POS
NCTR 32S	POS	POS	POS	NEG	NEG	NEG	POS	NEG	NEG	NEG	NEG	NEG	NEG	NEG	NEG	POS	POS	POS
SA02_A4 = Russia-06_0085_Moscow	POS	POS	POS	NEG	NEG	NEG	POS	NEG	NEG	NEG	NEG	NEG	NEG	NEG	NEG	POS	POS	POS
	POS	POS	POS	NEG	NEG	NEG	POS	NEG	NEG	NEG	NEG	NEG	NEG	NEG	NEG	POS	POS	POS
Dresden_08V35987 (Turkey)	POS	POS	POS	neg	NEG	NEG	POS	NEG	NEG	NEG	NEG	NEG	NEG	NEG	NEG	POS	POS	POS
Dresden_17ANR577152	POS	POS	POS	NEG	NEG	NEG	POS	NEG	NEG	NEG	NEG	NEG	NEG	NEG	NEG	POS	POS	POS
Dresden_17ANR580374 (Makedonia)	POS	POS	POS	NEG	NEG	NEG	POS	NEG	NEG	NEG	NEG	NEG	NEG	NEG	NEG	POS	POS	POS
Hong Kong_69-II	POS	POS	POS	neg	NEG	NEG	POS	NEG	NEG	NEG	NEG	NEG	NEG	NEG	NEG	POS	POS	POS
Hong Kong_93	POS	POS	POS	neg	NEG	NEG	POS	NEG	NEG	NEG	NEG	NEG	NEG	NEG	NEG	POS	POS	POS
Rawalpindi_Kidney Center_03_SK1	POS	POS	POS	NEG	NEG	NEG	POS	NEG	NEG	NEG	NEG	NEG	NEG	NEG	NEG	POS	POS	POS
Rawalpindi_Kidney Center_08	POS	POS	POS	NEG	NEG	NEG	POS	NEG	NEG	NEG	NEG	NEG	NEG	NEG	NEG	POS	POS	POS
Rawalpindi_Kidney Center_10	POS	POS	POS	neg	NEG	NEG	POS	NEG	NEG	NEG	NEG	NEG	NEG	NEG	NEG	POS	POS	POS
Rawalpindi_Kidney Center_19	POS	POS	POS	NEG	NEG	NEG	POS	NEG	NEG	NEG	NEG	NEG	NEG	NEG	NEG	POS	POS	POS
Rawalpindi_Kidney Center_50_SK1	POS	POS	POS	neg	NEG	NEG	POS	NEG	NEG	NEG	NEG	NEG	NEG	NEG	NEG	POS	POS	POS
Romania_Iasi_BC-49_430	POS	POS	POS	neg	NEG	NEG	POS	NEG	NEG	NEG	NEG	NEG	NEG	NEG	NEG	POS	POS	POS
Romania_Iasi_MRSA_284	POS	AMB	POS	neg	NEG	NEG	POS	NEG	NEG	NEG	NEG	NEG	NEG	NEG	NEG	POS	NEG	POS
Romania_Iasi_MRSA-32_318	POS	POS	POS	NEG	NEG	NEG	POS	NEG	NEG	NEG	NEG	NEG	NEG	NEG	NEG	POS	POS	POS
Romania_Iasi_MRSA-54_430	POS	POS	POS	neg	NEG	NEG	POS	NEG	NEG	NEG	NEG	NEG	NEG	NEG	NEG	POS	POS	POS
Romania_Iasi_SSTI-01_2	POS	POS	POS	neg	NEG	NEG	POS	NEG	NEG	NEG	NEG	NEG	NEG	NEG	NEG	POS	POS	POS
Romania_Iasi_SSTI-10_101	POS	POS	POS	neg	NEG	NEG	POS	NEG	NEG	NEG	NEG	NEG	NEG	NEG	NEG	POS	POS	POS
Romania_Iasi_SSTI-11_106	POS	POS	POS	NEG	NEG	NEG	POS	NEG	NEG	NEG	NEG	NEG	NEG	NEG	NEG	POS	POS	POS
Russia-01_0001_SaintPetersburg	POS	POS	POS	NEG	NEG	NEG	POS	NEG	NEG	NEG	NEG	NEG	NEG	NEG	NEG	POS	POS	POS
Russia-02_0004_SaintPetersburg	POS	POS	POS	NEG	NEG	NEG	POS	NEG	NEG	NEG	NEG	NEG	NEG	NEG	NEG	POS	POS	POS
Russia-03_0057_SaintPetersburg	POS	POS	POS	NEG	NEG	NEG	POS	NEG	NEG	NEG	NEG	NEG	NEG	NEG	NEG	POS	AMB	POS
Russia-04_0076_Moscow	POS	POS	POS	NEG	NEG	NEG	POS	NEG	NEG	NEG	NEG	NEG	NEG	NEG	NEG	POS	AMB	POS
Russia-05_0078_Moscow	POS	POS	POS	NEG	NEG	NEG	POS	NEG	NEG	NEG	NEG	NEG	NEG	NEG	NEG	POS	POS	POS
Russia-08_0135_Moscow	POS	POS	POS	NEG	NEG	NEG	POS	NEG	NEG	NEG	NEG	NEG	NEG	NEG	NEG	POS	POS	POS
Russia-09_0150_Kurgan	POS	POS	POS	NEG	NEG	NEG	POS	NEG	NEG	NEG	NEG	NEG	NEG	NEG	NEG	POS	POS	POS
Russia-10_0162_Kurgan	POS	POS	POS	NEG	NEG	NEG	POS	NEG	NEG	NEG	NEG	NEG	NEG	NEG	NEG	POS	POS	POS
Russia-14_0184_Moscow	POS	POS	POS	NEG	NEG	NEG	POS	NEG	NEG	NEG	NEG	NEG	NEG	NEG	NEG	POS	NEG	POS
Russia-15_0232_SaintPetersburg	POS	POS	POS	NEG	NEG	NEG	POS	NEG	NEG	NEG	NEG	NEG	NEG	NEG	NEG	POS	AMB	POS
Russia-22_0391_Chelyabinsk	POS	POS	POS	NEG	NEG	NEG	POS	NEG	NEG	NEG	NEG	NEG	NEG	NEG	NEG	POS	POS	POS
Russia-23_0392_Chelyabinsk	POS	POS	POS	NEG	NEG	NEG	POS	NEG	NEG	NEG	NEG	NEG	NEG	NEG	NEG	POS	AMB	POS
>"Eurasian Clade" : IU17																		
IU17 (Turkey)	POS	POS	POS	NEG	NEG	NEG	POS	NEG	NEG	NEG	NEG	NEG	NEG	NEG	NEG	POS	POS	POS
IU20 (Turkey)	POS	POS	POS	NEG	NEG	NEG	POS	NEG	NEG	NEG	NEG	NEG	NEG	NEG	NEG	POS	POS	POS

STRAIN / ISOLATE	METHICILLIN RESISTANCE AND SCCmec TYPING																	
	fudoh-PSM	cstB-SCC1 (ex Q2G1R6 - SCCmec	xylR/mecR2	mecC	blaZ (SCCmec XI)	plsSCC (COL)	mvaS-SCC	cstB-SCC2 (Q2G1R6)	Q9S0M4	kdpA-SCC	kdpB-SCC	kdpC-SCC	kdpD-SCC	kdpE-SCC	Q93IB7	D1GU38		Q933A2
	fudoh-PSM	cstB-SCC1 (Q2G1R6)	xylR/mecR2	mecC	blaZ (SCCmec XI)	pls-SCC (COL)	mvaS-SCC	cstB-SCC2 (Q2G1R6)	Q9S0M4	kdpA-SCC	kdpB-SCC	kdpC-SCC	kdpD-SCC	kdpE-SCC	Q93IB7	D1GU38	D1GU38 (TW20)	Q933A2
	Phenol soluble modulin from SCCmec	CsoR-like sulfur transferase-regulated genes B/metallo-beta-lactamase superfamily protein. Pseudogene containing two stop	Methicillin resistance operon repressor 2, Homolog of xylose repressor. Located next to mec operon downstream of mecI (not present if mecI is truncated)	Alternate gene encoding a modified penicillin binding protein . Present in, and characteristic for, SCCmec XI	Beta-lactamase from SCCmec XI	Plasmin-sensitive surface protein, prevents bacterial adhesion in vitro, located in SCC, close to mec operon. Subtyping SCCmec I	Truncated 3-hydroxy-3-methylglutaryl CoA synthase. Subtyping SCCmec I, II, IV, V	CsoR-like sulfur transferase-regulated genes B/metallo-beta-lactamase superfamily protein. Present in SCCmec IVa (truncated) and	Putative protein. Subtyping SCCmec I, SCCmec/ACME composites and SCCmec from WA40	Potassium-translocating ATPase A, chain 2	Potassium-transporting ATPase B, chain 1	Potassium-translocating ATPase C, chain 2	Sensor kinase protein	KDP operon transcriptional regulatory protein	LytTR domain DNA-binding regulator. Subtyping SCCmec III (HF569113.1)	Putative protein. Subtyping SCCmec III, identification of SCCmec VT, SCCmec ZH47, SCCmec VII because of an association with (additional/second)	Putative protein	Putative ADP-ribosyltransferase. Subtyping SCCmec III and SCCmec IX
>"Eurasian Clade": Spora																		
Dresden_16ANR581769	POS	POS	POS	NEG	NEG	NEG	POS	NEG	POS	NEG	NEG	NEG	NEG	NEG	NEG	POS	POS	POS
Dresden_17ANR578769	POS	POS	POS	NEG	NEG	NEG	POS	NEG	POS	NEG	NEG	NEG	NEG	NEG	NEG	NEG	NEG	POS
>"Eurasian Clade": Kuwa																		
Kuwait_306	POS	POS	POS	neg	NEG	NEG	POS	NEG	POS	NEG	NEG	NEG	NEG	NEG	NEG	POS	AMB	POS
Kuwait_301	POS	POS	POS	neg	NEG	NEG	POS	NEG	POS	NEG	NEG	NEG	NEG	NEG	NEG	POS	POS	POS
>"European Clade": "Gre																		
GRE18 (Greece)	POS	POS	POS	NEG	NEG	NEG	POS	NEG	NEG	NEG	NEG	NEG	NEG	NEG	POS	POS	POS	POS
GRE317 (Greece)	POS	POS	POS	NEG	NEG	NEG	POS	NEG	NEG	NEG	NEG	NEG	NEG	NEG	POS	POS	POS	POS
GRE4 (Greece)	POS	POS	POS	NEG	NEG	NEG	POS	NEG	NEG	NEG	NEG	NEG	NEG	NEG	POS	POS	POS	POS
R35 (USA)	POS	POS	POS	NEG	NEG	NEG	POS	NEG	NEG	NEG	NEG	NEG	NEG	NEG	POS	POS	POS	POS
Casablanca_HT20060548	POS	POS	POS	NEG	NEG	NEG	POS	NEG	NEG	NEG	NEG	NEG	NEG	NEG	POS	POS	POS	POS
Casablanca_HT20060550	POS	POS	POS	NEG	NEG	NEG	POS	NEG	NEG	NEG	NEG	NEG	NEG	NEG	POS	POS	POS	POS
Dresden_01V34572 (Greece)	POS	POS	POS	neg	NEG	NEG	POS	NEG	NEG	NEG	NEG	NEG	NEG	NEG	POS	POS	POS	POS
Dresden_01V36469	POS	POS	POS	neg	NEG	NEG	POS	NEG	NEG	NEG	NEG	NEG	NEG	NEG	POS	POS	POS	POS
Dresden_01V39123	POS	POS	POS	neg	NEG	NEG	POS	NEG	NEG	NEG	NEG	NEG	NEG	NEG	POS	POS	POS	POS
Dresden_01V39143	POS	POS	POS	neg	NEG	NEG	POS	NEG	NEG	NEG	NEG	NEG	NEG	NEG	POS	POS	POS	POS
Greece 1_3680_Harmony	POS	POS	POS	neg	NEG	NEG	POS	NEG	NEG	NEG	NEG	NEG	NEG	NEG	POS	POS	POS	POS
>"European Clade": UK-(																		
ANS46 (Australia)	POS	POS	POS	NEG	NEG	NEG	POS	NEG	NEG	NEG	NEG	NEG	NEG	NEG	POS	POS	POS	POS
LHH1 (USA)	POS	POS	POS	NEG	NEG	NEG	POS	NEG	NEG	NEG	NEG	NEG	NEG	NEG	POS	POS	POS	POS
Dublin-DSH_AR01_0-1085	POS	POS	POS	neg	NEG	NEG	POS	NEG	NEG	NEG	NEG	NEG	NEG	NEG	POS	POS	POS	POS
Dublin-DSH_AR01_0-1118	POS	POS	POS	neg	NEG	NEG	POS	NEG	NEG	NEG	NEG	NEG	NEG	NEG	POS	POS	POS	POS
Dublin-DSH_AR15_0098	POS	POS	POS	neg	NEG	NEG	POS	NEG	NEG	NEG	NEG	NEG	NEG	NEG	POS	POS	POS	POS
Dublin-DSH_AR15_0104	POS	POS	POS	neg	NEG	NEG	POS	NEG	NEG	NEG	NEG	NEG	NEG	NEG	POS	POS	POS	POS
UK_NCTC11939_UK-1_Harmony	POS	POS	POS	neg	NEG	NEG	POS	NEG	NEG	NEG	NEG	NEG	NEG	NEG	POS	POS	POS	POS
>"European Clade": BK2																		
BK2421 (USA)	POS	POS	POS	NEG	NEG	NEG	NEG	NEG	NEG	NEG	NEG	NEG	NEG	NEG	POS	POS	POS	POS
>"Aussi/NZ Clade", JKD6																		
JKD6009 (Australia)	POS	POS	POS	NEG	NEG	NEG	POS	NEG	NEG	NEG	NEG	NEG	NEG	NEG	NEG	NEG	NEG	POS
JKD6008 (New Zealand)	POS	POS	POS	NEG	NEG	NEG	POS	NEG	NEG	NEG	NEG	NEG	NEG	NEG	NEG	NEG	NEG	POS
Perth_2015-S20209	POS	POS	POS	NEG	NEG	NEG	POS	NEG	NEG	NEG	NEG	NEG	NEG	NEG	NEG	NEG	NEG	AMB
Perth_2015-S20305	POS	POS	POS	NEG	NEG	NEG	POS	NEG	NEG	NEG	NEG	NEG	NEG	NEG	NEG	NEG	NEG	POS
Perth_2015-S20603	POS	POS	POS	NEG	NEG	NEG	POS	NEG	NEG	NEG	NEG	NEG	NEG	NEG	NEG	NEG	NEG	POS
Perth_2015-S20607	POS	POS	POS	NEG	NEG	NEG	POS	NEG	NEG	NEG	NEG	NEG	NEG	NEG	NEG	NEG	NEG	POS
Perth_2015-S20608	POS	POS	POS	NEG	NEG	NEG	POS	NEG	NEG	NEG	NEG	NEG	NEG	NEG	NEG	NEG	NEG	POS
Perth_2015-S20610	POS	POS	POS	NEG	NEG	NEG	POS	NEG	NEG	NEG	NEG	NEG	NEG	NEG	NEG	NEG	NEG	POS
Perth_2015-S20613	POS	POS	POS	NEG	NEG	NEG	POS	NEG	NEG	NEG	NEG	NEG	NEG	NEG	NEG	NEG	NEG	POS
Perth_2015-S20617	POS	POS	POS	NEG	NEG	NEG	POS	NEG	NEG	NEG	NEG	NEG	NEG	NEG	NEG	NEG	NEG	POS
Perth_2015-S20622	POS	POS	NEG	NEG	NEG	NEG	POS	NEG	NEG	NEG	NEG	NEG	NEG	NEG	NEG	NEG	NEG	NEG
Perth_2015-S23619	POS	POS	POS	NEG	NEG	NEG	POS	NEG	NEG	NEG	NEG	NEG	NEG	NEG	NEG	NEG	NEG	POS
Perth_2015-S41210	POS	POS	POS	NEG	NEG	NEG	POS	NEG	NEG	NEG	NEG	NEG	NEG	NEG	NEG	NEG	NEG	POS
Perth_2015-S51001	POS	POS	POS	NEG	NEG	NEG	POS	NEG	NEG	NEG	NEG	NEG	NEG	NEG	NEG	NEG	NEG	POS
Perth_2015-S51003	POS	POS	POS	NEG	NEG	NEG	POS	NEG	NEG	NEG	NEG	NEG	NEG	NEG	NEG	NEG	NEG	POS
Perth_2015-S51006	POS	POS	POS	NEG	NEG	NEG	POS	NEG	NEG	NEG	NEG	NEG	NEG	NEG	NEG	NEG	NEG	POS
Perth_2015-S51007	POS	POS	POS	NEG	NEG	NEG	POS	NEG	NEG	NEG	NEG	NEG	NEG	NEG	NEG	NEG	NEG	POS
Perth_2015-S51008	POS	POS	POS	NEG	NEG	NEG	POS	NEG	NEG	NEG	NEG	NEG	NEG	NEG	NEG	NEG	NEG	POS
Perth_2015-S51014	POS	POS	POS	NEG	NEG	NEG	POS	NEG	NEG	NEG	NEG	NEG	NEG	NEG	NEG	NEG	NEG	POS
Perth_2015-S51408	POS	POS	POS	NEG	NEG	NEG	POS	NEG	NEG	NEG	NEG	NEG	NEG	NEG	NEG	NEG	NEG	POS
Perth_2016-S20614	POS	POS	POS	NEG	NEG	NEG	POS	NEG	NEG	NEG	NEG	NEG	NEG	NEG	NEG	NEG	NEG	POS
Perth_2016-S23614	POS	POS	POS	NEG	NEG	NEG	POS	NEG	NEG	NEG	NEG	NEG	NEG	NEG	NEG	NEG	NEG	POS
Perth_2016-S42009	POS	POS	POS	NEG	NEG	NEG	POS	NEG	NEG	NEG	NEG	NEG	NEG	NEG	NEG	NEG	NEG	POS
Perth_2016-S51001	POS	POS	POS	NEG	NEG	NEG	POS	NEG	NEG	NEG	NEG	NEG	NEG	NEG	NEG	NEG	NEG	POS
Perth_2016-S51403	POS	POS	POS	NEG	NEG	NEG	POS	NEG	NEG	NEG	NEG	NEG	NEG	NEG	NEG	NEG	NEG	POS
Perth_07 RPA 20	POS	POS	POS	neg	NEG	NEG	POS	NEG	NEG	NEG	NEG	NEG	NEG	NEG	NEG	NEG	NEG	POS
Perth_08-19088	POS	POS	POS	neg	NEG	NEG	POS	NEG	NEG	NEG	NEG	NEG	NEG	NEG	NEG	NEG	NEG	POS
Perth_01-15351	POS	POS	POS	neg	NEG	NEG	POS	NEG	NEG	NEG	NEG	NEG	NEG	NEG	NEG	NEG	NEG	POS
Perth_01-15357	POS	POS	POS	neg	NEG	NEG	POS	NEG	NEG	NEG	NEG	NEG	NEG	NEG	NEG	NEG	NEG	POS
Perth_01-15419	POS	POS	POS	neg	NEG	NEG	POS	NEG	NEG	NEG	NEG	NEG	NEG	NEG	NEG	NEG	NEG	POS
Perth_01-16309	POS	POS	POS	neg	NEG	NEG	POS	NEG	NEG	NEG	NEG	NEG	NEG	NEG	NEG	NEG	NEG	POS
Perth_03 SNP 95	POS	POS	POS	neg	NEG	NEG	POS	NEG	NEG	NEG	NEG	NEG	NEG	NEG	NEG	NEG	NEG	POS
Perth_2003_AH_47	POS	POS	POS	neg	NEG	NEG	POS	NEG	NEG	NEG	NEG	NEG	NEG	NEG	NEG	NEG	NEG	POS
>"Australian/NZ Clade":																		
Perth_2015-S20623	POS	POS	POS	NEG	NEG	NEG	POS	NEG	POS	NEG	NEG	NEG	NEG	NEG	NEG	NEG	NEG	POS
Perth_2015-S20826	POS	POS	POS	NEG	NEG	NEG	POS	NEG	POS	NEG	NEG	NEG	NEG	NEG	NEG	NEG	NEG	POS
Perth_05 NH 56	POS	POS	POS	neg	NEG	NEG	POS	NEG	POS	NEG	NEG	NEG	NEG	NEG	NEG	NEG	NEG	POS
>CC5-MRSA-III LA MRSA																		
Delbrück 38 isolate from domestic turkey	POS	POS	POS	neg	NEG	NEG	POS	NEG	NEG	NEG	NEG	NEG	NEG	NEG	NEG	NEG	NEG	POS
> Staph. pseudintermed																		
KM1381	POS	POS	POS	NEG	NEG	NEG	POS	NEG	NEG	NEG	NEG	NEG	NEG	NEG	NEG	NEG	NEG	POS
>"South-East Asian Clad																		
M92 (Canada)	POS	POS	POS	NEG	NEG	NEG	POS	NEG	NEG	NEG	NEG	NEG	NEG	NEG	POS	POS	POS	POS
CHI59 (China)	POS	POS	POS	NEG	NEG	NEG	POS	NEG	NEG	NEG	NEG	NEG	NEG	NEG	POS	POS	POS	POS
CHI61 (China)	POS	POS	POS	NEG	NEG	NEG	POS	NEG	NEG	NEG	NEG	NEG	NEG	NEG	POS	POS	POS	POS
CUHK_HK1997 (China)	POS	POS	POS	NEG	NEG	NEG	POS	NEG	NEG	NEG	NEG	NEG	NEG	NEG	POS	POS	POS	POS
M996 (China)	POS	POS	POS	NEG	NEG	NEG	POS	NEG	NEG	NEG	NEG	NEG	NEG	NEG	POS	POS	POS	POS
H211 (Denmark)	POS	POS	POS	NEG	NEG	NEG	POS	NEG	NEG	NEG	NEG	NEG	NEG	NEG	POS	POS	POS	POS
H216 (Denmark)	POS	POS	POS	NEG	NEG	NEG	POS	NEG	NEG	NEG	NEG	NEG	NEG	NEG	POS	POS	POS	POS
D71 (Germany)	POS	POS	POS	NEG	NEG	NEG	POS	NEG	NEG	NEG	NEG	NEG	NEG	NEG	POS	POS	POS	POS
NMR05 (India)	POS	POS	POS	NEG	NEG	NEG	POS	NEG	NEG	NEG	NEG	NEG	NEG	NEG	POS	POS	POS	POS
MAL11 (Malaysia)	POS	POS	POS	NEG	NEG	NEG	POS	NEG	NEG	NEG	NEG	NEG	NEG	NEG	POS	POS	POS	POS
MAL9 (Malaysia)	POS	POS	POS	NEG	NEG	NEG	POS	NEG	NEG	NEG	NEG	NEG	NEG	NEG	POS	POS	POS	POS
Na21 (Sri Lanka)	POS	POS	POS	NEG	NEG	NEG	POS	NEG	NEG	NEG	NEG	NEG	NEG	NEG	POS	POS	POS	POS
M592 (Syria)	POS	POS	POS	NEG	NEG	NEG	POS	NEG	NEG	NEG	NEG	NEG	NEG	NEG	POS	POS	POS	POS
H202 (Thailand)	POS	POS	POS	NEG	NEG	NEG	POS	NEG	NEG	NEG	NEG	NEG	NEG	NEG	POS	POS	POS	POS
S102 (Thailand)	POS	POS	POS	NEG	NEG	NEG	POS	NEG	NEG	NEG	NEG	NEG	NEG	NEG	POS	POS	POS	POS
S130 (Thailand)	POS	POS	POS	NEG	NEG	NEG	POS	NEG	NEG	NEG	NEG	NEG	NEG	NEG	POS	POS	POS	POS
S40 (Thailand)	POS	POS	POS	NEG	NEG	NEG	POS	NEG	NEG	NEG	NEG	NEG	NEG	NEG	POS	POS	POS	POS
S71 (Thailand)	POS	POS	POS	NEG	NEG	NEG	POS	NEG	NEG	NEG	NEG	NEG	NEG	NEG	POS	POS	POS	POS
S87 (Thailand)	POS	POS	POS	NEG	NEG	NEG	POS	NEG	NEG	NEG	NEG	NEG	NEG	NEG	POS	POS	POS	POS
S93 (Thailand)	POS	POS	POS	NEG	NEG	NEG	POS	NEG	NEG	NEG	NEG	NEG	NEG	NEG	POS	POS	POS	POS
US_002 (Thailand)	POS	POS	POS	NEG	NEG	NEG	POS	NEG	NEG	NEG	NEG	NEG	NEG	NEG	POS	POS	POS	POS
US_017 (Thailand)	POS	POS	POS	NEG	NEG	NEG	POS	NEG	NEG	NEG	NEG	NEG	NEG	NEG	POS	POS	POS	POS
WAMC6102 (Thailand)	POS	POS	POS	NEG	NEG	NEG	POS	NEG	NEG	NEG	NEG	NEG	NEG	NEG	POS	POS	POS	POS
NCTC_13626_TW20 (UK)	POS	POS	POS	NEG	NEG	NEG	POS	NEG	NEG	NEG	NEG	NEG	NEG	NEG	POS	POS	POS	POS
BSAC3021 (UK)	POS	POS	POS	NEG	NEG	NEG	POS	NEG	NEG	NEG	NEG	NEG	NEG	NEG	POS	POS	POS	POS
BSAC3046 (UK)	POS	POS	POS	NEG	NEG	NEG	POS	NEG	NEG	NEG	NEG	NEG	NEG	NEG	POS	POS	POS	POS
1193_SAUR (USA)	POS	POS	POS	NEG	NEG	NEG	POS	NEG	NEG	NEG	NEG	NEG	NEG	NEG	POS	POS	POS	POS
1194_SAUR (USA)	POS	POS	POS	NEG	NEG	NEG	POS	NEG	NEG	NEG	NEG	NEG	NEG	NEG	POS	POS	POS	POS

STRAIN / ISOLATE	METHICILLIN RESISTANCE AND SCCmec TYPING																	
	fudoh-PSM	cstB-SCC1 (ex Q2G1R6 - sCCmec	xylR/mecR2	mecC	blaZ (SCCmec XI)	plsSCC (COL)	mvaS-SCC	cstB-SCC2 (Q2G1R6)	Q9S0M4	kdpA-SCC	kdpB-SCC	kdpC-SCC	kdpD-SCC	kdpE-SCC	Q93IB7	D1GU38		Q933A2
	fudoh-PSM	cstB-SCC1 (Q2G1R6)	xylR/mecR2	mecC	blaZ (SCCmec XI)	pls-SCC (COL)	mvaS-SCC	cstB-SCC2 (Q2G1R6)	Q9S0M4	kdpA-SCC	kdpB-SCC	kdpC-SCC	kdpD-SCC	kdpE-SCC	Q93IB7	D1GU38	D1GU38 (TW20)	Q933A2
	Phenol soluble modulin from SCCmec	CsoR-like sulfur transferase-regulated genes B/metallo-beta-lactamase superfamily protein. Pseudogene containing two stop	Methicillin resistance operon repressor 2, Homolog of xylose repressor. Located next to mec operon downstream of mecI (not present if mecI is truncated)	Alternate gene encoding a modified penicillin binding protein . Present in, and characteristic for, SCCmec XI	Beta-lactamase from SCCmec XI	Plasmin-sensitive surface protein, prevents bacterial adhesion in vitro, located in SCC, close to mec operon. Subtyping SCCmec I	Truncated 3-hydroxy-3-methylglutaryl CoA synthase. Subtyping SCCmec I, II, IV, V	CsoR-like sulfur transferase-regulated genes B/metallo-beta-lactamase superfamily protein. Present in SCCmec IVa (truncated) and	Putative protein. Subtyping SCCmec I, SCCmec/ACME composites and SCCmec from WA40	Potassium-translocating ATPase A, chain 2	Potassium-transporting ATPase B, chain 1	Potassium-translocating ATPase C, chain 2	Sensor kinase protein	KDP operon transcriptional regulatory protein	LytTR domain DNA-binding regulator. Subtyping SCCmec III (HF569113.1)	Putative protein. Subtyping SCCmec III, identification of SCCmec VT, SCCmec ZH47, SCCmec VII because of an association with (additional/second)	Putative protein	Putative ADP-ribosyltransferase. Subtyping SCCmec III and SCCmec IX
>"South-East Asian Clade": TW20 (Irish AR44), c																		
1195_SAUR (USA)	POS	POS	POS	NEG	NEG	NEG	POS	NEG	NEG	NEG	NEG	NEG	NEG	NEG	POS	POS	POS	POS
FVRH6002 (USA)	POS	POS	POS	NEG	NEG	NEG	POS	NEG	NEG	NEG	NEG	NEG	NEG	NEG	POS	POS	POS	POS
KINW6048 (USA)	POS	POS	POS	NEG	NEG	NEG	POS	NEG	NEG	NEG	NEG	NEG	NEG	NEG	POS	POS	POS	POS
LAMC0011 (USA)	POS	POS	POS	NEG	NEG	NEG	POS	NEG	NEG	NEG	NEG	NEG	NEG	NEG	POS	POS	POS	POS
SJOS6053 (USA)	POS	POS	POS	NEG	NEG	NEG	POS	NEG	NEG	NEG	NEG	NEG	NEG	NEG	POS	POS	POS	POS
SJOS6072 (USA)	POS	POS	POS	NEG	NEG	NEG	POS	NEG	NEG	NEG	NEG	NEG	NEG	NEG	POS	POS	POS	POS
UCIM6042 (USA)	POS	POS	POS	NEG	NEG	NEG	POS	NEG	NEG	NEG	NEG	NEG	NEG	NEG	POS	POS	POS	POS
Perth_2015-S31902	NEG	POS	NEG	NEG	NEG	NEG	NEG	NEG	NEG	NEG	NEG	NEG	NEG	NEG	NEG	POS	POS	NEG
Perth_2015-S31904	POS	POS	POS	NEG	NEG	NEG	POS	NEG	NEG	NEG	NEG	NEG	NEG	NEG	POS	POS	POS	POS
Perth_2015-S31913	POS	POS	POS	NEG	NEG	NEG	POS	NEG	NEG	NEG	NEG	NEG	NEG	NEG	POS	POS	POS	POS
Perth_2015-S31918	NEG	POS	NEG	NEG	NEG	NEG	POS	NEG	NEG	NEG	NEG	NEG	NEG	NEG	NEG	NEG	NEG	NEG
Dublin-DSH_AR44_E1183	POS	POS	POS	neg	NEG	NEG	POS	NEG	NEG	NEG	NEG	NEG	NEG	NEG	POS	POS	POS	POS
Dublin-DSH_Unfamiliar-3_E1520	POS	POS	POS	neg	NEG	NEG	POS	NEG	NEG	NEG	NEG	NEG	NEG	NEG	POS	POS	POS	POS
Hong Kong_042	POS	POS	POS	neg	NEG	NEG	POS	NEG	NEG	NEG	NEG	NEG	NEG	NEG	POS	POS	POS	POS
Hong Kong_101	POS	POS	POS	neg	NEG	NEG	POS	NEG	NEG	NEG	NEG	NEG	NEG	NEG	POS	POS	POS	POS
Hong Kong_111	POS	POS	POS	neg	NEG	NEG	POS	NEG	NEG	NEG	NEG	NEG	NEG	NEG	POS	POS	POS	POS
Hong Kong_113	POS	POS	POS	neg	NEG	NEG	POS	NEG	NEG	NEG	NEG	NEG	NEG	NEG	POS	POS	POS	POS
Hong Kong_115	POS	POS	POS	NEG	NEG	NEG	POS	NEG	NEG	NEG	NEG	NEG	NEG	NEG	POS	POS	POS	POS
Hong Kong_118	POS	POS	POS	neg	NEG	NEG	POS	NEG	NEG	NEG	NEG	NEG	NEG	NEG	POS	POS	POS	POS
Hong Kong_125	POS	POS	POS	neg	NEG	NEG	POS	NEG	NEG	NEG	NEG	NEG	NEG	NEG	POS	POS	POS	POS
Hong Kong_129	POS	POS	POS	NEG	NEG	NEG	POS	NEG	NEG	NEG	NEG	NEG	NEG	NEG	POS	POS	POS	POS
Hong Kong_133	POS	POS	POS	neg	NEG	NEG	POS	NEG	NEG	NEG	NEG	NEG	NEG	NEG	POS	POS	POS	POS
Kuwait_009	POS	POS	POS	neg	NEG	NEG	POS	NEG	NEG	NEG	NEG	NEG	NEG	NEG	POS	POS	POS	POS
Kuwait_103	POS	POS	POS	neg	NEG	NEG	POS	NEG	NEG	NEG	NEG	NEG	NEG	NEG	POS	POS	POS	POS
Kuwait_107	POS	POS	POS	neg	NEG	NEG	POS	NEG	NEG	NEG	NEG	NEG	NEG	NEG	POS	POS	POS	POS
Perth_03 AH 88	POS	POS	POS	neg	NEG	NEG	POS	NEG	NEG	NEG	NEG	NEG	NEG	NEG	POS	POS	POS	POS
Perth_08-16905	POS	POS	POS	neg	NEG	NEG	POS	NEG	NEG	NEG	NEG	NEG	NEG	NEG	POS	POS	POS	POS
Riyadh_Alfaisal-10_SICU_13_B5672_1117353	POS	POS	POS	neg	NEG	NEG	POS	NEG	NEG	NEG	NEG	NEG	NEG	NEG	POS	POS	POS	POS
Riyadh_SSTI_17_3508602	POS	POS	POS	neg	NEG	NEG	POS	NEG	NEG	NEG	NEG	NEG	NEG	NEG	POS	POS	POS	POS
Riyadh-2822088-R	POS	POS	POS	neg	NEG	NEG	POS	NEG	NEG	NEG	NEG	NEG	NEG	NEG	POS	POS	POS	POS
Trinidad&Tobago_2012_195 (619/12)	POS	POS	POS	neg	NEG	NEG	POS	NEG	NEG	NEG	NEG	NEG	NEG	NEG	POS	POS	POS	POS
Trinidad&Tobago_2012_370 (1112-52177)	POS	POS	POS	neg	NEG	NEG	POS	NEG	NEG	NEG	NEG	NEG	NEG	NEG	POS	POS	POS	POS
Trinidad&Tobago_2012_378 (4139)	POS	POS	POS	neg	NEG	NEG	POS	NEG	NEG	NEG	NEG	NEG	NEG	NEG	POS	POS	POS	POS
Trinidad&Tobago_2013_PA21	POS	POS	POS	neg	NEG	NEG	POS	NEG	NEG	NEG	NEG	NEG	NEG	NEG	POS	POS	POS	POS
Trinidad&Tobago_2013_PA22	POS	POS	POS	neg	NEG	NEG	POS	NEG	NEG	NEG	NEG	NEG	NEG	NEG	POS	POS	POS	POS
Trinidad&Tobago_2912_340 (8523/12)	POS	POS	POS	neg	NEG	NEG	POS	NEG	NEG	NEG	NEG	NEG	NEG	NEG	POS	POS	AMB	POS
Trinidad&Tobago_MRSA2010_105	POS	POS	POS	neg	NEG	NEG	POS	NEG	NEG	NEG	NEG	NEG	NEG	NEG	POS	POS	POS	POS
Trinidad&Tobago_MRSA2010_108	POS	POS	POS	neg	NEG	NEG	POS	NEG	NEG	NEG	NEG	NEG	NEG	NEG	POS	POS	POS	POS
Trinidad&Tobago_MRSA2010_33	POS	POS	POS	neg	NEG	NEG	POS	NEG	NEG	NEG	NEG	NEG	NEG	NEG	POS	POS	POS	POS
Trinidad&Tobago_MRSA2010_79	POS	POS	POS	neg	NEG	NEG	POS	NEG	NEG	NEG	NEG	NEG	NEG	NEG	POS	POS	POS	POS
Trinidad&Tobago_MRSA2010_88	POS	POS	POS	neg	NEG	NEG	POS	NEG	NEG	NEG	NEG	NEG	NEG	NEG	POS	POS	POS	POS
Trinidad&Tobago_MRSA2010_97	POS	POS	POS	neg	NEG	NEG	POS	NEG	NEG	NEG	NEG	NEG	NEG	NEG	POS	POS	POS	POS
Trinidad&Tobago_SSTI 2012_031	POS	POS	POS	neg	NEG	NEG	POS	NEG	NEG	NEG	NEG	NEG	NEG	NEG	POS	POS	POS	POS
Uganda-29_17148_250811	POS	POS	POS	neg	NEG	NEG	POS	NEG	NEG	NEG	NEG	NEG	NEG	NEG	POS	POS	POS	POS
>"South-East Asian Clad																		
UCIM6015	POS	POS	POS	NEG	NEG	NEG	POS	NEG	NEG	NEG	NEG	NEG	NEG	NEG	POS	POS	POS	NEG
Hong Kong_121	POS	POS	POS	neg	NEG	NEG	POS	NEG	NEG	NEG	NEG	NEG	NEG	NEG	POS	POS	POS	NEG
>"South-East Asian Clad																		
Z172 (Taiwan)	POS	POS	POS	NEG	NEG	NEG	POS	NEG	NEG	NEG	NEG	NEG	NEG	NEG	NEG	POS	NEG	POS
Russia-19_0342_Kurgan	POS	POS	POS	NEG	NEG	NEG	POS	NEG	NEG	NEG	NEG	NEG	NEG	NEG	NEG	POS	NEG	POS
>"South-East Asian Clad																		
Perth_01-15392	POS	POS	POS	neg	NEG	NEG	NEG	NEG	NEG	NEG	NEG	NEG	NEG	NEG	NEG	POS	POS	POS
>"South-East Asian Clad																		
XN108 (China)	POS	POS	POS	NEG	NEG	NEG	POS	NEG	NEG	NEG	NEG	NEG	NEG	NEG	NEG	POS	POS	POS
NMR09 (India)	POS	POS	POS	NEG	NEG	NEG	POS	NEG	NEG	NEG	NEG	NEG	NEG	NEG	NEG	POS	POS	POS
346 (Malaysia)	POS	POS	POS	NEG	NEG	NEG	POS	NEG	NEG	NEG	NEG	NEG	NEG	NEG	NEG	POS	POS	POS
DS_009 (Thailand)	POS	POS	POS	NEG	NEG	NEG	POS	NEG	NEG	NEG	NEG	NEG	NEG	NEG	NEG	POS	POS	POS
18ANR581005 (Ind)	POS	POS	POS	NEG	NEG	NEG	POS	NEG	NEG	NEG	NEG	NEG	NEG	NEG	NEG	POS	POS	POS
Bengalaru_ST20141405	POS	POS	POS	NEG	AMB	NEG	POS	NEG	NEG	NEG	NEG	NEG	NEG	NEG	NEG	POS	POS	POS
Bengalaru_ST20141406	POS	POS	POS	NEG	NEG	NEG	POS	NEG	NEG	NEG	NEG	NEG	NEG	NEG	NEG	POS	POS	POS
Bengalaru_ST20141417	POS	POS	POS	NEG	NEG	NEG	POS	NEG	NEG	NEG	NEG	NEG	NEG	NEG	NEG	POS	POS	POS
Hong Kong_107	POS	POS	POS	neg	NEG	NEG	POS	NEG	NEG	NEG	NEG	NEG	NEG	NEG	NEG	POS	POS	POS
Hong Kong_12	POS	POS	POS	neg	NEG	NEG	POS	NEG	NEG	NEG	NEG	NEG	NEG	NEG	NEG	POS	POS	POS
Hong Kong_132	POS	POS	POS	neg	NEG	NEG	POS	NEG	NEG	NEG	NEG	NEG	NEG	NEG	NEG	POS	POS	POS
Riyadh-3028763-R	POS	POS	POS	NEG	NEG	NEG	POS	NEG	NEG	NEG	NEG	NEG	NEG	NEG	NEG	POS	POS	POS
Trinidad&Tobago_MRSA2010_94	POS	POS	POS	neg	NEG	NEG	POS	NEG	NEG	NEG	NEG	NEG	NEG	NEG	NEG	POS	POS	POS
>"South-East Asian Clad																		
M705 (Thailand)	POS	POS	POS	NEG	NEG	NEG	POS	NEG	NEG	NEG	NEG	NEG	NEG	NEG	POS	POS	POS	POS
S85 (Thailand)	POS	POS	POS	NEG	NEG	NEG	POS	NEG	NEG	NEG	NEG	NEG	NEG	NEG	POS	POS	POS	POS
Riyadh_Alfaisal-04_281014_S5076	POS	POS	POS	neg	NEG	NEG	POS	NEG	NEG	NEG	NEG	NEG	NEG	NEG	POS	POS	NEG	POS
Riyadh_Alfaisal-26_S15145_1104163	POS	POS	POS	neg	NEG	NEG	POS	NEG	NEG	NEG	NEG	NEG	NEG	NEG	POS	POS	POS	POS
Riyadh-2817437-W	POS	POS	POS	neg	NEG	NEG	POS	NEG	NEG	NEG	NEG	NEG	NEG	NEG	POS	POS	POS	POS
>"South-East Asian Clad																		
PPUKM-775-2009 (Malaysia)	POS	POS	POS	NEG	NEG	NEG	NEG	NEG	NEG	NEG	NEG	NEG	NEG	NEG	NEG	POS	POS	POS
Hong Kong_138	POS	POS	POS	neg	NEG	NEG	NEG	NEG	NEG	NEG	NEG	NEG	NEG	NEG	NEG	POS	POS	POS
>"South-East Asian Clad																		
Kuwait_001	POS	POS	POS	NEG	NEG	NEG	POS	NEG	NEG	NEG	NEG	NEG	NEG	NEG	POS	POS	POS	POS
Kuwait_337	POS	POS	POS	neg	NEG	NEG	POS	POS	NEG	NEG	NEG	NEG	NEG	NEG	POS	POS	AMB	POS
>"South-East Asian Clad																		
CUHK_HK2007 (China)	POS	POS	POS	NEG	NEG	NEG	POS	NEG	NEG	NEG	NEG	NEG	NEG	NEG	NEG	POS	POS	POS
NMR02 (India)	POS	POS	POS	NEG	NEG	NEG	POS	NEG	NEG	NEG	NEG	NEG	NEG	NEG	NEG	POS	POS	POS
VB1490 (India)	POS	POS	POS	NEG	NEG	NEG	POS	NEG	NEG	NEG	NEG	NEG	NEG	NEG	NEG	POS	POS	POS
V521 (Korea)	POS	POS	POS	NEG	NEG	NEG	POS	NEG	NEG	NEG	NEG	NEG	NEG	NEG	NEG	POS	POS	POS
HST-077 (Lebanon)	POS	POS	POS	NEG	NEG	NEG	POS	NEG	NEG	NEG	NEG	NEG	NEG	NEG	NEG	POS	POS	POS
MAL1 (Malaysia)	POS	POS	POS	NEG	NEG	NEG	POS	NEG	NEG	NEG	NEG	NEG	NEG	NEG	NEG	POS	POS	POS
MAL3 (Malaysia)	POS	POS	POS	NEG	NEG	NEG	POS	NEG	NEG	NEG	NEG	NEG	NEG	NEG	NEG	POS	POS	POS
PPUKM-261-2009 (Malaysia)	POS	POS	POS	NEG	NEG	NEG	POS	NEG	NEG	NEG	NEG	NEG	NEG	NEG	NEG	POS	POS	POS
PPUKM-332-2009 (Malaysia)	POS	POS	POS	NEG	NEG	NEG	POS	NEG	NEG	NEG	NEG	NEG	NEG	NEG	NEG	POS	POS	POS
S38 (Thailand)	POS	POS	POS	NEG	NEG	NEG	POS	NEG	NEG	NEG	NEG	NEG	NEG	NEG	NEG	POS	POS	POS
S7 (Thailand)	POS	AMB	POS	NEG	NEG	NEG	POS	NEG	NEG	NEG	NEG	NEG	NEG	NEG	NEG	POS	POS	POS
US_008 (Thailand)	POS	POS	POS	NEG	NEG	NEG	POS	NEG	NEG	NEG	NEG	NEG	NEG	NEG	NEG	POS	POS	POS
US_030 (Thailand)	POS	POS	POS	NEG	NEG	NEG	POS	NEG	NEG	NEG	NEG	NEG	NEG	NEG	NEG	POS	POS	POS
M170 (Tsunami-related)	POS	POS	POS	NEG	NEG	NEG	POS	NEG	NEG	NEG	NEG	NEG	NEG	NEG	NEG	POS	POS	POS
BSAC697 (UK)	POS	POS	POS	NEG	NEG	NEG	POS	NEG	NEG	NEG	NEG	NEG	NEG	NEG	NEG	POS	POS	POS
IS-189	POS	POS	POS	NEG	NEG	NEG	POS	NEG	NEG	NEG	NEG	NEG	NEG	NEG	NEG	POS	POS	POS
MRGR3	POS	POS	POS	NEG	NEG	NEG	POS	NEG	NEG	NEG	NEG	NEG	NEG	NEG	NEG	POS	POS	POS
smg07	POS	POS	POS	NEG	NEG	NEG	POS	NEG	NEG	NEG	NEG	NEG	NEG	NEG	NEG	POS	POS	POS
AH1-AUS-EMRSA-3	POS	POS	POS	neg	NEG	NEG	POS	NEG	NEG	NEG	NEG	NEG	NEG	NEG	NEG	POS	POS	POS
Bengalaru_ST20141403	POS	POS	POS	NEG	NEG	NEG	POS	NEG	NEG	NEG	NEG	NEG	NEG	NEG	NEG	POS	POS	POS
Finland E24_98541_Harmony	POS	POS	POS	neg	NEG	NEG	POS	NEG	NEG	NEG	NEG	NEG	NEG	NEG	NEG	POS	POS	POS
Hong Kong_10	POS	POS	POS	neg	NEG	NEG	POS	NEG	NEG	NEG	NEG	NEG	NEG	NEG	NEG	POS	POS	POS
Hong Kong_100	POS	POS	POS	neg	NEG	NEG	POS	NEG	NEG	NEG	NEG	NEG	NEG	NEG	NEG	POS	POS	POS
Hong Kong_199	POS	POS	POS	neg	NEG	NEG	POS	NEG	NEG	NEG	NEG	NEG	NEG	NEG	NEG	POS	POS	POS
Hong Kong_34	POS	POS	POS	neg	NEG	NEG	POS	NEG	NEG	NEG	NEG	NEG	NEG	NEG	NEG	POS	POS	POS
Hong Kong_88	POS	POS	POS	neg	NEG	NEG	POS	NEG	NEG	NEG	NEG	NEG	NEG	NEG	NEG	POS	POS	POS
Hong Kong_92	POS	POS	POS	neg	NEG	NEG	POS	NEG	NEG	NEG	NEG	NEG	NEG	NEG	NEG	POS	POS	POS
Hong Kong_94	POS	POS	POS	neg	NEG	NEG	POS	NEG	NEG	NEG	NEG	NEG	NEG	NEG	NEG	POS	POS	POS
Kuwait_257	POS	POS	POS	neg	NEG	NEG	POS	NEG	NEG	NEG	NEG	NEG	NEG	NEG	NEG	POS	POS	POS
Perth_01-15330	POS	POS	POS	neg	NEG	NEG	POS	NEG	NEG	NEG	NEG	NEG	NEG	NEG	NEG	POS	POS	POS
Perth_01-15349	POS	POS	POS	neg	NEG	NEG	POS	NEG	NEG	NEG	NEG	NEG	NEG	NEG	NEG	POS	POS	POS
Perth_01-16176	POS	POS	POS	neg	NEG	NEG	POS	NEG	NEG	NEG	NEG	NEG	NEG	NEG	NEG	POS	POS	POS
Perth_WBG8379	POS	POS	POS	neg	NEG	NEG	POS	NEG	NEG	NEG	NEG	NEG	NEG	NEG	NEG	POS	POS	POS
Rawalpindi_Kidney Center_56	POS	POS	POS	NEG	NEG	NEG	POS	NEG	NEG	NEG	NEG	NEG	NEG	NEG	NEG	POS	POS	POS
Riyadh_SSTI_05_3464614	POS	POS	POS	neg	NEG	NEG	POS	NEG	NEG	NEG	NEG	NEG	NEG	NEG	NEG	POS	POS	POS
Trinidad&Tobago_2012_157	POS	POS	POS	neg	NEG	NEG	POS	NEG	NEG	NEG	NEG	NEG	NEG	NEG	NEG	POS	POS	POS

STRAIN / ISOLATE	METHICILLIN RESISTANCE AND SCCmec TYPING																	
	fudoh-PSM	cstB-SCC1 (ex Q2G1R6 - SCCmec	xylR/mecR2	mecC	blaZ (SCCmec XI)	plsSCC (COL)	mvaS-SCC	cstB-SCC2 (Q2G1R6)	Q9S0M4	kdpA-SCC	kdpB-SCC	kdpC-SCC	kdpD-SCC	kdpE-SCC	Q93IB7	D1GU38		Q933A2
	fudoh-PSM	cstB-SCC1 (Q2G1R6)	xylR/mecR2	mecC	blaZ (SCCmec XI)	pls-SCC (COL)	mvaS-SCC	cstB-SCC2 (Q2G1R6)	Q9S0M4	kdpA-SCC	kdpB-SCC	kdpC-SCC	kdpD-SCC	kdpE-SCC	Q93IB7	D1GU38	D1GU38 (TW20)	Q933A2
	Phenol soluble modulin from SCCmec	CsoR-like sulfur transferase-regulated genes B/metallo-beta-lactamase superfamily protein. Pseudogene containing two stop	Methicillin resistance operon repressor 2, Homolog of xylose repressor. Located next to mec operon downstream of mecI (not present if mecI is truncated)	Alternate gene encoding a modified penicillin binding protein . Present in, and characteristic for, SCCmec XI	Beta-lactamase from SCCmec XI	Plasmin-sensitive surface protein, prevents bacterial adhesion in vitro, located in SCC, close to mec operon. Subtyping SCCmec I	Truncated 3-hydroxy-3-methylglutaryl CoA synthase. Subtyping SCCmec I, II, IV, V	CsoR-like sulfur transferase-regulated genes B/metallo-beta-lactamase superfamily protein. Present in SCCmec IVa (truncated) and	Putative protein. Subtyping SCCmec I, SCCmec/ACME composites and SCCmec from WA40	Potassium-translocating ATPase A, chain 2	Potassium-transporting ATPase B, chain 1	Potassium-translocating ATPase C, chain 2	Sensor kinase protein	KDP operon transcriptional regulatory protein	LytTR domain DNA-binding regulator. Subtyping SCCmec III (HF569113.1)	Putative protein. Subtyping SCCmec III, identification of SCCmec VT, SCCmec ZH47, SCCmec VII because of an association with (additional/second)	Putative protein	Putative ADP-ribosyltransferase. Subtyping SCCmec III and SCCmec IX
>"South-East Asian Clad																		
M418	POS	POS	POS	NEG	NEG	NEG	POS	NEG	POS	NEG	NEG	NEG	NEG	NEG	NEG	POS	POS	POS
>"South-East Asian Clad																		
NMR07 (India)	POS	POS	POS	NEG	NEG	NEG	POS	NEG	POS	NEG	NEG	NEG	NEG	NEG	NEG	POS	POS	POS
NMR08 (India)	POS	POS	POS	NEG	NEG	NEG	POS	NEG	POS	NEG	NEG	NEG	NEG	NEG	NEG	POS	POS	POS
Bengalaru_ST20121978	POS	POS	POS	NEG	NEG	NEG	POS	NEG	POS	NEG	NEG	NEG	NEG	NEG	NEG	POS	POS	POS
Hong Kong_89	POS	POS	POS	neg	NEG	NEG	POS	NEG	POS	NEG	NEG	NEG	NEG	NEG	NEG	POS	NEG	POS
>"South-East Asian Clad																		
Perth_2005_AH_5	POS	POS	POS	neg	NEG	NEG	POS	NEG	POS	NEG	NEG	NEG	NEG	NEG	POS	NEG	NEG	POS
>"South-East Asian Clad																		
Trinidad&Tobago_MRSA2010_103	POS	POS	POS	neg	NEG	NEG	POS	NEG	NEG	NEG	NEG	NEG	NEG	NEG	NEG	POS	POS	POS
Trinidad&Tobago_2013_PA25	POS	POS	POS	neg	NEG	NEG	POS	NEG	NEG	NEG	NEG	NEG	NEG	NEG	NEG	POS	POS	POS
Trinidad&Tobago_MRSA2010_110	POS	POS	POS	neg	NEG	NEG	POS	NEG	NEG	NEG	NEG	NEG	NEG	NEG	NEG	POS	POS	POS
Trinidad&Tobago_SSTI 2012_111	POS	POS	POS	neg	NEG	NEG	POS	NEG	NEG	NEG	NEG	NEG	NEG	NEG	NEG	POS	POS	POS
Trinidad&Tobago_MRSA2010_158	POS	POS	POS	neg	NEG	NEG	POS	NEG	NEG	NEG	NEG	NEG	NEG	NEG	NEG	POS	POS	POS
>"South-East Asian Clad																		
Kuwait_122	POS	POS	POS	neg	NEG	NEG	POS	NEG	NEG	NEG	NEG	NEG	NEG	NEG	POS	POS	POS	POS
Kuwait_002	POS	POS	POS	NEG	NEG	NEG	POS	NEG	NEG	NEG	NEG	NEG	NEG	NEG	POS	POS	POS	POS
Kuwait_196	POS	POS	POS	neg	NEG	NEG	POS	NEG	NEG	NEG	NEG	NEG	NEG	NEG	NEG	POS	POS	POS
Kuwait_199	POS	POS	POS	NEG	NEG	NEG	POS	NEG	NEG	NEG	NEG	NEG	NEG	NEG	NEG	POS	POS	POS
Hong Kong_136	POS	POS	POS	neg	NEG	NEG	POS	NEG	NEG	NEG	NEG	NEG	NEG	NEG	POS	NEG	POS	POS
>"South-East Asian Clad																		
P32 (Poland)	POS	POS	POS	NEG	NEG	NEG	POS	NEG	NEG	NEG	NEG	NEG	NEG	NEG	POS	NEG	NEG	POS
>"South-East Asian Clad																		
Perth_2015-S32313	POS	POS	POS	NEG	NEG	NEG	POS	NEG	NEG	NEG	NEG	NEG	NEG	NEG	POS	NEG	NEG	POS
>"hla-negative South-Ea																		
DEN907 (Denmark)	POS	POS	POS	NEG	NEG	NEG	NEG	NEG	NEG	NEG	NEG	NEG	NEG	NEG	NEG	NEG	NEG	POS
>"hla-negative South-Ea																		
S26 (Thailand)	POS	POS	POS	NEG	NEG	NEG	NEG	NEG	NEG	NEG	NEG	NEG	NEG	NEG	NEG	NEG	NEG	POS
S42 (Thailand)	POS	POS	POS	NEG	NEG	NEG	NEG	NEG	NEG	NEG	NEG	NEG	NEG	NEG	NEG	NEG	NEG	POS
S97 (Thailand)	POS	POS	POS	NEG	NEG	NEG	NEG	NEG	NEG	NEG	NEG	NEG	NEG	NEG	NEG	NEG	NEG	POS
S78 (Thailand)	POS	POS	POS	NEG	NEG	NEG	NEG	NEG	NEG	NEG	NEG	NEG	NEG	NEG	NEG	NEG	NEG	POS
S25 (Thailand)	POS	POS	POS	NEG	NEG	NEG	NEG	NEG	NEG	NEG	NEG	NEG	NEG	NEG	NEG	NEG	NEG	POS
S21 (Thailand)	POS	POS	POS	NEG	NEG	NEG	NEG	NEG	NEG	NEG	NEG	NEG	NEG	NEG	NEG	NEG	NEG	POS
S106 (Thailand)	POS	POS	POS	NEG	NEG	NEG	NEG	NEG	NEG	NEG	NEG	NEG	NEG	NEG	NEG	NEG	NEG	POS
S24 (Thailand)	POS	POS	POS	NEG	NEG	NEG	NEG	NEG	NEG	NEG	NEG	NEG	NEG	NEG	NEG	NEG	NEG	POS
S39 (Thailand)	POS	POS	POS	NEG	NEG	NEG	NEG	NEG	NEG	NEG	NEG	NEG	NEG	NEG	NEG	NEG	NEG	POS
S2 (Thailand)	POS	POS	POS	NEG	NEG	NEG	NEG	NEG	NEG	NEG	NEG	NEG	NEG	NEG	NEG	NEG	NEG	POS
S81 (Thailand)	POS	POS	POS	NEG	NEG	NEG	NEG	NEG	NEG	NEG	NEG	NEG	NEG	NEG	NEG	NEG	NEG	POS
M116 (Vietnam)	POS	POS	POS	NEG	NEG	NEG	NEG	NEG	NEG	NEG	NEG	NEG	NEG	NEG	NEG	NEG	NEG	POS
>"Portuguese Clade": Pc																		
FFP103 (Portugal)	POS	POS	POS	NEG	NEG	NEG	POS	NEG	NEG	NEG	NEG	NEG	NEG	NEG	POS	NEG	NEG	POS
HDG2 (Portugal)	POS	POS	POS	NEG	NEG	NEG	POS	NEG	NEG	NEG	NEG	NEG	NEG	NEG	POS	NEG	NEG	POS
HSA10 (Portugal)	POS	POS	POS	NEG	NEG	NEG	POS	NEG	NEG	NEG	NEG	NEG	NEG	NEG	POS	NEG	NEG	POS
HSA11 (Portugal)	POS	POS	POS	NEG	NEG	NEG	POS	NEG	NEG	NEG	NEG	NEG	NEG	NEG	POS	NEG	NEG	POS
ICP5011 (Portugal)	POS	POS	POS	NEG	NEG	NEG	POS	NEG	NEG	NEG	NEG	NEG	NEG	NEG	POS	NEG	NEG	POS
ICP5014 (Portugal)	POS	POS	POS	NEG	NEG	NEG	POS	NEG	NEG	NEG	NEG	NEG	NEG	NEG	POS	NEG	NEG	POS
ICP5062 (Portugal)	POS	POS	POS	NEG	NEG	NEG	POS	NEG	NEG	NEG	NEG	NEG	NEG	NEG	POS	NEG	NEG	POS
Strain 1063_ATCC 33592 FDAARGOS_3 (USA)	POS	POS	POS	NEG	NEG	NEG	POS	NEG	NEG	NEG	NEG	NEG	NEG	NEG	POS	NEG	NEG	POS
	POS	POS	POS			NEG	POS	NEG	NEG	NEG	NEG	NEG	NEG	NEG	POS	NEG	NEG	POS
Russia-07_0110_Kurgan	POS	AMB	POS	NEG	NEG	NEG	POS	NEG	NEG	NEG	NEG	NEG	NEG	NEG	POS	NEG	NEG	POS
Russia-11_0164_Kurgan	POS	POS	POS	NEG	NEG	NEG	POS	NEG	NEG	NEG	NEG	NEG	NEG	NEG	POS	NEG	NEG	POS
Russia-20_0367_Kurgan	POS	POS	POS	NEG	NEG	NEG	POS	NEG	NEG	NEG	NEG	NEG	NEG	NEG	POS	NEG	NEG	POS
Russia-21_0390_Chelyabinsk	POS	POS	POS	NEG	NEG	NEG	POS	NEG	NEG	NEG	NEG	NEG	NEG	NEG	POS	NEG	NEG	POS
>"South American/ Mid																		
AGT1 (Argentina)	POS	POS	POS	NEG	NEG	NEG	POS	NEG	NEG	NEG	NEG	NEG	NEG	NEG	NEG	POS	POS	POS
AGT120 (Argentina)	POS	POS	POS	NEG	NEG	NEG	POS	NEG	NEG	NEG	NEG	NEG	NEG	NEG	NEG	POS	POS	POS
AGT67 (Argentina)	POS	POS	POS	NEG	NEG	NEG	POS	NEG	NEG	NEG	NEG	NEG	NEG	NEG	NEG	POS	POS	POS
AGT9 (Argentina)	POS	POS	POS	NEG	NEG	NEG	POS	NEG	NEG	NEG	NEG	NEG	NEG	NEG	NEG	POS	POS	POS
RA3 (Argentina)	POS	POS	POS	NEG	NEG	NEG	POS	NEG	NEG	NEG	NEG	NEG	NEG	NEG	NEG	POS	POS	POS
RA6 (Argentina)	POS	POS	POS	NEG	NEG	NEG	POS	NEG	NEG	NEG	NEG	NEG	NEG	NEG	NEG	POS	POS	POS
RA7 (Argentina)	POS	POS	POS	NEG	NEG	NEG	POS	NEG	NEG	NEG	NEG	NEG	NEG	NEG	NEG	POS	POS	POS
Be62 (Brazil)	POS	POS	POS	NEG	NEG	NEG	POS	NEG	NEG	NEG	NEG	NEG	NEG	NEG	NEG	POS	POS	POS
Bmb9393 (Brazil)	POS	POS	POS	NEG	NEG	NEG	POS	NEG	NEG	NEG	NEG	NEG	NEG	NEG	NEG	POS	POS	POS
BRA36 (Brazil)	POS	POS	POS	NEG	NEG	NEG	POS	NEG	NEG	NEG	NEG	NEG	NEG	NEG	NEG	POS	POS	POS
BZ48 (Brazil)	POS	POS	POS	NEG	NEG	NEG	POS	NEG	NEG	NEG	NEG	NEG	NEG	NEG	NEG	POS	POS	POS
Gv51 (Brazil)	POS	POS	POS	NEG	NEG	NEG	POS	NEG	NEG	NEG	NEG	NEG	NEG	NEG	NEG	POS	POS	POS
Gv69 (Brazil)	POS	POS	POS	NEG	NEG	NEG	POS	NEG	NEG	NEG	NEG	NEG	NEG	NEG	NEG	POS	POS	POS
Gv88 (Brazil)	POS	POS	POS	NEG	NEG	NEG	POS	NEG	NEG	NEG	NEG	NEG	NEG	NEG	NEG	POS	POS	POS
HC1335 (Brazil)	POS	POS	POS	NEG	NEG	NEG	POS	NEG	NEG	NEG	NEG	NEG	NEG	NEG	NEG	POS	POS	POS
HC1340 (Brazil)	POS	POS	POS	NEG	NEG	NEG	POS	NEG	NEG	NEG	NEG	NEG	NEG	NEG	NEG	POS	POS	POS
HC556 (Brazil)	POS	POS	POS	NEG	NEG	NEG	POS	NEG	NEG	NEG	NEG	NEG	NEG	NEG	NEG	POS	POS	POS
HU25 (Brazil)	POS	POS	POS	NEG	NEG	NEG	POS	NEG	NEG	NEG	NEG	NEG	NEG	NEG	NEG	POS	POS	POS
UB563 (Brazil)	POS	POS	POS	NEG	NEG	NEG	POS	NEG	NEG	NEG	NEG	NEG	NEG	NEG	NEG	POS	POS	POS
2A8 (Czech Republic)	POS	POS	POS	NEG	NEG	NEG	POS	NEG	NEG	NEG	NEG	NEG	NEG	NEG	NEG	POS	POS	POS
M1229 (Denmark)	POS	POS	POS	NEG	NEG	NEG	POS	NEG	NEG	NEG	NEG	NEG	NEG	NEG	NEG	POS	POS	POS
FRICAR (France)	POS	POS	POS	NEG	NEG	NEG	POS	NEG	NEG	NEG	NEG	NEG	NEG	NEG	NEG	POS	POS	POS
D90 (Germany)	POS	POS	POS	NEG	NEG	NEG	POS	NEG	NEG	NEG	NEG	NEG	NEG	NEG	NEG	POS	POS	POS
LIT68 (Lithuania)	POS	POS	POS	NEG	NEG	NEG	POS	NEG	NEG	NEG	NEG	NEG	NEG	NEG	NEG	POS	POS	POS
LIT76 (Lithuania)	POS	AMB	POS	NEG	NEG	NEG	POS	NEG	NEG	NEG	NEG	NEG	NEG	NEG	NEG	POS	POS	POS
HGSA142 (Portugal)	POS	POS	POS	NEG	NEG	NEG	POS	NEG	NEG	NEG	NEG	NEG	NEG	NEG	NEG	POS	POS	POS
HGSA9 (Portugal)	POS	POS	POS	NEG	NEG	NEG	POS	NEG	NEG	NEG	NEG	NEG	NEG	NEG	NEG	POS	POS	POS
HSJ216 (Portugal)	POS	POS	POS	NEG	NEG	NEG	POS	NEG	NEG	NEG	NEG	NEG	NEG	NEG	NEG	POS	POS	POS
M278 (Portugal)	POS	POS	POS	NEG	NEG	NEG	POS	NEG	NEG	NEG	NEG	NEG	NEG	NEG	NEG	POS	POS	POS
ES26 (Spain)	POS	POS	POS	NEG	NEG	NEG	POS	NEG	NEG	NEG	NEG	NEG	NEG	NEG	NEG	POS	POS	POS
UK102 (UK)	POS	POS	POS	NEG	NEG	NEG	POS	NEG	NEG	NEG	NEG	NEG	NEG	NEG	NEG	POS	POS	POS
URU34 (Uruguay)	POS	POS	POS	NEG	NEG	NEG	POS	NEG	NEG	NEG	NEG	NEG	NEG	NEG	NEG	POS	POS	POS
IS-125	POS	POS	POS	NEG	NEG	NEG	POS	NEG	NEG	NEG	NEG	NEG	NEG	NEG	NEG	POS	POS	POS
IS-157	POS	POS	POS	NEG	NEG	NEG	POS	NEG	NEG	NEG	NEG	NEG	NEG	NEG	NEG	POS	POS	POS
Ecuador_1	POS	POS	POS	NEG	NEG	NEG	POS	NEG	NEG	NEG	NEG	NEG	NEG	NEG	NEG	POS	POS	POS
Ecuador_2	POS	POS	POS	NEG	NEG	NEG	POS	NEG	NEG	NEG	NEG	NEG	NEG	NEG	NEG	POS	POS	POS
Dresden_15ANR574263	POS	POS	POS	neg	NEG	NEG	POS	NEG	NEG	NEG	NEG	NEG	NEG	NEG	NEG	POS	POS	POS
UK-EMRSA-11	POS	POS	POS	neg	NEG	NEG	POS	NEG	NEG	NEG	NEG	NEG	NEG	NEG	NEG	POS	POS	POS
>"South American/ Mid																		
BRA2 (Brazil)	POS	POS	POS	NEG	NEG	NEG	POS	NEG	NEG	NEG	NEG	NEG	NEG	NEG	NEG	NEG	NEG	POS
CHL1 (Chile)	POS	POS	POS	NEG	NEG	NEG	POS	NEG	NEG	NEG	NEG	NEG	NEG	NEG	NEG	NEG	NEG	POS
CHL151 (Chile)	POS	POS	POS	NEG	NEG	NEG	POS	NEG	NEG	NEG	NEG	NEG	NEG	NEG	NEG	NEG	NEG	POS
UCL378 (Chile)	POS	POS	POS	NEG	NEG	NEG	POS	NEG	NEG	NEG	NEG	NEG	NEG	NEG	NEG	NEG	NEG	POS
UP72 (Peru)	POS	POS	POS	NEG	NEG	NEG	POS	NEG	NEG	NEG	NEG	NEG	NEG	NEG	NEG	NEG	NEG	POS
UP81 (Peru)	POS	POS	POS	NEG	NEG	NEG	POS	NEG	NEG	NEG	NEG	NEG	NEG	NEG	NEG	NEG	NEG	POS
>"South American/ Mid																		
UP1073 (Peru)	POS	POS	POS	NEG	NEG	NEG	POS	NEG	NEG	NEG	NEG	NEG	NEG	NEG	NEG	NEG	NEG	NEG

STRAIN / ISOLATE	METHICILLIN RESISTANCE AND SCCmec TYPING																	
	fudoh-PSM	cstB-SCC1 (ex Q2G1R6 - sCCmec	xylR/mecR2	mecC	blaZ (SCCmec XI)	plsSCC (COL)	mvaS-SCC	cstB-SCC2 (Q2G1R6)	Q9S0M4	kdpA-SCC	kdpB-SCC	kdpC-SCC	kdpD-SCC	kdpE-SCC	Q93IB7	D1GU38		Q933A2
	fudoh-PSM	cstB-SCC1 (Q2G1R6)	xylR/mecR2	mecC	blaZ (SCCmec XI)	pls-SCC (COL)	mvaS-SCC	cstB-SCC2 (Q2G1R6)	Q9S0M4	kdpA-SCC	kdpB-SCC	kdpC-SCC	kdpD-SCC	kdpE-SCC	Q93IB7	D1GU38	D1GU38 (TW20)	Q933A2
	Phenol soluble modulin from SCCmec	CsoR-like sulfur transferase-regulated genes B/metallo-beta-lactamase superfamily protein. Pseudogene containing two stop	Methicillin resistance operon repressor 2, Homolog of xylose repressor. Located next to mec operon downstream of mecI (not present if mecI is truncated)	Alternate gene encoding a modified penicillin binding protein . Present in, and characteristic for, SCCmec XI	Beta-lactamase from SCCmec XI	Plasmin-sensitive surface protein, prevents bacterial adhesion in vitro, located in SCC, close to mec operon. Subtyping SCCmec I	Truncated 3-hydroxy-3-methylglutaryl CoA synthase. Subtyping SCCmec I, II, IV, V	CsoR-like sulfur transferase-regulated genes B/metallo-beta-lactamase superfamily protein. Present in SCCmec IVa (truncated) and	Putative protein. Subtyping SCCmec I, SCCmec/ACME composites and SCCmec from WA40	Potassium-translocating ATPase A, chain 2	Potassium-transporting ATPase B, chain 1	Potassium-translocating ATPase C, chain 2	Sensor kinase protein	KDP operon transcriptional regulatory protein	LytTR domain DNA-binding regulator. Subtyping SCCmec III (HF569113.1)	Putative protein. Subtyping SCCmec III, identification of SCCmec VT, SCCmec ZH47, SCCmec VII because of an association with (additional/second)	Putative protein	Putative ADP-ribosyltransferase. Subtyping SCCmec III and SCCmec IX
>"South American/ Midd																		
LIT89 (Lithuania)	POS	POS	POS	NEG	NEG	NEG	POS	NEG	NEG	NEG	NEG	NEG	NEG	NEG	NEG	POS	POS	POS
MRSA_PR1 (Malaysia)	POS	POS	POS	NEG	NEG	NEG	POS	NEG	NEG	NEG	NEG	NEG	NEG	NEG	NEG	POS	POS	POS
Riyadh-2888905-R	POS	POS	POS	neg	NEG	NEG	POS	NEG	NEG	NEG	NEG	NEG	NEG	NEG	NEG	POS	POS	POS
Riyadh-2888915-BC	POS	POS	POS	NEG	NEG	NEG	POS	NEG	NEG	NEG	NEG	NEG	NEG	NEG	NEG	POS	POS	POS
Riyadh-2793706-R	POS	POS	POS	neg	NEG	NEG	POS	NEG	NEG	NEG	NEG	NEG	NEG	NEG	NEG	POS	POS	POS
>"South American/ Midd																		
Riyadh_SSTI_18_3502925	POS	POS	POS	neg	NEG	NEG	POS	NEG	NEG	NEG	NEG	NEG	NEG	NEG	POS	POS	POS	POS
Riyadh-2822825-W	POS	POS	POS	neg	NEG	NEG	POS	NEG	NEG	NEG	NEG	NEG	NEG	NEG	POS	POS	POS	POS
>"South American/ Midd																		
UK-EMRSA-9	POS	POS	POS	neg	NEG	NEG	POS	NEG	POS	NEG	NEG	NEG	NEG	NEG	NEG	POS	POS	POS
>"South American/ Midd																		
Lome_HT20020815	POS	POS	POS	NEG	NEG	NEG	POS	NEG	POS	NEG	NEG	NEG	NEG	NEG	NEG	NEG	NEG	POS
>"South American/ Midd																		
NA32 (Denmark)	POS	POS	POS	NEG	NEG	NEG	POS	NEG	NEG	NEG	NEG	NEG	NEG	NEG	POS	POS	POS	POS
H24 (Egypt)	POS	POS	POS	NEG	NEG	NEG	POS	NEG	NEG	NEG	NEG	NEG	NEG	NEG	POS	POS	POS	POS
ATCC BAA-39 (=HUSA304) (Hungary)	POS	POS	POS	NEG	NEG	NEG	POS	NEG	NEG	NEG	NEG	NEG	NEG	NEG	POS	POS	POS	POS
HUSA304 (Hungary)	POS	POS	POS	NEG	NEG	NEG	POS	NEG	NEG	NEG	NEG	NEG	NEG	NEG	POS	POS	POS	POS
HU106 (Hungary)	POS	POS	POS	NEG	NEG	NEG	POS	NEG	NEG	NEG	NEG	NEG	NEG	NEG	POS	POS	POS	POS
BSAC27 (UK)	POS	POS	POS	NEG	NEG	NEG	POS	NEG	NEG	NEG	NEG	NEG	NEG	NEG	POS	POS	POS	POS
NCTC13131, UK-EMRSA-4 (UK)	POS	POS	POS	NEG	NEG	NEG	POS	NEG	NEG	NEG	NEG	NEG	NEG	NEG	POS	POS	POS	POS
	POS	POS	POS	neg	NEG	NEG	POS	NEG	NEG	NEG	NEG	NEG	NEG	NEG	POS	POS	POS	POS
Algiers_HT20040080	POS	POS	POS	NEG	NEG	NEG	POS	NEG	NEG	NEG	NEG	NEG	NEG	NEG	POS	POS	POS	POS
Dublin-DSH_AR09_0.0066	POS	POS	POS	neg	NEG	NEG	POS	NEG	NEG	NEG	NEG	NEG	NEG	NEG	POS	POS	POS	POS
Dublin-DSH_AR09_0-0065	POS	POS	POS	neg	NEG	NEG	POS	NEG	NEG	NEG	NEG	NEG	NEG	NEG	POS	POS	POS	POS
Dublin-DSH_Phenotype-III_84	POS	POS	POS	neg	NEG	NEG	POS	NEG	NEG	NEG	NEG	NEG	NEG	NEG	POS	POS	POS	POS
Hong Kong_130	POS	POS	POS	neg	NEG	NEG	POS	NEG	NEG	NEG	NEG	NEG	NEG	NEG	POS	POS	POS	POS
Kuwait_018	POS	POS	POS	neg	NEG	NEG	POS	NEG	NEG	NEG	NEG	NEG	NEG	NEG	POS	POS	POS	POS
Perth_08-17726	POS	POS	POS	neg	NEG	NEG	POS	NEG	NEG	NEG	NEG	NEG	NEG	NEG	POS	POS	POS	POS
Riyadh_Alfaisal/KKKSUH_86_MRS-14-279	POS	POS	POS	neg	NEG	NEG	POS	NEG	NEG	NEG	NEG	NEG	NEG	NEG	POS	POS	POS	POS
Riyadh_Alfaisal-04_Z3861_831588	POS	POS	POS	neg	NEG	NEG	POS	NEG	NEG	NEG	NEG	NEG	NEG	NEG	POS	POS	POS	POS
Riyadh_Alfaisal-30_S15724_1108013	POS	POS	POS	neg	NEG	NEG	POS	NEG	NEG	NEG	NEG	NEG	NEG	NEG	POS	POS	POS	POS
Riyadh_Alfaisal-6_22A_13_B3992_397721	POS	POS	POS	neg	NEG	NEG	POS	NEG	NEG	NEG	NEG	NEG	NEG	NEG	POS	POS	AMB	POS
Riyadh_SSTI_52_3615482	POS	POS	POS	neg	NEG	NEG	POS	NEG	NEG	NEG	NEG	NEG	NEG	NEG	POS	POS	POS	POS
Riyadh-2817276-2	POS	POS	POS	neg	NEG	NEG	POS	NEG	NEG	NEG	NEG	NEG	NEG	NEG	POS	POS	POS	POS
Riyadh-2891670-W	POS	POS	POS	neg	NEG	NEG	POS	NEG	NEG	NEG	NEG	NEG	NEG	NEG	POS	POS	POS	POS
Riyadh-3006920-W	POS	POS	POS	neg	NEG	NEG	POS	NEG	NEG	NEG	NEG	NEG	NEG	NEG	POS	POS	POS	POS
Riyadh-R2567782	POS	POS	POS	neg	NEG	NEG	POS	NEG	NEG	NEG	NEG	NEG	NEG	NEG	POS	POS	POS	POS
Russia-18_0252_Krasnoyarsk_SK2	POS	POS	POS	NEG	NEG	NEG	POS	NEG	NEG	NEG	NEG	NEG	NEG	NEG	POS	POS	POS	POS
UK-EMRSA-7	POS	POS	POS	neg	NEG	NEG	POS	NEG	NEG	NEG	NEG	NEG	NEG	NEG	POS	POS	POS	POS
>"South American/ Midd																		
Dublin-DSH_AR23_0073	POS	POS	POS	neg	NEG	NEG	POS	NEG	NEG	NEG	NEG	NEG	NEG	NEG	POS	POS	POS	POS
>Related to "South Ame																		
MRSA-OC3 (Russia)	POS	POS	POS	NEG	NEG	NEG	POS	NEG	NEG	NEG	NEG	NEG	NEG	NEG	NEG	POS	POS	POS
Russia-12_0176_Krasnoyarsk	POS	POS	POS	NEG	NEG	NEG	POS	NEG	NEG	NEG	NEG	NEG	NEG	NEG	NEG	POS	POS	POS
Russia-13_0180_Krasnoyarsk	POS	POS	POS	NEG	NEG	NEG	POS	NEG	NEG	NEG	NEG	NEG	NEG	NEG	NEG	POS	POS	POS
Russia-16_0249_Krasnoyarsk	POS	POS	POS	NEG	NEG	NEG	POS	NEG	NEG	NEG	NEG	NEG	NEG	NEG	NEG	POS	POS	POS
Russia-17_0250_Krasnoyarsk	POS	POS	POS	NEG	NEG	NEG	POS	NEG	NEG	NEG	NEG	NEG	NEG	NEG	NEG	POS	NEG	POS
>Related to "South Ame																		
URU110 (Uruguay)	POS	POS	POS	NEG	NEG	NEG	POS	NEG	NEG	NEG	NEG	NEG	NEG	NEG	NEG	POS	POS	POS
>Related to "South Ame																		
DS_014 (Thailand)	POS	POS	POS	NEG	NEG	NEG	POS	NEG	NEG	NEG	NEG	NEG	NEG	NEG	NEG	POS	POS	POS
>Unassigned Middle Eas																		
Frankfurt_Oder_0490031797 (Libya)	POS	POS	POS	neg	NEG	NEG	POS	NEG	NEG	NEG	NEG	NEG	NEG	NEG	POS	NEG	NEG	POS
Kuwait_192	POS	POS	POS	neg	NEG	NEG	POS	NEG	NEG	NEG	NEG	NEG	NEG	NEG	POS	NEG	NEG	POS
Riyadh-9	POS	POS	POS	neg	NEG	NEG	POS	NEG	NEG	NEG	NEG	NEG	NEG	NEG	POS	NEG	NEG	POS
Russia-24_0407_Moscow	POS	POS	POS	NEG	NEG	NEG	POS	NEG	NEG	NEG	NEG	NEG	NEG	NEG	POS	NEG	NEG	POS

STRAIN / ISOLATE	METHICILLIN RESISTANCE AND SCCmec TYPING																
	D1GU55	B2Y834	B6VQU0	Q3YK51	ydhK	DUF1958	Q4LAG7			QAP8 (SCCmec	Q8CU82	D3JD07	cas1		fusC (Q6GD50)	tirS	yeeA
	D1GU55	B2Y834	B6VQU0	Q3YK51	ydhK (FPR3757)	DUF1958	Q4LAG7 (SCC consensus)	Q4LAG7 (SCCmecV, SO385)	Q4LAG7 (SCCfus 45394F/MSSA476)	C5QAP8 (SCCmec XI)	Q8CU82	D3JD07	cas1 (MSHR1132)	cas1 (M06-0171)		tirS	yeeA
	Putative membrane protein. Subtyping SCCmec III, additional marker for SCCmec VII	Abortive phage resistance protein. Subtyping SCCmec IV, i.e., identification of SCCmec IV A, G, c and SCCmec MRSAZH47	Putative protein. Subtyping SCCmec IV, i.e., identification of SCCmec IVh/j	Putative protein. Subtyping SCCmec IV, i.e., identification of SCCmec IV g	Putative lipoprotein. Present in some composite elements comprising SCCmec and heavy metal resistance genes including the one in FPR3757, GenBank:	Putative protein. Subtyping SCCmec VT. Present, e.g., in PM1, GenBank BAFA but absent, e.g., in Strain 3957, GenBank AOFU	Putative protein located within SCCmec type V/SCCfus elements	Putative protein located within SCCmec type V elements	Putative protein located within SCCfus elements	Putative protein. Identification of SCCmec XI	Putative protein. Present in some SCCmec/fus composite elements such as, e.g., CMFT120, GenBank HF569094.1 and CMFT2, GenBank	Putative protein. Present in some composite SCC elements	CRISPR-associated endonuclease 1. Present in MSHR1132	CRISPR-associated endonuclease 1. Present in M06/0171, GenBank HE980450.1	SCC-associated fusidic acid resistance gene. Present alone in "SCCfus" elements or together with mecA in composite elements. Most	Staphylococcal TIR-protein binding protein. Subtyping SCCfus because it is frequently, but not always, accompanying fusC	Putative DNA methyltransferase. Subtyping SCCmecIV/fus composite elements
**>"Eurasian Clade": TUR1**																	
TUR1 (Turkey)	POS	NEG	NEG	NEG	NEG	NEG	NEG	NEG	NEG	NEG	NEG	NEG	NEG	NEG	NEG	NEG	NEG
TUR9 (Turkey)	POS	NEG	NEG	NEG	NEG	NEG	NEG	NEG	NEG	NEG	NEG	NEG	NEG	NEG	NEG	NEG	NEG
**>"Eurasian Clade" : T013**																	
T0131 (China)	NEG	NEG	NEG	NEG	NEG	NEG	NEG	NEG	NEG	NEG	NEG	NEG	NEG	NEG	NEG	NEG	NEG
MU4 (Turkey)	NEG	NEG	NEG	NEG	NEG	NEG	NEG	NEG	NEG	NEG	NEG	NEG	NEG	NEG	NEG	NEG	NEG
DEU11 (Turkey)	NEG	NEG	NEG	NEG	NEG	NEG	NEG	NEG	NEG	NEG	NEG	NEG	NEG	NEG	NEG	NEG	NEG
DEU16 (Turkey)	NEG	NEG	NEG	NEG	NEG	NEG	NEG	NEG	NEG	NEG	NEG	NEG	NEG	NEG	NEG	NEG	NEG
Romania_Iasi_MRSA-07_54	NEG	NEG	NEG	NEG	NEG	NEG	NEG	NEG	NEG	NEG	NEG	NEG	NEG	NEG	NEG	NEG	NEG
**>"Eurasian Clade": arsC-**																	
Romania_Iasi_MRSA-05_46	NEG	NEG	NEG	NEG	NEG	NEG	NEG	NEG	NEG	NEG	NEG	NEG	NEG	NEG	NEG	NEG	NEG
Romania_Iasi_MRSA-06_47	POS	NEG	NEG	NEG	NEG	NEG	NEG	NEG	NEG	NEG	NEG	NEG	NEG	NEG	NEG	NEG	NEG
**>"Eurasian Clade": 16K/**																	
CN79 (China)	POS	NEG	NEG	NEG	NEG	NEG	NEG	NEG	NEG	NEG	NEG	NEG	NEG	NEG	NEG	NEG	NEG
CUHK_BJ2002 (China)	POS	NEG	NEG	NEG	NEG	NEG	NEG	NEG	NEG	NEG	NEG	NEG	NEG	NEG	NEG	NEG	NEG
CUHK_BJ2007 (China)	POS	NEG	NEG	NEG	NEG	NEG	NEG	NEG	NEG	NEG	NEG	NEG	NEG	NEG	NEG	NEG	NEG
3HK (Czech Republic)	POS	NEG	NEG	NEG	NEG	NEG	NEG	NEG	NEG	NEG	NEG	NEG	NEG	NEG	NEG	NEG	NEG
HU109 (Hungary)	POS	NEG	NEG	NEG	NEG	NEG	NEG	NEG	NEG	NEG	NEG	NEG	NEG	NEG	NEG	NEG	NEG
HUR18 (Hungary)	POS	NEG	NEG	NEG	NEG	NEG	NEG	NEG	NEG	NEG	NEG	NEG	NEG	NEG	NEG	NEG	NEG
H482 (Romania)	POS	NEG	NEG	NEG	NEG	NEG	NEG	NEG	NEG	NEG	NEG	NEG	NEG	NEG	NEG	NEG	NEG
16K (Russia)	POS	NEG	NEG	NEG	NEG	NEG	NEG	NEG	NEG	NEG	NEG	NEG	NEG	NEG	NEG	NEG	NEG
DEU10 (Turkey)	POS	NEG	NEG	NEG	NEG	NEG	NEG	NEG	NEG	NEG	NEG	NEG	NEG	NEG	NEG	NEG	NEG
DEU12 (Turkey)	POS	NEG	NEG	NEG	NEG	NEG	NEG	NEG	NEG	NEG	NEG	NEG	NEG	NEG	NEG	NEG	NEG
DEU14 (Turkey)	POS	NEG	NEG	NEG	NEG	NEG	NEG	NEG	NEG	NEG	NEG	NEG	NEG	NEG	NEG	NEG	NEG
DEU15 (Turkey)	POS	NEG	NEG	NEG	NEG	NEG	NEG	NEG	NEG	NEG	NEG	NEG	NEG	NEG	NEG	NEG	NEG
DEU17 (Turkey)	POS	NEG	NEG	NEG	NEG	NEG	NEG	NEG	NEG	NEG	NEG	NEG	NEG	NEG	NEG	NEG	NEG
DEU19 (Turkey)	POS	NEG	NEG	NEG	NEG	NEG	NEG	NEG	NEG	NEG	NEG	NEG	NEG	NEG	NEG	NEG	NEG
DEU2 (Turkey)	POS	NEG	NEG	NEG	NEG	NEG	NEG	NEG	NEG	NEG	NEG	NEG	NEG	NEG	NEG	NEG	NEG
DEU20 (Turkey)	POS	NEG	NEG	NEG	NEG	NEG	NEG	NEG	NEG	NEG	NEG	NEG	NEG	NEG	NEG	NEG	NEG
DEU23 (Turkey)	POS	NEG	NEG	NEG	NEG	NEG	NEG	NEG	NEG	NEG	NEG	NEG	NEG	NEG	NEG	NEG	NEG
DEU3 (Turkey)	POS	NEG	NEG	NEG	NEG	NEG	NEG	NEG	NEG	NEG	NEG	NEG	NEG	NEG	NEG	NEG	NEG
DEU5 (Turkey)	POS	NEG	NEG	NEG	NEG	NEG	NEG	NEG	NEG	NEG	NEG	NEG	NEG	NEG	NEG	NEG	NEG
DEU6 (Turkey)	POS	NEG	NEG	NEG	NEG	NEG	NEG	NEG	NEG	NEG	NEG	NEG	NEG	NEG	NEG	NEG	NEG
DEU8 (Turkey)	POS	NEG	NEG	NEG	NEG	NEG	NEG	NEG	NEG	NEG	NEG	NEG	NEG	NEG	NEG	NEG	NEG
HU11 (Turkey)	POS	NEG	NEG	NEG	NEG	NEG	NEG	NEG	NEG	NEG	NEG	NEG	NEG	NEG	NEG	NEG	NEG
HU13 (Turkey)	POS	NEG	NEG	NEG	NEG	NEG	NEG	NEG	NEG	NEG	NEG	NEG	NEG	NEG	NEG	NEG	NEG
HU14 (Turkey)	POS	NEG	NEG	NEG	NEG	NEG	NEG	NEG	NEG	NEG	NEG	NEG	NEG	NEG	NEG	NEG	NEG
HU15 (Turkey)	POS	NEG	NEG	NEG	NEG	NEG	NEG	NEG	NEG	NEG	NEG	NEG	NEG	NEG	NEG	NEG	NEG
HU16 (Turkey)	POS	NEG	NEG	NEG	NEG	NEG	NEG	NEG	NEG	NEG	NEG	NEG	NEG	NEG	NEG	NEG	NEG
HU17 (Turkey)	POS	NEG	NEG	NEG	NEG	NEG	NEG	NEG	NEG	NEG	NEG	NEG	NEG	NEG	NEG	NEG	NEG
HU21 (Turkey)	POS	NEG	NEG	NEG	NEG	NEG	NEG	NEG	NEG	NEG	NEG	NEG	NEG	NEG	NEG	NEG	NEG
HU23 (Turkey)	POS	NEG	NEG	NEG	NEG	NEG	NEG	NEG	NEG	NEG	NEG	NEG	NEG	NEG	NEG	NEG	NEG
HU26 (Turkey)	POS	NEG	NEG	NEG	NEG	NEG	NEG	NEG	NEG	NEG	NEG	NEG	NEG	NEG	NEG	NEG	NEG
HU4 (Turkey)	POS	NEG	NEG	NEG	NEG	NEG	NEG	NEG	NEG	NEG	NEG	NEG	NEG	NEG	NEG	NEG	NEG
HU5 (Turkey)	POS	NEG	NEG	NEG	NEG	NEG	NEG	NEG	NEG	NEG	NEG	NEG	NEG	NEG	NEG	NEG	NEG
HU6 (Turkey)	POS	NEG	NEG	NEG	NEG	NEG	NEG	NEG	NEG	NEG	NEG	NEG	NEG	NEG	NEG	NEG	NEG
HU7 (Turkey)	POS	NEG	NEG	NEG	NEG	NEG	NEG	NEG	NEG	NEG	NEG	NEG	NEG	NEG	NEG	NEG	NEG
HU8 (Turkey)	POS	NEG	NEG	NEG	NEG	NEG	NEG	NEG	NEG	NEG	NEG	NEG	NEG	NEG	NEG	NEG	NEG
HU9 (Turkey)	POS	NEG	NEG	NEG	NEG	NEG	NEG	NEG	NEG	NEG	NEG	NEG	NEG	NEG	NEG	NEG	NEG
IU1 (Turkey)	POS	NEG	NEG	NEG	NEG	NEG	NEG	NEG	NEG	NEG	NEG	NEG	NEG	NEG	NEG	NEG	NEG
IU10 (Turkey)	POS	NEG	NEG	NEG	NEG	NEG	NEG	NEG	NEG	NEG	NEG	NEG	NEG	NEG	NEG	NEG	NEG
IU11 (Turkey)	POS	NEG	NEG	NEG	NEG	NEG	NEG	NEG	NEG	NEG	NEG	NEG	NEG	NEG	NEG	NEG	NEG
IU12 (Turkey)	POS	NEG	NEG	NEG	NEG	NEG	NEG	NEG	NEG	NEG	NEG	NEG	NEG	NEG	NEG	NEG	NEG
IU13 (Turkey)	POS	NEG	NEG	NEG	NEG	NEG	NEG	NEG	NEG	NEG	NEG	NEG	NEG	NEG	NEG	NEG	NEG
IU15 (Turkey)	POS	NEG	NEG	NEG	NEG	NEG	NEG	NEG	NEG	NEG	NEG	NEG	NEG	NEG	NEG	NEG	NEG
IU18 (Turkey)	POS	NEG	NEG	NEG	NEG	NEG	NEG	NEG	NEG	NEG	NEG	NEG	NEG	NEG	NEG	NEG	NEG
IU19 (Turkey)	POS	NEG	NEG	NEG	NEG	NEG	NEG	NEG	NEG	NEG	NEG	NEG	NEG	NEG	NEG	NEG	NEG
IU2 (Turkey)	POS	NEG	NEG	NEG	NEG	NEG	NEG	NEG	NEG	NEG	NEG	NEG	NEG	NEG	NEG	NEG	NEG
IU4 (Turkey)	POS	NEG	NEG	NEG	NEG	NEG	NEG	NEG	NEG	NEG	NEG	NEG	NEG	NEG	NEG	NEG	NEG
IU6 (Turkey)	POS	NEG	NEG	NEG	NEG	NEG	NEG	NEG	NEG	NEG	NEG	NEG	NEG	NEG	NEG	NEG	NEG
IU7 (Turkey)	POS	NEG	NEG	NEG	NEG	NEG	NEG	NEG	NEG	NEG	NEG	NEG	NEG	NEG	NEG	NEG	NEG
IU9 (Turkey)	POS	NEG	NEG	NEG	NEG	NEG	NEG	NEG	NEG	NEG	NEG	NEG	NEG	NEG	NEG	NEG	NEG
MU1 (Turkey)	POS	NEG	NEG	NEG	NEG	NEG	NEG	NEG	NEG	NEG	NEG	NEG	NEG	NEG	NEG	NEG	NEG
MU10 (Turkey)	POS	NEG	NEG	NEG	NEG	NEG	NEG	NEG	NEG	NEG	NEG	NEG	NEG	NEG	NEG	NEG	NEG
MU20 (Turkey)	POS	NEG	NEG	NEG	NEG	NEG	NEG	NEG	NEG	NEG	NEG	NEG	NEG	NEG	NEG	NEG	NEG
Mu3 (Turkey)	POS	NEG	NEG	NEG	NEG	NEG	NEG	NEG	NEG	NEG	NEG	NEG	NEG	NEG	NEG	NEG	NEG
MU5 (Turkey)	POS	NEG	NEG	NEG	NEG	NEG	NEG	NEG	NEG	NEG	NEG	NEG	NEG	NEG	NEG	NEG	NEG
MU6 (Turkey)	POS	NEG	NEG	NEG	NEG	NEG	NEG	NEG	NEG	NEG	NEG	NEG	NEG	NEG	NEG	NEG	NEG
MU7 (Turkey)	POS	NEG	NEG	NEG	NEG	NEG	NEG	NEG	NEG	NEG	NEG	NEG	NEG	NEG	NEG	NEG	NEG
TUR27 (Turkey)	POS	NEG	NEG	NEG	NEG	NEG	NEG	NEG	NEG	NEG	NEG	NEG	NEG	NEG	NEG	NEG	NEG
NCTR 32S	POS	NEG	NEG	NEG	NEG	NEG	NEG	NEG	NEG	NEG	NEG	NEG	NEG	NEG	NEG	NEG	NEG
SA02_A4 = Russia-06_0085_Moscow	POS	NEG	NEG	NEG	NEG	NEG	NEG	NEG	NEG	NEG	NEG	NEG	NEG	NEG	NEG	NEG	NEG
	POS	NEG	NEG	NEG	NEG	NEG	NEG	NEG	NEG	NEG	NEG	NEG	NEG	NEG	NEG	NEG	NEG
Dresden_08V35987 (Turkey)	POS	NEG	NEG	NEG	NEG	NEG	NEG	NEG	NEG	NEG	NEG	NEG	NEG	NEG	NEG	NEG	NEG
Dresden_17ANR577152	POS	NEG	NEG	NEG	NEG	NEG	NEG	NEG	NEG	NEG	NEG	NEG	NEG	NEG	NEG	NEG	NEG
Dresden_17ANR580374 (Makedonia)	POS	NEG	NEG	NEG	NEG	NEG	NEG	NEG	NEG	NEG	NEG	NEG	NEG	NEG	NEG	NEG	NEG
Hong Kong_69-II	POS	NEG	NEG	NEG	NEG	NEG	NEG	NEG	NEG	NEG	NEG	NEG	NEG	NEG	NEG	NEG	NEG
Hong Kong_93	POS	NEG	NEG	NEG	NEG	NEG	NEG	NEG	NEG	NEG	NEG	NEG	NEG	NEG	NEG	NEG	NEG
Rawalpindi_Kidney Center_03_SK1	POS	NEG	NEG	NEG	NEG	NEG	NEG	NEG	NEG	NEG	NEG	NEG	NEG	NEG	NEG	NEG	NEG
Rawalpindi_Kidney Center_08	POS	NEG	NEG	NEG	NEG	NEG	NEG	NEG	NEG	NEG	NEG	NEG	NEG	NEG	NEG	NEG	NEG
Rawalpindi_Kidney Center_10	POS	NEG	NEG	NEG	NEG	NEG	NEG	NEG	NEG	NEG	NEG	NEG	NEG	NEG	NEG	NEG	NEG
Rawalpindi_Kidney Center_19	POS	NEG	NEG	NEG	NEG	NEG	NEG	NEG	NEG	NEG	NEG	NEG	NEG	NEG	NEG	NEG	NEG
Rawalpindi_Kidney Center_50_SK1	POS	NEG	NEG	NEG	NEG	NEG	NEG	NEG	NEG	NEG	NEG	NEG	neg	NEG	NEG	NEG	NEG
Romania_Iasi_BC-49_430	POS	NEG	NEG	NEG	NEG	NEG	NEG	NEG	NEG	NEG	NEG	NEG	NEG	NEG	NEG	NEG	NEG
Romania_Iasi_MRSA_284	POS	NEG	NEG	NEG	NEG	NEG	NEG	NEG	NEG	NEG	NEG	NEG	NEG	NEG	NEG	NEG	NEG
Romania_Iasi_MRSA-32_318	POS	NEG	NEG	NEG	NEG	NEG	NEG	NEG	NEG	NEG	NEG	NEG	NEG	NEG	NEG	NEG	NEG
Romania_Iasi_MRSA-54_430	POS	NEG	NEG	NEG	NEG	NEG	NEG	NEG	NEG	NEG	NEG	NEG	NEG	NEG	NEG	NEG	NEG
Romania_Iasi_SSTI-01_2	POS	NEG	NEG	NEG	NEG	NEG	NEG	NEG	NEG	NEG	NEG	NEG	NEG	NEG	NEG	NEG	NEG
Romania_Iasi_SSTI-10_101	POS	NEG	NEG	NEG	NEG	NEG	NEG	NEG	NEG	NEG	NEG	NEG	NEG	NEG	NEG	NEG	NEG
Romania_Iasi_SSTI-11_106	POS	NEG	NEG	NEG	NEG	NEG	NEG	NEG	NEG	NEG	NEG	NEG	NEG	NEG	NEG	NEG	NEG
Russia-01_0001_SaintPetersburg	POS	NEG	NEG	NEG	NEG	NEG	NEG	NEG	NEG	NEG	NEG	NEG	NEG	NEG	NEG	NEG	NEG
Russia-02_0004_SaintPetersburg	POS	NEG	NEG	NEG	NEG	NEG	NEG	NEG	NEG	NEG	NEG	NEG	NEG	NEG	NEG	NEG	NEG
Russia-03_0057_SaintPetersburg	POS	NEG	NEG	NEG	NEG	NEG	NEG	NEG	NEG	NEG	NEG	NEG	NEG	NEG	NEG	NEG	NEG
Russia-04_0076_Moscow	POS	NEG	NEG	NEG	NEG	NEG	NEG	NEG	NEG	NEG	NEG	NEG	NEG	NEG	NEG	NEG	NEG
Russia-05_0078_Moscow	POS	NEG	NEG	NEG	NEG	NEG	NEG	NEG	NEG	NEG	NEG	NEG	NEG	NEG	NEG	NEG	NEG
Russia-08_0135_Moscow	POS	NEG	NEG	NEG	NEG	NEG	NEG	NEG	NEG	NEG	NEG	NEG	NEG	NEG	NEG	NEG	NEG
Russia-09_0150_Kurgan	POS	NEG	NEG	NEG	NEG	NEG	NEG	NEG	NEG	NEG	NEG	NEG	NEG	NEG	NEG	NEG	NEG
Russia-10_0162_Kurgan	POS	NEG	NEG	NEG	NEG	NEG	NEG	NEG	NEG	NEG	NEG	NEG	NEG	NEG	NEG	NEG	NEG
Russia-14_0184_Moscow	POS	NEG	NEG	NEG	NEG	NEG	NEG	NEG	NEG	NEG	NEG	NEG	NEG	NEG	NEG	NEG	NEG
Russia-15_0232_SaintPetersburg	POS	NEG	NEG	NEG	NEG	NEG	NEG	NEG	NEG	NEG	NEG	NEG	NEG	NEG	NEG	NEG	NEG
Russia-22_0391_Chelyabinsk	POS	NEG	NEG	NEG	NEG	NEG	NEG	NEG	NEG	NEG	NEG	NEG	NEG	NEG	NEG	NEG	NEG
Russia-23_0392_Chelyabinsk	POS	NEG	NEG	NEG	NEG	NEG	NEG	NEG	NEG	NEG	NEG	NEG	NEG	NEG	NEG	NEG	NEG
**>"Eurasian Clade" : IU17**																	
IU17 (Turkey)	POS	NEG	NEG	NEG	NEG	NEG	NEG	NEG	NEG	NEG	NEG	NEG	NEG	NEG	NEG	NEG	NEG
IU20 (Turkey)	POS	NEG	NEG	NEG	NEG	NEG	NEG	NEG	NEG	NEG	NEG	NEG	NEG	NEG	NEG	NEG	NEG

STRAIN / ISOLATE	METHICILLIN RESISTANCE AND SCCmec TYPING																
	D1GU55	B2Y834	B6VQU0	Q3YK51	ydhK	DUF1958	Q4LAG7			QAP8 (SCCmec	Q8CU82	D3JD07	cas1		fusC (Q6GD50)	tirS	yeeA
	D1GU55	B2Y834	B6VQU0	Q3YK51	ydhK (FPR3757)	DUF1958	Q4LAG7 (SCC consensus)	Q4LAG7 (SCCmecV, SO385)	Q4LAG7 (SCCfus 45394F/MSSA476)	C5QAP8 (SCCmec XI)	Q8CU82	D3JD07	cas1 (MSHR1132)	cas1 (M06-0171)		tirS	yeeA
	Putative membrane protein. Subtyping SCCmec III, additional marker for SCCmec VII	Abortive phage resistance protein. Subtyping SCCmec IV, i.e., identification of SCCmec IV A, G, c and SCCmec MRSAZH47	Putative protein. Subtyping SCCmec IV, i.e., identification of SCCmec IVh/j	Putative protein. Subtyping SCCmec IV, i.e., identification of SCCmec IV g	Putative lipoprotein. Present in some composite elements comprising SCCmec and heavy metal resistance genes including the one in FPR3757, GenBank:	Putative protein. Subtyping SCCmec VT. Present, e.g., in PM1, GenBank BAFA but absent, e.g., in Strain 3957, GenBank AOFU	Putative protein located within SCCmec type V/SCCfus elements	Putative protein located within SCCmec type V elements	Putative protein located within SCCfus elements	Putative protein. Identification of SCCmec XI	Putative protein. Present in some SCCmec/fus composite elements such as, e.g., CMFT120, GenBank HF569094.1 and CMFT2, GenBank	Putative protein. Present in some composite SCC elements	CRISPR-associated endonuclease 1. Present in MSHR1132	CRISPR-associated endonuclease 1. Present in M06/0171, GenBank HE980450.1	SCC-associated fusidic acid resistance gene. Present alone in "SCCfus" elements or together with mecA in composite elements. Most	Staphylococcal TIR-protein binding protein. Subtyping SCCfus because it is frequently, but not always, accompanying fusC	Putative DNA methyltransferase. Subtyping SCCmecIV/fus composite elements
>"Eurasian Clade": Spora																	
Dresden_16ANR581769	POS	NEG	NEG	NEG	NEG	NEG	POS	POS	NEG	NEG	NEG	NEG	NEG	NEG	NEG	NEG	NEG
Dresden_17ANR578769	POS	NEG	NEG	NEG	NEG	NEG	POS	POS	NEG	NEG	NEG	NEG	NEG	NEG	NEG	NEG	NEG
>"Eurasian Clade": Kuwa																	
Kuwait_306	POS	NEG	NEG	NEG	NEG	NEG	NEG	NEG	NEG	NEG	NEG	NEG	NEG	NEG	NEG	NEG	NEG
Kuwait_301	POS	NEG	NEG	NEG	NEG	NEG	NEG	NEG	NEG	NEG	NEG	NEG	NEG	NEG	NEG	NEG	NEG
>"European Clade": "Gre																	
GRE18 (Greece)	POS	NEG	NEG	NEG	NEG	NEG	NEG	NEG	NEG	NEG	NEG	NEG	NEG	NEG	NEG	NEG	NEG
GRE317 (Greece)	POS	NEG	NEG	NEG	NEG	NEG	NEG	NEG	NEG	NEG	NEG	NEG	NEG	NEG	NEG	NEG	NEG
GRE4 (Greece)	POS	NEG	NEG	NEG	NEG	NEG	NEG	NEG	NEG	NEG	NEG	NEG	NEG	NEG	NEG	NEG	NEG
R35 (USA)	POS	NEG	NEG	NEG	NEG	NEG	NEG	NEG	NEG	NEG	NEG	NEG	NEG	NEG	NEG	NEG	NEG
Casablanca_HT20060548	POS	NEG	NEG	NEG	NEG	NEG	NEG	NEG	NEG	NEG	NEG	NEG	NEG	NEG	NEG	NEG	NEG
Casablanca_HT20060550	POS	NEG	NEG	NEG	NEG	NEG	NEG	NEG	NEG	NEG	NEG	NEG	neg	NEG	NEG	NEG	NEG
Dresden_01V34572 (Greece)	POS	NEG	NEG	NEG	NEG	NEG	NEG	NEG	NEG	NEG	NEG	NEG	NEG	NEG	NEG	NEG	NEG
Dresden_01V36469	POS	NEG	NEG	NEG	NEG	NEG	NEG	NEG	NEG	NEG	NEG	NEG	NEG	NEG	NEG	NEG	NEG
Dresden_01V39123	POS	NEG	NEG	NEG	NEG	NEG	NEG	NEG	NEG	NEG	NEG	NEG	NEG	NEG	NEG	NEG	NEG
Dresden_01V39143	POS	NEG	NEG	NEG	NEG	NEG	NEG	NEG	NEG	NEG	NEG	NEG	NEG	NEG	NEG	NEG	NEG
Greece 1_3680_Harmony	POS	NEG	NEG	NEG	NEG	NEG	NEG	NEG	NEG	NEG	NEG	NEG	NEG	NEG	NEG	NEG	NEG
>"European Clade": UK-(																	
ANS46 (Australia)	POS	NEG	NEG	NEG	NEG	NEG	NEG	NEG	NEG	NEG	NEG	NEG	NEG	NEG	NEG	NEG	NEG
LHH1 (USA)	POS	NEG	NEG	NEG	NEG	NEG	NEG	NEG	NEG	NEG	NEG	NEG	NEG	NEG	NEG	NEG	NEG
Dublin-DSH_AR01_0-1085	POS	NEG	NEG	NEG	NEG	NEG	NEG	NEG	NEG	NEG	NEG	NEG	NEG	NEG	NEG	NEG	NEG
Dublin-DSH_AR01_0-1118	POS	NEG	NEG	NEG	NEG	NEG	NEG	NEG	NEG	NEG	NEG	NEG	NEG	NEG	NEG	NEG	NEG
Dublin-DSH_AR15_0098	POS	NEG	NEG	NEG	NEG	NEG	NEG	NEG	NEG	NEG	NEG	NEG	NEG	NEG	NEG	NEG	NEG
Dublin-DSH_AR15_0104	POS	NEG	NEG	NEG	NEG	NEG	NEG	NEG	NEG	NEG	NEG	NEG	NEG	NEG	NEG	NEG	NEG
UK_NCTC11939_UK-1_Harmony	POS	NEG	NEG	NEG	NEG	NEG	NEG	NEG	NEG	NEG	NEG	NEG	NEG	NEG	NEG	NEG	NEG
>"European Clade": BK2																	
BK2421 (USA)	POS	NEG	NEG	NEG	NEG	NEG	NEG	NEG	NEG	NEG	NEG	NEG	NEG	NEG	NEG	NEG	NEG
>"Aussi/NZ Clade", JKD6																	
JKD6009 (Australia)	NEG	NEG	NEG	NEG	NEG	NEG	NEG	NEG	NEG	NEG	NEG	NEG	NEG	NEG	NEG	NEG	NEG
JKD6008 (New Zealand)	NEG	NEG	NEG	NEG	NEG	NEG	NEG	NEG	NEG	NEG	NEG	NEG	NEG	NEG	NEG	NEG	NEG
Perth_2015-S20209	NEG	NEG	NEG	NEG	NEG	NEG	NEG	NEG	NEG	NEG	NEG	NEG	NEG	NEG	NEG	NEG	NEG
Perth_2015-S20305	NEG	NEG	NEG	NEG	NEG	NEG	NEG	NEG	NEG	NEG	NEG	NEG	NEG	NEG	NEG	NEG	NEG
Perth_2015-S20603	NEG	NEG	NEG	NEG	NEG	NEG	NEG	NEG	NEG	NEG	NEG	NEG	NEG	NEG	NEG	NEG	NEG
Perth_2015-S20607	NEG	NEG	NEG	NEG	NEG	NEG	NEG	NEG	NEG	NEG	NEG	NEG	NEG	NEG	NEG	NEG	NEG
Perth_2015-S20608	NEG	NEG	NEG	NEG	NEG	NEG	NEG	NEG	NEG	NEG	NEG	NEG	NEG	NEG	NEG	NEG	NEG
Perth_2015-S20610	NEG	NEG	NEG	NEG	NEG	NEG	NEG	NEG	NEG	NEG	NEG	NEG	NEG	NEG	NEG	NEG	NEG
Perth_2015-S20613	NEG	NEG	NEG	NEG	NEG	NEG	NEG	NEG	NEG	NEG	NEG	NEG	NEG	NEG	NEG	NEG	NEG
Perth_2015-S20617	NEG	NEG	NEG	NEG	NEG	NEG	NEG	NEG	NEG	NEG	NEG	NEG	NEG	NEG	NEG	NEG	NEG
Perth_2015-S20622	NEG	NEG	NEG	NEG	NEG	NEG	NEG	NEG	NEG	NEG	NEG	NEG	NEG	NEG	NEG	NEG	NEG
Perth_2015-S23619	NEG	NEG	NEG	NEG	NEG	NEG	NEG	NEG	NEG	NEG	NEG	NEG	NEG	NEG	NEG	NEG	NEG
Perth_2015-S41210	NEG	NEG	NEG	NEG	NEG	NEG	NEG	NEG	NEG	NEG	NEG	NEG	NEG	NEG	NEG	NEG	NEG
Perth_2015-S51001	NEG	NEG	NEG	NEG	NEG	NEG	NEG	NEG	NEG	NEG	NEG	NEG	NEG	NEG	NEG	NEG	NEG
Perth_2015-S51003	NEG	NEG	NEG	NEG	NEG	NEG	NEG	NEG	NEG	NEG	NEG	NEG	NEG	NEG	NEG	NEG	NEG
Perth_2015-S51006	NEG	NEG	NEG	NEG	NEG	NEG	NEG	NEG	NEG	NEG	NEG	NEG	NEG	NEG	NEG	NEG	NEG
Perth_2015-S51007	NEG	NEG	NEG	NEG	NEG	NEG	NEG	NEG	NEG	NEG	NEG	NEG	NEG	NEG	NEG	NEG	NEG
Perth_2015-S51008	NEG	NEG	NEG	NEG	NEG	NEG	NEG	NEG	NEG	NEG	NEG	NEG	NEG	NEG	NEG	NEG	NEG
Perth_2015-S51014	NEG	NEG	NEG	NEG	NEG	NEG	NEG	NEG	NEG	NEG	NEG	NEG	NEG	NEG	NEG	NEG	NEG
Perth_2015-S51408	NEG	NEG	NEG	NEG	NEG	NEG	NEG	NEG	NEG	NEG	NEG	NEG	NEG	NEG	NEG	NEG	NEG
Perth_2016-S20614	NEG	NEG	NEG	NEG	NEG	NEG	NEG	NEG	NEG	NEG	NEG	NEG	NEG	NEG	NEG	NEG	NEG
Perth_2016-S23614	NEG	NEG	NEG	NEG	NEG	NEG	NEG	NEG	NEG	NEG	NEG	NEG	NEG	NEG	NEG	NEG	NEG
Perth_2016-S42009	NEG	NEG	NEG	NEG	NEG	NEG	NEG	NEG	NEG	NEG	NEG	NEG	NEG	NEG	NEG	NEG	NEG
Perth_2016-S51001	NEG	NEG	NEG	NEG	NEG	NEG	NEG	NEG	NEG	NEG	NEG	NEG	NEG	NEG	NEG	NEG	NEG
Perth_2016-S51403	NEG	NEG	NEG	NEG	NEG	NEG	NEG	NEG	NEG	NEG	NEG	NEG	NEG	NEG	NEG	NEG	NEG
Perth_07 RPA 20	NEG	NEG	NEG	NEG	NEG	NEG	NEG	NEG	NEG	NEG	NEG	NEG	NEG	NEG	NEG	NEG	NEG
Perth_08-19088	NEG	NEG	NEG	NEG	NEG	NEG	NEG	NEG	NEG	NEG	NEG	NEG	NEG	NEG	NEG	NEG	NEG
Perth_01-15351	NEG	NEG	NEG	NEG	NEG	NEG	NEG	NEG	NEG	NEG	NEG	NEG	NEG	NEG	NEG	NEG	NEG
Perth_01-15357	NEG	NEG	NEG	NEG	NEG	NEG	NEG	NEG	NEG	NEG	NEG	NEG	NEG	NEG	NEG	NEG	NEG
Perth_01-15419	NEG	NEG	NEG	NEG	NEG	NEG	NEG	NEG	NEG	NEG	NEG	NEG	NEG	NEG	NEG	NEG	NEG
Perth_01-16309	NEG	NEG	NEG	NEG	NEG	NEG	NEG	NEG	NEG	NEG	NEG	NEG	NEG	NEG	NEG	NEG	NEG
Perth_03 SNP 95	NEG	NEG	NEG	NEG	NEG	NEG	NEG	NEG	NEG	NEG	NEG	NEG	NEG	NEG	NEG	NEG	NEG
Perth_2003_AH_47	NEG	NEG	NEG	NEG	NEG	NEG	NEG	NEG	NEG	NEG	NEG	NEG	NEG	NEG	NEG	NEG	NEG
>"Australian/NZ Clade":																	
Perth_2015-S20623	NEG	NEG	NEG	NEG	NEG	NEG	NEG	NEG	NEG	NEG	NEG	NEG	NEG	NEG	NEG	NEG	NEG
Perth_2015-S20826	NEG	NEG	NEG	NEG	NEG	NEG	NEG	NEG	NEG	NEG	NEG	NEG	NEG	NEG	NEG	NEG	NEG
Perth_05 NH 56	NEG	NEG	NEG	NEG	NEG	NEG	NEG	NEG	NEG	NEG	NEG	NEG	NEG	NEG	NEG	NEG	NEG
>CC5-MRSA-III LA MRSA																	
Delbrück 38 isolate from domestic turkey	NEG	NEG	NEG	NEG	NEG	NEG	NEG	NEG	NEG	NEG	NEG	NEG	NEG	NEG	NEG	NEG	NEG
> Staph. pseudintermed																	
KM1381	NEG	NEG	NEG	NEG	NEG	NEG	NEG	NEG	NEG	NEG	NEG	NEG	NEG	NEG	NEG	NEG	NEG
>"South-East Asian Clad																	
M92 (Canada)	NEG	NEG	NEG	NEG	NEG	NEG	NEG	NEG	NEG	NEG	NEG	NEG	NEG	NEG	NEG	NEG	NEG
CHI59 (China)	NEG	NEG	NEG	NEG	NEG	NEG	NEG	NEG	NEG	NEG	NEG	NEG	NEG	NEG	NEG	NEG	NEG
CHI61 (China)	NEG	NEG	NEG	NEG	NEG	NEG	NEG	NEG	NEG	NEG	NEG	NEG	NEG	NEG	NEG	NEG	NEG
CUHK_HK1997 (China)	NEG	NEG	NEG	NEG	NEG	NEG	NEG	NEG	NEG	NEG	NEG	NEG	NEG	NEG	NEG	NEG	NEG
M996 (China)	NEG	NEG	NEG	NEG	NEG	NEG	NEG	NEG	NEG	NEG	NEG	NEG	NEG	NEG	NEG	NEG	NEG
H211 (Denmark)	NEG	NEG	NEG	NEG	NEG	NEG	NEG	NEG	NEG	NEG	NEG	NEG	NEG	NEG	NEG	NEG	NEG
H216 (Denmark)	NEG	NEG	NEG	NEG	NEG	NEG	NEG	NEG	NEG	NEG	NEG	NEG	NEG	NEG	NEG	NEG	NEG
D71 (Germany)	NEG	NEG	NEG	NEG	NEG	NEG	NEG	NEG	NEG	NEG	NEG	NEG	NEG	NEG	NEG	NEG	NEG
NMR05 (India)	NEG	NEG	NEG	NEG	NEG	NEG	NEG	NEG	NEG	NEG	NEG	NEG	NEG	NEG	NEG	NEG	NEG
MAL11 (Malaysia)	NEG	NEG	NEG	NEG	NEG	NEG	NEG	NEG	NEG	NEG	NEG	NEG	NEG	NEG	NEG	NEG	NEG
MAL9 (Malaysia)	NEG	NEG	NEG	NEG	NEG	NEG	NEG	NEG	NEG	NEG	NEG	NEG	NEG	NEG	NEG	NEG	NEG
Na21 (Sri Lanka)	NEG	NEG	NEG	NEG	NEG	NEG	NEG	NEG	NEG	NEG	NEG	NEG	NEG	NEG	NEG	NEG	NEG
M592 (Syria)	NEG	NEG	NEG	NEG	NEG	NEG	NEG	NEG	NEG	NEG	NEG	NEG	NEG	NEG	NEG	NEG	NEG
H202 (Thailand)	NEG	NEG	NEG	NEG	NEG	NEG	NEG	NEG	NEG	NEG	NEG	NEG	NEG	NEG	NEG	NEG	NEG
S102 (Thailand)	NEG	NEG	NEG	NEG	NEG	NEG	NEG	NEG	NEG	NEG	NEG	NEG	NEG	NEG	NEG	NEG	NEG
S130 (Thailand)	NEG	NEG	NEG	NEG	NEG	NEG	NEG	NEG	NEG	NEG	NEG	NEG	NEG	NEG	NEG	NEG	NEG
S40 (Thailand)	NEG	NEG	NEG	NEG	NEG	NEG	NEG	NEG	NEG	NEG	NEG	NEG	NEG	NEG	NEG	NEG	NEG
S71 (Thailand)	NEG	NEG	NEG	NEG	NEG	NEG	NEG	NEG	NEG	NEG	NEG	NEG	NEG	NEG	NEG	NEG	NEG
S87 (Thailand)	NEG	NEG	NEG	NEG	NEG	NEG	NEG	NEG	NEG	NEG	NEG	NEG	NEG	NEG	NEG	NEG	NEG
S93 (Thailand)	NEG	NEG	NEG	NEG	NEG	NEG	NEG	NEG	NEG	NEG	NEG	NEG	NEG	NEG	NEG	NEG	NEG
US_002 (Thailand)	NEG	NEG	NEG	NEG	NEG	NEG	NEG	NEG	NEG	NEG	NEG	NEG	NEG	NEG	NEG	NEG	NEG
US_017 (Thailand)	NEG	NEG	NEG	NEG	NEG	NEG	NEG	NEG	NEG	NEG	NEG	NEG	NEG	NEG	NEG	NEG	NEG
WAMC6102 (Thailand)	NEG	NEG	NEG	NEG	NEG	NEG	NEG	NEG	NEG	NEG	NEG	NEG	NEG	NEG	NEG	NEG	NEG
NCTC_13626_TW20 (UK)	NEG	NEG	NEG	NEG	NEG	NEG	NEG	NEG	NEG	NEG	NEG	NEG	NEG	NEG	NEG	NEG	NEG
BSAC3021 (UK)	NEG	NEG	NEG	NEG	NEG	NEG	NEG	NEG	NEG	NEG	NEG	NEG	NEG	NEG	NEG	NEG	NEG
BSAC3046 (UK)	NEG	NEG	NEG	NEG	NEG	NEG	NEG	NEG	NEG	NEG	NEG	NEG	NEG	NEG	NEG	NEG	NEG
1193_SAUR (USA)	NEG	NEG	NEG	NEG	NEG	NEG	NEG	NEG	NEG	NEG	NEG	NEG	NEG	NEG	NEG	NEG	NEG
1194_SAUR (USA)	NEG	NEG	NEG	NEG	NEG	NEG	NEG	NEG	NEG	NEG	NEG	NEG	NEG	NEG	NEG	NEG	NEG

STRAIN / ISOLATE	METHICILLIN RESISTANCE AND SCCmec TYPING																
	D1GU55	B2Y834	B6VQU0	Q3YK51	ydhK	DUF1958	Q4LAG7			QAP8 (SCCmec	Q8CU82	D3JD07	cas1		fusC (Q6GD50)	tirS	yeeA
	D1GU55	B2Y834	B6VQU0	Q3YK51	ydhK (FPR3757)	DUF1958	Q4LAG7 (SCC consensus)	Q4LAG7 (SCCmecV, SO385)	Q4LAG7 (SCCfus 45394F/MSSA476)	C5QAP8 (SCCmec XI)	Q8CU82	D3JD07	cas1 (MSHR1132)	cas1 (M06-0171)		tirS	yeeA
	Putative membrane protein. Subtyping SCCmec III, additional marker for SCCmec VII	Abortive phage resistance protein. Subtyping SCCmec IV, i.e., identification of SCCmec IV A, G, c and SCCmec MRSAZH47	Putative protein. Subtyping SCCmec IV, i.e., identification of SCCmec IVh/j	Putative protein. Subtyping SCCmec IV, i.e., identification of SCCmec IV g	Putative lipoprotein. Present in some composite elements comprising SCCmec and heavy metal resistance genes including the one in FPR3757, GenBank:	Putative protein. Subtyping SCCmec VT. Present, e.g., in PM1, GenBank BAFA but absent, e.g., in Strain 3957, GenBank AOFU	Putative protein located within SCCmec type V/SCCfus elements	Putative protein located within SCCmec type V elements	Putative protein located within SCCfus elements	Putative protein. Identification of SCCmec XI	Putative protein. Present in some SCCmec/fus composite elements such as, e.g., CMFT120, GenBank HF569094.1 and CMFT2, GenBank	Putative protein. Present in some composite SCC elements	CRISPR-associated endonuclease 1. Present in MSHR1132	CRISPR-associated endonuclease 1. Present in M06/0171, GenBank HE980450.1	SCC-associated fusidic acid resistance gene. Present alone in "SCCfus" elements or together with mecA in composite elements. Most	Staphylococcal TIR-protein binding protein. Subtyping SCCfus because it is frequently, but not always, accompanying fusC	Putative DNA methyltransferase. Subtyping SCCmecIV/fus composite elements
>"South-East Asian Clade": TW20 (Irish AR44), cc																	
1195_SAUR (USA)	NEG	NEG	NEG	NEG	NEG	NEG	NEG	NEG	NEG	NEG	NEG	NEG	NEG	NEG	NEG	NEG	NEG
FVRH6002 (USA)	NEG	NEG	NEG	NEG	NEG	NEG	NEG	NEG	NEG	NEG	NEG	NEG	NEG	NEG	NEG	NEG	NEG
KINW6048 (USA)	NEG	NEG	NEG	NEG	NEG	NEG	NEG	NEG	NEG	NEG	NEG	NEG	NEG	NEG	NEG	NEG	NEG
LAMC0011 (USA)	NEG	NEG	NEG	NEG	NEG	NEG	NEG	NEG	NEG	NEG	NEG	NEG	NEG	NEG	NEG	NEG	NEG
SJOS6053 (USA)	NEG	NEG	NEG	NEG	NEG	NEG	NEG	NEG	NEG	NEG	NEG	NEG	NEG	NEG	NEG	NEG	NEG
SJOS6072 (USA)	NEG	NEG	NEG	NEG	NEG	NEG	NEG	NEG	NEG	NEG	NEG	NEG	NEG	NEG	NEG	NEG	NEG
UCIM6042 (USA)	NEG	NEG	NEG	NEG	NEG	NEG	NEG	NEG	NEG	NEG	NEG	NEG	NEG	NEG	NEG	NEG	NEG
Perth_2015-S31902	NEG	NEG	NEG	NEG	NEG	NEG	NEG	NEG	NEG	NEG	NEG	NEG	NEG	NEG	NEG	NEG	NEG
Perth_2015-S31904	NEG	NEG	NEG	NEG	NEG	NEG	NEG	NEG	NEG	NEG	NEG	NEG	NEG	NEG	NEG	NEG	NEG
Perth_2015-S31913	NEG	NEG	NEG	NEG	NEG	NEG	NEG	NEG	NEG	NEG	NEG	NEG	NEG	NEG	NEG	NEG	NEG
Perth_2015-S31918	NEG	NEG	NEG	NEG	NEG	NEG	NEG	NEG	NEG	NEG	NEG	NEG	NEG	NEG	NEG	NEG	NEG
Dublin-DSH_AR44_E1183	AMB	NEG	NEG	NEG	NEG	NEG	NEG	NEG	NEG	NEG	NEG	NEG	NEG	NEG	NEG	NEG	NEG
Dublin-DSH_Unfamiliar-3_E1520	NEG	NEG	NEG	NEG	NEG	NEG	NEG	NEG	NEG	NEG	NEG	NEG	NEG	NEG	NEG	NEG	NEG
Hong Kong_042	NEG	NEG	NEG	NEG	NEG	NEG	NEG	NEG	NEG	NEG	NEG	NEG	NEG	NEG	NEG	NEG	NEG
Hong Kong_101	NEG	NEG	NEG	NEG	NEG	NEG	NEG	NEG	NEG	NEG	NEG	NEG	NEG	NEG	NEG	NEG	NEG
Hong Kong_111	NEG	NEG	NEG	NEG	NEG	NEG	NEG	NEG	NEG	NEG	NEG	NEG	NEG	NEG	NEG	NEG	NEG
Hong Kong_113	NEG	NEG	NEG	NEG	NEG	NEG	NEG	NEG	NEG	NEG	NEG	NEG	NEG	NEG	NEG	NEG	NEG
Hong Kong_115	NEG	NEG	NEG	NEG	NEG	NEG	NEG	NEG	NEG	NEG	NEG	NEG	NEG	NEG	NEG	NEG	NEG
Hong Kong_118	NEG	NEG	NEG	NEG	NEG	NEG	NEG	NEG	NEG	NEG	NEG	NEG	NEG	NEG	NEG	NEG	NEG
Hong Kong_125	NEG	NEG	NEG	NEG	NEG	NEG	NEG	NEG	NEG	NEG	NEG	NEG	NEG	NEG	NEG	NEG	NEG
Hong Kong_129	NEG	NEG	NEG	NEG	NEG	NEG	NEG	NEG	NEG	NEG	NEG	NEG	NEG	NEG	NEG	NEG	NEG
Hong Kong_133	NEG	NEG	NEG	NEG	NEG	NEG	NEG	NEG	NEG	NEG	NEG	NEG	NEG	NEG	NEG	NEG	NEG
Kuwait_009	NEG	NEG	NEG	NEG	NEG	NEG	NEG	NEG	NEG	NEG	NEG	NEG	NEG	NEG	NEG	NEG	NEG
Kuwait_103	NEG	NEG	NEG	NEG	NEG	NEG	NEG	NEG	NEG	NEG	NEG	NEG	NEG	NEG	NEG	NEG	NEG
Kuwait_107	NEG	NEG	NEG	NEG	NEG	NEG	NEG	NEG	NEG	NEG	NEG	NEG	NEG	NEG	NEG	NEG	NEG
Perth_03 AH 88	NEG	NEG	NEG	NEG	NEG	NEG	NEG	NEG	NEG	NEG	NEG	NEG	NEG	NEG	NEG	NEG	NEG
Perth_08-16905	NEG	NEG	NEG	NEG	NEG	NEG	NEG	NEG	NEG	NEG	NEG	NEG	NEG	NEG	NEG	NEG	NEG
Riyadh_Alfaisal-10_SICU_13_B5672_1117353	NEG	NEG	NEG	NEG	NEG	NEG	NEG	NEG	NEG	NEG	NEG	NEG	NEG	NEG	NEG	NEG	NEG
Riyadh_SSTI_17_3508602	NEG	NEG	NEG	NEG	NEG	NEG	NEG	NEG	NEG	NEG	NEG	NEG	NEG	NEG	NEG	NEG	NEG
Riyadh-2822088-R	NEG	NEG	NEG	NEG	NEG	NEG	NEG	NEG	NEG	NEG	NEG	NEG	NEG	NEG	NEG	NEG	NEG
Trinidad&Tobago_2012_195 (619/12)	NEG	NEG	NEG	NEG	NEG	NEG	NEG	NEG	NEG	NEG	NEG	NEG	NEG	NEG	NEG	NEG	NEG
Trinidad&Tobago_2012_370 (1112-52177)	NEG	NEG	NEG	NEG	NEG	NEG	NEG	NEG	NEG	NEG	NEG	NEG	NEG	NEG	NEG	NEG	NEG
Trinidad&Tobago_2012_378 (4139)	NEG	NEG	NEG	NEG	NEG	NEG	NEG	NEG	NEG	NEG	NEG	NEG	NEG	NEG	NEG	NEG	NEG
Trinidad&Tobago_2013_PA21	NEG	NEG	NEG	NEG	NEG	NEG	NEG	NEG	NEG	NEG	NEG	NEG	NEG	NEG	NEG	NEG	NEG
Trinidad&Tobago_2013_PA22	NEG	NEG	NEG	NEG	NEG	NEG	NEG	NEG	NEG	NEG	NEG	NEG	NEG	NEG	NEG	NEG	NEG
Trinidad&Tobago_2912_340 (8523/12)	NEG	NEG	NEG	NEG	NEG	NEG	NEG	NEG	NEG	NEG	NEG	NEG	NEG	NEG	NEG	NEG	NEG
Trinidad&Tobago_MRSA2010_105	NEG	NEG	NEG	NEG	NEG	NEG	NEG	NEG	NEG	NEG	NEG	NEG	NEG	NEG	NEG	NEG	NEG
Trinidad&Tobago_MRSA2010_108	NEG	NEG	NEG	NEG	NEG	NEG	NEG	NEG	NEG	NEG	NEG	NEG	NEG	NEG	NEG	NEG	NEG
Trinidad&Tobago_MRSA2010_33	NEG	NEG	NEG	NEG	NEG	NEG	NEG	NEG	NEG	NEG	NEG	NEG	NEG	NEG	NEG	NEG	NEG
Trinidad&Tobago_MRSA2010_79	NEG	NEG	NEG	NEG	NEG	NEG	NEG	NEG	NEG	NEG	NEG	NEG	NEG	NEG	NEG	NEG	NEG
Trinidad&Tobago_MRSA2010_88	NEG	NEG	NEG	NEG	NEG	NEG	NEG	NEG	NEG	NEG	NEG	NEG	NEG	NEG	NEG	NEG	NEG
Trinidad&Tobago_MRSA2010_97	NEG	NEG	NEG	NEG	NEG	NEG	NEG	NEG	NEG	NEG	NEG	NEG	NEG	NEG	NEG	NEG	NEG
Trinidad&Tobago_SSTI 2012_031	AMB	NEG	NEG	NEG	NEG	NEG	NEG	NEG	NEG	NEG	NEG	NEG	NEG	NEG	NEG	NEG	NEG
Uganda-29_17148_250811	NEG	NEG	NEG	NEG	NEG	NEG	NEG	NEG	NEG	NEG	NEG	NEG	NEG	NEG	NEG	NEG	NEG
>"South-East Asian Clad																	
UCIM6015	NEG	NEG	NEG	NEG	NEG	NEG	NEG	NEG	NEG	NEG	NEG	NEG	NEG	NEG	NEG	NEG	NEG
Hong Kong_121	NEG	NEG	NEG	NEG	NEG	NEG	NEG	NEG	NEG	NEG	NEG	NEG	NEG	NEG	NEG	NEG	NEG
>"South-East Asian Clad																	
Z172 (Taiwan)	NEG	NEG	NEG	NEG	NEG	NEG	NEG	NEG	NEG	NEG	NEG	NEG	NEG	NEG	NEG	NEG	NEG
Russia-19_0342_Kurgan	NEG	NEG	NEG	NEG	NEG	NEG	NEG	NEG	NEG	NEG	NEG	NEG	NEG	NEG	NEG	NEG	NEG
>"South-East Asian Clad																	
Perth_01-15392	NEG	NEG	NEG	NEG	NEG	NEG	NEG	NEG	NEG	NEG	NEG	NEG	NEG	NEG	NEG	NEG	NEG
>"South-East Asian Clad																	
XN108 (China)	NEG	NEG	NEG	NEG	NEG	NEG	NEG	NEG	NEG	NEG	NEG	NEG	NEG	NEG	NEG	NEG	NEG
NMR09 (India)	NEG	NEG	NEG	NEG	NEG	NEG	NEG	NEG	NEG	NEG	NEG	NEG	NEG	NEG	NEG	NEG	NEG
346 (Malaysia)	NEG	NEG	NEG	NEG	NEG	NEG	NEG	NEG	NEG	NEG	NEG	NEG	NEG	NEG	NEG	NEG	NEG
DS_009 (Thailand)	NEG	NEG	NEG	NEG	NEG	NEG	NEG	NEG	NEG	NEG	NEG	NEG	NEG	NEG	NEG	NEG	NEG
18ANR581005 (Ind)	NEG	NEG	NEG	NEG	NEG	NEG	NEG	NEG	NEG	NEG	NEG	NEG	NEG	NEG	NEG	NEG	NEG
Bengalaru_ST20141405	NEG	NEG	NEG	NEG	NEG	NEG	NEG	NEG	NEG	NEG	NEG	NEG	NEG	NEG	NEG	NEG	NEG
Bengalaru_ST20141406	NEG	NEG	NEG	NEG	NEG	NEG	NEG	NEG	NEG	NEG	NEG	NEG	NEG	NEG	NEG	NEG	NEG
Bengalaru_ST20141417	NEG	NEG	NEG	NEG	NEG	NEG	NEG	NEG	NEG	NEG	NEG	NEG	NEG	NEG	NEG	NEG	NEG
Hong Kong_107	NEG	NEG	NEG	NEG	NEG	NEG	NEG	NEG	NEG	NEG	NEG	NEG	NEG	NEG	NEG	NEG	NEG
Hong Kong_12	NEG	NEG	NEG	NEG	NEG	NEG	NEG	NEG	NEG	NEG	NEG	NEG	NEG	NEG	NEG	NEG	NEG
Hong Kong_132	NEG	NEG	NEG	NEG	NEG	NEG	NEG	NEG	NEG	NEG	NEG	NEG	NEG	NEG	NEG	NEG	NEG
Riyadh-3028763-R	NEG	NEG	NEG	NEG	NEG	NEG	NEG	NEG	NEG	NEG	NEG	NEG	NEG	NEG	NEG	NEG	NEG
Trinidad&Tobago_MRSA2010_94	NEG	NEG	NEG	NEG	NEG	NEG	NEG	NEG	NEG	NEG	NEG	NEG	NEG	NEG	NEG	NEG	NEG
>"South-East Asian Clad																	
M705 (Thailand)	NEG	NEG	NEG	NEG	NEG	NEG	NEG	NEG	NEG	NEG	NEG	NEG	NEG	NEG	NEG	NEG	NEG
S85 (Thailand)	NEG	NEG	NEG	NEG	NEG	NEG	NEG	NEG	NEG	NEG	NEG	NEG	NEG	NEG	NEG	NEG	NEG
Riyadh_Alfaisal-04_281014_S5076	NEG	NEG	NEG	NEG	NEG	NEG	NEG	NEG	NEG	NEG	NEG	NEG	NEG	NEG	NEG	NEG	NEG
Riyadh_Alfaisal-26_S15145_1104163	NEG	NEG	NEG	NEG	NEG	NEG	NEG	NEG	NEG	NEG	NEG	NEG	NEG	NEG	NEG	NEG	NEG
Riyadh-2817437-W	NEG	NEG	NEG	NEG	NEG	NEG	NEG	NEG	NEG	NEG	NEG	NEG	NEG	NEG	NEG	NEG	NEG
>"South-East Asian Clad																	
PPUKM-775-2009 (Malaysia)	NEG	NEG	NEG	NEG	NEG	NEG	NEG	NEG	NEG	NEG	NEG	NEG	NEG	NEG	NEG	NEG	NEG
Hong Kong_138	NEG	NEG	NEG	NEG	NEG	NEG	NEG	NEG	NEG	NEG	NEG	NEG	NEG	NEG	NEG	NEG	NEG
>"South-East Asian Clad																	
Kuwait_001	NEG	NEG	NEG	NEG	NEG	NEG	NEG	NEG	NEG	NEG	NEG	NEG	NEG	NEG	NEG	NEG	NEG
Kuwait_337	NEG	NEG	NEG	NEG	NEG	NEG	NEG	NEG	NEG	NEG	NEG	NEG	NEG	NEG	NEG	NEG	NEG
>"South-East Asian Clad																	
CUHK_HK2007 (China)	NEG	NEG	NEG	NEG	NEG	NEG	NEG	NEG	NEG	NEG	NEG	NEG	NEG	NEG	NEG	NEG	NEG
NMR02 (India)	NEG	NEG	NEG	NEG	NEG	NEG	NEG	NEG	NEG	NEG	NEG	NEG	NEG	NEG	NEG	NEG	NEG
VB1490 (India)	NEG	NEG	NEG	NEG	NEG	NEG	NEG	NEG	NEG	NEG	NEG	NEG	NEG	NEG	NEG	NEG	NEG
V521 (Korea)	NEG	NEG	NEG	NEG	NEG	NEG	NEG	NEG	NEG	NEG	NEG	NEG	NEG	NEG	NEG	NEG	NEG
HST-077 (Lebanon)	NEG	NEG	NEG	NEG	NEG	NEG	NEG	NEG	NEG	NEG	NEG	NEG	NEG	NEG	NEG	NEG	NEG
MAL1 (Malaysia)	NEG	NEG	NEG	NEG	NEG	NEG	NEG	NEG	NEG	NEG	NEG	NEG	NEG	NEG	NEG	NEG	NEG
MAL3 (Malaysia)	NEG	NEG	NEG	NEG	NEG	NEG	NEG	NEG	NEG	NEG	NEG	NEG	NEG	NEG	NEG	NEG	NEG
PPUKM-261-2009 (Malaysia)	NEG	NEG	NEG	NEG	NEG	NEG	NEG	NEG	NEG	NEG	NEG	NEG	NEG	NEG	NEG	NEG	NEG
PPUKM-332-2009 (Malaysia)	NEG	NEG	NEG	NEG	NEG	NEG	NEG	NEG	NEG	NEG	NEG	NEG	NEG	NEG	NEG	NEG	NEG
S38 (Thailand)	NEG	NEG	NEG	NEG	NEG	NEG	NEG	NEG	NEG	NEG	NEG	NEG	NEG	NEG	NEG	NEG	NEG
S7 (Thailand)	NEG	NEG	NEG	NEG	NEG	NEG	NEG	NEG	NEG	NEG	NEG	NEG	NEG	NEG	NEG	NEG	NEG
US_008 (Thailand)	NEG	NEG	NEG	NEG	NEG	NEG	NEG	NEG	NEG	NEG	NEG	NEG	NEG	NEG	NEG	NEG	NEG
US_030 (Thailand)	NEG	NEG	NEG	NEG	NEG	NEG	NEG	NEG	NEG	NEG	NEG	NEG	NEG	NEG	NEG	NEG	NEG
M170 (Tsunami-related)	NEG	NEG	NEG	NEG	NEG	NEG	NEG	NEG	NEG	NEG	NEG	NEG	NEG	NEG	NEG	NEG	NEG
BSAC697 (UK)	NEG	NEG	NEG	NEG	NEG	NEG	NEG	NEG	NEG	NEG	NEG	NEG	NEG	NEG	NEG	NEG	NEG
IS-189	NEG	NEG	NEG	NEG	NEG	NEG	NEG	NEG	NEG	NEG	NEG	NEG	NEG	NEG	NEG	NEG	NEG
MRGR3	NEG	NEG	NEG	NEG	NEG	NEG	NEG	NEG	NEG	NEG	NEG	NEG	NEG	NEG	NEG	NEG	NEG
smg07	NEG	NEG	NEG	NEG	NEG	NEG	NEG	NEG	NEG	NEG	NEG	NEG	NEG	NEG	NEG	NEG	NEG
AH1-AUS-EMRSA-3	NEG	NEG	NEG	NEG	NEG	NEG	NEG	NEG	NEG	NEG	NEG	NEG	NEG	NEG	NEG	NEG	NEG
Bengalaru_ST20141403	NEG	NEG	NEG	NEG	NEG	NEG	NEG	NEG	NEG	NEG	NEG	NEG	NEG	NEG	NEG	NEG	NEG
Finland E24_98541_Harmony	NEG	NEG	NEG	NEG	NEG	NEG	NEG	NEG	NEG	NEG	NEG	NEG	NEG	NEG	NEG	NEG	NEG
Hong Kong_10	NEG	NEG	NEG	NEG	NEG	NEG	NEG	NEG	NEG	NEG	NEG	NEG	NEG	NEG	NEG	NEG	NEG
Hong Kong_100	NEG	NEG	NEG	NEG	NEG	NEG	NEG	NEG	NEG	NEG	NEG	NEG	NEG	NEG	NEG	NEG	NEG
Hong Kong_199	NEG	NEG	NEG	NEG	NEG	NEG	NEG	NEG	NEG	NEG	NEG	NEG	NEG	NEG	NEG	NEG	NEG
Hong Kong_34	NEG	NEG	NEG	NEG	NEG	NEG	NEG	NEG	NEG	NEG	NEG	NEG	NEG	NEG	NEG	NEG	NEG
Hong Kong_88	NEG	NEG	NEG	neg	NEG	NEG	NEG	NEG	NEG	NEG	NEG	NEG	NEG	NEG	NEG	NEG	NEG
Hong Kong_92	NEG	NEG	NEG	NEG	NEG	NEG	NEG	NEG	NEG	NEG	NEG	NEG	NEG	NEG	NEG	NEG	NEG
Hong Kong_94	NEG	NEG	NEG	NEG	NEG	NEG	NEG	NEG	NEG	NEG	NEG	NEG	NEG	NEG	NEG	NEG	NEG
Kuwait_257	NEG	NEG	NEG	NEG	NEG	NEG	NEG	NEG	NEG	NEG	NEG	NEG	NEG	NEG	NEG	NEG	NEG
Perth_01-15330	NEG	NEG	NEG	NEG	NEG	NEG	NEG	NEG	NEG	NEG	NEG	NEG	NEG	NEG	NEG	NEG	NEG
Perth_01-15349	NEG	NEG	NEG	NEG	NEG	NEG	NEG	NEG	NEG	NEG	NEG	NEG	NEG	NEG	NEG	NEG	NEG
Perth_01-16176	NEG	NEG	NEG	NEG	NEG	NEG	NEG	NEG	NEG	NEG	NEG	NEG	NEG	NEG	NEG	NEG	NEG
Perth_WBG8379	NEG	NEG	NEG	NEG	NEG	NEG	NEG	NEG	NEG	NEG	NEG	NEG	NEG	NEG	NEG	NEG	NEG
Rawalpindi_Kidney Center_56	NEG	NEG	NEG	NEG	NEG	NEG	NEG	NEG	NEG	NEG	NEG	NEG	NEG	NEG	NEG	NEG	NEG
Riyadh_SSTI_05_3464614	NEG	NEG	NEG	NEG	NEG	NEG	NEG	NEG	NEG	NEG	NEG	NEG	NEG	NEG	NEG	NEG	NEG
Trinidad&Tobago_2012_157	NEG	NEG	NEG	NEG	NEG	NEG	NEG	NEG	NEG	NEG	NEG	NEG	NEG	NEG	NEG	NEG	NEG

STRAIN / ISOLATE	METHICILLIN RESISTANCE AND SCCmec TYPING																
	D1GU55	B2Y834	B6VQU0	Q3YK51	ydhK	DUF1958	Q4LAG7			QAP8 (SCCmec	Q8CU82	D3JD07	cas1		fusC (Q6GD50)	tirS	yeeA
	D1GU55	B2Y834	B6VQU0	Q3YK51	ydhK (FPR3757)	DUF1958	Q4LAG7 (SCC consensus)	Q4LAG7 (SCCmecV, SO385)	Q4LAG7 (SCCfus 45394F/MSSA476)	C5QAP8 (SCCmec XI)	Q8CU82	D3JD07	cas1 (MSHR1132)	cas1 (M06-0171)		tirS	yeeA
	Putative membrane protein. Subtyping SCCmec III, additional marker for SCCmec VII	Abortive phage resistance protein. Subtyping SCCmec IV, i.e., identification of SCCmec IV A, G, c and SCCmec MRSAZH47	Putative protein. Subtyping SCCmec IV, i.e., identification of SCCmec IVh/j	Putative protein. Subtyping SCCmec IV, i.e., identification of SCCmec IV g	Putative lipoprotein. Present in some composite elements comprising SCCmec and heavy metal resistance genes including the one in FPR3757, GenBank:	Putative protein. Subtyping SCCmec VT. Present, e.g., in PM1, GenBank BAFA but absent, e.g., in Strain 3957, GenBank AOFU	Putative protein located within SCCmec type V/SCCfus elements	Putative protein located within SCCmec type V elements	Putative protein located within SCCfus elements	Putative protein. Identification of SCCmec XI	Putative protein. Present in some SCCmec/fus composite elements such as, e.g., CMFT120, GenBank HF569094.1 and CMFT2, GenBank	Putative protein. Present in some composite SCC elements	CRISPR-associated endonuclease 1. Present in MSHR1132	CRISPR-associated endonuclease 1. Present in M06/0171, GenBank HE980450.1	SCC-associated fusidic acid resistance gene. Present alone in "SCCfus" elements or together with mecA in composite elements. Most	Staphylococcal TIR-protein binding protein. Subtyping SCCfus because it is frequently, but not always, accompanying fusC	Putative DNA methyltransferase. Subtyping SCCmecIV/fus composite elements
>"South-East Asian Clad																	
M418	NEG	NEG	NEG	NEG	NEG	NEG	NEG	NEG	NEG	NEG	NEG	NEG	NEG	NEG	NEG	NEG	NEG
>"South-East Asian Clad																	
NMR07 (India)	NEG	NEG	NEG	NEG	NEG	NEG	NEG	NEG	NEG	NEG	NEG	NEG	NEG	NEG	NEG	NEG	NEG
NMR08 (India)	NEG	NEG	NEG	NEG	NEG	NEG	NEG	NEG	NEG	NEG	NEG	NEG	NEG	NEG	NEG	NEG	NEG
Bengalaru_ST20121978	NEG	NEG	NEG	NEG	NEG	NEG	NEG	NEG	NEG	NEG	NEG	NEG	NEG	NEG	NEG	NEG	NEG
Hong Kong_89	NEG	NEG	NEG	NEG	NEG	NEG	NEG	NEG	NEG	NEG	POS	NEG	NEG	NEG	NEG	NEG	NEG
>"South-East Asian Clad																	
Perth_2005_AH_5	NEG	NEG	NEG	NEG	NEG	NEG	NEG	NEG	NEG	NEG	NEG	NEG	NEG	NEG	NEG	NEG	NEG
>"South-East Asian Clad																	
Trinidad&Tobago_MRSA2010_103	NEG	NEG	NEG	NEG	NEG	NEG	NEG	NEG	NEG	NEG	NEG	NEG	NEG	NEG	NEG	NEG	NEG
Trinidad&Tobago_2013_PA25	NEG	NEG	NEG	NEG	NEG	NEG	NEG	NEG	NEG	NEG	NEG	NEG	NEG	NEG	NEG	NEG	NEG
Trinidad&Tobago_MRSA2010_110	NEG	NEG	NEG	NEG	NEG	NEG	NEG	NEG	NEG	NEG	NEG	NEG	NEG	NEG	NEG	NEG	NEG
Trinidad&Tobago_SSTI 2012_111	NEG	NEG	NEG	NEG	NEG	NEG	NEG	NEG	NEG	NEG	NEG	NEG	NEG	NEG	NEG	NEG	NEG
Trinidad&Tobago_MRSA2010_158	NEG	NEG	NEG	NEG	NEG	NEG	NEG	NEG	NEG	NEG	NEG	NEG	NEG	NEG	NEG	NEG	NEG
>"South-East Asian Clad																	
Kuwait_122	NEG	NEG	NEG	NEG	NEG	NEG	NEG	NEG	NEG	NEG	NEG	NEG	NEG	NEG	NEG	NEG	NEG
Kuwait_002	NEG	NEG	NEG	NEG	NEG	NEG	NEG	NEG	NEG	NEG	NEG	NEG	NEG	NEG	NEG	NEG	NEG
Kuwait_196	NEG	NEG	NEG	NEG	NEG	NEG	NEG	NEG	NEG	NEG	NEG	NEG	NEG	NEG	NEG	NEG	NEG
Kuwait_199	NEG	NEG	NEG	NEG	NEG	NEG	NEG	NEG	NEG	NEG	NEG	NEG	NEG	NEG	NEG	NEG	NEG
Hong Kong_136	NEG	NEG	NEG	NEG	NEG	NEG	NEG	NEG	NEG	NEG	NEG	NEG	NEG	NEG	NEG	NEG	NEG
>"South-East Asian Clad																	
P32 (Poland)	NEG	NEG	NEG	NEG	NEG	NEG	NEG	NEG	NEG	NEG	NEG	NEG	NEG	NEG	NEG	NEG	NEG
>"South-East Asian Clad																	
Perth_2015-S32313	NEG	NEG	NEG	NEG	NEG	NEG	NEG	NEG	NEG	NEG	NEG	NEG	NEG	NEG	NEG	NEG	NEG
>"hla-negative South-Ea																	
DEN907 (Denmark)	NEG	NEG	NEG	NEG	NEG	NEG	NEG	NEG	NEG	NEG	NEG	NEG	NEG	NEG	NEG	NEG	NEG
>"hla-negative South-Ea																	
S26 (Thailand)	NEG	NEG	NEG	NEG	NEG	NEG	NEG	NEG	NEG	NEG	NEG	NEG	NEG	NEG	NEG	NEG	NEG
S42 (Thailand)	NEG	NEG	NEG	NEG	NEG	NEG	NEG	NEG	NEG	NEG	NEG	NEG	NEG	NEG	NEG	NEG	NEG
S97 (Thailand)	NEG	NEG	NEG	NEG	NEG	NEG	NEG	NEG	NEG	NEG	NEG	NEG	NEG	NEG	NEG	NEG	NEG
S78 (Thailand)	NEG	NEG	NEG	NEG	NEG	NEG	NEG	NEG	NEG	NEG	NEG	NEG	NEG	NEG	NEG	NEG	NEG
S25 (Thailand)	NEG	NEG	NEG	NEG	NEG	NEG	NEG	NEG	NEG	NEG	NEG	NEG	NEG	NEG	NEG	NEG	NEG
S21 (Thailand)	NEG	NEG	NEG	NEG	NEG	NEG	NEG	NEG	NEG	NEG	NEG	NEG	NEG	NEG	NEG	NEG	NEG
S106 (Thailand)	NEG	NEG	NEG	NEG	NEG	NEG	NEG	NEG	NEG	NEG	NEG	NEG	NEG	NEG	NEG	NEG	NEG
S24 (Thailand)	NEG	NEG	NEG	NEG	NEG	NEG	NEG	NEG	NEG	NEG	NEG	NEG	NEG	NEG	NEG	NEG	NEG
S39 (Thailand)	NEG	NEG	NEG	NEG	NEG	NEG	NEG	NEG	NEG	NEG	NEG	NEG	NEG	NEG	NEG	NEG	NEG
S2 (Thailand)	NEG	NEG	NEG	NEG	NEG	NEG	NEG	NEG	NEG	NEG	NEG	NEG	NEG	NEG	NEG	NEG	NEG
S81 (Thailand)	NEG	NEG	NEG	NEG	NEG	NEG	NEG	NEG	NEG	NEG	NEG	NEG	NEG	NEG	NEG	NEG	NEG
M116 (Vietnam)	NEG	NEG	NEG	NEG	NEG	NEG	NEG	NEG	NEG	NEG	NEG	NEG	NEG	NEG	NEG	NEG	NEG
>"Portuguese Clade": Pc																	
FFP103 (Portugal)	NEG	NEG	NEG	NEG	NEG	NEG	NEG	NEG	NEG	NEG	NEG	NEG	NEG	NEG	NEG	NEG	NEG
HDG2 (Portugal)	NEG	NEG	NEG	NEG	NEG	NEG	NEG	NEG	NEG	NEG	NEG	NEG	NEG	NEG	NEG	NEG	NEG
HSA10 (Portugal)	NEG	NEG	NEG	NEG	NEG	NEG	NEG	NEG	NEG	NEG	NEG	NEG	NEG	NEG	NEG	NEG	NEG
HSA11 (Portugal)	NEG	NEG	NEG	NEG	NEG	NEG	NEG	NEG	NEG	NEG	NEG	NEG	NEG	NEG	NEG	NEG	NEG
ICP5011 (Portugal)	NEG	NEG	NEG	NEG	NEG	NEG	NEG	NEG	NEG	NEG	NEG	NEG	NEG	NEG	NEG	NEG	NEG
ICP5014 (Portugal)	NEG	NEG	NEG	NEG	NEG	NEG	NEG	NEG	NEG	NEG	NEG	NEG	NEG	NEG	NEG	NEG	NEG
ICP5062 (Portugal)	NEG	NEG	NEG	NEG	NEG	NEG	NEG	NEG	NEG	NEG	NEG	NEG	NEG	NEG	NEG	NEG	NEG
Strain 1063_ATCC 33592 FDAARGOS_3 (USA)	NEG	NEG	NEG	NEG	NEG	NEG	NEG	NEG	NEG	NEG	NEG	NEG	NEG	NEG	NEG	NEG	NEG
	NEG	NEG	NEG	NEG	NEG	NEG	NEG	NEG	NEG	NEG	NEG	NEG	NEG	NEG	NEG	NEG	NEG
Russia-07_0110_Kurgan	NEG	NEG	NEG	NEG	NEG	NEG	NEG	NEG	NEG	NEG	NEG	NEG	NEG	NEG	NEG	NEG	NEG
Russia-11_0164_Kurgan	NEG	NEG	NEG	NEG	NEG	NEG	NEG	NEG	NEG	NEG	NEG	NEG	NEG	NEG	NEG	NEG	NEG
Russia-20_0367_Kurgan	NEG	NEG	NEG	NEG	NEG	NEG	NEG	NEG	NEG	NEG	NEG	NEG	NEG	NEG	NEG	NEG	NEG
Russia-21_0390_Chelyabinsk	NEG	NEG	NEG	NEG	NEG	NEG	NEG	NEG	NEG	NEG	NEG	NEG	NEG	NEG	NEG	NEG	NEG
>"South American/ Midc																	
AGT1 (Argentina)	NEG	NEG	NEG	NEG	NEG	NEG	NEG	NEG	NEG	NEG	NEG	NEG	NEG	NEG	NEG	NEG	NEG
AGT120 (Argentina)	NEG	NEG	NEG	NEG	NEG	NEG	NEG	NEG	NEG	NEG	NEG	NEG	NEG	NEG	NEG	NEG	NEG
AGT67 (Argentina)	NEG	NEG	NEG	NEG	NEG	NEG	NEG	NEG	NEG	NEG	NEG	NEG	NEG	NEG	NEG	NEG	NEG
AGT9 (Argentina)	NEG	NEG	NEG	NEG	NEG	NEG	NEG	NEG	NEG	NEG	NEG	NEG	NEG	NEG	NEG	NEG	NEG
RA3 (Argentina)	NEG	NEG	NEG	NEG	NEG	NEG	NEG	NEG	NEG	NEG	NEG	NEG	NEG	NEG	NEG	NEG	NEG
RA6 (Argentina)	NEG	NEG	NEG	NEG	NEG	NEG	NEG	NEG	NEG	NEG	NEG	NEG	NEG	NEG	NEG	NEG	NEG
RA7 (Argentina)	NEG	NEG	NEG	NEG	NEG	NEG	NEG	NEG	NEG	NEG	NEG	NEG	NEG	NEG	NEG	NEG	NEG
Be62 (Brazil)	NEG	NEG	NEG	NEG	NEG	NEG	NEG	NEG	NEG	NEG	NEG	NEG	NEG	NEG	NEG	NEG	NEG
Bmb9393 (Brazil)	NEG	NEG	NEG	NEG	NEG	NEG	NEG	NEG	NEG	NEG	NEG	NEG	NEG	NEG	NEG	NEG	NEG
BRA36 (Brazil)	NEG	NEG	NEG	NEG	NEG	NEG	NEG	NEG	NEG	NEG	NEG	NEG	NEG	NEG	NEG	NEG	NEG
BZ48 (Brazil)	NEG	NEG	NEG	NEG	NEG	NEG	NEG	NEG	NEG	NEG	NEG	NEG	NEG	NEG	NEG	NEG	NEG
Gv51 (Brazil)	NEG	NEG	NEG	NEG	NEG	NEG	NEG	NEG	NEG	NEG	NEG	NEG	NEG	NEG	NEG	NEG	NEG
Gv69 (Brazil)	NEG	NEG	NEG	NEG	NEG	NEG	NEG	NEG	NEG	NEG	NEG	NEG	NEG	NEG	NEG	NEG	NEG
Gv88 (Brazil)	NEG	NEG	NEG	NEG	NEG	NEG	NEG	NEG	NEG	NEG	NEG	NEG	NEG	NEG	NEG	NEG	NEG
HC1335 (Brazil)	NEG	NEG	NEG	NEG	NEG	NEG	NEG	NEG	NEG	NEG	NEG	NEG	NEG	NEG	NEG	NEG	NEG
HC1340 (Brazil)	NEG	NEG	NEG	NEG	NEG	NEG	NEG	NEG	NEG	NEG	NEG	NEG	NEG	NEG	NEG	NEG	NEG
HC556 (Brazil)	NEG	NEG	NEG	NEG	NEG	NEG	NEG	NEG	NEG	NEG	NEG	NEG	NEG	NEG	NEG	NEG	NEG
HU25 (Brazil)	NEG	NEG	NEG	NEG	NEG	NEG	NEG	NEG	NEG	NEG	NEG	NEG	NEG	NEG	NEG	NEG	NEG
UB563 (Brazil)	NEG	NEG	NEG	NEG	NEG	NEG	NEG	NEG	NEG	NEG	NEG	NEG	NEG	NEG	NEG	NEG	NEG
2A8 (Czech Republic)	NEG	NEG	NEG	NEG	NEG	NEG	NEG	NEG	NEG	NEG	NEG	NEG	NEG	NEG	NEG	NEG	NEG
M1229 (Denmark)	NEG	NEG	NEG	NEG	NEG	NEG	NEG	NEG	NEG	NEG	NEG	NEG	NEG	NEG	NEG	NEG	NEG
FRICAR (France)	NEG	NEG	NEG	NEG	NEG	NEG	NEG	NEG	NEG	NEG	NEG	NEG	NEG	NEG	NEG	NEG	NEG
D90 (Germany)	NEG	NEG	NEG	NEG	NEG	NEG	NEG	NEG	NEG	NEG	NEG	NEG	NEG	NEG	NEG	NEG	NEG
LIT68 (Lithuania)	NEG	NEG	NEG	NEG	NEG	NEG	NEG	NEG	NEG	NEG	NEG	NEG	NEG	NEG	NEG	NEG	NEG
LIT76 (Lithuania)	NEG	NEG	NEG	NEG	NEG	NEG	NEG	NEG	NEG	NEG	NEG	NEG	NEG	NEG	NEG	NEG	NEG
HGSA142 (Portugal)	NEG	NEG	NEG	NEG	NEG	NEG	NEG	NEG	NEG	NEG	NEG	NEG	NEG	NEG	NEG	NEG	NEG
HGSA9 (Portugal)	NEG	NEG	NEG	NEG	NEG	NEG	NEG	NEG	NEG	NEG	NEG	NEG	NEG	NEG	NEG	NEG	NEG
HSJ216 (Portugal)	NEG	NEG	NEG	NEG	NEG	NEG	NEG	NEG	NEG	NEG	NEG	NEG	NEG	NEG	NEG	NEG	NEG
M278 (Portugal)	NEG	NEG	NEG	NEG	NEG	NEG	NEG	NEG	NEG	NEG	NEG	NEG	NEG	NEG	NEG	NEG	NEG
ES26 (Spain)	NEG	NEG	NEG	NEG	NEG	NEG	NEG	NEG	NEG	NEG	NEG	NEG	NEG	NEG	NEG	NEG	NEG
UK102 (UK)	NEG	NEG	NEG	NEG	NEG	NEG	NEG	NEG	NEG	NEG	NEG	NEG	NEG	NEG	NEG	NEG	NEG
URU34 (Uruguay)	NEG	NEG	NEG	NEG	NEG	NEG	NEG	NEG	NEG	NEG	NEG	NEG	NEG	NEG	NEG	NEG	NEG
IS-125	NEG	NEG	NEG	NEG	NEG	NEG	NEG	NEG	NEG	NEG	NEG	NEG	NEG	NEG	NEG	NEG	NEG
IS-157	NEG	NEG	NEG	NEG	NEG	NEG	NEG	NEG	NEG	NEG	NEG	NEG	NEG	NEG	NEG	NEG	NEG
Ecuador_1	NEG	NEG	NEG	NEG	NEG	NEG	NEG	NEG	NEG	NEG	NEG	NEG	NEG	NEG	NEG	NEG	NEG
Ecuador_2	NEG	NEG	NEG	NEG	NEG	NEG	NEG	NEG	NEG	NEG	NEG	NEG	NEG	NEG	NEG	NEG	NEG
Dresden_15ANR574263	NEG	NEG	NEG	NEG	NEG	NEG	NEG	NEG	NEG	NEG	NEG	NEG	NEG	NEG	NEG	NEG	NEG
UK-EMRSA-11	NEG	NEG	NEG	NEG	NEG	NEG	NEG	NEG	NEG	NEG	NEG	NEG	NEG	NEG	NEG	NEG	NEG
>"South American/ Midc																	
BRA2 (Brazil)	NEG	NEG	NEG	NEG	NEG	NEG	NEG	NEG	NEG	NEG	NEG	NEG	NEG	NEG	NEG	NEG	NEG
CHL1 (Chile)	NEG	NEG	NEG	NEG	NEG	NEG	NEG	NEG	NEG	NEG	NEG	NEG	NEG	NEG	NEG	NEG	NEG
CHL151 (Chile)	NEG	NEG	NEG	NEG	NEG	NEG	NEG	NEG	NEG	NEG	NEG	NEG	NEG	NEG	NEG	NEG	NEG
UCL378 (Chile)	NEG	NEG	NEG	NEG	NEG	NEG	NEG	NEG	NEG	NEG	NEG	NEG	NEG	NEG	NEG	NEG	NEG
UP72 (Peru)	NEG	NEG	NEG	NEG	NEG	NEG	NEG	NEG	NEG	NEG	NEG	NEG	NEG	NEG	NEG	NEG	NEG
UP81 (Peru)	NEG	NEG	NEG	NEG	NEG	NEG	NEG	NEG	NEG	NEG	NEG	NEG	NEG	NEG	NEG	NEG	NEG
>"South American/ Midc																	
UP1073 (Peru)	NEG	NEG	NEG	NEG	NEG	NEG	NEG	NEG	NEG	NEG	NEG	NEG	NEG	NEG	NEG	NEG	NEG

STRAIN / ISOLATE	METHICILLIN RESISTANCE AND SCCmec TYPING																	
	D1GU55	B2Y834	B6VQU0	Q3YK51	ydhK	DUF1958	Q4LAG7			QAP8 (SCCmec	Q8CU82	D3JD07	cas1		fusC (Q6GD50)	tirS	yeeA
	D1GU55	B2Y834	B6VQU0	Q3YK51	ydhK (FPR3757)	DUF1958	Q4LAG7 (SCC consensus)	Q4LAG7 (SCCmecV, SO385)	Q4LAG7 (SCCfus 45394F/MSSA476)	C5QAP8 (SCCmec XI)	Q8CU82	D3JD07	cas1 (MSHR1132)	cas1 (M06-0171)		tirS	yeeA
	Putative membrane protein. Subtyping SCCmec III, additional marker for SCCmec VII	Abortive phage resistance protein. Subtyping SCCmec IV, i.e., identification of SCCmec IV A, G, c and SCCmec MRSAZH47	Putative protein. Subtyping SCCmec IV, i.e., identification of SCCmec IVh/j	Putative protein. Subtyping SCCmec IV, i.e., identification of SCCmec IV g	Putative lipoprotein. Present in some composite elements comprising SCCmec and heavy metal resistance genes including the one in FPR3757, GenBank:	Putative protein. Subtyping SCCmec VT. Present, e.g., in PM1, GenBank BAFA but absent, e.g., in Strain 3957, GenBank AOFU	Putative protein located within SCCmec type V/SCCfus elements	Putative protein located within SCCmec type V elements	Putative protein located within SCCfus elements	Putative protein. Identification of SCCmec XI	Putative protein. Present in some SCCmec/fus composite elements such as, e.g., CMFT120, GenBank HF569094.1 and CMFT2, GenBank	Putative protein. Present in some composite SCC elements	CRISPR-associated endonuclease 1. Present in MSHR1132	CRISPR-associated endonuclease 1. Present in M06/0171, GenBank HE980450.1	SCC-associated fusidic acid resistance gene. Present alone in "SCCfus" elements or together with mecA in composite elements. Most	Staphylococcal TIR-protein binding protein. Subtyping SCCfus because it is frequently, but not always, accompanying fusC	Putative DNA methyltransferase. Subtyping SCCmecIV/fus composite elements
**>"South American/ Mid**																		
LIT89 (Lithuania)	NEG	NEG	NEG	NEG	NEG	NEG	NEG	NEG	NEG	NEG	NEG	NEG	NEG	NEG	NEG	NEG	NEG	NEG
MRSA_PR1 (Malaysia)	NEG	NEG	NEG	NEG	NEG	NEG	NEG	NEG	NEG	NEG	NEG	NEG	NEG	NEG	NEG	NEG	NEG	NEG
Riyadh-2888905-R	NEG	NEG	NEG	NEG	NEG	NEG	NEG	NEG	NEG	NEG	NEG	NEG	NEG	NEG	NEG	NEG	NEG	NEG
Riyadh-2888915-BC	NEG	NEG	NEG	NEG	NEG	NEG	NEG	NEG	NEG	NEG	NEG	NEG	NEG	NEG	NEG	NEG	NEG	NEG
Riyadh-2793706-R	NEG	NEG	NEG	NEG	NEG	NEG	NEG	NEG	NEG	NEG	NEG	NEG	NEG	NEG	NEG	NEG	NEG	NEG
**>"South American/ Mid**																		
Riyadh_SSTI_18_3502925	NEG	NEG	NEG	NEG	NEG	NEG	NEG	NEG	NEG	NEG	NEG	NEG	NEG	NEG	NEG	NEG	NEG	NEG
Riyadh-2822825-W	NEG	NEG	NEG	NEG	NEG	NEG	NEG	NEG	NEG	NEG	NEG	NEG	NEG	NEG	NEG	NEG	NEG	NEG
**>"South American/ Mid**																		
UK-EMRSA-9	NEG	NEG	NEG	NEG	NEG	NEG	NEG	NEG	NEG	NEG	NEG	NEG	NEG	NEG	NEG	NEG	NEG	NEG
**>"South American/ Mid**																		
Lome_HT20020815	NEG	NEG	NEG	NEG	NEG	NEG	NEG	NEG	NEG	NEG	NEG	NEG	NEG	NEG	NEG	NEG	NEG	NEG
**>"South American/ Mid**																		
NA32 (Denmark)	NEG	NEG	NEG	NEG	NEG	NEG	NEG	NEG	NEG	NEG	NEG	NEG	NEG	NEG	NEG	NEG	NEG	NEG
H24 (Egypt)	NEG	NEG	NEG	NEG	NEG	NEG	NEG	NEG	NEG	NEG	NEG	NEG	NEG	NEG	NEG	NEG	NEG	NEG
ATCC BAA-39 (=HUSA304) (Hungary)	NEG	NEG	NEG	NEG	NEG	NEG	NEG	NEG	NEG	NEG	NEG	NEG	NEG	NEG	NEG	NEG	NEG	NEG
HUSA304 (Hungary)	NEG	NEG	NEG	NEG	NEG	NEG	NEG	NEG	NEG	NEG	NEG	NEG	NEG	NEG	NEG	NEG	NEG	NEG
HU106 (Hungary)	NEG	NEG	NEG	NEG	NEG	NEG	NEG	NEG	NEG	NEG	NEG	NEG	NEG	NEG	NEG	NEG	NEG	NEG
BSAC27 (UK)	NEG	NEG	NEG	NEG	NEG	NEG	NEG	NEG	NEG	NEG	NEG	NEG	NEG	NEG	NEG	NEG	NEG	NEG
NCTC13131, UK-EMRSA-4 (UK)	NEG	NEG	NEG	NEG	NEG	NEG	NEG	NEG	NEG	NEG	NEG	NEG	NEG	NEG	NEG	NEG	NEG	NEG
	AMB	NEG	NEG	NEG	NEG	NEG	NEG	NEG	NEG	NEG	NEG	NEG	NEG	NEG	NEG	NEG	NEG	NEG
Algiers_HT20040080	NEG	NEG	NEG	NEG	NEG	NEG	NEG	NEG	NEG	NEG	NEG	NEG	NEG	NEG	NEG	NEG	NEG	NEG
Dublin-DSH_AR09_0.0066	NEG	NEG	NEG	NEG	NEG	NEG	NEG	NEG	NEG	NEG	NEG	NEG	NEG	NEG	NEG	NEG	NEG	NEG
Dublin-DSH_AR09_0-0065	NEG	NEG	NEG	NEG	NEG	NEG	NEG	NEG	NEG	NEG	NEG	NEG	NEG	NEG	NEG	NEG	NEG	NEG
Dublin-DSH_Phenotype-III_84	NEG	NEG	NEG	NEG	NEG	NEG	NEG	NEG	NEG	NEG	NEG	NEG	NEG	NEG	NEG	NEG	NEG	NEG
Hong Kong_130	NEG	NEG	NEG	NEG	NEG	NEG	NEG	NEG	NEG	NEG	NEG	NEG	NEG	NEG	NEG	NEG	NEG	NEG
Kuwait_018	NEG	NEG	NEG	NEG	NEG	NEG	NEG	NEG	NEG	NEG	NEG	NEG	NEG	NEG	NEG	NEG	NEG	NEG
Perth_08-17726	NEG	NEG	NEG	NEG	NEG	NEG	NEG	NEG	NEG	NEG	NEG	NEG	NEG	NEG	NEG	NEG	NEG	NEG
Riyadh_Alfaisal/KKKSUH_86_MRS-14-279	NEG	NEG	NEG	NEG	NEG	NEG	NEG	NEG	NEG	NEG	NEG	NEG	NEG	NEG	NEG	NEG	NEG	NEG
Riyadh_Alfaisal-04_Z3861_831588	NEG	NEG	NEG	NEG	NEG	NEG	NEG	NEG	NEG	NEG	NEG	NEG	NEG	NEG	NEG	NEG	NEG	NEG
Riyadh_Alfaisal-30_S15724_1108013	NEG	NEG	NEG	NEG	NEG	NEG	NEG	NEG	NEG	NEG	NEG	NEG	NEG	NEG	NEG	NEG	NEG	NEG
Riyadh_Alfaisal-6_22A_13_B3992_397721	NEG	NEG	NEG	NEG	NEG	NEG	NEG	NEG	NEG	NEG	NEG	NEG	NEG	NEG	NEG	NEG	NEG	NEG
Riyadh_SSTI_52_3615482	NEG	NEG	NEG	NEG	NEG	NEG	NEG	NEG	NEG	NEG	NEG	NEG	NEG	NEG	NEG	NEG	NEG	NEG
Riyadh-2817276-2	NEG	NEG	NEG	NEG	NEG	NEG	NEG	NEG	NEG	NEG	NEG	NEG	NEG	NEG	NEG	NEG	NEG	NEG
Riyadh-2891670-W	NEG	NEG	NEG	NEG	NEG	NEG	NEG	NEG	NEG	NEG	NEG	NEG	NEG	NEG	NEG	NEG	NEG	NEG
Riyadh-3006920-W	NEG	NEG	NEG	NEG	NEG	NEG	NEG	NEG	NEG	NEG	NEG	NEG	NEG	NEG	NEG	NEG	NEG	NEG
Riyadh-R2567782	NEG	NEG	NEG	NEG	NEG	NEG	NEG	NEG	NEG	NEG	NEG	NEG	NEG	NEG	NEG	NEG	NEG	NEG
Russia-18_0252_Krasnoyarsk_SK2	NEG	NEG	NEG	NEG	NEG	NEG	NEG	NEG	NEG	NEG	NEG	NEG	NEG	NEG	NEG	NEG	NEG	NEG
UK-EMRSA-7	NEG	NEG	NEG	NEG	NEG	NEG	NEG	NEG	NEG	NEG	NEG	NEG	NEG	NEG	NEG	NEG	NEG	NEG
**>"South American/ Mid**																		
Dublin-DSH_AR23_0073	NEG	NEG	NEG	NEG	NEG	NEG	NEG	NEG	NEG	NEG	NEG	NEG	NEG	NEG	NEG	NEG	NEG	NEG
**>Related to "South Ame**																		
MRSA-OC3 (Russia)	NEG	NEG	NEG	NEG	NEG	NEG	NEG	NEG	NEG	NEG	NEG	NEG	NEG	NEG	NEG	NEG	NEG	NEG
Russia-12_0176_Krasnoyarsk	NEG	NEG	NEG	NEG	NEG	NEG	NEG	NEG	NEG	NEG	NEG	NEG	NEG	NEG	NEG	NEG	NEG	NEG
Russia-13_0180_Krasnoyarsk	NEG	NEG	NEG	NEG	NEG	NEG	NEG	NEG	NEG	NEG	NEG	NEG	NEG	NEG	NEG	NEG	NEG	NEG
Russia-16_0249_Krasnoyarsk	NEG	NEG	NEG	NEG	NEG	NEG	NEG	NEG	NEG	NEG	NEG	NEG	NEG	NEG	NEG	NEG	NEG	NEG
Russia-17_0250_Krasnoyarsk	NEG	NEG	NEG	NEG	NEG	NEG	NEG	NEG	NEG	NEG	NEG	NEG	NEG	NEG	NEG	NEG	NEG	NEG
**>Related to "South Ame**																		
URU110 (Uruguay)	NEG	NEG	NEG	NEG	NEG	NEG	NEG	NEG	NEG	NEG	NEG	NEG	NEG	NEG	NEG	NEG	NEG	NEG
**>Related to "South Ame**																		
DS_014 (Thailand)	NEG	NEG	NEG	NEG	NEG	NEG	NEG	NEG	NEG	NEG	NEG	NEG	NEG	NEG	NEG	NEG	NEG	NEG
**>Unassigned Middle Eas**																		
Frankfurt_Oder_0490031797 (Libya)	NEG	NEG	NEG	NEG	NEG	NEG	NEG	NEG	NEG	NEG	NEG	NEG	NEG	NEG	NEG	NEG	NEG	NEG
Kuwait_192	NEG	NEG	NEG	NEG	NEG	NEG	NEG	NEG	NEG	NEG	NEG	NEG	NEG	NEG	NEG	NEG	NEG	NEG
Riyadh-9	NEG	NEG	NEG	NEG	NEG	NEG	NEG	NEG	NEG	NEG	NEG	NEG	NEG	NEG	NEG	NEG	NEG	NEG
Russia-24_0407_Moscow	NEG	NEG	NEG	NEG	NEG	NEG	NEG	NEG	NEG	NEG	NEG	NEG	NEG	NEG	NEG	NEG	NEG	NEG

STRAIN / ISOLATE	METHICILLIN RESISTANCE AND SCCmec TYPING																		
	ACME total	arcA-SCC	arcB-SCC	arcC-SCC	arcD-SCC	opp3B	opp3C	adhC	speG	ccrA/B-1	ccrA/B-2	ccrA-3	ccrB-3	ccrAA		ccrC		ccrA-4	ccrB-4
	ACME total	arcA-SCC	arcB-SCC	arcC-SCC	arcD-SCC	opp3B	opp3C (FPR3757)	adhC	speG (FPR3757)					ccrAA (MRSAZH47) probe 1	ccrAA (MRSAZH47) probe 2	ccrC	ccrC allele	ccrA-4	ccrB-4
	ACME total	Arginine deiminase	Ornithine carbamoyltransferase	Carbamate kinase	Arginine/ornithine antiporter	Oligopeptide permease, channel-forming protein	Oligopeptide permease, channel-forming protein	Alcohol dehydrogenase, zinc-containing	Spermidine N-acetyltransferase. Usually associated with ACME or composite SCCmec/ACME elements	Cassette chromosome recombinase A/B, type 1	Cassette chromosome recombinase A/B, type 2	Cassette chromosome recombinase A, type 3	Cassette chromosome recombinase B, type 3	Cassette chromosome recombinase homologue, accompanying ccrC		Cassette chromosome recombinase C		Cassette chromosome recombinase A/B, type 4	
**>"Eurasian Clade": TUR1**																			
TUR1 (Turkey)	NEG	NEG	NEG	NEG	NEG	NEG	NEG	NEG	NEG	NEG	NEG	POS	POS	NEG	NEG	POS	PM1	NEG	NEG
TUR9 (Turkey)	NEG	NEG	NEG	NEG	NEG	NEG	NEG	NEG	NEG	NEG	NEG	POS	POS	NEG	NEG	POS	PM1	NEG	NEG
**>"Eurasian Clade" : T013**																			
T0131 (China)	NEG	NEG	NEG	NEG	NEG	NEG	NEG	NEG	NEG	NEG	NEG	POS	POS	NEG	NEG	POS	PM1	NEG	NEG
MU4 (Turkey)	NEG	NEG	NEG	NEG	NEG	NEG	NEG	NEG	NEG	NEG	NEG	POS	POS	NEG	NEG	POS	PM1	NEG	NEG
DEU11 (Turkey)	NEG	NEG	NEG	NEG	NEG	NEG	NEG	NEG	NEG	NEG	NEG	POS	POS	NEG	NEG	POS	PM1	NEG	NEG
DEU16 (Turkey)	NEG	NEG	NEG	NEG	NEG	NEG	NEG	NEG	NEG	NEG	NEG	POS	POS	NEG	NEG	POS	PM1	NEG	NEG
Romania_Iasi_MRSA-07_54	NEG	NEG	NEG	NEG	NEG	NEG	NEG	NEG	NEG	NEG	NEG	POS	POS	NEG	NEG	POS		NEG	NEG
**>"Eurasian Clade": arsC-**																			
Romania_Iasi_MRSA-05_46	NEG	NEG	NEG	NEG	NEG	NEG	NEG	NEG	NEG	NEG	NEG	POS	POS	NEG	NEG	POS		NEG	NEG
Romania_Iasi_MRSA-06_47	NEG	NEG	NEG	NEG	NEG	NEG	NEG	NEG	NEG	NEG	NEG	POS	POS	NEG	NEG	POS		NEG	NEG
**>"Eurasian Clade": 16K/**																			
CN79 (China)	NEG	NEG	NEG	NEG	NEG	NEG	NEG	NEG	NEG	NEG	NEG	POS	POS	NEG	NEG	POS	PM1	NEG	NEG
CUHK_BJ2002 (China)	NEG	NEG	NEG	NEG	NEG	NEG	NEG	NEG	NEG	NEG	NEG	POS	POS	NEG	NEG	POS	PM1	NEG	NEG
CUHK_BJ2007 (China)	NEG	NEG	NEG	NEG	NEG	NEG	NEG	NEG	NEG	NEG	NEG	POS	POS	NEG	NEG	POS	PM1	NEG	NEG
3HK (Czech Republic)	NEG	NEG	NEG	NEG	NEG	NEG	NEG	NEG	NEG	NEG	NEG	POS	POS	NEG	NEG	POS	PM1	NEG	NEG
HU109 (Hungary)	NEG	NEG	NEG	NEG	NEG	NEG	NEG	NEG	NEG	NEG	NEG	POS	POS	NEG	NEG	POS	PM1	NEG	NEG
HUR18 (Hungary)	NEG	NEG	NEG	NEG	NEG	NEG	NEG	NEG	NEG	NEG	NEG	POS	POS	NEG	NEG	POS		NEG	NEG
H482 (Romania)	NEG	NEG	NEG	NEG	NEG	NEG	NEG	NEG	NEG	NEG	NEG	POS	POS	NEG	NEG	POS	PM1	NEG	NEG
16K (Russia)	NEG	NEG	NEG	NEG	NEG	NEG	NEG	NEG	NEG	NEG	NEG	POS	POS	NEG	NEG	POS	PM1	NEG	NEG
DEU10 (Turkey)	NEG	NEG	NEG	NEG	NEG	NEG	NEG	NEG	NEG	NEG	NEG	POS	POS	NEG	NEG	POS	PM1	NEG	NEG
DEU12 (Turkey)	NEG	NEG	NEG	NEG	NEG	NEG	NEG	NEG	NEG	NEG	NEG	POS	POS	NEG	NEG	POS	PM1	NEG	NEG
DEU14 (Turkey)	NEG	NEG	NEG	NEG	NEG	NEG	NEG	NEG	NEG	NEG	NEG	POS	POS	NEG	NEG	POS	PM1	NEG	NEG
DEU15 (Turkey)	NEG	NEG	NEG	NEG	NEG	NEG	NEG	NEG	NEG	NEG	NEG	POS	POS	NEG	NEG	POS	PM1	NEG	NEG
DEU17 (Turkey)	NEG	NEG	NEG	NEG	NEG	NEG	NEG	NEG	NEG	NEG	NEG	POS	POS	NEG	NEG	POS	PM1	NEG	NEG
DEU19 (Turkey)	NEG	NEG	NEG	NEG	NEG	NEG	NEG	NEG	NEG	NEG	NEG	POS	POS	NEG	NEG	POS	PM1	NEG	NEG
DEU2 (Turkey)	NEG	NEG	NEG	NEG	NEG	NEG	NEG	NEG	NEG	NEG	NEG	POS	POS	NEG	NEG	POS	PM1	NEG	NEG
DEU20 (Turkey)	NEG	NEG	NEG	NEG	NEG	NEG	NEG	NEG	NEG	NEG	NEG	POS	POS	NEG	NEG	POS	PM1	NEG	NEG
DEU23 (Turkey)	NEG	NEG	NEG	NEG	NEG	NEG	NEG	NEG	NEG	NEG	NEG	POS	POS	NEG	NEG	POS	PM1	NEG	NEG
DEU3 (Turkey)	NEG	NEG	NEG	NEG	NEG	NEG	NEG	NEG	NEG	NEG	NEG	POS	POS	NEG	NEG	POS	PM1	NEG	NEG
DEU5 (Turkey)	NEG	NEG	NEG	NEG	NEG	NEG	NEG	NEG	NEG	NEG	NEG	POS	POS	NEG	NEG	POS	PM1	NEG	NEG
DEU6 (Turkey)	NEG	NEG	NEG	NEG	NEG	NEG	NEG	NEG	NEG	NEG	NEG	POS	POS	NEG	NEG	POS	PM1	NEG	NEG
DEU8 (Turkey)	NEG	NEG	NEG	NEG	NEG	NEG	NEG	NEG	NEG	NEG	NEG	POS	POS	NEG	NEG	POS	PM1	NEG	NEG
HU11 (Turkey)	NEG	NEG	NEG	NEG	NEG	NEG	NEG	NEG	NEG	NEG	NEG	POS	POS	NEG	NEG	POS	PM1	NEG	NEG
HU13 (Turkey)	NEG	NEG	NEG	NEG	NEG	NEG	NEG	NEG	NEG	NEG	NEG	POS	POS	NEG	NEG	POS	PM1	NEG	NEG
HU14 (Turkey)	NEG	NEG	NEG	NEG	NEG	NEG	NEG	NEG	NEG	NEG	NEG	POS	POS	NEG	NEG	POS	PM1	NEG	NEG
HU15 (Turkey)	NEG	NEG	NEG	NEG	NEG	NEG	NEG	NEG	NEG	NEG	NEG	POS	POS	NEG	NEG	POS	PM1	NEG	NEG
HU16 (Turkey)	NEG	NEG	NEG	NEG	NEG	NEG	NEG	NEG	NEG	NEG	NEG	POS	POS	NEG	NEG	POS	PM1	NEG	NEG
HU17 (Turkey)	NEG	NEG	NEG	NEG	NEG	NEG	NEG	NEG	NEG	NEG	NEG	POS	POS	NEG	NEG	POS	PM1	NEG	NEG
HU21 (Turkey)	NEG	NEG	NEG	NEG	NEG	NEG	NEG	NEG	NEG	NEG	NEG	POS	POS	NEG	NEG	POS	PM1	NEG	NEG
HU23 (Turkey)	NEG	NEG	NEG	NEG	NEG	NEG	NEG	NEG	NEG	NEG	NEG	POS	POS	NEG	NEG	POS	PM1	NEG	NEG
HU26 (Turkey)	NEG	NEG	NEG	NEG	NEG	NEG	NEG	NEG	NEG	NEG	NEG	POS	POS	NEG	NEG	POS	PM1	NEG	NEG
HU4 (Turkey)	NEG	NEG	NEG	NEG	NEG	NEG	NEG	NEG	NEG	NEG	NEG	POS	POS	NEG	NEG	POS	PM1	NEG	NEG
HU5 (Turkey)	NEG	NEG	NEG	NEG	NEG	NEG	NEG	NEG	NEG	NEG	NEG	POS	POS	NEG	NEG	POS	PM1	NEG	NEG
HU6 (Turkey)	NEG	NEG	NEG	NEG	NEG	NEG	NEG	NEG	NEG	NEG	NEG	POS	POS	NEG	NEG	POS	PM1	NEG	NEG
HU7 (Turkey)	NEG	NEG	NEG	NEG	NEG	NEG	NEG	NEG	NEG	NEG	NEG	POS	POS	NEG	NEG	POS	PM1	NEG	NEG
HU8 (Turkey)	NEG	NEG	NEG	NEG	NEG	NEG	NEG	NEG	NEG	NEG	NEG	POS	POS	NEG	NEG	POS	PM1	NEG	NEG
HU9 (Turkey)	NEG	NEG	NEG	NEG	NEG	NEG	NEG	NEG	NEG	NEG	NEG	POS	POS	NEG	NEG	POS	PM1	NEG	NEG
IU1 (Turkey)	NEG	NEG	NEG	NEG	NEG	NEG	NEG	NEG	NEG	NEG	NEG	POS	POS	NEG	NEG	POS	PM1	NEG	NEG
IU10 (Turkey)	NEG	NEG	NEG	NEG	NEG	NEG	NEG	NEG	NEG	NEG	NEG	POS	POS	NEG	NEG	POS	PM1	NEG	NEG
IU11 (Turkey)	NEG	NEG	NEG	NEG	NEG	NEG	NEG	NEG	NEG	NEG	NEG	POS	POS	NEG	NEG	POS	PM1	NEG	NEG
IU12 (Turkey)	NEG	NEG	NEG	NEG	NEG	NEG	NEG	NEG	NEG	NEG	NEG	POS	POS	NEG	NEG	POS	PM1	NEG	NEG
IU13 (Turkey)	NEG	NEG	NEG	NEG	NEG	NEG	NEG	NEG	NEG	NEG	NEG	POS	POS	NEG	NEG	POS	PM1	NEG	NEG
IU15 (Turkey)	NEG	NEG	NEG	NEG	NEG	NEG	NEG	NEG	NEG	NEG	NEG	POS	POS	NEG	NEG	POS	PM1	NEG	NEG
IU18 (Turkey)	NEG	NEG	NEG	NEG	NEG	NEG	NEG	NEG	NEG	NEG	NEG	POS	POS	NEG	NEG	POS	PM1	NEG	NEG
IU19 (Turkey)	NEG	NEG	NEG	NEG	NEG	NEG	NEG	NEG	NEG	NEG	NEG	POS	POS	NEG	NEG	POS	PM1	NEG	NEG
IU2 (Turkey)	NEG	NEG	NEG	NEG	NEG	NEG	NEG	NEG	NEG	NEG	NEG	POS	POS	NEG	NEG	POS	PM1	NEG	NEG
IU4 (Turkey)	NEG	NEG	NEG	NEG	NEG	NEG	NEG	NEG	NEG	NEG	NEG	POS	POS	NEG	NEG	POS	PM1	NEG	NEG
IU6 (Turkey)	NEG	NEG	NEG	NEG	NEG	NEG	NEG	NEG	NEG	NEG	NEG	POS	POS	NEG	NEG	POS	PM1	NEG	NEG
IU7 (Turkey)	NEG	NEG	NEG	NEG	NEG	NEG	NEG	NEG	NEG	NEG	NEG	POS	POS	NEG	NEG	POS	PM1	NEG	NEG
IU9 (Turkey)	NEG	NEG	NEG	NEG	NEG	NEG	NEG	NEG	NEG	NEG	NEG	POS	POS	NEG	NEG	POS	PM1	NEG	NEG
MU1 (Turkey)	NEG	NEG	NEG	NEG	NEG	NEG	NEG	NEG	NEG	NEG	NEG	POS	POS	NEG	NEG	POS	PM1	NEG	NEG
MU10 (Turkey)	NEG	NEG	NEG	NEG	NEG	NEG	NEG	NEG	NEG	NEG	NEG	POS	POS	NEG	NEG	POS	PM1	NEG	NEG
MU20 (Turkey)	NEG	NEG	NEG	NEG	NEG	NEG	NEG	NEG	NEG	NEG	NEG	POS	POS	NEG	NEG	POS	PM1	NEG	NEG
Mu3 (Turkey)	NEG	NEG	NEG	NEG	NEG	NEG	NEG	NEG	NEG	NEG	NEG	POS	POS	NEG	NEG	POS	PM1	NEG	NEG
MU5 (Turkey)	NEG	NEG	NEG	NEG	NEG	NEG	NEG	NEG	NEG	NEG	NEG	POS	POS	NEG	NEG	POS	PM1	NEG	NEG
MU6 (Turkey)	NEG	NEG	NEG	NEG	NEG	NEG	NEG	NEG	NEG	NEG	NEG	POS	POS	NEG	NEG	POS	PM1	NEG	NEG
MU7 (Turkey)	NEG	NEG	NEG	NEG	NEG	NEG	NEG	NEG	NEG	NEG	NEG	POS	POS	NEG	NEG	POS	PM1	NEG	NEG
TUR27 (Turkey)	NEG	NEG	NEG	NEG	NEG	NEG	NEG	NEG	NEG	NEG	NEG	POS	POS	NEG	NEG	POS	PM1	NEG	NEG
NCTR 32S	NEG	NEG	NEG	NEG	NEG	NEG	NEG	NEG	NEG	NEG	NEG	POS	POS	NEG	NEG	POS	PM1	NEG	NEG
SA02_A4 = Russia-06_0085_Moscow	NEG	NEG	NEG	NEG	NEG	NEG	NEG	NEG	NEG	NEG	NEG	POS	POS	NEG	NEG	POS	PM1	NEG	NEG
	NEG	NEG	NEG	NEG	NEG	NEG	NEG	NEG	NEG	NEG	NEG	POS	POS	NEG	NEG	POS		NEG	NEG
Dresden_08V35987 (Turkey)	NEG	NEG	NEG	NEG	NEG	NEG	NEG	NEG	NEG	NEG	NEG	POS	POS	NEG	NEG	POS		NEG	NEG
Dresden_17ANR577152	NEG	NEG	NEG	NEG	NEG	NEG	NEG	NEG	NEG	NEG	NEG	POS	POS	NEG	NEG	POS		NEG	NEG
Dresden_17ANR580374 (Makedonia)	NEG	NEG	NEG	NEG	NEG	NEG	NEG	NEG	NEG	NEG	NEG	POS	POS	NEG	NEG	POS		NEG	NEG
Hong Kong_69-II	NEG	NEG	NEG	NEG	NEG	NEG	NEG	NEG	NEG	NEG	NEG	POS	POS	NEG	NEG	POS		NEG	NEG
Hong Kong_93	NEG	NEG	NEG	NEG	NEG	NEG	NEG	NEG	NEG	NEG	NEG	POS	POS	NEG	AMB	POS		NEG	NEG
Rawalpindi_Kidney Center_03_SK1	NEG	NEG	NEG	NEG	NEG	NEG	NEG	NEG	NEG	NEG	NEG	POS	POS	NEG	NEG	POS		NEG	NEG
Rawalpindi_Kidney Center_08	NEG	NEG	NEG	NEG	NEG	NEG	NEG	NEG	NEG	NEG	NEG	POS	POS	NEG	NEG	POS		NEG	NEG
Rawalpindi_Kidney Center_10	NEG	NEG	NEG	NEG	NEG	NEG	NEG	NEG	NEG	NEG	NEG	POS	POS	NEG	NEG	POS		NEG	NEG
Rawalpindi_Kidney Center_19	NEG	NEG	NEG	NEG	NEG	NEG	NEG	NEG	NEG	NEG	NEG	POS	POS	NEG	AMB	POS		NEG	NEG
Rawalpindi_Kidney Center_50_SK1	NEG	NEG	NEG	NEG	NEG	NEG	NEG	NEG	NEG	NEG	NEG	POS	POS	NEG	NEG	POS		NEG	NEG
Romania_Iasi_BC-49_430	NEG	NEG	NEG	NEG	NEG	NEG	NEG	NEG	NEG	NEG	NEG	POS	POS	NEG	NEG	POS		NEG	NEG
Romania_Iasi_MRSA_284	NEG	NEG	NEG	NEG	NEG	NEG	NEG	NEG	NEG	NEG	NEG	POS	POS	NEG	NEG	POS		NEG	NEG
Romania_Iasi_MRSA-32_318	NEG	NEG	NEG	NEG	NEG	NEG	NEG	NEG	NEG	NEG	NEG	POS	POS	NEG	NEG	POS		NEG	NEG
Romania_Iasi_MRSA-54_430	NEG	NEG	NEG	NEG	NEG	NEG	NEG	NEG	NEG	NEG	NEG	POS	POS	NEG	AMB	POS		NEG	NEG
Romania_Iasi_SSTI-01_2	NEG	NEG	NEG	NEG	NEG	NEG	NEG	NEG	NEG	NEG	NEG	POS	POS	NEG	NEG	POS		NEG	NEG
Romania_Iasi_SSTI-10_101	NEG	NEG	NEG	NEG	NEG	NEG	NEG	NEG	NEG	NEG	NEG	POS	POS	NEG	NEG	POS		NEG	NEG
Romania_Iasi_SSTI-11_106	NEG	NEG	NEG	NEG	NEG	NEG	NEG	NEG	NEG	NEG	NEG	POS	POS	NEG	NEG	POS		NEG	NEG
Russia-01_0001_SaintPetersburg	NEG	NEG	NEG	NEG	NEG	NEG	NEG	NEG	NEG	NEG	NEG	POS	POS	NEG	NEG	POS		NEG	NEG
Russia-02_0004_SaintPetersburg	NEG	NEG	NEG	NEG	NEG	NEG	NEG	NEG	NEG	NEG	NEG	POS	POS	NEG	NEG	POS		NEG	NEG
Russia-03_0057_SaintPetersburg	NEG	NEG	NEG	NEG	NEG	NEG	NEG	NEG	NEG	NEG	NEG	POS	POS	NEG	NEG	POS		NEG	NEG
Russia-04_0076_Moscow	NEG	NEG	NEG	NEG	NEG	NEG	NEG	NEG	NEG	NEG	NEG	POS	POS	NEG	NEG	POS		NEG	NEG
Russia-05_0078_Moscow	NEG	NEG	NEG	NEG	NEG	NEG	NEG	NEG	NEG	NEG	NEG	POS	POS	NEG	NEG	POS		NEG	NEG
Russia-08_0135_Moscow	NEG	NEG	NEG	NEG	NEG	NEG	NEG	NEG	NEG	NEG	NEG	POS	POS	NEG	NEG	POS		NEG	NEG
Russia-09_0150_Kurgan	NEG	NEG	NEG	NEG	NEG	NEG	NEG	NEG	NEG	NEG	NEG	POS	POS	NEG	NEG	POS		NEG	NEG
Russia-10_0162_Kurgan	NEG	NEG	NEG	NEG	NEG	NEG	NEG	NEG	NEG	NEG	NEG	POS	POS	NEG	NEG	POS		NEG	NEG
Russia-14_0184_Moscow	NEG	NEG	NEG	NEG	NEG	NEG	NEG	NEG	NEG	NEG	NEG	POS	POS	NEG	NEG	POS		NEG	NEG
Russia-15_0232_SaintPetersburg	NEG	NEG	NEG	NEG	NEG	NEG	NEG	NEG	NEG	NEG	NEG	POS	POS	NEG	NEG	POS		NEG	NEG
Russia-22_0391_Chelyabinsk	NEG	NEG	NEG	NEG	NEG	NEG	NEG	NEG	NEG	NEG	NEG	POS	POS	NEG	NEG	POS		NEG	NEG
Russia-23_0392_Chelyabinsk	NEG	NEG	NEG	NEG	NEG	NEG	NEG	NEG	NEG	NEG	NEG	POS	POS	NEG	NEG	POS		NEG	NEG
**>"Eurasian Clade" : IU17**																			
IU17 (Turkey)	NEG	NEG	NEG	NEG	NEG	NEG	NEG	NEG	NEG	NEG	NEG	POS	POS	NEG	NEG	POS	PM1	NEG	NEG
IU20 (Turkey)	NEG	NEG	NEG	NEG	NEG	NEG	NEG	NEG	NEG	NEG	NEG	POS	POS	NEG	NEG	POS	PM1	NEG	NEG

STRAIN / ISOLATE	METHICILLIN RESISTANCE AND SCCmec TYPING																		
	ACME total	arcA-SCC	arcB-SCC	arcC-SCC	arcD-SCC	opp3B	opp3C	adhC	speG	ccrA/B-1	ccrA/B-2	ccrA-3	ccrB-3	ccrAA		ccrC		ccrA-4	ccrB-4
	ACME total	arcA-SCC	arcB-SCC	arcC-SCC	arcD-SCC	opp3B	opp3C (FPR3757)	adhC	speG (FPR3757)					ccrAA (MRSAZH47) probe 1	ccrAA (MRSAZH47) probe 2	ccrC	ccrC allele	ccrA-4	ccrB-4
	ACME total	Arginine deiminase	Ornithine carbamoyltransferase	Carbamate kinase	Arginine/ornithine antiporter	Oligopeptide permease, channel-forming protein	Oligopeptide permease, channel-forming protein	Alcohol dehydrogenase, zinc-containing	Spermidine N-acetyltransferase. Usually associated with ACME or composite SCCmec/ACME elements	Cassette chromosome recombinase A/B, type 1	Cassette chromosome recombinase A/B, type 2	Cassette chromosome recombinase A, type 3	Cassette chromosome recombinase B, type 3	Cassette chromosome recombinase homologue, accompanying ccrC		Cassette chromosome recombinase C		Cassette chromosome recombinase A/B, type 4	
**>"Eurasian Clade": Spora**																			
Dresden_16ANR581769	NEG	NEG	NEG	NEG	NEG	NEG	NEG	NEG	NEG	NEG	NEG	POS	POS	NEG	POS	POS		NEG	NEG
Dresden_17ANR578769	NEG	NEG	NEG	NEG	NEG	NEG	NEG	NEG	NEG	NEG	NEG	POS	POS	NEG	NEG	POS		NEG	NEG
**>"Eurasian Clade": Kuwa**																			
Kuwait_306	POS	POS	POS	POS	POS	NEG	NEG	NEG	NEG	NEG	NEG	POS	POS	NEG	POS	POS		NEG	NEG
Kuwait_301	POS	POS	POS	POS	POS	NEG	NEG	NEG	NEG	NEG	NEG	POS	POS	NEG	NEG	POS		NEG	NEG
**>"European Clade": "Gre**																			
GRE18 (Greece)	NEG	NEG	NEG	NEG	NEG	NEG	NEG	NEG	NEG	NEG	NEG	POS	POS	NEG	NEG	POS	PM1	NEG	NEG
GRE317 (Greece)	NEG	NEG	NEG	NEG	NEG	NEG	NEG	NEG	NEG	NEG	NEG	POS	POS	NEG	NEG	POS	PM1	NEG	NEG
GRE4 (Greece)	NEG	NEG	NEG	NEG	NEG	NEG	NEG	NEG	NEG	NEG	NEG	POS	POS	NEG	NEG	POS	PM1	NEG	NEG
R35 (USA)	NEG	NEG	NEG	NEG	NEG	NEG	NEG	NEG	NEG	NEG	NEG	POS	POS	NEG	NEG	POS	PM1	NEG	NEG
Casablanca HT20060548	NEG	NEG	NEG	NEG	NEG	NEG	NEG	NEG	NEG	NEG	NEG	POS	POS	NEG	NEG	POS		NEG	NEG
Casablanca HT20060550	NEG	NEG	NEG	NEG	NEG	NEG	NEG	NEG	NEG	NEG	NEG	POS	POS	NEG	NEG	POS		NEG	NEG
Dresden_01V34572 (Greece)	NEG	NEG	NEG	neg	NEG	NEG	NEG	NEG	NEG	NEG	NEG	POS	POS	NEG	NEG	POS		NEG	NEG
Dresden_01V36469	NEG	NEG	NEG	NEG	NEG	NEG	NEG	NEG	NEG	NEG	NEG	POS	POS	NEG	NEG	POS		NEG	NEG
Dresden 01V39123	NEG	NEG	NEG	NEG	NEG	NEG	NEG	NEG	NEG	NEG	NEG	POS	POS	NEG	NEG	POS		NEG	NEG
Dresden 01V39143	NEG	NEG	NEG	NEG	NEG	NEG	NEG	NEG	NEG	NEG	NEG	POS	POS	NEG	NEG	POS		NEG	NEG
Greece 1 3680 Harmony	NEG	NEG	NEG	NEG	NEG	NEG	NEG	NEG	NEG	NEG	NEG	POS	POS	NEG	NEG	POS		NEG	NEG
**>"European Clade": UK-(**																			
ANS46 (Australia)	NEG	NEG	NEG	NEG	NEG	NEG	NEG	NEG	NEG	NEG	NEG	POS	POS	NEG	NEG	POS	PM1	NEG	NEG
LHH1 (USA)	NEG	NEG	NEG	NEG	NEG	NEG	NEG	NEG	NEG	NEG	NEG	POS	POS	NEG	NEG	POS	PM1	NEG	NEG
Dublin-DSH_AR01_0-1085	NEG	NEG	NEG	NEG	NEG	NEG	NEG	NEG	NEG	NEG	NEG	POS	POS	NEG	NEG	POS		NEG	NEG
Dublin-DSH_AR01_0-1118	NEG	NEG	NEG	NEG	NEG	NEG	NEG	NEG	NEG	NEG	NEG	POS	POS	NEG	NEG	POS		NEG	NEG
Dublin-DSH_AR15_0098	NEG	NEG	NEG	NEG	NEG	NEG	NEG	NEG	NEG	NEG	NEG	POS	POS	NEG	NEG	POS		NEG	NEG
Dublin-DSH_AR15_0104	NEG	NEG	NEG	neg	NEG	NEG	NEG	NEG	NEG	NEG	NEG	POS	POS	NEG	NEG	POS		NEG	NEG
UK_NCTC11939_UK-1_Harmony	NEG	NEG	NEG	NEG	NEG	NEG	NEG	NEG	NEG	NEG	NEG	POS	POS	NEG	NEG	POS		NEG	NEG
**>"European Clade": BK2**																			
BK2421 (USA)	NEG	NEG	NEG	NEG	NEG	NEG	NEG	NEG	NEG	NEG	NEG	POS	POS	NEG	NEG	POS	PM1	NEG	NEG
**>"Aussi/NZ Clade", JKD6**																			
JKD6009 (Australia)	NEG	NEG	NEG	NEG	NEG	NEG	NEG	NEG	NEG	NEG	NEG	POS	POS	NEG	NEG	NEG		NEG	NEG
JKD6008 (New Zealand)	NEG	NEG	NEG	NEG	NEG	NEG	NEG	NEG	NEG	NEG	NEG	POS	POS	NEG	NEG	NEG		NEG	NEG
Perth_2015-S20209	NEG	NEG	NEG	NEG	NEG	NEG	NEG	NEG	NEG	NEG	NEG	POS	POS	NEG	NEG	NEG		NEG	NEG
Perth_2015-S20305	NEG	NEG	NEG	NEG	NEG	NEG	NEG	NEG	NEG	NEG	NEG	POS	POS	NEG	NEG	NEG		NEG	NEG
Perth_2015-S20603	NEG	NEG	NEG	NEG	NEG	NEG	NEG	NEG	NEG	NEG	NEG	POS	POS	NEG	NEG	NEG		NEG	NEG
Perth_2015-S20607	NEG	NEG	NEG	NEG	NEG	NEG	NEG	NEG	NEG	NEG	NEG	POS	POS	NEG	NEG	NEG		NEG	NEG
Perth_2015-S20608	NEG	NEG	NEG	NEG	NEG	NEG	NEG	NEG	NEG	NEG	NEG	POS	POS	NEG	NEG	NEG		NEG	NEG
Perth_2015-S20610	NEG	NEG	NEG	NEG	NEG	NEG	NEG	NEG	NEG	NEG	NEG	POS	POS	NEG	NEG	NEG		NEG	NEG
Perth_2015-S20613	NEG	NEG	NEG	NEG	NEG	NEG	NEG	NEG	NEG	NEG	NEG	POS	POS	NEG	NEG	NEG		NEG	NEG
Perth_2015-S20617	NEG	NEG	NEG	NEG	NEG	NEG	NEG	NEG	NEG	NEG	NEG	POS	POS	NEG	NEG	NEG		NEG	NEG
Perth_2015-S20622	NEG	NEG	NEG	NEG	NEG	NEG	NEG	NEG	NEG	NEG	NEG	POS	POS	NEG	NEG	NEG		NEG	NEG
Perth_2015-S23619	NEG	NEG	NEG	NEG	NEG	NEG	NEG	NEG	NEG	NEG	NEG	POS	POS	NEG	NEG	NEG		NEG	NEG
Perth_2015-S41210	NEG	NEG	NEG	NEG	NEG	NEG	NEG	NEG	NEG	NEG	NEG	POS	POS	NEG	NEG	NEG		NEG	NEG
Perth_2015-S51001	NEG	NEG	NEG	NEG	NEG	NEG	NEG	NEG	NEG	NEG	NEG	POS	POS	NEG	NEG	NEG		NEG	NEG
Perth_2015-S51003	NEG	NEG	NEG	NEG	NEG	NEG	NEG	NEG	NEG	NEG	NEG	POS	POS	NEG	NEG	NEG		NEG	NEG
Perth_2015-S51006	NEG	NEG	NEG	NEG	NEG	NEG	NEG	NEG	NEG	NEG	NEG	POS	POS	NEG	NEG	NEG		NEG	NEG
Perth_2015-S51007	NEG	NEG	NEG	NEG	NEG	NEG	NEG	NEG	NEG	NEG	NEG	POS	POS	NEG	NEG	NEG		NEG	NEG
Perth_2015-S51008	NEG	NEG	NEG	NEG	NEG	NEG	NEG	NEG	NEG	NEG	NEG	POS	POS	NEG	NEG	NEG		NEG	NEG
Perth_2015-S51014	NEG	NEG	NEG	NEG	NEG	NEG	NEG	NEG	NEG	NEG	NEG	POS	POS	NEG	NEG	NEG		NEG	NEG
Perth_2015-S51408	NEG	NEG	NEG	NEG	NEG	NEG	NEG	NEG	NEG	NEG	NEG	POS	POS	NEG	NEG	NEG		NEG	NEG
Perth_2016-S20614	NEG	NEG	NEG	NEG	NEG	NEG	NEG	NEG	NEG	NEG	NEG	POS	POS	NEG	NEG	NEG		NEG	NEG
Perth_2016-S23614	NEG	NEG	NEG	NEG	NEG	NEG	NEG	NEG	NEG	NEG	NEG	POS	POS	NEG	NEG	NEG		NEG	NEG
Perth_2016-S42009	NEG	NEG	NEG	NEG	NEG	NEG	NEG	NEG	NEG	NEG	NEG	POS	POS	NEG	NEG	NEG		NEG	NEG
Perth_2016-S51001	NEG	NEG	NEG	NEG	NEG	NEG	NEG	NEG	NEG	NEG	NEG	POS	POS	NEG	NEG	NEG		NEG	NEG
Perth_2016-S51403	NEG	NEG	NEG	NEG	NEG	NEG	NEG	NEG	NEG	NEG	NEG	POS	POS	NEG	NEG	NEG		NEG	NEG
Perth_07 RPA 20	NEG	NEG	NEG	NEG	NEG	NEG	NEG	NEG	NEG	NEG	NEG	POS	POS	NEG	NEG	NEG		NEG	NEG
Perth_08-19088	NEG	NEG	NEG	NEG	NEG	NEG	NEG	NEG	NEG	NEG	NEG	POS	POS	NEG	NEG	NEG		NEG	NEG
Perth_01-15351	NEG	NEG	NEG	NEG	NEG	NEG	NEG	NEG	NEG	NEG	NEG	POS	POS	NEG	NEG	NEG		NEG	NEG
Perth_01-15357	NEG	NEG	NEG	NEG	NEG	NEG	NEG	NEG	NEG	NEG	NEG	POS	POS	NEG	NEG	NEG		NEG	NEG
Perth_01-15419	NEG	NEG	NEG	neg	NEG	NEG	NEG	NEG	NEG	NEG	NEG	POS	POS	NEG	NEG	NEG		NEG	NEG
Perth_01-16309	NEG	NEG	NEG	NEG	NEG	NEG	NEG	NEG	NEG	NEG	NEG	POS	POS	NEG	NEG	NEG		NEG	NEG
Perth_03 SNP 95	NEG	NEG	NEG	NEG	NEG	NEG	NEG	NEG	NEG	NEG	NEG	POS	POS	NEG	NEG	NEG		NEG	NEG
Perth_2003_AH_47	NEG	NEG	NEG	NEG	NEG	NEG	NEG	NEG	NEG	NEG	NEG	POS	POS	NEG	NEG	NEG		NEG	NEG
**>"Australian/NZ Clade":**																			
Perth_2015-S20623	POS	POS	POS	POS	POS	NEG	NEG	NEG	NEG	NEG	NEG	POS	POS	NEG	NEG	NEG		NEG	NEG
Perth_2015-S20826	POS	POS	POS	POS	POS	NEG	NEG	NEG	NEG	NEG	NEG	POS	POS	NEG	NEG	NEG		NEG	NEG
Perth_05 NH 56	POS	POS	POS	POS	POS	NEG	NEG	NEG	NEG	NEG	NEG	POS	POS	NEG	NEG	NEG		NEG	NEG
**>CC5-MRSA-III LA MRSA**																			
Delbrück 38 isolate from domestic turkey	NEG	NEG	NEG	NEG	NEG	NEG	NEG	NEG	NEG	NEG	NEG	POS	POS	NEG	NEG	NEG		NEG	NEG
**> Staph. pseudintermed**																			
KM1381	NEG	NEG	NEG	NEG	NEG	NEG	NEG	NEG	NEG	NEG	NEG	POS	POS	NEG	NEG	NEG		NEG	NEG
**>"South-East Asian Clad**																			
M92 (Canada)	NEG	NEG	NEG	NEG	NEG	NEG	NEG	NEG	NEG	NEG	NEG	POS	POS	NEG	NEG	POS	TSGH17	NEG	NEG
CHI59 (China)	NEG	NEG	NEG	NEG	NEG	NEG	NEG	NEG	NEG	NEG	NEG	POS	POS	NEG	NEG	POS	TSGH17	NEG	NEG
CHI61 (China)	NEG	NEG	NEG	NEG	NEG	NEG	NEG	NEG	NEG	NEG	NEG	POS	POS	NEG	NEG	POS	TSGH17	NEG	NEG
CUHK_HK1997 (China)	NEG	NEG	NEG	NEG	NEG	NEG	NEG	NEG	NEG	NEG	NEG	POS	POS	NEG	NEG	POS	TSGH17	NEG	NEG
M996 (China)	NEG	NEG	NEG	NEG	NEG	NEG	NEG	NEG	NEG	NEG	NEG	POS	POS	NEG	NEG	POS	TSGH17	NEG	NEG
H211 (Denmark)	NEG	NEG	NEG	NEG	NEG	NEG	NEG	NEG	NEG	NEG	NEG	POS	POS	NEG	NEG	POS	TSGH17	NEG	NEG
H216 (Denmark)	NEG	NEG	NEG	NEG	NEG	NEG	NEG	NEG	NEG	NEG	NEG	POS	POS	NEG	NEG	POS	TSGH17	NEG	NEG
D71 (Germany)	NEG	NEG	NEG	NEG	NEG	NEG	NEG	NEG	NEG	NEG	NEG	POS	POS	NEG	NEG	POS	TSGH17	NEG	NEG
NMR05 (India)	NEG	NEG	NEG	NEG	NEG	NEG	NEG	NEG	NEG	NEG	NEG	POS	POS	NEG	NEG	POS	TSGH17	NEG	NEG
MAL11 (Malaysia)	NEG	NEG	NEG	NEG	NEG	NEG	NEG	NEG	NEG	NEG	NEG	POS	POS	NEG	NEG	POS	TSGH17	NEG	NEG
MAL9 (Malaysia)	NEG	NEG	NEG	NEG	NEG	NEG	NEG	NEG	NEG	NEG	NEG	POS	POS	NEG	NEG	POS	TSGH17	NEG	NEG
Na21 (Sri Lanka)	NEG	NEG	NEG	NEG	NEG	NEG	NEG	NEG	NEG	NEG	NEG	POS	POS	NEG	NEG	POS	TSGH17	NEG	NEG
M592 (Syria)	NEG	NEG	NEG	NEG	NEG	NEG	NEG	NEG	NEG	NEG	NEG	POS	POS	NEG	NEG	POS	TSGH17	NEG	NEG
H202 (Thailand)	NEG	NEG	NEG	NEG	NEG	NEG	NEG	NEG	NEG	NEG	NEG	POS	POS	NEG	NEG	POS	TSGH17	NEG	NEG
S102 (Thailand)	NEG	NEG	NEG	NEG	NEG	NEG	NEG	NEG	NEG	NEG	NEG	POS	POS	NEG	NEG	POS	TSGH17	NEG	NEG
S130 (Thailand)	NEG	NEG	NEG	NEG	NEG	NEG	NEG	NEG	NEG	NEG	NEG	POS	POS	NEG	NEG	POS	TSGH17	NEG	NEG
S40 (Thailand)	NEG	NEG	NEG	NEG	NEG	NEG	NEG	NEG	NEG	NEG	NEG	POS	POS	NEG	NEG	POS	TSGH17	NEG	NEG
S71 (Thailand)	NEG	NEG	NEG	NEG	NEG	NEG	NEG	NEG	NEG	NEG	NEG	POS	POS	NEG	NEG	POS	TSGH17	NEG	NEG
S87 (Thailand)	NEG	NEG	NEG	NEG	NEG	NEG	NEG	NEG	NEG	NEG	NEG	POS	POS	NEG	NEG	POS	TSGH17	NEG	NEG
S93 (Thailand)	NEG	NEG	NEG	NEG	NEG	NEG	NEG	NEG	NEG	NEG	NEG	POS	POS	NEG	NEG	POS	TSGH17	NEG	NEG
US_002 (Thailand)	NEG	NEG	NEG	NEG	NEG	NEG	NEG	NEG	NEG	NEG	NEG	POS	POS	NEG	NEG	POS	TSGH17	NEG	NEG
US_017 (Thailand)	NEG	NEG	NEG	NEG	NEG	NEG	NEG	NEG	NEG	NEG	NEG	POS	POS	NEG	NEG	POS	TSGH17	NEG	NEG
WAMC6102 (Thailand)	NEG	NEG	NEG	NEG	NEG	NEG	NEG	NEG	NEG	NEG	NEG	POS	POS	NEG	NEG	POS	TSGH17	NEG	NEG
NCTC_13626_TW20 (UK)	NEG	NEG	NEG	NEG	NEG	NEG	NEG	NEG	NEG	NEG	NEG	POS	POS	NEG	NEG	POS	TSGH17	NEG	NEG
BSAC3021 (UK)	NEG	NEG	NEG	NEG	NEG	NEG	NEG	NEG	NEG	NEG	NEG	POS	POS	NEG	NEG	POS	TSGH17	NEG	NEG
BSAC3046 (UK)	NEG	NEG	NEG	NEG	NEG	NEG	NEG	NEG	NEG	NEG	NEG	POS	POS	NEG	NEG	POS	TSGH17	NEG	NEG
1193_SAUR (USA)	NEG	NEG	NEG	NEG	NEG	NEG	NEG	NEG	NEG	NEG	NEG	POS	POS	NEG	NEG	POS	TSGH17	NEG	NEG
1194_SAUR (USA)	NEG	NEG	NEG	NEG	NEG	NEG	NEG	NEG	NEG	NEG	NEG	POS	POS	NEG	NEG	POS	TSGH17	NEG	NEG

METHICILLIN RESISTANCE AND SCCmec TYPING

STRAIN / ISOLATE	ACME total	arcA-SCC	arcB-SCC	arcC-SCC	arcD-SCC	opp3B	opp3C	adhC	speG	ccrA/B-1	ccrA/B-2	ccrA-3	ccrB-3	ccrAA		ccrC		ccrA-4	ccrB-4
	ACME total	arcA-SCC	arcB-SCC	arcC-SCC	arcD-SCC	opp3B	opp3C (FPR3757)	adhC	speG (FPR3757)					ccrAA (MRSAZH47) probe 1	ccrAA (MRSAZH47) probe 2	ccrC	ccrC allele	ccrA-4	ccrB-4
	ACME total	Arginine deiminase	Ornithine carbamoyltransferase	Carbamate kinase	Arginine/ornithine antiporter	Oligopeptide permease, channel-forming protein	Oligopeptide permease, channel-forming protein	Alcohol dehydrogenase, zinc-containing	Spermidine N-acetyltransferase. Usually associated with ACME or composite SCCmec/ACME elements	Cassette chromosome recombinase A/B, type 1	Cassette chromosome recombinase A/B, type 2	Cassette chromosome recombinase A, type 3	Cassette chromosome recombinase B, type 3	Cassette chromosome recombinase homologue, accompanying ccrC		Cassette chromosome recombinase C		Cassette chromosome recombinase A/B, type 4	
>"South-East Asian Clade": TW20 (Irish AR44), c																			
1195_SAUR (USA)	NEG	NEG	NEG	NEG	NEG	NEG	NEG	NEG	NEG	NEG	NEG	POS	POS	NEG	NEG	POS	TSGH17	NEG	NEG
FVRH6002 (USA)	NEG	NEG	NEG	NEG	NEG	NEG	NEG	NEG	NEG	NEG	NEG	POS	POS	NEG	NEG	POS	TSGH17	NEG	NEG
KINW6048 (USA)	NEG	NEG	NEG	NEG	NEG	NEG	NEG	NEG	NEG	NEG	NEG	POS	POS	NEG	NEG	POS	TSGH17	NEG	NEG
LAMC0011 (USA)	NEG	NEG	NEG	NEG	NEG	NEG	NEG	NEG	NEG	NEG	NEG	POS	POS	NEG	NEG	POS	TSGH17	NEG	NEG
SJOS6053 (USA)	NEG	NEG	NEG	NEG	NEG	NEG	NEG	NEG	NEG	NEG	NEG	POS	POS	NEG	NEG	POS	TSGH17	NEG	NEG
SJOS6072 (USA)	NEG	NEG	NEG	NEG	NEG	NEG	NEG	NEG	NEG	NEG	NEG	POS	POS	NEG	NEG	POS	TSGH17	NEG	NEG
UCIM6042 (USA)	NEG	NEG	NEG	NEG	NEG	NEG	NEG	NEG	NEG	NEG	NEG	POS	POS	NEG	NEG	POS	TSGH17	NEG	NEG
Perth_2015-S31902	NEG	NEG	NEG	NEG	NEG	NEG	NEG	NEG	NEG	NEG	NEG	POS	POS	NEG	NEG	POS		NEG	NEG
Perth_2015-S31904	NEG	NEG	NEG	NEG	NEG	NEG	NEG	NEG	NEG	NEG	NEG	POS	POS	NEG	NEG	POS		NEG	NEG
Perth_2015-S31913	NEG	NEG	NEG	NEG	NEG	NEG	NEG	NEG	NEG	NEG	NEG	POS	POS	NEG	NEG	POS		NEG	NEG
Perth_2015-S31918	NEG	NEG	NEG	NEG	NEG	NEG	NEG	NEG	NEG	NEG	NEG	POS	POS	NEG	NEG	POS		NEG	NEG
Dublin-DSH_AR44_E1183	NEG	NEG	NEG	NEG	NEG	NEG	NEG	NEG	NEG	NEG	NEG	POS	POS	NEG	NEG	POS		NEG	NEG
Dublin-DSH_Unfamiliar-3_E1520	NEG	NEG	NEG	NEG	NEG	NEG	NEG	NEG	NEG	NEG	NEG	POS	POS	NEG	NEG	POS		NEG	NEG
Hong Kong_042	NEG	NEG	NEG	NEG	NEG	NEG	NEG	NEG	NEG	NEG	NEG	POS	POS	NEG	NEG	POS		NEG	NEG
Hong Kong_101	NEG	NEG	NEG	NEG	NEG	NEG	NEG	NEG	NEG	NEG	NEG	POS	POS	NEG	NEG	POS		NEG	NEG
Hong Kong_111	NEG	NEG	NEG	NEG	NEG	NEG	NEG	NEG	NEG	NEG	NEG	POS	POS	NEG	AMB	POS		NEG	
Hong Kong_113	NEG	NEG	NEG	NEG	NEG	NEG	NEG	NEG	NEG	NEG	NEG	POS	POS	NEG	NEG	POS		NEG	NEG
Hong Kong_115	NEG	NEG	NEG	NEG	NEG	NEG	NEG	NEG	NEG	NEG	NEG	POS	POS	NEG	NEG	POS		NEG	NEG
Hong Kong_118	NEG	NEG	NEG	NEG	NEG	NEG	NEG	NEG	NEG	NEG	NEG	POS	POS	NEG	NEG	POS		NEG	NEG
Hong Kong_125	NEG	NEG	NEG	NEG	NEG	NEG	NEG	NEG	NEG	NEG	NEG	POS	POS	NEG	AMB	POS		NEG	NEG
Hong Kong_129	NEG	NEG	NEG	NEG	NEG	NEG	NEG	NEG	NEG	NEG	NEG	POS	POS	NEG	NEG	POS		NEG	NEG
Hong Kong_133	NEG	NEG	NEG	NEG	NEG	NEG	NEG	NEG	NEG	NEG	NEG	POS	POS	NEG	NEG	POS		NEG	NEG
Kuwait_009	NEG	NEG	NEG	NEG	NEG	NEG	NEG	NEG	NEG	NEG	NEG	POS	POS	NEG	NEG	POS		NEG	NEG
Kuwait_103	NEG	NEG	NEG	NEG	POS	NEG	NEG	NEG	NEG	NEG	NEG	POS	POS	NEG	AMB	POS		NEG	NEG
Kuwait_107	NEG	NEG	NEG	NEG	POS	NEG	NEG	NEG	NEG	NEG	NEG	POS	POS	NEG	NEG	POS		NEG	NEG
Perth_03 AH 88	NEG	NEG	NEG	NEG	NEG	NEG	NEG	NEG	NEG	NEG	NEG	POS	POS	NEG	NEG	POS		NEG	NEG
Perth_08-16905	NEG	NEG	NEG	NEG	NEG	NEG	NEG	NEG	NEG	NEG	NEG	POS	POS	NEG	NEG	POS		NEG	NEG
Riyadh_Alfaisal-10_SICU_13_B5672_1117353	NEG	NEG	NEG	NEG	NEG	NEG	NEG	NEG	NEG	NEG	NEG	POS	POS	NEG	NEG	POS		NEG	NEG
Riyadh_SSTI_17_3508602	NEG	NEG	NEG	NEG	NEG	NEG	NEG	NEG	NEG	NEG	NEG	POS	POS	NEG	NEG	POS		NEG	NEG
Riyadh-2822088-R	NEG	NEG	NEG	NEG	NEG	NEG	NEG	NEG	NEG	NEG	NEG	POS	POS	NEG	NEG	POS		NEG	NEG
Trinidad&Tobago_2012_195 (619/12)	NEG	NEG	NEG	NEG	NEG	NEG	NEG	NEG	NEG	NEG	NEG	POS	POS	NEG	AMB	POS		NEG	NEG
Trinidad&Tobago_2012_370 (1112-52177)	NEG	NEG	NEG	NEG	NEG	NEG	NEG	NEG	NEG	NEG	NEG	POS	POS	NEG	AMB	POS		NEG	NEG
Trinidad&Tobago_2012_378 (4139)	NEG	NEG	NEG	NEG	NEG	NEG	NEG	NEG	NEG	NEG	NEG	POS	POS	NEG	AMB	POS		NEG	NEG
Trinidad&Tobago_2013_PA21	NEG	NEG	NEG	NEG	NEG	NEG	NEG	NEG	NEG	NEG	NEG	POS	POS	NEG	NEG	POS		NEG	NEG
Trinidad&Tobago_2013_PA22	NEG	NEG	NEG	NEG	NEG	NEG	NEG	NEG	NEG	NEG	NEG	POS	POS	NEG	AMB	POS		NEG	NEG
Trinidad&Tobago_2912_340 (8523/12)	NEG	NEG	NEG	NEG	NEG	NEG	NEG	NEG	NEG	NEG	NEG	POS	POS	NEG	NEG	POS		NEG	NEG
Trinidad&Tobago_MRSA2010_105	NEG	NEG	NEG	NEG	NEG	NEG	NEG	NEG	NEG	NEG	NEG	POS	POS	NEG	AMB	POS		NEG	NEG
Trinidad&Tobago_MRSA2010_108	NEG	NEG	NEG	NEG	NEG	NEG	NEG	NEG	NEG	NEG	NEG	POS	POS	NEG	POS	POS		NEG	NEG
Trinidad&Tobago_MRSA2010_33	NEG	NEG	NEG	neg	NEG	NEG	NEG	NEG	NEG	NEG	NEG	POS	POS	NEG	NEG	POS		NEG	NEG
Trinidad&Tobago_MRSA2010_79	NEG	NEG	NEG	NEG	NEG	NEG	NEG	NEG	NEG	NEG	NEG	POS	POS	NEG	NEG	POS		NEG	NEG
Trinidad&Tobago_MRSA2010_88	NEG	NEG	NEG	NEG	NEG	NEG	NEG	NEG	NEG	NEG	NEG	POS	POS	NEG	NEG	POS		NEG	NEG
Trinidad&Tobago_MRSA2010_97	NEG	NEG	NEG	NEG	NEG	NEG	NEG	NEG	NEG	NEG	NEG	POS	POS	NEG	AMB	POS		NEG	NEG
Trinidad&Tobago_SSTI 2012_031	NEG	NEG	NEG	NEG	NEG	NEG	NEG	NEG	NEG	NEG	NEG	POS	POS	NEG	NEG	POS		NEG	NEG
Uganda-29_17148_250811	NEG	NEG	NEG	NEG	NEG	NEG	NEG	NEG	NEG	NEG	NEG	POS	POS	NEG	NEG	POS		NEG	NEG
>"South-East Asian Clad																			
UCIM6015	NEG	NEG	NEG	NEG	NEG	NEG	NEG	NEG	NEG	NEG	NEG	NEG	NEG	NEG	NEG	POS	TSGH17	NEG	NEG
Hong Kong_121	NEG	NEG	NEG	NEG	NEG	NEG	NEG	NEG	NEG	NEG	NEG	NEG	NEG	NEG	NEG	POS		NEG	NEG
>"South-East Asian Clad																			
Z172 (Taiwan)	NEG	NEG	NEG	NEG	NEG	NEG	NEG	NEG	NEG	NEG	NEG	POS	POS	NEG	NEG	POS	TSGH17	NEG	NEG
Russia-19_0342_Kurgan	NEG	NEG	NEG	NEG	NEG	NEG	NEG	NEG	NEG	NEG	NEG	POS	POS	NEG	NEG	POS		NEG	NEG
>"South-East Asian Clad																			
Perth_01-15392	NEG	NEG	NEG	NEG	NEG	NEG	NEG	NEG	NEG	NEG	NEG	POS	POS	NEG	NEG	POS		NEG	NEG
>"South-East Asian Clad																			
XN108 (China)	NEG	NEG	NEG	NEG	NEG	NEG	NEG	NEG	NEG	NEG	NEG	POS	POS	NEG	NEG	POS	TSGH17	NEG	NEG
NMR09 (India)	NEG	NEG	NEG	NEG	NEG	NEG	NEG	NEG	NEG	NEG	NEG	POS	POS	NEG	NEG	POS	TSGH17	NEG	NEG
346 (Malaysia)	NEG	NEG	NEG	NEG	NEG	NEG	NEG	NEG	NEG	NEG	NEG	POS	POS	NEG	NEG	POS	TSGH17	NEG	NEG
DS_009 (Thailand)	NEG	NEG	NEG	NEG	NEG	NEG	NEG	NEG	NEG	NEG	NEG	POS	POS	NEG	NEG	POS	TSGH17	NEG	NEG
18ANR581005 (Ind)	NEG	NEG	NEG	NEG	NEG	NEG	NEG	NEG	NEG	NEG	NEG	POS	POS	NEG	NEG	POS		NEG	NEG
Bengalaru_ST20141405	NEG	NEG	NEG	NEG	NEG	NEG	NEG	NEG	NEG	NEG	NEG	POS	POS	NEG	NEG	POS		NEG	NEG
Bengalaru_ST20141406	NEG	NEG	NEG	NEG	NEG	NEG	NEG	NEG	NEG	NEG	NEG	POS	POS	NEG	NEG	POS		NEG	NEG
Bengalaru_ST20141417	NEG	NEG	NEG	NEG	NEG	NEG	NEG	NEG	NEG	NEG	NEG	POS	POS	NEG	NEG	POS		NEG	NEG
Hong Kong_107	NEG	NEG	NEG	NEG	NEG	NEG	NEG	NEG	NEG	NEG	NEG	POS	POS	NEG	NEG	POS		NEG	NEG
Hong Kong_12	NEG	NEG	NEG	NEG	NEG	NEG	NEG	NEG	NEG	NEG	NEG	POS	POS	NEG	AMB	POS		NEG	NEG
Hong Kong_132	NEG	NEG	NEG	NEG	NEG	NEG	NEG	NEG	NEG	NEG	NEG	POS	POS	NEG	NEG	POS		NEG	neg
Riyadh-3028763-R	NEG	NEG	NEG	NEG	NEG	NEG	NEG	NEG	NEG	NEG	NEG	POS	POS	NEG	NEG	POS		NEG	NEG
Trinidad&Tobago_MRSA2010_94	NEG	NEG	NEG	NEG	NEG	NEG	NEG	NEG	NEG	NEG	NEG	POS	POS	NEG	NEG	POS		NEG	NEG
>"South-East Asian Clad																			
M705 (Thailand)	NEG	NEG	NEG	NEG	NEG	NEG	NEG	NEG	NEG	NEG	NEG	POS	POS	NEG	NEG	POS	TSGH17	NEG	NEG
S85 (Thailand)	NEG	NEG	NEG	NEG	NEG	NEG	NEG	NEG	NEG	NEG	NEG	POS	POS	NEG	NEG	POS	TSGH17	NEG	NEG
Riyadh_Alfaisal-04_281014_S5076	NEG	NEG	NEG	NEG	NEG	NEG	NEG	NEG	NEG	NEG	NEG	POS	POS	NEG	POS	POS		NEG	NEG
Riyadh_Alfaisal-26_S15145_1104163	NEG	NEG	NEG	NEG	NEG	NEG	NEG	NEG	NEG	NEG	NEG	POS	POS	NEG	NEG	POS		NEG	NEG
Riyadh-2817437-W	NEG	NEG	NEG	NEG	NEG	NEG	NEG	NEG	NEG	NEG	NEG	POS	POS	NEG	AMB	POS		NEG	NEG
>"South-East Asian Clad																			
PPUKM-775-2009 (Malaysia)	NEG	NEG	NEG	NEG	NEG	NEG	NEG	NEG	NEG	NEG	NEG	POS	POS	NEG	NEG	POS	TSGH17	NEG	NEG
Hong Kong_138	NEG	NEG	NEG	NEG	NEG	NEG	NEG	NEG	NEG	NEG	NEG	POS	POS	NEG	NEG	POS		NEG	NEG
>"South-East Asian Clad																			
Kuwait_001	NEG	NEG	NEG	NEG	NEG	NEG	NEG	NEG	NEG	NEG	NEG	POS	POS	NEG	NEG	NEG		NEG	NEG
Kuwait_337	NEG	NEG	NEG	NEG	POS	NEG	NEG	NEG	NEG	NEG	AMB	POS	POS	NEG	NEG	NEG		NEG	NEG
>"South-East Asian Clad																			
CUHK_HK2007 (China)	NEG	NEG	NEG	NEG	NEG	NEG	NEG	NEG	NEG	NEG	NEG	POS	POS	NEG	NEG	POS	TSGH17	NEG	NEG
NMR02 (India)	NEG	NEG	NEG	NEG	NEG	NEG	NEG	NEG	NEG	NEG	NEG	POS	POS	NEG	NEG	POS	TSGH17	NEG	NEG
VB1490 (India)	NEG	NEG	NEG	NEG	NEG	NEG	NEG	NEG	NEG	NEG	NEG	POS	POS	NEG	NEG	POS	TSGH17	NEG	NEG
V521 (Korea)	NEG	NEG	NEG	NEG	NEG	NEG	NEG	NEG	NEG	NEG	NEG	POS	POS	NEG	NEG	POS	TSGH17	NEG	NEG
HST-077 (Lebanon)	NEG	NEG	NEG	NEG	NEG	NEG	NEG	NEG	NEG	NEG	NEG	POS	POS	NEG	NEG	POS	TSGH17	NEG	NEG
MAL1 (Malaysia)	NEG	NEG	NEG	NEG	NEG	NEG	NEG	NEG	NEG	NEG	NEG	POS	POS	NEG	NEG	POS	TSGH17	NEG	NEG
MAL3 (Malaysia)	NEG	NEG	NEG	NEG	NEG	NEG	NEG	NEG	NEG	NEG	NEG	POS	POS	NEG	NEG	POS	TSGH17	NEG	NEG
PPUKM-261-2009 (Malaysia)	NEG	NEG	NEG	NEG	NEG	NEG	NEG	NEG	NEG	NEG	NEG	POS	POS	NEG	NEG	POS	TSGH17	NEG	NEG
PPUKM-332-2009 (Malaysia)	NEG	NEG	NEG	NEG	NEG	NEG	NEG	NEG	NEG	NEG	NEG	POS	POS	NEG	NEG	POS	TSGH17	NEG	NEG
S38 (Thailand)	NEG	NEG	NEG	NEG	NEG	NEG	NEG	NEG	NEG	NEG	NEG	POS	POS	NEG	NEG	POS	TSGH17	NEG	NEG
S7 (Thailand)	NEG	NEG	NEG	NEG	NEG	NEG	NEG	NEG	NEG	NEG	NEG	POS	POS	NEG	NEG	POS	TSGH17	NEG	NEG
US_008 (Thailand)	NEG	NEG	NEG	NEG	NEG	NEG	NEG	NEG	NEG	NEG	NEG	POS	POS	NEG	NEG	POS	TSGH17	NEG	NEG
US_030 (Thailand)	NEG	NEG	NEG	NEG	NEG	NEG	NEG	NEG	NEG	NEG	NEG	POS	POS	NEG	NEG	POS	TSGH17	NEG	NEG
M170 (Tsunami-related)	NEG	NEG	NEG	NEG	NEG	NEG	NEG	NEG	NEG	NEG	NEG	POS	POS	NEG	NEG	POS	TSGH17	NEG	NEG
BSAC697 (UK)	NEG	NEG	NEG	NEG	NEG	NEG	NEG	NEG	NEG	NEG	NEG	POS	POS	NEG	NEG	POS	TSGH17	NEG	NEG
IS-189	NEG	NEG	NEG	NEG	NEG	NEG	NEG	NEG	NEG	NEG	NEG	POS	POS	NEG	NEG	POS	TSGH17	NEG	NEG
MRGR3	NEG	NEG	NEG	NEG	NEG	NEG	NEG	NEG	NEG	NEG	NEG	POS	POS	NEG	NEG	POS	TSGH17	NEG	NEG
smg07	NEG	NEG	NEG	NEG	NEG	NEG	NEG	NEG	NEG	NEG	NEG	POS	POS	NEG	NEG	POS	TSGH17	NEG	NEG
AH1-AUS-EMRSA-3	NEG	NEG	NEG	NEG	NEG	NEG	NEG	NEG	NEG	NEG	NEG	POS	POS	NEG	NEG	POS		NEG	NEG
Bengalaru_ST20141403	NEG	NEG	NEG	NEG	NEG	NEG	NEG	NEG	NEG	NEG	NEG	POS	POS	NEG	NEG	POS		NEG	NEG
Finland E24_98541_Harmony	NEG	NEG	NEG	NEG	NEG	NEG	NEG	NEG	NEG	NEG	NEG	POS	POS	NEG	NEG	POS		NEG	NEG
Hong Kong_10	NEG	NEG	NEG	NEG	NEG	NEG	NEG	NEG	NEG	NEG	NEG	POS	POS	NEG	AMB	POS		NEG	NEG
Hong Kong_100	NEG	NEG	NEG	NEG	NEG	NEG	NEG	NEG	NEG	NEG	NEG	POS	POS	NEG	NEG	POS		NEG	NEG
Hong Kong_199	NEG	NEG	NEG	NEG	NEG	NEG	NEG	NEG	NEG	NEG	NEG	POS	POS	NEG	POS	POS		NEG	NEG
Hong Kong_34	NEG	NEG	NEG	neg	NEG	NEG	NEG	NEG	NEG	NEG	NEG	POS	POS	NEG	NEG	POS		NEG	NEG
Hong Kong_88	NEG	NEG	NEG	NEG	NEG	NEG	NEG	NEG	NEG	NEG	NEG	POS	POS	NEG	POS	POS		NEG	NEG
Hong Kong_92	NEG	NEG	NEG	NEG	NEG	NEG	NEG	NEG	NEG	NEG	NEG	POS	POS	NEG	AMB	POS		NEG	NEG
Hong Kong_94	NEG	NEG	NEG	NEG	NEG	NEG	NEG	NEG	NEG	NEG	NEG	POS	POS	NEG	NEG	POS		NEG	NEG
Kuwait_257	NEG	NEG	NEG	NEG	NEG	NEG	NEG	NEG	NEG	NEG	NEG	POS	POS	NEG	NEG	POS		NEG	NEG
Perth_01-15330	NEG	NEG	NEG	NEG	NEG	NEG	NEG	NEG	NEG	NEG	NEG	POS	POS	NEG	NEG	POS		NEG	NEG
Perth_01-15349	NEG	NEG	NEG	NEG	NEG	NEG	NEG	NEG	NEG	NEG	NEG	POS	POS	NEG	NEG	POS		NEG	NEG
Perth_01-16176	NEG	NEG	NEG	NEG	NEG	NEG	NEG	NEG	NEG	NEG	NEG	POS	POS	NEG	NEG	POS		NEG	NEG
Perth_WBG8379	NEG	NEG	NEG	NEG	NEG	NEG	NEG	NEG	NEG	NEG	NEG	POS	POS	NEG	NEG	POS		NEG	NEG
Rawalpindi_Kidney Center_56	NEG	NEG	NEG	NEG	NEG	NEG	NEG	NEG	NEG	NEG	NEG	POS	POS	NEG	NEG	POS		NEG	NEG
Riyadh_SSTI_05_3464614	NEG	NEG	NEG	NEG	NEG	NEG	NEG	NEG	NEG	NEG	NEG	POS	POS	NEG	NEG	POS		NEG	NEG
Trinidad&Tobago_2012_157	NEG	NEG	NEG	NEG	NEG	NEG	NEG	NEG	NEG	NEG	NEG	POS	POS	NEG	AMB	POS		NEG	NEG

STRAIN / ISOLATE	ACME total	arcA-SCC	arcB-SCC	arcC-SCC	arcD-SCC	opp3B	opp3C	adhC	speG	ccrA/B-1	ccrA/B-2	ccrA-3	ccrB-3	ccrAA		ccrC		ccrA-4	ccrB-4
METHICILLIN RESISTANCE AND SCCmec TYPING																			
	ACME total	arcA-SCC	arcB-SCC	arcC-SCC	arcD-SCC	opp3B	opp3C (FPR3757)	adhC	speG (FPR3757)					ccrAA (MRSAZH47) probe 1	ccrAA (MRSAZH47) probe 2	ccrC	ccrC allele	ccrA-4	ccrB-4
	ACME total	Arginine deiminase	Ornithine carbamoyltransferase	Carbamate kinase	Arginine/ornithine antiporter	Oligopeptide permease, channel-forming protein	Oligopeptide permease, channel-forming protein	Alcohol dehydrogenase, zinc-containing	Spermidine N-acetyltransferase. Usually associated with ACME or composite SCCmec/ACME elements	Cassette chromosome recombinase A/B, type 1	Cassette chromosome recombinase A/B, type 2	Cassette chromosome recombinase A, type 3	Cassette chromosome recombinase B, type 3	Cassette chromosome recombinase homologue, accompanying ccrC		Cassette chromosome recombinase C		Cassette chromosome recombinase A/B, type 4	
>"South-East Asian Clad																			
M418	NEG	NEG	NEG	NEG	NEG	NEG	NEG	NEG	NEG	NEG	NEG	POS	POS	NEG	NEG	POS	TSGH17	NEG	NEG
>"South-East Asian Clad																			
NMR07 (India)	NEG	NEG	NEG	NEG	NEG	NEG	NEG	NEG	NEG	NEG	NEG	POS	POS	NEG	NEG	POS	TSGH17	NEG	NEG
NMR08 (India)	NEG	NEG	NEG	NEG	NEG	NEG	NEG	NEG	NEG	NEG	NEG	POS	POS	NEG	NEG	POS	TSGH17	NEG	NEG
Bengalaru_ST20121978	NEG	NEG	NEG	NEG	NEG	NEG	NEG	NEG	NEG	NEG	NEG	POS	POS	NEG	AMB	POS		NEG	NEG
Hong Kong_89	NEG	NEG	NEG	NEG	NEG	NEG	NEG	NEG	NEG	NEG	NEG	POS	POS	NEG	POS	POS		NEG	NEG
>"South-East Asian Clad																			
Perth_2005_AH_5	POS	POS	POS	POS	AMB	NEG	NEG	NEG	NEG	NEG	NEG	POS	POS	NEG	NEG	NEG		NEG	NEG
>"South-East Asian Clad																			
Trinidad&Tobago_MRSA2010_103	NEG	NEG	NEG	NEG	NEG	NEG	NEG	NEG	POS	NEG	NEG	POS	POS	NEG	AMB	POS		AMB	POS
Trinidad&Tobago_2013_PA25	NEG	NEG	NEG	NEG	NEG	NEG	NEG	NEG	POS	NEG	NEG	POS	POS	NEG	NEG	POS		POS	POS
Trinidad&Tobago_MRSA2010_110	NEG	NEG	NEG	neg	NEG	NEG	NEG	NEG	POS	NEG	NEG	POS	POS	NEG	AMB	POS		POS	POS
Trinidad&Tobago_SSTI 2012_111	NEG	NEG	NEG	NEG	NEG	NEG	NEG	NEG	POS	NEG	NEG	POS	POS	NEG	NEG	POS		POS	POS
Trinidad&Tobago_MRSA2010_158	NEG	NEG	NEG	NEG	NEG	NEG	NEG	NEG	POS	NEG	NEG	POS	POS	NEG	NEG	POS		NEG	POS
>"South-East Asian Clad																			
Kuwait_122	NEG	NEG	NEG	NEG	NEG	NEG	NEG	NEG	NEG	NEG	NEG	POS	POS	NEG	NEG	NEG		NEG	NEG
Kuwait_002	NEG	NEG	NEG	NEG	NEG	NEG	NEG	NEG	NEG	NEG	NEG	POS	POS	NEG	NEG	NEG		NEG	NEG
Kuwait_196	NEG	NEG	NEG	NEG	NEG	NEG	NEG	NEG	NEG	NEG	NEG	POS	POS	NEG	NEG	NEG		NEG	NEG
Kuwait_199	NEG	NEG	NEG	NEG	NEG	NEG	NEG	NEG	NEG	NEG	NEG	POS	POS	NEG	NEG	NEG		NEG	NEG
Hong Kong_136	NEG	NEG	NEG	NEG	NEG	NEG	NEG	NEG	NEG	NEG	NEG	POS	POS	NEG	NEG	NEG		NEG	NEG
>"South-East Asian Clad																			
P32 (Poland)	NEG	NEG	NEG	NEG	NEG	NEG	NEG	NEG	NEG	NEG	NEG	POS	POS	NEG	NEG	NEG	0	NEG	NEG
>"South-East Asian Clad																			
Perth_2015-S32313	POS	POS	POS	POS	POS	NEG	NEG	NEG	NEG	NEG	NEG	POS	POS	NEG	NEG	NEG		NEG	NEG
>"hla-negative South-Ea																			
DEN907 (Denmark)	NEG	NEG	NEG	NEG	NEG	NEG	NEG	NEG	NEG	NEG	NEG	POS	POS	NEG	NEG	NEG	0	NEG	NEG
>"hla-negative South-Ea																			
S26 (Thailand)	NEG	NEG	NEG	NEG	NEG	NEG	NEG	NEG	NEG	NEG	NEG	POS	POS	NEG	NEG	NEG	0	NEG	NEG
S42 (Thailand)	NEG	NEG	NEG	NEG	NEG	NEG	NEG	NEG	NEG	NEG	NEG	POS	POS	NEG	NEG	NEG	0	NEG	NEG
S97 (Thailand)	NEG	NEG	NEG	NEG	NEG	NEG	NEG	NEG	NEG	NEG	NEG	POS	POS	NEG	NEG	NEG	0	NEG	NEG
S78 (Thailand)	NEG	NEG	NEG	NEG	NEG	NEG	NEG	NEG	NEG	NEG	NEG	POS	POS	NEG	NEG	NEG	0	NEG	NEG
S25 (Thailand)	NEG	NEG	NEG	NEG	NEG	NEG	NEG	NEG	NEG	NEG	NEG	POS	POS	NEG	NEG	NEG	0	NEG	NEG
S21 (Thailand)	NEG	NEG	NEG	NEG	NEG	NEG	NEG	NEG	NEG	NEG	NEG	POS	POS	NEG	NEG	NEG	0	NEG	NEG
S106 (Thailand)	NEG	NEG	NEG	NEG	NEG	NEG	NEG	NEG	NEG	NEG	NEG	POS	POS	NEG	NEG	NEG	0	NEG	NEG
S24 (Thailand)	NEG	NEG	NEG	NEG	NEG	NEG	NEG	NEG	NEG	NEG	NEG	POS	POS	NEG	NEG	NEG	0	NEG	NEG
S39 (Thailand)	NEG	NEG	NEG	NEG	NEG	NEG	NEG	NEG	NEG	NEG	NEG	POS	POS	NEG	NEG	NEG	0	NEG	NEG
S2 (Thailand)	NEG	NEG	NEG	NEG	NEG	NEG	NEG	NEG	NEG	NEG	NEG	POS	POS	NEG	NEG	NEG	0	NEG	NEG
S81 (Thailand)	NEG	NEG	NEG	NEG	NEG	NEG	NEG	NEG	NEG	NEG	NEG	POS	POS	NEG	NEG	NEG	0	NEG	NEG
M116 (Vietnam)	NEG	NEG	NEG	NEG	NEG	NEG	NEG	NEG	NEG	NEG	NEG	POS	POS	NEG	NEG	NEG	0	NEG	NEG
>"Portuguese Clade": Pc																			
FFP103 (Portugal)	NEG	NEG	NEG	NEG	NEG	NEG	NEG	NEG	NEG	NEG	NEG	POS	POS	NEG	NEG	NEG	0	NEG	NEG
HDG2 (Portugal)	NEG	NEG	NEG	NEG	NEG	NEG	NEG	NEG	NEG	NEG	NEG	POS	POS	NEG	NEG	NEG	0	NEG	NEG
HSA10 (Portugal)	NEG	NEG	NEG	NEG	NEG	NEG	NEG	NEG	NEG	NEG	NEG	POS	POS	NEG	NEG	NEG	0	NEG	NEG
HSA11 (Portugal)	NEG	NEG	NEG	NEG	NEG	NEG	NEG	NEG	NEG	NEG	NEG	POS	POS	NEG	NEG	NEG	0	NEG	NEG
ICP5011 (Portugal)	NEG	NEG	NEG	NEG	NEG	NEG	NEG	NEG	NEG	NEG	NEG	POS	POS	NEG	NEG	NEG	0	NEG	NEG
ICP5014 (Portugal)	NEG	NEG	NEG	NEG	NEG	NEG	NEG	NEG	NEG	NEG	NEG	POS	POS	NEG	NEG	NEG	0	NEG	NEG
ICP5062 (Portugal)	NEG	NEG	NEG	NEG	NEG	NEG	NEG	NEG	NEG	NEG	NEG	POS	POS	NEG	NEG	NEG	0	NEG	NEG
Strain 1063_ATCC 33592 FDAARGOS_3 (USA)	NEG	NEG	NEG	NEG	NEG	NEG	NEG	NEG	NEG	NEG	NEG	POS	POS	NEG	NEG	NEG	0	NEG	NEG
	NEG	NEG	NEG	neg	NEG	NEG	NEG	NEG	NEG	NEG	NEG	POS	POS	NEG	NEG	NEG		NEG	NEG
Russia-07_0110_Kurgan	NEG	NEG	NEG	NEG	NEG	NEG	NEG	NEG	NEG	NEG	NEG	POS	POS	NEG	NEG	NEG		NEG	NEG
Russia-11_0164_Kurgan	NEG	NEG	NEG	NEG	NEG	NEG	NEG	NEG	NEG	NEG	NEG	POS	POS	NEG	NEG	NEG		NEG	NEG
Russia-20_0367_Kurgan	NEG	NEG	NEG	NEG	NEG	NEG	NEG	NEG	NEG	NEG	NEG	POS	POS	NEG	NEG	NEG		NEG	NEG
Russia-21_0390_Chelyabinsk	NEG	NEG	NEG	NEG	NEG	NEG	NEG	NEG	NEG	NEG	NEG	POS	POS	NEG	NEG	NEG		NEG	NEG
>"South American/ Mid																			
AGT1 (Argentina)	NEG	NEG	NEG	NEG	NEG	NEG	NEG	NEG	NEG	NEG	NEG	POS	POS	NEG	NEG	POS	TSGH17	NEG	NEG
AGT120 (Argentina)	NEG	NEG	NEG	NEG	NEG	NEG	NEG	NEG	NEG	NEG	NEG	POS	POS	NEG	NEG	POS	TSGH17	NEG	NEG
AGT67 (Argentina)	NEG	NEG	NEG	NEG	NEG	NEG	NEG	NEG	NEG	NEG	NEG	POS	POS	NEG	NEG	POS	TSGH17	NEG	NEG
AGT9 (Argentina)	NEG	NEG	NEG	NEG	NEG	NEG	NEG	NEG	NEG	NEG	NEG	POS	POS	NEG	NEG	POS	TSGH17	NEG	NEG
RA3 (Argentina)	NEG	NEG	NEG	NEG	NEG	NEG	NEG	NEG	NEG	NEG	NEG	POS	POS	NEG	NEG	POS	TSGH17	NEG	NEG
RA6 (Argentina)	NEG	NEG	NEG	NEG	NEG	NEG	NEG	NEG	NEG	NEG	NEG	POS	POS	NEG	NEG	POS	TSGH17	NEG	NEG
RA7 (Argentina)	NEG	NEG	NEG	NEG	NEG	NEG	NEG	NEG	NEG	NEG	NEG	POS	POS	NEG	NEG	POS	TSGH17	NEG	NEG
Be62 (Brazil)	NEG	NEG	NEG	NEG	NEG	NEG	NEG	NEG	NEG	NEG	NEG	POS	POS	NEG	NEG	POS	TSGH17	NEG	NEG
Bmb9393 (Brazil)	NEG	NEG	NEG	NEG	NEG	NEG	NEG	NEG	NEG	NEG	NEG	POS	POS	NEG	NEG	POS	TSGH17	NEG	NEG
BRA36 (Brazil)	NEG	NEG	NEG	NEG	NEG	NEG	NEG	NEG	NEG	NEG	NEG	POS	POS	NEG	NEG	POS	TSGH17	NEG	NEG
BZ48 (Brazil)	NEG	NEG	NEG	NEG	NEG	NEG	NEG	NEG	NEG	NEG	NEG	POS	POS	NEG	NEG	POS	TSGH17	NEG	NEG
Gv51 (Brazil)	NEG	NEG	NEG	NEG	NEG	NEG	NEG	NEG	NEG	NEG	NEG	POS	POS	NEG	NEG	POS	TSGH17	NEG	NEG
Gv69 (Brazil)	NEG	NEG	NEG	NEG	NEG	NEG	NEG	NEG	NEG	NEG	NEG	POS	POS	NEG	NEG	POS	TSGH17	NEG	NEG
Gv88 (Brazil)	NEG	NEG	NEG	NEG	NEG	NEG	NEG	NEG	NEG	NEG	NEG	POS	POS	NEG	NEG	POS	TSGH17	NEG	NEG
HC1335 (Brazil)	NEG	NEG	NEG	NEG	NEG	NEG	NEG	NEG	NEG	NEG	NEG	POS	POS	NEG	NEG	POS	TSGH17	NEG	NEG
HC1340 (Brazil)	NEG	NEG	NEG	NEG	NEG	NEG	NEG	NEG	NEG	NEG	NEG	POS	POS	NEG	NEG	POS	TSGH17	NEG	NEG
HC556 (Brazil)	NEG	NEG	NEG	NEG	NEG	NEG	NEG	NEG	NEG	NEG	NEG	POS	POS	NEG	NEG	POS	TSGH17	NEG	NEG
HU25 (Brazil)	NEG	NEG	NEG	NEG	NEG	NEG	NEG	NEG	NEG	NEG	NEG	POS	POS	NEG	NEG	POS	TSGH17	NEG	NEG
UB563 (Brazil)	NEG	NEG	NEG	NEG	NEG	NEG	NEG	NEG	NEG	NEG	NEG	POS	POS	NEG	NEG	POS	TSGH17	NEG	NEG
2A8 (Czech Republic)	NEG	NEG	NEG	NEG	NEG	NEG	NEG	NEG	NEG	NEG	NEG	POS	POS	NEG	NEG	POS	TSGH17	NEG	NEG
M1229 (Denmark)	NEG	NEG	NEG	NEG	NEG	NEG	NEG	NEG	NEG	NEG	NEG	POS	POS	NEG	NEG	POS	TSGH17	NEG	NEG
FRICAR (France)	NEG	NEG	NEG	NEG	NEG	NEG	NEG	NEG	NEG	NEG	NEG	POS	POS	NEG	NEG	POS	TSGH17	NEG	NEG
D90 (Germany)	NEG	NEG	NEG	NEG	NEG	NEG	NEG	NEG	NEG	NEG	NEG	POS	POS	NEG	NEG	POS	TSGH17	NEG	NEG
LIT68 (Lithuania)	NEG	NEG	NEG	NEG	NEG	NEG	NEG	NEG	NEG	NEG	NEG	POS	POS	NEG	NEG	POS	TSGH17	NEG	NEG
LIT76 (Lithuania)	NEG	NEG	NEG	NEG	NEG	NEG	NEG	NEG	NEG	NEG	NEG	POS	POS	NEG	NEG	POS	TSGH17	NEG	NEG
HGSA142 (Portugal)	NEG	NEG	NEG	NEG	NEG	NEG	NEG	NEG	NEG	NEG	NEG	POS	POS	NEG	NEG	POS	TSGH17	NEG	NEG
HGSA9 (Portugal)	NEG	NEG	NEG	NEG	NEG	NEG	NEG	NEG	NEG	NEG	NEG	POS	POS	NEG	NEG	POS	TSGH17	NEG	NEG
HSJ216 (Portugal)	NEG	NEG	NEG	NEG	NEG	NEG	NEG	NEG	NEG	NEG	NEG	POS	POS	NEG	NEG	POS	TSGH17	NEG	NEG
M278 (Portugal)	NEG	NEG	NEG	NEG	NEG	NEG	NEG	NEG	NEG	NEG	NEG	POS	POS	NEG	NEG	POS	TSGH17	NEG	NEG
ES26 (Spain)	NEG	NEG	NEG	NEG	NEG	NEG	NEG	NEG	NEG	NEG	NEG	POS	POS	NEG	NEG	POS	TSGH17	NEG	NEG
UK102 (UK)	NEG	NEG	NEG	NEG	NEG	NEG	NEG	NEG	NEG	NEG	NEG	POS	POS	NEG	NEG	POS	TSGH17	NEG	NEG
URU34 (Uruguay)	NEG	NEG	NEG	NEG	NEG	NEG	NEG	NEG	NEG	NEG	NEG	POS	POS	NEG	NEG	POS		NEG	NEG
IS-125	NEG	NEG	NEG	NEG	NEG	NEG	NEG	NEG	NEG	NEG	NEG	POS	POS	NEG	NEG	POS	TSGH17	NEG	NEG
IS-157	NEG	NEG	NEG	NEG	NEG	NEG	NEG	NEG	NEG	NEG	NEG	POS	POS	NEG	NEG	POS	TSGH17	NEG	NEG
Ecuador_1	NEG	NEG	NEG	NEG	NEG	NEG	NEG	NEG	NEG	NEG	NEG	POS	POS	NEG	NEG	POS		NEG	NEG
Ecuador_2	NEG	NEG	NEG	NEG	NEG	NEG	NEG	NEG	NEG	NEG	NEG	POS	POS	NEG	NEG	POS		NEG	NEG
Dresden_15ANR574263	NEG	NEG	NEG	NEG	NEG	NEG	NEG	NEG	NEG	NEG	NEG	POS	POS	NEG	NEG	POS		NEG	NEG
UK-EMRSA-11	NEG	NEG	NEG	NEG	NEG	NEG	NEG	NEG	NEG	NEG	NEG	POS	POS	NEG	NEG	POS		NEG	NEG
>"South American/ Mid																			
BRA2 (Brazil)	NEG	NEG	NEG	NEG	NEG	NEG	NEG	NEG	NEG	NEG	NEG	POS	POS	NEG	NEG	NEG		NEG	NEG
CHL1 (Chile)	NEG	NEG	NEG	NEG	NEG	NEG	NEG	NEG	NEG	NEG	NEG	POS	POS	NEG	NEG	NEG		NEG	NEG
CHL151 (Chile)	NEG	NEG	NEG	NEG	NEG	NEG	NEG	NEG	NEG	NEG	NEG	POS	POS	NEG	NEG	NEG		NEG	NEG
UCL378 (Chile)	NEG	NEG	NEG	NEG	NEG	NEG	NEG	NEG	NEG	NEG	NEG	POS	POS	NEG	NEG	NEG		NEG	NEG
UP72 (Peru)	NEG	NEG	NEG	NEG	NEG	NEG	NEG	NEG	NEG	NEG	NEG	POS	POS	NEG	NEG	NEG		NEG	NEG
UP81 (Peru)	NEG	NEG	NEG	NEG	NEG	NEG	NEG	NEG	NEG	NEG	NEG	POS	POS	NEG	NEG	NEG		NEG	NEG
>"South American/ Mid																			
UP1073 (Peru)	NEG	NEG	NEG	NEG	NEG	NEG	NEG	NEG	NEG	NEG	NEG	NEG	NEG	NEG	NEG	NEG		NEG	NEG

STRAIN / ISOLATE	METHICILLIN RESISTANCE AND SCCmec TYPING																		
	ACME total	arcA-SCC	arcB-SCC	arcC-SCC	arcD-SCC	opp3B	opp3C	adhC	speG	ccrA/B-1	ccrA/B-2	ccrA-3	ccrB-3	ccrAA		ccrC		ccrA-4	ccrB-4
	ACME total	arcA-SCC	arcB-SCC	arcC-SCC	arcD-SCC	opp3B	opp3C (FPR3757)	adhC	speG (FPR3757)					ccrAA (MRSAZH47) probe 1	ccrAA (MRSAZH47) probe 2	ccrC	ccrC allele	ccrA-4	ccrB-4
	ACME total	Arginine deiminase	Ornithine carbamoyltransferase	Carbamate kinase	Arginine/ornithine antiporter	Oligopeptide permease, channel-forming protein	Oligopeptide permease, channel-forming protein	Alcohol dehydrogenase, zinc-containing	Spermidine N-acetyltransferase. Usually associated with ACME or composite SCCmec/ACME elements	Cassette chromosome recombinase A/B, type 1	Cassette chromosome recombinase A/B, type 2	Cassette chromosome recombinase A, type 3	Cassette chromosome recombinase B, type 3	Cassette chromosome recombinase homologue, accompanying ccrC		Cassette chromosome recombinase C		Cassette chromosome recombinase A/B, type 4	
>"South American/ Mid																			
LIT89 (Lithuania)	NEG	NEG	NEG	NEG	NEG	NEG	NEG	NEG	NEG	NEG	NEG	POS	POS	NEG	NEG	POS	TSGH17	NEG	NEG
MRSA_PR1 (Malaysia)	NEG	NEG	NEG	NEG	NEG	NEG	NEG	NEG	NEG	NEG	NEG	POS	POS	NEG	NEG	POS	TSGH17	NEG	NEG
Riyadh-2888905-R	NEG	NEG	NEG	NEG	NEG	NEG	NEG	NEG	NEG	NEG	NEG	POS	POS	NEG	NEG	POS		NEG	NEG
Riyadh-2888915-BC	NEG	NEG	NEG	NEG	NEG	NEG	NEG	NEG	NEG	NEG	NEG	POS	POS	NEG	NEG	POS		NEG	NEG
Riyadh-2793706-R	NEG	NEG	NEG	NEG	NEG	NEG	NEG	NEG	NEG	NEG	NEG	POS	POS	NEG	NEG	POS		NEG	NEG
>"South American/ Mid																			
Riyadh_SSTI_18_3502925	NEG	NEG	NEG	NEG	NEG	NEG	NEG	NEG	NEG	NEG	NEG	POS	POS	NEG	POS	POS		NEG	NEG
Riyadh-2822825-W	NEG	NEG	NEG	NEG	NEG	NEG	NEG	NEG	NEG	NEG	NEG	POS	POS	NEG	AMB	POS		NEG	NEG
>"South American/ Mid																			
UK-EMRSA-9	NEG	NEG	NEG	neg	NEG	NEG	NEG	NEG	NEG	NEG	NEG	POS	POS	NEG	NEG	POS		NEG	NEG
>"South American/ Mid																			
Lome_HT20020815	NEG	NEG	NEG	NEG	NEG	NEG	NEG	NEG	NEG	NEG	NEG	POS	POS	NEG	NEG	NEG		NEG	NEG
>"South American/ Mid																			
NA32 (Denmark)	NEG	NEG	NEG	NEG	NEG	NEG	NEG	NEG	NEG	NEG	NEG	POS	POS	NEG	NEG	POS	TSGH17	NEG	NEG
H24 (Egypt)	NEG	NEG	NEG	NEG	NEG	NEG	NEG	NEG	NEG	NEG	NEG	POS	POS	NEG	NEG	POS	TSGH17	NEG	NEG
ATCC BAA-39 (=HUSA304) (Hungary)	NEG	NEG	NEG	NEG	NEG	NEG	NEG	NEG	NEG	NEG	NEG	POS	POS	NEG	NEG	POS	TSGH17	NEG	NEG
HUSA304 (Hungary)	NEG	NEG	NEG	NEG	NEG	NEG	NEG	NEG	NEG	NEG	NEG	POS	POS	NEG	NEG	POS	TSGH17	NEG	NEG
HU106 (Hungary)	NEG	NEG	NEG	NEG	NEG	NEG	NEG	NEG	NEG	NEG	NEG	POS	POS	NEG	NEG	POS	TSGH17	NEG	NEG
BSAC27 (UK)	NEG	NEG	NEG	NEG	NEG	NEG	NEG	NEG	NEG	NEG	NEG	POS	POS	NEG	NEG	POS	TSGH17	NEG	NEG
NCTC13131, UK-EMRSA-4 (UK)	NEG	NEG	NEG	NEG	NEG	NEG	NEG	NEG	NEG	NEG	NEG	POS	POS	NEG	NEG	POS		NEG	NEG
	NEG	NEG	NEG	NEG	NEG	NEG	NEG	NEG	NEG	NEG	NEG	POS	POS	NEG	NEG	POS		NEG	NEG
Algiers_HT20040080	NEG	NEG	NEG	NEG	NEG	NEG	NEG	NEG	NEG	NEG	NEG	POS	POS	NEG	NEG	POS		NEG	NEG
Dublin-DSH_AR09_0.0066	NEG	NEG	NEG	neg	NEG	NEG	NEG	NEG	NEG	NEG	NEG	POS	POS	NEG	NEG	POS		NEG	NEG
Dublin-DSH_AR09_0-0065	NEG	NEG	NEG	NEG	NEG	NEG	NEG	NEG	NEG	NEG	NEG	POS	POS	NEG	NEG	POS		NEG	NEG
Dublin-DSH_Phenotype-III_84	NEG	NEG	NEG	NEG	NEG	NEG	NEG	NEG	NEG	NEG	NEG	POS	POS	NEG	NEG	POS		NEG	NEG
Hong Kong_130	NEG	NEG	NEG	NEG	NEG	NEG	NEG	NEG	NEG	NEG	NEG	POS	POS	NEG	NEG	POS		NEG	NEG
Kuwait_018	NEG	NEG	NEG	NEG	NEG	NEG	NEG	NEG	NEG	NEG	NEG	POS	POS	NEG	NEG	POS		NEG	NEG
Perth_08-17726	NEG	NEG	NEG	NEG	NEG	NEG	NEG	NEG	NEG	NEG	NEG	POS	POS	NEG	NEG	POS		NEG	NEG
Riyadh_Alfaisal/KKKSUH_86_MRS-14-279	NEG	NEG	NEG	NEG	NEG	NEG	NEG	NEG	NEG	NEG	NEG	POS	POS	NEG	NEG	POS		NEG	NEG
Riyadh_Alfaisal-04_Z3861_831588	NEG	NEG	NEG	NEG	NEG	NEG	NEG	NEG	NEG	NEG	NEG	POS	POS	NEG	NEG	POS		NEG	NEG
Riyadh_Alfaisal-30_S15724_1108013	NEG	NEG	NEG	NEG	NEG	NEG	NEG	NEG	NEG	NEG	NEG	POS	POS	NEG	NEG	POS		NEG	NEG
Riyadh_Alfaisal-6_22A_13_B3992_397721	NEG	NEG	NEG	NEG	NEG	NEG	NEG	NEG	NEG	NEG	NEG	POS	POS	NEG	NEG	POS		NEG	NEG
Riyadh_SSTI_52_3615482	NEG	NEG	NEG	NEG	NEG	NEG	NEG	NEG	NEG	NEG	NEG	POS	POS	NEG	NEG	POS		NEG	NEG
Riyadh-2817276-2	NEG	NEG	NEG	NEG	NEG	NEG	NEG	NEG	NEG	NEG	NEG	POS	POS	NEG	AMB	POS		NEG	NEG
Riyadh-2891670-W	NEG	NEG	NEG	NEG	NEG	NEG	NEG	NEG	NEG	NEG	NEG	POS	POS	NEG	AMB	POS		NEG	NEG
Riyadh-3006920-W	NEG	NEG	NEG	NEG	NEG	NEG	NEG	NEG	NEG	NEG	NEG	POS	POS	NEG	NEG	POS		NEG	NEG
Riyadh-R2567782	NEG	NEG	NEG	NEG	NEG	NEG	NEG	NEG	NEG	NEG	NEG	POS	POS	NEG	NEG	POS		NEG	NEG
Russia-18_0252_Krasnoyarsk_SK2	NEG	NEG	NEG	NEG	NEG	NEG	NEG	NEG	NEG	NEG	NEG	POS	POS	NEG	NEG	POS		NEG	NEG
UK-EMRSA-7	NEG	NEG	NEG	NEG	NEG	NEG	NEG	NEG	NEG	NEG	NEG	POS	POS	NEG	NEG	POS		NEG	NEG
>"South American/ Mid																			
Dublin-DSH_AR23_0073	NEG	NEG	NEG	NEG	NEG	NEG	NEG	NEG	NEG	NEG	NEG	POS	POS	NEG	NEG	POS		NEG	NEG
>Related to "South Ame																			
MRSA-OC3 (Russia)	NEG	NEG	NEG	NEG	NEG	NEG	NEG	NEG	NEG	NEG	NEG	POS	POS	NEG	NEG	POS	TSGH17	NEG	NEG
Russia-12_0176_Krasnoyarsk	NEG	NEG	NEG	NEG	NEG	NEG	NEG	NEG	NEG	NEG	NEG	POS	POS	NEG	NEG	POS		NEG	NEG
Russia-13_0180_Krasnoyarsk	NEG	NEG	NEG	NEG	NEG	NEG	NEG	NEG	NEG	NEG	NEG	POS	POS	NEG	NEG	POS		NEG	NEG
Russia-16_0249_Krasnoyarsk	NEG	NEG	NEG	NEG	NEG	NEG	NEG	NEG	NEG	NEG	NEG	POS	POS	NEG	NEG	POS		NEG	NEG
Russia-17_0250_Krasnoyarsk	NEG	NEG	NEG	NEG	NEG	NEG	NEG	NEG	NEG	NEG	NEG	POS	POS	NEG	NEG	POS		NEG	NEG
>Related to "South Ame																			
URU110 (Uruguay)	NEG	NEG	NEG	NEG	NEG	NEG	NEG	NEG	NEG	NEG	NEG	POS	POS	NEG	NEG	POS	TSGH17	NEG	NEG
>Related to "South Ame																			
DS_014 (Thailand)	NEG	NEG	NEG	NEG	NEG	NEG	NEG	NEG	NEG	NEG	NEG	POS	POS	NEG	NEG	POS	TSGH17	NEG	NEG
>Unassigned Middle Eas																			
Frankfurt_Oder_0490031797 (Libya)	NEG	NEG	NEG	NEG	NEG	NEG	NEG	NEG	NEG	NEG	NEG	POS	POS	NEG	POS	POS		NEG	NEG
Kuwait_192	NEG	NEG	NEG	NEG	NEG	NEG	NEG	NEG	NEG	NEG	NEG	POS	POS	NEG	POS	POS		NEG	NEG
Riyadh-9	NEG	NEG	NEG	NEG	NEG	NEG	NEG	NEG	NEG	NEG	NEG	POS	POS	NEG	POS	POS		NEG	NEG
Russia-24_0407_Moscow	NEG	NEG	NEG	NEG	NEG	NEG	NEG	NEG	NEG	NEG	NEG	POS	POS	NEG	POS	POS		NEG	NEG

STRAIN / ISOLATE	METHICILLIN RESISTANCE AND SCCmec TYPING															HEAVY METAL RESIST		
	Q9XB68-dcs	nsaB status	SCCmec Terminus 1	SCCmec Terminus 2	SCCmec Terminus 3	SCCmec Terminus 4	SCCmec Terminus 5	SCCmec Terminus 6	SCCmec Terminus 7	SCCmec Terminus 9	SCCmec Terminus 10	SCCmec Terminus 11	mec Terminu	mec Terminu	mec Terminu	merA	merB	mco-plasmid
	Q9XB68-dcs		SCCmec Terminus 1	SCCmec Terminus 2	SCCmec Terminus 3	SCCmec Terminus 4	SCCmec Terminus 5	SCCmec Terminus 6	SCCmec Terminus 7	SCCmec Terminus 9	SCCmec Terminus 10	SCCmec Terminus 11	SCCmec Terminus 12	SCCmec Terminus 13	SCCmec Terminus 14	merA	merB	mco-plasmid (MRSA252)
	Located at the terminus of SCCmec directly next to orfX.This locus comprises the downstream constant segment (dcs) that in turn comprises a copy of the SCC direct repeat DR_SCC (AGAAGCTTATCATAAGTAA)		SCC integration site alternate to dcs													mercury resistance operon		Multi copper oxidase
**>"Eurasian Clade": TUR1**																		
TUR1 (Turkey)	NEG	nsaB inserted	POS	POS	NEG	NEG	NEG	NEG	NEG	NEG	NEG	NEG	NEG	NEG	NEG	NEG	NEG	NEG
TUR9 (Turkey)	NEG	nsaB inserted	POS	POS	NEG	NEG	NEG	NEG	NEG	NEG	NEG	NEG	NEG	NEG	NEG	NEG	NEG	NEG
**>"Eurasian Clade" : T013**																		
T0131 (China)	POS	nsaB inserted	NEG	POS	NEG	NEG	NEG	NEG	NEG	NEG	NEG	NEG	NEG	NEG	NEG	NEG	NEG	NEG
MU4 (Turkey)	POS	nsaB inserted	NEG	NEG	NEG	NEG	NEG	NEG	NEG	NEG	NEG	NEG	NEG	NEG	NEG	NEG	NEG	NEG
DEU11 (Turkey)	POS	nsaB inserted	NEG	POS	NEG	NEG	NEG	NEG	NEG	NEG	NEG	NEG	NEG	NEG	NEG	NEG	NEG	NEG
DEU16 (Turkey)	POS	nsaB inserted	NEG	POS	NEG	NEG	NEG	NEG	NEG	NEG	NEG	NEG	NEG	NEG	NEG	NEG	NEG	NEG
Romania_Iasi_MRSA-07_54	POS		NEG	POS	NEG	NEG	NEG	NEG	NEG	NEG	NEG	NEG	NEG	NEG	NEG	NEG	NEG	NEG
**>"Eurasian Clade": arsC-**																		
Romania_Iasi_MRSA-05_46	POS		NEG	POS	NEG	NEG	NEG	NEG	NEG	NEG	NEG	NEG	NEG	NEG	NEG	NEG	NEG	NEG
Romania_Iasi_MRSA-06_47	POS		NEG	POS	AMB	NEG	NEG	NEG	NEG	NEG	NEG	NEG	NEG	NEG	NEG	NEG	NEG	NEG
**>"Eurasian Clade": 16K/**																		
CN79 (China)	POS	nsaB inserted	NEG	POS	NEG	NEG	NEG	NEG	NEG	NEG	NEG	NEG	NEG	NEG	NEG	NEG	NEG	NEG
CUHK_BJ2002 (China)	POS	nsaB inserted	NEG	POS	NEG	NEG	NEG	NEG	NEG	NEG	NEG	NEG	NEG	NEG	NEG	NEG	NEG	NEG
CUHK_BJ2007 (China)	POS	nsaB inserted	NEG	POS	NEG	NEG	NEG	NEG	NEG	NEG	NEG	NEG	NEG	NEG	NEG	NEG	NEG	NEG
3HK (Czech Republic)	POS	nsaB inserted	NEG	POS	NEG	NEG	NEG	NEG	NEG	NEG	NEG	NEG	NEG	NEG	NEG	NEG	NEG	NEG
HU109 (Hungary)	POS	nsaB inserted	NEG	POS	NEG	NEG	NEG	NEG	NEG	NEG	NEG	NEG	NEG	NEG	NEG	NEG	NEG	NEG
HUR18 (Hungary)	POS	nsaB inserted	NEG	POS	NEG	NEG	NEG	NEG	NEG	NEG	NEG	NEG	NEG	NEG	NEG	NEG	NEG	NEG
H482 (Romania)	POS	nsaB inserted	NEG	POS	NEG	NEG	NEG	NEG	NEG	NEG	NEG	NEG	NEG	NEG	NEG	NEG	NEG	NEG
16K (Russia)	POS	nsaB inserted	NEG	POS	NEG	NEG	NEG	NEG	NEG	NEG	NEG	NEG	NEG	NEG	NEG	NEG	NEG	NEG
DEU10 (Turkey)	POS	nsaB inserted	NEG	POS	NEG	NEG	NEG	NEG	NEG	NEG	NEG	NEG	NEG	NEG	NEG	NEG	NEG	NEG
DEU12 (Turkey)	POS	nsaB inserted	NEG	POS	NEG	NEG	NEG	NEG	NEG	NEG	NEG	NEG	NEG	NEG	NEG	NEG	NEG	NEG
DEU14 (Turkey)	POS	nsaB inserted	NEG	POS	NEG	NEG	NEG	NEG	NEG	NEG	NEG	NEG	NEG	NEG	NEG	NEG	NEG	NEG
DEU15 (Turkey)	POS	nsaB inserted	NEG	POS	NEG	NEG	NEG	NEG	NEG	NEG	NEG	NEG	NEG	NEG	NEG	NEG	NEG	NEG
DEU17 (Turkey)	POS	nsaB inserted	NEG	POS	NEG	NEG	NEG	NEG	NEG	NEG	NEG	NEG	NEG	NEG	NEG	NEG	NEG	NEG
DEU19 (Turkey)	POS	nsaB inserted	NEG	POS	NEG	NEG	NEG	NEG	NEG	NEG	NEG	NEG	NEG	NEG	NEG	NEG	NEG	NEG
DEU2 (Turkey)	POS	nsaB inserted	NEG	POS	NEG	NEG	NEG	NEG	NEG	NEG	NEG	NEG	NEG	NEG	NEG	NEG	NEG	NEG
DEU20 (Turkey)	POS	nsaB inserted	NEG	POS	NEG	NEG	NEG	NEG	NEG	NEG	NEG	NEG	NEG	NEG	NEG	NEG	NEG	NEG
DEU23 (Turkey)	POS	nsaB inserted	NEG	POS	NEG	NEG	NEG	NEG	NEG	NEG	NEG	NEG	NEG	NEG	NEG	NEG	NEG	NEG
DEU3 (Turkey)	POS	nsaB inserted	NEG	POS	NEG	NEG	NEG	NEG	NEG	NEG	NEG	NEG	NEG	NEG	NEG	NEG	NEG	NEG
DEU5 (Turkey)	POS	nsaB inserted	NEG	POS	NEG	NEG	NEG	NEG	NEG	NEG	NEG	NEG	NEG	NEG	NEG	NEG	NEG	NEG
DEU6 (Turkey)	POS	nsaB inserted	NEG	POS	NEG	NEG	NEG	NEG	NEG	NEG	NEG	NEG	NEG	NEG	NEG	NEG	NEG	NEG
DEU8 (Turkey)	POS	nsaB inserted	NEG	POS	NEG	NEG	NEG	NEG	NEG	NEG	NEG	NEG	NEG	NEG	NEG	NEG	NEG	NEG
HU11 (Turkey)	POS	nsaB inserted	NEG	POS	NEG	NEG	NEG	NEG	NEG	NEG	NEG	NEG	NEG	NEG	NEG	NEG	NEG	NEG
HU13 (Turkey)	POS	nsaB inserted	NEG	POS	NEG	NEG	NEG	NEG	NEG	NEG	NEG	NEG	NEG	NEG	NEG	NEG	NEG	NEG
HU14 (Turkey)	POS	nsaB inserted	NEG	POS	NEG	NEG	NEG	NEG	NEG	NEG	NEG	NEG	NEG	NEG	NEG	NEG	NEG	NEG
HU15 (Turkey)	POS	nsaB inserted	NEG	POS	NEG	NEG	NEG	NEG	NEG	NEG	NEG	NEG	NEG	NEG	NEG	NEG	NEG	NEG
HU16 (Turkey)	POS	nsaB inserted	NEG	POS	NEG	NEG	NEG	NEG	NEG	NEG	NEG	NEG	NEG	NEG	NEG	NEG	NEG	NEG
HU17 (Turkey)	POS	nsaB inserted	NEG	POS	NEG	NEG	NEG	NEG	NEG	NEG	NEG	NEG	NEG	NEG	NEG	NEG	NEG	NEG
HU21 (Turkey)	POS	nsaB inserted	NEG	POS	NEG	NEG	NEG	NEG	NEG	NEG	NEG	NEG	NEG	NEG	NEG	NEG	NEG	NEG
HU23 (Turkey)	POS	nsaB inserted	NEG	POS	NEG	NEG	NEG	NEG	NEG	NEG	NEG	NEG	NEG	NEG	NEG	NEG	NEG	NEG
HU26 (Turkey)	POS	nsaB inserted	NEG	POS	NEG	NEG	NEG	NEG	NEG	NEG	NEG	NEG	NEG	NEG	NEG	NEG	NEG	NEG
HU4 (Turkey)	POS	nsaB inserted	NEG	POS	NEG	NEG	NEG	NEG	NEG	NEG	NEG	NEG	NEG	NEG	NEG	NEG	NEG	NEG
HU5 (Turkey)	POS	nsaB inserted	NEG	POS	NEG	NEG	NEG	NEG	NEG	NEG	NEG	NEG	NEG	NEG	NEG	NEG	NEG	NEG
HU6 (Turkey)	POS	nsaB inserted	NEG	POS	NEG	NEG	NEG	NEG	NEG	NEG	NEG	NEG	NEG	NEG	NEG	NEG	NEG	NEG
HU7 (Turkey)	POS	nsaB inserted	NEG	POS	NEG	NEG	NEG	NEG	NEG	NEG	NEG	NEG	NEG	NEG	NEG	NEG	NEG	NEG
HU8 (Turkey)	POS	nsaB inserted	NEG	POS	NEG	NEG	NEG	NEG	NEG	NEG	NEG	NEG	NEG	NEG	NEG	NEG	NEG	NEG
HU9 (Turkey)	POS	nsaB inserted	NEG	POS	NEG	NEG	NEG	NEG	NEG	NEG	NEG	NEG	NEG	NEG	NEG	NEG	NEG	NEG
IU1 (Turkey)	POS	nsaB inserted	NEG	POS	NEG	NEG	NEG	NEG	NEG	NEG	NEG	NEG	NEG	NEG	NEG	NEG	NEG	NEG
IU10 (Turkey)	POS	nsaB inserted	NEG	POS	NEG	NEG	NEG	NEG	NEG	NEG	NEG	NEG	NEG	NEG	NEG	NEG	NEG	NEG
IU11 (Turkey)	POS	nsaB inserted	NEG	POS	NEG	NEG	NEG	NEG	NEG	NEG	NEG	NEG	NEG	NEG	NEG	NEG	NEG	NEG
IU12 (Turkey)	POS	nsaB inserted	NEG	POS	NEG	NEG	NEG	NEG	NEG	NEG	NEG	NEG	NEG	NEG	NEG	NEG	NEG	NEG
IU13 (Turkey)	POS	nsaB inserted	NEG	POS	NEG	NEG	NEG	NEG	NEG	NEG	NEG	NEG	NEG	NEG	NEG	NEG	NEG	NEG
IU15 (Turkey)	POS	nsaB inserted	NEG	POS	NEG	NEG	NEG	NEG	NEG	NEG	NEG	NEG	NEG	NEG	NEG	NEG	NEG	NEG
IU18 (Turkey)	POS	nsaB inserted	NEG	POS	NEG	NEG	NEG	NEG	NEG	NEG	NEG	NEG	NEG	NEG	NEG	NEG	NEG	NEG
IU19 (Turkey)	POS	nsaB inserted	NEG	POS	NEG	NEG	NEG	NEG	NEG	NEG	NEG	NEG	NEG	NEG	NEG	NEG	NEG	NEG
IU2 (Turkey)	POS	nsaB inserted	NEG	POS	NEG	NEG	NEG	NEG	NEG	NEG	NEG	NEG	NEG	NEG	NEG	NEG	NEG	NEG
IU4 (Turkey)	POS	nsaB inserted	NEG	POS	NEG	NEG	NEG	NEG	NEG	NEG	NEG	NEG	NEG	NEG	NEG	NEG	NEG	NEG
IU6 (Turkey)	POS	nsaB inserted	NEG	POS	NEG	NEG	NEG	NEG	NEG	NEG	NEG	NEG	NEG	NEG	NEG	NEG	NEG	NEG
IU7 (Turkey)	POS	nsaB inserted	NEG	POS	NEG	NEG	NEG	NEG	NEG	NEG	NEG	NEG	NEG	NEG	NEG	NEG	NEG	NEG
IU9 (Turkey)	POS	nsaB inserted	NEG	POS	NEG	NEG	NEG	NEG	NEG	NEG	NEG	NEG	NEG	NEG	NEG	NEG	NEG	NEG
MU1 (Turkey)	POS	nsaB inserted	NEG	POS	NEG	NEG	NEG	NEG	NEG	NEG	NEG	NEG	NEG	NEG	NEG	NEG	NEG	NEG
MU10 (Turkey)	POS	nsaB inserted	NEG	POS	NEG	NEG	NEG	NEG	NEG	NEG	NEG	NEG	NEG	NEG	NEG	NEG	NEG	NEG
MU20 (Turkey)	POS	nsaB inserted	NEG	POS	NEG	NEG	NEG	NEG	NEG	NEG	NEG	NEG	NEG	NEG	NEG	NEG	NEG	NEG
Mu3 (Turkey)	POS	nsaB inserted	NEG	POS	NEG	NEG	NEG	NEG	NEG	NEG	NEG	NEG	NEG	NEG	NEG	NEG	NEG	NEG
MU5 (Turkey)	POS	nsaB inserted	NEG	POS	NEG	NEG	NEG	NEG	NEG	NEG	NEG	NEG	NEG	NEG	NEG	NEG	NEG	NEG
MU6 (Turkey)	POS	nsaB inserted	NEG	POS	NEG	NEG	NEG	NEG	NEG	NEG	NEG	NEG	NEG	NEG	NEG	NEG	NEG	NEG
MU7 (Turkey)	POS	nsaB inserted	NEG	POS	NEG	NEG	NEG	NEG	NEG	NEG	NEG	NEG	NEG	NEG	NEG	NEG	NEG	NEG
TUR27 (Turkey)	POS	nsaB inserted	NEG	POS	NEG	NEG	NEG	NEG	NEG	NEG	NEG	NEG	NEG	NEG	NEG	NEG	NEG	NEG
NCTR 32S	POS	nsaB inserted	NEG	POS	NEG	NEG	NEG	NEG	NEG	NEG	NEG	NEG	NEG	NEG	NEG	NEG	NEG	NEG
SA02_A4 = Russia-06_0085_Moscow	POS		NEG	POS	NEG	NEG	NEG	NEG	NEG	NEG	NEG	NEG	NEG	NEG	NEG	NEG	NEG	NEG
	POS		NEG	POS	NEG	NEG	NEG	NEG	NEG	NEG	NEG	NEG	NEG	NEG	NEG	NEG	NEG	NEG
Dresden_08V35987 (Turkey)	POS		NEG	POS	NEG	NEG	NEG	NEG	NEG	NEG	NEG	NEG	NEG	NEG	NEG	NEG	NEG	NEG
Dresden_17ANR577152	POS		NEG	POS	NEG	NEG	NEG	NEG	NEG	NEG	NEG	NEG	NEG	NEG	NEG	NEG	NEG	NEG
Dresden_17ANR580374 (Makedonia)	POS		NEG	POS	NEG	NEG	NEG	NEG	NEG	NEG	NEG	NEG	NEG	NEG	NEG	NEG	NEG	NEG
Hong Kong_69-II	POS		NEG	POS	NEG	NEG	NEG	NEG	NEG	NEG	NEG	NEG	NEG	NEG	NEG	NEG	NEG	NEG
Hong Kong_93	POS		NEG	POS	NEG	NEG	NEG	NEG	NEG	NEG	NEG	NEG	NEG	NEG	NEG	NEG	NEG	NEG
Rawalpindi_Kidney Center_03_SK1	POS		NEG	POS	NEG	NEG	NEG		NEG	NEG	NEG	NEG	NEG	NEG	NEG	NEG	NEG	NEG
Rawalpindi_Kidney Center_08	POS		NEG	POS	NEG	NEG	NEG	NEG	NEG	NEG	NEG	NEG	NEG	NEG	NEG	NEG	NEG	NEG
Rawalpindi_Kidney Center_10	POS		NEG	POS	NEG	NEG	NEG	NEG	NEG	NEG	NEG	NEG	NEG	NEG	NEG	NEG	NEG	NEG
Rawalpindi_Kidney Center_19	POS		NEG	POS	NEG	NEG	NEG	NEG	NEG	NEG	NEG	NEG	NEG	NEG	NEG	NEG	NEG	NEG
Rawalpindi_Kidney Center_50_SK1	POS		NEG	POS	NEG	NEG	NEG	NEG	NEG	NEG	NEG	NEG	NEG	NEG	NEG	NEG	NEG	NEG
Romania_Iasi_BC-49_430	POS		NEG	POS	NEG	NEG	NEG	NEG	NEG	NEG	NEG	NEG	NEG	NEG	NEG	NEG	NEG	NEG
Romania_Iasi_MRSA_284	POS		NEG	POS	NEG	NEG	NEG	NEG	NEG	NEG	NEG	NEG	NEG	NEG	NEG	NEG	NEG	NEG
Romania_Iasi_MRSA-32_318	POS		NEG	POS	NEG	NEG	NEG	NEG	NEG	NEG	NEG	NEG	NEG	NEG	NEG	NEG	NEG	NEG
Romania_Iasi_MRSA-54_430	POS		NEG	AMB	NEG	NEG	NEG	NEG	NEG	NEG	NEG	NEG	NEG	NEG	NEG	NEG	NEG	NEG
Romania_Iasi_SSTI-01_2	POS		NEG	POS	NEG	NEG	NEG	NEG	NEG	NEG	NEG	NEG	NEG	NEG	NEG	NEG	NEG	NEG
Romania_Iasi_SSTI-10_101	POS		NEG	POS	NEG	NEG	NEG	NEG	NEG	NEG	NEG	NEG	NEG	NEG	NEG	NEG	NEG	NEG
Romania_Iasi_SSTI-11_106	POS		NEG	POS	NEG	NEG	NEG	NEG	NEG	NEG	NEG	NEG	NEG	NEG	NEG	NEG	NEG	NEG
Russia-01_0001_SaintPetersburg	POS		NEG	POS	NEG	NEG	NEG	NEG	NEG	NEG	NEG	NEG	NEG	NEG	NEG	NEG	NEG	NEG
Russia-02_0004_SaintPetersburg	POS		NEG	POS	NEG	NEG	NEG	NEG	NEG	NEG	NEG	NEG	NEG	NEG	NEG	NEG	NEG	NEG
Russia-03_0057_SaintPetersburg	POS		NEG	POS	NEG	NEG	NEG	NEG	NEG	NEG	NEG	NEG	NEG	NEG	NEG	NEG	NEG	NEG
Russia-04_0076_Moscow	POS		NEG	POS	NEG	NEG	NEG	NEG	NEG	NEG	NEG	NEG	NEG	NEG	NEG	NEG	NEG	NEG
Russia-05_0078_Moscow	POS		NEG	POS	NEG	NEG	NEG	NEG	NEG	NEG	NEG	NEG	NEG	NEG	NEG	NEG	NEG	NEG
Russia-08_0135_Moscow	POS		NEG	POS	NEG	NEG	NEG	NEG	NEG	NEG	NEG	NEG	NEG	NEG	NEG	NEG	NEG	NEG
Russia-09_0150_Kurgan	POS		NEG	POS	NEG	NEG	NEG	NEG	NEG	NEG	NEG	NEG	NEG	NEG	NEG	NEG	NEG	NEG
Russia-10_0162_Kurgan	POS		NEG	POS	NEG	NEG	NEG	NEG	NEG	NEG	NEG	NEG	NEG	NEG	NEG	NEG	NEG	NEG
Russia-14_0184_Moscow	AMB		NEG	POS	NEG	NEG	NEG	NEG	NEG	NEG	NEG	NEG	NEG	NEG	NEG	NEG	NEG	NEG
Russia-15_0232_SaintPetersburg	POS		NEG	POS	NEG	NEG	NEG	NEG	NEG	NEG	NEG	NEG	NEG	NEG	NEG	NEG	NEG	NEG
Russia-22_0391_Chelyabinsk	POS		NEG	POS	NEG	NEG	NEG	NEG	NEG	NEG	NEG	NEG	NEG	NEG	NEG	NEG	NEG	NEG
Russia-23_0392_Chelyabinsk	POS		NEG	POS	NEG	NEG	NEG	NEG	NEG	NEG	NEG	NEG	NEG	NEG	NEG	NEG	NEG	NEG
**>"Eurasian Clade" : IU17**																		
IU17 (Turkey)	NEG	nsaB inserted	NEG	POS	NEG	NEG	NEG	NEG	NEG	NEG	NEG	NEG	NEG	NEG	NEG	NEG	NEG	NEG
IU20 (Turkey)	NEG	nsaB inserted	NEG	POS	NEG	NEG	NEG	NEG	NEG	NEG	NEG	NEG	NEG	NEG	NEG	NEG	NEG	NEG

STRAIN / ISOLATE	METHICILLIN RESISTANCE AND SCCmec TYPING															HEAVY METAL RESIST		
	Q9XB68-dcs	nsaB status	SCCmec Terminus 1	SCCmec Terminus 2	SCCmec Terminus 3	SCCmec Terminus 4	SCCmec Terminus 5	SCCmec Terminus 6	SCCmec Terminus 7	SCCmec Terminus 9	SCCmec Terminus 10	SCCmec Terminus 11	mec Terminu	mec Terminu	mec Terminu	merA	merB	mco-plasmid
	Q9XB68-dcs		SCCmec Terminus 1	SCCmec Terminus 2	SCCmec Terminus 3	SCCmec Terminus 4	SCCmec Terminus 5	SCCmec Terminus 6	SCCmec Terminus 7	SCCmec Terminus 9	SCCmec Terminus 10	SCCmec Terminus 11	SCCmec Terminus 12	SCCmec Terminus 13	SCCmec Terminus 14	merA	merB	mco-plasmid (MRSA252)
	Located at the terminus of SCCmec directly next to orfX.This locus comprises the downstream constant segment (dcs) that in turn comprises a copy of the SCC direct repeat DR_SCC (AGAAGCTTATCATAAGTAA)		SCC integration site alternate to dcs													mercury resistance operon		Multi copper oxidase
>"Eurasian Clade": Spora																		
Dresden_16ANR581769	POS		NEG	POS	NEG	NEG	POS	NEG	NEG	NEG	NEG	NEG	NEG	NEG	NEG	NEG	NEG	NEG
Dresden_17ANR578769	POS		NEG	NEG	NEG	NEG	POS	NEG	NEG	NEG	NEG	NEG	NEG	NEG	NEG	NEG	NEG	NEG
>"Eurasian Clade": Kuwa																		
Kuwait_306	POS		NEG	POS	POS	NEG	POS	NEG	NEG	NEG	NEG	NEG	NEG	NEG	NEG	NEG	NEG	NEG
Kuwait_301	POS		NEG	POS	POS	NEG	POS	NEG	NEG	NEG	NEG	NEG	NEG	NEG	NEG	NEG	NEG	NEG
>"European Clade": "Gr																		
GRE18 (Greece)	NEG	nsaB wildtype	POS	POS	NEG	NEG	NEG	NEG	NEG	NEG	NEG	NEG	NEG	NEG	NEG	POS	POS	NEG
GRE317 (Greece)	NEG	nsaB wildtype	POS	POS	NEG	NEG	NEG	NEG	NEG	NEG	NEG	NEG	NEG	NEG	NEG	POS	POS	NEG
GRE4 (Greece)	NEG	nsaB wildtype	POS	POS	NEG	NEG	NEG	NEG	NEG	NEG	NEG	NEG	NEG	NEG	NEG	POS	POS	NEG
R35 (USA)	NEG	nsaB wildtype	POS	POS	NEG	NEG	NEG	NEG	NEG	NEG	NEG	NEG	NEG	NEG	NEG	POS	POS	NEG
Casablanca HT20060548	NEG		POS	POS	NEG	NEG	NEG	NEG	NEG	NEG	NEG	NEG	NEG	NEG	NEG	POS	POS	NEG
Casablanca HT20060550	NEG		POS	POS	NEG	NEG	NEG	NEG	NEG	NEG	NEG	NEG	NEG	NEG	NEG	POS	POS	NEG
Dresden_01V34572 (Greece)	NEG		AMB	POS	NEG	NEG	NEG	NEG	NEG	NEG	NEG	NEG	NEG	NEG	NEG	POS	POS	NEG
Dresden_01V36469	NEG		POS	POS	NEG	NEG	NEG	NEG	NEG	NEG	NEG	NEG	NEG	NEG	NEG	POS	POS	NEG
Dresden 01V39123	NEG		POS	POS	NEG	NEG	NEG	NEG	NEG	NEG	NEG	NEG	NEG	NEG	NEG	POS	POS	NEG
Dresden 01V39143	NEG		POS	POS	NEG	NEG	NEG	NEG	NEG	NEG	NEG	NEG	NEG	NEG	NEG	POS	POS	NEG
Greece 1 3680 Harmony	NEG		POS	POS	NEG	NEG	NEG	NEG	NEG	NEG	NEG	NEG	NEG	NEG	NEG	AMB	POS	NEG
>"European Clade": UK-																		
ANS46 (Australia)	NEG	nsaB wildtype	POS	POS	NEG	NEG	NEG	NEG	NEG	NEG	NEG	NEG	NEG	NEG	NEG	POS	POS	NEG
LHH1 (USA)	NEG	nsaB wildtype	POS	POS	NEG	NEG	NEG	NEG	NEG	NEG	NEG	NEG	NEG	NEG	NEG	POS	POS	NEG
Dublin-DSH_AR01_0-1085	NEG		POS	POS	NEG	NEG	NEG	NEG	NEG	NEG	NEG	NEG	NEG	NEG	NEG	POS	POS	NEG
Dublin-DSH_AR01_0-1118	NEG		POS	POS	NEG	NEG	NEG	NEG	NEG	NEG	NEG	NEG	NEG	NEG	NEG	POS	POS	NEG
Dublin-DSH_AR15_0098	NEG		POS	POS	NEG	NEG	NEG	NEG	NEG	NEG	NEG	NEG	NEG	neg	NEG	POS	POS	NEG
Dublin-DSH_AR15_0104	NEG		AMB	POS	NEG	NEG	NEG	NEG	NEG	NEG	NEG	NEG	NEG	NEG	NEG	POS	POS	NEG
UK_NCTC11939_UK-1_Harmony	NEG		POS	POS	NEG	NEG	NEG	NEG	NEG	NEG	NEG	NEG	NEG	NEG	NEG	POS	POS	NEG
>"European Clade": BK2																		
BK2421 (USA)	NEG		POS	POS	NEG	NEG	NEG	NEG	NEG	NEG	NEG	NEG	NEG	NEG	NEG	POS	POS	NEG
>"Aussi/NZ Clade", JKD6																		
JKD6009 (Australia)	POS	nsaB wildtype	NEG	NEG	NEG	NEG	NEG	NEG	NEG	NEG	NEG	NEG	NEG	NEG	NEG	NEG	NEG	NEG
JKD6008 (New Zealand)	POS	nsaB wildtype	NEG	NEG	NEG	NEG	NEG	NEG	NEG	NEG	NEG	NEG	NEG	NEG	NEG	NEG	NEG	NEG
Perth_2015-S20209	POS	nsaB wildtype	NEG	NEG	NEG	NEG	NEG	NEG	NEG	NEG	NEG	NEG	NEG	NEG	NEG	NEG	NEG	NEG
Perth_2015-S20305	POS	nsaB wildtype	NEG	NEG	NEG	NEG	NEG	NEG	NEG	NEG	NEG	NEG	NEG	NEG	NEG	NEG	NEG	NEG
Perth_2015-S20603	POS	nsaB wildtype	NEG	NEG	NEG	NEG	NEG	NEG	NEG	NEG	NEG	NEG	NEG	NEG	NEG	NEG	NEG	NEG
Perth_2015-S20607	POS	nsaB wildtype	NEG	NEG	NEG	NEG	NEG	NEG	NEG	NEG	NEG	NEG	NEG	NEG	NEG	NEG	NEG	NEG
Perth_2015-S20608	POS	nsaB wildtype	NEG	NEG	NEG	NEG	NEG	NEG	NEG	NEG	NEG	NEG	NEG	NEG	NEG	NEG	NEG	NEG
Perth_2015-S20610	POS	nsaB wildtype	NEG	NEG	NEG	NEG	NEG	NEG	NEG	NEG	NEG	NEG	NEG	NEG	NEG	NEG	NEG	NEG
Perth_2015-S20613	POS	nsaB wildtype	NEG	NEG	NEG	NEG	NEG	NEG	NEG	NEG	NEG	NEG	NEG	NEG	NEG	NEG	NEG	NEG
Perth_2015-S20617	POS	nsaB wildtype	NEG	NEG	NEG	NEG	NEG	NEG	NEG	NEG	NEG	NEG	NEG	NEG	NEG	NEG	NEG	NEG
Perth_2015-S20622	POS	nsaB wildtype	NEG	NEG	NEG	NEG	NEG	NEG	NEG	NEG	NEG	NEG	NEG	NEG	NEG	NEG	NEG	NEG
Perth_2015-S23619	POS	nsaB wildtype	NEG	NEG	NEG	NEG	NEG	NEG	NEG	NEG	NEG	NEG	NEG	NEG	NEG	NEG	NEG	NEG
Perth_2015-S41210	POS	nsaB wildtype	NEG	NEG	NEG	NEG	NEG	NEG	NEG	NEG	NEG	NEG	NEG	NEG	NEG	NEG	NEG	NEG
Perth_2015-S51001	POS	nsaB wildtype	NEG	NEG	NEG	NEG	NEG	NEG	NEG	NEG	NEG	NEG	NEG	NEG	NEG	NEG	NEG	NEG
Perth_2015-S51003	POS	nsaB wildtype	NEG	NEG	NEG	NEG	NEG	NEG	NEG	NEG	NEG	NEG	NEG	NEG	NEG	NEG	NEG	NEG
Perth_2015-S51006	POS	nsaB wildtype	NEG	NEG	NEG	NEG	NEG	NEG	NEG	NEG	NEG	NEG	NEG	NEG	NEG	NEG	NEG	NEG
Perth_2015-S51007	POS	nsaB wildtype	NEG	NEG	NEG	NEG	NEG	NEG	NEG	NEG	NEG	NEG	NEG	NEG	NEG	NEG	NEG	NEG
Perth_2015-S51008	POS	nsaB wildtype	NEG	NEG	NEG	NEG	NEG	NEG	NEG	NEG	NEG	NEG	NEG	NEG	NEG	NEG	NEG	NEG
Perth_2015-S51014	POS	nsaB wildtype	NEG	NEG	NEG	NEG	NEG	NEG	NEG	NEG	NEG	NEG	NEG	NEG	NEG	NEG	NEG	NEG
Perth_2015-S51408	POS	nsaB wildtype	NEG	NEG	NEG	NEG	NEG	NEG	NEG	NEG	NEG	NEG	NEG	NEG	NEG	NEG	NEG	NEG
Perth_2016-S20614	POS	nsaB wildtype	NEG	NEG	NEG	NEG	NEG	NEG	NEG	NEG	NEG	NEG	NEG	NEG	NEG	NEG	NEG	NEG
Perth_2016-S23614	POS	nsaB wildtype	NEG	NEG	NEG	NEG	NEG	NEG	NEG	NEG	NEG	NEG	NEG	NEG	NEG	NEG	NEG	NEG
Perth_2016-S42009	AMB	nsaB wildtype	NEG	NEG	NEG	NEG	NEG	NEG	NEG	NEG	NEG	NEG	NEG	NEG	NEG	NEG	NEG	NEG
Perth_2016-S51001	POS	nsaB wildtype	NEG	NEG	NEG	NEG	NEG	NEG	NEG	NEG	NEG	NEG	NEG	NEG	NEG	NEG	NEG	NEG
Perth_2016-S51403	POS	nsaB wildtype	NEG	NEG	NEG	NEG	NEG	NEG	NEG	NEG	NEG	NEG	NEG	NEG	NEG	NEG	NEG	NEG
Perth_07 RPA 20	POS		NEG	NEG	NEG	NEG	NEG	NEG	NEG	NEG	NEG	NEG	NEG	NEG	NEG	NEG	NEG	NEG
Perth_08-19088	POS		NEG	NEG	NEG	NEG	NEG	NEG	NEG	NEG	NEG	NEG	NEG	NEG	NEG	NEG	NEG	NEG
Perth_01-15351	POS		NEG	NEG	NEG	NEG	NEG	NEG	NEG	NEG	NEG	NEG	NEG	NEG	NEG	NEG	NEG	NEG
Perth_01-15357	POS		NEG	NEG	NEG	NEG	NEG	NEG	NEG	NEG	NEG	NEG	NEG	NEG	NEG	NEG	NEG	NEG
Perth_01-15419	POS		NEG	NEG	NEG	NEG	NEG	NEG	NEG	NEG	NEG	NEG	NEG	NEG	NEG	NEG	NEG	NEG
Perth_01-16309	POS		NEG	NEG	NEG	NEG	NEG	NEG	NEG	NEG	NEG	NEG	NEG	NEG	NEG	NEG	NEG	NEG
Perth_03 SNP 95	POS		NEG	NEG	NEG	NEG	NEG	NEG	NEG	NEG	NEG	NEG	NEG	NEG	NEG	NEG	NEG	NEG
Perth_2003_AH_47	POS		NEG	NEG	NEG	NEG	NEG	NEG	NEG	NEG	NEG	NEG	NEG	NEG	NEG	NEG	NEG	NEG
>"Australian/NZ Clade":																		
Perth_2015-S20623	POS	nsaB wildtype	NEG	NEG	POS	NEG	POS	NEG	NEG	NEG	NEG	NEG	NEG	NEG	NEG	NEG	NEG	NEG
Perth_2015-S20826	POS	nsaB wildtype	NEG	NEG	POS	NEG	POS	NEG	NEG	NEG	NEG	NEG	NEG	NEG	NEG	NEG	NEG	NEG
Perth_05 NH 56	POS		NEG	NEG	POS	NEG	POS	NEG	NEG	NEG	NEG	NEG	NEG	NEG	NEG	NEG	NEG	NEG
>CC5-MRSA-III LA MRSA																		
Delbrück 38 isolate from domestic turkey	POS		NEG	NEG	NEG	NEG	NEG	NEG	NEG	NEG	NEG	NEG	NEG	NEG	NEG	NEG	NEG	NEG
> Staph. pseudintermed																		
KM1381	POS		NEG	NEG	NEG	NEG	NEG	NEG	NEG	NEG	NEG	NEG	NEG	NEG	NEG	NEG	NEG	NEG
>"South-East Asian Clad																		
M92 (Canada)	NEG	nsaB wildtype	POS	POS	NEG	NEG	NEG	NEG	NEG	NEG	NEG	NEG	NEG	NEG	NEG	POS	POS	NEG
CHI59 (China)	NEG	nsaB wildtype	POS	POS	NEG	NEG	NEG	NEG	NEG	NEG	NEG	NEG	NEG	NEG	NEG	POS	POS	NEG
CHI61 (China)	NEG	nsaB wildtype	POS	POS	NEG	NEG	NEG	NEG	NEG	NEG	NEG	NEG	NEG	NEG	NEG	POS	POS	NEG
CUHK_HK1997 (China)	NEG	nsaB wildtype	POS	POS	NEG	NEG	NEG	NEG	NEG	NEG	NEG	NEG	NEG	NEG	NEG	POS	POS	NEG
M996 (China)	NEG	nsaB wildtype	POS	POS	NEG	NEG	NEG	NEG	NEG	NEG	NEG	NEG	NEG	NEG	NEG	POS	POS	NEG
H211 (Denmark)	NEG	nsaB wildtype	POS	POS	NEG	NEG	NEG	NEG	NEG	NEG	NEG	NEG	NEG	NEG	NEG	POS	POS	NEG
H216 (Denmark)	NEG	nsaB wildtype	POS	POS	NEG	NEG	NEG	NEG	NEG	NEG	NEG	NEG	NEG	NEG	NEG	POS	POS	NEG
D71 (Germany)	NEG	nsaB wildtype	POS	POS	NEG	NEG	NEG	NEG	NEG	NEG	NEG	NEG	NEG	NEG	NEG	POS	POS	NEG
NMR05 (India)	NEG	nsaB wildtype	POS	POS	NEG	NEG	NEG	NEG	NEG	NEG	NEG	NEG	NEG	NEG	NEG	POS	POS	NEG
MAL11 (Malaysia)	NEG	nsaB wildtype	POS	POS	NEG	NEG	NEG	NEG	NEG	NEG	NEG	NEG	NEG	NEG	NEG	POS	POS	NEG
MAL9 (Malaysia)	NEG	nsaB wildtype	POS	POS	NEG	NEG	NEG	NEG	NEG	NEG	NEG	NEG	NEG	NEG	NEG	POS	POS	NEG
Na21 (Sri Lanka)	NEG	nsaB wildtype	POS	POS	NEG	NEG	NEG	NEG	NEG	NEG	NEG	NEG	NEG	NEG	NEG	POS	POS	NEG
M592 (Syria)	NEG	nsaB wildtype	POS	POS	NEG	NEG	NEG	NEG	NEG	NEG	NEG	NEG	NEG	NEG	NEG	POS	POS	NEG
H202 (Thailand)	NEG	nsaB wildtype	POS	POS	NEG	NEG	NEG	NEG	NEG	NEG	NEG	NEG	NEG	NEG	NEG	POS	POS	NEG
S102 (Thailand)	NEG	nsaB wildtype	POS	POS	NEG	NEG	NEG	NEG	NEG	NEG	NEG	NEG	NEG	NEG	NEG	POS	POS	NEG
S130 (Thailand)	NEG	nsaB wildtype	POS	POS	NEG	NEG	NEG	NEG	NEG	NEG	NEG	NEG	NEG	NEG	NEG	POS	POS	NEG
S40 (Thailand)	NEG	nsaB wildtype	POS	POS	NEG	NEG	NEG	NEG	NEG	NEG	NEG	NEG	NEG	NEG	NEG	POS	POS	NEG
S71 (Thailand)	NEG	nsaB wildtype	POS	POS	NEG	NEG	NEG	NEG	NEG	NEG	NEG	NEG	NEG	NEG	NEG	POS	POS	NEG
S87 (Thailand)	NEG	nsaB wildtype	POS	POS	NEG	NEG	NEG	NEG	NEG	NEG	NEG	NEG	NEG	NEG	NEG	POS	POS	NEG
S93 (Thailand)	NEG	nsaB wildtype	POS	POS	NEG	NEG	NEG	NEG	NEG	NEG	NEG	NEG	NEG	NEG	NEG	POS	POS	NEG
US_002 (Thailand)	NEG	nsaB wildtype	POS	POS	NEG	NEG	NEG	NEG	NEG	NEG	NEG	NEG	NEG	NEG	NEG	POS	POS	NEG
US_017 (Thailand)	NEG	nsaB wildtype	POS	POS	NEG	NEG	NEG	NEG	NEG	NEG	NEG	NEG	NEG	NEG	NEG	POS	POS	NEG
WAMC6102 (Thailand)	NEG	nsaB wildtype	POS	POS	NEG	NEG	NEG	NEG	NEG	NEG	NEG	NEG	NEG	NEG	NEG	POS	POS	NEG
NCTC_13626_TW20 (UK)	NEG	nsaB wildtype	POS	POS	NEG	NEG	NEG	NEG	NEG	NEG	NEG	NEG	NEG	NEG	NEG	POS	POS	NEG
BSAC3021 (UK)	NEG	nsaB wildtype	POS	POS	NEG	NEG	NEG	NEG	NEG	NEG	NEG	NEG	NEG	NEG	NEG	POS	POS	NEG
BSAC3046 (UK)	NEG	nsaB wildtype	POS	POS	NEG	NEG	NEG	NEG	NEG	NEG	NEG	NEG	NEG	NEG	NEG	POS	POS	NEG
1193_SAUR (USA)	NEG	nsaB wildtype	POS	POS	NEG	NEG	NEG	NEG	NEG	NEG	NEG	NEG	NEG	NEG	NEG	POS	POS	NEG
1194_SAUR (USA)	NEG	nsaB wildtype	POS	POS	NEG	NEG	NEG	NEG	NEG	NEG	NEG	NEG	NEG	NEG	NEG	POS	POS	NEG

STRAIN / ISOLATE	METHICILLIN RESISTANCE AND SCCmec TYPING															HEAVY METAL RESIST		
	Q9XB68-dcs	nsaB status	SCCmec Terminus 1	SCCmec Terminus 2	SCCmec Terminus 3	SCCmec Terminus 4	SCCmec Terminus 5	SCCmec Terminus 6	SCCmec Terminus 7	SCCmec Terminus 9	SCCmec Terminus 10	SCCmec Terminus 11	:mec Terminu	:mec Terminu	:mec Terminu	merA	merB	mco-plasmid
	Q9XB68-dcs		SCCmec Terminus 1	SCCmec Terminus 2	SCCmec Terminus 3	SCCmec Terminus 4	SCCmec Terminus 5	SCCmec Terminus 6	SCCmec Terminus 7	SCCmec Terminus 9	SCCmec Terminus 10	SCCmec Terminus 11	SCCmec Terminus 12	SCCmec Terminus 13	SCCmec Terminus 14	merA	merB	mco-plasmid (MRSA252)
	Located at the terminus of SCCmec directly next to orfX.This locus comprises the downstream constant segment (dcs) that in turn comprises a copy of the SCC direct repeat DR_SCC (AGAAGCTTATCATAAGTAA)		SCC integration site alternate to dcs													mercury resistance operon		Multi copper oxidase
>"South-East Asian Clade": TW20 (Irish AR44), cc																		
1195_SAUR (USA)	NEG	nsaB wildtype	POS	POS	NEG	NEG	NEG	NEG	NEG	NEG	NEG	NEG	NEG	NEG	NEG	POS	POS	NEG
FVRH6002 (USA)	NEG	nsaB wildtype	POS	POS	NEG	NEG	NEG	NEG	NEG	NEG	NEG	NEG	NEG	NEG	NEG	POS	POS	NEG
KINW6048 (USA)	NEG	nsaB wildtype	POS	POS	NEG	NEG	NEG	NEG	NEG	NEG	NEG	NEG	NEG	NEG	NEG	POS	POS	NEG
LAMC0011 (USA)	NEG	nsaB wildtype	POS	POS	NEG	NEG	NEG	NEG	NEG	NEG	NEG	NEG	NEG	NEG	NEG	POS	POS	NEG
SJOS6053 (USA)	NEG	nsaB wildtype	POS	POS	NEG	NEG	NEG	NEG	NEG	NEG	NEG	NEG	NEG	NEG	NEG	POS	POS	NEG
SJOS6072 (USA)	NEG	nsaB wildtype	POS	POS	NEG	NEG	NEG	NEG	NEG	NEG	NEG	NEG	NEG	NEG	NEG	POS	POS	NEG
UCIM6042 (USA)	NEG	nsaB wildtype	POS	POS	NEG	NEG	NEG	NEG	NEG	NEG	NEG	NEG	NEG	NEG	NEG	POS	POS	NEG
Perth_2015-S31902	NEG	nsaB wildtype	NEG	POS	NEG	NEG	NEG	NEG	NEG	NEG	NEG	NEG	NEG	NEG	NEG	POS	POS	NEG
Perth_2015-S31904	NEG	nsaB wildtype	NEG	POS	NEG	NEG	NEG	NEG	NEG	NEG	NEG	NEG	NEG	NEG	NEG	POS	POS	NEG
Perth_2015-S31913	NEG	nsaB wildtype	NEG	POS	NEG	NEG	NEG	NEG	NEG	NEG	NEG	NEG	NEG	NEG	NEG	POS	POS	NEG
Perth_2015-S31918	NEG	nsaB wildtype	NEG	POS	NEG	NEG	NEG	NEG	NEG	NEG	NEG	NEG	NEG	NEG	NEG	POS	POS	NEG
Dublin-DSH_AR44_E1183	NEG		POS	POS	NEG	NEG	NEG	NEG	NEG	NEG	NEG	NEG	NEG	NEG	NEG	POS	POS	NEG
Dublin-DSH_Unfamiliar-3_E1520	NEG		POS	POS	NEG	NEG	NEG	NEG	NEG	NEG	NEG	NEG	NEG	NEG	NEG	POS	POS	NEG
Hong Kong_042	NEG		POS	POS	NEG	NEG	NEG	NEG	NEG	NEG	NEG	NEG	NEG	NEG	NEG	POS	POS	NEG
Hong Kong_101	NEG		POS	POS	NEG	NEG	NEG	NEG	NEG	NEG	NEG	NEG	NEG	NEG	NEG	POS	POS	NEG
Hong Kong_111	NEG		POS	POS	NEG	NEG	NEG	NEG	NEG	NEG	NEG	NEG	NEG	NEG	NEG	POS	POS	NEG
Hong Kong_113	NEG		POS	POS	NEG	NEG	NEG	NEG	NEG	NEG	NEG	NEG	NEG	NEG	NEG	POS	POS	NEG
Hong Kong_115	NEG		POS	POS	NEG	NEG	NEG	NEG	NEG	NEG	NEG	NEG	NEG	NEG	NEG	POS	POS	NEG
Hong Kong_118	NEG		POS	POS	NEG	NEG	NEG	NEG	NEG	NEG	NEG	NEG	NEG	NEG	NEG	POS	POS	NEG
Hong Kong_125	NEG		POS	POS	NEG	NEG	NEG	NEG	NEG	NEG	NEG	NEG	NEG	NEG	NEG	POS	POS	NEG
Hong Kong_129	NEG		POS	POS	NEG	NEG	NEG	NEG	NEG	NEG	NEG	NEG	NEG	NEG	NEG	POS	POS	NEG
Hong Kong_133	NEG		POS	POS	NEG	NEG	NEG	NEG	NEG	NEG	NEG	NEG	NEG	NEG	NEG	POS	POS	NEG
Kuwait_009	NEG		POS	POS	NEG	NEG	NEG	NEG	NEG	NEG	NEG	NEG	NEG	NEG	NEG	POS	POS	NEG
Kuwait_103	NEG		POS	POS	NEG	NEG	NEG	NEG	NEG	NEG	NEG	NEG	NEG	NEG	NEG	POS	POS	NEG
Kuwait_107	NEG		POS	POS	NEG	NEG	NEG	NEG	NEG	NEG	NEG	NEG	NEG	NEG	NEG	POS	POS	NEG
Perth_03 AH 88	NEG		NEG	POS	NEG	NEG	NEG	NEG	NEG	NEG	NEG	NEG	NEG	NEG	NEG	POS	POS	NEG
Perth_08-16905	NEG		POS	POS	NEG	NEG	NEG	NEG	NEG	NEG	NEG	NEG	NEG	NEG	NEG	POS	POS	NEG
Riyadh_Alfaisal-10_SICU_13_B5672_1117353	NEG		POS	POS	NEG	NEG	NEG	NEG	NEG	NEG	NEG	NEG	NEG	NEG	NEG	POS	POS	NEG
Riyadh_SSTI_17_3508602	NEG		NEG	POS	NEG	NEG	NEG	NEG	NEG	NEG	NEG	NEG	NEG	NEG	NEG	POS	POS	NEG
Riyadh-2822088-R	NEG		POS	POS	NEG	NEG	NEG	NEG	NEG	NEG	NEG	NEG	NEG	NEG	NEG	POS	POS	NEG
Trinidad&Tobago_2012_195 (619/12)	NEG		POS	POS	NEG	NEG	NEG	NEG	NEG	NEG	NEG	NEG	NEG	NEG	NEG	POS	POS	NEG
Trinidad&Tobago_2012_370 (1112-52177)	NEG		POS	POS	NEG	NEG	NEG	NEG	NEG	NEG	NEG	NEG	NEG	NEG	NEG	POS	POS	NEG
Trinidad&Tobago_2012_378 (4139)	NEG		AMB	POS	NEG	NEG	NEG	NEG	NEG	NEG	NEG	NEG	NEG	NEG	NEG	POS	POS	NEG
Trinidad&Tobago_2013_PA21	NEG		POS	POS	NEG	NEG	NEG	NEG	NEG	NEG	NEG	NEG	NEG	NEG	NEG	POS	POS	NEG
Trinidad&Tobago_2013_PA22	NEG		POS	POS	NEG	NEG	NEG	NEG	NEG	NEG	NEG	NEG	NEG	NEG	NEG	POS	POS	NEG
Trinidad&Tobago_2912_340 (8523/12)	NEG		AMB	POS	NEG	NEG	NEG	NEG	NEG	NEG	NEG	NEG	NEG	NEG	NEG	POS	POS	NEG
Trinidad&Tobago_MRSA2010_105	NEG		POS	POS	NEG	NEG	NEG	NEG	POS	NEG	AMB	NEG	NEG	NEG	NEG	POS	POS	NEG
Trinidad&Tobago_MRSA2010_108	NEG		POS	POS	NEG	NEG	NEG	NEG	NEG	NEG	NEG	NEG	NEG	NEG	NEG	POS	POS	NEG
Trinidad&Tobago_MRSA2010_33	NEG		AMB	POS	NEG	NEG	NEG	NEG	NEG	NEG	NEG	NEG	NEG	NEG	NEG	POS	POS	NEG
Trinidad&Tobago_MRSA2010_79	NEG		POS	POS	NEG	NEG	NEG	NEG	NEG	NEG	NEG	NEG	NEG	NEG	NEG	POS	POS	NEG
Trinidad&Tobago_MRSA2010_88	NEG		POS	POS	NEG	NEG	NEG	NEG	NEG	NEG	NEG	NEG	NEG	NEG	NEG	POS	POS	NEG
Trinidad&Tobago_MRSA2010_97	NEG		POS	POS	NEG	NEG	NEG	NEG	NEG	NEG	NEG	NEG	NEG	NEG	NEG	POS	POS	NEG
Trinidad&Tobago_SSTI 2012_031	NEG		POS	POS	NEG	NEG	NEG	NEG	AMB	NEG	NEG	NEG	NEG	NEG	NEG	POS	POS	NEG
Uganda-29_17148_250811	NEG		POS	POS	NEG	NEG	NEG	NEG	NEG	NEG	NEG	NEG	NEG	NEG	NEG	POS	POS	NEG
>"South-East Asian Clad																		
UCIM6015	NEG	nsaB wildtype	POS	POS	NEG	NEG	NEG	NEG	NEG	NEG	NEG	NEG	NEG	NEG	NEG	POS	POS	NEG
Hong Kong_121	NEG		POS	POS	NEG	NEG	NEG	NEG	NEG	NEG	NEG	NEG	NEG	NEG	NEG	POS	POS	NEG
>"South-East Asian Clad																		
Z172 (Taiwan)	NEG	nsaB wildtype	NEG	POS	NEG	NEG	NEG	NEG	NEG	NEG	NEG	NEG	NEG	NEG	NEG	POS	POS	NEG
Russia-19_0342_Kurgan	NEG		NEG	POS	NEG	NEG	NEG	NEG	NEG	NEG	NEG	NEG	NEG	NEG	NEG	POS	POS	NEG
>"South-East Asian Clad																		
Perth_01-15392	NEG		NEG	POS	NEG	NEG	NEG	NEG	NEG	NEG	NEG	NEG	NEG	NEG	NEG	POS	POS	NEG
>"South-East Asian Clad																		
XN108 (China)	NEG	nsaB wildtype	NEG	POS	NEG	NEG	NEG	NEG	NEG	NEG	NEG	NEG	NEG	NEG	NEG	NEG	NEG	NEG
NMR09 (India)	NEG	nsaB wildtype	NEG	POS	NEG	NEG	NEG	NEG	NEG	NEG	NEG	NEG	NEG	NEG	NEG	NEG	NEG	NEG
346 (Malaysia)	NEG	nsaB wildtype	NEG	POS	NEG	NEG	NEG	NEG	NEG	NEG	NEG	NEG	NEG	NEG	NEG	NEG	NEG	NEG
DS_009 (Thailand)	NEG	nsaB wildtype	NEG	POS	NEG	NEG	NEG	NEG	NEG	NEG	NEG	NEG	NEG	NEG	NEG	NEG	NEG	NEG
18ANR581005 (Ind)	NEG		NEG	POS	NEG	NEG	NEG	NEG	NEG	NEG	NEG	NEG	NEG	NEG	NEG	NEG	NEG	NEG
Bengalaru_ST20141405	NEG		NEG	POS	NEG	NEG	NEG	NEG	NEG	NEG	NEG	NEG	NEG	NEG	NEG	NEG	NEG	NEG
Bengalaru_ST20141406	NEG		NEG	POS	NEG	NEG	NEG	NEG	NEG	NEG	NEG	NEG	NEG	NEG	NEG	NEG	NEG	NEG
Bengalaru_ST20141417	NEG		NEG	POS	NEG	NEG	NEG	NEG	NEG	NEG	NEG	NEG	NEG	NEG	NEG	NEG	NEG	NEG
Hong Kong_107	NEG		NEG	POS	NEG	NEG	NEG	NEG	NEG	NEG	NEG	NEG	NEG	NEG	NEG	NEG	NEG	NEG
Hong Kong_12	NEG		NEG	POS	NEG	NEG	NEG	NEG	NEG	NEG	NEG	NEG	NEG	NEG	NEG	NEG	NEG	NEG
Hong Kong_132	NEG		NEG	POS	NEG	NEG	NEG	NEG	NEG	NEG	NEG	NEG	NEG	NEG	NEG	NEG	NEG	NEG
Riyadh-3028763-R	NEG		NEG	POS	NEG	NEG	NEG	NEG	NEG	NEG	NEG	NEG	NEG	NEG	NEG	NEG	NEG	NEG
Trinidad&Tobago_MRSA2010_94	NEG		NEG	POS	NEG	NEG	NEG	NEG	NEG	NEG	NEG	NEG	NEG	NEG	NEG	NEG	NEG	NEG
>"South-East Asian Clad																		
M705 (Thailand)	NEG	nsaB wildtype	POS	POS	NEG	NEG	NEG	NEG	NEG	NEG	NEG	NEG	NEG	NEG	NEG	NEG	NEG	NEG
S85 (Thailand)	NEG	nsaB wildtype	POS	POS	NEG	NEG	NEG	NEG	NEG	NEG	NEG	NEG	NEG	NEG	NEG	NEG	NEG	NEG
Riyadh_Alfaisal-04_281014_S5076	NEG		NEG	POS	NEG	NEG	NEG	NEG	NEG	NEG	NEG	NEG	NEG	NEG	NEG	NEG	NEG	NEG
Riyadh_Alfaisal-26_S15145_1104163	NEG		POS	POS	NEG	NEG	NEG	NEG	NEG	NEG	NEG	NEG	NEG	NEG	NEG	NEG	NEG	NEG
Riyadh-2817437-W	NEG		POS	POS	NEG	NEG	NEG	NEG	NEG	NEG	NEG	NEG	NEG	NEG	NEG	NEG	NEG	NEG
>"South-East Asian Clad																		
PPUKM-775-2009 (Malaysia)	NEG	nsaB wildtype	NEG	POS	NEG	NEG	NEG	NEG	NEG	NEG	NEG	NEG	NEG	NEG	NEG	NEG	NEG	NEG
Hong Kong_138	NEG		NEG	POS	NEG	NEG	NEG	NEG	NEG	NEG	NEG	NEG	NEG	NEG	NEG	NEG	NEG	NEG
>"South-East Asian Clad																		
Kuwait_001	NEG		POS	POS	NEG	NEG	NEG	NEG	NEG	NEG	NEG	NEG	NEG	NEG	NEG	POS	POS	NEG
Kuwait_337	NEG		POS	POS	NEG	NEG	NEG	NEG	NEG	NEG	NEG	NEG	NEG	NEG	NEG	POS	POS	NEG
>"South-East Asian Clad																		
CUHK_HK2007 (China)	NEG	nsaB wildtype	NEG	POS	NEG	NEG	NEG	NEG	NEG	NEG	NEG	NEG	NEG	NEG	NEG	POS	POS	NEG
NMR02 (India)	NEG	nsaB wildtype	NEG	POS	NEG	NEG	NEG	NEG	NEG	NEG	NEG	NEG	NEG	NEG	NEG	POS	POS	NEG
VB1490 (India)	NEG	nsaB wildtype	NEG	POS	NEG	NEG	NEG	NEG	NEG	NEG	NEG	NEG	NEG	NEG	NEG	POS	POS	NEG
V521 (Korea)	NEG	nsaB wildtype	NEG	POS	NEG	NEG	NEG	NEG	NEG	NEG	NEG	NEG	NEG	NEG	NEG	POS	POS	NEG
HST-077 (Lebanon)	NEG	nsaB wildtype	NEG	POS	NEG	NEG	NEG	NEG	NEG	NEG	NEG	NEG	NEG	NEG	NEG	POS	POS	NEG
MAL1 (Malaysia)	NEG	nsaB wildtype	NEG	POS	NEG	NEG	NEG	NEG	NEG	NEG	NEG	NEG	NEG	NEG	NEG	POS	POS	NEG
MAL3 (Malaysia)	NEG	nsaB wildtype	NEG	POS	NEG	NEG	NEG	NEG	NEG	NEG	NEG	NEG	NEG	NEG	NEG	POS	POS	NEG
PPUKM-261-2009 (Malaysia)	NEG	nsaB wildtype	NEG	POS	NEG	NEG	NEG	NEG	NEG	NEG	NEG	NEG	NEG	NEG	NEG	POS	POS	NEG
PPUKM-332-2009 (Malaysia)	NEG	nsaB wildtype	NEG	POS	NEG	NEG	NEG	NEG	NEG	NEG	NEG	NEG	NEG	NEG	NEG	POS	POS	NEG
S38 (Thailand)	NEG	nsaB wildtype	NEG	POS	NEG	NEG	NEG	NEG	NEG	NEG	NEG	NEG	NEG	NEG	NEG	POS	POS	NEG
S7 (Thailand)	NEG	nsaB wildtype	NEG	POS	NEG	NEG	NEG	NEG	NEG	NEG	NEG	NEG	NEG	NEG	NEG	POS	POS	NEG
US_008 (Thailand)	NEG	nsaB wildtype	NEG	POS	NEG	NEG	NEG	NEG	NEG	NEG	NEG	NEG	NEG	NEG	NEG	POS	POS	NEG
US_030 (Thailand)	NEG	nsaB wildtype	NEG	POS	NEG	NEG	NEG	NEG	NEG	NEG	NEG	NEG	NEG	NEG	NEG	POS	POS	NEG
M170 (Tsunami-related)	NEG	nsaB wildtype	NEG	POS	NEG	NEG	NEG	NEG	NEG	NEG	NEG	NEG	NEG	NEG	NEG	POS	POS	NEG
BSAC697 (UK)	NEG	nsaB wildtype	NEG	POS	NEG	NEG	NEG	NEG	NEG	NEG	NEG	NEG	NEG	NEG	NEG	POS	POS	NEG
IS-189	NEG	nsaB wildtype	NEG	POS	NEG	NEG	NEG	NEG	NEG	NEG	NEG	NEG	NEG	NEG	NEG	POS	POS	NEG
MRGR3	NEG	nsaB wildtype	NEG	POS	NEG	NEG	NEG	NEG	NEG	NEG	NEG	NEG	NEG	NEG	NEG	POS	POS	NEG
smg07	NEG	nsaB wildtype	NEG	POS	NEG	NEG	NEG	NEG	NEG	NEG	NEG	NEG	NEG	NEG	NEG	POS	POS	NEG
AH1-AUS-EMRSA-3	NEG		NEG	POS	NEG	NEG	NEG	NEG	NEG	NEG	NEG	NEG	NEG	NEG	NEG	POS	POS	NEG
Bengalaru_ST20141403	NEG		NEG	POS	NEG	NEG	NEG	NEG	NEG	NEG	NEG	NEG	NEG	NEG	NEG	POS	POS	NEG
Finland E24_98541_Harmony	NEG		NEG	POS	NEG	NEG	NEG	NEG	NEG	NEG	NEG	NEG	NEG	NEG	NEG	POS	POS	NEG
Hong Kong_10	NEG		NEG	POS	NEG	NEG	NEG	NEG	NEG	NEG	NEG	NEG	NEG	NEG	NEG	POS	POS	NEG
Hong Kong_100	NEG		NEG	POS	NEG	NEG	NEG	NEG	NEG	NEG	NEG	NEG	NEG	NEG	NEG	POS	POS	NEG
Hong Kong_199	NEG		NEG	POS	NEG	NEG	NEG	NEG	NEG	NEG	NEG	NEG	NEG	NEG	NEG	POS	POS	NEG
Hong Kong_34	NEG		NEG	POS	NEG	NEG	NEG	NEG	NEG	NEG	NEG	NEG	NEG	NEG	NEG	POS	POS	NEG
Hong Kong_88	NEG		NEG	POS	NEG	NEG	NEG	NEG	NEG	NEG	NEG	NEG	NEG	NEG	NEG	POS	POS	NEG
Hong Kong_92	NEG		NEG	POS	NEG	NEG	NEG	NEG	NEG	NEG	NEG	NEG	NEG	NEG	NEG	POS	POS	NEG
Hong Kong_94	NEG		NEG	POS	NEG	NEG	NEG	NEG	NEG	NEG	NEG	NEG	NEG	NEG	NEG	POS	POS	NEG
Kuwait_257	NEG		NEG	POS	NEG	NEG	NEG	NEG	NEG	NEG	NEG	NEG	NEG	NEG	NEG	POS	POS	NEG
Perth_01-15330	NEG		NEG	POS	NEG	NEG	NEG	NEG	NEG	NEG	NEG	NEG	NEG	NEG	NEG	POS	POS	NEG
Perth_01-15349	NEG		NEG	POS	NEG	NEG	NEG	NEG	NEG	NEG	NEG	NEG	NEG	NEG	NEG	POS	POS	NEG
Perth_01-16176	NEG		NEG	POS	NEG	NEG	NEG	NEG	NEG	NEG	NEG	NEG	NEG	NEG	NEG	POS	POS	NEG
Perth_WBG8379	NEG		NEG	POS	NEG	NEG	NEG	NEG	NEG	NEG	NEG	NEG	NEG	NEG	NEG	POS	POS	NEG
Rawalpindi_Kidney Center_56	NEG		NEG	POS	NEG	NEG	NEG	NEG	NEG	NEG	NEG	NEG	NEG	NEG	NEG	POS	POS	NEG
Riyadh_SSTI_05_3464614	NEG		NEG	POS	NEG	NEG	NEG	NEG	NEG	NEG	NEG	NEG	NEG	NEG	NEG	POS	POS	NEG
Trinidad&Tobago_2012_157	NEG		NEG	POS	NEG	NEG	NEG	NEG	NEG	NEG	NEG	NEG	NEG	NEG	NEG	POS	POS	NEG

STRAIN / ISOLATE	METHICILLIN RESISTANCE AND SCCmec TYPING															HEAVY METAL RESIST		
	Q9XB68-dcs	nsaB status	SCCmec Terminus 1	SCCmec Terminus 2	SCCmec Terminus 3	SCCmec Terminus 4	SCCmec Terminus 5	SCCmec Terminus 6	SCCmec Terminus 7	SCCmec Terminus 9	SCCmec Terminus 10	SCCmec Terminus 11	:mec Terminu	:mec Terminu	:mec Terminu	merA	merB	mco-plasmid
	Q9XB68-dcs		SCCmec Terminus 1	SCCmec Terminus 2	SCCmec Terminus 3	SCCmec Terminus 4	SCCmec Terminus 5	SCCmec Terminus 6	SCCmec Terminus 7	SCCmec Terminus 9	SCCmec Terminus 10	SCCmec Terminus 11	SCCmec Terminus 12	SCCmec Terminus 13	SCCmec Terminus 14	merA	merB	mco-plasmid (MRSA252)
	Located at the terminus of SCCmec directly next to orfX.This locus comprises the downstream constant segment (dcs) that in turn comprises a copy of the SCC direct repeat DR_SCC (AGAAGCTTATCATAAGTAA)		SCC integration site alternate to dcs													mercury resistance operon		Multi copper oxidase
>"South-East Asian Clad																		
M418	NEG	nsaB wildtype	NEG	POS	NEG	NEG	POS	NEG	NEG	NEG	NEG	NEG	NEG	NEG	NEG	POS	POS	NEG
>"South-East Asian Clad																		
NMR07 (India)	NEG	nsaB wildtype	NEG	POS	NEG	NEG	POS	NEG	NEG	NEG	NEG	NEG	NEG	NEG	NEG	NEG	NEG	NEG
NMR08 (India)	NEG	nsaB wildtype	NEG	POS	NEG	NEG	POS	NEG	NEG	NEG	NEG	NEG	NEG	NEG	NEG	NEG	NEG	NEG
Bengalaru_ST20121978	NEG		NEG	POS	NEG	NEG	POS	NEG	NEG	NEG	NEG	NEG	NEG	NEG	NEG	NEG	NEG	NEG
Hong Kong_89	NEG		NEG	POS	NEG	NEG	POS	NEG	NEG	NEG	NEG	NEG	NEG	NEG	NEG	NEG	NEG	NEG
>"South-East Asian Clad																		
Perth_2005_AH_5	NEG		NEG	NEG	AMB	NEG	POS	NEG	NEG	NEG	NEG	NEG	NEG	NEG	NEG	POS	POS	NEG
>"South-East Asian Clad																		
Trinidad&Tobago_MRSA2010_103	NEG		NEG	POS	NEG	NEG	NEG	NEG	POS	NEG	NEG	NEG	NEG	NEG	NEG	NEG	NEG	NEG
Trinidad&Tobago_2013_PA25	NEG		NEG	AMB	NEG	NEG	NEG	NEG	POS	NEG	NEG	NEG	NEG	NEG	NEG	POS	POS	NEG
Trinidad&Tobago_MRSA2010_110	NEG		NEG	POS	NEG	NEG	NEG	NEG	POS	NEG	NEG	NEG	NEG	NEG	NEG	POS	POS	NEG
Trinidad&Tobago_SSTI 2012_111	NEG		NEG	POS	NEG	NEG	NEG	NEG	POS	NEG	NEG	NEG	NEG	NEG	NEG	POS	POS	NEG
Trinidad&Tobago_MRSA2010_158	NEG		NEG	AMB	NEG	NEG	NEG	NEG	POS	NEG	NEG	NEG	NEG	NEG	NEG	NEG	NEG	NEG
>"South-East Asian Clad																		
Kuwait_122	NEG		POS	POS	NEG	NEG	NEG	NEG	NEG	NEG	NEG	NEG	NEG	NEG	NEG	POS	POS	NEG
Kuwait_002	NEG		POS	POS	NEG	NEG	NEG	NEG	NEG	NEG	NEG	NEG	NEG	NEG	NEG	POS	POS	NEG
Kuwait_196	NEG		NEG	POS	NEG	NEG	NEG	NEG	NEG	NEG	NEG	NEG	NEG	NEG	NEG	POS	POS	NEG
Kuwait_199	NEG		NEG	POS	NEG	NEG	NEG	NEG	NEG	NEG	NEG	NEG	NEG	NEG	NEG	POS	POS	NEG
Hong Kong_136	NEG		POS	POS	NEG	NEG	NEG	NEG	NEG	NEG	NEG	NEG	NEG	NEG	NEG	POS	POS	NEG
>"South-East Asian Clad																		
P32 (Poland)	NEG	nsaB wildtype	POS	NEG	NEG	NEG	NEG	NEG	NEG	NEG	NEG	NEG	NEG	NEG	NEG	NEG	NEG	NEG
>"South-East Asian Clad																		
Perth_2015-S32313	NEG	nsaB wildtype	POS	NEG	POS	NEG	NEG	NEG	NEG	NEG	NEG	NEG	NEG	NEG	NEG	NEG	NEG	NEG
>"hla-negative South-Ea																		
DEN907 (Denmark)	POS	nsaB wildtype	NEG	NEG	NEG	NEG	NEG	NEG	NEG	NEG	NEG	NEG	NEG	NEG	NEG	POS	POS	NEG
>"hla-negative South-Ea																		
S26 (Thailand)	POS	nsaB wildtype	NEG	NEG	NEG	NEG	NEG	NEG	NEG	NEG	NEG	NEG	NEG	NEG	NEG	NEG	NEG	NEG
S42 (Thailand)	POS	nsaB wildtype	NEG	NEG	NEG	NEG	NEG	NEG	NEG	NEG	NEG	NEG	NEG	NEG	NEG	NEG	NEG	NEG
S97 (Thailand)	POS	nsaB wildtype	NEG	NEG	NEG	NEG	NEG	NEG	NEG	NEG	NEG	NEG	NEG	NEG	NEG	NEG	NEG	NEG
S78 (Thailand)	POS	nsaB wildtype	NEG	NEG	NEG	NEG	NEG	NEG	NEG	NEG	NEG	NEG	NEG	NEG	NEG	NEG	NEG	NEG
S25 (Thailand)	POS	nsaB wildtype	NEG	NEG	NEG	NEG	NEG	NEG	NEG	NEG	NEG	NEG	NEG	NEG	NEG	NEG	NEG	NEG
S21 (Thailand)	POS	nsaB wildtype	NEG	NEG	NEG	NEG	NEG	NEG	NEG	NEG	NEG	NEG	NEG	NEG	NEG	NEG	NEG	NEG
S106 (Thailand)	POS	nsaB wildtype	NEG	NEG	NEG	NEG	NEG	NEG	NEG	NEG	NEG	NEG	NEG	NEG	NEG	NEG	NEG	NEG
S24 (Thailand)	POS	nsaB wildtype	NEG	NEG	NEG	NEG	NEG	NEG	NEG	NEG	NEG	NEG	NEG	NEG	NEG	NEG	NEG	NEG
S39 (Thailand)	POS	nsaB wildtype	NEG	NEG	NEG	NEG	NEG	NEG	NEG	NEG	NEG	NEG	NEG	NEG	NEG	NEG	NEG	NEG
S2 (Thailand)	POS	nsaB wildtype	NEG	NEG	NEG	NEG	NEG	NEG	NEG	NEG	NEG	NEG	NEG	NEG	NEG	NEG	NEG	NEG
S81 (Thailand)	POS	nsaB wildtype	NEG	NEG	NEG	NEG	NEG	NEG	NEG	NEG	NEG	NEG	NEG	NEG	NEG	NEG	NEG	NEG
M116 (Vietnam)	POS	nsaB wildtype	NEG	NEG	NEG	NEG	NEG	NEG	NEG	NEG	NEG	NEG	NEG	NEG	NEG	NEG	NEG	NEG
>"Portuguese Clade": Pc																		
FFP103 (Portugal)	NEG	nsaB wildtype	POS	NEG	NEG	NEG	NEG	NEG	NEG	NEG	NEG	NEG	NEG	NEG	NEG	NEG	NEG	NEG
HDG2 (Portugal)	NEG	nsaB wildtype	POS	NEG	NEG	NEG	NEG	NEG	NEG	NEG	NEG	NEG	NEG	NEG	NEG	NEG	NEG	NEG
HSA10 (Portugal)	NEG	nsaB wildtype	POS	NEG	NEG	NEG	NEG	NEG	NEG	NEG	NEG	NEG	NEG	NEG	NEG	NEG	NEG	NEG
HSA11 (Portugal)	NEG	nsaB wildtype	POS	NEG	NEG	NEG	NEG	NEG	NEG	NEG	NEG	NEG	NEG	NEG	NEG	NEG	NEG	NEG
ICP5011 (Portugal)	NEG	nsaB wildtype	POS	NEG	NEG	NEG	NEG	NEG	NEG	NEG	NEG	NEG	NEG	NEG	NEG	NEG	NEG	NEG
ICP5014 (Portugal)	NEG	nsaB wildtype	POS	NEG	NEG	NEG	NEG	NEG	NEG	NEG	NEG	NEG	NEG	NEG	NEG	NEG	NEG	NEG
ICP5062 (Portugal)	NEG	nsaB wildtype	POS	NEG	NEG	NEG	NEG	NEG	NEG	NEG	NEG	NEG	NEG	NEG	NEG	NEG	NEG	NEG
Strain 1063_ATCC 33592 FDAARGOS_3 (USA)	NEG	nsaB wildtype	POS	NEG	NEG	NEG	NEG	NEG	NEG	NEG	NEG	NEG	NEG	NEG	NEG	NEG	NEG	NEG
	NEG		POS	NEG	NEG	NEG	NEG	NEG	NEG	NEG	NEG	NEG	NEG	NEG	NEG	NEG	NEG	NEG
Russia-07_0110_Kurgan	NEG		NEG	NEG	NEG	NEG	NEG	NEG	NEG	NEG	NEG	NEG	NEG	NEG	NEG	NEG	NEG	NEG
Russia-11_0164_Kurgan	NEG		POS	NEG	NEG	NEG	NEG	NEG	NEG	NEG	NEG	NEG	NEG	NEG	NEG	NEG	NEG	NEG
Russia-20_0367_Kurgan	NEG		AMB	NEG	NEG	NEG	NEG	NEG	NEG	NEG	NEG	NEG	NEG	NEG	NEG	NEG	NEG	NEG
Russia-21_0390_Chelyabinsk	NEG		AMB	NEG	NEG	NEG	NEG	NEG	NEG	NEG	NEG	NEG	NEG	NEG	NEG	NEG	NEG	NEG
>"South American/ Mid																		
AGT1 (Argentina)	NEG	nsaB wildtype	NEG	POS	NEG	NEG	NEG	NEG	NEG	NEG	NEG	NEG	NEG	NEG	NEG	POS	POS	NEG
AGT120 (Argentina)	NEG	nsaB wildtype	NEG	POS	NEG	NEG	NEG	NEG	NEG	NEG	NEG	NEG	NEG	NEG	NEG	POS	POS	NEG
AGT67 (Argentina)	NEG	nsaB wildtype	NEG	POS	NEG	NEG	NEG	NEG	NEG	NEG	NEG	NEG	NEG	NEG	NEG	POS	POS	NEG
AGT9 (Argentina)	NEG	nsaB wildtype	NEG	POS	NEG	NEG	NEG	NEG	NEG	NEG	NEG	NEG	NEG	NEG	NEG	POS	POS	NEG
RA3 (Argentina)	NEG	nsaB wildtype	NEG	POS	NEG	NEG	NEG	NEG	NEG	NEG	NEG	NEG	NEG	NEG	NEG	POS	POS	NEG
RA6 (Argentina)	NEG	nsaB wildtype	NEG	POS	NEG	NEG	NEG	NEG	NEG	NEG	NEG	NEG	NEG	NEG	NEG	POS	POS	NEG
RA7 (Argentina)	NEG	nsaB wildtype	NEG	POS	NEG	NEG	NEG	NEG	NEG	NEG	NEG	NEG	NEG	NEG	NEG	POS	POS	NEG
Be62 (Brazil)	NEG	nsaB wildtype	NEG	POS	NEG	NEG	NEG	NEG	NEG	NEG	NEG	NEG	NEG	NEG	NEG	POS	POS	NEG
Bmb9393 (Brazil)	NEG	nsaB wildtype	NEG	POS	NEG	NEG	NEG	NEG	NEG	NEG	NEG	NEG	NEG	NEG	NEG	POS	POS	NEG
BRA36 (Brazil)	NEG	nsaB wildtype	NEG	POS	NEG	NEG	NEG	NEG	NEG	NEG	NEG	NEG	NEG	NEG	NEG	POS	POS	NEG
BZ48 (Brazil)	NEG	nsaB wildtype	NEG	POS	NEG	NEG	NEG	NEG	NEG	NEG	NEG	NEG	NEG	NEG	NEG	POS	POS	NEG
Gv51 (Brazil)	NEG	nsaB wildtype	NEG	POS	NEG	NEG	NEG	NEG	NEG	NEG	NEG	NEG	NEG	NEG	NEG	POS	POS	NEG
Gv69 (Brazil)	NEG	nsaB wildtype	NEG	POS	NEG	NEG	NEG	NEG	NEG	NEG	NEG	NEG	NEG	NEG	NEG	POS	POS	NEG
Gv88 (Brazil)	NEG	nsaB wildtype	NEG	POS	NEG	NEG	NEG	NEG	NEG	NEG	NEG	NEG	NEG	NEG	NEG	POS	POS	NEG
HC1335 (Brazil)	NEG	nsaB wildtype	NEG	POS	NEG	NEG	NEG	NEG	NEG	NEG	NEG	NEG	NEG	NEG	NEG	POS	POS	NEG
HC1340 (Brazil)	NEG	nsaB wildtype	NEG	POS	NEG	NEG	NEG	NEG	NEG	NEG	NEG	NEG	NEG	NEG	NEG	POS	POS	NEG
HC556 (Brazil)	NEG	nsaB wildtype	NEG	POS	NEG	NEG	NEG	NEG	NEG	NEG	NEG	NEG	NEG	NEG	NEG	POS	POS	NEG
HU25 (Brazil)	NEG	nsaB wildtype	NEG	POS	NEG	NEG	NEG	NEG	NEG	NEG	NEG	NEG	NEG	NEG	NEG	POS	POS	NEG
UB563 (Brazil)	NEG	nsaB wildtype	NEG	POS	NEG	NEG	NEG	NEG	NEG	NEG	NEG	NEG	NEG	NEG	NEG	POS	POS	NEG
2A8 (Czech Republic)	NEG	nsaB wildtype	NEG	POS	NEG	NEG	NEG	NEG	NEG	NEG	NEG	NEG	NEG	NEG	NEG	POS	POS	NEG
M1229 (Denmark)	NEG	nsaB wildtype	NEG	POS	NEG	NEG	NEG	NEG	NEG	NEG	NEG	NEG	NEG	NEG	NEG	POS	POS	NEG
FRICAR (France)	NEG	nsaB wildtype	NEG	POS	NEG	NEG	NEG	NEG	NEG	NEG	NEG	NEG	NEG	NEG	NEG	POS	POS	NEG
D90 (Germany)	NEG	nsaB wildtype	NEG	POS	NEG	NEG	NEG	NEG	NEG	NEG	NEG	NEG	NEG	NEG	NEG	POS	POS	NEG
LIT68 (Lithuania)	NEG	nsaB wildtype	NEG	POS	NEG	NEG	NEG	NEG	NEG	NEG	NEG	NEG	NEG	NEG	NEG	POS	POS	NEG
LIT76 (Lithuania)	NEG	nsaB wildtype	NEG	POS	NEG	NEG	NEG	NEG	NEG	NEG	NEG	NEG	NEG	NEG	NEG	POS	POS	NEG
HGSA142 (Portugal)	NEG	nsaB wildtype	NEG	POS	NEG	NEG	NEG	NEG	NEG	NEG	NEG	NEG	NEG	NEG	NEG	POS	POS	NEG
HGSA9 (Portugal)	NEG	nsaB wildtype	NEG	POS	NEG	NEG	NEG	NEG	NEG	NEG	NEG	NEG	NEG	NEG	NEG	POS	POS	NEG
HSJ216 (Portugal)	NEG	nsaB wildtype	NEG	POS	NEG	NEG	NEG	NEG	NEG	NEG	NEG	NEG	NEG	NEG	NEG	POS	POS	NEG
M278 (Portugal)	NEG	nsaB wildtype	NEG	POS	NEG	NEG	NEG	NEG	NEG	NEG	NEG	NEG	NEG	NEG	NEG	POS	POS	NEG
ES26 (Spain)	NEG	nsaB wildtype	NEG	POS	NEG	NEG	NEG	NEG	NEG	NEG	NEG	NEG	NEG	NEG	NEG	POS	POS	NEG
UK102 (UK)	NEG	nsaB wildtype	NEG	POS	NEG	NEG	NEG	NEG	NEG	NEG	NEG	NEG	NEG	NEG	NEG	POS	POS	NEG
URU34 (Uruguay)	NEG	nsaB wildtype	NEG	POS	NEG	NEG	NEG	NEG	NEG	NEG	NEG	NEG	NEG	NEG	NEG	POS	POS	NEG
IS-125	NEG	nsaB wildtype	NEG	POS	NEG	NEG	NEG	NEG	NEG	NEG	NEG	NEG	NEG	NEG	NEG	POS	POS	NEG
IS-157	NEG	nsaB wildtype	NEG	POS	NEG	NEG	NEG	NEG	NEG	NEG	NEG	NEG	NEG	NEG	NEG	POS	POS	NEG
Ecuador_1	NEG		NEG	POS	NEG	NEG	NEG	NEG	NEG	NEG	NEG	NEG	NEG	NEG	NEG	POS	POS	NEG
Ecuador_2	NEG		NEG	POS	NEG	NEG	NEG	NEG	NEG	NEG	NEG	NEG	NEG	NEG	NEG	POS	POS	NEG
Dresden_15ANR574263	NEG		NEG	POS	NEG	NEG	NEG	NEG	NEG	NEG	NEG	NEG	NEG	NEG	NEG	POS	POS	NEG
UK-EMRSA-11	NEG		NEG	POS	NEG	NEG	NEG	NEG	NEG	NEG	NEG	NEG	NEG	NEG	NEG	POS	POS	NEG
>"South American/ Mid																		
BRA2 (Brazil)	NEG	nsaB wildtype	NEG	POS	NEG	NEG	NEG	NEG	NEG	NEG	NEG	NEG	NEG	NEG	NEG	POS	POS	NEG
CHL1 (Chile)	NEG	nsaB wildtype	NEG	POS	NEG	NEG	NEG	NEG	NEG	NEG	NEG	NEG	NEG	NEG	NEG	POS	POS	NEG
CHL151 (Chile)	NEG	nsaB wildtype	NEG	POS	NEG	NEG	NEG	NEG	NEG	NEG	NEG	NEG	NEG	NEG	NEG	POS	POS	NEG
UCL378 (Chile)	NEG	nsaB wildtype	NEG	POS	NEG	NEG	NEG	NEG	NEG	NEG	NEG	NEG	NEG	NEG	NEG	POS	POS	NEG
UP72 (Peru)	NEG	nsaB wildtype	NEG	POS	NEG	NEG	NEG	NEG	NEG	NEG	NEG	NEG	NEG	NEG	NEG	POS	POS	NEG
UP81 (Peru)	NEG	nsaB wildtype	NEG	POS	NEG	NEG	NEG	NEG	NEG	NEG	NEG	NEG	NEG	NEG	NEG	POS	POS	NEG
>"South American/ Mid																		
UP1073 (Peru)	NEG	nsaB wildtype	NEG	POS	NEG	NEG	NEG	NEG	NEG	NEG	NEG	NEG	NEG	NEG	NEG	POS	POS	NEG

STRAIN / ISOLATE	METHICILLIN RESISTANCE AND SCCmec TYPING															HEAVY METAL RESIST		
	Q9XB68-dcs	nsaB status	SCCmec Terminus 1	SCCmec Terminus 2	SCCmec Terminus 3	SCCmec Terminus 4	SCCmec Terminus 5	SCCmec Terminus 6	SCCmec Terminus 7	SCCmec Terminus 9	SCCmec Terminus 10	SCCmec Terminus 11	mec Terminu	mec Terminu	mec Terminu	merA	merB	mco-plasmid
	Q9XB68-dcs		SCCmec Terminus 1	SCCmec Terminus 2	SCCmec Terminus 3	SCCmec Terminus 4	SCCmec Terminus 5	SCCmec Terminus 6	SCCmec Terminus 7	SCCmec Terminus 9	SCCmec Terminus 10	SCCmec Terminus 11	SCCmec Terminus 12	SCCmec Terminus 13	SCCmec Terminus 14	merA	merB	mco-plasmid (MRSA252)
	Located at the terminus of SCCmec directly next to orfX.This locus comprises the downstream constant segment (dcs) that in turn comprises a copy of the SCC direct repeat DR_SCC (AGAAGCTTATCATAAGTAA)		SCC integration site alternate to dcs													mercury resistance operon		Multi copper oxidase
>"South American/ Mid																		
LIT89 (Lithuania)	NEG	nsaB wildtype	NEG	POS	NEG	NEG	NEG	NEG	NEG	NEG	NEG	NEG	NEG	NEG	NEG	NEG	NEG	NEG
MRSA_PR1 (Malaysia)	NEG	nsaB wildtype	NEG	POS	NEG	NEG	NEG	NEG	NEG	NEG	NEG	NEG	NEG	NEG	NEG	NEG	NEG	NEG
Riyadh-2888905-R	NEG		NEG	POS	NEG	NEG	NEG	NEG	NEG	NEG	NEG	NEG	NEG	NEG	NEG	NEG	NEG	NEG
Riyadh-2888915-BC	NEG		NEG	POS	NEG	NEG	NEG	NEG	NEG	NEG	NEG	NEG	NEG	NEG	NEG	NEG	NEG	NEG
Riyadh-2793706-R	NEG		NEG	POS	NEG	NEG	NEG	NEG	neg	NEG	neg	NEG	NEG	NEG	NEG	NEG	NEG	NEG
>"South American/ Mid																		
Riyadh_SSTI_18_3502925	NEG		AMB	POS	NEG	NEG	NEG	NEG	NEG	NEG	NEG	NEG	NEG	NEG	NEG	NEG	NEG	NEG
Riyadh-2822825-W	NEG		POS	POS	NEG	NEG	NEG	NEG	NEG	NEG	NEG	NEG	NEG	NEG	NEG	NEG	NEG	NEG
>"South American/ Mid																		
UK-EMRSA-9	NEG		NEG	POS	NEG	NEG	POS	NEG	NEG	NEG	NEG	NEG	NEG	NEG	NEG	POS	POS	NEG
>"South American/ Mid																		
Lome_HT20020815	NEG		NEG	NEG	NEG	NEG	POS	NEG	NEG	NEG	NEG	NEG	NEG	NEG	NEG	POS	POS	NEG
>"South American/ Mid																		
NA32 (Denmark)	NEG	nsaB wildtype	POS	POS	NEG	NEG	NEG	NEG	NEG	NEG	NEG	NEG	NEG	NEG	NEG	POS	POS	NEG
H24 (Egypt)	NEG	nsaB wildtype	POS	POS	NEG	NEG	NEG	NEG	NEG	NEG	NEG	NEG	NEG	NEG	NEG	POS	POS	NEG
ATCC BAA-39 (=HUSA304) (Hungary)	NEG	nsaB wildtype	POS	POS	NEG	NEG	NEG	NEG	NEG	NEG	NEG	NEG	NEG	NEG	NEG	POS	POS	NEG
HUSA304 (Hungary)	NEG	nsaB wildtype	POS	POS	NEG	NEG	NEG	NEG	NEG	NEG	NEG	NEG	NEG	NEG	NEG	POS	POS	NEG
HU106 (Hungary)	NEG	nsaB wildtype	POS	POS	NEG	NEG	NEG	NEG	NEG	NEG	NEG	NEG	NEG	NEG	NEG	POS	POS	NEG
BSAC27 (UK)	NEG	nsaB wildtype	POS	POS	NEG	NEG	NEG	NEG	NEG	NEG	NEG	NEG	NEG	NEG	NEG	POS	POS	NEG
NCTC13131, UK-EMRSA-4 (UK)	NEG		POS	POS	NEG	NEG	NEG	NEG	NEG	NEG	NEG	NEG	NEG	NEG	NEG	POS	POS	NEG
	NEG		POS	POS	NEG	NEG	NEG	NEG	NEG	NEG	NEG	NEG	NEG	NEG	NEG	POS	POS	NEG
Algiers_HT20040080	NEG		POS	POS	NEG	NEG	NEG	NEG	NEG	NEG	NEG	NEG	NEG	NEG	NEG	POS	POS	NEG
Dublin-DSH_AR09_0.0066	NEG		AMB	POS	NEG	NEG	NEG	NEG	NEG	NEG	NEG	NEG	NEG	NEG	NEG	POS	POS	NEG
Dublin-DSH_AR09_0-0065	NEG		POS	POS	NEG	NEG	NEG	NEG	NEG	NEG	NEG	NEG	NEG	NEG	NEG	POS	POS	NEG
Dublin-DSH_Phenotype-III_84	NEG		POS	POS	NEG	NEG	NEG	NEG	NEG	NEG	NEG	NEG	NEG	NEG	NEG	POS	POS	NEG
Hong Kong_130	NEG		POS	POS	NEG	NEG	NEG	NEG	NEG	NEG	NEG	NEG	NEG	NEG	NEG	POS	POS	NEG
Kuwait_018	NEG		POS	POS	NEG	NEG	NEG	NEG	NEG	NEG	NEG	NEG	NEG	NEG	NEG	POS	POS	NEG
Perth_08-17726	NEG		POS	POS	NEG	NEG	NEG	NEG	NEG	NEG	NEG	NEG	NEG	NEG	NEG	POS	POS	NEG
Riyadh_Alfaisal/KKKSUH_86_MRS-14-279	NEG		POS	POS	NEG	NEG	NEG	NEG	NEG	NEG	NEG	NEG	NEG	NEG	NEG	POS	POS	NEG
Riyadh_Alfaisal-04_Z3861_831588	NEG		POS	POS	NEG	NEG	NEG	NEG	NEG	NEG	NEG	NEG	NEG	NEG	NEG	POS	POS	NEG
Riyadh_Alfaisal-30_S15724_1108013	NEG		POS	POS	NEG	NEG	NEG	NEG	NEG	NEG	NEG	NEG	NEG	NEG	NEG	POS	POS	NEG
Riyadh_Alfaisal-6_22A_13_B3992_397721	NEG		POS	POS	NEG	NEG	NEG	NEG	NEG	NEG	NEG	NEG	NEG	NEG	NEG	POS	POS	NEG
Riyadh_SSTI_52_3615482	NEG		AMB	POS	NEG	NEG	NEG	NEG	NEG	NEG	NEG	NEG	NEG	NEG	NEG	POS	POS	NEG
Riyadh-2817276-2	NEG		POS	POS	NEG	NEG	NEG	NEG	NEG	NEG	NEG	NEG	NEG	NEG	NEG	POS	POS	NEG
Riyadh-2891670-W	NEG		POS	POS	NEG	NEG	NEG	NEG	NEG	NEG	NEG	NEG	NEG	NEG	NEG	POS	POS	NEG
Riyadh-3006920-W	NEG		POS	POS	NEG	NEG	NEG	NEG	NEG	NEG	NEG	NEG	NEG	NEG	NEG	POS	POS	NEG
Riyadh-R2567782	NEG		POS	POS	NEG	NEG	NEG	NEG	NEG	NEG	NEG	NEG	NEG	NEG	NEG	POS	POS	NEG
Russia-18_0252_Krasnoyarsk_SK2	NEG		POS	POS	NEG	NEG	NEG	NEG	NEG	NEG	NEG	NEG	NEG	NEG	NEG	POS	POS	NEG
UK-EMRSA-7	NEG		POS	POS	NEG	NEG	NEG	NEG	NEG	NEG	NEG	NEG	NEG	NEG	NEG	POS	POS	NEG
>"South American/ Mid																		
Dublin-DSH_AR23_0073	NEG		POS	POS	NEG	NEG	NEG	NEG	NEG	NEG	NEG	NEG	NEG	NEG	NEG	POS	POS	NEG
>Related to "South Ame																		
MRSA-OC3 (Russia)	NEG	nsaB wildtype	NEG	POS	NEG	NEG	NEG	NEG	NEG	NEG	NEG	NEG	NEG	NEG	NEG	POS	POS	NEG
Russia-12_0176_Krasnoyarsk	NEG		NEG	POS	NEG	NEG	NEG	NEG	NEG	NEG	NEG	NEG	NEG	NEG	NEG	POS	POS	NEG
Russia-13_0180_Krasnoyarsk	NEG		NEG	POS	NEG	NEG	NEG	NEG	NEG	NEG	NEG	NEG	NEG	NEG	NEG	POS	POS	NEG
Russia-16_0249_Krasnoyarsk	NEG		NEG	POS	NEG	NEG	NEG	NEG	NEG	NEG	NEG	NEG	NEG	NEG	NEG	POS	POS	NEG
Russia-17_0250_Krasnoyarsk	NEG		NEG	POS	NEG	NEG	NEG	NEG	NEG	NEG	NEG	NEG	NEG	NEG	NEG	POS	POS	NEG
>Related to "South Ame																		
URU110 (Uruguay)	NEG	nsaB wildtype	NEG	POS	NEG	NEG	NEG	NEG	NEG	NEG	NEG	NEG	NEG	NEG	NEG	POS	POS	NEG
>Related to "South Ame																		
DS_014 (Thailand)	NEG	nsaB wildtype	NEG	POS	NEG	NEG	NEG	NEG	NEG	NEG	NEG	NEG	NEG	NEG	NEG	NEG	NEG	NEG
>Unassigned Middle Eas																		
Frankfurt_Oder_0490031797 (Libya)	NEG		AMB	NEG	NEG	NEG	NEG	NEG	NEG	NEG	NEG	NEG	NEG	NEG	NEG	NEG	NEG	NEG
Kuwait_192	NEG		AMB	NEG	NEG	NEG	NEG	NEG	NEG	NEG	NEG	NEG	NEG	NEG	NEG	NEG	NEG	NEG
Riyadh-9	NEG		POS	NEG	NEG	NEG	NEG	NEG	NEG	NEG	NEG	NEG	NEG	NEG	NEG	NEG	NEG	NEG
Russia-24_0407_Moscow	NEG		NEG	NEG	NEG	NEG	NEG	NEG	NEG	NEG	NEG	NEG	NEG	NEG	NEG	NEG	NEG	NEG

STRAIN / ISOLATE	HEAVY METAL RESIST																
	copA2-plasmid	copA2-SCC	mco-SCC	arsA	arsD	arsD2	arsB				arsC					czrB	czrC
	copA2_pla	copA2-SCC	mco-SCC	arsA	arsD	arsD2	arsB (SCC)			arsB (chromosomal)	arsC (chromosomal)	arsC (SCC or plasmidic)				czrB	czrC
	Copper exporting ATPase	Copper exporting ATPase	Multi copper oxidase	arsenical pump-driving ATPase	Putative dehydrogenase	trans-acting repressor of arsenic resistance operon	arsenical pump membrane protein				arsenate reductase					zink and cobalt transporter protein	cadmium and zinc resistance gene C, heavy metal translocating P-type ATPase. Frequently associated with SCCmec elements from livestock MRSA
**>"Eurasian Clade": TUR1**																	
TUR1 (Turkey)	NEG	NEG	NEG	NEG	NEG	NEG	NEG	NEG	NEG	POS	POS	NEG	NEG	NEG	NEG	POS	NEG
TUR9 (Turkey)	NEG	NEG	NEG	NEG	NEG	NEG	NEG	NEG	NEG	POS	POS	NEG	NEG	NEG	NEG	POS	NEG
**>"Eurasian Clade" : T013**																	
T0131 (China)	NEG	NEG	NEG	NEG	NEG	NEG	NEG	NEG	NEG	POS	POS	NEG	NEG	NEG	NEG	POS	NEG
MU4 (Turkey)	NEG	NEG	NEG	NEG	NEG	NEG	NEG	NEG	NEG	POS	POS	NEG	NEG	NEG	NEG	POS	NEG
DEU11 (Turkey)	NEG	NEG	NEG	NEG	NEG	NEG	NEG	NEG	NEG	POS	POS	NEG	NEG	NEG	NEG	POS	NEG
DEU16 (Turkey)	NEG	NEG	NEG	NEG	NEG	NEG	NEG	NEG	NEG	POS	POS	NEG	NEG	NEG	NEG	POS	NEG
Romania_Iasi_MRSA-07_54	NEG	NEG	NEG	NEG	NEG	NEG	NEG	NEG	NEG	POS	POS	NEG	NEG	NEG	NEG	POS	NEG
**>"Eurasian Clade": arsC-**																	
Romania_Iasi_MRSA-05_46	NEG	NEG	NEG	NEG	NEG	NEG	NEG	NEG	NEG	POS	POS	POS	NEG	POS	NEG	POS	NEG
Romania_Iasi_MRSA-06_47	NEG	NEG	NEG	NEG	NEG	NEG	NEG	NEG	NEG	POS	POS	POS	NEG	AMB	NEG	POS	NEG
**>"Eurasian Clade": 16K/**																	
CN79 (China)	NEG	NEG	NEG	NEG	NEG	NEG	NEG	NEG	NEG	POS	POS	NEG	NEG	NEG	NEG	POS	NEG
CUHK_BJ2002 (China)	NEG	NEG	NEG	NEG	NEG	NEG	NEG	NEG	NEG	POS	POS	NEG	NEG	NEG	NEG	POS	NEG
CUHK_BJ2007 (China)	NEG	NEG	NEG	NEG	NEG	NEG	NEG	NEG	NEG	POS	POS	NEG	NEG	NEG	NEG	POS	NEG
3HK (Czech Republic)	NEG	NEG	NEG	NEG	NEG	NEG	NEG	NEG	NEG	POS	POS	NEG	NEG	NEG	NEG	POS	NEG
HU109 (Hungary)	NEG	NEG	NEG	NEG	NEG	NEG	NEG	NEG	NEG	POS	POS	NEG	NEG	NEG	NEG	POS	NEG
HUR18 (Hungary)	NEG	NEG	NEG	NEG	NEG	NEG	NEG	NEG	NEG	POS	POS	NEG	NEG	NEG	NEG	POS	NEG
H482 (Romania)	NEG	NEG	NEG	NEG	NEG	NEG	NEG	NEG	NEG	POS	POS	NEG	NEG	NEG	NEG	POS	NEG
16K (Russia)	NEG	NEG	NEG	NEG	NEG	NEG	NEG	NEG	NEG	POS	POS	NEG	NEG	NEG	NEG	POS	NEG
DEU10 (Turkey)	NEG	NEG	NEG	NEG	NEG	NEG	NEG	NEG	NEG	POS	POS	NEG	NEG	NEG	NEG	POS	NEG
DEU12 (Turkey)	NEG	NEG	NEG	NEG	NEG	NEG	NEG	NEG	NEG	POS	POS	NEG	NEG	NEG	NEG	POS	NEG
DEU14 (Turkey)	NEG	NEG	NEG	NEG	NEG	NEG	NEG	NEG	NEG	POS	POS	NEG	NEG	NEG	NEG	POS	NEG
DEU15 (Turkey)	NEG	NEG	NEG	NEG	NEG	NEG	NEG	NEG	NEG	POS	POS	NEG	NEG	NEG	NEG	POS	NEG
DEU17 (Turkey)	NEG	NEG	NEG	NEG	NEG	NEG	NEG	NEG	NEG	POS	POS	NEG	NEG	NEG	NEG	POS	NEG
DEU19 (Turkey)	NEG	NEG	NEG	NEG	NEG	NEG	NEG	NEG	NEG	POS	POS	NEG	NEG	NEG	NEG	POS	NEG
DEU2 (Turkey)	NEG	NEG	NEG	NEG	NEG	NEG	NEG	NEG	NEG	POS	POS	NEG	NEG	NEG	NEG	POS	NEG
DEU20 (Turkey)	NEG	NEG	NEG	NEG	NEG	NEG	NEG	NEG	NEG	POS	POS	NEG	NEG	NEG	NEG	POS	NEG
DEU23 (Turkey)	NEG	NEG	NEG	NEG	NEG	NEG	NEG	NEG	NEG	POS	POS	NEG	NEG	NEG	NEG	POS	NEG
DEU3 (Turkey)	NEG	NEG	NEG	NEG	NEG	NEG	NEG	NEG	NEG	POS	POS	NEG	NEG	NEG	NEG	POS	NEG
DEU5 (Turkey)	NEG	NEG	NEG	NEG	NEG	NEG	NEG	NEG	NEG	POS	POS	NEG	NEG	NEG	NEG	POS	NEG
DEU6 (Turkey)	NEG	NEG	NEG	NEG	NEG	NEG	NEG	NEG	NEG	POS	POS	NEG	NEG	NEG	NEG	POS	NEG
DEU8 (Turkey)	NEG	NEG	NEG	NEG	NEG	NEG	NEG	NEG	NEG	POS	POS	NEG	NEG	NEG	NEG	POS	NEG
HU11 (Turkey)	NEG	NEG	NEG	NEG	NEG	NEG	NEG	NEG	NEG	POS	POS	NEG	NEG	NEG	NEG	POS	NEG
HU13 (Turkey)	NEG	NEG	NEG	NEG	NEG	NEG	NEG	NEG	NEG	POS	POS	NEG	NEG	NEG	NEG	POS	NEG
HU14 (Turkey)	NEG	NEG	NEG	NEG	NEG	NEG	NEG	NEG	NEG	POS	POS	NEG	NEG	NEG	NEG	POS	NEG
HU15 (Turkey)	NEG	NEG	NEG	NEG	NEG	NEG	NEG	NEG	NEG	POS	POS	NEG	NEG	NEG	NEG	POS	NEG
HU16 (Turkey)	NEG	NEG	NEG	NEG	NEG	NEG	NEG	NEG	NEG	POS	POS	NEG	NEG	NEG	NEG	POS	NEG
HU17 (Turkey)	NEG	NEG	NEG	NEG	NEG	NEG	NEG	NEG	NEG	POS	POS	NEG	NEG	NEG	NEG	POS	NEG
HU21 (Turkey)	NEG	NEG	NEG	NEG	NEG	NEG	NEG	NEG	NEG	POS	POS	NEG	NEG	NEG	NEG	POS	NEG
HU23 (Turkey)	NEG	NEG	NEG	NEG	NEG	NEG	NEG	NEG	NEG	POS	POS	NEG	NEG	NEG	NEG	POS	NEG
HU26 (Turkey)	NEG	NEG	NEG	NEG	NEG	NEG	NEG	NEG	NEG	POS	POS	NEG	NEG	NEG	NEG	POS	NEG
HU4 (Turkey)	NEG	NEG	NEG	NEG	NEG	NEG	NEG	NEG	NEG	POS	POS	NEG	NEG	NEG	NEG	POS	NEG
HU5 (Turkey)	NEG	NEG	NEG	NEG	NEG	NEG	NEG	NEG	NEG	POS	POS	NEG	NEG	NEG	NEG	POS	NEG
HU6 (Turkey)	NEG	NEG	NEG	NEG	NEG	NEG	NEG	NEG	NEG	POS	POS	NEG	NEG	NEG	NEG	POS	NEG
HU7 (Turkey)	NEG	NEG	NEG	NEG	NEG	NEG	NEG	NEG	NEG	POS	POS	NEG	NEG	NEG	NEG	POS	NEG
HU8 (Turkey)	NEG	NEG	NEG	NEG	NEG	NEG	NEG	NEG	NEG	POS	POS	NEG	NEG	NEG	NEG	POS	NEG
HU9 (Turkey)	NEG	NEG	NEG	NEG	NEG	NEG	NEG	NEG	NEG	POS	POS	NEG	NEG	NEG	NEG	POS	NEG
IU1 (Turkey)	NEG	NEG	NEG	NEG	NEG	NEG	NEG	NEG	NEG	POS	POS	NEG	NEG	NEG	NEG	POS	NEG
IU10 (Turkey)	NEG	NEG	NEG	NEG	NEG	NEG	NEG	NEG	NEG	POS	POS	NEG	NEG	NEG	NEG	POS	NEG
IU11 (Turkey)	NEG	NEG	NEG	NEG	NEG	NEG	NEG	NEG	NEG	POS	POS	NEG	NEG	NEG	NEG	POS	NEG
IU12 (Turkey)	NEG	NEG	NEG	NEG	NEG	NEG	NEG	NEG	NEG	POS	POS	NEG	NEG	NEG	NEG	POS	NEG
IU13 (Turkey)	NEG	NEG	NEG	NEG	NEG	NEG	NEG	NEG	NEG	POS	POS	NEG	NEG	NEG	NEG	POS	NEG
IU15 (Turkey)	NEG	NEG	NEG	NEG	NEG	NEG	NEG	NEG	NEG	POS	POS	NEG	NEG	NEG	NEG	POS	NEG
IU18 (Turkey)	NEG	NEG	NEG	NEG	NEG	NEG	NEG	NEG	NEG	POS	POS	NEG	NEG	NEG	NEG	POS	NEG
IU19 (Turkey)	NEG	NEG	NEG	NEG	NEG	NEG	NEG	NEG	NEG	POS	POS	NEG	NEG	NEG	NEG	POS	NEG
IU2 (Turkey)	NEG	NEG	NEG	NEG	NEG	NEG	NEG	NEG	NEG	POS	POS	NEG	NEG	NEG	NEG	POS	NEG
IU4 (Turkey)	NEG	NEG	NEG	NEG	NEG	NEG	NEG	NEG	NEG	POS	POS	NEG	NEG	NEG	NEG	POS	NEG
IU6 (Turkey)	NEG	NEG	NEG	NEG	NEG	NEG	NEG	NEG	NEG	POS	POS	NEG	NEG	NEG	NEG	POS	NEG
IU7 (Turkey)	NEG	NEG	NEG	NEG	NEG	NEG	NEG	NEG	NEG	POS	POS	NEG	NEG	NEG	NEG	POS	NEG
IU9 (Turkey)	NEG	NEG	NEG	NEG	NEG	NEG	NEG	NEG	NEG	POS	POS	NEG	NEG	NEG	NEG	POS	NEG
MU1 (Turkey)	NEG	NEG	NEG	NEG	NEG	NEG	NEG	NEG	NEG	POS	POS	NEG	NEG	NEG	NEG	POS	NEG
MU10 (Turkey)	NEG	NEG	NEG	NEG	NEG	NEG	NEG	NEG	NEG	POS	POS	NEG	NEG	NEG	NEG	POS	NEG
MU20 (Turkey)	NEG	NEG	NEG	NEG	NEG	NEG	NEG	NEG	NEG	POS	POS	NEG	NEG	NEG	NEG	POS	NEG
Mu3 (Turkey)	NEG	NEG	NEG	NEG	NEG	NEG	NEG	NEG	NEG	POS	POS	NEG	NEG	NEG	NEG	POS	NEG
MU5 (Turkey)	NEG	NEG	NEG	NEG	NEG	NEG	NEG	NEG	NEG	POS	POS	NEG	NEG	NEG	NEG	POS	NEG
MU6 (Turkey)	NEG	NEG	NEG	NEG	NEG	NEG	NEG	NEG	NEG	POS	POS	NEG	NEG	NEG	NEG	POS	NEG
MU7 (Turkey)	NEG	NEG	NEG	NEG	NEG	NEG	NEG	NEG	NEG	POS	POS	NEG	NEG	NEG	NEG	POS	NEG
TUR27 (Turkey)	NEG	NEG	NEG	NEG	NEG	NEG	NEG	NEG	NEG	POS	POS	NEG	NEG	NEG	NEG	POS	NEG
NCTR 32S	NEG	NEG	NEG	NEG	NEG	NEG	NEG	NEG	NEG	POS	POS	NEG	NEG	NEG	NEG	POS	NEG
SA02_A4 = Russia-06_0085_Moscow	NEG	NEG	NEG	NEG	NEG	NEG	NEG	NEG	NEG	POS	POS	NEG	NEG	NEG	NEG	POS	NEG
	NEG	NEG	NEG	NEG	NEG	NEG	NEG	NEG	NEG	POS	POS	NEG	NEG	NEG	NEG	POS	NEG
Dresden_08V35987 (Turkey)	NEG	NEG	NEG	NEG	NEG	NEG	NEG	NEG	NEG	POS	POS	NEG	NEG	NEG	NEG	POS	NEG
Dresden_17ANR577152	NEG	NEG	NEG	NEG	NEG	NEG	NEG	NEG	NEG	POS	POS	NEG	NEG	NEG	NEG	POS	NEG
Dresden_17ANR580374 (Makedonia)	NEG	NEG	NEG	NEG	NEG	NEG	NEG	NEG	NEG	POS	POS	NEG	NEG	NEG	NEG	POS	NEG
Hong Kong_69-II	NEG	NEG	NEG	NEG	NEG	NEG	NEG	NEG	NEG	POS	POS	NEG	NEG	NEG	NEG	POS	NEG
Hong Kong_93	NEG	NEG	NEG	NEG	NEG	NEG	NEG	NEG	NEG	POS	POS	NEG	AMB	NEG	NEG	POS	NEG
Rawalpindi_Kidney Center_03_SK1	NEG	NEG	NEG	NEG	NEG	NEG	NEG	NEG	NEG	POS	POS	NEG	NEG	NEG	NEG	POS	NEG
Rawalpindi_Kidney Center_08	NEG	NEG	NEG	NEG	NEG	NEG	NEG	NEG	NEG	POS	POS	NEG	NEG	NEG	NEG	POS	NEG
Rawalpindi_Kidney Center_10	NEG	neg	NEG	NEG	NEG	NEG	NEG	NEG	NEG	POS	POS	NEG	NEG	NEG	NEG	POS	NEG
Rawalpindi_Kidney Center_19	NEG	NEG	NEG	NEG	NEG	NEG	NEG	NEG	NEG	POS	POS	NEG	NEG	NEG	NEG	POS	NEG
Rawalpindi_Kidney Center_50_SK1	NEG	NEG	NEG	NEG	NEG	NEG	NEG	NEG	NEG	POS	POS	NEG	NEG	NEG	NEG	POS	NEG
Romania_Iasi_BC-49_430	NEG	NEG	NEG	NEG	NEG	NEG	NEG	NEG	NEG	POS	POS	NEG	NEG	NEG	NEG	POS	NEG
Romania_Iasi_MRSA_284	NEG	NEG	NEG	NEG	NEG	NEG	NEG	NEG	NEG	POS	POS	NEG	NEG	NEG	NEG	POS	NEG
Romania_Iasi_MRSA-32_318	NEG	NEG	NEG	NEG	NEG	NEG	NEG	NEG	NEG	POS	POS	NEG	NEG	NEG	NEG	POS	NEG
Romania_Iasi_MRSA-54_430	NEG	NEG	NEG	NEG	NEG	NEG	NEG	NEG	NEG	POS	POS	NEG	NEG	NEG	NEG	POS	NEG
Romania_Iasi_SSTI-01_2	NEG	NEG	NEG	NEG	NEG	NEG	NEG	NEG	NEG	POS	POS	NEG	NEG	NEG	NEG	POS	NEG
Romania_Iasi_SSTI-10_101	NEG	NEG	NEG	NEG	NEG	NEG	NEG	NEG	NEG	POS	POS	NEG	NEG	NEG	NEG	POS	NEG
Romania_Iasi_SSTI-11_106	NEG	NEG	NEG	NEG	NEG	NEG	NEG	NEG	NEG	POS	POS	NEG	NEG	NEG	NEG	POS	NEG
Russia-01_0001_SaintPetersburg	NEG	NEG	NEG	NEG	NEG	NEG	NEG	NEG	NEG	POS	POS	NEG	NEG	NEG	NEG	POS	NEG
Russia-02_0004_SaintPetersburg	NEG	NEG	NEG	NEG	NEG	NEG	NEG	NEG	NEG	POS	POS	NEG	NEG	NEG	NEG	POS	NEG
Russia-03_0057_SaintPetersburg	NEG	NEG	NEG	NEG	NEG	NEG	NEG	NEG	NEG	POS	POS	NEG	NEG	NEG	NEG	POS	NEG
Russia-04_0076_Moscow	NEG	NEG	NEG	NEG	NEG	NEG	NEG	NEG	NEG	POS	POS	NEG	NEG	NEG	NEG	POS	NEG
Russia-05_0078_Moscow	NEG	NEG	NEG	NEG	NEG	NEG	NEG	NEG	NEG	POS	POS	NEG	NEG	NEG	NEG	POS	NEG
Russia-08_0135_Moscow	NEG	NEG	NEG	NEG	NEG	NEG	NEG	NEG	NEG	POS	POS	NEG	NEG	NEG	NEG	POS	NEG
Russia-09_0150_Kurgan	NEG	NEG	NEG	NEG	NEG	NEG	NEG	NEG	NEG	POS	POS	NEG	NEG	NEG	NEG	POS	NEG
Russia-10_0162_Kurgan	NEG	NEG	NEG	NEG	NEG	NEG	NEG	NEG	NEG	POS	POS	NEG	NEG	NEG	NEG	POS	NEG
Russia-14_0184_Moscow	NEG	NEG	NEG	NEG	NEG	NEG	NEG	NEG	NEG	POS	POS	NEG	NEG	NEG	NEG	POS	NEG
Russia-15_0232_SaintPetersburg	NEG	NEG	NEG	NEG	NEG	NEG	NEG	NEG	NEG	POS	POS	NEG	NEG	NEG	NEG	POS	NEG
Russia-22_0391_Chelyabinsk	NEG	NEG	NEG	NEG	NEG	NEG	NEG	NEG	NEG	POS	POS	NEG	NEG	NEG	NEG	POS	NEG
Russia-23_0392_Chelyabinsk	NEG	NEG	NEG	NEG	NEG	NEG	NEG	NEG	NEG	POS	POS	NEG	NEG	NEG	NEG	POS	NEG
**>"Eurasian Clade" : IU17**																	
IU17 (Turkey)	NEG	NEG	NEG	NEG	NEG	NEG	NEG	NEG	NEG	POS	POS	NEG	NEG	NEG	NEG	POS	NEG
IU20 (Turkey)	NEG	NEG	NEG	NEG	NEG	NEG	NEG	NEG	NEG	POS	POS	NEG	NEG	NEG	NEG	POS	NEG

STRAIN / ISOLATE	HEAVY METAL RESIST																
	copA2-plasmid	copA2-SCC	mco-SCC	arsA	arsD	arsD2	arsB				arsC					czrB	czrC
	copA2_pla	copA2-SCC	mco-SCC	arsA	arsD	arsD2	arsB (SCC)			arsB (chromosomal)	arsC (chromosomal)	arsC (SCC or plasmidic)				czrB	czrC
	Copper exporting ATPase	Copper exporting ATPase	Multi copper oxidase	arsenical pump-driving ATPase	Putative dehydrogenase	trans-acting repressor of arsenic resistance operon	arsenical pump membrane protein				arsenate reductase					zink and cobalt transporter protein	cadmium and zinc resistance gene C, heavy metal translocating P-type ATPase. Frequently associated with SCCmec elements from livestock MRSA
>"Eurasian Clade": Spora																	
Dresden_16ANR581769	NEG	NEG	NEG	NEG	NEG	NEG	NEG	NEG	NEG	POS	POS	NEG	NEG	NEG	NEG	POS	NEG
Dresden_17ANR578769	NEG	NEG	NEG	NEG	NEG	NEG	NEG	NEG	NEG	POS	POS	NEG	NEG	NEG	NEG	POS	NEG
>"Eurasian Clade": Kuwa																	
Kuwait_306	NEG	NEG	NEG	NEG	NEG	NEG	NEG	NEG	NEG	POS	POS	NEG	NEG	NEG	NEG	POS	NEG
Kuwait_301	NEG	NEG	NEG	NEG	NEG	NEG	NEG	NEG	NEG	POS	POS	NEG	NEG	NEG	NEG	POS	NEG
>"European Clade": "Gre																	
GRE18 (Greece)	NEG	NEG	NEG	NEG	NEG	NEG	NEG	NEG	NEG	POS	POS	NEG	NEG	NEG	NEG	POS	NEG
GRE317 (Greece)	NEG	NEG	NEG	NEG	NEG	NEG	NEG	NEG	NEG	POS	POS	NEG	NEG	NEG	NEG	POS	NEG
GRE4 (Greece)	NEG	NEG	NEG	NEG	NEG	NEG	NEG	NEG	NEG	POS	POS	NEG	NEG	NEG	NEG	POS	NEG
R35 (USA)	NEG	NEG	NEG	NEG	NEG	NEG	NEG	NEG	NEG	POS	POS	NEG	NEG	NEG	NEG	POS	NEG
Casablanca_HT20060548	NEG	NEG	NEG	NEG	NEG	NEG	NEG	NEG	NEG	POS	POS	NEG	NEG	NEG	NEG	POS	NEG
Casablanca_HT20060550	NEG	neg	NEG	NEG	NEG	NEG	NEG	NEG	NEG	POS	POS	NEG	NEG	NEG	NEG	POS	NEG
Dresden_01V34572 (Greece)	NEG	NEG	NEG	NEG	NEG	NEG	NEG	NEG	NEG	POS	POS	NEG	NEG	NEG	NEG	POS	NEG
Dresden_01V36469	NEG	NEG	NEG	NEG	NEG	NEG	NEG	NEG	NEG	POS	POS	NEG	NEG	NEG	NEG	POS	NEG
Dresden_01V39123	NEG	NEG	NEG	NEG	NEG	NEG	NEG	NEG	NEG	POS	POS	NEG	NEG	NEG	NEG	POS	NEG
Dresden_01V39143	NEG	NEG	NEG	NEG	NEG	NEG	NEG	NEG	NEG	POS	POS	NEG	AMB	POS	NEG	POS	NEG
Greece 1_3680_Harmony	NEG	NEG	NEG	NEG	NEG	NEG	NEG	NEG	NEG	POS	POS	NEG	NEG	NEG	NEG	POS	NEG
>"European Clade": UK-(																	
ANS46 (Australia)	NEG	NEG	NEG	NEG	NEG	NEG	NEG	NEG	NEG	POS	POS	NEG	NEG	NEG	NEG	POS	NEG
LHH1 (USA)	NEG	NEG	NEG	NEG	NEG	NEG	NEG	NEG	NEG	POS	POS	NEG	NEG	NEG	NEG	POS	NEG
Dublin-DSH_AR01_0-1085	NEG	NEG	NEG	NEG	NEG	NEG	NEG	NEG	NEG	POS	POS	NEG	NEG	NEG	NEG	POS	NEG
Dublin-DSH_AR01_0-1118	NEG	NEG	NEG	NEG	NEG	NEG	NEG	NEG	NEG	POS	POS	NEG	NEG	NEG	NEG	POS	NEG
Dublin-DSH_AR15_0098	NEG	NEG	NEG	NEG	NEG	NEG	NEG	NEG	NEG	POS	POS	NEG	NEG	NEG	NEG	POS	NEG
Dublin-DSH_AR15_0104	NEG	NEG	NEG	NEG	NEG	NEG	NEG	NEG	NEG	POS	POS	NEG	NEG	NEG	NEG	POS	NEG
UK_NCTC11939_UK-1_Harmony	NEG	NEG	NEG	NEG	NEG	NEG	NEG	NEG	NEG	POS	POS	NEG	NEG	NEG	NEG	POS	NEG
>"European Clade": BK2																	
BK2421 (USA)	NEG	NEG	NEG	NEG	NEG	NEG	NEG	NEG	NEG	POS	POS	NEG	NEG	NEG	NEG	POS	NEG
>"Aussi/NZ Clade", JKD6																	
JKD6009 (Australia)	NEG	NEG	NEG	NEG	NEG	NEG	NEG	NEG	NEG	POS	POS	NEG	NEG	NEG	NEG	POS	NEG
JKD6008 (New Zealand)	NEG	NEG	NEG	NEG	NEG	NEG	NEG	NEG	NEG	POS	POS	NEG	NEG	NEG	NEG	POS	NEG
Perth_2015-S20209	NEG	NEG	NEG	NEG	NEG	NEG	NEG	NEG	NEG	POS	POS	NEG	NEG	NEG	NEG	POS	NEG
Perth_2015-S20305	NEG	NEG	NEG	NEG	NEG	NEG	NEG	NEG	NEG	POS	POS	NEG	NEG	NEG	NEG	POS	NEG
Perth_2015-S20603	NEG	NEG	NEG	NEG	NEG	NEG	NEG	NEG	NEG	POS	POS	NEG	NEG	NEG	NEG	POS	NEG
Perth_2015-S20607	NEG	NEG	NEG	NEG	NEG	NEG	NEG	NEG	NEG	POS	POS	NEG	NEG	NEG	NEG	POS	NEG
Perth_2015-S20608	NEG	NEG	NEG	NEG	NEG	NEG	NEG	NEG	NEG	POS	POS	NEG	NEG	NEG	NEG	POS	NEG
Perth_2015-S20610	NEG	NEG	NEG	NEG	NEG	NEG	NEG	NEG	NEG	POS	POS	NEG	NEG	NEG	NEG	POS	NEG
Perth_2015-S20613	NEG	NEG	NEG	NEG	NEG	NEG	NEG	NEG	NEG	POS	POS	NEG	NEG	NEG	NEG	POS	NEG
Perth_2015-S20617	NEG	NEG	NEG	NEG	NEG	NEG	NEG	NEG	NEG	POS	POS	NEG	NEG	NEG	NEG	POS	NEG
Perth_2015-S20622	NEG	NEG	NEG	NEG	NEG	NEG	NEG	NEG	NEG	POS	POS	NEG	NEG	NEG	NEG	POS	NEG
Perth_2015-S23619	NEG	NEG	NEG	NEG	NEG	NEG	NEG	NEG	NEG	POS	POS	NEG	NEG	NEG	NEG	POS	NEG
Perth_2015-S41210	NEG	NEG	NEG	NEG	NEG	NEG	NEG	NEG	NEG	POS	POS	NEG	NEG	NEG	NEG	POS	NEG
Perth_2015-S51001	NEG	NEG	NEG	NEG	NEG	NEG	NEG	NEG	NEG	POS	POS	NEG	NEG	NEG	NEG	POS	NEG
Perth_2015-S51003	NEG	NEG	NEG	NEG	NEG	NEG	NEG	NEG	NEG	POS	POS	NEG	NEG	NEG	NEG	POS	NEG
Perth_2015-S51006	NEG	NEG	NEG	NEG	NEG	NEG	NEG	NEG	NEG	POS	POS	NEG	NEG	NEG	NEG	POS	NEG
Perth_2015-S51007	NEG	NEG	NEG	NEG	NEG	NEG	NEG	NEG	NEG	POS	POS	NEG	NEG	NEG	NEG	POS	NEG
Perth_2015-S51008	NEG	NEG	NEG	NEG	NEG	NEG	NEG	NEG	NEG	POS	POS	NEG	NEG	NEG	NEG	POS	NEG
Perth_2015-S51014	NEG	NEG	NEG	NEG	NEG	NEG	NEG	NEG	NEG	POS	POS	NEG	NEG	NEG	NEG	POS	NEG
Perth_2015-S51408	NEG	NEG	NEG	NEG	NEG	NEG	NEG	NEG	NEG	POS	POS	NEG	NEG	NEG	NEG	POS	NEG
Perth_2016-S20614	NEG	NEG	NEG	NEG	NEG	NEG	NEG	NEG	NEG	POS	POS	NEG	NEG	NEG	NEG	POS	NEG
Perth_2016-S23614	NEG	NEG	NEG	NEG	NEG	NEG	NEG	NEG	NEG	POS	POS	NEG	NEG	NEG	NEG	POS	NEG
Perth_2016-S42009	NEG	NEG	NEG	NEG	NEG	NEG	NEG	NEG	NEG	POS	POS	NEG	NEG	NEG	NEG	POS	NEG
Perth_2016-S51001	NEG	NEG	NEG	NEG	NEG	NEG	NEG	NEG	NEG	POS	POS	NEG	NEG	NEG	NEG	POS	NEG
Perth_2016-S51403	NEG	NEG	NEG	NEG	NEG	NEG	NEG	NEG	NEG	POS	POS	NEG	NEG	NEG	NEG	POS	NEG
Perth_07 RPA 20	NEG	NEG	NEG	NEG	NEG	NEG	NEG	NEG	NEG	POS	POS	NEG	NEG	NEG	NEG	POS	NEG
Perth_08-19088	NEG	NEG	NEG	NEG	NEG	NEG	NEG	NEG	NEG	POS	POS	NEG	NEG	NEG	NEG	POS	NEG
Perth_01-15351	NEG	NEG	NEG	NEG	NEG	NEG	NEG	NEG	NEG	POS	POS	NEG	NEG	NEG	NEG	POS	NEG
Perth_01-15357	NEG	neg	NEG	NEG	NEG	NEG	NEG	NEG	NEG	POS	POS	NEG	NEG	NEG	NEG	POS	NEG
Perth_01-15419	NEG	NEG	NEG	NEG	NEG	NEG	NEG	NEG	NEG	POS	POS	NEG	NEG	NEG	NEG	POS	NEG
Perth_01-16309	NEG	NEG	NEG	NEG	NEG	NEG	NEG	NEG	NEG	POS	POS	NEG	NEG	NEG	NEG	POS	NEG
Perth_03 SNP 95	NEG	NEG	NEG	NEG	NEG	NEG	NEG	NEG	NEG	POS	POS	NEG	NEG	NEG	NEG	POS	NEG
Perth_2003_AH_47	NEG	NEG	NEG	NEG	NEG	NEG	NEG	NEG	NEG	POS	POS	NEG	NEG	NEG	NEG	POS	NEG
>"Australian/NZ Clade":																	
Perth_2015-S20623	NEG	NEG	NEG	NEG	NEG	NEG	NEG	NEG	NEG	POS	POS	NEG	NEG	NEG	NEG	POS	NEG
Perth_2015-S20826	NEG	NEG	NEG	NEG	NEG	NEG	NEG	NEG	NEG	POS	POS	NEG	NEG	NEG	NEG	POS	NEG
Perth_05 NH 56	NEG	NEG	NEG	NEG	NEG	NEG	NEG	NEG	NEG	POS	POS	NEG	NEG	NEG	NEG	POS	NEG
>CC5-MRSA-III LA MRSA																	
Delbrück 38 isolate from domestic turkey	NEG	NEG	NEG	NEG	NEG	NEG	NEG	NEG	NEG	POS	NEG	NEG	NEG	NEG	NEG	POS	NEG
> Staph. pseudintermed																	
KM1381	NEG	NEG	NEG	NEG	NEG	NEG	NEG	NEG	NEG	NEG	NEG	NEG	NEG	NEG	NEG	NEG	NEG
>"South-East Asian Clad																	
M92 (Canada)	NEG	NEG	NEG	NEG	NEG	NEG	NEG	NEG	NEG	POS	POS	NEG	NEG	NEG	NEG	POS	NEG
CHI59 (China)	NEG	NEG	NEG	NEG	NEG	NEG	NEG	NEG	NEG	POS	POS	NEG	NEG	NEG	NEG	POS	NEG
CHI61 (China)	NEG	NEG	NEG	NEG	NEG	NEG	NEG	NEG	NEG	POS	POS	NEG	NEG	NEG	NEG	POS	NEG
CUHK_HK1997 (China)	NEG	NEG	NEG	NEG	NEG	NEG	NEG	NEG	NEG	POS	POS	NEG	NEG	NEG	NEG	POS	NEG
M996 (China)	NEG	NEG	NEG	NEG	NEG	NEG	NEG	NEG	NEG	POS	POS	NEG	NEG	NEG	NEG	POS	NEG
H211 (Denmark)	NEG	NEG	NEG	NEG	NEG	NEG	NEG	NEG	NEG	POS	POS	NEG	NEG	NEG	NEG	POS	NEG
H216 (Denmark)	NEG	NEG	NEG	NEG	NEG	NEG	NEG	NEG	NEG	POS	POS	NEG	NEG	NEG	NEG	POS	NEG
D71 (Germany)	NEG	NEG	NEG	NEG	NEG	NEG	NEG	NEG	NEG	POS	POS	NEG	NEG	NEG	NEG	POS	NEG
NMR05 (India)	NEG	NEG	NEG	NEG	NEG	NEG	NEG	NEG	NEG	POS	POS	NEG	NEG	NEG	NEG	POS	NEG
MAL11 (Malaysia)	NEG	NEG	NEG	NEG	NEG	NEG	NEG	NEG	NEG	POS	POS	NEG	NEG	NEG	NEG	POS	NEG
MAL9 (Malaysia)	NEG	NEG	NEG	NEG	NEG	NEG	NEG	NEG	NEG	POS	POS	NEG	NEG	NEG	NEG	POS	NEG
Na21 (Sri Lanka)	NEG	NEG	NEG	NEG	NEG	NEG	NEG	NEG	NEG	POS	POS	NEG	NEG	NEG	NEG	POS	NEG
M592 (Syria)	NEG	NEG	NEG	NEG	NEG	NEG	NEG	NEG	NEG	POS	POS	NEG	NEG	NEG	NEG	POS	NEG
H202 (Thailand)	NEG	NEG	NEG	NEG	NEG	NEG	NEG	NEG	NEG	POS	POS	NEG	NEG	NEG	NEG	POS	NEG
S102 (Thailand)	NEG	NEG	NEG	NEG	NEG	NEG	NEG	NEG	NEG	POS	POS	NEG	NEG	NEG	NEG	POS	NEG
S130 (Thailand)	NEG	NEG	NEG	NEG	NEG	NEG	NEG	NEG	NEG	POS	POS	NEG	NEG	NEG	NEG	POS	NEG
S40 (Thailand)	NEG	NEG	NEG	NEG	NEG	NEG	NEG	NEG	NEG	POS	POS	NEG	NEG	NEG	NEG	POS	NEG
S71 (Thailand)	NEG	NEG	NEG	NEG	NEG	NEG	NEG	NEG	NEG	POS	POS	NEG	NEG	NEG	NEG	POS	NEG
S87 (Thailand)	NEG	NEG	NEG	NEG	NEG	NEG	NEG	NEG	NEG	POS	POS	NEG	NEG	NEG	NEG	POS	NEG
S93 (Thailand)	NEG	NEG	NEG	NEG	NEG	NEG	NEG	NEG	NEG	POS	POS	NEG	NEG	NEG	NEG	POS	NEG
US_002 (Thailand)	NEG	NEG	NEG	NEG	NEG	NEG	NEG	NEG	NEG	POS	POS	NEG	NEG	NEG	NEG	POS	NEG
US_017 (Thailand)	NEG	NEG	NEG	NEG	NEG	NEG	NEG	NEG	NEG	POS	POS	NEG	NEG	NEG	NEG	POS	NEG
WAMC6102 (Thailand)	NEG	NEG	NEG	NEG	NEG	NEG	NEG	NEG	NEG	POS	POS	NEG	NEG	NEG	NEG	POS	NEG
NCTC_13626_TW20 (UK)	NEG	NEG	NEG	NEG	NEG	NEG	NEG	NEG	NEG	POS	POS	NEG	NEG	NEG	NEG	POS	NEG
BSAC3021 (UK)	NEG	NEG	NEG	NEG	NEG	NEG	NEG	NEG	NEG	POS	POS	NEG	NEG	NEG	NEG	POS	NEG
BSAC3046 (UK)	NEG	NEG	NEG	NEG	NEG	NEG	NEG	NEG	NEG	POS	POS	NEG	NEG	NEG	NEG	POS	NEG
1193_SAUR (USA)	NEG	NEG	NEG	NEG	NEG	NEG	NEG	NEG	NEG	POS	POS	NEG	NEG	NEG	NEG	POS	NEG
1194_SAUR (USA)	NEG	NEG	NEG	NEG	NEG	NEG	NEG	NEG	NEG	POS	POS	NEG	NEG	NEG	NEG	POS	NEG

STRAIN / ISOLATE	HEAVY METAL RESIST																
	copA2-plasmi	copA2-SCC	mco-SCC	arsA	arsD	arsD2	arsB				arsC					czrB	czrC
	copA2_pla	copA2-SCC	mco-SCC	arsA	arsD	arsD2	arsB (SCC)			arsB (chromosomal)	arsC (chromosomal)	arsC (SCC or plasmidic)				czrB	czrC
	Copper exporting ATPase	Copper exporting ATPase	Multi copper oxidase	arsenical pump-driving ATPase	Putative dehydrogenase	trans-acting repressor of arsenic resistance operon	arsenical pump membrane protein				arsenate reductase					zink and cobalt transporter protein	cadmium and zinc resistance gene C, heavy metal translocating P-type ATPase. Frequently associated with SCCmec elements from livestock MRSA
>"South-East Asian Clade": TW20 (Irish AR44), cl																	
1195_SAUR (USA)	NEG	NEG	NEG	NEG	NEG	NEG	NEG	NEG	NEG	POS	POS	NEG	NEG	NEG	NEG	POS	NEG
FVRH6002 (USA)	NEG	NEG	NEG	NEG	NEG	NEG	NEG	NEG	NEG	POS	POS	NEG	NEG	NEG	NEG	POS	NEG
KINW6048 (USA)	NEG	NEG	NEG	NEG	NEG	NEG	NEG	NEG	NEG	POS	POS	NEG	NEG	NEG	NEG	POS	NEG
LAMC0011 (USA)	NEG	NEG	NEG	NEG	NEG	NEG	NEG	NEG	NEG	POS	POS	NEG	NEG	NEG	NEG	POS	NEG
SJOS6053 (USA)	NEG	NEG	NEG	NEG	NEG	NEG	NEG	NEG	NEG	POS	POS	NEG	NEG	NEG	NEG	POS	NEG
SJOS6072 (USA)	NEG	NEG	NEG	NEG	NEG	NEG	NEG	NEG	NEG	POS	POS	NEG	NEG	NEG	NEG	POS	NEG
UCIM6042 (USA)	NEG	NEG	NEG	NEG	NEG	NEG	NEG	NEG	NEG	POS	POS	NEG	NEG	NEG	NEG	POS	NEG
Perth_2015-S31902	NEG	NEG	NEG	NEG	NEG	NEG	NEG	NEG	NEG	POS	POS	NEG	NEG	NEG	NEG	POS	NEG
Perth_2015-S31904	NEG	NEG	NEG	NEG	NEG	NEG	NEG	NEG	NEG	POS	POS	NEG	NEG	NEG	NEG	POS	NEG
Perth_2015-S31913	NEG	NEG	NEG	NEG	NEG	NEG	NEG	NEG	NEG	POS	POS	NEG	NEG	NEG	NEG	POS	NEG
Perth_2015-S31918	NEG	NEG	NEG	NEG	NEG	NEG	NEG	NEG	NEG	POS	POS	NEG	NEG	NEG	NEG	POS	NEG
Dublin-DSH_AR44_E1183	NEG	NEG	NEG	NEG	NEG	NEG	NEG	NEG	NEG	POS	POS	NEG	POS	AMB	NEG	POS	NEG
Dublin-DSH_Unfamiliar-3_E1520	NEG	NEG	NEG	NEG	NEG	NEG	NEG	NEG	NEG	POS	POS	NEG	POS	POS	NEG	POS	NEG
Hong Kong_042	NEG	NEG	NEG	NEG	NEG	NEG	NEG	NEG	NEG	POS	POS	NEG	NEG	NEG	NEG	POS	NEG
Hong Kong_101	NEG	NEG	NEG	NEG	NEG	NEG	NEG	NEG	NEG	POS	POS	NEG	NEG	NEG	NEG	POS	NEG
Hong Kong_111	NEG	NEG	NEG	NEG	NEG	NEG	NEG	NEG	NEG	POS	POS	NEG	NEG	NEG	NEG	POS	NEG
Hong Kong_113	NEG	NEG	NEG	NEG	NEG	NEG	NEG	NEG	NEG	POS	POS	NEG	NEG	NEG	NEG	POS	NEG
Hong Kong_115	NEG	NEG	NEG	NEG	NEG	NEG	NEG	NEG	NEG	POS	POS	NEG	NEG	NEG	NEG	POS	NEG
Hong Kong_118	NEG	NEG	NEG	NEG	NEG	NEG	NEG	NEG	NEG	POS	POS	NEG	NEG	NEG	NEG	POS	NEG
Hong Kong_125	NEG	NEG	NEG	NEG	NEG	NEG	NEG	NEG	NEG	POS	POS	NEG	NEG	NEG	NEG	POS	NEG
Hong Kong_129	NEG	NEG	NEG	NEG	NEG	NEG	NEG	NEG	NEG	POS	POS	NEG	NEG	NEG	NEG	POS	NEG
Hong Kong_133	NEG	NEG	NEG	NEG	NEG	NEG	NEG	NEG	NEG	POS	POS	NEG	NEG	NEG	NEG	POS	NEG
Kuwait_009	NEG	NEG	NEG	NEG	NEG	NEG	NEG	NEG	NEG	POS	POS	NEG	NEG	NEG	NEG	POS	NEG
Kuwait_103	NEG	NEG	NEG	NEG	NEG	NEG	NEG	NEG	NEG	POS	POS	NEG	NEG	NEG	NEG	POS	NEG
Kuwait_107	NEG	NEG	NEG	NEG	NEG	NEG	NEG	NEG	NEG	POS	POS	NEG	NEG	NEG	NEG	POS	NEG
Perth_03 AH 88	NEG	NEG	NEG	NEG	NEG	NEG	NEG	NEG	NEG	POS	POS	NEG	NEG	NEG	NEG	POS	NEG
Perth_08-16905	NEG	NEG	NEG	NEG	NEG	NEG	NEG	NEG	NEG	POS	POS	NEG	NEG	NEG	NEG	POS	NEG
Riyadh_Alfaisal-10_SICU_13_B5672_1117353	NEG	NEG	NEG	NEG	NEG	NEG	NEG	NEG	NEG	POS	POS	NEG	NEG	NEG	NEG	POS	NEG
Riyadh_SSTI_17_3508602	NEG	NEG	NEG	NEG	NEG	NEG	NEG	NEG	NEG	POS	POS	NEG	NEG	NEG	NEG	POS	NEG
Riyadh-2822088-R	NEG	NEG	NEG	NEG	NEG	NEG	NEG	NEG	NEG	POS	POS	NEG	NEG	NEG	NEG	POS	NEG
Trinidad&Tobago_2012_195 (619/12)	NEG	NEG	NEG	NEG	NEG	NEG	NEG	NEG	NEG	POS	POS	NEG	NEG	NEG	NEG	POS	NEG
Trinidad&Tobago_2012_370 (1112-52177)	NEG	NEG	NEG	NEG	NEG	NEG	NEG	NEG	NEG	POS	POS	NEG	NEG	NEG	NEG	POS	NEG
Trinidad&Tobago_2012_378 (4139)	NEG	NEG	NEG	NEG	NEG	NEG	NEG	NEG	NEG	POS	POS	NEG	NEG	NEG	NEG	POS	NEG
Trinidad&Tobago_2013_PA21	NEG	NEG	NEG	NEG	NEG	NEG	NEG	NEG	NEG	POS	POS	NEG	POS	AMB	NEG	POS	NEG
Trinidad&Tobago_2013_PA22	NEG	NEG	NEG	NEG	NEG	NEG	NEG	NEG	NEG	POS	POS	NEG	NEG	NEG	NEG	POS	NEG
Trinidad&Tobago_2912_340 (8523/12)	NEG	NEG	NEG	NEG	NEG	NEG	NEG	NEG	NEG	POS	POS	NEG	NEG	NEG	NEG	POS	NEG
Trinidad&Tobago_MRSA2010_105	NEG	NEG	NEG	NEG	NEG	NEG	NEG	NEG	NEG	POS	POS	NEG	POS	POS	NEG	POS	NEG
Trinidad&Tobago_MRSA2010_108	NEG	NEG	NEG	NEG	NEG	NEG	NEG	NEG	NEG	POS	POS	NEG	NEG	NEG	NEG	POS	NEG
Trinidad&Tobago_MRSA2010_33	NEG	NEG	NEG	NEG	NEG	NEG	NEG	NEG	NEG	POS	POS	NEG	NEG	NEG	NEG	POS	NEG
Trinidad&Tobago_MRSA2010_79	NEG	NEG	NEG	NEG	NEG	NEG	NEG	NEG	NEG	POS	POS	NEG	AMB	NEG	NEG	POS	NEG
Trinidad&Tobago_MRSA2010_88	NEG	NEG	NEG	NEG	NEG	NEG	NEG	NEG	NEG	POS	POS	NEG	NEG	NEG	NEG	POS	NEG
Trinidad&Tobago_MRSA2010_97	NEG	NEG	NEG	NEG	NEG	NEG	NEG	NEG	NEG	POS	POS	NEG	NEG	NEG	NEG	POS	NEG
Trinidad&Tobago_SSTI 2012_031	NEG	NEG	NEG	NEG	NEG	NEG	NEG	NEG	NEG	POS	POS	NEG	POS	POS	NEG	POS	NEG
Uganda-29_17148_250811	NEG	NEG	NEG	NEG	NEG	NEG	NEG	NEG	NEG	POS	POS	NEG	NEG	NEG	NEG	POS	NEG
>"South-East Asian Clad																	
UCIM6015	NEG	NEG	NEG	NEG	NEG	NEG	NEG	NEG	NEG	POS	POS	NEG	NEG	NEG	NEG	POS	NEG
Hong Kong_121	NEG	NEG	NEG	NEG	NEG	NEG	NEG	NEG	NEG	POS	POS	NEG	NEG	NEG	NEG	POS	NEG
>"South-East Asian Clad																	
Z172 (Taiwan)	NEG	NEG	NEG	NEG	NEG	NEG	NEG	NEG	NEG	POS	POS	NEG	NEG	NEG	NEG	POS	NEG
Russia-19_0342_Kurgan	NEG	NEG	NEG	NEG	NEG	NEG	NEG	NEG	NEG	POS	POS	NEG	NEG	NEG	NEG	POS	NEG
>"South-East Asian Clad																	
Perth_01-15392	NEG	neg	NEG	NEG		NEG	NEG	NEG	NEG	POS	POS	NEG	NEG	NEG	NEG	POS	NEG
>"South-East Asian Clad																	
XN108 (China)	NEG	NEG	NEG	NEG	NEG	NEG	NEG	NEG	NEG	POS	POS	NEG	NEG	NEG	NEG	POS	NEG
NMR09 (India)	NEG	NEG	NEG	NEG	NEG	NEG	NEG	NEG	NEG	POS	POS	NEG	NEG	NEG	NEG	POS	NEG
346 (Malaysia)	NEG	NEG	NEG	NEG	NEG	NEG	NEG	NEG	NEG	POS	POS	NEG	NEG	NEG	NEG	POS	NEG
DS_009 (Thailand)	NEG	NEG	NEG	NEG	NEG	NEG	NEG	NEG	NEG	POS	POS	NEG	NEG	NEG	NEG	POS	NEG
18ANR581005 (Ind)	NEG	neg	NEG	NEG	neg	NEG	NEG	NEG	NEG	POS	POS	NEG	NEG	NEG	NEG	POS	NEG
Bengalaru_ST20141405	NEG	NEG	NEG	NEG	NEG	NEG	NEG	NEG	NEG	POS	POS	NEG	NEG	NEG	NEG	POS	NEG
Bengalaru_ST20141406	NEG	NEG	NEG	NEG	NEG	NEG	NEG	NEG	NEG	POS	POS	NEG	NEG	NEG	NEG	POS	NEG
Bengalaru_ST20141417	NEG	NEG	NEG	NEG	NEG	NEG	NEG	NEG	NEG	POS	POS	NEG	NEG	NEG	NEG	POS	NEG
Hong Kong_107	NEG	NEG	NEG	NEG	NEG	NEG	NEG	NEG	NEG	POS	POS	NEG	NEG	NEG	NEG	POS	NEG
Hong Kong_12	NEG	NEG	NEG	NEG	NEG	NEG	NEG	NEG	NEG	POS	POS	NEG	NEG	NEG	NEG	POS	NEG
Hong Kong_132	NEG	NEG	NEG	NEG	NEG	NEG	NEG	NEG	NEG	POS	POS	NEG	NEG	NEG	NEG	POS	NEG
Riyadh-3028763-R	NEG	NEG	NEG	NEG	NEG	NEG	NEG	NEG	NEG	POS	POS	NEG	NEG	NEG	NEG	POS	NEG
Trinidad&Tobago_MRSA2010_94	NEG	NEG	NEG	NEG	NEG	NEG	NEG	NEG	NEG	POS	POS	NEG	AMB	NEG	NEG	POS	NEG
>"South-East Asian Clad																	
M705 (Thailand)	NEG	NEG	NEG	NEG	NEG	NEG	NEG	NEG	NEG	POS	POS	NEG	NEG	NEG	NEG	POS	NEG
S85 (Thailand)	NEG	NEG	NEG	NEG	NEG	NEG	NEG	NEG	NEG	POS	POS	NEG	NEG	NEG	NEG	POS	NEG
Riyadh_Alfaisal-04_281014_S5076	NEG	NEG	NEG	NEG	NEG	NEG	NEG	NEG	NEG	POS	POS	NEG	NEG	NEG	NEG	POS	NEG
Riyadh_Alfaisal-26_S15145_1104163	NEG	NEG	NEG	NEG	NEG	NEG	NEG	NEG	NEG	POS	POS	NEG	NEG	NEG	NEG	POS	NEG
Riyadh-2817437-W	NEG	NEG	NEG	NEG	NEG	NEG	NEG	NEG	NEG	POS	POS	NEG	NEG	NEG	NEG	POS	NEG
>"South-East Asian Clad																	
PPUKM-775-2009 (Malaysia)	NEG	NEG	NEG	NEG	NEG	NEG	NEG	NEG	NEG	POS	POS	NEG	NEG	NEG	NEG	POS	NEG
Hong Kong_138	NEG	NEG	NEG	NEG	NEG	NEG	NEG	NEG	NEG	POS	POS	NEG	NEG	NEG	NEG	POS	NEG
>"South-East Asian Clad																	
Kuwait_001	NEG	NEG	NEG	NEG	NEG	NEG	NEG	NEG	NEG	POS	POS	NEG	NEG	NEG	NEG	POS	NEG
Kuwait_337	NEG	NEG	NEG	NEG	NEG	NEG	NEG	NEG	NEG	POS	POS	NEG	NEG	NEG	NEG	POS	NEG
>"South-East Asian Clad																	
CUHK_HK2007 (China)	NEG	NEG	NEG	NEG	NEG	NEG	NEG	NEG	NEG	POS	POS	NEG	NEG	NEG	NEG	POS	NEG
NMR02 (India)	NEG	NEG	NEG	NEG	NEG	NEG	NEG	NEG	NEG	POS	POS	NEG	NEG	NEG	NEG	POS	NEG
VB1490 (India)	NEG	NEG	NEG	NEG	NEG	NEG	NEG	NEG	NEG	POS	POS	NEG	NEG	NEG	NEG	POS	NEG
V521 (Korea)	NEG	NEG	NEG	NEG	NEG	NEG	NEG	NEG	NEG	POS	POS	NEG	NEG	NEG	NEG	POS	NEG
HST-077 (Lebanon)	NEG	NEG	NEG	NEG	NEG	NEG	NEG	NEG	NEG	POS	POS	NEG	NEG	NEG	NEG	POS	NEG
MAL1 (Malaysia)	NEG	NEG	NEG	NEG	NEG	NEG	NEG	NEG	NEG	POS	POS	NEG	NEG	NEG	NEG	POS	NEG
MAL3 (Malaysia)	NEG	NEG	NEG	NEG	NEG	NEG	NEG	NEG	NEG	POS	POS	NEG	NEG	NEG	NEG	POS	NEG
PPUKM-261-2009 (Malaysia)	NEG	NEG	NEG	NEG	NEG	NEG	NEG	NEG	NEG	POS	POS	NEG	NEG	NEG	NEG	POS	NEG
PPUKM-332-2009 (Malaysia)	NEG	NEG	NEG	NEG	NEG	NEG	NEG	NEG	NEG	POS	POS	NEG	NEG	NEG	NEG	POS	NEG
S38 (Thailand)	NEG	NEG	NEG	NEG	NEG	NEG	NEG	NEG	NEG	POS	POS	NEG	NEG	NEG	NEG	POS	NEG
S7 (Thailand)	NEG	NEG	NEG	NEG	NEG	NEG	NEG	NEG	NEG	POS	POS	NEG	NEG	NEG	NEG	POS	NEG
US_008 (Thailand)	NEG	NEG	NEG	NEG	NEG	NEG	NEG	NEG	NEG	POS	POS	NEG	NEG	NEG	NEG	POS	NEG
US_030 (Thailand)	NEG	NEG	NEG	NEG	NEG	NEG	NEG	NEG	NEG	POS	POS	NEG	NEG	NEG	NEG	POS	NEG
M170 (Tsunami-related)	NEG	NEG	NEG	NEG	NEG	NEG	NEG	NEG	NEG	POS	POS	NEG	NEG	NEG	NEG	POS	NEG
BSAC697 (UK)	NEG	NEG	NEG	NEG	NEG	NEG	NEG	NEG	NEG	POS	POS	NEG	NEG	NEG	NEG	POS	NEG
IS-189	NEG	NEG	NEG	NEG	NEG	NEG	NEG	NEG	NEG	POS	POS	NEG	NEG	NEG	NEG	POS	NEG
MRGR3	NEG	NEG	NEG	NEG	NEG	NEG	NEG	NEG	NEG	POS	POS	NEG	NEG	NEG	NEG	POS	NEG
smg07	NEG	NEG	NEG	NEG	NEG	NEG	NEG	NEG	NEG	POS	POS	NEG	NEG	NEG	NEG	POS	NEG
AH1-AUS-EMRSA-3	NEG	NEG	NEG	NEG	NEG	NEG	NEG	NEG	NEG	POS	POS	NEG	NEG	NEG	NEG	POS	NEG
Bengalaru_ST20141403	NEG	NEG	NEG	NEG	NEG	NEG	NEG	NEG	NEG	POS	POS	NEG	NEG	NEG	NEG	POS	NEG
Finland E24_98541_Harmony	NEG	NEG	NEG	NEG	NEG	NEG	NEG	NEG	NEG	POS	POS	NEG	NEG	NEG	NEG	POS	NEG
Hong Kong_10	NEG	NEG	NEG	NEG	NEG	NEG	NEG	NEG	NEG	POS	POS	NEG	NEG	NEG	NEG	POS	NEG
Hong Kong_100	NEG	NEG	NEG	NEG	NEG	NEG	NEG	NEG	NEG	POS	POS	NEG	NEG	NEG	NEG	POS	NEG
Hong Kong_199	NEG	NEG	NEG	NEG	NEG	NEG	NEG	NEG	NEG	POS	POS	NEG	NEG	NEG	NEG	POS	NEG
Hong Kong_34	NEG	NEG	NEG	NEG	NEG	NEG	NEG	NEG	NEG	POS	POS	NEG	NEG	NEG	NEG	POS	NEG
Hong Kong_88	NEG	NEG	NEG	NEG	NEG	NEG	NEG	NEG	NEG	POS	POS	NEG	POS	AMB	NEG	POS	NEG
Hong Kong_92	NEG	NEG	NEG	NEG	NEG	NEG	NEG	NEG	NEG	POS	POS	NEG	NEG	NEG	NEG	POS	NEG
Hong Kong_94	NEG	NEG	NEG	NEG	NEG	NEG	NEG	NEG	NEG	POS	POS	NEG	NEG	AMB	NEG	POS	NEG
Kuwait_257	NEG	NEG	NEG	NEG	NEG	NEG	NEG	NEG	NEG	POS	POS	NEG	NEG	AMB	NEG	POS	NEG
Perth_01-15330	NEG	NEG	NEG	NEG		NEG	NEG	NEG	NEG	POS	POS	NEG	NEG	NEG	NEG	POS	NEG
Perth_01-15349	NEG	NEG	NEG	NEG		NEG	NEG	NEG	NEG	POS	POS	NEG	NEG	NEG	NEG	POS	NEG
Perth_01-16176	NEG	NEG	NEG	NEG	NEG	NEG	NEG	NEG	NEG	POS	POS	NEG	NEG	NEG	NEG	POS	NEG
Perth_WBG8379	NEG	NEG	NEG	NEG	NEG	NEG	NEG	NEG	NEG	POS	POS	NEG	NEG	NEG	NEG	POS	NEG
Rawalpindi_Kidney Center_56	NEG	NEG	NEG	NEG	NEG	NEG	NEG	NEG	NEG	POS	POS	NEG	AMB	NEG	NEG	POS	NEG
Riyadh_SSTI_05_3464614	NEG	NEG	NEG	NEG	NEG	NEG	NEG	NEG	NEG	POS	POS	NEG	NEG	NEG	NEG	POS	NEG
Trinidad&Tobago_2012_157	NEG	NEG	NEG	NEG	NEG	NEG	NEG	NEG	NEG	POS	POS	NEG	POS	AMB	NEG	POS	NEG

STRAIN / ISOLATE	HEAVY METAL RESIST																
	copA2-plasmid	copA2-SCC	mco-SCC	arsA	arsD	arsD2	arsB				arsC					czrB	czrC
	copA2_pla	copA2-SCC	mco-SCC	arsA	arsD	arsD2	arsB (SCC)			arsB (chromosomal)	arsC (chromosomal)	arsC (SCC or plasmidic)				czrB	czrC
	Copper exporting ATPase	Copper exporting ATPase	Multi copper oxidase	arsenical pump-driving ATPase	Putative dehydrogenase	trans-acting repressor of arsenic resistance operon	arsenical pump membrane protein				arsenate reductase					zink and cobalt transporter protein	cadmium and zinc resistance gene C, heavy metal translocating P-type ATPase. Frequently associated with SCCmec elements from livestock MRSA
>"South-East Asian Clad																	
M418	NEG	NEG	NEG	NEG	NEG	NEG	NEG	NEG	NEG	POS	POS	NEG	NEG	NEG	NEG	POS	NEG
>"South-East Asian Clad																	
NMR07 (India)	NEG	NEG	NEG	NEG	NEG	NEG	NEG	NEG	NEG	POS	POS	NEG	NEG	NEG	NEG	POS	NEG
NMR08 (India)	NEG	NEG	NEG	NEG	NEG	NEG	NEG	NEG	NEG	POS	POS	NEG	NEG	NEG	NEG	POS	NEG
Bengalaru_ST20121978	NEG	NEG	NEG	NEG	NEG	NEG	NEG	NEG	NEG	POS	POS	NEG	NEG	NEG	NEG	POS	NEG
Hong Kong_89	NEG	NEG	NEG	NEG	NEG	NEG	NEG	NEG	NEG	POS	POS	NEG	NEG	NEG	NEG	POS	NEG
>"South-East Asian Clad																	
Perth_2005_AH_5	NEG	NEG	NEG	NEG	NEG	NEG	NEG	NEG	NEG	POS	POS	NEG	NEG	NEG	NEG	POS	NEG
>"South-East Asian Clad																	
Trinidad&Tobago_MRSA2010_103	NEG	NEG	NEG	NEG	NEG	NEG	NEG	NEG	NEG	POS	POS	NEG	NEG	NEG	NEG	POS	POS
Trinidad&Tobago_2013_PA25	NEG	NEG	NEG	NEG	NEG	NEG	NEG	NEG	NEG	POS	POS	NEG	NEG	NEG	NEG	POS	POS
Trinidad&Tobago_MRSA2010_110	NEG	NEG	NEG	NEG	NEG	NEG	NEG	NEG	NEG	POS	POS	NEG	NEG	NEG	NEG	POS	POS
Trinidad&Tobago_SSTI 2012_111	NEG	NEG	NEG	NEG	NEG	NEG	NEG	NEG	NEG	POS	POS	NEG	NEG	NEG	NEG	POS	POS
Trinidad&Tobago_MRSA2010_158	NEG	NEG	NEG	NEG	NEG	NEG	NEG	NEG	NEG	POS	POS	NEG	AMB	POS	NEG	POS	POS
>"South-East Asian Clad																	
Kuwait_122	NEG	NEG	NEG	NEG	NEG	NEG	NEG	NEG	NEG	POS	POS	NEG	NEG	NEG	NEG	POS	NEG
Kuwait_002	NEG	NEG	NEG	NEG	NEG	NEG	NEG	NEG	NEG	POS	POS	NEG	NEG	NEG	NEG	POS	NEG
Kuwait_196	NEG	NEG	NEG	NEG		NEG	NEG	NEG	NEG	POS	POS	NEG	NEG	NEG	NEG	POS	NEG
Kuwait_199	NEG	NEG	NEG	NEG	NEG	NEG	NEG	NEG	NEG	POS	POS	NEG	NEG	NEG	NEG	POS	NEG
Hong Kong_136	NEG	NEG	NEG	NEG	NEG	NEG	NEG	NEG	NEG	POS	POS	NEG	NEG	NEG	NEG	POS	NEG
>"South-East Asian Clad																	
P32 (Poland)	NEG	NEG	NEG	NEG	NEG	NEG	NEG	NEG	NEG	POS	POS	NEG	NEG	NEG	NEG	POS	NEG
>"South-East Asian Clad																	
Perth_2015-S32313	NEG	NEG	NEG	NEG	NEG	NEG	NEG	NEG	NEG	POS	POS	NEG	NEG	NEG	NEG	POS	NEG
>"hla-negative South-Ea																	
DEN907 (Denmark)	NEG	NEG	NEG	NEG	NEG	NEG	NEG	NEG	NEG	POS	POS	NEG	NEG	NEG	NEG	POS	NEG
>"hla-negative South-Ea																	
S26 (Thailand)	NEG	NEG	NEG	NEG	NEG	NEG	NEG	NEG	NEG	POS	POS	NEG	NEG	NEG	NEG	POS	NEG
S42 (Thailand)	NEG	NEG	NEG	NEG	NEG	NEG	NEG	NEG	NEG	POS	POS	NEG	NEG	NEG	NEG	POS	NEG
S97 (Thailand)	NEG	NEG	NEG	NEG	NEG	NEG	NEG	NEG	NEG	POS	POS	NEG	NEG	NEG	NEG	POS	NEG
S78 (Thailand)	NEG	NEG	NEG	NEG	NEG	NEG	NEG	NEG	NEG	POS	POS	NEG	NEG	NEG	NEG	POS	NEG
S25 (Thailand)	NEG	NEG	NEG	NEG	NEG	NEG	NEG	NEG	NEG	POS	POS	NEG	NEG	NEG	NEG	POS	NEG
S21 (Thailand)	NEG	NEG	NEG	NEG	NEG	NEG	NEG	NEG	NEG	POS	POS	NEG	NEG	NEG	NEG	POS	NEG
S106 (Thailand)	NEG	NEG	NEG	NEG	NEG	NEG	NEG	NEG	NEG	POS	POS	NEG	NEG	NEG	NEG	POS	NEG
S24 (Thailand)	NEG	NEG	NEG	NEG	NEG	NEG	NEG	NEG	NEG	POS	POS	NEG	NEG	NEG	NEG	POS	NEG
S39 (Thailand)	NEG	NEG	NEG	NEG	NEG	NEG	NEG	NEG	NEG	POS	POS	NEG	NEG	NEG	NEG	POS	NEG
S2 (Thailand)	NEG	NEG	NEG	NEG	NEG	NEG	NEG	NEG	NEG	POS	POS	NEG	NEG	NEG	NEG	POS	NEG
S81 (Thailand)	NEG	NEG	NEG	NEG	NEG	NEG	NEG	NEG	NEG	POS	POS	NEG	NEG	NEG	NEG	POS	NEG
M116 (Vietnam)	NEG	NEG	NEG	NEG	NEG	NEG	NEG	NEG	NEG	POS	POS	NEG	NEG	NEG	NEG	POS	NEG
>"Portuguese Clade": Pc																	
FFP103 (Portugal)	NEG	NEG	NEG	NEG	NEG	NEG	NEG	NEG	NEG	POS	POS	NEG	NEG	NEG	NEG	POS	NEG
HDG2 (Portugal)	NEG	NEG	NEG	NEG	NEG	NEG	NEG	NEG	NEG	POS	POS	NEG	NEG	NEG	NEG	POS	NEG
HSA10 (Portugal)	NEG	NEG	NEG	NEG	NEG	NEG	NEG	NEG	NEG	POS	POS	NEG	NEG	NEG	NEG	POS	NEG
HSA11 (Portugal)	NEG	NEG	NEG	NEG	NEG	NEG	NEG	NEG	NEG	POS	POS	NEG	NEG	NEG	NEG	POS	NEG
ICP5011 (Portugal)	NEG	NEG	NEG	NEG	NEG	NEG	NEG	NEG	NEG	POS	POS	NEG	NEG	NEG	NEG	POS	NEG
ICP5014 (Portugal)	NEG	NEG	NEG	NEG	NEG	NEG	NEG	NEG	NEG	POS	POS	NEG	NEG	NEG	NEG	POS	NEG
ICP5062 (Portugal)	NEG	NEG	NEG	NEG	NEG	NEG	NEG	NEG	NEG	POS	POS	NEG	NEG	NEG	NEG	POS	NEG
Strain 1063_ATCC 33592 FDAARGOS_3 (USA)	NEG	NEG	NEG	NEG	NEG	NEG	NEG	NEG	NEG	POS	POS	NEG	NEG	NEG	NEG	POS	NEG
	NEG	NEG	NEG	NEG	NEG	NEG	NEG	NEG	NEG	POS	POS	NEG	NEG	NEG	NEG	POS	NEG
Russia-07_0110_Kurgan	NEG	NEG	NEG	NEG	NEG	NEG	NEG	NEG	NEG	POS	POS	NEG	NEG	NEG	NEG	POS	NEG
Russia-11_0164_Kurgan	NEG	NEG	NEG	NEG	NEG	NEG	NEG	NEG	NEG	POS	POS	NEG	NEG	NEG	NEG	POS	NEG
Russia-20_0367_Kurgan	NEG	NEG	NEG	NEG	NEG	NEG	NEG	NEG	NEG	POS	POS	NEG	NEG	NEG	NEG	POS	NEG
Russia-21_0390_Chelyabinsk	NEG	NEG	NEG	NEG	NEG	NEG	NEG	NEG	NEG	POS	POS	NEG	NEG	NEG	NEG	POS	NEG
>"South American/ Mid																	
AGT1 (Argentina)	NEG	NEG	NEG	NEG	NEG	NEG	NEG	NEG	NEG	POS	POS	NEG	NEG	NEG	NEG	POS	NEG
AGT120 (Argentina)	NEG	NEG	NEG	NEG	NEG	NEG	NEG	NEG	NEG	POS	POS	NEG	NEG	NEG	NEG	POS	NEG
AGT67 (Argentina)	NEG	NEG	NEG	NEG	NEG	NEG	NEG	NEG	NEG	POS	POS	NEG	NEG	NEG	NEG	POS	NEG
AGT9 (Argentina)	NEG	NEG	NEG	NEG	NEG	NEG	NEG	NEG	NEG	POS	POS	NEG	NEG	NEG	NEG	POS	NEG
RA3 (Argentina)	NEG	NEG	NEG	NEG	NEG	NEG	NEG	NEG	NEG	POS	POS	NEG	NEG	NEG	NEG	POS	NEG
RA6 (Argentina)	NEG	NEG	NEG	NEG	NEG	NEG	NEG	NEG	NEG	POS	POS	NEG	NEG	NEG	NEG	POS	NEG
RA7 (Argentina)	NEG	NEG	NEG	NEG	NEG	NEG	NEG	NEG	NEG	POS	POS	NEG	NEG	NEG	NEG	POS	NEG
Be62 (Brazil)	NEG	NEG	NEG	NEG	NEG	NEG	NEG	NEG	NEG	POS	POS	NEG	NEG	NEG	NEG	POS	NEG
Bmb9393 (Brazil)	NEG	NEG	NEG	NEG	NEG	NEG	NEG	NEG	NEG	POS	POS	NEG	NEG	NEG	NEG	POS	NEG
BRA36 (Brazil)	NEG	NEG	NEG	NEG	NEG	NEG	NEG	NEG	NEG	POS	POS	NEG	NEG	NEG	NEG	POS	NEG
BZ48 (Brazil)	NEG	NEG	NEG	NEG	NEG	NEG	NEG	NEG	NEG	POS	POS	NEG	NEG	NEG	NEG	POS	NEG
Gv51 (Brazil)	NEG	NEG	NEG	NEG	NEG	NEG	NEG	NEG	NEG	POS	POS	NEG	NEG	NEG	NEG	POS	NEG
Gv69 (Brazil)	NEG	NEG	NEG	NEG	NEG	NEG	NEG	NEG	NEG	POS	POS	NEG	NEG	NEG	NEG	POS	NEG
Gv88 (Brazil)	NEG	NEG	NEG	NEG	NEG	NEG	NEG	NEG	NEG	POS	POS	NEG	NEG	NEG	NEG	POS	NEG
HC1335 (Brazil)	NEG	NEG	NEG	NEG	NEG	NEG	NEG	NEG	NEG	POS	POS	NEG	NEG	NEG	NEG	POS	NEG
HC1340 (Brazil)	NEG	NEG	NEG	NEG	NEG	NEG	NEG	NEG	NEG	POS	POS	NEG	NEG	NEG	NEG	POS	NEG
HC556 (Brazil)	NEG	NEG	NEG	NEG	NEG	NEG	NEG	NEG	NEG	POS	POS	NEG	NEG	NEG	NEG	POS	NEG
HU25 (Brazil)	NEG	NEG	NEG	NEG	NEG	NEG	NEG	NEG	NEG	POS	POS	NEG	NEG	NEG	NEG	POS	NEG
UB563 (Brazil)	NEG	NEG	NEG	NEG	NEG	NEG	NEG	NEG	NEG	POS	POS	NEG	NEG	NEG	NEG	POS	NEG
2A8 (Czech Republic)	NEG	NEG	NEG	NEG	NEG	NEG	NEG	NEG	NEG	POS	POS	NEG	NEG	NEG	NEG	POS	NEG
M1229 (Denmark)	NEG	NEG	NEG	NEG	NEG	NEG	NEG	NEG	NEG	POS	POS	NEG	NEG	NEG	NEG	POS	NEG
FRICAR (France)	NEG	NEG	NEG	NEG	NEG	NEG	NEG	NEG	NEG	POS	POS	NEG	NEG	NEG	NEG	POS	NEG
D90 (Germany)	NEG	NEG	NEG	NEG	NEG	NEG	NEG	NEG	NEG	POS	POS	NEG	NEG	NEG	NEG	POS	NEG
LIT68 (Lithuania)	NEG	NEG	NEG	NEG	NEG	NEG	NEG	NEG	NEG	POS	POS	NEG	NEG	NEG	NEG	POS	NEG
LIT76 (Lithuania)	NEG	NEG	NEG	NEG	NEG	NEG	NEG	NEG	NEG	POS	POS	NEG	NEG	NEG	NEG	POS	NEG
HGSA142 (Portugal)	NEG	NEG	NEG	NEG	NEG	NEG	NEG	NEG	NEG	POS	POS	NEG	NEG	NEG	NEG	POS	NEG
HGSA9 (Portugal)	NEG	NEG	NEG	NEG	NEG	NEG	NEG	NEG	NEG	POS	POS	NEG	NEG	NEG	NEG	POS	NEG
HSJ216 (Portugal)	NEG	NEG	NEG	NEG	NEG	NEG	NEG	NEG	NEG	POS	POS	NEG	NEG	NEG	NEG	POS	NEG
M278 (Portugal)	NEG	NEG	NEG	NEG	NEG	NEG	NEG	NEG	NEG	POS	POS	NEG	NEG	NEG	NEG	POS	NEG
ES26 (Spain)	NEG	NEG	NEG	NEG	NEG	NEG	NEG	NEG	NEG	POS	POS	NEG	NEG	NEG	NEG	POS	NEG
UK102 (UK)	NEG	NEG	NEG	NEG	NEG	NEG	NEG	NEG	NEG	POS	POS	NEG	NEG	NEG	NEG	POS	NEG
URU34 (Uruguay)	NEG	NEG	NEG	NEG	NEG	NEG	NEG	NEG	NEG	POS	POS	NEG	NEG	NEG	NEG	POS	NEG
IS-125	NEG	NEG	NEG	NEG	NEG	NEG	NEG	NEG	NEG	POS	POS	NEG	NEG	NEG	NEG	POS	NEG
IS-157	NEG	NEG	NEG	NEG	NEG	NEG	NEG	NEG	NEG	POS	POS	NEG	NEG	NEG	NEG	POS	NEG
Ecuador_1	NEG	NEG	NEG	NEG	NEG	NEG	NEG	NEG	NEG	POS	POS	NEG	NEG	NEG	NEG	POS	NEG
Ecuador_2	NEG	NEG	NEG	NEG	NEG	NEG	NEG	NEG	NEG	POS	POS	NEG	NEG	NEG	NEG	POS	NEG
Dresden_15ANR574263	NEG	NEG	NEG	NEG	NEG	NEG	NEG	NEG	NEG	POS	POS	NEG	NEG	AMB	NEG	POS	NEG
UK-EMRSA-11	NEG	NEG	NEG	NEG	NEG	NEG	NEG	NEG	NEG	POS	POS	NEG	NEG	NEG	NEG	POS	NEG
>"South American/ Mid																	
BRA2 (Brazil)	NEG	NEG	NEG	NEG	NEG	NEG	NEG	NEG	NEG	POS	POS	NEG	NEG	NEG	NEG	POS	NEG
CHL1 (Chile)	NEG	NEG	NEG	NEG	NEG	NEG	NEG	NEG	NEG	POS	POS	NEG	NEG	NEG	NEG	POS	NEG
CHL151 (Chile)	NEG	NEG	NEG	NEG	NEG	NEG	NEG	NEG	NEG	POS	POS	NEG	NEG	NEG	NEG	POS	NEG
UCL378 (Chile)	NEG	NEG	NEG	NEG	NEG	NEG	NEG	NEG	NEG	POS	POS	NEG	NEG	NEG	NEG	POS	NEG
UP72 (Peru)	NEG	NEG	NEG	NEG	NEG	NEG	NEG	NEG	NEG	AMB	POS	NEG	NEG	NEG	NEG	POS	NEG
UP81 (Peru)	NEG	NEG	NEG	NEG	NEG	NEG	NEG	NEG	NEG	POS	POS	NEG	NEG	NEG	NEG	POS	NEG
>"South American/ Mid																	
UP1073 (Peru)	NEG	NEG	NEG	NEG	NEG	NEG	NEG	NEG	NEG	POS	POS	NEG	NEG	NEG	NEG	POS	NEG

STRAIN / ISOLATE	HEAVY METAL RESIST																
	copA2-plasmid	copA2-SCC	mco-SCC	arsA	arsD	arsD2	arsB				arsC					czrB	czrC
	copA2_pla	copA2-SCC	mco-SCC	arsA	arsD	arsD2	arsB (SCC)			arsB (chromosomal)	arsC (chromosomal)	arsC (SCC or plasmidic)				czrB	czrC
	Copper exporting ATPase	Copper exporting ATPase	Multi copper oxidase	arsenical pump-driving ATPase	Putative dehydrogenase	trans-acting repressor of arsenic resistance operon	arsenical pump membrane protein				arsenate reductase					zink and cobalt transporter protein	cadmium and zinc resistance gene C, heavy metal translocating P-type ATPase. Frequently associated with SCCmec elements from livestock MRSA
>"South American/ Mid																	
LIT89 (Lithuania)	NEG	NEG	NEG	NEG	NEG	NEG	NEG	NEG	NEG	POS	POS	NEG	NEG	NEG	NEG	POS	NEG
MRSA_PR1 (Malaysia)	NEG	NEG	NEG	NEG	NEG	NEG	NEG	NEG	NEG	POS	POS	NEG	NEG	NEG	NEG	POS	NEG
Riyadh-2888905-R	NEG	NEG	NEG	NEG		NEG	NEG	NEG	NEG	POS	POS	NEG	NEG	NEG	NEG	POS	NEG
Riyadh-2888915-BC	NEG	NEG	NEG	NEG	NEG	NEG	NEG	NEG	NEG	POS	POS	NEG	NEG	NEG	NEG	POS	NEG
Riyadh-2793706-R	NEG	NEG	NEG	NEG	NEG	NEG	NEG	NEG	NEG	POS	POS	NEG	NEG	NEG	NEG	POS	NEG
>"South American/ Mid																	
Riyadh_SSTI_18_3502925	NEG	NEG	NEG	NEG	NEG	NEG	NEG	NEG	NEG	POS	POS	NEG	NEG	NEG	NEG	POS	NEG
Riyadh-2822825-W	NEG	NEG	NEG	NEG	NEG	NEG	NEG	NEG	NEG	POS	POS	NEG	NEG	NEG	NEG	POS	NEG
>"South American/ Mid																	
UK-EMRSA-9	NEG	NEG	NEG	NEG	NEG	NEG	NEG	NEG	NEG	POS	POS	NEG	NEG	NEG	NEG	POS	NEG
>"South American/ Mid																	
Lome_HT20020815	NEG	NEG	NEG	NEG	NEG	NEG	NEG	NEG	NEG	POS	POS	NEG	NEG	NEG	NEG	POS	NEG
>"South American/ Mid																	
NA32 (Denmark)	NEG	NEG	NEG	NEG	NEG	NEG	NEG	NEG	NEG	POS	POS	NEG	NEG	NEG	NEG	POS	NEG
H24 (Egypt)	NEG	NEG	NEG	NEG	NEG	NEG	NEG	NEG	NEG	POS	POS	NEG	NEG	NEG	NEG	POS	NEG
ATCC BAA-39 (=HUSA304) (Hungary)	NEG	NEG	NEG	NEG	NEG	NEG	NEG	NEG	NEG	POS	POS	NEG	NEG	NEG	NEG	POS	NEG
HUSA304 (Hungary)	NEG	NEG	NEG	NEG	NEG	NEG	NEG	NEG	NEG	POS	POS	NEG	NEG	NEG	NEG	POS	NEG
HU106 (Hungary)	NEG	NEG	NEG	NEG	NEG	NEG	NEG	NEG	NEG	POS	POS	NEG	NEG	NEG	NEG	POS	NEG
BSAC27 (UK)	NEG	NEG	NEG	NEG	NEG	NEG	NEG	NEG	NEG	POS	POS	NEG	NEG	NEG	NEG	POS	NEG
NCTC13131, UK-EMRSA-4 (UK)	NEG	NEG	NEG	NEG	NEG	NEG	NEG	NEG	NEG	POS	POS	NEG	NEG	NEG	NEG	POS	NEG
	NEG	NEG	NEG	NEG	NEG	NEG	NEG	NEG	NEG	POS	POS	NEG	AMB	NEG	NEG	POS	NEG
Algiers_HT20040080	NEG	NEG	NEG	NEG	NEG	NEG	NEG	NEG	NEG	POS	POS	NEG	NEG	NEG	NEG	POS	NEG
Dublin-DSH_AR09_0.0066	NEG	NEG	NEG	NEG	NEG	NEG	NEG	NEG	NEG	POS	POS	NEG	NEG	NEG	NEG	POS	NEG
Dublin-DSH_AR09_0-0065	NEG	NEG	NEG	NEG	NEG	NEG	NEG	NEG	NEG	POS	POS	NEG	NEG	NEG	NEG	POS	NEG
Dublin-DSH_Phenotype-III_84	NEG	NEG	NEG	NEG	NEG	NEG	NEG	NEG	NEG	POS	POS	NEG	NEG	NEG	NEG	POS	NEG
Hong Kong_130	NEG	NEG	NEG	NEG	NEG	NEG	NEG	NEG	NEG	POS	POS	NEG	NEG	NEG	NEG	POS	NEG
Kuwait_018	NEG	NEG	NEG	NEG	NEG	NEG	NEG	NEG	NEG	POS	POS	NEG	NEG	NEG	NEG	POS	NEG
Perth_08-17726	NEG	NEG	NEG	NEG	NEG	NEG	NEG	NEG	NEG	POS	POS	NEG	NEG	NEG	NEG	POS	NEG
Riyadh_Alfaisal/KKKSUH_86_MRS-14-279	NEG	NEG	NEG	NEG	NEG	NEG	NEG	NEG	NEG	POS	POS	NEG	NEG	NEG	NEG	POS	NEG
Riyadh_Alfaisal-04_Z3861_831588	NEG	NEG	NEG	NEG	NEG	NEG	NEG	NEG	NEG	POS	POS	NEG	NEG	NEG	NEG	POS	NEG
Riyadh_Alfaisal-30_S15724_1108013	NEG	NEG	NEG	NEG	NEG	NEG	NEG	NEG	NEG	POS	POS	NEG	NEG	NEG	NEG	POS	NEG
Riyadh_Alfaisal-6_22A_13_B3992_397721	NEG	NEG	NEG	NEG	NEG	NEG	NEG	NEG	NEG	POS	POS	NEG	NEG	NEG	NEG	POS	NEG
Riyadh_SSTI_52_3615482	NEG	NEG	NEG	NEG	NEG	NEG	NEG	NEG	NEG	POS	POS	NEG	NEG	NEG	NEG	POS	NEG
Riyadh-2817276-2	NEG	NEG	NEG	NEG	NEG	NEG	NEG	NEG	NEG	POS	POS	NEG	NEG	NEG	NEG	POS	NEG
Riyadh-2891670-W	NEG	NEG	NEG	NEG	NEG	NEG	NEG	NEG	NEG	POS	POS	NEG	NEG	NEG	NEG	POS	NEG
Riyadh-3006920-W	NEG	NEG	NEG	NEG	NEG	NEG	NEG	NEG	NEG	POS	POS	NEG	NEG	NEG	NEG	POS	NEG
Riyadh-R2567782	NEG	NEG	NEG	NEG	NEG	NEG	AMB	NEG	NEG	POS	POS	NEG	NEG	NEG	NEG	POS	NEG
Russia-18_0252_Krasnoyarsk_SK2	NEG	NEG	NEG	NEG	NEG	NEG	NEG	NEG	NEG	POS	POS	NEG	NEG	NEG	NEG	POS	NEG
UK-EMRSA-7	NEG	NEG	NEG	NEG	NEG	NEG	NEG	NEG	NEG	POS	POS	NEG	NEG	NEG	NEG	POS	NEG
>"South American/ Mid																	
Dublin-DSH_AR23_0073	NEG	NEG	NEG	NEG	NEG	NEG	NEG	NEG	NEG	POS	POS	NEG	NEG	NEG	NEG	POS	NEG
>Related to "South Ame																	
MRSA-OC3 (Russia)	NEG	NEG	NEG	NEG	NEG	NEG	NEG	NEG	NEG	POS	POS	NEG	NEG	NEG	NEG	POS	NEG
Russia-12_0176_Krasnoyarsk	NEG	NEG	NEG	NEG	NEG	NEG	NEG	NEG	NEG	POS	POS	NEG	NEG	NEG	NEG	POS	NEG
Russia-13_0180_Krasnoyarsk	NEG	NEG	NEG	NEG	NEG	NEG	NEG	NEG	NEG	POS	POS	NEG	NEG	NEG	NEG	POS	NEG
Russia-16_0249_Krasnoyarsk	NEG	NEG	NEG	NEG	NEG	NEG	NEG	NEG	NEG	POS	POS	NEG	NEG	NEG	NEG	POS	NEG
Russia-17_0250_Krasnoyarsk	NEG	NEG	NEG	NEG	NEG	NEG	NEG	NEG	NEG	POS	POS	NEG	NEG	NEG	NEG	POS	NEG
>Related to "South Ame																	
URU110 (Uruguay)	NEG	NEG	NEG	NEG	NEG	NEG	NEG	NEG	NEG	POS	POS	NEG	NEG	NEG	NEG	POS	NEG
>Related to "South Ame																	
DS_014 (Thailand)	NEG	NEG	NEG	NEG	NEG	NEG	NEG	NEG	NEG	POS	POS	NEG	NEG	NEG	NEG	POS	NEG
>Unassigned Middle Eas																	
Frankfurt_Oder_0490031797 (Libya)	NEG	NEG	NEG	NEG	NEG	NEG	NEG	NEG	NEG	POS	POS	NEG	AMB	NEG	NEG	POS	NEG
Kuwait_192	NEG	NEG	NEG	NEG	NEG	NEG	NEG	NEG	NEG	POS	POS	NEG	NEG	NEG	NEG	POS	NEG
Riyadh-9	NEG	NEG	NEG	NEG	NEG	NEG	NEG	NEG	NEG	POS	POS	NEG	NEG	NEG	NEG	POS	NEG
Russia-24_0407_Moscow	NEG	NEG	NEG	NEG	NEG	NEG	NEG	NEG	NEG	POS	POS	NEG	NEG	NEG	NEG	POS	NEG

STRAIN / ISOLATE	HEAVY METAL RESIST																
	cadA						cadD								cadX (plasmid)		cadX (SCC)
	cadA (cons)	cadA (pTW20)	cadA (TN554)	cadA (Sepi)	cadC (TN554)	cadC (pI258)	cadD (total)	hp1548_cadD	hp1340_cadD	hp1339_cadD	hp1466_cadD	hp1547_cadD	cadD (R35)	cadD (Smas)	cadX (plasmid probe I)	cadX (plasmid probe II)	cadX (JCSC6943)
	Cadmium transport protein D						cadmium efflux adenosine triphosphatase								Putative regulator of cadmium efflux		
>"Eurasian Clade": TUR1																NEG	
TUR1 (Turkey)	POS	NEG	POS	NEG	POS	NEG	NEG	NEG	NEG	NEG	NEG	NEG	POS	NEG	NEG	NEG	NEG
TUR9 (Turkey)	POS	NEG	POS	NEG	POS	NEG	NEG	NEG	NEG	NEG	NEG	NEG	POS	NEG	NEG	NEG	NEG
>"Eurasian Clade" : T013																NEG	
T0131 (China)	POS	NEG	POS	NEG	POS	NEG	NEG	NEG	NEG	NEG	NEG	NEG	POS	NEG	NEG	NEG	NEG
MU4 (Turkey)	POS	NEG	POS	NEG	POS	NEG	NEG	NEG	NEG	NEG	NEG	NEG	POS	NEG	NEG	NEG	NEG
DEU11 (Turkey)	POS	NEG	POS	NEG	POS	NEG	NEG	NEG	NEG	NEG	NEG	NEG	POS	NEG	NEG	NEG	NEG
DEU16 (Turkey)	POS	NEG	POS	NEG	POS	NEG	NEG	NEG	NEG	NEG	NEG	NEG	POS	NEG	NEG	NEG	NEG
Romania_Iasi_MRSA-07_54	POS	NEG	POS	NEG	POS	NEG	NEG	NEG	NEG	NEG	NEG	NEG	POS	NEG	NEG	NEG	NEG
>"Eurasian Clade": arsC-																NEG	
Romania_Iasi_MRSA-05_46	POS	NEG	POS	NEG	POS	NEG	NEG	NEG	NEG	NEG	NEG	NEG	POS	AMB	NEG	NEG	NEG
Romania_Iasi_MRSA-06_47	POS	NEG	POS	NEG	POS	NEG	NEG	NEG	NEG	NEG	NEG	NEG	POS	AMB	POS	NEG	NEG
>"Eurasian Clade": 16K/(																NEG	
CN79 (China)	POS	NEG	POS	NEG	POS	NEG	NEG	NEG	NEG	NEG	NEG	NEG	POS	NEG	NEG	NEG	NEG
CUHK_BJ2002 (China)	POS	NEG	POS	NEG	POS	NEG	NEG	NEG	NEG	NEG	NEG	NEG	POS	NEG	NEG	NEG	NEG
CUHK_BJ2007 (China)	POS	NEG	POS	NEG	POS	NEG	NEG	NEG	NEG	NEG	NEG	NEG	POS	NEG	NEG	NEG	NEG
3HK (Czech Republic)	POS	NEG	POS	NEG	POS	NEG	NEG	NEG	NEG	NEG	NEG	NEG	POS	NEG	NEG	NEG	NEG
HU109 (Hungary)	POS	NEG	POS	NEG	POS	NEG	NEG	NEG	NEG	NEG	NEG	NEG	POS	NEG	NEG	NEG	NEG
HUR18 (Hungary)	POS	NEG	POS	NEG	POS	NEG	NEG	NEG	NEG	NEG	NEG	NEG	POS	NEG	NEG	NEG	NEG
H482 (Romania)	POS	NEG	POS	NEG	POS	NEG	NEG	NEG	NEG	NEG	NEG	NEG	POS	NEG	NEG	NEG	NEG
16K (Russia)	POS	NEG	POS	NEG	POS	NEG	NEG	NEG	NEG	NEG	NEG	NEG	POS	NEG	NEG	NEG	NEG
DEU10 (Turkey)	POS	NEG	POS	NEG	POS	NEG	NEG	NEG	NEG	NEG	NEG	NEG	POS	NEG	NEG	NEG	NEG
DEU12 (Turkey)	POS	NEG	POS	NEG	POS	NEG	NEG	NEG	NEG	NEG	NEG	NEG	POS	NEG	NEG	NEG	NEG
DEU14 (Turkey)	POS	NEG	POS	NEG	POS	NEG	NEG	NEG	NEG	NEG	NEG	NEG	POS	NEG	NEG	NEG	NEG
DEU15 (Turkey)	POS	NEG	POS	NEG	POS	NEG	NEG	NEG	NEG	NEG	NEG	NEG	POS	NEG	NEG	NEG	NEG
DEU17 (Turkey)	POS	NEG	POS	NEG	POS	NEG	NEG	NEG	NEG	NEG	NEG	NEG	POS	NEG	NEG	NEG	NEG
DEU19 (Turkey)	POS	NEG	POS	NEG	POS	NEG	NEG	NEG	NEG	NEG	NEG	NEG	POS	NEG	NEG	NEG	NEG
DEU2 (Turkey)	POS	NEG	POS	NEG	POS	NEG	NEG	NEG	NEG	NEG	NEG	NEG	POS	NEG	NEG	NEG	NEG
DEU20 (Turkey)	POS	NEG	POS	NEG	POS	NEG	NEG	NEG	NEG	NEG	NEG	NEG	POS	NEG	NEG	NEG	NEG
DEU23 (Turkey)	POS	NEG	POS	NEG	POS	NEG	NEG	NEG	NEG	NEG	NEG	NEG	POS	NEG	NEG	NEG	NEG
DEU3 (Turkey)	POS	NEG	POS	NEG	POS	NEG	NEG	NEG	NEG	NEG	NEG	NEG	POS	NEG	NEG	NEG	NEG
DEU5 (Turkey)	POS	NEG	POS	NEG	POS	NEG	NEG	NEG	NEG	NEG	NEG	NEG	POS	NEG	NEG	NEG	NEG
DEU6 (Turkey)	POS	NEG	POS	NEG	POS	NEG	NEG	NEG	NEG	NEG	NEG	NEG	POS	NEG	NEG	NEG	NEG
DEU8 (Turkey)	POS	NEG	POS	NEG	POS	NEG	NEG	NEG	NEG	NEG	NEG	NEG	POS	NEG	NEG	NEG	NEG
HU11 (Turkey)	POS	NEG	POS	NEG	POS	NEG	NEG	NEG	NEG	NEG	NEG	NEG	POS	NEG	NEG	NEG	NEG
HU13 (Turkey)	POS	NEG	POS	NEG	POS	NEG	NEG	NEG	NEG	NEG	NEG	NEG	POS	NEG	NEG	NEG	NEG
HU14 (Turkey)	POS	NEG	POS	NEG	POS	NEG	NEG	NEG	NEG	NEG	NEG	NEG	POS	NEG	NEG	NEG	NEG
HU15 (Turkey)	POS	NEG	POS	NEG	POS	NEG	NEG	NEG	NEG	NEG	NEG	NEG	POS	NEG	NEG	NEG	NEG
HU16 (Turkey)	POS	NEG	POS	NEG	POS	NEG	NEG	NEG	NEG	NEG	NEG	NEG	POS	NEG	NEG	NEG	NEG
HU17 (Turkey)	POS	NEG	POS	NEG	POS	NEG	NEG	NEG	NEG	NEG	NEG	NEG	POS	NEG	NEG	NEG	NEG
HU21 (Turkey)	POS	NEG	POS	NEG	POS	NEG	NEG	NEG	NEG	NEG	NEG	NEG	POS	NEG	NEG	NEG	NEG
HU23 (Turkey)	POS	NEG	POS	NEG	POS	NEG	NEG	NEG	NEG	NEG	NEG	NEG	POS	NEG	NEG	NEG	NEG
HU26 (Turkey)	POS	NEG	POS	NEG	POS	NEG	NEG	NEG	NEG	NEG	NEG	NEG	POS	NEG	NEG	NEG	NEG
HU4 (Turkey)	POS	NEG	POS	NEG	POS	NEG	NEG	NEG	NEG	NEG	NEG	NEG	POS	NEG	NEG	NEG	NEG
HU5 (Turkey)	POS	NEG	POS	NEG	POS	NEG	NEG	NEG	NEG	NEG	NEG	NEG	POS	NEG	NEG	NEG	NEG
HU6 (Turkey)	POS	NEG	POS	NEG	POS	NEG	NEG	NEG	NEG	NEG	NEG	NEG	POS	NEG	NEG	NEG	NEG
HU7 (Turkey)	POS	NEG	POS	NEG	POS	NEG	NEG	NEG	NEG	NEG	NEG	NEG	POS	NEG	NEG	NEG	NEG
HU8 (Turkey)	POS	NEG	POS	NEG	POS	NEG	NEG	NEG	NEG	NEG	NEG	NEG	POS	NEG	NEG	NEG	NEG
HU9 (Turkey)	POS	NEG	POS	NEG	POS	NEG	NEG	NEG	NEG	NEG	NEG	NEG	POS	NEG	NEG	NEG	NEG
IU1 (Turkey)	POS	NEG	POS	NEG	POS	NEG	NEG	NEG	NEG	NEG	NEG	NEG	POS	NEG	NEG	NEG	NEG
IU10 (Turkey)	POS	NEG	POS	NEG	POS	NEG	NEG	NEG	NEG	NEG	NEG	NEG	POS	NEG	NEG	NEG	NEG
IU11 (Turkey)	POS	NEG	POS	NEG	POS	NEG	NEG	NEG	NEG	NEG	NEG	NEG	POS	NEG	NEG	NEG	NEG
IU12 (Turkey)	POS	NEG	POS	NEG	POS	NEG	NEG	NEG	NEG	NEG	NEG	NEG	POS	NEG	NEG	NEG	NEG
IU13 (Turkey)	POS	NEG	POS	NEG	POS	NEG	NEG	NEG	NEG	NEG	NEG	NEG	POS	NEG	NEG	NEG	NEG
IU15 (Turkey)	POS	NEG	POS	NEG	POS	NEG	NEG	NEG	NEG	NEG	NEG	NEG	POS	NEG	NEG	NEG	NEG
IU18 (Turkey)	POS	NEG	POS	NEG	POS	NEG	NEG	NEG	NEG	NEG	NEG	NEG	POS	NEG	NEG	NEG	NEG
IU19 (Turkey)	POS	NEG	POS	NEG	POS	NEG	NEG	NEG	NEG	NEG	NEG	NEG	POS	NEG	NEG	NEG	NEG
IU2 (Turkey)	POS	NEG	POS	NEG	POS	NEG	NEG	NEG	NEG	NEG	NEG	NEG	POS	NEG	NEG	NEG	NEG
IU4 (Turkey)	POS	NEG	POS	NEG	POS	NEG	NEG	NEG	NEG	NEG	NEG	NEG	POS	NEG	NEG	NEG	NEG
IU6 (Turkey)	POS	NEG	POS	NEG	POS	NEG	NEG	NEG	NEG	NEG	NEG	NEG	POS	NEG	NEG	NEG	NEG
IU7 (Turkey)	POS	NEG	POS	NEG	POS	NEG	NEG	NEG	NEG	NEG	NEG	NEG	POS	NEG	NEG	NEG	NEG
IU9 (Turkey)	POS	NEG	POS	NEG	POS	NEG	NEG	NEG	NEG	NEG	NEG	NEG	POS	NEG	NEG	NEG	NEG
MU1 (Turkey)	POS	NEG	POS	NEG	POS	NEG	NEG	NEG	NEG	NEG	NEG	NEG	POS	NEG	NEG	NEG	NEG
MU10 (Turkey)	POS	NEG	POS	NEG	POS	NEG	NEG	NEG	NEG	NEG	NEG	NEG	POS	NEG	NEG	NEG	NEG
MU20 (Turkey)	POS	NEG	POS	NEG	POS	NEG	NEG	NEG	NEG	NEG	NEG	NEG	POS	NEG	NEG	NEG	NEG
Mu3 (Turkey)	POS	NEG	POS	NEG	POS	NEG	NEG	NEG	NEG	NEG	NEG	NEG	POS	NEG	NEG	NEG	NEG
MU5 (Turkey)	POS	NEG	POS	NEG	POS	NEG	NEG	NEG	NEG	NEG	NEG	NEG	POS	NEG	NEG	NEG	NEG
MU6 (Turkey)	POS	NEG	POS	NEG	POS	NEG	NEG	NEG	NEG	NEG	NEG	NEG	POS	NEG	NEG	NEG	NEG
MU7 (Turkey)	POS	NEG	POS	NEG	POS	NEG	NEG	NEG	NEG	NEG	NEG	NEG	POS	NEG	NEG	NEG	NEG
TUR27 (Turkey)	POS	NEG	POS	NEG	POS	NEG	NEG	NEG	NEG	NEG	NEG	NEG	POS	NEG	NEG	NEG	NEG
NCTR 32S	POS	NEG	POS	NEG	POS	NEG	NEG	NEG	NEG	NEG	NEG	NEG	POS	NEG	NEG	NEG	NEG
SA02_A4 = Russia-06_0085_Moscow	POS	NEG	POS	NEG	POS	NEG	NEG	NEG	NEG	NEG	NEG	NEG	POS	NEG	NEG	NEG	NEG
	POS	NEG	POS	NEG	POS	NEG	NEG	NEG	NEG	NEG	NEG	NEG	POS	NEG	NEG	NEG	NEG
Dresden_08V35987 (Turkey)	POS	NEG	POS	NEG	POS	NEG	NEG	NEG	NEG	NEG	NEG	NEG	POS	NEG	NEG	NEG	NEG
Dresden_17ANR577152	POS	NEG	POS	NEG	POS	NEG	NEG	NEG	NEG	NEG	NEG	NEG	POS	AMB	NEG	NEG	NEG
Dresden_17ANR580374 (Makedonia)	POS	NEG	POS	NEG	POS	NEG	NEG	NEG	NEG	NEG	NEG	NEG	POS	NEG	NEG	NEG	NEG
Hong Kong_69-II	POS	NEG	POS	NEG	POS	NEG	AMB	NEG	AMB	AMB	AMB	NEG	POS	NEG	AMB	NEG	NEG
Hong Kong_93	POS	NEG	POS	NEG	POS	NEG	NEG	NEG	NEG	NEG	NEG	NEG	POS	NEG	NEG	NEG	NEG
Rawalpindi_Kidney Center_03_SK1	POS	NEG	POS	NEG	POS	NEG	NEG	NEG	NEG	NEG	NEG	NEG	POS	NEG	AMB	NEG	NEG
Rawalpindi_Kidney Center_08	POS	NEG	POS	NEG	POS	NEG	NEG	NEG	NEG	NEG	NEG	NEG	POS	NEG	NEG	NEG	NEG
Rawalpindi_Kidney Center_10	POS	NEG	POS	NEG	POS	NEG	NEG	NEG	NEG	NEG	NEG	NEG	POS	AMB	NEG	NEG	NEG
Rawalpindi_Kidney Center_19	POS	NEG	POS	NEG	POS	NEG	NEG	NEG	NEG	NEG	NEG	NEG	POS	AMB	POS	NEG	NEG
Rawalpindi_Kidney Center_50_SK1	POS	NEG	POS	NEG	POS	NEG	NEG	NEG	NEG	NEG	NEG	NEG	POS	AMB	NEG	NEG	NEG
Romania_Iasi_BC-49_430	POS	NEG	POS	NEG	POS	NEG	NEG	NEG	NEG	NEG	NEG	NEG	POS	NEG	NEG	NEG	NEG
Romania_Iasi_MRSA_284	POS	NEG	POS	NEG	POS	NEG	NEG	NEG	NEG	NEG	NEG	NEG	POS	NEG	NEG	NEG	NEG
Romania_Iasi_MRSA-32_318	POS	NEG	POS	NEG	POS	#ZAHL!	NEG	NEG	NEG	NEG	NEG	NEG	POS	NEG	NEG	NEG	NEG
Romania_Iasi_MRSA-54_430	POS	NEG	POS	NEG	POS	NEG	NEG	NEG	NEG	NEG	NEG	NEG	POS	NEG	NEG	NEG	NEG
Romania_Iasi_SSTI-01_2	POS	NEG	POS	NEG	POS	NEG	NEG	NEG	NEG	NEG	NEG	NEG	POS	NEG	NEG	NEG	NEG
Romania_Iasi_SSTI-10_101	POS	NEG	POS	NEG	POS	NEG	NEG	NEG	NEG	NEG	NEG	NEG	POS	AMB	NEG	NEG	NEG
Romania_Iasi_SSTI-11_106	POS	NEG	POS	NEG	POS	NEG	NEG	NEG	NEG	NEG	NEG	NEG	POS	NEG	NEG	NEG	NEG
Russia-01_0001_SaintPetersburg	POS	NEG	POS	NEG	POS	NEG	NEG	NEG	NEG	NEG	NEG	NEG	POS	NEG	NEG	NEG	NEG
Russia-02_0004_SaintPetersburg	POS	NEG	POS	NEG	POS	NEG	NEG	NEG	NEG	NEG	NEG	NEG	POS	NEG	NEG	NEG	NEG
Russia-03_0057_SaintPetersburg	POS	NEG	POS	NEG	POS	NEG	NEG	NEG	NEG	NEG	NEG	NEG	POS	NEG	NEG	NEG	NEG
Russia-04_0076_Moscow	POS	NEG	POS	NEG	POS	NEG	NEG	NEG	NEG	NEG	NEG	NEG	POS	NEG	NEG	NEG	NEG
Russia-05_0078_Moscow	POS	NEG	POS	NEG	POS	NEG	NEG	NEG	NEG	NEG	NEG	NEG	POS	NEG	NEG	NEG	NEG
Russia-08_0135_Moscow	POS	NEG	POS	NEG	POS	NEG	NEG	NEG	NEG	NEG	NEG	NEG	POS	NEG	NEG	NEG	NEG
Russia-09_0150_Kurgan	POS	NEG	POS	NEG	POS	NEG	NEG	NEG	NEG	NEG	NEG	NEG	POS	NEG	NEG	NEG	NEG
Russia-10_0162_Kurgan	POS	NEG	POS	NEG	POS	NEG	NEG	NEG	NEG	NEG	NEG	NEG	POS	NEG	NEG	NEG	NEG
Russia-14_0184_Moscow	POS	NEG	POS	NEG	POS	NEG	NEG	NEG	NEG	NEG	NEG	NEG	POS	NEG	NEG	NEG	NEG
Russia-15_0232_SaintPetersburg	POS	NEG	POS	NEG	POS	NEG	NEG	NEG	NEG	NEG	NEG	NEG	POS	NEG	NEG	NEG	NEG
Russia-22_0391_Chelyabinsk	POS	NEG	POS	NEG	POS	NEG	NEG	NEG	NEG	NEG	NEG	NEG	POS	NEG	NEG	NEG	NEG
Russia-23_0392_Chelyabinsk	POS	NEG	POS	NEG	POS	NEG	NEG	NEG	NEG	NEG	NEG	NEG	POS	NEG	NEG	NEG	NEG
>"Eurasian Clade" : IU17																NEG	
IU17 (Turkey)	POS	NEG	POS	NEG	POS	NEG	NEG	NEG	NEG	NEG	NEG	NEG	POS	NEG	NEG	NEG	NEG
IU20 (Turkey)	POS	NEG	POS	NEG	POS	NEG	NEG	NEG	NEG	NEG	NEG	NEG	POS	NEG	NEG	NEG	NEG

STRAIN / ISOLATE	HEAVY METAL RESIST																
	cadA						cadD								cadX (plasmid)		cadX (SCC)
	cadA (cons)	cadA (pTW20)	cadA (TN554)	cadA (Sepi)	cadC (TN554)	cadC (pI258)	cadD (total)	hp1548_cadD	hp1340_cadD	hp1339_cadD	hp1466_cadD	hp1547_cadD	cadD (R35)	cadD (Smas)	cadX (plasmid probe I)	cadX (plasmid probe II)	cadX (JCSC6943)
	Cadmium transport protein D						cadmium efflux adenosine triphosphatase								Putative regulator of cadmium efflux		
**>"Eurasian Clade": Spora**																NEG	
Dresden_16ANR581769	POS	NEG	POS	NEG	POS	NEG	NEG	NEG	NEG	NEG	NEG	NEG	POS	NEG	NEG	NEG	NEG
Dresden_17ANR578769	POS	NEG	POS	NEG	POS	NEG	NEG	NEG	NEG	NEG	NEG	NEG	POS	NEG	NEG	NEG	NEG
**>"Eurasian Clade": Kuwa**																NEG	
Kuwait_306	POS	NEG	POS	NEG	POS	NEG	NEG	NEG	NEG	NEG	NEG	NEG	POS	NEG	NEG	NEG	NEG
Kuwait_301	POS	NEG	POS	NEG	POS	NEG	NEG	NEG	NEG	NEG	NEG	NEG	POS	NEG	NEG	NEG	NEG
**>"European Clade": "Gre**																VAR	
GRE18 (Greece)	POS	NEG	POS	NEG	POS	NEG	POS	NEG	POS	POS	POS	NEG	POS	NEG	POS	POS	NEG
GRE317 (Greece)	POS	NEG	POS	NEG	POS	NEG	POS	NEG	POS	POS	POS	NEG	POS	NEG	POS	POS	NEG
GRE4 (Greece)	POS	NEG	POS	NEG	POS	NEG	POS	NEG	POS	POS	POS	NEG	POS	NEG	POS	POS	NEG
R35 (USA)	POS	NEG	POS	NEG	POS	NEG	NEG	NEG	NEG	NEG	NEG	NEG	POS	NEG	NEG	NEG	NEG
Casablanca_HT20060548	POS	#ZAHL!	POS	NEG	POS	#ZAHL!	NEG	NEG	NEG	NEG	NEG	NEG	POS	NEG	NEG	NEG	NEG
Casablanca_HT20060550	POS	NEG	POS	NEG	POS	#ZAHL!	NEG	NEG	NEG	NEG	NEG	NEG	POS	NEG	NEG	NEG	NEG
Dresden_01V34572 (Greece)	POS	NEG	POS	NEG	POS	NEG	POS	AMB	POS	POS	POS	NEG	POS	NEG	POS	POS	NEG
Dresden_01V36469	POS	NEG	POS	NEG	POS	NEG	POS	POS	POS	POS	POS	NEG	POS	NEG	POS	POS	NEG
Dresden_01V39123	POS	NEG	POS	NEG	POS	NEG	POS	POS	POS	POS	POS	NEG	POS	NEG	POS	POS	NEG
Dresden_01V39143	POS	NEG	POS	NEG	POS	AMB	POS	POS	POS	POS	POS	AMB	POS	NEG	POS	POS	NEG
Greece 1_3680_Harmony	POS	NEG	POS	NEG	POS	NEG	POS	POS	POS	POS	POS	NEG	POS	AMB	POS	POS	NEG
**>"European Clade": UK-(**																NEG	
ANS46 (Australia)	POS	NEG	POS	NEG	POS	NEG	NEG	NEG	NEG	NEG	NEG	NEG	POS	NEG	NEG	NEG	NEG
LHH1 (USA)	POS	NEG	POS	NEG	POS	NEG	NEG	NEG	NEG	NEG	NEG	NEG	POS	NEG	NEG	NEG	NEG
Dublin-DSH_AR01_0-1085	POS	NEG	POS	NEG	POS	NEG	POS	NEG	NEG	NEG	NEG	NEG	POS	NEG	NEG	NEG	NEG
Dublin-DSH_AR01_0-1118	POS	NEG	POS	NEG	POS	NEG	NEG	NEG	NEG	NEG	NEG	NEG	POS	NEG	NEG	NEG	NEG
Dublin-DSH_AR15_0098	POS	NEG	POS	NEG	POS	NEG	NEG	NEG	NEG	NEG	NEG	NEG	POS	NEG	NEG	NEG	NEG
Dublin-DSH_AR15_0104	POS	NEG	POS	NEG	POS	NEG	NEG	NEG	NEG	NEG	NEG	NEG	POS	NEG	NEG	NEG	NEG
UK_NCTC11939_UK-1_Harmony	POS	NEG	POS	NEG	POS	NEG	NEG	NEG	NEG	NEG	NEG	NEG	POS	NEG	NEG	NEG	NEG
**>"European Clade": BK2**																NEG	
BK2421 (USA)	POS	NEG	POS	NEG	POS	NEG	NEG	NEG	NEG	NEG	NEG	NEG	POS	NEG	NEG	NEG	NEG
**>"Aussi/NZ Clade", JKD6**																NEG	
JKD6009 (Australia)	POS	NEG	POS	NEG	POS	NEG	NEG	NEG	NEG	NEG	NEG	NEG	POS	NEG	NEG	NEG	NEG
JKD6008 (New Zealand)	POS	NEG	POS	NEG	POS	NEG	NEG	NEG	NEG	NEG	NEG	NEG	POS	NEG	NEG	NEG	NEG
Perth_2015-S20209	POS	NEG	POS	NEG	POS	NEG	NEG	NEG	NEG	NEG	NEG	NEG	POS	NEG	NEG	NEG	NEG
Perth_2015-S20305	POS	NEG	POS	NEG	POS	NEG	NEG	NEG	NEG	NEG	NEG	NEG	POS	NEG	NEG	NEG	NEG
Perth_2015-S20603	POS	NEG	POS	NEG	POS	NEG	NEG	NEG	NEG	NEG	NEG	NEG	POS	NEG	NEG	NEG	NEG
Perth_2015-S20607	POS	NEG	POS	NEG	POS	NEG	NEG	NEG	NEG	NEG	NEG	NEG	POS	NEG	NEG	NEG	NEG
Perth_2015-S20608	POS	NEG	POS	NEG	POS	NEG	NEG	NEG	NEG	NEG	NEG	NEG	POS	NEG	NEG	NEG	NEG
Perth_2015-S20610	POS	NEG	POS	NEG	POS	NEG	NEG	NEG	NEG	NEG	NEG	NEG	POS	NEG	NEG	NEG	NEG
Perth_2015-S20613	POS	NEG	POS	NEG	POS	NEG	NEG	NEG	NEG	NEG	NEG	NEG	POS	NEG	NEG	NEG	NEG
Perth_2015-S20617	POS	NEG	POS	NEG	POS	NEG	NEG	NEG	NEG	NEG	NEG	NEG	POS	NEG	NEG	NEG	NEG
Perth_2015-S20622	POS	NEG	POS	NEG	POS	NEG	NEG	NEG	NEG	NEG	NEG	NEG	POS	NEG	NEG	NEG	NEG
Perth_2015-S23619	POS	NEG	POS	NEG	POS	NEG	NEG	NEG	NEG	NEG	NEG	NEG	POS	NEG	NEG	NEG	NEG
Perth_2015-S41210	POS	NEG	POS	NEG	POS	NEG	NEG	NEG	NEG	NEG	NEG	NEG	POS	NEG	NEG	NEG	NEG
Perth_2015-S51001	POS	NEG	POS	NEG	POS	NEG	NEG	NEG	NEG	NEG	NEG	NEG	POS	NEG	NEG	NEG	NEG
Perth_2015-S51003	POS	NEG	POS	NEG	POS	NEG	NEG	NEG	NEG	NEG	NEG	NEG	POS	NEG	NEG	NEG	NEG
Perth_2015-S51006	POS	NEG	POS	NEG	POS	NEG	NEG	NEG	NEG	NEG	NEG	NEG	POS	NEG	NEG	NEG	NEG
Perth_2015-S51007	POS	NEG	POS	NEG	POS	NEG	NEG	NEG	NEG	NEG	NEG	NEG	POS	NEG	NEG	NEG	NEG
Perth_2015-S51008	POS	NEG	POS	NEG	POS	NEG	NEG	NEG	NEG	NEG	NEG	NEG	POS	NEG	NEG	NEG	NEG
Perth_2015-S51014	POS	NEG	POS	NEG	POS	NEG	NEG	NEG	NEG	NEG	NEG	NEG	POS	NEG	NEG	NEG	NEG
Perth_2015-S51408	POS	NEG	POS	NEG	POS	NEG	NEG	NEG	NEG	NEG	NEG	NEG	POS	NEG	NEG	NEG	NEG
Perth_2016-S20614	POS	NEG	POS	NEG	POS	NEG	NEG	NEG	NEG	NEG	NEG	NEG	POS	NEG	NEG	NEG	NEG
Perth_2016-S23614	POS	NEG	POS	NEG	POS	NEG	NEG	NEG	NEG	NEG	NEG	NEG	POS	NEG	NEG	NEG	NEG
Perth_2016-S42009	POS	NEG	POS	NEG	POS	NEG	NEG	NEG	NEG	NEG	NEG	NEG	POS	NEG	NEG	NEG	NEG
Perth_2016-S51001	POS	NEG	POS	NEG	POS	NEG	NEG	NEG	NEG	NEG	NEG	NEG	POS	NEG	NEG	NEG	NEG
Perth_2016-S51403	POS	NEG	POS	NEG	POS	NEG	NEG	NEG	NEG	NEG	NEG	NEG	POS	NEG	NEG	NEG	NEG
Perth_07 RPA 20	POS	NEG	POS	NEG	POS	NEG	NEG	NEG	NEG	NEG	NEG	NEG	POS	NEG	NEG	NEG	NEG
Perth_08-19088	POS	NEG	POS	NEG	POS	NEG	NEG	NEG	NEG	NEG	NEG	NEG	POS	AMB	NEG	NEG	NEG
Perth_01-15351	POS	NEG	POS	NEG	POS	NEG	POS	NEG	NEG	NEG	NEG	NEG	POS	NEG	NEG	NEG	NEG
Perth_01-15357	POS	NEG	POS	NEG	POS	NEG	NEG	NEG	NEG	NEG	NEG	NEG	POS	NEG	NEG	NEG	NEG
Perth_01-15419	POS	NEG	POS	NEG	POS	NEG	NEG	NEG	NEG	NEG	NEG	NEG	POS	NEG	NEG	NEG	NEG
Perth_01-16309	POS	NEG	POS	NEG	POS	NEG	NEG	NEG	NEG	NEG	NEG	NEG	POS	NEG	NEG	NEG	NEG
Perth_03 SNP 95	POS	NEG	POS	NEG	POS	NEG	NEG	NEG	NEG	NEG	NEG	NEG	POS	NEG	NEG	NEG	NEG
Perth_2003_AH_47	POS	NEG	POS	NEG	POS	NEG	NEG	NEG	NEG	NEG	NEG	NEG	POS	NEG	NEG	NEG	NEG
**>"Australian/NZ Clade":**																NEG	
Perth_2015-S20623	POS	NEG	POS	NEG	POS	NEG	NEG	NEG	NEG	NEG	NEG	NEG	POS	NEG	NEG	NEG	NEG
Perth_2015-S20826	POS	NEG	POS	NEG	POS	NEG	NEG	NEG	NEG	NEG	NEG	NEG	POS	NEG	NEG	NEG	NEG
Perth_05 NH 56	POS	NEG	POS	NEG	POS	NEG	NEG	NEG	NEG	NEG	NEG	NEG	POS	NEG	NEG	NEG	NEG
**>CC5-MRSA-III LA MRSA**																NEG	
Delbrück 38 isolate from domestic turkey	POS	NEG	POS	NEG	POS	NEG	NEG	NEG	NEG	NEG	NEG	NEG	POS	AMB	NEG	NEG	NEG
**> Staph. pseudintermedi**																NEG	
KM1381	NEG	NEG	NEG	NEG	NEG	NEG	NEG	NEG	NEG	NEG	NEG	NEG	NEG	NEG	NEG	NEG	NEG
**>"South-East Asian Clad**																VAR	
M92 (Canada)	POS	NEG	POS	NEG	POS	NEG	NEG	NEG	NEG	NEG	NEG	NEG	POS	NEG	NEG	NEG	NEG
CHI59 (China)	POS	NEG	POS	NEG	POS	NEG	NEG	NEG	NEG	NEG	NEG	NEG	POS	NEG	NEG	NEG	NEG
CHI61 (China)	POS	POS	POS	NEG	POS	POS	POS	NEG	POS	AMB	POS	NEG	POS	NEG	POS	POS	NEG
CUHK_HK1997 (China)	POS	NEG	POS	NEG	POS	NEG	NEG	NEG	NEG	NEG	NEG	NEG	POS	NEG	NEG	NEG	NEG
M996 (China)	POS	POS	POS	NEG	POS	POS	POS	NEG	POS	AMB	POS	NEG	POS	NEG	POS	POS	NEG
H211 (Denmark)	POS	NEG	POS	NEG	POS	NEG	NEG	NEG	NEG	NEG	NEG	NEG	POS	NEG	NEG	NEG	NEG
H216 (Denmark)	POS	NEG	POS	NEG	POS	NEG	NEG	NEG	NEG	NEG	NEG	NEG	POS	NEG	NEG	NEG	NEG
D71 (Germany)	POS	POS	POS	NEG	POS	POS	POS	NEG	POS	AMB	POS	NEG	POS	NEG	POS	POS	NEG
NMR05 (India)	POS	NEG	POS	NEG	POS	NEG	NEG	NEG	NEG	NEG	NEG	NEG	POS	NEG	POS	NEG	NEG
MAL11 (Malaysia)	POS	POS	POS	NEG	POS	POS	POS	NEG	POS	AMB	POS	NEG	POS	NEG	POS	POS	NEG
MAL9 (Malaysia)	POS	POS	POS	NEG	POS	POS	POS	NEG	POS	AMB	POS	NEG	POS	NEG	POS	POS	NEG
Na21 (Sri Lanka)	POS	NEG	POS	NEG	POS	NEG	NEG	NEG	NEG	NEG	NEG	NEG	POS	NEG	NEG	NEG	NEG
M592 (Syria)	POS	POS	POS	NEG	POS	POS	POS	NEG	POS	AMB	POS	NEG	POS	NEG	POS	POS	NEG
H202 (Thailand)	POS	NEG	POS	NEG	POS	NEG	NEG	NEG	NEG	NEG	NEG	NEG	POS	NEG	NEG	NEG	NEG
S102 (Thailand)	POS	NEG	POS	NEG	POS	NEG	NEG	NEG	NEG	NEG	NEG	NEG	POS	NEG	NEG	NEG	NEG
S130 (Thailand)	POS	NEG	POS	NEG	POS	NEG	NEG	NEG	NEG	NEG	NEG	NEG	POS	NEG	NEG	NEG	NEG
S40 (Thailand)	POS	NEG	POS	NEG	POS	NEG	NEG	NEG	NEG	NEG	NEG	NEG	POS	NEG	NEG	NEG	NEG
S71 (Thailand)	POS	NEG	POS	NEG	POS	NEG	NEG	NEG	NEG	NEG	NEG	NEG	POS	NEG	NEG	NEG	NEG
S87 (Thailand)	POS	NEG	POS	NEG	POS	NEG	NEG	NEG	NEG	NEG	NEG	NEG	POS	NEG	NEG	NEG	NEG
S93 (Thailand)	POS	NEG	POS	NEG	POS	NEG	NEG	NEG	NEG	NEG	NEG	NEG	POS	NEG	NEG	NEG	NEG
US_002 (Thailand)	POS	NEG	POS	NEG	POS	NEG	NEG	NEG	NEG	NEG	NEG	NEG	POS	NEG	NEG	NEG	NEG
US_017 (Thailand)	POS	NEG	POS	NEG	POS	NEG	NEG	NEG	NEG	NEG	NEG	NEG	POS	NEG	NEG	NEG	NEG
WAMC6102 (Thailand)	POS	POS	POS	NEG	POS	POS	POS	NEG	POS	AMB	POS	NEG	POS	NEG	POS	POS	NEG
NCTC_13626_TW20 (UK)	POS	POS	POS	NEG	POS	POS	POS	NEG	POS	AMB	POS	NEG	POS	NEG	POS	POS	NEG
BSAC3021 (UK)	POS	POS	POS	NEG	POS	POS	POS	NEG	POS	AMB	POS	NEG	POS	NEG	POS	POS	NEG
BSAC3046 (UK)	POS	POS	POS	NEG	POS	POS	POS	NEG	POS	AMB	POS	NEG	POS	NEG	POS	POS	NEG
1193_SAUR (USA)	POS	NEG	POS	NEG	POS	NEG	NEG	NEG	NEG	NEG	NEG	NEG	POS	NEG	NEG	NEG	NEG
1194_SAUR (USA)	POS	NEG	POS	NEG	POS	NEG	NEG	NEG	NEG	NEG	NEG	NEG	POS	NEG	NEG	NEG	NEG

STRAIN / ISOLATE	HEAVY METAL RESIST																
	cadA						cadD								cadX (plasmid)		cadX (SCC)
	cadA (cons)	cadA (pTW20)	cadA (TN554)	cadA (Sepi)	cadC (TN554)	cadC (pI258)	cadD (total)	hp1548_cadD	hp1340_cadD	hp1339_cadD	hp1466_cadD	hp1547_cadD	cadD (R35)	cadD (Smas)	cadX (plasmid probe I)	cadX (plasmid probe II)	cadX (JCSC6943)
	Cadmium transport protein D						cadmium efflux adenosine triphosphatase								Putative regulator of cadmium efflux		
>"South-East Asian Clade": TW20 (Irish AR44), co																	
1195_SAUR (USA)	POS	NEG	POS	NEG	POS	NEG	NEG	NEG	NEG	NEG	NEG	NEG	POS	NEG	NEG	NEG	NEG
FVRH6002 (USA)	POS	NEG	POS	NEG	POS	NEG	NEG	NEG	NEG	NEG	NEG	NEG	POS	NEG	NEG	NEG	NEG
KINW6048 (USA)	NEG	POS	POS	NEG	POS	POS	POS	NEG	POS	AMB	POS	NEG	POS	NEG	POS	POS	NEG
LAMC0011 (USA)	POS	POS	POS	NEG	POS	POS	POS	NEG	POS	AMB	POS	NEG	POS	NEG	POS	POS	NEG
SJOS6053 (USA)	POS	POS	POS	NEG	POS	POS	POS	NEG	POS	AMB	POS	NEG	POS	NEG	POS	POS	NEG
SJOS6072 (USA)	POS	NEG	POS	NEG	POS	NEG	NEG	NEG	NEG	NEG	NEG	NEG	POS	NEG	NEG	NEG	NEG
UCIM6042 (USA)	POS	POS	POS	NEG	NEG	POS	POS	NEG	POS	AMB	POS	NEG	POS	NEG	POS	POS	NEG
Perth_2015-S31902	POS	POS	POS	NEG	POS	POS	POS	NEG	POS	AMB	POS	NEG	POS	NEG	POS	POS	NEG
Perth_2015-S31904	POS	POS	POS	NEG	POS	POS	POS	NEG	POS	AMB	POS	NEG	POS	NEG	POS	POS	NEG
Perth_2015-S31913	POS	POS	POS	NEG	POS	POS	POS	NEG	POS	AMB	POS	NEG	POS	NEG	POS	POS	NEG
Perth_2015-S31918	POS	POS	POS	NEG	POS	POS	POS	NEG	POS	AMB	POS	NEG	AMB	NEG	POS	POS	NEG
Dublin-DSH_AR44_E1183	POS	POS	POS	NEG	POS	POS	POS	POS	POS	POS	POS	POS	POS	AMB	POS	POS	NEG
Dublin-DSH_Unfamiliar-3_E1520	POS	POS	POS	NEG	POS	POS	POS	POS	POS	POS	POS	NEG	POS	AMB	POS	POS	NEG
Hong Kong_042	POS	NEG	POS	NEG	POS	NEG	NEG	NEG	NEG	NEG	NEG	NEG	POS	NEG	NEG	NEG	NEG
Hong Kong_101	POS	NEG	POS	NEG	POS	NEG	NEG	NEG	NEG	NEG	NEG	NEG	POS	NEG	NEG	NEG	NEG
Hong Kong_111	POS	NEG	POS	NEG	POS	NEG	NEG	NEG	NEG	NEG	NEG	NEG	POS	NEG	POS	NEG	NEG
Hong Kong_113	POS	NEG	POS	NEG	POS	NEG	NEG	NEG	NEG	NEG	NEG	NEG	POS	NEG	NEG	NEG	NEG
Hong Kong_115	POS	NEG	POS	NEG	POS	NEG	NEG	NEG	NEG	NEG	NEG	NEG	POS	NEG	NEG	NEG	NEG
Hong Kong_118	POS	NEG	POS	NEG	POS	NEG	NEG	NEG	NEG	NEG	NEG	NEG	POS	NEG	NEG	NEG	NEG
Hong Kong_125	POS	NEG	POS	NEG	POS	NEG	NEG	NEG	NEG	NEG	NEG	NEG	POS	NEG	NEG	NEG	NEG
Hong Kong_129	POS	NEG	POS	NEG	POS	NEG	NEG	NEG	NEG	NEG	NEG	NEG	POS	NEG	NEG	NEG	NEG
Hong Kong_133	POS	POS	POS	NEG	POS	POS	POS	AMB	POS	POS	POS	NEG	POS	NEG	POS	POS	NEG
Kuwait_009	POS	POS	NEG	NEG	POS	POS	POS	POS	POS	POS	POS	NEG	POS	NEG	POS	POS	NEG
Kuwait_103	POS	POS	POS	NEG	POS	POS	POS	POS	POS	POS	POS	NEG	POS	NEG	POS	POS	NEG
Kuwait_107	POS	POS	POS	NEG	POS	POS	POS	AMB	POS	POS	POS	NEG	POS	NEG	POS	POS	NEG
Perth_03 AH 88	POS	POS	POS	NEG	POS	POS	POS	NEG	POS	POS	POS	NEG	POS	NEG	POS	POS	NEG
Perth_08-16905	POS	POS	POS	NEG	POS	POS	POS	AMB	POS	POS	POS	NEG	POS	NEG	POS	POS	NEG
Riyadh_Alfaisal-10_SICU_13_B5672_1117353	POS	POS	POS	NEG	POS	POS	POS	NEG	POS	POS	POS	NEG	POS	NEG	POS	POS	NEG
Riyadh_SSTI_17_3508602	POS	POS	POS	NEG	POS	POS	POS	NEG	POS	POS	POS	NEG	POS	NEG	POS	POS	NEG
Riyadh-2822088-R	POS	POS	POS	NEG	POS	POS	POS	AMB	POS	POS	POS	NEG	POS	NEG	POS	POS	NEG
Trinidad&Tobago_2012_195 (619/12)	POS	NEG	POS	NEG	POS	NEG	NEG	NEG	NEG	NEG	NEG	NEG	POS	NEG	NEG	NEG	NEG
Trinidad&Tobago_2012_370 (1112-52177)	POS	NEG	POS	NEG	POS	NEG	NEG	NEG	NEG	NEG	NEG	NEG	POS	NEG	NEG	NEG	NEG
Trinidad&Tobago_2012_378 (4139)	POS	POS	POS	NEG	POS	POS	POS	NEG	POS	POS	POS	NEG	POS	NEG	POS	POS	NEG
Trinidad&Tobago_2013_PA21	POS	POS	POS	NEG	POS	POS	POS	POS	POS	POS	POS	AMB	POS	AMB	POS	POS	NEG
Trinidad&Tobago_2013_PA22	POS	POS	POS	NEG	POS	POS	POS	POS	POS	POS	POS	NEG	POS	NEG	POS	POS	NEG
Trinidad&Tobago_2912_340 (8523/12)	POS	POS	POS	NEG	POS	POS	POS	NEG	POS	POS	POS	NEG	POS	NEG	POS	POS	NEG
Trinidad&Tobago_MRSA2010_105	POS	POS	POS	NEG	POS	POS	POS	POS	POS	POS	POS	NEG	POS	AMB	POS	POS	NEG
Trinidad&Tobago_MRSA2010_108	POS	POS	POS	NEG	POS	POS	POS	POS	POS	POS	POS	NEG	POS	NEG	POS	POS	NEG
Trinidad&Tobago_MRSA2010_33	POS	POS	POS	NEG	POS	POS	POS	AMB	POS	POS	POS	NEG	POS	NEG	POS	POS	NEG
Trinidad&Tobago_MRSA2010_79	POS	POS	POS	NEG	POS	POS	POS	POS	POS	POS	POS	AMB	POS	NEG	POS	POS	NEG
Trinidad&Tobago_MRSA2010_88	POS	POS	POS	NEG	POS	POS	POS	NEG	POS	POS	POS	NEG	POS	NEG	POS	POS	NEG
Trinidad&Tobago_MRSA2010_97	POS	POS	POS	NEG	POS	POS	POS	POS	POS	POS	POS	AMB	POS	NEG	POS	POS	NEG
Trinidad&Tobago_SSTI 2012_031	POS	POS	POS	NEG	POS	POS	POS	POS	POS	POS	POS	POS	POS	AMB	POS	POS	NEG
Uganda-29_17148_250811	POS	POS	POS	NEG	POS	POS	POS	NEG	POS	POS	POS	NEG	POS	NEG	POS	POS	NEG
>"South-East Asian Clad																VAR	
UCIM6015	POS	POS	POS	NEG	POS	POS	POS	NEG	POS	AMB	POS	NEG	POS	NEG	POS	POS	NEG
Hong Kong_121	POS	NEG	POS	NEG	POS	NEG	NEG	NEG	NEG	NEG	NEG	NEG	POS	NEG	NEG	NEG	NEG
>"South-East Asian Clad																POS	
Z172 (Taiwan)	POS	POS	NEG	NEG	POS	POS	POS	NEG	POS	AMB	POS	NEG	POS	NEG	POS	POS	NEG
Russia-19_0342_Kurgan	POS	POS	POS	NEG	POS	POS	POS	NEG	POS	POS	POS	NEG	POS	NEG	POS	POS	NEG
>"South-East Asian Clad																POS	
Perth_01-15392	POS	POS	POS	NEG	POS	POS	POS	POS	POS	POS	POS	NEG	POS	NEG	POS	POS	NEG
>"South-East Asian Clad																RARE	
XN108 (China)	POS	NEG	POS	NEG	POS	NEG	NEG	NEG	NEG	NEG	NEG	NEG	POS	NEG	NEG	NEG	NEG
NMR09 (India)	POS	NEG	POS	NEG	POS	NEG	NEG	NEG	NEG	NEG	NEG	NEG	POS	NEG	NEG	NEG	NEG
346 (Malaysia)	POS	NEG	POS	NEG	POS	NEG	NEG	NEG	NEG	NEG	NEG	NEG	POS	NEG	NEG	NEG	NEG
DS_009 (Thailand)	POS	NEG	POS	NEG	POS	NEG	NEG	NEG	NEG	NEG	NEG	NEG	POS	NEG	NEG	NEG	NEG
18ANR581005 (Ind)	POS	neg	POS	NEG	POS	neg	POS	NEG	POS	POS	POS	NEG	POS	NEG	POS	POS	NEG
Bengalaru_ST20141405	POS	NEG	POS	NEG	POS	NEG	NEG	NEG	NEG	NEG	NEG	NEG	POS	AMB	NEG	NEG	NEG
Bengalaru_ST20141406	POS	NEG	POS	NEG	POS	NEG	NEG	NEG	NEG	NEG	NEG	NEG	POS	NEG	NEG	NEG	NEG
Bengalaru_ST20141417	POS	NEG	POS	NEG	POS	NEG	NEG	NEG	NEG	NEG	NEG	NEG	POS	NEG	NEG	NEG	NEG
Hong Kong_107	POS	NEG	POS	NEG	POS	NEG	NEG	NEG	NEG	NEG	NEG	NEG	POS	NEG	NEG	NEG	NEG
Hong Kong_12	POS	NEG	POS	NEG	POS	NEG	NEG	NEG	NEG	NEG	NEG	NEG	AMB	NEG	NEG	NEG	NEG
Hong Kong_132	POS	NEG	POS	NEG	POS	NEG	NEG	NEG	NEG	NEG	NEG	NEG	POS	NEG	POS	NEG	NEG
Riyadh-3028763-R	POS	POS	NEG	NEG	POS	POS	POS	POS	POS	POS	POS	NEG	POS	NEG	POS	POS	NEG
Trinidad&Tobago_MRSA2010_94	POS	NEG	POS	NEG	POS	NEG	NEG	NEG	NEG	NEG	NEG	NEG	POS	AMB	POS	NEG	NEG
>"South-East Asian Clad																VAR	
M705 (Thailand)	POS	NEG	POS	NEG	POS	NEG	NEG	NEG	NEG	NEG	NEG	NEG	POS	NEG	NEG	NEG	NEG
S85 (Thailand)	POS	NEG	POS	NEG	POS	NEG	NEG	NEG	NEG	NEG	NEG	NEG	POS	NEG	NEG	NEG	NEG
Riyadh_Alfaisal-04_281014_S5076	POS	POS	NEG	NEG	POS	POS	POS	NEG	POS	POS	POS	NEG	POS	NEG	POS	POS	NEG
Riyadh_Alfaisal-26_S15145_1104163	POS	POS	NEG	NEG	POS	POS	POS	NEG	POS	POS	POS	NEG	POS	NEG	POS	POS	NEG
Riyadh-2817437-W	POS	POS	NEG	NEG	POS	POS	POS	POS	POS	POS	POS	NEG	POS	NEG	POS	POS	NEG
>"South-East Asian Clad																VAR	
PPUKM-775-2009 (Malaysia)	NEG	POS	POS	NEG	POS	POS	POS	NEG	POS	AMB	POS	NEG	POS	NEG	POS	POS	NEG
Hong Kong_138	POS	NEG	POS	NEG	POS	NEG	NEG	NEG	NEG	NEG	NEG	NEG	POS	NEG	NEG	NEG	NEG
>"South-East Asian Clad																POS	
Kuwait_001	POS	POS	NEG	NEG	NEG	POS	POS	POS	POS	POS	POS	NEG	NEG	NEG	POS	POS	NEG
Kuwait_337	POS	POS	NEG	NEG	NEG	POS	POS	NEG	POS	POS	POS	NEG	NEG	NEG	POS	POS	NEG
>"South-East Asian Clad																VAR	
CUHK_HK2007 (China)	POS	POS	POS	NEG	POS	POS	POS	NEG	POS	AMB	POS	NEG	POS	NEG	POS	POS	NEG
NMR02 (India)	POS	NEG	POS	NEG	POS	NEG	POS	NEG	POS	POS	POS	NEG	POS	NEG	POS	POS	NEG
VB1490 (India)	POS	NEG	POS	NEG	POS	NEG	NEG	NEG	NEG	NEG	NEG	NEG	POS	NEG	NEG	NEG	NEG
V521 (Korea)	POS	POS	POS	NEG	POS	POS	POS	NEG	POS	AMB	POS	NEG	POS	NEG	POS	POS	NEG
HST-077 (Lebanon)	POS	NEG	POS	NEG	POS	NEG	NEG	NEG	NEG	NEG	NEG	NEG	POS	NEG	NEG	NEG	NEG
MAL1 (Malaysia)	POS	POS	POS	NEG	POS	POS	POS	NEG	POS	AMB	POS	NEG	POS	NEG	POS	POS	NEG
MAL3 (Malaysia)	POS	NEG	POS	NEG	POS	NEG	NEG	NEG	NEG	NEG	NEG	NEG	POS	NEG	NEG	NEG	NEG
PPUKM-261-2009 (Malaysia)	POS	POS	POS	NEG	POS	POS	POS	NEG	POS	AMB	POS	NEG	POS	NEG	POS	POS	NEG
PPUKM-332-2009 (Malaysia)	POS	POS	POS	NEG	POS	POS	POS	NEG	POS	AMB	POS	NEG	POS	NEG	POS	POS	NEG
S38 (Thailand)	POS	POS	POS	NEG	POS	POS	POS	NEG	POS	AMB	POS	NEG	POS	NEG	POS	POS	NEG
S7 (Thailand)	POS	POS	POS	NEG	POS	POS	POS	NEG	POS	AMB	POS	NEG	POS	NEG	POS	POS	NEG
US_008 (Thailand)	POS	NEG	POS	NEG	POS	NEG	NEG	NEG	NEG	NEG	NEG	NEG	POS	NEG	NEG	NEG	NEG
US_030 (Thailand)	POS	NEG	POS	NEG	POS	NEG	NEG	NEG	NEG	NEG	NEG	NEG	POS	NEG	NEG	NEG	NEG
M170 (Tsunami-related)	POS	NEG	POS	NEG	POS	NEG	NEG	NEG	NEG	NEG	NEG	NEG	POS	NEG	NEG	NEG	NEG
BSAC697 (UK)	POS	NEG	POS	NEG	POS	NEG	NEG	NEG	NEG	NEG	NEG	NEG	POS	NEG	NEG	NEG	NEG
IS-189	POS	NEG	POS	NEG	POS	NEG	NEG	NEG	NEG	NEG	NEG	NEG	POS	NEG	NEG	NEG	NEG
MRGR3	POS	NEG	POS	NEG	POS	NEG	NEG	NEG	NEG	NEG	NEG	NEG	POS	NEG	NEG	NEG	NEG
smg07	POS	NEG	POS	NEG	POS	NEG	NEG	NEG	NEG	NEG	NEG	NEG	POS	NEG	NEG	NEG	NEG
AH1-AUS-EMRSA-3	POS	POS	POS	NEG	POS	POS	POS	POS	POS	POS	POS	NEG	POS	NEG	POS	POS	NEG
Bengalaru_ST20141403	POS	POS	POS	NEG	POS	POS	POS	NEG	POS	POS	POS	NEG	POS	NEG	POS	POS	NEG
Finland E24_98541_Harmony	POS	POS	POS	NEG	POS	POS	POS	POS	POS	POS	POS	NEG	POS	NEG	POS	POS	NEG
Hong Kong_10	POS	POS	POS	NEG	POS	POS	POS	POS	POS	POS	POS	NEG	POS	AMB	POS	POS	NEG
Hong Kong_100	POS	NEG	POS	NEG	POS	NEG	NEG	NEG	NEG	NEG	NEG	NEG	POS	NEG	NEG	NEG	NEG
Hong Kong_199	POS	NEG	POS	NEG	POS	NEG	NEG	NEG	NEG	NEG	NEG	NEG	POS	NEG	NEG	NEG	NEG
Hong Kong_34	POS	NEG	POS	NEG	POS	NEG	NEG	NEG	NEG	NEG	NEG	NEG	POS	NEG	NEG	NEG	NEG
Hong Kong_88	POS	NEG	POS	NEG	POS	NEG	AMB	NEG	NEG	NEG	POS	NEG	POS	AMB	AMB	NEG	NEG
Hong Kong_92	POS	NEG	POS	NEG	POS	NEG	NEG	NEG	NEG	NEG	NEG	NEG	POS	NEG	NEG	NEG	NEG
Hong Kong_94	POS	NEG	POS	NEG	POS	NEG	AMB	NEG	NEG	NEG	POS	NEG	POS	AMB	POS	NEG	NEG
Kuwait_257	POS	NEG	POS	NEG	POS	NEG	NEG	NEG	NEG	NEG	NEG	NEG	POS	NEG	AMB	NEG	NEG
Perth_01-15330	POS	POS	POS	NEG	POS	POS	POS	POS	POS	POS	POS	NEG	POS	NEG	POS	POS	NEG
Perth_01-15349	POS	POS	POS	NEG	POS	POS	POS	POS	POS	POS	POS	NEG	POS	NEG	POS	POS	NEG
Perth_01-16176	POS	POS	POS	NEG	POS	POS	POS	POS	POS	POS	POS	NEG	POS	NEG	POS	POS	NEG
Perth_WBG8379	POS	POS	POS	NEG	POS	POS	POS	POS	POS	POS	POS	NEG	POS	NEG	POS	POS	NEG
Rawalpindi_Kidney Center_56	POS	NEG	POS	NEG	POS	NEG	NEG	NEG	NEG	NEG	NEG	NEG	POS	AMB	POS	NEG	NEG
Riyadh_SSTI_05_3464614	POS	POS	POS	NEG	POS	POS	POS	NEG	POS	POS	POS	NEG	POS	NEG	POS	POS	NEG
Trinidad&Tobago_2012_157	POS	NEG	POS	NEG	POS	NEG	AMB	NEG	NEG	NEG	POS	NEG	POS	AMB	POS	NEG	NEG

STRAIN / ISOLATE	HEAVY METAL RESIST																
	cadA						cadD								cadX (plasmid)		cadX (SCC)
	cadA (cons)	cadA (pTW20)	cadA (TN554)	cadA (Sepi)	cadC (TN554)	cadC (pI258)	cadD (total)	hp1548_cadD	hp1340_cadD	hp1339_cadD	hp1466_cadD	hp1547_cadD	cadD (R35)	cadD (Smas)	cadX (plasmid probe I)	cadX (plasmid probe II)	cadX (JCSC6943)
	Cadmium transport protein D						cadmium efflux adenosine triphosphatase								Putative regulator of cadmium efflux		
>"South-East Asian Clad																NEG	
M418	POS	NEG	POS	NEG	POS	NEG	NEG	NEG	NEG	NEG	NEG	NEG	POS	NEG	NEG	NEG	NEG
>"South-East Asian Clad																NEG	
NMR07 (India)	POS	NEG	POS	NEG	POS	NEG	NEG	NEG	NEG	NEG	NEG	NEG	POS	NEG	NEG	NEG	NEG
NMR08 (India)	POS	NEG	POS	NEG	POS	NEG	NEG	NEG	NEG	NEG	NEG	NEG	POS	NEG	NEG	NEG	NEG
Bengalaru_ST20121978	POS	NEG	POS	NEG	POS	NEG	NEG	NEG	NEG	NEG	NEG	NEG	POS	NEG	NEG	NEG	NEG
Hong Kong_89	POS	NEG	POS	NEG	POS	NEG	NEG	NEG	NEG	NEG	NEG	NEG	POS	NEG	NEG	NEG	NEG
>"South-East Asian Clad																POS	
Perth_2005_AH_5	POS	POS	POS	NEG	POS	POS	POS	NEG	POS	POS	POS	NEG	POS	NEG	POS	POS	NEG
>"South-East Asian Clad																VAR	
Trinidad&Tobago_MRSA2010_103	POS	NEG	POS	NEG	POS	NEG	NEG	NEG	NEG	NEG	NEG	NEG	POS	NEG	AMB	NEG	NEG
Trinidad&Tobago_2013_PA25	POS	POS	POS	NEG	POS	POS	POS	POS	POS	POS	POS	NEG	POS	NEG	POS	POS	NEG
Trinidad&Tobago_MRSA2010_110	POS	POS	POS	NEG	POS	POS	POS	NEG	POS	POS	POS	NEG	POS	NEG	POS	POS	NEG
Trinidad&Tobago_SSTI 2012_111	POS	POS	POS	NEG	POS	POS	POS	NEG	POS	POS	POS	NEG	POS	NEG	POS	POS	NEG
Trinidad&Tobago_MRSA2010_158	POS	NEG	POS	NEG	POS	NEG	NEG	NEG	NEG	NEG	NEG	NEG	POS	AMB	POS	NEG	NEG
>"South-East Asian Clad																VAR	
Kuwait_122	POS	POS	POS	NEG	POS	POS	POS	NEG	POS	POS	POS	NEG	POS	NEG	POS	POS	NEG
Kuwait_002	POS	POS	POS	NEG	POS	POS	POS	NEG	POS	POS	POS	NEG	POS	NEG	POS	POS	NEG
Kuwait_196	POS	POS	POS	NEG	POS	POS	POS	POS	POS	POS	POS	NEG	POS	NEG	POS	POS	NEG
Kuwait_199	POS	POS	POS	NEG	POS	POS	POS	AMB	POS	POS	POS	NEG	POS	NEG	POS	POS	NEG
Hong Kong_136	POS	NEG	POS	NEG	POS	NEG	NEG	NEG	NEG	NEG	NEG	NEG	POS	NEG	AMB	NEG	NEG
>"South-East Asian Clad																NEG	
P32 (Poland)	POS	NEG	POS	NEG	POS	NEG	NEG	NEG	NEG	NEG	NEG	NEG	POS	NEG	NEG	NEG	NEG
>"South-East Asian Clad																NEG	
Perth_2015-S32313	POS	NEG	POS	NEG	POS	NEG	NEG	NEG	NEG	NEG	NEG	NEG	POS	NEG	NEG	NEG	NEG
>"hla-negative South-Ea																POS	
DEN907 (Denmark)	POS	POS	POS	NEG	POS	POS	POS	NEG	POS	AMB	POS	NEG	POS	NEG	POS	POS	NEG
>"hla-negative South-Ea																COMM	
S26 (Thailand)	NEG	POS	POS	NEG	POS	POS	POS	NEG	POS	AMB	POS	NEG	POS	NEG	POS	POS	NEG
S42 (Thailand)	NEG	POS	POS	NEG	POS	POS	POS	NEG	POS	AMB	POS	NEG	POS	NEG	POS	POS	NEG
S97 (Thailand)	POS	POS	POS	NEG	POS	POS	POS	NEG	POS	AMB	POS	NEG	POS	NEG	POS	POS	NEG
S78 (Thailand)	POS	POS	NEG	NEG	POS	POS	POS	NEG	POS	AMB	POS	NEG	POS	NEG	POS	POS	NEG
S25 (Thailand)	POS	POS	POS	NEG	POS	POS	POS	NEG	POS	AMB	POS	NEG	POS	NEG	POS	POS	NEG
S21 (Thailand)	POS	POS	POS	NEG	POS	POS	POS	NEG	POS	AMB	POS	NEG	POS	NEG	POS	POS	NEG
S106 (Thailand)	POS	POS	POS	NEG	POS	POS	POS	NEG	POS	AMB	POS	NEG	POS	NEG	POS	POS	NEG
S24 (Thailand)	POS	POS	POS	NEG	POS	POS	POS	NEG	POS	AMB	POS	NEG	POS	NEG	POS	POS	NEG
S39 (Thailand)	POS	POS	POS	NEG	POS	POS	POS	NEG	POS	AMB	POS	NEG	POS	NEG	POS	POS	NEG
S2 (Thailand)	POS	POS	POS	NEG	POS	POS	POS	NEG	POS	AMB	POS	NEG	POS	NEG	POS	POS	NEG
S81 (Thailand)	POS	POS	POS	NEG	POS	POS	POS	NEG	POS	AMB	POS	NEG	POS	NEG	POS	POS	NEG
M116 (Vietnam)	POS	NEG	POS	NEG	POS	NEG	NEG	NEG	NEG	NEG	NEG	NEG	POS	NEG	NEG	NEG	NEG
>"Portuguese Clade": Po																NEG	
FFP103 (Portugal)	POS	NEG	POS	NEG	POS	NEG	NEG	NEG	NEG	NEG	NEG	NEG	POS	NEG	NEG	NEG	NEG
HDG2 (Portugal)	POS	NEG	POS	NEG	POS	NEG	NEG	NEG	NEG	NEG	NEG	NEG	POS	NEG	NEG	NEG	NEG
HSA10 (Portugal)	POS	NEG	POS	NEG	POS	NEG	NEG	NEG	NEG	NEG	NEG	NEG	POS	NEG	NEG	NEG	NEG
HSA11 (Portugal)	POS	NEG	POS	NEG	POS	NEG	NEG	NEG	NEG	NEG	NEG	NEG	POS	NEG	NEG	NEG	NEG
ICP5011 (Portugal)	POS	NEG	POS	NEG	POS	NEG	NEG	NEG	NEG	NEG	NEG	NEG	POS	NEG	NEG	NEG	NEG
ICP5014 (Portugal)	POS	NEG	POS	NEG	POS	NEG	NEG	NEG	NEG	NEG	NEG	NEG	POS	NEG	NEG	NEG	NEG
ICP5062 (Portugal)	POS	NEG	POS	NEG	POS	NEG	NEG	NEG	NEG	NEG	NEG	NEG	POS	NEG	NEG	NEG	NEG
Strain 1063_ATCC 33592 FDAARGOS_3 (USA)	POS	NEG	POS	NEG	POS	NEG	NEG	NEG	NEG	NEG	NEG	NEG	POS	NEG	NEG	NEG	NEG
	POS	NEG	POS	NEG	POS	NEG	NEG	NEG	NEG	NEG	NEG	NEG	POS	NEG	NEG	NEG	NEG
Russia-07_0110_Kurgan	POS	NEG	POS	NEG	POS	NEG	NEG	NEG	NEG	NEG	NEG	NEG	POS	NEG	NEG	NEG	NEG
Russia-11_0164_Kurgan	POS	NEG	POS	NEG	POS	NEG	NEG	NEG	NEG	NEG	NEG	NEG	POS	NEG	NEG	NEG	NEG
Russia-20_0367_Kurgan	POS	NEG	POS	NEG	POS	NEG	NEG	NEG	NEG	NEG	NEG	NEG	POS	NEG	NEG	NEG	NEG
Russia-21_0390_Chelyabinsk	POS	NEG	POS	NEG	POS	NEG	NEG	NEG	NEG	NEG	NEG	NEG	POS	NEG	NEG	NEG	NEG
>"South American/ Midd																RARE	
AGT1 (Argentina)	POS	NEG	POS	NEG	POS	NEG	NEG	NEG	NEG	NEG	NEG	NEG	POS	NEG	NEG	NEG	NEG
AGT120 (Argentina)	POS	NEG	POS	NEG	POS	NEG	NEG	NEG	NEG	NEG	NEG	NEG	POS	NEG	NEG	NEG	NEG
AGT67 (Argentina)	POS	NEG	POS	NEG	POS	NEG	NEG	NEG	NEG	NEG	NEG	NEG	POS	NEG	NEG	NEG	NEG
AGT9 (Argentina)	POS	NEG	POS	NEG	POS	NEG	NEG	NEG	NEG	NEG	NEG	NEG	POS	NEG	NEG	NEG	NEG
RA3 (Argentina)	POS	NEG	POS	NEG	POS	NEG	NEG	NEG	NEG	NEG	NEG	NEG	POS	NEG	NEG	NEG	NEG
RA6 (Argentina)	POS	NEG	POS	NEG	POS	NEG	NEG	NEG	NEG	NEG	NEG	NEG	POS	NEG	NEG	NEG	NEG
RA7 (Argentina)	POS	NEG	POS	NEG	POS	NEG	NEG	NEG	NEG	NEG	NEG	NEG	POS	NEG	NEG	NEG	NEG
Be62 (Brazil)	POS	NEG	POS	NEG	POS	NEG	NEG	NEG	NEG	NEG	NEG	NEG	POS	NEG	NEG	NEG	NEG
Bmb9393 (Brazil)	POS	NEG	POS	NEG	POS	NEG	NEG	NEG	NEG	NEG	NEG	NEG	POS	NEG	NEG	NEG	NEG
BRA36 (Brazil)	POS	NEG	POS	NEG	POS	NEG	NEG	NEG	NEG	NEG	NEG	NEG	POS	NEG	NEG	NEG	NEG
BZ48 (Brazil)	POS	NEG	POS	NEG	POS	NEG	NEG	NEG	NEG	NEG	NEG	NEG	POS	NEG	NEG	NEG	NEG
Gv51 (Brazil)	POS	NEG	POS	NEG	POS	NEG	NEG	NEG	NEG	NEG	NEG	NEG	POS	NEG	NEG	NEG	NEG
Gv69 (Brazil)	POS	NEG	POS	NEG	POS	NEG	NEG	NEG	NEG	NEG	NEG	NEG	POS	NEG	NEG	NEG	NEG
Gv88 (Brazil)	POS	NEG	POS	NEG	POS	NEG	NEG	NEG	NEG	NEG	NEG	NEG	POS	NEG	NEG	NEG	NEG
HC1335 (Brazil)	POS	NEG	POS	NEG	POS	NEG	NEG	NEG	NEG	NEG	NEG	NEG	POS	NEG	NEG	NEG	NEG
HC1340 (Brazil)	POS	NEG	POS	NEG	POS	NEG	NEG	NEG	NEG	NEG	NEG	NEG	POS	NEG	NEG	NEG	NEG
HC556 (Brazil)	POS	NEG	POS	NEG	POS	NEG	NEG	NEG	NEG	NEG	NEG	NEG	POS	NEG	NEG	NEG	NEG
HU25 (Brazil)	POS	NEG	POS	NEG	POS	NEG	NEG	NEG	NEG	NEG	NEG	NEG	POS	NEG	NEG	NEG	NEG
UB563 (Brazil)	POS	NEG	POS	NEG	POS	NEG	NEG	NEG	NEG	NEG	NEG	NEG	POS	NEG	NEG	NEG	NEG
2A8 (Czech Republic)	POS	NEG	POS	NEG	POS	NEG	NEG	NEG	NEG	NEG	NEG	NEG	POS	NEG	NEG	NEG	NEG
M1229 (Denmark)	POS	NEG	POS	NEG	POS	NEG	NEG	NEG	NEG	NEG	NEG	NEG	POS	NEG	NEG	NEG	NEG
FRICAR (France)	POS	NEG	POS	NEG	POS	NEG	NEG	NEG	NEG	NEG	NEG	NEG	POS	NEG	NEG	NEG	NEG
D90 (Germany)	POS	NEG	POS	NEG	POS	NEG	NEG	NEG	NEG	NEG	NEG	NEG	POS	NEG	NEG	NEG	NEG
LIT68 (Lithuania)	POS	NEG	POS	NEG	POS	NEG	NEG	NEG	NEG	NEG	NEG	NEG	POS	NEG	NEG	NEG	NEG
LIT76 (Lithuania)	POS	NEG	POS	NEG	POS	NEG	NEG	NEG	NEG	NEG	NEG	NEG	POS	NEG	NEG	NEG	NEG
HGSA142 (Portugal)	POS	NEG	POS	NEG	POS	NEG	NEG	NEG	NEG	NEG	NEG	NEG	POS	NEG	NEG	NEG	NEG
HGSA9 (Portugal)	POS	NEG	POS	NEG	POS	NEG	NEG	NEG	NEG	NEG	NEG	NEG	POS	NEG	NEG	NEG	NEG
HSJ216 (Portugal)	POS	NEG	POS	NEG	POS	NEG	NEG	NEG	NEG	NEG	NEG	NEG	POS	NEG	NEG	NEG	NEG
M278 (Portugal)	POS	NEG	POS	NEG	POS	NEG	NEG	NEG	NEG	NEG	NEG	NEG	POS	NEG	NEG	NEG	NEG
ES26 (Spain)	POS	NEG	POS	NEG	POS	NEG	NEG	NEG	NEG	NEG	NEG	NEG	POS	NEG	NEG	NEG	NEG
UK102 (UK)	POS	POS	POS	NEG	POS	POS	POS	NEG	POS	AMB	POS	NEG	POS	NEG	POS	POS	NEG
URU34 (Uruguay)	POS	NEG	POS	NEG	POS	NEG	NEG	NEG	NEG	NEG	NEG	NEG	POS	NEG	NEG	NEG	NEG
IS-125	POS	NEG	POS	NEG	POS	NEG	NEG	NEG	NEG	NEG	NEG	NEG	POS	NEG	NEG	NEG	NEG
IS-157	POS	NEG	POS	NEG	POS	NEG	NEG	NEG	NEG	NEG	NEG	NEG	POS	NEG	NEG	NEG	NEG
Ecuador_1	POS	NEG	POS	NEG	POS	NEG	NEG	NEG	NEG	NEG	NEG	NEG	POS	NEG	NEG	NEG	NEG
Ecuador_2	POS	NEG	POS	NEG	POS	NEG	NEG	NEG	NEG	NEG	NEG	NEG	POS	NEG	NEG	NEG	NEG
Dresden_15ANR574263	POS	NEG	POS	NEG	POS	NEG	NEG	NEG	NEG	NEG	NEG	NEG	POS	AMB	POS	NEG	NEG
UK-EMRSA-11	POS	NEG	POS	NEG	POS	NEG	NEG	NEG	NEG	NEG	NEG	NEG	POS	NEG	NEG	NEG	NEG
>"South American/ Midd																NEG	
BRA2 (Brazil)	POS	NEG	POS	NEG	POS	NEG	NEG	NEG	NEG	NEG	NEG	NEG	POS	NEG	NEG	NEG	NEG
CHL1 (Chile)	POS	NEG	POS	NEG	POS	NEG	NEG	NEG	NEG	NEG	NEG	NEG	POS	NEG	NEG	NEG	NEG
CHL151 (Chile)	POS	NEG	POS	NEG	POS	NEG	NEG	NEG	NEG	NEG	NEG	NEG	POS	NEG	NEG	NEG	NEG
UCL378 (Chile)	POS	NEG	POS	NEG	POS	NEG	NEG	NEG	NEG	NEG	NEG	NEG	POS	NEG	NEG	NEG	NEG
UP72 (Peru)	POS	NEG	POS	NEG	POS	NEG	NEG	NEG	NEG	NEG	NEG	NEG	POS	NEG	NEG	NEG	NEG
UP81 (Peru)	POS	NEG	POS	NEG	POS	NEG	NEG	NEG	NEG	NEG	NEG	NEG	POS	NEG	NEG	NEG	NEG
>"South American/ Midd																NEG	
UP1073 (Peru)	POS	NEG	POS	NEG	POS	NEG	NEG	NEG	NEG	NEG	NEG	NEG	POS	NEG	NEG	NEG	NEG

STRAIN / ISOLATE	HEAVY METAL RESIST																
	cadA						cadD								cadX (plasmid)		cadX (SCC)
	cadA (cons)	cadA (pTW20)	cadA (TN554)	cadA (Sepi)	cadC (TN554)	cadC (pI258)	cadD (total)	hp1548_cadD	hp1340_cadD	hp1339_cadD	hp1466_cadD	hp1547_cadD	cadD (R35)	cadD (Smas)	cadX (plasmid probe I)	cadX (plasmid probe II)	cadX (JCSC6943)
	Cadmium transport protein D						cadmium efflux adenosine triphosphatase								Putative regulator of cadmium efflux		
>"South American/ Mid																VAR	
LIT89 (Lithuania)	POS	NEG	POS	NEG	POS	NEG	NEG	NEG	NEG	NEG	NEG	NEG	POS	NEG	NEG	NEG	NEG
MRSA_PR1 (Malaysia)	NEG	NEG	POS	NEG	NEG	NEG	NEG	NEG	NEG	NEG	NEG	NEG	POS	NEG	NEG	NEG	NEG
Riyadh-2888905-R	POS	POS	NEG	NEG	POS	POS	POS	AMB	POS	POS	POS	NEG	POS	NEG	POS	POS	NEG
Riyadh-2888915-BC	POS	POS	NEG	NEG	POS	POS	POS	AMB	POS	POS	POS	NEG	POS	NEG	POS	POS	NEG
Riyadh-2793706-R	POS	POS	NEG	NEG	POS	POS	POS	NEG	POS	POS	POS	NEG	POS	NEG	POS	POS	NEG
>"South American/ Mid																POS	
Riyadh_SSTI_18_3502925	POS	AMB	NEG	NEG	POS	POS	POS	POS	POS	POS	POS	NEG	POS	NEG	NEG	POS	NEG
Riyadh-2822825-W	POS	POS	NEG	NEG	POS	POS	POS	POS	POS	POS	POS	NEG	POS	NEG	POS	POS	NEG
>"South American/ Mid																NEG	
UK-EMRSA-9	POS	NEG	POS	NEG	POS	NEG	NEG	NEG	NEG	NEG	NEG	NEG	POS	NEG	NEG	NEG	NEG
>"South American/ Mid																NEG	
Lome_HT20020815	POS	NEG	POS	NEG	POS	NEG	NEG	NEG	NEG	NEG	NEG	NEG	POS	NEG	NEG	NEG	NEG
>"South American/ Mid																VAR	
NA32 (Denmark)	POS	NEG	POS	NEG	POS	NEG	NEG	NEG	NEG	NEG	NEG	NEG	POS	NEG	NEG	NEG	NEG
H24 (Egypt)	POS	NEG	POS	NEG	POS	NEG	NEG	NEG	NEG	NEG	NEG	NEG	POS	NEG	NEG	NEG	NEG
ATCC BAA-39 (=HUSA304) (Hungary)	POS	NEG	POS	NEG	POS	NEG	NEG	NEG	NEG	NEG	NEG	NEG	POS	NEG	NEG	NEG	NEG
HUSA304 (Hungary)	POS	NEG	POS	NEG	POS	NEG	NEG	NEG	NEG	NEG	NEG	NEG	POS	NEG	NEG	NEG	NEG
HU106 (Hungary)	POS	NEG	POS	NEG	POS	NEG	NEG	NEG	NEG	NEG	NEG	NEG	POS	NEG	NEG	NEG	NEG
BSAC27 (UK)	POS	POS	POS	NEG	POS	POS	NEG	NEG	NEG	NEG	NEG	NEG	POS	NEG	NEG	NEG	NEG
NCTC13131, UK-EMRSA-4 (UK)	POS	POS	POS	NEG	POS	POS	POS	NEG	POS	AMB	POS	NEG	POS	NEG	POS	POS	NEG
	POS	POS	POS	NEG	POS	POS	POS	AMB	POS	POS	POS	AMB	POS	NEG	POS	POS	NEG
Algiers_HT20040080	POS	NEG	POS	NEG	POS	NEG	NEG	NEG	NEG	NEG	NEG	NEG	POS	NEG	NEG	NEG	NEG
Dublin-DSH_AR09_0.0066	POS	NEG	POS	NEG	POS	NEG	NEG	NEG	NEG	NEG	NEG	NEG	POS	NEG	NEG	NEG	NEG
Dublin-DSH_AR09_0-0065	POS	NEG	POS	NEG	POS	NEG	NEG	NEG	NEG	NEG	NEG	NEG	POS	NEG	NEG	NEG	NEG
Dublin-DSH_Phenotype-III_84	POS	NEG	POS	NEG	POS	NEG	NEG	NEG	NEG	NEG	NEG	NEG	POS	NEG	NEG	NEG	NEG
Hong Kong_130	POS	NEG	POS	NEG	POS	NEG	NEG	NEG	NEG	NEG	NEG	NEG	POS	NEG	NEG	NEG	NEG
Kuwait_018	POS	NEG	POS	NEG	POS	NEG	NEG	NEG	NEG	NEG	NEG	NEG	POS	NEG	NEG	NEG	NEG
Perth_08-17726	POS	NEG	POS	NEG	POS	NEG	NEG	NEG	NEG	NEG	NEG	NEG	POS	NEG	NEG	NEG	NEG
Riyadh_Alfaisal/KKKSUH_86_MRS-14-279	POS	POS	POS	NEG	POS	POS	POS	NEG	POS	POS	POS	NEG	POS	NEG	POS	POS	NEG
Riyadh_Alfaisal-04_Z3861_831588	POS	POS	POS	NEG	POS	POS	POS	NEG	POS	POS	POS	NEG	POS	NEG	POS	POS	NEG
Riyadh_Alfaisal-30_S15724_1108013	POS	POS	POS	NEG	POS	POS	POS	NEG	POS	POS	POS	NEG	POS	NEG	POS	POS	NEG
Riyadh_Alfaisal-6_22A_13_B3992_397721	POS	POS	POS	NEG	POS	POS	POS	NEG	POS	POS	POS	NEG	POS	NEG	POS	POS	NEG
Riyadh_SSTI_52_3615482	POS	POS	POS	NEG	POS	POS	POS	AMB	POS	POS	POS	NEG	POS	NEG	POS	POS	NEG
Riyadh-2817276-2	POS	POS	POS	NEG	POS	POS	POS	POS	POS	POS	POS	NEG	POS	NEG	POS	POS	NEG
Riyadh-2891670-W	POS	POS	POS	NEG	POS	POS	POS	POS	POS	POS	POS	NEG	POS	NEG	POS	POS	NEG
Riyadh-3006920-W	POS	POS	POS	NEG	POS	POS	POS	AMB	POS	POS	POS	NEG	POS	NEG	POS	POS	NEG
Riyadh-R2567782	POS	POS	POS	NEG	POS	POS	POS	POS	POS	POS	POS	NEG	POS	NEG	POS	POS	NEG
Russia-18_0252_Krasnoyarsk_SK2	POS	NEG	POS	NEG	POS	NEG	NEG	NEG	NEG	NEG	NEG	NEG	POS	NEG	NEG	NEG	NEG
UK-EMRSA-7	POS	POS	POS	NEG	POS	POS	POS	POS	POS	POS	POS	POS	POS	NEG	POS	POS	NEG
>"South American/ Mid																NEG	
Dublin-DSH_AR23_0073	NEG	NEG	NEG	NEG	NEG	NEG	NEG	NEG	NEG	NEG	NEG	NEG	NEG	NEG	NEG	NEG	NEG
>Related to "South Ame																NEG	
MRSA-OC3 (Russia)	POS	NEG	POS	NEG	POS	NEG	NEG	NEG	NEG	NEG	NEG	NEG	POS	NEG	NEG	NEG	NEG
Russia-12_0176_Krasnoyarsk	POS	NEG	POS	NEG	POS	NEG	NEG	NEG	NEG	NEG	NEG	NEG	POS	NEG	NEG	NEG	NEG
Russia-13_0180_Krasnoyarsk	POS	NEG	POS	NEG	POS	NEG	NEG	NEG	NEG	NEG	NEG	NEG	POS	NEG	NEG	NEG	NEG
Russia-16_0249_Krasnoyarsk	POS	NEG	POS	NEG	POS	NEG	NEG	NEG	NEG	NEG	NEG	NEG	POS	NEG	NEG	NEG	NEG
Russia-17_0250_Krasnoyarsk	POS	NEG	POS	NEG	POS	NEG	NEG	NEG	NEG	NEG	NEG	NEG	POS	NEG	NEG	NEG	NEG
>Related to "South Ame																NEG	
URU110 (Uruguay)	POS	NEG	POS	NEG	POS	NEG	NEG	NEG	NEG	NEG	NEG	NEG	POS	NEG	NEG	NEG	NEG
>Related to "South Ame																NEG	
DS_014 (Thailand)	POS	NEG	POS	NEG	POS	NEG	NEG	NEG	NEG	NEG	NEG	NEG	POS	NEG	NEG	NEG	NEG
>Unassigned Middle Eas																NEG	
Frankfurt_Oder_0490031797 (Libya)	POS	NEG	POS	NEG	POS	NEG	POS	NEG	AMB	AMB	POS	NEG	POS	AMB	POS	NEG	neg
Kuwait_192	POS	NEG	POS	NEG	POS	NEG	NEG	NEG	NEG	NEG	NEG	NEG	POS	NEG	NEG	NEG	NEG
Riyadh-9	POS	NEG	POS	NEG	POS	NEG	NEG	NEG	NEG	NEG	NEG	NEG	POS	NEG	NEG	NEG	NEG
Russia-24_0407_Moscow	POS	NEG	POS	NEG	POS	NEG	NEG	NEG	NEG	NEG	NEG	NEG	POS	NEG	NEG	NEG	NEG

STRAIN / ISOLATE	RESISTANCE : PENICILLINASE							RESISTANCE : MLS-ANTIBIOTICS										
	blaZ	blaI	blaR	hp_blaR-811	hp_blaR-812	blaZ (Slent)	blaZ (Ssap)	ermA	ermA-43	ermB	ermC		ermF	ermT	linA/lnuA	lnuB	lsa-B	lsa-E
	blaZ	blaI	blaR	hp_blaR-811	hp_blaR-812	blaZ (Slent)	blaZ (Ssap)	ermA	ermA-43	ermB	ermC	ermC-GM	ermF	ermT	linA/lnuA	lnuB	lsa-B	lsa-E
	beta-lactamase	beta lactamase repressor (inhibitor)	beta-lactamase regulatory protein					rRNA adenine N-6-methyl-transferase, erythromycin/clindamycin resistance		erythro-mycin/clindamycin resistance	0,00	rRNA adenine methylase-like protein ErmGM	rRNA adenine N-6-methyltransferase	0,00	Lincosamid-Nucleotidyltransferase	lincosamide nucleotidyltransferase	lincosamide ABC transporter	lincosamide ABC transporter
>"Eurasian Clade": TUR1	POS							POS			POS							
TUR1 (Turkey)	POS	POS	POS	NEG	POS	NEG	NEG	POS	NEG	NEG	POS	NEG	NEG	NEG	NEG	NEG	NEG	NEG
TUR9 (Turkey)	POS	POS	POS	NEG	POS	NEG	NEG	POS	NEG	NEG	POS	NEG	NEG	NEG	NEG	NEG	NEG	NEG
>"Eurasian Clade" : T013	POS							POS			NEG							
T0131 (China)	POS	POS	POS	NEG	POS	NEG	NEG	POS	NEG	NEG	NEG	NEG	NEG	NEG	NEG	NEG	NEG	NEG
MU4 (Turkey)	POS	POS	POS	NEG	POS	NEG	NEG	POS	NEG	NEG	POS	NEG	NEG	NEG	NEG	NEG	NEG	NEG
DEU11 (Turkey)	POS	POS	POS	NEG	POS	NEG	NEG	POS	NEG	NEG	POS	NEG	NEG	NEG	NEG	NEG	NEG	NEG
DEU16 (Turkey)	POS	POS	POS	NEG	POS	NEG	NEG	POS	NEG	NEG	POS	NEG	NEG	NEG	NEG	NEG	NEG	NEG
Romania_Iasi_MRSA-07_54	POS	POS	POS	NEG	POS	NEG	NEG	POS	NEG	NEG	NEG	NEG	NEG	NEG	NEG	NEG	NEG	NEG
>"Eurasian Clade": arsC-	POS							POS			NEG							
Romania_Iasi_MRSA-05_46	POS	POS	POS	NEG	POS	NEG	NEG	POS	NEG	NEG	NEG	NEG	NEG	NEG	NEG	NEG	NEG	NEG
Romania_Iasi_MRSA-06_47	POS	POS	POS	NEG	POS	NEG	NEG	POS	NEG	NEG	NEG	NEG	NEG	NEG	NEG	NEG	NEG	NEG
>"Eurasian Clade": 16K/(	POS							COMM			VAR							
CN79 (China)	POS	POS	POS	NEG	POS	NEG	NEG	NEG	NEG	NEG	NEG	NEG	NEG	NEG	NEG	NEG	NEG	NEG
CUHK_BJ2002 (China)	POS	POS	POS	NEG	POS	NEG	NEG	POS	NEG	NEG	NEG	NEG	NEG	NEG	NEG	NEG	NEG	NEG
CUHK_BJ2007 (China)	POS	POS	POS	NEG	POS	NEG	NEG	POS	NEG	NEG	POS	NEG	NEG	NEG	NEG	NEG	NEG	NEG
3HK (Czech Republic)	POS	POS	POS	NEG	POS	NEG	NEG	POS	NEG	NEG	POS	NEG	NEG	NEG	NEG	NEG	NEG	NEG
HU109 (Hungary)	POS	POS	POS	NEG	POS	NEG	NEG	POS	NEG	NEG	POS	NEG	NEG	NEG	NEG	NEG	NEG	NEG
HUR18 (Hungary)	POS	POS	POS	NEG	POS	NEG	NEG	POS	NEG	NEG	NEG	NEG	NEG	NEG	NEG	NEG	NEG	NEG
H482 (Romania)	POS	POS	POS	NEG	POS	NEG	NEG	POS	NEG	NEG	NEG	NEG	NEG	NEG	NEG	NEG	NEG	NEG
16K (Russia)	POS	POS	POS	NEG	POS	NEG	NEG	POS	NEG	NEG	NEG	NEG	NEG	NEG	NEG	NEG	NEG	NEG
DEU10 (Turkey)	POS	POS	POS	NEG	POS	NEG	NEG	POS	NEG	NEG	NEG	NEG	NEG	NEG	NEG	NEG	NEG	NEG
DEU12 (Turkey)	POS	POS	POS	NEG	POS	NEG	NEG	POS	NEG	NEG	NEG	NEG	NEG	NEG	NEG	NEG	NEG	NEG
DEU14 (Turkey)	POS	POS	POS	NEG	POS	NEG	NEG	POS	NEG	NEG	NEG	NEG	NEG	NEG	NEG	NEG	NEG	NEG
DEU15 (Turkey)	POS	POS	POS	NEG	POS	NEG	NEG	POS	NEG	NEG	NEG	NEG	NEG	NEG	NEG	NEG	NEG	NEG
DEU17 (Turkey)	POS	POS	POS	NEG	POS	NEG	NEG	POS	NEG	NEG	NEG	NEG	NEG	NEG	NEG	NEG	NEG	NEG
DEU19 (Turkey)	POS	POS	POS	NEG	POS	NEG	NEG	POS	NEG	NEG	NEG	NEG	NEG	NEG	NEG	NEG	NEG	NEG
DEU2 (Turkey)	POS	POS	POS	NEG	POS	NEG	NEG	POS	NEG	NEG	POS	NEG	NEG	NEG	NEG	NEG	NEG	NEG
DEU20 (Turkey)	POS	POS	POS	NEG	POS	NEG	NEG	POS	NEG	NEG	NEG	NEG	NEG	NEG	NEG	NEG	NEG	NEG
DEU23 (Turkey)	POS	POS	POS	NEG	POS	NEG	NEG	POS	NEG	NEG	NEG	NEG	NEG	NEG	NEG	NEG	NEG	NEG
DEU3 (Turkey)	POS	POS	POS	NEG	POS	NEG	NEG	POS	NEG	NEG	NEG	NEG	NEG	NEG	NEG	NEG	NEG	NEG
DEU5 (Turkey)	POS	POS	POS	NEG	POS	NEG	NEG	POS	NEG	NEG	NEG	NEG	NEG	NEG	NEG	NEG	NEG	NEG
DEU6 (Turkey)	POS	POS	POS	NEG	POS	NEG	NEG	POS	NEG	NEG	POS	NEG	NEG	NEG	NEG	NEG	NEG	NEG
DEU8 (Turkey)	POS	POS	POS	NEG	POS	NEG	NEG	POS	NEG	NEG	NEG	NEG	NEG	NEG	NEG	NEG	NEG	NEG
HU11 (Turkey)	POS	POS	POS	NEG	POS	NEG	NEG	POS	NEG	NEG	NEG	NEG	NEG	NEG	NEG	NEG	NEG	NEG
HU13 (Turkey)	POS	POS	POS	NEG	POS	NEG	NEG	POS	NEG	NEG	POS	NEG	NEG	NEG	NEG	NEG	NEG	NEG
HU14 (Turkey)	POS	POS	POS	NEG	POS	NEG	NEG	POS	NEG	NEG	NEG	NEG	NEG	NEG	NEG	NEG	NEG	NEG
HU15 (Turkey)	POS	POS	POS	NEG	POS	NEG	NEG	POS	NEG	NEG	NEG	NEG	NEG	NEG	NEG	NEG	NEG	NEG
HU16 (Turkey)	POS	POS	POS	NEG	POS	NEG	NEG	POS	NEG	NEG	NEG	NEG	NEG	NEG	NEG	NEG	NEG	NEG
HU17 (Turkey)	POS	POS	POS	NEG	POS	NEG	NEG	POS	NEG	NEG	POS	NEG	NEG	NEG	NEG	NEG	NEG	NEG
HU21 (Turkey)	POS	POS	POS	NEG	POS	NEG	NEG	POS	NEG	NEG	NEG	NEG	NEG	NEG	NEG	NEG	NEG	NEG
HU23 (Turkey)	POS	POS	POS	NEG	POS	NEG	NEG	POS	NEG	NEG	NEG	NEG	NEG	NEG	NEG	NEG	NEG	NEG
HU26 (Turkey)	POS	POS	POS	NEG	POS	NEG	NEG	POS	NEG	NEG	NEG	NEG	NEG	NEG	NEG	NEG	NEG	NEG
HU4 (Turkey)	POS	POS	POS	NEG	POS	NEG	NEG	POS	NEG	NEG	NEG	NEG	NEG	NEG	NEG	NEG	NEG	NEG
HU5 (Turkey)	POS	POS	POS	NEG	POS	NEG	NEG	POS	NEG	NEG	POS	NEG	NEG	NEG	NEG	NEG	NEG	NEG
HU6 (Turkey)	POS	POS	POS	NEG	POS	NEG	NEG	POS	NEG	NEG	NEG	NEG	NEG	NEG	NEG	NEG	NEG	NEG
HU7 (Turkey)	POS	POS	POS	NEG	POS	NEG	NEG	POS	NEG	NEG	NEG	NEG	NEG	NEG	NEG	NEG	NEG	NEG
HU8 (Turkey)	POS	POS	POS	NEG	POS	NEG	NEG	POS	NEG	NEG	NEG	NEG	NEG	NEG	NEG	NEG	NEG	NEG
HU9 (Turkey)	POS	POS	POS	NEG	POS	NEG	NEG	POS	NEG	NEG	NEG	NEG	NEG	NEG	NEG	NEG	NEG	NEG
IU1 (Turkey)	POS	POS	POS	NEG	POS	NEG	NEG	POS	NEG	NEG	POS	NEG	NEG	NEG	NEG	NEG	NEG	NEG
IU10 (Turkey)	POS	POS	POS	NEG	POS	NEG	NEG	POS	NEG	NEG	POS	NEG	NEG	NEG	NEG	NEG	NEG	NEG
IU11 (Turkey)	POS	POS	POS	NEG	POS	NEG	NEG	POS	NEG	NEG	POS	NEG	NEG	NEG	NEG	NEG	NEG	NEG
IU12 (Turkey)	POS	POS	POS	NEG	POS	NEG	NEG	POS	NEG	NEG	POS	NEG	NEG	NEG	NEG	NEG	NEG	NEG
IU13 (Turkey)	POS	POS	POS	NEG	POS	NEG	NEG	POS	NEG	NEG	NEG	NEG	NEG	NEG	NEG	NEG	NEG	NEG
IU15 (Turkey)	POS	POS	POS	NEG	POS	NEG	NEG	POS	NEG	NEG	NEG	NEG	NEG	NEG	NEG	NEG	NEG	NEG
IU18 (Turkey)	POS	POS	POS	NEG	POS	NEG	NEG	POS	NEG	NEG	NEG	NEG	NEG	NEG	NEG	NEG	NEG	NEG
IU19 (Turkey)	POS	POS	POS	NEG	POS	NEG	NEG	POS	NEG	NEG	NEG	NEG	NEG	NEG	NEG	NEG	NEG	NEG
IU2 (Turkey)	POS	POS	POS	NEG	POS	NEG	NEG	POS	NEG	NEG	NEG	NEG	NEG	NEG	NEG	NEG	NEG	NEG
IU4 (Turkey)	POS	POS	POS	NEG	POS	NEG	NEG	POS	NEG	NEG	NEG	NEG	NEG	NEG	NEG	NEG	NEG	NEG
IU6 (Turkey)	POS	POS	POS	NEG	POS	NEG	NEG	POS	NEG	NEG	NEG	NEG	NEG	NEG	NEG	NEG	NEG	NEG
IU7 (Turkey)	POS	POS	POS	NEG	POS	NEG	NEG	POS	NEG	NEG	POS	NEG	NEG	NEG	NEG	NEG	NEG	NEG
IU9 (Turkey)	POS	POS	POS	NEG	POS	NEG	NEG	POS	NEG	NEG	POS	NEG	NEG	NEG	NEG	NEG	NEG	NEG
MU1 (Turkey)	POS	POS	POS	NEG	POS	NEG	NEG	POS	NEG	NEG	NEG	NEG	NEG	NEG	NEG	NEG	NEG	NEG
MU10 (Turkey)	POS	POS	POS	NEG	POS	NEG	NEG	POS	NEG	NEG	POS	NEG	NEG	NEG	NEG	NEG	NEG	NEG
MU20 (Turkey)	POS	POS	POS	NEG	POS	NEG	NEG	POS	NEG	NEG	NEG	NEG	NEG	NEG	NEG	NEG	NEG	NEG
Mu3 (Turkey)	POS	POS	POS	NEG	POS	NEG	NEG	POS	NEG	NEG	POS	NEG	NEG	NEG	NEG	NEG	NEG	NEG
MU5 (Turkey)	POS	POS	POS	NEG	POS	NEG	NEG	POS	NEG	NEG	NEG	NEG	NEG	NEG	NEG	NEG	NEG	NEG
MU6 (Turkey)	POS	POS	POS	NEG	POS	NEG	NEG	POS	NEG	NEG	NEG	NEG	NEG	NEG	NEG	NEG	NEG	NEG
MU7 (Turkey)	POS	POS	POS	NEG	POS	NEG	NEG	POS	NEG	NEG	NEG	NEG	NEG	NEG	NEG	NEG	NEG	NEG
TUR27 (Turkey)	POS	POS	POS	NEG	POS	NEG	NEG	POS	NEG	NEG	NEG	NEG	NEG	NEG	NEG	NEG	NEG	NEG
NCTR 32S	POS	POS	POS	NEG	POS	NEG	NEG	POS	NEG	NEG	NEG	NEG	NEG	NEG	NEG	NEG	NEG	NEG
SA02_A4 = Russia-06_0085_Moscow	POS	POS	POS	NEG	POS	NEG	NEG	POS	NEG	NEG	NEG	NEG	NEG	NEG	NEG	NEG	NEG	NEG
	POS	POS	POS	NEG	POS	NEG	NEG	POS	NEG	NEG	NEG	NEG	NEG	NEG	NEG	NEG	NEG	NEG
Dresden_08V35987 (Turkey)	POS	POS	POS	NEG	POS	NEG	NEG	POS	NEG	NEG	NEG	NEG	NEG	NEG	NEG	NEG	NEG	NEG
Dresden_17ANR577152	POS	POS	POS	AMB	POS	NEG	NEG	POS	NEG	NEG	NEG	NEG	NEG	NEG	NEG	NEG	NEG	NEG
Dresden_17ANR580374 (Makedonia)	POS	POS	POS	NEG	POS	NEG	NEG	POS	NEG	NEG	NEG	NEG	NEG	NEG	NEG	NEG	NEG	NEG
Hong Kong_69-II	POS	POS	POS	NEG	POS	NEG	NEG	POS	NEG	NEG	NEG	NEG	NEG	NEG	NEG	NEG	NEG	NEG
Hong Kong_93	POS	POS	POS	AMB	POS	NEG	NEG	POS	NEG	NEG	NEG	NEG	NEG	NEG	NEG	NEG	NEG	NEG
Rawalpindi_Kidney Center_03_SK1	POS	POS	POS	NEG	POS	NEG	NEG	POS	NEG	NEG	POS	NEG	NEG	NEG	NEG	NEG	NEG	NEG
Rawalpindi_Kidney Center_08	POS	POS	POS	NEG	POS	NEG	NEG	POS	NEG	NEG	POS	NEG	NEG	NEG	NEG	NEG	NEG	NEG
Rawalpindi_Kidney Center_10	POS	POS	POS	NEG	POS	NEG	NEG	POS	NEG	NEG	POS	NEG	NEG	NEG	NEG	NEG	NEG	NEG
Rawalpindi_Kidney Center_19	POS	POS	POS	POS	POS	NEG	NEG	POS	NEG	NEG	NEG	NEG	NEG	NEG	NEG	NEG	NEG	NEG
Rawalpindi_Kidney Center_50_SK1	POS	POS	POS	NEG	POS	NEG	NEG	POS	NEG	NEG	POS	NEG	NEG	NEG	NEG	NEG	NEG	NEG
Romania_Iasi_BC-49_430	POS	POS	POS	NEG	POS	NEG	NEG	POS	NEG	NEG	NEG	NEG	NEG	NEG	NEG	NEG	NEG	NEG
Romania_Iasi_MRSA_284	POS	POS	POS	NEG	NEG	NEG	NEG	POS	NEG	NEG	NEG	NEG	NEG	NEG	NEG	NEG	NEG	NEG
Romania_Iasi_MRSA-32_318	POS	POS	POS	NEG	POS	NEG	NEG	POS	NEG	NEG	NEG	NEG	NEG	NEG	NEG	#ZAHL!	#ZAHL!	NEG
Romania_Iasi_MRSA-54_430	POS	POS	POS	NEG	POS	NEG	NEG	POS	NEG	NEG	NEG	NEG	NEG	NEG	NEG	NEG	NEG	NEG
Romania_Iasi_SSTI-01_2	POS	POS	POS	NEG	POS	NEG	NEG	POS	NEG	NEG	NEG	NEG	NEG	NEG	NEG	NEG	NEG	NEG
Romania_Iasi_SSTI-10_101	POS	POS	POS	NEG	POS	NEG	NEG	POS	NEG	NEG	NEG	NEG	NEG	NEG	NEG	NEG	NEG	NEG
Romania_Iasi_SSTI-11_106	POS	POS	POS	NEG	POS	NEG	NEG	POS	NEG	NEG	NEG	NEG	NEG	NEG	NEG	NEG	NEG	NEG
Russia-01_0001_SaintPetersburg	POS	POS	POS	NEG	POS	NEG	NEG	POS	NEG	NEG	NEG	NEG	NEG	NEG	NEG	NEG	NEG	NEG
Russia-02_0004_SaintPetersburg	POS	POS	POS	NEG	POS	NEG	NEG	POS	NEG	NEG	NEG	NEG	NEG	NEG	NEG	NEG	NEG	NEG
Russia-03_0057_SaintPetersburg	POS	POS	POS	NEG	POS	NEG	NEG	POS	NEG	NEG	NEG	NEG	NEG	NEG	NEG	NEG	NEG	NEG
Russia-04_0076_Moscow	POS	POS	POS	NEG	POS	NEG	NEG	POS	NEG	NEG	NEG	NEG	NEG	NEG	NEG	NEG	NEG	NEG
Russia-05_0078_Moscow	POS	POS	POS	NEG	POS	NEG	NEG	POS	NEG	NEG	NEG	NEG	NEG	NEG	NEG	NEG	NEG	NEG
Russia-08_0135_Moscow	POS	POS	POS	NEG	POS	NEG	NEG	POS	NEG	NEG	NEG	NEG	NEG	NEG	NEG	NEG	NEG	NEG
Russia-09_0150_Kurgan	POS	POS	POS	NEG	POS	NEG	NEG	POS	NEG	NEG	NEG	NEG	NEG	NEG	NEG	NEG	NEG	NEG
Russia-10_0162_Kurgan	POS	POS	POS	NEG	POS	NEG	NEG	POS	NEG	NEG	NEG	NEG	NEG	NEG	NEG	NEG	NEG	NEG
Russia-14_0184_Moscow	POS	POS	POS	NEG	AMB	NEG	NEG	POS	NEG	NEG	NEG	NEG	NEG	NEG	NEG	NEG	NEG	NEG
Russia-15_0232_SaintPetersburg	POS	POS	POS	NEG	POS	NEG	NEG	POS	NEG	NEG	POS	NEG	NEG	NEG	NEG	NEG	NEG	NEG
Russia-22_0391_Chelyabinsk	POS	POS	POS	NEG	POS	NEG	NEG	POS	NEG	NEG	NEG	NEG	NEG	NEG	NEG	NEG	NEG	NEG
Russia-23_0392_Chelyabinsk	POS	POS	POS	NEG	POS	NEG	NEG	POS	NEG	NEG	NEG	NEG	NEG	NEG	NEG	NEG	NEG	NEG
>"Eurasian Clade" : IU17	POS							POS			POS							
IU17 (Turkey)	POS	POS	POS	NEG	POS	NEG	NEG	POS	NEG	NEG	POS	NEG	NEG	NEG	NEG	NEG	NEG	NEG
IU20 (Turkey)	POS	POS	POS	NEG	POS	NEG	NEG	POS	NEG	NEG	POS	NEG	NEG	NEG	NEG	NEG	NEG	NEG

STRAIN / ISOLATE	RESISTANCE : PENICILLINASE							RESISTANCE : MLS-ANTIBIOTICS										
	blaZ	blaI	blaR	hp_blaR-811	hp_blaR-812	blaZ (Slent)	blaZ (Ssap)	ermA	ermA-43	ermB	ermC		ermF	ermT	linA/lnuA	lnuB	lsa-B	lsa-E
	blaZ	blaI	blaR	hp_blaR-811	hp_blaR-812	blaZ (Slent)	blaZ (Ssap)	ermA	ermA-43	ermB	ermC	ermC-GM	ermF	ermT	linA/lnuA	lnuB	lsa-B	lsa-E
	beta-lactamase	beta lactamase repressor (inhibitor)	beta-lactamase regulatory protein					rRNA adenine N-6-methyl-transferase, erythromycin/clindamycin resistance		erythro-mycin/clindamycin resistance	0,00	rRNA adenine methylase-like protein ErmGM	rRNA adenine N-6-methyltransferase	0,00	Lincosamid-Nucleotidyltransferase	lincosamide nucleotidyltransferase	lincosamide ABC transporter	lincosamide ABC transporter
**>"Eurasian Clade": Spora**	**POS**							**POS**			**VAR**							
Dresden_16ANR581769	POS	POS	POS	NEG	POS	NEG	NEG	POS	NEG	NEG	NEG	NEG	NEG	NEG	NEG	NEG	NEG	NEG
Dresden_17ANR578769	POS	POS	POS	NEG	POS	NEG	NEG	POS	NEG	NEG	POS	NEG	NEG	NEG	NEG	NEG	NEG	NEG
**>"Eurasian Clade": Kuwa**	**POS**							**POS**			**POS**							
Kuwait_306	POS	POS	POS	NEG	POS	NEG	NEG	POS	NEG	NEG	POS	NEG	NEG	NEG	NEG	NEG	NEG	NEG
Kuwait_301	POS	POS	POS	NEG	POS	NEG	NEG	POS	NEG	NEG	POS	NEG	NEG	NEG	NEG	NEG	NEG	NEG
**>"European Clade": "Gre**		**POS**						**NEG**			**COMM**							
GRE18 (Greece)	POS	POS	POS	NEG	POS	NEG	NEG	NEG	NEG	NEG	POS	NEG	NEG	NEG	NEG	NEG	NEG	NEG
GRE317 (Greece)	POS	POS	POS	NEG	POS	NEG	NEG	NEG	NEG	NEG	POS	NEG	NEG	NEG	NEG	NEG	NEG	NEG
GRE4 (Greece)	POS	POS	POS	NEG	POS	NEG	NEG	NEG	NEG	NEG	POS	NEG	NEG	NEG	NEG	NEG	NEG	NEG
R35 (USA)	AMB	POS	POS	NEG	POS	NEG	NEG	NEG	NEG	NEG	POS	NEG	NEG	NEG	NEG	NEG	NEG	NEG
Casablanca_HT20060548	POS	POS	POS	NEG	POS	NEG	NEG	NEG	NEG	NEG	NEG	NEG	NEG	NEG	NEG	NEG	NEG	NEG
Casablanca_HT20060550	POS	POS	POS	NEG	POS	NEG	NEG	NEG	NEG	NEG	NEG	NEG	NEG	NEG	NEG	NEG	#ZAHL!	NEG
Dresden_01V34572 (Greece)	POS	POS	POS	NEG	POS	NEG	NEG	NEG	NEG		POS	NEG	NEG	NEG	NEG	NEG	NEG	NEG
Dresden_01V36469	POS	POS	POS	NEG	POS	NEG	NEG	NEG	NEG	NEG	POS	NEG	NEG	NEG	NEG	NEG	NEG	NEG
Dresden_01V39123	POS	POS	POS	NEG	POS	NEG	NEG	NEG	NEG	NEG	POS	NEG	NEG	NEG	NEG	NEG	NEG	NEG
Dresden_01V39143	POS	POS	POS	NEG	POS	NEG	NEG	NEG	NEG	NEG	POS	NEG	NEG	NEG	NEG	NEG	NEG	NEG
Greece 1_3680_Harmony	POS	POS	POS	NEG	POS	NEG	NEG	NEG	NEG	NEG	POS	NEG	NEG	NEG	NEG	NEG	NEG	NEG
**>"European Clade": UK-(**	**POS**							**POS**			**NEG**							
ANS46 (Australia)	POS	POS	POS	NEG	POS	NEG	NEG	POS	NEG	NEG	NEG	NEG	NEG	NEG	NEG	NEG	NEG	NEG
LHH1 (USA)	POS	POS	POS	NEG	POS	NEG	NEG	POS	NEG	NEG	NEG	NEG	NEG	NEG	NEG	NEG	NEG	NEG
Dublin-DSH_AR01_0-1085	POS	POS	POS	NEG	POS	NEG	NEG	POS	NEG	NEG	NEG	NEG	NEG	NEG	NEG	NEG	NEG	NEG
Dublin-DSH_AR01_0-1118	POS	POS	POS	NEG	POS	NEG	NEG	POS	NEG	NEG	NEG	NEG	NEG	NEG	NEG	NEG	NEG	NEG
Dublin-DSH_AR15_0098	POS	POS	POS	NEG	POS	NEG	NEG	POS	NEG	NEG	NEG	NEG	NEG	NEG	NEG	NEG	NEG	NEG
Dublin-DSH_AR15_0104	POS	POS	POS	NEG	POS	NEG	NEG	POS	NEG	NEG	NEG	NEG	NEG	NEG	NEG	NEG	NEG	NEG
UK_NCTC11939_UK-1_Harmony	POS	POS	POS	NEG	POS	NEG	NEG	POS	NEG	NEG	NEG	NEG	NEG	NEG	NEG	NEG	NEG	NEG
**>"European Clade": BK2**	**POS**							**POS**			**NEG**							
BK2421 (USA)	POS	POS	POS	NEG	POS	NEG	NEG	POS	NEG	NEG	NEG	NEG	NEG	NEG	NEG	NEG	NEG	NEG
**>"Aussi/NZ Clade", JKD6**	**pos**							**POS**			**NEG**							
JKD6009 (Australia)	POS	POS	POS	NEG	POS	NEG	NEG	POS	NEG	NEG	NEG	NEG	NEG	NEG	NEG	NEG	NEG	NEG
JKD6008 (New Zealand)	POS	POS	POS	NEG	POS	NEG	NEG	POS	NEG	NEG	NEG	NEG	NEG	NEG	NEG	NEG	NEG	NEG
Perth_2015-S20209	POS	POS	POS	NEG	POS	NEG	NEG	POS	NEG	NEG	NEG	NEG	NEG	NEG	NEG	NEG	NEG	NEG
Perth_2015-S20305	POS	POS	POS	NEG	POS	NEG	NEG	POS	NEG	NEG	NEG	NEG	NEG	NEG	NEG	NEG	NEG	NEG
Perth_2015-S20603	POS	POS	POS	NEG	POS	NEG	NEG	POS	NEG	NEG	NEG	NEG	NEG	NEG	NEG	NEG	NEG	NEG
Perth_2015-S20607	POS	POS	AMB	NEG	POS	NEG	NEG	POS	NEG	NEG	NEG	NEG	NEG	NEG	NEG	NEG	NEG	NEG
Perth_2015-S20608	POS	POS	POS	NEG	POS	NEG	NEG	POS	NEG	NEG	NEG	NEG	NEG	NEG	NEG	NEG	NEG	NEG
Perth_2015-S20610	POS	POS	AMB	NEG	POS	NEG	NEG	POS	NEG	NEG	NEG	NEG	NEG	NEG	NEG	NEG	NEG	NEG
Perth_2015-S20613	POS	POS	POS	NEG	POS	NEG	NEG	POS	NEG	NEG	NEG	NEG	NEG	NEG	NEG	NEG	NEG	NEG
Perth_2015-S20617	AMB	POS	AMB	NEG	POS	NEG	NEG	POS	NEG	NEG	NEG	NEG	NEG	NEG	NEG	NEG	NEG	NEG
Perth_2015-S20622	AMB	POS	AMB	NEG	NEG	NEG	NEG	POS	NEG	NEG	NEG	NEG	NEG	NEG	NEG	NEG	NEG	NEG
Perth_2015-S23619	POS	POS	POS	NEG	POS	NEG	NEG	POS	NEG	NEG	NEG	NEG	NEG	NEG	NEG	NEG	NEG	NEG
Perth_2015-S41210	POS	POS	POS	NEG	POS	NEG	NEG	POS	NEG	NEG	NEG	NEG	NEG	NEG	NEG	NEG	NEG	NEG
Perth_2015-S51001	POS	POS	POS	NEG	POS	NEG	NEG	POS	NEG	NEG	NEG	NEG	NEG	NEG	NEG	NEG	NEG	NEG
Perth_2015-S51003	POS	POS	POS	NEG	POS	NEG	NEG	POS	NEG	NEG	NEG	NEG	NEG	NEG	NEG	NEG	NEG	NEG
Perth_2015-S51006	POS	POS	POS	NEG	POS	NEG	NEG	POS	NEG	NEG	NEG	NEG	NEG	NEG	NEG	NEG	NEG	NEG
Perth_2015-S51007	POS	POS	POS	NEG	POS	NEG	NEG	POS	NEG	NEG	NEG	NEG	NEG	NEG	NEG	NEG	NEG	NEG
Perth_2015-S51008	POS	POS	POS	NEG	POS	NEG	NEG	POS	NEG	NEG	NEG	NEG	NEG	NEG	NEG	NEG	NEG	NEG
Perth_2015-S51014	POS	POS	AMB	NEG	POS	NEG	NEG	POS	NEG	NEG	NEG	NEG	NEG	NEG	NEG	NEG	NEG	NEG
Perth_2015-S51408	POS	POS	POS	NEG	POS	NEG	NEG	POS	NEG	NEG	NEG	NEG	NEG	NEG	NEG	NEG	NEG	NEG
Perth_2016-S20614	POS	POS	POS	NEG	POS	NEG	NEG	POS	NEG	NEG	NEG	NEG	NEG	NEG	NEG	NEG	NEG	NEG
Perth_2016-S23614	POS	POS	POS	NEG	POS	NEG	NEG	POS	NEG	NEG	NEG	NEG	NEG	NEG	NEG	NEG	NEG	NEG
Perth_2016-S42009	POS	POS	POS	NEG	POS	NEG	NEG	POS	NEG	NEG	NEG	NEG	NEG	NEG	NEG	NEG	NEG	NEG
Perth_2016-S51001	POS	POS	POS	NEG	POS	NEG	NEG	POS	NEG	NEG	NEG	NEG	NEG	NEG	NEG	NEG	NEG	NEG
Perth_2016-S51403	POS	POS	POS	NEG	POS	NEG	NEG	POS	NEG	NEG	NEG	NEG	NEG	NEG	NEG	NEG	NEG	NEG
Perth_07 RPA 20	POS	POS	POS	NEG	POS	NEG	NEG	POS	NEG	NEG	NEG	NEG	NEG	NEG	NEG	NEG	NEG	NEG
Perth_08-19088	POS	POS	POS	NEG	POS	NEG	NEG	POS	NEG	NEG	NEG	NEG	NEG	NEG	NEG	NEG	NEG	NEG
Perth_01-15351	POS	POS	POS	NEG	POS	NEG	NEG	POS	NEG	NEG	NEG	NEG	NEG	NEG	NEG	NEG	NEG	NEG
Perth_01-15357	POS	POS	POS	NEG	POS	NEG	NEG	POS	NEG	NEG	NEG	NEG	NEG	NEG	NEG	NEG	NEG	NEG
Perth_01-15419	POS	POS	POS	NEG	POS	NEG	NEG	POS	NEG	NEG	NEG	NEG	NEG	NEG	NEG	NEG	NEG	NEG
Perth_01-16309	POS	POS	POS	NEG	POS	NEG	NEG	POS	NEG	NEG	NEG	NEG	NEG	NEG	NEG	NEG	NEG	NEG
Perth_03 SNP 95	POS	POS	POS	NEG	POS	NEG	NEG	POS	NEG	NEG	NEG	NEG	NEG	NEG	NEG	NEG	NEG	NEG
Perth_2003_AH_47	POS	POS	POS	NEG	POS	NEG	NEG	POS	NEG	NEG	NEG	NEG	NEG	NEG	NEG	NEG	NEG	NEG
**>"Australian/NZ Clade":**	**POS**							**POS**			**NEG**							
Perth_2015-S20623	POS	POS	POS	NEG	POS	NEG	NEG	POS	NEG	NEG	NEG	NEG	NEG	NEG	NEG	NEG	NEG	NEG
Perth_2015-S20826	POS	POS	POS	NEG	POS	NEG	NEG	POS	NEG	NEG	NEG	NEG	NEG	NEG	NEG	NEG	NEG	NEG
Perth_05 NH 56	POS	POS	POS	NEG	POS	NEG	NEG	POS	NEG	NEG	NEG	NEG	NEG	NEG	NEG	NEG	NEG	NEG
**>CC5-MRSA-III LA MRSA**	**POS**							**POS**			**NEG**							
Delbrück 38 isolate from domestic turkey	POS	POS	POS	POS	POS	NEG	NEG	POS	NEG	NEG	NEG	NEG	NEG	NEG	NEG	NEG	NEG	NEG
**> Staph. pseudintermedi**	**NEG**							**NEG**			**NEG**							
KM1381	NEG	NEG	NEG	NEG	NEG	NEG	NEG	NEG	NEG	NEG	NEG	NEG	NEG	NEG	NEG	NEG	NEG	NEG
**>"South-East Asian Clad**	**COMM**							**POS**			**RARE**							
M92 (Canada)	POS	POS	POS	NEG	POS	NEG	NEG	POS	NEG	NEG	NEG	NEG	NEG	NEG	NEG	NEG	NEG	NEG
CHI59 (China)	POS	POS	POS	NEG	POS	NEG	NEG	POS	NEG	NEG	NEG	NEG	NEG	NEG	NEG	NEG	NEG	NEG
CHI61 (China)	POS	POS	POS	NEG	POS	NEG	NEG	POS	NEG	NEG	NEG	NEG	NEG	NEG	NEG	NEG	NEG	NEG
CUHK_HK1997 (China)	POS	POS	POS	NEG	POS	NEG	NEG	POS	NEG	NEG	NEG	NEG	NEG	NEG	NEG	NEG	NEG	NEG
M996 (China)	POS	POS	POS	NEG	POS	NEG	NEG	POS	NEG	NEG	NEG	NEG	NEG	NEG	NEG	NEG	NEG	NEG
H211 (Denmark)	POS	POS	POS	NEG	POS	NEG	NEG	POS	NEG	NEG	NEG	NEG	NEG	NEG	NEG	NEG	NEG	NEG
H216 (Denmark)	POS	POS	POS	NEG	POS	NEG	NEG	POS	NEG	NEG	NEG	NEG	NEG	NEG	NEG	NEG	NEG	NEG
D71 (Germany)	POS	POS	POS	NEG	POS	NEG	NEG	POS	NEG	NEG	NEG	NEG	NEG	NEG	NEG	NEG	NEG	NEG
NMR05 (India)	POS	POS	POS	NEG	POS	NEG	NEG	POS	NEG	NEG	NEG	NEG	NEG	NEG	NEG	NEG	NEG	NEG
MAL11 (Malaysia)	POS	POS	POS	NEG	POS	NEG	NEG	POS	NEG	NEG	NEG	NEG	NEG	NEG	NEG	NEG	NEG	NEG
MAL9 (Malaysia)	POS	POS	POS	NEG	POS	NEG	NEG	POS	NEG	NEG	NEG	NEG	NEG	NEG	NEG	NEG	NEG	NEG
Na21 (Sri Lanka)	POS	POS	POS	NEG	POS	NEG	NEG	POS	NEG	NEG	NEG	NEG	NEG	NEG	NEG	NEG	NEG	NEG
M592 (Syria)	POS	POS	POS	NEG	POS	NEG	NEG	POS	NEG	NEG	NEG	NEG	NEG	NEG	NEG	NEG	NEG	NEG
H202 (Thailand)	POS	POS	POS	NEG	POS	NEG	NEG	POS	NEG	NEG	NEG	NEG	NEG	NEG	NEG	NEG	NEG	NEG
S102 (Thailand)	POS	POS	POS	NEG	POS	NEG	NEG	POS	NEG	NEG	NEG	NEG	NEG	NEG	NEG	NEG	NEG	NEG
S130 (Thailand)	POS	POS	POS	NEG	POS	NEG	NEG	POS	NEG	NEG	NEG	NEG	NEG	NEG	NEG	NEG	NEG	NEG
S40 (Thailand)	POS	POS	POS	NEG	POS	NEG	NEG	POS	NEG	NEG	NEG	NEG	NEG	NEG	NEG	NEG	NEG	NEG
S71 (Thailand)	POS	POS	POS	NEG	POS	NEG	NEG	POS	NEG	NEG	NEG	NEG	NEG	NEG	NEG	NEG	NEG	NEG
S87 (Thailand)	POS	POS	POS	NEG	POS	NEG	NEG	POS	NEG	NEG	NEG	NEG	NEG	NEG	NEG	NEG	NEG	NEG
S93 (Thailand)	POS	POS	POS	NEG	POS	NEG	NEG	POS	NEG	NEG	NEG	NEG	NEG	NEG	NEG	NEG	NEG	NEG
US_002 (Thailand)	POS	POS	POS	NEG	POS	NEG	NEG	POS	NEG	NEG	NEG	NEG	NEG	NEG	NEG	NEG	NEG	NEG
US_017 (Thailand)	POS	POS	POS	NEG	POS	NEG	NEG	POS	NEG	NEG	NEG	NEG	NEG	NEG	NEG	NEG	NEG	NEG
WAMC6102 (Thailand)	POS	POS	POS	NEG	POS	NEG	NEG	POS	NEG	NEG	NEG	NEG	NEG	NEG	NEG	NEG	NEG	NEG
NCTC_13626_TW20 (UK)	POS	POS	POS	NEG	POS	NEG	NEG	POS	NEG	NEG	NEG	NEG	NEG	NEG	NEG	NEG	NEG	NEG
BSAC3021 (UK)	POS	POS	POS	NEG	POS	NEG	NEG	POS	NEG	NEG	NEG	NEG	NEG	NEG	NEG	NEG	NEG	NEG
BSAC3046 (UK)	POS	POS	POS	NEG	POS	NEG	NEG	POS	NEG	NEG	POS	NEG	NEG	NEG	NEG	NEG	NEG	NEG
1193_SAUR (USA)	POS	POS	POS	NEG	POS	NEG	NEG	POS	NEG	NEG	NEG	NEG	NEG	NEG	NEG	NEG	NEG	NEG
1194_SAUR (USA)	POS	POS	POS	NEG	POS	NEG	NEG	POS	NEG	NEG	NEG	NEG	NEG	NEG	NEG	NEG	NEG	NEG

STRAIN / ISOLATE	RESISTANCE : PENICILLINASE							RESISTANCE : MLS-ANTIBIOTICS										
	blaZ	blaI	blaR	hp_blaR-811	hp_blaR-812	blaZ (Slent)	blaZ (Ssap)	ermA	ermA-43	ermB	ermC		ermF	ermT	linA/lnuA	lnuB	lsa-B	lsa-E
	blaZ	blaI	blaR	hp_blaR-811	hp_blaR-812	blaZ (Slent)	blaZ (Ssap)	ermA	ermA-43	ermB	ermC	ermC-GM	ermF	ermT	linA/lnuA	lnuB	lsa-B	lsa-E
	beta-lactamase	beta lactamase repressor (inhibitor)	beta-lactamase regulatory protein					rRNA adenine N-6-methyl-transferase, erythromycin/clindamycin resistance		erythro-mycin/clindamycin resistance	0,00	rRNA adenine methylase-like protein ErmGM	rRNA adenine N-6-methyltransferase	0,00	Lincosamid-Nucleotidyltransferase	lincosamide nucleotidyltransferase	lincosamide ABC transporter	lincosamide ABC transporter
>"South-East Asian Clade": TW20 (Irish AR44), cc																		
1195_SAUR (USA)	POS	POS	POS	NEG	POS	NEG	NEG	POS	NEG	NEG	NEG	NEG	NEG	NEG	NEG	NEG	NEG	NEG
FVRH6002 (USA)	POS	POS	POS	NEG	POS	NEG	NEG	POS	NEG	NEG	NEG	NEG	NEG	NEG	NEG	NEG	NEG	NEG
KINW6048 (USA)	POS	POS	POS	NEG	POS	NEG	NEG	POS	NEG	NEG	NEG	NEG	NEG	NEG	NEG	NEG	NEG	NEG
LAMC0011 (USA)	NEG	NEG	NEG	NEG	NEG	NEG	NEG	POS	NEG	NEG	NEG	NEG	NEG	NEG	NEG	NEG	NEG	NEG
SJOS6053 (USA)	NEG	POS	NEG	NEG	NEG	NEG	NEG	POS	NEG	NEG	NEG	NEG	NEG	NEG	NEG	NEG	NEG	NEG
SJOS6072 (USA)	POS	POS	POS	NEG	POS	NEG	NEG	POS	NEG	NEG	NEG	NEG	NEG	NEG	NEG	NEG	NEG	NEG
UCIM6042 (USA)	NEG	POS	NEG	NEG	NEG	NEG	NEG	POS	NEG	NEG	NEG	NEG	NEG	NEG	NEG	NEG	NEG	NEG
Perth_2015-S31902	NEG	POS	NEG	NEG	NEG	NEG	NEG	POS	NEG	NEG	NEG	NEG	NEG	NEG	NEG	NEG	NEG	NEG
Perth_2015-S31904	POS	POS	POS	NEG	POS	NEG	NEG	POS	NEG	NEG	NEG	NEG	NEG	NEG	NEG	NEG	NEG	NEG
Perth_2015-S31913	POS	POS	AMB	NEG	POS	NEG	NEG	POS	NEG	NEG	NEG	NEG	NEG	NEG	NEG	NEG	NEG	NEG
Perth_2015-S31918	AMB	NEG	NEG	NEG	NEG	NEG	NEG	POS	NEG	NEG	NEG	NEG	NEG	NEG	NEG	NEG	NEG	NEG
Dublin-DSH_AR44_E1183	POS	POS	POS	AMB	POS	NEG	NEG	POS	NEG	NEG	NEG	NEG	NEG	NEG	NEG	NEG	NEG	NEG
Dublin-DSH_Unfamiliar-3_E1520	POS	POS	POS	AMB	POS	NEG	NEG	POS	NEG	NEG	NEG	NEG	NEG	NEG	NEG	NEG	NEG	NEG
Hong Kong_042	POS	POS	POS	NEG	POS	NEG	NEG	POS	NEG	NEG	NEG	NEG	NEG	NEG	NEG	NEG	NEG	NEG
Hong Kong_101	POS	POS	POS	NEG	POS	NEG	NEG	POS	NEG	NEG	NEG	NEG	NEG	NEG	NEG	NEG	NEG	NEG
Hong Kong_111	POS	POS	POS	NEG	POS	NEG	NEG	POS	NEG	NEG	NEG	NEG	NEG	NEG	NEG	NEG	NEG	NEG
Hong Kong_113	POS	POS	POS	NEG	POS	NEG	NEG	POS	NEG	NEG	NEG	NEG	NEG	NEG	NEG	NEG	NEG	NEG
Hong Kong_115	POS	POS	POS	NEG	POS	NEG	NEG	POS	NEG	NEG	NEG	NEG	NEG	NEG	NEG	NEG	NEG	NEG
Hong Kong_118	POS	POS	POS	NEG	POS	NEG	NEG	POS	NEG	NEG	NEG	NEG	NEG	NEG	NEG	NEG	NEG	NEG
Hong Kong_125	POS	POS	POS	NEG	POS	NEG	NEG	POS	NEG	NEG	NEG	NEG	NEG	NEG	NEG	NEG	NEG	NEG
Hong Kong_129	POS	POS	POS	NEG	POS	NEG	NEG	POS	NEG	NEG	NEG	NEG	NEG	NEG	NEG	NEG	NEG	NEG
Hong Kong_133	POS	POS	POS	NEG	POS	NEG	NEG	POS	NEG	NEG	NEG	NEG	NEG	NEG	NEG	NEG	NEG	NEG
Kuwait_009	POS	POS	POS	NEG	POS	NEG	NEG	POS	NEG	NEG	NEG	NEG	NEG	NEG	NEG	NEG	NEG	NEG
Kuwait_103	POS	POS	POS	NEG	POS	NEG	NEG	POS	NEG	NEG	NEG	NEG	NEG	NEG	NEG	NEG	NEG	NEG
Kuwait_107	POS	POS	POS	NEG	POS	NEG	NEG	POS	NEG	NEG	NEG	NEG	NEG	NEG	NEG	NEG	NEG	NEG
Perth_03 AH 88	POS	POS	POS	NEG	POS	NEG	NEG	POS	NEG	NEG	NEG	NEG	NEG	NEG	NEG	NEG	NEG	NEG
Perth_08-16905	POS	POS	POS	NEG	POS	NEG	NEG	POS	NEG	NEG	NEG	NEG	NEG	NEG	NEG	NEG	NEG	NEG
Riyadh_Alfaisal-10_SICU_13_B5672_1117353	POS	POS	POS	NEG	POS	NEG	NEG	POS	NEG	NEG	NEG	NEG	NEG	NEG	NEG	NEG	NEG	NEG
Riyadh_SSTI_17_3508602	POS	POS	POS	NEG	POS	NEG	NEG	POS	NEG	NEG	NEG	NEG	NEG	NEG	NEG	NEG	NEG	NEG
Riyadh-2822088-R	POS	POS	POS	NEG	POS	NEG	NEG	POS	NEG	NEG	NEG	NEG	NEG	NEG	NEG	NEG	NEG	NEG
Trinidad&Tobago_2012_195 (619/12)	POS	POS	POS	NEG	POS	NEG	NEG	POS	NEG	NEG	NEG	NEG	NEG	NEG	NEG	NEG	NEG	NEG
Trinidad&Tobago_2012_370 (1112-52177)	POS	POS	POS	NEG	POS	NEG	NEG	POS	NEG	NEG	NEG	NEG	NEG	NEG	NEG	NEG	NEG	NEG
Trinidad&Tobago_2012_378 (4139)	POS	POS	POS	POS	POS	NEG	NEG	POS	NEG	NEG	NEG	NEG	NEG	NEG	POS	NEG	NEG	NEG
Trinidad&Tobago_2013_PA21	POS	POS	POS	POS	POS	NEG	NEG	POS	NEG	NEG	NEG	NEG	NEG	NEG	NEG	AMB	NEG	NEG
Trinidad&Tobago_2013_PA22	POS	POS	POS	NEG	POS	NEG	NEG	POS	NEG	NEG	NEG	NEG	NEG	NEG	NEG	NEG	NEG	NEG
Trinidad&Tobago_2912_340 (8523/12)	POS	POS	POS	NEG	POS	NEG	NEG	POS	NEG	NEG	NEG	NEG	NEG	NEG	NEG	NEG	NEG	NEG
Trinidad&Tobago_MRSA2010_105	POS	POS	POS	POS	POS	NEG	NEG	POS	NEG	NEG	NEG	NEG	NEG	NEG	NEG	NEG	NEG	NEG
Trinidad&Tobago_MRSA2010_108	POS	POS	POS	AMB	POS	NEG	NEG	POS	NEG	NEG	NEG	NEG	NEG	NEG	NEG	NEG	NEG	NEG
Trinidad&Tobago_MRSA2010_33	POS	POS	POS	NEG	POS	NEG	NEG	POS	NEG	NEG	NEG	NEG	NEG	NEG	NEG	NEG	NEG	NEG
Trinidad&Tobago_MRSA2010_79	POS	POS	POS	POS	POS	NEG	NEG	POS	NEG	NEG	NEG	NEG	NEG	NEG	NEG	NEG	NEG	NEG
Trinidad&Tobago_MRSA2010_88	POS	POS	POS	NEG	POS	NEG	NEG	POS	NEG	NEG	NEG	NEG	NEG	NEG	NEG	NEG	NEG	NEG
Trinidad&Tobago_MRSA2010_97	POS	POS	POS	AMB	POS	NEG	NEG	POS	NEG	NEG	NEG	NEG	NEG	NEG	NEG	NEG	NEG	NEG
Trinidad&Tobago_SSTI 2012_031	POS	POS	POS	POS	POS	NEG	NEG	POS	NEG	NEG	NEG	NEG	NEG	NEG	NEG	NEG	NEG	NEG
Uganda-29_17148_250811	POS	POS	POS	NEG	POS	NEG	NEG	POS	NEG	NEG	NEG	NEG	NEG	NEG	NEG	NEG	NEG	NEG
>"South-East Asian Clad	POS							POS			NEG							
UCIM6015	POS	POS	POS	NEG	POS	NEG	NEG	POS	NEG	NEG	NEG	NEG	NEG	NEG	NEG	NEG	NEG	NEG
Hong Kong_121	POS	POS	POS	NEG	POS	NEG	NEG	POS	NEG	NEG	NEG	NEG	NEG	NEG	NEG	NEG	NEG	NEG
>"South-East Asian Clad	POS							POS			NEG							
Z172 (Taiwan)	POS	POS	POS	NEG	POS	NEG	NEG	POS	NEG	NEG	NEG	NEG	NEG	NEG	NEG	NEG	NEG	NEG
Russia-19_0342_Kurgan	POS	POS	POS	NEG	AMB	NEG	NEG	POS	NEG	NEG	NEG	NEG	NEG	NEG	NEG	NEG	NEG	NEG
>"South-East Asian Clad	POS							POS			NEG							
Perth_01-15392	POS	POS	POS	NEG	POS	NEG	NEG	POS	NEG	NEG	NEG	NEG	NEG	NEG	NEG	NEG	NEG	NEG
>"South-East Asian Clad	POS							POS			NEG							
XN108 (China)	POS	POS	POS	NEG	POS	NEG	NEG	POS	NEG	NEG	NEG	NEG	NEG	NEG	NEG	NEG	NEG	NEG
NMR09 (India)	POS	POS	POS	NEG	POS	NEG	NEG	POS	NEG	NEG	NEG	NEG	NEG	NEG	NEG	NEG	NEG	NEG
346 (Malaysia)	POS	POS	AMB	NEG	POS	NEG	NEG	POS	NEG	NEG	NEG	NEG	NEG	NEG	NEG	NEG	NEG	NEG
DS_009 (Thailand)	POS	POS	POS	NEG	POS	NEG	NEG	POS	NEG	NEG	NEG	NEG	NEG	NEG	NEG	NEG	NEG	NEG
18ANR581005 (Ind)	POS	POS	POS	NEG	POS	NEG	NEG	POS	NEG	NEG	NEG	NEG	NEG	NEG	NEG	NEG	#ZAHL!	NEG
Bengalaru_ST20141405	POS	POS	POS	NEG	POS	NEG	NEG	POS	NEG	NEG	NEG	NEG	NEG	NEG	NEG	NEG	NEG	NEG
Bengalaru_ST20141406	POS	POS	POS	NEG	POS	NEG	NEG	POS	NEG	NEG	NEG	NEG	NEG	NEG	NEG	NEG	NEG	NEG
Bengalaru_ST20141417	POS	POS	POS	NEG	POS	NEG	NEG	POS	NEG	NEG	NEG	NEG	NEG	NEG	NEG	NEG	NEG	NEG
Hong Kong_107	POS	POS	POS	NEG	POS	NEG	NEG	POS	NEG	NEG	NEG	NEG	NEG	NEG	NEG	NEG	NEG	NEG
Hong Kong_12	POS	POS	POS	NEG	POS	NEG	NEG	POS	NEG	NEG	NEG	NEG	NEG	NEG	NEG	NEG	NEG	NEG
Hong Kong_132	POS	POS	POS	AMB	POS	NEG	NEG	POS	NEG	NEG	NEG	NEG	NEG	NEG	NEG	NEG	NEG	NEG
Riyadh-3028763-R	POS	POS	POS	NEG	POS	NEG	NEG	POS	NEG	NEG	NEG	NEG	NEG	NEG	NEG	NEG	NEG	NEG
Trinidad&Tobago_MRSA2010_94	POS	POS	POS	NEG	POS	NEG	NEG	POS	NEG	NEG	NEG	NEG	NEG	NEG	NEG	NEG	NEG	NEG
>"South-East Asian Clad	POS							POS			VAR							
M705 (Thailand)	POS	POS	POS	NEG	POS	NEG	NEG	POS	NEG	NEG	POS	NEG	NEG	NEG	NEG	NEG	NEG	NEG
S85 (Thailand)	POS	POS	POS	NEG	POS	NEG	NEG	POS	NEG	NEG	NEG	NEG	NEG	NEG	NEG	NEG	NEG	NEG
Riyadh_Alfaisal-04_281014_S5076	POS	POS	POS	NEG	POS	NEG	NEG	POS	NEG	NEG	NEG	NEG	NEG	NEG	NEG	NEG	NEG	NEG
Riyadh_Alfaisal-26_S15145_1104163	POS	POS	POS	NEG	POS	NEG	NEG	POS	NEG	NEG	NEG	NEG	NEG	NEG	NEG	NEG	NEG	NEG
Riyadh-2817437-W	POS	POS	POS	NEG	POS	NEG	NEG	POS	NEG	NEG	NEG	NEG	NEG	NEG	NEG	NEG	NEG	NEG
>"South-East Asian Clad	POS							POS			NEG							
PPUKM-775-2009 (Malaysia)	POS	POS	POS	NEG	POS	NEG	NEG	POS	NEG	NEG	NEG	NEG	NEG	NEG	NEG	NEG	NEG	NEG
Hong Kong_138	POS	POS	POS	NEG	POS	NEG	NEG	POS	NEG	NEG	NEG	NEG	NEG	NEG	NEG	NEG	NEG	NEG
>"South-East Asian Clad	POS							POS			NEG							
Kuwait_001	POS	POS	POS	NEG	POS	NEG	NEG	POS	NEG	NEG	NEG	NEG	NEG	NEG	NEG	NEG	NEG	NEG
Kuwait_337	POS	POS	POS	NEG	POS	NEG	NEG	POS	NEG	NEG	NEG	NEG	NEG	NEG	NEG	NEG	NEG	NEG
>"South-East Asian Clad	POS							POS			RARE							
CUHK_HK2007 (China)	POS	POS	POS	NEG	POS	NEG	NEG	POS	NEG	NEG	NEG	NEG	NEG	NEG	NEG	NEG	NEG	NEG
NMR02 (India)	POS	POS	POS	NEG	POS	NEG	NEG	POS	NEG	NEG	NEG	NEG	NEG	NEG	NEG	NEG	NEG	NEG
VB1490 (India)	POS	POS	POS	NEG	POS	NEG	NEG	POS	NEG	NEG	NEG	NEG	NEG	NEG	NEG	NEG	NEG	NEG
V521 (Korea)	POS	POS	POS	NEG	POS	NEG	NEG	POS	NEG	NEG	NEG	NEG	NEG	NEG	NEG	NEG	NEG	NEG
HST-077 (Lebanon)	POS	POS	POS	NEG	POS	NEG	NEG	POS	NEG	NEG	NEG	NEG	NEG	NEG	NEG	NEG	NEG	NEG
MAL1 (Malaysia)	POS	POS	POS	NEG	POS	NEG	NEG	POS	NEG	NEG	NEG	NEG	NEG	NEG	NEG	NEG	NEG	NEG
MAL3 (Malaysia)	POS	POS	POS	NEG	POS	NEG	NEG	POS	NEG	NEG	NEG	NEG	NEG	NEG	NEG	NEG	NEG	NEG
PPUKM-261-2009 (Malaysia)	POS	POS	POS	NEG	POS	NEG	NEG	POS	NEG	NEG	NEG	NEG	NEG	NEG	NEG	NEG	NEG	NEG
PPUKM-332-2009 (Malaysia)	POS	POS	POS	NEG	POS	NEG	NEG	POS	NEG	NEG	NEG	NEG	NEG	NEG	NEG	NEG	NEG	NEG
S38 (Thailand)	POS	POS	POS	NEG	POS	NEG	NEG	POS	NEG	NEG	NEG	NEG	NEG	NEG	NEG	NEG	NEG	NEG
S7 (Thailand)	POS	POS	POS	NEG	POS	NEG	NEG	POS	NEG	NEG	NEG	NEG	NEG	NEG	NEG	NEG	NEG	NEG
US_008 (Thailand)	POS	POS	POS	NEG	POS	NEG	NEG	POS	NEG	NEG	NEG	NEG	NEG	NEG	NEG	NEG	NEG	NEG
US_030 (Thailand)	POS	POS	POS	NEG	POS	NEG	NEG	POS	NEG	NEG	NEG	NEG	NEG	NEG	NEG	NEG	NEG	NEG
M170 (Tsunami-related)	POS	POS	POS	NEG	POS	NEG	NEG	POS	NEG	NEG	NEG	NEG	NEG	NEG	NEG	NEG	NEG	NEG
BSAC697 (UK)	POS	POS	POS	NEG	POS	NEG	NEG	POS	NEG	NEG	POS	NEG	NEG	NEG	NEG	NEG	NEG	NEG
IS-189	POS	POS	POS	NEG	POS	NEG	NEG	POS	NEG	NEG	NEG	NEG	NEG	NEG	NEG	NEG	NEG	NEG
MRGR3	POS	POS	POS	NEG	POS	NEG	NEG	POS	NEG	NEG	AMB	NEG	NEG	NEG	NEG	NEG	NEG	NEG
smg07	POS	POS	POS	NEG	POS	NEG	NEG	POS	NEG	NEG	NEG	NEG	NEG	NEG	NEG	NEG	NEG	NEG
AH1-AUS-EMRSA-3	POS	POS	POS	NEG	POS	NEG	NEG	POS	NEG	NEG	NEG	NEG	NEG	NEG	NEG	NEG	NEG	NEG
Bengalaru_ST20141403	POS	POS	POS	NEG	POS	NEG	NEG	POS	NEG	NEG	NEG	NEG	NEG	NEG	NEG	NEG	NEG	NEG
Finland E24_98541_Harmony	POS	POS	POS	NEG	POS	NEG	NEG	POS	NEG	NEG	NEG	NEG	NEG	NEG	NEG	NEG	NEG	NEG
Hong Kong_10	POS	POS	POS	AMB	POS	NEG	NEG	POS	NEG	NEG	NEG	NEG	NEG	NEG	NEG	NEG	NEG	NEG
Hong Kong_100	POS	POS	POS	NEG	POS	NEG	NEG	POS	NEG	NEG	NEG	NEG	NEG	NEG	NEG	NEG	NEG	NEG
Hong Kong_199	POS	POS	POS	NEG	POS	NEG	NEG	POS	NEG	NEG	NEG	NEG	NEG	NEG	NEG	NEG	NEG	NEG
Hong Kong_34	POS	POS	POS	NEG	POS	NEG	NEG	POS	NEG	NEG	NEG	NEG	NEG	NEG	NEG	NEG	NEG	NEG
Hong Kong_88	POS	POS	POS	POS	POS	NEG	NEG	POS	NEG	NEG	NEG	NEG	NEG	NEG	NEG	NEG	NEG	NEG
Hong Kong_92	POS	POS	POS	NEG	POS	NEG	NEG	POS	NEG	NEG	NEG	NEG	NEG	NEG	NEG	NEG	NEG	NEG
Hong Kong_94	POS	POS	POS	AMB	POS	NEG	NEG	POS	NEG	NEG	NEG	NEG	NEG	NEG	NEG	NEG	NEG	NEG
Kuwait_257	POS	POS	POS	POS	POS	NEG	NEG	POS	NEG	NEG	NEG	NEG	NEG	NEG	NEG	NEG	NEG	NEG
Perth_01-15330	POS	POS	POS	NEG	POS	NEG	NEG	POS	NEG	NEG	NEG	NEG	NEG	NEG	NEG	NEG	NEG	NEG
Perth_01-15349	POS	POS	POS	NEG	POS	NEG	NEG	POS	NEG	NEG	NEG	NEG	NEG	NEG	NEG	NEG	NEG	NEG
Perth_01-16176	POS	POS	POS	NEG	POS	NEG	NEG	POS	NEG	NEG	NEG	NEG	NEG	NEG	NEG	NEG	NEG	NEG
Perth_WBG8379	POS	POS	POS	NEG	POS	NEG	NEG	POS	NEG	NEG	NEG	NEG	NEG	NEG	NEG	NEG	NEG	NEG
Rawalpindi_Kidney Center_56	POS	POS	POS	POS	POS	NEG	NEG	POS	NEG	NEG	NEG	NEG	NEG	NEG	NEG	NEG	NEG	NEG
Riyadh_SSTI_05_3464614	POS	POS	POS	NEG	POS	NEG	NEG	POS	NEG	NEG	NEG	NEG	NEG	NEG	NEG	NEG	NEG	NEG
Trinidad&Tobago_2012_157	POS	POS	POS	POS	POS	NEG	NEG	POS	NEG	NEG	NEG	NEG	NEG	NEG	NEG	NEG	NEG	NEG

STRAIN / ISOLATE	RESISTANCE : PENICILLINASE							RESISTANCE : MLS-ANTIBIOTICS										
	blaZ	blaI	blaR	hp_blaR-811	hp_blaR-812	blaZ (Slent)	blaZ (Ssap)	ermA	ermA-43	ermB	ermC		ermF	ermT	linA/lnuA	lnuB	lsa-B	lsa-E
	blaZ	blaI	blaR	hp_blaR-811	hp_blaR-812	blaZ (Slent)	blaZ (Ssap)	ermA	ermA-43	ermB	ermC	ermC-GM	ermF	ermT	linA/lnuA	lnuB	lsa-B	lsa-E
	beta-lactamase	beta lactamase repressor (inhibitor)	beta-lactamase regulatory protein					rRNA adenine N-6-methyl-transferase, erythromycin/clindamycin resistance		erythro-mycin/clindamycin resistance	0,00	rRNA adenine methylase-like protein ErmGM	rRNA adenine N-6-methyltransferase	0,00	Lincosamid-Nucleotidyltransferase	lincosamide nucleotidyltransferase	lincosamide ABC transporter	lincosamide ABC transporter
>"South-East Asian Clad	POS							POS			NEG							
M418	POS	POS	POS	NEG	POS	NEG	NEG	POS	NEG	NEG	NEG	NEG	NEG	NEG	NEG	NEG	NEG	NEG
>"South-East Asian Clad	POS							POS			NEG							
NMR07 (India)	POS	POS	POS	NEG	POS	NEG	NEG	POS	NEG	NEG	NEG	NEG	NEG	NEG	NEG	NEG	NEG	NEG
NMR08 (India)	POS	POS	POS	NEG	POS	NEG	NEG	POS	NEG	NEG	NEG	NEG	NEG	NEG	NEG	NEG	NEG	NEG
Bengalaru_ST20121978	POS	POS	POS	NEG	POS	NEG	NEG	POS	NEG	NEG	NEG	NEG	NEG	NEG	NEG	NEG	NEG	NEG
Hong Kong_89	POS	POS	POS	NEG	POS	NEG	NEG	POS	NEG	NEG	NEG	NEG	NEG	NEG	NEG	NEG	NEG	NEG
>"South-East Asian Clad	POS							POS			NEG							
Perth_2005_AH_5	POS	POS	POS	NEG	POS	NEG	NEG	POS	NEG	NEG	NEG	NEG	NEG	NEG	NEG	NEG	NEG	NEG
>"South-East Asian Clad	POS							POS			NEG							
Trinidad&Tobago_MRSA2010_103	POS	POS	POS	NEG	POS	NEG	NEG	POS	NEG	NEG	NEG	NEG	NEG	NEG	NEG	NEG	NEG	NEG
Trinidad&Tobago_2013_PA25	POS	POS	POS	NEG	POS	NEG	NEG	POS	NEG	NEG	NEG	NEG	NEG	NEG	NEG	NEG	NEG	NEG
Trinidad&Tobago_MRSA2010_110	POS	POS	POS	NEG	POS	NEG	NEG	POS	NEG	NEG	NEG	NEG	NEG	NEG	NEG	NEG	NEG	NEG
Trinidad&Tobago_SSTI 2012_111	POS	POS	POS	NEG	POS	NEG	NEG	POS	NEG	NEG	NEG	NEG	NEG	NEG	NEG	NEG	NEG	NEG
Trinidad&Tobago_MRSA2010_158	POS	POS	POS	POS	POS	NEG	NEG	POS	NEG	NEG	NEG	NEG	NEG	NEG	NEG	NEG	NEG	NEG
>"South-East Asian Clad	POS							POS			VAR							
Kuwait_122	POS	POS	POS	NEG	POS	NEG	NEG	POS	NEG	NEG	NEG	NEG	NEG	NEG	NEG	NEG	NEG	NEG
Kuwait_002	POS	POS	POS	NEG	POS	NEG	NEG	POS	NEG	NEG	NEG	NEG	NEG	NEG	NEG	NEG	NEG	NEG
Kuwait_196	POS	POS	POS	NEG	POS	NEG	NEG	POS	NEG	NEG	NEG	NEG	NEG	NEG	NEG	NEG	NEG	NEG
Kuwait_199	POS	POS	POS	NEG	POS	NEG	NEG	POS	NEG	NEG	POS	NEG	NEG	NEG	POS	NEG	NEG	NEG
Hong Kong_136	POS	POS	POS	NEG	POS	NEG	NEG	POS	NEG	NEG	NEG	NEG	NEG	NEG	NEG	NEG	NEG	NEG
>"South-East Asian Clad	POS							POS			NEG							
P32 (Poland)	POS	POS	POS	NEG	POS	NEG	NEG	POS	NEG	NEG	NEG	NEG	NEG	NEG	NEG	NEG	NEG	NEG
>"South-East Asian Clad	pos							POS			NEG							
Perth_2015-S32313	AMB	POS	AMB	NEG	POS	NEG	NEG	POS	NEG	NEG	NEG	NEG	NEG	0,00	NEG	NEG	NEG	NEG
>"hla-negative South-Ea	POS							POS			NEG							
DEN907 (Denmark)	POS	POS	POS	NEG	POS	NEG	NEG	POS	NEG	NEG	NEG	NEG	NEG	NEG	NEG	NEG	NEG	NEG
>"hla-negative South-Ea	POS							POS			NEG							
S26 (Thailand)	POS	POS	POS	NEG	POS	NEG	NEG	POS	NEG	NEG	NEG	NEG	NEG	NEG	NEG	NEG	NEG	NEG
S42 (Thailand)	POS	POS	POS	NEG	POS	NEG	NEG	POS	NEG	NEG	NEG	NEG	NEG	NEG	NEG	NEG	NEG	NEG
S97 (Thailand)	POS	POS	POS	NEG	POS	NEG	NEG	POS	NEG	NEG	NEG	NEG	NEG	NEG	NEG	NEG	NEG	NEG
S78 (Thailand)	POS	POS	POS	NEG	POS	NEG	NEG	POS	NEG	NEG	NEG	NEG	NEG	NEG	NEG	NEG	NEG	NEG
S25 (Thailand)	POS	POS	POS	NEG	POS	NEG	NEG	POS	NEG	NEG	NEG	NEG	NEG	NEG	NEG	NEG	NEG	NEG
S21 (Thailand)	POS	POS	POS	NEG	POS	NEG	NEG	POS	NEG	NEG	NEG	NEG	NEG	NEG	NEG	NEG	NEG	NEG
S106 (Thailand)	POS	POS	POS	NEG	POS	NEG	NEG	POS	NEG	NEG	NEG	NEG	NEG	NEG	NEG	NEG	NEG	NEG
S24 (Thailand)	POS	POS	POS	NEG	POS	NEG	NEG	POS	NEG	NEG	NEG	NEG	NEG	NEG	NEG	NEG	NEG	NEG
S39 (Thailand)	POS	POS	POS	NEG	POS	NEG	NEG	POS	NEG	NEG	NEG	NEG	NEG	NEG	NEG	NEG	NEG	NEG
S2 (Thailand)	POS	POS	POS	NEG	POS	NEG	NEG	POS	NEG	NEG	NEG	NEG	NEG	NEG	NEG	NEG	NEG	NEG
S81 (Thailand)	POS	POS	POS	NEG	POS	NEG	NEG	POS	NEG	NEG	NEG	NEG	NEG	NEG	NEG	NEG	NEG	NEG
M116 (Vietnam)	POS	POS	POS	NEG	POS	NEG	NEG	POS	NEG	NEG	NEG	NEG	NEG	NEG	NEG	NEG	NEG	NEG
>"Portuguese Clade": Po	POS							POS			RARE							
FFP103 (Portugal)	POS	POS	POS	NEG	POS	NEG	NEG	POS	NEG	NEG	NEG	NEG	NEG	NEG	NEG	NEG	NEG	NEG
HDG2 (Portugal)	POS	POS	POS	NEG	POS	NEG	NEG	POS	NEG	NEG	NEG	NEG	NEG	NEG	NEG	NEG	NEG	NEG
HSA10 (Portugal)	POS	POS	POS	NEG	POS	NEG	NEG	POS	NEG	NEG	NEG	NEG	NEG	NEG	NEG	NEG	NEG	NEG
HSA11 (Portugal)	POS	POS	POS	NEG	POS	NEG	NEG	POS	NEG	NEG	NEG	NEG	NEG	NEG	NEG	NEG	NEG	NEG
ICP5011 (Portugal)	POS	POS	POS	NEG	POS	NEG	NEG	POS	NEG	NEG	POS	NEG	NEG	NEG	NEG	NEG	NEG	NEG
ICP5014 (Portugal)	POS	POS	POS	NEG	POS	NEG	NEG	POS	NEG	NEG	NEG	NEG	NEG	NEG	NEG	NEG	NEG	NEG
ICP5062 (Portugal)	POS	POS	POS	NEG	POS	NEG	NEG	POS	NEG	NEG	NEG	NEG	NEG	NEG	NEG	NEG	NEG	NEG
Strain 1063_ATCC 33592 FDAARGOS_3 (USA)	POS	POS	POS	NEG	POS	NEG	NEG	POS	NEG	NEG	NEG	NEG	NEG	NEG	NEG	NEG	NEG	NEG
	POS	POS	POS	NEG	POS	NEG	NEG	POS	NEG	NEG	POS	NEG	NEG	NEG	NEG	NEG	NEG	NEG
Russia-07_0110_Kurgan	POS	POS	POS	NEG	NEG	NEG	NEG	POS	NEG	NEG	NEG	NEG	NEG	NEG	NEG	NEG	NEG	NEG
Russia-11_0164_Kurgan	POS	POS	POS	NEG	POS	NEG	NEG	POS	NEG	NEG	NEG	NEG	NEG	NEG	NEG	NEG	NEG	NEG
Russia-20_0367_Kurgan	POS	POS	POS	NEG	POS	NEG	NEG	POS	NEG	NEG	NEG	NEG	NEG	NEG	NEG	NEG	NEG	NEG
Russia-21_0390_Chelyabinsk	POS	POS	POS	NEG	POS	NEG	NEG	POS	NEG	NEG	NEG	NEG	NEG	NEG	NEG	NEG	NEG	NEG
>"South American/ Midd	POS							POS			NEG							
AGT1 (Argentina)	POS	POS	POS	NEG	POS	NEG	NEG	POS	NEG	NEG	NEG	NEG	NEG	NEG	NEG	NEG	NEG	NEG
AGT120 (Argentina)	POS	POS	POS	NEG	POS	NEG	NEG	POS	NEG	NEG	NEG	NEG	NEG	NEG	NEG	NEG	NEG	NEG
AGT67 (Argentina)	POS	POS	POS	NEG	POS	NEG	NEG	POS	NEG	NEG	NEG	NEG	NEG	NEG	NEG	NEG	NEG	NEG
AGT9 (Argentina)	POS	POS	POS	NEG	POS	NEG	NEG	POS	NEG	NEG	NEG	NEG	NEG	NEG	NEG	NEG	NEG	NEG
RA3 (Argentina)	POS	POS	POS	NEG	POS	NEG	NEG	POS	NEG	NEG	NEG	NEG	NEG	NEG	NEG	NEG	NEG	NEG
RA6 (Argentina)	POS	POS	POS	NEG	POS	NEG	NEG	POS	NEG	NEG	NEG	NEG	NEG	NEG	NEG	NEG	NEG	NEG
RA7 (Argentina)	POS	POS	POS	NEG	POS	NEG	NEG	POS	NEG	NEG	NEG	NEG	NEG	NEG	NEG	NEG	NEG	NEG
Be62 (Brazil)	POS	POS	POS	NEG	POS	NEG	NEG	POS	NEG	NEG	NEG	NEG	NEG	NEG	NEG	NEG	NEG	NEG
Bmb9393 (Brazil)	POS	POS	POS	NEG	POS	NEG	NEG	POS	NEG	NEG	NEG	NEG	NEG	NEG	NEG	NEG	NEG	NEG
BRA36 (Brazil)	POS	POS	POS	NEG	POS	NEG	NEG	POS	NEG	NEG	NEG	NEG	NEG	NEG	NEG	NEG	NEG	NEG
BZ48 (Brazil)	POS	POS	POS	NEG	POS	NEG	NEG	POS	NEG	NEG	NEG	NEG	NEG	NEG	NEG	NEG	NEG	NEG
Gv51 (Brazil)	POS	POS	POS	NEG	POS	NEG	NEG	POS	NEG	NEG	NEG	NEG	NEG	NEG	NEG	NEG	NEG	NEG
Gv69 (Brazil)	POS	POS	POS	NEG	POS	NEG	NEG	POS	NEG	NEG	NEG	NEG	NEG	NEG	NEG	NEG	NEG	NEG
Gv88 (Brazil)	POS	POS	POS	NEG	POS	NEG	NEG	POS	NEG	NEG	NEG	NEG	NEG	NEG	NEG	NEG	NEG	NEG
HC1335 (Brazil)	POS	POS	POS	NEG	POS	NEG	NEG	POS	NEG	NEG	NEG	NEG	NEG	NEG	NEG	NEG	NEG	NEG
HC1340 (Brazil)	POS	POS	POS	NEG	POS	NEG	NEG	POS	NEG	NEG	NEG	NEG	NEG	NEG	NEG	NEG	NEG	NEG
HC556 (Brazil)	POS	POS	POS	NEG	POS	NEG	NEG	POS	NEG	NEG	NEG	NEG	NEG	NEG	NEG	NEG	NEG	NEG
HU25 (Brazil)	POS	POS	POS	NEG	POS	NEG	NEG	POS	NEG	NEG	NEG	NEG	NEG	NEG	NEG	NEG	NEG	NEG
UB563 (Brazil)	POS	POS	POS	NEG	POS	NEG	NEG	POS	NEG	NEG	NEG	NEG	NEG	NEG	NEG	NEG	NEG	NEG
2A8 (Czech Republic)	POS	POS	POS	NEG	POS	NEG	NEG	POS	NEG	NEG	NEG	NEG	NEG	NEG	NEG	NEG	NEG	NEG
M1229 (Denmark)	POS	POS	POS	NEG	POS	NEG	NEG	POS	NEG	NEG	NEG	NEG	NEG	NEG	NEG	NEG	NEG	NEG
FRICAR (France)	POS	POS	POS	NEG	POS	NEG	NEG	POS	NEG	NEG	NEG	NEG	NEG	NEG	NEG	NEG	NEG	NEG
D90 (Germany)	POS	POS	POS	NEG	POS	NEG	NEG	POS	NEG	NEG	NEG	NEG	NEG	NEG	NEG	NEG	NEG	NEG
LIT68 (Lithuania)	POS	POS	POS	NEG	POS	NEG	NEG	POS	NEG	NEG	NEG	NEG	NEG	NEG	NEG	NEG	NEG	NEG
LIT76 (Lithuania)	POS	POS	POS	NEG	POS	NEG	NEG	POS	NEG	NEG	NEG	NEG	NEG	NEG	NEG	NEG	NEG	NEG
HGSA142 (Portugal)	POS	POS	POS	NEG	POS	NEG	NEG	POS	NEG	NEG	NEG	NEG	NEG	NEG	NEG	NEG	NEG	NEG
HGSA9 (Portugal)	POS	POS	POS	NEG	POS	NEG	NEG	POS	NEG	NEG	NEG	NEG	NEG	NEG	NEG	NEG	NEG	NEG
HSJ216 (Portugal)	POS	POS	POS	NEG	POS	NEG	NEG	POS	NEG	NEG	NEG	NEG	NEG	NEG	NEG	NEG	NEG	NEG
M278 (Portugal)	POS	POS	POS	NEG	POS	NEG	NEG	POS	NEG	NEG	NEG	NEG	NEG	NEG	NEG	NEG	NEG	NEG
ES26 (Spain)	POS	POS	POS	NEG	POS	NEG	NEG	POS	NEG	NEG	NEG	NEG	NEG	NEG	NEG	NEG	NEG	NEG
UK102 (UK)	POS	POS	POS	NEG	POS	NEG	NEG	POS	NEG	NEG	NEG	NEG	NEG	NEG	NEG	NEG	NEG	NEG
URU34 (Uruguay)	POS	POS	POS	NEG	POS	NEG	NEG	POS	NEG	POS	NEG	NEG	NEG	NEG	NEG	NEG	NEG	NEG
IS-125	POS	POS	POS	NEG	POS	NEG	NEG	POS	NEG	NEG	NEG	NEG	NEG	NEG	NEG	NEG	NEG	NEG
IS-157	POS	POS	POS	NEG	POS	NEG	NEG	POS	NEG	NEG	NEG	NEG	NEG	NEG	NEG	NEG	NEG	NEG
Ecuador_1	POS	POS	POS	NEG	POS	NEG	NEG	POS	NEG	NEG	NEG	NEG	NEG	NEG	NEG	NEG	NEG	NEG
Ecuador_2	POS	POS	POS	NEG	POS	NEG	NEG	POS	NEG	NEG	NEG	NEG	NEG	NEG	NEG	NEG	NEG	NEG
Dresden_15ANR574263	POS	POS	POS	POS	POS	NEG	NEG	POS	NEG	NEG	NEG	NEG	NEG	NEG	NEG	AMB	NEG	NEG
UK-EMRSA-11	POS	POS	POS	NEG	POS	NEG	NEG	POS	NEG	NEG	NEG	NEG	NEG	NEG	NEG	NEG	NEG	NEG
>"South American/ Midd	POS							POS			VAR							
BRA2 (Brazil)	POS	POS	POS	NEG	POS	NEG	NEG	POS	NEG	NEG	NEG	NEG	NEG	NEG	NEG	NEG	NEG	NEG
CHL1 (Chile)	POS	POS	POS	NEG	POS	NEG	NEG	POS	NEG	NEG	NEG	NEG	NEG	NEG	NEG	NEG	NEG	NEG
CHL151 (Chile)	POS	POS	POS	NEG	POS	NEG	NEG	POS	NEG	NEG	NEG	NEG	NEG	NEG	NEG	NEG	NEG	NEG
UCL378 (Chile)	POS	POS	POS	NEG	POS	NEG	NEG	POS	NEG	NEG	NEG	NEG	NEG	NEG	NEG	NEG	NEG	NEG
UP72 (Peru)	POS	POS	POS	NEG	POS	NEG	NEG	POS	NEG	NEG	POS	NEG	NEG	NEG	NEG	NEG	NEG	NEG
UP81 (Peru)	POS	POS	POS	NEG	POS	NEG	NEG	POS	NEG	NEG	POS	NEG	NEG	NEG	NEG	NEG	NEG	NEG
>"South American/ Midd	POS							POS			POS							
UP1073 (Peru)	POS	POS	POS	NEG	POS	NEG	NEG	POS	NEG	NEG	POS	NEG	NEG	NEG	NEG	NEG	NEG	NEG

STRAIN / ISOLATE	RESISTANCE : PENICILLINASE							RESISTANCE : MLS-ANTIBIOTICS										
	blaZ	blaI	blaR	hp_blaR-811	hp_blaR-812	blaZ (Slent)	blaZ (Ssap)	ermA	ermA-43	ermB	ermC		ermF	ermT	linA/lnuA	lnuB	lsa-B	lsa-E
	blaZ	blaI	blaR	hp_blaR-811	hp_blaR-812	blaZ (Slent)	blaZ (Ssap)	ermA	ermA-43	ermB	ermC	ermC-GM	ermF	ermT	linA/lnuA	lnuB	lsa-B	lsa-E
	beta-lactamase	beta lactamase repressor (inhibitor)	beta-lactamase regulatory protein					rRNA adenine N-6-methyl-transferase, erythromycin/clindamycin resistance		erythro-mycin/clindamycin resistance	0,00	rRNA adenine methylase-like protein ErmGM	rRNA adenine N-6-methyltransferase	0,00	Lincosamid-Nucleotidyltransferase	lincosamide nucleotidyltransferase	lincosamide ABC transporter	lincosamide ABC transporter
>"South American/ Midc	POS							POS			NEG							
LIT89 (Lithuania)	POS	POS	POS	NEG	POS	NEG	NEG	POS	NEG	NEG	NEG	NEG	NEG	NEG	NEG	NEG	NEG	NEG
MRSA_PR1 (Malaysia)	POS	POS	POS	NEG	POS	NEG	NEG	POS	NEG	NEG	NEG	NEG	NEG	NEG	NEG	NEG	NEG	NEG
Riyadh-2888905-R	POS	POS	POS	NEG	POS	NEG	NEG	POS	NEG	NEG	NEG	NEG	NEG	NEG	NEG	NEG	NEG	NEG
Riyadh-2888915-BC	POS	POS	POS	NEG	POS	NEG	NEG	POS	NEG	NEG	NEG	NEG	NEG	NEG	NEG	NEG	NEG	NEG
Riyadh-2793706-R	POS	POS	POS	NEG	POS	NEG	NEG	POS	NEG	NEG	NEG	NEG	NEG	NEG	NEG	NEG	NEG	NEG
>"South American/ Midc	POS							POS			NEG							
Riyadh_SSTI_18_3502925	POS	POS	POS	NEG	POS	NEG	NEG	POS	NEG	NEG	NEG	NEG	NEG	NEG	NEG	NEG	NEG	NEG
Riyadh-2822825-W	POS	POS	POS	NEG	POS	NEG	NEG	POS	NEG	NEG	NEG	NEG	NEG	NEG	NEG	NEG	NEG	NEG
>"South American/ Midc	POS							POS			NEG							
UK-EMRSA-9	POS	POS	POS	NEG	POS	NEG	NEG	POS	NEG	NEG	NEG	NEG	NEG	NEG	NEG	NEG	NEG	NEG
>"South American/ Midc	POS							POS			NEG							
Lome_HT20020815	POS	POS	POS	NEG	POS	NEG	NEG	POS	NEG	NEG	NEG	NEG	NEG	NEG	NEG	NEG	NEG	NEG
>"South American/ Midc	POS							COMM			NEG							
NA32 (Denmark)	POS	POS	POS	NEG	POS	NEG	NEG	POS	NEG	NEG	NEG	NEG	NEG	NEG	NEG	NEG	NEG	NEG
H24 (Egypt)	POS	POS	POS	NEG	POS	NEG	NEG	POS	NEG	NEG	NEG	NEG	NEG	NEG	NEG	NEG	NEG	NEG
ATCC BAA-39 (=HUSA304) (Hungary)	POS	POS	POS	NEG	POS	NEG	NEG	POS	NEG	NEG	NEG	NEG	NEG	NEG	NEG	NEG	NEG	NEG
HUSA304 (Hungary)	POS	POS	POS	NEG	POS	NEG	NEG	POS	NEG	NEG	NEG	NEG	NEG	NEG	NEG	NEG	NEG	NEG
HU106 (Hungary)	POS	POS	POS	NEG	POS	NEG	NEG	POS	NEG	NEG	NEG	NEG	NEG	NEG	NEG	NEG	NEG	NEG
BSAC27 (UK)	POS	POS	POS	NEG	POS	NEG	NEG	POS	NEG	NEG	NEG	NEG	NEG	NEG	NEG	NEG	NEG	NEG
NCTC13131, UK-EMRSA-4 (UK)	POS	POS	POS	NEG	POS	NEG	NEG	POS	NEG	NEG	NEG	NEG	NEG	NEG	NEG	NEG	NEG	NEG
	POS	POS	POS	AMB	POS	NEG	NEG	POS	NEG	NEG	NEG	NEG	NEG	NEG	NEG	NEG	NEG	NEG
Algiers_HT20040080	POS	POS	POS	NEG	POS	NEG	NEG	NEG	NEG	NEG	NEG	NEG	NEG	NEG	NEG	NEG	NEG	NEG
Dublin-DSH_AR09_0.0066	POS	POS	POS	NEG	POS		NEG	POS	NEG	NEG	NEG	NEG	NEG	NEG	NEG	NEG	NEG	NEG
Dublin-DSH_AR09_0-0065	POS	POS	POS	NEG	POS	NEG	NEG	POS	NEG	NEG	NEG	NEG	NEG	NEG	NEG	NEG	NEG	NEG
Dublin-DSH_Phenotype-III_84	POS	POS	POS	NEG	POS	NEG	NEG	POS	NEG	NEG	NEG	NEG	NEG	NEG	NEG	NEG	NEG	NEG
Hong Kong_130	POS	POS	POS	NEG	POS	NEG	NEG	POS	NEG	NEG	NEG	NEG	NEG	NEG	NEG	NEG	NEG	NEG
Kuwait_018	POS	POS	POS	NEG	POS	NEG	NEG	POS	NEG	NEG	NEG	NEG	NEG	NEG	NEG	NEG	NEG	NEG
Perth_08-17726	POS	POS	POS	NEG	POS	NEG	NEG	POS	NEG	NEG	NEG	NEG	NEG	NEG	NEG	NEG	NEG	NEG
Riyadh_Alfaisal/KKKSUH_86_MRS-14-279	POS	POS	POS	NEG	POS	NEG	NEG	POS	NEG	NEG	NEG	NEG	NEG	NEG	NEG	NEG	NEG	NEG
Riyadh_Alfaisal-04_Z3861_831588	POS	POS	POS	NEG	POS	NEG	NEG	POS	NEG	NEG	NEG	NEG	NEG	NEG	NEG	NEG	NEG	NEG
Riyadh_Alfaisal-30_S15724_1108013	POS	POS	POS	NEG	POS	NEG	NEG	POS	NEG	NEG	NEG	NEG	NEG	NEG	NEG	NEG	NEG	NEG
Riyadh_Alfaisal-6_22A_13_B3992_397721	POS	POS	POS	NEG	POS	NEG	NEG	POS	NEG	NEG	NEG	NEG	NEG	NEG	NEG	NEG	NEG	NEG
Riyadh_SSTI_52_3615482	POS	POS	POS	NEG	POS	NEG	NEG	POS	NEG	NEG	NEG	NEG	NEG	NEG	NEG	NEG	NEG	NEG
Riyadh-2817276-2	POS	POS	POS	NEG	POS	NEG	NEG	POS	NEG	NEG	NEG	NEG	NEG	NEG	NEG	NEG	NEG	NEG
Riyadh-2891670-W	POS	POS	POS	NEG	POS	NEG	NEG	POS	NEG	NEG	NEG	NEG	NEG	NEG	NEG	NEG	NEG	NEG
Riyadh-3006920-W	POS	POS	POS	NEG	POS	NEG	NEG	POS	NEG	NEG	NEG	NEG	NEG	NEG	NEG	NEG	NEG	NEG
Riyadh-R2567782	POS	POS	POS	NEG	POS	NEG	NEG	POS	NEG	NEG	NEG	NEG	NEG	NEG	NEG	AMB	NEG	NEG
Russia-18_0252_Krasnoyarsk_SK2	POS	POS	POS	NEG	POS	NEG	NEG	POS	NEG	NEG	NEG	NEG	NEG	NEG	NEG	NEG	NEG	NEG
UK-EMRSA-7	POS	POS	POS	AMB	POS	NEG	NEG	POS	NEG	NEG	NEG	NEG	NEG	NEG	NEG	AMB	NEG	NEG
>"South American/ Midc	POS							POS			NEG							
Dublin-DSH_AR23_0073	POS	POS	POS	NEG	POS	NEG	NEG	POS	NEG	NEG	NEG	NEG	NEG	NEG	NEG	NEG	NEG	NEG
>Related to "South Ame	POS							POS			NEG							
MRSA-OC3 (Russia)	POS	POS	POS	NEG	POS	NEG	NEG	POS	NEG	NEG	NEG	NEG	NEG	NEG	NEG	NEG	NEG	NEG
Russia-12_0176_Krasnoyarsk	POS	POS	POS	NEG	POS	NEG	NEG	POS	NEG	NEG	NEG	NEG	NEG	NEG	NEG	NEG	NEG	NEG
Russia-13_0180_Krasnoyarsk	POS	POS	POS	NEG	POS	NEG	NEG	POS	NEG	NEG	NEG	NEG	NEG	NEG	NEG	NEG	NEG	NEG
Russia-16_0249_Krasnoyarsk	POS	POS	POS	NEG	POS	NEG	NEG	POS	NEG	NEG	NEG	NEG	NEG	NEG	NEG	NEG	NEG	NEG
Russia-17_0250_Krasnoyarsk	POS	POS	POS	NEG	POS	NEG	NEG	POS	NEG	NEG	NEG	NEG	NEG	NEG	NEG	NEG	NEG	NEG
>Related to "South Ame	POS							POS			NEG							
URU110 (Uruguay)	POS	POS	POS	NEG	POS	NEG	NEG	POS	NEG	NEG	NEG	NEG	NEG	NEG	NEG	NEG	NEG	NEG
>Related to "South Ame	POS							POS			NEG							
DS_014 (Thailand)	POS	POS	POS	NEG	POS	NEG	NEG	POS	NEG	NEG	NEG	NEG	NEG	NEG	NEG	NEG	NEG	NEG
>Unassigned Middle Eas	POS							NEG			VAR							
Frankfurt_Oder_0490031797 (Libya)	POS	POS	POS	AMB	POS	NEG	NEG	NEG	NEG	NEG	POS	NEG	NEG	NEG	NEG	NEG	NEG	NEG
Kuwait_192	POS	POS	POS	NEG	POS	NEG	NEG	NEG	NEG	NEG	NEG	NEG	NEG	NEG	NEG	NEG	NEG	NEG
Riyadh-9	POS	POS	POS	NEG	POS	NEG	NEG	NEG	NEG	NEG	POS	NEG	NEG	NEG	NEG	NEG	NEG	NEG
Russia-24_0407_Moscow	POS	POS	POS	NEG	POS	NEG	NEG	NEG	NEG	NEG	NEG	NEG	NEG	NEG	NEG	NEG	NEG	NEG

STRAIN / ISOLATE	RESISTANCE : MLS-ANTIBIOTICS												RESISTANCE : AMINOGLYSOSIDES					
	msrA	mefA	mph(C)	vat(A)	vat(B)	vga(A)	vga(A) (BM 3327)	vgaB	vgaC	vgaD	vgaE	vgb	aacA-aphD	aadD	ant9		ble	aad6
	msrA	mefA	mph(C)	vat(A)	vat(B)	vga(A)	vga(A) (BM 3327)	vgaB	vgaC	vgaD	vgaE	vgb	aacA-aphD	aadD	ant9-C2944	ant9	ble	aad6
	energy-dependent efflux of erythro-mycin	macrolide efflux protein A	probable lysylphos-phatidyl-glycerol synthetase	virginia-mycin A acetyltransferase	acetyl-transferase inactivating streptogramin A	ATP binding protein, streptogramin-A-resistance		streptogramin A ABC transporter	streptogramin A ABC transporter	#NV	streptogramin A ABC transporter	virginia-mycin B hydrolase	bifunctional enzyme Aac/Aph, gentamicin resistance	amino-glycoside adenyl-transferase,tobramycin resistance	adenyltransferase AAd9		bleomycin resistance protein	aminoglycoside 6-adenylyltransferase
**>"Eurasian Clade": TUR1**													**POS**	**NEG**		**POS**	**NEG**	
TUR1 (Turkey)	NEG	NEG	NEG	NEG	NEG	NEG	NEG	NEG	NEG	NEG	NEG	NEG	POS	NEG	NEG	POS	NEG	NEG
TUR9 (Turkey)	NEG	NEG	NEG	NEG	NEG	NEG	NEG	NEG	NEG	NEG	NEG	NEG	POS	NEG	NEG	POS	NEG	NEG
**>"Eurasian Clade" : T013**													**NEG**	**NEG**		**POS**	**NEG**	
T0131 (China)	NEG	NEG	NEG	NEG	NEG	NEG	NEG	NEG	NEG	NEG	NEG	NEG	NEG	NEG	NEG	POS	NEG	NEG
MU4 (Turkey)	NEG	NEG	NEG	NEG	NEG	NEG	NEG	NEG	NEG	NEG	NEG	NEG	NEG	NEG	NEG	POS	NEG	NEG
DEU11 (Turkey)	NEG	NEG	NEG	NEG	NEG	NEG	NEG	NEG	NEG	NEG	NEG	NEG	NEG	NEG	NEG	POS	NEG	NEG
DEU16 (Turkey)	NEG	NEG	NEG	NEG	NEG	NEG	NEG	NEG	NEG	NEG	NEG	NEG	NEG	NEG	NEG	POS	NEG	NEG
Romania_Iasi_MRSA-07_54	NEG	NEG	NEG	NEG	NEG	NEG	NEG	NEG	NEG	NEG	NEG	NEG	NEG	NEG	NEG	POS	NEG	NEG
**>"Eurasian Clade": arsC-**													**VAR**	**NEG**		**POS**	**NEG**	
Romania_Iasi_MRSA-05_46	NEG	NEG	NEG	NEG	NEG	NEG	NEG	NEG	NEG	NEG	NEG	NEG	NEG	NEG	NEG	POS	NEG	NEG
Romania_Iasi_MRSA-06_47	NEG	NEG	NEG	NEG	NEG	NEG	NEG	NEG	NEG	NEG	NEG	NEG	POS	NEG	NEG	POS	NEG	NEG
**>"Eurasian Clade": 16K/**													**COMM**	**RARE**		**COMM**	**RARE**	
CN79 (China)	NEG	NEG	NEG	NEG	NEG	NEG	NEG	NEG	NEG	NEG	NEG	NEG	NEG	NEG	NEG	NEG	NEG	NEG
CUHK_BJ2002 (China)	NEG	NEG	NEG	NEG	NEG	NEG	NEG	NEG	NEG	NEG	NEG	NEG	POS	NEG	NEG	POS	NEG	NEG
CUHK_BJ2007 (China)	NEG	NEG	NEG	NEG	NEG	NEG	NEG	NEG	NEG	NEG	NEG	NEG	POS	NEG	NEG	POS	NEG	NEG
3HK (Czech Republic)	NEG	NEG	NEG	NEG	NEG	NEG	NEG	NEG	NEG	NEG	NEG	NEG	POS	NEG	NEG	POS	NEG	NEG
HU109 (Hungary)	NEG	NEG	NEG	NEG	NEG	NEG	NEG	NEG	NEG	NEG	NEG	NEG	POS	NEG	NEG	POS	NEG	NEG
HUR18 (Hungary)	NEG	NEG	NEG	NEG	NEG	NEG	NEG	NEG	NEG	NEG	NEG	NEG	POS	NEG	NEG	POS	NEG	NEG
H482 (Romania)	NEG	NEG	NEG	NEG	NEG	NEG	NEG	NEG	NEG	NEG	NEG	NEG	POS	NEG	NEG	POS	NEG	NEG
16K (Russia)	NEG	NEG	NEG	NEG	NEG	NEG	NEG	NEG	NEG	NEG	NEG	NEG	POS	NEG	NEG	POS	NEG	NEG
DEU10 (Turkey)	NEG	NEG	NEG	NEG	NEG	NEG	NEG	NEG	NEG	NEG	NEG	NEG	POS	NEG	NEG	POS	NEG	NEG
DEU12 (Turkey)	NEG	NEG	NEG	NEG	NEG	NEG	NEG	NEG	NEG	NEG	NEG	NEG	POS	NEG	NEG	POS	NEG	NEG
DEU14 (Turkey)	NEG	NEG	NEG	NEG	NEG	NEG	NEG	NEG	NEG	NEG	NEG	NEG	POS	NEG	NEG	POS	NEG	NEG
DEU15 (Turkey)	NEG	NEG	NEG	NEG	NEG	NEG	NEG	NEG	NEG	NEG	NEG	NEG	POS	NEG	NEG	POS	NEG	NEG
DEU17 (Turkey)	NEG	NEG	NEG	NEG	NEG	NEG	NEG	NEG	NEG	NEG	NEG	NEG	POS	NEG	NEG	POS	NEG	NEG
DEU19 (Turkey)	NEG	NEG	NEG	NEG	NEG	NEG	NEG	NEG	NEG	NEG	NEG	NEG	POS	NEG	NEG	POS	NEG	NEG
DEU2 (Turkey)	NEG	NEG	NEG	NEG	NEG	NEG	NEG	NEG	NEG	NEG	NEG	NEG	POS	NEG	NEG	POS	NEG	NEG
DEU20 (Turkey)	NEG	NEG	NEG	NEG	NEG	NEG	NEG	NEG	NEG	NEG	NEG	NEG	POS	NEG	NEG	POS	NEG	NEG
DEU23 (Turkey)	NEG	NEG	NEG	NEG	NEG	NEG	NEG	NEG	NEG	NEG	NEG	NEG	POS	NEG	NEG	POS	NEG	NEG
DEU3 (Turkey)	NEG	NEG	NEG	NEG	NEG	NEG	NEG	NEG	NEG	NEG	NEG	NEG	POS	NEG	NEG	POS	NEG	NEG
DEU5 (Turkey)	NEG	NEG	NEG	NEG	NEG	NEG	NEG	NEG	NEG	NEG	NEG	NEG	POS	NEG	NEG	POS	NEG	NEG
DEU6 (Turkey)	NEG	NEG	NEG	NEG	NEG	NEG	NEG	NEG	NEG	NEG	NEG	NEG	POS	NEG	NEG	POS	NEG	NEG
DEU8 (Turkey)	NEG	NEG	NEG	NEG	NEG	NEG	NEG	NEG	NEG	NEG	NEG	NEG	POS	NEG	NEG	POS	NEG	NEG
HU11 (Turkey)	NEG	NEG	NEG	NEG	NEG	NEG	NEG	NEG	NEG	NEG	NEG	NEG	POS	NEG	NEG	POS	NEG	NEG
HU13 (Turkey)	NEG	NEG	NEG	NEG	NEG	NEG	NEG	NEG	NEG	NEG	NEG	NEG	POS	NEG	NEG	POS	NEG	NEG
HU14 (Turkey)	NEG	NEG	NEG	NEG	NEG	NEG	NEG	NEG	NEG	NEG	NEG	NEG	POS	NEG	NEG	POS	NEG	NEG
HU15 (Turkey)	NEG	NEG	NEG	NEG	NEG	NEG	NEG	NEG	NEG	NEG	NEG	NEG	POS	NEG	NEG	POS	NEG	NEG
HU16 (Turkey)	NEG	NEG	NEG	NEG	NEG	NEG	NEG	NEG	NEG	NEG	NEG	NEG	POS	NEG	NEG	POS	NEG	NEG
HU17 (Turkey)	NEG	NEG	NEG	NEG	NEG	NEG	NEG	NEG	NEG	NEG	NEG	NEG	POS	NEG	NEG	POS	NEG	NEG
HU21 (Turkey)	NEG	NEG	NEG	NEG	NEG	NEG	NEG	NEG	NEG	NEG	NEG	NEG	POS	NEG	NEG	POS	NEG	NEG
HU23 (Turkey)	NEG	NEG	NEG	NEG	NEG	NEG	NEG	NEG	NEG	NEG	NEG	NEG	POS	NEG	NEG	POS	NEG	NEG
HU26 (Turkey)	NEG	NEG	NEG	NEG	NEG	NEG	NEG	NEG	NEG	NEG	NEG	NEG	POS	NEG	NEG	POS	NEG	NEG
HU4 (Turkey)	NEG	NEG	NEG	NEG	NEG	NEG	NEG	NEG	NEG	NEG	NEG	NEG	POS	NEG	NEG	POS	NEG	NEG
HU5 (Turkey)	NEG	NEG	NEG	NEG	NEG	NEG	NEG	NEG	NEG	NEG	NEG	NEG	POS	NEG	NEG	POS	NEG	NEG
HU6 (Turkey)	NEG	NEG	NEG	NEG	NEG	NEG	NEG	NEG	NEG	NEG	NEG	NEG	POS	NEG	NEG	POS	NEG	NEG
HU7 (Turkey)	NEG	NEG	NEG	NEG	NEG	NEG	NEG	NEG	NEG	NEG	NEG	NEG	POS	NEG	NEG	POS	NEG	NEG
HU8 (Turkey)	NEG	NEG	NEG	NEG	NEG	NEG	NEG	NEG	NEG	NEG	NEG	NEG	POS	NEG	NEG	POS	NEG	NEG
HU9 (Turkey)	NEG	NEG	NEG	NEG	NEG	NEG	NEG	NEG	NEG	NEG	NEG	NEG	POS	NEG	NEG	POS	NEG	NEG
IU1 (Turkey)	NEG	NEG	NEG	NEG	NEG	NEG	NEG	NEG	NEG	NEG	NEG	NEG	POS	NEG	NEG	POS	NEG	NEG
IU10 (Turkey)	NEG	NEG	NEG	NEG	NEG	NEG	NEG	NEG	NEG	NEG	NEG	NEG	POS	NEG	NEG	POS	NEG	NEG
IU11 (Turkey)	NEG	NEG	NEG	NEG	NEG	NEG	NEG	NEG	NEG	NEG	NEG	NEG	POS	NEG	NEG	POS	NEG	NEG
IU12 (Turkey)	NEG	NEG	NEG	NEG	NEG	NEG	NEG	NEG	NEG	NEG	NEG	NEG	POS	NEG	NEG	POS	NEG	NEG
IU13 (Turkey)	NEG	NEG	NEG	NEG	NEG	NEG	NEG	NEG	NEG	NEG	NEG	NEG	POS	NEG	NEG	POS	NEG	NEG
IU15 (Turkey)	NEG	NEG	NEG	NEG	NEG	NEG	NEG	NEG	NEG	NEG	NEG	NEG	POS	NEG	NEG	POS	NEG	NEG
IU18 (Turkey)	NEG	NEG	NEG	NEG	NEG	NEG	NEG	NEG	NEG	NEG	NEG	NEG	POS	NEG	NEG	POS	NEG	NEG
IU19 (Turkey)	NEG	NEG	NEG	NEG	NEG	NEG	NEG	NEG	NEG	NEG	NEG	NEG	NEG	NEG	NEG	POS	NEG	NEG
IU2 (Turkey)	NEG	NEG	NEG	NEG	NEG	NEG	NEG	NEG	NEG	NEG	NEG	NEG	POS	NEG	NEG	POS	NEG	NEG
IU4 (Turkey)	NEG	NEG	NEG	NEG	NEG	NEG	NEG	NEG	NEG	NEG	NEG	NEG	POS	NEG	NEG	POS	NEG	NEG
IU6 (Turkey)	NEG	NEG	NEG	NEG	NEG	NEG	NEG	NEG	NEG	NEG	NEG	NEG	POS	NEG	NEG	POS	NEG	NEG
IU7 (Turkey)	NEG	NEG	NEG	NEG	NEG	NEG	NEG	NEG	NEG	NEG	NEG	NEG	POS	NEG	NEG	POS	NEG	NEG
IU9 (Turkey)	NEG	NEG	NEG	NEG	NEG	NEG	NEG	NEG	NEG	NEG	NEG	NEG	POS	NEG	NEG	POS	NEG	NEG
MU1 (Turkey)	NEG	NEG	NEG	NEG	NEG	NEG	NEG	NEG	NEG	NEG	NEG	NEG	POS	NEG	NEG	POS	NEG	NEG
MU10 (Turkey)	NEG	NEG	NEG	NEG	NEG	NEG	NEG	NEG	NEG	NEG	NEG	NEG	POS	NEG	NEG	POS	NEG	NEG
MU20 (Turkey)	NEG	NEG	NEG	NEG	NEG	NEG	NEG	NEG	NEG	NEG	NEG	NEG	POS	NEG	NEG	POS	NEG	NEG
Mu3 (Turkey)	NEG	NEG	NEG	NEG	NEG	NEG	NEG	NEG	NEG	NEG	NEG	NEG	POS	POS	NEG	POS	POS	NEG
MU5 (Turkey)	NEG	NEG	NEG	NEG	NEG	NEG	NEG	NEG	NEG	NEG	NEG	NEG	POS	NEG	NEG	POS	NEG	NEG
MU6 (Turkey)	NEG	NEG	NEG	NEG	NEG	NEG	NEG	NEG	NEG	NEG	NEG	NEG	POS	NEG	NEG	POS	NEG	NEG
MU7 (Turkey)	NEG	NEG	NEG	NEG	NEG	NEG	NEG	NEG	NEG	NEG	NEG	NEG	POS	NEG	NEG	POS	NEG	NEG
TUR27 (Turkey)	NEG	NEG	NEG	NEG	NEG	NEG	NEG	NEG	NEG	NEG	NEG	NEG	POS	NEG	NEG	POS	NEG	NEG
NCTR 32S	NEG	NEG	NEG	NEG	NEG	NEG	NEG	NEG	NEG	NEG	NEG	NEG	POS	NEG	NEG	POS	NEG	NEG
SA02_A4 = Russia-06_0085_Moscow	NEG	NEG	NEG	NEG	NEG	NEG	NEG	NEG	NEG	NEG	NEG	NEG	POS	NEG	NEG	POS	NEG	NEG
	NEG	NEG	NEG	NEG	NEG	NEG	NEG	NEG	NEG	NEG	NEG	NEG	POS	NEG	NEG	POS	NEG	NEG
Dresden_08V35987 (Turkey)	NEG	NEG	NEG	NEG	NEG	NEG	NEG	NEG	NEG	NEG	NEG	NEG	POS	NEG	NEG	POS	NEG	NEG
Dresden_17ANR577152	NEG	NEG	NEG	NEG	NEG	NEG	NEG	NEG	NEG	NEG	NEG	NEG	POS	NEG	NEG	POS	NEG	NEG
Dresden_17ANR580374 (Makedonia)	NEG	NEG	NEG	NEG	NEG	NEG	NEG	NEG	NEG	NEG	NEG	NEG	POS	NEG	NEG	POS	NEG	NEG
Hong Kong_69-II	NEG	NEG	NEG	NEG	NEG	NEG	NEG	NEG	NEG	NEG	NEG	NEG	POS	NEG	NEG	POS	NEG	NEG
Hong Kong_93	NEG	NEG	NEG	NEG	NEG	NEG	NEG	NEG	NEG	NEG	NEG	NEG	POS	NEG	NEG	POS	NEG	NEG
Rawalpindi_Kidney Center_03_SK1	NEG	NEG	NEG	NEG	NEG	NEG	NEG	NEG	NEG	NEG	NEG	NEG	POS	NEG	NEG	POS	NEG	NEG
Rawalpindi_Kidney Center_08	NEG	NEG	NEG	NEG	NEG	NEG	NEG	NEG	NEG	NEG	NEG	NEG	POS	NEG	NEG	POS	NEG	NEG
Rawalpindi_Kidney Center_10	NEG	NEG	NEG	NEG	NEG	NEG	NEG	NEG	NEG	NEG	NEG	NEG	POS	NEG	NEG	POS	NEG	NEG
Rawalpindi_Kidney Center_19	NEG	NEG	NEG	NEG	NEG	NEG	NEG	NEG	NEG	NEG	NEG	NEG	POS	NEG	NEG	POS	NEG	NEG
Rawalpindi_Kidney Center_50_SK1	NEG	NEG	NEG	NEG	NEG	NEG	NEG	NEG	NEG	NEG	NEG	NEG	POS	NEG	NEG	POS	NEG	NEG
Romania_Iasi_BC-49_430	NEG	NEG	NEG	NEG	NEG	NEG	NEG	NEG	NEG	NEG	NEG	NEG	POS	NEG	NEG	POS	NEG	NEG
Romania_Iasi_MRSA_284	NEG	NEG	NEG	NEG	NEG	NEG	NEG	NEG	NEG	NEG	NEG	NEG	POS	NEG	NEG	POS	NEG	NEG
Romania_Iasi_MRSA-32_318	NEG	NEG	NEG	NEG	NEG	NEG	NEG	NEG	NEG	NEG	NEG	NEG	POS	NEG	NEG	POS	NEG	NEG
Romania_Iasi_MRSA-54_430	NEG	NEG	NEG	NEG	NEG	AMB	NEG	NEG	NEG	NEG	NEG	NEG	POS	NEG	NEG	POS	NEG	NEG
Romania_Iasi_SSTI-01_2	NEG	NEG	NEG	NEG	NEG	NEG	NEG	NEG	NEG	NEG	NEG	NEG	POS	NEG	NEG	POS	NEG	NEG
Romania_Iasi_SSTI-10_101	NEG	NEG	NEG	NEG	NEG	NEG	NEG	NEG	NEG	NEG	NEG	NEG	POS	NEG	NEG	POS	NEG	NEG
Romania_Iasi_SSTI-11_106	NEG	NEG	NEG	NEG	NEG	NEG	NEG	NEG	NEG	NEG	NEG	NEG	POS	NEG	NEG	POS	NEG	NEG
Russia-01_0001_SaintPetersburg	NEG	NEG	NEG	NEG	NEG	NEG	NEG	NEG	NEG	NEG	NEG	NEG	POS	NEG	NEG	POS	NEG	NEG
Russia-02_0004_SaintPetersburg	NEG	NEG	NEG	NEG	NEG	NEG	NEG	NEG	NEG	NEG	NEG	NEG	POS	NEG	NEG	POS	NEG	NEG
Russia-03_0057_SaintPetersburg	NEG	NEG	NEG	NEG	NEG	NEG	NEG	NEG	NEG	NEG	NEG	NEG	POS	NEG	NEG	POS	NEG	NEG
Russia-04_0076_Moscow	NEG	NEG	NEG	NEG	NEG	NEG	NEG	NEG	NEG	NEG	NEG	NEG	POS	NEG	NEG	POS	NEG	NEG
Russia-05_0078_Moscow	NEG	NEG	NEG	NEG	NEG	NEG	NEG	NEG	NEG	NEG	NEG	NEG	POS	NEG	NEG	POS	NEG	NEG
Russia-08_0135_Moscow	NEG	NEG	NEG	NEG	NEG	NEG	NEG	NEG	NEG	NEG	NEG	NEG	POS	NEG	NEG	POS	NEG	NEG
Russia-09_0150_Kurgan	NEG	NEG	NEG	NEG	NEG	NEG	NEG	NEG	NEG	NEG	NEG	NEG	POS	NEG	NEG	POS	NEG	NEG
Russia-10_0162_Kurgan	NEG	NEG	NEG	NEG	NEG	NEG	NEG	NEG	NEG	NEG	NEG	NEG	POS	NEG	NEG	POS	NEG	NEG
Russia-14_0184_Moscow	NEG	NEG	NEG	NEG	NEG	NEG	NEG	NEG	NEG	NEG	NEG	NEG	POS	NEG	NEG	POS	NEG	NEG
Russia-15_0232_SaintPetersburg	NEG	NEG	NEG	NEG	NEG	NEG	NEG	NEG	NEG	NEG	NEG	NEG	POS	NEG	NEG	POS	NEG	NEG
Russia-22_0391_Chelyabinsk	NEG	NEG	NEG	NEG	NEG	NEG	NEG	NEG	NEG	NEG	NEG	NEG	POS	NEG	NEG	POS	NEG	NEG
Russia-23_0392_Chelyabinsk	NEG	NEG	NEG	NEG	NEG	NEG	NEG	NEG	NEG	NEG	NEG	NEG	POS	NEG	NEG	POS	NEG	NEG
**>"Eurasian Clade" : IU17**													**POS**	**NEG**		**POS**	**NEG**	
IU17 (Turkey)	NEG	NEG	NEG	NEG	NEG	NEG	NEG	NEG	NEG	NEG	NEG	NEG	POS	NEG	NEG	POS	NEG	NEG
IU20 (Turkey)	NEG	NEG	NEG	NEG	NEG	NEG	NEG	NEG	NEG	NEG	NEG	NEG	POS	NEG	NEG	POS	NEG	NEG

STRAIN / ISOLATE	RESISTANCE : MLS-ANTIBIOTICS												RESISTANCE : AMINOGLYSOSIDES					
	msrA	mefA	mph(C)	vat(A)	vat(B)	vga(A)	vga(A) (BM 3327)	vgaB	vgaC	vgaD	vgaE	vgb	aacA-aphD	aadD	ant9		ble	aad6
	msrA	mefA	mph(C)	vat(A)	vat(B)	vga(A)	vga(A) (BM 3327)	vgaB	vgaC	vgaD	vgaE	vgb	aacA-aphD	aadD	ant9-C2944	ant9	ble	aad6
	energy-dependent efflux of erythro-mycin	macrolide efflux protein A	probable lysylphos-phatidyl-glycerol synthetase	virginia-mycin A acetyltransferase	acetyl-transferase inactivating streptogramin A	ATP binding protein, streptogramin-A-resistance		streptogramin A ABC transporter	streptogramin A ABC transporter	#NV	streptogramin A ABC transporter	virginia-mycin B hydrolase	bifunctional enzyme Aac/Aph, gentamicin resistance	amino-glycoside adenyl-transferase,tobramycin resistance	adenyltransferase AAd9		bleomycin resistance protein	aminoglycoside 6-adenylyltransferase
>"Eurasian Clade": Spor													POS	NEG		POS	NEG	
Dresden_16ANR581769	NEG	NEG	NEG	NEG	NEG	NEG	NEG	NEG	NEG	NEG	NEG	NEG	POS	NEG	NEG	POS	NEG	NEG
Dresden_17ANR578769	NEG	NEG	NEG	NEG	NEG	NEG	NEG	NEG	NEG	NEG	NEG	NEG	POS	NEG	NEG	POS	NEG	NEG
>"Eurasian Clade": Kuwa													POS	NEG		VAR	NEG	
Kuwait_306	NEG	NEG	NEG	NEG	NEG	AMB	NEG	NEG	NEG	NEG	NEG	NEG	POS	NEG	NEG	POS	NEG	NEG
Kuwait_301	NEG	NEG	NEG	NEG	NEG	NEG	NEG	NEG	NEG	NEG	NEG	NEG	POS	NEG	NEG	NEG	NEG	NEG
>"European Clade": "Gr													COMM	NEG		NEG	NEG	
GRE18 (Greece)	NEG	NEG	NEG	NEG	NEG	NEG	NEG	NEG	NEG	NEG	NEG	NEG	POS	NEG	NEG	NEG	NEG	NEG
GRE317 (Greece)	NEG	NEG	NEG	NEG	NEG	NEG	NEG	NEG	NEG	NEG	NEG	NEG	POS	NEG	NEG	NEG	NEG	NEG
GRE4 (Greece)	NEG	NEG	NEG	NEG	NEG	NEG	NEG	NEG	NEG	NEG	NEG	NEG	POS	NEG	NEG	NEG	NEG	NEG
R35 (USA)	NEG	NEG	NEG	NEG	NEG	NEG	NEG	NEG	NEG	NEG	NEG	NEG	POS	NEG	NEG	NEG	NEG	NEG
Casablanca_HT20060548	NEG	NEG	NEG	NEG	NEG	NEG	NEG	NEG	NEG	NEG	NEG	NEG	POS	NEG	NEG	NEG	NEG	NEG
Casablanca_HT20060550	NEG	NEG	NEG	NEG	NEG	NEG	NEG	NEG	NEG	NEG	NEG	NEG	POS	NEG	NEG	NEG	NEG	NEG
Dresden_01V34572 (Greece)	NEG	NEG	NEG	NEG	NEG	NEG	NEG	NEG	NEG	NEG	NEG	NEG	POS	NEG	NEG	NEG	NEG	NEG
Dresden_01V36469	NEG	NEG	NEG	NEG	NEG	NEG	NEG	NEG	NEG	NEG	NEG	NEG	POS	NEG	NEG	NEG	NEG	NEG
Dresden_01V39123	NEG	NEG	NEG	NEG	NEG	NEG	NEG	NEG	NEG	NEG	NEG	NEG	POS	NEG	NEG	NEG	NEG	NEG
Dresden_01V39143	NEG	NEG	NEG	NEG	NEG	NEG	NEG	NEG	NEG	NEG	NEG	NEG	POS	NEG	NEG	NEG	NEG	NEG
Greece 1_3680_Harmony	NEG	NEG	NEG	NEG	NEG	NEG	NEG	NEG	NEG	NEG	NEG	NEG	NEG	NEG	NEG	NEG	NEG	NEG
>"European Clade": UK-													COMM	NEG		POS	NEG	
ANS46 (Australia)	NEG	NEG	NEG	NEG	NEG	NEG	NEG	NEG	NEG	NEG	NEG	NEG	POS	NEG	NEG	POS	NEG	NEG
LHH1 (USA)	NEG	NEG	NEG	NEG	NEG	NEG	NEG	NEG	NEG	NEG	NEG	NEG	POS	NEG	NEG	POS	NEG	NEG
Dublin-DSH_AR01_0-1085	NEG	NEG	NEG	NEG	NEG	NEG	NEG	NEG	NEG	NEG	NEG	NEG	POS	NEG	NEG	POS	NEG	NEG
Dublin-DSH_AR01_0-1118	NEG	NEG	NEG	NEG	NEG	NEG	NEG	NEG	NEG	NEG	NEG	NEG	NEG	NEG	NEG	POS	NEG	NEG
Dublin-DSH_AR15_0098	NEG	NEG	NEG	NEG	NEG	NEG	NEG	NEG	NEG	NEG	NEG	NEG	POS	NEG	NEG	POS	NEG	NEG
Dublin-DSH_AR15_0104	NEG	NEG	NEG	NEG	NEG	NEG	NEG	NEG	NEG	NEG	NEG	NEG	POS	NEG	NEG	POS	NEG	NEG
UK_NCTC11939_UK-1_Harmony	NEG	NEG	NEG	NEG	NEG	NEG	NEG	NEG	NEG	NEG	NEG	NEG	POS	NEG	NEG	POS	NEG	NEG
>"European Clade": BK2													POS	NEG		POS	NEG	
BK2421 (USA)	NEG	NEG	NEG	NEG	NEG	NEG	NEG	NEG	NEG	NEG	NEG	NEG	POS	NEG	NEG	POS	NEG	NEG
>"Aussi/NZ Clade", JKD6													COMM	COMM		POS	POS	
JKD6009 (Australia)	NEG	NEG	NEG	NEG	NEG	NEG	NEG	NEG	NEG	NEG	NEG	NEG	POS	POS	NEG	POS	POS	NEG
JKD6008 (New Zealand)	NEG	NEG	NEG	NEG	NEG	NEG	NEG	NEG	NEG	NEG	NEG	NEG	POS	POS	NEG	POS	POS	NEG
Perth_2015-S20209	NEG	NEG	NEG	NEG	NEG	NEG	NEG	NEG	NEG	NEG	NEG	NEG	POS	POS	NEG	POS	POS	NEG
Perth_2015-S20305	NEG	NEG	NEG	NEG	NEG	NEG	NEG	NEG	NEG	NEG	NEG	NEG	POS	POS	NEG	POS	POS	NEG
Perth_2015-S20603	NEG	NEG	NEG	NEG	NEG	NEG	NEG	NEG	NEG	NEG	NEG	NEG	POS	POS	NEG	POS	POS	NEG
Perth_2015-S20607	NEG	NEG	NEG	NEG	NEG	NEG	NEG	NEG	NEG	NEG	NEG	NEG	POS	POS	NEG	POS	POS	NEG
Perth_2015-S20608	NEG	NEG	NEG	NEG	NEG	NEG	NEG	NEG	NEG	NEG	NEG	NEG	POS	POS	NEG	POS	POS	NEG
Perth_2015-S20610	NEG	NEG	NEG	NEG	NEG	NEG	NEG	NEG	NEG	NEG	NEG	NEG	POS	POS	NEG	POS	POS	NEG
Perth_2015-S20613	NEG	NEG	NEG	NEG	NEG	NEG	NEG	NEG	NEG	NEG	NEG	NEG	POS	POS	NEG	POS	POS	NEG
Perth_2015-S20617	NEG	NEG	NEG	NEG	NEG	NEG	NEG	NEG	NEG	NEG	NEG	NEG	POS	POS	NEG	POS	POS	NEG
Perth_2015-S20622	NEG	NEG	NEG	NEG	NEG	NEG	NEG	NEG	NEG	NEG	NEG	NEG	NEG	POS	NEG	POS	POS	NEG
Perth_2015-S23619	NEG	NEG	NEG	NEG	NEG	NEG	NEG	NEG	NEG	NEG	NEG	NEG	POS	POS	NEG	POS	POS	NEG
Perth_2015-S41210	NEG	NEG	NEG	NEG	NEG	NEG	NEG	NEG	NEG	NEG	NEG	NEG	POS	POS	NEG	POS	POS	NEG
Perth_2015-S51001	NEG	NEG	NEG	NEG	NEG	NEG	NEG	NEG	NEG	NEG	NEG	NEG	POS	POS	NEG	POS	POS	NEG
Perth_2015-S51003	NEG	NEG	NEG	NEG	NEG	NEG	NEG	NEG	NEG	NEG	NEG	NEG	POS	POS	NEG	POS	POS	NEG
Perth_2015-S51006	NEG	NEG	NEG	NEG	NEG	NEG	NEG	NEG	NEG	NEG	NEG	NEG	POS	POS	NEG	POS	POS	NEG
Perth_2015-S51007	NEG	NEG	NEG	NEG	NEG	NEG	NEG	NEG	NEG	NEG	NEG	NEG	POS	POS	NEG	POS	POS	NEG
Perth_2015-S51008	NEG	NEG	NEG	NEG	NEG	NEG	NEG	NEG	NEG	NEG	NEG	NEG	NEG	POS	NEG	POS	POS	NEG
Perth_2015-S51014	NEG	NEG	NEG	NEG	NEG	NEG	NEG	NEG	NEG	NEG	NEG	NEG	POS	POS	NEG	POS	POS	NEG
Perth_2015-S51408	NEG	NEG	NEG	NEG	NEG	NEG	NEG	NEG	NEG	NEG	NEG	NEG	POS	POS	NEG	POS	POS	NEG
Perth_2016-S20614	NEG	NEG	NEG	NEG	NEG	NEG	NEG	NEG	NEG	NEG	NEG	NEG	POS	POS	NEG	POS	POS	NEG
Perth_2016-S23614	NEG	NEG	NEG	NEG	NEG	NEG	NEG	NEG	NEG	NEG	NEG	NEG	POS	POS	NEG	POS	POS	NEG
Perth_2016-S42009	NEG	NEG	NEG	NEG	NEG	NEG	NEG	NEG	NEG	NEG	NEG	NEG	POS	POS	NEG	POS	POS	NEG
Perth_2016-S51001	NEG	NEG	NEG	NEG	NEG	NEG	NEG	NEG	NEG	NEG	NEG	NEG	POS	POS	NEG	POS	POS	NEG
Perth_2016-S51403	NEG	NEG	NEG	NEG	NEG	NEG	NEG	NEG	NEG	NEG	NEG	NEG	POS	POS	NEG	POS	POS	NEG
Perth_07 RPA 20	NEG	NEG	NEG	NEG	NEG	NEG	NEG	NEG	NEG	NEG	NEG	NEG	POS	POS	NEG	POS	POS	NEG
Perth_08-19088	NEG	NEG	NEG	NEG	NEG	NEG	NEG	NEG	NEG	NEG	NEG	NEG	POS	POS	NEG	POS	POS	NEG
Perth_01-15351	NEG	NEG	NEG	NEG	NEG	NEG	NEG	NEG	NEG	NEG	NEG	NEG	AMB	POS	NEG	POS	POS	NEG
Perth_01-15357	NEG	NEG	NEG	NEG	NEG	NEG	NEG	NEG	NEG	NEG	NEG	NEG	POS	POS	NEG	POS	POS	NEG
Perth_01-15419	NEG	NEG	NEG	NEG	NEG	NEG	NEG	NEG	NEG	NEG	NEG	NEG	POS	AMB	NEG	POS	POS	NEG
Perth_01-16309	NEG	NEG	NEG	NEG	NEG	NEG	NEG	NEG	NEG	NEG	NEG	NEG	AMB	POS	NEG	POS	POS	NEG
Perth_03 SNP 95	NEG	NEG	NEG	NEG	NEG	NEG	NEG	NEG	NEG	NEG	NEG	NEG	POS	POS	NEG	POS	POS	NEG
Perth_2003_AH_47	NEG	NEG	NEG	NEG	NEG	NEG	NEG	NEG	NEG	NEG	NEG	NEG	POS	POS	NEG	POS	POS	NEG
>"Australian/NZ Clade":													POS	POS		POS	POS	
Perth_2015-S20623	NEG	NEG	NEG	NEG	NEG	NEG	NEG	NEG	NEG	NEG	NEG	NEG	POS	POS	NEG	POS	POS	NEG
Perth_2015-S20826	NEG	NEG	NEG	NEG	NEG	NEG	NEG	NEG	NEG	NEG	NEG	NEG	POS	POS	NEG	POS	POS	NEG
Perth_05 NH 56	NEG	NEG	NEG	NEG	NEG	NEG	NEG	NEG	NEG	NEG	NEG	NEG	POS	POS	NEG	POS	POS	NEG
>CC5-MRSA-III LA MRSA													NEG	NEG		POS	NEG	
Delbrück 38 isolate from domestic turkey	NEG	NEG	NEG	NEG	NEG	NEG	NEG	NEG	NEG	NEG	NEG	NEG	NEG	NEG	NEG	POS	NEG	NEG
> Staph. pseudintermed													NEG	NEG		NEG	NEG	
KM1381	NEG	NEG	NEG	NEG	NEG	NEG	NEG	NEG	NEG	NEG	NEG	NEG	NEG	NEG	NEG	NEG	NEG	NEG
>"South-East Asian Clad													COMM	RARE		COMM	NEG	
M92 (Canada)	NEG	NEG	NEG	NEG	NEG	NEG	NEG	NEG	NEG	NEG	NEG	NEG	POS	NEG	NEG	POS	NEG	NEG
CHI59 (China)	NEG	NEG	NEG	NEG	NEG	NEG	NEG	NEG	NEG	NEG	NEG	NEG	POS	NEG	NEG	POS	NEG	NEG
CHI61 (China)	NEG	NEG	NEG	NEG	NEG	NEG	NEG	NEG	NEG	NEG	NEG	NEG	POS	NEG	NEG	POS	NEG	NEG
CUHK_HK1997 (China)	NEG	NEG	NEG	NEG	NEG	NEG	NEG	NEG	NEG	NEG	NEG	NEG	POS	NEG	NEG	POS	NEG	NEG
M996 (China)	NEG	NEG	NEG	NEG	NEG	NEG	NEG	NEG	NEG	NEG	NEG	NEG	POS	NEG	NEG	POS	NEG	NEG
H211 (Denmark)	NEG	NEG	NEG	NEG	NEG	NEG	NEG	NEG	NEG	NEG	NEG	NEG	POS	NEG	NEG	POS	NEG	NEG
H216 (Denmark)	NEG	NEG	NEG	NEG	NEG	NEG	NEG	NEG	NEG	NEG	NEG	NEG	POS	NEG	NEG	POS	NEG	NEG
D71 (Germany)	NEG	NEG	NEG	NEG	NEG	NEG	NEG	NEG	NEG	NEG	NEG	NEG	POS	POS	NEG	POS	NEG	NEG
NMR05 (India)	NEG	NEG	NEG	NEG	NEG	NEG	NEG	NEG	NEG	NEG	NEG	NEG	POS	NEG	NEG	POS	NEG	NEG
MAL11 (Malaysia)	NEG	NEG	NEG	NEG	NEG	NEG	NEG	NEG	NEG	NEG	NEG	NEG	POS	NEG	NEG	POS	NEG	NEG
MAL9 (Malaysia)	NEG	NEG	NEG	NEG	NEG	NEG	NEG	NEG	NEG	NEG	NEG	NEG	POS	NEG	NEG	POS	NEG	NEG
Na21 (Sri Lanka)	NEG	NEG	NEG	NEG	NEG	NEG	NEG	NEG	NEG	NEG	NEG	NEG	POS	NEG	NEG	POS	NEG	NEG
M592 (Syria)	NEG	NEG	NEG	NEG	NEG	NEG	NEG	NEG	NEG	NEG	NEG	NEG	POS	NEG	NEG	POS	NEG	NEG
H202 (Thailand)	NEG	NEG	NEG	NEG	NEG	NEG	NEG	NEG	NEG	NEG	NEG	NEG	POS	NEG	NEG	POS	NEG	NEG
S102 (Thailand)	NEG	NEG	NEG	NEG	NEG	NEG	NEG	NEG	NEG	NEG	NEG	NEG	POS	NEG	NEG	POS	NEG	NEG
S130 (Thailand)	NEG	NEG	NEG	NEG	NEG	NEG	NEG	NEG	NEG	NEG	NEG	NEG	POS	NEG	NEG	POS	NEG	NEG
S40 (Thailand)	NEG	NEG	NEG	NEG	NEG	NEG	NEG	NEG	NEG	NEG	NEG	NEG	POS	NEG	NEG	POS	NEG	NEG
S71 (Thailand)	NEG	NEG	NEG	NEG	NEG	NEG	NEG	NEG	NEG	NEG	NEG	NEG	POS	NEG	NEG	POS	NEG	NEG
S87 (Thailand)	NEG	NEG	NEG	NEG	NEG	NEG	NEG	NEG	NEG	NEG	NEG	NEG	POS	NEG	NEG	POS	NEG	NEG
S93 (Thailand)	NEG	NEG	NEG	NEG	NEG	NEG	NEG	NEG	NEG	NEG	NEG	NEG	POS	NEG	NEG	POS	NEG	NEG
US_002 (Thailand)	NEG	NEG	NEG	NEG	NEG	NEG	NEG	NEG	NEG	NEG	NEG	NEG	POS	NEG	NEG	POS	NEG	NEG
US_017 (Thailand)	NEG	NEG	NEG	NEG	NEG	NEG	NEG	NEG	NEG	NEG	NEG	NEG	POS	NEG	NEG	POS	NEG	NEG
WAMC6102 (Thailand)	NEG	NEG	NEG	NEG	NEG	NEG	NEG	NEG	NEG	NEG	NEG	NEG	POS	NEG	NEG	NEG	NEG	NEG
NCTC_13626_TW20 (UK)	NEG	NEG	NEG	NEG	NEG	NEG	NEG	NEG	NEG	NEG	NEG	NEG	POS	NEG	NEG	POS	NEG	NEG
BSAC3021 (UK)	NEG	NEG	NEG	NEG	NEG	NEG	NEG	NEG	NEG	NEG	NEG	NEG	POS	NEG	NEG	POS	NEG	NEG
BSAC3046 (UK)	NEG	NEG	NEG	NEG	NEG	NEG	NEG	NEG	NEG	NEG	NEG	NEG	POS	NEG	NEG	POS	NEG	NEG
1193_SAUR (USA)	NEG	NEG	NEG	NEG	NEG	NEG	NEG	NEG	NEG	NEG	NEG	NEG	POS	NEG	NEG	POS	NEG	NEG
1194_SAUR (USA)	NEG	NEG	NEG	NEG	NEG	NEG	NEG	NEG	NEG	NEG	NEG	NEG	POS	NEG	NEG	POS	NEG	NEG

STRAIN / ISOLATE	RESISTANCE : MLS-ANTIBIOTICS												RESISTANCE : AMINOGLYSOSIDES					
	msrA	mefA	mph(C)	vat(A)	vat(B)	vga(A)	vga(A) (BM 3327)	vgaB	vgaC	vgaD	vgaE	vgb	aacA-aphD	aadD	ant9		ble	aad6
	msrA	mefA	mph(C)	vat(A)	vat(B)	vga(A)	vga(A) (BM 3327)	vgaB	vgaC	vgaD	vgaE	vgb	aacA-aphD	aadD	ant9-C2944	ant9	ble	aad6
	energy-dependent efflux of erythro-mycin	macrolide efflux protein A	probable lysylphos-phatidyl-glycerol synthetase	virginia-mycin A acetyltransferase	acetyl-transferase inactivating streptogramin A	ATP binding protein, streptogramin-A-resistance		streptogramin A ABC transporter	streptogramin A ABC transporter	#NV	streptogramin A ABC transporter	virginia-mycin B hydrolase	bifunctional enzyme Aac/Aph, gentamicin resistance	amino-glycoside adenyl-transferase,tobramycin resistance	adenyltransferase AAd9		bleomycin resistance protein	aminoglycoside 6-adenylyltransferase
>"South-East Asian Clade": TW20 (Irish AR44), c																		
1195_SAUR (USA)	NEG	NEG	NEG	NEG	NEG	NEG	NEG	NEG	NEG	NEG	NEG	NEG	POS	NEG	NEG	POS	NEG	NEG
FVRH6002 (USA)	NEG	NEG	NEG	NEG	NEG	NEG	NEG	NEG	NEG	NEG	NEG	NEG	POS	NEG	NEG	NEG	NEG	NEG
KINW6048 (USA)	NEG	NEG	NEG	NEG	NEG	NEG	NEG	NEG	NEG	NEG	NEG	NEG	POS	NEG	NEG	POS	NEG	NEG
LAMC0011 (USA)	POS	NEG	NEG	NEG	NEG	NEG	NEG	NEG	NEG	NEG	NEG	NEG	POS	NEG	NEG	POS	NEG	NEG
SJOS6053 (USA)	POS	NEG	POS	NEG	NEG	NEG	NEG	NEG	NEG	NEG	NEG	NEG	POS	NEG	NEG	POS	NEG	NEG
SJOS6072 (USA)	NEG	NEG	NEG	NEG	NEG	NEG	NEG	NEG	NEG	NEG	NEG	NEG	POS	NEG	NEG	NEG	NEG	NEG
UCIM6042 (USA)	NEG	NEG	NEG	NEG	NEG	NEG	NEG	NEG	NEG	NEG	NEG	NEG	POS	NEG	NEG	POS	NEG	NEG
Perth_2015-S31902	NEG	NEG	NEG	NEG	NEG	NEG	NEG	NEG	NEG	NEG	NEG	NEG	NEG	NEG	NEG	POS	NEG	NEG
Perth_2015-S31904	NEG	NEG	NEG	NEG	NEG	NEG	NEG	NEG	NEG	NEG	NEG	NEG	POS	NEG	NEG	POS	NEG	NEG
Perth_2015-S31913	NEG	NEG	NEG	NEG	NEG	NEG	NEG	NEG	NEG	NEG	NEG	NEG	NEG	NEG	NEG	POS	NEG	NEG
Perth_2015-S31918	NEG	NEG	NEG	NEG	NEG	NEG	NEG	NEG	NEG	NEG	NEG	NEG	NEG	NEG	NEG	POS	NEG	NEG
Dublin-DSH_AR44_E1183	NEG	NEG	NEG	NEG	NEG	NEG	NEG	NEG	NEG	NEG	NEG	NEG	POS	POS	NEG	POS	NEG	NEG
Dublin-DSH_Unfamiliar-3_E1520	NEG	NEG	NEG	NEG	NEG	NEG	NEG	NEG	NEG	NEG	NEG	NEG	POS	NEG	NEG	POS	NEG	NEG
Hong Kong_042	NEG	NEG	NEG	NEG	NEG	NEG	NEG	NEG	NEG	NEG	NEG	NEG	POS	NEG	NEG	POS	NEG	NEG
Hong Kong_101	NEG	NEG	NEG	NEG	NEG	NEG	NEG	NEG	NEG	NEG	NEG	NEG	POS	NEG	NEG	POS	NEG	NEG
Hong Kong_111	NEG	NEG	NEG	NEG	NEG	NEG	NEG	NEG	NEG	NEG	NEG	NEG	POS	NEG	NEG	POS	NEG	NEG
Hong Kong_113	NEG	NEG	NEG	NEG	NEG	NEG	NEG	NEG	NEG	NEG	NEG	NEG	POS	NEG	NEG	POS	NEG	NEG
Hong Kong_115	NEG	NEG	NEG	NEG	NEG	NEG	NEG	NEG	NEG	NEG	NEG	NEG	POS	NEG	NEG	POS	NEG	NEG
Hong Kong_118	NEG	NEG	NEG	NEG	NEG	NEG	NEG	NEG	NEG	NEG	NEG	NEG	POS	NEG	NEG	POS	NEG	NEG
Hong Kong_125	NEG	NEG	NEG	NEG	NEG	NEG	NEG	NEG	NEG	NEG	NEG	NEG	POS	NEG	NEG	POS	NEG	NEG
Hong Kong_129	NEG	NEG	NEG	NEG	NEG	NEG	NEG	NEG	NEG	NEG	NEG	NEG	POS	NEG	NEG	POS	NEG	NEG
Hong Kong_133	NEG	NEG	NEG	NEG	NEG	NEG	NEG	NEG	NEG	NEG	NEG	NEG	POS	NEG	NEG	POS	NEG	NEG
Kuwait_009	NEG	NEG	NEG	NEG	NEG	NEG	NEG	NEG	NEG	NEG	NEG	NEG	POS	NEG	NEG	POS	NEG	NEG
Kuwait_103	NEG	NEG	NEG	NEG	NEG	NEG	NEG	NEG	NEG	NEG	NEG	NEG	POS	NEG	NEG	POS	NEG	NEG
Kuwait_107	NEG	NEG	NEG	NEG	NEG	NEG	NEG	NEG	NEG	NEG	NEG	NEG	POS	NEG	NEG	POS	NEG	NEG
Perth_03 AH 88	NEG	NEG	NEG	NEG	NEG	NEG	NEG	NEG	NEG	NEG	NEG	NEG	POS	NEG	NEG	POS	NEG	NEG
Perth_08-16905	NEG	NEG	NEG	NEG	NEG	NEG	NEG	NEG	NEG	NEG	NEG	NEG	POS	NEG	NEG	POS	NEG	NEG
Riyadh_Alfaisal-10_SICU_13_B5672_1117353	NEG	NEG	NEG	NEG	NEG	NEG	NEG	NEG	NEG	NEG	NEG	NEG	POS	NEG	NEG	POS	NEG	NEG
Riyadh_SSTI_17_3508602	NEG	NEG	NEG	NEG	NEG	NEG	NEG	NEG	NEG	NEG	NEG	NEG	POS	NEG	NEG	POS	NEG	NEG
Riyadh-2822088-R	NEG	NEG	NEG	NEG	NEG	NEG	NEG	NEG	NEG	NEG	NEG	NEG	POS	NEG	NEG	POS	NEG	NEG
Trinidad&Tobago_2012_195 (619/12)	NEG	NEG	NEG	NEG	NEG	NEG	NEG	NEG	NEG	NEG	NEG	NEG	POS	NEG	NEG	POS	NEG	NEG
Trinidad&Tobago_2012_370 (1112-52177)	NEG	NEG	NEG	NEG	NEG	NEG	NEG	NEG	NEG	NEG	NEG	NEG	POS	POS	NEG	POS	NEG	NEG
Trinidad&Tobago_2012_378 (4139)	NEG	NEG	NEG	NEG	NEG	NEG	NEG	NEG	NEG	NEG	NEG	NEG	POS	POS	NEG	POS	NEG	NEG
Trinidad&Tobago_2013_PA21	NEG	NEG	NEG	NEG	NEG	NEG	NEG	AMB	NEG	NEG	NEG	NEG	POS	NEG	NEG	POS	NEG	NEG
Trinidad&Tobago_2013_PA22	NEG	NEG	NEG	NEG	NEG	NEG	NEG	NEG	NEG	NEG	NEG	NEG	POS	NEG	NEG	POS	NEG	NEG
Trinidad&Tobago_2912_340 (8523/12)	NEG	NEG	NEG	NEG	NEG	NEG	NEG	NEG	NEG	NEG	NEG	NEG	POS	NEG	NEG	POS	NEG	NEG
Trinidad&Tobago_MRSA2010_105	NEG	NEG	NEG	NEG	NEG	NEG	NEG	AMB	NEG	NEG	NEG	NEG	POS	POS	NEG	POS	NEG	NEG
Trinidad&Tobago_MRSA2010_108	NEG	NEG	NEG	NEG	NEG	NEG	NEG	NEG	NEG	NEG	NEG	NEG	POS	NEG	NEG	POS	NEG	NEG
Trinidad&Tobago_MRSA2010_33	NEG	NEG	NEG	NEG	NEG	NEG	NEG	NEG	NEG	NEG	NEG	NEG	POS	POS	NEG	POS	NEG	NEG
Trinidad&Tobago_MRSA2010_79	NEG	NEG	NEG	NEG	NEG	NEG	NEG	NEG	NEG	NEG	NEG	NEG	POS	POS	NEG	POS	NEG	NEG
Trinidad&Tobago_MRSA2010_88	NEG	NEG	NEG	NEG	NEG	NEG	NEG	NEG	NEG	NEG	NEG	NEG	POS	NEG	NEG	POS	NEG	NEG
Trinidad&Tobago_MRSA2010_97	NEG	NEG	NEG	NEG	NEG	NEG	NEG	NEG	NEG	NEG	NEG	NEG	POS	NEG	NEG	POS	NEG	NEG
Trinidad&Tobago_SSTI 2012_031	NEG	NEG	NEG	NEG	NEG	NEG	NEG	AMB	NEG	NEG	NEG	NEG	POS	NEG	NEG	POS	NEG	NEG
Uganda-29_17148_250811	NEG	NEG	NEG	NEG	NEG	NEG	NEG	NEG	NEG	NEG	NEG	NEG	POS	NEG	NEG	POS	NEG	NEG
>"South-East Asian Clad													POS	NEG		POS	NEG	
UCIM6015	NEG	NEG	NEG	NEG	NEG	NEG	NEG	NEG	NEG	NEG	NEG	NEG	POS	NEG	NEG	POS	NEG	NEG
Hong Kong_121	NEG	NEG	NEG	NEG	NEG	NEG	NEG	NEG	NEG	NEG	NEG	NEG	POS	NEG	NEG	POS	NEG	NEG
>"South-East Asian Clad													POS	NEG		POS	NEG	
Z172 (Taiwan)	NEG	NEG	NEG	NEG	NEG	NEG	NEG	NEG	NEG	NEG	NEG	NEG	POS	NEG	NEG	POS	NEG	NEG
Russia-19_0342_Kurgan	NEG	NEG	NEG	NEG	NEG	NEG	NEG	NEG	NEG	NEG	NEG	NEG	POS	NEG	NEG	POS	NEG	NEG
>"South-East Asian Clad													POS	NEG		POS	NEG	
Perth_01-15392	NEG	NEG	NEG	NEG	NEG	NEG	NEG	NEG	NEG	NEG	NEG	NEG	POS	NEG	NEG	POS	NEG	NEG
>"South-East Asian Clad													POS	NEG		POS	NEG	
XN108 (China)	NEG	NEG	NEG	NEG	NEG	NEG	NEG	NEG	NEG	NEG	NEG	NEG	POS	NEG	NEG	POS	NEG	NEG
NMR09 (India)	NEG	NEG	NEG	NEG	NEG	NEG	NEG	NEG	NEG	NEG	NEG	NEG	POS	NEG	NEG	POS	NEG	NEG
346 (Malaysia)	NEG	NEG	NEG	NEG	NEG	NEG	NEG	NEG	NEG	NEG	NEG	NEG	POS	NEG	NEG	POS	NEG	NEG
DS_009 (Thailand)	NEG	NEG	NEG	NEG	NEG	NEG	NEG	NEG	NEG	NEG	NEG	NEG	POS	NEG	NEG	POS	NEG	NEG
18ANR581005 (Ind)	NEG	NEG	NEG	NEG	NEG	NEG	NEG	#ZAHL!	NEG	NEG	#ZAHL!	NEG	POS	NEG	NEG	POS	NEG	NEG
Bengalaru_ST20141405	NEG	NEG	NEG	NEG	NEG	NEG	NEG	NEG	NEG	NEG	NEG	NEG	POS	NEG	NEG	POS	NEG	NEG
Bengalaru_ST20141406	NEG	NEG	NEG	NEG	NEG	NEG	NEG	NEG	NEG	NEG	NEG	NEG	POS	NEG	NEG	POS	NEG	NEG
Bengalaru_ST20141417	NEG	NEG	NEG	NEG	NEG	NEG	NEG	NEG	NEG	NEG	NEG	NEG	POS	NEG	NEG	POS	NEG	NEG
Hong Kong_107	NEG	NEG	NEG	NEG	NEG	NEG	NEG	NEG	NEG	NEG	NEG	NEG	POS	NEG	NEG	POS	NEG	NEG
Hong Kong_12	NEG	NEG	NEG	NEG	NEG	NEG	NEG	NEG	NEG	NEG	NEG	NEG	POS	NEG	NEG	POS	NEG	NEG
Hong Kong_132	NEG	NEG	NEG	NEG	NEG	NEG	NEG	NEG	NEG	NEG	NEG	NEG	POS	NEG	NEG	POS	NEG	NEG
Riyadh-3028763-R	NEG	NEG	NEG	NEG	NEG	NEG	NEG	NEG	NEG	NEG	NEG	NEG	POS	NEG	NEG	POS	NEG	NEG
Trinidad&Tobago_MRSA2010_94	NEG	NEG	NEG	NEG	NEG	NEG	NEG	NEG	NEG	NEG	NEG	NEG	POS	NEG	NEG	POS	NEG	NEG
>"South-East Asian Clad													POS	NEG		POS	NEG	
M705 (Thailand)	NEG	NEG	NEG	NEG	NEG	NEG	NEG	NEG	NEG	NEG	NEG	NEG	POS	NEG	NEG	POS	NEG	NEG
S85 (Thailand)	NEG	NEG	NEG	NEG	NEG	NEG	NEG	NEG	NEG	NEG	NEG	NEG	POS	NEG	NEG	POS	NEG	NEG
Riyadh_Alfaisal-04_281014_S5076	NEG	NEG	NEG	NEG	NEG	NEG	NEG	NEG	NEG	NEG	NEG	NEG	POS	NEG	NEG	POS	NEG	NEG
Riyadh_Alfaisal-26_S15145_1104163	NEG	NEG	NEG	NEG	NEG	NEG	NEG	NEG	NEG	NEG	NEG	NEG	POS	NEG	NEG	POS	NEG	NEG
Riyadh-2817437-W	NEG	NEG	NEG	NEG	NEG	AMB	NEG	NEG	NEG	NEG	NEG	NEG	POS	NEG	NEG	POS	NEG	NEG
>"South-East Asian Clad													POS	NEG		POS	NEG	
PPUKM-775-2009 (Malaysia)	NEG	NEG	NEG	NEG	NEG	NEG	NEG	NEG	NEG	NEG	NEG	NEG	POS	NEG	NEG	POS	NEG	NEG
Hong Kong_138	NEG	NEG	NEG	NEG	NEG	NEG	NEG	NEG	NEG	NEG	NEG	NEG	POS	NEG	NEG	POS	NEG	NEG
>"South-East Asian Clad													POS	NEG		POS	NEG	
Kuwait_001	NEG	NEG	NEG	NEG	NEG	NEG	NEG	NEG	NEG	NEG	NEG	NEG	POS	NEG	NEG	POS	NEG	NEG
Kuwait_337	NEG	NEG	NEG	NEG	NEG	NEG	NEG	NEG	NEG	NEG	NEG	NEG	POS	NEG	NEG	POS	NEG	NEG
>"South-East Asian Clad													POS	RARE		POS	RARE	
CUHK_HK2007 (China)	NEG	NEG	NEG	NEG	NEG	NEG	NEG	NEG	NEG	NEG	NEG	NEG	POS	NEG	NEG	POS	NEG	NEG
NMR02 (India)	NEG	NEG	NEG	NEG	NEG	NEG	NEG	NEG	NEG	NEG	NEG	NEG	POS	NEG	NEG	POS	NEG	NEG
VB1490 (India)	NEG	NEG	NEG	NEG	NEG	NEG	NEG	NEG	NEG	NEG	NEG	NEG	POS	NEG	NEG	POS	NEG	NEG
V521 (Korea)	NEG	NEG	NEG	NEG	NEG	NEG	NEG	NEG	NEG	NEG	NEG	NEG	POS	NEG	NEG	POS	NEG	NEG
HST-077 (Lebanon)	NEG	NEG	NEG	NEG	NEG	NEG	NEG	NEG	NEG	NEG	NEG	NEG	POS	NEG	NEG	POS	NEG	NEG
MAL1 (Malaysia)	NEG	NEG	NEG	NEG	NEG	NEG	NEG	NEG	NEG	NEG	NEG	NEG	POS	NEG	NEG	POS	NEG	NEG
MAL3 (Malaysia)	NEG	NEG	NEG	NEG	NEG	NEG	NEG	NEG	NEG	NEG	NEG	NEG	POS	NEG	NEG	POS	NEG	NEG
PPUKM-261-2009 (Malaysia)	NEG	NEG	NEG	NEG	NEG	NEG	NEG	NEG	NEG	NEG	NEG	NEG	POS	NEG	NEG	POS	NEG	NEG
PPUKM-332-2009 (Malaysia)	NEG	NEG	NEG	NEG	NEG	NEG	NEG	NEG	NEG	NEG	NEG	NEG	POS	NEG	NEG	POS	NEG	NEG
S38 (Thailand)	NEG	NEG	NEG	NEG	NEG	NEG	NEG	NEG	NEG	NEG	NEG	NEG	POS	NEG	NEG	POS	NEG	NEG
S7 (Thailand)	NEG	NEG	NEG	NEG	NEG	NEG	NEG	NEG	NEG	NEG	NEG	NEG	POS	NEG	NEG	POS	NEG	NEG
US_008 (Thailand)	NEG	NEG	NEG	NEG	NEG	NEG	NEG	NEG	NEG	NEG	NEG	NEG	POS	NEG	NEG	POS	NEG	NEG
US_030 (Thailand)	NEG	NEG	NEG	NEG	NEG	NEG	NEG	NEG	NEG	NEG	NEG	NEG	POS	NEG	NEG	POS	NEG	NEG
M170 (Tsunami-related)	NEG	NEG	NEG	NEG	NEG	POS	NEG	NEG	NEG	NEG	NEG	NEG	POS	NEG	NEG	POS	NEG	NEG
BSAC697 (UK)	NEG	NEG	NEG	NEG	NEG	NEG	NEG	NEG	NEG	NEG	NEG	NEG	POS	NEG	NEG	POS	NEG	NEG
IS-189	NEG	NEG	NEG	NEG	NEG	NEG	NEG	NEG	NEG	NEG	NEG	NEG	POS	NEG	NEG	POS	NEG	NEG
MRGR3	NEG	NEG	NEG	NEG	NEG	NEG	NEG	NEG	NEG	NEG	NEG	NEG	POS	NEG	NEG	POS	NEG	NEG
smg07	NEG	NEG	NEG	NEG	NEG	NEG	NEG	NEG	NEG	NEG	NEG	NEG	POS	NEG	NEG	POS	NEG	NEG
AH1-AUS-EMRSA-3	NEG	NEG	NEG	NEG	NEG	NEG	NEG	NEG	NEG	NEG	NEG	NEG	POS	NEG	NEG	POS	NEG	NEG
Bengalaru_ST20141403	NEG	NEG	NEG	NEG	NEG	NEG	NEG	NEG	NEG	NEG	NEG	NEG	POS	NEG	NEG	POS	NEG	NEG
Finland E24_98541_Harmony	NEG	NEG	NEG	NEG	NEG	AMB	NEG	NEG	NEG	NEG	NEG	NEG	POS	NEG	NEG	POS	NEG	NEG
Hong Kong_10	NEG	NEG	NEG	NEG	NEG	NEG	NEG	NEG	NEG	NEG	NEG	NEG	POS	NEG	NEG	POS	NEG	NEG
Hong Kong_100	NEG	NEG	NEG	NEG	NEG	NEG	NEG	NEG	NEG	NEG	NEG	NEG	POS	NEG	NEG	POS	NEG	NEG
Hong Kong_199	NEG	NEG	NEG	NEG	NEG	NEG	NEG	NEG	NEG	NEG	NEG	NEG	POS	NEG	NEG	POS	NEG	NEG
Hong Kong_34	NEG	NEG	NEG	NEG	NEG	NEG	NEG	NEG	NEG	NEG	NEG	NEG	POS	NEG	NEG	POS	NEG	NEG
Hong Kong_88	NEG	NEG	NEG	NEG	NEG	NEG	NEG	NEG	NEG	NEG	NEG	NEG	POS	POS	NEG	POS	NEG	NEG
Hong Kong_92	NEG	NEG	NEG	NEG	NEG	NEG	NEG	NEG	NEG	NEG	NEG	NEG	POS	NEG	NEG	POS	NEG	NEG
Hong Kong_94	NEG	NEG	NEG	NEG	NEG	NEG	NEG	NEG	NEG	NEG	NEG	NEG	POS	POS	NEG	POS	POS	NEG
Kuwait_257	NEG	NEG	NEG	NEG	NEG	NEG	NEG	NEG	NEG	NEG	NEG	NEG	POS	NEG	NEG	POS	NEG	NEG
Perth_01-15330	NEG	NEG	NEG	NEG	NEG	NEG	NEG	NEG	NEG	NEG	NEG	NEG	POS	NEG	NEG	POS	NEG	NEG
Perth_01-15349	NEG	NEG	NEG	NEG	NEG	NEG	NEG	NEG	NEG	NEG	NEG	NEG	POS	NEG	NEG	POS	NEG	NEG
Perth_01-16176	NEG	NEG	NEG	NEG	NEG	NEG	NEG	NEG	NEG	NEG	NEG	NEG	POS	NEG	NEG	POS	NEG	NEG
Perth_WBG8379	NEG	NEG	NEG	NEG	NEG	NEG	NEG	NEG	NEG	NEG	NEG	NEG	POS	NEG	NEG	POS	NEG	NEG
Rawalpindi_Kidney Center_56	NEG	NEG	NEG	NEG	NEG	NEG	NEG	NEG	NEG	NEG	NEG	NEG	POS	NEG	NEG	POS	NEG	NEG
Riyadh_SSTI_05_3464614	NEG	NEG	NEG	NEG	NEG	NEG	NEG	NEG	NEG	NEG	NEG	NEG	POS	NEG	NEG	POS	NEG	NEG
Trinidad&Tobago_2012_157	NEG	NEG	NEG	NEG	NEG	NEG	NEG	NEG	NEG	NEG	NEG	NEG	POS	NEG	NEG	POS	NEG	NEG

STRAIN / ISOLATE	RESISTANCE : MLS-ANTIBIOTICS												RESISTANCE : AMINOGLYSOSIDES					
	msrA	mefA	mph(C)	vat(A)	vat(B)	vga(A)	vga(A) (BM 3327)	vgaB	vgaC	vgaD	vgaE	vgb	aacA-aphD	aadD	ant9		ble	aad6
	msrA	mefA	mph(C)	vat(A)	vat(B)	vga(A)	vga(A) (BM 3327)	vgaB	vgaC	vgaD	vgaE	vgb	aacA-aphD	aadD	ant9-C2944	ant9	ble	aad6
	energy-dependent efflux of erythro-mycin	macrolide efflux protein A	probable lysylphos-phatidyl-glycerol synthetase	virginia-mycin A acetyltransferase	acetyl-transferase inactivating streptogramin A	ATP binding protein, streptogramin-A-resistance		streptogramin A ABC transporter	streptogramin A ABC transporter	#NV	streptogramin A ABC transporter	virginia-mycin B hydrolase	bifunctional enzyme Aac/Aph, gentamicin resistance	amino-glycoside adenyl-transferase,tobramycin resistance	adenyltransferase AAd9		bleomycin resistance protein	aminoglycoside 6-adenylyltransferase
>"South-East Asian Clad													POS	NEG		POS	NEG	
M418	NEG	NEG	NEG	NEG	NEG	NEG	NEG	NEG	NEG	NEG	NEG	NEG	POS	NEG	NEG	POS	NEG	NEG
>"South-East Asian Clad													POS	NEG		POS	NEG	
NMR07 (India)	NEG	NEG	NEG	NEG	NEG	NEG	NEG	NEG	NEG	NEG	NEG	NEG	POS	NEG	NEG	POS	NEG	NEG
NMR08 (India)	NEG	NEG	NEG	NEG	NEG	NEG	NEG	NEG	NEG	NEG	NEG	NEG	POS	NEG	NEG	POS	NEG	NEG
Bengalaru_ST20121978	NEG	NEG	NEG	NEG	NEG	NEG	NEG	NEG	NEG	NEG	NEG	NEG	POS	NEG	NEG	POS	NEG	NEG
Hong Kong_89	NEG	NEG	NEG	NEG	NEG	NEG	NEG	NEG	NEG	NEG	NEG	NEG	POS	NEG	NEG	POS	NEG	NEG
>"South-East Asian Clad													POS	NEG		POS	NEG	
Perth_2005_AH_5	NEG	NEG	NEG	NEG	NEG	NEG	NEG	NEG	NEG	NEG	NEG	NEG	POS	NEG	NEG	POS	NEG	NEG
>"South-East Asian Clad													POS	NEG		POS	NEG	
Trinidad&Tobago_MRSA2010_103	NEG	NEG	NEG	NEG	NEG	NEG	NEG	NEG	NEG	NEG	NEG	NEG	POS	NEG	NEG	POS	NEG	NEG
Trinidad&Tobago_2013_PA25	NEG	NEG	NEG	NEG	NEG	NEG	NEG	NEG	NEG	NEG	NEG	NEG	POS	NEG	NEG	POS	NEG	NEG
Trinidad&Tobago_MRSA2010_110	NEG	NEG	NEG	NEG	NEG	NEG	NEG	NEG	NEG	NEG	NEG	NEG	POS	NEG	NEG	POS	NEG	NEG
Trinidad&Tobago_SSTI 2012_111	NEG	NEG	NEG	NEG	NEG	NEG	NEG	NEG	NEG	NEG	NEG	NEG	POS	NEG	NEG	POS	NEG	NEG
Trinidad&Tobago_MRSA2010_158	NEG	NEG	NEG	NEG	NEG	NEG	NEG	POS	NEG	NEG	NEG	NEG	POS	NEG	NEG	POS	NEG	NEG
>"South-East Asian Clad													POS	NEG		POS	NEG	
Kuwait_122	NEG	NEG	NEG	NEG	NEG	NEG	NEG	NEG	NEG	NEG	NEG	NEG	POS	NEG	NEG	POS	NEG	NEG
Kuwait_002	NEG	NEG	NEG	NEG	NEG	NEG	NEG	NEG	NEG	NEG	NEG	NEG	POS	NEG	NEG	POS	NEG	NEG
Kuwait_196	NEG	NEG	NEG	NEG	NEG	NEG	NEG	NEG	NEG	NEG	NEG	NEG	POS	NEG	NEG	POS	NEG	NEG
Kuwait_199	NEG	NEG	NEG	NEG	NEG	NEG	NEG	NEG	NEG	NEG	NEG	NEG	POS	NEG	NEG	POS	NEG	NEG
Hong Kong_136	NEG	NEG	NEG	NEG	NEG	NEG	NEG	NEG	NEG	NEG	NEG	NEG	POS	NEG	NEG	POS	NEG	NEG
>"South-East Asian Clad													POS	NEG		POS	NEG	
P32 (Poland)	NEG	NEG	NEG	NEG	NEG	NEG	NEG	NEG	NEG	NEG	NEG	NEG	POS	NEG	NEG	POS	NEG	NEG
>"South-East Asian Clad													POS	NEG		POS	NEG	
Perth_2015-S32313	NEG	NEG	NEG	NEG	NEG	NEG	NEG	NEG	NEG	NEG	NEG	NEG	POS	NEG	NEG	POS	NEG	NEG
>"hla-negative South-Ea													POS	NEG		POS	NEG	
DEN907 (Denmark)	NEG	NEG	NEG	NEG	NEG	NEG	NEG	NEG	NEG	NEG	NEG	NEG	POS	NEG	NEG	POS	NEG	NEG
>"hla-negative South-Ea													POS	VAR		POS	NEG	
S26 (Thailand)	NEG	NEG	NEG	NEG	NEG	NEG	NEG	NEG	NEG	NEG	NEG	NEG	POS	NEG	NEG	POS	NEG	NEG
S42 (Thailand)	NEG	NEG	NEG	NEG	NEG	NEG	NEG	NEG	NEG	NEG	NEG	NEG	POS	NEG	NEG	POS	NEG	NEG
S97 (Thailand)	NEG	NEG	NEG	NEG	NEG	NEG	NEG	NEG	NEG	NEG	NEG	NEG	POS	POS	NEG	POS	NEG	NEG
S78 (Thailand)	NEG	NEG	NEG	NEG	NEG	NEG	NEG	NEG	NEG	NEG	NEG	NEG	POS	NEG	NEG	POS	NEG	NEG
S25 (Thailand)	NEG	NEG	NEG	NEG	NEG	NEG	NEG	NEG	NEG	NEG	NEG	NEG	POS	POS	NEG	POS	NEG	NEG
S21 (Thailand)	NEG	NEG	NEG	NEG	NEG	NEG	NEG	NEG	NEG	NEG	NEG	NEG	POS	NEG	NEG	POS	NEG	NEG
S106 (Thailand)	NEG	NEG	NEG	NEG	NEG	NEG	NEG	NEG	NEG	NEG	NEG	NEG	POS	NEG	NEG	POS	NEG	NEG
S24 (Thailand)	NEG	NEG	NEG	NEG	NEG	NEG	NEG	NEG	NEG	NEG	NEG	NEG	POS	NEG	NEG	POS	NEG	NEG
S39 (Thailand)	NEG	NEG	NEG	NEG	NEG	NEG	NEG	NEG	NEG	NEG	NEG	NEG	POS	NEG	NEG	POS	NEG	NEG
S2 (Thailand)	NEG	NEG	NEG	NEG	NEG	NEG	NEG	NEG	NEG	NEG	NEG	NEG	POS	POS	NEG	POS	NEG	NEG
S81 (Thailand)	NEG	NEG	NEG	NEG	NEG	NEG	NEG	NEG	NEG	NEG	NEG	NEG	POS	NEG	NEG	POS	NEG	NEG
M116 (Vietnam)	NEG	NEG	NEG	NEG	NEG	NEG	NEG	NEG	NEG	NEG	NEG	NEG	POS	NEG	NEG	POS	NEG	NEG
>"Portuguese Clade": Pc													COMM	RARE		POS	RARE	
FFP103 (Portugal)	NEG	NEG	NEG	NEG	NEG	NEG	NEG	NEG	NEG	NEG	NEG	NEG	POS	NEG	NEG	POS	NEG	NEG
HDG2 (Portugal)	NEG	NEG	NEG	NEG	NEG	NEG	NEG	NEG	NEG	NEG	NEG	NEG	POS	NEG	NEG	POS	NEG	NEG
HSA10 (Portugal)	NEG	NEG	NEG	NEG	NEG	NEG	NEG	NEG	NEG	NEG	NEG	NEG	POS	NEG	NEG	POS	NEG	NEG
HSA11 (Portugal)	NEG	NEG	NEG	NEG	NEG	NEG	NEG	NEG	NEG	NEG	NEG	NEG	POS	NEG	NEG	POS	NEG	NEG
ICP5011 (Portugal)	NEG	NEG	NEG	NEG	NEG	NEG	NEG	NEG	NEG	NEG	NEG	NEG	POS	NEG	NEG	POS	NEG	NEG
ICP5014 (Portugal)	NEG	NEG	NEG	NEG	NEG	NEG	NEG	NEG	NEG	NEG	NEG	NEG	POS	NEG	NEG	POS	NEG	NEG
ICP5062 (Portugal)	NEG	NEG	NEG	NEG	NEG	NEG	NEG	NEG	NEG	NEG	NEG	NEG	POS	NEG	NEG	POS	NEG	NEG
Strain 1063_ATCC 33592 FDAARGOS_3 (USA)	NEG	NEG	NEG	NEG	NEG	NEG	NEG	NEG	NEG	NEG	NEG	NEG	NEG	NEG	NEG	POS	NEG	NEG
	NEG	NEG	NEG	NEG	NEG	NEG	NEG	NEG	NEG	NEG	NEG	NEG	POS	POS	NEG	POS	POS	NEG
Russia-07_0110_Kurgan	NEG	NEG	NEG	NEG	NEG	NEG	NEG	NEG	NEG	NEG	NEG	NEG	POS	NEG	NEG	POS	NEG	NEG
Russia-11_0164_Kurgan	NEG	NEG	NEG	NEG	NEG	NEG	NEG	NEG	NEG	NEG	NEG	NEG	POS	NEG	NEG	POS	NEG	NEG
Russia-20_0367_Kurgan	NEG	NEG	NEG	NEG	NEG	NEG	NEG	NEG	NEG	NEG	NEG	NEG	POS	NEG	NEG	POS	NEG	NEG
Russia-21_0390_Chelyabinsk	NEG	NEG	NEG	NEG	NEG	NEG	NEG	NEG	NEG	NEG	NEG	NEG	POS	NEG	NEG	POS	NEG	NEG
>"South American/ Mid													COMM	NEG		POS	NEG	
AGT1 (Argentina)	NEG	NEG	NEG	NEG	NEG	NEG	NEG	NEG	NEG	NEG	NEG	NEG	POS	NEG	NEG	POS	NEG	NEG
AGT120 (Argentina)	NEG	NEG	NEG	NEG	NEG	NEG	NEG	NEG	NEG	NEG	NEG	NEG	POS	NEG	NEG	POS	NEG	NEG
AGT67 (Argentina)	NEG	NEG	NEG	NEG	NEG	NEG	NEG	NEG	NEG	NEG	NEG	NEG	POS	NEG	NEG	POS	NEG	NEG
AGT9 (Argentina)	NEG	NEG	NEG	NEG	NEG	NEG	NEG	NEG	NEG	NEG	NEG	NEG	POS	NEG	NEG	POS	NEG	NEG
RA3 (Argentina)	NEG	NEG	NEG	NEG	NEG	NEG	NEG	NEG	NEG	NEG	NEG	NEG	POS	NEG	NEG	POS	NEG	NEG
RA6 (Argentina)	NEG	NEG	NEG	NEG	NEG	NEG	NEG	NEG	NEG	NEG	NEG	NEG	POS	NEG	NEG	POS	NEG	NEG
RA7 (Argentina)	NEG	NEG	NEG	NEG	NEG	NEG	NEG	NEG	NEG	NEG	NEG	NEG	POS	NEG	NEG	POS	NEG	NEG
Be62 (Brazil)	NEG	NEG	NEG	NEG	NEG	NEG	NEG	NEG	NEG	NEG	NEG	NEG	POS	NEG	NEG	POS	NEG	NEG
Bmb9393 (Brazil)	NEG	NEG	NEG	NEG	NEG	NEG	NEG	NEG	NEG	NEG	NEG	NEG	POS	NEG	NEG	POS	NEG	NEG
BRA36 (Brazil)	NEG	NEG	NEG	NEG	NEG	NEG	NEG	NEG	NEG	NEG	NEG	NEG	POS	NEG	NEG	POS	NEG	NEG
BZ48 (Brazil)	NEG	NEG	NEG	NEG	NEG	NEG	NEG	NEG	NEG	NEG	NEG	NEG	POS	NEG	NEG	POS	NEG	NEG
Gv51 (Brazil)	NEG	NEG	NEG	NEG	NEG	NEG	NEG	NEG	NEG	NEG	NEG	NEG	POS	NEG	NEG	POS	NEG	NEG
Gv69 (Brazil)	NEG	NEG	NEG	NEG	NEG	NEG	NEG	NEG	NEG	NEG	NEG	NEG	POS	NEG	NEG	POS	NEG	NEG
Gv88 (Brazil)	NEG	NEG	NEG	NEG	NEG	NEG	NEG	NEG	NEG	NEG	NEG	NEG	POS	NEG	NEG	POS	NEG	NEG
HC1335 (Brazil)	NEG	NEG	NEG	NEG	NEG	NEG	NEG	NEG	NEG	NEG	NEG	NEG	NEG	NEG	NEG	POS	NEG	NEG
HC1340 (Brazil)	NEG	NEG	NEG	NEG	NEG	NEG	NEG	NEG	NEG	NEG	NEG	NEG	POS	NEG	NEG	POS	NEG	NEG
HC556 (Brazil)	NEG	NEG	NEG	NEG	NEG	NEG	NEG	NEG	NEG	NEG	NEG	NEG	POS	NEG	NEG	POS	NEG	NEG
HU25 (Brazil)	NEG	NEG	NEG	NEG	NEG	NEG	NEG	NEG	NEG	NEG	NEG	NEG	POS	NEG	NEG	POS	NEG	NEG
UB563 (Brazil)	NEG	NEG	NEG	NEG	NEG	NEG	NEG	NEG	NEG	NEG	NEG	NEG	POS	NEG	NEG	POS	NEG	NEG
2A8 (Czech Republic)	NEG	NEG	NEG	NEG	NEG	NEG	NEG	NEG	NEG	NEG	NEG	NEG	POS	NEG	NEG	POS	NEG	NEG
M1229 (Denmark)	NEG	NEG	NEG	NEG	NEG	NEG	NEG	NEG	NEG	NEG	NEG	NEG	NEG	NEG	NEG	POS	NEG	NEG
FRICAR (France)	NEG	NEG	NEG	NEG	NEG	NEG	NEG	NEG	NEG	NEG	NEG	NEG	POS	NEG	NEG	POS	NEG	NEG
D90 (Germany)	NEG	NEG	NEG	NEG	NEG	NEG	NEG	NEG	NEG	NEG	NEG	NEG	NEG	NEG	NEG	POS	NEG	NEG
LIT68 (Lithuania)	NEG	NEG	NEG	NEG	NEG	NEG	NEG	NEG	NEG	NEG	NEG	NEG	POS	NEG	NEG	POS	NEG	NEG
LIT76 (Lithuania)	NEG	NEG	NEG	NEG	NEG	NEG	NEG	NEG	NEG	NEG	NEG	NEG	POS	NEG	NEG	POS	NEG	NEG
HGSA142 (Portugal)	NEG	NEG	NEG	NEG	NEG	NEG	NEG	NEG	NEG	NEG	NEG	NEG	POS	NEG	NEG	POS	NEG	NEG
HGSA9 (Portugal)	NEG	NEG	NEG	NEG	NEG	NEG	NEG	NEG	NEG	NEG	NEG	NEG	POS	NEG	NEG	POS	NEG	NEG
HSJ216 (Portugal)	NEG	NEG	NEG	NEG	NEG	NEG	NEG	NEG	NEG	NEG	NEG	NEG	POS	NEG	NEG	POS	NEG	NEG
M278 (Portugal)	NEG	NEG	NEG	NEG	NEG	NEG	NEG	NEG	NEG	NEG	NEG	NEG	POS	NEG	NEG	POS	NEG	NEG
ES26 (Spain)	NEG	NEG	NEG	NEG	NEG	NEG	NEG	NEG	NEG	NEG	NEG	NEG	POS	NEG	NEG	POS	NEG	NEG
UK102 (UK)	NEG	NEG	NEG	NEG	NEG	NEG	NEG	NEG	NEG	NEG	NEG	NEG	NEG	NEG	NEG	POS	NEG	NEG
URU34 (Uruguay)	NEG	NEG	NEG	NEG	NEG	NEG	NEG	NEG	NEG	NEG	NEG	NEG	POS	NEG	NEG	POS	NEG	NEG
IS-125	NEG	NEG	NEG	NEG	NEG	NEG	NEG	NEG	NEG	NEG	NEG	NEG	POS	NEG	NEG	POS	NEG	NEG
IS-157	NEG	NEG	NEG	NEG	NEG	NEG	NEG	NEG	NEG	NEG	NEG	NEG	POS	NEG	NEG	POS	NEG	NEG
Ecuador_1	NEG	NEG	NEG	NEG	NEG	NEG	NEG	NEG	NEG	NEG	NEG	NEG	POS	NEG	NEG	POS	NEG	NEG
Ecuador_2	NEG	NEG	NEG	NEG	NEG	NEG	NEG	NEG	NEG	NEG	NEG	NEG	POS	NEG	NEG	POS	NEG	NEG
Dresden_15ANR574263	NEG	NEG	NEG	NEG	NEG	NEG	NEG	NEG	NEG	NEG	NEG	NEG	POS	NEG	NEG	POS	NEG	NEG
UK-EMRSA-11	NEG	NEG	NEG	NEG	NEG	NEG	NEG	NEG	NEG	NEG	NEG	NEG	POS	NEG	NEG	POS	NEG	NEG
>"South American/ Mid													POS	NEG		POS	NEG	
BRA2 (Brazil)	NEG	NEG	NEG	NEG	NEG	NEG	NEG	NEG	NEG	NEG	NEG	NEG	POS	NEG	NEG	POS	NEG	NEG
CHL1 (Chile)	NEG	NEG	NEG	NEG	NEG	NEG	NEG	NEG	NEG	NEG	NEG	NEG	POS	NEG	NEG	POS	NEG	NEG
CHL151 (Chile)	NEG	NEG	NEG	NEG	NEG	NEG	NEG	NEG	NEG	NEG	NEG	NEG	POS	NEG	NEG	POS	NEG	NEG
UCL378 (Chile)	NEG	NEG	NEG	NEG	NEG	NEG	NEG	NEG	NEG	NEG	NEG	NEG	POS	NEG	NEG	POS	NEG	NEG
UP72 (Peru)	NEG	NEG	NEG	NEG	NEG	NEG	NEG	NEG	NEG	NEG	NEG	NEG	POS	NEG	NEG	POS	NEG	NEG
UP81 (Peru)	NEG	NEG	NEG	NEG	NEG	NEG	NEG	NEG	NEG	NEG	NEG	NEG	POS	NEG	NEG	POS	NEG	NEG
>"South American/ Mid													POS	NEG		POS	NEG	
UP1073 (Peru)	NEG	NEG	NEG	NEG	NEG	NEG	NEG	NEG	NEG	NEG	NEG	NEG	POS	NEG	NEG	POS	NEG	NEG

STRAIN / ISOLATE	RESISTANCE : MLS-ANTIBIOTICS												RESISTANCE : AMINOGLYSOSIDES					
	msrA	mefA	mph(C)	vat(A)	vat(B)	vga(A)	vga(A) (BM 3327)	vgaB	vgaC	vgaD	vgaE	vgb	aacA-aphD	aadD	ant9		ble	aad6
	msrA	mefA	mph(C)	vat(A)	vat(B)	vga(A)	vga(A) (BM 3327)	vgaB	vgaC	vgaD	vgaE	vgb	aacA-aphD	aadD	ant9-C2944	ant9	ble	aad6
	energy-dependent efflux of erythro-mycin	macrolide efflux protein A	probable lysylphos-phatidyl-glycerol synthetase	virginia-mycin A acetyltransferase	acetyl-transferase inactivating streptogramin A	ATP binding protein, streptogramin-A-resistance		streptogramin A ABC transporter	streptogramin A ABC transporter	#NV	streptogramin A ABC transporter	virginia-mycin B hydrolase	bifunctional enzyme Aac/Aph, gentamicin resistance	amino-glycoside adenyl-transferase,tobramy cin resistance	adenyltransferase AAd9		bleomycin resistance protein	aminoglycoside 6-adenylyltransferase
>"South American/ Mid													VAR	NEG		POS	NEG	
LIT89 (Lithuania)	NEG	NEG	NEG	NEG	NEG	NEG	NEG	NEG	NEG	NEG	NEG	NEG	POS	NEG	NEG	POS	NEG	NEG
MRSA_PR1 (Malaysia)	NEG	NEG	NEG	NEG	NEG	NEG	NEG	NEG	NEG	NEG	NEG	NEG	NEG	NEG	NEG	POS	NEG	NEG
Riyadh-2888905-R	NEG	NEG	NEG	NEG	NEG	NEG	NEG	NEG	NEG	NEG	NEG	NEG	NEG	NEG	NEG	POS	NEG	NEG
Riyadh-2888915-BC	NEG	NEG	NEG	NEG	NEG	NEG	NEG	NEG	NEG	NEG	NEG	NEG	NEG	NEG	NEG	POS	NEG	NEG
Riyadh-2793706-R	NEG	NEG	NEG	NEG	NEG	NEG	NEG	NEG	NEG	NEG	NEG	NEG	NEG	NEG	NEG	POS	NEG	NEG
>"South American/ Mid													NEG	NEG		POS	NEG	
Riyadh_SSTI_18_3502925	NEG	NEG	NEG	NEG	NEG	AMB	NEG	NEG	NEG	NEG	NEG	NEG	NEG	NEG	NEG	POS	NEG	NEG
Riyadh-2822825-W	NEG	NEG	NEG	NEG	NEG	AMB	NEG	NEG	NEG	NEG	NEG	NEG	NEG	NEG	NEG	POS	NEG	NEG
>"South American/ Mid													POS	NEG		POS	NEG	
UK-EMRSA-9	NEG	NEG	NEG	NEG	NEG	NEG	NEG	NEG	NEG	NEG	NEG	NEG	POS	NEG	NEG	POS	NEG	NEG
>"South American/ Mid													NEG	NEG		POS	NEG	
Lome_HT20020815	NEG	NEG	NEG	NEG	NEG	NEG	NEG	NEG	NEG	NEG	NEG	NEG	NEG	NEG	NEG	POS	NEG	NEG
>"South American/ Mid													VAR	RARE		COMM	RARE	
NA32 (Denmark)	NEG	NEG	NEG	NEG	NEG	NEG	NEG	NEG	NEG	NEG	NEG	NEG	NEG	NEG	NEG	POS	NEG	NEG
H24 (Egypt)	NEG	NEG	NEG	NEG	NEG	NEG	NEG	NEG	NEG	NEG	NEG	NEG	POS	NEG	NEG	POS	NEG	NEG
ATCC BAA-39 (=HUSA304) (Hungary)	NEG	NEG	NEG	NEG	NEG	NEG	NEG	NEG	NEG	NEG	NEG	NEG	POS	NEG	NEG	POS	NEG	NEG
HUSA304 (Hungary)	NEG	NEG	NEG	NEG	NEG	NEG	NEG	NEG	NEG	NEG	NEG	NEG	POS	NEG	NEG	POS	NEG	NEG
HU106 (Hungary)	NEG	NEG	NEG	NEG	NEG	NEG	NEG	NEG	NEG	NEG	NEG	NEG	POS	NEG	NEG	POS	NEG	NEG
BSAC27 (UK)	NEG	NEG	NEG	NEG	NEG	NEG	NEG	NEG	NEG	NEG	NEG	NEG	NEG	NEG	NEG	POS	NEG	NEG
NCTC13131, UK-EMRSA-4 (UK)	NEG	NEG	NEG	NEG	NEG	NEG	NEG	NEG	NEG	NEG	NEG	NEG	NEG	NEG	NEG	POS	NEG	NEG
	NEG	NEG	NEG	NEG	NEG	NEG	NEG	AMB	NEG	NEG	NEG	NEG	NEG	NEG	NEG	POS	NEG	NEG
Algiers_HT20040080	NEG	NEG	NEG	NEG	NEG	NEG	NEG	NEG	NEG	NEG	NEG	NEG	POS	NEG	NEG	NEG	NEG	NEG
Dublin-DSH_AR09_0.0066	NEG	NEG	NEG	NEG	NEG	NEG	NEG	NEG	NEG	NEG	NEG	NEG	POS	NEG	NEG	POS	NEG	NEG
Dublin-DSH_AR09_0-0065	NEG	NEG	NEG	NEG	NEG	NEG	NEG	NEG	NEG	NEG	NEG	NEG	POS	NEG	NEG	POS	NEG	NEG
Dublin-DSH_Phenotype-III_84	NEG	NEG	NEG	NEG	NEG	NEG	NEG	NEG	NEG	NEG	NEG	NEG	POS	NEG	NEG	POS	NEG	NEG
Hong Kong_130	NEG	NEG	NEG	NEG	NEG	NEG	NEG	NEG	NEG	NEG	NEG	NEG	NEG	NEG	NEG	POS	NEG	NEG
Kuwait_018	NEG	NEG	NEG	NEG	NEG	NEG	NEG	NEG	NEG	NEG	NEG	NEG	NEG	NEG	NEG	POS	NEG	NEG
Perth_08-17726	NEG	NEG	NEG	NEG	NEG	NEG	NEG	NEG	NEG	NEG	NEG	NEG	NEG	NEG	NEG	POS	NEG	NEG
Riyadh_Alfaisal/KKKSUH_86_MRS-14-279	NEG	NEG	NEG	NEG	NEG	NEG	NEG	NEG	NEG	NEG	NEG	NEG	NEG	NEG	NEG	POS	NEG	NEG
Riyadh_Alfaisal-04_Z3861_831588	NEG	NEG	NEG	NEG	NEG	NEG	NEG	NEG	NEG	NEG	NEG	NEG	NEG	NEG	NEG	POS	NEG	NEG
Riyadh_Alfaisal-30_S15724_1108013	NEG	NEG	NEG	NEG	NEG	NEG	NEG	NEG	NEG	NEG	NEG	NEG	NEG	NEG	NEG	POS	NEG	NEG
Riyadh_Alfaisal-6_22A_13_B3992_397721	NEG	NEG	NEG	NEG	NEG	NEG	NEG	NEG	NEG	NEG	NEG	NEG	NEG	NEG	NEG	POS	NEG	NEG
Riyadh_SSTI_52_3615482	NEG	NEG	NEG	NEG	NEG	NEG	NEG	NEG	NEG	NEG	NEG	NEG	NEG	NEG	NEG	POS	NEG	NEG
Riyadh-2817276-2	NEG	NEG	NEG	NEG	NEG	NEG	NEG	NEG	NEG	NEG	NEG	NEG	NEG	NEG	NEG	POS	NEG	NEG
Riyadh-2891670-W	NEG	NEG	NEG	NEG	NEG	AMB	NEG	NEG	NEG	NEG	NEG	NEG	NEG	NEG	NEG	POS	NEG	NEG
Riyadh-3006920-W	NEG	NEG	NEG	NEG	NEG	NEG	NEG	NEG	NEG	NEG	NEG	NEG	NEG	NEG	NEG	POS	NEG	NEG
Riyadh-R2567782	NEG	NEG	NEG	NEG	NEG	NEG	NEG	POS	NEG	NEG	NEG	NEG	NEG	NEG	NEG	POS	NEG	NEG
Russia-18_0252_Krasnoyarsk_SK2	NEG	NEG	NEG	NEG	NEG	NEG	NEG	NEG	NEG	NEG	NEG	NEG	NEG	POS	NEG	POS	POS	NEG
UK-EMRSA-7	NEG	NEG	NEG	NEG	NEG	NEG	NEG	POS	NEG	NEG	NEG	NEG	NEG	NEG	NEG	POS	NEG	NEG
>"South American/ Mid													POS	NEG		POS	NEG	
Dublin-DSH_AR23_0073	NEG	NEG	NEG	NEG	NEG	NEG	NEG	NEG	NEG	NEG	NEG	NEG	POS	NEG	NEG	POS	NEG	NEG
>Related to "South Ame													POS	POS		POS	VAR	
MRSA-OC3 (Russia)	NEG	NEG	NEG	NEG	NEG	NEG	NEG	NEG	NEG	NEG	NEG	NEG	POS	POS	NEG	POS	POS	NEG
Russia-12_0176_Krasnoyarsk	NEG	NEG	NEG	NEG	NEG	NEG	NEG	NEG	NEG	NEG	NEG	NEG	POS	POS	NEG	POS	POS	NEG
Russia-13_0180_Krasnoyarsk	NEG	NEG	NEG	NEG	NEG	NEG	NEG	NEG	NEG	NEG	NEG	NEG	POS	POS	NEG	POS	POS	NEG
Russia-16_0249_Krasnoyarsk	NEG	NEG	NEG	NEG	NEG	NEG	NEG	NEG	NEG	NEG	NEG	NEG	POS	POS	NEG	POS	POS	NEG
Russia-17_0250_Krasnoyarsk	NEG	NEG	NEG	NEG	NEG	NEG	NEG	NEG	NEG	NEG	NEG	NEG	POS	POS	NEG	POS	AMB	NEG
>Related to "South Ame													POS	POS		POS	POS	
URU110 (Uruguay)	NEG	NEG	NEG	NEG	NEG	NEG	NEG	NEG	NEG	NEG	NEG	NEG	POS	POS	NEG	POS	POS	NEG
>Related to "South Ame													NEG	NEG		POS	NEG	
DS_014 (Thailand)	NEG	NEG	NEG	NEG	NEG	NEG	NEG	NEG	NEG	NEG	NEG	NEG	NEG	NEG	NEG	POS	NEG	NEG
>Unassigned Middle Eas													POS	NEG		NEG	NEG	
Frankfurt_Oder_0490031797 (Libya)	NEG	NEG	NEG	NEG	NEG	NEG	NEG	NEG	NEG	NEG	NEG	NEG	POS	NEG	NEG	NEG	NEG	NEG
Kuwait_192	NEG	NEG	NEG	NEG	NEG	AMB	NEG	NEG	NEG	NEG	NEG	NEG	POS	NEG	NEG	NEG	NEG	NEG
Riyadh-9	NEG	NEG	NEG	NEG	NEG	NEG	NEG	NEG	NEG	NEG	NEG	NEG	POS	NEG	NEG	NEG	NEG	NEG
Russia-24_0407_Moscow	NEG	NEG	NEG	NEG	NEG	NEG	NEG	NEG	NEG	NEG	NEG	NEG	POS	NEG	NEG	NEG	NEG	NEG

STRAIN / ISOLATE	RESISTANCE : AMINOGLYSOSIDES				RESISTANCE : MISCELLANEOUS GENES													
	aadA		aadE	aphA3	sat	dfrA	dfrG				far1	mupA	mupB	tetK	tetL	tetM		
	aadA-var1	aadA-var2	aadE	aphA3	sat	dfrA	dfrG	dfrG-Tn559	dfrG-K	dfrG-D	far1	mupA	mupB	tetK	tetL	tetM (com-bined)	tetM-O	tetM-S
	aminoglycoside-3'-adenylyltransferase			3'5'-aminog-lycoside phospho-transferase, neo-/ kanamycin resistance	strepto-thricine-acetyl-transferase	dihydro-folate reductase type 1	dihydrofolate reductase				fusidic acid resistence	mupirocin resistence protein	plasmidic isoleucyl-tRNA synthase	tetracycline resistance	Tetracycline resistance gene	tetracycline resistance		
**>"Eurasian Clade": TUR1**			**NEG**	**NEG**		**NEG**	**NEG**					**NEG**		**POS**		**POS**		
TUR1 (Turkey)	NEG	NEG	NEG	NEG	NEG	NEG	NEG	NEG	NEG	NEG	NEG	NEG	NEG	POS	NEG	POS	NEG	NEG
TUR9 (Turkey)	NEG	NEG	NEG	NEG	NEG	NEG	NEG	NEG	NEG	NEG	NEG	NEG	NEG	POS	NEG	POS	NEG	NEG
**>"Eurasian Clade" : T013**			**NEG**	**NEG**		**NEG**	**NEG**					**NEG**		**NEG**		**POS**		
T0131 (China)	NEG	NEG	NEG	NEG	NEG	NEG	NEG	NEG	NEG	NEG	NEG	NEG	NEG	NEG	NEG	POS	NEG	NEG
MU4 (Turkey)	NEG	NEG	NEG	NEG	NEG	NEG	NEG	NEG	NEG	NEG	NEG	NEG	NEG	NEG	NEG	POS	NEG	NEG
DEU11 (Turkey)	NEG	NEG	NEG	NEG	NEG	NEG	NEG	NEG	NEG	NEG	NEG	NEG	NEG	NEG	NEG	POS	NEG	NEG
DEU16 (Turkey)	NEG	NEG	NEG	NEG	NEG	NEG	NEG	NEG	NEG	NEG	NEG	NEG	NEG	NEG	NEG	POS	NEG	NEG
Romania_Iasi_MRSA-07_54	NEG	NEG	NEG	NEG	NEG	NEG	NEG	NEG	NEG	NEG	NEG	NEG	NEG	NEG	NEG	POS	NEG	NEG
**>"Eurasian Clade": arsC-**			**NEG**	**NEG**		**NEG**	**NEG**					**NEG**		**NEG**		**POS**		
Romania_Iasi_MRSA-05_46	NEG	NEG	NEG	NEG	NEG	NEG	NEG	NEG	NEG	NEG	NEG	NEG	NEG	NEG	NEG	POS	NEG	NEG
Romania_Iasi_MRSA-06_47	NEG	NEG	NEG	NEG	NEG	NEG	NEG	NEG	NEG	NEG	NEG	NEG	NEG	NEG	NEG	POS	NEG	NEG
**>"Eurasian Clade": 16K/**			**NEG**	**NEG**		**RARE**	**NEG**					**RARE**		**NEG**		**POS**		
CN79 (China)	NEG	NEG	NEG	NEG	NEG	NEG	NEG	NEG	NEG	NEG	NEG	NEG	NEG	NEG	NEG	POS	NEG	NEG
CUHK_BJ2002 (China)	NEG	NEG	NEG	NEG	NEG	NEG	NEG	NEG	NEG	NEG	NEG	NEG	NEG	NEG	NEG	POS	NEG	NEG
CUHK_BJ2007 (China)	NEG	NEG	NEG	NEG	NEG	NEG	NEG	NEG	NEG	NEG	NEG	NEG	NEG	NEG	NEG	POS	NEG	NEG
3HK (Czech Republic)	NEG	NEG	NEG	NEG	NEG	NEG	NEG	NEG	NEG	NEG	NEG	NEG	NEG	NEG	NEG	POS	NEG	NEG
HU109 (Hungary)	NEG	NEG	NEG	NEG	NEG	NEG	NEG	NEG	NEG	NEG	NEG	NEG	NEG	NEG	NEG	POS	NEG	NEG
HUR18 (Hungary)	NEG	NEG	NEG	NEG	NEG	NEG	NEG	NEG	NEG	NEG	NEG	NEG	NEG	NEG	NEG	POS	NEG	NEG
H482 (Romania)	NEG	NEG	NEG	NEG	NEG	NEG	NEG	NEG	NEG	NEG	NEG	NEG	NEG	NEG	NEG	POS	NEG	NEG
16K (Russia)	NEG	NEG	NEG	NEG	NEG	NEG	NEG	NEG	NEG	NEG	NEG	NEG	NEG	NEG	NEG	POS	NEG	NEG
DEU10 (Turkey)	NEG	NEG	NEG	NEG	NEG	NEG	NEG	NEG	NEG	NEG	NEG	NEG	NEG	NEG	NEG	POS	NEG	NEG
DEU12 (Turkey)	NEG	NEG	NEG	NEG	NEG	NEG	NEG	NEG	NEG	NEG	NEG	NEG	NEG	NEG	NEG	POS	NEG	NEG
DEU14 (Turkey)	NEG	NEG	NEG	NEG	NEG	NEG	NEG	NEG	NEG	NEG	NEG	NEG	NEG	NEG	NEG	POS	NEG	NEG
DEU15 (Turkey)	NEG	NEG	NEG	NEG	NEG	NEG	NEG	NEG	NEG	NEG	NEG	NEG	NEG	NEG	NEG	POS	NEG	NEG
DEU17 (Turkey)	NEG	NEG	NEG	NEG	NEG	NEG	NEG	NEG	NEG	NEG	NEG	NEG	NEG	NEG	NEG	POS	NEG	NEG
DEU19 (Turkey)	NEG	NEG	NEG	NEG	NEG	NEG	NEG	NEG	NEG	NEG	NEG	NEG	NEG	NEG	NEG	POS	NEG	NEG
DEU2 (Turkey)	NEG	NEG	NEG	NEG	NEG	NEG	NEG	NEG	NEG	NEG	NEG	POS	NEG	NEG	NEG	POS	NEG	NEG
DEU20 (Turkey)	NEG	NEG	NEG	NEG	NEG	NEG	NEG	NEG	NEG	NEG	NEG	NEG	NEG	NEG	NEG	POS	NEG	NEG
DEU23 (Turkey)	NEG	NEG	NEG	NEG	NEG	NEG	NEG	NEG	NEG	NEG	NEG	NEG	NEG	NEG	NEG	POS	NEG	NEG
DEU3 (Turkey)	NEG	NEG	NEG	NEG	NEG	NEG	NEG	NEG	NEG	NEG	NEG	NEG	NEG	NEG	NEG	POS	NEG	NEG
DEU5 (Turkey)	NEG	NEG	NEG	NEG	NEG	NEG	NEG	NEG	NEG	NEG	NEG	NEG	NEG	NEG	NEG	POS	NEG	NEG
DEU6 (Turkey)	NEG	NEG	NEG	NEG	NEG	NEG	NEG	NEG	NEG	NEG	NEG	NEG	NEG	NEG	NEG	POS	NEG	NEG
DEU8 (Turkey)	NEG	NEG	NEG	NEG	NEG	NEG	NEG	NEG	NEG	NEG	NEG	NEG	NEG	NEG	NEG	POS	NEG	NEG
HU11 (Turkey)	NEG	NEG	NEG	NEG	NEG	NEG	NEG	NEG	NEG	NEG	NEG	NEG	NEG	NEG	NEG	POS	NEG	NEG
HU13 (Turkey)	NEG	NEG	NEG	NEG	NEG	NEG	NEG	NEG	NEG	NEG	NEG	NEG	NEG	NEG	NEG	POS	NEG	NEG
HU14 (Turkey)	NEG	NEG	NEG	NEG	NEG	POS	NEG	NEG	NEG	NEG	NEG	NEG	NEG	NEG	NEG	POS	NEG	NEG
HU15 (Turkey)	NEG	NEG	NEG	NEG	NEG	POS	NEG	NEG	NEG	NEG	NEG	NEG	NEG	NEG	NEG	POS	NEG	NEG
HU16 (Turkey)	NEG	NEG	NEG	NEG	NEG	NEG	NEG	NEG	NEG	NEG	NEG	NEG	NEG	NEG	NEG	POS	NEG	NEG
HU17 (Turkey)	NEG	NEG	NEG	NEG	NEG	NEG	NEG	NEG	NEG	NEG	NEG	NEG	NEG	NEG	NEG	POS	NEG	NEG
HU21 (Turkey)	NEG	NEG	NEG	NEG	NEG	NEG	NEG	NEG	NEG	NEG	NEG	NEG	NEG	NEG	NEG	POS	NEG	NEG
HU23 (Turkey)	NEG	NEG	NEG	NEG	NEG	NEG	NEG	NEG	NEG	NEG	NEG	NEG	NEG	NEG	NEG	POS	NEG	NEG
HU26 (Turkey)	NEG	NEG	NEG	NEG	NEG	NEG	NEG	NEG	NEG	NEG	NEG	NEG	NEG	NEG	NEG	POS	NEG	NEG
HU4 (Turkey)	NEG	NEG	NEG	NEG	NEG	NEG	NEG	NEG	NEG	NEG	NEG	NEG	NEG	NEG	NEG	POS	NEG	NEG
HU5 (Turkey)	NEG	NEG	NEG	NEG	NEG	NEG	NEG	NEG	NEG	NEG	NEG	NEG	NEG	NEG	NEG	POS	NEG	NEG
HU6 (Turkey)	NEG	NEG	NEG	NEG	NEG	NEG	NEG	NEG	NEG	NEG	NEG	NEG	NEG	NEG	NEG	POS	NEG	NEG
HU7 (Turkey)	NEG	NEG	NEG	NEG	NEG	NEG	NEG	NEG	NEG	NEG	NEG	NEG	NEG	NEG	NEG	POS	NEG	NEG
HU8 (Turkey)	NEG	NEG	NEG	NEG	NEG	NEG	NEG	NEG	NEG	NEG	NEG	NEG	NEG	NEG	NEG	POS	NEG	NEG
HU9 (Turkey)	NEG	NEG	NEG	NEG	NEG	NEG	NEG	NEG	NEG	NEG	NEG	NEG	NEG	NEG	NEG	POS	NEG	NEG
IU1 (Turkey)	NEG	NEG	NEG	NEG	NEG	NEG	NEG	NEG	NEG	NEG	NEG	NEG	NEG	NEG	NEG	POS	NEG	NEG
IU10 (Turkey)	NEG	NEG	NEG	NEG	NEG	NEG	NEG	NEG	NEG	NEG	NEG	NEG	NEG	NEG	NEG	POS	NEG	NEG
IU11 (Turkey)	NEG	NEG	NEG	NEG	NEG	NEG	NEG	NEG	NEG	NEG	NEG	NEG	NEG	NEG	NEG	POS	NEG	NEG
IU12 (Turkey)	NEG	NEG	NEG	NEG	NEG	NEG	NEG	NEG	NEG	NEG	NEG	NEG	NEG	NEG	NEG	POS	NEG	NEG
IU13 (Turkey)	NEG	NEG	NEG	NEG	NEG	NEG	NEG	NEG	NEG	NEG	NEG	NEG	NEG	NEG	NEG	POS	NEG	NEG
IU15 (Turkey)	NEG	NEG	NEG	NEG	NEG	NEG	NEG	NEG	NEG	NEG	NEG	NEG	NEG	NEG	NEG	POS	NEG	NEG
IU18 (Turkey)	NEG	NEG	NEG	NEG	NEG	NEG	NEG	NEG	NEG	NEG	NEG	NEG	NEG	NEG	NEG	POS	NEG	NEG
IU19 (Turkey)	NEG	NEG	NEG	NEG	NEG	NEG	NEG	NEG	NEG	NEG	NEG	NEG	NEG	NEG	NEG	POS	NEG	NEG
IU2 (Turkey)	NEG	NEG	NEG	NEG	NEG	NEG	NEG	NEG	NEG	NEG	NEG	NEG	NEG	NEG	NEG	POS	NEG	NEG
IU4 (Turkey)	NEG	NEG	NEG	NEG	NEG	NEG	NEG	NEG	NEG	NEG	NEG	NEG	NEG	NEG	NEG	POS	NEG	NEG
IU6 (Turkey)	NEG	NEG	NEG	NEG	NEG	NEG	NEG	NEG	NEG	NEG	NEG	NEG	NEG	NEG	NEG	POS	NEG	NEG
IU7 (Turkey)	NEG	NEG	NEG	NEG	NEG	NEG	NEG	NEG	NEG	NEG	NEG	NEG	NEG	NEG	NEG	POS	NEG	NEG
IU9 (Turkey)	NEG	NEG	NEG	NEG	NEG	NEG	NEG	NEG	NEG	NEG	NEG	NEG	NEG	NEG	NEG	POS	NEG	NEG
MU1 (Turkey)	NEG	NEG	NEG	NEG	NEG	NEG	NEG	NEG	NEG	NEG	NEG	NEG	NEG	NEG	NEG	POS	NEG	NEG
MU10 (Turkey)	NEG	NEG	NEG	NEG	NEG	NEG	NEG	NEG	NEG	NEG	NEG	NEG	NEG	NEG	NEG	POS	NEG	NEG
MU20 (Turkey)	NEG	NEG	NEG	NEG	NEG	NEG	NEG	NEG	NEG	NEG	NEG	NEG	NEG	NEG	NEG	POS	NEG	NEG
Mu3 (Turkey)	NEG	NEG	NEG	NEG	NEG	NEG	NEG	NEG	NEG	NEG	NEG	POS	NEG	NEG	NEG	POS	NEG	NEG
MU5 (Turkey)	NEG	NEG	NEG	NEG	NEG	POS	NEG	NEG	NEG	NEG	NEG	NEG	NEG	NEG	NEG	POS	NEG	NEG
MU6 (Turkey)	NEG	NEG	NEG	NEG	NEG	NEG	NEG	NEG	NEG	NEG	NEG	NEG	NEG	NEG	NEG	POS	NEG	NEG
MU7 (Turkey)	NEG	NEG	NEG	NEG	NEG	NEG	NEG	NEG	NEG	NEG	NEG	NEG	NEG	NEG	NEG	POS	NEG	NEG
TUR27 (Turkey)	NEG	NEG	NEG	NEG	NEG	NEG	NEG	NEG	NEG	NEG	NEG	NEG	NEG	NEG	NEG	POS	NEG	NEG
NCTR 32S	NEG	NEG	NEG	NEG	NEG	NEG	NEG	NEG	NEG	NEG	NEG	NEG	NEG	NEG	NEG	POS	NEG	NEG
SA02_A4 = Russia-06_0085_Moscow	NEG	NEG	NEG	NEG	NEG	NEG	NEG	NEG	NEG	NEG	NEG	NEG	NEG	NEG	NEG	POS	NEG	NEG
	NEG	NEG	NEG	NEG	NEG	NEG	NEG	NEG	NEG	NEG	NEG	NEG	NEG	NEG	NEG	POS	NEG	NEG
Dresden_08V35987 (Turkey)	NEG	NEG	NEG	NEG	NEG	NEG	NEG	NEG	NEG	NEG	NEG	NEG	NEG	NEG	NEG	POS	NEG	NEG
Dresden_17ANR577152	NEG	NEG	NEG	NEG	NEG	NEG	NEG	NEG	NEG	NEG	NEG	NEG	NEG	NEG	NEG	POS	NEG	NEG
Dresden_17ANR580374 (Makedonia)	NEG	NEG	NEG	NEG	NEG	NEG	NEG	NEG	NEG	NEG	NEG	NEG	NEG	NEG	NEG	POS	NEG	NEG
Hong Kong_69-II	NEG	NEG	NEG	NEG	NEG	NEG	NEG	NEG	NEG	NEG	NEG	NEG	NEG	NEG	NEG	POS	NEG	NEG
Hong Kong_93	NEG	NEG	NEG	NEG	NEG	NEG	NEG	NEG	NEG	NEG	NEG	NEG	NEG	NEG	NEG	POS	NEG	NEG
Rawalpindi_Kidney Center_03_SK1	NEG	NEG	NEG	NEG	NEG	NEG	NEG	NEG	NEG	NEG	NEG	NEG	NEG	NEG	NEG	POS	NEG	NEG
Rawalpindi_Kidney Center_08	NEG	NEG	NEG	NEG	NEG	NEG	NEG	NEG	NEG	NEG	NEG	NEG	NEG	NEG	NEG	POS	NEG	NEG
Rawalpindi_Kidney Center_10	NEG	NEG	NEG	NEG	NEG	NEG	NEG	NEG	NEG	NEG	NEG	NEG	NEG	NEG	NEG	POS	NEG	NEG
Rawalpindi_Kidney Center_19	NEG	NEG	NEG	NEG	NEG	NEG	NEG	NEG	NEG	NEG	NEG	NEG	NEG	NEG	NEG	POS	NEG	NEG
Rawalpindi_Kidney Center_50_SK1	NEG	NEG		NEG	NEG	NEG	NEG	NEG	NEG	NEG	NEG	NEG	NEG	NEG	NEG	POS	NEG	NEG
Romania_Iasi_BC-49_430	NEG	NEG	NEG	NEG	NEG	NEG	NEG	NEG	NEG	NEG	NEG	NEG	NEG	NEG	NEG	POS	NEG	NEG
Romania_Iasi_MRSA_284	NEG	NEG	NEG	NEG	NEG	NEG	NEG	NEG	NEG	NEG	NEG	NEG	NEG	NEG	NEG	POS	NEG	NEG
Romania_Iasi_MRSA-32_318	NEG	NEG	NEG	NEG	NEG	POS	NEG	NEG	NEG	NEG	NEG	NEG	NEG	NEG	NEG	POS	NEG	NEG
Romania_Iasi_MRSA-54_430	NEG	NEG	NEG	NEG	NEG	AMB	NEG	NEG	NEG	NEG	NEG	NEG	NEG	NEG	NEG	POS	NEG	NEG
Romania_Iasi_SSTI-01_2	NEG	NEG	NEG	NEG	NEG	NEG	NEG	NEG	NEG	NEG	NEG	NEG	NEG	NEG	NEG	POS	NEG	NEG
Romania_Iasi_SSTI-10_101	NEG	NEG	NEG	NEG	NEG	NEG	NEG	NEG	NEG	NEG	NEG	NEG	NEG	NEG	NEG	POS	NEG	NEG
Romania_Iasi_SSTI-11_106	NEG	NEG	NEG	NEG	NEG	NEG	NEG	NEG	NEG	NEG	NEG	NEG	NEG	NEG	NEG	POS	NEG	NEG
Russia-01_0001_SaintPetersburg	NEG	NEG	NEG	NEG	NEG	POS	NEG	NEG	NEG	NEG	NEG	NEG	NEG	NEG	NEG	POS	NEG	NEG
Russia-02_0004_SaintPetersburg	NEG	NEG	NEG	NEG	NEG	POS	NEG	NEG	NEG	NEG	NEG	NEG	NEG	NEG	NEG	POS	NEG	NEG
Russia-03_0057_SaintPetersburg	NEG	NEG	NEG	NEG	NEG	POS	NEG	NEG	NEG	NEG	NEG	NEG	NEG	NEG	NEG	POS	NEG	NEG
Russia-04_0076_Moscow	NEG	NEG	NEG	NEG	NEG	NEG	NEG	NEG	NEG	NEG	NEG	NEG	NEG	NEG	NEG	POS	NEG	NEG
Russia-05_0078_Moscow	NEG	NEG	NEG	NEG	NEG	NEG	NEG	NEG	NEG	NEG	NEG	NEG	NEG	NEG	NEG	POS	NEG	NEG
Russia-08_0135_Moscow	NEG	NEG	NEG	NEG	NEG	NEG	NEG	NEG	NEG	NEG	NEG	NEG	NEG	NEG	NEG	POS	NEG	NEG
Russia-09_0150_Kurgan	NEG	NEG	NEG	NEG	NEG	NEG	NEG	NEG	NEG	NEG	NEG	NEG	NEG	NEG	NEG	POS	NEG	NEG
Russia-10_0162_Kurgan	NEG	NEG	NEG	NEG	NEG	NEG	NEG	NEG	NEG	NEG	NEG	NEG	NEG	NEG	NEG	POS	NEG	NEG
Russia-14_0184_Moscow	NEG	NEG	NEG	NEG	NEG	NEG	NEG	NEG	NEG	NEG	NEG	NEG	NEG	NEG	NEG	POS	NEG	NEG
Russia-15_0232_SaintPetersburg	NEG	NEG	NEG	NEG	NEG	NEG	NEG	NEG	NEG	NEG	NEG	NEG	NEG	NEG	NEG	POS	NEG	NEG
Russia-22_0391_Chelyabinsk	NEG	NEG	NEG	NEG	NEG	NEG	NEG	NEG	NEG	NEG	NEG	NEG	NEG	NEG	NEG	POS	NEG	NEG
Russia-23_0392_Chelyabinsk	NEG	NEG	NEG	NEG	NEG	NEG	NEG	NEG	NEG	NEG	NEG	NEG	NEG	NEG	NEG	POS	NEG	NEG
**>"Eurasian Clade" : IU17**			**NEG**	**NEG**		**NEG**	**NEG**					**NEG**		**NEG**		**POS**		
IU17 (Turkey)	NEG	NEG	NEG	NEG	NEG	NEG	NEG	NEG	NEG	NEG	NEG	NEG	NEG	NEG	NEG	POS	NEG	NEG
IU20 (Turkey)	NEG	NEG	NEG	NEG	NEG	NEG	NEG	NEG	NEG	NEG	NEG	NEG	NEG	NEG	NEG	POS	NEG	NEG

STRAIN / ISOLATE	RESISTANCE : AMINOGLYSOSIDES				RESISTANCE : MISCELLANEOUS GENES													
	aadA		aadE	aphA3	sat	dfrA	dfrG				far1	mupA	mupB	tetK	tetL	tetM		
	aadA-var1	aadA-var2	aadE	aphA3	sat	dfrA	dfrG	dfrG-Tn559	dfrG-K	dfrG-D	far1	mupA	mupB	tetK	tetL	tetM (com-bined)	tetM-O	tetM-S
	aminoglycoside-3'-adenylyltransferase			3'5'-aminog-lycoside phospho-transferase, neo-/ kanamycin resistance	strepto-thricine-acetyl-transferase	dihydro-folate reductase type 1	dihydrofolate reductase				fusidic acid resistence	mupirocin resistence protein	plasmidic isoleucyl-tRNA synthase	tetracycline resistance	Tetracycline resistance gene	tetracycline resistance		
**>"Eurasian Clade": Spor**			NEG	NEG		NEG	NEG					NEG		NEG		POS		
Dresden_16ANR581769	NEG	NEG	NEG	NEG	NEG	NEG	NEG	NEG	NEG	NEG	NEG	NEG	NEG	NEG	NEG	POS	NEG	NEG
Dresden_17ANR578769	NEG	NEG	NEG	NEG	NEG	NEG	NEG	NEG	NEG	NEG	NEG	NEG	NEG	NEG	NEG	POS	NEG	NEG
**>"Eurasian Clade": Kuwa**			NEG	NEG		NEG	NEG					NEG		NEG		POS		
Kuwait_306	NEG	NEG	NEG	NEG	NEG	NEG	NEG	NEG	NEG	NEG	NEG	NEG	NEG	NEG	NEG	POS	NEG	NEG
Kuwait_301	NEG	NEG	NEG	NEG	NEG	NEG	NEG	NEG	NEG	NEG	NEG	NEG	NEG	NEG	NEG	POS	NEG	NEG
**>"European Clade": "Gr**			COMM	COMM		NEG	NEG					NEG		VAR		COMM		
GRE18 (Greece)	NEG	NEG	POS	POS	POS	NEG	NEG	NEG	NEG	NEG	NEG	NEG	NEG	POS	NEG	POS	NEG	NEG
GRE317 (Greece)	NEG	NEG	POS	POS	POS	NEG	NEG	NEG	NEG	NEG	NEG	NEG	NEG	NEG	NEG	POS	NEG	NEG
GRE4 (Greece)	NEG	NEG	POS	POS	POS	NEG	NEG	NEG	NEG	NEG	NEG	NEG	NEG	NEG	NEG	POS	NEG	NEG
R35 (USA)	NEG	NEG	NEG	NEG	NEG	NEG	NEG	NEG	NEG	NEG	NEG	NEG	NEG	POS	NEG	POS	NEG	NEG
Casablanca_HT20060548	NEG	NEG	POS	POS	POS	NEG	NEG	NEG	NEG	NEG	NEG	NEG	NEG	POS	NEG	NEG	NEG	NEG
Casablanca_HT20060550	NEG	NEG	POS	POS	POS	NEG	NEG	NEG	NEG	NEG	NEG	NEG	NEG	NEG	NEG	NEG	NEG	NEG
Dresden_01V34572 (Greece)	NEG	NEG	POS	POS	POS	NEG	NEG	NEG	NEG	NEG	NEG	NEG	NEG	NEG	NEG	POS	NEG	NEG
Dresden_01V36469	NEG	NEG	POS	POS	POS	NEG	NEG	NEG	NEG	NEG	NEG	NEG	NEG	NEG	NEG	POS	NEG	NEG
Dresden_01V39123	NEG	NEG	POS	POS	POS	NEG	NEG	NEG	NEG	NEG	NEG	NEG	NEG	NEG	NEG	POS	NEG	NEG
Dresden_01V39143	NEG	NEG	POS	POS	POS	NEG	NEG	NEG	NEG	NEG	NEG	NEG	NEG	NEG	NEG	POS	NEG	NEG
Greece_1_3680_Harmony	NEG	NEG	POS	POS	POS	NEG	NEG	NEG	NEG	NEG	NEG	NEG	NEG	AMB	NEG	POS	NEG	NEG
**>"European Clade": UK-**			NEG	NEG		VAR	NEG					NEG		POS		POS		
ANS46 (Australia)	NEG	NEG	NEG	NEG	NEG	NEG	NEG	NEG	NEG	NEG	NEG	NEG	NEG	POS	NEG	POS	NEG	NEG
LHH1 (USA)	NEG	NEG	NEG	NEG	NEG	POS	NEG	NEG	NEG	NEG	NEG	NEG	NEG	POS	NEG	POS	NEG	NEG
Dublin-DSH_AR01_0-1085	NEG	NEG	NEG	NEG	NEG	POS	NEG	NEG	NEG	NEG	NEG	NEG	NEG	POS	NEG	POS	NEG	NEG
Dublin-DSH_AR01_0-1118	NEG	NEG	NEG	NEG	NEG	NEG	NEG	NEG	NEG	NEG	NEG	NEG	NEG	POS	NEG	POS	NEG	NEG
Dublin-DSH_AR15_0098	NEG	NEG	NEG	NEG	NEG	POS	NEG	NEG	NEG	NEG	NEG	NEG	NEG	POS	NEG	POS	NEG	NEG
Dublin-DSH_AR15_0104	NEG	NEG	NEG	NEG	NEG	POS	NEG	NEG	NEG	NEG	NEG	NEG	NEG	POS	NEG	POS	NEG	NEG
UK_NCTC11939_UK-1_Harmony	NEG	NEG	NEG	NEG	NEG	POS	NEG	NEG	NEG	NEG	NEG	NEG	NEG	POS	NEG	POS	NEG	NEG
**>"European Clade": BK2**			NEG	POS		POS	NEG					NEG		POS		POS		
BK2421 (USA)	NEG	NEG	NEG	POS	POS	POS	NEG	NEG	NEG	NEG	NEG	NEG	NEG	POS	NEG	POS	NEG	NEG
**>"Aussi/NZ Clade", JKD6**			NEG	NEG		VAR	NEG					NEG		NEG		POS		
JKD6009 (Australia)	NEG	NEG	NEG	NEG	NEG	POS	NEG	NEG	NEG	NEG	NEG	NEG	NEG	NEG	NEG	POS	NEG	NEG
JKD6008 (New Zealand)	NEG	NEG	NEG	NEG	NEG	POS	NEG	NEG	NEG	NEG	NEG	NEG	NEG	NEG	NEG	POS	NEG	NEG
Perth_2015-S20209	NEG	NEG	NEG	NEG	NEG	POS	NEG	NEG	NEG	NEG	NEG	NEG	NEG	NEG	NEG	POS	NEG	NEG
Perth_2015-S20305	NEG	NEG	NEG	NEG	NEG	POS	NEG	NEG	NEG	NEG	NEG	NEG	NEG	NEG	NEG	POS	NEG	NEG
Perth_2015-S20603	NEG	NEG	NEG	NEG	NEG	POS	NEG	NEG	NEG	NEG	NEG	NEG	NEG	NEG	NEG	POS	NEG	NEG
Perth_2015-S20607	NEG	NEG	NEG	NEG	NEG	POS	NEG	NEG	NEG	NEG	NEG	NEG	NEG	NEG	NEG	POS	NEG	NEG
Perth_2015-S20608	NEG	NEG	NEG	NEG	NEG	POS	NEG	NEG	NEG	NEG	NEG	NEG	NEG	NEG	NEG	POS	NEG	NEG
Perth_2015-S20610	NEG	NEG	NEG	NEG	NEG	POS	NEG	NEG	NEG	NEG	NEG	NEG	NEG	NEG	NEG	POS	NEG	NEG
Perth_2015-S20613	NEG	NEG	NEG	NEG	NEG	POS	NEG	NEG	NEG	NEG	NEG	NEG	NEG	NEG	NEG	POS	NEG	NEG
Perth_2015-S20617	NEG	NEG	NEG	NEG	NEG	POS	NEG	NEG	NEG	NEG	NEG	NEG	NEG	NEG	NEG	POS	NEG	NEG
Perth_2015-S20622	NEG	NEG	NEG	NEG	NEG	POS	NEG	NEG	NEG	NEG	NEG	NEG	NEG	NEG	NEG	POS	NEG	NEG
Perth_2015-S23619	NEG	NEG	NEG	NEG	NEG	POS	NEG	NEG	NEG	NEG	NEG	NEG	NEG	NEG	NEG	POS	NEG	NEG
Perth_2015-S41210	NEG	NEG	NEG	NEG	NEG	POS	NEG	NEG	NEG	NEG	NEG	NEG	NEG	NEG	NEG	POS	NEG	NEG
Perth_2015-S51001	NEG	NEG	NEG	NEG	NEG	NEG	NEG	NEG	NEG	NEG	NEG	NEG	NEG	NEG	NEG	POS	NEG	NEG
Perth_2015-S51003	NEG	NEG	NEG	NEG	NEG	NEG	NEG	NEG	NEG	NEG	NEG	NEG	NEG	NEG	NEG	POS	NEG	NEG
Perth_2015-S51006	NEG	NEG	NEG	NEG	NEG	NEG	NEG	NEG	NEG	NEG	NEG	NEG	NEG	NEG	NEG	POS	NEG	NEG
Perth_2015-S51007	NEG	NEG	NEG	NEG	NEG	NEG	NEG	NEG	NEG	NEG	NEG	NEG	NEG	NEG	NEG	POS	NEG	NEG
Perth_2015-S51008	NEG	NEG	NEG	NEG	NEG	NEG	NEG	NEG	NEG	NEG	NEG	NEG	NEG	NEG	NEG	POS	NEG	NEG
Perth_2015-S51014	NEG	NEG	NEG	NEG	NEG	NEG	NEG	NEG	NEG	NEG	NEG	NEG	NEG	NEG	NEG	POS	NEG	NEG
Perth_2015-S51408	NEG	NEG	NEG	NEG	NEG	POS	NEG	NEG	NEG	NEG	NEG	NEG	NEG	NEG	NEG	POS	NEG	NEG
Perth_2016-S20614	NEG	NEG	NEG	NEG	NEG	POS	NEG	NEG	NEG	NEG	NEG	NEG	NEG	NEG	NEG	POS	NEG	NEG
Perth_2016-S23614	NEG	NEG	NEG	NEG	NEG	POS	NEG	NEG	NEG	NEG	NEG	NEG	NEG	NEG	NEG	POS	NEG	NEG
Perth_2016-S42009	NEG	NEG	NEG	NEG	NEG	POS	NEG	NEG	NEG	NEG	NEG	NEG	NEG	NEG	NEG	POS	NEG	NEG
Perth_2016-S51001	NEG	NEG	NEG	NEG	NEG	NEG	NEG	NEG	NEG	NEG	NEG	NEG	NEG	NEG	NEG	POS	NEG	NEG
Perth_2016-S51403	NEG	NEG	NEG	NEG	NEG	NEG	NEG	NEG	NEG	NEG	NEG	NEG	NEG	NEG	NEG	POS	NEG	NEG
Perth_07 RPA 20	NEG	NEG	NEG	NEG	NEG	POS	NEG	NEG	NEG	NEG	NEG	NEG	NEG	NEG	NEG	POS	NEG	NEG
Perth_08-19088	NEG	NEG	NEG	NEG	NEG	POS	NEG	NEG	NEG	NEG	NEG	NEG	NEG	NEG	NEG	POS	NEG	NEG
Perth_01-15351	NEG	NEG	NEG	NEG	NEG	NEG	NEG	NEG	NEG	NEG	NEG	NEG	NEG	NEG	NEG	POS	NEG	NEG
Perth_01-15357	NEG	NEG	NEG	NEG	NEG	NEG	NEG	NEG	NEG	NEG	NEG	NEG	NEG	NEG	NEG	POS	NEG	NEG
Perth_01-15419	NEG	NEG	NEG	NEG	NEG	NEG	NEG	NEG	NEG	NEG	NEG	NEG	NEG	NEG	NEG	POS	NEG	NEG
Perth_01-16309	NEG	NEG	NEG	NEG	NEG	NEG	NEG	NEG	NEG	NEG	NEG	NEG	NEG	NEG	NEG	POS	NEG	NEG
Perth_03 SNP 95	NEG	NEG	NEG	NEG	NEG	NEG	NEG	NEG	NEG	NEG	NEG	NEG	NEG	NEG	NEG	POS	NEG	NEG
Perth_2003_AH_47	NEG	NEG	NEG	NEG	NEG	POS	NEG	NEG	NEG	NEG	NEG	NEG	NEG	NEG	NEG	POS	NEG	NEG
**>"Australian/NZ Clade":**			NEG	NEG		POS	NEG					NEG		NEG		POS		
Perth_2015-S20623	NEG	NEG	NEG	NEG	NEG	POS	NEG	NEG	NEG	NEG	NEG	NEG	NEG	NEG	NEG	POS	NEG	NEG
Perth_2015-S20826	NEG	NEG	NEG	NEG	NEG	POS	NEG	NEG	NEG	NEG	NEG	NEG	NEG	NEG	NEG	POS	NEG	NEG
Perth_05 NH 56	NEG	NEG	NEG	NEG	NEG	POS	NEG	NEG	NEG	NEG	NEG	NEG	NEG	NEG	NEG	POS	NEG	NEG
**>CC5-MRSA-III LA MRSA**			NEG	NEG		NEG	NEG					NEG		NEG		POS		
Delbrück 38 isolate from domestic turkey	NEG	NEG	NEG	NEG	NEG	NEG	NEG	NEG	NEG	NEG	NEG	NEG	NEG	NEG	NEG	POS	NEG	NEG
**> Staph. pseudintermed**			NEG	NEG		NEG	NEG					NEG		NEG		NEG		
KM1381	NEG	NEG	NEG	NEG	NEG	NEG	NEG	NEG	NEG	NEG	NEG	NEG	NEG	NEG	NEG	NEG	NEG	NEG
**>"South-East Asian Clad**			pos	COMM		RARE	VAR					RARE		VAR		POS		
M92 (Canada)	NEG	NEG	POS	POS	POS	NEG	NEG	NEG	NEG	NEG	NEG	NEG	NEG	POS	NEG	POS	NEG	NEG
CHI59 (China)	NEG	NEG	POS	POS	POS	NEG	NEG	NEG	NEG	NEG	NEG	NEG	NEG	POS	NEG	POS	NEG	NEG
CHI61 (China)	NEG	NEG	POS	POS	POS	NEG	NEG	NEG	NEG	NEG	NEG	NEG	NEG	NEG	NEG	POS	NEG	NEG
CUHK_HK1997 (China)	NEG	NEG	POS	POS	POS	NEG	NEG	NEG	NEG	NEG	NEG	NEG	NEG	POS	NEG	POS	NEG	NEG
M996 (China)	NEG	NEG	POS	POS	POS	NEG	NEG	NEG	NEG	NEG	NEG	NEG	NEG	NEG	NEG	POS	NEG	NEG
H211 (Denmark)	NEG	NEG	POS	POS	POS	NEG	POS	AMB	AMB	AMB	NEG	NEG	NEG	NEG	NEG	POS	NEG	NEG
H216 (Denmark)	NEG	NEG	POS	POS	POS	NEG	POS	AMB	AMB	AMB	NEG	NEG	NEG	NEG	NEG	POS	NEG	NEG
D71 (Germany)	NEG	NEG	POS	POS	POS	NEG	NEG	NEG	NEG	NEG	NEG	POS	NEG	NEG	NEG	POS	NEG	NEG
NMR05 (India)	NEG	NEG	POS	POS	POS	POS	NEG	NEG	NEG	NEG	NEG	NEG	NEG	POS	NEG	POS	NEG	NEG
MAL11 (Malaysia)	NEG	NEG	POS	POS	POS	NEG	NEG	NEG	NEG	NEG	NEG	NEG	NEG	POS	NEG	POS	NEG	NEG
MAL9 (Malaysia)	NEG	NEG	POS	POS	POS	NEG	POS	AMB	AMB	AMB	NEG	NEG	NEG	POS	NEG	POS	NEG	NEG
Na21 (Sri Lanka)	NEG	NEG	POS	POS	POS	POS	NEG	NEG	NEG	NEG	NEG	NEG	NEG	POS	NEG	POS	NEG	NEG
M592 (Syria)	NEG	NEG	POS	POS	POS	NEG	POS	AMB	AMB	AMB	NEG	NEG	NEG	POS	NEG	POS	NEG	NEG
H202 (Thailand)	NEG	NEG	POS	POS	POS	NEG	POS	AMB	AMB	AMB	NEG	NEG	NEG	NEG	NEG	POS	NEG	NEG
S102 (Thailand)	NEG	NEG	POS	POS	POS	NEG	POS	AMB	AMB	AMB	NEG	NEG	NEG	POS	NEG	POS	NEG	NEG
S130 (Thailand)	NEG	NEG	POS	POS	POS	NEG	POS	AMB	AMB	AMB	NEG	NEG	NEG	NEG	NEG	POS	NEG	NEG
S40 (Thailand)	NEG	NEG	POS	POS	POS	NEG	POS	AMB	AMB	AMB	NEG	NEG	NEG	POS	NEG	POS	NEG	NEG
S71 (Thailand)	NEG	NEG	POS	POS	POS	NEG	POS	AMB	AMB	AMB	NEG	NEG	NEG	POS	NEG	POS	NEG	NEG
S87 (Thailand)	NEG	NEG	POS	POS	POS	NEG	POS	AMB	AMB	AMB	NEG	NEG	NEG	NEG	NEG	POS	NEG	NEG
S93 (Thailand)	NEG	NEG	POS	POS	POS	NEG	POS	AMB	AMB	AMB	NEG	NEG	NEG	POS	NEG	POS	NEG	NEG
US_002 (Thailand)	NEG	NEG	POS	POS	POS	NEG	POS	AMB	AMB	AMB	NEG	NEG	NEG	NEG	NEG	POS	NEG	NEG
US_017 (Thailand)	NEG	NEG	POS	POS	POS	NEG	POS	AMB	AMB	AMB	NEG	NEG	NEG	POS	NEG	POS	NEG	NEG
WAMC6102 (Thailand)	NEG	NEG	POS	POS	POS	NEG	POS	AMB	AMB	AMB	NEG	NEG	NEG	POS	NEG	POS	NEG	NEG
NCTC_13626_TW20 (UK)	NEG	NEG	POS	POS	POS	NEG	POS	AMB	AMB	AMB	NEG	NEG	NEG	POS	NEG	POS	NEG	NEG
BSAC3021 (UK)	NEG	NEG	POS	POS	POS	NEG	POS	AMB	AMB	AMB	NEG	NEG	NEG	POS	NEG	POS	NEG	NEG
BSAC3046 (UK)	NEG	NEG	POS	POS	POS	NEG	POS	AMB	AMB	AMB	NEG	NEG	NEG	POS	NEG	POS	NEG	NEG
1193_SAUR (USA)	NEG	NEG	POS	POS	POS	NEG	POS	AMB	AMB	AMB	NEG	NEG	NEG	NEG	NEG	POS	NEG	NEG
1194_SAUR (USA)	NEG	NEG	POS	POS	POS	NEG	POS	AMB	AMB	AMB	NEG	NEG	NEG	NEG	NEG	POS	NEG	NEG

STRAIN / ISOLATE	RESISTANCE : AMINOGLYSOSIDES				RESISTANCE : MISCELLANEOUS GENES													
	aadA		aadE	aphA3	sat	dfrA	dfrG				far1	mupA	mupB	tetK	tetL	tetM		
	aadA-var1	aadA-var2	aadE	aphA3	sat	dfrA	dfrG	dfrG-Tn559	dfrG-K	dfrG-D	far1	mupA	mupB	tetK	tetL	tetM (com-bined)	tetM-O	tetM-S
	aminoglycoside-3'-adenylyltransferase			3'5'-aminog-lycoside phospho-transferase, neo-/ kanamycin resistance	strepto-thricine-acetyl-transferase	dihydro-folate reductase type 1	dihydrofolate reductase				fusidic acid resistence	mupirocin resistence protein	plasmidic isoleucyl-tRNA synthase	tetracycline resistance	Tetracycline resistance gene	tetracycline resistance		
>"South-East Asian Clade": TW20 (Irish AR44), c																		
1195_SAUR (USA)	NEG	NEG	POS	POS	POS	NEG	POS	AMB	AMB	AMB	NEG	NEG	NEG	NEG	NEG	POS	NEG	NEG
FVRH6002 (USA)	NEG	NEG	POS	POS	POS	NEG	POS	AMB	AMB	AMB	NEG	NEG	NEG	POS	NEG	POS	NEG	NEG
KINW6048 (USA)	NEG	NEG	POS	POS	POS	NEG	POS	AMB	AMB	AMB	NEG	NEG	NEG	POS	NEG	POS	NEG	NEG
LAMC0011 (USA)	NEG	NEG	POS	POS	POS	NEG	POS	AMB	AMB	AMB	NEG	NEG	NEG	POS	NEG	POS	NEG	NEG
SJOS6053 (USA)	NEG	NEG	NEG	POS	NEG	NEG	POS	AMB	AMB	AMB	NEG	NEG	NEG	POS	NEG	POS	NEG	NEG
SJOS6072 (USA)	NEG	NEG	POS	POS	POS	NEG	POS	AMB	AMB	AMB	NEG	NEG	NEG	POS	NEG	POS	NEG	NEG
UCIM6042 (USA)	NEG	NEG	POS	POS	POS	NEG	POS	AMB	AMB	AMB	NEG	NEG	NEG	POS	NEG	POS	NEG	NEG
Perth_2015-S31902	NEG	NEG	POS	POS	POS	NEG	POS	AMB	AMB	AMB	NEG	NEG	NEG	NEG	NEG	POS	NEG	NEG
Perth_2015-S31904	NEG	NEG	POS	POS	POS	NEG	POS	AMB	AMB	AMB	NEG	NEG	NEG	POS	NEG	POS	NEG	NEG
Perth_2015-S31913	NEG	NEG	POS	POS	POS	NEG	POS	AMB	AMB	AMB	NEG	NEG	NEG	POS	NEG	POS	NEG	NEG
Perth_2015-S31918	NEG	NEG	POS	POS	POS	NEG	POS	AMB	AMB	AMB	NEG	NEG	NEG	POS	NEG	POS	NEG	NEG
Dublin-DSH_AR44_E1183	NEG	NEG	POS	POS	POS	NEG	AMB	POS	AMB	AMB	NEG	POS	NEG	POS	NEG	POS	NEG	NEG
Dublin-DSH_Unfamiliar-3_E1520	NEG	NEG	POS	POS	POS	NEG	AMB	POS	AMB	AMB	NEG	NEG	NEG	POS	NEG	POS	NEG	NEG
Hong Kong_042	NEG	NEG	POS	POS	POS	NEG	NEG	NEG	NEG	NEG	NEG	NEG	NEG	NEG	NEG	POS	NEG	NEG
Hong Kong_101	NEG	NEG	POS	POS	POS	NEG	NEG	NEG	NEG	NEG	NEG	NEG	NEG	POS	NEG	POS	NEG	NEG
Hong Kong_111	NEG	NEG	POS	POS	POS	NEG	NEG	NEG	NEG	NEG	NEG	NEG	NEG	POS	NEG	POS	NEG	NEG
Hong Kong_113	NEG	NEG	POS	POS	POS	NEG	NEG	NEG	NEG	NEG	NEG	NEG	NEG	NEG	NEG	POS	NEG	NEG
Hong Kong_115	NEG	NEG	POS	POS	POS	NEG	NEG	NEG	NEG	NEG	NEG	NEG	NEG	POS	NEG	POS	NEG	NEG
Hong Kong_118	NEG	NEG	POS	POS	POS	NEG	NEG	NEG	NEG	NEG	NEG	NEG	NEG	POS	NEG	POS	NEG	NEG
Hong Kong_125	NEG	NEG	POS	POS	POS	NEG	NEG	NEG	NEG	NEG	NEG	NEG	NEG	POS	NEG	POS	NEG	NEG
Hong Kong_129	NEG	NEG	NEG	NEG	NEG	NEG	NEG	NEG	NEG	NEG	NEG	NEG	NEG	POS	NEG	POS	NEG	NEG
Hong Kong_133	NEG	NEG	POS	POS	POS	NEG	NEG	NEG	NEG	NEG	NEG	NEG	NEG	POS	NEG	POS	NEG	NEG
Kuwait_009	NEG	NEG	POS	POS	POS	NEG	AMB	POS	AMB	AMB	NEG	NEG	NEG	POS	NEG	POS	NEG	NEG
Kuwait_103	NEG	NEG	POS	POS	POS	NEG	POS	NEG	NEG	NEG	NEG	NEG	NEG	POS	NEG	POS	NEG	NEG
Kuwait_107	NEG	NEG	POS	POS	POS	NEG	POS	AMB	AMB	AMB	NEG	NEG	NEG	POS	NEG	POS	NEG	NEG
Perth_03 AH 88	NEG	NEG	POS	POS	POS	NEG	POS	AMB	AMB	NEG	NEG	NEG	NEG	POS	NEG	POS	NEG	NEG
Perth_08-16905	NEG	NEG	POS	POS	POS	NEG	POS	AMB	AMB	NEG	NEG	NEG	NEG	POS	NEG	POS	NEG	NEG
Riyadh_Alfaisal-10_SICU_13_B5672_1117353	NEG	NEG	POS	POS	POS	NEG	POS	AMB	AMB	NEG	NEG	NEG	NEG	POS	NEG	POS	NEG	NEG
Riyadh_SSTI_17_3508602	NEG	NEG	POS	POS	POS	NEG	POS	AMB	AMB	NEG	NEG	NEG	NEG	NEG	NEG	POS	NEG	NEG
Riyadh-2822088-R	NEG	NEG	POS	POS	POS	NEG	AMB	AMB	AMB	AMB	NEG	NEG	NEG	NEG	NEG	POS	NEG	NEG
Trinidad&Tobago_2012_195 (619/12)	NEG	NEG	POS	POS	POS	NEG	POS	AMB	AMB	NEG	NEG	NEG	NEG	NEG	NEG	POS	NEG	NEG
Trinidad&Tobago_2012_370 (1112-52177)	NEG	NEG	POS	POS	POS	NEG	POS	AMB	AMB	NEG	NEG	POS	NEG	POS	NEG	POS	NEG	NEG
Trinidad&Tobago_2012_378 (4139)	NEG	NEG	POS	POS	POS	NEG	POS	AMB	AMB	AMB	NEG	POS	NEG	POS	NEG	POS	NEG	NEG
Trinidad&Tobago_2013_PA21	NEG	NEG	POS	POS	POS	NEG	AMB	POS	AMB	AMB	NEG	POS	NEG	POS	NEG	POS	NEG	NEG
Trinidad&Tobago_2013_PA22	NEG	NEG	POS	POS	POS	NEG	POS	AMB	AMB	AMB	NEG	NEG	NEG	POS	NEG	POS	NEG	NEG
Trinidad&Tobago_2912_340 (8523/12)	NEG	NEG	POS	POS	POS	NEG	POS	AMB	AMB	NEG	NEG	NEG	NEG	POS	NEG	POS	NEG	NEG
Trinidad&Tobago_MRSA2010_105	NEG	NEG	POS	POS	POS	NEG	AMB	POS	AMB	AMB	NEG	POS	NEG	POS	NEG	POS	NEG	NEG
Trinidad&Tobago_MRSA2010_108	NEG	NEG	POS	POS	POS	NEG	POS	AMB	AMB	AMB	NEG	NEG	NEG	POS	NEG	POS	NEG	NEG
Trinidad&Tobago_MRSA2010_33	NEG	NEG	POS	POS	POS	NEG	POS	AMB	AMB	AMB	NEG	POS	NEG	POS	NEG	POS	NEG	NEG
Trinidad&Tobago_MRSA2010_79	NEG	NEG	POS	POS	POS	NEG	AMB	AMB	AMB	AMB	NEG	POS	NEG	POS	NEG	POS	NEG	NEG
Trinidad&Tobago_MRSA2010_88	NEG	NEG	POS	POS	POS	NEG	AMB	AMB	AMB	AMB	NEG	NEG	NEG	POS	NEG	POS	NEG	NEG
Trinidad&Tobago_MRSA2010_97	NEG	NEG	POS	POS	POS	NEG	AMB	POS	AMB	AMB	NEG	NEG	NEG	POS	NEG	POS	NEG	NEG
Trinidad&Tobago_SSTI 2012_031	NEG	NEG	POS	POS	POS	NEG	AMB	POS	AMB	AMB	NEG	NEG	NEG	POS	NEG	POS	NEG	NEG
Uganda-29_17148_250811	NEG	NEG	POS	POS	POS	NEG	POS	AMB	AMB	NEG	NEG	NEG	NEG	POS	NEG	POS	NEG	NEG
>"South-East Asian Clad			POS	POS		NEG	VAR					NEG		VAR		POS		
UCIM6015	NEG	NEG	POS	POS	POS	NEG	POS	AMB	AMB	AMB	NEG	NEG	NEG	POS	NEG	POS	NEG	NEG
Hong Kong_121	NEG	NEG	POS	POS	POS	NEG	NEG	NEG	NEG	NEG	NEG	NEG	NEG	NEG	NEG	POS	NEG	NEG
>"South-East Asian Clad			POS	POS		NEG	POS					NEG		POS		POS		
Z172 (Taiwan)	NEG	NEG	POS	POS	POS	NEG	POS	AMB	AMB	AMB	NEG	NEG	NEG	POS	NEG	POS	NEG	NEG
Russia-19_0342_Kurgan	NEG	NEG	POS	POS	POS	NEG	POS	AMB	AMB	NEG	NEG	NEG	NEG	POS	NEG	POS	NEG	NEG
>"South-East Asian Clad			POS	POS		NEG	RARE					NEG		POS		POS		
Perth_01-15392	NEG	NEG	POS	POS	POS	NEG	AMB	POS	AMB	AMB	NEG	NEG	NEG	POS	NEG	POS	NEG	NEG
>"South-East Asian Clad			COMM	COMM		VAR	VAR					NEG		RARE		POS		
XN108 (China)	NEG	NEG	POS	POS	POS	NEG	POS	AMB	AMB	AMB	NEG	NEG	NEG	NEG	NEG	POS	NEG	NEG
NMR09 (India)	NEG	NEG	POS	POS	POS	POS	NEG	NEG	NEG	NEG	NEG	NEG	NEG	NEG	NEG	POS	NEG	NEG
346 (Malaysia)	NEG	NEG	NEG	NEG	NEG	NEG	POS	AMB	AMB	AMB	NEG	NEG	NEG	NEG	NEG	POS	NEG	NEG
DS_009 (Thailand)	NEG	NEG	POS	POS	POS	NEG	POS	AMB	AMB	AMB	NEG	NEG	NEG	NEG	NEG	POS	NEG	NEG
18ANR581005 (Ind)	NEG	NEG	POS	POS	POS	POS	NEG	NEG	NEG	NEG	NEG	NEG	NEG	NEG	NEG	POS	NEG	NEG
Bengalaru_ST20141405	NEG	NEG	POS	POS	POS	POS	NEG	NEG	NEG	NEG	NEG	NEG	NEG	NEG	NEG	POS	NEG	NEG
Bengalaru_ST20141406	NEG	NEG	POS	POS	POS	POS	NEG	NEG	NEG	NEG	NEG	NEG	NEG	NEG	NEG	POS	NEG	NEG
Bengalaru_ST20141417	NEG	NEG	POS	POS	POS	POS	NEG	NEG	NEG	NEG	NEG	NEG	NEG	NEG	NEG	POS	NEG	NEG
Hong Kong_107	NEG	NEG	POS	POS	POS	NEG	NEG	NEG	NEG	NEG	NEG	NEG	NEG	NEG	NEG	POS	NEG	NEG
Hong Kong_12	NEG	NEG	POS	POS	POS	NEG	POS	NEG	NEG	NEG	NEG	NEG	NEG	POS	NEG	POS	NEG	NEG
Hong Kong_132	NEG	NEG	POS	POS	POS	NEG	NEG	NEG	NEG	NEG	NEG	NEG	NEG	NEG	NEG	POS	NEG	NEG
Riyadh-3028763-R	NEG	NEG	POS	POS	POS	NEG	POS	AMB	AMB	AMB	NEG	NEG	NEG	NEG	NEG	POS	NEG	NEG
Trinidad&Tobago_MRSA2010_94	NEG	NEG	POS	POS	POS	NEG	AMB	POS	AMB	AMB	NEG	NEG	NEG	NEG	NEG	POS	NEG	NEG
>"South-East Asian Clad			POS	POS		NEG	VAR					NEG		NEG		POS		
M705 (Thailand)	NEG	NEG	POS	POS	POS	NEG	POS	AMB	AMB	AMB	NEG	NEG	NEG	NEG	NEG	POS	NEG	NEG
S85 (Thailand)	NEG	NEG	POS	POS	POS	NEG	POS	AMB	AMB	AMB	NEG	NEG	NEG	NEG	NEG	POS	NEG	NEG
Riyadh_Alfaisal-04_281014_S5076	NEG	NEG	POS	POS	POS	NEG	POS	AMB	AMB	NEG	NEG	NEG	NEG	NEG	NEG	POS	NEG	NEG
Riyadh_Alfaisal-26_S15145_1104163	NEG	NEG	POS	POS	POS	NEG	POS	AMB	AMB	AMB	NEG	NEG	NEG	NEG	NEG	POS	NEG	NEG
Riyadh-2817437-W	NEG	NEG	POS	POS	POS	NEG	AMB	POS	AMB	AMB	NEG	NEG	NEG	NEG	NEG	POS	NEG	NEG
>"South-East Asian Clad			POS	POS		NEG	VAR					NEG		NEG		POS		
PPUKM-775-2009 (Malaysia)	NEG	NEG	POS	POS	POS	NEG	POS	AMB	AMB	AMB	NEG	NEG	NEG	NEG	NEG	POS	NEG	NEG
Hong Kong_138	NEG	NEG	POS	POS	POS	NEG	NEG	NEG	NEG	NEG	NEG	NEG	NEG	NEG	NEG	POS	NEG	NEG
>"South-East Asian Clad			POS	POS		NEG	NEG					NEG		POS		POS		
Kuwait_001	NEG	NEG	POS	POS	POS	NEG	NEG	NEG	NEG	NEG	NEG	NEG	NEG	POS	NEG	POS	NEG	NEG
Kuwait_337	NEG	NEG	POS	POS	POS	NEG	NEG	NEG	NEG	NEG	NEG	NEG	NEG	POS	NEG	POS	NEG	NEG
>"South-East Asian Clad			COMM	COMM		RARE	VAR					RARE		VAR		POS		
CUHK_HK2007 (China)	NEG	NEG	POS	POS	POS	NEG	POS	AMB	AMB	AMB	NEG	NEG	NEG	POS	NEG	POS	NEG	NEG
NMR02 (India)	NEG	NEG	POS	POS	POS	POS	NEG	NEG	NEG	NEG	NEG	NEG	NEG	NEG	NEG	POS	NEG	NEG
VB1490 (India)	NEG	NEG	POS	POS	POS	NEG	NEG	NEG	NEG	NEG	NEG	NEG	NEG	NEG	NEG	POS	NEG	NEG
V521 (Korea)	NEG	NEG	POS	POS	POS	NEG	POS	AMB	AMB	AMB	NEG	NEG	NEG	POS	NEG	POS	NEG	NEG
HST-077 (Lebanon)	NEG	NEG	POS	POS	POS	NEG	POS	AMB	AMB	AMB	NEG	NEG	NEG	POS	NEG	POS	NEG	NEG
MAL1 (Malaysia)	NEG	NEG	POS	POS	POS	NEG	POS	AMB	AMB	AMB	NEG	NEG	NEG	POS	NEG	POS	NEG	NEG
MAL3 (Malaysia)	NEG	NEG	POS	POS	POS	NEG	POS	AMB	AMB	AMB	NEG	NEG	NEG	POS	NEG	POS	NEG	NEG
PPUKM-261-2009 (Malaysia)	NEG	NEG	POS	POS	POS	NEG	POS	AMB	AMB	AMB	NEG	NEG	NEG	NEG	NEG	POS	NEG	NEG
PPUKM-332-2009 (Malaysia)	NEG	NEG	POS	POS	POS	NEG	POS	AMB	AMB	AMB	NEG	NEG	NEG	NEG	NEG	POS	NEG	NEG
S38 (Thailand)	NEG	NEG	POS	POS	POS	NEG	POS	AMB	AMB	AMB	NEG	NEG	NEG	POS	NEG	POS	NEG	NEG
S7 (Thailand)	NEG	NEG	POS	POS	POS	NEG	POS	AMB	AMB	AMB	NEG	NEG	NEG	POS	NEG	POS	NEG	NEG
US_008 (Thailand)	NEG	NEG	POS	POS	POS	NEG	POS	AMB	AMB	AMB	NEG	POS	NEG	POS	NEG	POS	NEG	NEG
US_030 (Thailand)	NEG	NEG	POS	POS	POS	NEG	POS	AMB	AMB	AMB	NEG	NEG	NEG	NEG	NEG	POS	NEG	NEG
M170 (Tsunami-related)	NEG	NEG	POS	POS	POS	NEG	POS	AMB	AMB	AMB	NEG	NEG	NEG	POS	NEG	POS	NEG	NEG
BSAC697 (UK)	NEG	NEG	POS	POS	POS	NEG	POS	AMB	AMB	AMB	NEG	NEG	NEG	POS	NEG	POS	NEG	NEG
IS-189	NEG	NEG	POS	POS	POS	NEG	POS	AMB	AMB	AMB	NEG	NEG	NEG	POS	NEG	POS	NEG	NEG
MRGR3	NEG	NEG	POS	POS	POS	NEG	NEG	NEG	NEG	NEG	NEG	NEG	NEG	POS	NEG	POS	NEG	NEG
smg07	NEG	NEG	POS	POS	POS	NEG	POS	AMB	AMB	AMB	NEG	NEG	NEG	NEG	NEG	POS	NEG	NEG
AH1-AUS-EMRSA-3	NEG	NEG	POS	POS	POS	NEG	AMB	POS	AMB	AMB	NEG	NEG	NEG	POS	NEG	POS	NEG	NEG
Bengalaru_ST20141403	NEG	NEG	POS	POS	POS	POS	NEG	NEG	NEG	NEG	NEG	NEG	NEG	NEG	NEG	POS	NEG	NEG
Finland E24_98541_Harmony	NEG	NEG	POS	POS	POS	NEG	AMB	AMB	AMB	AMB	NEG	NEG	NEG	NEG	NEG	POS	NEG	NEG
Hong Kong_10	NEG	NEG	POS	POS	POS	NEG	AMB	POS	AMB	AMB	NEG	NEG	NEG	POS	NEG	POS	NEG	NEG
Hong Kong_100	NEG	NEG	POS	POS	POS	NEG	NEG	NEG	NEG	NEG	NEG	NEG	NEG	NEG	NEG	POS	NEG	NEG
Hong Kong_199	NEG	NEG	POS	POS	POS	NEG	POS	AMB	AMB	AMB	NEG	NEG	NEG	POS	NEG	POS	NEG	NEG
Hong Kong_34	NEG	NEG	POS	POS	POS	NEG	NEG	NEG	NEG	NEG	NEG	NEG	NEG	NEG	NEG	POS	NEG	NEG
Hong Kong_88	NEG	NEG	NEG	NEG	NEG	NEG	AMB	POS	AMB	AMB	NEG	NEG	NEG	POS	NEG	POS	NEG	NEG
Hong Kong_92	NEG	NEG	POS	POS	POS	NEG	POS	AMB	AMB	AMB	NEG	NEG	NEG	POS	NEG	POS	NEG	NEG
Hong Kong_94	NEG	NEG	POS	POS	POS	NEG	AMB	POS	AMB	AMB	NEG	NEG	NEG	POS	POS	POS	NEG	NEG
Kuwait_257	NEG	NEG	POS	POS	POS	NEG	AMB	POS	AMB	AMB	NEG	NEG	NEG	POS	NEG	POS	NEG	NEG
Perth_01-15330	NEG	NEG	POS	POS	POS	NEG	POS	AMB	AMB	AMB	NEG	NEG	NEG	AMB	NEG	POS	NEG	NEG
Perth_01-15349	NEG	NEG	POS	POS	POS	NEG	POS	AMB	AMB	AMB	NEG	NEG	NEG	AMB	NEG	POS	NEG	NEG
Perth_01-16176	NEG	NEG	POS	AMB	POS	NEG	AMB	AMB	AMB	AMB	NEG	NEG	NEG	AMB	NEG	POS	NEG	NEG
Perth_WBG8379	NEG	NEG	POS	POS	POS	NEG	POS	AMB	AMB	AMB	NEG	NEG	NEG	POS	NEG	POS	NEG	NEG
Rawalpindi_Kidney Center_56	NEG	NEG	POS	POS	POS	NEG	AMB	POS	AMB	AMB	NEG	NEG	NEG	NEG	NEG	POS	NEG	NEG
Riyadh_SSTI_05_3464614	NEG	NEG	POS	POS	POS	NEG	POS	AMB	AMB	NEG	NEG	NEG	NEG	NEG	NEG	POS	NEG	NEG
Trinidad&Tobago_2012_157	NEG	NEG	POS	POS	POS	NEG	AMB	POS	AMB	AMB	NEG	NEG	NEG	POS	NEG	POS	NEG	NEG

STRAIN / ISOLATE	RESISTANCE : AMINOGLYSOSIDES				RESISTANCE : MISCELLANEOUS GENES													
	aadA		aadE	aphA3	sat	dfrA	dfrG				far1	mupA	mupB	tetK	tetL	tetM		
	aadA-var1	aadA-var2	aadE	aphA3	sat	dfrA	dfrG	dfrG-Tn559	dfrG-K	dfrG-D	far1	mupA	mupB	tetK	tetL	tetM (com-bined)	tetM-O	tetM-S
	aminoglycoside-3'-adenylyltransferase			3'5'-aminog-lycoside phospho-transferase, neo-/ kanamycin resistance	strepto-thricine-acetyl-transferase	dihydro-folate reductase type 1	dihydrofolate reductase				fusidic acid resistence	mupirocin resistence protein	plasmidic isoleucyl-tRNA synthase	tetracycline resistance	Tetracycline resistance gene	tetracycline resistance		
>"South-East Asian Clad			POS	POS		POS	NEG					NEG		POS		POS		
M418	NEG	NEG	POS	POS	POS	POS	NEG	NEG	NEG	NEG	NEG	NEG	NEG	POS	NEG	POS	NEG	NEG
>"South-East Asian Clad			POS	POS		VAR	VAR					NEG		NEG		POS		
NMR07 (India)	NEG	NEG	POS	POS	POS	POS	NEG	NEG	NEG	NEG	NEG	NEG	NEG	NEG	NEG	POS	NEG	NEG
NMR08 (India)	NEG	NEG	POS	POS	POS	POS	NEG	NEG	NEG	NEG	NEG	NEG	NEG	NEG	NEG	POS	NEG	NEG
Bengalaru_ST20121978	NEG	NEG	POS	POS	POS	POS	NEG	NEG	NEG	NEG	NEG	NEG	NEG	NEG	NEG	POS	NEG	NEG
Hong Kong_89	NEG	NEG	POS	POS	POS	NEG	POS	AMB	AMB	AMB	NEG	NEG	NEG	NEG	NEG	POS	NEG	NEG
>"South-East Asian Clad			POS	POS		NEG	POS					NEG		POS		POS		
Perth_2005_AH_5	NEG	NEG	POS	POS	POS	NEG	POS	AMB	AMB	AMB	NEG	NEG	NEG	POS	NEG	POS	NEG	NEG
>"South-East Asian Clad			POS	POS		NEG	VAR					NEG		NEG		POS		
Trinidad&Tobago_MRSA2010_103	NEG	NEG	POS	POS	POS	NEG	POS	AMB	AMB	NEG	NEG	NEG	NEG	NEG	NEG	POS	NEG	NEG
Trinidad&Tobago_2013_PA25	NEG	NEG	POS	POS	POS	NEG	AMB	POS	AMB	AMB	NEG	NEG	NEG	NEG	NEG	POS	NEG	NEG
Trinidad&Tobago_MRSA2010_110	NEG	NEG	POS	POS	POS	NEG	AMB	AMB	AMB	AMB	NEG	NEG	NEG	NEG	NEG	POS	NEG	NEG
Trinidad&Tobago_SSTI 2012_111	NEG	NEG	POS	POS	POS	NEG	POS	AMB	AMB	NEG	NEG	NEG	NEG	NEG	NEG	POS	NEG	NEG
Trinidad&Tobago_MRSA2010_158	NEG	NEG	POS	POS	POS	NEG	AMB	POS	AMB	AMB	NEG	NEG	NEG	NEG	NEG	POS	NEG	NEG
>"South-East Asian Clad			POS	POS		NEG	NEG					VAR		VAR		POS		
Kuwait_122	NEG	NEG	POS	POS	POS	NEG	NEG	NEG	NEG	NEG	NEG	NEG	NEG	POS	NEG	POS	NEG	NEG
Kuwait_002	NEG	NEG	POS	POS	POS	NEG	NEG	NEG	NEG	NEG	NEG	NEG	NEG	NEG	NEG	POS	NEG	NEG
Kuwait_196	NEG	NEG	POS	POS	POS	NEG	NEG	NEG	NEG	NEG	NEG	NEG	NEG	NEG	NEG	POS	NEG	NEG
Kuwait_199	NEG	NEG	POS	POS	POS	NEG	NEG	NEG	NEG	NEG	NEG	POS	NEG	NEG	NEG	POS	NEG	NEG
Hong Kong_136	NEG	NEG	POS	POS	POS	NEG	NEG	NEG	NEG	NEG	NEG	NEG	NEG	POS	NEG	POS	NEG	NEG
>"South-East Asian Clad			POS	POS		NEG	NEG					NEG		POS		POS		
P32 (Poland)	NEG	NEG	POS	POS	POS	NEG	NEG	NEG	NEG	NEG	NEG	NEG	NEG	POS	NEG	POS	NEG	NEG
>"South-East Asian Clad			POS	POS		NEG	POS					NEG		POS		POS		
Perth_2015-S32313	NEG	NEG	POS	POS	POS	NEG	POS	AMB	AMB	AMB	NEG	NEG	NEG	POS	NEG	POS	NEG	NEG
>"hla-negative South-Ea			POS	POS		NEG	POS					NEG		NEG		POS		
DEN907 (Denmark)	NEG	NEG	POS	POS	POS	NEG	POS	AMB	AMB	AMB	NEG	NEG	NEG	NEG	NEG	POS	NEG	NEG
>"hla-negative South-Ea			POS	POS		NEG	POS					VAR		NEG		POS		
S26 (Thailand)	NEG	NEG	POS	POS	POS	NEG	POS	AMB	AMB	AMB	NEG	NEG	NEG	NEG	NEG	POS	NEG	NEG
S42 (Thailand)	NEG	NEG	POS	POS	POS	NEG	POS	AMB	AMB	AMB	NEG	NEG	NEG	NEG	NEG	POS	NEG	NEG
S97 (Thailand)	NEG	NEG	POS	POS	POS	NEG	POS	AMB	AMB	AMB	NEG	POS	NEG	NEG	NEG	POS	NEG	NEG
S78 (Thailand)	NEG	NEG	POS	POS	POS	NEG	POS	AMB	AMB	AMB	NEG	NEG	NEG	NEG	NEG	POS	NEG	NEG
S25 (Thailand)	NEG	NEG	POS	POS	POS	NEG	POS	AMB	AMB	AMB	NEG	POS	NEG	NEG	NEG	POS	NEG	NEG
S21 (Thailand)	NEG	NEG	POS	POS	POS	NEG	POS	AMB	AMB	AMB	NEG	NEG	NEG	NEG	NEG	POS	NEG	NEG
S106 (Thailand)	NEG	NEG	POS	POS	POS	NEG	POS	AMB	AMB	AMB	NEG	NEG	NEG	NEG	NEG	POS	NEG	NEG
S24 (Thailand)	NEG	NEG	POS	POS	POS	NEG	POS	AMB	AMB	AMB	NEG	NEG	NEG	NEG	NEG	POS	NEG	NEG
S39 (Thailand)	NEG	NEG	POS	POS	POS	NEG	POS	AMB	AMB	AMB	NEG	NEG	NEG	NEG	NEG	POS	NEG	NEG
S2 (Thailand)	NEG	NEG	POS	POS	POS	NEG	POS	AMB	AMB	AMB	NEG	POS	NEG	NEG	NEG	POS	NEG	NEG
S81 (Thailand)	NEG	NEG	POS	POS	POS	NEG	POS	AMB	AMB	AMB	NEG	NEG	NEG	NEG	NEG	POS	NEG	NEG
M116 (Vietnam)	NEG	NEG	POS	POS	POS	NEG	POS	AMB	AMB	AMB	NEG	NEG	NEG	NEG	NEG	POS	NEG	NEG
>"Portuguese Clade": Pc			VAR	COMM		NEG	NEG					NEG		VAR		POS		
FFP103 (Portugal)	NEG	NEG	NEG	POS	POS	NEG	NEG	NEG	NEG	NEG	NEG	NEG	NEG	POS	NEG	POS	NEG	NEG
HDG2 (Portugal)	NEG	NEG	NEG	POS	POS	NEG	NEG	NEG	NEG	NEG	NEG	NEG	NEG	NEG	NEG	POS	NEG	NEG
HSA10 (Portugal)	NEG	NEG	NEG	POS	POS	NEG	NEG	NEG	NEG	NEG	NEG	NEG	NEG	POS	NEG	POS	NEG	NEG
HSA11 (Portugal)	NEG	NEG	NEG	POS	POS	NEG	NEG	NEG	NEG	NEG	NEG	NEG	NEG	POS	NEG	POS	NEG	NEG
ICP5011 (Portugal)	NEG	NEG	NEG	POS	POS	NEG	NEG	NEG	NEG	NEG	NEG	NEG	NEG	POS	NEG	POS	NEG	NEG
ICP5014 (Portugal)	NEG	NEG	NEG	POS	POS	NEG	NEG	NEG	NEG	NEG	NEG	NEG	NEG	POS	NEG	POS	NEG	NEG
ICP5062 (Portugal)	NEG	NEG	NEG	POS	POS	NEG	NEG	NEG	NEG	NEG	NEG	NEG	NEG	POS	NEG	POS	NEG	NEG
Strain 1063_ATCC 33592 FDAARGOS_3 (USA)	NEG	NEG	POS	POS	POS	NEG	NEG	NEG	NEG	NEG	NEG	NEG	NEG	POS	NEG	POS	NEG	NEG
	NEG	NEG	POS	POS	POS	NEG	NEG	NEG	NEG	NEG	NEG	NEG	NEG	POS	NEG	POS	NEG	NEG
Russia-07_0110_Kurgan	NEG	NEG	NEG	NEG	NEG	NEG	NEG	NEG	NEG	NEG	NEG	NEG	NEG	NEG	NEG	POS	NEG	NEG
Russia-11_0164_Kurgan	NEG	NEG	POS	POS	POS	NEG	NEG	NEG	NEG	NEG	NEG	NEG	NEG	POS	NEG	POS	NEG	NEG
Russia-20_0367_Kurgan	NEG	NEG	POS	POS	POS	NEG	NEG	NEG	NEG	NEG	NEG	NEG	NEG	NEG	NEG	POS	NEG	NEG
Russia-21_0390_Chelyabinsk	NEG	NEG	POS	POS	POS	NEG	NEG	NEG	NEG	NEG	NEG	NEG	NEG	POS	NEG	POS	NEG	NEG
>"South American/ Mid			COMM	COMM		RARE	RARE					NEG		RARE		POS		
AGT1 (Argentina)	NEG	NEG	POS	POS	POS	NEG	NEG	NEG	NEG	NEG	NEG	NEG	NEG	NEG	NEG	POS	NEG	NEG
AGT120 (Argentina)	NEG	NEG	POS	POS	POS	NEG	NEG	NEG	NEG	NEG	NEG	NEG	NEG	NEG	NEG	POS	NEG	NEG
AGT67 (Argentina)	NEG	NEG	POS	POS	POS	NEG	NEG	NEG	NEG	NEG	NEG	NEG	NEG	NEG	NEG	POS	NEG	NEG
AGT9 (Argentina)	NEG	NEG	POS	POS	POS	NEG	NEG	NEG	NEG	NEG	NEG	NEG	NEG	NEG	NEG	POS	NEG	NEG
RA3 (Argentina)	NEG	NEG	NEG	NEG	NEG	NEG	NEG	NEG	NEG	NEG	NEG	NEG	NEG	NEG	NEG	POS	NEG	NEG
RA6 (Argentina)	NEG	NEG	POS	POS	POS	NEG	NEG	NEG	NEG	NEG	NEG	NEG	NEG	NEG	NEG	POS	NEG	NEG
RA7 (Argentina)	NEG	NEG	POS	POS	POS	NEG	NEG	NEG	NEG	NEG	NEG	NEG	NEG	NEG	NEG	POS	NEG	NEG
Be62 (Brazil)	NEG	NEG	POS	POS	POS	NEG	NEG	NEG	NEG	NEG	NEG	NEG	NEG	NEG	NEG	POS	NEG	NEG
Bmb9393 (Brazil)	NEG	NEG	POS	POS	POS	NEG	NEG	NEG	NEG	NEG	NEG	NEG	NEG	NEG	NEG	POS	NEG	NEG
BRA36 (Brazil)	NEG	NEG	POS	POS	POS	NEG	NEG	NEG	NEG	NEG	NEG	NEG	NEG	NEG	NEG	POS	NEG	NEG
BZ48 (Brazil)	NEG	NEG	POS	POS	POS	NEG	NEG	NEG	NEG	NEG	NEG	NEG	NEG	NEG	NEG	POS	NEG	NEG
Gv51 (Brazil)	NEG	NEG	POS	POS	POS	NEG	NEG	NEG	NEG	NEG	NEG	NEG	NEG	NEG	NEG	POS	NEG	NEG
Gv69 (Brazil)	NEG	NEG	POS	POS	POS	NEG	NEG	NEG	NEG	NEG	NEG	NEG	NEG	NEG	NEG	POS	NEG	NEG
Gv88 (Brazil)	NEG	NEG	POS	POS	POS	NEG	NEG	NEG	NEG	NEG	NEG	NEG	NEG	NEG	NEG	POS	NEG	NEG
HC1335 (Brazil)	NEG	NEG	POS	POS	POS	NEG	NEG	NEG	NEG	NEG	NEG	NEG	NEG	NEG	NEG	POS	NEG	NEG
HC1340 (Brazil)	NEG	NEG	POS	POS	POS	NEG	NEG	NEG	NEG	NEG	NEG	NEG	NEG	NEG	NEG	POS	NEG	NEG
HC556 (Brazil)	NEG	NEG	POS	POS	POS	NEG	NEG	NEG	NEG	NEG	NEG	NEG	NEG	NEG	NEG	POS	NEG	NEG
HU25 (Brazil)	NEG	NEG	POS	POS	POS	NEG	NEG	NEG	NEG	NEG	NEG	NEG	NEG	NEG	NEG	POS	NEG	NEG
UB563 (Brazil)	NEG	NEG	POS	POS	POS	NEG	NEG	NEG	NEG	NEG	NEG	NEG	NEG	NEG	NEG	POS	NEG	NEG
2A8 (Czech Republic)	NEG	NEG	POS	POS	POS	POS	NEG	NEG	NEG	NEG	NEG	NEG	NEG	NEG	NEG	POS	NEG	NEG
M1229 (Denmark)	NEG	NEG	NEG	NEG	NEG	NEG	POS	AMB	AMB	AMB	NEG	NEG	NEG	NEG	NEG	POS	NEG	NEG
FRICAR (France)	NEG	NEG	POS	POS	POS	NEG	NEG	NEG	NEG	NEG	NEG	NEG	NEG	NEG	NEG	POS	NEG	NEG
D90 (Germany)	NEG	NEG	NEG	NEG	NEG	NEG	POS	AMB	AMB	AMB	NEG	NEG	NEG	POS	NEG	POS	NEG	NEG
LIT68 (Lithuania)	NEG	NEG	NEG	NEG	NEG	NEG	NEG	NEG	NEG	NEG	NEG	NEG	NEG	POS	NEG	POS	NEG	NEG
LIT76 (Lithuania)	NEG	NEG	NEG	NEG	NEG	NEG	NEG	NEG	NEG	NEG	NEG	NEG	NEG	POS	NEG	POS	NEG	NEG
HGSA142 (Portugal)	NEG	NEG	POS	POS	POS	NEG	NEG	NEG	NEG	NEG	NEG	NEG	NEG	NEG	NEG	POS	NEG	NEG
HGSA9 (Portugal)	NEG	NEG	POS	POS	POS	NEG	NEG	NEG	NEG	NEG	NEG	NEG	NEG	NEG	NEG	POS	NEG	NEG
HSJ216 (Portugal)	NEG	NEG	POS	POS	POS	NEG	NEG	NEG	NEG	NEG	NEG	NEG	NEG	NEG	NEG	POS	NEG	NEG
M278 (Portugal)	NEG	NEG	POS	POS	POS	NEG	NEG	NEG	NEG	NEG	NEG	NEG	NEG	NEG	NEG	POS	NEG	NEG
ES26 (Spain)	NEG	NEG	POS	POS	POS	NEG	NEG	NEG	NEG	NEG	NEG	NEG	NEG	NEG	NEG	POS	NEG	NEG
UK102 (UK)	NEG	NEG	NEG	NEG	NEG	NEG	POS	AMB	AMB	AMB	NEG	NEG	NEG	POS	NEG	POS	NEG	NEG
URU34 (Uruguay)	NEG	NEG	POS	POS	POS	NEG	NEG	NEG	NEG	NEG	NEG	NEG	NEG	NEG	POS	POS	NEG	NEG
IS-125	NEG	NEG	POS	POS	POS	NEG	NEG	NEG	NEG	NEG	NEG	NEG	NEG	NEG	NEG	POS	NEG	NEG
IS-157	NEG	NEG	POS	POS	POS	NEG	NEG	NEG	NEG	NEG	NEG	NEG	NEG	NEG	NEG	POS	NEG	NEG
Ecuador_1	NEG	NEG	POS	POS	POS	NEG	NEG	NEG	NEG	NEG	NEG	NEG	NEG	NEG	NEG	POS	NEG	NEG
Ecuador_2	NEG	NEG	POS	POS	POS	NEG	NEG	NEG	NEG	NEG	NEG	NEG	NEG	NEG	NEG	POS	NEG	NEG
Dresden_15ANR574263	NEG	NEG	POS	POS	POS	NEG	NEG	NEG	NEG	NEG	NEG	NEG	NEG	NEG	NEG	POS	NEG	NEG
UK-EMRSA-11	NEG	NEG	POS	POS	POS	POS	NEG	NEG	NEG	NEG	NEG	NEG	NEG	NEG	NEG	POS	NEG	NEG
>"South American/ Mid			POS	POS		NEG	NEG					NEG		NEG		POS		
BRA2 (Brazil)	NEG	NEG	POS	POS	POS	NEG	NEG	NEG	NEG	NEG	NEG	NEG	NEG	NEG	NEG	POS	NEG	NEG
CHL1 (Chile)	NEG	NEG	POS	POS	POS	NEG	NEG	NEG	NEG	NEG	NEG	NEG	NEG	NEG	NEG	POS	NEG	NEG
CHL151 (Chile)	NEG	NEG	POS	POS	POS	NEG	NEG	NEG	NEG	NEG	NEG	NEG	NEG	NEG	NEG	POS	NEG	NEG
UCL378 (Chile)	NEG	NEG	POS	POS	POS	NEG	NEG	NEG	NEG	NEG	NEG	NEG	NEG	NEG	NEG	POS	NEG	NEG
UP72 (Peru)	NEG	NEG	POS	POS	POS	NEG	NEG	NEG	NEG	NEG	NEG	NEG	NEG	NEG	NEG	POS	NEG	NEG
UP81 (Peru)	NEG	NEG	POS	POS	POS	NEG	NEG	NEG	NEG	NEG	NEG	NEG	NEG	NEG	NEG	POS	NEG	NEG
>"South American/ Mid			NEG	NEG		NEG	NEG					NEG		NEG		POS		
UP1073 (Peru)	NEG	NEG	NEG	NEG	NEG	NEG	NEG	NEG	NEG	NEG	NEG	NEG	NEG	NEG	NEG	POS	NEG	NEG

STRAIN / ISOLATE	RESISTANCE : AMINOGLYSOSIDES				RESISTANCE : MISCELLANEOUS GENES													
	aadA		aadE	aphA3	sat	dfrA	dfrG				far1	mupA	mupB	tetK	tetL	tetM		
	aadA-var1	aadA-var2	aadE	aphA3	sat	dfrA	dfrG	dfrG-Tn559	dfrG-K	dfrG-D	far1	mupA	mupB	tetK	tetL	tetM (com-bined)	tetM-O	tetM-S
	aminoglycoside-3'-adenylyltransferase			3'5'-aminog-lycoside phospho-transferase, neo-/ kanamycin resistance	strepto-thricine-acetyl-transferase	dihydro-folate reductase type 1	dihydrofolate reductase				fusidic acid resistence	mupirocin resistence protein	plasmidic isoleucyl-tRNA synthase	tetracycline resistance	Tetracycline resistance gene	tetracycline resistance		
>"South American/ Mid			NEG	NEG		NEG	VAR					NEG		NEG		POS		
LIT89 (Lithuania)	NEG	NEG	NEG	NEG	NEG	NEG	NEG	NEG	NEG	NEG	NEG	NEG	NEG	NEG	NEG	POS	NEG	NEG
MRSA_PR1 (Malaysia)	NEG	NEG	NEG	NEG	NEG	NEG	POS	AMB	AMB	AMB	NEG	NEG	NEG	NEG	NEG	POS	NEG	NEG
Riyadh-2888905-R	NEG	NEG	NEG	NEG	NEG	NEG	AMB	AMB	AMB	AMB	NEG	NEG	NEG	NEG	NEG	POS	NEG	NEG
Riyadh-2888915-BC	NEG	NEG	NEG	NEG	NEG	NEG	POS	AMB	AMB	AMB	NEG	NEG	NEG	NEG	NEG	POS	NEG	NEG
Riyadh-2793706-R	NEG	NEG	NEG	NEG	NEG	NEG	AMB	AMB	AMB	AMB	NEG	NEG	NEG	NEG	NEG	POS	NEG	NEG
>"South American/ Mid			NEG	NEG		NEG	pos					NEG		NEG		POS		
Riyadh_SSTI_18_3502925	NEG	NEG	NEG	NEG	NEG	NEG	AMB	AMB	AMB	AMB	NEG	NEG	NEG	NEG	NEG	POS	NEG	NEG
Riyadh-2822825-W	NEG	NEG	NEG	NEG	NEG	NEG	AMB	POS	AMB	NEG	NEG	NEG	NEG	NEG	NEG	POS	NEG	NEG
>"South American/ Mid			NEG	NEG		NEG	POS					NEG		POS		POS		
UK-EMRSA-9	NEG	NEG	NEG	NEG	NEG	NEG	POS	AMB	AMB	AMB	NEG	NEG	NEG	POS	NEG	POS	NEG	NEG
>"South American/ Mid			NEG	NEG		NEG	NEG					NEG		POS		POS		
Lome_HT20020815	NEG	NEG	NEG	NEG	NEG	NEG	NEG	NEG	NEG	NEG	NEG	NEG	NEG	POS	NEG	POS	NEG	NEG
>"South American/ Mid			RARE	RARE		RARE	VAR					NEG		VAR		POS		
NA32 (Denmark)	NEG	NEG	NEG	NEG	NEG	NEG	NEG	NEG	NEG	NEG	NEG	NEG	NEG	POS	NEG	POS	NEG	NEG
H24 (Egypt)	NEG	NEG	POS	POS	POS	POS	NEG	NEG	NEG	NEG	NEG	NEG	NEG	POS	NEG	POS	NEG	NEG
ATCC BAA-39 (=HUSA304) (Hungary)	NEG	NEG	NEG	NEG	NEG	NEG	NEG	NEG	NEG	NEG	NEG	NEG	NEG	POS	NEG	POS	NEG	NEG
HUSA304 (Hungary)	NEG	NEG	NEG	NEG	NEG	NEG	NEG	NEG	NEG	NEG	NEG	NEG	NEG	POS	NEG	POS	NEG	NEG
HU106 (Hungary)	NEG	NEG	NEG	NEG	NEG	NEG	NEG	NEG	NEG	NEG	NEG	NEG	NEG	POS	NEG	POS	NEG	NEG
BSAC27 (UK)	NEG	NEG	NEG	NEG	NEG	NEG	POS	AMB	AMB	AMB	NEG	NEG	NEG	POS	NEG	POS	NEG	NEG
NCTC13131, UK-EMRSA-4 (UK)	NEG	NEG	NEG	NEG	NEG	NEG	NEG	NEG	NEG	NEG	NEG	NEG	NEG	POS	NEG	POS	NEG	NEG
	NEG	NEG	NEG	NEG	NEG	NEG	NEG	NEG	NEG	NEG	NEG	NEG	NEG	POS	NEG	POS	NEG	NEG
Algiers_HT20040080	NEG	NEG	POS	POS	POS	NEG	NEG	NEG	NEG	NEG	NEG	NEG	NEG	POS	NEG	POS	NEG	NEG
Dublin-DSH_AR09_0.0066	NEG	NEG	POS	POS	POS	NEG	NEG	NEG	NEG	NEG	NEG	NEG	NEG	POS	NEG	POS	NEG	NEG
Dublin-DSH_AR09_0-0065	NEG	NEG	NEG	NEG	NEG	NEG	NEG	NEG	NEG	NEG	NEG	NEG	NEG	AMB	NEG	POS	NEG	NEG
Dublin-DSH_Phenotype-III_84	NEG	NEG	POS	POS	POS	NEG	NEG	NEG	NEG	NEG	NEG	NEG	NEG	AMB	NEG	POS	NEG	NEG
Hong Kong_130	NEG	NEG	NEG	NEG	NEG	NEG	NEG	NEG	NEG	NEG	NEG	NEG	NEG	POS	NEG	POS	NEG	NEG
Kuwait_018	NEG	NEG	NEG	NEG	NEG	NEG	POS	AMB	AMB	NEG	NEG	NEG	NEG	POS	NEG	POS	NEG	NEG
Perth_08-17726	NEG	NEG	NEG	NEG	NEG	NEG	POS	AMB	AMB	AMB	NEG	NEG	NEG	NEG	NEG	POS	NEG	NEG
Riyadh_Alfaisal/KKKSUH_86_MRS-14-279	NEG	NEG	NEG	NEG	NEG	NEG	POS	AMB	AMB	NEG	NEG	NEG	NEG	NEG	NEG	POS	NEG	NEG
Riyadh_Alfaisal-04_Z3861_831588	NEG	NEG	NEG	NEG	NEG	NEG	POS	AMB	AMB	AMB	NEG	NEG	NEG	NEG	NEG	POS	NEG	NEG
Riyadh_Alfaisal-30_S15724_1108013	NEG	NEG	NEG	NEG	NEG	NEG	POS	AMB	AMB	AMB	NEG	NEG	NEG	NEG	NEG	POS	NEG	NEG
Riyadh_Alfaisal-6_22A_13_B3992_397721	NEG	NEG	NEG	NEG	NEG	NEG	POS	AMB	AMB	NEG	NEG	NEG	NEG	POS	NEG	POS	NEG	NEG
Riyadh_SSTI_52_3615482	NEG	NEG	NEG	NEG	NEG	NEG	AMB	AMB	AMB	AMB	NEG	NEG	NEG	NEG	NEG	POS	NEG	NEG
Riyadh-2817276-2	NEG	NEG	NEG	NEG	NEG	NEG	POS	AMB	AMB	AMB	NEG	NEG	NEG	POS	NEG	POS	NEG	NEG
Riyadh-2891670-W	NEG	NEG	NEG	NEG	NEG	NEG	POS	AMB	AMB	AMB	NEG	NEG	NEG	NEG	NEG	POS	NEG	NEG
Riyadh-3006920-W	NEG	NEG	NEG	NEG	NEG	NEG	POS	AMB	AMB	AMB	NEG	NEG	NEG	NEG	NEG	POS	NEG	NEG
Riyadh-R2567782	NEG	NEG	NEG	NEG	NEG	NEG	POS	AMB	AMB	AMB	NEG	NEG	NEG	NEG	NEG	POS	NEG	NEG
Russia-18_0252_Krasnoyarsk_SK2	NEG	NEG	NEG	NEG	NEG	NEG	NEG	NEG	NEG	NEG	NEG	NEG	NEG	POS	NEG	POS	NEG	NEG
UK-EMRSA-7	NEG	NEG	NEG	NEG	NEG	NEG	NEG	NEG	NEG	NEG	NEG	NEG	NEG	POS	NEG	POS	NEG	NEG
>"South American/ Mid			POS	POS		NEG	NEG					NEG		RARE		POS		
Dublin-DSH_AR23_0073	NEG	NEG	POS	POS	POS	NEG	NEG	NEG	NEG	NEG	NEG	NEG	NEG	AMB	NEG	POS	NEG	NEG
>Related to "South Ame			NEG	NEG		NEG	NEG					NEG		NEG		POS		
MRSA-OC3 (Russia)	NEG	NEG	NEG	NEG	NEG	NEG	NEG	NEG	NEG	NEG	NEG	NEG	NEG	NEG	NEG	POS	NEG	NEG
Russia-12_0176_Krasnoyarsk	NEG	NEG	NEG	NEG	NEG	NEG	NEG	NEG	NEG	NEG	NEG	NEG	NEG	NEG	NEG	POS	NEG	NEG
Russia-13_0180_Krasnoyarsk	NEG	NEG	NEG	NEG	NEG	NEG	NEG	NEG	NEG	NEG	NEG	NEG	NEG	NEG	NEG	POS	NEG	NEG
Russia-16_0249_Krasnoyarsk	NEG	NEG	NEG	NEG	NEG	NEG	NEG	NEG	NEG	NEG	NEG	NEG	NEG	NEG	NEG	POS	NEG	NEG
Russia-17_0250_Krasnoyarsk	NEG	NEG	NEG	NEG	NEG	NEG	NEG	NEG	NEG	NEG	NEG	NEG	NEG	NEG	NEG	POS	NEG	NEG
>Related to "South Ame			NEG	NEG		NEG	NEG					NEG		POS		POS		
URU110 (Uruguay)	NEG	NEG	NEG	NEG	NEG	NEG	NEG	NEG	NEG	NEG	NEG	NEG	NEG	POS	NEG	POS	NEG	NEG
>Related to "South Ame			NEG	NEG		NEG	POS					NEG		NEG		POS		
DS_014 (Thailand)	NEG	NEG	NEG	NEG	NEG	NEG	POS	AMB	AMB	AMB	NEG	NEG	NEG	NEG	NEG	POS	NEG	NEG
>Unassigned Middle Eas			POS	POS		NEG	NEG					NEG		POS		POS		
Frankfurt_Oder_0490031797 (Libya)	NEG	NEG	POS	POS	POS	NEG	NEG	NEG	NEG	NEG	NEG	NEG	NEG	POS	NEG	POS	NEG	NEG
Kuwait_192	NEG	NEG	POS	POS	POS	NEG	NEG	NEG	NEG	NEG	NEG	NEG	NEG	POS	NEG	POS	NEG	NEG
Riyadh-9	NEG	NEG	POS	POS	POS	NEG	NEG	NEG	NEG	NEG	NEG	NEG	NEG	POS	NEG	POS	NEG	NEG
Russia-24_0407_Moscow	NEG	NEG	POS	POS	POS	NEG	NEG	NEG	NEG	NEG	NEG	NEG	NEG	POS	NEG	POS	NEG	NEG

STRAIN / ISOLATE	RESISTANCE : MISCELLANEOUS GENES																	
	cat					cfr	fexA	apmA	fosB			qacA	qacC					
	cat	cat (pC221)	cat (pc223)	cat (pMC524)	cat (pSBK203R)	cfr	fexA	apmA	fosB	fosB (Sint)	fosB (plasmid 1)	qacA	qacC	qacC (cons)	qacC (equine)	qacC (SA5)	qacC (Ssap)	qacC (ST94)
	chloramphenicol acetyltransferase					23S rRNA methyltransferase	chloramphenicol/florfenicol exporter	aminocyclitol acetyltransferase, confers apramycin resistance	metallothiol transferase			quaternary ammonium compound resistance protein A	quaternary ammonium compound resistance protein C					
>"Eurasian Clade": TUR1	VAR											NEG	POS					
TUR1 (Turkey)	POS	NEG	NEG	POS	NEG	NEG	NEG	NEG	POS	NEG	NEG	NEG	POS	POS	NEG	NEG	NEG	NEG
TUR9 (Turkey)	NEG	NEG	NEG	NEG	NEG	NEG	NEG	NEG	POS	NEG	NEG	NEG	POS	POS	NEG	NEG	NEG	NEG
>"Eurasian Clade" : T013	NEG											NEG	NEG					
T0131 (China)	NEG	NEG	NEG	NEG	NEG	NEG	NEG	NEG	POS	NEG	NEG	NEG	NEG	NEG	NEG	NEG	NEG	NEG
MU4 (Turkey)	NEG	NEG	NEG	NEG	NEG	NEG	NEG	NEG	POS	NEG	NEG	NEG	POS	POS	NEG	NEG	NEG	NEG
DEU11 (Turkey)	NEG	NEG	NEG	NEG	NEG	NEG	NEG	NEG	POS	NEG	NEG	NEG	POS	POS	NEG	NEG	NEG	NEG
DEU16 (Turkey)	NEG	NEG	NEG	NEG	NEG	NEG	NEG	NEG	POS	NEG	NEG	NEG	NEG	NEG	NEG	NEG	NEG	NEG
Romania_Iasi_MRSA-07_54	NEG	NEG	NEG	NEG	NEG	NEG	NEG	NEG	POS	NEG	NEG	NEG	NEG	NEG	NEG	NEG	NEG	NEG
>"Eurasian Clade": arsC-	NEG											NEG	NEG					
Romania_Iasi_MRSA-05_46	NEG	NEG	NEG	NEG	NEG	NEG	NEG	NEG	POS	NEG	NEG	NEG	NEG	NEG	NEG	NEG	NEG	NEG
Romania_Iasi_MRSA-06_47	NEG	NEG	NEG	NEG	NEG	NEG	NEG	NEG	POS	NEG	NEG	NEG	NEG	NEG	NEG	NEG	NEG	NEG
>"Eurasian Clade": 16K/	RARE											NEG	VAR					
CN79 (China)	NEG	NEG	NEG	NEG	NEG	NEG	NEG	NEG	POS	NEG	NEG	NEG	NEG	NEG	NEG	NEG	NEG	NEG
CUHK_BJ2002 (China)	NEG	NEG	NEG	NEG	NEG	NEG	NEG	NEG	POS	NEG	NEG	NEG	NEG	NEG	NEG	NEG	NEG	NEG
CUHK_BJ2007 (China)	NEG	NEG	NEG	NEG	NEG	NEG	NEG	NEG	POS	NEG	NEG	NEG	NEG	NEG	NEG	NEG	NEG	NEG
3HK (Czech Republic)	NEG	NEG	NEG	NEG	NEG	NEG	NEG	NEG	POS	NEG	NEG	NEG	POS	POS	NEG	NEG	NEG	NEG
HU109 (Hungary)	POS	NEG	NEG	POS	NEG	NEG	NEG	NEG	POS	NEG	NEG	NEG	POS	POS	NEG	NEG	NEG	NEG
HUR18 (Hungary)	NEG	NEG	NEG	NEG	NEG	NEG	NEG	NEG	POS	NEG	NEG	NEG	NEG	NEG	NEG	NEG	NEG	NEG
H482 (Romania)	NEG	NEG	NEG	NEG	NEG	NEG	NEG	NEG	POS	NEG	NEG	NEG	NEG	NEG	NEG	NEG	NEG	NEG
16K (Russia)	POS	NEG	NEG	POS	NEG	NEG	NEG	NEG	POS	NEG	NEG	NEG	POS	POS	NEG	NEG	NEG	NEG
DEU10 (Turkey)	NEG	NEG	NEG	NEG	NEG	NEG	NEG	NEG	POS	NEG	NEG	NEG	POS	POS	NEG	NEG	NEG	NEG
DEU12 (Turkey)	NEG	NEG	NEG	NEG	NEG	NEG	NEG	NEG	POS	NEG	NEG	NEG	POS	POS	NEG	NEG	NEG	NEG
DEU14 (Turkey)	NEG	NEG	NEG	NEG	NEG	NEG	NEG	NEG	POS	NEG	NEG	NEG	POS	POS	NEG	NEG	NEG	NEG
DEU15 (Turkey)	NEG	NEG	NEG	NEG	NEG	NEG	NEG	NEG	POS	NEG	NEG	NEG	POS	POS	NEG	NEG	NEG	NEG
DEU17 (Turkey)	NEG	NEG	NEG	NEG	NEG	NEG	NEG	NEG	POS	NEG	NEG	NEG	POS	POS	NEG	NEG	NEG	NEG
DEU19 (Turkey)	NEG	NEG	NEG	NEG	NEG	NEG	NEG	NEG	POS	NEG	NEG	NEG	NEG	NEG	NEG	NEG	NEG	NEG
DEU2 (Turkey)	NEG	NEG	NEG	NEG	NEG	NEG	NEG	NEG	POS	NEG	NEG	NEG	POS	POS	NEG	NEG	NEG	NEG
DEU20 (Turkey)	NEG	NEG	NEG	NEG	NEG	NEG	NEG	NEG	POS	NEG	NEG	NEG	POS	POS	NEG	NEG	NEG	NEG
DEU23 (Turkey)	NEG	NEG	NEG	NEG	NEG	NEG	NEG	NEG	POS	NEG	NEG	NEG	POS	POS	NEG	NEG	NEG	NEG
DEU3 (Turkey)	NEG	NEG	NEG	NEG	NEG	NEG	NEG	NEG	POS	NEG	NEG	NEG	POS	POS	NEG	NEG	NEG	NEG
DEU5 (Turkey)	NEG	NEG	NEG	NEG	NEG	NEG	NEG	NEG	POS	NEG	NEG	NEG	NEG	NEG	NEG	NEG	NEG	NEG
DEU6 (Turkey)	NEG	NEG	NEG	NEG	NEG	NEG	NEG	NEG	POS	NEG	NEG	NEG	POS	POS	NEG	NEG	NEG	NEG
DEU8 (Turkey)	NEG	NEG	NEG	NEG	NEG	NEG	NEG	NEG	POS	NEG	NEG	NEG	POS	POS	NEG	NEG	NEG	NEG
HU11 (Turkey)	NEG	NEG	NEG	NEG	NEG	NEG	NEG	NEG	POS	NEG	NEG	NEG	NEG	NEG	NEG	NEG	NEG	NEG
HU13 (Turkey)	NEG	NEG	NEG	NEG	NEG	NEG	NEG	NEG	POS	NEG	NEG	NEG	NEG	NEG	NEG	NEG	NEG	NEG
HU14 (Turkey)	NEG	NEG	NEG	NEG	NEG	NEG	NEG	NEG	POS	NEG	NEG	NEG	NEG	NEG	NEG	NEG	NEG	NEG
HU15 (Turkey)	NEG	NEG	NEG	NEG	NEG	NEG	NEG	NEG	POS	NEG	NEG	NEG	NEG	NEG	NEG	NEG	NEG	NEG
HU16 (Turkey)	NEG	NEG	NEG	NEG	NEG	NEG	NEG	NEG	POS	NEG	NEG	NEG	NEG	NEG	NEG	NEG	NEG	NEG
HU17 (Turkey)	NEG	NEG	NEG	NEG	NEG	NEG	NEG	NEG	POS	NEG	NEG	NEG	NEG	NEG	NEG	NEG	NEG	NEG
HU21 (Turkey)	NEG	NEG	NEG	NEG	NEG	NEG	NEG	NEG	POS	NEG	NEG	NEG	NEG	NEG	NEG	NEG	NEG	NEG
HU23 (Turkey)	NEG	NEG	NEG	NEG	NEG	NEG	NEG	NEG	POS	NEG	NEG	NEG	NEG	NEG	NEG	NEG	NEG	NEG
HU26 (Turkey)	NEG	NEG	NEG	NEG	NEG	NEG	NEG	NEG	POS	NEG	NEG	NEG	NEG	NEG	NEG	NEG	NEG	NEG
HU4 (Turkey)	NEG	NEG	NEG	NEG	NEG	NEG	NEG	NEG	POS	NEG	NEG	NEG	NEG	NEG	NEG	NEG	NEG	NEG
HU5 (Turkey)	NEG	NEG	NEG	NEG	NEG	NEG	NEG	NEG	POS	NEG	NEG	NEG	NEG	NEG	NEG	NEG	NEG	NEG
HU6 (Turkey)	NEG	NEG	NEG	NEG	NEG	NEG	NEG	NEG	POS	NEG	NEG	NEG	POS	POS	NEG	NEG	NEG	NEG
HU7 (Turkey)	NEG	NEG	NEG	NEG	NEG	NEG	NEG	NEG	POS	NEG	NEG	NEG	NEG	NEG	NEG	NEG	NEG	NEG
HU8 (Turkey)	NEG	NEG	NEG	NEG	NEG	NEG	NEG	NEG	POS	NEG	NEG	NEG	POS	POS	NEG	NEG	NEG	NEG
HU9 (Turkey)	NEG	NEG	NEG	NEG	NEG	NEG	NEG	NEG	POS	NEG	NEG	NEG	NEG	NEG	NEG	NEG	NEG	NEG
IU1 (Turkey)	NEG	NEG	NEG	NEG	NEG	NEG	NEG	NEG	POS	NEG	NEG	NEG	POS	POS	NEG	NEG	NEG	NEG
IU10 (Turkey)	NEG	NEG	NEG	NEG	NEG	NEG	NEG	NEG	POS	NEG	NEG	NEG	NEG	NEG	NEG	NEG	NEG	NEG
IU11 (Turkey)	NEG	NEG	NEG	NEG	NEG	NEG	NEG	NEG	POS	NEG	NEG	NEG	NEG	NEG	NEG	NEG	NEG	NEG
IU12 (Turkey)	NEG	NEG	NEG	NEG	NEG	NEG	NEG	NEG	POS	NEG	NEG	NEG	NEG	NEG	NEG	NEG	NEG	NEG
IU13 (Turkey)	NEG	NEG	NEG	NEG	NEG	NEG	NEG	NEG	POS	NEG	NEG	NEG	NEG	NEG	NEG	NEG	NEG	NEG
IU15 (Turkey)	NEG	NEG	NEG	NEG	NEG	NEG	NEG	NEG	POS	NEG	NEG	NEG	NEG	NEG	NEG	NEG	NEG	NEG
IU18 (Turkey)	NEG	NEG	NEG	NEG	NEG	NEG	NEG	NEG	POS	NEG	NEG	NEG	NEG	NEG	NEG	NEG	NEG	NEG
IU19 (Turkey)	POS	NEG	NEG	POS	NEG	NEG	NEG	NEG	POS	NEG	NEG	NEG	NEG	NEG	NEG	NEG	NEG	NEG
IU2 (Turkey)	NEG	NEG	NEG	NEG	NEG	NEG	NEG	NEG	POS	NEG	NEG	NEG	NEG	NEG	NEG	NEG	NEG	NEG
IU4 (Turkey)	NEG	NEG	NEG	NEG	NEG	NEG	NEG	NEG	POS	NEG	NEG	NEG	NEG	NEG	NEG	NEG	NEG	NEG
IU6 (Turkey)	NEG	NEG	NEG	NEG	NEG	NEG	NEG	NEG	POS	NEG	NEG	NEG	POS	POS	NEG	NEG	NEG	NEG
IU7 (Turkey)	NEG	NEG	NEG	NEG	NEG	NEG	NEG	NEG	POS	NEG	NEG	NEG	NEG	NEG	NEG	NEG	NEG	NEG
IU9 (Turkey)	NEG	NEG	NEG	NEG	NEG	NEG	NEG	NEG	POS	NEG	NEG	NEG	NEG	NEG	NEG	NEG	NEG	NEG
MU1 (Turkey)	NEG	NEG	NEG	NEG	NEG	NEG	NEG	NEG	POS	NEG	NEG	NEG	POS	POS	NEG	NEG	NEG	NEG
MU10 (Turkey)	NEG	NEG	NEG	NEG	NEG	NEG	NEG	NEG	POS	NEG	NEG	NEG	POS	POS	NEG	NEG	NEG	NEG
MU20 (Turkey)	NEG	NEG	NEG	NEG	NEG	NEG	NEG	NEG	POS	NEG	NEG	NEG	NEG	NEG	NEG	NEG	NEG	NEG
Mu3 (Turkey)	NEG	NEG	NEG	NEG	NEG	NEG	NEG	NEG	POS	NEG	NEG	NEG	POS	POS	NEG	NEG	NEG	NEG
MU5 (Turkey)	NEG	NEG	NEG	NEG	NEG	NEG	NEG	NEG	POS	NEG	NEG	NEG	NEG	NEG	NEG	NEG	NEG	NEG
MU6 (Turkey)	NEG	NEG	NEG	NEG	NEG	NEG	NEG	NEG	POS	NEG	NEG	NEG	NEG	NEG	NEG	NEG	NEG	NEG
MU7 (Turkey)	NEG	NEG	NEG	NEG	NEG	NEG	NEG	NEG	POS	NEG	NEG	NEG	NEG	NEG	NEG	NEG	NEG	NEG
TUR27 (Turkey)	NEG	NEG	NEG	NEG	NEG	NEG	NEG	NEG	POS	NEG	NEG	NEG	POS	POS	NEG	NEG	NEG	NEG
NCTR 32S	NEG	NEG	NEG	NEG	NEG	NEG	NEG	NEG	POS	NEG	NEG	NEG	NEG	NEG	NEG	NEG	NEG	NEG
SA02_A4 = Russia-06_0085_Moscow	NEG	NEG	NEG	NEG	NEG	NEG	NEG	NEG	POS	NEG	NEG	NEG	POS	POS	NEG	NEG	NEG	NEG
	NEG	NEG	NEG	NEG	NEG	NEG	NEG	NEG	POS	NEG	NEG	NEG	POS	POS	NEG	NEG	NEG	NEG
Dresden_08V35987 (Turkey)	NEG	NEG	NEG	NEG	NEG	NEG	NEG	NEG	POS	NEG	NEG	NEG	POS	POS	NEG	NEG	NEG	NEG
Dresden_17ANR577152	NEG	NEG	NEG	NEG	NEG	NEG	NEG	NEG	POS	NEG	NEG	NEG	NEG	NEG	NEG	NEG	NEG	NEG
Dresden_17ANR580374 (Makedonia)	NEG	NEG	NEG	NEG	NEG	NEG	NEG	NEG	POS	NEG	NEG	NEG	NEG	NEG	NEG	NEG	NEG	NEG
Hong Kong_69-II	NEG	NEG	NEG	NEG	NEG	NEG	NEG	NEG	POS	NEG	NEG	NEG	NEG	NEG	NEG	NEG	NEG	NEG
Hong Kong_93	NEG	NEG	NEG	NEG	NEG	NEG	NEG	NEG	POS	NEG	AMB	NEG	NEG	NEG	NEG	NEG	NEG	NEG
Rawalpindi_Kidney Center_03_SK1	NEG	NEG	NEG	NEG	NEG	NEG	NEG	NEG	POS	NEG	NEG	NEG	NEG	NEG	NEG	NEG	NEG	NEG
Rawalpindi_Kidney Center_08	NEG	NEG	NEG	NEG	NEG	NEG	NEG	NEG	POS	NEG	NEG	NEG	NEG	NEG	NEG	NEG	NEG	NEG
Rawalpindi_Kidney Center_10	NEG	NEG	NEG	NEG	NEG	NEG	NEG	NEG	POS	NEG	NEG	NEG	NEG	NEG	NEG	NEG	NEG	NEG
Rawalpindi_Kidney Center_19	NEG	NEG	NEG	NEG	NEG	NEG	NEG	NEG	POS	NEG	NEG	NEG	NEG	NEG	NEG	NEG	NEG	NEG
Rawalpindi_Kidney Center_50_SK1	NEG	NEG	NEG	NEG	NEG	NEG	NEG	NEG	POS	NEG	NEG	NEG	NEG	NEG	NEG	NEG	NEG	NEG
Romania_Iasi_BC-49_430	NEG	NEG	NEG	NEG	NEG	NEG	NEG	NEG	POS	NEG	AMB	NEG	NEG	NEG	NEG	NEG	NEG	NEG
Romania_Iasi_MRSA_284	NEG	NEG	NEG	NEG	NEG	NEG	NEG	NEG	POS	NEG	NEG	NEG	NEG	NEG	NEG	NEG	NEG	NEG
Romania_Iasi_MRSA-32_318	NEG	NEG	NEG	NEG	NEG	NEG	NEG	NEG	POS	NEG	NEG	NEG	NEG	NEG	NEG	NEG	NEG	NEG
Romania_Iasi_MRSA-54_430	NEG	NEG	NEG	NEG	NEG	NEG	NEG	NEG	POS	NEG	AMB	NEG	NEG	NEG	NEG	NEG	NEG	NEG
Romania_Iasi_SSTI-01_2	NEG	NEG	NEG	NEG	NEG	NEG	NEG	NEG	POS	NEG	NEG	NEG	NEG	NEG	NEG	NEG	NEG	NEG
Romania_Iasi_SSTI-10_101	NEG	NEG	NEG	NEG	NEG	NEG	NEG	NEG	POS	NEG	NEG	NEG	POS	POS	NEG	NEG	NEG	NEG
Romania_Iasi_SSTI-11_106	NEG	NEG	NEG	NEG	NEG	NEG	NEG	NEG	POS	NEG	NEG	NEG	NEG	NEG	NEG	NEG	NEG	NEG
Russia-01_0001_SaintPetersburg	POS	NEG	NEG	POS	NEG	NEG	NEG	NEG	POS	NEG	NEG	NEG	POS	POS	NEG	NEG	NEG	NEG
Russia-02_0004_SaintPetersburg	POS	NEG	NEG	POS	NEG	NEG	NEG	NEG	POS	NEG	NEG	NEG	POS	POS	NEG	NEG	NEG	NEG
Russia-03_0057_SaintPetersburg	POS	NEG	NEG	POS	NEG	NEG	NEG	NEG	POS	NEG	NEG	NEG	POS	POS	NEG	NEG	NEG	NEG
Russia-04_0076_Moscow	NEG	NEG	NEG	NEG	NEG	NEG	NEG	NEG	POS	NEG	NEG	NEG	POS	POS	NEG	NEG	NEG	NEG
Russia-05_0078_Moscow	NEG	NEG	NEG	NEG	NEG	NEG	NEG	NEG	POS	NEG	NEG	NEG	POS	POS	NEG	NEG	NEG	NEG
Russia-08_0135_Moscow	NEG	NEG	NEG	NEG	NEG	NEG	NEG	NEG	POS	NEG	NEG	NEG	POS	POS	NEG	NEG	NEG	NEG
Russia-09_0150_Kurgan	NEG	NEG	NEG	NEG	NEG	NEG	NEG	NEG	POS	NEG	NEG	NEG	POS	POS	NEG	NEG	NEG	NEG
Russia-10_0162_Kurgan	POS	NEG	NEG	POS	NEG	NEG	NEG	NEG	POS	NEG	NEG	NEG	NEG	NEG	NEG	NEG	NEG	NEG
Russia-14_0184_Moscow	NEG	NEG	NEG	NEG	NEG	NEG	NEG	NEG	POS	NEG	NEG	NEG	NEG	NEG	NEG	NEG	NEG	NEG
Russia-15_0232_SaintPetersburg	NEG	NEG	NEG	NEG	NEG	NEG	NEG	NEG	POS	NEG	NEG	NEG	NEG	NEG	NEG	NEG	NEG	NEG
Russia-22_0391_Chelyabinsk	POS	NEG	NEG	POS	NEG	NEG	NEG	NEG	POS	NEG	NEG	NEG	NEG	NEG	NEG	NEG	NEG	NEG
Russia-23_0392_Chelyabinsk	POS	NEG	NEG	POS	NEG	NEG	NEG	NEG	POS	NEG	NEG	NEG	NEG	NEG	NEG	NEG	NEG	NEG
>"Eurasian Clade" : IU17	NEG											NEG	POS					
IU17 (Turkey)	NEG	NEG	NEG	NEG	NEG	NEG	NEG	NEG	POS	NEG	NEG	NEG	POS	POS	NEG	NEG	NEG	NEG
IU20 (Turkey)	NEG	NEG	NEG	NEG	NEG	NEG	NEG	NEG	POS	NEG	NEG	NEG	POS	POS	NEG	NEG	NEG	NEG

STRAIN / ISOLATE	RESISTANCE : MISCELLANEOUS GENES																	
	cat					cfr	fexA	apmA	fosB			qacA	qacC					
	cat	cat (pC221)	cat (pc223)	cat (pMC524)	cat (pSBK203R)	cfr	fexA	apmA	fosB	fosB (Sint)	fosB (plasmid 1)	qacA	qacC	qacC (cons)	qacC (equine)	qacC (SA5)	qacC (Ssap)	qacC (ST94)
	chloramphenicol acetyltransferase					23S rRNA methyltransferase	chloramphenicol/florfenicol exporter	aminocyclitol acetyltransferase, confers apramycin resistance	metallothiol transferase			quaternary ammonium compound resistance protein A	quaternary ammonium compound resistance protein C					
**>"Eurasian Clade": Spor[illegible]**	NEG											NEG	NEG					
Dresden_16ANR581769	NEG	NEG	NEG	NEG	NEG	NEG	NEG	NEG	POS	NEG	NEG	NEG	NEG	NEG	NEG	NEG	NEG	NEG
Dresden_17ANR578769	NEG	NEG	NEG	NEG	NEG	NEG	NEG	NEG	POS	NEG	NEG	NEG	NEG	NEG	NEG	NEG	NEG	NEG
**>"Eurasian Clade": Kuwa[illegible]**	NEG											NEG	NEG					
Kuwait_306	NEG	NEG	NEG	NEG	NEG	NEG	NEG	NEG	POS	NEG	POS	NEG	NEG	NEG	NEG	NEG	NEG	NEG
Kuwait_301	NEG	NEG	NEG	NEG	NEG	NEG	NEG	NEG	POS	NEG	AMB	NEG	NEG	NEG	NEG	NEG	NEG	NEG
**>"European Clade": "Gr[illegible]**	RARE											NEG	RARE					
GRE18 (Greece)	POS	AMB	NEG	NEG	NEG	NEG	NEG	NEG	POS	NEG	NEG	NEG	NEG	NEG	NEG	NEG	NEG	NEG
GRE317 (Greece)	NEG	NEG	NEG	NEG	NEG	NEG	NEG	NEG	POS	NEG	NEG	NEG	NEG	NEG	NEG	NEG	NEG	NEG
GRE4 (Greece)	NEG	NEG	NEG	NEG	NEG	NEG	NEG	NEG	POS	NEG	NEG	NEG	NEG	NEG	NEG	NEG	NEG	NEG
R35 (USA)	NEG	NEG	NEG	NEG	NEG	NEG	NEG	NEG	POS	NEG	NEG	NEG	POS	POS	NEG	NEG	NEG	NEG
Casablanca_HT20060548	POS	POS	NEG	NEG	NEG	NEG	NEG	NEG	POS	NEG	NEG	NEG	NEG	NEG	NEG	NEG	NEG	NEG
Casablanca_HT20060550	NEG	NEG	NEG	NEG	NEG	NEG	NEG	NEG	POS	NEG	NEG	NEG	NEG	NEG	NEG	NEG	NEG	NEG
Dresden_01V34572 (Greece)	NEG	NEG	NEG	NEG	NEG	NEG	NEG	NEG	POS	NEG	NEG	NEG	NEG	NEG	NEG	NEG	NEG	NEG
Dresden_01V36469	NEG	NEG	NEG	NEG	NEG	NEG	NEG	NEG	POS	NEG	NEG	NEG	NEG	NEG	NEG	NEG	NEG	NEG
Dresden_01V39123	NEG	NEG	NEG	NEG	NEG	NEG	NEG	NEG	POS	NEG	AMB	NEG	NEG	NEG	NEG	NEG	NEG	NEG
Dresden_01V39143	NEG	NEG	NEG	NEG	NEG	NEG	NEG	NEG	POS	NEG	NEG	NEG	NEG	NEG	NEG	NEG	NEG	NEG
Greece 1_3680_Harmony	NEG	NEG	NEG	NEG	NEG	NEG	NEG	NEG	POS	NEG	NEG	NEG	NEG	NEG	NEG	NEG	NEG	NEG
**>"European Clade": UK-[illegible]**	VAR											VAR	NEG					
ANS46 (Australia)	POS	POS	NEG	NEG	NEG	NEG	NEG	NEG	POS	NEG	NEG	NEG	NEG	NEG	NEG	NEG	NEG	NEG
LHH1 (USA)	NEG	NEG	NEG	NEG	NEG	NEG	NEG	NEG	POS	NEG	NEG	POS	NEG	NEG	NEG	NEG	NEG	NEG
Dublin-DSH_AR01_0-1085	NEG	NEG	NEG	NEG	NEG	NEG	NEG	NEG	POS	NEG	NEG	POS	NEG	NEG	NEG	NEG	NEG	NEG
Dublin-DSH_AR01_0-1118	NEG	NEG	NEG	NEG	NEG	NEG	NEG	NEG	POS	NEG	NEG	NEG	NEG	NEG	NEG	NEG	NEG	NEG
Dublin-DSH_AR15_0098	NEG	NEG	NEG	NEG	NEG	NEG	NEG	NEG	POS	NEG	NEG	POS	NEG	NEG	NEG	NEG	NEG	NEG
Dublin-DSH_AR15_0104	NEG	NEG	NEG	NEG	NEG	NEG	NEG	NEG	POS	NEG	NEG	POS	NEG	NEG	NEG	NEG	NEG	NEG
UK_NCTC11939_UK-1_Harmony	POS	POS	NEG	NEG	NEG	NEG	NEG	NEG	POS	NEG	NEG	POS	NEG	NEG	NEG	NEG	NEG	NEG
**>"European Clade": BK2**	NEG											POS	NEG					
BK2421 (USA)	NEG	NEG	NEG	NEG	NEG	NEG	NEG	NEG	POS	NEG	NEG	POS	NEG	NEG	NEG	NEG	NEG	NEG
**>"Aussi/NZ Clade", JKD[illegible]**	NEG											COMM	NEG					
JKD6009 (Australia)	NEG	NEG	NEG	NEG	NEG	NEG	NEG	NEG	POS	NEG	NEG	POS	NEG	NEG	NEG	NEG	NEG	NEG
JKD6008 (New Zealand)	NEG	NEG	NEG	NEG	NEG	NEG	NEG	NEG	POS	NEG	NEG	POS	NEG	NEG	NEG	NEG	NEG	NEG
Perth_2015-S20209	NEG	NEG	NEG	NEG	NEG	NEG	NEG	NEG	POS	NEG	NEG	POS	NEG	NEG	NEG	NEG	NEG	NEG
Perth_2015-S20305	NEG	NEG	NEG	NEG	NEG	NEG	NEG	NEG	POS	NEG	NEG	POS	NEG	NEG	NEG	NEG	NEG	NEG
Perth_2015-S20603	NEG	NEG	NEG	NEG	NEG	NEG	NEG	NEG	POS	NEG	NEG	POS	NEG	NEG	NEG	NEG	NEG	NEG
Perth_2015-S20607	NEG	NEG	NEG	NEG	NEG	NEG	NEG	NEG	POS	NEG	NEG	POS	NEG	NEG	NEG	NEG	NEG	NEG
Perth_2015-S20608	NEG	NEG	NEG	NEG	NEG	NEG	NEG	NEG	POS	NEG	NEG	POS	NEG	NEG	NEG	NEG	NEG	NEG
Perth_2015-S20610	NEG	NEG	NEG	NEG	NEG	NEG	NEG	NEG	POS	NEG	NEG	POS	NEG	NEG	NEG	NEG	NEG	NEG
Perth_2015-S20613	NEG	NEG	NEG	NEG	NEG	NEG	NEG	NEG	POS	NEG	NEG	POS	NEG	NEG	NEG	NEG	NEG	NEG
Perth_2015-S20617	NEG	NEG	NEG	NEG	NEG	NEG	NEG	NEG	POS	NEG	NEG	POS	NEG	NEG	NEG	NEG	NEG	NEG
Perth_2015-S20622	NEG	NEG	NEG	NEG	NEG	NEG	NEG	NEG	POS	NEG	NEG	POS	NEG	NEG	NEG	NEG	NEG	NEG
Perth_2015-S23619	NEG	NEG	NEG	NEG	NEG	NEG	NEG	NEG	POS	NEG	NEG	POS	NEG	NEG	NEG	NEG	NEG	NEG
Perth_2015-S41210	NEG	NEG	NEG	NEG	NEG	NEG	NEG	NEG	POS	NEG	NEG	POS	NEG	NEG	NEG	NEG	NEG	NEG
Perth_2015-S51001	NEG	NEG	NEG	NEG	NEG	NEG	NEG	NEG	POS	NEG	NEG	POS	NEG	NEG	NEG	NEG	NEG	NEG
Perth_2015-S51003	NEG	NEG	NEG	NEG	NEG	NEG	NEG	NEG	POS	NEG	NEG	POS	NEG	NEG	NEG	NEG	NEG	NEG
Perth_2015-S51006	NEG	NEG	NEG	NEG	NEG	NEG	NEG	NEG	POS	NEG	NEG	POS	NEG	NEG	NEG	NEG	NEG	NEG
Perth_2015-S51007	NEG	NEG	NEG	NEG	NEG	NEG	NEG	NEG	POS	NEG	NEG	POS	NEG	NEG	NEG	NEG	NEG	NEG
Perth_2015-S51008	NEG	NEG	NEG	NEG	NEG	NEG	NEG	NEG	POS	NEG	NEG	POS	NEG	NEG	NEG	NEG	NEG	NEG
Perth_2015-S51014	NEG	NEG	NEG	NEG	NEG	NEG	NEG	NEG	POS	NEG	NEG	POS	NEG	NEG	NEG	NEG	NEG	NEG
Perth_2015-S51408	NEG	NEG	NEG	NEG	NEG	NEG	NEG	NEG	POS	NEG	NEG	POS	NEG	NEG	NEG	NEG	NEG	NEG
Perth_2016-S20614	NEG	NEG	NEG	NEG	NEG	NEG	NEG	NEG	POS	NEG	NEG	POS	NEG	NEG	NEG	NEG	NEG	NEG
Perth_2016-S23614	NEG	NEG	NEG	NEG	NEG	NEG	NEG	NEG	POS	NEG	NEG	POS	NEG	NEG	NEG	NEG	NEG	NEG
Perth_2016-S42009	NEG	NEG	NEG	NEG	NEG	NEG	NEG	NEG	POS	NEG	NEG	POS	NEG	NEG	NEG	NEG	NEG	NEG
Perth_2016-S51001	NEG	NEG	NEG	NEG	NEG	NEG	NEG	NEG	POS	NEG	NEG	POS	NEG	NEG	NEG	NEG	NEG	NEG
Perth_2016-S51403	NEG	NEG	NEG	NEG	NEG	NEG	NEG	NEG	POS	NEG	NEG	POS	NEG	NEG	NEG	NEG	NEG	NEG
Perth_07 RPA 20	NEG	NEG	NEG	NEG	NEG	NEG	NEG	NEG	POS	NEG	NEG	POS	NEG	NEG	NEG	NEG	NEG	NEG
Perth_08-19088	NEG	NEG	NEG	NEG	NEG	NEG	NEG	NEG	POS	NEG	NEG	POS	NEG	NEG	NEG	NEG	NEG	NEG
Perth_01-15351	NEG	NEG	NEG	NEG	NEG	NEG	NEG	NEG	POS	NEG	NEG	AMB	NEG	NEG	NEG	NEG	NEG	NEG
Perth_01-15357	NEG	NEG	NEG	NEG	NEG	NEG	NEG	NEG	POS	NEG	NEG	POS	NEG	NEG	NEG	NEG	NEG	NEG
Perth_01-15419	NEG	NEG	NEG	NEG	NEG	NEG	NEG	NEG	POS	NEG	NEG	POS	NEG	NEG	NEG	NEG	NEG	NEG
Perth_01-16309	NEG	NEG	NEG	NEG	NEG	NEG	NEG	NEG	POS	NEG	NEG	POS	NEG	NEG	NEG	NEG	NEG	NEG
Perth_03 SNP 95	NEG	NEG	NEG	NEG	NEG	NEG	NEG	NEG	POS	NEG	AMB	POS	NEG	NEG	NEG	NEG	NEG	NEG
Perth_2003_AH_47	NEG	NEG	NEG	NEG	NEG	NEG	NEG	NEG	POS	NEG	AMB	POS	NEG	NEG	NEG	NEG	NEG	NEG
**>"Australian/NZ Clade":**	NEG											POS	VAR					
Perth_2015-S20623	NEG	NEG	NEG	NEG	NEG	NEG	NEG	NEG	POS	NEG	NEG	POS	POS	POS	NEG	NEG	NEG	NEG
Perth_2015-S20826	NEG	NEG	NEG	NEG	NEG	NEG	NEG	NEG	POS	NEG	NEG	POS	NEG	NEG	NEG	NEG	NEG	NEG
Perth_05 NH 56	NEG	NEG	NEG	NEG	NEG	NEG	NEG	NEG	POS	NEG	NEG	POS	NEG	NEG	NEG	NEG	NEG	NEG
**>CC5-MRSA-III LA MRSA**	NEG											NEG	NEG					
Delbrück 38 isolate from domestic turkey	NEG	NEG	NEG	NEG	NEG	NEG	NEG	NEG	POS	NEG	NEG	NEG	NEG	NEG	NEG	NEG	NEG	NEG
**> Staph. pseudintermed**	NEG											NEG	NEG					
KM1381	NEG	NEG	NEG	NEG	NEG	NEG	NEG	NEG	NEG	NEG	NEG	NEG	NEG	NEG	NEG	NEG	NEG	NEG
**>"South-East Asian Clad[illegible]**	RARE											VAR	VAR					
M92 (Canada)	NEG	NEG	NEG	NEG	NEG	NEG	NEG	NEG	POS	NEG	NEG	NEG	NEG	NEG	NEG	NEG	NEG	NEG
CHI59 (China)	POS	POS	NEG	NEG	NEG	NEG	NEG	NEG	POS	NEG	POS	NEG	NEG	NEG	NEG	NEG	NEG	NEG
CHI61 (China)	POS	POS	NEG	NEG	NEG	NEG	NEG	NEG	POS	NEG	NEG	POS	NEG	NEG	NEG	NEG	NEG	NEG
CUHK_HK1997 (China)	NEG	NEG	NEG	NEG	NEG	NEG	NEG	NEG	POS	NEG	NEG	NEG	NEG	NEG	NEG	NEG	NEG	NEG
M996 (China)	NEG	NEG	NEG	NEG	NEG	NEG	NEG	NEG	POS	NEG	NEG	POS	NEG	NEG	NEG	NEG	NEG	NEG
H211 (Denmark)	NEG	NEG	NEG	NEG	NEG	NEG	NEG	NEG	POS	NEG	NEG	NEG	POS	NEG	NEG	POS	NEG	NEG
H216 (Denmark)	NEG	NEG	NEG	NEG	NEG	NEG	NEG	NEG	POS	NEG	NEG	NEG	POS	NEG	NEG	POS	NEG	NEG
D71 (Germany)	NEG	NEG	NEG	NEG	NEG	NEG	NEG	NEG	POS	NEG	NEG	POS	NEG	NEG	NEG	NEG	NEG	NEG
NMR05 (India)	NEG	NEG	NEG	NEG	NEG	NEG	NEG	NEG	POS	NEG	NEG	NEG	NEG	NEG	NEG	NEG	NEG	NEG
MAL11 (Malaysia)	NEG	NEG	NEG	NEG	NEG	NEG	NEG	NEG	POS	NEG	NEG	POS	NEG	NEG	NEG	NEG	NEG	NEG
MAL9 (Malaysia)	NEG	NEG	NEG	NEG	NEG	NEG	NEG	NEG	POS	NEG	NEG	POS	POS	NEG	NEG	POS	NEG	NEG
Na21 (Sri Lanka)	NEG	NEG	NEG	NEG	NEG	NEG	NEG	NEG	POS	NEG	NEG	NEG	POS	NEG	NEG	POS	NEG	NEG
M592 (Syria)	NEG	NEG	NEG	NEG	NEG	NEG	NEG	NEG	POS	NEG	NEG	POS	NEG	NEG	NEG	NEG	NEG	NEG
H202 (Thailand)	NEG	NEG	NEG	NEG	NEG	NEG	NEG	NEG	POS	NEG	NEG	NEG	POS	NEG	NEG	POS	NEG	NEG
S102 (Thailand)	NEG	NEG	NEG	NEG	NEG	NEG	NEG	NEG	POS	NEG	NEG	NEG	POS	NEG	NEG	POS	NEG	NEG
S130 (Thailand)	NEG	NEG	NEG	NEG	NEG	NEG	NEG	NEG	POS	NEG	NEG	NEG	POS	NEG	NEG	POS	NEG	NEG
S40 (Thailand)	NEG	NEG	NEG	NEG	NEG	NEG	NEG	NEG	POS	NEG	NEG	NEG	NEG	NEG	NEG	NEG	NEG	NEG
S71 (Thailand)	NEG	NEG	NEG	NEG	NEG	NEG	NEG	NEG	POS	NEG	NEG	NEG	POS	NEG	NEG	POS	NEG	NEG
S87 (Thailand)	NEG	NEG	NEG	NEG	NEG	NEG	NEG	NEG	POS	NEG	NEG	NEG	POS	NEG	NEG	POS	NEG	NEG
S93 (Thailand)	NEG	NEG	NEG	NEG	NEG	NEG	NEG	NEG	POS	NEG	NEG	NEG	POS	NEG	NEG	POS	NEG	NEG
US_002 (Thailand)	NEG	NEG	NEG	NEG	NEG	NEG	NEG	NEG	POS	NEG	NEG	NEG	POS	NEG	NEG	POS	NEG	NEG
US_017 (Thailand)	NEG	NEG	NEG	NEG	NEG	NEG	NEG	NEG	POS	NEG	NEG	NEG	NEG	NEG	NEG	NEG	NEG	NEG
WAMC6102 (Thailand)	NEG	NEG	NEG	NEG	NEG	NEG	NEG	NEG	POS	NEG	NEG	POS	NEG	NEG	NEG	NEG	NEG	NEG
NCTC_13626_TW20 (UK)	NEG	NEG	NEG	NEG	NEG	NEG	NEG	NEG	POS	NEG	NEG	POS	NEG	NEG	NEG	NEG	NEG	NEG
BSAC3021 (UK)	NEG	NEG	NEG	NEG	NEG	NEG	NEG	NEG	POS	NEG	NEG	POS	NEG	NEG	NEG	NEG	NEG	NEG
BSAC3046 (UK)	POS	POS	NEG	NEG	NEG	NEG	NEG	NEG	POS	NEG	NEG	POS	NEG	NEG	NEG	NEG	NEG	NEG
1193_SAUR (USA)	NEG	NEG	NEG	NEG	NEG	NEG	NEG	NEG	POS	NEG	NEG	NEG	NEG	NEG	NEG	NEG	NEG	NEG
1194_SAUR (USA)	NEG	NEG	NEG	NEG	NEG	NEG	NEG	NEG	POS	NEG	NEG	NEG	NEG	NEG	NEG	NEG	NEG	NEG

STRAIN / ISOLATE	RESISTANCE : MISCELLANEOUS GENES																	
	cat					cfr	fexA	apmA	fosB			qacA	qacC					
	cat	cat (pC221)	cat (pc223)	cat (pMC524)	cat (pSBK203R)	cfr	fexA	apmA	fosB	fosB (Sint)	fosB (plasmid 1)	qacA	qacC	qacC (cons)	qacC (equine)	qacC (SA5)	qacC (Ssap)	qacC (ST94)
	chloramphenicol acetyltransferase					23S rRNA methyltransferase	chloramphenicol/florfenicol exporter	aminocyclitol acetyltransferase, confers apramycin resistance	metallothiol transferase			quaternary ammonium compound resistance protein A	quaternary ammonium compound resistance protein C					
>"South-East Asian Clade": TW20 (Irish AR44), c																		
1195_SAUR (USA)	NEG	NEG	NEG	NEG	NEG	NEG	NEG	NEG	POS	NEG	NEG	NEG	NEG	NEG	NEG	NEG	NEG	NEG
FVRH6002 (USA)	NEG	NEG	NEG	NEG	NEG	NEG	NEG	NEG	POS	NEG	NEG	NEG	NEG	NEG	NEG	NEG	NEG	NEG
KINW6048 (USA)	NEG	NEG	NEG	NEG	NEG	NEG	NEG	NEG	POS	NEG	NEG	POS	NEG	NEG	NEG	NEG	NEG	NEG
LAMC0011 (USA)	NEG	NEG	NEG	NEG	NEG	NEG	NEG	NEG	POS	NEG	NEG	POS	NEG	NEG	NEG	NEG	NEG	NEG
SJOS6053 (USA)	NEG	NEG	NEG	NEG	NEG	NEG	NEG	NEG	POS	NEG	NEG	POS	NEG	NEG	NEG	NEG	NEG	NEG
SJOS6072 (USA)	NEG	NEG	NEG	NEG	NEG	NEG	NEG	NEG	POS	NEG	NEG	NEG	NEG	NEG	NEG	NEG	NEG	NEG
UCIM6042 (USA)	NEG	NEG	NEG	NEG	NEG	NEG	NEG	NEG	POS	NEG	NEG	POS	NEG	NEG	NEG	NEG	NEG	NEG
Perth_2015-S31902	NEG	NEG	NEG	NEG	NEG	NEG	NEG	NEG	POS	NEG	NEG	POS	NEG	NEG	NEG	NEG	NEG	NEG
Perth_2015-S31904	NEG	NEG	NEG	NEG	NEG	NEG	NEG	NEG	POS	NEG	NEG	POS	NEG	NEG	NEG	NEG	NEG	NEG
Perth_2015-S31913	NEG	NEG	NEG	NEG	NEG	NEG	NEG	NEG	POS	NEG	NEG	POS	NEG	NEG	NEG	NEG	NEG	NEG
Perth_2015-S31918	NEG	NEG	NEG	NEG	NEG	NEG	NEG	NEG	POS	NEG	NEG	POS	NEG	NEG	NEG	NEG	NEG	NEG
Dublin-DSH_AR44_E1183	NEG	NEG	NEG	NEG	NEG	NEG	NEG	NEG	POS	NEG	NEG	POS	NEG	NEG	NEG	NEG	NEG	NEG
Dublin-DSH_Unfamiliar-3_E1520	NEG	NEG	NEG	NEG	NEG	NEG	NEG	NEG	POS	NEG	NEG	POS	NEG	NEG	NEG	NEG	NEG	NEG
Hong Kong_042	NEG	NEG	NEG	NEG	NEG	NEG	NEG	NEG	POS	NEG	NEG	NEG	NEG	NEG	NEG	NEG	NEG	NEG
Hong Kong_101	POS	NEG	NEG	POS	NEG	NEG	NEG	NEG	POS	NEG	NEG	NEG	NEG	NEG	NEG	NEG	NEG	NEG
Hong Kong_111	NEG	NEG	NEG	NEG	NEG	NEG	NEG	NEG	POS	NEG	AMB	NEG	NEG	NEG	NEG	NEG	NEG	NEG
Hong Kong_113	NEG	NEG	NEG	NEG	NEG	NEG	NEG	NEG	POS	NEG	NEG	NEG	NEG	NEG	NEG	NEG	NEG	NEG
Hong Kong_115	NEG	NEG	NEG	NEG	NEG	NEG	NEG	NEG	POS	NEG	NEG	NEG	NEG	NEG	NEG	NEG	NEG	NEG
Hong Kong_118	NEG	NEG	NEG	NEG	NEG	NEG	NEG	NEG	POS	NEG	NEG	NEG	NEG	NEG	NEG	NEG	NEG	NEG
Hong Kong_125	NEG	NEG	NEG	NEG	NEG	NEG	NEG	NEG	POS	NEG	NEG	NEG	NEG	NEG	NEG	NEG	NEG	NEG
Hong Kong_129	POS	NEG	NEG	POS	NEG	NEG	NEG	NEG	POS	NEG	NEG	NEG	NEG	NEG	NEG	NEG	NEG	NEG
Hong Kong_133	NEG	NEG	NEG	NEG	NEG	NEG	NEG	NEG	POS	NEG	NEG	POS	NEG	NEG	NEG	NEG	NEG	NEG
Kuwait_009	NEG	NEG	NEG	NEG	NEG	NEG	NEG	NEG	POS	NEG	NEG	POS	POS	NEG	NEG	POS	NEG	NEG
Kuwait_103	POS	NEG	NEG	POS	NEG	NEG	NEG	NEG	POS	NEG	AMB	POS	POS	NEG	NEG	POS	NEG	NEG
Kuwait_107	POS	NEG	NEG	POS	NEG	NEG	NEG	NEG	POS	NEG	AMB	POS	POS	NEG		POS	NEG	NEG
Perth_03 AH 88	NEG	NEG	NEG	NEG	NEG	NEG	NEG	NEG	POS	NEG	NEG	POS	NEG	NEG	NEG	NEG	NEG	NEG
Perth_08-16905	NEG	NEG	NEG	NEG	NEG	NEG	NEG	NEG	POS	NEG	NEG	POS	NEG	NEG	NEG	NEG	NEG	NEG
Riyadh_Alfaisal-10_SICU_13_B5672_1117353	NEG	NEG	NEG	NEG	NEG	NEG	NEG	NEG	POS	NEG	NEG	POS	NEG	NEG	NEG	NEG	NEG	NEG
Riyadh_SSTI_17_3508602	NEG	NEG	NEG	NEG	NEG	NEG	NEG	NEG	POS	NEG	NEG	POS	NEG	NEG	NEG	NEG	NEG	NEG
Riyadh-2822088-R	NEG	NEG	NEG	NEG	NEG	NEG	NEG	NEG	POS	NEG	NEG	POS	NEG	NEG	NEG	NEG	NEG	NEG
Trinidad&Tobago_2012_195 (619/12)	NEG	NEG	NEG	NEG	NEG	NEG	NEG	NEG	POS	NEG	AMB	NEG	NEG	NEG	NEG	NEG	NEG	NEG
Trinidad&Tobago_2012_370 (1112-52177)	NEG	NEG	NEG	NEG	NEG	NEG	NEG	NEG	POS	NEG	AMB	NEG	NEG	NEG	NEG	NEG	NEG	NEG
Trinidad&Tobago_2012_378 (4139)	NEG	NEG	NEG	NEG	NEG	NEG	NEG	NEG	POS	NEG	AMB	POS	POS	NEG	NEG	NEG	NEG	POS
Trinidad&Tobago_2013_PA21	NEG	NEG	NEG	NEG	NEG	NEG	NEG	NEG	POS	NEG	NEG	POS	NEG	NEG	NEG	NEG	NEG	NEG
Trinidad&Tobago_2013_PA22	NEG	NEG	NEG	NEG	NEG	NEG	NEG	NEG	POS	NEG	NEG	POS	NEG	NEG	NEG	NEG	NEG	NEG
Trinidad&Tobago_2912_340 (8523/12)	NEG	NEG	NEG	NEG	NEG	NEG	NEG	NEG	POS	NEG	NEG	POS	NEG	NEG	NEG	NEG	NEG	NEG
Trinidad&Tobago_MRSA2010_105	NEG	NEG	NEG	NEG	NEG	NEG	NEG	NEG	POS	NEG	NEG	POS	NEG	NEG	NEG	NEG	NEG	NEG
Trinidad&Tobago_MRSA2010_108	NEG	NEG	NEG	NEG	NEG	NEG	NEG	NEG	POS	NEG	AMB	POS	NEG	NEG	NEG	NEG	NEG	NEG
Trinidad&Tobago_MRSA2010_33	NEG	NEG	NEG	NEG	NEG	NEG	NEG	NEG	POS	NEG	NEG	POS	NEG	NEG	NEG	NEG	NEG	NEG
Trinidad&Tobago_MRSA2010_79	NEG	NEG	NEG	NEG	NEG	NEG	NEG	NEG	POS	NEG	NEG	POS	NEG	NEG	NEG	NEG	NEG	NEG
Trinidad&Tobago_MRSA2010_88	NEG	NEG	NEG	NEG	NEG	NEG	NEG	NEG	POS	NEG	NEG	POS	NEG	NEG	NEG	NEG	NEG	NEG
Trinidad&Tobago_MRSA2010_97	NEG	NEG	NEG	NEG	NEG	NEG	NEG	NEG	POS	NEG	AMB	POS	NEG	NEG	NEG	NEG	NEG	NEG
Trinidad&Tobago_SSTI 2012_031	NEG	NEG	NEG	NEG	NEG	NEG	NEG	NEG	POS	NEG	AMB	POS	NEG	NEG	NEG	NEG	NEG	NEG
Uganda-29_17148_250811	POS	POS	NEG	POS	NEG	NEG	NEG	NEG	POS	NEG	NEG	POS	NEG	NEG	NEG	NEG	NEG	NEG
>"South-East Asian Clad	NEG											VAR	NEG					
UCIM6015	NEG	NEG	NEG	NEG	NEG	NEG	NEG	NEG	POS	NEG	NEG	POS	NEG	NEG	NEG	NEG	NEG	NEG
Hong Kong_121	NEG	NEG	NEG	NEG	NEG	NEG	NEG	NEG	POS	NEG	NEG	NEG	NEG	NEG	NEG	NEG	NEG	NEG
>"South-East Asian Clad	NEG											POS	NEG					
Z172 (Taiwan)	NEG	NEG	NEG	NEG	NEG	NEG	NEG	NEG	POS	NEG	NEG	POS	NEG	NEG	NEG	NEG	NEG	NEG
Russia-19_0342_Kurgan	NEG	NEG	NEG	NEG	NEG	NEG	NEG	NEG	POS	NEG	NEG	POS	NEG	NEG	NEG	NEG	NEG	NEG
>"South-East Asian Clad	NEG											POS	NEG					
Perth_01-15392	NEG	NEG	NEG	NEG	NEG	NEG	NEG	NEG	POS	NEG	NEG	POS	NEG	NEG	NEG	NEG	NEG	NEG
>"South-East Asian Clad	RARE											RARE	RARE					
XN108 (China)	NEG	NEG	NEG	NEG	NEG	NEG	NEG	NEG	POS	NEG	NEG	NEG	NEG	NEG	NEG	NEG	NEG	NEG
NMR09 (India)	NEG	NEG	NEG	NEG	NEG	NEG	NEG	NEG	POS	NEG	NEG	NEG	NEG	NEG	NEG	NEG	NEG	NEG
346 (Malaysia)	POS	NEG	POS	NEG	AMB	NEG	NEG	NEG	POS	NEG	NEG	NEG	NEG	NEG	NEG	NEG	NEG	NEG
DS_009 (Thailand)	NEG	NEG	NEG	NEG	NEG	NEG	NEG	NEG	POS	NEG	NEG	NEG	POS	NEG	NEG	POS	NEG	NEG
18ANR581005 (Ind)	NEG	NEG	NEG	NEG	NEG	NEG	NEG	NEG	POS	NEG	NEG	NEG	NEG	NEG	NEG	NEG	NEG	NEG
Bengalaru_ST20141405	NEG	NEG	NEG	NEG	NEG	NEG	NEG	NEG	POS	NEG	NEG	NEG	NEG	NEG	NEG	NEG	NEG	NEG
Bengalaru_ST20141406	NEG	NEG	NEG	NEG	NEG	NEG	NEG	NEG	POS	NEG	NEG	NEG	NEG	NEG	NEG	NEG	NEG	NEG
Bengalaru_ST20141417	NEG	NEG	NEG	NEG	NEG	NEG	NEG	NEG	POS	NEG	NEG	NEG	NEG	NEG	NEG	NEG	NEG	NEG
Hong Kong_107	NEG	NEG	NEG	NEG	NEG	NEG	NEG	NEG	POS	NEG	NEG	NEG	NEG	NEG	NEG	NEG	NEG	NEG
Hong Kong_12	NEG	NEG	NEG	NEG	NEG	NEG	NEG	NEG	POS	NEG	NEG	NEG	NEG	NEG	NEG	NEG	NEG	NEG
Hong Kong_132	NEG	NEG	NEG	NEG	NEG	NEG	NEG	NEG	POS	NEG	NEG	NEG	NEG	NEG	NEG	NEG	NEG	NEG
Riyadh-3028763-R	NEG	NEG	NEG	NEG	NEG	NEG	NEG	NEG	POS	NEG	NEG	POS	NEG	NEG	NEG	NEG	NEG	NEG
Trinidad&Tobago_MRSA2010_94	NEG	NEG	NEG	NEG	NEG	NEG	NEG	NEG	POS	NEG	NEG	NEG	NEG	NEG	NEG	NEG	NEG	NEG
>"South-East Asian Clad	VAR											VAR	NEG					
M705 (Thailand)	POS	POS	NEG	NEG	NEG	NEG	NEG	NEG	POS	NEG	NEG	NEG	NEG	NEG	NEG	NEG	NEG	NEG
S85 (Thailand)	NEG	NEG	NEG	NEG	NEG	NEG	NEG	NEG	POS	NEG	NEG	NEG	NEG	NEG	NEG	NEG	NEG	NEG
Riyadh_Alfaisal-04_281014_S5076	NEG	NEG	NEG	NEG	NEG	NEG	NEG	NEG	POS	NEG	POS	POS	NEG	NEG	NEG	NEG	NEG	NEG
Riyadh_Alfaisal-26_S15145_1104163	NEG	NEG	NEG	NEG	NEG	NEG	NEG	NEG	POS	NEG	NEG	POS	NEG	NEG	NEG	NEG	NEG	NEG
Riyadh-2817437-W	NEG	NEG	NEG	NEG	NEG	NEG	NEG	NEG	POS	NEG	AMB	POS	NEG	NEG	NEG	NEG	NEG	NEG
>"South-East Asian Clad	VAR											VAR	NEG					
PPUKM-775-2009 (Malaysia)	NEG	NEG	NEG	NEG	NEG	NEG	NEG	NEG	POS	NEG	NEG	POS	NEG	NEG	NEG	NEG	NEG	NEG
Hong Kong_138	POS	NEG	NEG	POS	NEG	NEG	NEG	NEG	POS	NEG	NEG	NEG	NEG	NEG	NEG	NEG	NEG	NEG
>"South-East Asian Clad	NEG											POS	NEG					
Kuwait_001	NEG	NEG	NEG	NEG	NEG	NEG	NEG	NEG	POS	NEG	NEG	POS	NEG	NEG	NEG	NEG	NEG	NEG
Kuwait_337	NEG	NEG	NEG	NEG	NEG	NEG	NEG	NEG	POS	NEG	NEG	POS	NEG	NEG	NEG	NEG	NEG	NEG
>"South-East Asian Clad	VAR											VAR	RARE					
CUHK_HK2007 (China)	NEG	NEG	NEG	NEG	NEG	NEG	NEG	NEG	POS	NEG	NEG	POS	NEG	NEG	NEG	NEG	NEG	NEG
NMR02 (India)	NEG	NEG	NEG	NEG	NEG	NEG	NEG	NEG	POS	NEG	NEG	POS	NEG	NEG	NEG	NEG	NEG	NEG
VB1490 (India)	NEG	NEG	NEG	NEG	NEG	NEG	NEG	NEG	POS	NEG	NEG	NEG	NEG	NEG	NEG	NEG	NEG	NEG
V521 (Korea)	NEG	NEG	NEG	NEG	NEG	NEG	NEG	NEG	POS	NEG	NEG	POS	NEG	NEG	NEG	NEG	NEG	NEG
HST-077 (Lebanon)	NEG	NEG	NEG	NEG	NEG	NEG	NEG	NEG	POS	NEG	NEG	NEG	NEG	NEG	NEG	NEG	NEG	NEG
MAL1 (Malaysia)	POS	NEG	NEG	NEG	POS	NEG	NEG	NEG	POS	NEG	NEG	POS	NEG	NEG	NEG	NEG	NEG	NEG
MAL3 (Malaysia)	NEG	NEG	NEG	NEG	NEG	NEG	NEG	NEG	POS	NEG	NEG	NEG	NEG	NEG	NEG	NEG	NEG	NEG
PPUKM-261-2009 (Malaysia)	NEG	NEG	NEG	NEG	NEG	NEG	NEG	NEG	POS	NEG	NEG	POS	NEG	NEG	NEG	NEG	NEG	NEG
PPUKM-332-2009 (Malaysia)	POS	NEG	NEG	NEG	POS	NEG	NEG	NEG	POS	NEG	NEG	POS	NEG	NEG	NEG	NEG	NEG	NEG
S38 (Thailand)	NEG	NEG	NEG	NEG	NEG	NEG	NEG	NEG	POS	NEG	NEG	POS	NEG	NEG	NEG	NEG	NEG	NEG
S7 (Thailand)	NEG	NEG	NEG	NEG	NEG	NEG	NEG	NEG	POS	NEG	NEG	POS	NEG	NEG	NEG	NEG	NEG	NEG
US_008 (Thailand)	POS	POS	NEG	NEG	NEG	NEG	NEG	NEG	POS	NEG	NEG	NEG	NEG	NEG	NEG	NEG	NEG	NEG
US_030 (Thailand)	NEG	NEG	NEG	NEG	NEG	NEG	NEG	NEG	POS	NEG	NEG	NEG	NEG	NEG	NEG	NEG	NEG	NEG
M170 (Tsunami-related)	NEG	NEG	NEG	NEG	NEG	NEG	NEG	NEG	POS	NEG	NEG	POS	NEG	NEG	NEG	NEG	NEG	NEG
BSAC697 (UK)	NEG	NEG	NEG	NEG	NEG	NEG	NEG	NEG	POS	NEG	NEG	NEG	NEG	NEG	NEG	NEG	NEG	NEG
IS-189	POS	POS	NEG	NEG	NEG	NEG	NEG	NEG	POS	NEG	NEG	POS	NEG	NEG	NEG	NEG	NEG	NEG
MRGR3	POS	NEG	NEG	POS	NEG	NEG	NEG	NEG	POS	NEG	NEG	NEG	NEG	NEG	NEG	NEG	NEG	NEG
smg07	POS	NEG	NEG	POS	NEG	NEG	NEG	NEG	POS	NEG	NEG	NEG	POS	NEG	NEG	POS	NEG	NEG
AH1-AUS-EMRSA-3	NEG	NEG	NEG	NEG	NEG	NEG	NEG	NEG	POS	NEG	NEG	POS	NEG	NEG	NEG	NEG	NEG	NEG
Bengalaru_ST20141403	NEG	NEG	NEG	NEG	NEG	NEG	NEG	NEG	POS	NEG	NEG	POS	NEG	NEG	NEG	NEG	NEG	NEG
Finland E24_98541_Harmony	POS	NEG	NEG	POS	NEG	NEG	NEG	NEG	POS	NEG	NEG	POS	NEG	NEG	NEG	NEG	NEG	NEG
Hong Kong_10	NEG	NEG	NEG	NEG	NEG	NEG	NEG	NEG	POS	NEG	AMB	POS	NEG	NEG	NEG	NEG	NEG	NEG
Hong Kong_100	NEG	NEG	NEG	NEG	NEG	NEG	NEG	NEG	POS	NEG	NEG	NEG	NEG	NEG	NEG	NEG	NEG	NEG
Hong Kong_199	NEG	NEG	NEG	NEG	NEG	NEG	NEG	NEG	POS	NEG	AMB	NEG	NEG	NEG	NEG	NEG	NEG	NEG
Hong Kong_34	NEG	NEG	NEG	NEG	NEG	NEG	NEG	NEG	POS	NEG	AMB	NEG	NEG	NEG	NEG	NEG	NEG	NEG
Hong Kong_88	NEG	NEG	NEG	NEG	NEG	NEG	NEG	NEG	POS	NEG	POS	POS	NEG	NEG	NEG	NEG	NEG	NEG
Hong Kong_92	NEG	NEG	NEG	NEG	NEG	NEG	NEG	NEG	POS	NEG	AMB	NEG	NEG	NEG	NEG	NEG	NEG	NEG
Hong Kong_94	NEG	NEG	NEG	NEG	NEG	NEG	NEG	NEG	POS	NEG	NEG	NEG	POS	POS	NEG	NEG	NEG	NEG
Kuwait_257	POS	POS	NEG	NEG	NEG	NEG	NEG	NEG	POS	NEG	NEG	NEG	NEG	NEG	NEG	NEG	NEG	NEG
Perth_01-15330	NEG	NEG	NEG	NEG	NEG	NEG	NEG	NEG	POS	NEG	NEG	POS	NEG	NEG	NEG	NEG	NEG	NEG
Perth_01-15349	NEG	NEG	NEG	NEG	NEG	NEG	NEG	NEG	POS	NEG	NEG	POS	NEG	NEG	NEG	NEG	NEG	NEG
Perth_01-16176	NEG	NEG	NEG	NEG	NEG	NEG	NEG	NEG	POS	NEG	NEG	POS	NEG	NEG	NEG	NEG	NEG	NEG
Perth_WBG8379	NEG	NEG	NEG	NEG	NEG	NEG	NEG	NEG	POS	NEG	NEG	POS	NEG	NEG	NEG	NEG	NEG	NEG
Rawalpindi_Kidney Center_56	NEG	NEG	NEG	NEG	NEG	NEG	NEG	NEG	POS	NEG	NEG	NEG	NEG	NEG	NEG	NEG	NEG	NEG
Riyadh_SSTI_05_3464614	NEG	NEG	NEG	NEG	NEG	NEG	NEG	NEG	POS	NEG	NEG	POS	NEG	NEG	NEG	NEG	NEG	NEG
Trinidad&Tobago_2012_157	NEG	NEG	NEG	NEG	NEG	NEG	NEG	NEG	POS	NEG	AMB	NEG	NEG	NEG	NEG	NEG	NEG	NEG

STRAIN / ISOLATE	RESISTANCE : MISCELLANEOUS GENES																	
	cat					cfr	fexA	apmA	fosB			qacA	qacC					
	cat	cat (pC221)	cat (pc223)	cat (pMC524)	cat (pSBK203R)	cfr	fexA	apmA	fosB	fosB (Sint)	fosB (plasmid 1)	qacA	qacC	qacC (cons)	qacC (equine)	qacC (SA5)	qacC (Ssap)	qacC (ST94)
	chloramphenicol acetyltransferase					23S rRNA methyltransferase	chloramphenicol/florfenicol exporter	aminocyclitol acetyltransferase, confers apramycin resistance	metallothiol transferase			quaternary ammonium compound resistance protein A	quaternary ammonium compound resistance protein C					
>"South-East Asian Clad	NEG											NEG	NEG					
M418	NEG	NEG	NEG	NEG	NEG	NEG	NEG	NEG	POS	NEG	NEG	NEG	NEG	NEG	NEG	NEG	NEG	NEG
>"South-East Asian Clad	NEG											NEG	NEG					
NMR07 (India)	NEG	NEG	NEG	NEG	NEG	NEG	NEG	NEG	POS	NEG	NEG	NEG	NEG	NEG	NEG	NEG	NEG	NEG
NMR08 (India)	NEG	NEG	NEG	NEG	NEG	NEG	NEG	NEG	POS	NEG	NEG	NEG	NEG	NEG	NEG	NEG	NEG	NEG
Bengalaru_ST20121978	NEG	NEG	NEG	NEG	NEG	NEG	NEG	NEG	POS	NEG	NEG	NEG	NEG	NEG	NEG	NEG	NEG	NEG
Hong Kong_89	NEG	NEG	NEG	NEG	NEG	NEG	NEG	NEG	POS	NEG	POS	NEG	NEG	NEG	NEG	NEG	NEG	NEG
>"South-East Asian Clad	NEG											POS	NEG					
Perth_2005_AH_5	NEG	NEG	NEG	NEG	NEG	NEG	NEG	NEG	POS	NEG	NEG	POS	NEG	NEG	NEG	NEG	NEG	NEG
>"South-East Asian Clad	NEG											VAR	NEG					
Trinidad&Tobago_MRSA2010_103	NEG	NEG	NEG	NEG	NEG	NEG	NEG	NEG	POS	NEG	NEG	NEG	NEG	NEG	NEG	NEG	NEG	NEG
Trinidad&Tobago_2013_PA25	NEG	NEG	NEG	NEG	NEG	NEG	NEG	NEG	POS	NEG	NEG	POS	NEG	NEG	NEG	NEG	NEG	NEG
Trinidad&Tobago_MRSA2010_110	NEG	NEG	NEG	NEG	NEG	NEG	NEG	NEG	POS	NEG	NEG	POS	NEG	NEG	NEG	NEG	NEG	NEG
Trinidad&Tobago_SSTI 2012_111	NEG	NEG	NEG	NEG	NEG	NEG	NEG	NEG	POS	NEG	NEG	POS	NEG	NEG	NEG	NEG	NEG	NEG
Trinidad&Tobago_MRSA2010_158	NEG	NEG	NEG	NEG	NEG	NEG	NEG	NEG	POS	NEG	NEG	NEG	NEG	NEG	NEG	NEG	NEG	NEG
>"South-East Asian Clad	NEG											VAR	NEG					
Kuwait_122	NEG	NEG	NEG	NEG	NEG	NEG	NEG	NEG	POS	NEG	NEG	POS	NEG	NEG	NEG	NEG	NEG	NEG
Kuwait_002	NEG	NEG	NEG	NEG	NEG	NEG	NEG	NEG	POS	NEG	NEG	POS	NEG	NEG	NEG	NEG	NEG	NEG
Kuwait_196	NEG	NEG	NEG	NEG	NEG	NEG	NEG	NEG	POS	NEG	NEG	POS	NEG	NEG	NEG	NEG	NEG	NEG
Kuwait_199	NEG	NEG	NEG	NEG	NEG	NEG	NEG	NEG	POS	NEG	NEG	POS	NEG	NEG	NEG	NEG	NEG	NEG
Hong Kong_136	NEG	NEG	NEG	NEG	NEG	NEG	NEG	NEG	POS	NEG	NEG	NEG	NEG	NEG	NEG	NEG	NEG	NEG
>"South-East Asian Clad	NEG											NEG	NEG					
P32 (Poland)	NEG	NEG	NEG	NEG	NEG	NEG	NEG	NEG	POS	NEG	NEG	NEG	NEG	NEG	NEG	NEG	NEG	NEG
>"South-East Asian Clad	NEG											NEG	NEG					
Perth_2015-S32313	NEG	NEG	NEG	NEG	NEG	NEG	NEG	NEG	POS	NEG	NEG	NEG	NEG	NEG	NEG	NEG	NEG	NEG
>"hla-negative South-Ea	NEG											POS	NEG					
DEN907 (Denmark)	NEG	NEG	NEG	NEG	NEG	NEG	NEG	NEG	POS	NEG	NEG	POS	NEG	NEG	NEG	NEG	NEG	NEG
>"hla-negative South-Ea	NEG											COMM	RARE					
S26 (Thailand)	NEG	NEG	NEG	NEG	NEG	NEG	NEG	NEG	POS	NEG	NEG	POS	NEG	NEG	NEG	NEG	NEG	NEG
S42 (Thailand)	NEG	NEG	NEG	NEG	NEG	NEG	NEG	NEG	POS	NEG	NEG	POS	NEG	NEG	NEG	NEG	NEG	NEG
S97 (Thailand)	NEG	NEG	NEG	NEG	NEG	NEG	NEG	NEG	POS	NEG	NEG	POS	NEG	NEG	NEG	NEG	NEG	NEG
S78 (Thailand)	NEG	NEG	NEG	NEG	NEG	NEG	NEG	NEG	POS	NEG	NEG	POS	NEG	NEG	NEG	NEG	NEG	NEG
S25 (Thailand)	NEG	NEG	NEG	NEG	NEG	NEG	NEG	NEG	POS	NEG	NEG	POS	NEG	NEG	NEG	NEG	NEG	NEG
S21 (Thailand)	NEG	NEG	NEG	NEG	NEG	NEG	NEG	NEG	POS	NEG	NEG	POS	NEG	NEG	NEG	NEG	NEG	NEG
S106 (Thailand)	NEG	NEG	NEG	NEG	NEG	NEG	NEG	NEG	POS	NEG	NEG	POS	NEG	NEG	NEG	NEG	NEG	NEG
S24 (Thailand)	NEG	NEG	NEG	NEG	NEG	NEG	NEG	NEG	POS	NEG	NEG	POS	NEG	NEG	NEG	NEG	NEG	NEG
S39 (Thailand)	NEG	NEG	NEG	NEG	NEG	NEG	NEG	NEG	POS	NEG	NEG	POS	NEG	NEG	NEG	NEG	NEG	NEG
S2 (Thailand)	NEG	NEG	NEG	NEG	NEG	NEG	NEG	NEG	POS	NEG	NEG	POS	POS	NEG	NEG	POS	NEG	NEG
S81 (Thailand)	NEG	NEG	NEG	NEG	NEG	NEG	NEG	NEG	POS	NEG	NEG	POS	NEG	NEG	NEG	NEG	NEG	NEG
M116 (Vietnam)	NEG	NEG	NEG	NEG	NEG	NEG	NEG	NEG	POS	NEG	NEG	NEG	NEG	NEG	NEG	NEG	NEG	NEG
>"Portuguese Clade": Pc	VAR											NEG	RARE					
FFP103 (Portugal)	NEG	NEG	NEG	NEG	NEG	NEG	NEG	NEG	POS	NEG	NEG	NEG	NEG	NEG	NEG	NEG	NEG	NEG
HDG2 (Portugal)	NEG	NEG	NEG	NEG	NEG	NEG	NEG	NEG	POS	NEG	NEG	NEG	NEG	NEG	NEG	NEG	NEG	NEG
HSA10 (Portugal)	NEG	NEG	NEG	NEG	NEG	NEG	NEG	NEG	POS	NEG	NEG	NEG	NEG	NEG	NEG	NEG	NEG	NEG
HSA11 (Portugal)	NEG	NEG	NEG	NEG	NEG	NEG	NEG	NEG	POS	NEG	NEG	NEG	NEG	NEG	NEG	NEG	NEG	NEG
ICP5011 (Portugal)	NEG	NEG	NEG	NEG	NEG	NEG	NEG	NEG	POS	NEG	NEG	NEG	NEG	NEG	NEG	NEG	NEG	NEG
ICP5014 (Portugal)	NEG	NEG	NEG	NEG	NEG	NEG	NEG	NEG	POS	NEG	NEG	NEG	NEG	NEG	NEG	NEG	NEG	NEG
ICP5062 (Portugal)	NEG	NEG	NEG	NEG	NEG	NEG	NEG	NEG	POS	NEG	NEG	NEG	NEG	NEG	NEG	NEG	NEG	NEG
Strain 1063_ATCC 33592 FDAARGOS_3 (USA)	NEG	NEG	NEG	NEG	NEG	NEG	NEG	NEG	POS	NEG	NEG	NEG	NEG	NEG	NEG	NEG	NEG	NEG
	POS	NEG	NEG	POS	NEG	NEG	NEG	NEG	POS	NEG	AMB	NEG	POS	POS	NEG	NEG	NEG	NEG
Russia-07_0110_Kurgan	POS	NEG	NEG	POS	NEG	NEG	NEG	NEG	POS	NEG	NEG	NEG	NEG	NEG	NEG	NEG	NEG	NEG
Russia-11_0164_Kurgan	POS	NEG	NEG	POS	NEG	NEG	NEG	NEG	POS	NEG	NEG	NEG	NEG	NEG	NEG	NEG	NEG	NEG
Russia-20_0367_Kurgan	POS	NEG	NEG	POS	NEG	NEG	NEG	NEG	POS	NEG	NEG	NEG	NEG	NEG	NEG	NEG	NEG	NEG
Russia-21_0390_Chelyabinsk	POS	NEG	NEG	POS	NEG	NEG	NEG	NEG	POS	NEG	NEG	NEG	NEG	NEG	NEG	NEG	NEG	NEG
>"South American/ Mid	VAR											RARE	RARE					
AGT1 (Argentina)	NEG	NEG	NEG	NEG	NEG	NEG	NEG	NEG	POS	NEG	NEG	NEG	NEG	NEG	NEG	NEG	NEG	NEG
AGT120 (Argentina)	POS	NEG	NEG	POS	NEG	NEG	NEG	NEG	POS	NEG	NEG	NEG	NEG	NEG	NEG	NEG	NEG	NEG
AGT67 (Argentina)	NEG	NEG	NEG	NEG	NEG	NEG	NEG	NEG	POS	NEG	NEG	NEG	NEG	NEG	NEG	NEG	NEG	NEG
AGT9 (Argentina)	POS	NEG	NEG	POS	NEG	NEG	NEG	NEG	POS	NEG	NEG	NEG	NEG	NEG	NEG	NEG	NEG	NEG
RA3 (Argentina)	POS	NEG	NEG	POS	NEG	NEG	NEG	NEG	POS	NEG	NEG	NEG	NEG	NEG	NEG	NEG	NEG	NEG
RA6 (Argentina)	NEG	NEG	NEG	NEG	NEG	NEG	NEG	NEG	POS	NEG	NEG	NEG	NEG	NEG	NEG	NEG	NEG	NEG
RA7 (Argentina)	NEG	NEG	NEG	NEG	NEG	NEG	NEG	NEG	POS	NEG	NEG	NEG	POS	POS	NEG	NEG	NEG	NEG
Be62 (Brazil)	POS	NEG	NEG	POS	NEG	NEG	NEG	NEG	POS	NEG	NEG	NEG	NEG	NEG	NEG	NEG	NEG	NEG
Bmb9393 (Brazil)	POS	NEG	NEG	POS	NEG	NEG	NEG	NEG	POS	NEG	NEG	NEG	NEG	NEG	NEG	NEG	NEG	NEG
BRA36 (Brazil)	POS	NEG	NEG	POS	NEG	NEG	NEG	NEG	POS	NEG	NEG	NEG	NEG	NEG	NEG	NEG	NEG	NEG
BZ48 (Brazil)	POS	NEG	NEG	POS	NEG	NEG	NEG	NEG	POS	NEG	NEG	NEG	NEG	NEG	NEG	NEG	NEG	NEG
Gv51 (Brazil)	POS	NEG	NEG	POS	NEG	NEG	NEG	NEG	POS	NEG	NEG	NEG	NEG	NEG	NEG	NEG	NEG	NEG
Gv69 (Brazil)	NEG	NEG	NEG	NEG	NEG	NEG	NEG	NEG	POS	NEG	NEG	NEG	NEG	NEG	NEG	NEG	NEG	NEG
Gv88 (Brazil)	POS	NEG	NEG	POS	NEG	NEG	NEG	NEG	POS	NEG	NEG	NEG	NEG	NEG	NEG	NEG	NEG	NEG
HC1335 (Brazil)	NEG	NEG	NEG	NEG	NEG	NEG	NEG	NEG	POS	NEG	NEG	NEG	NEG	NEG	NEG	NEG	NEG	NEG
HC1340 (Brazil)	NEG	NEG	NEG	NEG	NEG	NEG	NEG	NEG	POS	NEG	NEG	NEG	NEG	NEG	NEG	NEG	NEG	NEG
HC556 (Brazil)	NEG	NEG	NEG	NEG	NEG	NEG	NEG	NEG	POS	NEG	NEG	NEG	NEG	NEG	NEG	NEG	NEG	NEG
HU25 (Brazil)	NEG	NEG	NEG	NEG	NEG	NEG	NEG	NEG	POS	NEG	NEG	NEG	NEG	NEG	NEG	NEG	NEG	NEG
UB563 (Brazil)	NEG	NEG	NEG	NEG	NEG	NEG	NEG	NEG	POS	NEG	NEG	NEG	NEG	NEG	NEG	NEG	NEG	NEG
2A8 (Czech Republic)	NEG	NEG	NEG	NEG	NEG	NEG	NEG	NEG	POS	NEG	NEG	NEG	POS	POS	NEG	NEG	NEG	NEG
M1229 (Denmark)	NEG	NEG	NEG	NEG	NEG	NEG	NEG	NEG	POS	NEG	NEG	NEG	NEG	NEG	NEG	NEG	NEG	NEG
FRICAR (France)	NEG	NEG	NEG	NEG	NEG	NEG	NEG	NEG	POS	NEG	NEG	NEG	NEG	NEG	NEG	NEG	NEG	NEG
D90 (Germany)	POS	NEG	NEG	POS	NEG	NEG	NEG	NEG	POS	NEG	NEG	NEG	NEG	NEG	NEG	NEG	NEG	NEG
LIT68 (Lithuania)	POS	NEG	NEG	POS	NEG	NEG	NEG	NEG	POS	NEG	NEG	NEG	NEG	NEG	NEG	NEG	NEG	NEG
LIT76 (Lithuania)	POS	NEG	NEG	POS	NEG	NEG	NEG	NEG	POS	NEG	NEG	NEG	NEG	NEG	NEG	NEG	NEG	NEG
HGSA142 (Portugal)	POS	NEG	NEG	POS	NEG	NEG	NEG	NEG	POS	NEG	NEG	NEG	NEG	NEG	NEG	NEG	NEG	NEG
HGSA9 (Portugal)	NEG	NEG	NEG	NEG	NEG	NEG	NEG	NEG	POS	NEG	NEG	NEG	NEG	NEG	NEG	NEG	NEG	NEG
HSJ216 (Portugal)	POS	NEG	NEG	POS	NEG	NEG	NEG	NEG	POS	NEG	NEG	NEG	NEG	NEG	NEG	NEG	NEG	NEG
M278 (Portugal)	NEG	NEG	NEG	NEG	NEG	NEG	NEG	NEG	POS	NEG	NEG	NEG	POS	NEG	NEG	POS	NEG	NEG
ES26 (Spain)	NEG	NEG	NEG	NEG	NEG	NEG	NEG	NEG	POS	NEG	NEG	NEG	NEG	NEG	NEG	NEG	NEG	NEG
UK102 (UK)	NEG	NEG	NEG	NEG	NEG	NEG	NEG	NEG	POS	NEG	NEG	POS	POS	NEG	NEG	POS	NEG	NEG
URU34 (Uruguay)	POS	AMB	NEG	POS	NEG	NEG	NEG	NEG	POS	NEG	NEG	NEG	NEG	NEG	NEG	NEG	NEG	NEG
IS-125	NEG	NEG	NEG	NEG	NEG	NEG	NEG	NEG	POS	NEG	NEG	NEG	NEG	NEG	NEG	NEG	NEG	NEG
IS-157	NEG	NEG	NEG	NEG	NEG	NEG	NEG	NEG	POS	NEG	NEG	POS	NEG	NEG	NEG	NEG	NEG	NEG
Ecuador_1	NEG	NEG	NEG	NEG	NEG	NEG	NEG	NEG	POS	NEG	NEG	NEG	NEG	NEG	NEG	NEG	NEG	NEG
Ecuador_2	POS	POS	NEG	NEG	NEG	NEG	NEG	NEG	POS	NEG	NEG	NEG	NEG	NEG	NEG	NEG	NEG	NEG
Dresden_15ANR574263	NEG	NEG	NEG	NEG	NEG	NEG	NEG	NEG	POS	NEG	NEG	NEG	NEG	NEG	NEG	NEG	NEG	NEG
UK-EMRSA-11	NEG	NEG	NEG	NEG	NEG	NEG	NEG	NEG	POS	NEG	NEG	POS	NEG	NEG	NEG	NEG	NEG	NEG
>"South American/ Mid	VAR											NEG	NEG					
BRA2 (Brazil)	POS	NEG	NEG	POS	NEG	NEG	NEG	NEG	POS	NEG	NEG	NEG	NEG	NEG	NEG	NEG	NEG	NEG
CHL1 (Chile)	POS	NEG	NEG	POS	NEG	NEG	NEG	NEG	POS	NEG	NEG	NEG	NEG	NEG	NEG	NEG	NEG	NEG
CHL151 (Chile)	POS	NEG	NEG	POS	NEG	NEG	NEG	NEG	POS	NEG	NEG	NEG	NEG	NEG	NEG	NEG	NEG	NEG
UCL378 (Chile)	NEG	NEG	NEG	NEG	NEG	NEG	NEG	NEG	POS	NEG	NEG	NEG	NEG	NEG	NEG	NEG	NEG	NEG
UP72 (Peru)	NEG	NEG	NEG	NEG	NEG	NEG	NEG	NEG	POS	NEG	NEG	NEG	NEG	NEG	NEG	NEG	NEG	NEG
UP81 (Peru)	NEG	NEG	NEG	NEG	NEG	NEG	NEG	NEG	POS	NEG	NEG	NEG	NEG	NEG	NEG	NEG	NEG	NEG
>"South American/ Mid	NEG											NEG	NEG					
UP1073 (Peru)	NEG	NEG	NEG	NEG	NEG	NEG	NEG	NEG	POS	NEG	NEG	NEG	NEG	NEG	NEG	NEG	NEG	NEG

STRAIN / ISOLATE	RESISTANCE : MISCELLANEOUS GENES																	
	cat					cfr	fexA	apmA	fosB			qacA	qacC					
	cat	cat (pC221)	cat (pc223)	cat (pMC524)	cat (pSBK203R)	cfr	fexA	apmA	fosB	fosB (Sint)	fosB (plasmid 1)	qacA	qacC	qacC (cons)	qacC (equine)	qacC (SA5)	qacC (Ssap)	qacC (ST94)
	chloramphenicol acetyltransferase					23S rRNA methyltransferase	chloramphenicol/florfenicol exporter	aminocyclitol acetyltransferase, confers apramycin resistance	metallothiol transferase			quaternary ammonium compound resistance protein A	quaternary ammonium compound resistance protein C					
>"South American/ Midd	NEG											VAR	NEG					
LIT89 (Lithuania)	NEG	NEG	NEG	NEG	NEG	NEG	NEG	NEG	POS	NEG	NEG	NEG	NEG	NEG	NEG	NEG	NEG	NEG
MRSA_PR1 (Malaysia)	NEG	NEG	NEG	NEG	NEG	NEG	NEG	NEG	POS	NEG	NEG	NEG	NEG	NEG	NEG	NEG	NEG	NEG
Riyadh-2888905-R	NEG	NEG	NEG	NEG	NEG	NEG	NEG	NEG	POS	NEG	NEG	POS	NEG	NEG	NEG	NEG	NEG	NEG
Riyadh-2888915-BC	NEG	NEG	NEG	NEG	NEG	NEG	NEG	NEG	POS	NEG	NEG	POS	NEG	NEG	NEG	NEG	NEG	NEG
Riyadh-2793706-R	NEG	NEG	NEG	NEG	NEG	NEG	NEG	NEG	POS	NEG	AMB	POS	NEG	NEG	NEG	NEG	NEG	NEG
>"South American/ Midd	NEG											POS	NEG					
Riyadh_SSTI_18_3502925	NEG	NEG	NEG	NEG	NEG	NEG	NEG	NEG	POS	NEG	POS	POS	NEG	NEG	NEG	NEG	NEG	NEG
Riyadh-2822825-W	NEG	NEG	NEG	NEG	NEG	NEG	NEG	NEG	POS	NEG	AMB	POS	NEG	NEG	NEG	NEG	NEG	NEG
>"South American/ Midd	POS											NEG	POS					
UK-EMRSA-9	POS	NEG	NEG	POS	NEG	NEG	NEG	NEG	POS	NEG	NEG	NEG	POS	NEG	AMB	POS	NEG	NEG
>"South American/ Midd	NEG											NEG	NEG					
Lome_HT20020815	NEG	NEG	NEG	NEG	NEG	NEG	NEG	NEG	POS	NEG	NEG	NEG	NEG	NEG	NEG	NEG	NEG	NEG
>"South American/ Midd	RARE											VAR	RARE					
NA32 (Denmark)	NEG	NEG	NEG	NEG	NEG	NEG	NEG	NEG	POS	NEG	NEG	NEG	POS	NEG	NEG	POS	NEG	NEG
H24 (Egypt)	POS	NEG	NEG	POS	NEG	NEG	NEG	NEG	POS	NEG	NEG	POS	NEG	NEG	NEG	NEG	NEG	NEG
ATCC BAA-39 (=HUSA304) (Hungary)	NEG	NEG	NEG	NEG	NEG	NEG	NEG	NEG	POS	NEG	NEG	NEG	NEG	NEG	NEG	NEG	NEG	NEG
HUSA304 (Hungary)	NEG	NEG	NEG	NEG	NEG	NEG	NEG	NEG	POS	NEG	NEG	NEG	NEG	NEG	NEG	NEG	NEG	NEG
HU106 (Hungary)	NEG	NEG	NEG	NEG	NEG	NEG	NEG	NEG	POS	NEG	NEG	NEG	NEG	NEG	NEG	NEG	NEG	NEG
BSAC27 (UK)	NEG	NEG	NEG	NEG	NEG	NEG	NEG	NEG	POS	NEG	NEG	POS	NEG	NEG	NEG	NEG	NEG	NEG
NCTC13131, UK-EMRSA-4 (UK)	NEG	NEG	NEG	NEG	NEG	NEG	NEG	NEG	POS	NEG	NEG	POS	NEG	NEG	NEG	NEG	NEG	NEG
	NEG	NEG	NEG	NEG	NEG	NEG	NEG	NEG	POS	NEG	NEG	POS	NEG	NEG	NEG	NEG	NEG	NEG
Algiers_HT20040080	NEG	NEG	NEG	NEG	NEG	NEG	NEG	NEG	POS	NEG	NEG	NEG	NEG	NEG	NEG	NEG	NEG	NEG
Dublin-DSH_AR09_0.0066	NEG	NEG	NEG	NEG	NEG	NEG	NEG	NEG	POS	NEG	NEG	NEG	NEG	NEG	NEG	NEG	NEG	NEG
Dublin-DSH_AR09_0-0065	NEG	NEG	NEG	NEG	NEG	NEG	NEG	NEG	POS	NEG	NEG	NEG	NEG	NEG	NEG	NEG	NEG	NEG
Dublin-DSH_Phenotype-III_84	POS	NEG	NEG	POS	NEG	NEG	NEG	NEG	POS	NEG	NEG	POS	NEG	NEG	NEG	NEG	NEG	NEG
Hong Kong_130	POS	NEG	NEG	POS	NEG	NEG	NEG	NEG	POS	NEG	NEG	NEG	POS	NEG	NEG	POS	NEG	NEG
Kuwait_018	POS	NEG	NEG	POS	NEG	NEG	NEG	NEG	POS	NEG	NEG	NEG	NEG	NEG	NEG	NEG	NEG	NEG
Perth_08-17726	NEG	NEG	NEG	NEG	NEG	NEG	NEG	NEG	POS	NEG	NEG	NEG	NEG	NEG	NEG	NEG	NEG	NEG
Riyadh_Alfaisal/KKKSUH_86_MRS-14-279	NEG	NEG	NEG	NEG	NEG	NEG	NEG	NEG	POS	NEG	NEG	POS	NEG	NEG	NEG	NEG	NEG	NEG
Riyadh_Alfaisal-04_Z3861_831588	NEG	NEG	NEG	NEG	NEG	NEG	NEG	NEG	POS	NEG	NEG	POS	NEG	NEG	NEG	NEG	NEG	NEG
Riyadh_Alfaisal-30_S15724_1108013	NEG	NEG	NEG	NEG	NEG	NEG	NEG	NEG	POS	NEG	NEG	POS	NEG	NEG	NEG	NEG	NEG	NEG
Riyadh_Alfaisal-6_22A_13_B3992_397721	NEG	NEG	NEG	NEG	NEG	NEG	NEG	NEG	POS	NEG	NEG	POS	NEG	NEG	NEG	NEG	NEG	NEG
Riyadh_SSTI_52_3615482	NEG	NEG	NEG	NEG	NEG	NEG	NEG	NEG	POS	NEG	NEG	POS	NEG	NEG	NEG	NEG	NEG	NEG
Riyadh-2817276-2	NEG	NEG	NEG	NEG	NEG	NEG	NEG	NEG	POS	NEG	AMB	POS	NEG	NEG	NEG	NEG	NEG	NEG
Riyadh-2891670-W	NEG	NEG	NEG	NEG	NEG	NEG	NEG	NEG	POS	NEG	AMB	POS	NEG	NEG	NEG	NEG	NEG	NEG
Riyadh-3006920-W	NEG	NEG	NEG	NEG	NEG	NEG	NEG	NEG	POS	NEG	NEG	POS	NEG	NEG	NEG	NEG	NEG	NEG
Riyadh-R2567782	NEG	NEG	NEG	NEG	NEG	NEG	NEG	NEG	POS	NEG	NEG	POS	NEG	NEG	NEG	NEG	NEG	NEG
Russia-18_0252_Krasnoyarsk_SK2	NEG	NEG	NEG	NEG	NEG	NEG	NEG	NEG	POS	NEG	NEG	NEG	NEG	NEG	NEG	NEG	NEG	NEG
UK-EMRSA-7	NEG	NEG	NEG	NEG	NEG	NEG	NEG	NEG	POS	NEG	NEG	POS	AMB	NEG	NEG	POS	NEG	NEG
>"South American/ Midd	NEG											NEG	POS					
Dublin-DSH_AR23_0073	NEG	NEG	NEG	NEG	NEG	NEG	NEG	NEG	POS	NEG	NEG	NEG	POS	POS	NEG	NEG	NEG	NEG
>Related to "South Ame	VAR											NEG	NEG					
MRSA-OC3 (Russia)	POS	NEG	NEG	POS	NEG	NEG	NEG	NEG	POS	NEG	NEG	NEG	NEG	NEG	NEG	NEG	NEG	NEG
Russia-12_0176_Krasnoyarsk	POS	NEG	NEG	POS	NEG	NEG	NEG	NEG	POS	NEG	NEG	NEG	NEG	NEG	NEG	NEG	NEG	NEG
Russia-13_0180_Krasnoyarsk	POS	NEG	NEG	POS	NEG	NEG	NEG	NEG	POS	NEG	NEG	NEG	NEG	NEG	NEG	NEG	NEG	NEG
Russia-16_0249_Krasnoyarsk	NEG	NEG	NEG	NEG	NEG	NEG	NEG	NEG	POS	NEG	NEG	NEG	NEG	NEG	NEG	NEG	NEG	NEG
Russia-17_0250_Krasnoyarsk	POS	NEG	NEG	POS	NEG	NEG	NEG	NEG	POS	NEG	NEG	NEG	NEG	NEG	NEG	NEG	NEG	NEG
>Related to "South Ame	NEG											NEG	NEG					
URU110 (Uruguay)	NEG	NEG	NEG	NEG	NEG	NEG	NEG	NEG	POS	NEG	NEG	NEG	NEG	NEG	NEG	NEG	NEG	NEG
>Related to "South Ame	NEG											NEG	NEG					
DS_014 (Thailand)	NEG	NEG	NEG	NEG	NEG	NEG	NEG	NEG	POS	NEG	NEG	NEG	NEG	NEG	NEG	NEG	NEG	NEG
>Unassigned Middle Eas	VAR											NEG	NEG					
Frankfurt_Oder_0490031797 (Libya)	POS	POS	NEG	NEG	NEG	NEG	NEG	NEG	POS	NEG	NEG	NEG	NEG	NEG	NEG	NEG	NEG	NEG
Kuwait_192	NEG	NEG	NEG	NEG	NEG	NEG	NEG	NEG	POS	NEG	AMB	NEG	NEG	NEG	NEG	NEG	NEG	NEG
Riyadh-9	NEG	NEG	NEG	NEG	NEG	NEG	NEG	NEG	POS	NEG	AMB	NEG	NEG	NEG	NEG	NEG	NEG	NEG
Russia-24_0407_Moscow	NEG	NEG	NEG	NEG	NEG	NEG	NEG	NEG	POS	NEG	NEG	NEG	NEG	NEG	NEG	NEG	NEG	NEG

STRAIN / ISOLATE	RESISTANCE : MISCELLANEOUS GENES				TOX.SCHOCK.TOXIN			VIRULENCE : ENTEROTOXINS									
	smr	vanA	vanB	vanZ	tst1			sea			seB	seC/L	seD	seD2	seE	seH	seJ
	smr (AL1)	vanA	vanB	vanZ	tst1 (consensus)	tst1 ("human" allele)	tst1 ("bovine" allele)	entA	entA (320E)	entA (N315) / entP	entB	entC	entD	entD2	entE	entH	entJ
		vancomycin resistance gene	vancomycin resistance gene from enterococci and Clostridium	teicoplanin resistance gene from enterococci	toxic shock syndrome toxin 1			Enterotoxin A	Enterotoxin A, allele from strain 320E	Enterotoxin A, allele from strain N315 = Entero-toxin P	Enterotoxin B	Enterotoxin C	Enterotoxin D	Enterotoxin similar to EntD	Enterotoxin E	Enterotoxin H	Enterotoxin J
**>"Eurasian Clade": TUR1**																	
TUR1 (Turkey)	NEG	NEG	NEG	NEG	NEG	NEG	NEG	POS	NEG	NEG	NEG	NEG	NEG	NEG	NEG	NEG	NEG
TUR9 (Turkey)	NEG	NEG	NEG	NEG	NEG	NEG	NEG	POS	NEG	NEG	NEG	NEG	NEG	NEG	NEG	NEG	NEG
**>"Eurasian Clade" : T013**																	
T0131 (China)	NEG	NEG	NEG	NEG	NEG	NEG	NEG	POS	NEG	NEG	NEG	NEG	NEG	NEG	NEG	NEG	NEG
MU4 (Turkey)	NEG	NEG	NEG	NEG	NEG	NEG	NEG	POS	NEG	NEG	NEG	NEG	NEG	NEG	NEG	NEG	NEG
DEU11 (Turkey)	NEG	NEG	NEG	NEG	NEG	NEG	NEG	POS	NEG	NEG	NEG	NEG	NEG	NEG	NEG	NEG	NEG
DEU16 (Turkey)	NEG	NEG	NEG	NEG	NEG	NEG	NEG	POS	NEG	NEG	NEG	NEG	NEG	NEG	NEG	NEG	NEG
Romania_Iasi_MRSA-07_54	NEG	NEG	NEG	NEG	NEG	NEG	NEG	POS	NEG	NEG	NEG	NEG	NEG	NEG	NEG	NEG	NEG
**>"Eurasian Clade": arsC-**																	
Romania_Iasi_MRSA-05_46	NEG	NEG	NEG	NEG	NEG	NEG	NEG	POS	NEG	NEG	NEG	NEG	NEG	NEG	NEG	NEG	NEG
Romania_Iasi_MRSA-06_47	NEG	NEG	NEG	NEG	NEG	NEG	NEG	POS	NEG	NEG	NEG	NEG	NEG	NEG	NEG	NEG	NEG
**>"Eurasian Clade": 16K/**																	
CN79 (China)	NEG	NEG	NEG	NEG	NEG	NEG	NEG	POS	NEG	NEG	NEG	NEG	NEG	NEG	NEG	NEG	NEG
CUHK_BJ2002 (China)	NEG	NEG	NEG	NEG	NEG	NEG	NEG	POS	NEG	NEG	NEG	NEG	NEG	NEG	NEG	NEG	NEG
CUHK_BJ2007 (China)	NEG	NEG	NEG	NEG	NEG	NEG	NEG	POS	NEG	NEG	NEG	NEG	NEG	NEG	NEG	NEG	NEG
3HK (Czech Republic)	NEG	NEG	NEG	NEG	NEG	NEG	NEG	POS	NEG	NEG	NEG	NEG	NEG	NEG	NEG	NEG	NEG
HU109 (Hungary)	NEG	NEG	NEG	NEG	NEG	NEG	NEG	POS	NEG	NEG	NEG	NEG	NEG	NEG	NEG	NEG	NEG
HUR18 (Hungary)	NEG	NEG	NEG	NEG	NEG	NEG	NEG	POS	NEG	NEG	NEG	NEG	NEG	NEG	NEG	NEG	NEG
H482 (Romania)	NEG	NEG	NEG	NEG	NEG	NEG	NEG	POS	NEG	NEG	NEG	NEG	NEG	NEG	NEG	NEG	NEG
16K (Russia)	NEG	NEG	NEG	NEG	NEG	NEG	NEG	POS	NEG	NEG	NEG	NEG	NEG	NEG	NEG	NEG	NEG
DEU10 (Turkey)	NEG	NEG	NEG	NEG	NEG	NEG	NEG	POS	NEG	NEG	NEG	NEG	NEG	NEG	NEG	NEG	NEG
DEU12 (Turkey)	NEG	NEG	NEG	NEG	NEG	NEG	NEG	POS	NEG	NEG	NEG	NEG	NEG	NEG	NEG	NEG	NEG
DEU14 (Turkey)	NEG	NEG	NEG	NEG	NEG	NEG	NEG	POS	NEG	NEG	NEG	NEG	NEG	NEG	NEG	NEG	NEG
DEU15 (Turkey)	NEG	NEG	NEG	NEG	NEG	NEG	NEG	POS	NEG	NEG	NEG	NEG	NEG	NEG	NEG	NEG	NEG
DEU17 (Turkey)	NEG	NEG	NEG	NEG	NEG	NEG	NEG	POS	NEG	NEG	NEG	NEG	NEG	NEG	NEG	NEG	NEG
DEU19 (Turkey)	NEG	NEG	NEG	NEG	NEG	NEG	NEG	POS	NEG	NEG	NEG	NEG	NEG	NEG	NEG	NEG	NEG
DEU2 (Turkey)	NEG	NEG	NEG	NEG	NEG	NEG	NEG	POS	NEG	NEG	NEG	NEG	NEG	NEG	NEG	NEG	NEG
DEU20 (Turkey)	NEG	NEG	NEG	NEG	NEG	NEG	NEG	POS	NEG	NEG	NEG	NEG	NEG	NEG	NEG	NEG	NEG
DEU23 (Turkey)	NEG	NEG	NEG	NEG	NEG	NEG	NEG	POS	NEG	NEG	NEG	NEG	NEG	NEG	NEG	NEG	NEG
DEU3 (Turkey)	NEG	NEG	NEG	NEG	NEG	NEG	NEG	POS	NEG	NEG	NEG	NEG	NEG	NEG	NEG	NEG	NEG
DEU5 (Turkey)	NEG	NEG	NEG	NEG	NEG	NEG	NEG	POS	NEG	NEG	NEG	NEG	NEG	NEG	NEG	NEG	NEG
DEU6 (Turkey)	NEG	NEG	NEG	NEG	NEG	NEG	NEG	POS	NEG	NEG	NEG	NEG	NEG	NEG	NEG	NEG	NEG
DEU8 (Turkey)	NEG	NEG	NEG	NEG	NEG	NEG	NEG	POS	NEG	NEG	NEG	NEG	NEG	NEG	NEG	NEG	NEG
HU11 (Turkey)	NEG	NEG	NEG	NEG	NEG	NEG	NEG	POS	NEG	NEG	NEG	NEG	NEG	NEG	NEG	NEG	NEG
HU13 (Turkey)	NEG	NEG	NEG	NEG	NEG	NEG	NEG	POS	NEG	NEG	NEG	NEG	NEG	NEG	NEG	NEG	NEG
HU14 (Turkey)	NEG	NEG	NEG	NEG	NEG	NEG	NEG	POS	NEG	NEG	NEG	NEG	NEG	NEG	NEG	NEG	NEG
HU15 (Turkey)	NEG	NEG	NEG	NEG	NEG	NEG	NEG	POS	NEG	NEG	NEG	NEG	NEG	NEG	NEG	NEG	NEG
HU16 (Turkey)	NEG	NEG	NEG	NEG	NEG	NEG	NEG	POS	NEG	NEG	NEG	NEG	NEG	NEG	NEG	NEG	NEG
HU17 (Turkey)	NEG	NEG	NEG	NEG	NEG	NEG	NEG	POS	NEG	NEG	NEG	NEG	NEG	NEG	NEG	NEG	NEG
HU21 (Turkey)	NEG	NEG	NEG	NEG	NEG	NEG	NEG	POS	NEG	NEG	NEG	NEG	NEG	NEG	NEG	NEG	NEG
HU23 (Turkey)	NEG	NEG	NEG	NEG	NEG	NEG	NEG	POS	NEG	NEG	NEG	NEG	NEG	NEG	NEG	NEG	NEG
HU26 (Turkey)	NEG	NEG	NEG	NEG	NEG	NEG	NEG	POS	NEG	NEG	NEG	NEG	NEG	NEG	NEG	NEG	NEG
HU4 (Turkey)	NEG	NEG	NEG	NEG	NEG	NEG	NEG	POS	NEG	NEG	NEG	NEG	NEG	NEG	NEG	NEG	NEG
HU5 (Turkey)	NEG	NEG	NEG	NEG	NEG	NEG	NEG	POS	NEG	NEG	NEG	NEG	NEG	NEG	NEG	NEG	NEG
HU6 (Turkey)	NEG	NEG	NEG	NEG	NEG	NEG	NEG	POS	NEG	NEG	NEG	NEG	NEG	NEG	NEG	NEG	NEG
HU7 (Turkey)	NEG	NEG	NEG	NEG	NEG	NEG	NEG	POS	NEG	NEG	NEG	NEG	NEG	NEG	NEG	NEG	NEG
HU8 (Turkey)	NEG	NEG	NEG	NEG	NEG	NEG	NEG	POS	NEG	NEG	NEG	NEG	NEG	NEG	NEG	NEG	NEG
HU9 (Turkey)	NEG	NEG	NEG	NEG	NEG	NEG	NEG	POS	NEG	NEG	NEG	NEG	NEG	NEG	NEG	NEG	NEG
IU1 (Turkey)	NEG	NEG	NEG	NEG	NEG	NEG	NEG	POS	NEG	NEG	NEG	NEG	NEG	NEG	NEG	NEG	NEG
IU10 (Turkey)	NEG	NEG	NEG	NEG	NEG	NEG	NEG	POS	NEG	NEG	NEG	NEG	NEG	NEG	NEG	NEG	NEG
IU11 (Turkey)	NEG	NEG	NEG	NEG	NEG	NEG	NEG	POS	NEG	NEG	NEG	NEG	NEG	NEG	NEG	NEG	NEG
IU12 (Turkey)	NEG	NEG	NEG	NEG	NEG	NEG	NEG	POS	NEG	NEG	NEG	NEG	NEG	NEG	NEG	NEG	NEG
IU13 (Turkey)	NEG	NEG	NEG	NEG	NEG	NEG	NEG	POS	NEG	NEG	NEG	NEG	NEG	NEG	NEG	NEG	NEG
IU15 (Turkey)	NEG	NEG	NEG	NEG	NEG	NEG	NEG	POS	NEG	NEG	NEG	NEG	NEG	NEG	NEG	NEG	NEG
IU18 (Turkey)	NEG	NEG	NEG	NEG	NEG	NEG	NEG	POS	NEG	NEG	NEG	NEG	NEG	NEG	NEG	NEG	NEG
IU19 (Turkey)	NEG	NEG	NEG	NEG	NEG	NEG	NEG	POS	NEG	NEG	NEG	NEG	NEG	NEG	NEG	NEG	NEG
IU2 (Turkey)	NEG	NEG	NEG	NEG	NEG	NEG	NEG	POS	NEG	NEG	NEG	NEG	NEG	NEG	NEG	NEG	NEG
IU4 (Turkey)	NEG	NEG	NEG	NEG	NEG	NEG	NEG	POS	NEG	NEG	NEG	NEG	NEG	NEG	NEG	NEG	NEG
IU6 (Turkey)	NEG	NEG	NEG	NEG	NEG	NEG	NEG	POS	NEG	NEG	NEG	NEG	NEG	NEG	NEG	NEG	NEG
IU7 (Turkey)	NEG	NEG	NEG	NEG	NEG	NEG	NEG	POS	NEG	NEG	NEG	NEG	NEG	NEG	NEG	NEG	NEG
IU9 (Turkey)	NEG	NEG	NEG	NEG	NEG	NEG	NEG	POS	NEG	NEG	NEG	NEG	NEG	NEG	NEG	NEG	NEG
MU1 (Turkey)	NEG	NEG	NEG	NEG	NEG	NEG	NEG	POS	NEG	NEG	NEG	NEG	NEG	NEG	NEG	NEG	NEG
MU10 (Turkey)	NEG	NEG	NEG	NEG	NEG	NEG	NEG	POS	NEG	NEG	NEG	NEG	NEG	NEG	NEG	NEG	NEG
MU20 (Turkey)	NEG	NEG	NEG	NEG	NEG	NEG	NEG	POS	NEG	NEG	NEG	NEG	NEG	NEG	NEG	NEG	NEG
Mu3 (Turkey)	NEG	NEG	NEG	NEG	NEG	NEG	NEG	POS	NEG	NEG	NEG	NEG	NEG	NEG	NEG	NEG	NEG
MU5 (Turkey)	NEG	NEG	NEG	NEG	NEG	NEG	NEG	POS	NEG	NEG	NEG	NEG	NEG	NEG	NEG	NEG	NEG
MU6 (Turkey)	NEG	NEG	NEG	NEG	NEG	NEG	NEG	POS	NEG	NEG	NEG	NEG	NEG	NEG	NEG	NEG	NEG
MU7 (Turkey)	NEG	NEG	NEG	NEG	NEG	NEG	NEG	NEG	NEG	NEG	NEG	NEG	NEG	NEG	NEG	NEG	NEG
TUR27 (Turkey)	NEG	NEG	NEG	NEG	NEG	NEG	NEG	POS	NEG	NEG	NEG	NEG	NEG	NEG	NEG	NEG	NEG
NCTR 32S	NEG	NEG	NEG	NEG	NEG	NEG	NEG	POS	NEG	NEG	NEG	NEG	NEG	NEG	NEG	NEG	NEG
SA02_A4 = Russia-06_0085_Moscow	NEG	NEG	NEG	NEG	NEG	NEG	NEG	POS	NEG	NEG	NEG	NEG	NEG	NEG	NEG	NEG	NEG
	NEG	NEG	NEG	NEG	NEG	NEG	NEG	POS	NEG	NEG	NEG	NEG	NEG	NEG	NEG	NEG	NEG
Dresden_08V35987 (Turkey)	NEG	NEG	NEG	NEG	NEG	NEG	NEG	POS	NEG	NEG	NEG	NEG	NEG	NEG	NEG	NEG	NEG
Dresden_17ANR577152	NEG	NEG	NEG	NEG	NEG	NEG	NEG	POS	NEG	NEG	NEG	NEG	NEG	NEG	NEG	NEG	NEG
Dresden_17ANR580374 (Makedonia)	NEG	NEG	NEG	NEG	NEG	NEG	NEG	NEG	NEG	NEG	NEG	NEG	NEG	NEG	NEG	NEG	NEG
Hong Kong_69-II	NEG	NEG	NEG	NEG	NEG	NEG	NEG	POS	NEG	NEG	NEG	NEG	NEG	NEG	NEG	NEG	NEG
Hong Kong_93	NEG	NEG	NEG	NEG	NEG	NEG	NEG	POS	NEG	NEG	NEG	NEG	NEG	NEG	NEG	NEG	NEG
Rawalpindi_Kidney Center_03_SK1	NEG	NEG	NEG	NEG	NEG	NEG	NEG	POS	NEG	NEG	NEG	NEG	NEG	NEG	NEG	NEG	NEG
Rawalpindi_Kidney Center_08	NEG	NEG	NEG	NEG	NEG	NEG	NEG	POS	NEG	NEG	NEG	NEG	NEG	NEG	NEG	NEG	NEG
Rawalpindi_Kidney Center_10	NEG	NEG	NEG	NEG	NEG	NEG	NEG	POS	NEG	NEG	NEG	NEG	NEG	NEG	NEG	NEG	NEG
Rawalpindi_Kidney Center_19	NEG	NEG	NEG	NEG	NEG	NEG	NEG	POS	AMB	NEG	NEG	NEG	AMB	NEG	NEG	NEG	NEG
Rawalpindi_Kidney Center_50_SK1	NEG	NEG	NEG	NEG	NEG	NEG	NEG	POS	NEG	NEG	NEG	NEG	NEG	NEG	NEG	NEG	NEG
Romania_Iasi_BC-49_430	NEG	NEG	NEG	NEG	NEG	NEG	NEG	POS	AMB	NEG	NEG	NEG	NEG	NEG	NEG	NEG	NEG
Romania_Iasi_MRSA_284	NEG	NEG	NEG	NEG	NEG	NEG	NEG	POS	NEG	NEG	NEG	NEG	NEG	NEG	NEG	NEG	NEG
Romania_Iasi_MRSA-32_318	NEG	NEG	NEG	NEG	NEG	NEG	NEG	POS	NEG	NEG	NEG	NEG	NEG	NEG	NEG	NEG	NEG
Romania_Iasi_MRSA-54_430	NEG	NEG	NEG	NEG	NEG	AMB	NEG	POS	AMB	NEG	NEG	NEG	NEG	NEG	NEG	NEG	NEG
Romania_Iasi_SSTI-01_2	NEG	NEG	NEG	NEG	NEG	NEG	NEG	POS	NEG	NEG	NEG	NEG	NEG	NEG	NEG	NEG	NEG
Romania_Iasi_SSTI-10_101	NEG	NEG	NEG	NEG	NEG	NEG	NEG	POS	NEG	NEG	NEG	NEG	NEG	NEG	NEG	NEG	NEG
Romania_Iasi_SSTI-11_106	NEG	NEG	NEG	NEG	NEG	NEG	NEG	POS	NEG	NEG	NEG	NEG	NEG	NEG	NEG	NEG	NEG
Russia-01_0001_SaintPetersburg	NEG	NEG	NEG	NEG	NEG	NEG	NEG	NEG	NEG	POS	NEG	NEG	NEG	NEG	NEG	NEG	NEG
Russia-02_0004_SaintPetersburg	NEG	NEG	NEG	NEG	NEG	NEG	NEG	NEG	NEG	POS	NEG	NEG	NEG	NEG	NEG	NEG	NEG
Russia-03_0057_SaintPetersburg	NEG	NEG	NEG	NEG	NEG	NEG	NEG	NEG	NEG	POS	NEG	NEG	NEG	NEG	NEG	NEG	NEG
Russia-04_0076_Moscow	NEG	NEG	NEG	NEG	NEG	NEG	NEG	POS	NEG	NEG	NEG	NEG	NEG	NEG	NEG	NEG	NEG
Russia-05_0078_Moscow	NEG	NEG	NEG	NEG	NEG	NEG	NEG	POS	NEG	NEG	NEG	NEG	NEG	NEG	NEG	NEG	NEG
Russia-08_0135_Moscow	NEG	NEG	NEG	NEG	NEG	NEG	NEG	POS	NEG	NEG	NEG	NEG	NEG	NEG	NEG	NEG	NEG
Russia-09_0150_Kurgan	NEG	NEG	NEG	NEG	NEG	NEG	NEG	POS	NEG	NEG	NEG	NEG	NEG	NEG	NEG	NEG	NEG
Russia-10_0162_Kurgan	NEG	NEG	NEG	NEG	NEG	NEG	NEG	POS	NEG	NEG	NEG	NEG	NEG	NEG	NEG	NEG	NEG
Russia-14_0184_Moscow	NEG	NEG	NEG	NEG	NEG	NEG	NEG	POS	NEG	NEG	NEG	NEG	NEG	NEG	NEG	NEG	NEG
Russia-15_0232_SaintPetersburg	NEG	NEG	NEG	NEG	NEG	NEG	NEG	POS	NEG	NEG	NEG	NEG	NEG	NEG	NEG	NEG	NEG
Russia-22_0391_Chelyabinsk	NEG	NEG	NEG	NEG	NEG	NEG	NEG	POS	NEG	NEG	NEG	NEG	NEG	NEG	NEG	NEG	NEG
Russia-23_0392_Chelyabinsk	NEG	NEG	NEG	NEG	NEG	NEG	NEG	POS	NEG	NEG	NEG	NEG	NEG	NEG	NEG	NEG	NEG
**>"Eurasian Clade" : IU17**																	
IU17 (Turkey)	NEG	NEG	NEG	NEG	NEG	NEG	NEG	POS	NEG	NEG	NEG	NEG	NEG	NEG	NEG	NEG	NEG
IU20 (Turkey)	NEG	NEG	NEG	NEG	NEG	NEG	NEG	POS	NEG	NEG	NEG	NEG	NEG	NEG	NEG	NEG	NEG

STRAIN / ISOLATE	RESISTANCE : MISCELLANEOUS GENES				TOX.SCHOCK.TOXIN			VIRULENCE : ENTEROTOXINS									
	smr	vanA	vanB	vanZ	tst1			sea			seB	seC/L	seD	seD2	seE	seH	seJ
	smr (AL1)	vanA	vanB	vanZ	tst1 (consensus)	tst1 ("human" allele)	tst1 ("bovine" allele)	entA	entA (320E)	entA (N315) / entP	entB	entC	entD	entD2	entE	entH	entJ
		vancomycin resistance gene	vancomycin resistance gene from enterococci and Clostridium	teicoplanin resistance gene from enterococci	toxic shock syndrome toxin 1			Enterotoxin A	Enterotoxin A, allele from strain 320E	Enterotoxin A, allele from strain N315 = Entero-toxin P	Enterotoxin B	Enterotoxin C	Enterotoxin D	Enterotoxin similar to EntD	Enterotoxin E	Enterotoxin H	Enterotoxin J
>"Eurasian Clade": Spor																	
Dresden_16ANR581769	NEG	NEG	NEG	NEG	NEG	NEG	NEG	POS	NEG	NEG	NEG	NEG	NEG	NEG	NEG	NEG	NEG
Dresden_17ANR578769	NEG	NEG	NEG	NEG	NEG	NEG	NEG	POS	NEG	NEG	NEG	NEG	NEG	NEG	NEG	NEG	NEG
>"Eurasian Clade": Kuwa																	
Kuwait_306	NEG	NEG	NEG	NEG	NEG	NEG	NEG	POS	POS	NEG	NEG	NEG	POS	NEG	NEG	NEG	NEG
Kuwait_301	NEG	NEG	NEG	NEG	NEG	NEG	NEG	POS	AMB	NEG	NEG	NEG	NEG	NEG	NEG	NEG	NEG
>"European Clade": "Gr																	
GRE18 (Greece)	NEG	NEG	NEG	NEG	NEG	NEG	NEG	POS	NEG	NEG	NEG	NEG	NEG	NEG	NEG	NEG	NEG
GRE317 (Greece)	NEG	NEG	NEG	NEG	NEG	NEG	NEG	POS	NEG	NEG	NEG	NEG	NEG	NEG	NEG	NEG	NEG
GRE4 (Greece)	NEG	NEG	NEG	NEG	NEG	NEG	NEG	POS	NEG	NEG	NEG	NEG	NEG	NEG	NEG	NEG	NEG
R35 (USA)	NEG	NEG	NEG	NEG	NEG	NEG	NEG	POS	NEG	NEG	NEG	NEG	NEG	NEG	NEG	NEG	NEG
Casablanca_HT20060548	NEG	NEG	NEG	NEG	NEG	NEG	NEG	POS	NEG	NEG	NEG	NEG	NEG	NEG	NEG	NEG	NEG
Casablanca_HT20060550	NEG	NEG	NEG	NEG	NEG	NEG	NEG	POS	NEG	NEG	NEG	NEG	NEG	NEG	NEG	NEG	NEG
Dresden_01V34572 (Greece)	NEG	NEG	NEG	NEG	NEG	NEG	NEG	POS	NEG	NEG	NEG	NEG	NEG	NEG	NEG	NEG	NEG
Dresden_01V36469	NEG	NEG	NEG	NEG	NEG	NEG	NEG	POS	NEG	NEG	NEG	NEG	NEG	NEG	NEG	NEG	NEG
Dresden_01V39123	NEG	NEG	NEG	NEG	NEG	AMB	NEG	POS	NEG	NEG	NEG	NEG	NEG	NEG	NEG	NEG	NEG
Dresden_01V39143	NEG	NEG	NEG	NEG	NEG	NEG	NEG	POS	NEG	NEG	NEG	NEG	NEG	NEG	NEG	NEG	NEG
Greece 1_3680_Harmony	NEG	NEG	NEG	NEG	NEG	NEG	NEG	AMB	NEG	NEG	NEG	NEG	NEG	NEG	NEG	NEG	NEG
>"European Clade": UK-																	
ANS46 (Australia)	NEG	NEG	NEG	NEG	NEG	NEG	NEG	POS	NEG	NEG	NEG	NEG	NEG	NEG	NEG	NEG	NEG
LHH1 (USA)	NEG	NEG	NEG	NEG	NEG	NEG	NEG	POS	NEG	NEG	NEG	NEG	NEG	NEG	NEG	NEG	NEG
Dublin-DSH_AR01_0-1085	NEG	NEG	NEG	NEG	NEG	NEG	NEG	POS	NEG	NEG	NEG	NEG	NEG	NEG	NEG	NEG	NEG
Dublin-DSH_AR01_0-1118	NEG	NEG	NEG	NEG	NEG	NEG	NEG	POS	NEG	NEG	NEG	NEG	NEG	NEG	NEG	NEG	NEG
Dublin-DSH_AR15_0098	NEG	NEG	NEG	NEG	NEG	NEG	NEG	POS	NEG	NEG	NEG	NEG	NEG	NEG	NEG	NEG	NEG
Dublin-DSH_AR15_0104	NEG	NEG	NEG	NEG	NEG	NEG	NEG	POS	NEG	NEG	NEG	NEG	NEG	NEG	NEG	NEG	NEG
UK_NCTC11939_UK-1_Harmony	NEG	NEG	NEG	NEG	NEG	NEG	NEG	POS	NEG	NEG	NEG	NEG	NEG	NEG	NEG	NEG	NEG
>"European Clade": BK2																	
BK2421 (USA)	NEG	NEG	NEG	NEG	NEG	NEG	NEG	POS	NEG	NEG	NEG	NEG	NEG	NEG	NEG	NEG	NEG
>"Aussi/NZ Clade", JKD6																	
JKD6009 (Australia)	NEG	NEG	NEG	NEG	NEG	NEG	NEG	POS	NEG	NEG	NEG	NEG	NEG	NEG	NEG	NEG	NEG
JKD6008 (New Zealand)	NEG	NEG	NEG	NEG	NEG	NEG	NEG	POS	NEG	NEG	NEG	NEG	NEG	NEG	NEG	NEG	NEG
Perth_2015-S20209	NEG	NEG	NEG	NEG	NEG	NEG	NEG	POS	NEG	NEG	NEG	NEG	NEG	NEG	NEG	NEG	NEG
Perth_2015-S20305	NEG	NEG	NEG	NEG	NEG	NEG	NEG	POS	NEG	NEG	NEG	NEG	NEG	NEG	NEG	NEG	NEG
Perth_2015-S20603	NEG	NEG	NEG	NEG	NEG	NEG	NEG	POS	NEG	NEG	NEG	NEG	NEG	NEG	NEG	NEG	NEG
Perth_2015-S20607	NEG	NEG	NEG	NEG	NEG	NEG	NEG	POS	NEG	NEG	NEG	NEG	NEG	NEG	NEG	NEG	NEG
Perth_2015-S20608	NEG	NEG	NEG	NEG	NEG	NEG	NEG	POS	NEG	NEG	NEG	NEG	NEG	NEG	NEG	NEG	NEG
Perth_2015-S20610	NEG	NEG	NEG	NEG	NEG	NEG	NEG	POS	NEG	NEG	NEG	NEG	NEG	NEG	NEG	NEG	NEG
Perth_2015-S20613	NEG	NEG	NEG	NEG	NEG	NEG	NEG	POS	NEG	NEG	NEG	NEG	NEG	NEG	NEG	NEG	NEG
Perth_2015-S20617	NEG	NEG	NEG	NEG	NEG	NEG	NEG	POS	NEG	NEG	NEG	NEG	NEG	NEG	NEG	NEG	NEG
Perth_2015-S20622	NEG	NEG	NEG	NEG	NEG	NEG	NEG	POS	NEG	NEG	NEG	NEG	NEG	NEG	NEG	NEG	NEG
Perth_2015-S23619	NEG	NEG	NEG	NEG	NEG	NEG	NEG	POS	NEG	NEG	NEG	NEG	NEG	NEG	NEG	NEG	NEG
Perth_2015-S41210	NEG	NEG	NEG	NEG	NEG	NEG	NEG	POS	NEG	NEG	NEG	NEG	NEG	NEG	NEG	NEG	NEG
Perth_2015-S51001	NEG	NEG	NEG	NEG	NEG	NEG	NEG	NEG	NEG	NEG	NEG	NEG	NEG	NEG	NEG	NEG	NEG
Perth_2015-S51003	NEG	NEG	NEG	NEG	NEG	NEG	NEG	NEG	NEG	NEG	NEG	NEG	NEG	NEG	NEG	NEG	NEG
Perth_2015-S51006	NEG	NEG	NEG	NEG	NEG	NEG	NEG	NEG	NEG	NEG	NEG	NEG	NEG	NEG	NEG	NEG	NEG
Perth_2015-S51007	NEG	NEG	NEG	NEG	NEG	NEG	NEG	NEG	NEG	NEG	NEG	NEG	NEG	NEG	NEG	NEG	NEG
Perth_2015-S51008	NEG	NEG	NEG	NEG	NEG	NEG	NEG	NEG	NEG	NEG	NEG	NEG	NEG	NEG	NEG	NEG	NEG
Perth_2015-S51014	NEG	NEG	NEG	NEG	NEG	NEG	NEG	NEG	NEG	NEG	NEG	NEG	NEG	NEG	NEG	NEG	NEG
Perth_2015-S51408	NEG	NEG	NEG	NEG	NEG	NEG	NEG	POS	NEG	NEG	NEG	NEG	NEG	NEG	NEG	NEG	NEG
Perth_2016-S20614	NEG	NEG	NEG	NEG	NEG	NEG	NEG	POS	NEG	NEG	NEG	NEG	NEG	NEG	NEG	NEG	NEG
Perth_2016-S23614	NEG	NEG	NEG	NEG	NEG	NEG	NEG	POS	NEG	NEG	NEG	NEG	NEG	NEG	NEG	NEG	NEG
Perth_2016-S42009	NEG	NEG	NEG	NEG	NEG	NEG	NEG	POS	NEG	NEG	NEG	NEG	NEG	NEG	NEG	NEG	NEG
Perth_2016-S51001	NEG	NEG	NEG	NEG	NEG	NEG	NEG	NEG	NEG	NEG	NEG	NEG	NEG	NEG	NEG	NEG	NEG
Perth_2016-S51403	NEG	NEG	NEG	NEG	NEG	NEG	NEG	NEG	NEG	NEG	NEG	NEG	NEG	NEG	NEG	NEG	NEG
Perth_07 RPA 20	NEG	NEG	NEG	NEG	NEG	NEG	NEG	POS	NEG	NEG	NEG	NEG	NEG	NEG	NEG	NEG	NEG
Perth_08-19088	NEG	NEG	NEG	NEG	NEG	NEG	NEG	POS	NEG	NEG	NEG	NEG	NEG	NEG	NEG	NEG	NEG
Perth_01-15351	NEG	NEG	NEG	NEG	NEG	NEG	NEG	POS	NEG	NEG	NEG	NEG	NEG	NEG	NEG	NEG	NEG
Perth_01-15357	NEG	NEG	NEG	NEG	NEG	NEG	NEG	POS	NEG	NEG	NEG	NEG	NEG	NEG	NEG	NEG	NEG
Perth_01-15419	NEG	NEG	NEG	NEG	NEG	NEG	NEG	POS	NEG	NEG	NEG	NEG	NEG	NEG	NEG	NEG	NEG
Perth_01-16309	NEG	NEG	NEG	NEG	NEG	NEG	NEG	NEG	NEG	NEG	NEG	NEG	NEG	NEG	NEG	NEG	NEG
Perth_03 SNP 95	NEG	NEG	NEG	NEG	NEG	NEG	NEG	NEG	NEG	NEG	NEG	NEG	NEG	NEG	NEG	NEG	NEG
Perth_2003_AH_47	NEG	NEG	NEG	NEG	NEG	NEG	NEG	POS	NEG	NEG	NEG	NEG	NEG	NEG	NEG	NEG	NEG
>"Australian/NZ Clade":																	
Perth_2015-S20623	NEG	NEG	NEG	NEG	NEG	NEG	NEG	POS	NEG	NEG	NEG	NEG	NEG	NEG	NEG	NEG	NEG
Perth_2015-S20826	NEG	NEG	NEG	NEG	NEG	NEG	NEG	POS	NEG	NEG	NEG	NEG	NEG	NEG	NEG	NEG	NEG
Perth_05 NH 56	NEG	NEG	NEG	NEG	NEG	NEG	NEG	POS	NEG	NEG	NEG	NEG	NEG	NEG	NEG	NEG	NEG
>CC5-MRSA-III LA MRSA																	
Delbrück 38 isolate from domestic turkey	NEG	NEG	NEG	NEG	NEG	NEG	NEG	NEG	NEG	NEG	NEG	NEG	NEG	NEG	NEG	NEG	NEG
> Staph. pseudintermed																	
KM1381	NEG	NEG	NEG	NEG	NEG	NEG	NEG	NEG	NEG	NEG	NEG	NEG	NEG	NEG	NEG	NEG	NEG
>"South-East Asian Clad																	
M92 (Canada)	NEG	NEG	NEG	NEG	NEG	NEG	NEG	NEG	NEG	NEG	NEG	NEG	NEG	NEG	NEG	NEG	NEG
CHI59 (China)	NEG	NEG	NEG	NEG	NEG	NEG	NEG	NEG	NEG	NEG	NEG	NEG	NEG	NEG	NEG	NEG	NEG
CHI61 (China)	NEG	NEG	NEG	NEG	NEG	NEG	NEG	NEG	NEG	NEG	NEG	NEG	NEG	NEG	NEG	NEG	NEG
CUHK_HK1997 (China)	NEG	NEG	NEG	NEG	NEG	NEG	NEG	POS	NEG	NEG	NEG	NEG	NEG	NEG	NEG	NEG	NEG
M996 (China)	NEG	NEG	NEG	NEG	NEG	NEG	NEG	NEG	NEG	NEG	NEG	NEG	NEG	NEG	NEG	NEG	NEG
H211 (Denmark)	NEG	NEG	NEG	NEG	NEG	NEG	NEG	NEG	NEG	NEG	NEG	NEG	NEG	NEG	NEG	NEG	NEG
H216 (Denmark)	NEG	NEG	NEG	NEG	NEG	NEG	NEG	NEG	NEG	NEG	NEG	NEG	NEG	NEG	NEG	NEG	NEG
D71 (Germany)	NEG	NEG	NEG	NEG	NEG	NEG	NEG	NEG	NEG	NEG	NEG	NEG	NEG	NEG	NEG	NEG	NEG
NMR05 (India)	NEG	NEG	NEG	NEG	NEG	NEG	NEG	POS	NEG	NEG	NEG	NEG	NEG	NEG	NEG	NEG	NEG
MAL11 (Malaysia)	NEG	NEG	NEG	NEG	NEG	NEG	NEG	POS	NEG	NEG	NEG	NEG	NEG	NEG	NEG	NEG	NEG
MAL9 (Malaysia)	NEG	NEG	NEG	NEG	NEG	NEG	NEG	POS	NEG	NEG	NEG	NEG	NEG	NEG	NEG	NEG	NEG
Na21 (Sri Lanka)	NEG	NEG	NEG	NEG	NEG	NEG	NEG	POS	NEG	NEG	NEG	NEG	NEG	NEG	NEG	NEG	NEG
M592 (Syria)	NEG	NEG	NEG	NEG	NEG	NEG	NEG	NEG	NEG	NEG	NEG	NEG	NEG	NEG	NEG	NEG	NEG
H202 (Thailand)	NEG	NEG	NEG	NEG	NEG	NEG	NEG	NEG	NEG	NEG	NEG	NEG	NEG	NEG	NEG	NEG	NEG
S102 (Thailand)	NEG	NEG	NEG	NEG	NEG	NEG	NEG	NEG	NEG	NEG	NEG	NEG	NEG	NEG	NEG	NEG	NEG
S130 (Thailand)	NEG	NEG	NEG	NEG	NEG	NEG	NEG	NEG	NEG	NEG	NEG	NEG	NEG	NEG	NEG	NEG	NEG
S40 (Thailand)	NEG	NEG	NEG	NEG	NEG	NEG	NEG	NEG	NEG	NEG	NEG	NEG	NEG	NEG	NEG	NEG	NEG
S71 (Thailand)	NEG	NEG	NEG	NEG	NEG	NEG	NEG	NEG	NEG	NEG	NEG	NEG	NEG	NEG	NEG	NEG	NEG
S87 (Thailand)	NEG	NEG	NEG	NEG	NEG	NEG	NEG	NEG	NEG	NEG	NEG	NEG	NEG	NEG	NEG	NEG	NEG
S93 (Thailand)	NEG	NEG	NEG	NEG	NEG	NEG	NEG	NEG	NEG	NEG	NEG	NEG	NEG	NEG	NEG	NEG	NEG
US_002 (Thailand)	NEG	NEG	NEG	NEG	NEG	NEG	NEG	NEG	NEG	NEG	NEG	NEG	NEG	NEG	NEG	NEG	NEG
US_017 (Thailand)	NEG	NEG	NEG	NEG	NEG	NEG	NEG	NEG	NEG	NEG	NEG	NEG	NEG	NEG	NEG	NEG	NEG
WAMC6102 (Thailand)	NEG	NEG	NEG	NEG	NEG	NEG	NEG	POS	NEG	NEG	NEG	NEG	NEG	NEG	NEG	NEG	NEG
NCTC_13626_TW20 (UK)	NEG	NEG	NEG	NEG	NEG	NEG	NEG	POS	NEG	NEG	NEG	NEG	NEG	NEG	NEG	NEG	NEG
BSAC3021 (UK)	NEG	NEG	NEG	NEG	NEG	NEG	NEG	POS	NEG	NEG	NEG	NEG	NEG	NEG	NEG	NEG	NEG
BSAC3046 (UK)	NEG	NEG	NEG	NEG	NEG	NEG	NEG	POS	NEG	NEG	NEG	NEG	NEG	NEG	NEG	NEG	NEG
1193_SAUR (USA)	NEG	NEG	NEG	NEG	NEG	NEG	NEG	POS	NEG	NEG	NEG	NEG	NEG	NEG	NEG	NEG	NEG
1194_SAUR (USA)	NEG	NEG	NEG	NEG	NEG	NEG	NEG	POS	NEG	NEG	NEG	NEG	NEG	NEG	NEG	NEG	NEG

STRAIN / ISOLATE	RESISTANCE : MISCELLANEOUS GENES				TOX.SCHOCK.TOXIN			VIRULENCE : ENTEROTOXINS									
	smr	vanA	vanB	vanZ	tst1			sea			seB	seC/L	seD	seD2	seE	seH	seJ
	smr (AL1)	vanA	vanB	vanZ	tst1 (consensus)	tst1 ("human" allele)	tst1 ("bovine" allele)	entA	entA (320E)	entA (N315) / entP	entB	entC	entD	entD2	entE	entH	entJ
		vancomycin resistance gene	vancomycin resistance gene from enterococci and Clostridium	teicoplanin resistance gene from enterococci	toxic shock syndrome toxin 1			Enterotoxin A	Enterotoxin A, allele from strain 320E	Enterotoxin A, allele from strain N315 = Entero-toxin P	Enterotoxin B	Enterotoxin C	Enterotoxin D	Enterotoxin similar to EntD	Enterotoxin E	Enterotoxin H	Enterotoxin J
>"South-East Asian Clade": TW20 (Irish AR44), c																	
1195_SAUR (USA)	NEG	NEG	NEG	NEG	NEG	NEG	NEG	POS	NEG	NEG	NEG	NEG	NEG	NEG	NEG	NEG	NEG
FVRH6002 (USA)	NEG	NEG	NEG	NEG	NEG	NEG	NEG	POS	NEG	NEG	NEG	NEG	NEG	NEG	NEG	NEG	NEG
KINW6048 (USA)	NEG	NEG	NEG	NEG	NEG	NEG	NEG	POS	NEG	NEG	NEG	NEG	NEG	NEG	NEG	NEG	NEG
LAMC0011 (USA)	NEG	NEG	NEG	NEG	NEG	NEG	NEG	POS	NEG	NEG	NEG	NEG	NEG	NEG	NEG	NEG	NEG
SJOS6053 (USA)	NEG	NEG	NEG	NEG	NEG	NEG	NEG	POS	NEG	NEG	NEG	NEG	NEG	NEG	NEG	NEG	NEG
SJOS6072 (USA)	NEG	NEG	NEG	NEG	NEG	NEG	NEG	POS	NEG	NEG	NEG	NEG	NEG	NEG	NEG	NEG	NEG
UCIM6042 (USA)	NEG	NEG	NEG	NEG	NEG	NEG	NEG	POS	NEG	NEG	NEG	NEG	NEG	NEG	NEG	NEG	NEG
Perth_2015-S31902	NEG	NEG	NEG	NEG	NEG	NEG	NEG	POS	NEG	NEG	NEG	NEG	NEG	NEG	NEG	NEG	NEG
Perth_2015-S31904	NEG	NEG	NEG	NEG	NEG	NEG	NEG	POS	NEG	NEG	NEG	NEG	NEG	NEG	NEG	NEG	NEG
Perth_2015-S31913	NEG	NEG	NEG	NEG	NEG	NEG	NEG	POS	NEG	NEG	NEG	NEG	NEG	NEG	NEG	NEG	NEG
Perth_2015-S31918	NEG	NEG	NEG	NEG	NEG	NEG	NEG	POS	NEG	NEG	NEG	NEG	NEG	NEG	NEG	NEG	NEG
Dublin-DSH_AR44_E1183	NEG	NEG	NEG	NEG	NEG	NEG	NEG	POS	NEG	NEG	NEG	NEG	NEG	NEG	NEG	NEG	NEG
Dublin-DSH_Unfamiliar-3_E1520	NEG	NEG	NEG	NEG	NEG	NEG	NEG	POS	NEG	NEG	NEG	NEG	NEG	NEG	NEG	NEG	NEG
Hong Kong_042	NEG	NEG	NEG	NEG	NEG	NEG	NEG	NEG	NEG	NEG	NEG	NEG	NEG	NEG	NEG	NEG	NEG
Hong Kong_101	NEG	NEG	NEG	NEG	NEG	NEG	NEG	NEG	NEG	NEG	NEG	NEG	NEG	NEG	NEG	NEG	NEG
Hong Kong_111	NEG	NEG	NEG	NEG	NEG	NEG	NEG	POS	NEG	NEG	NEG	NEG	NEG	NEG	NEG	NEG	NEG
Hong Kong_113	NEG	NEG	NEG	NEG	NEG	NEG	NEG	NEG	NEG	NEG	NEG	NEG	NEG	NEG	NEG	NEG	NEG
Hong Kong_115	NEG	NEG	NEG	NEG	NEG	NEG	NEG	POS	NEG	NEG	NEG	NEG	NEG	NEG	NEG	NEG	NEG
Hong Kong_118	NEG	NEG	NEG	NEG	NEG	NEG	NEG	POS	NEG	NEG	NEG	NEG	NEG	NEG	NEG	NEG	NEG
Hong Kong_125	NEG	NEG	NEG	NEG	NEG	NEG	NEG	NEG	NEG	NEG	NEG	NEG	NEG	NEG	NEG	NEG	NEG
Hong Kong_129	NEG	NEG	NEG	NEG	NEG	NEG	NEG	NEG	NEG	NEG	NEG	NEG	NEG	NEG	NEG	NEG	NEG
Hong Kong_133	NEG	NEG	NEG	NEG	NEG	NEG	NEG	POS	NEG	NEG	NEG	NEG	NEG	NEG	NEG	NEG	NEG
Kuwait_009	NEG	NEG	NEG	NEG	NEG	NEG	NEG	NEG	NEG	NEG	NEG	NEG	NEG	NEG	NEG	NEG	NEG
Kuwait_103	NEG	NEG	NEG	NEG	NEG	NEG	NEG	NEG	NEG	NEG	NEG	NEG	AMB	NEG	NEG	NEG	NEG
Kuwait_107	NEG	NEG	NEG	NEG	NEG	NEG	NEG	NEG	NEG	NEG	NEG	NEG	AMB	NEG	NEG	NEG	NEG
Perth_03 AH 88	NEG	NEG	NEG	NEG	NEG	NEG	NEG	POS	NEG	NEG	NEG	NEG	NEG	NEG	NEG	NEG	NEG
Perth_08-16905	NEG	NEG	NEG	NEG	NEG	NEG	NEG	POS	NEG	NEG	NEG	NEG	NEG	NEG	NEG	NEG	NEG
Riyadh_Alfaisal-10_SICU_13_B5672_1117353	NEG	NEG	NEG	NEG	NEG	NEG	NEG	NEG	NEG	NEG	NEG	NEG	NEG	NEG	NEG	NEG	NEG
Riyadh_SSTI_17_3508602	NEG	NEG	NEG	NEG	NEG	NEG	NEG	NEG	NEG	NEG	NEG	NEG	NEG	NEG	NEG	NEG	NEG
Riyadh-2822088-R	NEG	NEG	NEG	NEG	NEG	NEG	NEG	NEG	NEG	NEG	NEG	NEG	NEG	NEG	NEG	NEG	NEG
Trinidad&Tobago_2012_195 (619/12)	NEG	NEG	NEG	NEG	NEG	NEG	NEG	NEG	NEG	NEG	NEG	NEG	NEG	NEG	NEG	NEG	NEG
Trinidad&Tobago_2012_370 (1112-52177)	NEG	NEG	NEG	NEG	NEG	NEG	NEG	NEG	NEG	NEG	NEG	NEG	NEG	NEG	NEG	NEG	NEG
Trinidad&Tobago_2012_378 (4139)	NEG	NEG	NEG	NEG	NEG	NEG	NEG	POS	NEG	NEG	NEG	NEG	NEG	NEG	NEG	NEG	NEG
Trinidad&Tobago_2013_PA21	NEG	NEG	NEG	NEG	NEG	NEG	NEG	POS	NEG	NEG	NEG	NEG	NEG	NEG	NEG	NEG	NEG
Trinidad&Tobago_2013_PA22	NEG	NEG	NEG	NEG	NEG	NEG	NEG	POS	AMB	NEG	NEG	NEG	NEG	NEG	NEG	NEG	NEG
Trinidad&Tobago_2912_340 (8523/12)	NEG	NEG	NEG	NEG	NEG	NEG	NEG	POS	NEG	NEG	NEG	NEG	NEG	NEG	NEG	NEG	NEG
Trinidad&Tobago_MRSA2010_105	NEG	NEG	NEG	NEG	NEG	NEG	NEG	POS	NEG	NEG	NEG	NEG	NEG	NEG	NEG	NEG	NEG
Trinidad&Tobago_MRSA2010_108	NEG	NEG	NEG	NEG	NEG	NEG	NEG	POS	NEG	NEG	NEG	NEG	NEG	NEG	NEG	NEG	NEG
Trinidad&Tobago_MRSA2010_33	NEG	NEG	NEG	NEG	NEG	NEG	NEG	POS	NEG	NEG	NEG	NEG	NEG	NEG	NEG	NEG	NEG
Trinidad&Tobago_MRSA2010_79	NEG	NEG	NEG	NEG	NEG	NEG	NEG	POS	NEG	NEG	NEG	NEG	NEG	NEG	NEG	NEG	NEG
Trinidad&Tobago_MRSA2010_88	NEG	NEG	NEG	NEG	NEG	NEG	NEG	POS	NEG	NEG	NEG	NEG	NEG	NEG	NEG	NEG	NEG
Trinidad&Tobago_MRSA2010_97	NEG	NEG	NEG	NEG	NEG	NEG	NEG	POS	NEG	NEG	NEG	NEG	NEG	NEG	NEG	NEG	NEG
Trinidad&Tobago_SSTI 2012_031	NEG	NEG	NEG	NEG	NEG	NEG	NEG	NEG	NEG	NEG	NEG	NEG	NEG	NEG	NEG	NEG	NEG
Uganda-29_17148_250811	NEG	NEG	NEG	NEG	NEG	NEG	NEG	NEG	NEG	NEG	NEG	NEG	NEG	NEG	NEG	NEG	NEG
>"South-East Asian Clad																	
UCIM6015	NEG	NEG	NEG	NEG	NEG	NEG	NEG	POS	NEG	NEG	NEG	NEG	NEG	NEG	NEG	NEG	NEG
Hong Kong_121	NEG	NEG	NEG	NEG	NEG	NEG	NEG	NEG	NEG	NEG	NEG	NEG	NEG	NEG	NEG	NEG	NEG
>"South-East Asian Clad																	
Z172 (Taiwan)	NEG	NEG	NEG	NEG	NEG	NEG	NEG	POS	NEG	NEG	NEG	NEG	NEG	NEG	NEG	NEG	NEG
Russia-19_0342_Kurgan	NEG	NEG	NEG	NEG	NEG	NEG	NEG	POS	NEG	NEG	NEG	NEG	NEG	NEG	NEG	NEG	NEG
>"South-East Asian Clad																	
Perth_01-15392	NEG	NEG	NEG	NEG	NEG	NEG	NEG	POS	NEG	NEG	NEG	NEG	NEG	NEG	NEG	NEG	NEG
>"South-East Asian Clad																	
XN108 (China)	NEG	NEG	NEG	NEG	NEG	NEG	NEG	POS	NEG	NEG	NEG	NEG	NEG	NEG	NEG	NEG	NEG
NMR09 (India)	NEG	NEG	NEG	NEG	NEG	NEG	NEG	POS	NEG	NEG	NEG	NEG	NEG	NEG	NEG	NEG	NEG
346 (Malaysia)	NEG	NEG	NEG	NEG	NEG	NEG	NEG	POS	NEG	NEG	NEG	NEG	NEG	NEG	NEG	NEG	NEG
DS_009 (Thailand)	NEG	NEG	NEG	NEG	NEG	NEG	NEG	NEG	NEG	NEG	NEG	NEG	NEG	NEG	NEG	NEG	NEG
18ANR581005 (Ind)	NEG	NEG	NEG	NEG	NEG	NEG	NEG	POS	NEG	NEG	NEG	NEG	NEG	NEG	NEG	NEG	NEG
Bengalaru_ST20141405	NEG	NEG	NEG	NEG	NEG	NEG	NEG	POS	NEG	NEG	NEG	NEG	NEG	NEG	NEG	NEG	NEG
Bengalaru_ST20141406	AMB	NEG	NEG	NEG	NEG	NEG	NEG	POS	NEG	NEG	NEG	NEG	NEG	NEG	NEG	NEG	NEG
Bengalaru_ST20141417	NEG	NEG	NEG	NEG	NEG	NEG	NEG	POS	NEG	NEG	NEG	NEG	NEG	NEG	NEG	NEG	NEG
Hong Kong_107	NEG	NEG	NEG	NEG	NEG	NEG	NEG	NEG	NEG	NEG	NEG	NEG	NEG	NEG	NEG	NEG	NEG
Hong Kong_12	NEG	NEG	NEG	NEG	NEG	NEG	NEG	POS	NEG	NEG	NEG	NEG	NEG	NEG	NEG	NEG	NEG
Hong Kong_132	NEG	NEG	NEG	NEG	NEG	NEG	NEG	NEG	NEG	NEG	NEG	NEG	NEG	NEG	NEG	NEG	NEG
Riyadh-3028763-R	NEG	NEG	NEG	NEG	NEG	NEG	NEG	NEG	NEG	NEG	NEG	NEG	NEG	NEG	NEG	NEG	NEG
Trinidad&Tobago_MRSA2010_94	NEG	NEG	NEG	NEG	NEG	NEG	NEG	NEG	NEG	NEG	NEG	NEG	NEG	NEG	NEG	NEG	NEG
>"South-East Asian Clad																	
M705 (Thailand)	NEG	NEG	NEG	NEG	NEG	NEG	NEG	NEG	NEG	NEG	NEG	NEG	NEG	NEG	NEG	NEG	NEG
S85 (Thailand)	NEG	NEG	NEG	NEG	NEG	NEG	NEG	NEG	NEG	NEG	NEG	NEG	NEG	NEG	NEG	NEG	NEG
Riyadh_Alfaisal-04_281014_S5076	NEG	NEG	NEG	NEG	NEG	NEG	NEG	NEG	NEG	NEG	NEG	NEG	NEG	NEG	NEG	NEG	NEG
Riyadh_Alfaisal-26_S15145_1104163	NEG	NEG	NEG	NEG	NEG	NEG	NEG	NEG	NEG	NEG	NEG	NEG	NEG	NEG	NEG	NEG	NEG
Riyadh-2817437-W	NEG	NEG	NEG	NEG	NEG	NEG	NEG	NEG	NEG	NEG	NEG	NEG	NEG	NEG	NEG	NEG	NEG
>"South-East Asian Clad																	
PPUKM-775-2009 (Malaysia)	NEG	NEG	NEG	NEG	NEG	NEG	NEG	POS	NEG	NEG	NEG	NEG	NEG	NEG	NEG	NEG	NEG
Hong Kong_138	NEG	NEG	NEG	NEG	NEG	NEG	NEG	NEG	NEG	NEG	NEG	NEG	NEG	NEG	NEG	NEG	NEG
>"South-East Asian Clad																	
Kuwait_001	NEG	NEG	NEG	NEG	NEG	NEG	NEG	POS	NEG	NEG	NEG	NEG	NEG	NEG	NEG	NEG	NEG
Kuwait_337	NEG	NEG	NEG	NEG	NEG	NEG	NEG	NEG	NEG	NEG	NEG	NEG	NEG	NEG	NEG	NEG	NEG
>"South-East Asian Clad								VAR									
CUHK_HK2007 (China)	NEG	NEG	NEG	NEG	NEG	NEG	NEG	POS	NEG	NEG	NEG	NEG	NEG	NEG	NEG	NEG	NEG
NMR02 (India)	NEG	NEG	NEG	NEG	NEG	NEG	NEG	POS	NEG	NEG	NEG	NEG	NEG	NEG	NEG	NEG	NEG
VB1490 (India)	NEG	NEG	NEG	NEG	NEG	NEG	NEG	NEG	NEG	NEG	NEG	NEG	NEG	NEG	NEG	NEG	NEG
V521 (Korea)	NEG	NEG	NEG	NEG	NEG	NEG	NEG	POS	NEG	NEG	NEG	NEG	NEG	NEG	NEG	NEG	NEG
HST-077 (Lebanon)	NEG	NEG	NEG	NEG	NEG	NEG	NEG	POS	NEG	NEG	NEG	NEG	NEG	NEG	NEG	NEG	NEG
MAL1 (Malaysia)	NEG	NEG	NEG	NEG	NEG	NEG	NEG	POS	NEG	NEG	NEG	NEG	NEG	NEG	NEG	NEG	NEG
MAL3 (Malaysia)	NEG	NEG	NEG	NEG	NEG	NEG	NEG	POS	NEG	NEG	NEG	NEG	NEG	NEG	NEG	NEG	NEG
PPUKM-261-2009 (Malaysia)	NEG	NEG	NEG	NEG	NEG	NEG	NEG	POS	NEG	NEG	NEG	NEG	NEG	NEG	NEG	NEG	NEG
PPUKM-332-2009 (Malaysia)	NEG	NEG	NEG	NEG	NEG	NEG	NEG	POS	NEG	NEG	NEG	NEG	NEG	NEG	NEG	NEG	NEG
S38 (Thailand)	NEG	NEG	NEG	NEG	NEG	NEG	NEG	POS	NEG	NEG	NEG	NEG	NEG	NEG	NEG	NEG	NEG
S7 (Thailand)	NEG	NEG	NEG	NEG	NEG	NEG	NEG	POS	NEG	NEG	NEG	NEG	NEG	NEG	NEG	NEG	NEG
US_008 (Thailand)	NEG	NEG	NEG	NEG	NEG	NEG	NEG	POS	NEG	NEG	NEG	NEG	NEG	NEG	NEG	NEG	NEG
US_030 (Thailand)	NEG	NEG	NEG	NEG	NEG	NEG	NEG	NEG	NEG	NEG	NEG	NEG	NEG	NEG	NEG	NEG	NEG
M170 (Tsunami-related)	NEG	NEG	NEG	NEG	NEG	NEG	NEG	NEG	NEG	NEG	NEG	NEG	NEG	NEG	NEG	NEG	NEG
BSAC697 (UK)	NEG	NEG	NEG	NEG	NEG	NEG	NEG	POS	NEG	NEG	NEG	NEG	NEG	NEG	NEG	NEG	NEG
IS-189	NEG	NEG	NEG	NEG	NEG	NEG	NEG	POS	NEG	NEG	NEG	NEG	NEG	NEG	NEG	NEG	NEG
MRGR3	NEG	NEG	NEG	NEG	NEG	NEG	NEG	POS	NEG	NEG	NEG	NEG	NEG	NEG	NEG	NEG	NEG
smg07	NEG	NEG	NEG	NEG	NEG	NEG	NEG	POS	NEG	NEG	NEG	NEG	NEG	NEG	NEG	NEG	NEG
AH1-AUS-EMRSA-3	NEG	NEG	NEG	NEG	NEG	NEG	NEG	POS	NEG	NEG	NEG	NEG	NEG	NEG	NEG	NEG	NEG
Bengalaru_ST20141403	NEG	NEG	NEG	NEG	NEG	NEG	NEG	POS	NEG	NEG	NEG	NEG	NEG	NEG	NEG	NEG	NEG
Finland E24_98541_Harmony	NEG	NEG	NEG	NEG	NEG	NEG	NEG	NEG	NEG	NEG	NEG	NEG	NEG	NEG	NEG	NEG	NEG
Hong Kong_10	NEG	NEG	NEG	NEG	NEG	NEG	NEG	POS	NEG	NEG	NEG	NEG	NEG	NEG	NEG	NEG	NEG
Hong Kong_100	NEG	NEG	NEG	NEG	NEG	NEG	NEG	NEG	NEG	NEG	NEG	NEG	NEG	NEG	NEG	NEG	NEG
Hong Kong_199	NEG	NEG	NEG	NEG	NEG	NEG	NEG	POS	NEG	NEG	NEG	NEG	NEG	NEG	NEG	NEG	NEG
Hong Kong_34	NEG	NEG	NEG	NEG	NEG	NEG	NEG	NEG	NEG	NEG	NEG	NEG	NEG	NEG	NEG	NEG	NEG
Hong Kong_88	NEG	NEG	NEG	NEG	NEG	NEG	NEG	POS	NEG	NEG	NEG	NEG	NEG	NEG	NEG	NEG	NEG
Hong Kong_92	NEG	NEG	NEG	NEG	NEG	NEG	NEG	POS	NEG	NEG	NEG	NEG	NEG	NEG	NEG	NEG	NEG
Hong Kong_94	NEG	NEG	NEG	NEG	NEG	NEG	NEG	POS	NEG	NEG	NEG	NEG	NEG	NEG	NEG	NEG	NEG
Kuwait_257	NEG	NEG	NEG	NEG	NEG	NEG	NEG	POS	NEG	NEG	NEG	NEG	NEG	NEG	NEG	NEG	NEG
Perth_01-15330	NEG	NEG	NEG	NEG	NEG	NEG	NEG	POS	NEG	NEG	NEG	NEG	NEG	NEG	NEG	NEG	NEG
Perth_01-15349	NEG	NEG	NEG	NEG	NEG	NEG	NEG	POS	NEG	NEG	NEG	NEG	NEG	NEG	NEG	NEG	NEG
Perth_01-16176	NEG	NEG	NEG	NEG	NEG	NEG	NEG	POS	NEG	NEG	NEG	NEG	NEG	NEG	NEG	NEG	NEG
Perth_WBG8379	NEG	NEG	NEG	NEG	NEG	NEG	NEG	POS	NEG	NEG	NEG	NEG	NEG	NEG	NEG	NEG	NEG
Rawalpindi_Kidney Center_56	NEG	NEG	NEG	NEG	NEG	NEG	NEG	NEG	AMB	NEG	NEG	NEG	AMB	NEG	NEG	AMB	NEG
Riyadh_SSTI_05_3464614	NEG	NEG	NEG	NEG	NEG	NEG	NEG	NEG	NEG	NEG	NEG	NEG	NEG	NEG	NEG	NEG	NEG
Trinidad&Tobago_2012_157	NEG	NEG	NEG	NEG	NEG	NEG	NEG	POS	AMB	NEG	NEG	NEG	NEG	NEG	NEG	NEG	NEG

STRAIN / ISOLATE	RESISTANCE : MISCELLANEOUS GENES				TOX.SCHOCK.TOXIN			VIRULENCE : ENTEROTOXINS									
	smr	vanA	vanB	vanZ	tst1			sea			seB	seC/L	seD	seD2	seE	seH	seJ
	smr (AL1)	vanA	vanB	vanZ	tst1 (consensus)	tst1 ("human" allele)	tst1 ("bovine" allele)	entA	entA (320E)	entA (N315) / entP	entB	entC	entD	entD2	entE	entH	entJ
		vancomycin resistance gene	vancomycin resistance gene from enterococci and Clostridium	teicoplanin resistance gene from enterococci	toxic shock syndrome toxin 1			Enterotoxin A	Enterotoxin A, allele from strain 320E	Enterotoxin A, allele from strain N315 = Entero-toxin P	Enterotoxin B	Enterotoxin C	Enterotoxin D	Enterotoxin similar to EntD	Enterotoxin E	Enterotoxin H	Enterotoxin J
>"South-East Asian Clad																	
M418	NEG	NEG	NEG	NEG	NEG	NEG	NEG	POS	NEG	NEG	NEG	NEG	NEG	NEG	NEG	NEG	NEG
>"South-East Asian Clad																	
NMR07 (India)	NEG	NEG	NEG	NEG	NEG	NEG	NEG	POS	NEG	NEG	NEG	NEG	NEG	NEG	NEG	NEG	NEG
NMR08 (India)	NEG	NEG	NEG	NEG	NEG	NEG	NEG	POS	NEG	NEG	NEG	NEG	NEG	NEG	NEG	NEG	NEG
Bengalaru_ST20121978	AMB	NEG	NEG	NEG	NEG	NEG	NEG	POS	NEG	NEG	NEG	NEG	NEG	NEG	NEG	NEG	NEG
Hong Kong_89	NEG	NEG	NEG	NEG	NEG	NEG	NEG	POS	NEG	NEG	NEG	NEG	NEG	NEG	NEG	NEG	NEG
>"South-East Asian Clad																	
Perth_2005_AH_5	NEG	NEG	NEG	NEG	NEG	NEG	NEG	POS	NEG	NEG	NEG	NEG	NEG	NEG	NEG	NEG	NEG
>"South-East Asian Clad																	
Trinidad&Tobago_MRSA2010_103	NEG	NEG	NEG	NEG	NEG	NEG	NEG	NEG	NEG	NEG	NEG	NEG	NEG	NEG	NEG	NEG	NEG
Trinidad&Tobago_2013_PA25	NEG	NEG	NEG	NEG	NEG	NEG	NEG	NEG	NEG	NEG	NEG	NEG	NEG	NEG	NEG	NEG	NEG
Trinidad&Tobago_MRSA2010_110	NEG	NEG	NEG	NEG	NEG	NEG	NEG	NEG	NEG	NEG	NEG	NEG	NEG	NEG	NEG	NEG	NEG
Trinidad&Tobago_SSTI 2012_111	NEG	NEG	NEG	NEG	NEG	NEG	NEG	NEG	NEG	NEG	NEG	NEG	NEG	NEG	NEG	NEG	NEG
Trinidad&Tobago_MRSA2010_158	NEG	NEG	NEG	NEG	NEG	NEG	NEG	NEG	NEG	NEG	NEG	NEG	NEG	NEG	NEG	NEG	NEG
>"South-East Asian Clad																	
Kuwait_122	NEG	NEG	NEG	NEG	NEG	NEG	NEG	POS	NEG	NEG	NEG	NEG	NEG	NEG	NEG	NEG	NEG
Kuwait_002	NEG	NEG	NEG	NEG	NEG	NEG	NEG	POS	NEG	NEG	NEG	NEG	NEG	NEG	NEG	NEG	NEG
Kuwait_196	NEG	NEG	NEG	NEG	NEG	NEG	NEG	NEG	NEG	NEG	NEG	NEG	NEG	NEG	NEG	NEG	NEG
Kuwait_199	NEG	NEG	NEG	NEG	NEG	NEG	NEG	NEG	NEG	NEG	NEG	NEG	NEG	NEG	NEG	NEG	NEG
Hong Kong_136	NEG	NEG	NEG	NEG	NEG	NEG	NEG	NEG	NEG	NEG	NEG	NEG	NEG	NEG	NEG	NEG	NEG
>"South-East Asian Clad																	
P32 (Poland)	NEG	NEG	NEG	NEG	NEG	NEG	NEG	NEG	NEG	NEG	NEG	NEG	NEG	NEG	NEG	NEG	NEG
>"South-East Asian Clad																	
Perth_2015-S32313	NEG	NEG	NEG	NEG	NEG	NEG	NEG	NEG	NEG	NEG	NEG	NEG	NEG	NEG	NEG	NEG	NEG
>"hla-negative South-Ea																	
DEN907 (Denmark)	NEG	NEG	NEG	NEG	NEG	NEG	NEG	POS	NEG	NEG	NEG	NEG	NEG	NEG	NEG	NEG	NEG
>"hla-negative South-Ea																	
S26 (Thailand)	NEG	NEG	NEG	NEG	NEG	NEG	NEG	POS	NEG	NEG	NEG	NEG	NEG	NEG	NEG	NEG	NEG
S42 (Thailand)	NEG	NEG	NEG	NEG	NEG	NEG	NEG	POS	NEG	NEG	NEG	NEG	NEG	NEG	NEG	NEG	NEG
S97 (Thailand)	NEG	NEG	NEG	NEG	NEG	NEG	NEG	POS	NEG	NEG	NEG	NEG	NEG	NEG	NEG	NEG	NEG
S78 (Thailand)	NEG	NEG	NEG	NEG	NEG	NEG	NEG	POS	NEG	NEG	NEG	NEG	NEG	NEG	NEG	NEG	NEG
S25 (Thailand)	NEG	NEG	NEG	NEG	NEG	NEG	NEG	POS	NEG	NEG	NEG	NEG	NEG	NEG	NEG	NEG	NEG
S21 (Thailand)	NEG	NEG	NEG	NEG	NEG	NEG	NEG	POS	NEG	NEG	NEG	NEG	NEG	NEG	NEG	NEG	NEG
S106 (Thailand)	NEG	NEG	NEG	NEG	NEG	NEG	NEG	POS	NEG	NEG	NEG	NEG	NEG	NEG	NEG	NEG	NEG
S24 (Thailand)	NEG	NEG	NEG	NEG	NEG	NEG	NEG	POS	NEG	NEG	NEG	NEG	NEG	NEG	NEG	NEG	NEG
S39 (Thailand)	NEG	NEG	NEG	NEG	NEG	NEG	NEG	POS	NEG	NEG	NEG	NEG	NEG	NEG	NEG	NEG	NEG
S2 (Thailand)	NEG	NEG	NEG	NEG	NEG	NEG	NEG	POS	NEG	NEG	NEG	NEG	NEG	NEG	NEG	NEG	NEG
S81 (Thailand)	NEG	NEG	NEG	NEG	NEG	NEG	NEG	POS	NEG	NEG	NEG	NEG	NEG	NEG	NEG	NEG	NEG
M116 (Vietnam)	NEG	NEG	NEG	NEG	NEG	NEG	NEG	POS	NEG	NEG	NEG	NEG	NEG	NEG	NEG	NEG	NEG
>"Portuguese Clade": Pc																	
FFP103 (Portugal)	NEG	NEG	NEG	NEG	NEG	NEG	NEG	NEG	NEG	NEG	NEG	NEG	NEG	NEG	NEG	NEG	NEG
HDG2 (Portugal)	NEG	NEG	NEG	NEG	NEG	NEG	NEG	NEG	NEG	NEG	NEG	NEG	NEG	NEG	NEG	NEG	NEG
HSA10 (Portugal)	NEG	NEG	NEG	NEG	NEG	NEG	NEG	NEG	NEG	NEG	NEG	NEG	NEG	NEG	NEG	NEG	NEG
HSA11 (Portugal)	NEG	NEG	NEG	NEG	NEG	NEG	NEG	NEG	NEG	NEG	NEG	NEG	NEG	NEG	NEG	NEG	NEG
ICP5011 (Portugal)	NEG	NEG	NEG	NEG	NEG	NEG	NEG	NEG	NEG	NEG	NEG	NEG	NEG	NEG	NEG	NEG	NEG
ICP5014 (Portugal)	NEG	NEG	NEG	NEG	NEG	NEG	NEG	NEG	NEG	NEG	NEG	NEG	NEG	NEG	NEG	NEG	NEG
ICP5062 (Portugal)	NEG	NEG	NEG	NEG	NEG	NEG	NEG	NEG	NEG	NEG	NEG	NEG	NEG	NEG	NEG	NEG	NEG
Strain 1063_ATCC 33592 FDAARGOS_3 (USA)	NEG	NEG	NEG	NEG	NEG	NEG	NEG	POS	NEG	NEG	NEG	NEG	NEG	NEG	NEG	NEG	NEG
	NEG	NEG	NEG	NEG	NEG	NEG	NEG	POS	NEG	NEG	NEG	NEG	NEG	NEG	NEG	NEG	NEG
Russia-07_0110_Kurgan	NEG	NEG	NEG	NEG	NEG	NEG	NEG	NEG	NEG	NEG	NEG	NEG	NEG	NEG	NEG	NEG	NEG
Russia-11_0164_Kurgan	NEG	NEG	NEG	NEG	NEG	NEG	NEG	NEG	NEG	NEG	NEG	NEG	NEG	NEG	NEG	NEG	NEG
Russia-20_0367_Kurgan	NEG	NEG	NEG	NEG	NEG	NEG	NEG	NEG	NEG	NEG	NEG	NEG	NEG	NEG	NEG	NEG	NEG
Russia-21_0390_Chelyabinsk	NEG	NEG	NEG	NEG	NEG	NEG	NEG	NEG	NEG	NEG	NEG	NEG	NEG	NEG	NEG	NEG	NEG
>"South American/ Mid																	
AGT1 (Argentina)	NEG	NEG	NEG	NEG	NEG	NEG	NEG	NEG	NEG	NEG	NEG	NEG	NEG	NEG	NEG	NEG	NEG
AGT120 (Argentina)	NEG	NEG	NEG	NEG	NEG	NEG	NEG	NEG	NEG	NEG	NEG	NEG	NEG	NEG	NEG	NEG	NEG
AGT67 (Argentina)	NEG	NEG	NEG	NEG	NEG	NEG	NEG	NEG	NEG	NEG	NEG	NEG	NEG	NEG	NEG	NEG	NEG
AGT9 (Argentina)	NEG	NEG	NEG	NEG	NEG	NEG	NEG	NEG	NEG	NEG	NEG	NEG	NEG	NEG	NEG	NEG	NEG
RA3 (Argentina)	NEG	NEG	NEG	NEG	NEG	NEG	NEG	NEG	NEG	NEG	NEG	NEG	NEG	NEG	NEG	NEG	NEG
RA6 (Argentina)	NEG	NEG	NEG	NEG	NEG	NEG	NEG	NEG	NEG	NEG	NEG	NEG	NEG	NEG	NEG	NEG	NEG
RA7 (Argentina)	NEG	NEG	NEG	NEG	NEG	NEG	NEG	NEG	NEG	NEG	NEG	NEG	NEG	NEG	NEG	NEG	NEG
Be62 (Brazil)	NEG	NEG	NEG	NEG	NEG	NEG	NEG	NEG	NEG	NEG	NEG	NEG	NEG	NEG	NEG	NEG	NEG
Bmb9393 (Brazil)	NEG	NEG	NEG	NEG	NEG	NEG	NEG	NEG	NEG	NEG	NEG	NEG	NEG	NEG	NEG	NEG	NEG
BRA36 (Brazil)	NEG	NEG	NEG	NEG	NEG	NEG	NEG	NEG	NEG	NEG	NEG	NEG	NEG	NEG	NEG	NEG	NEG
BZ48 (Brazil)	NEG	NEG	NEG	NEG	NEG	NEG	NEG	NEG	NEG	NEG	NEG	NEG	NEG	NEG	NEG	NEG	NEG
Gv51 (Brazil)	NEG	NEG	NEG	NEG	NEG	NEG	NEG	NEG	NEG	NEG	NEG	NEG	NEG	NEG	NEG	NEG	NEG
Gv69 (Brazil)	NEG	NEG	NEG	NEG	NEG	NEG	NEG	NEG	NEG	NEG	NEG	NEG	NEG	NEG	NEG	NEG	NEG
Gv88 (Brazil)	NEG	NEG	NEG	NEG	NEG	NEG	NEG	NEG	NEG	NEG	NEG	NEG	NEG	NEG	NEG	NEG	NEG
HC1335 (Brazil)	NEG	NEG	NEG	NEG	NEG	NEG	NEG	NEG	NEG	NEG	NEG	NEG	NEG	NEG	NEG	NEG	NEG
HC1340 (Brazil)	NEG	NEG	NEG	NEG	NEG	NEG	NEG	NEG	NEG	NEG	NEG	NEG	NEG	NEG	NEG	NEG	NEG
HC556 (Brazil)	NEG	NEG	NEG	NEG	NEG	NEG	NEG	NEG	NEG	NEG	NEG	NEG	NEG	NEG	NEG	NEG	NEG
HU25 (Brazil)	NEG	NEG	NEG	NEG	NEG	NEG	NEG	NEG	NEG	NEG	NEG	NEG	NEG	NEG	NEG	NEG	NEG
UB563 (Brazil)	NEG	NEG	NEG	NEG	NEG	NEG	NEG	NEG	NEG	NEG	NEG	NEG	NEG	NEG	NEG	NEG	NEG
2A8 (Czech Republic)	NEG	NEG	NEG	NEG	NEG	NEG	NEG	NEG	NEG	NEG	NEG	NEG	NEG	NEG	NEG	NEG	NEG
M1229 (Denmark)	NEG	NEG	NEG	NEG	NEG	NEG	NEG	NEG	NEG	NEG	NEG	NEG	NEG	NEG	NEG	NEG	NEG
FRICAR (France)	NEG	NEG	NEG	NEG	NEG	NEG	NEG	NEG	NEG	NEG	NEG	NEG	NEG	NEG	NEG	NEG	NEG
D90 (Germany)	NEG	NEG	NEG	NEG	NEG	NEG	NEG	NEG	NEG	NEG	NEG	NEG	NEG	NEG	NEG	NEG	NEG
LIT68 (Lithuania)	NEG	NEG	NEG	NEG	NEG	NEG	NEG	POS	NEG	NEG	NEG	NEG	NEG	NEG	NEG	NEG	NEG
LIT76 (Lithuania)	NEG	NEG	NEG	NEG	NEG	NEG	NEG	POS	NEG	NEG	NEG	NEG	NEG	NEG	NEG	NEG	NEG
HGSA142 (Portugal)	NEG	NEG	NEG	NEG	NEG	NEG	NEG	NEG	NEG	NEG	NEG	NEG	NEG	NEG	NEG	NEG	NEG
HGSA9 (Portugal)	NEG	NEG	NEG	NEG	NEG	NEG	NEG	NEG	NEG	NEG	NEG	NEG	NEG	NEG	NEG	NEG	NEG
HSJ216 (Portugal)	NEG	NEG	NEG	NEG	NEG	NEG	NEG	NEG	NEG	NEG	NEG	NEG	NEG	NEG	NEG	NEG	NEG
M278 (Portugal)	NEG	NEG	NEG	NEG	NEG	NEG	NEG	NEG	NEG	NEG	NEG	NEG	NEG	NEG	NEG	NEG	NEG
ES26 (Spain)	NEG	NEG	NEG	NEG	NEG	NEG	NEG	NEG	NEG	NEG	NEG	NEG	NEG	NEG	NEG	NEG	NEG
UK102 (UK)	NEG	NEG	NEG	NEG	NEG	NEG	NEG	NEG	NEG	NEG	NEG	NEG	NEG	NEG	NEG	NEG	NEG
URU34 (Uruguay)	NEG	NEG	NEG	NEG	NEG	NEG	NEG	NEG	NEG	NEG	NEG	NEG	NEG	NEG	NEG	NEG	NEG
IS-125	NEG	NEG	NEG	NEG	NEG	NEG	NEG	POS	NEG	NEG	NEG	NEG	NEG	NEG	NEG	NEG	NEG
IS-157	NEG	NEG	NEG	NEG	NEG	NEG	NEG	POS	NEG	NEG	NEG	NEG	NEG	NEG	NEG	NEG	NEG
Ecuador_1	NEG	NEG	NEG	NEG	NEG	NEG	NEG	NEG	NEG	NEG	NEG	NEG	NEG	NEG	NEG	NEG	NEG
Ecuador_2	NEG	NEG	NEG	NEG	NEG	NEG	NEG	NEG	NEG	NEG	NEG	NEG	NEG	NEG	NEG	NEG	NEG
Dresden_15ANR574263	NEG	NEG	NEG	NEG	NEG	NEG	NEG	POS	NEG	NEG	NEG	NEG	NEG	NEG	NEG	NEG	NEG
UK-EMRSA-11	NEG	NEG	NEG	NEG	NEG	NEG	NEG	POS	NEG	NEG	NEG	NEG	NEG	NEG	NEG	NEG	NEG
>"South American/ Mid																	
BRA2 (Brazil)	NEG	NEG	NEG	NEG	NEG	NEG	NEG	NEG	NEG	NEG	NEG	NEG	NEG	NEG	NEG	NEG	NEG
CHL1 (Chile)	NEG	NEG	NEG	NEG	NEG	NEG	NEG	NEG	NEG	NEG	NEG	NEG	NEG	NEG	NEG	NEG	NEG
CHL151 (Chile)	NEG	NEG	NEG	NEG	NEG	NEG	NEG	NEG	NEG	NEG	NEG	NEG	NEG	NEG	NEG	NEG	NEG
UCL378 (Chile)	NEG	NEG	NEG	NEG	NEG	NEG	NEG	NEG	NEG	NEG	NEG	NEG	NEG	NEG	NEG	NEG	NEG
UP72 (Peru)	NEG	NEG	NEG	NEG	NEG	NEG	NEG	NEG	NEG	NEG	NEG	NEG	NEG	NEG	NEG	NEG	NEG
UP81 (Peru)	NEG	NEG	NEG	NEG	NEG	NEG	NEG	NEG	NEG	NEG	NEG	NEG	NEG	NEG	NEG	NEG	NEG
>"South American/ Mid																	
UP1073 (Peru)	NEG	NEG	NEG	NEG	NEG	NEG	NEG	NEG	NEG	NEG	NEG	NEG	NEG	NEG	NEG	NEG	NEG

STRAIN / ISOLATE	RESISTANCE : MISCELLANEOUS GENES				TOX.SCHOCK.TOXIN			VIRULENCE : ENTEROTOXINS									
	smr	vanA	vanB	vanZ	tst1			sea			seB	seC/L	seD	seD2	seE	seH	seJ
	smr (AL1)	vanA	vanB	vanZ	tst1 (consensus)	tst1 ("human" allele)	tst1 ("bovine" allele)	entA	entA (320E)	entA (N315) / entP	entB	entC	entD	entD2	entE	entH	entJ
		vancomycin resistance gene	vancomycin resistance gene from enterococci and Clostridium	teicoplanin resistance gene from enterococci	toxic shock syndrome toxin 1			Enterotoxin A	Enterotoxin A, allele from strain 320E	Enterotoxin A, allele from strain N315 = Entero-toxin P	Enterotoxin B	Enterotoxin C	Enterotoxin D	Enterotoxin similar to EntD	Enterotoxin E	Enterotoxin H	Enterotoxin J
>"South American/ Mid																	
LIT89 (Lithuania)	NEG	NEG	NEG	NEG	NEG	NEG	NEG	POS	NEG	NEG	NEG	NEG	NEG	NEG	NEG	NEG	NEG
MRSA_PR1 (Malaysia)	NEG	NEG	NEG	NEG	NEG	NEG	NEG	POS	NEG	NEG	NEG	NEG	NEG	NEG	NEG	NEG	NEG
Riyadh-2888905-R	NEG	NEG	NEG	NEG	NEG	NEG	NEG	NEG	NEG	NEG	NEG	NEG	NEG	NEG	NEG	NEG	NEG
Riyadh-2888915-BC	NEG	NEG	NEG	NEG	NEG	NEG	NEG	NEG	NEG	NEG	NEG	NEG	NEG	NEG	NEG	NEG	NEG
Riyadh-2793706-R	NEG	NEG	NEG	NEG	NEG	NEG	NEG	NEG	NEG	NEG	NEG	NEG	NEG	NEG	NEG	NEG	NEG
>"South American/ Mid																	
Riyadh_SSTI_18_3502925	NEG	NEG	NEG	NEG	NEG	NEG	NEG	NEG	NEG	NEG	NEG	NEG	NEG	NEG	NEG	NEG	NEG
Riyadh-2822825-W	NEG	NEG	NEG	NEG	AMB	NEG	NEG	NEG	NEG	NEG	NEG	NEG	NEG	NEG	NEG	NEG	NEG
>"South American/ Mid																	
UK-EMRSA-9	NEG	NEG	NEG	NEG	NEG	NEG	NEG	NEG	NEG	NEG	NEG	NEG	NEG	NEG	NEG	NEG	NEG
>"South American/ Mid																	
Lome_HT20020815	NEG	NEG	NEG	NEG	NEG	NEG	NEG	POS	NEG	NEG	NEG	NEG	NEG	NEG	NEG	NEG	NEG
>"South American/ Mid																	
NA32 (Denmark)	NEG	NEG	NEG	NEG	NEG	NEG	NEG	POS	NEG	NEG	NEG	NEG	NEG	NEG	NEG	NEG	NEG
H24 (Egypt)	NEG	NEG	NEG	NEG	NEG	NEG	NEG	POS	NEG	NEG	NEG	NEG	NEG	NEG	NEG	NEG	NEG
ATCC BAA-39 (=HUSA304) (Hungary)	NEG	NEG	NEG	NEG	NEG	NEG	NEG	POS	NEG	NEG	NEG	NEG	NEG	NEG	NEG	NEG	NEG
HUSA304 (Hungary)	NEG	NEG	NEG	NEG	NEG	NEG	NEG	POS	NEG	NEG	NEG	NEG	NEG	NEG	NEG	NEG	NEG
HU106 (Hungary)	NEG	NEG	NEG	NEG	NEG	NEG	NEG	POS	NEG	NEG	NEG	NEG	NEG	NEG	NEG	NEG	NEG
BSAC27 (UK)	NEG	NEG	NEG	NEG	NEG	NEG	NEG	NEG	NEG	NEG	NEG	NEG	NEG	NEG	NEG	NEG	NEG
NCTC13131, UK-EMRSA-4 (UK)	NEG	NEG	NEG	NEG	NEG	NEG	NEG	POS	NEG	NEG	NEG	NEG	NEG	NEG	NEG	NEG	NEG
	NEG	NEG	NEG	NEG	NEG	NEG	NEG	POS	NEG	NEG	NEG	NEG	NEG	NEG	NEG	NEG	NEG
Algiers_HT20040080	NEG	NEG	NEG	NEG	NEG	NEG	NEG	NEG	NEG	POS	NEG	NEG	NEG	NEG	NEG	NEG	NEG
Dublin-DSH_AR09_0.0066	NEG	NEG	NEG	NEG	NEG	NEG	NEG	POS	NEG	NEG	NEG	NEG	NEG	NEG	NEG	NEG	NEG
Dublin-DSH_AR09_0-0065	NEG	NEG	NEG	NEG	NEG	NEG	NEG	POS	NEG	NEG	NEG	NEG	NEG	NEG	NEG	NEG	NEG
Dublin-DSH_Phenotype-III_84	NEG	NEG	NEG	NEG	NEG	NEG	NEG	POS	NEG	NEG	NEG	NEG	NEG	NEG	NEG	NEG	NEG
Hong Kong_130	NEG	NEG	NEG	NEG	NEG	NEG	NEG	POS	NEG	NEG	NEG	NEG	NEG	NEG	NEG	NEG	NEG
Kuwait_018	NEG	NEG	NEG	NEG	NEG	NEG	NEG	NEG	NEG	NEG	NEG	NEG	NEG	NEG	NEG	NEG	NEG
Perth_08-17726	NEG	NEG	NEG	NEG	NEG	NEG	NEG	NEG	NEG	NEG	NEG	NEG	NEG	NEG	NEG	NEG	NEG
Riyadh_Alfaisal/KKKSUH_86_MRS-14-279	NEG	NEG	NEG	NEG	NEG	NEG	NEG	NEG	NEG	NEG	NEG	NEG	NEG	NEG	NEG	NEG	NEG
Riyadh_Alfaisal-04_Z3861_831588	NEG	NEG	NEG	NEG	NEG	NEG	NEG	NEG	NEG	NEG	NEG	NEG	NEG	NEG	NEG	NEG	NEG
Riyadh_Alfaisal-30_S15724_1108013	NEG	NEG	NEG	NEG	NEG	NEG	NEG	NEG	NEG	NEG	NEG	NEG	NEG	NEG	NEG	NEG	NEG
Riyadh_Alfaisal-6_22A_13_B3992_397721	NEG	NEG	NEG	NEG	NEG	NEG	NEG	NEG	NEG	NEG	NEG	NEG	NEG	NEG	NEG	NEG	NEG
Riyadh_SSTI_52_3615482	NEG	NEG	NEG	NEG	NEG	NEG	NEG	NEG	NEG	NEG	NEG	NEG	NEG	NEG	NEG	NEG	NEG
Riyadh-2817276-2	NEG	NEG	NEG	NEG	NEG	NEG	NEG	NEG	NEG	NEG	NEG	NEG	NEG	NEG	NEG	NEG	NEG
Riyadh-2891670-W	NEG	NEG	NEG	NEG	AMB	NEG	NEG	NEG	NEG	NEG	NEG	NEG	NEG	NEG	NEG	NEG	NEG
Riyadh-3006920-W	NEG	NEG	NEG	NEG	NEG	NEG	NEG	NEG	NEG	NEG	NEG	NEG	NEG	NEG	NEG	NEG	NEG
Riyadh-R2567782	AMB	NEG	NEG	NEG	NEG	NEG	NEG	NEG	NEG	NEG	NEG	NEG	NEG	NEG	NEG	NEG	NEG
Russia-18_0252_Krasnoyarsk_SK2	NEG	NEG	NEG	NEG	NEG	NEG	NEG	POS	NEG	NEG	NEG	NEG	NEG	NEG	NEG	NEG	NEG
UK-EMRSA-7	NEG	NEG	NEG	NEG	NEG	NEG	NEG	POS	NEG	NEG	NEG	NEG	NEG	NEG	NEG	NEG	NEG
>"South American/ Mid																	
Dublin-DSH_AR23_0073	NEG	NEG	NEG	NEG	NEG	NEG	NEG	POS	NEG	NEG	NEG	NEG	NEG	NEG	NEG	NEG	NEG
>Related to "South Ame																	
MRSA-OC3 (Russia)	NEG	NEG	NEG	NEG	POS	POS	NEG	NEG	NEG	NEG	NEG	NEG	NEG	NEG	NEG	NEG	NEG
Russia-12_0176_Krasnoyarsk	NEG	NEG	NEG	NEG	POS	POS	NEG	NEG	NEG	NEG	NEG	NEG	NEG	NEG	NEG	NEG	NEG
Russia-13_0180_Krasnoyarsk	NEG	NEG	NEG	NEG	POS	POS	NEG	NEG	NEG	NEG	NEG	NEG	NEG	NEG	NEG	NEG	NEG
Russia-16_0249_Krasnoyarsk	NEG	NEG	NEG	NEG	POS	POS	NEG	NEG	NEG	NEG	NEG	NEG	NEG	NEG	NEG	NEG	NEG
Russia-17_0250_Krasnoyarsk	NEG	NEG	NEG	NEG	POS	POS	NEG	NEG	NEG	NEG	NEG	NEG	NEG	NEG	NEG	NEG	NEG
>Related to "South Ame																	
URU110 (Uruguay)	NEG	NEG	NEG	NEG	NEG	NEG	NEG	POS	NEG	NEG	NEG	NEG	NEG	NEG	NEG	NEG	NEG
>Related to "South Ame																	
DS_014 (Thailand)	NEG	NEG	NEG	NEG	NEG	NEG	NEG	NEG	NEG	NEG	NEG	NEG	NEG	NEG	NEG	NEG	NEG
>Unassigned Middle Eas																	
Frankfurt_Oder_0490031797 (Libya)	NEG	NEG	NEG	NEG	NEG	NEG	NEG	POS	NEG	NEG	NEG	NEG	NEG	NEG	NEG	NEG	NEG
Kuwait_192	NEG	NEG	NEG	NEG	NEG	NEG	NEG	POS	AMB	NEG	NEG	NEG	NEG	NEG	NEG	NEG	NEG
Riyadh-9	NEG	NEG	NEG	NEG	NEG	NEG	NEG	POS	NEG	NEG	NEG	NEG	NEG	NEG	NEG	NEG	NEG
Russia-24_0407_Moscow	NEG	NEG	NEG	NEG	NEG	NEG	NEG	POS	NEG	NEG	NEG	NEG	NEG	NEG	NEG	NEG	NEG

STRAIN / ISOLATE	VIRULENCE : ENTEROTOXINS									VIRULENCE : HLG AND LEUKOCIDINS							
	seK	seN2	seQ	seR	seS	seT	seU2	seW	egc (total)	ORF CM14	lukF	lukS		hlgA	PVL	lukF-PV (P83) / lukM	lukD / lukE
	entK	entN2	entQ	entR	entS	entT	entU2	entW	seg, sei, selm, seln, selo , selu		lukF	lukS	lukS (ST22+ST45)	hlgA	lukF-PV, lukS-PV		
	Enterotoxin K	Putative Enterotoxin N2	Enterotoxin Q	Enterotoxin R	Enterotoxin S	Enterotoxin T	Enterotoxin U2	Putative Enterotoxin	egc cluster	Enterotoxin-like protein ORF CM14	Haemolysin gamma / leukocidin, component B	Haemolysin gamma / leukocidin, component C		Haemolysin gamma, component A	Panton Valentine leukocidin F component	F component from ruminant leukocidin	leukocidin E component
>"Eurasian Clade": TUR1			NEG														
TUR1 (Turkey)	NEG	NEG	NEG	NEG	NEG	NEG	NEG	NEG	NEG	NEG	POS	POS	AMB	POS	NEG	NEG	POS
TUR9 (Turkey)	NEG	NEG	NEG	NEG	NEG	NEG	NEG	NEG	NEG	NEG	POS	POS	AMB	POS	NEG	NEG	POS
>"Eurasian Clade" : T013			POS														
T0131 (China)	POS	NEG	POS	NEG	NEG	NEG	NEG	NEG	NEG	NEG	POS	POS	AMB	POS	NEG	NEG	POS
MU4 (Turkey)	POS	NEG	POS	NEG	NEG	NEG	NEG	NEG	NEG	NEG	POS	POS	AMB	POS	NEG	NEG	POS
DEU11 (Turkey)	POS	NEG	POS	NEG	NEG	NEG	NEG	NEG	NEG	NEG	POS	POS	AMB	POS	NEG	NEG	POS
DEU16 (Turkey)	POS	NEG	POS	NEG	NEG	NEG	NEG	NEG	NEG	NEG	POS	POS	AMB	POS	NEG	NEG	POS
Romania_Iasi_MRSA-07_54	POS	NEG	POS	NEG	NEG	NEG	NEG	NEG	NEG	NEG	POS	POS	POS	POS	NEG	NEG	POS
>"Eurasian Clade": arsC-			POS														
Romania_Iasi_MRSA-05_46	POS	NEG	POS	NEG	NEG	NEG	NEG	NEG	NEG	NEG	POS	POS	AMB	POS	NEG	NEG	POS
Romania_Iasi_MRSA-06_47	POS	NEG	POS	NEG	NEG	NEG	NEG	NEG	NEG	NEG	POS	POS	POS	POS	NEG	NEG	POS
>"Eurasian Clade": 16K/			COMM														
CN79 (China)	POS	NEG	POS	NEG	NEG	NEG	NEG	NEG	NEG	NEG	POS	POS	AMB	POS	NEG	NEG	POS
CUHK_BJ2002 (China)	POS	NEG	POS	NEG	NEG	NEG	NEG	NEG	NEG	NEG	POS	POS	AMB	POS	NEG	NEG	POS
CUHK_BJ2007 (China)	POS	NEG	POS	NEG	NEG	NEG	NEG	NEG	NEG	NEG	POS	POS	AMB	POS	NEG	NEG	POS
3HK (Czech Republic)	POS	NEG	POS	NEG	NEG	NEG	NEG	NEG	NEG	NEG	POS	POS	AMB	POS	NEG	NEG	POS
HU109 (Hungary)	POS	NEG	POS	NEG	NEG	NEG	NEG	NEG	NEG	NEG	POS	POS	AMB	POS	NEG	NEG	POS
HUR18 (Hungary)	POS	NEG	POS	NEG	NEG	NEG	NEG	NEG	NEG	NEG	POS	POS	AMB	POS	NEG	NEG	POS
H482 (Romania)	POS	NEG	POS	NEG	NEG	NEG	NEG	NEG	NEG	NEG	POS	POS	AMB	POS	NEG	NEG	POS
16K (Russia)	POS	NEG	POS	NEG	NEG	NEG	NEG	NEG	NEG	NEG	POS	POS	AMB	POS	NEG	NEG	POS
DEU10 (Turkey)	POS	NEG	POS	NEG	NEG	NEG	NEG	NEG	NEG	NEG	POS	POS	AMB	POS	NEG	NEG	POS
DEU12 (Turkey)	POS	NEG	POS	NEG	NEG	NEG	NEG	NEG	NEG	NEG	POS	POS	AMB	POS	NEG	NEG	POS
DEU14 (Turkey)	POS	NEG	POS	NEG	NEG	NEG	NEG	NEG	NEG	NEG	POS	POS	AMB	POS	NEG	NEG	POS
DEU15 (Turkey)	POS	NEG	POS	NEG	NEG	NEG	NEG	NEG	NEG	NEG	POS	POS	AMB	POS	NEG	NEG	POS
DEU17 (Turkey)	POS	NEG	POS	NEG	NEG	NEG	NEG	NEG	NEG	NEG	POS	POS	AMB	POS	NEG	NEG	POS
DEU19 (Turkey)	POS	NEG	POS	NEG	NEG	NEG	NEG	NEG	NEG	NEG	POS	POS	AMB	POS	NEG	NEG	POS
DEU2 (Turkey)	POS	NEG	POS	NEG	NEG	NEG	NEG	NEG	NEG	NEG	POS	POS	AMB	POS	NEG	NEG	POS
DEU20 (Turkey)	POS	NEG	POS	NEG	NEG	NEG	NEG	NEG	NEG	NEG	POS	POS	AMB	POS	NEG	NEG	POS
DEU23 (Turkey)	POS	NEG	POS	NEG	NEG	NEG	NEG	NEG	NEG	NEG	POS	POS	AMB	POS	NEG	NEG	POS
DEU3 (Turkey)	POS	NEG	POS	NEG	NEG	NEG	NEG	NEG	NEG	NEG	POS	POS	AMB	POS	NEG	NEG	POS
DEU5 (Turkey)	POS	NEG	POS	NEG	NEG	NEG	NEG	NEG	NEG	NEG	POS	POS	AMB	POS	NEG	NEG	POS
DEU6 (Turkey)	POS	NEG	POS	NEG	NEG	NEG	NEG	NEG	NEG	NEG	POS	POS	AMB	POS	NEG	NEG	POS
DEU8 (Turkey)	POS	NEG	POS	NEG	NEG	NEG	NEG	NEG	NEG	NEG	POS	POS	AMB	POS	NEG	NEG	POS
HU11 (Turkey)	POS	NEG	POS	NEG	NEG	NEG	NEG	NEG	NEG	NEG	POS	POS	AMB	POS	NEG	NEG	POS
HU13 (Turkey)	POS	NEG	POS	NEG	NEG	NEG	NEG	NEG	NEG	NEG	POS	POS	AMB	POS	NEG	NEG	POS
HU14 (Turkey)	POS	NEG	POS	NEG	NEG	NEG	NEG	NEG	NEG	NEG	POS	POS	AMB	POS	NEG	NEG	POS
HU15 (Turkey)	POS	NEG	POS	NEG	NEG	NEG	NEG	NEG	NEG	NEG	POS	POS	AMB	POS	NEG	NEG	POS
HU16 (Turkey)	POS	NEG	POS	NEG	NEG	NEG	NEG	NEG	NEG	NEG	POS	POS	AMB	POS	NEG	NEG	POS
HU17 (Turkey)	POS	NEG	POS	NEG	NEG	NEG	NEG	NEG	NEG	NEG	POS	POS	AMB	POS	NEG	NEG	POS
HU21 (Turkey)	POS	NEG	POS	NEG	NEG	NEG	NEG	NEG	NEG	NEG	POS	POS	AMB	POS	NEG	NEG	POS
HU23 (Turkey)	POS	NEG	POS	NEG	NEG	NEG	NEG	NEG	NEG	NEG	POS	POS	AMB	POS	NEG	NEG	POS
HU26 (Turkey)	POS	NEG	POS	NEG	NEG	NEG	NEG	NEG	NEG	NEG	POS	POS	AMB	POS	NEG	NEG	POS
HU4 (Turkey)	POS	NEG	POS	NEG	NEG	NEG	NEG	NEG	NEG	NEG	POS	POS	AMB	POS	NEG	NEG	POS
HU5 (Turkey)	POS	NEG	POS	NEG	NEG	NEG	NEG	NEG	NEG	NEG	POS	POS	AMB	POS	NEG	NEG	POS
HU6 (Turkey)	POS	NEG	POS	NEG	NEG	NEG	NEG	NEG	NEG	NEG	POS	POS	AMB	POS	NEG	NEG	POS
HU7 (Turkey)	NEG	NEG	NEG	NEG	NEG	NEG	NEG	NEG	NEG	NEG	POS	POS	AMB	POS	NEG	NEG	POS
HU8 (Turkey)	POS	NEG	POS	NEG	NEG	NEG	NEG	NEG	NEG	NEG	POS	POS	AMB	POS	NEG	NEG	POS
HU9 (Turkey)	POS	NEG	POS	NEG	NEG	NEG	NEG	NEG	NEG	NEG	POS	POS	AMB	POS	NEG	NEG	POS
IU1 (Turkey)	POS	NEG	POS	NEG	NEG	NEG	NEG	NEG	NEG	NEG	POS	POS	AMB	POS	NEG	NEG	POS
IU10 (Turkey)	POS	NEG	POS	NEG	NEG	NEG	NEG	NEG	NEG	NEG	POS	POS	AMB	POS	NEG	NEG	POS
IU11 (Turkey)	POS	NEG	POS	NEG	NEG	NEG	NEG	NEG	NEG	NEG	POS	POS	AMB	POS	NEG	NEG	POS
IU12 (Turkey)	POS	NEG	POS	NEG	NEG	NEG	NEG	NEG	NEG	NEG	POS	POS	AMB	POS	NEG	NEG	POS
IU13 (Turkey)	POS	NEG	POS	NEG	NEG	NEG	NEG	NEG	NEG	NEG	POS	POS	AMB	POS	NEG	NEG	POS
IU15 (Turkey)	POS	NEG	POS	NEG	NEG	NEG	NEG	NEG	NEG	NEG	POS	POS	AMB	POS	NEG	NEG	POS
IU18 (Turkey)	POS	NEG	POS	NEG	NEG	NEG	NEG	NEG	NEG	NEG	POS	POS	AMB	POS	NEG	NEG	POS
IU19 (Turkey)	POS	NEG	POS	NEG	NEG	NEG	NEG	NEG	NEG	NEG	POS	POS	AMB	POS	NEG	NEG	POS
IU2 (Turkey)	POS	NEG	POS	NEG	NEG	NEG	NEG	NEG	NEG	NEG	POS	POS	AMB	POS	NEG	NEG	POS
IU4 (Turkey)	POS	NEG	POS	NEG	NEG	NEG	NEG	NEG	NEG	NEG	POS	POS	AMB	POS	NEG	NEG	POS
IU6 (Turkey)	POS	NEG	POS	NEG	NEG	NEG	NEG	NEG	NEG	NEG	POS	POS	AMB	POS	NEG	NEG	POS
IU7 (Turkey)	POS	NEG	POS	NEG	NEG	NEG	NEG	NEG	NEG	NEG	POS	POS	AMB	POS	NEG	NEG	POS
IU9 (Turkey)	POS	NEG	POS	NEG	NEG	NEG	NEG	NEG	NEG	NEG	POS	POS	AMB	POS	NEG	NEG	POS
MU1 (Turkey)	POS	NEG	POS	NEG	NEG	NEG	NEG	NEG	NEG	NEG	POS	POS	AMB	POS	NEG	NEG	POS
MU10 (Turkey)	POS	NEG	POS	NEG	NEG	NEG	NEG	NEG	NEG	NEG	POS	POS	AMB	POS	NEG	NEG	POS
MU20 (Turkey)	POS	NEG	POS	NEG	NEG	NEG	NEG	NEG	NEG	NEG	POS	POS	AMB	POS	NEG	NEG	POS
Mu3 (Turkey)	POS	NEG	POS	NEG	NEG	NEG	NEG	NEG	NEG	NEG	POS	POS	AMB	POS	NEG	NEG	POS
MU5 (Turkey)	POS	NEG	POS	NEG	NEG	NEG	NEG	NEG	NEG	NEG	POS	POS	AMB	POS	NEG	NEG	POS
MU6 (Turkey)	POS	NEG	POS	NEG	NEG	NEG	NEG	NEG	NEG	NEG	POS	POS	AMB	POS	NEG	NEG	POS
MU7 (Turkey)	POS	NEG	POS	NEG	NEG	NEG	NEG	NEG	NEG	NEG	POS	POS	AMB	POS	NEG	NEG	POS
TUR27 (Turkey)	POS	NEG	POS	NEG	NEG	NEG	NEG	NEG	NEG	NEG	POS	POS	AMB	POS	NEG	NEG	POS
NCTR 32S	POS	NEG	POS	NEG	NEG	NEG	NEG	NEG	NEG	NEG	POS	POS	AMB	POS	NEG	NEG	POS
SA02_A4 = Russia-06_0085_Moscow	POS	NEG	POS	NEG	NEG	NEG	NEG	NEG	NEG	NEG	POS	POS	AMB	POS	NEG	NEG	POS
	POS	NEG	POS	NEG	NEG	NEG	NEG	NEG	NEG	NEG	POS	POS	POS	POS	NEG	NEG	POS
Dresden_08V35987 (Turkey)	POS	NEG	POS	NEG	NEG	NEG	NEG	NEG		NEG	POS	POS	AMB	POS	NEG	NEG	POS
Dresden_17ANR577152	POS	NEG	POS	NEG	NEG	NEG	NEG	NEG	NEG	NEG	POS	POS	POS	POS	NEG	NEG	POS
Dresden_17ANR580374 (Makedonia)	POS	NEG	POS	NEG	NEG	NEG	NEG	NEG	NEG	NEG	POS	POS	POS	POS	NEG	NEG	POS
Hong Kong_69-II	POS	NEG	POS	NEG	NEG	NEG	NEG	NEG	NEG	NEG	POS	POS	POS	POS	NEG	NEG	POS
Hong Kong_93	POS	NEG	POS	NEG	NEG	NEG	NEG	NEG	NEG	NEG	POS	POS	POS	POS	NEG	NEG	POS
Rawalpindi_Kidney Center_03_SK1	POS	NEG	POS	NEG	NEG	NEG	NEG	NEG	NEG	NEG	POS	POS	POS	POS	NEG	NEG	POS
Rawalpindi_Kidney Center_08	POS	NEG	POS	NEG	NEG	NEG	NEG	NEG	NEG	NEG	POS	POS	POS	POS	NEG	NEG	POS
Rawalpindi_Kidney Center_10	POS	NEG	POS	NEG	NEG	NEG	NEG	NEG	NEG	NEG	POS	POS	POS	POS	NEG	NEG	POS
Rawalpindi_Kidney Center_19	POS	NEG	POS	NEG	NEG	NEG	NEG	NEG	NEG	NEG	POS	POS	POS	POS	NEG	NEG	POS
Rawalpindi_Kidney Center_50_SK1	POS	NEG	POS	NEG	NEG	NEG	NEG	NEG	NEG	NEG	POS	POS	POS	POS	NEG	NEG	POS
Romania_Iasi_BC-49_430	POS	NEG	POS	NEG	NEG	NEG	NEG	NEG	NEG	NEG	POS	POS	POS	POS	NEG	NEG	POS
Romania_Iasi_MRSA_284	POS	NEG	POS	NEG	NEG	NEG	NEG	NEG	NEG	NEG	POS	POS	AMB	POS	NEG	NEG	POS
Romania_Iasi_MRSA-32_318	POS	NEG	POS	NEG	NEG	NEG	NEG	NEG	NEG	NEG	POS	POS	POS	POS	NEG	NEG	POS
Romania_Iasi_MRSA-54_430	POS	NEG	POS	NEG	NEG	NEG	NEG	NEG	NEG	NEG	POS	POS	POS	POS	NEG	NEG	POS
Romania_Iasi_SSTI-01_2	POS	NEG	POS	NEG	NEG	NEG	NEG	NEG	NEG	NEG	POS	POS	POS	POS	NEG	NEG	POS
Romania_Iasi_SSTI-10_101	POS	NEG	POS	NEG	NEG	NEG	NEG	NEG	NEG	NEG	POS	POS	AMB	POS	NEG	NEG	POS
Romania_Iasi_SSTI-11_106	POS	NEG	POS	NEG	NEG	NEG	NEG	NEG	NEG	NEG	POS	POS	POS	POS	NEG	NEG	POS
Russia-01_0001_SaintPetersburg	NEG	NEG	NEG	NEG	NEG	NEG	NEG	NEG	NEG	NEG	POS	POS	POS	POS	NEG	NEG	POS
Russia-02_0004_SaintPetersburg	NEG	NEG	NEG	NEG	NEG	NEG	NEG	NEG	NEG	NEG	POS	POS	POS	POS	NEG	NEG	POS
Russia-03_0057_SaintPetersburg	NEG	NEG	NEG	NEG	NEG	NEG	NEG	NEG	NEG	NEG	POS	POS	NEG	POS	NEG	NEG	POS
Russia-04_0076_Moscow	POS	NEG	POS	NEG	NEG	NEG	NEG	NEG	NEG	NEG	POS	POS	POS	POS	NEG	NEG	POS
Russia-05_0078_Moscow	POS	NEG	POS	NEG	NEG	NEG	NEG	NEG	NEG	NEG	POS	POS	POS	POS	NEG	NEG	POS
Russia-08_0135_Moscow	POS	NEG	POS	NEG	NEG	NEG	NEG	NEG	NEG	NEG	POS	POS	POS	POS	NEG	NEG	POS
Russia-09_0150_Kurgan	NEG	NEG	NEG	NEG	NEG	NEG	NEG	NEG	NEG	NEG	POS	POS	POS	POS	NEG	NEG	POS
Russia-10_0162_Kurgan	NEG	NEG	NEG	NEG	NEG	NEG	NEG	NEG	NEG	NEG	POS	POS	POS	POS	NEG	NEG	POS
Russia-14_0184_Moscow	POS	NEG	POS	NEG	NEG	NEG	NEG	NEG	NEG	NEG	POS	POS	POS	POS	NEG	NEG	POS
Russia-15_0232_SaintPetersburg	NEG	NEG	NEG	NEG	NEG	NEG	NEG	NEG	NEG	NEG	POS	POS	POS	POS	NEG	NEG	POS
Russia-22_0391_Chelyabinsk	NEG	NEG	NEG	NEG	NEG	NEG	NEG	NEG	NEG	NEG	POS	POS	POS	POS	NEG	NEG	POS
Russia-23_0392_Chelyabinsk	NEG	NEG	NEG	NEG	NEG	NEG	NEG	NEG	NEG	NEG	POS	POS	POS	POS	NEG	NEG	POS
>"Eurasian Clade" : IU17			POS														
IU17 (Turkey)	POS	NEG	POS	NEG	NEG	NEG	NEG	NEG	NEG	NEG	POS	POS	AMB	POS	NEG	NEG	POS
IU20 (Turkey)	POS	NEG	POS	NEG	NEG	NEG	NEG	NEG	NEG	NEG	POS	POS	AMB	POS	NEG	NEG	POS

STRAIN / ISOLATE	VIRULENCE : ENTEROTOXINS									VIRULENCE : HLG AND LEUKOCIDINS							
	seK	seN2	seQ	seR	seS	seT	seU2	seW	egc (total)	ORF CM14	lukF	lukS		hlgA	PVL	lukF-PV (P83) / lukM	lukD / lukE
	entK	entN2	entQ	entR	entS	entT	entU2	entW	seg, sei, selm, seln, selo , selu		lukF	lukS	lukS (ST22+ST45)	hlgA	lukF-PV, lukS-PV		
	Enterotoxin K	Putative Enterotoxin N2	Enterotoxin Q	Enterotoxin R	Enterotoxin S	Enterotoxin T	Enterotoxin U2	Putative Enterotoxin	egc cluster	Enterotoxin-like protein ORF CM14	Haemolysin gamma / leukocidin, component B	Haemolysin gamma / leukocidin, component C		Haemolysin gamma, component A	Panton Valentine leukocidin F component	F component from ruminant leukocidin	leukocidin E component
>"Eurasian Clade": Spor			POS														
Dresden_16ANR581769	POS	NEG	POS	NEG	NEG	NEG	NEG	NEG	NEG	NEG	POS	POS	NEG	POS	NEG	NEG	POS
Dresden_17ANR578769	POS	NEG	POS	NEG	NEG	NEG	NEG	NEG	NEG	NEG	POS	POS	POS	POS	NEG	NEG	POS
>"Eurasian Clade": Kuw			POS														
Kuwait_306	POS	NEG	POS	NEG	NEG	NEG	NEG	NEG	NEG	NEG	POS	POS	POS	POS	NEG	NEG	POS
Kuwait_301	POS	NEG	POS	NEG	NEG	NEG	NEG	NEG	NEG	NEG	POS	POS	POS	POS	NEG	NEG	POS
>"European Clade": "Gr			RARE														
GRE18 (Greece)	NEG	NEG	NEG	NEG	NEG	NEG	NEG	NEG	NEG	NEG	POS	POS	AMB	POS	NEG	NEG	POS
GRE317 (Greece)	NEG	NEG	NEG	NEG	NEG	NEG	NEG	NEG	NEG	NEG	POS	POS	AMB	POS	NEG	NEG	POS
GRE4 (Greece)	NEG	NEG	NEG	NEG	NEG	NEG	NEG	NEG	NEG	NEG	POS	POS	AMB	POS	NEG	NEG	NEG
R35 (USA)	NEG	NEG	NEG	NEG	NEG	NEG	NEG	NEG	NEG	NEG	POS	POS	AMB	POS	NEG	NEG	POS
Casablanca_HT20060548	POS	NEG	POS	NEG	NEG	NEG	NEG	NEG	NEG	NEG	POS	POS	POS	POS	NEG	NEG	POS
Casablanca_HT20060550	POS	NEG	POS	NEG	NEG	NEG	NEG	NEG	NEG	NEG	POS	POS	POS	NEG	NEG	NEG	POS
Dresden_01V34572 (Greece)	NEG	NEG	NEG	NEG	NEG	NEG	NEG	NEG	NEG	NEG	POS	POS	NEG	POS	NEG	NEG	POS
Dresden_01V36469	NEG	NEG	NEG	NEG	NEG	NEG	NEG	NEG	NEG	NEG	POS	POS	AMB	POS	NEG	NEG	POS
Dresden_01V39123	NEG	NEG	NEG	NEG	NEG	NEG	NEG	NEG	NEG	NEG	POS	POS	POS	POS	NEG	NEG	POS
Dresden_01V39143	NEG	NEG	NEG	NEG	NEG	NEG	NEG	NEG	NEG	NEG	POS	POS	AMB	POS	NEG	NEG	POS
Greece 1_3680_Harmony	NEG	NEG	NEG	NEG	NEG	NEG	NEG	NEG	NEG	NEG	POS	POS	NEG	POS	NEG	NEG	AMB
>"European Clade": UK-			POS														
ANS46 (Australia)	POS	NEG	POS	NEG	NEG	NEG	NEG	NEG	NEG	NEG	POS	POS	AMB	POS	NEG	NEG	POS
LHH1 (USA)	POS	NEG	POS	NEG	NEG	NEG	NEG	NEG	NEG	NEG	POS	POS	AMB	POS	NEG	NEG	POS
Dublin-DSH_AR01_0-1085	POS	NEG	POS	NEG	NEG	NEG	NEG	NEG	NEG	NEG	POS	POS	AMB	POS	NEG	NEG	POS
Dublin-DSH_AR01_0-1118	POS	NEG	POS	NEG	NEG	NEG	NEG	NEG	NEG	NEG	POS	POS	AMB	POS	NEG	NEG	POS
Dublin-DSH_AR15_0098	POS	NEG	POS	NEG	NEG	NEG	NEG	NEG	NEG	NEG	POS	POS	AMB	POS	NEG	NEG	POS
Dublin-DSH_AR15_0104	POS	NEG	POS	NEG	NEG	NEG	NEG	NEG	NEG	NEG	POS	POS	POS	POS	NEG	NEG	POS
UK_NCTC11939_UK-1_Harmony	POS	NEG	POS	NEG	NEG	NEG	NEG	NEG	NEG	NEG	POS	POS	POS	POS	NEG	NEG	POS
>"European Clade": BK2			POS														
BK2421 (USA)	POS	NEG	POS	NEG	NEG	NEG	NEG	NEG	NEG	NEG	POS	POS	AMB	POS	NEG	NEG	POS
>"Aussi/NZ Clade", JKD6			VAR														
JKD6009 (Australia)	POS	NEG	POS	NEG	NEG	NEG	NEG	NEG	NEG	NEG	POS	POS	AMB	POS	NEG	NEG	NEG
JKD6008 (New Zealand)	POS	NEG	POS	NEG	NEG	NEG	NEG	NEG	NEG	NEG	POS	POS	AMB	POS	NEG	NEG	NEG
Perth_2015-S20209	POS	NEG	AMB	NEG	NEG	NEG	NEG	NEG	NEG	NEG	POS	POS	AMB	POS	NEG	NEG	POS
Perth_2015-S20305	NEG	NEG	NEG	NEG	NEG	NEG	NEG	NEG	NEG	NEG	POS	POS	AMB	POS	NEG	NEG	POS
Perth_2015-S20603	NEG	NEG	NEG	NEG	NEG	NEG	NEG	NEG	NEG	NEG	POS	POS	AMB	POS	NEG	NEG	POS
Perth_2015-S20607	NEG	NEG	NEG	NEG	NEG	NEG	NEG	NEG	NEG	NEG	POS	POS	AMB	POS	NEG	NEG	POS
Perth_2015-S20608	NEG	NEG	NEG	NEG	NEG	NEG	NEG	NEG	NEG	NEG	POS	POS	AMB	POS	NEG	NEG	POS
Perth_2015-S20610	NEG	NEG	NEG	NEG	NEG	NEG	NEG	NEG	NEG	NEG	POS	POS	AMB	POS	NEG	NEG	POS
Perth_2015-S20613	NEG	NEG	NEG	NEG	NEG	NEG	NEG	NEG	NEG	NEG	POS	POS	AMB	POS	NEG	NEG	POS
Perth_2015-S20617	NEG	NEG	NEG	NEG	NEG	NEG	NEG	NEG	NEG	NEG	POS	POS	AMB	POS	NEG	NEG	NEG
Perth_2015-S20622	NEG	NEG	NEG	NEG	NEG	NEG	NEG	NEG	NEG	NEG	POS	POS	AMB	POS	NEG	NEG	POS
Perth_2015-S23619	NEG	NEG	NEG	NEG	NEG	NEG	NEG	NEG	NEG	NEG	POS	POS	AMB	POS	NEG	NEG	NEG
Perth_2015-S41210	NEG	NEG	NEG	NEG	NEG	NEG	NEG	NEG	NEG	NEG	POS	POS	AMB	POS	NEG	NEG	POS
Perth_2015-S51001	POS	NEG	POS	NEG	NEG	NEG	NEG	NEG	NEG	NEG	POS	POS	AMB	POS	NEG	NEG	POS
Perth_2015-S51003	POS	NEG	POS	NEG	NEG	NEG	NEG	NEG	NEG	NEG	POS	POS	AMB	POS	NEG	NEG	POS
Perth_2015-S51006	POS	NEG	POS	NEG	NEG	NEG	NEG	NEG	NEG	NEG	POS	POS	AMB	POS	NEG	NEG	POS
Perth_2015-S51007	POS	NEG	POS	NEG	NEG	NEG	NEG	NEG	NEG	NEG	POS	POS	AMB	POS	NEG	NEG	POS
Perth_2015-S51008	POS	NEG	POS	NEG	NEG	NEG	NEG	NEG	NEG	NEG	POS	POS	AMB	POS	NEG	NEG	POS
Perth_2015-S51014	POS	NEG	POS	NEG	NEG	NEG	NEG	NEG	NEG	NEG	POS	POS	AMB	POS	NEG	NEG	POS
Perth_2015-S51408	POS	NEG	POS	NEG	NEG	NEG	NEG	NEG	NEG	NEG	POS	POS	AMB	POS	NEG	NEG	POS
Perth_2016-S20614	NEG	NEG	NEG	NEG	NEG	NEG	NEG	NEG	NEG	NEG	POS	POS	AMB	POS	NEG	NEG	POS
Perth_2016-S23614	NEG	NEG	NEG	NEG	NEG	NEG	NEG	NEG	NEG	NEG	POS	POS	AMB	POS	NEG	NEG	NEG
Perth_2016-S42009	NEG	NEG	NEG	NEG	NEG	NEG	NEG	NEG	NEG	NEG	POS	POS	AMB	POS	NEG	NEG	POS
Perth_2016-S51001	POS	NEG	POS	NEG	NEG	NEG	NEG	NEG	NEG	NEG	POS	POS	AMB	POS	NEG	NEG	POS
Perth_2016-S51403	POS	NEG	POS	NEG	NEG	NEG	NEG	NEG	NEG	NEG	POS	POS	AMB	POS	NEG	NEG	POS
Perth_07 RPA 20	NEG	NEG	NEG	NEG	NEG	NEG	NEG	NEG	NEG	NEG	POS	POS	AMB	POS	NEG	NEG	NEG
Perth_08-19088	NEG	NEG	NEG	NEG	NEG	NEG	NEG	NEG	NEG	NEG	POS	POS	AMB	POS	NEG	NEG	POS
Perth_01-15351	POS	NEG	POS	NEG	NEG	NEG	NEG	NEG	NEG	NEG	POS	POS	AMB	POS	NEG	NEG	POS
Perth_01-15357	POS	NEG	POS	NEG	NEG	NEG	NEG	NEG	NEG	NEG	POS	POS	NEG	AMB	NEG	NEG	POS
Perth_01-15419	POS	NEG	POS	NEG	NEG	NEG	NEG	NEG	NEG	NEG	POS	POS	NEG	NEG	NEG	NEG	POS
Perth_01-16309	POS	NEG	POS	NEG	NEG	NEG	NEG	NEG	NEG	NEG	POS	POS	AMB	POS	NEG	NEG	POS
Perth_03 SNP 95	POS	NEG	POS	NEG	NEG	NEG	NEG	NEG	NEG	NEG	POS	AMB	POS	POS	NEG	NEG	POS
Perth_2003_AH_47	POS	NEG	POS	NEG	NEG	NEG	NEG	NEG	NEG	NEG	POS	POS	AMB	POS	NEG	NEG	POS
>"Australian/NZ Clade":			NEG														
Perth_2015-S20623	NEG	NEG	NEG	NEG	NEG	NEG	NEG	NEG	NEG	NEG	POS	POS	AMB	POS	NEG	NEG	POS
Perth_2015-S20826	NEG	NEG	NEG	NEG	NEG	NEG	NEG	NEG	NEG	NEG	POS	POS	AMB	POS	NEG	NEG	POS
Perth_05 NH 56	NEG	NEG	NEG	NEG	NEG	NEG	NEG	NEG	NEG	NEG	POS	POS	AMB	POS	NEG	NEG	POS
>CC5-MRSA-III LA MRSA			NEG														
Delbrück 38 isolate from domestic turkey	NEG	NEG	NEG	NEG	NEG	NEG	NEG	NEG	POS	NEG	POS	POS	AMB	POS	NEG	NEG	POS
> Staph. pseudintermed			NEG														
KM1381	NEG	NEG	NEG	NEG	NEG	NEG	NEG	NEG	NEG	NEG	NEG	NEG	NEG	NEG	NEG	NEG	NEG
>"South-East Asian Clad			VAR														
M92 (Canada)	POS	NEG	POS	NEG	NEG	NEG	NEG	NEG	NEG	NEG	POS	POS	AMB	POS	NEG	NEG	POS
CHI59 (China)	POS	NEG	POS	NEG	NEG	NEG	NEG	NEG	NEG	NEG	POS	POS	AMB	POS	NEG	NEG	POS
CHI61 (China)	POS	NEG	POS	NEG	NEG	NEG	NEG	NEG	NEG	NEG	POS	POS	AMB	POS	NEG	NEG	POS
CUHK_HK1997 (China)	NEG	NEG	NEG	NEG	NEG	NEG	NEG	NEG	NEG	NEG	POS	POS	AMB	POS	NEG	NEG	POS
M996 (China)	POS	NEG	POS	NEG	NEG	NEG	NEG	NEG	NEG	NEG	POS	POS	AMB	POS	NEG	NEG	POS
H211 (Denmark)	POS	NEG	POS	NEG	NEG	NEG	NEG	NEG	NEG	NEG	POS	POS	AMB	POS	NEG	NEG	POS
H216 (Denmark)	POS	NEG	POS	NEG	NEG	NEG	NEG	NEG	NEG	NEG	POS	POS	AMB	POS	NEG	NEG	POS
D71 (Germany)	POS	NEG	POS	NEG	NEG	NEG	NEG	NEG	NEG	NEG	POS	POS	AMB	POS	NEG	NEG	POS
NMR05 (India)	NEG	NEG	NEG	NEG	NEG	NEG	NEG	NEG	NEG	NEG	POS	POS	AMB	POS	NEG	NEG	POS
MAL11 (Malaysia)	NEG	NEG	NEG	NEG	NEG	NEG	NEG	NEG	NEG	NEG	POS	POS	AMB	POS	NEG	NEG	POS
MAL9 (Malaysia)	POS	NEG	POS	NEG	NEG	NEG	NEG	NEG	NEG	NEG	POS	POS	AMB	POS	NEG	NEG	POS
Na21 (Sri Lanka)	NEG	NEG	NEG	NEG	NEG	NEG	NEG	NEG	NEG	NEG	POS	POS	AMB	POS	NEG	NEG	POS
M592 (Syria)	POS	NEG	POS	NEG	NEG	NEG	NEG	NEG	NEG	NEG	POS	POS	AMB	POS	NEG	NEG	POS
H202 (Thailand)	POS	NEG	POS	NEG	NEG	NEG	NEG	NEG	NEG	NEG	POS	POS	AMB	POS	NEG	NEG	POS
S102 (Thailand)	POS	NEG	POS	NEG	NEG	NEG	NEG	NEG	NEG	NEG	POS	POS	AMB	POS	NEG	NEG	POS
S130 (Thailand)	POS	NEG	POS	NEG	NEG	NEG	NEG	NEG	NEG	NEG	POS	POS	AMB	POS	NEG	NEG	POS
S40 (Thailand)	POS	NEG	POS	NEG	NEG	NEG	NEG	NEG	NEG	NEG	POS	POS	AMB	POS	NEG	NEG	POS
S71 (Thailand)	POS	NEG	POS	NEG	NEG	NEG	NEG	NEG	NEG	NEG	POS	POS	AMB	POS	NEG	NEG	POS
S87 (Thailand)	POS	NEG	POS	NEG	NEG	NEG	NEG	NEG	NEG	NEG	POS	POS	AMB	POS	NEG	NEG	POS
S93 (Thailand)	POS	NEG	POS	NEG	NEG	NEG	NEG	NEG	NEG	NEG	POS	POS	AMB	POS	NEG	NEG	POS
US_002 (Thailand)	POS	NEG	POS	NEG	NEG	NEG	NEG	NEG	NEG	NEG	POS	POS	AMB	POS	NEG	NEG	POS
US_017 (Thailand)	POS	NEG	POS	NEG	NEG	NEG	NEG	NEG	NEG	NEG	POS	POS	AMB	POS	NEG	NEG	POS
WAMC6102 (Thailand)	POS	NEG	POS	NEG	NEG	NEG	NEG	NEG	NEG	NEG	POS	POS	AMB	POS	NEG	NEG	POS
NCTC_13626_TW20 (UK)	POS	NEG	POS	NEG	NEG	NEG	NEG	NEG	NEG	NEG	POS	POS	AMB	POS	NEG	NEG	POS
BSAC3021 (UK)	POS	NEG	POS	NEG	NEG	NEG	NEG	NEG	NEG	NEG	POS	POS	AMB	POS	NEG	NEG	POS
BSAC3046 (UK)	POS	NEG	POS	NEG	NEG	NEG	NEG	NEG	NEG	NEG	POS	POS	AMB	POS	NEG	NEG	POS
1193_SAUR (USA)	POS	NEG	POS	NEG	NEG	NEG	NEG	NEG	NEG	NEG	POS	POS	AMB	POS	NEG	NEG	POS
1194_SAUR (USA)	POS	NEG	POS	NEG	NEG	NEG	NEG	NEG	NEG	NEG	POS	POS	AMB	POS	NEG	NEG	POS

STRAIN / ISOLATE	VIRULENCE : ENTEROTOXINS									VIRULENCE : HLG AND LEUKOCIDINS							
	seK	seN2	seQ	seR	seS	seT	seU2	seW	egc (total)	ORF CM14	lukF	lukS		hlgA	PVL	lukF-PV (P83) / lukM	lukD / lukE
	entK	entN2	entQ	entR	entS	entT	entU2	entW	seg, sei, selm, seln, selo , selu		lukF	lukS	lukS (ST22+ST45)	hlgA	lukF-PV, lukS-PV		
	Enterotoxin K	Putative Enterotoxin N2	Enterotoxin Q	Enterotoxin R	Enterotoxin S	Enterotoxin T	Enterotoxin U2	Putative Enterotoxin	egc cluster	Enterotoxin-like protein ORF CM14	Haemolysin gamma / leukocidin, component B	Haemolysin gamma / leukocidin, component C		Haemolysin gamma, component A	Panton Valentine leukocidin F component	F component from ruminant leukocidin	leukocidin E component
>"South-East Asian Clade": TW20 (Irish AR44), c																	
1195_SAUR (USA)	POS	NEG	POS	NEG	NEG	NEG	NEG	NEG	NEG	NEG	POS	POS	AMB	POS	NEG	NEG	POS
FVRH6002 (USA)	POS	NEG	POS	NEG	NEG	NEG	NEG	NEG	NEG	NEG	POS	POS	AMB	POS	NEG	NEG	POS
KINW6048 (USA)	POS	NEG	POS	NEG	NEG	NEG	NEG	NEG	NEG	NEG	POS	POS	AMB	POS	NEG	NEG	POS
LAMC0011 (USA)	POS	NEG	POS	NEG	NEG	NEG	NEG	NEG	NEG	NEG	POS	POS	AMB	POS	NEG	NEG	POS
SJOS6053 (USA)	POS	NEG	POS	NEG	NEG	NEG	NEG	NEG	NEG	NEG	POS	POS	AMB	POS	NEG	NEG	POS
SJOS6072 (USA)	POS	NEG	POS	NEG	NEG	NEG	NEG	NEG	NEG	NEG	POS	POS	AMB	POS	NEG	NEG	POS
UCIM6042 (USA)	POS	NEG	POS	NEG	NEG	NEG	NEG	NEG	NEG	NEG	POS	POS	AMB	POS	NEG	NEG	POS
Perth_2015-S31902	AMB	NEG	NEG	NEG	NEG	NEG	NEG	NEG	NEG	NEG	POS	POS	AMB	POS	NEG	NEG	POS
Perth_2015-S31904	NEG	NEG	NEG	NEG	NEG	NEG	NEG	NEG	NEG	NEG	POS	POS	AMB	POS	NEG	NEG	POS
Perth_2015-S31913	NEG	NEG	NEG	NEG	NEG	NEG	NEG	NEG	NEG	NEG	POS	POS	AMB	POS	NEG	NEG	POS
Perth_2015-S31918	NEG	NEG	NEG	NEG	NEG	NEG	NEG	NEG	NEG	NEG	POS	POS	AMB	POS	NEG	NEG	POS
Dublin-DSH_AR44_E1183	NEG	NEG	NEG	NEG	NEG	NEG	NEG	NEG	NEG	NEG	POS	POS	AMB	POS	NEG	NEG	POS
Dublin-DSH_Unfamiliar-3_E1520	POS	NEG	POS	NEG	NEG	NEG	NEG	NEG	NEG	NEG	POS	POS	NEG	POS	NEG	NEG	POS
Hong Kong_042	POS	NEG	POS	NEG	NEG	NEG	NEG	NEG	NEG	NEG	POS	POS	AMB	POS	NEG	NEG	POS
Hong Kong_101	POS	NEG	POS	NEG	NEG	NEG	NEG	NEG	NEG	NEG	POS	POS	POS	POS	NEG	NEG	POS
Hong Kong_111	NEG	NEG	NEG	NEG	NEG	NEG	NEG	NEG	NEG	NEG	POS	POS	POS	POS	NEG	NEG	POS
Hong Kong_113	POS	NEG	POS	NEG	NEG	NEG	NEG	NEG	NEG	NEG	POS	POS	NEG	POS	NEG	NEG	POS
Hong Kong_115	NEG	NEG	NEG	NEG	NEG	NEG	NEG	NEG	NEG	NEG	POS	POS	POS	POS	NEG	NEG	POS
Hong Kong_118	NEG	NEG	NEG	NEG	NEG	NEG	NEG	NEG	NEG	NEG	POS	POS	POS	POS	NEG	NEG	POS
Hong Kong_125	POS	NEG	POS	NEG	NEG	NEG	NEG	NEG	NEG	NEG	POS	POS	POS	POS	NEG	NEG	POS
Hong Kong_129	POS	NEG	POS	NEG	NEG	NEG	NEG	NEG	NEG	NEG	POS	POS	POS	POS	NEG	NEG	POS
Hong Kong_133	NEG	NEG	NEG	NEG	NEG	NEG	NEG	NEG	NEG	NEG	POS	POS	POS	POS	NEG	NEG	POS
Kuwait_009	POS	NEG	POS	NEG	NEG	NEG	NEG	NEG	NEG	NEG	POS	POS	POS	POS	NEG	NEG	POS
Kuwait_103	POS	NEG	POS	NEG	NEG	NEG	NEG	NEG	NEG	NEG	POS	POS	POS	POS	NEG	NEG	POS
Kuwait_107	POS	NEG	POS	NEG	NEG	NEG	NEG	NEG	NEG	NEG	POS	POS	POS	POS	NEG	NEG	POS
Perth_03 AH 88	NEG	NEG	NEG	NEG	NEG	NEG	NEG	NEG	NEG	NEG	POS	POS	AMB	POS	NEG	NEG	POS
Perth_08-16905	POS	NEG	POS	NEG	NEG	NEG	NEG	NEG	NEG	NEG	POS	POS	POS	POS	NEG	NEG	POS
Riyadh_Alfaisal-10_SICU_13_B5672_1117353	POS	NEG	POS	NEG	NEG	NEG	NEG	NEG	NEG	NEG	POS	POS	POS	POS	NEG	NEG	POS
Riyadh_SSTI_17_3508602	NEG	NEG	NEG	NEG	NEG	NEG	NEG	NEG	NEG	NEG	POS	POS	POS	POS	NEG	NEG	POS
Riyadh-2822088-R	POS	NEG	POS	NEG	NEG	NEG	NEG	NEG	NEG	NEG	POS	POS	POS	POS	NEG	NEG	POS
Trinidad&Tobago_2012_195 (619/12)	POS	NEG	POS	NEG	NEG	NEG	NEG	NEG	NEG	NEG	POS	POS	POS	POS	NEG	NEG	POS
Trinidad&Tobago_2012_370 (1112-52177)	POS	NEG	POS	NEG	NEG	NEG	NEG	NEG	NEG	NEG	POS	POS	POS	POS	NEG	NEG	POS
Trinidad&Tobago_2012_378 (4139)	POS	NEG	POS	NEG	NEG	NEG	NEG	NEG	NEG	NEG	POS	POS	POS	POS	NEG	NEG	POS
Trinidad&Tobago_2013_PA21	POS	NEG	POS	NEG	NEG	NEG	NEG	NEG	NEG	NEG	POS	POS	AMB	POS	NEG	NEG	POS
Trinidad&Tobago_2013_PA22	POS	NEG	POS	NEG	NEG	NEG	NEG	NEG	NEG	NEG	POS	POS	POS	POS	NEG	NEG	POS
Trinidad&Tobago_2912_340 (8523/12)	POS	NEG	POS	NEG	NEG	NEG	NEG	NEG	NEG	NEG	POS	POS	POS	POS	NEG	NEG	POS
Trinidad&Tobago_MRSA2010_105	POS	NEG	POS	NEG	NEG	NEG	NEG	NEG	NEG	NEG	POS	POS	POS	POS	NEG	NEG	POS
Trinidad&Tobago_MRSA2010_108	POS	NEG	POS	NEG	NEG	NEG	NEG	NEG	NEG	NEG	POS	POS	POS	POS	NEG	NEG	POS
Trinidad&Tobago_MRSA2010_33	POS	NEG	POS	NEG	NEG	NEG	NEG	NEG	NEG	NEG	POS	POS	AMB	POS	NEG	NEG	POS
Trinidad&Tobago_MRSA2010_79	POS	NEG	POS	NEG	NEG	NEG	NEG	NEG	NEG	NEG	POS	POS	NEG	POS	NEG	NEG	POS
Trinidad&Tobago_MRSA2010_88	POS	NEG	POS	NEG	NEG	NEG	NEG	NEG	NEG	NEG	POS	POS	POS	POS	NEG	NEG	POS
Trinidad&Tobago_MRSA2010_97	AMB	NEG	POS	NEG	NEG	NEG	NEG	NEG	NEG	NEG	POS	POS	POS	POS	NEG	NEG	POS
Trinidad&Tobago_SSTI 2012_031	POS	NEG	POS	NEG	NEG	NEG	NEG	NEG	NEG	NEG	POS	POS	POS	POS	NEG	NEG	POS
Uganda-29_17148_250811	POS	NEG	POS	NEG	NEG	NEG	NEG	NEG	NEG	NEG	POS	POS	AMB	POS	NEG	NEG	POS
>"South-East Asian Clad			POS														
UCIM6015	POS	NEG	POS	NEG	NEG	NEG	NEG	NEG	NEG	NEG	POS	POS	AMB	POS	NEG	NEG	POS
Hong Kong_121	POS	NEG	POS	NEG	NEG	NEG	NEG	NEG	NEG	NEG	POS	POS	AMB	POS	NEG	NEG	POS
>"South-East Asian Clad			POS														
Z172 (Taiwan)	POS	NEG	POS	NEG	NEG	NEG	NEG	NEG	NEG	NEG	POS	POS	AMB	POS	NEG	NEG	POS
Russia-19_0342_Kurgan	POS	NEG	POS	NEG	NEG	NEG	NEG	NEG	NEG	NEG	POS	POS	POS	POS	NEG	NEG	POS
>"South-East Asian Clad			POS														
Perth_01-15392	POS	NEG	POS	NEG	NEG	NEG	NEG	NEG	NEG	NEG	POS	POS	POS	POS	NEG	NEG	POS
>"South-East Asian Clad			VAR														
XN108 (China)	POS	NEG	POS	NEG	NEG	NEG	NEG	NEG	NEG	NEG	POS	POS	AMB	POS	NEG	NEG	POS
NMR09 (India)	NEG	NEG	NEG	NEG	NEG	NEG	NEG	NEG	NEG	NEG	POS	POS	AMB	POS	NEG	NEG	POS
346 (Malaysia)	NEG	NEG	NEG	NEG	NEG	NEG	NEG	NEG	NEG	NEG	POS	POS	NEG	POS	NEG	NEG	POS
DS_009 (Thailand)	POS	NEG	POS	NEG	NEG	NEG	NEG	NEG	NEG	NEG	POS	POS	AMB	POS	NEG	NEG	POS
18ANR581005 (Ind)	NEG	NEG	NEG	NEG	NEG	NEG	NEG	NEG	NEG	NEG	POS	POS	AMB	POS	NEG	NEG	POS
Bengalaru_ST20141405	NEG	NEG	NEG	NEG	NEG	NEG	NEG	NEG	NEG	NEG	POS	POS	POS	POS	NEG	NEG	POS
Bengalaru_ST20141406	NEG	NEG	NEG	NEG	NEG	NEG	NEG	NEG	NEG	NEG	POS	POS	POS	POS	NEG	NEG	POS
Bengalaru_ST20141417	NEG	NEG	NEG	NEG	NEG	NEG	NEG	NEG	NEG	NEG	POS	POS	POS	POS	NEG	NEG	POS
Hong Kong_107	NEG	NEG	NEG	NEG	NEG	NEG	NEG	NEG	NEG	NEG	POS	POS	AMB	POS	NEG	NEG	POS
Hong Kong_12	POS	NEG	POS	NEG	NEG	NEG	NEG	NEG	NEG	NEG	POS	POS	POS	POS	NEG	NEG	POS
Hong Kong_132	POS	NEG	POS	NEG	NEG	NEG	NEG	NEG	NEG	NEG	POS	POS	POS	POS	NEG	NEG	POS
Riyadh-3028763-R	POS	NEG	POS	NEG	NEG	NEG	NEG	NEG	NEG	NEG	POS	POS	POS	POS	NEG	NEG	POS
Trinidad&Tobago_MRSA2010_94	POS	NEG	POS	NEG	NEG	NEG	NEG	NEG	NEG	NEG	POS	POS	POS	POS	NEG	NEG	POS
>"South-East Asian Clad			POS														
M705 (Thailand)	POS	NEG	POS	NEG	NEG	NEG	NEG	NEG	NEG	NEG	POS	POS	AMB	POS	NEG	NEG	POS
S85 (Thailand)	POS	NEG	POS	NEG	NEG	NEG	NEG	NEG	NEG	NEG	POS	POS	AMB	POS	NEG	NEG	POS
Riyadh_Alfaisal-04_281014_S5076	POS	NEG	POS	NEG	NEG	NEG	NEG	NEG	NEG	NEG	POS	POS	POS	POS	NEG	NEG	POS
Riyadh_Alfaisal-26_S15145_1104163	POS	NEG	POS	NEG	NEG	NEG	NEG	NEG	NEG	NEG	POS	POS	POS	POS	NEG	NEG	POS
Riyadh-2817437-W	POS	NEG	POS	NEG	NEG	NEG	NEG	NEG	NEG	NEG	POS	POS	POS	POS	NEG	NEG	POS
>"South-East Asian Clad			VAR														
PPUKM-775-2009 (Malaysia)	NEG	NEG	NEG	NEG	NEG	NEG	NEG	NEG	NEG	NEG	POS	POS	AMB	POS	NEG	NEG	POS
Hong Kong_138	POS	NEG	POS	NEG	NEG	NEG	NEG	NEG	NEG	NEG	POS	POS	POS	POS	NEG	NEG	POS
>"South-East Asian Clad			POS														
Kuwait_001	POS	NEG	POS	NEG	NEG	NEG	NEG	NEG	NEG	NEG	POS	POS	POS	POS	NEG	NEG	POS
Kuwait_337	POS	NEG	POS	NEG	NEG	NEG	NEG	NEG	NEG	NEG	POS	POS	POS	POS	NEG	NEG	POS
>"South-East Asian Clad			COMM														
CUHK_HK2007 (China)	POS	NEG	POS	NEG	NEG	NEG	NEG	NEG	NEG	NEG	POS	POS	AMB	POS	NEG	NEG	POS
NMR02 (India)	POS	NEG	POS	NEG	NEG	NEG	NEG	NEG	NEG	NEG	POS	POS	AMB	POS	NEG	NEG	POS
VB1490 (India)	POS	NEG	POS	NEG	NEG	NEG	NEG	NEG	NEG	NEG	POS	POS	AMB	POS	NEG	NEG	POS
V521 (Korea)	POS	NEG	POS	NEG	NEG	NEG	NEG	NEG	NEG	NEG	POS	POS	AMB	POS	NEG	NEG	POS
HST-077 (Lebanon)	POS	NEG	POS	NEG	NEG	NEG	NEG	NEG	NEG	NEG	POS	POS	AMB	POS	NEG	NEG	POS
MAL1 (Malaysia)	POS	NEG	POS	NEG	NEG	NEG	NEG	NEG	NEG	NEG	POS	POS	AMB	POS	NEG	NEG	POS
MAL3 (Malaysia)	POS	NEG	POS	NEG	NEG	NEG	NEG	NEG	NEG	NEG	POS	POS	AMB	POS	NEG	NEG	POS
PPUKM-261-2009 (Malaysia)	NEG	NEG	NEG	NEG	NEG	NEG	NEG	NEG	NEG	NEG	POS	POS	AMB	POS	NEG	NEG	POS
PPUKM-332-2009 (Malaysia)	POS	NEG	POS	NEG	NEG	NEG	NEG	NEG	NEG	NEG	POS	POS	AMB	POS	NEG	NEG	POS
S38 (Thailand)	POS	NEG	POS	NEG	NEG	NEG	NEG	NEG	NEG	NEG	POS	POS	AMB	POS	NEG	NEG	POS
S7 (Thailand)	POS	NEG	POS	NEG	NEG	NEG	NEG	NEG	NEG	NEG	POS	POS	AMB	POS	NEG	NEG	POS
US_008 (Thailand)	POS	NEG	POS	NEG	NEG	NEG	NEG	NEG	NEG	NEG	POS	POS	AMB	POS	NEG	NEG	POS
US_030 (Thailand)	POS	NEG	POS	NEG	NEG	NEG	NEG	NEG	NEG	NEG	POS	POS	AMB	POS	NEG	NEG	POS
M170 (Tsunami-related)	POS	NEG	POS	NEG	NEG	NEG	NEG	NEG	NEG	NEG	POS	POS	AMB	POS	NEG	NEG	POS
BSAC697 (UK)	POS	NEG	POS	NEG	NEG	NEG	NEG	NEG	NEG	NEG	POS	POS	AMB	POS	NEG	NEG	POS
IS-189	POS	NEG	POS	NEG	NEG	NEG	NEG	NEG	NEG	NEG	POS	POS	AMB	POS	NEG	NEG	POS
MRGR3	POS	NEG	POS	NEG	NEG	NEG	NEG	NEG	NEG	NEG	POS	POS	AMB	POS	NEG	NEG	POS
smg07	POS	NEG	POS	NEG	NEG	NEG	NEG	NEG	NEG	NEG	POS	POS	AMB	POS	NEG	NEG	POS
AH1-AUS-EMRSA-3	POS	NEG	POS	NEG	NEG	NEG	NEG	NEG	NEG	NEG	POS	POS	AMB	POS	NEG	NEG	POS
Bengalaru_ST20141403	NEG	NEG	NEG	NEG	NEG	NEG	NEG	NEG	NEG	NEG	POS	POS	POS	POS	NEG	NEG	POS
Finland E24_98541_Harmony	POS	NEG	POS	NEG	NEG	NEG	NEG	NEG	NEG	NEG	POS	POS	POS	POS	NEG	NEG	POS
Hong Kong_10	POS	NEG	POS	NEG	NEG	NEG	NEG	NEG	NEG	NEG	POS	POS	POS	POS	NEG	NEG	POS
Hong Kong_100	POS	NEG	POS	NEG	NEG	NEG	NEG	NEG	NEG	NEG	POS	POS	POS	POS	NEG	NEG	POS
Hong Kong_199	POS	NEG	POS	NEG	NEG	NEG	NEG	NEG	NEG	NEG	POS	POS	POS	POS	NEG	NEG	POS
Hong Kong_34	POS	NEG	POS	NEG	NEG	NEG	NEG	NEG	NEG	NEG	POS	POS	POS	POS	NEG	NEG	POS
Hong Kong_88	POS	NEG	POS	NEG	NEG	NEG	NEG	NEG	NEG	NEG	POS	POS	POS	POS	NEG	NEG	POS
Hong Kong_92	POS	NEG	POS	NEG	NEG	NEG	NEG	NEG	NEG	NEG	POS	POS	POS	POS	NEG	NEG	POS
Hong Kong_94	POS	NEG	POS	NEG	NEG	NEG	NEG	NEG	NEG	NEG	POS	POS	POS	POS	NEG	NEG	POS
Kuwait_257	POS	NEG	POS	NEG	NEG	NEG	NEG	NEG	NEG	NEG	POS	POS	POS	POS	NEG	NEG	POS
Perth_01-15330	POS	NEG	POS	NEG	NEG	NEG	NEG	NEG	NEG	NEG	POS	POS	NEG	POS	NEG	NEG	POS
Perth_01-15349	POS	NEG	POS	NEG	NEG	NEG	NEG	NEG	NEG	NEG	POS	POS	NEG	AMB	NEG	NEG	POS
Perth_01-16176	POS	NEG	POS	NEG	NEG	NEG	NEG	NEG	NEG	NEG	POS	POS	AMB	AMB	NEG	NEG	POS
Perth_WBG8379	POS	NEG	POS	NEG	NEG	NEG	NEG	NEG	NEG	NEG	POS	POS	POS	POS	NEG	NEG	POS
Rawalpindi_Kidney Center_56	NEG	NEG	NEG	NEG	NEG	NEG	NEG	NEG	NEG	NEG	POS	POS	POS	POS	NEG	NEG	POS
Riyadh_SSTI_05_3464614	POS	NEG	POS	NEG	NEG	NEG	NEG	NEG	NEG	NEG	POS	POS	AMB	POS	NEG	NEG	POS
Trinidad&Tobago_2012_157	POS	NEG	POS	NEG	NEG	NEG	NEG	NEG	NEG	NEG	POS	POS	POS	POS	NEG	NEG	POS

STRAIN / ISOLATE	VIRULENCE : ENTEROTOXINS									VIRULENCE : HLG AND LEUKOCIDINS							
	seK	seN2	seQ	seR	seS	seT	seU2	seW	egc (total)	ORF CM14	lukF	lukS		hlgA	PVL	lukF-PV (P83) / lukM	lukD / lukE
	entK	entN2	entQ	entR	entS	entT	entU2	entW	seg, sei, selm, seln, selo , selu		lukF	lukS	lukS (ST22+ST45)	hlgA	lukF-PV, lukS-PV		
	Enterotoxin K	Putative Enterotoxin N2	Enterotoxin Q	Enterotoxin R	Enterotoxin S	Enterotoxin T	Enterotoxin U2	Putative Enterotoxin	egc cluster	Enterotoxin-like protein ORF CM14	Haemolysin gamma / leukocidin, component B	Haemolysin gamma / leukocidin, component C		Haemolysin gamma, component A	Panton Valentine leukocidin F component	F component from ruminant leukocidin	leukocidin E component
>"South-East Asian Clad			POS														
M418	POS	NEG	POS	NEG	NEG	NEG	NEG	NEG	NEG	NEG	POS	POS	AMB	POS	NEG	NEG	POS
>"South-East Asian Clad			VAR														
NMR07 (India)	NEG	NEG	NEG	NEG	NEG	NEG	NEG	NEG	NEG	NEG	POS	POS	AMB	POS	NEG	NEG	POS
NMR08 (India)	NEG	NEG	NEG	NEG	NEG	NEG	NEG	NEG	NEG	NEG	POS	POS	AMB	POS	NEG	NEG	POS
Bengalaru_ST20121978	NEG	NEG	NEG	NEG	NEG	NEG	NEG	NEG	NEG	NEG	POS	POS	POS	POS	NEG	NEG	POS
Hong Kong_89	POS	NEG	POS	NEG	NEG	NEG	NEG	NEG	NEG	NEG	POS	POS	POS	POS	NEG	AMB	POS
>"South-East Asian Clad			POS														
Perth_2005_AH_5	POS	NEG	POS	NEG	NEG	NEG	NEG	NEG	NEG	NEG	POS	POS	NEG	POS	NEG	NEG	POS
>"South-East Asian Clad			POS														
Trinidad&Tobago_MRSA2010_103	POS	NEG	POS	NEG	NEG	NEG	NEG	NEG	NEG	NEG	POS	POS	POS	POS	NEG	NEG	POS
Trinidad&Tobago_2013_PA25	POS	NEG	POS	NEG	NEG	NEG	NEG	NEG	NEG	NEG	POS	POS	POS	POS	NEG	NEG	POS
Trinidad&Tobago_MRSA2010_110	POS	NEG	POS	NEG	NEG	NEG	NEG	NEG	NEG	NEG	POS	POS	POS	POS	NEG	NEG	POS
Trinidad&Tobago_SSTI 2012_111	POS	NEG	POS	NEG	NEG	NEG	NEG	NEG	NEG	NEG	POS	POS	POS	POS	NEG	NEG	POS
Trinidad&Tobago_MRSA2010_158	POS	NEG	POS	NEG	NEG	NEG	NEG	NEG	NEG	NEG	POS	POS	AMB	POS	NEG	NEG	POS
>"South-East Asian Clad			POS														
Kuwait_122	POS	NEG	POS	NEG	NEG	NEG	NEG	NEG	NEG	NEG	POS	POS	AMB	POS	NEG	NEG	POS
Kuwait_002	POS	NEG	POS	NEG	NEG	NEG	NEG	NEG	NEG	NEG	POS	POS	POS	POS	NEG	NEG	POS
Kuwait_196	POS	NEG	POS	NEG	NEG	NEG	NEG	NEG	NEG	NEG	POS	POS	POS	POS	NEG	NEG	POS
Kuwait_199	POS	NEG	POS	NEG	NEG	NEG	NEG	NEG	NEG	NEG	POS	POS	POS	POS	NEG	NEG	POS
Hong Kong_136	POS	NEG	POS	NEG	NEG	NEG	NEG	NEG	NEG	NEG	POS	POS	POS	POS	NEG	NEG	POS
>"South-East Asian Clad			POS														
P32 (Poland)	POS	NEG	POS	NEG	NEG	NEG	NEG	NEG	NEG	NEG	POS	POS	AMB	POS	NEG	NEG	POS
>"South-East Asian Clad			POS														
Perth_2015-S32313	POS	NEG	POS	NEG	NEG	NEG	NEG	NEG	NEG	NEG	POS	POS	AMB	POS	NEG	NEG	POS
>"hla-negative South-Ea			POS														
DEN907 (Denmark)	POS	NEG	POS	NEG	NEG	NEG	NEG	NEG	NEG	NEG	POS	POS	AMB	POS	NEG	NEG	POS
>"hla-negative South-Ea			POS														
S26 (Thailand)	POS	NEG	POS	NEG	NEG	NEG	NEG	NEG	NEG	NEG	POS	POS	AMB	POS	NEG	NEG	POS
S42 (Thailand)	POS	NEG	POS	NEG	NEG	NEG	NEG	NEG	NEG	NEG	POS	POS	AMB	POS	NEG	NEG	POS
S97 (Thailand)	POS	NEG	POS	NEG	NEG	NEG	NEG	NEG	NEG	NEG	POS	POS	AMB	POS	NEG	NEG	POS
S78 (Thailand)	POS	NEG	POS	NEG	NEG	NEG	NEG	NEG	NEG	NEG	POS	POS	AMB	POS	NEG	NEG	POS
S25 (Thailand)	POS	NEG	POS	NEG	NEG	NEG	NEG	NEG	NEG	NEG	POS	POS	AMB	POS	NEG	NEG	POS
S21 (Thailand)	POS	NEG	POS	NEG	NEG	NEG	NEG	NEG	NEG	NEG	POS	POS	AMB	POS	NEG	NEG	POS
S106 (Thailand)	POS	NEG	POS	NEG	NEG	NEG	NEG	NEG	NEG	NEG	POS	POS	AMB	POS	NEG	NEG	POS
S24 (Thailand)	POS	NEG	POS	NEG	NEG	NEG	NEG	NEG	NEG	NEG	POS	POS	AMB	POS	NEG	NEG	POS
S39 (Thailand)	POS	NEG	POS	NEG	NEG	NEG	NEG	NEG	NEG	NEG	POS	POS	AMB	POS	NEG	NEG	POS
S2 (Thailand)	POS	NEG	POS	NEG	NEG	NEG	NEG	NEG	NEG	NEG	POS	POS	AMB	POS	NEG	NEG	POS
S81 (Thailand)	POS	NEG	POS	NEG	NEG	NEG	NEG	NEG	NEG	NEG	POS	POS	AMB	POS	NEG	NEG	POS
M116 (Vietnam)	POS	NEG	POS	NEG	NEG	NEG	NEG	NEG	NEG	NEG	POS	POS	AMB	POS	NEG	NEG	POS
>"Portuguese Clade": Pc			VAR														
FFP103 (Portugal)	NEG	NEG	NEG	NEG	NEG	NEG	NEG	NEG	NEG	NEG	POS	POS	AMB	POS	NEG	NEG	POS
HDG2 (Portugal)	NEG	NEG	NEG	NEG	NEG	NEG	NEG	NEG	NEG	NEG	POS	POS	AMB	POS	NEG	NEG	POS
HSA10 (Portugal)	NEG	NEG	NEG	NEG	NEG	NEG	NEG	NEG	NEG	NEG	POS	POS	AMB	POS	NEG	NEG	POS
HSA11 (Portugal)	POS	NEG	POS	NEG	NEG	NEG	NEG	NEG	NEG	NEG	POS	POS	AMB	POS	NEG	NEG	POS
ICP5011 (Portugal)	NEG	NEG	NEG	NEG	NEG	NEG	NEG	NEG	NEG	NEG	POS	POS	AMB	POS	NEG	NEG	POS
ICP5014 (Portugal)	NEG	NEG	NEG	NEG	NEG	NEG	NEG	NEG	NEG	NEG	POS	POS	AMB	POS	NEG	NEG	POS
ICP5062 (Portugal)	NEG	NEG	NEG	NEG	NEG	NEG	NEG	NEG	NEG	NEG	POS	POS	AMB	POS	NEG	NEG	POS
Strain 1063_ATCC 33592 FDAARGOS_3 (USA)	POS	NEG	POS	NEG	NEG	NEG	NEG	NEG	NEG	NEG	POS	POS	AMB	POS	NEG	NEG	POS
	POS	NEG	POS	NEG	NEG	NEG	NEG	NEG	NEG	NEG	POS	POS	POS	POS	NEG	NEG	POS
Russia-07_0110_Kurgan	POS	NEG	POS	NEG	NEG	NEG	NEG	NEG	NEG	NEG	POS	POS	POS	POS	NEG	NEG	POS
Russia-11_0164_Kurgan	POS	NEG	POS	NEG	NEG	NEG	NEG	NEG	NEG	NEG	POS	POS	POS	POS	NEG	NEG	POS
Russia-20_0367_Kurgan	POS	NEG	POS	NEG	NEG	NEG	NEG	NEG	NEG	NEG	POS	POS	POS	POS	NEG	NEG	POS
Russia-21_0390_Chelyabinsk	POS	NEG	POS	NEG	NEG	NEG	NEG	NEG	NEG	NEG	POS	POS	POS	POS	NEG	NEG	POS
>"South American/ Mid			RARE														
AGT1 (Argentina)	NEG	NEG	NEG	NEG	NEG	NEG	NEG	NEG	NEG	NEG	POS	POS	AMB	POS	NEG	NEG	POS
AGT120 (Argentina)	NEG	NEG	NEG	NEG	NEG	NEG	NEG	NEG	NEG	NEG	POS	POS	AMB	POS	NEG	NEG	POS
AGT67 (Argentina)	NEG	NEG	NEG	NEG	NEG	NEG	NEG	NEG	NEG	NEG	POS	POS	AMB	POS	NEG	NEG	POS
AGT9 (Argentina)	NEG	NEG	NEG	NEG	NEG	NEG	NEG	NEG	NEG	NEG	POS	POS	AMB	POS	NEG	NEG	POS
RA3 (Argentina)	NEG	NEG	NEG	NEG	NEG	NEG	NEG	NEG	NEG	NEG	POS	POS	AMB	POS	NEG	NEG	POS
RA6 (Argentina)	NEG	NEG	NEG	NEG	NEG	NEG	NEG	NEG	NEG	NEG	POS	POS	AMB	POS	NEG	NEG	POS
RA7 (Argentina)	NEG	NEG	NEG	NEG	NEG	NEG	NEG	NEG	NEG	NEG	POS	POS	AMB	POS	NEG	NEG	POS
Be62 (Brazil)	NEG	NEG	NEG	NEG	NEG	NEG	NEG	NEG	NEG	NEG	POS	POS	AMB	POS	NEG	NEG	POS
Bmb9393 (Brazil)	NEG	NEG	NEG	NEG	NEG	NEG	NEG	NEG	NEG	NEG	POS	POS	AMB	POS	NEG	NEG	POS
BRA36 (Brazil)	NEG	NEG	NEG	NEG	NEG	NEG	NEG	NEG	NEG	NEG	POS	POS	AMB	POS	NEG	NEG	POS
BZ48 (Brazil)	NEG	NEG	NEG	NEG	NEG	NEG	NEG	NEG	NEG	NEG	POS	POS	AMB	POS	NEG	NEG	POS
Gv51 (Brazil)	NEG	NEG	NEG	NEG	NEG	NEG	NEG	NEG	NEG	NEG	POS	POS	AMB	POS	NEG	NEG	POS
Gv69 (Brazil)	NEG	NEG	NEG	NEG	NEG	NEG	NEG	NEG	NEG	NEG	POS	POS	AMB	POS	NEG	NEG	POS
Gv88 (Brazil)	NEG	NEG	NEG	NEG	NEG	NEG	NEG	NEG	NEG	NEG	POS	POS	AMB	POS	NEG	NEG	POS
HC1335 (Brazil)	NEG	NEG	NEG	NEG	NEG	NEG	NEG	NEG	NEG	NEG	POS	POS	AMB	POS	NEG	NEG	POS
HC1340 (Brazil)	NEG	NEG	NEG	NEG	NEG	NEG	NEG	NEG	NEG	NEG	POS	POS	AMB	POS	NEG	NEG	POS
HC556 (Brazil)	NEG	NEG	NEG	NEG	NEG	NEG	NEG	NEG	NEG	NEG	POS	POS	AMB	POS	NEG	NEG	POS
HU25 (Brazil)	NEG	NEG	NEG	NEG	NEG	NEG	NEG	NEG	NEG	NEG	POS	POS	AMB	POS	NEG	NEG	POS
UB563 (Brazil)	NEG	NEG	NEG	NEG	NEG	NEG	NEG	NEG	NEG	NEG	POS	POS	AMB	POS	NEG	NEG	POS
2A8 (Czech Republic)	NEG	NEG	NEG	NEG	NEG	NEG	NEG	NEG	NEG	NEG	POS	POS	AMB	POS	NEG	NEG	POS
M1229 (Denmark)	POS	NEG	POS	NEG	NEG	NEG	NEG	NEG	NEG	NEG	POS	POS	AMB	POS	NEG	NEG	POS
FRICAR (France)	NEG	NEG	NEG	NEG	NEG	NEG	NEG	NEG	NEG	NEG	POS	POS	AMB	POS	NEG	NEG	POS
D90 (Germany)	POS	NEG	POS	NEG	NEG	NEG	NEG	NEG	NEG	NEG	POS	POS	AMB	POS	NEG	NEG	POS
LIT68 (Lithuania)	POS	NEG	POS	NEG	NEG	NEG	NEG	NEG	NEG	NEG	POS	POS	AMB	POS	NEG	NEG	POS
LIT76 (Lithuania)	POS	NEG	POS	NEG	NEG	NEG	NEG	NEG	NEG	NEG	POS	POS	AMB	POS	NEG	NEG	POS
HGSA142 (Portugal)	NEG	NEG	NEG	NEG	NEG	NEG	NEG	NEG	NEG	NEG	POS	POS	AMB	POS	NEG	NEG	POS
HGSA9 (Portugal)	NEG	NEG	NEG	NEG	NEG	NEG	NEG	NEG	NEG	NEG	POS	POS	AMB	POS	NEG	NEG	POS
HSJ216 (Portugal)	NEG	NEG	NEG	NEG	NEG	NEG	NEG	NEG	NEG	NEG	POS	POS	AMB	POS	NEG	NEG	POS
M278 (Portugal)	NEG	NEG	NEG	NEG	NEG	NEG	NEG	NEG	NEG	NEG	POS	POS	AMB	POS	NEG	NEG	POS
ES26 (Spain)	NEG	NEG	NEG	NEG	NEG	NEG	NEG	NEG	NEG	NEG	POS	POS	AMB	POS	NEG	NEG	POS
UK102 (UK)	POS	NEG	POS	NEG	NEG	NEG	NEG	NEG	NEG	NEG	POS	POS	AMB	POS	NEG	NEG	POS
URU34 (Uruguay)	NEG	NEG	NEG	NEG	NEG	NEG	NEG	NEG	NEG	NEG	POS	POS	AMB	POS	NEG	NEG	POS
IS-125	NEG	NEG	NEG	NEG	NEG	NEG	NEG	NEG	NEG	NEG	POS	POS	AMB	POS	NEG	NEG	POS
IS-157	NEG	NEG	NEG	NEG	NEG	NEG	NEG	NEG	NEG	NEG	POS	POS	AMB	POS	NEG	NEG	POS
Ecuador_1	NEG	NEG	NEG	NEG	NEG	NEG	NEG	NEG	NEG	NEG	POS	POS	POS	POS	NEG	NEG	POS
Ecuador_2	NEG	NEG	NEG	NEG	NEG	NEG	NEG	NEG	NEG	NEG	POS	POS	POS	POS	NEG	NEG	POS
Dresden_15ANR574263	NEG	NEG	NEG	NEG	NEG	NEG	NEG	NEG	NEG	NEG	POS	POS	POS	POS	NEG	NEG	POS
UK-EMRSA-11	NEG	NEG	NEG	NEG	NEG	NEG	NEG	NEG	NEG	NEG	POS	POS	AMB	POS	NEG	NEG	POS
>"South American/ Mid			NEG														
BRA2 (Brazil)	NEG	NEG	NEG	NEG	NEG	NEG	NEG	NEG	NEG	NEG	POS	POS	AMB	POS	NEG	NEG	POS
CHL1 (Chile)	NEG	NEG	NEG	NEG	NEG	NEG	NEG	NEG	NEG	NEG	POS	POS	AMB	POS	NEG	NEG	POS
CHL151 (Chile)	NEG	NEG	NEG	NEG	NEG	NEG	NEG	NEG	NEG	NEG	POS	POS	AMB	POS	NEG	NEG	POS
UCL378 (Chile)	NEG	NEG	NEG	NEG	NEG	NEG	NEG	NEG	NEG	NEG	POS	POS	AMB	POS	NEG	NEG	POS
UP72 (Peru)	NEG	NEG	NEG	NEG	NEG	NEG	NEG	NEG	NEG	NEG	POS	POS	AMB	POS	NEG	NEG	POS
UP81 (Peru)	NEG	NEG	NEG	NEG	NEG	NEG	NEG	NEG	NEG	NEG	POS	POS	AMB	POS	NEG	NEG	POS
>"South American/ Mid			NEG														
UP1073 (Peru)	NEG	NEG	NEG	NEG	NEG	NEG	NEG	NEG	NEG	NEG	POS	POS	AMB	POS	NEG	NEG	POS

STRAIN / ISOLATE	VIRULENCE : ENTEROTOXINS									VIRULENCE : HLG AND LEUKOCIDINS							
	seK	seN2	seQ	seR	seS	seT	seU2	seW	egc (total)	ORF CM14	lukF	lukS		hlgA	PVL	lukF-PV (P83) / lukM	lukD / lukE
	entK	entN2	entQ	entR	entS	entT	entU2	entW	seg, sei, selm, seln, selo , selu		lukF	lukS	lukS (ST22+ST45)	hlgA	lukF-PV, lukS-PV		
	Enterotoxin K	Putative Enterotoxin N2	Enterotoxin Q	Enterotoxin R	Enterotoxin S	Enterotoxin T	Enterotoxin U2	Putative Enterotoxin	egc cluster	Enterotoxin-like protein ORF CM14	Haemolysin gamma / leukocidin, component B	Haemolysin gamma / leukocidin, component C		Haemolysin gamma, component A	Panton Valentine leukocidin F component	F component from ruminant leukocidin	leukocidin E component
>"South American/ Mid			POS														
LIT89 (Lithuania)	POS	NEG	POS	NEG	NEG	NEG	NEG	NEG	NEG	NEG	POS	POS	AMB	POS	NEG	NEG	POS
MRSA_PR1 (Malaysia)	POS	NEG	POS	NEG	NEG	NEG	NEG	NEG	NEG	NEG	POS	POS	AMB	POS	NEG	NEG	POS
Riyadh-2888905-R	POS	NEG	POS	NEG	NEG	NEG	NEG	NEG	NEG	NEG	POS	POS	POS	POS	NEG	NEG	POS
Riyadh-2888915-BC	POS	NEG	POS	NEG	NEG	NEG	NEG	NEG	NEG	NEG	POS	POS	POS	POS	NEG	NEG	POS
Riyadh-2793706-R	POS	NEG	POS	NEG	NEG	NEG	NEG	NEG	NEG	NEG	POS	POS	POS	POS	NEG	NEG	POS
>"South American/ Mid			POS														
Riyadh_SSTI_18_3502925	POS	NEG	POS	NEG	NEG	NEG	NEG	NEG	NEG	NEG	POS	POS	POS	POS	NEG	NEG	POS
Riyadh-2822825-W	POS	NEG	POS	NEG	NEG	NEG	NEG	NEG	NEG	NEG	POS	POS	POS	POS	NEG	NEG	POS
>"South American/ Mid			NEG														
UK-EMRSA-9	NEG	NEG	NEG	NEG	NEG	NEG	NEG	NEG	NEG	NEG	POS	POS	AMB	POS	NEG	NEG	POS
>"South American/ Mid			POS														
Lome_HT20020815	POS	NEG	POS	NEG	NEG	NEG	NEG	NEG	NEG	NEG	POS	POS	POS	POS	NEG	NEG	POS
>"South American/ Mid			COMM														
NA32 (Denmark)	NEG	NEG	NEG	NEG	NEG	NEG	NEG	NEG	NEG	NEG	POS	POS	AMB	POS	NEG	NEG	POS
H24 (Egypt)	NEG	NEG	NEG	NEG	NEG	NEG	NEG	NEG	NEG	NEG	POS	POS	AMB	POS	NEG	NEG	POS
ATCC BAA-39 (=HUSA304) (Hungary)	POS	NEG	POS	NEG	NEG	NEG	NEG	NEG	NEG	NEG	POS	POS	AMB	POS	NEG	NEG	POS
HUSA304 (Hungary)	POS	NEG	POS	NEG	NEG	NEG	NEG	NEG	NEG	NEG	POS	POS	AMB	POS	NEG	NEG	POS
HU106 (Hungary)	POS	NEG	POS	NEG	NEG	NEG	NEG	NEG	NEG	NEG	POS	POS	AMB	POS	NEG	NEG	POS
BSAC27 (UK)	POS	NEG	POS	NEG	NEG	NEG	NEG	NEG	NEG	NEG	POS	POS	AMB	POS	NEG	NEG	POS
NCTC13131, UK-EMRSA-4 (UK)	POS	NEG	POS	NEG	NEG	NEG	NEG	NEG	NEG	NEG	POS	POS	AMB	POS	NEG	NEG	POS
	POS	NEG	POS	NEG	NEG	NEG	NEG	NEG	NEG	NEG	POS	POS	AMB	POS	NEG	NEG	POS
Algiers_HT20040080	NEG	NEG	NEG	NEG	NEG	NEG	NEG	NEG	NEG	NEG	POS	POS	POS	POS	NEG	NEG	POS
Dublin-DSH_AR09_0.0066	POS	NEG	POS	NEG	NEG	NEG	NEG	NEG	NEG	NEG	POS	POS	POS	POS	NEG	NEG	POS
Dublin-DSH_AR09_0-0065	POS	NEG	POS	NEG	NEG	NEG	NEG	NEG	NEG	NEG	AMB	POS	NEG	POS	NEG	NEG	AMB
Dublin-DSH_Phenotype-III_84	POS	NEG	POS	NEG	NEG	NEG	NEG	NEG	NEG	NEG	POS	POS	AMB	POS	NEG	NEG	POS
Hong Kong_130	POS	NEG	POS	NEG	NEG	NEG	NEG	NEG	NEG	NEG	POS	POS	POS	POS	NEG	NEG	POS
Kuwait_018	POS	NEG	POS	NEG	NEG	NEG	NEG	NEG	NEG	NEG	POS	POS	AMB	POS	NEG	NEG	POS
Perth_08-17726	POS	NEG	POS	NEG	NEG	NEG	NEG	NEG	NEG	NEG	POS	POS	POS	POS	NEG	NEG	POS
Riyadh_Alfaisal/KKKSUH_86_MRS-14-279	POS	NEG	POS	NEG	NEG	NEG	NEG	NEG	NEG	NEG	POS	POS	AMB	POS	NEG	NEG	POS
Riyadh_Alfaisal-04_Z3861_831588	NEG	NEG	NEG	NEG	NEG	NEG	NEG	NEG	NEG	NEG	POS	POS	POS	POS	NEG	NEG	POS
Riyadh_Alfaisal-30_S15724_1108013	NEG	NEG	NEG	NEG	NEG	NEG	NEG	NEG	NEG	NEG	POS	POS	POS	POS	NEG	NEG	POS
Riyadh_Alfaisal-6_22A_13_B3992_397721	POS	NEG	POS	NEG	NEG	NEG	NEG	NEG	NEG	NEG	POS	POS	POS	POS	NEG	NEG	POS
Riyadh_SSTI_52_3615482	POS	NEG	POS	NEG	NEG	NEG	NEG	NEG	NEG	NEG	POS	POS	POS	POS	NEG	NEG	POS
Riyadh-2817276-2	POS	NEG	POS	NEG	NEG	NEG	NEG	NEG	NEG	NEG	POS	POS	POS	POS	NEG	NEG	POS
Riyadh-2891670-W	POS	NEG	POS	NEG	NEG	NEG	NEG	NEG	NEG	NEG	POS	POS	POS	POS	NEG	NEG	POS
Riyadh-3006920-W	POS	NEG	POS	NEG	NEG	NEG	NEG	NEG	NEG	NEG	POS	POS	POS	POS	NEG	NEG	POS
Riyadh-R2567782	POS	NEG	POS	NEG	NEG	NEG	NEG	NEG	NEG	NEG	POS	POS	POS	POS	NEG	NEG	POS
Russia-18_0252_Krasnoyarsk_SK2	POS	NEG	POS	NEG	NEG	NEG	NEG	NEG	NEG	NEG	POS	POS	POS	POS	NEG	NEG	POS
UK-EMRSA-7	POS	NEG	POS	NEG	NEG	NEG	NEG	NEG	NEG	NEG	POS	POS	NEG	POS	NEG	NEG	POS
>"South American/ Mid			POS														
Dublin-DSH_AR23_0073	POS	NEG	POS	NEG	NEG	NEG	NEG	NEG	NEG	NEG	POS	POS	AMB	POS	NEG	NEG	POS
>Related to "South Ame			POS														
MRSA-OC3 (Russia)	POS	NEG	POS	NEG	NEG	NEG	NEG	NEG	NEG	NEG	POS	POS	AMB	POS	NEG	NEG	POS
Russia-12_0176_Krasnoyarsk	POS	NEG	POS	NEG	NEG	NEG	NEG	NEG	NEG	NEG	POS	POS	POS	POS	NEG	NEG	POS
Russia-13_0180_Krasnoyarsk	POS	NEG	POS	NEG	NEG	NEG	NEG	NEG	NEG	NEG	POS	POS	POS	POS	NEG	NEG	POS
Russia-16_0249_Krasnoyarsk	POS	NEG	POS	NEG	NEG	NEG	NEG	NEG	NEG	NEG	POS	POS	POS	POS	NEG	NEG	POS
Russia-17_0250_Krasnoyarsk	POS	NEG	POS	NEG	NEG	NEG	NEG	NEG	NEG	NEG	POS	POS	POS	POS	NEG	NEG	POS
>Related to "South Ame			POS														
URU110 (Uruguay)	POS	NEG	POS	NEG	NEG	NEG	NEG	NEG	NEG	NEG	POS	POS	AMB	POS	NEG	NEG	POS
>Related to "South Ame			NEG														
DS_014 (Thailand)	NEG	NEG	NEG	NEG	NEG	NEG	NEG	NEG	NEG	NEG	POS	POS	AMB	POS	NEG	NEG	POS
>Unassigned Middle Eas			VAR														
Frankfurt_Oder_0490031797 (Libya)	POS	NEG	POS	NEG	NEG	NEG	NEG	NEG	NEG	NEG	POS	POS	POS	POS	NEG	NEG	POS
Kuwait_192	POS	NEG	POS	NEG	NEG	NEG	NEG	NEG	NEG	NEG	POS	POS	POS	POS	NEG	NEG	POS
Riyadh-9	POS	NEG	POS	NEG	NEG	NEG	NEG	NEG	NEG	NEG	POS	POS	POS	POS	NEG	NEG	POS
Russia-24_0407_Moscow	NEG	NEG	NEG	NEG	NEG	NEG	NEG	NEG	NEG	NEG	POS	POS	POS	POS	NEG	NEG	POS

STRAIN / ISOLATE	VIRULENCE : HLG AND LEUKOCIDINS				VIRULENCE : HAEMOLYSINS					VIRULENCE : HLB-CONV PHAGES			VIRULENCE : OTHER FACTORS			
	lukX	lukY			corB	hla	hlIII	hlIII	hlb	sak	chp	scn	etA	etB	etD	etD2
	lukX	lukY	lukY (ST30+ST45)	lukY (ST1850)	corB (=hl)	hla	hlIII (cons)	hlIII (other than RF122)	hlb-probe 1	sak	chp	scn	etA	etB	etD	etD2
	leukocidin/ haemolysin toxin family protein	leukocidin/haemolysin toxin family protein			Putative membrane protein	Haemolysin alpha	Putative membrane protein		haemolysin beta	staphylo-kinase	chemotaxis-inhibiting protein (CHIPS)	Staphyl. Complement inhibitor	exfoliative toxin serotype A	exfoliative toxin serotype B	exfoliative toxin D	exfoliative toxin D2
>"Eurasian Clade": TUR1																
TUR1 (Turkey)	POS	POS	NEG	NEG	POS	POS	POS	POS	POS	POS	NEG	POS	NEG	NEG	NEG	NEG
TUR9 (Turkey)	POS	POS	NEG	NEG	POS	POS	POS	POS	POS	POS	NEG	POS	NEG	NEG	NEG	NEG
>"Eurasian Clade" : T013																
T0131 (China)	POS	POS	NEG	NEG	POS	POS	POS	POS	POS	POS	NEG	POS	NEG	NEG	NEG	NEG
MU4 (Turkey)	POS	POS	NEG	NEG	POS	POS	POS	POS	POS	POS	NEG	POS	NEG	NEG	NEG	NEG
DEU11 (Turkey)	POS	POS	NEG	NEG	POS	POS	POS	POS	POS	NEG	NEG	NEG	NEG	NEG	NEG	NEG
DEU16 (Turkey)	POS	POS	NEG	NEG	POS	POS	POS	POS	POS	NEG	NEG	NEG	NEG	NEG	NEG	NEG
Romania_Iasi_MRSA-07_54	POS	POS	NEG	NEG	POS	POS	POS	POS	POS	POS	NEG	POS	NEG	NEG	NEG	NEG
>"Eurasian Clade": arsC-																
Romania_Iasi_MRSA-05_46	POS	POS	NEG	NEG	POS	POS	POS	POS	POS	POS	NEG	POS	NEG	NEG	NEG	NEG
Romania_Iasi_MRSA-06_47	POS	POS	NEG	NEG	POS	POS	POS	POS	POS	POS	NEG	POS	NEG	NEG	NEG	NEG
>"Eurasian Clade": 16K/																
CN79 (China)	POS	POS	NEG	NEG	POS	POS	POS	POS	POS	POS	NEG	POS	NEG	NEG	NEG	NEG
CUHK_BJ2002 (China)	POS	POS	NEG	NEG	POS	POS	POS	POS	POS	POS	NEG	POS	NEG	NEG	NEG	NEG
CUHK_BJ2007 (China)	POS	POS	NEG	NEG	POS	POS	POS	POS	POS	POS	NEG	POS	NEG	NEG	NEG	NEG
3HK (Czech Republic)	POS	POS	NEG	NEG	POS	POS	POS	POS	POS	POS	NEG	POS	NEG	NEG	NEG	NEG
HU109 (Hungary)	POS	POS	NEG	NEG	POS	POS	POS	POS	POS	POS	NEG	POS	NEG	NEG	NEG	NEG
HUR18 (Hungary)	POS	POS	NEG	NEG	POS	POS	POS	POS	POS	POS	NEG	POS	NEG	NEG	NEG	NEG
H482 (Romania)	POS	POS	NEG	NEG	POS	POS	POS	POS	POS	POS	NEG	POS	NEG	NEG	NEG	NEG
16K (Russia)	POS	POS	NEG	NEG	POS	POS	POS	POS	POS	POS	NEG	POS	NEG	NEG	NEG	NEG
DEU10 (Turkey)	POS	POS	NEG	NEG	POS	POS	POS	POS	POS	NEG	NEG	NEG	NEG	NEG	NEG	NEG
DEU12 (Turkey)	POS	POS	NEG	NEG	POS	POS	POS	POS	POS	NEG	NEG	NEG	NEG	NEG	NEG	NEG
DEU14 (Turkey)	POS	POS	NEG	NEG	POS	POS	POS	POS	POS	NEG	NEG	NEG	NEG	NEG	NEG	NEG
DEU15 (Turkey)	POS	POS	NEG	NEG	POS	POS	POS	POS	POS	NEG	NEG	NEG	NEG	NEG	NEG	NEG
DEU17 (Turkey)	POS	POS	NEG	NEG	POS	POS	POS	POS	POS	NEG	NEG	NEG	NEG	NEG	NEG	NEG
DEU19 (Turkey)	POS	POS	NEG	NEG	POS	POS	POS	POS	POS	NEG	NEG	NEG	NEG	NEG	NEG	NEG
DEU2 (Turkey)	POS	POS	NEG	NEG	POS	POS	POS	POS	POS	NEG	NEG	NEG	NEG	NEG	NEG	NEG
DEU20 (Turkey)	POS	POS	NEG	NEG	POS	POS	POS	POS	POS	NEG	NEG	NEG	NEG	NEG	NEG	NEG
DEU23 (Turkey)	POS	POS	NEG	NEG	POS	POS	POS	POS	POS	NEG	NEG	NEG	NEG	NEG	NEG	NEG
DEU3 (Turkey)	POS	POS	NEG	NEG	POS	POS	POS	POS	POS	NEG	NEG	NEG	NEG	NEG	NEG	NEG
DEU5 (Turkey)	POS	POS	NEG	NEG	POS	POS	POS	POS	POS	NEG	NEG	NEG	NEG	NEG	NEG	NEG
DEU6 (Turkey)	POS	POS	NEG	NEG	POS	POS	POS	POS	POS	NEG	NEG	NEG	NEG	NEG	NEG	NEG
DEU8 (Turkey)	POS	POS	NEG	NEG	POS	POS	POS	POS	POS	NEG	NEG	NEG	NEG	NEG	NEG	NEG
HU11 (Turkey)	POS	POS	NEG	NEG	POS	POS	POS	POS	POS	NEG	NEG	NEG	NEG	NEG	NEG	NEG
HU13 (Turkey)	POS	POS	NEG	NEG	POS	POS	POS	POS	POS	NEG	NEG	NEG	NEG	NEG	NEG	NEG
HU14 (Turkey)	POS	POS	NEG	NEG	POS	POS	POS	POS	POS	NEG	NEG	NEG	NEG	NEG	NEG	NEG
HU15 (Turkey)	POS	POS	NEG	NEG	POS	POS	POS	POS	POS	NEG	NEG	NEG	NEG	NEG	NEG	NEG
HU16 (Turkey)	POS	POS	NEG	NEG	POS	POS	POS	POS	POS	NEG	NEG	NEG	NEG	NEG	NEG	NEG
HU17 (Turkey)	POS	POS	NEG	NEG	POS	POS	POS	POS	POS	NEG	NEG	NEG	NEG	NEG	NEG	NEG
HU21 (Turkey)	POS	POS	NEG	NEG	POS	POS	POS	POS	POS	NEG	NEG	NEG	NEG	NEG	NEG	NEG
HU23 (Turkey)	POS	POS	NEG	NEG	POS	POS	POS	POS	POS	NEG	NEG	NEG	NEG	NEG	NEG	NEG
HU26 (Turkey)	POS	POS	NEG	NEG	POS	POS	POS	POS	POS	NEG	NEG	NEG	NEG	NEG	NEG	NEG
HU4 (Turkey)	POS	POS	NEG	NEG	POS	POS	POS	POS	POS	NEG	NEG	NEG	NEG	NEG	NEG	NEG
HU5 (Turkey)	POS	POS	NEG	NEG	POS	POS	POS	POS	POS	NEG	NEG	NEG	NEG	NEG	NEG	NEG
HU6 (Turkey)	POS	POS	NEG	NEG	POS	POS	POS	POS	POS	NEG	NEG	NEG	NEG	NEG	NEG	NEG
HU7 (Turkey)	POS	POS	NEG	NEG	POS	POS	POS	POS	POS	POS	NEG	POS	NEG	NEG	NEG	NEG
HU8 (Turkey)	POS	POS	NEG	NEG	POS	POS	POS	POS	POS	NEG	NEG	NEG	NEG	NEG	NEG	NEG
HU9 (Turkey)	POS	POS	NEG	NEG	POS	POS	POS	POS	POS	NEG	NEG	NEG	NEG	NEG	NEG	NEG
IU1 (Turkey)	POS	POS	NEG	NEG	POS	POS	POS	POS	POS	NEG	NEG	NEG	NEG	NEG	NEG	NEG
IU10 (Turkey)	POS	POS	NEG	NEG	POS	POS	POS	POS	POS	POS	NEG	POS	NEG	NEG	NEG	NEG
IU11 (Turkey)	POS	POS	NEG	NEG	POS	POS	POS	POS	POS	POS	NEG	POS	NEG	NEG	NEG	NEG
IU12 (Turkey)	POS	POS	NEG	NEG	POS	POS	POS	POS	POS	NEG	NEG	NEG	NEG	NEG	NEG	NEG
IU13 (Turkey)	POS	POS	NEG	NEG	POS	POS	POS	POS	POS	NEG	NEG	NEG	NEG	NEG	NEG	NEG
IU15 (Turkey)	POS	POS	NEG	NEG	POS	POS	POS	POS	POS	NEG	NEG	NEG	NEG	NEG	NEG	NEG
IU18 (Turkey)	POS	POS	NEG	NEG	POS	POS	POS	POS	POS	NEG	NEG	NEG	NEG	NEG	NEG	NEG
IU19 (Turkey)	POS	POS	NEG	NEG	POS	POS	POS	POS	POS	NEG	NEG	NEG	NEG	NEG	NEG	NEG
IU2 (Turkey)	POS	POS	NEG	NEG	POS	POS	POS	POS	POS	POS	NEG	POS	NEG	NEG	NEG	NEG
IU4 (Turkey)	POS	POS	NEG	NEG	POS	POS	POS	POS	POS	POS	NEG	POS	NEG	NEG	NEG	NEG
IU6 (Turkey)	POS	POS	NEG	NEG	POS	POS	POS	POS	POS	NEG	NEG	NEG	NEG	NEG	NEG	NEG
IU7 (Turkey)	POS	POS	NEG	NEG	POS	POS	POS	POS	POS	NEG	NEG	NEG	NEG	NEG	NEG	NEG
IU9 (Turkey)	POS	POS	NEG	NEG	POS	POS	POS	POS	POS	POS	NEG	POS	NEG	NEG	NEG	NEG
MU1 (Turkey)	POS	POS	NEG	NEG	POS	POS	POS	POS	POS	NEG	NEG	NEG	NEG	NEG	NEG	NEG
MU10 (Turkey)	POS	POS	NEG	NEG	POS	POS	POS	POS	POS	NEG	NEG	NEG	NEG	NEG	NEG	NEG
MU20 (Turkey)	POS	POS	NEG	NEG	POS	POS	POS	POS	POS	NEG	NEG	NEG	NEG	NEG	NEG	NEG
Mu3 (Turkey)	POS	POS	NEG	NEG	POS	POS	POS	POS	POS	NEG	NEG	NEG	NEG	NEG	NEG	NEG
MU5 (Turkey)	POS	POS	NEG	NEG	POS	POS	POS	POS	POS	NEG	NEG	NEG	NEG	NEG	NEG	NEG
MU6 (Turkey)	POS	POS	NEG	NEG	POS	POS	POS	POS	POS	NEG	NEG	NEG	NEG	NEG	NEG	NEG
MU7 (Turkey)	POS	POS	NEG	NEG	POS	POS	POS	POS	POS	NEG	NEG	NEG	NEG	NEG	NEG	NEG
TUR27 (Turkey)	POS	POS	NEG	NEG	POS	POS	POS	POS	POS	POS	NEG	POS	NEG	NEG	NEG	NEG
NCTR 32S	POS	POS	NEG	NEG	POS	POS	POS	POS	POS	POS	NEG	POS	NEG	NEG	NEG	NEG
SA02_A4 = Russia-06_0085_Moscow	POS	POS	NEG	NEG	POS	POS	POS	POS	POS	POS	NEG	POS	NEG	NEG	NEG	NEG
	POS	POS	NEG	NEG	POS	POS	POS	POS	POS	POS	NEG	POS	NEG	NEG	NEG	NEG
Dresden_08V35987 (Turkey)	POS	POS	NEG	NEG	POS	POS	POS	POS	POS	NEG	NEG	NEG	NEG	NEG	NEG	NEG
Dresden_17ANR577152	POS	POS	NEG	NEG	POS	POS	POS	POS	POS	POS	NEG	POS	NEG	NEG	NEG	NEG
Dresden_17ANR580374 (Makedonia)	POS	AMB	NEG	NEG	POS	POS	POS	POS	POS	NEG	NEG	NEG	NEG	NEG	NEG	NEG
Hong Kong_69-II	POS	POS	NEG	NEG	POS	POS	POS	POS	POS	POS	NEG	POS	NEG	NEG	NEG	NEG
Hong Kong_93	POS	POS	NEG	NEG	POS	POS	POS	POS	POS	POS	NEG	POS	NEG	NEG	NEG	NEG
Rawalpindi_Kidney Center_03_SK1	POS	POS	NEG	NEG	POS	POS	POS	POS	POS	POS	NEG	POS	NEG	NEG	NEG	NEG
Rawalpindi_Kidney Center_08	POS	POS	NEG	NEG	POS	POS	POS	POS	POS	POS	NEG	POS	NEG	NEG	NEG	NEG
Rawalpindi_Kidney Center_10	NEG	POS	NEG	NEG	POS	POS	POS	POS	POS	POS	NEG	POS	NEG	NEG	NEG	NEG
Rawalpindi_Kidney Center_19	POS	POS	NEG	NEG	POS	POS	POS	POS	POS	POS	NEG	POS	NEG	NEG	NEG	NEG
Rawalpindi_Kidney Center_50_SK1	POS	POS	NEG	NEG	POS	POS	POS	POS	POS	POS	NEG	POS	NEG	NEG	NEG	NEG
Romania_Iasi_BC-49_430	POS	POS	NEG	NEG	POS	POS	POS	POS	POS	POS	NEG	POS	NEG	NEG	NEG	NEG
Romania_Iasi_MRSA_284	POS	POS	NEG	NEG	POS	POS	POS	POS	POS	POS	NEG	POS	NEG	NEG	NEG	NEG
Romania_Iasi_MRSA-32_318	POS	POS	NEG	NEG	POS	POS	POS	POS	POS	NEG	NEG	NEG	NEG	NEG	NEG	NEG
Romania_Iasi_MRSA-54_430	POS	POS	NEG	NEG	POS	POS	POS	POS	POS	POS	NEG	POS	NEG	NEG	NEG	NEG
Romania_Iasi_SSTI-01_2	POS	POS	NEG	NEG	POS	POS	POS	POS	POS	NEG	NEG	NEG	NEG	NEG	NEG	NEG
Romania_Iasi_SSTI-10_101	POS	POS	NEG	NEG	POS	POS	POS	POS	POS	POS	NEG	POS	NEG	NEG	NEG	NEG
Romania_Iasi_SSTI-11_106	POS	POS	NEG	NEG	POS	POS	POS	POS	POS	POS	NEG	POS	NEG	NEG	NEG	#ZAHL!
Russia-01_0001_SaintPetersburg	POS	POS	NEG	NEG	POS	POS	POS	POS	POS	POS	POS	POS	NEG	NEG	NEG	NEG
Russia-02_0004_SaintPetersburg	POS	POS	NEG	NEG	POS	POS	POS	POS	POS	POS	POS	POS	NEG	NEG	NEG	NEG
Russia-03_0057_SaintPetersburg	POS	POS	NEG	NEG	POS	POS	POS	POS	POS	POS	POS	POS	NEG	NEG	NEG	NEG
Russia-04_0076_Moscow	POS	POS	NEG	NEG	POS	POS	POS	POS	POS	POS	NEG	POS	NEG	NEG	NEG	NEG
Russia-05_0078_Moscow	POS	POS	NEG	NEG	POS	POS	POS	POS	POS	POS	NEG	POS	NEG	NEG	NEG	NEG
Russia-08_0135_Moscow	POS	POS	NEG	NEG	POS	POS	POS	POS	POS	POS	NEG	POS	NEG	NEG	NEG	NEG
Russia-09_0150_Kurgan	POS	POS	NEG	NEG	POS	POS	POS	POS	POS	POS	NEG	POS	NEG	NEG	NEG	NEG
Russia-10_0162_Kurgan	POS	POS	NEG	NEG	POS	POS	POS	POS	POS	POS	NEG	POS	NEG	NEG	NEG	NEG
Russia-14_0184_Moscow	POS	POS	NEG	NEG	POS	POS	POS	POS	POS	POS	NEG	POS	NEG	NEG	NEG	NEG
Russia-15_0232_SaintPetersburg	POS	POS	NEG	NEG	POS	POS	POS	POS	POS	POS	NEG	POS	NEG	NEG	NEG	NEG
Russia-22_0391_Chelyabinsk	POS	POS	NEG	NEG	POS	POS	POS	POS	POS	POS	NEG	POS	NEG	NEG	NEG	NEG
Russia-23_0392_Chelyabinsk	POS	POS	NEG	NEG	POS	POS	POS	POS	POS	POS	NEG	POS	NEG	NEG	NEG	NEG
>"Eurasian Clade" : IU17																
IU17 (Turkey)	POS	POS	NEG	NEG	POS	POS	POS	POS	POS	POS	NEG	POS	NEG	NEG	NEG	NEG
IU20 (Turkey)	POS	POS	NEG	NEG	POS	POS	POS	POS	POS	POS	NEG	POS	NEG	NEG	NEG	NEG

STRAIN / ISOLATE	VIRULENCE : HLG AND LEUKOCIDINS				VIRULENCE : HAEMOLYSINS					VIRULENCE : HLB-CONV PHAGES			VIRULENCE : OTHER FACTORS			
	lukX	lukY			corB	hla	hlIII	hlIII	hlb	sak	chp	scn	etA	etB	etD	etD2
	lukX	lukY	lukY (ST30+ST45)	lukY (ST1850)	corB (=hl)	hla	hlIII (cons)	hlIII (other than RF122)	hlb-probe 1	sak	chp	scn	etA	etB	etD	etD2
	leukocidin/ haemolysin toxin family protein	leukocidin/haemolysin toxin family protein			Putative membrane protein	Haemolysin alpha	Putative membrane protein		haemolysin beta	staphylo-kinase	chemotaxis-inhibiting protein (CHIPS)	Staphyl. Comple-ment inhibitor	exfoliative toxin serotype A	exfoliative toxin serotype B	exfoliative toxin D	exfoliative toxin D2
>"Eurasian Clade": Spora																
Dresden_16ANR581769	POS	POS	NEG	NEG	POS	POS	POS	POS	POS	NEG	NEG	NEG	NEG	NEG	NEG	NEG
Dresden_17ANR578769	POS	POS	NEG	NEG	POS	POS	POS	POS	POS	NEG	NEG	NEG	NEG	NEG	NEG	NEG
>"Eurasian Clade": Kuwa																
Kuwait_306	POS	POS	NEG	NEG	POS	POS	POS	POS	POS	NEG	NEG	NEG	NEG	NEG	NEG	NEG
Kuwait_301	POS	POS	NEG	NEG	POS	POS	POS	POS	POS	NEG	NEG	NEG	NEG	NEG	NEG	NEG
>"European Clade": "Gr																
GRE18 (Greece)	POS	POS	NEG	NEG	POS	POS	POS	POS	POS	POS	NEG	POS	NEG	NEG	NEG	NEG
GRE317 (Greece)	POS	POS	NEG	NEG	POS	POS	POS	POS	POS	POS	NEG	POS	NEG	NEG	NEG	NEG
GRE4 (Greece)	POS	POS	NEG	NEG	POS	POS	POS	POS	POS	POS	NEG	POS	NEG	NEG	NEG	NEG
R35 (USA)	POS	POS	NEG	NEG	POS	NEG	POS	POS	POS	POS	NEG	POS	NEG	NEG	NEG	NEG
Casablanca_HT20060548	POS	POS	NEG	NEG	POS	POS	POS	POS	POS	POS	NEG	POS	NEG	NEG	NEG	NEG
Casablanca_HT20060550	POS	POS	NEG	NEG	POS	POS	POS	POS	POS	POS	NEG	POS	NEG	NEG	NEG	NEG
Dresden_01V34572 (Greece)	POS	POS	NEG	NEG	POS	POS	POS	POS	POS	POS	NEG	POS	NEG	NEG	NEG	NEG
Dresden_01V36469	POS	POS	NEG	NEG	POS	POS	POS	POS	POS	POS	NEG	POS	NEG	NEG	NEG	NEG
Dresden_01V39123	POS	POS	NEG	NEG	POS	POS	POS	POS	POS	POS	NEG	POS	NEG	NEG	NEG	NEG
Dresden_01V39143	POS	POS	NEG	NEG	POS	POS	POS	POS	POS	POS	NEG	POS	NEG	NEG	NEG	NEG
Greece 1_3680_Harmony	AMB	POS	NEG	NEG	POS	AMB	POS	POS	POS	POS	NEG	AMB	NEG	NEG	NEG	NEG
>"European Clade": UK-(																
ANS46 (Australia)	POS	POS	NEG	NEG	POS	POS	POS	POS	POS	POS	NEG	POS	NEG	NEG	NEG	NEG
LHH1 (USA)	POS	POS	NEG	NEG	POS	POS	POS	POS	POS	POS	NEG	POS	NEG	NEG	NEG	NEG
Dublin-DSH_AR01_0-1085	POS	POS	NEG	NEG	POS	POS	POS	POS	POS	POS	NEG	POS	NEG	NEG	NEG	NEG
Dublin-DSH_AR01_0-1118	POS	POS	NEG	NEG	POS	POS	POS	POS	POS	POS	NEG	POS	NEG	NEG	NEG	NEG
Dublin-DSH_AR15_0098	POS	POS	NEG	NEG	POS	POS	POS	POS	POS	POS	NEG	POS	NEG	NEG	NEG	NEG
Dublin-DSH_AR15_0104	POS	POS	NEG	NEG	POS	POS	POS	POS	POS	POS	NEG	POS	NEG	NEG	NEG	NEG
UK_NCTC11939_UK-1_Harmony	POS	POS	NEG	NEG	POS	POS	POS	POS	POS	POS	NEG	POS	NEG	NEG	NEG	NEG
>"European Clade": BK2																
BK2421 (USA)	POS	POS	NEG	NEG	POS	POS	POS	POS	POS	POS	NEG	POS	NEG	NEG	NEG	NEG
>"Aussi/NZ Clade", JKD6																
JKD6009 (Australia)	POS	POS	NEG	NEG	POS	POS	POS	POS	POS	POS	NEG	POS	NEG	NEG	NEG	NEG
JKD6008 (New Zealand)	POS	POS	NEG	NEG	POS	POS	POS	POS	POS	POS	NEG	POS	NEG	NEG	NEG	NEG
Perth_2015-S20209	POS	POS	NEG	NEG	POS	POS	POS	POS	POS	POS	NEG	POS	NEG	NEG	NEG	NEG
Perth_2015-S20305	POS	POS	NEG	NEG	POS	POS	POS	POS	POS	POS	NEG	POS	NEG	NEG	NEG	NEG
Perth_2015-S20603	POS	POS	NEG	NEG	POS	POS	POS	POS	POS	POS	NEG	POS	NEG	NEG	NEG	NEG
Perth_2015-S20607	POS	POS	NEG	NEG	POS	POS	POS	POS	POS	POS	NEG	POS	NEG	NEG	NEG	NEG
Perth_2015-S20608	POS	POS	NEG	NEG	POS	POS	POS	POS	POS	POS	NEG	POS	NEG	NEG	NEG	NEG
Perth_2015-S20610	POS	POS	NEG	NEG	POS	POS	POS	POS	POS	POS	NEG	POS	NEG	NEG	NEG	NEG
Perth_2015-S20613	POS	POS	NEG	NEG	POS	POS	POS	POS	POS	POS	NEG	POS	NEG	NEG	NEG	NEG
Perth_2015-S20617	POS	NEG	NEG	NEG	POS	POS	POS	POS	POS	POS	NEG	NEG	NEG	NEG	NEG	NEG
Perth_2015-S20622	POS	POS	NEG	NEG	POS	POS	POS	POS	POS	POS	NEG	NEG	NEG	NEG	NEG	NEG
Perth_2015-S23619	POS	POS	NEG	NEG	POS	POS	POS	POS	POS	POS	NEG	POS	NEG	NEG	NEG	NEG
Perth_2015-S41210	POS	POS	NEG	NEG	POS	POS	POS	POS	POS	POS	NEG	POS	NEG	NEG	NEG	NEG
Perth_2015-S51001	POS	POS	NEG	NEG	POS	POS	POS	POS	POS	NEG	NEG	NEG	NEG	NEG	NEG	NEG
Perth_2015-S51003	POS	POS	NEG	NEG	POS	POS	POS	POS	POS	NEG	NEG	NEG	NEG	NEG	NEG	NEG
Perth_2015-S51006	POS	POS	NEG	NEG	POS	POS	POS	POS	POS	NEG	NEG	NEG	NEG	NEG	NEG	NEG
Perth_2015-S51007	POS	POS	NEG	NEG	POS	POS	POS	POS	POS	NEG	NEG	NEG	NEG	NEG	NEG	NEG
Perth_2015-S51008	POS	POS	NEG	NEG	POS	POS	POS	POS	POS	NEG	NEG	NEG	NEG	NEG	NEG	NEG
Perth_2015-S51014	POS	POS	NEG	NEG	POS	NEG	POS	POS	POS	NEG	NEG	NEG	NEG	NEG	NEG	NEG
Perth_2015-S51408	POS	POS	NEG	NEG	POS	POS	POS	POS	POS	POS	NEG	POS	NEG	NEG	NEG	NEG
Perth_2016-S20614	POS	POS	NEG	NEG	POS	NEG	POS	POS	POS	POS	NEG	POS	NEG	NEG	NEG	NEG
Perth_2016-S23614	POS	POS	NEG	NEG	POS	POS	POS	POS	POS	POS	NEG	POS	NEG	NEG	NEG	NEG
Perth_2016-S42009	POS	POS	NEG	NEG	POS	POS	POS	POS	POS	POS	NEG	POS	NEG	NEG	NEG	NEG
Perth_2016-S51001	POS	POS	NEG	NEG	POS	POS	POS	POS	POS	NEG	NEG	NEG	NEG	NEG	NEG	NEG
Perth_2016-S51403	POS	POS	NEG	NEG	POS	POS	POS	POS	POS	POS	NEG	POS	NEG	NEG	NEG	NEG
Perth_07 RPA 20	POS	POS	NEG	NEG	POS	POS	POS	POS	POS	POS	NEG	POS	NEG	NEG	NEG	NEG
Perth_08-19088	POS	POS	NEG	NEG	POS	POS	POS	POS	POS	POS	NEG	POS	NEG	NEG	NEG	NEG
Perth_01-15351	POS	POS	NEG	NEG	POS	POS	POS	POS	POS	POS	NEG	POS	NEG	NEG	NEG	NEG
Perth_01-15357	POS	POS	NEG	NEG	POS	POS	POS	POS	POS	POS	NEG	POS	NEG	NEG	NEG	NEG
Perth_01-15419	AMB	POS	NEG	NEG	POS	POS	POS	AMB	POS	POS	NEG	POS	NEG	NEG	NEG	NEG
Perth_01-16309	AMB	POS	NEG	NEG	POS	POS	POS	POS	POS	NEG	NEG	NEG	NEG	NEG	NEG	NEG
Perth_03 SNP 95	POS	POS	NEG	NEG	POS	POS	POS	POS	POS	NEG	NEG	AMB	NEG	NEG	NEG	NEG
Perth_2003_AH_47	POS	POS	NEG	NEG	POS	POS	POS	POS	POS	POS	NEG	POS	NEG	NEG	NEG	NEG
>"Australian/NZ Clade":																
Perth_2015-S20623	POS	POS	NEG	NEG	POS	POS	POS	POS	POS	POS	NEG	POS	NEG	NEG	NEG	NEG
Perth_2015-S20826	POS	POS	NEG	NEG	POS	POS	POS	POS	POS	POS	NEG	POS	NEG	NEG	NEG	NEG
Perth_05 NH 56	POS	POS	NEG	NEG	POS	POS	POS	POS	POS	POS	NEG	POS	NEG	NEG	NEG	NEG
>CC5-MRSA-III LA MRSA																
Delbrück 38 isolate from domestic turkey	POS	POS	NEG	NEG	POS	POS	POS	POS	POS	NEG	AMB	AMB	NEG	NEG	NEG	NEG
> Staph. pseudintermed																
KM1381	NEG	NEG	NEG	NEG	NEG	NEG	NEG	NEG	NEG	NEG	NEG	NEG	NEG	NEG	NEG	NEG
>"South-East Asian Clad										COMM	VAR	COMM				
M92 (Canada)	POS	POS	NEG	NEG	POS	POS	POS	POS	POS	POS	NEG	POS	NEG	NEG	NEG	NEG
CHI59 (China)	POS	POS	NEG	NEG	POS	POS	POS	POS	POS	POS	NEG	POS	NEG	NEG	NEG	NEG
CHI61 (China)	POS	POS	NEG	NEG	POS	POS	POS	POS	POS	POS	NEG	POS	NEG	NEG	NEG	NEG
CUHK_HK1997 (China)	POS	POS	NEG	NEG	POS	NEG	POS	POS	POS	POS	NEG	POS	NEG	NEG	NEG	NEG
M996 (China)	POS	POS	NEG	NEG	POS	POS	POS	POS	POS	NEG	NEG	NEG	NEG	NEG	NEG	NEG
H211 (Denmark)	POS	POS	NEG	NEG	POS	POS	POS	POS	POS	POS	POS	POS	NEG	NEG	NEG	NEG
H216 (Denmark)	POS	POS	NEG	NEG	POS	POS	POS	POS	POS	POS	POS	POS	NEG	NEG	NEG	NEG
D71 (Germany)	POS	POS	NEG	NEG	POS	POS	POS	POS	POS	POS	NEG	NEG	NEG	NEG	NEG	NEG
NMR05 (India)	POS	POS	NEG	NEG	POS	POS	POS	POS	POS	POS	NEG	POS	NEG	NEG	NEG	NEG
MAL11 (Malaysia)	POS	POS	NEG	NEG	POS	POS	POS	POS	POS	POS	NEG	POS	NEG	NEG	NEG	NEG
MAL9 (Malaysia)	POS	POS	NEG	NEG	POS	POS	POS	POS	POS	POS	NEG	POS	NEG	NEG	NEG	NEG
Na21 (Sri Lanka)	POS	POS	NEG	NEG	POS	POS	POS	POS	POS	POS	NEG	POS	NEG	NEG	NEG	NEG
M592 (Syria)	POS	POS	NEG	NEG	POS	POS	POS	POS	POS	POS	POS	POS	NEG	NEG	NEG	NEG
H202 (Thailand)	POS	POS	NEG	NEG	POS	POS	POS	POS	POS	POS	POS	POS	NEG	NEG	NEG	NEG
S102 (Thailand)	POS	POS	NEG	NEG	POS	POS	POS	POS	POS	POS	POS	POS	NEG	NEG	NEG	NEG
S130 (Thailand)	POS	POS	NEG	NEG	POS	POS	POS	POS	POS	POS	POS	POS	NEG	NEG	NEG	NEG
S40 (Thailand)	POS	POS	NEG	NEG	POS	POS	POS	POS	POS	POS	POS	POS	NEG	NEG	NEG	NEG
S71 (Thailand)	POS	POS	NEG	NEG	POS	POS	POS	POS	POS	POS	POS	POS	NEG	NEG	NEG	NEG
S87 (Thailand)	POS	POS	NEG	NEG	POS	POS	POS	POS	POS	POS	POS	POS	NEG	NEG	NEG	NEG
S93 (Thailand)	POS	POS	NEG	NEG	POS	POS	POS	POS	POS	POS	POS	POS	NEG	NEG	NEG	NEG
US_002 (Thailand)	POS	POS	NEG	NEG	POS	POS	POS	POS	POS	POS	POS	POS	NEG	NEG	NEG	NEG
US_017 (Thailand)	POS	POS	NEG	NEG	POS	POS	POS	POS	POS	POS	POS	POS	NEG	NEG	NEG	NEG
WAMC6102 (Thailand)	POS	POS	NEG	NEG	POS	POS	POS	POS	POS	POS	NEG	POS	NEG	NEG	NEG	NEG
NCTC_13626_TW20 (UK)	POS	POS	NEG	NEG	POS	POS	POS	POS	POS	POS	NEG	POS	NEG	NEG	NEG	NEG
BSAC3021 (UK)	POS	POS	NEG	NEG	POS	POS	POS	POS	POS	POS	NEG	POS	NEG	NEG	NEG	NEG
BSAC3046 (UK)	POS	POS	NEG	NEG	POS	POS	POS	POS	POS	POS	NEG	POS	NEG	NEG	NEG	NEG
1193_SAUR (USA)	POS	POS	NEG	NEG	POS	POS	POS	POS	POS	POS	NEG	POS	NEG	NEG	NEG	NEG
1194_SAUR (USA)	POS	POS	NEG	NEG	POS	POS	POS	POS	POS	POS	NEG	POS	NEG	NEG	NEG	NEG

STRAIN / ISOLATE	VIRULENCE : HLG AND LEUKOCIDINS				VIRULENCE : HAEMOLYSINS					VIRULENCE : HLB-CONV PHAGES			VIRULENCE : OTHER FACTORS			
	lukX	lukY			corB	hla	hlIII	hlIII	hlb	sak	chp	scn	etA	etB	etD	etD2
	lukX	lukY	lukY (ST30+ST45)	lukY (ST1850)	corB (=hl)	hla	hlIII (cons)	hlIII (other than RF122)	hlb-probe 1	sak	chp	scn	etA	etB	etD	etD2
	leukocidin/ haemolysin toxin family protein	leukocidin/haemolysin toxin family protein			Putative membrane protein	Haemolysin alpha	Putative membrane protein		haemolysin beta	staphylo-kinase	chemotaxis-inhibiting protein (CHIPS)	Staphyl. Complement inhibitor	exfoliative toxin serotype A	exfoliative toxin serotype B	exfoliative toxin D	exfoliative toxin D2
>"South-East Asian Clade": TW20 (Irish AR44), c																
1195_SAUR (USA)	POS	POS	NEG	NEG	POS	POS	POS	POS	POS	POS	NEG	POS	NEG	NEG	NEG	NEG
FVRH6002 (USA)	POS	POS	NEG	NEG	POS	POS	POS	POS	POS	POS	NEG	POS	NEG	NEG	NEG	NEG
KINW6048 (USA)	POS	POS	NEG	NEG	POS	POS	POS	POS	POS	POS	NEG	POS	NEG	NEG	NEG	NEG
LAMC0011 (USA)	POS	POS	NEG	NEG	POS	POS	POS	POS	POS	POS	NEG	POS	NEG	NEG	NEG	NEG
SJOS6053 (USA)	POS	POS	NEG	NEG	POS	POS	POS	POS	POS	POS	NEG	POS	NEG	NEG	NEG	NEG
SJOS6072 (USA)	POS	POS	NEG	NEG	POS	POS	POS	POS	POS	POS	NEG	POS	NEG	NEG	NEG	NEG
UCIM6042 (USA)	POS	POS	NEG	NEG	POS	POS	POS	POS	POS	POS	NEG	POS	NEG	NEG	NEG	NEG
Perth_2015-S31902	NEG	NEG	NEG	NEG	POS	POS	POS	POS	POS	POS	NEG	NEG	NEG	NEG	NEG	NEG
Perth_2015-S31904	POS	POS	NEG	NEG	POS	POS	POS	POS	POS	POS	NEG	POS	NEG	NEG	NEG	NEG
Perth_2015-S31913	POS	POS	NEG	NEG	POS	POS	POS	POS	POS	POS	NEG	NEG	NEG	NEG	NEG	NEG
Perth_2015-S31918	POS	POS	NEG	NEG	POS	NEG	POS	POS	POS	POS	NEG	NEG	NEG	NEG	NEG	NEG
Dublin-DSH_AR44_E1183	POS	POS	NEG	NEG	POS	POS	POS	POS	POS	POS	NEG	POS	NEG	NEG	NEG	NEG
Dublin-DSH_Unfamiliar-3_E1520	AMB	POS	NEG	NEG	POS	NEG	POS	POS	POS	POS	NEG	POS	NEG	NEG	NEG	NEG
Hong Kong_042	POS	POS	NEG	NEG	POS	POS	POS	POS	POS	POS	NEG	POS	NEG	NEG	NEG	NEG
Hong Kong_101	POS	POS	NEG	NEG	POS	POS	POS	POS	AMB	NEG	NEG	NEG	NEG	NEG	NEG	NEG
Hong Kong_111	POS	POS	NEG	NEG	POS	POS	POS	POS	POS	POS	NEG	POS	NEG	NEG	NEG	NEG
Hong Kong_113	POS	POS	NEG	NEG	POS	POS	POS	POS	AMB	NEG	NEG	NEG	NEG	NEG	NEG	NEG
Hong Kong_115	POS	POS	NEG	NEG	POS	POS	POS	POS	POS	POS	NEG	POS	NEG	NEG	NEG	NEG
Hong Kong_118	POS	POS	NEG	NEG	POS	POS	POS	POS	POS	POS	NEG	POS	NEG	NEG	NEG	NEG
Hong Kong_125	POS	POS	NEG	NEG	POS	POS	POS	POS	POS	NEG	NEG	NEG	NEG	NEG	NEG	NEG
Hong Kong_129	POS	POS	NEG	NEG	POS	POS	POS	POS	POS	NEG	NEG	NEG	NEG	NEG	NEG	NEG
Hong Kong_133	POS	POS	NEG	NEG	POS	POS	POS	POS	POS	POS	NEG	POS	NEG	NEG	NEG	NEG
Kuwait_009	POS	POS	NEG	NEG	POS	POS	POS	POS	POS	POS	POS	POS	NEG	NEG	NEG	NEG
Kuwait_103	POS	POS	NEG	NEG	POS	POS	POS	POS	POS	POS	POS	POS	NEG	NEG	NEG	NEG
Kuwait_107	POS	POS	NEG	NEG	POS	POS	POS	POS	POS	POS	POS	POS	NEG	NEG	NEG	NEG
Perth_03 AH 88	AMB	POS	NEG	NEG	POS	POS	POS	POS	POS	POS	NEG	POS	NEG	NEG	NEG	NEG
Perth_08-16905	POS	POS	NEG	NEG	POS	POS	POS	POS	POS	POS	NEG	POS	NEG	NEG	NEG	NEG
Riyadh_Alfaisal-10_SICU_13_B5672_1117353	POS	POS	NEG	NEG	POS	POS	POS	POS	POS	POS	POS	POS	NEG	NEG	NEG	NEG
Riyadh_SSTI_17_3508602	POS	POS	NEG	NEG	POS	POS	POS	POS	POS	POS	POS	POS	NEG	NEG	NEG	NEG
Riyadh-2822088-R	POS	POS	NEG	NEG	POS	POS	POS	POS	POS	POS	POS	POS	NEG	NEG	NEG	NEG
Trinidad&Tobago_2012_195 (619/12)	POS	POS	NEG	NEG	POS	POS	POS	POS	POS	NEG	NEG	NEG	NEG	NEG	NEG	NEG
Trinidad&Tobago_2012_370 (1112-52177)	POS	POS	NEG	NEG	POS	POS	POS	POS	POS	NEG	NEG	NEG	NEG	NEG	NEG	NEG
Trinidad&Tobago_2012_378 (4139)	POS	POS	NEG	NEG	POS	POS	POS	POS	POS	POS	NEG	POS	NEG	NEG	NEG	NEG
Trinidad&Tobago_2013_PA21	POS	POS	NEG	NEG	POS	POS	POS	POS	POS	POS	NEG	POS	NEG	NEG	NEG	NEG
Trinidad&Tobago_2013_PA22	POS	POS	NEG	NEG	POS	POS	POS	POS	POS	POS	NEG	POS	NEG	NEG	NEG	NEG
Trinidad&Tobago_2912_340 (8523/12)	POS	POS	NEG	NEG	POS	POS	POS	POS	POS	POS	NEG	POS	NEG	NEG	NEG	NEG
Trinidad&Tobago_MRSA2010_105	POS	POS	NEG	NEG	POS	POS	POS	POS	POS	POS	NEG	POS	NEG	NEG	NEG	NEG
Trinidad&Tobago_MRSA2010_108	POS	POS	NEG	NEG	POS	POS	POS	POS	POS	POS	NEG	POS	NEG	NEG	NEG	NEG
Trinidad&Tobago_MRSA2010_33	POS	POS	NEG	NEG	POS	POS	POS	POS	POS	POS	NEG	POS	NEG	NEG	NEG	NEG
Trinidad&Tobago_MRSA2010_79	POS	POS	NEG	NEG	POS	POS	POS	POS	POS	POS	NEG	POS	NEG	NEG	NEG	NEG
Trinidad&Tobago_MRSA2010_88	POS	POS	NEG	NEG	POS	POS	POS	POS	POS	POS	NEG	POS	NEG	NEG	NEG	NEG
Trinidad&Tobago_MRSA2010_97	POS	POS	NEG	NEG	POS	POS	POS	POS	POS	POS	NEG	POS	NEG	NEG	NEG	NEG
Trinidad&Tobago_SSTI 2012_031	POS	POS	NEG	NEG	POS	POS	POS	POS	POS	NEG	NEG	NEG	NEG	NEG	NEG	NEG
Uganda-29_17148_250811	POS	POS	NEG	NEG	POS	POS	POS	POS	POS	POS	POS	POS	NEG	NEG	NEG	NEG
>"South-East Asian Clad																
UCIM6015	POS	POS	NEG	NEG	POS	POS	POS	POS	POS	POS	NEG	POS	NEG	NEG	NEG	NEG
Hong Kong_121	POS	POS	NEG	NEG	POS	POS	POS	POS	POS	NEG	NEG	NEG	NEG	NEG	NEG	NEG
>"South-East Asian Clad																
Z172 (Taiwan)	POS	POS	NEG	NEG	POS	POS	POS	POS	POS	POS	NEG	POS	NEG	NEG	NEG	NEG
Russia-19_0342_Kurgan	POS	POS	NEG	NEG	POS	POS	POS	POS	POS	POS	NEG	POS	NEG	NEG	NEG	NEG
>"South-East Asian Clad																
Perth_01-15392	POS	POS	NEG	NEG	POS	POS	POS	POS	POS	POS	NEG	POS	NEG	NEG	NEG	NEG
>"South-East Asian Clad																
XN108 (China)	POS	POS	NEG	NEG	POS	POS	POS	POS	POS	POS	NEG	POS	NEG	NEG	NEG	NEG
NMR09 (India)	POS	POS	NEG	NEG	POS	POS	POS	POS	POS	POS	NEG	POS	NEG	NEG	NEG	NEG
346 (Malaysia)	POS	POS	NEG	NEG	POS	POS	POS	POS	POS	NEG	NEG	NEG	NEG	NEG	NEG	NEG
DS_009 (Thailand)	POS	POS	NEG	NEG	POS	POS	POS	POS	POS	POS	POS	POS	NEG	NEG	NEG	NEG
18ANR581005 (Ind)	POS	POS	NEG	NEG	POS	POS	POS	POS	POS	POS	NEG	POS	NEG	NEG	NEG	NEG
Bengalaru_ST20141405	POS	POS	NEG	NEG	POS	POS	POS	POS	POS	POS	NEG	POS	NEG	NEG	NEG	NEG
Bengalaru_ST20141406	POS	POS	NEG	NEG	POS	POS	POS	POS	POS	POS	NEG	POS	NEG	NEG	NEG	NEG
Bengalaru_ST20141417	POS	POS	NEG	NEG	POS	POS	POS	POS	POS	POS	NEG	POS	NEG	NEG	NEG	NEG
Hong Kong_107	POS	POS	NEG	NEG	POS	POS	POS	POS	POS	NEG	NEG	NEG	NEG	NEG	NEG	NEG
Hong Kong_12	POS	POS	NEG	NEG	POS	POS	POS	POS	POS	POS	NEG	POS	NEG	NEG	NEG	NEG
Hong Kong_132	POS	POS	NEG	NEG	POS	POS	POS	POS	POS	NEG	NEG	NEG	NEG	NEG	NEG	NEG
Riyadh-3028763-R	POS	POS	NEG	NEG	POS	POS	POS	POS	POS	POS	POS	POS	NEG	NEG	NEG	NEG
Trinidad&Tobago_MRSA2010_94	POS	POS	NEG	NEG	POS	POS	POS	POS	POS	NEG	NEG	NEG	NEG	NEG	NEG	NEG
>"South-East Asian Clad																
M705 (Thailand)	POS	POS	NEG	NEG	POS	POS	POS	POS	POS	POS	POS	POS	NEG	NEG	NEG	NEG
S85 (Thailand)	POS	POS	NEG	NEG	POS	POS	POS	POS	POS	POS	POS	POS	NEG	NEG	NEG	NEG
Riyadh_Alfaisal-04_281014_S5076	POS	POS	NEG	NEG	POS	POS	POS	POS	POS	POS	POS	POS	NEG	NEG	NEG	NEG
Riyadh_Alfaisal-26_S15145_1104163	POS	POS	NEG	NEG	POS	POS	POS	POS	POS	POS	POS	POS	NEG	NEG	NEG	NEG
Riyadh-2817437-W	POS	POS	NEG	NEG	POS	POS	POS	POS	POS	POS	POS	POS	NEG	NEG	NEG	NEG
>"South-East Asian Clad																
PPUKM-775-2009 (Malaysia)	POS	POS	NEG	NEG	POS	POS	POS	POS	POS	POS	NEG	POS	NEG	NEG	NEG	NEG
Hong Kong_138	POS	POS	NEG	NEG	POS	POS	POS	POS	POS	POS	POS	POS	NEG	NEG	NEG	NEG
>"South-East Asian Clad																
Kuwait_001	POS	POS	NEG	NEG	POS	POS	POS	POS	POS	POS	NEG	POS	NEG	NEG	NEG	NEG
Kuwait_337	POS	POS	NEG	NEG	POS	POS	POS	POS	POS	NEG	NEG	NEG	NEG	NEG	NEG	NEG
>"South-East Asian Clad																
CUHK_HK2007 (China)	POS	POS	NEG	NEG	POS	POS	POS	POS	POS	POS	NEG	POS	NEG	NEG	NEG	NEG
NMR02 (India)	POS	POS	NEG	NEG	POS	POS	POS	POS	POS	POS	NEG	POS	NEG	NEG	NEG	NEG
VB1490 (India)	POS	POS	NEG	NEG	POS	POS	POS	POS	POS	POS	NEG	POS	NEG	NEG	NEG	NEG
V521 (Korea)	POS	POS	NEG	NEG	POS	POS	POS	POS	POS	POS	NEG	POS	NEG	NEG	NEG	NEG
HST-077 (Lebanon)	POS	POS	NEG	NEG	POS	POS	POS	POS	POS	POS	NEG	POS	NEG	NEG	NEG	NEG
MAL1 (Malaysia)	POS	POS	NEG	NEG	POS	POS	POS	POS	POS	POS	NEG	POS	NEG	NEG	NEG	NEG
MAL3 (Malaysia)	POS	POS	NEG	NEG	POS	POS	POS	POS	POS	POS	NEG	POS	NEG	NEG	NEG	NEG
PPUKM-261-2009 (Malaysia)	POS	POS	NEG	NEG	POS	POS	POS	POS	POS	POS	NEG	POS	NEG	NEG	NEG	NEG
PPUKM-332-2009 (Malaysia)	POS	POS	NEG	NEG	POS	POS	POS	POS	POS	POS	NEG	POS	NEG	NEG	NEG	NEG
S38 (Thailand)	POS	POS	NEG	NEG	POS	POS	POS	POS	POS	POS	NEG	POS	NEG	NEG	NEG	NEG
S7 (Thailand)	POS	POS	NEG	NEG	POS	POS	POS	POS	POS	POS	NEG	POS	NEG	NEG	NEG	NEG
US_008 (Thailand)	POS	POS	NEG	NEG	POS	POS	POS	POS	POS	POS	NEG	POS	NEG	NEG	NEG	NEG
US_030 (Thailand)	POS	POS	NEG	NEG	POS	POS	POS	POS	POS	POS	POS	POS	NEG	NEG	NEG	NEG
M170 (Tsunami-related)	POS	POS	NEG	NEG	POS	POS	POS	POS	POS	POS	POS	POS	NEG	NEG	NEG	NEG
BSAC697 (UK)	POS	POS	NEG	NEG	POS	POS	POS	POS	POS	POS	NEG	POS	NEG	NEG	NEG	NEG
IS-189	POS	POS	NEG	NEG	POS	POS	POS	POS	POS	POS	NEG	POS	NEG	NEG	NEG	NEG
MRGR3	POS	POS	NEG	NEG	POS	POS	POS	POS	POS	POS	NEG	POS	NEG	NEG	NEG	NEG
smg07	POS	POS	NEG	NEG	POS	POS	POS	POS	POS	POS	NEG	POS	NEG	NEG	NEG	NEG
AH1-AUS-EMRSA-3	POS	POS	NEG	NEG	POS	POS	POS	POS	POS	POS	NEG	POS	NEG	NEG	NEG	NEG
Bengalaru_ST20141403	POS	POS	NEG	NEG	POS	POS	POS	POS	POS	POS	NEG	POS	NEG	NEG	NEG	NEG
Finland E24_98541_Harmony	POS	POS	NEG	NEG	POS	POS	POS	POS	POS	POS	POS	POS	NEG	NEG	NEG	NEG
Hong Kong_10	POS	POS	NEG	NEG	POS	POS	POS	POS	POS	POS	NEG	POS	NEG	NEG	NEG	NEG
Hong Kong_100	POS	POS	NEG	NEG	POS	POS	POS	POS	POS	NEG	NEG	NEG	NEG	NEG	NEG	NEG
Hong Kong_199	POS	POS	NEG	NEG	POS	POS	POS	POS	POS	POS	NEG	POS	NEG	NEG	NEG	NEG
Hong Kong_34	POS	POS	NEG	NEG	POS	POS	POS	POS	POS	POS	NEG	POS	NEG	NEG	NEG	NEG
Hong Kong_88	POS	POS	NEG	NEG	POS	POS	POS	POS	POS	POS	NEG	POS	NEG	NEG	NEG	NEG
Hong Kong_92	POS	POS	NEG	NEG	POS	POS	POS	POS	POS	POS	NEG	POS	NEG	NEG	NEG	NEG
Hong Kong_94	POS	POS	NEG	NEG	POS	POS	POS	POS	POS	POS	NEG	POS	NEG	NEG	NEG	NEG
Kuwait_257	POS	POS	NEG	NEG	POS	POS	POS	POS	POS	POS	NEG	POS	NEG	NEG	NEG	NEG
Perth_01-15330	POS	POS	NEG	NEG	POS	POS	POS	POS	POS	POS	NEG	POS	NEG	NEG	NEG	NEG
Perth_01-15349	AMB	POS	NEG	NEG	POS	POS	POS	POS	POS	POS	NEG	POS	NEG	NEG	NEG	NEG
Perth_01-16176	POS	POS	NEG	NEG	POS	POS	POS	POS	POS	POS	NEG	POS	NEG	NEG	NEG	NEG
Perth_WBG8379	POS	POS	NEG	NEG	POS	POS	POS	POS	POS	POS	NEG	POS	NEG	NEG	NEG	NEG
Rawalpindi_Kidney Center_56	POS	POS	NEG	NEG	POS	POS	POS	POS	POS	NEG	NEG	NEG	NEG	NEG	NEG	NEG
Riyadh_SSTI_05_3464614	POS	POS	NEG	NEG	POS	POS	POS	POS	POS	POS	POS	POS	NEG	NEG	NEG	NEG
Trinidad&Tobago_2012_157	POS	POS	NEG	NEG	POS	POS	POS	POS	POS	POS	NEG	POS	NEG	NEG	NEG	NEG

STRAIN / ISOLATE	VIRULENCE : HLG AND LEUKOCIDINS				VIRULENCE : HAEMOLYSINS					VIRULENCE : HLB-CONV PHAGES			VIRULENCE : OTHER FACTORS			
	lukX	lukY			corB	hla	hlIII	hlIII	hlb	sak	chp	scn	etA	etB	etD	etD2
	lukX	lukY	lukY (ST30+ST45)	lukY (ST1850)	corB (=hl)	hla	hlIII (cons)	hlIII (other than RF122)	hlb-probe 1	sak	chp	scn	etA	etB	etD	etD2
	leukocidin/ haemolysin toxin family protein	leukocidin/haemolysin toxin family protein			Putative membrane protein	Haemolysin alpha	Putative membrane protein		haemolysin beta	staphylo-kinase	chemotaxis-inhibiting protein (CHIPS)	Staphyl. Comple-ment inhibitor	exfoliative toxin serotype A	exfoliative toxin serotype B	exfoliative toxin D	exfoliative toxin D2
>"South-East Asian Clad																
M418	POS	POS	NEG	NEG	POS	POS	POS	POS	POS	POS	NEG	POS	NEG	NEG	NEG	NEG
>"South-East Asian Clad																
NMR07 (India)	POS	POS	NEG	NEG	POS	POS	POS	POS	POS	POS	NEG	POS	NEG	NEG	NEG	NEG
NMR08 (India)	POS	POS	NEG	NEG	POS	POS	POS	POS	POS	POS	NEG	POS	NEG	NEG	NEG	NEG
Bengalaru_ST20121978	POS	POS	NEG	NEG	POS	POS	POS	POS	POS	POS	NEG	POS	NEG	NEG	NEG	NEG
Hong Kong_89	POS	POS	NEG	NEG	POS	POS	POS	POS	POS	POS	NEG	POS	NEG	NEG	NEG	NEG
>"South-East Asian Clad																
Perth_2005_AH_5	POS	POS	NEG	NEG	POS	POS	POS	POS	POS	POS	NEG	POS	NEG	NEG	NEG	NEG
>"South-East Asian Clad																
Trinidad&Tobago_MRSA2010_103	POS	POS	NEG	NEG	POS	POS	POS	POS	POS	NEG	NEG	NEG	NEG	NEG	NEG	NEG
Trinidad&Tobago_2013_PA25	POS	POS	NEG	NEG	POS	POS	POS	POS	POS	NEG	NEG	NEG	NEG	NEG	NEG	NEG
Trinidad&Tobago_MRSA2010_110	POS	POS	NEG	NEG	POS	POS	POS	POS	POS	NEG	NEG	AMB	NEG	NEG	NEG	NEG
Trinidad&Tobago_SSTI 2012_111	POS	POS	NEG	NEG	POS	POS	POS	POS	POS	NEG	NEG	NEG	NEG	NEG	NEG	NEG
Trinidad&Tobago_MRSA2010_158	POS	POS	NEG	NEG	POS	POS	POS	POS	POS	NEG	NEG	NEG	NEG	NEG	NEG	NEG
>"South-East Asian Clad																
Kuwait_122	POS	POS	NEG	NEG	POS	POS	POS	POS	POS	POS	NEG	POS	NEG	NEG	NEG	NEG
Kuwait_002	POS	POS	NEG	NEG	POS	POS	POS	POS	POS	POS	NEG	POS	NEG	NEG	NEG	NEG
Kuwait_196	POS	POS	NEG	NEG	POS	POS	POS	POS	POS	POS	POS	POS	NEG	NEG	NEG	NEG
Kuwait_199	POS	POS	NEG	NEG	POS	POS	POS	POS	POS	POS	POS	POS	NEG	NEG	NEG	NEG
Hong Kong_136	POS	POS	NEG	NEG	POS	POS	POS	POS	POS	NEG	NEG	NEG	NEG	NEG	NEG	NEG
>"South-East Asian Clad																
P32 (Poland)	POS	POS	NEG	NEG	POS	POS	POS	POS	POS	NEG	NEG	NEG	NEG	NEG	NEG	NEG
>"South-East Asian Clad																
Perth_2015-S32313	POS	POS	NEG	NEG	POS	POS	POS	POS	POS	POS	NEG	POS	NEG	NEG	NEG	NEG
>"hla-negative South-Ea																
DEN907 (Denmark)	POS	POS	NEG	NEG	POS	NEG	POS	POS	POS	POS	NEG	POS	NEG	NEG	NEG	NEG
>"hla-negative South-Ea																
S26 (Thailand)	POS	POS	NEG	NEG	POS	NEG	POS	POS	POS	POS	NEG	POS	NEG	NEG	NEG	NEG
S42 (Thailand)	POS	POS	NEG	NEG	POS	NEG	POS	POS	POS	POS	NEG	POS	NEG	NEG	NEG	NEG
S97 (Thailand)	POS	POS	NEG	NEG	POS	NEG	POS	POS	POS	POS	NEG	POS	NEG	NEG	NEG	NEG
S78 (Thailand)	POS	POS	NEG	NEG	POS	NEG	POS	POS	POS	POS	NEG	POS	NEG	NEG	NEG	NEG
S25 (Thailand)	POS	POS	NEG	NEG	POS	NEG	POS	POS	POS	POS	NEG	POS	NEG	NEG	NEG	NEG
S21 (Thailand)	POS	POS	NEG	NEG	POS	NEG	POS	POS	POS	POS	NEG	POS	NEG	NEG	NEG	NEG
S106 (Thailand)	POS	POS	NEG	NEG	POS	NEG	POS	POS	POS	POS	NEG	POS	NEG	NEG	NEG	NEG
S24 (Thailand)	POS	POS	NEG	NEG	POS	NEG	POS	POS	POS	POS	NEG	POS	NEG	NEG	NEG	NEG
S39 (Thailand)	POS	POS	NEG	NEG	POS	NEG	POS	POS	POS	POS	NEG	POS	NEG	NEG	NEG	NEG
S2 (Thailand)	POS	POS	NEG	NEG	POS	NEG	POS	POS	POS	POS	NEG	POS	NEG	NEG	NEG	NEG
S81 (Thailand)	POS	POS	NEG	NEG	POS	NEG	POS	POS	POS	POS	NEG	POS	NEG	NEG	NEG	NEG
M116 (Vietnam)	POS	POS	NEG	NEG	POS	NEG	POS	POS	POS	POS	NEG	POS	NEG	NEG	NEG	NEG
>"Portuguese Clade": Pc																
FFP103 (Portugal)	POS	POS	NEG	NEG	POS	POS	POS	POS	POS	NEG	NEG	NEG	NEG	NEG	NEG	NEG
HDG2 (Portugal)	POS	POS	NEG	NEG	POS	POS	POS	POS	POS	POS	POS	POS	NEG	NEG	NEG	NEG
HSA10 (Portugal)	POS	POS	NEG	NEG	POS	POS	POS	POS	POS	NEG	NEG	NEG	NEG	NEG	NEG	NEG
HSA11 (Portugal)	POS	POS	NEG	NEG	POS	POS	POS	POS	POS	NEG	NEG	NEG	NEG	NEG	NEG	NEG
ICP5011 (Portugal)	POS	POS	NEG	NEG	POS	POS	POS	POS	POS	NEG	NEG	NEG	NEG	NEG	NEG	NEG
ICP5014 (Portugal)	POS	POS	NEG	NEG	POS	POS	POS	POS	POS	NEG	NEG	NEG	NEG	NEG	NEG	NEG
ICP5062 (Portugal)	POS	POS	NEG	NEG	POS	POS	POS	POS	POS	NEG	NEG	NEG	NEG	NEG	NEG	NEG
Strain 1063_ATCC 33592 FDAARGOS_3 (USA)	POS	POS	NEG	NEG	POS	POS	POS	POS	POS	POS	NEG	POS	NEG	NEG	NEG	NEG
	POS	POS	NEG	NEG	POS	POS	POS	POS	POS	POS	NEG	POS	NEG	NEG	NEG	NEG
Russia-07_0110_Kurgan	POS	POS	NEG	NEG	POS	POS	POS	POS	POS	POS	NEG	POS	NEG	NEG	NEG	NEG
Russia-11_0164_Kurgan	POS	POS	NEG	NEG	POS	POS	POS	POS	POS	NEG	NEG	NEG	NEG	NEG	NEG	NEG
Russia-20_0367_Kurgan	POS	POS	NEG	NEG	POS	POS	POS	POS	POS	POS	NEG	POS	NEG	NEG	NEG	NEG
Russia-21_0390_Chelyabinsk	POS	POS	NEG	NEG	POS	POS	POS	POS	POS	POS	NEG	POS	NEG	NEG	NEG	NEG
>"South American/ Mid																
AGT1 (Argentina)	POS	POS	NEG	NEG	POS	POS	POS	POS	POS	POS	POS	POS	NEG	NEG	NEG	NEG
AGT120 (Argentina)	POS	POS	NEG	NEG	POS	POS	POS	POS	POS	POS	POS	POS	NEG	NEG	NEG	NEG
AGT67 (Argentina)	POS	POS	NEG	NEG	POS	POS	POS	POS	POS	NEG	NEG	NEG	NEG	NEG	NEG	NEG
AGT9 (Argentina)	POS	POS	NEG	NEG	POS	POS	POS	POS	POS	POS	POS	POS	NEG	NEG	NEG	NEG
RA3 (Argentina)	POS	POS	NEG	NEG	POS	POS	POS	POS	POS	POS	POS	POS	NEG	NEG	NEG	NEG
RA6 (Argentina)	POS	POS	NEG	NEG	POS	POS	POS	POS	POS	POS	POS	POS	NEG	NEG	NEG	NEG
RA7 (Argentina)	POS	POS	NEG	NEG	POS	POS	POS	POS	POS	POS	POS	POS	NEG	NEG	NEG	NEG
Be62 (Brazil)	POS	POS	NEG	NEG	POS	POS	POS	POS	POS	POS	POS	POS	NEG	NEG	NEG	NEG
Bmb9393 (Brazil)	POS	POS	NEG	NEG	POS	POS	POS	POS	POS	POS	POS	POS	NEG	NEG	NEG	NEG
BRA36 (Brazil)	POS	POS	NEG	NEG	POS	POS	POS	POS	POS	POS	POS	POS	NEG	NEG	NEG	NEG
BZ48 (Brazil)	POS	POS	NEG	NEG	POS	POS	POS	POS	POS	POS	POS	POS	NEG	NEG	NEG	NEG
Gv51 (Brazil)	POS	POS	NEG	NEG	POS	POS	POS	POS	POS	POS	POS	POS	NEG	NEG	NEG	NEG
Gv69 (Brazil)	POS	POS	NEG	NEG	POS	POS	POS	POS	POS	POS	POS	POS	NEG	NEG	NEG	NEG
Gv88 (Brazil)	POS	POS	NEG	NEG	POS	POS	POS	POS	POS	POS	POS	POS	NEG	NEG	NEG	NEG
HC1335 (Brazil)	POS	POS	NEG	NEG	POS	POS	POS	POS	POS	POS	POS	POS	NEG	NEG	NEG	NEG
HC1340 (Brazil)	POS	POS	NEG	NEG	POS	POS	POS	POS	POS	POS	POS	POS	NEG	NEG	NEG	NEG
HC556 (Brazil)	POS	POS	NEG	NEG	POS	POS	POS	POS	POS	POS	POS	POS	NEG	NEG	NEG	NEG
HU25 (Brazil)	POS	POS	NEG	NEG	POS	POS	POS	POS	POS	POS	POS	POS	NEG	NEG	NEG	NEG
UB563 (Brazil)	POS	POS	NEG	NEG	POS	POS	POS	POS	POS	POS	POS	POS	NEG	NEG	NEG	NEG
2A8 (Czech Republic)	POS	POS	NEG	NEG	POS	POS	POS	POS	POS	POS	POS	POS	NEG	NEG	NEG	NEG
M1229 (Denmark)	POS	POS	NEG	NEG	POS	POS	POS	POS	POS	NEG	NEG	NEG	NEG	NEG	NEG	NEG
FRICAR (France)	POS	POS	NEG	NEG	POS	POS	POS	POS	POS	POS	POS	POS	NEG	NEG	NEG	NEG
D90 (Germany)	POS	POS	NEG	NEG	POS	POS	POS	POS	POS	POS	POS	POS	NEG	NEG	NEG	NEG
LIT68 (Lithuania)	POS	POS	NEG	NEG	POS	POS	POS	POS	POS	POS	NEG	POS	NEG	NEG	NEG	NEG
LIT76 (Lithuania)	POS	POS	NEG	NEG	POS	POS	POS	POS	POS	POS	NEG	POS	NEG	NEG	NEG	NEG
HGSA142 (Portugal)	POS	POS	NEG	NEG	POS	POS	POS	POS	POS	POS	POS	POS	NEG	NEG	NEG	NEG
HGSA9 (Portugal)	POS	POS	NEG	NEG	POS	POS	POS	POS	POS	POS	POS	POS	NEG	NEG	NEG	NEG
HSJ216 (Portugal)	POS	POS	NEG	NEG	POS	POS	POS	POS	POS	POS	POS	POS	NEG	NEG	NEG	NEG
M278 (Portugal)	POS	POS	NEG	NEG	POS	POS	POS	POS	POS	POS	POS	POS	NEG	NEG	NEG	NEG
ES26 (Spain)	POS	POS	NEG	NEG	POS	POS	POS	POS	POS	POS	POS	POS	NEG	NEG	NEG	NEG
UK102 (UK)	POS	POS	NEG	NEG	POS	POS	POS	POS	POS	POS	POS	POS	NEG	NEG	NEG	NEG
URU34 (Uruguay)	POS	POS	NEG	NEG	POS	POS	POS	POS	POS	POS	POS	POS	NEG	NEG	NEG	NEG
IS-125	POS	POS	NEG	NEG	POS	POS	POS	POS	POS	POS	NEG	POS	NEG	NEG	NEG	NEG
IS-157	POS	POS	NEG	NEG	POS	POS	POS	POS	POS	POS	NEG	POS	NEG	NEG	NEG	NEG
Ecuador_1	POS	POS	NEG	NEG	POS	NEG	POS	POS	POS	POS	POS	POS	NEG	NEG	NEG	NEG
Ecuador_2	POS	POS	NEG	NEG	POS	POS	POS	POS	POS	POS	POS	POS	NEG	NEG	NEG	NEG
Dresden_15ANR574263	POS	POS	NEG	NEG	POS	POS	POS	POS	POS	POS	NEG	POS	NEG	NEG	NEG	NEG
UK-EMRSA-11	POS	POS	NEG	NEG	POS	POS	POS	POS	POS	POS	NEG	POS	NEG	NEG	NEG	NEG
>"South American/ Mid																
BRA2 (Brazil)	POS	POS	NEG	NEG	POS	POS	POS	POS	POS	POS	POS	POS	NEG	NEG	NEG	NEG
CHL1 (Chile)	POS	POS	NEG	NEG	POS	POS	POS	POS	POS	POS	POS	POS	NEG	NEG	NEG	NEG
CHL151 (Chile)	POS	POS	NEG	NEG	POS	POS	POS	POS	POS	POS	POS	POS	NEG	NEG	NEG	NEG
UCL378 (Chile)	POS	POS	NEG	NEG	POS	POS	POS	POS	POS	NEG	NEG	NEG	NEG	NEG	NEG	NEG
UP72 (Peru)	POS	POS	NEG	NEG	POS	POS	POS	POS	POS	POS	POS	POS	NEG	NEG	NEG	NEG
UP81 (Peru)	POS	POS	NEG	NEG	POS	POS	POS	POS	POS	POS	POS	POS	NEG	NEG	NEG	NEG
>"South American/ Mid																
UP1073 (Peru)	POS	POS	NEG	NEG	POS	POS	POS	POS	POS	POS	POS	POS	NEG	NEG	NEG	NEG

STRAIN / ISOLATE	VIRULENCE : HLG AND LEUKOCIDINS				VIRULENCE : HAEMOLYSINS					VIRULENCE : HLB-CONV PHAGES			VIRULENCE : OTHER FACTORS			
	lukX	lukY			corB	hla	hlIII	hlIII	hlb	sak	chp	scn	etA	etB	etD	etD2
	lukX	lukY	lukY (ST30+ST45)	lukY (ST1850)	corB (=hl)	hla	hlIII (cons)	hlIII (other than RF122)	hlb-probe 1	sak	chp	scn	etA	etB	etD	etD2
	leukocidin/ haemolysin toxin family protein	leukocidin/haemolysin toxin family protein			Putative membrane protein	Haemolysin alpha	Putative membrane protein		haemolysin beta	staphylo-kinase	chemotaxis-inhibiting protein (CHIPS)	Staphyl. Comple-ment inhibitor	exfoliative toxin serotype A	exfoliative toxin serotype B	exfoliative toxin D	exfoliative toxin D2
>"South American/ Mid																
LIT89 (Lithuania)	POS	POS	NEG	NEG	POS	POS	POS	POS	POS	POS	NEG	POS	NEG	NEG	NEG	NEG
MRSA_PR1 (Malaysia)	POS	POS	NEG	NEG	POS	POS	POS	POS	POS	POS	NEG	POS	NEG	NEG	NEG	NEG
Riyadh-2888905-R	POS	POS	NEG	NEG	POS	POS	POS	POS	POS	POS	POS	POS	NEG	NEG	NEG	NEG
Riyadh-2888915-BC	POS	POS	NEG	NEG	POS	POS	POS	POS	POS	POS	POS	POS	NEG	NEG	NEG	NEG
Riyadh-2793706-R	POS	POS	NEG	NEG	POS	POS	POS	POS	POS	POS	POS	POS	NEG	NEG	NEG	NEG
>"South American/ Mid																
Riyadh_SSTI_18_3502925	POS	POS	NEG	NEG	POS	POS	POS	POS	POS	POS	POS	POS	NEG	NEG	NEG	NEG
Riyadh-2822825-W	POS	POS	NEG	NEG	POS	POS	POS	POS	POS	POS	POS	POS	NEG	NEG	NEG	NEG
>"South American/ Mid																
UK-EMRSA-9	POS	POS	NEG	NEG	POS	POS	POS	POS	POS	POS	NEG	POS	NEG	NEG	NEG	NEG
>"South American/ Mid																
Lome_HT20020815	POS	POS	NEG	NEG	POS	POS	POS	POS	POS	POS	NEG	POS	NEG	NEG	NEG	NEG
>"South American/ Mid																
NA32 (Denmark)	POS	POS	NEG	NEG	POS	POS	POS	POS	POS	POS	NEG	POS	NEG	NEG	NEG	NEG
H24 (Egypt)	POS	POS	NEG	NEG	POS	POS	POS	POS	POS	POS	NEG	POS	NEG	NEG	NEG	NEG
ATCC BAA-39 (=HUSA304) (Hungary)	POS	POS	NEG	NEG	POS	POS	POS	POS	POS	POS	NEG	POS	NEG	NEG	NEG	NEG
HUSA304 (Hungary)	POS	POS	NEG	NEG	POS	POS	POS	POS	POS	POS	NEG	POS	NEG	NEG	NEG	NEG
HU106 (Hungary)	POS	POS	NEG	NEG	POS	POS	POS	POS	POS	POS	NEG	POS	NEG	NEG	NEG	NEG
BSAC27 (UK)	POS	POS	NEG	NEG	POS	POS	POS	POS	POS	POS	POS	POS	NEG	NEG	NEG	NEG
NCTC13131, UK-EMRSA-4 (UK)	POS	POS	NEG	NEG	POS	POS	POS	POS	POS	POS	NEG	POS	NEG	NEG	NEG	NEG
	POS	POS	NEG	NEG	POS	POS	POS	POS	POS	POS	NEG	POS	NEG	NEG	NEG	NEG
Algiers_HT20040080	POS	POS	NEG	NEG	POS	POS	POS	POS	POS	POS	POS	POS	NEG	NEG	NEG	NEG
Dublin-DSH_AR09_0.0066	POS	POS	NEG	NEG	POS	POS	POS	POS	POS	POS	NEG	POS	NEG	NEG	NEG	NEG
Dublin-DSH_AR09_0-0065	NEG	POS	NEG	NEG	POS	POS	POS	POS	POS	POS	NEG	POS	NEG	NEG	NEG	NEG
Dublin-DSH_Phenotype-III_84	AMB	POS	NEG	NEG	POS	POS	POS	POS	POS	POS	NEG	POS	NEG	NEG	NEG	NEG
Hong Kong_130	POS	POS	NEG	NEG	POS	POS	POS	POS	POS	POS	NEG	POS	NEG	NEG	NEG	NEG
Kuwait_018	POS	POS	NEG	NEG	POS	POS	POS	POS	POS	POS	POS	POS	NEG	NEG	NEG	NEG
Perth_08-17726	POS	POS	NEG	NEG	POS	POS	POS	POS	POS	POS	POS	POS	NEG	NEG	NEG	NEG
Riyadh_Alfaisal/KKKSUH_86_MRS-14-279	POS	POS	NEG	NEG	POS	POS	POS	POS	POS	POS	POS	POS	NEG	NEG	NEG	NEG
Riyadh_Alfaisal-04_Z3861_831588	POS	POS	NEG	NEG	POS	POS	POS	POS	POS	POS	POS	POS	NEG	NEG	NEG	NEG
Riyadh_Alfaisal-30_S15724_1108013	POS	POS	NEG	NEG	POS	POS	POS	POS	POS	POS	POS	POS	NEG	NEG	NEG	NEG
Riyadh_Alfaisal-6_22A_13_B3992_397721	POS	POS	NEG	NEG	POS	POS	POS	POS	POS	POS	POS	POS	NEG	NEG	NEG	NEG
Riyadh_SSTI_52_3615482	POS	POS	NEG	NEG	POS	POS	POS	POS	POS	NEG	NEG	NEG	NEG	NEG	NEG	NEG
Riyadh-2817276-2	POS	POS	NEG	NEG	POS	POS	POS	POS	POS	POS	POS	POS	NEG	NEG	NEG	NEG
Riyadh-2891670-W	POS	POS	NEG	NEG	POS	POS	POS	POS	POS	POS	POS	POS	NEG	NEG	NEG	NEG
Riyadh-3006920-W	POS	POS	NEG	NEG	POS	POS	POS	POS	POS	POS	POS	POS	NEG	NEG	NEG	NEG
Riyadh-R2567782	POS	POS	NEG	NEG	POS	POS	POS	POS	POS	POS	POS	POS	NEG	NEG	NEG	NEG
Russia-18_0252_Krasnoyarsk_SK2	POS	POS	NEG	NEG	POS	POS	POS	POS	POS	POS	NEG	POS	NEG	NEG	NEG	NEG
UK-EMRSA-7	POS	POS	NEG	NEG	POS	POS	POS	POS	POS	POS	NEG	POS	NEG	NEG	NEG	NEG
>"South American/ Mid																
Dublin-DSH_AR23_0073	AMB	POS	NEG	NEG	POS	POS	POS	POS	POS	POS	NEG	POS	NEG	NEG	NEG	NEG
>Related to "South Ame																
MRSA-OC3 (Russia)	POS	POS	NEG	NEG	POS	POS	POS	POS	POS	POS	NEG	POS	NEG	NEG	NEG	NEG
Russia-12_0176_Krasnoyarsk	POS	POS	NEG	NEG	POS	POS	POS	POS	POS	POS	NEG	POS	NEG	NEG	NEG	NEG
Russia-13_0180_Krasnoyarsk	POS	POS	NEG	NEG	POS	POS	POS	POS	POS	POS	NEG	POS	NEG	NEG	NEG	NEG
Russia-16_0249_Krasnoyarsk	POS	POS	NEG	NEG	POS	POS	POS	POS	POS	POS	NEG	POS	NEG	NEG	NEG	NEG
Russia-17_0250_Krasnoyarsk	POS	POS	NEG	NEG	POS	POS	POS	POS	POS	POS	NEG	POS	NEG	NEG	NEG	NEG
>Related to "South Ame																
URU110 (Uruguay)	POS	POS	NEG	NEG	POS	POS	POS	POS	POS	POS	NEG	POS	NEG	NEG	NEG	NEG
>Related to "South Ame																
DS_014 (Thailand)	POS	POS	NEG	NEG	POS	POS	POS	POS	POS	POS	POS	POS	NEG	NEG	NEG	NEG
>Unassigned Middle Eas																
Frankfurt_Oder_0490031797 (Libya)	POS	POS	NEG	NEG	POS	NEG	POS	POS	POS	POS	NEG	POS	NEG	NEG	NEG	NEG
Kuwait_192	POS	POS	NEG	NEG	POS	NEG	POS	POS	POS	POS	NEG	POS	NEG	NEG	NEG	NEG
Riyadh-9	POS	POS	NEG	NEG	POS	NEG	POS	POS	POS	POS	NEG	POS	NEG	NEG	NEG	NEG
Russia-24_0407_Moscow	POS	POS	NEG	NEG	POS	NEG	POS	POS	POS	POS	NEG	POS	NEG	NEG	NEG	NEG

STRAIN / ISOLATE	VIRULENCE : OTHER FACTORS					VIRULENCE : PROTEASES									
	edinA	edinB	edinC	esxA	esxB	aur			splA	splB	splE	sspA	sspB	sspP	
	edinA	edinB	edinC	esxA	esxB	aur (cons)	aur (Other than MRSA252)	aur (MRSA252)	splA	splB	splE	sspA	sspB	sspP (cons)	sspP (other than ST93)
	epidermal cell differen-tiation inhibitor	epidermal cell differen-tiation inhibitor B	epidermal cell differen-tiation inhibitor C	virulence factor esxA	virulence factor esxB	aureolysin			serin- protease A	serin- protease B	serin- protease E	glutamylendopeptid ase	Staphopain B, protease	Staphopain A (Staphylopain A), protease	
>"Eurasian Clade": TUR1															
TUR1 (Turkey)	NEG	NEG	NEG	POS	NEG	POS	NEG	POS	POS	POS	NEG	POS	POS	POS	POS
TUR9 (Turkey)	NEG	NEG	NEG	POS	NEG	POS	NEG	POS	POS	POS	NEG	POS	POS	POS	POS
>"Eurasian Clade" : T013															
T0131 (China)	NEG	NEG	NEG	POS	NEG	POS	NEG	POS	POS	POS	NEG	POS	POS	POS	POS
MU4 (Turkey)	NEG	NEG	NEG	POS	NEG	POS	NEG	POS	POS	POS	NEG	POS	POS	POS	POS
DEU11 (Turkey)	NEG	NEG	NEG	POS	NEG	POS	NEG	POS	POS	POS	NEG	POS	POS	POS	POS
DEU16 (Turkey)	NEG	NEG	NEG	POS	NEG	POS	NEG	POS	POS	POS	NEG	POS	POS	POS	POS
Romania_Iasi_MRSA-07_54	NEG	NEG	NEG	POS	NEG	POS	NEG	POS	POS	POS	NEG	POS	POS	POS	POS
>"Eurasian Clade": arsC-															
Romania_Iasi_MRSA-05_46	NEG	NEG	NEG	POS	NEG	POS	NEG	POS	POS	POS	NEG	POS	POS	POS	POS
Romania_Iasi_MRSA-06_47	NEG	NEG	NEG	POS	NEG	POS	NEG	POS	POS	POS	NEG	POS	POS	POS	POS
>"Eurasian Clade": 16K/(															
CN79 (China)	NEG	NEG	NEG	POS	NEG	POS	NEG	POS	POS	POS	NEG	POS	POS	POS	POS
CUHK_BJ2002 (China)	NEG	NEG	NEG	POS	NEG	POS	NEG	POS	POS	POS	NEG	POS	POS	POS	POS
CUHK_BJ2007 (China)	NEG	NEG	NEG	POS	NEG	POS	NEG	POS	POS	POS	NEG	POS	POS	POS	POS
3HK (Czech Republic)	NEG	NEG	NEG	POS	NEG	POS	NEG	POS	POS	POS	NEG	POS	POS	POS	POS
HU109 (Hungary)	NEG	NEG	NEG	POS	NEG	POS	NEG	POS	POS	POS	NEG	POS	POS	POS	POS
HUR18 (Hungary)	NEG	NEG	NEG	POS	NEG	POS	NEG	POS	POS	POS	NEG	POS	POS	POS	POS
H482 (Romania)	NEG	NEG	NEG	POS	NEG	POS	NEG	POS	POS	POS	NEG	POS	POS	POS	POS
16K (Russia)	NEG	NEG	NEG	POS	NEG	POS	NEG	POS	POS	POS	NEG	POS	POS	POS	POS
DEU10 (Turkey)	NEG	NEG	NEG	POS	NEG	POS	NEG	POS	POS	POS	NEG	POS	POS	POS	POS
DEU12 (Turkey)	NEG	NEG	NEG	POS	NEG	POS	NEG	POS	POS	POS	NEG	POS	POS	POS	POS
DEU14 (Turkey)	NEG	NEG	NEG	POS	NEG	POS	NEG	POS	POS	POS	NEG	POS	POS	POS	POS
DEU15 (Turkey)	NEG	NEG	NEG	POS	NEG	POS	NEG	POS	POS	POS	NEG	POS	POS	POS	POS
DEU17 (Turkey)	NEG	NEG	NEG	POS	NEG	POS	NEG	POS	POS	POS	NEG	POS	POS	POS	POS
DEU19 (Turkey)	NEG	NEG	NEG	POS	NEG	POS	NEG	POS	POS	POS	NEG	POS	POS	POS	POS
DEU2 (Turkey)	NEG	NEG	NEG	POS	NEG	POS	NEG	POS	POS	POS	NEG	POS	POS	POS	POS
DEU20 (Turkey)	NEG	NEG	NEG	POS	NEG	POS	NEG	POS	POS	POS	NEG	POS	POS	POS	POS
DEU23 (Turkey)	NEG	NEG	NEG	POS	NEG	POS	NEG	POS	POS	POS	NEG	POS	POS	POS	POS
DEU3 (Turkey)	NEG	NEG	NEG	POS	NEG	POS	NEG	POS	POS	POS	NEG	POS	POS	POS	POS
DEU5 (Turkey)	NEG	NEG	NEG	POS	NEG	POS	NEG	POS	POS	POS	NEG	POS	POS	POS	POS
DEU6 (Turkey)	NEG	NEG	NEG	POS	NEG	POS	NEG	POS	POS	POS	NEG	POS	POS	POS	POS
DEU8 (Turkey)	NEG	NEG	NEG	POS	NEG	POS	NEG	POS	POS	POS	NEG	POS	POS	POS	POS
HU11 (Turkey)	NEG	NEG	NEG	POS	NEG	POS	NEG	POS	POS	POS	NEG	POS	POS	POS	POS
HU13 (Turkey)	NEG	NEG	NEG	POS	NEG	POS	NEG	POS	POS	POS	NEG	POS	POS	POS	POS
HU14 (Turkey)	NEG	NEG	NEG	POS	NEG	POS	NEG	POS	POS	POS	NEG	POS	POS	POS	POS
HU15 (Turkey)	NEG	NEG	NEG	POS	NEG	POS	NEG	POS	POS	POS	NEG	POS	POS	POS	POS
HU16 (Turkey)	NEG	NEG	NEG	POS	NEG	POS	NEG	POS	POS	POS	NEG	POS	POS	POS	POS
HU17 (Turkey)	NEG	NEG	NEG	POS	NEG	POS	NEG	POS	POS	POS	NEG	POS	POS	POS	POS
HU21 (Turkey)	NEG	NEG	NEG	POS	NEG	POS	NEG	POS	POS	POS	NEG	POS	POS	POS	POS
HU23 (Turkey)	NEG	NEG	NEG	POS	NEG	POS	NEG	POS	POS	POS	NEG	POS	POS	POS	POS
HU26 (Turkey)	NEG	NEG	NEG	POS	NEG	POS	NEG	POS	POS	POS	NEG	POS	POS	POS	POS
HU4 (Turkey)	NEG	NEG	NEG	POS	NEG	POS	NEG	POS	POS	POS	NEG	POS	POS	POS	POS
HU5 (Turkey)	NEG	NEG	NEG	POS	NEG	POS	NEG	POS	POS	POS	NEG	POS	POS	POS	POS
HU6 (Turkey)	NEG	NEG	NEG	POS	NEG	POS	NEG	POS	POS	POS	NEG	POS	POS	POS	POS
HU7 (Turkey)	NEG	NEG	NEG	POS	NEG	POS	NEG	POS	POS	POS	NEG	POS	POS	POS	POS
HU8 (Turkey)	NEG	NEG	NEG	POS	NEG	POS	NEG	POS	POS	POS	NEG	POS	POS	POS	POS
HU9 (Turkey)	NEG	NEG	NEG	POS	NEG	POS	NEG	POS	POS	POS	NEG	POS	POS	POS	POS
IU1 (Turkey)	NEG	NEG	NEG	POS	NEG	POS	NEG	POS	POS	POS	NEG	POS	POS	POS	POS
IU10 (Turkey)	NEG	NEG	NEG	POS	NEG	POS	NEG	POS	POS	POS	NEG	POS	POS	POS	POS
IU11 (Turkey)	NEG	NEG	NEG	POS	NEG	POS	NEG	POS	POS	POS	NEG	POS	POS	POS	POS
IU12 (Turkey)	NEG	NEG	NEG	POS	NEG	POS	NEG	POS	POS	POS	NEG	POS	POS	POS	POS
IU13 (Turkey)	NEG	NEG	NEG	POS	NEG	POS	NEG	POS	POS	POS	NEG	POS	POS	POS	POS
IU15 (Turkey)	NEG	NEG	NEG	POS	NEG	POS	NEG	POS	POS	POS	NEG	POS	POS	POS	POS
IU18 (Turkey)	NEG	NEG	NEG	POS	NEG	POS	NEG	POS	POS	POS	NEG	POS	POS	POS	POS
IU19 (Turkey)	NEG	NEG	NEG	POS	NEG	POS	NEG	POS	POS	POS	NEG	POS	POS	POS	POS
IU2 (Turkey)	NEG	NEG	NEG	POS	NEG	POS	NEG	POS	POS	POS	NEG	POS	POS	POS	POS
IU4 (Turkey)	NEG	NEG	NEG	POS	NEG	POS	NEG	POS	POS	POS	NEG	POS	POS	POS	POS
IU6 (Turkey)	NEG	NEG	NEG	POS	NEG	POS	NEG	POS	POS	POS	NEG	POS	POS	POS	POS
IU7 (Turkey)	NEG	NEG	NEG	POS	NEG	POS	NEG	POS	POS	POS	NEG	POS	POS	POS	POS
IU9 (Turkey)	NEG	NEG	NEG	POS	NEG	POS	NEG	POS	POS	POS	NEG	POS	POS	POS	POS
MU1 (Turkey)	NEG	NEG	NEG	POS	NEG	POS	NEG	POS	POS	POS	NEG	POS	POS	POS	POS
MU10 (Turkey)	NEG	NEG	NEG	POS	NEG	POS	NEG	POS	POS	POS	NEG	POS	POS	POS	POS
MU20 (Turkey)	NEG	NEG	NEG	POS	NEG	POS	NEG	POS	POS	POS	NEG	POS	POS	POS	POS
Mu3 (Turkey)	NEG	NEG	NEG	POS	NEG	POS	NEG	POS	POS	POS	NEG	POS	POS	POS	POS
MU5 (Turkey)	NEG	NEG	NEG	POS	NEG	POS	NEG	POS	POS	POS	NEG	POS	POS	POS	POS
MU6 (Turkey)	NEG	NEG	NEG	POS	NEG	POS	NEG	POS	POS	POS	NEG	POS	POS	POS	POS
MU7 (Turkey)	NEG	NEG	NEG	POS	NEG	POS	NEG	POS	POS	POS	NEG	POS	POS	POS	POS
TUR27 (Turkey)	NEG	NEG	NEG	POS	NEG	POS	NEG	POS	POS	POS	NEG	POS	POS	POS	POS
NCTR 32S	NEG	NEG	NEG	POS	NEG	POS	NEG	POS	POS	POS	NEG	POS	POS	POS	POS
SA02_A4 = Russia-06_0085_Moscow	NEG	NEG	NEG	POS	NEG	POS	NEG	POS	POS	POS	NEG	POS	POS	POS	POS
	NEG	NEG	NEG	POS	NEG	POS	NEG	POS	POS	POS	NEG	POS	POS	POS	POS
Dresden_08V35987 (Turkey)	NEG	NEG	NEG	POS	NEG	POS	NEG	POS	POS	POS	NEG	POS	POS	POS	POS
Dresden_17ANR577152	NEG	NEG	NEG	POS	NEG	POS	NEG	POS	POS	POS	NEG	POS	POS	POS	POS
Dresden_17ANR580374 (Makedonia)	NEG	NEG	NEG	POS	NEG	POS	NEG	POS	POS	POS	NEG	POS	POS	POS	POS
Hong Kong_69-II	NEG	NEG	NEG	POS	NEG	POS	NEG	POS	POS	POS	NEG	POS	POS	POS	POS
Hong Kong_93	NEG	NEG	NEG	POS	NEG	POS	NEG	POS	POS	POS	NEG	POS	POS	POS	POS
Rawalpindi_Kidney Center_03_SK1	NEG	NEG	NEG	POS	NEG	POS	NEG	POS	POS	POS	NEG	POS	POS	POS	POS
Rawalpindi_Kidney Center_08	NEG	NEG	NEG	POS	NEG	POS	NEG	POS	POS	POS	NEG	POS	POS	POS	POS
Rawalpindi_Kidney Center_10	NEG	NEG	NEG	POS	NEG	POS	NEG	POS	POS	POS	NEG	POS	POS	POS	POS
Rawalpindi_Kidney Center_19	NEG	NEG	NEG	POS	NEG	POS	NEG	POS	POS	POS	NEG	POS	POS	POS	POS
Rawalpindi_Kidney Center_50_SK1	NEG	NEG	NEG	POS	NEG	POS	NEG	POS	POS	POS	NEG	POS	POS	POS	POS
Romania_Iasi_BC-49_430	NEG	NEG	NEG	POS	NEG	POS	NEG	POS	POS	POS	NEG	POS	POS	POS	POS
Romania_Iasi_MRSA_284	NEG	NEG	NEG	POS	NEG	POS	NEG	POS	POS	POS	NEG	POS	POS	POS	POS
Romania_Iasi_MRSA-32_318	NEG	NEG	NEG	POS	NEG	POS	NEG	POS	POS	POS	NEG	POS	POS	POS	POS
Romania_Iasi_MRSA-54_430	NEG	NEG	NEG	POS	NEG	POS	NEG	POS	POS	POS	NEG	POS	POS	POS	POS
Romania_Iasi_SSTI-01_2	NEG	NEG	NEG	POS	NEG	POS	NEG	POS	POS	POS	NEG	POS	POS	POS	POS
Romania_Iasi_SSTI-10_101	NEG	NEG	NEG	POS	NEG	POS	NEG	POS	POS	POS	NEG	POS	POS	POS	POS
Romania_Iasi_SSTI-11_106	NEG	NEG	NEG	POS	NEG	POS	NEG	POS	POS	POS	NEG	POS	POS	POS	POS
Russia-01_0001_SaintPetersburg	NEG	NEG	NEG	POS	NEG	POS	NEG	POS	POS	POS	NEG	POS	POS	POS	POS
Russia-02_0004_SaintPetersburg	NEG	NEG	NEG	POS	NEG	POS	NEG	POS	POS	POS	NEG	POS	POS	POS	POS
Russia-03_0057_SaintPetersburg	NEG	NEG	NEG	POS	NEG	POS	NEG	POS	POS	POS	NEG	POS	POS	POS	POS
Russia-04_0076_Moscow	NEG	NEG	NEG	POS	NEG	POS	NEG	POS	POS	POS	NEG	POS	POS	POS	POS
Russia-05_0078_Moscow	NEG	NEG	NEG	POS	NEG	POS	NEG	POS	POS	POS	NEG	POS	POS	POS	POS
Russia-08_0135_Moscow	NEG	NEG	NEG	POS	NEG	POS	NEG	POS	POS	POS	NEG	POS	POS	POS	POS
Russia-09_0150_Kurgan	NEG	NEG	NEG	POS	NEG	POS	NEG	POS	POS	POS	NEG	POS	POS	POS	POS
Russia-10_0162_Kurgan	NEG	NEG	NEG	POS	NEG	POS	NEG	POS	POS	POS	NEG	POS	POS	POS	POS
Russia-14_0184_Moscow	NEG	NEG	NEG	POS	NEG	POS	NEG	POS	POS	POS	NEG	POS	POS	POS	POS
Russia-15_0232_SaintPetersburg	NEG	NEG	NEG	POS	NEG	POS	NEG	POS	POS	POS	NEG	POS	POS	POS	POS
Russia-22_0391_Chelyabinsk	NEG	NEG	NEG	POS	NEG	POS	NEG	POS	POS	POS	NEG	POS	POS	POS	POS
Russia-23_0392_Chelyabinsk	NEG	NEG	NEG	POS	NEG	POS	NEG	POS	POS	POS	NEG	POS	POS	POS	POS
>"Eurasian Clade" : IU17															
IU17 (Turkey)	NEG	NEG	NEG	POS	NEG	POS	NEG	POS	POS	POS	NEG	POS	POS	POS	POS
IU20 (Turkey)	NEG	NEG	NEG	POS	NEG	POS	NEG	POS	POS	POS	NEG	POS	POS	POS	POS

STRAIN / ISOLATE	VIRULENCE : OTHER FACTORS					VIRULENCE : PROTEASES									
	edinA	edinB	edinC	esxA	esxB	aur			splA	splB	splE	sspA	sspB	sspP	
	edinA	edinB	edinC	esxA	esxB	aur (cons)	aur (Other than MRSA252)	aur (MRSA252)	splA	splB	splE	sspA	sspB	sspP (cons)	sspP (other than ST93)
	epidermal cell differen-tiation inhibitor	epidermal cell differen-tiation inhibitor B	epidermal cell differen-tiation inhibitor C	virulence factor esxA	virulence factor esxB	aureolysin			serin- protease A	serin- protease B	serin- protease E	glutamylendopeptidase	Staphopain B, protease	Staphopain A (Staphylopain A), protease	
>"Eurasian Clade": Spora															
Dresden_16ANR581769	NEG	NEG	NEG	POS	NEG	POS	NEG	POS	POS	POS	NEG	POS	POS	POS	POS
Dresden_17ANR578769	NEG	NEG	NEG	POS	NEG	POS	NEG	POS	POS	POS	NEG	POS	POS	POS	POS
>"Eurasian Clade": Kuwa															
Kuwait_306	NEG	NEG	NEG	POS	NEG	POS	NEG	POS	POS	POS	NEG	POS	POS	POS	POS
Kuwait_301	NEG	NEG	NEG	POS	NEG	POS	NEG	POS	POS	POS	NEG	POS	POS	POS	POS
>"European Clade": "Gre															
GRE18 (Greece)	NEG	NEG	NEG	POS	NEG	POS	NEG	POS	POS	POS	POS	POS	POS	POS	POS
GRE317 (Greece)	NEG	NEG	NEG	POS	NEG	POS	NEG	POS	POS	POS	POS	POS	POS	POS	POS
GRE4 (Greece)	NEG	NEG	NEG	POS	NEG	POS	NEG	POS	NEG	NEG	NEG	POS	POS	POS	POS
R35 (USA)	NEG	NEG	NEG	POS	NEG	POS	NEG	POS	POS	POS	POS	POS	POS	POS	POS
Casablanca HT20060548	NEG	NEG	NEG	POS	NEG	POS	NEG	POS	POS	POS	POS	POS	POS	POS	POS
Casablanca HT20060550	NEG	NEG	NEG	POS	NEG	POS	NEG	POS	POS	POS	POS	POS	POS	POS	POS
Dresden_01V34572 (Greece)	NEG	NEG	NEG	POS	NEG	NEG	NEG	POS	POS	POS	POS	POS	POS	POS	POS
Dresden_01V36469	NEG	NEG	NEG	POS	NEG	POS	NEG	POS	POS	POS	POS	POS	POS	POS	POS
Dresden_01V39123	NEG	NEG	NEG	POS	NEG	POS	NEG	POS	POS	POS	POS	POS	POS	POS	POS
Dresden_01V39143	NEG	NEG	NEG	POS	NEG	POS	NEG	POS	POS	POS	POS	POS	POS	POS	POS
Greece 1_3680_Harmony	NEG	NEG	NEG	POS	NEG	POS	NEG	POS	POS	POS	POS	POS	POS	POS	POS
>"European Clade": UK-(															
ANS46 (Australia)	NEG	NEG	NEG	POS	NEG	POS	NEG	POS	POS	POS	POS	POS	POS	POS	POS
LHH1 (USA)	NEG	NEG	NEG	POS	NEG	POS	NEG	POS	POS	POS	POS	POS	POS	POS	POS
Dublin-DSH_AR01_0-1085	NEG	NEG	NEG	POS	NEG	POS	NEG	POS	POS	POS	POS	POS	POS	POS	POS
Dublin-DSH_AR01_0-1118	NEG	NEG	NEG	POS	NEG	POS	NEG	POS	POS	POS	POS	POS	POS	POS	POS
Dublin-DSH_AR15_0098	NEG	NEG	NEG	POS	NEG	POS	NEG	POS	POS	POS	POS	POS	POS	POS	POS
Dublin-DSH_AR15_0104	NEG	NEG	NEG	POS	NEG	POS	NEG	POS	POS	POS	POS	POS	POS	POS	POS
UK_NCTC11939_UK-1_Harmony	NEG	NEG	NEG	POS	NEG	POS	NEG	POS	POS	POS	POS	POS	POS	POS	POS
>"European Clade": BK2															
BK2421 (USA)	NEG	NEG	NEG	POS	NEG	POS	NEG	POS	POS	POS	POS	POS	POS	POS	POS
>"Aussi/NZ Clade", JKD6															
JKD6009 (Australia)	NEG	NEG	NEG	POS	NEG	POS	NEG	POS	NEG	NEG	NEG	POS	POS	POS	POS
JKD6008 (New Zealand)	NEG	NEG	NEG	POS	NEG	POS	NEG	POS	NEG	NEG	NEG	POS	POS	POS	POS
Perth_2015-S20209	NEG	NEG	NEG	POS	NEG	POS	NEG	POS	POS	POS	POS	POS	POS	POS	POS
Perth_2015-S20305	NEG	NEG	NEG	POS	NEG	POS	NEG	POS	NEG	NEG	NEG	POS	POS	POS	POS
Perth_2015-S20603	NEG	NEG	NEG	POS	NEG	POS	NEG	POS	NEG	NEG	NEG	POS	POS	POS	POS
Perth_2015-S20607	NEG	NEG	NEG	POS	NEG	POS	NEG	POS	POS	POS	POS	POS	POS	POS	POS
Perth_2015-S20608	NEG	NEG	NEG	POS	NEG	POS	NEG	POS	NEG	NEG	NEG	POS	POS	POS	POS
Perth_2015-S20610	NEG	NEG	NEG	POS	NEG	POS	NEG	POS	NEG	NEG	NEG	POS	POS	POS	POS
Perth_2015-S20613	NEG	NEG	NEG	POS	NEG	POS	NEG	POS	NEG	NEG	NEG	POS	POS	POS	POS
Perth_2015-S20617	NEG	NEG	NEG	POS	NEG	POS	NEG	POS	NEG	NEG	NEG	POS	POS	POS	POS
Perth_2015-S20622	NEG	NEG	NEG	POS	NEG	POS	NEG	POS	NEG	NEG	NEG	POS	POS	POS	POS
Perth_2015-S23619	NEG	NEG	NEG	POS	NEG	POS	NEG	POS	NEG	NEG	NEG	POS	POS	POS	POS
Perth_2015-S41210	NEG	NEG	NEG	POS	NEG	POS	NEG	POS	POS	POS	POS	POS	POS	POS	POS
Perth_2015-S51001	NEG	NEG	NEG	POS	NEG	POS	NEG	POS	POS	POS	POS	POS	POS	POS	POS
Perth_2015-S51003	NEG	NEG	NEG	POS	NEG	POS	NEG	POS	POS	POS	POS	POS	POS	POS	POS
Perth_2015-S51006	NEG	NEG	NEG	POS	NEG	POS	NEG	POS	POS	POS	POS	POS	POS	POS	POS
Perth_2015-S51007	NEG	NEG	NEG	POS	NEG	POS	NEG	POS	POS	POS	POS	POS	POS	POS	POS
Perth_2015-S51008	NEG	NEG	NEG	POS	NEG	POS	NEG	POS	POS	POS	POS	POS	POS	POS	POS
Perth_2015-S51014	NEG	NEG	NEG	POS	NEG	POS	NEG	POS	POS	POS	POS	POS	POS	POS	POS
Perth_2015-S51408	NEG	NEG	NEG	POS	NEG	POS	NEG	POS	POS	POS	POS	POS	POS	POS	POS
Perth_2016-S20614	NEG	NEG	NEG	POS	NEG	POS	NEG	POS	NEG	NEG	NEG	POS	POS	POS	POS
Perth_2016-S23614	NEG	NEG	NEG	POS	NEG	POS	NEG	POS	NEG	NEG	NEG	POS	POS	POS	POS
Perth_2016-S42009	NEG	NEG	NEG	POS	NEG	POS	NEG	POS	POS	POS	POS	POS	POS	POS	POS
Perth_2016-S51001	NEG	NEG	NEG	POS	NEG	POS	NEG	POS	POS	POS	POS	POS	POS	POS	POS
Perth_2016-S51403	NEG	NEG	NEG	POS	NEG	POS	NEG	POS	POS	POS	POS	POS	POS	POS	POS
Perth_07 RPA 20	NEG	NEG	NEG	POS	NEG	POS	NEG	POS	NEG	NEG	NEG	POS	POS	POS	POS
Perth_08-19088	NEG	NEG	NEG	POS	NEG	POS	NEG	POS	POS	POS	POS	POS	POS	POS	POS
Perth_01-15351	NEG	NEG	NEG	POS	NEG	POS	NEG	POS	POS	POS	POS	POS	POS	POS	POS
Perth_01-15357	NEG	NEG	NEG	POS	NEG	POS	NEG	POS	POS	POS	POS	POS	POS	POS	POS
Perth_01-15419	NEG	NEG	NEG	POS	NEG	POS	NEG	POS	POS	POS	POS	POS	POS	POS	POS
Perth_01-16309	NEG	NEG	NEG	POS	NEG	POS	NEG	POS	POS	POS	POS	POS	POS	POS	POS
Perth_03 SNP 95	NEG	NEG	NEG	POS	NEG	POS	NEG	POS	POS	POS	POS	POS	POS	POS	POS
Perth_2003_AH_47	NEG	NEG	NEG	POS	NEG	POS	NEG	POS	POS	POS	POS	POS	POS	POS	POS
>"Australian/NZ Clade":															
Perth_2015-S20623	NEG	NEG	NEG	POS	NEG	POS	NEG	POS	NEG	NEG	NEG	POS	POS	POS	POS
Perth_2015-S20826	NEG	NEG	NEG	POS	NEG	POS	NEG	POS	POS	POS	POS	POS	POS	POS	POS
Perth_05 NH 56	NEG	NEG	NEG	POS	NEG	POS	NEG	POS	POS	POS	POS	POS	POS	POS	POS
>CC5-MRSA-III LA MRSA															
Delbrück 38 isolate from domestic turkey	NEG	NEG	NEG	POS	POS	POS	POS	NEG	POS	POS	NEG	POS	POS	POS	POS
> Staph. pseudintermedi															
KM1381	NEG	NEG	NEG	NEG	NEG	NEG	NEG	NEG	NEG	NEG	NEG	NEG	NEG	NEG	NEG
>"South-East Asian Clad															
M92 (Canada)	NEG	NEG	NEG	POS	NEG	POS	NEG	POS	POS	POS	POS	POS	POS	POS	POS
CHI59 (China)	NEG	NEG	NEG	POS	NEG	POS	NEG	POS	POS	POS	POS	POS	POS	POS	POS
CHI61 (China)	NEG	NEG	NEG	POS	NEG	POS	NEG	POS	POS	POS	POS	POS	POS	POS	POS
CUHK_HK1997 (China)	NEG	NEG	NEG	POS	NEG	POS	NEG	POS	POS	POS	POS	POS	POS	POS	POS
M996 (China)	NEG	NEG	NEG	POS	NEG	POS	NEG	POS	POS	POS	POS	POS	POS	POS	POS
H211 (Denmark)	NEG	NEG	NEG	POS	NEG	POS	NEG	POS	POS	POS	POS	POS	POS	POS	POS
H216 (Denmark)	NEG	NEG	NEG	POS	NEG	POS	NEG	POS	POS	POS	POS	POS	POS	POS	POS
D71 (Germany)	NEG	NEG	NEG	POS	NEG	POS	NEG	POS	POS	POS	POS	POS	POS	POS	POS
NMR05 (India)	NEG	NEG	NEG	POS	NEG	POS	NEG	POS	POS	POS	POS	POS	POS	POS	POS
MAL11 (Malaysia)	NEG	NEG	NEG	POS	NEG	POS	NEG	POS	POS	POS	POS	POS	POS	POS	POS
MAL9 (Malaysia)	NEG	NEG	NEG	POS	NEG	POS	NEG	POS	POS	POS	POS	POS	POS	POS	POS
Na21 (Sri Lanka)	NEG	NEG	NEG	POS	NEG	POS	NEG	POS	POS	POS	POS	POS	POS	POS	POS
M592 (Syria)	NEG	NEG	NEG	POS	NEG	POS	NEG	POS	POS	POS	NEG	POS	POS	POS	POS
H202 (Thailand)	NEG	NEG	NEG	POS	NEG	POS	NEG	POS	POS	POS	POS	POS	POS	POS	POS
S102 (Thailand)	NEG	NEG	NEG	POS	NEG	POS	NEG	POS	POS	POS	POS	POS	POS	POS	POS
S130 (Thailand)	NEG	NEG	NEG	POS	NEG	POS	NEG	POS	POS	POS	POS	POS	POS	POS	POS
S40 (Thailand)	NEG	NEG	NEG	POS	NEG	POS	NEG	POS	POS	POS	POS	POS	POS	POS	POS
S71 (Thailand)	NEG	NEG	NEG	POS	NEG	POS	NEG	POS	POS	POS	POS	POS	POS	POS	POS
S87 (Thailand)	NEG	NEG	NEG	POS	NEG	POS	NEG	POS	POS	POS	POS	POS	POS	POS	POS
S93 (Thailand)	NEG	NEG	NEG	POS	NEG	POS	NEG	POS	POS	POS	POS	POS	POS	POS	POS
US_002 (Thailand)	NEG	NEG	NEG	POS	NEG	POS	NEG	POS	POS	POS	POS	POS	POS	POS	POS
US_017 (Thailand)	NEG	NEG	NEG	POS	NEG	POS	NEG	POS	POS	POS	POS	POS	POS	POS	POS
WAMC6102 (Thailand)	NEG	NEG	NEG	POS	NEG	POS	NEG	POS	POS	POS	POS	POS	POS	POS	POS
NCTC_13626_TW20 (UK)	NEG	NEG	NEG	POS	NEG	POS	NEG	POS	POS	POS	POS	POS	POS	POS	POS
BSAC3021 (UK)	NEG	NEG	NEG	POS	NEG	POS	NEG	POS	POS	POS	POS	POS	POS	POS	POS
BSAC3046 (UK)	NEG	NEG	NEG	POS	NEG	POS	NEG	POS	POS	POS	POS	POS	POS	POS	POS
1193_SAUR (USA)	NEG	NEG	NEG	POS	NEG	POS	NEG	POS	POS	POS	POS	POS	POS	POS	POS
1194_SAUR (USA)	NEG	NEG	NEG	POS	NEG	POS	NEG	POS	POS	POS	POS	POS	POS	POS	POS

STRAIN / ISOLATE	VIRULENCE : OTHER FACTORS					VIRULENCE : PROTEASES									
	ednA	edinB	edinC	esxA	esxB	aur			splA	splB	splE	sspA	sspB	sspP	
	edinA	edinB	edinC	esxA	esxB	aur (cons)	aur (Other than MRSA252)	aur (MRSA252)	splA	splB	splE	sspA	sspB	sspP (cons)	sspP (other than ST93)
	epidermal cell differen-tiation inhibitor	epidermal cell differen-tiation inhibitor B	epidermal cell differen-tiation inhibitor C	virulence factor esxA	virulence factor esxB	aureolysin			serin- protease A	serin- protease B	serin- protease E	glutamylendopeptidase	Staphopain B, protease	Staphopain A (Staphylopain A), protease	
>"South-East Asian Clade": TW20 (Irish AR44), co															
1195_SAUR (USA)	NEG	NEG	NEG	POS	NEG	POS	NEG	POS	POS	POS	POS	POS	POS	POS	POS
FVRH6002 (USA)	NEG	NEG	NEG	POS	NEG	POS	NEG	POS	POS	POS	POS	POS	POS	POS	POS
KINW6048 (USA)	NEG	NEG	NEG	POS	NEG	POS	NEG	POS	POS	POS	POS	POS	POS	POS	POS
LAMC0011 (USA)	NEG	NEG	NEG	POS	NEG	POS	NEG	POS	POS	POS	POS	POS	POS	POS	POS
SJOS6053 (USA)	NEG	NEG	NEG	POS	NEG	POS	NEG	POS	POS	POS	POS	POS	POS	POS	POS
SJOS6072 (USA)	NEG	NEG	NEG	POS	NEG	POS	NEG	POS	POS	POS	POS	POS	POS	POS	POS
UCIM6042 (USA)	NEG	NEG	NEG	POS	NEG	POS	NEG	POS	POS	POS	POS	POS	POS	POS	POS
Perth_2015-S31902	NEG	NEG	NEG	POS	NEG	POS	NEG	POS	POS	POS	POS	POS	POS	NEG	POS
Perth_2015-S31904	NEG	NEG	NEG	POS	NEG	POS	NEG	POS	POS	POS	POS	POS	POS	POS	POS
Perth_2015-S31913	NEG	NEG	NEG	POS	NEG	POS	NEG	POS	POS	POS	POS	POS	POS	POS	POS
Perth_2015-S31918	NEG	NEG	NEG	POS	NEG	POS	NEG	POS	POS	POS	POS	POS	POS	POS	POS
Dublin-DSH_AR44_E1183	NEG	NEG	NEG	POS	NEG	POS	NEG	POS	POS	POS	POS	POS	POS	POS	POS
Dublin-DSH_Unfamiliar-3_E1520	NEG	NEG	NEG	POS	NEG	POS	NEG	POS	POS	POS	POS	POS	POS	POS	POS
Hong Kong_042	NEG	NEG	NEG	POS	NEG	POS	NEG	POS	POS	POS	POS	POS	POS	POS	POS
Hong Kong_101	NEG	NEG	NEG	POS	NEG	POS	NEG	POS	POS	POS	POS	POS	POS	POS	POS
Hong Kong_111	NEG	NEG	NEG	POS	NEG	POS	NEG	POS	POS	POS	POS	POS	POS	POS	POS
Hong Kong_113	NEG	NEG	NEG	POS	NEG	POS	NEG	POS	POS	POS	POS	POS	POS	POS	POS
Hong Kong_115	NEG	NEG	NEG	POS	NEG	POS	NEG	POS	POS	POS	POS	POS	POS	POS	POS
Hong Kong_118	NEG	NEG	NEG	POS	NEG	POS	NEG	POS	POS	POS	POS	POS	POS	POS	POS
Hong Kong_125	NEG	NEG	NEG	POS	NEG	POS	NEG	POS	POS	POS	POS	POS	POS	POS	POS
Hong Kong_129	NEG	NEG	NEG	POS	NEG	POS	NEG	POS	POS	POS	POS	POS	POS	POS	POS
Hong Kong_133	NEG	NEG	NEG	POS	NEG	POS	NEG	POS	POS	POS	POS	POS	POS	POS	POS
Kuwait_009	NEG	NEG	NEG	POS	NEG	POS	NEG	POS	POS	POS	NEG	POS	POS	POS	POS
Kuwait_103	NEG	NEG	NEG	POS	NEG	POS	NEG	POS	POS	POS	NEG	POS	POS	POS	POS
Kuwait_107	NEG	NEG	NEG	POS	NEG	POS	NEG	POS	POS	POS	NEG	POS	POS	POS	POS
Perth_03 AH 88	NEG	NEG	NEG	POS	NEG	POS	NEG	POS	POS	POS	POS	POS	POS	POS	POS
Perth_08-16905	NEG	NEG	NEG	POS	NEG	POS	NEG	POS	POS	POS	POS	POS	POS	POS	POS
Riyadh_Alfaisal-10_SICU_13_B5672_1117353	NEG	NEG	NEG	POS	NEG	POS	NEG	POS	POS	POS	NEG	POS	POS	POS	POS
Riyadh_SSTI_17_3508602	NEG	NEG	NEG	POS	NEG	POS	NEG	POS	POS	POS	NEG	POS	POS	POS	POS
Riyadh-2822088-R	NEG	NEG	NEG	POS	NEG	POS	NEG	POS	POS	POS	NEG	POS	POS	POS	POS
Trinidad&Tobago_2012_195 (619/12)	NEG	NEG	NEG	POS	NEG	POS	NEG	POS	POS	POS	POS	POS	POS	POS	POS
Trinidad&Tobago_2012_370 (1112-52177)	NEG	NEG	NEG	POS	NEG	POS	NEG	POS	POS	POS	POS	POS	POS	POS	POS
Trinidad&Tobago_2012_378 (4139)	NEG	NEG	NEG	POS	NEG	POS	NEG	POS	POS	POS	POS	POS	POS	POS	POS
Trinidad&Tobago_2013_PA21	NEG	NEG	NEG	POS	NEG	POS	NEG	POS	POS	POS	POS	POS	POS	POS	POS
Trinidad&Tobago_2013_PA22	NEG	NEG	NEG	POS	NEG	POS	NEG	POS	POS	POS	POS	POS	POS	POS	POS
Trinidad&Tobago_2912_340 (8523/12)	NEG	NEG	NEG	POS	NEG	POS	NEG	POS	POS	POS	POS	POS	POS	POS	POS
Trinidad&Tobago_MRSA2010_105	NEG	NEG	NEG	POS	NEG	POS	NEG	POS	POS	POS	POS	POS	POS	POS	POS
Trinidad&Tobago_MRSA2010_108	NEG	NEG	NEG	POS	NEG	POS	NEG	POS	POS	POS	POS	POS	POS	POS	POS
Trinidad&Tobago_MRSA2010_33	NEG	NEG	NEG	POS	NEG	POS	NEG	POS	POS	POS	POS	POS	POS	POS	POS
Trinidad&Tobago_MRSA2010_79	NEG	NEG	NEG	POS	NEG	POS	NEG	POS	POS	POS	POS	POS	POS	POS	POS
Trinidad&Tobago_MRSA2010_88	NEG	NEG	NEG	POS	NEG	POS	NEG	POS	POS	POS	POS	POS	POS	POS	POS
Trinidad&Tobago_MRSA2010_97	NEG	NEG	NEG	POS	NEG	POS	NEG	POS	POS	POS	POS	POS	POS	POS	POS
Trinidad&Tobago_SSTI 2012_031	NEG	NEG	NEG	POS	NEG	POS	NEG	POS	POS	POS	POS	POS	POS	POS	POS
Uganda-29_17148_250811	NEG	NEG	NEG	POS	NEG	POS	NEG	POS	POS	POS	POS	POS	POS	POS	POS
>"South-East Asian Clad															
UCIM6015	NEG	NEG	NEG	POS	NEG	POS	NEG	POS	POS	POS	POS	POS	POS	POS	POS
Hong Kong_121	NEG	NEG	NEG	POS	NEG	POS	NEG	POS	POS	POS	POS	POS	POS	POS	POS
>"South-East Asian Clad															
Z172 (Taiwan)	NEG	NEG	NEG	POS	NEG	POS	NEG	POS	POS	POS	NEG	POS	POS	POS	POS
Russia-19_0342_Kurgan	NEG	NEG	NEG	POS	NEG	POS	NEG	POS	POS	POS	POS	POS	POS	POS	POS
>"South-East Asian Clad															
Perth_01-15392	NEG	NEG	NEG	POS	NEG	POS	NEG	POS	POS	POS	POS	POS	POS	POS	POS
>"South-East Asian Clad															
XN108 (China)	NEG	NEG	NEG	POS	NEG	POS	NEG	POS	POS	POS	POS	POS	POS	POS	POS
NMR09 (India)	NEG	NEG	NEG	POS	NEG	POS	NEG	POS	POS	POS	POS	POS	POS	POS	POS
346 (Malaysia)	NEG	NEG	NEG	POS	NEG	POS	NEG	POS	POS	POS	POS	POS	POS	POS	POS
DS_009 (Thailand)	NEG	NEG	NEG	POS	NEG	POS	NEG	POS	POS	POS	POS	POS	POS	POS	POS
18ANR581005 (Ind)	NEG	NEG	NEG	POS	NEG	POS	NEG	POS	NEG	NEG	POS	POS	POS	POS	POS
Bengalaru_ST20141405	NEG	NEG	NEG	POS	NEG	POS	NEG	POS	POS	POS	POS	POS	POS	POS	POS
Bengalaru_ST20141406	NEG	NEG	NEG	POS	NEG	POS	NEG	POS	POS	POS	POS	POS	POS	POS	POS
Bengalaru_ST20141417	NEG	NEG	NEG	POS	NEG	POS	NEG	POS	POS	POS	POS	POS	POS	POS	POS
Hong Kong_107	NEG	NEG	NEG	POS	NEG	POS	NEG	POS	POS	POS	POS	POS	POS	POS	POS
Hong Kong_12	NEG	NEG	NEG	POS	NEG	POS	NEG	POS	POS	POS	POS	POS	POS	POS	POS
Hong Kong_132	NEG	NEG	NEG	POS	NEG	POS	NEG	POS	POS	POS	POS	POS	POS	POS	POS
Riyadh-3028763-R	NEG	NEG	NEG	POS	NEG	POS	NEG	POS	POS	POS	NEG	POS	POS	POS	POS
Trinidad&Tobago_MRSA2010_94	NEG	NEG	NEG	POS	NEG	POS	NEG	POS	POS	POS	POS	POS	POS	POS	POS
>"South-East Asian Clad															
M705 (Thailand)	NEG	NEG	NEG	POS	NEG	POS	NEG	POS	POS	POS	POS	POS	POS	POS	POS
S85 (Thailand)	NEG	NEG	NEG	POS	NEG	POS	NEG	POS	POS	POS	POS	POS	POS	POS	POS
Riyadh_Alfaisal-04_281014_S5076	NEG	NEG	NEG	POS	NEG	POS	NEG	POS	POS	POS	NEG	POS	POS	POS	POS
Riyadh_Alfaisal-26_S15145_1104163	NEG	NEG	NEG	POS	NEG	POS	NEG	POS	POS	POS	NEG	POS	POS	POS	POS
Riyadh-2817437-W	NEG	NEG	NEG	POS	NEG	POS	NEG	POS	POS	POS	NEG	POS	POS	POS	POS
>"South-East Asian Clad															
PPUKM-775-2009 (Malaysia)	NEG	NEG	NEG	POS	NEG	POS	NEG	POS	POS	POS	POS	POS	POS	POS	POS
Hong Kong_138	NEG	NEG	NEG	POS	NEG	POS	NEG	POS	POS	POS	POS	POS	POS	POS	POS
>"South-East Asian Clad															
Kuwait_001	NEG	NEG	NEG	POS	NEG	POS	NEG	POS	POS	POS	POS	POS	POS	POS	POS
Kuwait_337	NEG	NEG	NEG	POS	POS	POS	NEG	POS	POS	POS	POS	POS	POS	POS	POS
>"South-East Asian Clad															
CUHK_HK2007 (China)	NEG	NEG	NEG	POS	NEG	POS	NEG	POS	POS	POS	POS	POS	POS	POS	POS
NMR02 (India)	NEG	NEG	NEG	POS	NEG	POS	NEG	POS	POS	POS	POS	POS	POS	POS	POS
VB1490 (India)	NEG	NEG	NEG	POS	NEG	POS	NEG	POS	POS	POS	POS	POS	POS	POS	POS
V521 (Korea)	NEG	NEG	NEG	POS	NEG	POS	NEG	POS	POS	POS	POS	POS	POS	POS	POS
HST-077 (Lebanon)	NEG	NEG	NEG	POS	NEG	POS	NEG	POS	POS	POS	POS	POS	POS	POS	POS
MAL1 (Malaysia)	NEG	NEG	NEG	POS	NEG	POS	NEG	POS	POS	POS	POS	POS	POS	POS	POS
MAL3 (Malaysia)	NEG	NEG	NEG	POS	NEG	POS	NEG	POS	POS	POS	POS	POS	POS	POS	POS
PPUKM-261-2009 (Malaysia)	NEG	NEG	NEG	POS	NEG	POS	NEG	POS	POS	POS	POS	POS	POS	POS	POS
PPUKM-332-2009 (Malaysia)	NEG	NEG	NEG	POS	NEG	POS	NEG	POS	POS	POS	POS	POS	POS	POS	POS
S38 (Thailand)	NEG	NEG	NEG	POS	NEG	POS	NEG	POS	POS	POS	NEG	POS	POS	POS	POS
S7 (Thailand)	NEG	NEG	NEG	POS	NEG	POS	NEG	POS	POS	POS	NEG	POS	POS	POS	POS
US_008 (Thailand)	NEG	NEG	NEG	POS	NEG	POS	NEG	POS	POS	POS	NEG	POS	POS	POS	POS
US_030 (Thailand)	NEG	NEG	NEG	POS	NEG	POS	NEG	POS	POS	POS	POS	POS	POS	POS	POS
M170 (Tsunami-related)	NEG	NEG	NEG	POS	NEG	POS	NEG	POS	POS	POS	POS	POS	POS	POS	POS
BSAC697 (UK)	NEG	NEG	NEG	POS	NEG	POS	NEG	POS	POS	POS	POS	POS	POS	POS	POS
IS-189	NEG	NEG	NEG	POS	NEG	POS	NEG	POS	POS	POS	POS	POS	POS	POS	POS
MRGR3	NEG	NEG	NEG	POS	NEG	POS	NEG	POS	POS	POS	POS	POS	POS	POS	POS
smg07	NEG	NEG	NEG	POS	NEG	POS	NEG	POS	POS	POS	POS	POS	POS	POS	POS
AH1-AUS-EMRSA-3	NEG	NEG	NEG	POS	NEG	POS	NEG	POS	POS	POS	POS	POS	POS	POS	POS
Bengalaru_ST20141403	NEG	NEG	NEG	POS	NEG	POS	NEG	POS	POS	POS	POS	POS	POS	POS	POS
Finland E24_98541_Harmony	NEG	NEG	NEG	POS	NEG	POS	NEG	POS	POS	POS	POS	POS	POS	POS	POS
Hong Kong_10	NEG	NEG	NEG	POS	NEG	POS	NEG	POS	POS	POS	POS	POS	POS	POS	POS
Hong Kong_100	NEG	NEG	NEG	POS	NEG	POS	NEG	POS	POS	POS	POS	POS	POS	POS	POS
Hong Kong_199	NEG	NEG	NEG	POS	NEG	POS	NEG	POS	POS	POS	POS	POS	POS	POS	POS
Hong Kong_34	NEG	NEG	NEG	POS	NEG	POS	NEG	POS	POS	POS	POS	POS	POS	POS	POS
Hong Kong_88	NEG	NEG	NEG	POS	NEG	POS	NEG	POS	POS	POS	POS	POS	POS	POS	POS
Hong Kong_92	NEG	NEG	NEG	POS	NEG	POS	NEG	POS	POS	POS	POS	POS	POS	POS	POS
Hong Kong_94	NEG	NEG	NEG	POS	NEG	POS	NEG	POS	POS	POS	POS	POS	POS	POS	POS
Kuwait_257	NEG	NEG	NEG	POS	NEG	POS	NEG	POS	POS	POS	POS	POS	POS	POS	POS
Perth_01-15330	NEG	NEG	NEG	POS	NEG	POS	NEG	POS	POS	POS	POS	POS	POS	POS	POS
Perth_01-15349	NEG	NEG	NEG	POS	NEG	POS	NEG	POS	POS	POS	POS	POS	POS	POS	POS
Perth_01-16176	NEG	NEG	NEG	POS	NEG	POS	NEG	POS	POS	POS	POS	POS	POS	POS	POS
Perth_WBG8379	NEG	NEG	NEG	POS	NEG	POS	NEG	POS	POS	POS	POS	POS	POS	POS	POS
Rawalpindi_Kidney Center_56	NEG	NEG	NEG	POS	NEG	POS	NEG	POS	POS	POS	POS	POS	POS	POS	POS
Riyadh_SSTI_05_3464614	NEG	NEG	NEG	POS	NEG	POS	NEG	POS	POS	POS	NEG	POS	POS	POS	POS
Trinidad&Tobago_2012_157	NEG	NEG	NEG	POS	NEG	POS	NEG	POS	POS	POS	POS	POS	POS	POS	POS

STRAIN / ISOLATE	VIRULENCE : OTHER FACTORS					VIRULENCE : PROTEASES									
	edinA	edinB	edinC	esxA	esxB	aur			splA	splB	splE	sspA	sspB	sspP	
	edinA	edinB	edinC	esxA	esxB	aur (cons)	aur (Other than MRSA252)	aur (MRSA252)	splA	splB	splE	sspA	sspB	sspP (cons)	sspP (other than ST93)
	epidermal cell differen-tiation inhibitor	epidermal cell differen-tiation inhibitor B	epidermal cell differen-tiation inhibitor C	virulence factor esxA	virulence factor esxB	aureolysin			serin- protease A	serin- protease B	serin- protease E	glutamylendopeptidase	Staphopain B, protease	Staphopain A (Staphylopain A), protease	
>"South-East Asian Clad															
M418	NEG	NEG	NEG	POS	NEG	POS	NEG	POS	POS	POS	POS	POS	POS	POS	POS
>"South-East Asian Clad															
NMR07 (India)	NEG	NEG	NEG	POS	NEG	POS	NEG	POS	POS	POS	POS	POS	POS	POS	POS
NMR08 (India)	NEG	NEG	NEG	POS	NEG	POS	NEG	POS	POS	POS	POS	POS	POS	POS	POS
Bengalaru_ST20121978	NEG	NEG	NEG	POS	NEG	POS	NEG	POS	POS	POS	POS	POS	POS	POS	POS
Hong Kong_89	NEG	NEG	NEG	POS	NEG	POS	NEG	POS	POS	POS	NEG	POS	POS	POS	POS
>"South-East Asian Clad															
Perth_2005_AH_5	NEG	NEG	NEG	POS	NEG	POS	NEG	POS	POS	POS	POS	POS	POS	POS	POS
>"South-East Asian Clad															
Trinidad&Tobago_MRSA2010_103	NEG	NEG	NEG	POS	NEG	POS	NEG	POS	POS	POS	POS	POS	POS	POS	POS
Trinidad&Tobago_2013_PA25	NEG	NEG	NEG	POS	NEG	POS	NEG	POS	POS	POS	POS	POS	POS	POS	POS
Trinidad&Tobago_MRSA2010_110	NEG	NEG	NEG	POS	NEG	POS	NEG	POS	POS	POS	POS	POS	POS	POS	POS
Trinidad&Tobago_SSTI 2012_111	NEG	NEG	NEG	POS	NEG	POS	NEG	POS	POS	POS	POS	POS	POS	POS	POS
Trinidad&Tobago_MRSA2010_158	NEG	NEG	NEG	POS	NEG	POS	NEG	POS	POS	POS	POS	POS	POS	POS	POS
>"South-East Asian Clad															
Kuwait_122	NEG	NEG	NEG	POS	NEG	POS	NEG	POS	POS	POS	POS	POS	POS	POS	POS
Kuwait_002	NEG	NEG	NEG	POS	NEG	POS	NEG	POS	POS	POS	POS	POS	POS	POS	POS
Kuwait_196	NEG	NEG	NEG	POS	NEG	POS	NEG	POS	POS	POS	POS	POS	POS	POS	POS
Kuwait_199	NEG	NEG	NEG	POS	NEG	POS	NEG	POS	POS	POS	NEG	POS	POS	POS	POS
Hong Kong_136	NEG	NEG	NEG	POS	NEG	POS	NEG	POS	POS	POS	POS	POS	POS	POS	POS
>"South-East Asian Clad															
P32 (Poland)	NEG	NEG	NEG	POS	NEG	POS	NEG	POS	POS	POS	POS	POS	POS	POS	POS
>"South-East Asian Clad															
Perth_2015-S32313	NEG	NEG	NEG	POS	NEG	POS	NEG	POS	POS	POS	POS	POS	POS	POS	POS
>"hla-negative South-Ea															
DEN907 (Denmark)	NEG	NEG	NEG	POS	NEG	POS	NEG	POS	POS	POS	POS	POS	POS	POS	POS
>"hla-negative South-Ea															
S26 (Thailand)	NEG	NEG	NEG	POS	NEG	POS	NEG	POS	POS	POS	POS	POS	POS	POS	POS
S42 (Thailand)	NEG	NEG	NEG	POS	NEG	POS	NEG	POS	POS	POS	POS	POS	POS	POS	POS
S97 (Thailand)	NEG	NEG	NEG	POS	NEG	POS	NEG	POS	POS	POS	POS	POS	POS	POS	POS
S78 (Thailand)	NEG	NEG	NEG	POS	NEG	POS	NEG	POS	POS	POS	POS	POS	POS	POS	POS
S25 (Thailand)	NEG	NEG	NEG	POS	NEG	POS	NEG	POS	POS	POS	POS	POS	POS	POS	POS
S21 (Thailand)	NEG	NEG	NEG	POS	NEG	POS	NEG	POS	POS	POS	POS	POS	POS	POS	POS
S106 (Thailand)	NEG	NEG	NEG	POS	NEG	POS	NEG	POS	POS	POS	POS	POS	POS	POS	POS
S24 (Thailand)	NEG	NEG	NEG	POS	NEG	POS	NEG	POS	POS	POS	POS	POS	POS	POS	POS
S39 (Thailand)	NEG	NEG	NEG	POS	NEG	POS	NEG	POS	POS	POS	POS	POS	POS	POS	POS
S2 (Thailand)	NEG	NEG	NEG	POS	NEG	POS	NEG	POS	POS	POS	POS	POS	POS	POS	POS
S81 (Thailand)	NEG	NEG	NEG	POS	NEG	POS	NEG	POS	POS	POS	POS	POS	POS	POS	POS
M116 (Vietnam)	NEG	NEG	NEG	POS	NEG	POS	NEG	POS	POS	POS	POS	POS	POS	POS	POS
>"Portuguese Clade": Po															
FFP103 (Portugal)	NEG	NEG	NEG	POS	NEG	POS	NEG	POS	POS	POS	POS	POS	POS	POS	POS
HDG2 (Portugal)	NEG	NEG	NEG	POS	NEG	POS	NEG	POS	POS	POS	NEG	POS	POS	POS	POS
HSA10 (Portugal)	NEG	NEG	NEG	POS	NEG	POS	NEG	POS	POS	POS	POS	POS	POS	POS	POS
HSA11 (Portugal)	NEG	NEG	NEG	POS	NEG	POS	NEG	POS	POS	POS	POS	POS	POS	POS	POS
ICP5011 (Portugal)	NEG	NEG	NEG	POS	NEG	POS	NEG	POS	NEG	NEG	NEG	POS	POS	POS	POS
ICP5014 (Portugal)	NEG	NEG	NEG	POS	NEG	POS	NEG	POS	POS	POS	POS	POS	POS	POS	POS
ICP5062 (Portugal)	NEG	NEG	NEG	POS	NEG	POS	NEG	POS	POS	POS	POS	POS	POS	POS	POS
Strain 1063_ATCC 33592 FDAARGOS_3 (USA)	NEG	NEG	NEG	POS	NEG	POS	NEG	POS	POS	POS	POS	POS	POS	POS	POS
	NEG	NEG	NEG	POS	NEG	POS	NEG	POS	POS	POS	POS	POS	POS	POS	POS
Russia-07_0110_Kurgan	NEG	NEG	NEG	POS	NEG	POS	NEG	POS	POS	POS	POS	POS	POS	POS	POS
Russia-11_0164_Kurgan	NEG	NEG	NEG	POS	NEG	POS	NEG	POS	POS	POS	POS	POS	POS	POS	POS
Russia-20_0367_Kurgan	NEG	NEG	NEG	POS	NEG	POS	NEG	POS	POS	POS	NEG	POS	POS	POS	POS
Russia-21_0390_Chelyabinsk	NEG	NEG	NEG	POS	NEG	POS	NEG	POS	POS	POS	NEG	POS	POS	POS	POS
>"South American/ Midd															
AGT1 (Argentina)	NEG	NEG	NEG	POS	NEG	POS	NEG	POS	POS	POS	POS	POS	POS	POS	POS
AGT120 (Argentina)	NEG	NEG	NEG	POS	NEG	POS	NEG	POS	POS	POS	POS	POS	POS	POS	POS
AGT67 (Argentina)	NEG	NEG	NEG	POS	NEG	POS	NEG	POS	POS	POS	POS	POS	POS	POS	POS
AGT9 (Argentina)	NEG	NEG	NEG	POS	NEG	POS	NEG	POS	POS	POS	POS	POS	POS	POS	POS
RA3 (Argentina)	NEG	NEG	NEG	POS	NEG	POS	NEG	POS	POS	POS	POS	POS	POS	POS	POS
RA6 (Argentina)	NEG	NEG	NEG	POS	NEG	POS	NEG	POS	POS	POS	POS	POS	POS	POS	POS
RA7 (Argentina)	NEG	NEG	NEG	POS	NEG	POS	NEG	POS	POS	POS	POS	POS	POS	POS	POS
Be62 (Brazil)	NEG	NEG	NEG	POS	NEG	POS	NEG	POS	POS	POS	POS	POS	POS	POS	POS
Bmb9393 (Brazil)	NEG	NEG	NEG	POS	NEG	POS	NEG	POS	POS	POS	POS	POS	POS	POS	POS
BRA36 (Brazil)	NEG	NEG	NEG	POS	NEG	POS	NEG	POS	POS	POS	POS	POS	POS	POS	POS
BZ48 (Brazil)	NEG	NEG	NEG	POS	NEG	POS	NEG	POS	POS	POS	POS	POS	POS	POS	POS
Gv51 (Brazil)	NEG	NEG	NEG	POS	NEG	POS	NEG	POS	POS	POS	POS	POS	POS	POS	POS
Gv69 (Brazil)	NEG	NEG	NEG	POS	NEG	POS	NEG	POS	POS	POS	POS	POS	POS	POS	POS
Gv88 (Brazil)	NEG	NEG	NEG	POS	NEG	POS	NEG	POS	POS	POS	POS	POS	POS	POS	POS
HC1335 (Brazil)	NEG	NEG	NEG	POS	NEG	POS	NEG	POS	POS	POS	POS	POS	POS	POS	POS
HC1340 (Brazil)	NEG	NEG	NEG	POS	NEG	POS	NEG	POS	POS	POS	POS	POS	POS	POS	POS
HC556 (Brazil)	NEG	NEG	NEG	POS	NEG	POS	NEG	POS	POS	POS	POS	POS	POS	POS	POS
HU25 (Brazil)	NEG	NEG	NEG	POS	NEG	POS	NEG	POS	POS	POS	POS	POS	POS	POS	POS
UB563 (Brazil)	NEG	NEG	NEG	POS	NEG	POS	NEG	POS	POS	POS	POS	POS	POS	POS	POS
2A8 (Czech Republic)	NEG	NEG	NEG	POS	NEG	POS	NEG	POS	POS	POS	POS	POS	POS	POS	POS
M1229 (Denmark)	NEG	NEG	NEG	POS	NEG	POS	NEG	POS	POS	POS	POS	POS	POS	POS	POS
FRICAR (France)	NEG	NEG	NEG	POS	NEG	POS	NEG	POS	POS	POS	POS	POS	POS	POS	POS
D90 (Germany)	NEG	NEG	NEG	POS	NEG	POS	NEG	POS	POS	POS	POS	POS	POS	POS	POS
LIT68 (Lithuania)	NEG	NEG	NEG	POS	NEG	POS	NEG	POS	POS	POS	POS	POS	POS	POS	POS
LIT76 (Lithuania)	NEG	NEG	NEG	POS	NEG	POS	NEG	POS	POS	POS	POS	POS	POS	POS	POS
HGSA142 (Portugal)	NEG	NEG	NEG	POS	NEG	POS	NEG	POS	POS	POS	POS	POS	POS	POS	POS
HGSA9 (Portugal)	NEG	NEG	NEG	POS	NEG	POS	NEG	POS	POS	POS	POS	POS	POS	POS	POS
HSJ216 (Portugal)	NEG	NEG	NEG	POS	NEG	POS	NEG	POS	POS	POS	POS	POS	POS	POS	POS
M278 (Portugal)	NEG	NEG	NEG	POS	NEG	POS	NEG	POS	POS	POS	POS	POS	POS	POS	POS
ES26 (Spain)	NEG	NEG	NEG	POS	NEG	POS	NEG	POS	POS	POS	POS	POS	POS	POS	POS
UK102 (UK)	NEG	NEG	NEG	POS	NEG	POS	NEG	POS	POS	POS	POS	POS	POS	POS	POS
URU34 (Uruguay)	NEG	NEG	NEG	POS	NEG	POS	NEG	POS	POS	POS	POS	POS	POS	POS	POS
IS-125	NEG	NEG	NEG	POS	NEG	POS	NEG	POS	POS	POS	POS	POS	POS	POS	POS
IS-157	NEG	NEG	NEG	POS	NEG	POS	NEG	POS	POS	POS	POS	POS	POS	POS	POS
Ecuador_1	NEG	NEG	NEG	POS	NEG	POS	NEG	POS	POS	POS	POS	POS	POS	POS	POS
Ecuador_2	NEG	NEG	NEG	POS	NEG	POS	NEG	POS	POS	POS	POS	POS	POS	POS	POS
Dresden_15ANR574263	NEG	NEG	NEG	POS	NEG	POS	NEG	POS	POS	POS	POS	POS	POS	POS	POS
UK-EMRSA-11	NEG	NEG	NEG	POS	NEG	POS	NEG	POS	POS	POS	POS	POS	POS	POS	POS
>"South American/ Midd															
BRA2 (Brazil)	NEG	NEG	NEG	POS	NEG	POS	NEG	POS	POS	POS	POS	POS	POS	POS	POS
CHL1 (Chile)	NEG	NEG	NEG	POS	NEG	POS	NEG	POS	POS	POS	POS	POS	POS	POS	POS
CHL151 (Chile)	NEG	NEG	NEG	POS	NEG	POS	NEG	POS	POS	POS	POS	POS	POS	POS	POS
UCL378 (Chile)	NEG	NEG	NEG	POS	NEG	POS	NEG	POS	POS	POS	POS	POS	POS	POS	POS
UP72 (Peru)	NEG	NEG	NEG	POS	NEG	POS	NEG	POS	POS	POS	POS	POS	POS	POS	POS
UP81 (Peru)	NEG	NEG	NEG	POS	NEG	POS	NEG	POS	POS	POS	POS	POS	POS	POS	POS
>"South American/ Midd															
UP1073 (Peru)	NEG	NEG	NEG	POS	NEG	POS	NEG	POS	POS	POS	POS	POS	POS	POS	POS

STRAIN / ISOLATE	VIRULENCE : OTHER FACTORS					VIRULENCE : PROTEASES									
	edinA	edinB	edinC	esxA	esxB	aur			splA	splB	splE	sspA	sspB	sspP	
	edinA	edinB	edinC	esxA	esxB	aur (cons)	aur (Other than MRSA252)	aur (MRSA252)	splA	splB	splE	sspA	sspB	sspP (cons)	sspP (other than ST93)
	epidermal cell differen-tiation inhibitor	epidermal cell differen-tiation inhibitor B	epidermal cell differen-tiation inhibitor C	virulence factor esxA	virulence factor esxB	aureolysin			serin- protease A	serin- protease B	serin- protease E	glutamylendopeptidase	Staphopain B, protease	Staphopain A (Staphylopain A), protease	
>"South American/ Mid															
LIT89 (Lithuania)	NEG	NEG	NEG	POS	NEG	POS	NEG	POS	POS	POS	POS	POS	POS	POS	POS
MRSA_PR1 (Malaysia)	NEG	NEG	NEG	POS	NEG	POS	NEG	POS	POS	POS	POS	POS	POS	POS	POS
Riyadh-2888905-R	NEG	NEG	NEG	POS	NEG	POS	NEG	POS	POS	POS	NEG	POS	POS	POS	POS
Riyadh-2888915-BC	NEG	NEG	NEG	POS	NEG	POS	NEG	POS	POS	POS	NEG	POS	POS	POS	POS
Riyadh-2793706-R	NEG	NEG	NEG	POS	NEG	POS	NEG	POS	POS	POS	NEG	POS	POS	POS	POS
>"South American/ Mid															
Riyadh_SSTI_18_3502925	NEG	NEG	NEG	POS	NEG	POS	NEG	POS	POS	POS	NEG	POS	POS	POS	POS
Riyadh-2822825-W	NEG	NEG	NEG	POS	NEG	POS	NEG	POS	POS	POS	NEG	POS	POS	POS	POS
>"South American/ Mid															
UK-EMRSA-9	NEG	NEG	NEG	POS	NEG	POS	NEG	POS	POS	POS	POS	POS	POS	POS	POS
>"South American/ Mid															
Lome_HT20020815	NEG	NEG	NEG	POS	NEG	POS	NEG	POS	POS	POS	POS	POS	POS	POS	POS
>"South American/ Mid															
NA32 (Denmark)	NEG	NEG	NEG	POS	NEG	POS	NEG	POS	POS	POS	POS	POS	POS	POS	POS
H24 (Egypt)	NEG	NEG	NEG	POS	NEG	POS	NEG	POS	POS	POS	POS	POS	POS	POS	POS
ATCC BAA-39 (=HUSA304) (Hungary)	NEG	NEG	NEG	POS	NEG	POS	NEG	POS	POS	POS	POS	POS	POS	POS	POS
HUSA304 (Hungary)	NEG	NEG	NEG	POS	NEG	POS	NEG	POS	POS	POS	POS	POS	POS	POS	POS
HU106 (Hungary)	NEG	NEG	NEG	POS	NEG	POS	NEG	POS	POS	POS	POS	POS	POS	POS	POS
BSAC27 (UK)	NEG	NEG	NEG	POS	NEG	POS	NEG	POS	POS	POS	POS	POS	POS	POS	POS
NCTC13131, UK-EMRSA-4 (UK)	NEG	NEG	NEG	POS	NEG	POS	NEG	POS	POS	POS	POS	POS	POS	POS	POS
	NEG	NEG	NEG	POS	NEG	POS	NEG	POS	POS	POS	POS	POS	POS	POS	POS
Algiers_HT20040080	NEG	NEG	NEG	POS	NEG	POS	NEG	POS	POS	POS	POS	POS	POS	POS	POS
Dublin-DSH_AR09_0.0066	NEG	NEG	NEG	POS	NEG	POS	NEG	POS	POS	POS	POS	POS	POS	POS	POS
Dublin-DSH_AR09_0-0065	NEG	NEG	NEG	POS	NEG	AMB	NEG	POS	POS	POS	POS	POS	POS	POS	POS
Dublin-DSH_Phenotype-III_84	NEG	NEG	NEG	POS	NEG	POS	NEG	POS	POS	POS	POS	POS	POS	POS	POS
Hong Kong_130	NEG	NEG	NEG	POS	NEG	POS	NEG	POS	POS	POS	POS	POS	POS	POS	POS
Kuwait_018	NEG	NEG	NEG	POS	NEG	POS	NEG	POS	POS	POS	POS	POS	POS	POS	POS
Perth_08-17726	NEG	NEG	NEG	POS	NEG	POS	NEG	POS	POS	POS	POS	POS	POS	POS	POS
Riyadh_Alfaisal/KKKSUH_86_MRS-14-279	NEG	NEG	NEG	POS	NEG	POS	NEG	POS	POS	POS	NEG	POS	POS	POS	POS
Riyadh_Alfaisal-04_Z3861_831588	NEG	NEG	NEG	POS	NEG	POS	NEG	POS	POS	POS	NEG	POS	POS	POS	POS
Riyadh_Alfaisal-30_S15724_1108013	NEG	NEG	NEG	POS	NEG	POS	NEG	POS	POS	POS	NEG	POS	POS	POS	POS
Riyadh_Alfaisal-6_22A_13_B3992_397721	NEG	NEG	NEG	POS	NEG	POS	NEG	POS	POS	POS	NEG	POS	POS	POS	POS
Riyadh_SSTI_52_3615482	NEG	NEG	NEG	POS	NEG	POS	NEG	POS	POS	POS	NEG	POS	POS	POS	POS
Riyadh-2817276-2	NEG	NEG	NEG	POS	NEG	POS	NEG	POS	POS	POS	NEG	POS	POS	POS	POS
Riyadh-2891670-W	NEG	NEG	NEG	POS	NEG	POS	NEG	POS	POS	POS	NEG	POS	POS	POS	POS
Riyadh-3006920-W	NEG	NEG	NEG	POS	NEG	POS	NEG	POS	POS	POS	NEG	POS	POS	POS	POS
Riyadh-R2567782	NEG	NEG	NEG	POS	NEG	POS	NEG	POS	POS	POS	NEG	POS	POS	POS	POS
Russia-18_0252_Krasnoyarsk_SK2	NEG	NEG	NEG	POS	NEG	POS	NEG	POS	POS	POS	POS	POS	POS	POS	POS
UK-EMRSA-7	NEG	NEG	NEG	POS	NEG	POS	NEG	POS	POS	POS	POS	POS	POS	POS	POS
>"South American/ Mid															
Dublin-DSH_AR23_0073	NEG	NEG	NEG	POS	NEG	POS	NEG	POS	POS	POS	POS	POS	POS	POS	POS
>Related to "South Ame															
MRSA-OC3 (Russia)	NEG	NEG	NEG	POS	NEG	POS	NEG	POS	POS	POS	POS	POS	POS	POS	POS
Russia-12_0176_Krasnoyarsk	NEG	NEG	NEG	POS	NEG	POS	NEG	POS	POS	POS	POS	POS	POS	POS	POS
Russia-13_0180_Krasnoyarsk	NEG	NEG	NEG	POS	NEG	POS	NEG	POS	POS	POS	POS	POS	POS	POS	POS
Russia-16_0249_Krasnoyarsk	NEG	NEG	NEG	POS	NEG	POS	NEG	POS	POS	POS	POS	POS	POS	POS	POS
Russia-17_0250_Krasnoyarsk	NEG	NEG	NEG	POS	NEG	POS	NEG	POS	POS	POS	POS	POS	POS	POS	POS
>Related to "South Ame															
URU110 (Uruguay)	NEG	NEG	NEG	POS	NEG	POS	NEG	POS	POS	POS	POS	POS	POS	POS	POS
>Related to "South Ame															
DS_014 (Thailand)	NEG	NEG	NEG	POS	NEG	POS	NEG	POS	POS	POS	POS	POS	POS	POS	POS
>Unassigned Middle Eas															
Frankfurt_Oder_0490031797 (Libya)	NEG	NEG	NEG	POS	NEG	POS	NEG	POS	POS	POS	POS	POS	POS	POS	POS
Kuwait_192	NEG	NEG	NEG	POS	NEG	POS	NEG	POS	POS	POS	POS	POS	POS	POS	POS
Riyadh-9	NEG	NEG	NEG	POS	NEG	POS	NEG	POS	POS	POS	POS	POS	POS	POS	POS
Russia-24_0407_Moscow	NEG	NEG	NEG	POS	NEG	POS	NEG	POS	POS	POS	POS	POS	POS	POS	POS

STRAIN / ISOLATE	SPECIES MARKER							STAPHYLOXANTHIN BIOSYNTH. OPERON					REGULATORY GENES					
	rrnD1	gapA	katA	CoA	nuc1	spa	sbi	crtM		crtN	crtO	crtP	sarA	saeS	vraS	agrI	agrII	agrIII
	rrnD1 (S. aureus)	gapA	katA	CoA	nuc1	spa	sbi	crtM-nonST93	crtM-ST93	crtN	crtO	crtP	sarA	saeS	vraS			
	Domain 1 of 23S-rRNA	glyceraldehyde 3-phosphate dehydrogenase, locus 1	katalase A	coagulase	thermostable extracellular nuclease	staphylococcal protein A	IgG-binding protein	dehydrosqualene synthase		dehydrosqualene desaturase	staphyloxanthin acyltransferase	diaponeurosporene oxidase	staphylococcal accessory regulator A	histidine protein kinase, sae locus	sensor protein	accessory gene regulator allele I	accessory gene regulator allele II	accessory gene regulator allele III
>"Eurasian Clade": TUR1																		
TUR1 (Turkey)	POS	POS	POS	POS	POS	POS	POS	POS	NEG	POS	POS	POS	POS	POS	POS	POS	NEG	NEG
TUR9 (Turkey)	POS	POS	POS	POS	POS	POS	POS	POS	NEG	POS	POS	POS	POS	POS	POS	POS	NEG	NEG
>"Eurasian Clade" : T013																		
T0131 (China)	POS	POS	POS	POS	POS	POS	POS	POS	NEG	POS	POS	POS	POS	POS	POS	POS	NEG	NEG
MU4 (Turkey)	POS	POS	POS	POS	POS	POS	POS	POS	NEG	POS	POS	POS	POS	POS	POS	POS	NEG	NEG
DEU11 (Turkey)	POS	POS	POS	POS	POS	POS	POS	POS	NEG	POS	POS	POS	POS	POS	POS	POS	NEG	NEG
DEU16 (Turkey)	POS	POS	POS	POS	POS	POS	POS	POS	NEG	POS	POS	POS	POS	POS	POS	POS	NEG	NEG
Romania_Iasi_MRSA-07_54	POS	POS	POS	POS	POS	POS	POS	POS	NEG	POS	POS	POS	POS	POS	POS	POS	NEG	NEG
>"Eurasian Clade": arsC-																		
Romania_Iasi_MRSA-05_46	POS	POS	POS	POS	POS	POS	POS	POS	NEG	POS	POS	POS	POS	POS	POS	POS	NEG	NEG
Romania_Iasi_MRSA-06_47	POS	POS	POS	POS	POS	POS	POS	POS	NEG	POS	POS	POS	POS	POS	POS	POS	NEG	NEG
>"Eurasian Clade": 16K/																		
CN79 (China)	NEG	POS	POS	POS	POS	POS	POS	POS	NEG	POS	POS	POS	POS	POS	POS	POS	NEG	NEG
CUHK_BJ2002 (China)	POS	POS	POS	POS	POS	POS	POS	POS	NEG	POS	POS	POS	POS	POS	POS	POS	NEG	NEG
CUHK_BJ2007 (China)	POS	POS	POS	POS	POS	POS	POS	POS	NEG	POS	POS	POS	POS	POS	POS	POS	NEG	NEG
3HK (Czech Republic)	POS	POS	POS	POS	POS	POS	POS	POS	NEG	POS	POS	POS	POS	POS	POS	POS	NEG	NEG
HU109 (Hungary)	POS	POS	POS	POS	POS	POS	POS	POS	NEG	POS	POS	POS	POS	POS	POS	POS	NEG	NEG
HUR18 (Hungary)	POS	POS	POS	POS	POS	POS	POS	POS	NEG	POS	POS	POS	POS	POS	POS	POS	NEG	NEG
H482 (Romania)	POS	POS	POS	POS	POS	POS	POS	POS	NEG	POS	POS	POS	POS	POS	POS	POS	NEG	NEG
16K (Russia)	POS	POS	POS	POS	POS	POS	POS	POS	NEG	POS	POS	POS	POS	POS	POS	POS	NEG	NEG
DEU10 (Turkey)	POS	POS	POS	POS	POS	POS	POS	POS	NEG	POS	POS	POS	POS	POS	POS	POS	NEG	NEG
DEU12 (Turkey)	POS	POS	POS	POS	POS	POS	POS	POS	NEG	POS	POS	POS	POS	POS	POS	POS	NEG	NEG
DEU14 (Turkey)	POS	POS	POS	POS	POS	POS	POS	POS	NEG	POS	POS	POS	POS	POS	POS	POS	NEG	NEG
DEU15 (Turkey)	POS	POS	POS	POS	POS	POS	POS	POS	NEG	POS	POS	POS	POS	POS	POS	POS	NEG	NEG
DEU17 (Turkey)	POS	POS	POS	POS	POS	POS	POS	POS	NEG	POS	POS	POS	POS	POS	POS	POS	NEG	NEG
DEU19 (Turkey)	POS	POS	POS	POS	POS	POS	POS	POS	NEG	POS	POS	POS	POS	POS	POS	POS	NEG	NEG
DEU2 (Turkey)	POS	POS	POS	POS	POS	POS	POS	POS	NEG	POS	POS	POS	POS	POS	POS	POS	NEG	NEG
DEU20 (Turkey)	POS	POS	POS	POS	POS	POS	POS	POS	NEG	POS	POS	POS	POS	POS	POS	POS	NEG	NEG
DEU23 (Turkey)	POS	POS	POS	POS	POS	POS	POS	POS	NEG	POS	POS	POS	POS	POS	POS	POS	NEG	NEG
DEU3 (Turkey)	POS	POS	POS	POS	POS	POS	POS	POS	NEG	POS	POS	POS	POS	POS	POS	POS	NEG	NEG
DEU5 (Turkey)	POS	POS	POS	POS	POS	POS	POS	POS	NEG	POS	POS	POS	POS	POS	POS	POS	NEG	NEG
DEU6 (Turkey)	POS	POS	POS	POS	POS	POS	POS	POS	NEG	POS	POS	POS	POS	POS	POS	POS	NEG	NEG
DEU8 (Turkey)	POS	POS	POS	POS	POS	POS	POS	POS	NEG	POS	POS	POS	POS	POS	POS	POS	NEG	NEG
HU11 (Turkey)	POS	POS	POS	POS	POS	POS	POS	POS	NEG	POS	POS	POS	POS	POS	POS	POS	NEG	NEG
HU13 (Turkey)	POS	POS	POS	POS	POS	POS	POS	POS	NEG	POS	POS	POS	POS	POS	POS	POS	NEG	NEG
HU14 (Turkey)	POS	POS	POS	POS	POS	POS	POS	POS	NEG	POS	POS	POS	POS	POS	POS	POS	NEG	NEG
HU15 (Turkey)	POS	POS	POS	POS	POS	POS	POS	POS	NEG	POS	POS	POS	POS	POS	POS	POS	NEG	NEG
HU16 (Turkey)	POS	POS	POS	POS	POS	POS	POS	POS	NEG	POS	POS	POS	POS	POS	POS	POS	NEG	NEG
HU17 (Turkey)	POS	POS	POS	POS	POS	POS	POS	POS	NEG	POS	POS	POS	POS	POS	POS	POS	NEG	NEG
HU21 (Turkey)	POS	POS	POS	POS	POS	POS	POS	POS	NEG	POS	POS	POS	POS	POS	POS	POS	NEG	NEG
HU23 (Turkey)	POS	POS	POS	POS	POS	POS	POS	POS	NEG	POS	POS	POS	POS	POS	POS	POS	NEG	NEG
HU26 (Turkey)	POS	POS	POS	POS	POS	POS	POS	POS	NEG	POS	POS	POS	POS	POS	POS	POS	NEG	NEG
HU4 (Turkey)	POS	POS	POS	POS	POS	POS	POS	POS	NEG	POS	POS	POS	POS	POS	POS	POS	NEG	NEG
HU5 (Turkey)	POS	POS	POS	POS	POS	POS	POS	POS	NEG	POS	POS	POS	POS	POS	POS	POS	NEG	NEG
HU6 (Turkey)	POS	POS	POS	POS	POS	POS	POS	POS	NEG	POS	POS	POS	POS	POS	POS	POS	NEG	NEG
HU7 (Turkey)	POS	POS	POS	POS	POS	POS	POS	POS	NEG	POS	POS	POS	POS	POS	POS	POS	NEG	NEG
HU8 (Turkey)	NEG	POS	POS	POS	POS	POS	POS	POS	NEG	POS	POS	POS	POS	POS	POS	POS	NEG	NEG
HU9 (Turkey)	POS	POS	POS	POS	POS	POS	POS	POS	NEG	POS	POS	POS	POS	POS	POS	POS	NEG	NEG
IU1 (Turkey)	POS	POS	POS	POS	POS	POS	POS	POS	NEG	POS	POS	POS	POS	POS	POS	POS	NEG	NEG
IU10 (Turkey)	POS	POS	POS	POS	POS	POS	POS	POS	NEG	POS	POS	POS	POS	POS	POS	POS	NEG	NEG
IU11 (Turkey)	NEG	POS	POS	POS	POS	POS	POS	POS	NEG	POS	POS	POS	POS	POS	POS	POS	NEG	NEG
IU12 (Turkey)	POS	POS	POS	POS	POS	POS	POS	POS	NEG	POS	POS	POS	POS	POS	POS	POS	NEG	NEG
IU13 (Turkey)	POS	POS	POS	POS	POS	POS	POS	POS	NEG	POS	POS	POS	POS	POS	POS	POS	NEG	NEG
IU15 (Turkey)	POS	POS	POS	POS	POS	POS	POS	POS	NEG	POS	POS	POS	POS	POS	POS	POS	NEG	NEG
IU18 (Turkey)	POS	POS	POS	POS	POS	POS	POS	POS	NEG	POS	POS	POS	POS	POS	POS	POS	NEG	NEG
IU19 (Turkey)	POS	POS	POS	POS	POS	POS	POS	POS	NEG	POS	POS	POS	POS	POS	POS	POS	NEG	NEG
IU2 (Turkey)	POS	POS	POS	POS	POS	POS	POS	POS	NEG	POS	POS	POS	POS	POS	POS	POS	NEG	NEG
IU4 (Turkey)	POS	POS	POS	POS	POS	POS	POS	POS	NEG	POS	POS	POS	POS	POS	POS	POS	NEG	NEG
IU6 (Turkey)	POS	POS	POS	POS	POS	POS	POS	POS	NEG	POS	POS	POS	POS	POS	POS	POS	NEG	NEG
IU7 (Turkey)	NEG	POS	POS	POS	POS	POS	POS	POS	NEG	POS	POS	POS	POS	POS	POS	POS	NEG	NEG
IU9 (Turkey)	POS	POS	POS	POS	POS	POS	POS	POS	NEG	POS	POS	POS	POS	POS	POS	POS	NEG	NEG
MU1 (Turkey)	POS	POS	POS	POS	POS	POS	POS	POS	NEG	POS	POS	POS	POS	POS	POS	POS	NEG	NEG
MU10 (Turkey)	POS	POS	POS	POS	POS	POS	POS	POS	NEG	POS	POS	POS	POS	POS	POS	POS	NEG	NEG
MU20 (Turkey)	POS	POS	POS	POS	POS	POS	POS	POS	NEG	POS	POS	POS	POS	POS	POS	POS	NEG	NEG
Mu3 (Turkey)	POS	POS	POS	POS	POS	POS	POS	POS	NEG	POS	POS	POS	POS	POS	POS	POS	NEG	NEG
MU5 (Turkey)	POS	POS	POS	POS	POS	POS	POS	POS	NEG	POS	POS	POS	POS	POS	POS	POS	NEG	NEG
MU6 (Turkey)	POS	POS	POS	POS	POS	POS	POS	POS	NEG	POS	POS	POS	POS	POS	POS	POS	NEG	NEG
MU7 (Turkey)	POS	POS	POS	POS	POS	POS	POS	POS	NEG	POS	POS	POS	POS	POS	POS	POS	NEG	NEG
TUR27 (Turkey)	POS	POS	POS	POS	POS	POS	POS	POS	NEG	POS	POS	POS	POS	POS	POS	POS	NEG	NEG
NCTR 32S	POS	POS	POS	POS	POS	POS	POS	POS	NEG	POS	POS	POS	POS	POS	POS	POS	NEG	NEG
SA02_A4 = Russia-06_0085_Moscow	POS	POS	POS	POS	POS	POS	POS	POS	NEG	POS	POS	POS	POS	POS	POS	POS	NEG	NEG
	POS	POS	POS	POS	POS	POS	POS	POS	NEG	POS	POS	POS	POS	POS	POS	POS	NEG	NEG
Dresden_08V35987 (Turkey)	POS	POS	POS	POS	POS	POS	POS	POS	NEG	POS	POS	POS	POS	POS	POS	POS	NEG	NEG
Dresden_17ANR577152	POS	POS	POS	POS	POS	POS	POS	POS	NEG	POS	POS	POS	POS	POS	POS	POS	NEG	NEG
Dresden_17ANR580374 (Makedonia)	POS	POS	POS	POS	POS	POS	POS	POS	NEG	POS	POS	POS	POS	POS	POS	POS	NEG	NEG
Hong Kong_69-II	POS	POS	POS	POS	POS	POS	POS	POS	NEG	POS	POS	POS	POS	POS	POS	POS	NEG	NEG
Hong Kong_93	POS	POS	POS	POS	POS	POS	POS	POS	NEG	POS	POS	POS	POS	POS	POS	POS	NEG	NEG
Rawalpindi_Kidney Center_03_SK1	POS	POS	POS	POS	POS	POS	POS	POS	NEG	POS	POS	POS	POS	POS	POS	POS	NEG	NEG
Rawalpindi_Kidney Center_08	POS	POS	POS	POS	POS	POS	POS	POS	NEG	POS	POS	POS	POS	POS	POS	POS	NEG	NEG
Rawalpindi_Kidney Center_10	POS	POS	POS	POS	POS	POS	POS	POS	NEG	POS	POS	POS	POS	POS	POS	POS	NEG	NEG
Rawalpindi_Kidney Center_19	POS	POS	POS	POS	POS	POS	POS	POS	NEG	POS	POS	POS	POS	POS	POS	POS	NEG	NEG
Rawalpindi_Kidney Center_50_SK1	POS	POS	POS	POS	POS	POS	POS	POS	NEG	POS	POS	POS	POS	POS	POS	POS	NEG	NEG
Romania_Iasi_BC-49_430	POS	POS	POS	POS	POS	POS	POS	POS	NEG	POS	POS	POS	POS	POS	POS	POS	NEG	NEG
Romania_Iasi_MRSA_284	POS	POS	POS	AMB	POS	POS	POS	AMB	NEG	POS	AMB	POS	POS	POS	POS	POS	NEG	NEG
Romania_Iasi_MRSA-32_318	POS	POS	POS	POS	POS	POS	POS	POS	NEG	POS	POS	POS	POS	POS	POS	POS	NEG	NEG
Romania_Iasi_MRSA-54_430	POS	POS	POS	POS	POS	POS	POS	POS	NEG	POS	POS	POS	POS	POS	POS	POS	NEG	NEG
Romania_Iasi_SSTI-01_2	POS	POS	POS	POS	POS	POS	POS	POS	NEG	POS	POS	POS	POS	POS	POS	POS	NEG	NEG
Romania_Iasi_SSTI-10_101	POS	POS	POS	POS	POS	POS	POS	POS	NEG	POS	POS	POS	POS	POS	POS	POS	NEG	NEG
Romania_Iasi_SSTI-11_106	POS	POS	POS	POS	POS	POS	POS	POS	NEG	POS	POS	POS	POS	POS	POS	POS	NEG	NEG
Russia-01_0001_SaintPetersburg	POS	POS	POS	POS	POS	POS	POS	POS	NEG	POS	POS	POS	POS	POS	POS	POS	NEG	NEG
Russia-02_0004_SaintPetersburg	POS	POS	POS	POS	POS	POS	POS	POS	NEG	POS	POS	POS	POS	POS	POS	POS	NEG	NEG
Russia-03_0057_SaintPetersburg	POS	POS	POS	POS	POS	POS	POS	POS	NEG	POS	POS	POS	POS	POS	POS	POS	NEG	NEG
Russia-04_0076_Moscow	POS	POS	POS	POS	POS	POS	POS	POS	NEG	POS	POS	POS	POS	POS	POS	POS	NEG	NEG
Russia-05_0078_Moscow	POS	POS	POS	POS	POS	POS	POS	POS	NEG	POS	POS	POS	POS	POS	POS	POS	NEG	NEG
Russia-08_0135_Moscow	POS	POS	POS	POS	POS	POS	POS	POS	NEG	POS	POS	POS	POS	POS	POS	POS	NEG	NEG
Russia-09_0150_Kurgan	POS	POS	POS	POS	POS	POS	POS	POS	NEG	POS	POS	POS	POS	POS	POS	POS	NEG	NEG
Russia-10_0162_Kurgan	POS	POS	POS	POS	POS	POS	POS	POS	NEG	POS	POS	POS	POS	POS	POS	POS	NEG	NEG
Russia-14_0184_Moscow	POS	POS	POS	AMB	POS	POS	POS	POS	NEG	POS	POS	POS	POS	POS	POS	POS	NEG	NEG
Russia-15_0232_SaintPetersburg	POS	POS	POS	POS	POS	POS	POS	POS	NEG	POS	POS	POS	POS	POS	POS	POS	NEG	NEG
Russia-22_0391_Chelyabinsk	POS	POS	POS	POS	POS	POS	POS	POS	NEG	POS	POS	POS	POS	POS	POS	POS	NEG	NEG
Russia-23_0392_Chelyabinsk	POS	POS	POS	POS	POS	POS	POS	POS	NEG	POS	POS	POS	POS	POS	POS	POS	NEG	NEG
>"Eurasian Clade" : IU17																		
IU17 (Turkey)	POS	POS	POS	POS	POS	POS	POS	POS	NEG	POS	POS	POS	POS	POS	POS	POS	NEG	NEG
IU20 (Turkey)	POS	POS	POS	POS	POS	POS	POS	POS	NEG	POS	POS	POS	POS	POS	POS	POS	NEG	NEG

STRAIN / ISOLATE	SPECIES MARKER							STAPHYLOXANTHIN BIOSYNTH. OPERON					REGULATORY GENES					
	rrnD1	gapA	katA	CoA	nuc1	spa	sbi	crtM		crtN	crtO	crtP	sarA	saeS	vraS	agrI	agrII	agrIII
	rrnD1 (S. aureus)	gapA	katA	CoA	nuc1	spa	sbi	crtM-nonST93	crtM-ST93	crtN	crtO	crtP	sarA	saeS	vraS			
	Domain 1 of 23S-rRNA	glyceraldehyde 3-phosphate dehydrogenase, locus 1	katalase A	coagulase	thermostable extracellular nuclease	staphylococcal protein A	IgG-binding protein	dehydrosqualene synthase		dehydrosqualene desaturase	staphyloxanthin acyltransferase	diaponeurosporene oxidase	staphylococcal accessory regulator A	histidine protein kinase, sae locus	sensor protein	accessory gene regulator allele I	accessory gene regulator allele II	accessory gene regulator allele III
**>"Eurasian Clade": Spor[illegible]**																		
Dresden_16ANR581769	POS	POS	POS	POS	POS	POS	POS	POS	NEG	POS	POS	POS	POS	POS	POS	POS	NEG	NEG
Dresden_17ANR578769	POS	POS	POS	POS	POS	POS	POS	POS	NEG	POS	POS	POS	POS	POS	POS	POS	NEG	NEG
**>"Eurasian Clade": Kuw[illegible]**																		
Kuwait_306	POS	POS	POS	POS	POS	POS	POS	POS	NEG	POS	POS	POS	POS	POS	POS	POS	NEG	NEG
Kuwait_301	POS	POS	POS	POS	POS	POS	POS	POS	NEG	POS	POS	POS	POS	POS	POS	POS	NEG	NEG
**>"European Clade": "Gr[illegible]**																		
GRE18 (Greece)	POS	POS	POS	POS	POS	POS	POS	POS	NEG	POS	POS	POS	POS	POS	POS	POS	NEG	NEG
GRE317 (Greece)	POS	POS	POS	POS	POS	POS	POS	POS	NEG	POS	POS	POS	POS	POS	POS	POS	NEG	NEG
GRE4 (Greece)	POS	POS	POS	POS	POS	POS	POS	POS	NEG	POS	POS	POS	POS	POS	POS	POS	NEG	NEG
R35 (USA)	POS	POS	POS	POS	POS	POS	POS	POS	NEG	POS	POS	POS	POS	POS	POS	POS	NEG	NEG
Casablanca_HT20060548	POS	POS	POS	POS	POS	POS	POS	POS	NEG	POS	POS	POS	POS	POS	POS	POS	NEG	NEG
Casablanca_HT20060550	POS	POS	POS	POS	POS	POS	NEG	POS	NEG	POS	POS	POS	POS	POS	POS	POS	NEG	NEG
Dresden_01V34572 (Greece)	POS	POS	POS	POS	POS	POS	POS	POS	NEG	POS	POS	POS	POS	POS	POS	POS	NEG	NEG
Dresden_01V36469	POS	POS	POS	POS	POS	POS	POS	POS	NEG	POS	POS	POS	POS	POS	POS	POS	NEG	NEG
Dresden_01V39123	POS	POS	POS	POS	POS	POS	POS	POS	NEG	POS	POS	POS	POS	POS	POS	POS	NEG	NEG
Dresden_01V39143	POS	POS	POS	POS	POS	POS	POS	POS	NEG	POS	POS	POS	POS	POS	POS	POS	NEG	NEG
Greece 1_3680_Harmony	POS	POS	POS	POS	POS	POS	POS	POS	NEG	POS	POS	POS	POS	POS	POS	POS	NEG	NEG
**>"European Clade": UK-[illegible]**																		
ANS46 (Australia)	POS	POS	POS	POS	POS	POS	POS	POS	NEG	POS	POS	POS	POS	POS	POS	POS	NEG	NEG
LHH1 (USA)	POS	POS	POS	POS	POS	POS	POS	POS	NEG	POS	POS	POS	POS	POS	POS	POS	NEG	NEG
Dublin-DSH_AR01_0-1085	POS	POS	POS	POS	POS	POS	POS	POS	NEG	POS	POS	POS	POS	POS	POS	POS	NEG	NEG
Dublin-DSH_AR01_0-1118	POS	POS	POS	POS	POS	POS	POS	POS	NEG	POS	POS	POS	POS	POS	POS	POS	NEG	NEG
Dublin-DSH_AR15_0098	POS	POS	POS	POS	POS	POS	POS	POS	NEG	POS	POS	POS	POS	POS	POS	POS	NEG	NEG
Dublin-DSH_AR15_0104	POS	POS	POS	POS	POS	POS	POS	POS	NEG	POS	POS	POS	POS	POS	POS	POS	NEG	NEG
UK_NCTC11939_UK-1_Harmony	POS	POS	POS	POS	POS	POS	POS	POS	NEG	POS	POS	POS	POS	POS	POS	POS	NEG	NEG
**>"European Clade": BK2[illegible]**																		
BK2421 (USA)	POS	POS	POS	POS	POS	POS	POS	POS	NEG	POS	POS	POS	POS	POS	POS	POS	NEG	NEG
**>"Aussi/NZ Clade", JKD[illegible]**																		
JKD6009 (Australia)	NEG	POS	POS	POS	POS	POS	POS	POS	NEG	POS	POS	POS	POS	POS	POS	POS	NEG	NEG
JKD6008 (New Zealand)	POS	POS	POS	POS	POS	POS	POS	POS	NEG	POS	POS	POS	POS	POS	POS	POS	NEG	NEG
Perth_2015-S20209	POS	POS	POS	POS	POS	POS	POS	POS	NEG	POS	POS	POS	POS	POS	POS	POS	NEG	NEG
Perth_2015-S20305	POS	POS	POS	POS	POS	POS	POS	POS	NEG	POS	POS	POS	POS	POS	POS	POS	NEG	NEG
Perth_2015-S20603	POS	POS	POS	POS	POS	POS	POS	POS	NEG	POS	POS	POS	POS	POS	POS	POS	NEG	NEG
Perth_2015-S20607	POS	POS	POS	POS	POS	POS	POS	POS	NEG	POS	POS	POS	POS	POS	POS	POS	NEG	NEG
Perth_2015-S20608	POS	POS	POS	POS	POS	POS	POS	POS	NEG	POS	POS	POS	POS	POS	POS	POS	NEG	NEG
Perth_2015-S20610	POS	POS	POS	POS	POS	POS	POS	POS	NEG	POS	POS	POS	POS	POS	POS	POS	NEG	NEG
Perth_2015-S20613	POS	POS	POS	POS	POS	POS	POS	POS	NEG	POS	POS	POS	POS	POS	POS	POS	NEG	NEG
Perth_2015-S20617	POS	POS	POS	POS	POS	POS	POS	POS	NEG	POS	POS	POS	POS	POS	POS	POS	NEG	NEG
Perth_2015-S20622	POS	POS	POS	POS	POS	POS	POS	POS	NEG	POS	POS	POS	NEG	POS	POS	NEG	NEG	NEG
Perth_2015-S23619	POS	POS	POS	POS	POS	POS	POS	POS	NEG	POS	POS	POS	POS	POS	POS	POS	NEG	NEG
Perth_2015-S41210	POS	POS	POS	POS	POS	POS	POS	POS	NEG	POS	POS	POS	POS	POS	POS	POS	NEG	NEG
Perth_2015-S51001	POS	POS	POS	POS	POS	POS	POS	POS	NEG	POS	POS	POS	POS	POS	POS	POS	NEG	NEG
Perth_2015-S51003	POS	POS	POS	POS	POS	POS	POS	POS	NEG	POS	POS	POS	POS	POS	POS	POS	NEG	NEG
Perth_2015-S51006	POS	POS	POS	POS	POS	POS	POS	POS	NEG	POS	POS	POS	POS	POS	POS	POS	NEG	NEG
Perth_2015-S51007	POS	POS	POS	POS	POS	POS	POS	POS	NEG	POS	POS	POS	POS	POS	POS	POS	NEG	NEG
Perth_2015-S51008	POS	POS	POS	POS	POS	POS	POS	POS	NEG	POS	POS	POS	POS	POS	POS	POS	NEG	NEG
Perth_2015-S51014	POS	POS	POS	POS	POS	POS	POS	POS	NEG	POS	POS	POS	POS	POS	POS	POS	NEG	NEG
Perth_2015-S51408	POS	POS	POS	POS	POS	POS	POS	POS	NEG	POS	POS	POS	POS	POS	POS	POS	NEG	NEG
Perth_2016-S20614	POS	POS	POS	POS	POS	POS	POS	POS	NEG	POS	POS	POS	POS	POS	POS	POS	NEG	NEG
Perth_2016-S23614	POS	POS	POS	POS	POS	POS	POS	POS	NEG	POS	POS	POS	POS	POS	POS	POS	NEG	NEG
Perth_2016-S42009	POS	POS	POS	POS	NEG	POS	POS	POS	NEG	POS	POS	POS	POS	POS	POS	POS	NEG	NEG
Perth_2016-S51001	POS	POS	POS	POS	POS	POS	POS	POS	NEG	POS	POS	POS	POS	POS	POS	POS	NEG	NEG
Perth_2016-S51403	POS	POS	POS	POS	POS	POS	POS	POS	NEG	POS	POS	POS	POS	POS	POS	POS	NEG	NEG
Perth_07 RPA 20	POS	POS	POS	POS	POS	POS	POS	POS	NEG	POS	POS	POS	POS	POS	POS	POS	NEG	NEG
Perth_08-19088	POS	POS	POS	POS	POS	POS	POS	POS	NEG	POS	POS	POS	POS	POS	POS	POS	NEG	NEG
Perth_01-15351	POS	POS	POS	POS	POS	POS	POS	POS	NEG	POS	POS	POS	POS	POS	POS	POS	NEG	NEG
Perth_01-15357	POS	POS	POS	POS	POS	POS	POS	POS	NEG	POS	POS	POS	POS	POS	POS	POS	NEG	NEG
Perth_01-15419	POS	POS	POS	POS	POS	POS	POS	POS	NEG	POS	POS	POS	POS	POS	POS	POS	NEG	NEG
Perth_01-16309	POS	POS	POS	POS	POS	POS	POS	POS	NEG	POS	POS	POS	POS	POS	POS	POS	NEG	NEG
Perth_03 SNP 95	POS	POS	POS	POS	POS	POS	POS	POS	NEG	POS	POS	POS	POS	POS	POS	POS	NEG	NEG
Perth_2003_AH_47	POS	POS	POS	POS	POS	POS	POS	POS	NEG	POS	POS	POS	POS	POS	POS	POS	NEG	NEG
**>"Australian/NZ Clade":**																		
Perth_2015-S20623	POS	POS	POS	POS	POS	POS	POS	POS	NEG	POS	POS	POS	POS	POS	POS	POS	NEG	NEG
Perth_2015-S20826	POS	POS	POS	POS	POS	POS	POS	POS	NEG	POS	POS	POS	POS	POS	POS	POS	NEG	NEG
Perth_05 NH 56	POS	POS	POS	POS	POS	POS	POS	POS	NEG	POS	POS	POS	POS	POS	POS	POS	NEG	NEG
**>CC5-MRSA-III LA MRSA**																		
Delbrück 38 isolate from domestic turkey	POS	POS	POS	POS	POS	POS	POS	POS	NEG	POS	POS	POS	POS	POS	POS	NEG	POS	NEG
**> Staph. pseudintermed**																		
KM1381	NEG	NEG	NEG	NEG	NEG	NEG	NEG	NEG	NEG	NEG	NEG	NEG			NEG	NEG	NEG	NEG
**>"South-East Asian Clad[illegible]**																		
M92 (Canada)	POS	POS	POS	POS	POS	POS	POS	POS	NEG	POS	POS	POS	POS	POS	POS	POS	NEG	NEG
CHI59 (China)	POS	POS	POS	POS	POS	POS	POS	POS	NEG	POS	POS	POS	POS	POS	POS	POS	NEG	NEG
CHI61 (China)	POS	POS	POS	POS	POS	POS	POS	POS	NEG	POS	POS	POS	POS	POS	POS	POS	NEG	NEG
CUHK_HK1997 (China)	POS	POS	POS	POS	POS	POS	POS	POS	NEG	POS	POS	POS	POS	POS	POS	POS	NEG	NEG
M996 (China)	POS	POS	POS	POS	POS	POS	POS	POS	NEG	POS	POS	POS	POS	POS	POS	POS	NEG	NEG
H211 (Denmark)	POS	POS	POS	POS	POS	POS	POS	POS	NEG	POS	POS	POS	POS	POS	POS	POS	NEG	NEG
H216 (Denmark)	POS	POS	POS	POS	POS	POS	POS	POS	NEG	POS	POS	POS	POS	POS	POS	POS	NEG	NEG
D71 (Germany)	POS	POS	POS	POS	POS	POS	POS	POS	NEG	POS	POS	POS	POS	POS	POS	POS	NEG	NEG
NMR05 (India)	POS	POS	POS	POS	POS	POS	POS	POS	NEG	POS	POS	POS	POS	POS	POS	POS	NEG	NEG
MAL11 (Malaysia)	POS	POS	POS	POS	POS	POS	POS	POS	NEG	POS	POS	POS	POS	POS	POS	POS	NEG	NEG
MAL9 (Malaysia)	POS	POS	POS	POS	POS	POS	POS	POS	NEG	POS	POS	POS	POS	POS	POS	POS	NEG	NEG
Na21 (Sri Lanka)	POS	POS	POS	POS	POS	POS	POS	POS	NEG	POS	POS	POS	POS	POS	POS	POS	NEG	NEG
M592 (Syria)	POS	POS	POS	POS	POS	POS	POS	POS	NEG	POS	POS	POS	POS	POS	POS	POS	NEG	NEG
H202 (Thailand)	NEG	POS	POS	POS	POS	POS	POS	POS	NEG	POS	POS	POS	POS	POS	POS	POS	NEG	NEG
S102 (Thailand)	POS	POS	POS	POS	POS	POS	POS	POS	NEG	POS	POS	POS	POS	POS	POS	POS	NEG	NEG
S130 (Thailand)	POS	POS	POS	POS	POS	POS	POS	POS	NEG	POS	POS	POS	POS	POS	POS	POS	NEG	NEG
S40 (Thailand)	POS	POS	POS	POS	POS	POS	POS	POS	NEG	POS	POS	POS	POS	POS	POS	POS	NEG	NEG
S71 (Thailand)	POS	POS	POS	POS	POS	POS	POS	POS	NEG	POS	POS	POS	POS	POS	POS	POS	NEG	NEG
S87 (Thailand)	POS	POS	POS	POS	POS	POS	POS	POS	NEG	POS	POS	POS	POS	POS	POS	POS	NEG	NEG
S93 (Thailand)	POS	POS	POS	POS	POS	POS	POS	POS	NEG	POS	POS	POS	POS	POS	POS	POS	NEG	NEG
US_002 (Thailand)	POS	POS	POS	POS	POS	POS	POS	POS	NEG	POS	POS	POS	POS	POS	POS	POS	NEG	NEG
US_017 (Thailand)	POS	POS	POS	POS	POS	POS	POS	POS	NEG	POS	POS	POS	POS	POS	POS	POS	NEG	NEG
WAMC6102 (Thailand)	POS	POS	POS	POS	POS	POS	POS	POS	NEG	POS	POS	POS	POS	POS	POS	POS	NEG	NEG
NCTC_13626_TW20 (UK)	POS	POS	POS	POS	POS	POS	POS	POS	NEG	POS	POS	POS	POS	POS	POS	POS	NEG	NEG
BSAC3021 (UK)	POS	POS	POS	POS	POS	POS	POS	POS	NEG	POS	POS	POS	POS	POS	POS	POS	NEG	NEG
BSAC3046 (UK)	POS	POS	POS	POS	POS	POS	POS	POS	NEG	POS	POS	POS	POS	POS	POS	POS	NEG	NEG
1193_SAUR (USA)	POS	POS	POS	POS	POS	POS	POS	POS	NEG	POS	POS	POS	POS	POS	POS	POS	NEG	NEG
1194_SAUR (USA)	POS	POS	POS	POS	POS	POS	POS	POS	NEG	POS	POS	POS	POS	POS	POS	POS	NEG	NEG

STRAIN / ISOLATE	SPECIES MARKER							STAPHYLOXANTHIN BIOSYNTH. OPERON					REGULATORY GENES					
	rrnD1	gapA	katA	CoA	nuc1	spa	sbi	crtM		crtN	crtO	crtP	sarA	saeS	vraS	agrI	agrII	agrIII
	rrnD1 (S. aureus)	gapA	katA	CoA	nuc1	spa	sbi	crtM-nonST93	crtM-ST93	crtN	crtO	crtP	sarA	saeS	vraS			
	Domain 1 of 23S-rRNA	glyceraldehyde 3-phosphate dehydrogenase, locus 1	katalase A	coagulase	thermostable extracellular nuclease	staphylococcal protein A	IgG-binding protein	dehydrosqualene synthase		dehydrosqualene desaturase	staphyloxanthin acyltransferase	diaponeurosporene oxidase	staphylococcal accessory regulator A	histidine protein kinase, sae locus	sensor protein	accessory gene regulator allele I	accessory gene regulator allele II	accessory gene regulator allele III
>"South-East Asian Clade": TW20 (Irish AR44), c																		
1195_SAUR (USA)	POS	POS	POS	POS	POS	POS	POS	POS	NEG	POS	POS	POS	POS	POS	POS	POS	NEG	NEG
FVRH6002 (USA)	POS	POS	POS	POS	POS	POS	POS	POS	NEG	POS	POS	POS	POS	POS	POS	POS	NEG	NEG
KINW6048 (USA)	POS	POS	POS	POS	POS	POS	POS	POS	NEG	POS	POS	POS	POS	POS	POS	POS	NEG	NEG
LAMC0011 (USA)	POS	POS	POS	POS	POS	POS	POS	POS	NEG	POS	POS	POS	POS	POS	POS	POS	NEG	NEG
SJOS6053 (USA)	POS	POS	POS	POS	POS	POS	POS	POS	NEG	POS	POS	POS	POS	POS	POS	POS	NEG	NEG
SJOS6072 (USA)	POS	POS	POS	POS	POS	POS	POS	POS	NEG	POS	POS	POS	POS	POS	POS	POS	NEG	NEG
UCIM6042 (USA)	POS	POS	POS	POS	POS	POS	POS	POS	NEG	POS	POS	POS	POS	POS	POS	POS	NEG	NEG
Perth_2015-S31902	POS	POS	POS	POS	POS	POS	POS	POS	NEG	POS	POS	POS	NEG	POS	POS	POS	NEG	NEG
Perth_2015-S31904	POS	POS	POS	POS	POS	POS	POS	POS	NEG	POS	POS	POS	POS	POS	POS	POS	NEG	NEG
Perth_2015-S31913	POS	POS	POS	POS	POS	POS	POS	POS	NEG	POS	POS	POS	POS	POS	POS	POS	NEG	NEG
Perth_2015-S31918	POS	POS	POS	POS	POS	POS	POS	POS	NEG	POS	POS	POS	NEG	NEG	POS	POS	NEG	NEG
Dublin-DSH_AR44_E1183	POS	POS	POS	POS	POS	POS	POS	POS	NEG	POS	POS	POS	POS	POS	POS	POS	NEG	NEG
Dublin-DSH_Unfamiliar-3_E1520	POS	POS	POS	POS	POS	POS	POS	POS	NEG	POS	POS	POS	POS	POS	POS	POS	NEG	NEG
Hong Kong_042	POS	POS	POS	POS	POS	POS	POS	POS	NEG	POS	POS	POS	POS	POS	POS	POS	NEG	NEG
Hong Kong_101	POS	POS	POS	POS	POS	POS	POS	POS	NEG	POS	POS	POS	POS	POS	POS	POS	NEG	NEG
Hong Kong_111	POS	POS	POS	POS	POS	POS	POS	POS	NEG	POS	POS	POS	POS	POS	POS	POS	NEG	NEG
Hong Kong_113	POS	POS	POS	POS	POS	POS	POS	POS	NEG	POS	POS	POS	POS	POS	POS	POS	NEG	NEG
Hong Kong_115	POS	POS	POS	POS	POS	POS	POS	POS	NEG	POS	POS	POS	POS	POS	POS	POS	NEG	NEG
Hong Kong_118	POS	POS	POS	POS	POS	POS	POS	POS	NEG	POS	POS	POS	POS	POS	POS	POS	NEG	NEG
Hong Kong_125	POS	POS	POS	POS	POS	POS	POS	POS	NEG	POS	POS	POS	POS	POS	POS	POS	NEG	NEG
Hong Kong_129	POS	POS	POS	POS	POS	POS	POS	POS	NEG	POS	POS	POS	POS	POS	POS	POS	NEG	NEG
Hong Kong_133	POS	POS	POS	POS	POS	NEG	POS	POS	NEG	POS	POS	POS	POS	POS	POS	POS	NEG	NEG
Kuwait_009	POS	POS	POS	POS	POS	POS	POS	POS	NEG	POS	POS	POS	POS	POS	POS	POS	NEG	NEG
Kuwait_103	POS	POS	POS	POS	POS	POS	POS	POS		POS	POS	POS	POS	POS	POS	POS	NEG	NEG
Kuwait_107	POS	POS	POS	POS	POS	POS	POS	POS	NEG	POS	POS	POS	POS	POS	POS	POS	NEG	NEG
Perth_03 AH 88	POS	POS	POS	POS	POS	POS	POS	POS	NEG	POS	POS	POS	POS	POS	POS	POS	NEG	NEG
Perth_08-16905	POS	POS	POS	POS	POS	POS	POS	POS	NEG	POS	POS	POS	POS	POS	POS	POS	NEG	NEG
Riyadh_Alfaisal-10_SICU_13_B5672_1117353	POS	POS	POS	POS	POS	POS	POS	POS	NEG	POS	POS	POS	POS	POS	POS	POS	NEG	NEG
Riyadh_SSTI_17_3508602	POS	POS	POS	POS	POS	POS	POS	POS	NEG	POS	POS	POS	POS	POS	POS	POS	NEG	NEG
Riyadh-2822088-R	POS	POS	POS	POS	POS	POS	POS	POS	NEG	POS	POS	POS	POS	POS	POS	POS	NEG	NEG
Trinidad&Tobago_2012_195 (619/12)	POS	POS	POS	POS	POS	POS	POS	POS	NEG	POS	POS	POS	POS	POS	POS	POS	NEG	NEG
Trinidad&Tobago_2012_370 (1112-52177)	POS	POS	POS	POS	POS	POS	POS	POS	NEG	POS	POS	POS	POS	POS	POS	POS	NEG	NEG
Trinidad&Tobago_2012_378 (4139)	POS	POS	POS	POS	POS	POS	POS	POS	NEG	POS	POS	POS	POS	POS	POS	POS	NEG	NEG
Trinidad&Tobago_2013_PA21	POS	POS	POS	POS	POS	POS	POS	POS	NEG	POS	POS	POS	POS	POS	POS	POS	NEG	NEG
Trinidad&Tobago_2013_PA22	POS	POS	POS	POS	POS	POS	POS	POS	NEG	POS	POS	POS	POS	POS	POS	POS	NEG	NEG
Trinidad&Tobago_2912_340 (8523/12)	POS	POS	POS	POS	POS	POS	POS	POS	NEG	POS	POS	POS	POS	POS	POS	POS	NEG	NEG
Trinidad&Tobago_MRSA2010_105	POS	POS	POS	POS	POS	POS	POS	POS	NEG	POS	POS	POS	POS	POS	POS	POS	NEG	NEG
Trinidad&Tobago_MRSA2010_108	POS	POS	POS	POS	POS	POS	POS	POS	NEG	POS	POS	POS	POS	POS	POS	POS	NEG	NEG
Trinidad&Tobago_MRSA2010_33	POS	POS	POS	POS	POS	POS	POS	POS	NEG	POS	POS	POS	POS	POS	POS	POS	NEG	NEG
Trinidad&Tobago_MRSA2010_79	POS	POS	POS	POS	POS	POS	POS	POS	NEG	POS	POS	POS	POS	POS	POS	POS	NEG	NEG
Trinidad&Tobago_MRSA2010_88	POS	POS	POS	POS	POS	POS	POS	POS	NEG	POS	POS	POS	POS	POS	POS	POS	NEG	NEG
Trinidad&Tobago_MRSA2010_97	POS	POS	POS	POS	POS	POS	POS	POS	NEG	POS	POS	POS	POS	POS	POS	POS	NEG	NEG
Trinidad&Tobago_SSTI 2012_031	POS	POS	POS	POS	POS	POS	POS	POS	NEG	POS	POS	POS	POS	POS	POS	POS	NEG	NEG
Uganda-29_17148_250811	POS	POS	POS	POS	POS	POS	POS	POS	NEG	POS	POS	POS	POS	POS	POS	POS	NEG	NEG
>"South-East Asian Clad																		
UCIM6015	POS	POS	POS	POS	POS	POS	POS	POS	NEG	POS	POS	POS	POS	POS	POS	POS	NEG	NEG
Hong Kong_121	POS	POS	POS	POS	POS	NEG	POS	POS	NEG	POS	POS	POS	POS	POS	POS	POS	NEG	NEG
>"South-East Asian Clad																		
Z172 (Taiwan)	POS	POS	POS	POS	POS	POS	POS	POS	NEG	POS	POS	POS	POS	POS	POS	POS	NEG	NEG
Russia-19_0342_Kurgan	POS	POS	POS	AMB	POS	POS	POS	POS	NEG	POS	POS	POS	POS	POS	POS	POS	NEG	NEG
>"South-East Asian Clad																		
Perth_01-15392	POS	POS	POS	POS	POS	POS	POS	POS	NEG	POS	POS	POS	POS	POS	POS	POS	NEG	NEG
>"South-East Asian Clad																		
XN108 (China)	POS	POS	POS	POS	POS	POS	POS	POS	NEG	POS	POS	POS	POS	POS	POS	POS	NEG	NEG
NMR09 (India)	POS	POS	POS	POS	POS	POS	POS	POS	NEG	POS	POS	POS	POS	POS	POS	POS	NEG	NEG
346 (Malaysia)	POS	POS	POS	POS	POS	POS	POS	POS	NEG	POS	POS	POS	POS	POS	POS	POS	NEG	NEG
DS_009 (Thailand)	POS	POS	POS	POS	POS	POS	POS	POS	NEG	POS	POS	POS	POS	POS	POS	POS	NEG	NEG
18ANR581005 (Ind)	POS	POS	POS	POS	POS	POS	POS	POS	NEG	POS	POS	POS	POS	POS	POS	POS	NEG	NEG
Bengalaru_ST20141405	POS	POS	POS	POS	POS	POS	POS	POS	NEG	POS	POS	POS	POS	POS	POS	POS	NEG	NEG
Bengalaru_ST20141406	POS	POS	POS	POS	POS	POS	POS	POS	NEG	POS	POS	POS	POS	POS	POS	POS	NEG	NEG
Bengalaru_ST20141417	POS	POS	POS	POS	POS	POS	POS	POS	NEG	POS	POS	POS	POS	POS	POS	POS	NEG	NEG
Hong Kong_107	POS	POS	POS	POS	POS	POS	POS	POS	NEG	POS	POS	POS	POS	POS	POS	POS	NEG	NEG
Hong Kong_12	POS	POS	POS	POS	POS	POS	POS	POS	NEG	POS	POS	POS	POS	POS	POS	POS	NEG	NEG
Hong Kong_132	POS	POS	POS	POS	POS	POS	POS	POS	NEG	POS	POS	POS	POS	POS	POS	POS	NEG	NEG
Riyadh-3028763-R	POS	POS	POS	POS	POS	POS	POS	POS	NEG	POS	POS	POS	POS	POS	POS	POS	NEG	NEG
Trinidad&Tobago_MRSA2010_94	POS	POS	POS	POS	POS	POS	POS	POS	NEG	POS	POS	POS	POS	POS	POS	POS	NEG	NEG
>"South-East Asian Clad																		
M705 (Thailand)	POS	POS	POS	POS	POS	POS	POS	POS	NEG	POS	POS	POS	POS	POS	POS	POS	NEG	NEG
S85 (Thailand)	POS	POS	POS	POS	POS	POS	POS	POS	NEG	POS	POS	POS	POS	POS	POS	POS	NEG	NEG
Riyadh_Alfaisal-04_281014_S5076	POS	POS	POS	POS	POS	POS	POS	POS	NEG	POS	POS	POS	POS	POS	POS	POS	NEG	NEG
Riyadh_Alfaisal-26_S15145_1104163	POS	POS	POS	POS	POS	POS	POS	POS	NEG	POS	POS	POS	POS	POS	POS	POS	NEG	NEG
Riyadh-2817437-W	POS	POS	POS	POS	POS	POS	POS	POS	NEG	POS	POS	POS	POS	POS	POS	POS	NEG	NEG
>"South-East Asian Clad																		
PPUKM-775-2009 (Malaysia)	POS	POS	POS	POS	POS	POS	POS	POS	NEG	POS	POS	POS	POS	POS	POS	POS	NEG	NEG
Hong Kong_138	POS	POS	POS	POS	POS	POS	POS	POS	NEG	POS	POS	POS	POS	POS	POS	POS	NEG	NEG
>"South-East Asian Clad																		
Kuwait_001	POS	POS	POS	POS	POS	POS	POS	POS	NEG	POS	POS	POS	POS	POS	POS	POS	NEG	NEG
Kuwait_337	POS	POS	POS	POS	POS	POS	POS	POS	NEG	POS	POS	POS	POS	POS	POS	POS	NEG	NEG
>"South-East Asian Clad																		
CUHK_HK2007 (China)	POS	POS	POS	POS	POS	POS	POS	POS	NEG	POS	POS	POS	POS	POS	POS	POS	NEG	NEG
NMR02 (India)	POS	POS	POS	POS	POS	POS	POS	POS	NEG	POS	POS	POS	POS	POS	POS	POS	NEG	NEG
VB1490 (India)	POS	POS	POS	POS	POS	POS	POS	POS	NEG	POS	POS	POS	POS	POS	POS	POS	NEG	NEG
V521 (Korea)	POS	POS	POS	POS	POS	POS	POS	POS	NEG	POS	POS	POS	POS	POS	POS	POS	NEG	NEG
HST-077 (Lebanon)	POS	POS	POS	POS	POS	POS	POS	POS	NEG	POS	POS	POS	POS	POS	POS	POS	NEG	NEG
MAL1 (Malaysia)	POS	POS	POS	POS	POS	POS	POS	POS	NEG	POS	POS	POS	POS	POS	POS	POS	NEG	NEG
MAL3 (Malaysia)	POS	POS	POS	POS	POS	POS	POS	POS	NEG	POS	POS	POS	POS	POS	POS	POS	NEG	NEG
PPUKM-261-2009 (Malaysia)	POS	POS	POS	POS	POS	POS	POS	POS	NEG	POS	POS	POS	POS	POS	POS	POS	NEG	NEG
PPUKM-332-2009 (Malaysia)	POS	POS	POS	POS	POS	POS	POS	POS	NEG	POS	POS	POS	POS	POS	POS	POS	NEG	NEG
S38 (Thailand)	POS	POS	POS	POS	POS	POS	POS	POS	NEG	POS	POS	POS	POS	POS	POS	POS	NEG	NEG
S7 (Thailand)	POS	POS	POS	POS	POS	POS	POS	POS	NEG	POS	POS	POS	POS	POS	POS	POS	NEG	NEG
US_008 (Thailand)	POS	POS	POS	POS	POS	POS	POS	POS	NEG	POS	POS	POS	POS	POS	POS	POS	NEG	NEG
US_030 (Thailand)	POS	POS	POS	POS	POS	POS	POS	POS	NEG	POS	POS	POS	POS	POS	POS	POS	NEG	NEG
M170 (Tsunami-related)	POS	POS	POS	POS	POS	POS	POS	POS	NEG	POS	POS	POS	POS	POS	POS	POS	NEG	NEG
BSAC697 (UK)	POS	POS	POS	POS	POS	POS	POS	POS	NEG	POS	POS	POS	POS	POS	POS	POS	NEG	NEG
IS-189	POS	POS	POS	POS	POS	POS	POS	POS	NEG	POS	POS	POS	POS	POS	POS	POS	NEG	NEG
MRGR3	NEG	POS	POS	POS	POS	POS	POS	POS	NEG	POS	POS	POS	POS	POS	POS	POS	NEG	NEG
smg07	POS	POS	POS	POS	POS	POS	POS	POS	NEG	POS	POS	POS	POS	POS	POS	POS	NEG	NEG
AH1-AUS-EMRSA-3	POS	POS	POS	POS	POS	POS	POS	POS	NEG	POS	POS	POS	POS	POS	POS	POS	NEG	NEG
Bengalaru_ST20141403	POS	POS	POS	POS	POS	POS	POS	POS	NEG	POS	POS	POS	POS	POS	POS	POS	NEG	NEG
Finland E24_98541_Harmony	POS	POS	POS	POS	POS	POS	POS	POS	NEG	POS	POS	POS	POS	POS	POS	POS	NEG	NEG
Hong Kong_10	POS	POS	POS	POS	POS	POS	POS	POS	NEG	POS	POS	POS	POS	POS	POS	POS	NEG	NEG
Hong Kong_100	POS	POS	POS	POS	POS	POS	POS	POS	NEG	POS	POS	POS	POS	POS	POS	POS	NEG	NEG
Hong Kong_199	POS	POS	POS	POS	POS	POS	POS	POS	NEG	POS	POS	POS	POS	POS	POS	POS	NEG	NEG
Hong Kong_34	POS	POS	POS	POS	POS	POS	POS	POS	NEG	POS	POS	POS	POS	POS	POS	POS	NEG	NEG
Hong Kong_88	POS	POS	POS	POS	POS	POS	POS	POS	NEG	POS	POS	POS	POS	POS	POS	POS	NEG	NEG
Hong Kong_92	POS	POS	POS	POS	POS	POS	POS	POS	NEG	POS	POS	POS	POS	POS	POS	POS	NEG	NEG
Hong Kong_94	POS	POS	POS	POS	POS	POS	POS	POS	NEG	POS	POS	POS	POS	POS	POS	POS	NEG	NEG
Kuwait_257	POS	POS	POS	POS	POS	POS	POS	POS	NEG	POS	POS	POS	POS	POS	POS	POS	NEG	NEG
Perth_01-15330	POS	POS	POS	POS	POS	POS	POS	POS	NEG	POS	POS	POS	POS	POS	POS	POS	NEG	NEG
Perth_01-15349	POS	POS	POS	POS	POS	POS	POS	POS	NEG	POS	POS	POS	POS	POS	POS	POS	NEG	NEG
Perth_01-16176	POS	POS	POS	POS	POS	POS	POS	POS	NEG	POS	POS	POS	POS	POS	POS	POS	NEG	NEG
Perth_WBG8379	POS	POS	POS	POS	POS	POS	POS	POS		POS	POS	POS	POS	POS	POS	POS	NEG	NEG
Rawalpindi_Kidney Center_56	POS	POS	POS	POS	POS	POS	POS	POS	NEG	POS	POS	POS	POS	POS	POS	POS	NEG	NEG
Riyadh_SSTI_05_3464614	POS	POS	POS	POS	POS	POS	POS	POS	NEG	POS	POS	POS	POS	POS	POS	POS	NEG	NEG
Trinidad&Tobago_2012_157	POS	POS	POS	POS	POS	POS	POS	POS	NEG	POS	POS	POS	POS	POS	POS	POS	NEG	NEG

STRAIN / ISOLATE	SPECIES MARKER							STAPHYLOXANTHIN BIOSYNTH. OPERON					REGULATORY GENES					
	rrnD1	gapA	katA	CoA	nuc1	spa	sbi	crtM		crtN	crtO	crtP	sarA	saeS	vraS	agrI	agrII	agrIII
	rrnD1 (S. aureus)	gapA	katA	CoA	nuc1	spa	sbi	crtM-nonST93	crtM-ST93	crtN	crtO	crtP	sarA	saeS	vraS			
	Domain 1 of 23S-rRNA	glyceraldehyde 3-phosphate dehydrogenase, locus 1	katalase A	coagulase	thermostable extracellular nuclease	staphylococcal protein A	IgG-binding protein	dehydrosqualene synthase		dehydrosqualene desaturase	staphyloxanthin acyltransferase	diaponeurosporene oxidase	staphylococcal accessory regulator A	histidine protein kinase, sae locus	sensor protein	accessory gene regulator allele I	accessory gene regulator allele II	accessory gene regulator allele III
>"South-East Asian Clad																		
M418	POS	POS	POS	POS	POS	POS	POS	POS	NEG	POS	POS	POS	POS	POS	POS	POS	NEG	NEG
>"South-East Asian Clad																		
NMR07 (India)	POS	POS	POS	POS	POS	POS	POS	POS	NEG	POS	POS	POS	POS	POS	POS	POS	NEG	NEG
NMR08 (India)	POS	POS	POS	POS	POS	POS	POS	POS	NEG	POS	POS	POS	POS	POS	POS	POS	NEG	NEG
Bengalaru_ST20121978	POS	POS	POS	POS	POS	POS	POS	POS	NEG	POS	POS	POS	POS	POS	POS	POS	NEG	NEG
Hong Kong_89	POS	POS	POS	POS	POS	POS	POS	POS	NEG	POS	POS	POS	POS	POS	POS	POS	NEG	NEG
>"South-East Asian Clad																		
Perth_2005_AH_5	POS	POS	POS	POS	POS	POS	POS	POS	NEG	POS	POS	POS	POS	POS	POS	POS	NEG	NEG
>"South-East Asian Clad																		
Trinidad&Tobago_MRSA2010_103	POS	POS	POS	POS	POS	POS	POS	POS	NEG	POS	POS	POS	POS	POS	POS	POS	NEG	NEG
Trinidad&Tobago_2013_PA25	POS	POS	POS	POS	POS	POS	POS	POS	NEG	POS	POS	POS	POS	POS	POS	POS	NEG	NEG
Trinidad&Tobago_MRSA2010_110	POS	POS	POS	POS	POS	POS	POS	POS	NEG	POS	POS	POS	POS	POS	POS	POS	NEG	NEG
Trinidad&Tobago_SSTI 2012_111	POS	POS	POS	POS	POS	POS	POS	POS	NEG	POS	POS	POS	POS	POS	POS	POS	NEG	NEG
Trinidad&Tobago_MRSA2010_158	POS	POS	POS	POS	POS	POS	POS	POS	NEG	POS	POS	POS	POS	POS	POS	POS	NEG	NEG
>"South-East Asian Clad																		
Kuwait_122	POS	POS	POS	POS	POS	POS	POS	POS	NEG	POS	POS	POS	POS	POS	POS	POS	NEG	NEG
Kuwait_002	POS	POS	POS	POS	POS	POS	POS	POS	NEG	POS	POS	POS	POS	POS	POS	POS	NEG	NEG
Kuwait_196	POS	POS	POS	POS	POS	POS	POS	POS	NEG	POS	POS	POS	POS	POS	POS	POS	NEG	NEG
Kuwait_199	POS	POS	POS	POS	POS	POS	POS	POS	NEG	POS	POS	POS	POS	POS	POS	POS	NEG	NEG
Hong Kong_136	POS	POS	POS	POS	POS	POS	POS	POS	NEG	POS	POS	POS	POS	POS	POS	POS	NEG	NEG
>"South-East Asian Clad																		
P32 (Poland)	POS	POS	POS	POS	POS	POS	POS	POS	NEG	POS	POS	POS	POS	POS	POS	POS	NEG	NEG
>"South-East Asian Clad																		
Perth_2015-S32313	POS	POS	POS	POS	POS	POS	POS	POS	NEG	POS	POS	POS	NEG	POS	POS	NEG	NEG	NEG
>"hla-negative South-Ea																		
DEN907 (Denmark)	POS	POS	POS	POS	POS	POS	POS	POS	NEG	POS	POS	POS	POS	POS	POS	POS	NEG	NEG
>"hla-negative South-Ea																		
S26 (Thailand)	POS	POS	POS	POS	POS	POS	POS	POS	NEG	POS	POS	POS	POS	POS	POS	POS	NEG	NEG
S42 (Thailand)	POS	POS	POS	POS	POS	POS	POS	POS	NEG	POS	POS	POS	POS	POS	POS	POS	NEG	NEG
S97 (Thailand)	POS	POS	POS	POS	POS	POS	POS	POS	NEG	POS	POS	POS	POS	POS	POS	POS	NEG	NEG
S78 (Thailand)	POS	POS	POS	POS	POS	POS	POS	POS	NEG	POS	POS	POS	POS	POS	POS	POS	NEG	NEG
S25 (Thailand)	POS	POS	POS	POS	POS	POS	POS	POS	NEG	POS	POS	POS	POS	POS	POS	POS	NEG	NEG
S21 (Thailand)	POS	POS	POS	POS	POS	POS	POS	POS	NEG	POS	POS	POS	POS	POS	POS	POS	NEG	NEG
S106 (Thailand)	POS	POS	POS	POS	POS	POS	POS	POS	NEG	POS	POS	POS	POS	POS	POS	POS	NEG	NEG
S24 (Thailand)	POS	POS	POS	POS	POS	POS	POS	POS	NEG	POS	POS	POS	POS	POS	POS	POS	NEG	NEG
S39 (Thailand)	POS	POS	POS	POS	POS	POS	POS	POS	NEG	POS	POS	POS	POS	POS	POS	POS	NEG	NEG
S2 (Thailand)	POS	POS	POS	POS	POS	POS	POS	POS	NEG	POS	POS	POS	POS	POS	POS	POS	NEG	NEG
S81 (Thailand)	POS	POS	POS	POS	POS	POS	POS	POS	NEG	POS	POS	POS	POS	POS	POS	POS	NEG	NEG
M116 (Vietnam)	POS	POS	POS	POS	POS	POS	POS	POS	NEG	POS	POS	POS	POS	POS	POS	POS	NEG	NEG
>"Portuguese Clade": Pc																		
FFP103 (Portugal)	POS	POS	POS	POS	POS	POS	POS	POS	NEG	POS	POS	POS	POS	POS	POS	POS	NEG	NEG
HDG2 (Portugal)	POS	POS	POS	POS	POS	POS	POS	POS	NEG	POS	POS	POS	POS	POS	POS	POS	NEG	NEG
HSA10 (Portugal)	POS	POS	POS	POS	POS	POS	POS	POS	NEG	POS	POS	POS	POS	POS	POS	POS	NEG	NEG
HSA11 (Portugal)	POS	POS	POS	POS	POS	POS	POS	POS	NEG	POS	POS	POS	POS	POS	POS	POS	NEG	NEG
ICP5011 (Portugal)	POS	POS	POS	POS	POS	POS	POS	POS	NEG	POS	POS	POS	POS	POS	POS	POS	NEG	NEG
ICP5014 (Portugal)	POS	POS	POS	POS	POS	POS	POS	POS	NEG	POS	POS	POS	POS	POS	POS	POS	NEG	NEG
ICP5062 (Portugal)	POS	POS	POS	POS	POS	POS	POS	POS	NEG	POS	POS	POS	POS	POS	POS	POS	NEG	NEG
Strain 1063_ATCC 33592 FDAARGOS_3 (USA)	POS	POS	POS	POS	POS	POS	POS	POS	NEG	POS	POS	POS	POS	POS	POS	POS	NEG	NEG
	POS	POS	POS	POS	POS	POS	POS	POS	NEG	POS	POS	POS	POS	POS	POS	POS	NEG	NEG
Russia-07_0110_Kurgan	POS	POS	POS	AMB	POS	POS	POS	AMB	NEG	POS	POS	POS	POS	POS	POS	POS	NEG	NEG
Russia-11_0164_Kurgan	POS	POS	POS	POS	POS	POS	POS	POS	NEG	POS	POS	POS	POS	POS	POS	POS	NEG	NEG
Russia-20_0367_Kurgan	POS	POS	POS	POS	POS	POS	POS	POS	NEG	POS	POS	POS	POS	POS	POS	POS	NEG	NEG
Russia-21_0390_Chelyabinsk	POS	POS	POS	POS	POS	POS	POS	POS	NEG	POS	POS	POS	POS	POS	POS	POS	NEG	NEG
>"South American/ Mid																		
AGT1 (Argentina)	POS	POS	POS	POS	POS	POS	POS	POS	NEG	POS	POS	POS	POS	POS	POS	POS	NEG	NEG
AGT120 (Argentina)	POS	POS	POS	POS	POS	POS	POS	POS	NEG	POS	POS	POS	POS	POS	POS	POS	NEG	NEG
AGT67 (Argentina)	POS	POS	POS	POS	POS	POS	POS	POS	NEG	POS	POS	POS	POS	POS	POS	POS	NEG	NEG
AGT9 (Argentina)	POS	POS	POS	POS	POS	POS	POS	POS	NEG	POS	POS	POS	POS	POS	POS	POS	NEG	NEG
RA3 (Argentina)	POS	POS	POS	POS	POS	POS	POS	POS	NEG	POS	POS	POS	POS	POS	POS	POS	NEG	NEG
RA6 (Argentina)	POS	POS	POS	POS	POS	POS	POS	POS	NEG	POS	POS	POS	POS	POS	POS	POS	NEG	NEG
RA7 (Argentina)	POS	POS	POS	POS	POS	POS	POS	POS	NEG	POS	POS	POS	POS	POS	POS	POS	NEG	NEG
Be62 (Brazil)	POS	POS	POS	POS	POS	POS	POS	POS	NEG	POS	POS	POS	POS	POS	POS	POS	NEG	NEG
Bmb9393 (Brazil)	POS	POS	POS	POS	POS	POS	POS	POS	NEG	POS	POS	POS	POS	POS	POS	POS	NEG	NEG
BRA36 (Brazil)	POS	POS	POS	POS	POS	POS	POS	POS	NEG	POS	POS	POS	POS	POS	POS	POS	NEG	NEG
BZ48 (Brazil)	POS	POS	POS	POS	POS	POS	POS	POS	NEG	POS	POS	POS	POS	POS	POS	POS	NEG	NEG
Gv51 (Brazil)	POS	POS	POS	POS	POS	POS	POS	POS	NEG	POS	POS	POS	POS	POS	POS	POS	NEG	NEG
Gv69 (Brazil)	POS	POS	POS	POS	POS	POS	POS	POS	NEG	POS	POS	POS	POS	POS	POS	POS	NEG	NEG
Gv88 (Brazil)	POS	POS	POS	POS	POS	POS	POS	POS	NEG	POS	POS	POS	POS	POS	POS	POS	NEG	NEG
HC1335 (Brazil)	POS	POS	POS	POS	POS	POS	POS	POS	NEG	POS	POS	POS	POS	POS	POS	POS	NEG	NEG
HC1340 (Brazil)	POS	POS	POS	POS	POS	POS	POS	POS	NEG	POS	POS	POS	POS	POS	POS	POS	NEG	NEG
HC556 (Brazil)	POS	POS	POS	POS	POS	POS	POS	POS	NEG	POS	POS	POS	POS	POS	POS	POS	NEG	NEG
HU25 (Brazil)	POS	POS	POS	POS	POS	POS	POS	POS	NEG	POS	POS	POS	POS	POS	POS	POS	NEG	NEG
UB563 (Brazil)	POS	POS	POS	POS	POS	POS	POS	POS	NEG	POS	POS	POS	POS	POS	POS	POS	NEG	NEG
2A8 (Czech Republic)	POS	POS	POS	POS	POS	POS	POS	POS	NEG	POS	POS	POS	POS	POS	POS	POS	NEG	NEG
M1229 (Denmark)	POS	POS	POS	POS	POS	POS	POS	POS	NEG	POS	POS	POS	POS	POS	POS	POS	NEG	NEG
FRICAR (France)	POS	POS	POS	POS	POS	POS	POS	POS	NEG	POS	POS	POS	POS	POS	POS	POS	NEG	NEG
D90 (Germany)	POS	POS	POS	POS	POS	POS	POS	POS	NEG	POS	POS	POS	POS	POS	POS	POS	NEG	NEG
LIT68 (Lithuania)	POS	POS	POS	POS	POS	POS	POS	POS	NEG	POS	POS	POS	POS	POS	POS	POS	NEG	NEG
LIT76 (Lithuania)	POS	POS	POS	POS	POS	POS	POS	POS	NEG	POS	POS	POS	POS	POS	POS	POS	NEG	NEG
HGSA142 (Portugal)	POS	POS	POS	POS	POS	POS	POS	POS	NEG	POS	POS	POS	POS	POS	POS	POS	NEG	NEG
HGSA9 (Portugal)	POS	POS	POS	POS	POS	POS	POS	POS	NEG	POS	POS	POS	POS	POS	POS	POS	NEG	NEG
HSJ216 (Portugal)	POS	POS	POS	POS	POS	POS	POS	POS	NEG	POS	POS	POS	POS	POS	POS	POS	NEG	NEG
M278 (Portugal)	POS	POS	POS	POS	POS	POS	POS	POS	NEG	POS	POS	POS	POS	POS	POS	POS	NEG	NEG
ES26 (Spain)	POS	POS	POS	POS	POS	POS	POS	POS	NEG	POS	POS	POS	POS	POS	POS	POS	NEG	NEG
UK102 (UK)	POS	POS	POS	POS	POS	POS	POS	POS	NEG	POS	POS	POS	POS	POS	POS	POS	NEG	NEG
URU34 (Uruguay)	POS	POS	POS	POS	POS	POS	POS	POS	NEG	POS	POS	POS	POS	POS	POS	POS	NEG	NEG
IS-125	POS	POS	POS	POS	POS	POS	POS	POS	NEG	POS	POS	POS	POS	POS	POS	POS	NEG	NEG
IS-157	POS	POS	POS	POS	POS	POS	POS	POS	NEG	POS	POS	POS	POS	POS	POS	POS	NEG	NEG
Ecuador_1	POS	POS	POS	POS	POS	POS	POS	POS	NEG	POS	POS	POS	POS	POS	POS	POS	NEG	NEG
Ecuador_2	POS	POS	POS	POS	POS	POS	POS	POS	NEG	POS	POS	POS	POS	POS	POS	POS	NEG	NEG
Dresden_15ANR574263	POS	POS	POS	POS	POS	POS	POS	POS	NEG	POS	POS	POS	POS	POS	POS	POS	NEG	NEG
UK-EMRSA-11	POS	POS	POS	POS	POS	POS	POS	POS	NEG	POS	POS	POS	POS	POS	POS	POS	NEG	NEG
>"South American/ Mid																		
BRA2 (Brazil)	POS	POS	POS	POS	POS	POS	POS	POS	NEG	POS	POS	POS	POS	POS	POS	POS	NEG	NEG
CHL1 (Chile)	POS	POS	POS	POS	POS	POS	POS	POS	NEG	POS	POS	POS	POS	POS	POS	POS	NEG	NEG
CHL151 (Chile)	POS	POS	POS	POS	POS	POS	POS	POS	NEG	POS	POS	POS	POS	POS	POS	POS	NEG	NEG
UCL378 (Chile)	POS	POS	POS	POS	POS	POS	POS	POS	NEG	POS	POS	POS	POS	POS	POS	POS	NEG	NEG
UP72 (Peru)	POS	POS	POS	POS	POS	POS	POS	POS	NEG	POS	POS	POS	POS	POS	POS	POS	NEG	NEG
UP81 (Peru)	POS	POS	POS	POS	POS	POS	POS	POS	NEG	POS	POS	POS	POS	POS	POS	POS	NEG	NEG
>"South American/ Mid																		
UP1073 (Peru)	POS	POS	POS	POS	POS	POS	POS	POS	NEG	POS	POS	POS	POS	POS	POS	POS	NEG	NEG

STRAIN / ISOLATE	SPECIES MARKER							STAPHYLOXANTHIN BIOSYNTH. OPERON					REGULATORY GENES					
	rrnD1	gapA	katA	CoA	nuc1	spa	sbi	crtM		crtN	crtO	crtP	sarA	saeS	vraS	agrI	agrII	agrIII
	rrnD1 (S. aureus)	gapA	katA	CoA	nuc1	spa	sbi	crtM-nonST93	crtM-ST93	crtN	crtO	crtP	sarA	saeS	vraS			
	Domain 1 of 23S-rRNA	glyceraldehyde 3-phosphate dehydrogenase, locus 1	katalase A	coagulase	thermostable extracellular nuclease	staphylococcal protein A	IgG-binding protein	dehydrosqualene synthase		dehydrosqualene desaturase	staphyloxanthin acyltransferase	diaponeurosporene oxidase	staphylococcal accessory regulator A	histidine protein kinase, sae locus	sensor protein	accessory gene regulator allele I	accessory gene regulator allele II	accessory gene regulator allele III
>"South American/ Mid																		
LIT89 (Lithuania)	POS	POS	POS	POS	POS	POS	POS	POS	NEG	POS	POS	POS	POS	POS	POS	POS	NEG	NEG
MRSA_PR1 (Malaysia)	NEG	POS	POS	POS	POS	POS	POS	POS	NEG	POS	POS	POS	POS	POS	POS	POS	NEG	NEG
Riyadh-2888905-R	POS	POS	POS	POS	POS	POS	POS	POS	NEG	POS	POS	POS	POS	POS	POS	POS	NEG	NEG
Riyadh-2888915-BC	POS	POS	POS	POS	POS	POS	POS	POS	NEG	POS	POS	POS	POS	POS	POS	POS	NEG	NEG
Riyadh-2793706-R	POS	POS	POS	POS	POS	POS	POS	POS	NEG	POS	POS	POS	POS	POS	POS	POS	NEG	NEG
>"South American/ Mid																		
Riyadh_SSTI_18_3502925	POS	POS	POS	POS	POS	POS	POS	POS	NEG	POS	POS	POS	POS	POS	POS	POS	NEG	NEG
Riyadh-2822825-W	POS	POS	POS	POS	POS	POS	POS	POS		POS	POS	POS	POS	POS	POS	POS	NEG	NEG
>"South American/ Mid																		
UK-EMRSA-9	POS	POS	POS	POS	POS	POS	POS	POS	NEG	POS	POS	POS	POS	POS	POS	POS	NEG	NEG
>"South American/ Mid																		
Lome_HT20020815	POS	POS	POS	POS	POS	POS	POS	POS	NEG	POS	POS	POS	POS	POS	POS	POS	NEG	NEG
>"South American/ Mid																		
NA32 (Denmark)	NEG	POS	POS	POS	POS	POS	POS	POS	NEG	POS	POS	POS	POS	POS	POS	POS	NEG	NEG
H24 (Egypt)	POS	POS	POS	POS	POS	POS	POS	POS	NEG	POS	POS	POS	POS	POS	POS	POS	NEG	NEG
ATCC BAA-39 (=HUSA304) (Hungary)	POS	POS	POS	POS	POS	POS	POS	POS	NEG	POS	POS	POS	POS	POS	POS	POS	NEG	NEG
HUSA304 (Hungary)	POS	POS	POS	POS	POS	POS	POS	POS	NEG	POS	POS	POS	POS	POS	POS	POS	NEG	NEG
HU106 (Hungary)	POS	POS	POS	POS	POS	POS	POS	POS	NEG	POS	POS	POS	POS	POS	POS	POS	NEG	NEG
BSAC27 (UK)	POS	POS	POS	POS	POS	POS	POS	POS	NEG	POS	POS	POS	POS	POS	POS	POS	NEG	NEG
NCTC13131, UK-EMRSA-4 (UK)	POS	POS	POS	POS	POS	POS	POS	POS	NEG	POS	POS	POS	POS	POS	POS	POS	NEG	NEG
	POS	POS	POS	POS	POS	POS	POS	POS	NEG	POS	POS	POS	POS	POS	POS	POS	NEG	NEG
Algiers_HT20040080	POS	POS	POS	POS	POS	POS	POS	POS	NEG	POS	POS	POS	POS	POS	POS	POS	NEG	NEG
Dublin-DSH_AR09_0.0066	POS	POS	POS	POS	POS	POS	POS	POS	NEG	POS	POS	POS	POS	POS	POS	POS	NEG	NEG
Dublin-DSH_AR09_0-0065	POS	POS	POS	POS	POS	POS	POS	POS	NEG	POS	POS	POS	POS	POS	POS	POS	NEG	NEG
Dublin-DSH_Phenotype-III_84	POS	POS	POS	POS	POS	POS	POS	POS	NEG	POS	POS	POS	POS	POS	POS	POS	NEG	NEG
Hong Kong_130	POS	POS	POS	POS	POS	POS	POS	POS	NEG	POS	POS	POS	POS	POS	POS	POS	NEG	NEG
Kuwait_018	POS	POS	POS	POS	POS	POS	POS	POS	NEG	POS	POS	POS	POS	POS	POS	POS	NEG	NEG
Perth_08-17726	POS	POS	POS	POS	POS	POS	POS	POS	NEG	POS	POS	POS	POS	POS	POS	POS	NEG	NEG
Riyadh_Alfaisal/KKKSUH_86_MRS-14-279	POS	POS	POS	POS	POS	POS	POS	POS	NEG	POS	POS	POS	POS	POS	POS	POS	NEG	NEG
Riyadh_Alfaisal-04_Z3861_831588	POS	POS	POS	POS	POS	POS	POS	POS	NEG	POS	POS	POS	POS	POS	POS	POS	NEG	NEG
Riyadh_Alfaisal-30_S15724_1108013	POS	POS	POS	POS	POS	POS	POS	POS	NEG	POS	POS	POS	POS	POS	POS	POS	NEG	NEG
Riyadh_Alfaisal-6_22A_13_B3992_397721	POS	POS	POS	POS	POS	POS	POS	POS	NEG	POS	POS	POS	POS	POS	POS	POS	NEG	NEG
Riyadh_SSTI_52_3615482	POS	POS	POS	POS	POS	POS	POS	POS	NEG	POS	POS	POS	POS	POS	POS	POS	NEG	NEG
Riyadh-2817276-2	POS	POS	POS	POS	POS	POS	POS	POS	NEG	POS	POS	POS	POS	POS	POS	POS	NEG	NEG
Riyadh-2891670-W	POS	POS	POS	POS	POS	POS	POS	POS		POS	POS	POS	POS	POS	POS	POS	NEG	NEG
Riyadh-3006920-W	POS	POS	POS	POS	POS	POS	POS	POS	NEG	POS	POS	POS	POS	POS	POS	POS	NEG	NEG
Riyadh-R2567782	POS	POS	POS	POS	POS	POS	POS	POS	NEG	POS	POS	POS	POS	POS	POS	POS	NEG	NEG
Russia-18_0252_Krasnoyarsk_SK2	POS	POS	POS	POS	POS	POS	POS	POS	NEG	POS	POS	POS	POS	POS	POS	POS	NEG	NEG
UK-EMRSA-7	POS	POS	POS	POS	POS	POS	POS	POS	NEG	POS	POS	POS	POS	POS	POS	POS	NEG	NEG
>"South American/ Mid																		
Dublin-DSH_AR23_0073	POS	POS	POS	POS	POS	POS	POS	POS	NEG	POS	POS	POS	POS	POS	POS	POS	NEG	NEG
>Related to "South Ame																		
MRSA-OC3 (Russia)	POS	POS	POS	POS	POS	POS	POS	POS	NEG	POS	POS	POS	POS	POS	POS	POS	NEG	NEG
Russia-12_0176_Krasnoyarsk	POS	POS	POS	POS	POS	POS	POS	POS	NEG	POS	POS	POS	POS	POS	POS	POS	NEG	NEG
Russia-13_0180_Krasnoyarsk	POS	POS	POS	POS	POS	POS	POS	POS	NEG	POS	POS	POS	POS	POS	POS	POS	NEG	NEG
Russia-16_0249_Krasnoyarsk	POS	POS	POS	POS	POS	POS	POS	POS	NEG	POS	POS	POS	POS	POS	POS	POS	NEG	NEG
Russia-17_0250_Krasnoyarsk	POS	POS	POS	POS	POS	POS	POS	POS	NEG	POS	POS	POS	POS	POS	POS	POS	NEG	NEG
>Related to "South Ame																		
URU110 (Uruguay)	POS	POS	POS	POS	POS	POS	POS	POS	NEG	POS	POS	POS	POS	POS	POS	POS	NEG	NEG
>Related to "South Ame																		
DS_014 (Thailand)	POS	POS	POS	POS	POS	POS	POS	POS	NEG	POS	POS	POS	POS	POS	POS	POS	NEG	NEG
>Unassigned Middle Eas																		
Frankfurt_Oder_0490031797 (Libya)	POS	POS	POS	POS	POS	POS	POS	POS	NEG	POS	POS	POS	POS	POS	POS	POS	NEG	NEG
Kuwait_192	POS	POS	POS	POS	POS	POS	POS	POS	NEG	POS	POS	POS	POS	POS	POS	POS	NEG	NEG
Riyadh-9	POS	POS	POS	POS	POS	POS	POS	POS	NEG	POS	POS	POS	POS	POS	POS	POS	NEG	NEG
Russia-24_0407_Moscow	POS	POS	POS	POS	POS	POS	POS	POS	NEG	POS	POS	POS	POS	POS	POS	POS	NEG	NEG

STRAIN / ISOLATE	REGULATORY GENES				
	agrIV	agrV		hld	setC
		agrV-ST1850	agrV-ST2198		setC / selX
	accessory gene regulator allele IV	accessory gene regulator alleles from S. argenteus group		haemolysin delta	Staphyl. exotoxin-like protein
>"Eurasian Clade": TUR1					
TUR1 (Turkey)	NEG	NEG	NEG	POS	POS
TUR9 (Turkey)	NEG	NEG	NEG	POS	POS
>"Eurasian Clade" : T013					
T0131 (China)	NEG	NEG	NEG	POS	POS
MU4 (Turkey)	NEG	NEG	NEG	POS	POS
DEU11 (Turkey)	NEG	NEG	NEG	POS	POS
DEU16 (Turkey)	NEG	NEG	NEG	POS	POS
Romania_Iasi_MRSA-07_54	AMB	NEG	NEG	POS	POS
>"Eurasian Clade": arsC-					
Romania_Iasi_MRSA-05_46	AMB	NEG	NEG	POS	POS
Romania_Iasi_MRSA-06_47	AMB	NEG	NEG	POS	POS
>"Eurasian Clade": 16K/					
CN79 (China)	NEG	NEG	NEG	POS	POS
CUHK_BJ2002 (China)	NEG	NEG	NEG	POS	POS
CUHK_BJ2007 (China)	NEG	NEG	NEG	POS	POS
3HK (Czech Republic)	NEG	NEG	NEG	POS	POS
HU109 (Hungary)	NEG	NEG	NEG	POS	POS
HUR18 (Hungary)	NEG	NEG	NEG	POS	POS
H482 (Romania)	NEG	NEG	NEG	POS	POS
16K (Russia)	NEG	NEG	NEG	POS	POS
DEU10 (Turkey)	NEG	NEG	NEG	POS	POS
DEU12 (Turkey)	NEG	NEG	NEG	POS	POS
DEU14 (Turkey)	NEG	NEG	NEG	POS	POS
DEU15 (Turkey)	NEG	NEG	NEG	POS	POS
DEU17 (Turkey)	NEG	NEG	NEG	POS	POS
DEU19 (Turkey)	NEG	NEG	NEG	POS	POS
DEU2 (Turkey)	NEG	NEG	NEG	POS	POS
DEU20 (Turkey)	NEG	NEG	NEG	POS	POS
DEU23 (Turkey)	NEG	NEG	NEG	POS	POS
DEU3 (Turkey)	NEG	NEG	NEG	POS	POS
DEU5 (Turkey)	NEG	NEG	NEG	POS	POS
DEU6 (Turkey)	NEG	NEG	NEG	POS	POS
DEU8 (Turkey)	NEG	NEG	NEG	POS	POS
HU11 (Turkey)	NEG	NEG	NEG	POS	POS
HU13 (Turkey)	NEG	NEG	NEG	POS	POS
HU14 (Turkey)	NEG	NEG	NEG	POS	POS
HU15 (Turkey)	NEG	NEG	NEG	POS	POS
HU16 (Turkey)	NEG	NEG	NEG	POS	POS
HU17 (Turkey)	NEG	NEG	NEG	POS	POS
HU21 (Turkey)	NEG	NEG	NEG	POS	POS
HU23 (Turkey)	NEG	NEG	NEG	POS	POS
HU26 (Turkey)	NEG	NEG	NEG	POS	POS
HU4 (Turkey)	NEG	NEG	NEG	POS	POS
HU5 (Turkey)	NEG	NEG	NEG	POS	POS
HU6 (Turkey)	NEG	NEG	NEG	POS	POS
HU7 (Turkey)	NEG	NEG	NEG	POS	POS
HU8 (Turkey)	NEG	NEG	NEG	POS	POS
HU9 (Turkey)	NEG	NEG	NEG	POS	POS
IU1 (Turkey)	NEG	NEG	NEG	POS	POS
IU10 (Turkey)	NEG	NEG	NEG	POS	POS
IU11 (Turkey)	NEG	NEG	NEG	POS	POS
IU12 (Turkey)	NEG	NEG	NEG	POS	POS
IU13 (Turkey)	NEG	NEG	NEG	POS	POS
IU15 (Turkey)	NEG	NEG	NEG	POS	POS
IU18 (Turkey)	NEG	NEG	NEG	POS	POS
IU19 (Turkey)	NEG	NEG	NEG	POS	POS
IU2 (Turkey)	NEG	NEG	NEG	POS	POS
IU4 (Turkey)	NEG	NEG	NEG	POS	POS
IU6 (Turkey)	NEG	NEG	NEG	POS	POS
IU7 (Turkey)	NEG	NEG	NEG	POS	POS
IU9 (Turkey)	NEG	NEG	NEG	POS	POS
MU1 (Turkey)	NEG	NEG	NEG	POS	POS
MU10 (Turkey)	NEG	NEG	NEG	POS	POS
MU20 (Turkey)	NEG	NEG	NEG	POS	POS
Mu3 (Turkey)	NEG	NEG	NEG	POS	POS
MU5 (Turkey)	NEG	NEG	NEG	POS	POS
MU6 (Turkey)	NEG	NEG	NEG	POS	POS
MU7 (Turkey)	NEG	NEG	NEG	POS	POS
TUR27 (Turkey)	NEG	NEG	NEG	POS	POS
NCTR 32S	NEG	NEG	NEG	POS	POS
SA02_A4 = Russia-06_0085_Moscow	NEG	NEG	NEG	POS	POS
	AMB	NEG	NEG	POS	POS
Dresden_08V35987 (Turkey)	NEG	NEG	NEG	POS	POS
Dresden_17ANR577152	NEG	NEG	NEG	POS	POS
Dresden_17ANR580374 (Makedonia)	NEG	NEG	NEG	POS	POS
Hong Kong_69-II	AMB	NEG	NEG	POS	POS
Hong Kong_93	AMB	NEG	NEG	POS	POS
Rawalpindi_Kidney Center_03_SK1	AMB	NEG	NEG	POS	POS
Rawalpindi_Kidney Center_08	NEG	NEG	NEG	POS	POS
Rawalpindi_Kidney Center_10	AMB	NEG	NEG	POS	POS
Rawalpindi_Kidney Center_19	AMB	NEG	NEG	POS	POS
Rawalpindi_Kidney Center_50_SK1	NEG	NEG	NEG	POS	POS
Romania_Iasi_BC-49_430	AMB	NEG	NEG	POS	POS
Romania_Iasi_MRSA_284	AMB	NEG	NEG	POS	POS
Romania_Iasi_MRSA-32_318	NEG	NEG	NEG	POS	POS
Romania_Iasi_MRSA-54_430	POS	NEG	NEG	POS	POS
Romania_Iasi_SSTI-01_2	AMB	NEG	NEG	POS	POS
Romania_Iasi_SSTI-10_101	AMB	NEG	NEG	POS	POS
Romania_Iasi_SSTI-11_106	NEG	NEG	NEG	POS	POS
Russia-01_0001_SaintPetersburg	NEG	NEG	NEG	POS	POS
Russia-02_0004_SaintPetersburg	AMB	NEG	NEG	POS	POS
Russia-03_0057_SaintPetersburg	NEG	NEG	NEG	POS	POS
Russia-04_0076_Moscow	NEG	NEG	NEG	POS	POS
Russia-05_0078_Moscow	AMB	NEG	NEG	POS	POS
Russia-08_0135_Moscow	AMB	NEG	NEG	POS	POS
Russia-09_0150_Kurgan	NEG	NEG	NEG	POS	POS
Russia-10_0162_Kurgan	NEG	NEG	NEG	POS	POS
Russia-14_0184_Moscow	NEG	NEG	NEG	POS	POS
Russia-15_0232_SaintPetersburg	NEG	NEG	NEG	POS	POS
Russia-22_0391_Chelyabinsk	NEG	NEG	NEG	POS	POS
Russia-23_0392_Chelyabinsk	NEG	NEG	NEG	POS	POS
>"Eurasian Clade" : IU17					
IU17 (Turkey)	NEG	NEG	NEG	POS	POS
IU20 (Turkey)	NEG	NEG	NEG	POS	POS

STRAIN / ISOLATE	REGULATORY GENES				
	agrIV	agrV		hld	setC
		agrV-ST1850	agrV-ST2198		setC / selX
	accessory gene regulator allele IV	accessory gene regulator alleles from S. argenteus group		haemolysin delta	Staphyl. exotoxin-like protein
>"Eurasian Clade": Spora					
Dresden_16ANR581769	NEG	NEG	NEG	POS	POS
Dresden_17ANR578769	NEG	NEG	NEG	POS	POS
>"Eurasian Clade": Kuwa					
Kuwait_306	POS	NEG	NEG	POS	POS
Kuwait_301	AMB	NEG	NEG	POS	POS
>"European Clade": "Gre					
GRE18 (Greece)	NEG	NEG	NEG	POS	POS
GRE317 (Greece)	NEG	NEG	NEG	POS	POS
GRE4 (Greece)	NEG	NEG	NEG	POS	POS
R35 (USA)	NEG	NEG	NEG	POS	POS
Casablanca HT20060548	AMB	NEG	NEG	POS	POS
Casablanca HT20060550	AMB	NEG	NEG	POS	POS
Dresden_01V34572 (Greece)	NEG	NEG	NEG	POS	POS
Dresden_01V36469	NEG	NEG	NEG	POS	POS
Dresden 01V39123	POS	NEG	NEG	POS	POS
Dresden 01V39143	NEG	NEG	NEG	POS	POS
Greece 1 3680 Harmony	NEG	NEG	NEG	POS	POS
>"European Clade": UK-(					
ANS46 (Australia)	NEG	NEG	NEG	POS	POS
LHH1 (USA)	NEG	NEG	NEG	POS	POS
Dublin-DSH_AR01_0-1085	NEG			POS	POS
Dublin-DSH_AR01_0-1118	NEG	NEG	NEG	POS	POS
Dublin-DSH_AR15_0098	NEG	NEG	NEG	POS	POS
Dublin-DSH_AR15_0104	NEG	NEG	NEG	POS	POS
UK_NCTC11939_UK-1_Harmony	NEG	NEG	NEG	POS	POS
>"European Clade": BK2					
BK2421 (USA)	NEG	NEG	NEG	POS	POS
>"Aussi/NZ Clade", JKD6					
JKD6009 (Australia)	NEG	NEG	NEG	POS	POS
JKD6008 (New Zealand)	NEG	NEG	NEG	POS	POS
Perth_2015-S20209	NEG	NEG	NEG	POS	POS
Perth_2015-S20305	NEG	NEG	NEG	POS	POS
Perth_2015-S20603	NEG	NEG	NEG	POS	POS
Perth_2015-S20607	NEG	NEG	NEG	POS	POS
Perth_2015-S20608	NEG	NEG	NEG	POS	POS
Perth_2015-S20610	NEG	NEG	NEG	POS	POS
Perth_2015-S20613	NEG	NEG	NEG	POS	POS
Perth_2015-S20617	NEG	NEG	NEG	POS	POS
Perth_2015-S20622	NEG	NEG	NEG	POS	NEG
Perth_2015-S23619	NEG	NEG	NEG	POS	POS
Perth_2015-S41210	NEG	NEG	NEG	POS	POS
Perth_2015-S51001	NEG	NEG	NEG	POS	POS
Perth_2015-S51003	NEG	NEG	NEG	POS	POS
Perth_2015-S51006	NEG	NEG	NEG	POS	POS
Perth_2015-S51007	NEG	NEG	NEG	POS	POS
Perth_2015-S51008	NEG	NEG	NEG	POS	POS
Perth_2015-S51014	NEG	NEG	NEG	POS	POS
Perth_2015-S51408	NEG	NEG	NEG	POS	POS
Perth_2016-S20614	NEG	NEG	NEG	POS	POS
Perth_2016-S23614	NEG	NEG	NEG	POS	POS
Perth_2016-S42009	NEG	NEG	NEG	POS	POS
Perth_2016-S51001	NEG	NEG	NEG	POS	POS
Perth_2016-S51403	NEG	NEG	NEG	POS	POS
Perth_07 RPA 20	NEG	NEG	NEG	POS	POS
Perth_08-19088	AMB	NEG	NEG	POS	POS
Perth_01-15351	NEG			POS	POS
Perth_01-15357	NEG	NEG	NEG	POS	POS
Perth_01-15419	NEG	NEG	NEG	POS	POS
Perth_01-16309	NEG	NEG	NEG	POS	POS
Perth_03 SNP 95	AMB	NEG	NEG	POS	POS
Perth_2003_AH_47	AMB	NEG	NEG	POS	POS
>"Australian/NZ Clade":					
Perth_2015-S20623	NEG	NEG	NEG	POS	POS
Perth_2015-S20826	NEG	NEG	NEG	POS	POS
Perth_05 NH 56	NEG	NEG	NEG	POS	POS
>CC5-MRSA-III LA MRSA					
Delbrück 38 isolate from domestic turkey	NEG	NEG	NEG	POS	POS
> Staph. pseudintermed					
KM1381	NEG	NEG	NEG	NEG	NEG
>"South-East Asian Clad					
M92 (Canada)	NEG	NEG	NEG	POS	POS
CHI59 (China)	NEG	NEG	NEG	POS	POS
CHI61 (China)	NEG	NEG	NEG	POS	POS
CUHK_HK1997 (China)	NEG	NEG	NEG	POS	POS
M996 (China)	NEG	NEG	NEG	POS	POS
H211 (Denmark)	NEG	NEG	NEG	POS	POS
H216 (Denmark)	NEG	NEG	NEG	POS	POS
D71 (Germany)	NEG	NEG	NEG	POS	POS
NMR05 (India)	NEG	NEG	NEG	POS	POS
MAL11 (Malaysia)	NEG	NEG	NEG	POS	POS
MAL9 (Malaysia)	NEG	NEG	NEG	POS	POS
Na21 (Sri Lanka)	NEG	NEG	NEG	POS	POS
M592 (Syria)	NEG	NEG	NEG	POS	POS
H202 (Thailand)	NEG	NEG	NEG	POS	POS
S102 (Thailand)	NEG	NEG	NEG	POS	POS
S130 (Thailand)	NEG	NEG	NEG	POS	POS
S40 (Thailand)	NEG	NEG	NEG	POS	POS
S71 (Thailand)	NEG	NEG	NEG	POS	POS
S87 (Thailand)	NEG	NEG	NEG	POS	POS
S93 (Thailand)	NEG	NEG	NEG	POS	POS
US_002 (Thailand)	NEG	NEG	NEG	POS	POS
US_017 (Thailand)	NEG	NEG	NEG	POS	POS
WAMC6102 (Thailand)	NEG	NEG	NEG	POS	POS
NCTC_13626_TW20 (UK)	NEG	NEG	NEG	POS	POS
BSAC3021 (UK)	NEG	NEG	NEG	POS	POS
BSAC3046 (UK)	NEG	NEG	NEG	POS	POS
1193_SAUR (USA)	NEG	NEG	NEG	POS	POS
1194_SAUR (USA)	NEG	NEG	NEG	POS	POS

STRAIN / ISOLATE	REGULATORY GENES				
	agrIV	agrV		hld	setC
		agrV-ST1850	agrV-ST2198		setC / selX
	accessory gene regulator allele IV	accessory gene regulator alleles from S. argenteus group		haemolysin delta	Staphyl. exotoxin-like protein
>"South-East Asian Clade": TW20 (Irish AR44), c					
1195_SAUR (USA)	NEG	NEG	NEG	POS	POS
FVRH6002 (USA)	NEG	NEG	NEG	POS	POS
KINW6048 (USA)	NEG	NEG	NEG	POS	POS
LAMC0011 (USA)	NEG	NEG	NEG	POS	POS
SJOS6053 (USA)	NEG	NEG	NEG	POS	POS
SJOS6072 (USA)	NEG	NEG	NEG	POS	POS
UCIM6042 (USA)	NEG	NEG	NEG	POS	POS
Perth_2015-S31902	NEG	NEG	NEG	POS	NEG
Perth_2015-S31904	NEG	NEG	NEG	POS	POS
Perth_2015-S31913	NEG	NEG	NEG	POS	POS
Perth_2015-S31918	NEG	NEG	NEG	POS	NEG
Dublin-DSH_AR44_E1183	NEG	NEG	NEG	POS	POS
Dublin-DSH_Unfamiliar-3_E1520	NEG	NEG	NEG	POS	POS
Hong Kong_042	NEG	NEG	NEG	POS	POS
Hong Kong_101	NEG	NEG	NEG	POS	POS
Hong Kong_111	AMB	NEG	NEG	POS	POS
Hong Kong_113	NEG	NEG	NEG	POS	POS
Hong Kong_115	NEG	NEG	NEG	POS	POS
Hong Kong_118	NEG	NEG	NEG	POS	POS
Hong Kong_125	NEG	NEG	NEG	POS	POS
Hong Kong_129	NEG	NEG	NEG	POS	POS
Hong Kong_133	NEG	NEG	NEG	POS	POS
Kuwait_009	AMB	NEG	NEG	POS	POS
Kuwait_103	AMB			POS	POS
Kuwait_107	AMB	NEG	NEG	POS	POS
Perth_03 AH 88	NEG	NEG	NEG	POS	POS
Perth_08-16905	NEG	NEG	NEG	POS	POS
Riyadh_Alfaisal-10_SICU_13_B5672_1117353	AMB	NEG	NEG	POS	POS
Riyadh_SSTI_17_3508602	AMB	NEG	NEG	POS	POS
Riyadh-2822088-R	AMB	NEG	NEG	POS	POS
Trinidad&Tobago_2012_195 (619/12)	AMB	NEG	NEG	POS	POS
Trinidad&Tobago_2012_370 (1112-52177)	AMB	NEG	NEG	POS	POS
Trinidad&Tobago_2012_378 (4139)	AMB	NEG	NEG	POS	POS
Trinidad&Tobago_2013_PA21	AMB	NEG	NEG	POS	POS
Trinidad&Tobago_2013_PA22	POS	NEG	NEG	POS	POS
Trinidad&Tobago_2912_340 (8523/12)	AMB	NEG	NEG	POS	POS
Trinidad&Tobago_MRSA2010_105	AMB	NEG	NEG	POS	POS
Trinidad&Tobago_MRSA2010_108	AMB	NEG	NEG	POS	POS
Trinidad&Tobago_MRSA2010_33	AMB	NEG	NEG	POS	POS
Trinidad&Tobago_MRSA2010_79	NEG	NEG	NEG	POS	POS
Trinidad&Tobago_MRSA2010_88	AMB	NEG	NEG	POS	POS
Trinidad&Tobago_MRSA2010_97	AMB	NEG	NEG	POS	POS
Trinidad&Tobago_SSTI 2012_031	AMB	NEG	NEG	POS	POS
Uganda-29_17148_250811	NEG	NEG	NEG	POS	POS
>"South-East Asian Clad					
UCIM6015	NEG	NEG	NEG	POS	POS
Hong Kong_121	NEG	NEG	NEG	POS	POS
>"South-East Asian Clad					
Z172 (Taiwan)	NEG	NEG	NEG	POS	POS
Russia-19_0342_Kurgan	NEG	NEG	NEG	POS	POS
>"South-East Asian Clad					
Perth_01-15392	NEG	NEG	NEG	POS	POS
>"South-East Asian Clad					
XN108 (China)	NEG	NEG	NEG	POS	POS
NMR09 (India)	NEG	NEG	NEG	POS	POS
346 (Malaysia)	NEG	NEG	NEG	POS	POS
DS_009 (Thailand)	NEG	NEG	NEG	POS	POS
18ANR581005 (Ind)	NEG	NEG	NEG	POS	POS
Bengalaru_ST20141405	AMB	NEG	NEG	POS	POS
Bengalaru_ST20141406	AMB	NEG	NEG	POS	POS
Bengalaru_ST20141417	AMB	NEG	NEG	POS	POS
Hong Kong_107	NEG	NEG	NEG	POS	POS
Hong Kong_12	NEG	NEG	NEG	POS	POS
Hong Kong_132	NEG	NEG	NEG	POS	POS
Riyadh-3028763-R	NEG	NEG	NEG	POS	POS
Trinidad&Tobago_MRSA2010_94	AMB	NEG	NEG	POS	POS
>"South-East Asian Clad					
M705 (Thailand)	NEG	NEG	NEG	POS	POS
S85 (Thailand)	NEG	NEG	NEG	POS	POS
Riyadh_Alfaisal-04_281014_S5076	AMB	NEG	NEG	POS	POS
Riyadh_Alfaisal-26_S15145_1104163	AMB	NEG	NEG	POS	POS
Riyadh-2817437-W	AMB	NEG	NEG	POS	POS
>"South-East Asian Clad					
PPUKM-775-2009 (Malaysia)	NEG	NEG	NEG	POS	POS
Hong Kong_138	NEG	NEG	NEG	POS	POS
>"South-East Asian Clad					
Kuwait_001	AMB	NEG	NEG	POS	POS
Kuwait_337	AMB	NEG	NEG	POS	POS
>"South-East Asian Clad					
CUHK_HK2007 (China)	NEG	NEG	NEG	POS	POS
NMR02 (India)	NEG	NEG	NEG	POS	POS
VB1490 (India)	NEG	NEG	NEG	POS	POS
V521 (Korea)	NEG	NEG	NEG	POS	POS
HST-077 (Lebanon)	NEG	NEG	NEG	POS	POS
MAL1 (Malaysia)	NEG	NEG	NEG	POS	POS
MAL3 (Malaysia)	NEG	NEG	NEG	POS	POS
PPUKM-261-2009 (Malaysia)	NEG	NEG	NEG	POS	POS
PPUKM-332-2009 (Malaysia)	NEG	NEG	NEG	POS	POS
S38 (Thailand)	NEG	NEG	NEG	POS	POS
S7 (Thailand)	NEG	NEG	NEG	POS	POS
US_008 (Thailand)	NEG	NEG	NEG	POS	POS
US_030 (Thailand)	NEG	NEG	NEG	POS	POS
M170 (Tsunami-related)	NEG	NEG	NEG	POS	POS
BSAC697 (UK)	NEG	NEG	NEG	POS	POS
IS-189	NEG	NEG	NEG	POS	POS
MRGR3	NEG	NEG	NEG	POS	POS
smg07	NEG	NEG	NEG	POS	POS
AH1-AUS-EMRSA-3	NEG	NEG	NEG	POS	POS
Bengalaru_ST20141403	AMB	NEG	NEG	POS	POS
Finland E24_98541_Harmony	AMB	NEG	NEG	POS	POS
Hong Kong_10	AMB	NEG	NEG	POS	POS
Hong Kong_100	NEG	NEG	NEG	POS	POS
Hong Kong_199	AMB	NEG	NEG	POS	POS
Hong Kong_34	AMB	NEG	NEG	POS	POS
Hong Kong_88	AMB	NEG	NEG	POS	POS
Hong Kong_92	AMB	NEG	NEG	POS	POS
Hong Kong_94	NEG	NEG	NEG	POS	POS
Kuwait_257	AMB	NEG	NEG	POS	POS
Perth_01-15330	NEG	NEG	NEG	POS	POS
Perth_01-15349	NEG	NEG	NEG	POS	POS
Perth_01-16176	NEG	NEG	NEG	POS	POS
Perth_WBG8379	NEG			POS	POS
Rawalpindi_Kidney Center_56	POS	NEG	NEG	POS	POS
Riyadh_SSTI_05_3464614	NEG	NEG	NEG	POS	POS
Trinidad&Tobago_2012_157	AMB	NEG	NEG	POS	POS

STRAIN / ISOLATE	REGULATORY GENES				
	agrIV	agrV		hld	setC
		agrV-ST1850	agrV-ST2198		setC / selX
	accessory gene regulator allele IV	accessory gene regulator alleles from S. argenteus group		haemolysin delta	Staphyl. exotoxin-like protein
>"South-East Asian Clad					
M418	NEG	NEG	NEG	POS	POS
>"South-East Asian Clad					
NMR07 (India)	NEG	NEG	NEG	POS	POS
NMR08 (India)	NEG	NEG	NEG	POS	POS
Bengalaru_ST20121978	AMB	NEG	NEG	POS	POS
Hong Kong_89	AMB	NEG	NEG	POS	POS
>"South-East Asian Clad					
Perth_2005_AH_5	AMB	NEG	NEG	POS	POS
>"South-East Asian Clad					
Trinidad&Tobago_MRSA2010_103	NEG	NEG	NEG	POS	POS
Trinidad&Tobago_2013_PA25	POS	NEG	NEG	POS	POS
Trinidad&Tobago_MRSA2010_110	AMB	NEG	NEG	POS	POS
Trinidad&Tobago_SSTI 2012_111	AMB	NEG	NEG	POS	POS
Trinidad&Tobago_MRSA2010_158	NEG	NEG	NEG	POS	POS
>"South-East Asian Clad					
Kuwait_122	NEG	NEG	NEG	POS	POS
Kuwait_002	NEG	NEG	NEG	POS	POS
Kuwait_196	NEG	NEG	NEG	POS	POS
Kuwait_199	AMB	NEG	NEG	POS	POS
Hong Kong_136	AMB	NEG	NEG	POS	POS
>"South-East Asian Clad					
P32 (Poland)	NEG	NEG	NEG	POS	POS
>"South-East Asian Clad					
Perth_2015-S32313	NEG	NEG	NEG	POS	POS
>"hla-negative South-Ea					
DEN907 (Denmark)	NEG	NEG	NEG	POS	POS
>"hla-negative South-Ea					
S26 (Thailand)	NEG	NEG	NEG	POS	POS
S42 (Thailand)	NEG	NEG	NEG	POS	POS
S97 (Thailand)	NEG	NEG	NEG	POS	POS
S78 (Thailand)	NEG	NEG	NEG	POS	POS
S25 (Thailand)	NEG	NEG	NEG	POS	POS
S21 (Thailand)	NEG	NEG	NEG	POS	POS
S106 (Thailand)	NEG	NEG	NEG	POS	POS
S24 (Thailand)	NEG	NEG	NEG	POS	POS
S39 (Thailand)	NEG	NEG	NEG	POS	POS
S2 (Thailand)	NEG	NEG	NEG	POS	POS
S81 (Thailand)	NEG	NEG	NEG	POS	POS
M116 (Vietnam)	NEG	NEG	NEG	POS	POS
>"Portuguese Clade": Pc					
FFP103 (Portugal)	NEG	NEG	NEG	POS	POS
HDG2 (Portugal)	NEG	NEG	NEG	POS	POS
HSA10 (Portugal)	NEG	NEG	NEG	POS	POS
HSA11 (Portugal)	NEG	NEG	NEG	POS	POS
ICP5011 (Portugal)	NEG	NEG	NEG	POS	POS
ICP5014 (Portugal)	NEG	NEG	NEG	POS	POS
ICP5062 (Portugal)	NEG	NEG	NEG	POS	POS
Strain 1063_ATCC 33592 FDAARGOS_3 (USA)	NEG	NEG	NEG	POS	POS
	POS	NEG	NEG	POS	POS
Russia-07_0110_Kurgan	NEG	NEG	NEG	POS	POS
Russia-11_0164_Kurgan	NEG	NEG	NEG	POS	POS
Russia-20_0367_Kurgan	NEG	NEG	NEG	POS	POS
Russia-21_0390_Chelyabinsk	NEG	NEG	NEG	POS	POS
>"South American/ Mid					
AGT1 (Argentina)	NEG	NEG	NEG	POS	POS
AGT120 (Argentina)	NEG	NEG	NEG	POS	POS
AGT67 (Argentina)	NEG	NEG	NEG	POS	POS
AGT9 (Argentina)	NEG	NEG	NEG	POS	POS
RA3 (Argentina)	NEG	NEG	NEG	POS	POS
RA6 (Argentina)	NEG	NEG	NEG	POS	POS
RA7 (Argentina)	NEG	NEG	NEG	POS	POS
Be62 (Brazil)	NEG	NEG	NEG	POS	POS
Bmb9393 (Brazil)	NEG	NEG	NEG	POS	POS
BRA36 (Brazil)	NEG	NEG	NEG	POS	POS
BZ48 (Brazil)	NEG	NEG	NEG	POS	POS
Gv51 (Brazil)	NEG	NEG	NEG	POS	POS
Gv69 (Brazil)	NEG	NEG	NEG	POS	POS
Gv88 (Brazil)	NEG	NEG	NEG	POS	POS
HC1335 (Brazil)	NEG	NEG	NEG	POS	POS
HC1340 (Brazil)	NEG	NEG	NEG	POS	POS
HC556 (Brazil)	NEG	NEG	NEG	POS	POS
HU25 (Brazil)	NEG	NEG	NEG	POS	POS
UB563 (Brazil)	NEG	NEG	NEG	POS	POS
2A8 (Czech Republic)	NEG	NEG	NEG	POS	POS
M1229 (Denmark)	NEG	NEG	NEG	POS	POS
FRICAR (France)	NEG	NEG	NEG	POS	POS
D90 (Germany)	NEG	NEG	NEG	POS	POS
LIT68 (Lithuania)	NEG	NEG	NEG	POS	POS
LIT76 (Lithuania)	NEG	NEG	NEG	POS	POS
HGSA142 (Portugal)	NEG	NEG	NEG	POS	POS
HGSA9 (Portugal)	NEG	NEG	NEG	POS	POS
HSJ216 (Portugal)	NEG	NEG	NEG	POS	POS
M278 (Portugal)	NEG	NEG	NEG	POS	POS
ES26 (Spain)	NEG	NEG	NEG	POS	POS
UK102 (UK)	NEG	NEG	NEG	POS	POS
URU34 (Uruguay)	NEG	NEG	NEG	POS	POS
IS-125	NEG	NEG	NEG	POS	POS
IS-157	NEG	NEG	NEG	POS	POS
Ecuador_1	AMB	NEG	NEG	POS	POS
Ecuador_2	AMB	NEG	NEG	POS	POS
Dresden_15ANR574263	AMB	NEG	NEG	POS	POS
UK-EMRSA-11	NEG	NEG	NEG	POS	POS
>"South American/ Mid					
BRA2 (Brazil)	NEG	NEG	NEG	POS	POS
CHL1 (Chile)	NEG	NEG	NEG	POS	POS
CHL151 (Chile)	NEG	NEG	NEG	POS	POS
UCL378 (Chile)	NEG	NEG	NEG	POS	POS
UP72 (Peru)	NEG	NEG	NEG	POS	POS
UP81 (Peru)	NEG	NEG	NEG	POS	POS
>"South American/ Mid					
UP1073 (Peru)	NEG	NEG	NEG	POS	POS

STRAIN / ISOLATE	REGULATORY GENES				
	agrIV	agrV		hld	setC
		agrV-ST1850	agrV-ST2198		setC / selX
	accessory gene regulator allele IV	accessory gene regulator alleles from S. argenteus group		haemolysin delta	Staphyl. exotoxin-like protein
>"South American/ Midd					
LIT89 (Lithuania)	NEG	NEG	NEG	POS	POS
MRSA_PR1 (Malaysia)	NEG	NEG	NEG	POS	POS
Riyadh-2888905-R	NEG	NEG	NEG	POS	POS
Riyadh-2888915-BC	NEG	NEG	NEG	POS	POS
Riyadh-2793706-R	AMB	NEG	NEG	POS	POS
>"South American/ Midd					
Riyadh_SSTI_18_3502925	POS	NEG	NEG	POS	POS
Riyadh-2822825-W	AMB	NEG		POS	POS
>"South American/ Midd					
UK-EMRSA-9	AMB	NEG	NEG	POS	POS
>"South American/ Midd					
Lome_HT20020815	AMB	NEG	NEG	POS	POS
>"South American/ Midd					
NA32 (Denmark)	NEG	NEG	NEG	POS	POS
H24 (Egypt)	NEG	NEG	NEG	POS	POS
ATCC BAA-39 (=HUSA304) (Hungary)	NEG	NEG	NEG	POS	POS
HUSA304 (Hungary)	NEG	NEG	NEG	POS	POS
HU106 (Hungary)	NEG	NEG	NEG	POS	POS
BSAC27 (UK)	NEG	NEG	NEG	POS	POS
NCTC13131, UK-EMRSA-4 (UK)	NEG	NEG	NEG	POS	POS
	NEG	NEG	NEG	POS	POS
Algiers_HT20040080	AMB	NEG	NEG	POS	POS
Dublin-DSH_AR09_0.0066	AMB	NEG	NEG	POS	POS
Dublin-DSH_AR09_0-0065	AMB	NEG	NEG	POS	POS
Dublin-DSH_Phenotype-III_84	NEG	NEG	NEG	POS	POS
Hong Kong_130	NEG	NEG	NEG	POS	POS
Kuwait_018	NEG	NEG	NEG	POS	POS
Perth_08-17726	NEG	NEG	NEG	POS	POS
Riyadh_Alfaisal/KKKSUH_86_MRS-14-279	NEG	NEG	NEG	POS	POS
Riyadh_Alfaisal-04_Z3861_831588	AMB	NEG	NEG	POS	POS
Riyadh_Alfaisal-30_S15724_1108013		NEG	NEG	POS	POS
Riyadh_Alfaisal-6_22A_13_B3992_397721	AMB	NEG	NEG	POS	POS
Riyadh_SSTI_52_3615482	AMB	NEG	NEG	POS	POS
Riyadh-2817276-2	AMB	NEG		POS	POS
Riyadh-2891670-W	AMB	NEG	NEG	POS	POS
Riyadh-3006920-W	AMB	NEG	NEG	POS	POS
Riyadh-R2567782	AMB	NEG	NEG	POS	POS
Russia-18_0252_Krasnoyarsk_SK2	NEG	NEG	NEG	POS	POS
UK-EMRSA-7	NEG	NEG	NEG	POS	POS
>"South American/ Midd					
Dublin-DSH_AR23_0073	AMB	NEG	NEG	POS	POS
>Related to "South Ame					
MRSA-OC3 (Russia)	NEG	NEG	NEG	POS	POS
Russia-12_0176_Krasnoyarsk	NEG	NEG	NEG	POS	POS
Russia-13_0180_Krasnoyarsk	AMB	NEG	NEG	POS	POS
Russia-16_0249_Krasnoyarsk	AMB	NEG	NEG	POS	POS
Russia-17_0250_Krasnoyarsk	NEG	NEG	NEG	POS	POS
>Related to "South Ame					
URU110 (Uruguay)	NEG	NEG	NEG	POS	POS
>Related to "South Ame					
DS_014 (Thailand)	NEG	NEG	NEG	POS	POS
>Unassigned Middle Eas					
Frankfurt_Oder_0490031797 (Libya)	AMB	NEG	NEG	POS	POS
Kuwait_192	AMB	NEG	NEG	POS	POS
Riyadh-9	AMB	NEG	NEG	POS	POS
Russia-24_0407_Moscow	NEG	NEG	NEG	POS	POS

STRAIN / ISOLATE	VIRULENCE : STAPHYLOCOCCAL SUPERANTIGEN/ENTEROTOXIN-LIKE GENES (SET/SSL)																
	ssl01												ssl02		ssl03		
	set6-var1_11	set6-var2_11	set6-var1_12	set6-var2_12	set6-var4_11	ssl01-RF122	ssl01/set6 (COL)	ssl01/set6 (Mu50+N315)	ssl01/set6 (MW2+MSSA476)	ssl01/set6 (MRSA252)	ssl01/set6 (RF122)	ssl01/set6 (other alleles)	ssl02/set7	ssl02/set7 (MRSA252)	ssl03/set8_probe 1	ssl03/set8_probe 2	ssl03/set8 (MRSA252, SAR0424)
	Staphylococcal superantigen-like protein 1												Staphylococcal superantigen-like protein 2		Staphylococcal superantigen-like protein 3		
**>"Eurasian Clade": TUR1**																	
TUR1 (Turkey)	POS	NEG	AMB	NEG	POS	NEG	POS	AMB	NEG	NEG	NEG	NEG	POS	AMB	POS	POS	NEG
TUR9 (Turkey)	POS	NEG	AMB	NEG	POS	NEG	POS	AMB	NEG	NEG	NEG	NEG	POS	AMB	POS	POS	NEG
**>"Eurasian Clade" : T013**																	
T0131 (China)	POS	NEG	AMB	NEG	POS	NEG	POS	AMB	NEG	NEG	NEG	NEG	POS	AMB	POS	POS	NEG
MU4 (Turkey)	POS	NEG	AMB	NEG	POS	NEG	POS	AMB	NEG	NEG	NEG	NEG	POS	AMB	POS	POS	NEG
DEU11 (Turkey)	POS	NEG	AMB	NEG	POS	NEG	POS	AMB	NEG	NEG	NEG	NEG	POS	AMB	POS	POS	NEG
DEU16 (Turkey)	POS	NEG	AMB	NEG	POS	NEG	POS	AMB	NEG	NEG	NEG	NEG	POS	AMB	POS	POS	NEG
Romania_Iasi_MRSA-07_54	POS	NEG	POS	NEG	POS	NEG	POS	AMB	NEG	NEG	NEG	NEG	POS	AMB	POS	POS	NEG
**>"Eurasian Clade": arsC-**																	
Romania_Iasi_MRSA-05_46	POS	NEG	AMB	NEG	POS	NEG	POS	AMB	NEG	NEG	NEG	NEG	POS	AMB	POS	POS	NEG
Romania_Iasi_MRSA-06_47	POS	NEG	POS	NEG	POS	NEG	POS	AMB	NEG	NEG	NEG	NEG	POS	AMB	POS	POS	NEG
**>"Eurasian Clade": 16K/**																	
CN79 (China)	POS	NEG	AMB	NEG	POS	NEG	POS	AMB	NEG	NEG	NEG	NEG	POS	AMB	POS	POS	NEG
CUHK_BJ2002 (China)	POS	NEG	AMB	NEG	POS	NEG	POS	AMB	NEG	NEG	NEG	NEG	POS	AMB	POS	POS	NEG
CUHK_BJ2007 (China)	POS	NEG	AMB	NEG	POS	NEG	POS	AMB	NEG	NEG	NEG	NEG	POS	AMB	POS	POS	NEG
3HK (Czech Republic)	POS	NEG	AMB	NEG	POS	NEG	POS	AMB	NEG	NEG	NEG	NEG	POS	AMB	POS	POS	NEG
HU109 (Hungary)	POS	NEG	AMB	NEG	POS	NEG	POS	AMB	NEG	NEG	NEG	NEG	POS	AMB	POS	POS	NEG
HUR18 (Hungary)	POS	NEG	AMB	NEG	POS	NEG	POS	AMB	NEG	NEG	NEG	NEG	POS	AMB	POS	POS	NEG
H482 (Romania)	POS	NEG	AMB	NEG	POS	NEG	POS	AMB	NEG	NEG	NEG	NEG	POS	AMB	POS	POS	NEG
16K (Russia)	POS	NEG	AMB	NEG	POS	NEG	POS	AMB	NEG	NEG	NEG	NEG	POS	AMB	POS	POS	NEG
DEU10 (Turkey)	POS	NEG	AMB	NEG	POS	NEG	POS	AMB	NEG	NEG	NEG	NEG	POS	AMB	POS	POS	NEG
DEU12 (Turkey)	POS	NEG	AMB	NEG	POS	NEG	POS	AMB	NEG	NEG	NEG	NEG	POS	AMB	POS	POS	NEG
DEU14 (Turkey)	POS	NEG	AMB	NEG	POS	NEG	POS	AMB	NEG	NEG	NEG	NEG	POS	AMB	POS	POS	NEG
DEU15 (Turkey)	POS	NEG	AMB	NEG	POS	NEG	POS	AMB	NEG	NEG	NEG	NEG	POS	AMB	POS	POS	NEG
DEU17 (Turkey)	POS	NEG	AMB	NEG	POS	NEG	POS	AMB	NEG	NEG	NEG	NEG	POS	AMB	POS	POS	NEG
DEU19 (Turkey)	POS	NEG	AMB	NEG	POS	NEG	POS	AMB	NEG	NEG	NEG	NEG	POS	AMB	POS	POS	NEG
DEU2 (Turkey)	POS	NEG	AMB	NEG	POS	NEG	POS	AMB	NEG	NEG	NEG	NEG	POS	AMB	POS	POS	NEG
DEU20 (Turkey)	POS	NEG	AMB	NEG	POS	NEG	POS	AMB	NEG	NEG	NEG	NEG	POS	AMB	POS	POS	NEG
DEU23 (Turkey)	POS	NEG	AMB	NEG	POS	NEG	POS	AMB	NEG	NEG	NEG	NEG	POS	AMB	POS	POS	NEG
DEU3 (Turkey)	POS	NEG	AMB	NEG	POS	NEG	POS	AMB	NEG	NEG	NEG	NEG	POS	AMB	POS	POS	NEG
DEU5 (Turkey)	POS	NEG	AMB	NEG	POS	NEG	POS	AMB	NEG	NEG	NEG	NEG	POS	AMB	POS	POS	NEG
DEU6 (Turkey)	POS	NEG	AMB	NEG	POS	NEG	POS	AMB	NEG	NEG	NEG	NEG	POS	AMB	POS	POS	NEG
DEU8 (Turkey)	POS	NEG	AMB	NEG	POS	NEG	POS	AMB	NEG	NEG	NEG	NEG	POS	AMB	POS	POS	NEG
HU11 (Turkey)	POS	NEG	AMB	NEG	POS	NEG	POS	AMB	NEG	NEG	NEG	NEG	POS	AMB	POS	POS	NEG
HU13 (Turkey)	POS	NEG	AMB	NEG	POS	NEG	POS	AMB	NEG	NEG	NEG	NEG	POS	AMB	POS	POS	NEG
HU14 (Turkey)	POS	NEG	AMB	NEG	POS	NEG	POS	AMB	NEG	NEG	NEG	NEG	POS	AMB	POS	POS	NEG
HU15 (Turkey)	POS	NEG	AMB	NEG	POS	NEG	POS	AMB	NEG	NEG	NEG	NEG	POS	AMB	POS	POS	NEG
HU16 (Turkey)	POS	NEG	AMB	NEG	POS	NEG	POS	AMB	NEG	NEG	NEG	NEG	POS	AMB	POS	POS	NEG
HU17 (Turkey)	POS	NEG	AMB	NEG	POS	NEG	POS	AMB	NEG	NEG	NEG	NEG	POS	AMB	POS	POS	NEG
HU21 (Turkey)	POS	NEG	AMB	NEG	POS	NEG	POS	AMB	NEG	NEG	NEG	NEG	POS	AMB	POS	POS	NEG
HU23 (Turkey)	POS	NEG	AMB	NEG	POS	NEG	POS	AMB	NEG	NEG	NEG	NEG	POS	AMB	POS	POS	NEG
HU26 (Turkey)	POS	NEG	AMB	NEG	POS	NEG	POS	AMB	NEG	NEG	NEG	NEG	POS	AMB	POS	POS	NEG
HU4 (Turkey)	POS	NEG	AMB	NEG	POS	NEG	POS	AMB	NEG	NEG	NEG	NEG	POS	AMB	POS	POS	NEG
HU5 (Turkey)	POS	NEG	AMB	NEG	POS	NEG	POS	AMB	NEG	NEG	NEG	NEG	POS	AMB	POS	POS	NEG
HU6 (Turkey)	POS	NEG	AMB	NEG	POS	NEG	POS	AMB	NEG	NEG	NEG	NEG	POS	AMB	POS	POS	NEG
HU7 (Turkey)	POS	NEG	AMB	NEG	POS	NEG	POS	AMB	NEG	NEG	NEG	NEG	POS	AMB	POS	POS	NEG
HU8 (Turkey)	POS	NEG	AMB	NEG	POS	NEG	POS	AMB	NEG	NEG	NEG	NEG	POS	AMB	POS	POS	NEG
HU9 (Turkey)	POS	NEG	AMB	NEG	POS	NEG	POS	AMB	NEG	NEG	NEG	NEG	POS	AMB	POS	POS	NEG
IU1 (Turkey)	POS	NEG	AMB	NEG	POS	NEG	POS	AMB	NEG	NEG	NEG	NEG	POS	AMB	POS	POS	NEG
IU10 (Turkey)	POS	NEG	AMB	NEG	POS	NEG	POS	AMB	NEG	NEG	NEG	NEG	POS	AMB	POS	POS	NEG
IU11 (Turkey)	POS	NEG	AMB	NEG	POS	NEG	POS	AMB	NEG	NEG	NEG	NEG	POS	AMB	POS	POS	NEG
IU12 (Turkey)	POS	NEG	AMB	NEG	POS	NEG	POS	AMB	NEG	NEG	NEG	NEG	POS	AMB	POS	POS	NEG
IU13 (Turkey)	POS	NEG	AMB	NEG	POS	NEG	POS	AMB	NEG	NEG	NEG	NEG	POS	AMB	POS	POS	NEG
IU15 (Turkey)	POS	NEG	AMB	NEG	POS	NEG	POS	AMB	NEG	NEG	NEG	NEG	POS	AMB	POS	POS	NEG
IU18 (Turkey)	POS	NEG	AMB	NEG	POS	NEG	POS	AMB	NEG	NEG	NEG	NEG	POS	AMB	POS	POS	NEG
IU19 (Turkey)	POS	NEG	AMB	NEG	POS	NEG	POS	AMB	NEG	NEG	NEG	NEG	POS	AMB	POS	POS	NEG
IU2 (Turkey)	POS	NEG	AMB	NEG	POS	NEG	POS	AMB	NEG	NEG	NEG	NEG	POS	AMB	POS	POS	NEG
IU4 (Turkey)	POS	NEG	AMB	NEG	POS	NEG	POS	AMB	NEG	NEG	NEG	NEG	POS	AMB	POS	POS	NEG
IU6 (Turkey)	POS	NEG	AMB	NEG	POS	NEG	POS	AMB	NEG	NEG	NEG	NEG	POS	AMB	POS	POS	NEG
IU7 (Turkey)	POS	NEG	AMB	NEG	POS	NEG	POS	AMB	NEG	NEG	NEG	NEG	POS	AMB	POS	POS	NEG
IU9 (Turkey)	POS	NEG	AMB	NEG	POS	NEG	POS	AMB	NEG	NEG	NEG	NEG	POS	AMB	POS	POS	NEG
MU1 (Turkey)	POS	NEG	AMB	NEG	POS	NEG	POS	AMB	NEG	NEG	NEG	NEG	POS	AMB	POS	POS	NEG
MU10 (Turkey)	POS	NEG	AMB	NEG	POS	NEG	POS	AMB	NEG	NEG	NEG	NEG	POS	AMB	POS	POS	NEG
MU20 (Turkey)	POS	NEG	AMB	NEG	POS	NEG	POS	AMB	NEG	NEG	NEG	NEG	POS	AMB	POS	POS	NEG
Mu3 (Turkey)	POS	NEG	AMB	NEG	POS	NEG	POS	AMB	NEG	NEG	NEG	NEG	POS	AMB	POS	POS	NEG
MU5 (Turkey)	POS	NEG	AMB	NEG	POS	NEG	POS	AMB	NEG	NEG	NEG	NEG	POS	AMB	POS	POS	NEG
MU6 (Turkey)	POS	NEG	AMB	NEG	POS	NEG	POS	AMB	NEG	NEG	NEG	NEG	POS	AMB	POS	POS	NEG
MU7 (Turkey)	POS	NEG	AMB	NEG	POS	NEG	POS	AMB	NEG	NEG	NEG	NEG	POS	AMB	POS	POS	NEG
TUR27 (Turkey)	POS	NEG	AMB	NEG	POS	NEG	POS	AMB	NEG	NEG	NEG	NEG	POS	AMB	POS	POS	NEG
NCTR 32S	POS	NEG	AMB	NEG	POS	NEG	POS	AMB	NEG	NEG	NEG	NEG	POS	AMB	POS	POS	NEG
SA02_A4 = Russia-06_0085_Moscow	POS	NEG	AMB	NEG	POS	NEG	POS	AMB	NEG	NEG	NEG	NEG	POS	AMB	POS	POS	NEG
	POS	NEG	POS	NEG	POS	NEG	POS	AMB	NEG	NEG	NEG	NEG	POS	AMB	POS	POS	NEG
Dresden_08V35987 (Turkey)	POS	NEG	AMB	NEG	POS	NEG	POS	AMB	NEG	NEG	NEG	NEG	POS	NEG	POS	POS	NEG
Dresden_17ANR577152	POS	NEG	AMB	NEG	POS	NEG	POS	AMB	NEG	NEG	NEG	NEG	POS	NEG	POS	POS	NEG
Dresden_17ANR580374 (Makedonia)	POS	NEG	AMB	NEG	POS	NEG	POS	AMB	NEG	NEG	NEG	NEG	POS	NEG	POS	POS	NEG
Hong Kong_69-II	POS	NEG	POS	NEG	POS	NEG	POS	AMB	NEG	NEG	NEG	NEG	POS	AMB	POS	POS	NEG
Hong Kong_93	POS	NEG	POS	NEG	POS	NEG	POS	AMB	NEG	NEG	NEG	NEG	POS	AMB	POS	POS	NEG
Rawalpindi_Kidney Center_03_SK1	POS	NEG	POS	NEG	POS	NEG	POS	AMB	NEG	NEG	NEG	NEG	POS	AMB	POS	POS	NEG
Rawalpindi_Kidney Center_08	POS	NEG	POS	NEG	POS	NEG	POS	AMB	NEG	NEG	NEG	NEG	POS	NEG	POS	POS	NEG
Rawalpindi_Kidney Center_10	POS	NEG	POS	NEG	POS	NEG	POS	AMB	NEG	NEG	NEG	NEG	POS	AMB	POS	POS	NEG
Rawalpindi_Kidney Center_19	POS	NEG	POS	NEG	POS	NEG	POS	AMB	NEG	NEG	NEG	NEG	POS	AMB	POS	POS	NEG
Rawalpindi_Kidney Center_50_SK1	POS	NEG	AMB	NEG	POS	NEG	POS	AMB	NEG	NEG	NEG	NEG	POS	NEG	POS	POS	NEG
Romania_Iasi_BC-49_430	POS	NEG	POS	NEG	POS	NEG	POS	AMB	NEG	NEG	NEG	NEG	POS	AMB	POS	POS	NEG
Romania_Iasi_MRSA_284	POS	NEG	AMB	NEG	POS	NEG	POS	AMB	NEG	NEG	NEG	NEG	POS	AMB	POS	POS	NEG
Romania_Iasi_MRSA-32_318	POS	NEG	AMB	NEG	POS	NEG	POS	AMB	NEG	NEG	NEG	NEG	POS	NEG	POS	POS	NEG
Romania_Iasi_MRSA-54_430	POS	NEG	POS	NEG	POS	NEG	POS	AMB	NEG	NEG	NEG	NEG	POS	AMB	POS	POS	NEG
Romania_Iasi_SSTI-01_2	POS	NEG	POS	NEG	POS	NEG	POS	AMB	NEG	NEG	NEG	NEG	POS	AMB	POS	POS	NEG
Romania_Iasi_SSTI-10_101	POS	NEG	AMB	NEG	POS	NEG	POS	AMB	NEG	NEG	NEG	NEG	POS	AMB	POS	POS	NEG
Romania_Iasi_SSTI-11_106	POS	NEG	POS	NEG	POS	NEG	POS	AMB	NEG	NEG	NEG	NEG	POS	NEG	POS	POS	NEG
Russia-01_0001_SaintPetersburg	POS	NEG	POS	NEG	POS	NEG	POS	AMB	NEG	NEG	NEG	NEG	POS	AMB	POS	POS	NEG
Russia-02_0004_SaintPetersburg	POS	NEG	POS	NEG	POS	NEG	POS	AMB	NEG	NEG	NEG	NEG	POS	AMB	POS	POS	NEG
Russia-03_0057_SaintPetersburg	POS	NEG	NEG	NEG	POS	NEG	POS	AMB	NEG	NEG	NEG	NEG	POS	NEG	POS	POS	NEG
Russia-04_0076_Moscow	POS	NEG	POS	NEG	POS	NEG	POS	AMB	NEG	NEG	NEG	NEG	POS	NEG	POS	POS	NEG
Russia-05_0078_Moscow	POS	NEG	POS	NEG	POS	NEG	POS	AMB	NEG	NEG	NEG	NEG	POS	AMB	POS	POS	NEG
Russia-08_0135_Moscow	POS	NEG	POS	NEG	POS	NEG	POS	AMB	NEG	NEG	NEG	NEG	POS	AMB	POS	POS	NEG
Russia-09_0150_Kurgan	POS	NEG	POS	NEG	POS	NEG	POS	AMB	NEG	NEG	NEG	NEG	POS	NEG	POS	POS	NEG
Russia-10_0162_Kurgan	POS	NEG	POS	NEG	POS	NEG	POS	AMB	NEG	NEG	NEG	NEG	POS	NEG	POS	POS	NEG
Russia-14_0184_Moscow	POS	NEG	AMB	NEG	POS	NEG	POS	AMB	NEG	NEG	NEG	NEG	POS	NEG	POS	POS	NEG
Russia-15_0232_SaintPetersburg	POS	NEG	POS	NEG	POS	NEG	POS	AMB	NEG	NEG	NEG	NEG	POS	NEG	POS	POS	NEG
Russia-22_0391_Chelyabinsk	POS	NEG	POS	NEG	POS	NEG	POS	AMB	NEG	NEG	NEG	NEG	POS	AMB	POS	POS	NEG
Russia-23_0392_Chelyabinsk	POS	NEG	POS	NEG	POS	NEG	POS	AMB	NEG	NEG	NEG	NEG	POS	NEG	POS	POS	NEG
**>"Eurasian Clade" : IU17**																	
IU17 (Turkey)	POS	NEG	AMB	NEG	POS	NEG	POS	AMB	NEG	NEG	NEG	NEG	POS	AMB	POS	POS	NEG
IU20 (Turkey)	POS	NEG	AMB	NEG	POS	NEG	POS	AMB	NEG	NEG	NEG	NEG	POS	AMB	POS	POS	NEG

STRAIN / ISOLATE	VIRULENCE : STAPHYLOCOCCAL SUPERANTIGEN/ENTEROTOXIN-LIKE GENES (SET/SSL)																
	ssl01												ssl02		ssl03		
	set6-var1_11	set6-var2_11	set6-var1_12	set6-var2_12	set6-var4_11	ssl01-RF122	ssl01/set6 (COL)	ssl01/set6 (Mu50+N315)	ssl01/set6 (MW2+MSSA476)	ssl01/set6 (MRSA252)	ssl01/set6 (RF122)	ssl01/set6 (other alleles)	ssl02/set7	ssl02/set7 (MRSA252)	ssl03/set8_probe 1	ssl03/set8_probe 2	ssl03/set8 (MRSA252, SAR0424)
	Staphylococcal superantigen-like protein 1												Staphylococcal superantigen-like protein 2		Staphylococcal superantigen-like protein 3		
**>"Eurasian Clade": Spor[illegible]**																	
Dresden_16ANR581769	POS	NEG	NEG	NEG	POS	NEG	POS	AMB	NEG	NEG	NEG	NEG	POS	NEG	POS	POS	NEG
Dresden_17ANR578769	POS	NEG	POS	NEG	POS	NEG	POS	AMB	NEG	NEG	NEG	NEG	POS	NEG	POS	POS	NEG
**>"Eurasian Clade": Kuw[illegible]**																	
Kuwait_306	POS	NEG	POS	NEG	POS	NEG	POS	AMB	NEG	NEG	NEG	NEG	POS	AMB	POS	POS	NEG
Kuwait_301	POS	NEG	POS	NEG	POS	NEG	POS	AMB	NEG	NEG	NEG	NEG	POS	AMB	POS	POS	NEG
**>"European Clade": "Gr[illegible]**																	
GRE18 (Greece)	POS	NEG	AMB	NEG	POS	NEG	POS	AMB	NEG	NEG	NEG	NEG	POS	AMB	POS	POS	NEG
GRE317 (Greece)	POS	NEG	AMB	NEG	POS	NEG	POS	AMB	NEG	NEG	NEG	NEG	POS	AMB	POS	POS	NEG
GRE4 (Greece)	POS	NEG	AMB	NEG	POS	NEG	POS	AMB	NEG	NEG	NEG	NEG	POS	AMB	POS	POS	NEG
R35 (USA)	POS	NEG	AMB	NEG	POS	NEG	POS	AMB	NEG	NEG	NEG	NEG	POS	AMB	POS	POS	NEG
Casablanca_HT20060548	POS	NEG	POS	NEG	POS	NEG	POS	AMB	NEG	NEG	NEG	NEG	POS	AMB	POS	POS	NEG
Casablanca_HT20060550	POS	NEG	POS	NEG	POS	NEG	POS	AMB	NEG	NEG	NEG	NEG	POS	NEG	POS	POS	NEG
Dresden_01V34572 (Greece)						NEG	POS	NEG	NEG	NEG		NEG	POS	NEG	POS	POS	NEG
Dresden_01V36469	POS	NEG	POS	NEG	POS	NEG	POS	AMB	NEG	NEG	NEG	NEG	POS	NEG	POS	POS	NEG
Dresden_01V39123	POS	NEG	POS	NEG	POS	NEG	POS	AMB	NEG	NEG	NEG	NEG	POS	AMB	POS	POS	NEG
Dresden_01V39143	POS	NEG	AMB	NEG	POS	NEG	POS	AMB	NEG	NEG	NEG	NEG	POS	NEG	POS	POS	NEG
Greece 1_3680_Harmony	POS	NEG	NEG	NEG	POS	NEG	POS	AMB	NEG	NEG	NEG	NEG	POS	NEG	POS	POS	NEG
**>"European Clade": UK-[illegible]**																	
ANS46 (Australia)	POS	NEG	AMB	NEG	POS	NEG	POS	AMB	NEG	NEG	NEG	NEG	POS	AMB	POS	POS	NEG
LHH1 (USA)	POS	NEG	AMB	NEG	POS	NEG	POS	AMB	NEG	NEG	NEG	NEG	POS	AMB	POS	POS	NEG
Dublin-DSH_AR01_0-1085	POS	NEG	AMB	NEG	POS	NEG	POS	AMB	NEG	NEG		NEG	POS	NEG	POS	POS	NEG
Dublin-DSH_AR01_0-1118	POS	NEG	AMB	NEG	POS	NEG	POS	AMB	NEG	NEG	NEG	NEG	POS	NEG	POS	POS	NEG
Dublin-DSH_AR15_0098	POS	NEG	AMB	NEG	POS	NEG	POS	AMB	NEG	NEG		NEG	POS	AMB	POS	AMB	NEG
Dublin-DSH_AR15_0104	POS	NEG	AMB	NEG	POS	NEG	POS	AMB	NEG	NEG	NEG	NEG	POS	NEG	POS	POS	NEG
UK_NCTC11939_UK-1_Harmony	POS	NEG	NEG	NEG	POS	NEG	POS	AMB	NEG	NEG	NEG	NEG	POS	NEG	POS	POS	NEG
**>"European Clade": BK2**																	
BK2421 (USA)	POS	NEG	AMB	NEG	POS	NEG	POS	AMB	NEG	NEG	NEG	NEG	POS	AMB	POS	POS	NEG
**>"Aussi/NZ Clade", JKD6**																	
JKD6009 (Australia)	POS	NEG	AMB	NEG	POS	NEG	POS	AMB	NEG	NEG	NEG	NEG	POS	AMB	POS	POS	NEG
JKD6008 (New Zealand)	POS	NEG	AMB	NEG	POS	NEG	POS	AMB	NEG	NEG	NEG	NEG	POS	AMB	POS	POS	NEG
Perth_2015-S20209	POS	NEG	AMB	NEG	POS	NEG	POS	AMB	NEG	NEG	NEG	NEG	POS	AMB	POS	POS	NEG
Perth_2015-S20305	POS	NEG	AMB	NEG	POS	NEG	POS	AMB	NEG	NEG	NEG	NEG	POS	AMB	POS	POS	NEG
Perth_2015-S20603	POS	NEG	AMB	NEG	POS	NEG	POS	AMB	NEG	NEG	NEG	NEG	POS	AMB	POS	POS	NEG
Perth_2015-S20607	POS	NEG	AMB	NEG	POS	NEG	POS	AMB	NEG	NEG	NEG	NEG	POS	AMB	POS	POS	NEG
Perth_2015-S20608	POS	NEG	AMB	NEG	POS	NEG	POS	AMB	NEG	NEG	NEG	NEG	POS	AMB	POS	POS	NEG
Perth_2015-S20610	POS	NEG	AMB	NEG	POS	NEG	POS	AMB	NEG	NEG	NEG	NEG	POS	AMB	POS	POS	NEG
Perth_2015-S20613	POS	NEG	AMB	NEG	POS	NEG	POS	AMB	NEG	NEG	NEG	NEG	POS	AMB	POS	POS	NEG
Perth_2015-S20617	NEG	NEG	NEG	NEG	NEG	NEG	NEG	NEG	NEG	NEG	NEG	NEG	POS	AMB	POS	POS	NEG
Perth_2015-S20622	POS	NEG	AMB	NEG	POS	NEG	POS	AMB	NEG	NEG	NEG	NEG	POS	AMB	POS	POS	NEG
Perth_2015-S23619	POS	NEG	AMB	NEG	POS	NEG	POS	AMB	NEG	NEG	NEG	NEG	POS	AMB	POS	POS	NEG
Perth_2015-S41210	POS	NEG	AMB	NEG	POS	NEG	POS	AMB	NEG	NEG	NEG	NEG	POS	AMB	POS	POS	NEG
Perth_2015-S51001	POS	NEG	AMB	NEG	POS	NEG	POS	AMB	NEG	NEG	NEG	NEG	POS	AMB	POS	POS	NEG
Perth_2015-S51003	POS	NEG	AMB	NEG	POS	NEG	POS	AMB	NEG	NEG	NEG	NEG	POS	AMB	POS	POS	NEG
Perth_2015-S51006	POS	NEG	AMB	NEG	POS	NEG	POS	AMB	NEG	NEG	NEG	NEG	POS	AMB	POS	POS	NEG
Perth_2015-S51007	POS	NEG	AMB	NEG	POS	NEG	POS	AMB	NEG	NEG	NEG	NEG	POS	AMB	POS	POS	NEG
Perth_2015-S51008	POS	NEG	AMB	NEG	POS	NEG	POS	AMB	NEG	NEG	NEG	NEG	POS	AMB	POS	POS	NEG
Perth_2015-S51014	POS	NEG	AMB	NEG	POS	NEG	POS	AMB	NEG	NEG	NEG	NEG	POS	AMB	POS	POS	NEG
Perth_2015-S51408	POS	NEG	AMB	NEG	POS	NEG	POS	AMB	NEG	NEG	NEG	NEG	POS	AMB	POS	POS	NEG
Perth_2016-S20614	POS	NEG	AMB	NEG	POS	NEG	POS	AMB	NEG	NEG	NEG	NEG	POS	AMB	POS	POS	NEG
Perth_2016-S23614	POS	NEG	AMB	NEG	POS	NEG	POS	AMB	NEG	NEG	NEG	NEG	POS	AMB	POS	POS	NEG
Perth_2016-S42009	POS	NEG	AMB	NEG	POS	NEG	POS	AMB	NEG	NEG	NEG	NEG	POS	AMB	POS	POS	NEG
Perth_2016-S51001	POS	NEG	AMB	NEG	POS	NEG	POS	AMB	NEG	NEG	NEG	NEG	POS	AMB	POS	POS	NEG
Perth_2016-S51403	POS	NEG	AMB	NEG	POS	NEG	POS	AMB	NEG	NEG	NEG	NEG	POS	AMB	POS	POS	NEG
Perth_07 RPA 20	POS	NEG	NEG	NEG	POS	NEG	POS	AMB	NEG	NEG	NEG	NEG	POS	NEG	POS	POS	NEG
Perth_08-19088	POS	NEG	POS	NEG	POS	NEG	POS	AMB	NEG	NEG		NEG	POS	AMB	POS	POS	NEG
Perth_01-15351	POS	NEG	AMB	NEG	POS	NEG	POS	AMB	NEG	NEG		NEG	POS	NEG	POS	POS	NEG
Perth_01-15357	POS	NEG	NEG	NEG	POS	NEG	POS	AMB	NEG	NEG	NEG	NEG	POS	NEG	POS	POS	NEG
Perth_01-15419	POS	NEG	NEG	NEG	POS	NEG	POS	AMB	NEG	NEG		NEG	POS	NEG	POS	POS	NEG
Perth_01-16309	POS	NEG	NEG	NEG	POS	NEG	POS	AMB	NEG	NEG		NEG	POS	NEG	POS	POS	NEG
Perth_03 SNP 95	POS	NEG	POS	NEG	POS	NEG	NEG	POS	NEG	NEG		NEG	POS	AMB	POS	POS	NEG
Perth_2003_AH_47	POS	POS	POS	NEG	POS	NEG	POS	AMB	NEG	POS		NEG	POS	AMB	POS	POS	NEG
**>"Australian/NZ Clade":**																	
Perth_2015-S20623	POS	NEG	AMB	NEG	POS	NEG	POS	AMB	NEG	NEG	NEG	NEG	POS	AMB	POS	POS	NEG
Perth_2015-S20826	POS	NEG	AMB	NEG	POS	NEG	POS	AMB	NEG	NEG	NEG	NEG	POS	AMB	POS	POS	NEG
Perth_05 NH 56	POS	NEG	POS	NEG	POS	NEG	POS	AMB	NEG	NEG	NEG	NEG	POS	NEG	POS	POS	NEG
**>CC5-MRSA-III LA MRSA**																	
Delbrück 38 isolate from domestic turkey	POS	NEG	POS	NEG	POS	NEG	POS	AMB	NEG	NEG		NEG	POS	AMB	POS	POS	NEG
**> Staph. pseudintermed**																	
KM1381	NEG	NEG	NEG	NEG	NEG	NEG	NEG	NEG	NEG	NEG	NEG	NEG	NEG	NEG	NEG	NEG	NEG
**>"South-East Asian Clad**																	
M92 (Canada)	POS	NEG	AMB	NEG	POS	NEG	POS	AMB	NEG	NEG	NEG	NEG	POS	AMB	POS	POS	NEG
CHI59 (China)	NEG	NEG	NEG	NEG	NEG	NEG	NEG	NEG	NEG	NEG	NEG	NEG	NEG	NEG	NEG	NEG	NEG
CHI61 (China)	NEG	NEG	NEG	NEG	NEG	NEG	NEG	NEG	NEG	NEG	NEG	NEG	NEG	NEG	NEG	NEG	NEG
CUHK_HK1997 (China)	POS	NEG	AMB	NEG	POS	NEG	POS	AMB	NEG	NEG	NEG	NEG	POS	AMB	POS	POS	NEG
M996 (China)	POS	NEG	AMB	NEG	POS	NEG	POS	AMB	NEG	NEG	NEG	NEG	POS	AMB	POS	POS	NEG
H211 (Denmark)	POS	NEG	AMB	NEG	POS	NEG	POS	AMB	NEG	NEG	NEG	NEG	POS	AMB	POS	POS	NEG
H216 (Denmark)	POS	NEG	AMB	NEG	POS	NEG	POS	AMB	NEG	NEG	NEG	NEG	POS	AMB	POS	POS	NEG
D71 (Germany)	POS	NEG	AMB	NEG	POS	NEG	POS	AMB	NEG	NEG	NEG	NEG	POS	AMB	POS	POS	NEG
NMR05 (India)	POS	NEG	AMB	NEG	POS	NEG	POS	AMB	NEG	NEG	NEG	NEG	POS	AMB	POS	POS	NEG
MAL11 (Malaysia)	POS	NEG	AMB	NEG	POS	NEG	POS	AMB	NEG	NEG	NEG	NEG	POS	AMB	POS	POS	NEG
MAL9 (Malaysia)	POS	NEG	AMB	NEG	POS	NEG	POS	AMB	NEG	NEG	NEG	NEG	POS	AMB	POS	POS	NEG
Na21 (Sri Lanka)	POS	NEG	AMB	NEG	POS	NEG	POS	AMB	NEG	NEG	NEG	NEG	POS	AMB	POS	POS	NEG
M592 (Syria)	POS	NEG	AMB	NEG	POS	NEG	POS	AMB	NEG	NEG	NEG	NEG	POS	AMB	POS	POS	NEG
H202 (Thailand)	POS	NEG	AMB	NEG	POS	NEG	POS	AMB	NEG	NEG	NEG	NEG	POS	AMB	POS	POS	NEG
S102 (Thailand)	POS	NEG	AMB	NEG	POS	NEG	POS	AMB	NEG	NEG	NEG	NEG	POS	AMB	POS	POS	NEG
S130 (Thailand)	POS	NEG	AMB	NEG	POS	NEG	POS	AMB	NEG	NEG	NEG	NEG	POS	AMB	POS	POS	NEG
S40 (Thailand)	POS	NEG	AMB	NEG	POS	NEG	POS	AMB	NEG	NEG	NEG	NEG	POS	AMB	POS	POS	NEG
S71 (Thailand)	POS	NEG	AMB	NEG	POS	NEG	POS	AMB	NEG	NEG	NEG	NEG	POS	AMB	POS	POS	NEG
S87 (Thailand)	POS	NEG	AMB	NEG	POS	NEG	POS	AMB	NEG	NEG	NEG	NEG	POS	AMB	POS	POS	NEG
S93 (Thailand)	POS	NEG	AMB	NEG	POS	NEG	POS	AMB	NEG	NEG	NEG	NEG	POS	AMB	POS	POS	NEG
US_002 (Thailand)	POS	NEG	AMB	NEG	POS	NEG	POS	AMB	NEG	NEG	NEG	NEG	POS	AMB	POS	POS	NEG
US_017 (Thailand)	POS	NEG	AMB	NEG	POS	NEG	POS	AMB	NEG	NEG	NEG	NEG	POS	AMB	POS	POS	NEG
WAMC6102 (Thailand)	POS	NEG	AMB	NEG	POS	NEG	POS	AMB	NEG	NEG	NEG	NEG	POS	AMB	POS	POS	NEG
NCTC_13626_TW20 (UK)	POS	NEG	AMB	NEG	POS	NEG	POS	AMB	NEG	NEG	NEG	NEG	POS	AMB	POS	POS	NEG
BSAC3021 (UK)	POS	NEG	AMB	NEG	POS	NEG	POS	AMB	NEG	NEG	NEG	NEG	POS	AMB	POS	POS	NEG
BSAC3046 (UK)	POS	NEG	AMB	NEG	POS	NEG	POS	AMB	NEG	NEG	NEG	NEG	POS	AMB	POS	POS	NEG
1193_SAUR (USA)	POS	NEG	AMB	NEG	POS	NEG	POS	AMB	NEG	NEG	NEG	NEG	POS	AMB	POS	POS	NEG
1194_SAUR (USA)	POS	NEG	AMB	NEG	POS	NEG	POS	AMB	NEG	NEG	NEG	NEG	POS	AMB	POS	POS	NEG

STRAIN / ISOLATE	VIRULENCE : STAPHYLOCOCCAL SUPERANTIGEN/ENTEROTOXIN-LIKE GENES (SET/SSL)																
	ssl01												ssl02		ssl03		
	set6-var1_11	set6-var2_11	set6-var1_12	set6-var2_12	set6-var4_11	ssl01-RF122	ssl01/set6 (COL)	ssl01/set6 (Mu50+N315)	ssl01/set6 (MW2+MSSA476)	ssl01/set6 (MRSA252)	ssl01/set6 (RF122)	ssl01/set6 (other alleles)	ssl02/set7	ssl02/set7 (MRSA252)	ssl03/set8_probe 1	ssl03/set8_probe 2	ssl03/set8 (MRSA252, SAR0424)
	Staphylococcal superantigen-like protein 1												Staphylococcal superantigen-like protein 2		Staphylococcal superantigen-like protein 3		
>"South-East Asian Clade": TW20 (Irish AR44), c																	
1195_SAUR (USA)	POS	NEG	AMB	NEG	POS	NEG	POS	AMB	NEG	NEG	NEG	NEG	POS	AMB	POS	POS	NEG
FVRH6002 (USA)	POS	NEG	AMB	NEG	POS	NEG	POS	AMB	NEG	NEG	NEG	NEG	POS	AMB	POS	POS	NEG
KINW6048 (USA)	POS	NEG	AMB	NEG	POS	NEG	POS	AMB	NEG	NEG	NEG	NEG	POS	AMB	POS	POS	NEG
LAMC0011 (USA)	POS	NEG	AMB	NEG	POS	NEG	POS	AMB	NEG	NEG	NEG	NEG	POS	AMB	POS	POS	NEG
SJOS6053 (USA)	POS	NEG	AMB	NEG	POS	NEG	POS	AMB	NEG	NEG	NEG	NEG	POS	AMB	POS	POS	NEG
SJOS6072 (USA)	POS	NEG	AMB	NEG	POS	NEG	POS	AMB	NEG	NEG	NEG	NEG	POS	AMB	POS	POS	NEG
UCIM6042 (USA)	POS	NEG	AMB	NEG	POS	NEG	POS	AMB	NEG	NEG	NEG	NEG	POS	AMB	POS	POS	NEG
Perth_2015-S31902	POS	NEG	AMB	NEG	POS	NEG	POS	AMB	NEG	NEG	NEG	NEG	POS	AMB	POS	POS	NEG
Perth_2015-S31904	POS	NEG	AMB	NEG	POS	NEG	POS	AMB	NEG	NEG	NEG	NEG	POS	AMB	POS	POS	NEG
Perth_2015-S31913	POS	NEG	AMB	NEG	POS	NEG	POS	AMB	NEG	NEG	NEG	NEG	POS	AMB	POS	POS	NEG
Perth_2015-S31918	POS	NEG	AMB	NEG	POS	NEG	POS	AMB	NEG	NEG	NEG	NEG	POS	AMB	POS	POS	NEG
Dublin-DSH_AR44_E1183	POS	NEG	AMB	NEG	POS	NEG	POS	AMB	NEG	NEG	NEG	NEG	POS	NEG	POS	POS	NEG
Dublin-DSH_Unfamiliar-3_E1520	POS	NEG	NEG	NEG	POS	NEG	POS	AMB	NEG	NEG		NEG	POS	NEG	POS	POS	NEG
Hong Kong_042	POS	NEG	AMB	NEG	POS	NEG	POS	AMB	NEG	NEG	NEG	NEG	POS	NEG	POS	POS	NEG
Hong Kong_101	POS	NEG	AMB	NEG	POS	NEG	POS	AMB	NEG	NEG	NEG	NEG	POS	NEG	POS	POS	NEG
Hong Kong_111	POS	NEG	POS	NEG	POS	NEG	POS	AMB	NEG	NEG	NEG	NEG	POS	AMB	POS	POS	NEG
Hong Kong_113	POS	NEG	NEG	NEG	POS	NEG	POS	AMB	NEG	NEG	NEG	NEG	POS	NEG	POS	POS	NEG
Hong Kong_115	POS	NEG	POS	NEG	POS	NEG	POS	AMB	NEG	NEG	NEG	NEG	POS	NEG	POS	POS	NEG
Hong Kong_118	POS	NEG	POS	NEG	POS	NEG	POS	AMB	NEG	NEG	NEG	NEG	POS	AMB	POS	POS	NEG
Hong Kong_125	POS	NEG	POS	NEG	POS	NEG	POS	AMB	NEG	NEG	NEG	NEG	POS	AMB	POS	POS	NEG
Hong Kong_129	POS	NEG	POS	NEG	POS	NEG	POS	AMB	NEG	NEG	NEG	NEG	POS	NEG	POS	POS	NEG
Hong Kong_133	POS	NEG	POS	NEG	POS	NEG	POS	AMB	NEG	NEG	NEG	NEG	POS	AMB	POS	POS	NEG
Kuwait_009	POS	NEG	POS	NEG	POS	NEG	POS	AMB	NEG	NEG	NEG	NEG	POS	AMB	POS	POS	NEG
Kuwait_103	POS	NEG	POS	NEG	POS	NEG	POS	AMB	NEG	NEG	NEG	NEG	POS	AMB	POS	POS	NEG
Kuwait_107	POS	NEG	POS	NEG	POS	NEG	POS	AMB	NEG	NEG	NEG	NEG	POS	AMB	POS	POS	NEG
Perth_03 AH 88	POS	NEG	NEG	NEG	POS	NEG	POS	AMB	NEG	NEG		NEG	POS	NEG	POS	POS	NEG
Perth_08-16905	POS	NEG	AMB	NEG	POS	NEG	POS	AMB	NEG	NEG	NEG	NEG	POS	NEG	POS	POS	NEG
Riyadh_Alfaisal-10_SICU_13_B5672_1117353	POS	NEG	POS	NEG	POS	NEG	POS	AMB	NEG	NEG	NEG	NEG	POS	AMB	POS	POS	NEG
Riyadh_SSTI_17_3508602	POS	NEG	POS	NEG	POS	NEG	POS	AMB	NEG	NEG	NEG	NEG	POS	AMB	POS	POS	NEG
Riyadh-2822088-R	POS	NEG	POS	NEG	POS	NEG	POS	AMB	NEG	NEG	NEG	NEG	POS	AMB	POS	POS	NEG
Trinidad&Tobago_2012_195 (619/12)	POS	NEG	POS	NEG	POS	NEG	POS	AMB	NEG	NEG	NEG	NEG	POS	AMB	POS	POS	NEG
Trinidad&Tobago_2012_370 (1112-52177)	POS	NEG	POS	NEG	POS	NEG	NEG	POS	NEG	NEG	NEG	NEG	POS	AMB	POS	POS	NEG
Trinidad&Tobago_2012_378 (4139)	POS	NEG	POS	NEG	POS	NEG	POS	AMB	NEG	NEG	NEG	NEG	POS	AMB	POS	POS	NEG
Trinidad&Tobago_2013_PA21	POS	NEG	NEG	NEG	POS	NEG	POS	AMB	NEG	NEG	NEG	NEG	POS	NEG	POS	POS	NEG
Trinidad&Tobago_2013_PA22	POS	NEG	AMB	NEG	POS	NEG	POS	AMB	NEG	NEG	NEG	NEG	POS	AMB	POS	POS	NEG
Trinidad&Tobago_2912_340 (8523/12)	POS	NEG	POS	NEG	POS	NEG	POS	AMB	NEG	NEG	NEG	NEG	POS	AMB	POS	POS	NEG
Trinidad&Tobago_MRSA2010_105	POS	NEG	POS	NEG	POS	NEG	POS	AMB	NEG	NEG	NEG	NEG	POS	AMB	POS	POS	NEG
Trinidad&Tobago_MRSA2010_108	POS	NEG	POS	NEG	POS	NEG	POS	AMB	NEG	NEG	NEG	NEG	POS	AMB	POS	POS	NEG
Trinidad&Tobago_MRSA2010_33	POS	NEG	AMB	NEG	POS	NEG	POS	AMB	NEG	NEG	NEG	NEG	POS	AMB	POS	POS	NEG
Trinidad&Tobago_MRSA2010_79	POS	NEG	NEG	NEG	POS	NEG	POS	AMB	NEG	NEG	NEG	NEG	POS	NEG	POS	POS	NEG
Trinidad&Tobago_MRSA2010_88	POS	NEG	AMB	NEG	POS	NEG	POS	AMB	NEG	NEG	NEG	NEG	POS	AMB	POS	POS	NEG
Trinidad&Tobago_MRSA2010_97	POS	NEG	POS	NEG	POS	NEG	POS	AMB	NEG	NEG	NEG	NEG	POS	AMB	POS	POS	NEG
Trinidad&Tobago_SSTI 2012_031	POS	NEG	POS	NEG	POS	NEG	POS	AMB	NEG	NEG	NEG	NEG	POS	AMB	POS	POS	NEG
Uganda-29_17148_250811	POS	NEG	AMB	NEG	POS	NEG	POS	AMB	NEG	NEG	NEG	NEG	POS	NEG	POS	POS	NEG
>"South-East Asian Clad																	
UCIM6015	POS	NEG	AMB	NEG	POS	NEG	POS	AMB	NEG	NEG	NEG	NEG	POS	AMB	POS	POS	NEG
Hong Kong_121	POS	NEG	AMB	NEG	POS	NEG	POS	AMB	NEG	NEG	NEG	NEG	POS	NEG	POS	POS	NEG
>"South-East Asian Clad																	
Z172 (Taiwan)	POS	NEG	AMB	NEG	POS	NEG	POS	AMB	NEG	NEG	NEG	NEG	POS	AMB	POS	POS	NEG
Russia-19_0342_Kurgan	POS	NEG	AMB	NEG	POS	NEG	POS	AMB	NEG	NEG	NEG	NEG	POS	NEG	POS	POS	NEG
>"South-East Asian Clad																	
Perth_01-15392	POS	NEG	AMB	NEG	POS	NEG	POS	AMB	NEG	NEG	NEG	NEG	POS	NEG	POS	POS	NEG
>"South-East Asian Clad																	
XN108 (China)	POS	NEG	AMB	NEG	POS	NEG	POS	AMB	NEG	NEG	NEG	NEG	POS	AMB	POS	POS	NEG
NMR09 (India)	POS	NEG	AMB	NEG	POS	NEG	POS	AMB	NEG	NEG	NEG	NEG	POS	AMB	POS	POS	NEG
346 (Malaysia)	POS	NEG	AMB	NEG	POS	NEG	POS	AMB	NEG	NEG	NEG	NEG	POS	AMB	POS	POS	NEG
DS_009 (Thailand)	POS	NEG	AMB	NEG	POS	NEG	POS	AMB	NEG	NEG	NEG	NEG	POS	AMB	POS	POS	NEG
18ANR581005 (Ind)	POS	NEG	POS	NEG	POS	NEG	POS	AMB	NEG	NEG	NEG	NEG	POS	NEG	POS	POS	NEG
Bengalaru_ST20141405	POS	NEG	POS	NEG	POS	NEG	POS	AMB	NEG	NEG	NEG	NEG	POS	AMB	POS	POS	NEG
Bengalaru_ST20141406	POS	NEG	POS	NEG	POS	NEG	POS	AMB	NEG	NEG	NEG	NEG	POS	AMB	POS	POS	NEG
Bengalaru_ST20141417	POS	NEG	POS	NEG	POS	NEG	POS	AMB	NEG	NEG	NEG	NEG	POS	NEG	POS	POS	NEG
Hong Kong_107	POS	NEG	AMB	NEG	POS	NEG	POS	AMB	NEG	NEG	NEG	NEG	POS	NEG	POS	POS	NEG
Hong Kong_12	POS	NEG	POS	NEG	POS	NEG	POS	AMB	NEG	NEG	NEG	NEG	POS	AMB	POS	POS	NEG
Hong Kong_132	POS	NEG	POS	NEG	POS	NEG	POS	AMB	NEG	NEG	NEG	NEG	POS	AMB	POS	POS	NEG
Riyadh-3028763-R	POS	NEG	AMB	NEG	POS	NEG	POS	AMB	NEG	NEG	NEG	NEG	POS	NEG	POS	POS	NEG
Trinidad&Tobago_MRSA2010_94	POS	NEG	AMB	NEG	POS	NEG	POS	AMB	NEG	NEG	NEG	NEG	POS	AMB	POS	POS	NEG
>"South-East Asian Clad																	
M705 (Thailand)	POS	NEG	AMB	NEG	POS	NEG	POS	AMB	NEG	NEG	NEG	NEG	POS	AMB	POS	POS	NEG
S85 (Thailand)	POS	NEG	AMB	NEG	POS	NEG	POS	AMB	NEG	NEG	NEG	NEG	POS	AMB	POS	POS	NEG
Riyadh_Alfaisal-04_281014_S5076	POS	NEG	POS	NEG	POS	NEG	NEG	POS	NEG	NEG	NEG	NEG	POS	AMB	POS	POS	NEG
Riyadh_Alfaisal-26_S15145_1104163	POS	NEG	POS	NEG	POS	NEG	POS	AMB	NEG	NEG	NEG	NEG	POS	AMB	POS	POS	NEG
Riyadh-2817437-W	POS	NEG	POS	NEG	POS	NEG	POS	AMB	NEG	NEG	NEG	NEG	POS	AMB	POS	POS	NEG
>"South-East Asian Clad																	
PPUKM-775-2009 (Malaysia)	POS	NEG	AMB	NEG	POS	NEG	POS	AMB	NEG	NEG	NEG	NEG	POS	AMB	POS	POS	NEG
Hong Kong_138	POS	NEG	POS	NEG	POS	NEG	POS	AMB	NEG	NEG	NEG	NEG	POS	AMB	POS	POS	NEG
>"South-East Asian Clad																	
Kuwait_001	POS	NEG	POS	NEG	POS	NEG	POS	AMB	NEG	NEG	NEG	NEG	POS	AMB	POS	POS	NEG
Kuwait_337	POS	NEG	POS	NEG	POS	NEG	POS	AMB	NEG	NEG	NEG	NEG	POS	AMB	POS	POS	NEG
>"South-East Asian Clad																	
CUHK_HK2007 (China)	POS	NEG	AMB	NEG	POS	NEG	POS	AMB	NEG	NEG	NEG	NEG	POS	AMB	POS	POS	NEG
NMR02 (India)	POS	NEG	AMB	NEG	POS	NEG	POS	AMB	NEG	NEG	NEG	NEG	POS	AMB	POS	POS	NEG
VB1490 (India)	POS	NEG	AMB	NEG	POS	NEG	POS	AMB	NEG	NEG	NEG	NEG	POS	AMB	POS	POS	NEG
V521 (Korea)	POS	NEG	AMB	NEG	POS	NEG	POS	AMB	NEG	NEG	NEG	NEG	POS	AMB	POS	POS	NEG
HST-077 (Lebanon)	POS	NEG	AMB	NEG	POS	NEG	POS	AMB	NEG	NEG	NEG	NEG	POS	AMB	POS	POS	NEG
MAL1 (Malaysia)	POS	NEG	AMB	NEG	POS	NEG	POS	AMB	NEG	NEG	NEG	NEG	POS	AMB	POS	POS	NEG
MAL3 (Malaysia)	POS	NEG	AMB	NEG	POS	NEG	POS	AMB	NEG	NEG	NEG	NEG	POS	AMB	POS	POS	NEG
PPUKM-261-2009 (Malaysia)	POS	NEG	AMB	NEG	POS	NEG	POS	AMB	NEG	NEG	NEG	NEG	POS	AMB	POS	POS	NEG
PPUKM-332-2009 (Malaysia)	POS	NEG	AMB	NEG	POS	NEG	POS	AMB	NEG	NEG	NEG	NEG	POS	AMB	POS	POS	NEG
S38 (Thailand)	POS	NEG	AMB	NEG	POS	NEG	POS	AMB	NEG	NEG	NEG	NEG	POS	AMB	POS	POS	NEG
S7 (Thailand)	POS	NEG	AMB	NEG	POS	NEG	POS	AMB	NEG	NEG	NEG	NEG	POS	AMB	POS	POS	NEG
US_008 (Thailand)	POS	NEG	AMB	NEG	POS	NEG	POS	AMB	NEG	NEG	NEG	NEG	POS	AMB	POS	POS	NEG
US_030 (Thailand)	POS	NEG	AMB	NEG	POS	NEG	POS	AMB	NEG	NEG	NEG	NEG	POS	AMB	POS	POS	NEG
M170 (Tsunami-related)	POS	NEG	AMB	NEG	POS	NEG	POS	AMB	NEG	NEG	NEG	NEG	POS	AMB	POS	POS	NEG
BSAC697 (UK)	POS	NEG	AMB	NEG	POS	NEG	POS	AMB	NEG	NEG	NEG	NEG	POS	AMB	POS	POS	NEG
IS-189	POS	NEG	AMB	NEG	POS	NEG	POS	AMB	NEG	NEG	NEG	NEG	POS	AMB	POS	POS	NEG
MRGR3	POS	NEG	AMB	NEG	POS	NEG	POS	AMB	NEG	NEG	NEG	NEG	POS	AMB	POS	POS	NEG
smg07	POS	NEG	AMB	NEG	POS	NEG	POS	AMB	NEG	NEG	NEG	NEG	POS	AMB	POS	POS	NEG
AH1-AUS-EMRSA-3	POS	NEG	AMB	NEG	POS	NEG	POS	AMB	NEG	NEG		NEG	POS	NEG	POS	POS	NEG
Bengalaru_ST20141403	POS	NEG	POS	NEG	POS	NEG	POS	AMB	NEG	NEG	NEG	NEG	POS	AMB	POS	POS	NEG
Finland E24_98541_Harmony	POS	NEG	POS	NEG	POS	NEG	POS	AMB	NEG	NEG	NEG	NEG	POS	AMB	POS	POS	NEG
Hong Kong_10	POS	NEG	POS	NEG	POS	NEG	POS	AMB	NEG	NEG	NEG	NEG	POS	AMB	POS	POS	NEG
Hong Kong_100	POS	NEG	AMB	NEG	POS	NEG	POS	AMB	NEG	NEG	NEG	NEG	POS	NEG	POS	POS	NEG
Hong Kong_199	POS	NEG	POS	NEG	POS	NEG	POS	AMB	NEG	NEG	NEG	NEG	POS	AMB	POS	POS	NEG
Hong Kong_34	POS	NEG	POS	NEG	POS	NEG	POS	AMB	NEG	NEG	NEG	NEG	POS	AMB	POS	POS	NEG
Hong Kong_88	POS	NEG	POS	NEG	POS	NEG	POS	AMB	NEG	NEG	NEG	NEG	POS	AMB	POS	POS	NEG
Hong Kong_92	POS	NEG	POS	NEG	POS	NEG	POS	AMB	NEG	NEG	NEG	NEG	POS	AMB	POS	POS	NEG
Hong Kong_94	POS	NEG	POS	NEG	POS	NEG	POS	AMB	NEG	NEG	NEG	NEG	POS	AMB	POS	POS	NEG
Kuwait_257	POS	NEG	POS	NEG	POS	NEG	POS	AMB	NEG	NEG	NEG	NEG	POS	AMB	POS	POS	NEG
Perth_01-15330	POS	NEG	AMB	NEG	POS	NEG	POS	AMB	NEG	NEG	NEG	NEG	POS	NEG	POS	POS	NEG
Perth_01-15349	POS	NEG	NEG	NEG	POS	NEG	POS	AMB	NEG	NEG	NEG	NEG	POS	NEG	POS	POS	NEG
Perth_01-16176	POS	NEG	NEG	NEG	POS	NEG	POS	AMB	NEG	NEG		NEG	POS	NEG	POS	POS	NEG
Perth_WBG8379	POS	NEG	AMB	NEG	POS	NEG	POS	AMB	NEG	NEG		NEG	POS	NEG	POS	POS	NEG
Rawalpindi_Kidney Center_56	POS	NEG	POS	NEG	POS	NEG	POS	AMB	NEG	NEG	NEG	NEG	POS	AMB	POS	POS	NEG
Riyadh_SSTI_05_3464614	POS	NEG	AMB	NEG	POS	NEG	POS	AMB	NEG	NEG	NEG	NEG	POS	NEG	POS	POS	NEG
Trinidad&Tobago_2012_157	POS	NEG	POS	NEG	POS	NEG	NEG	POS	NEG	NEG	NEG	NEG	AMB	POS	POS	POS	NEG

STRAIN / ISOLATE	VIRULENCE : STAPHYLOCOCCAL SUPERANTIGEN/ENTEROTOXIN-LIKE GENES (SET/SSL)																
	ssl01												ssl02		ssl03		
	set6-var1_11	set6-var2_11	set6-var1_12	set6-var2_12	set6-var4_11	ssl01-RF122	ssl01/set6 (COL)	ssl01/set6 (Mu50+N315)	ssl01/set6 (MW2+MSSA476)	ssl01/set6 (MRSA252)	ssl01/set6 (RF122)	ssl01/set6 (other alleles)	ssl02/set7	ssl02/set7 (MRSA252)	ssl03/set8_probe 1	ssl03/set8_probe 2	ssl03/set8 (MRSA252, SAR0424)
	Staphylococcal superantigen-like protein 1												Staphylococcal superantigen-like protein 2		Staphylococcal superantigen-like protein 3		
>"South-East Asian Clad																	
M418	POS	NEG	AMB	NEG	POS	NEG	POS	AMB	NEG	NEG	NEG	NEG	POS	AMB	POS	POS	NEG
>"South-East Asian Clad																	
NMR07 (India)	POS	NEG	AMB	NEG	POS	NEG	POS	AMB	NEG	NEG	NEG	NEG	POS	AMB	POS	POS	NEG
NMR08 (India)	POS	NEG	AMB	NEG	POS	NEG	POS	AMB	NEG	NEG	NEG	NEG	POS	AMB	POS	POS	NEG
Bengalaru_ST20121978	POS	NEG	POS	NEG	POS	NEG	POS	AMB	NEG	NEG	NEG	NEG	POS	AMB	POS	POS	NEG
Hong Kong_89	NEG	NEG	POS	NEG	POS	NEG	NEG	NEG	NEG	NEG	NEG	POS	POS	AMB	POS	POS	NEG
>"South-East Asian Clad																	
Perth_2005_AH_5	POS	POS	AMB	NEG	POS	NEG	POS	AMB	NEG	NEG		NEG	POS	AMB	POS	POS	NEG
>"South-East Asian Clad																	
Trinidad&Tobago_MRSA2010_103	POS	NEG	AMB	NEG	POS	NEG	POS	AMB	NEG	NEG	NEG	NEG	POS	NEG	POS	POS	NEG
Trinidad&Tobago_2013_PA25	POS	NEG	POS	NEG	POS	NEG	POS	AMB	NEG	NEG	NEG	NEG	POS	AMB	POS	POS	NEG
Trinidad&Tobago_MRSA2010_110	POS	NEG	POS	NEG	POS	NEG	POS	AMB	NEG	NEG	NEG	NEG	POS	AMB	POS	POS	NEG
Trinidad&Tobago_SSTI 2012_111	POS	NEG	POS	NEG	POS	NEG	POS	AMB	NEG	NEG	NEG	NEG	POS	NEG	POS	POS	NEG
Trinidad&Tobago_MRSA2010_158	POS	NEG	AMB	NEG	POS	NEG	POS	AMB	NEG	NEG	NEG	NEG	POS	AMB	POS	POS	NEG
>"South-East Asian Clad																	
Kuwait_122	POS	NEG	AMB	NEG	POS	NEG	POS	AMB	NEG	NEG	NEG	NEG	POS	NEG	POS	POS	NEG
Kuwait_002	POS	NEG	AMB	NEG	POS	NEG	POS	AMB	NEG	NEG	NEG	NEG	POS	NEG	POS	POS	NEG
Kuwait_196	POS	NEG	POS	NEG	POS	NEG	POS	AMB	NEG	NEG	NEG	NEG	POS	NEG	POS	POS	NEG
Kuwait_199	POS	NEG	POS	NEG	POS	NEG	AMB	POS	NEG	NEG	NEG	NEG	POS	NEG	POS	POS	NEG
Hong Kong_136	NEG	NEG	NEG	NEG	NEG	NEG	NEG	NEG	NEG	NEG	NEG	NEG	NEG	NEG	NEG	NEG	NEG
>"South-East Asian Clad																	
P32 (Poland)	POS	NEG	AMB	NEG	POS	NEG	POS	AMB	NEG	NEG	NEG	NEG	POS	AMB	POS	POS	NEG
>"South-East Asian Clad																	
Perth_2015-S32313	POS	NEG	AMB	NEG	POS	NEG	POS	AMB	NEG	NEG	NEG	NEG	POS	AMB	POS	POS	NEG
>"hla-negative South-Ea																	
DEN907 (Denmark)	POS	NEG	AMB	NEG	POS	NEG	POS	AMB	NEG	NEG	NEG	NEG	POS	AMB	POS	POS	NEG
>"hla-negative South-Ea																	
S26 (Thailand)	POS	NEG	AMB	NEG	POS	NEG	POS	AMB	NEG	NEG	NEG	NEG	POS	AMB	POS	POS	NEG
S42 (Thailand)	POS	NEG	AMB	NEG	POS	NEG	POS	AMB	NEG	NEG	NEG	NEG	POS	AMB	POS	POS	NEG
S97 (Thailand)	POS	NEG	AMB	NEG	POS	NEG	POS	AMB	NEG	NEG	NEG	NEG	POS	AMB	POS	POS	NEG
S78 (Thailand)	POS	NEG	AMB	NEG	POS	NEG	POS	AMB	NEG	NEG	NEG	NEG	POS	AMB	POS	POS	NEG
S25 (Thailand)	POS	NEG	AMB	NEG	POS	NEG	POS	AMB	NEG	NEG	NEG	NEG	POS	AMB	POS	POS	NEG
S21 (Thailand)	POS	NEG	AMB	NEG	POS	NEG	POS	AMB	NEG	NEG	NEG	NEG	POS	AMB	POS	POS	NEG
S106 (Thailand)	POS	NEG	AMB	NEG	POS	NEG	POS	AMB	NEG	NEG	NEG	NEG	POS	AMB	POS	POS	NEG
S24 (Thailand)	POS	NEG	AMB	NEG	POS	NEG	POS	AMB	NEG	NEG	NEG	NEG	POS	AMB	POS	POS	NEG
S39 (Thailand)	POS	NEG	AMB	NEG	POS	NEG	POS	AMB	NEG	NEG	NEG	NEG	POS	AMB	POS	POS	NEG
S2 (Thailand)	POS	NEG	AMB	NEG	POS	NEG	POS	AMB	NEG	NEG	NEG	NEG	POS	AMB	POS	POS	NEG
S81 (Thailand)	POS	NEG	AMB	NEG	POS	NEG	POS	AMB	NEG	NEG	NEG	NEG	POS	AMB	POS	POS	NEG
M116 (Vietnam)	POS	NEG	AMB	NEG	POS	NEG	POS	AMB	NEG	NEG	NEG	NEG	POS	AMB	POS	POS	NEG
>"Portuguese Clade": Pc																	
FFP103 (Portugal)	POS	NEG	AMB	NEG	POS	NEG	POS	AMB	NEG	NEG	NEG	NEG	POS	AMB	POS	POS	NEG
HDG2 (Portugal)	POS	NEG	AMB	NEG	POS	NEG	POS	AMB	NEG	NEG	NEG	NEG	POS	AMB	POS	POS	NEG
HSA10 (Portugal)	POS	NEG	AMB	NEG	POS	NEG	POS	AMB	NEG	NEG	NEG	NEG	POS	AMB	POS	POS	NEG
HSA11 (Portugal)	POS	NEG	AMB	NEG	POS	NEG	POS	AMB	NEG	NEG	NEG	NEG	POS	AMB	POS	POS	NEG
ICP5011 (Portugal)	POS	NEG	AMB	NEG	POS	NEG	POS	AMB	NEG	NEG	NEG	NEG	POS	AMB	POS	POS	NEG
ICP5014 (Portugal)	POS	NEG	AMB	NEG	POS	NEG	POS	AMB	NEG	NEG	NEG	NEG	POS	AMB	POS	POS	NEG
ICP5062 (Portugal)	POS	NEG	AMB	NEG	POS	NEG	POS	AMB	NEG	NEG	NEG	NEG	POS	AMB	POS	POS	NEG
Strain 1063_ATCC 33592 FDAARGOS_3 (USA)	POS	NEG	AMB	NEG	POS	NEG	POS	AMB	NEG	NEG	NEG	NEG	POS	AMB	POS	POS	NEG
	POS	NEG	POS	NEG	POS	NEG	POS	AMB	NEG	NEG	NEG	NEG	POS	AMB	POS	POS	NEG
Russia-07_0110_Kurgan	POS	NEG	AMB	NEG	POS	NEG	POS	AMB	NEG	NEG	NEG	NEG	POS	NEG	POS	POS	NEG
Russia-11_0164_Kurgan	POS	NEG	POS	NEG	POS	NEG	POS	AMB	NEG	NEG	NEG	NEG	POS	AMB	POS	POS	NEG
Russia-20_0367_Kurgan	POS	NEG	POS	NEG	POS	NEG	POS	AMB	NEG	NEG	NEG	NEG	POS	AMB	POS	POS	NEG
Russia-21_0390_Chelyabinsk	POS	NEG	POS	NEG	POS	NEG	POS	AMB	NEG	NEG	NEG	NEG	POS	AMB	POS	POS	NEG
>"South American/ Mid																	
AGT1 (Argentina)	POS	NEG	AMB	NEG	POS	NEG	POS	AMB	NEG	NEG	NEG	NEG	POS	AMB	POS	POS	NEG
AGT120 (Argentina)	POS	NEG	AMB	NEG	POS	NEG	POS	AMB	NEG	NEG	NEG	NEG	POS	AMB	POS	POS	NEG
AGT67 (Argentina)	POS	NEG	AMB	NEG	POS	NEG	POS	AMB	NEG	NEG	NEG	NEG	POS	AMB	POS	POS	NEG
AGT9 (Argentina)	POS	NEG	AMB	NEG	POS	NEG	POS	AMB	NEG	NEG	NEG	NEG	POS	AMB	POS	POS	NEG
RA3 (Argentina)	POS	NEG	AMB	NEG	POS	NEG	POS	AMB	NEG	NEG	NEG	NEG	POS	AMB	POS	POS	NEG
RA6 (Argentina)	POS	NEG	AMB	NEG	POS	NEG	POS	AMB	NEG	NEG	NEG	NEG	POS	AMB	POS	POS	NEG
RA7 (Argentina)	POS	NEG	AMB	NEG	POS	NEG	POS	AMB	NEG	NEG	NEG	NEG	POS	AMB	POS	POS	NEG
Be62 (Brazil)	POS	NEG	AMB	NEG	POS	NEG	POS	AMB	NEG	NEG	NEG	NEG	POS	AMB	POS	POS	NEG
Bmb9393 (Brazil)	POS	NEG	AMB	NEG	POS	NEG	POS	AMB	NEG	NEG	NEG	NEG	POS	AMB	POS	POS	NEG
BRA36 (Brazil)	POS	NEG	AMB	NEG	POS	NEG	POS	AMB	NEG	NEG	NEG	NEG	POS	AMB	POS	POS	NEG
BZ48 (Brazil)	POS	NEG	AMB	NEG	POS	NEG	POS	AMB	NEG	NEG	NEG	NEG	POS	AMB	POS	POS	NEG
Gv51 (Brazil)	POS	NEG	AMB	NEG	POS	NEG	POS	AMB	NEG	NEG	NEG	NEG	POS	AMB	POS	POS	NEG
Gv69 (Brazil)	POS	NEG	AMB	NEG	POS	NEG	POS	AMB	NEG	NEG	NEG	NEG	POS	AMB	POS	POS	NEG
Gv88 (Brazil)	POS	NEG	AMB	NEG	POS	NEG	POS	AMB	NEG	NEG	NEG	NEG	POS	AMB	POS	POS	NEG
HC1335 (Brazil)	POS	NEG	AMB	NEG	POS	NEG	POS	AMB	NEG	NEG	NEG	NEG	POS	AMB	POS	POS	NEG
HC1340 (Brazil)	POS	NEG	AMB	NEG	POS	NEG	POS	AMB	NEG	NEG	NEG	NEG	POS	AMB	POS	POS	NEG
HC556 (Brazil)	POS	NEG	AMB	NEG	POS	NEG	POS	AMB	NEG	NEG	NEG	NEG	POS	AMB	POS	POS	NEG
HU25 (Brazil)	POS	NEG	AMB	NEG	POS	NEG	POS	AMB	NEG	NEG	NEG	NEG	POS	AMB	POS	POS	NEG
UB563 (Brazil)	POS	NEG	AMB	NEG	POS	NEG	POS	AMB	NEG	NEG	NEG	NEG	POS	AMB	POS	POS	NEG
2A8 (Czech Republic)	POS	NEG	AMB	NEG	POS	NEG	POS	AMB	NEG	NEG	NEG	NEG	POS	AMB	POS	POS	NEG
M1229 (Denmark)	POS	NEG	AMB	NEG	POS	NEG	POS	AMB	NEG	NEG	NEG	NEG	POS	AMB	POS	POS	NEG
FRICAR (France)	POS	NEG	AMB	NEG	POS	NEG	POS	AMB	NEG	NEG	NEG	NEG	POS	AMB	POS	POS	NEG
D90 (Germany)	POS	NEG	AMB	NEG	POS	NEG	POS	AMB	NEG	NEG	NEG	NEG	POS	AMB	POS	POS	NEG
LIT68 (Lithuania)	POS	NEG	AMB	NEG	POS	NEG	POS	AMB	NEG	NEG	NEG	NEG	POS	AMB	POS	POS	NEG
LIT76 (Lithuania)	POS	NEG	AMB	NEG	POS	NEG	POS	AMB	NEG	NEG	NEG	NEG	POS	AMB	POS	POS	NEG
HGSA142 (Portugal)	POS	NEG	AMB	NEG	POS	NEG	POS	AMB	NEG	NEG	NEG	NEG	POS	AMB	POS	POS	NEG
HGSA9 (Portugal)	POS	NEG	AMB	NEG	POS	NEG	POS	AMB	NEG	NEG	NEG	NEG	POS	AMB	POS	POS	NEG
HSJ216 (Portugal)	POS	NEG	AMB	NEG	POS	NEG	POS	AMB	NEG	NEG	NEG	NEG	POS	AMB	POS	POS	NEG
M278 (Portugal)	POS	NEG	AMB	NEG	POS	NEG	POS	AMB	NEG	NEG	NEG	NEG	POS	AMB	POS	POS	NEG
ES26 (Spain)	POS	NEG	AMB	NEG	POS	NEG	POS	AMB	NEG	NEG	NEG	NEG	POS	AMB	POS	POS	NEG
UK102 (UK)	POS	NEG	AMB	NEG	POS	NEG	POS	AMB	NEG	NEG	NEG	NEG	POS	AMB	POS	POS	NEG
URU34 (Uruguay)	POS	NEG	AMB	NEG	POS	NEG	POS	AMB	NEG	NEG	NEG	NEG	POS	AMB	POS	POS	NEG
IS-125	POS	NEG	AMB	NEG	POS	NEG	POS	AMB	NEG	NEG	NEG	NEG	POS	AMB	POS	POS	NEG
IS-157	POS	NEG	AMB	NEG	POS	NEG	POS	AMB	NEG	NEG	NEG	NEG	POS	AMB	POS	POS	NEG
Ecuador_1	POS	NEG	POS	NEG	POS	NEG	POS	AMB	NEG	NEG	NEG	NEG	POS	AMB	POS	POS	NEG
Ecuador_2	POS	NEG	POS	NEG	POS	NEG	POS	AMB	NEG	NEG	NEG	NEG	POS	AMB	POS	POS	NEG
Dresden_15ANR574263	POS	NEG	POS	NEG	POS	NEG	POS	AMB	NEG	NEG	NEG	NEG	POS	AMB	POS	POS	NEG
UK-EMRSA-11	POS	NEG	POS	NEG	POS	NEG	POS	AMB	NEG	NEG		NEG	POS	AMB	POS	POS	NEG
>"South American/ Mid																	
BRA2 (Brazil)	POS	NEG	AMB	NEG	POS	NEG	POS	AMB	NEG	NEG	NEG	NEG	POS	AMB	POS	POS	NEG
CHL1 (Chile)	POS	NEG	AMB	NEG	POS	NEG	POS	AMB	NEG	NEG	NEG	NEG	POS	AMB	POS	POS	NEG
CHL151 (Chile)	POS	NEG	AMB	NEG	POS	NEG	POS	AMB	NEG	NEG	NEG	NEG	POS	AMB	POS	POS	NEG
UCL378 (Chile)	POS	NEG	AMB	NEG	POS	NEG	POS	AMB	NEG	NEG	NEG	NEG	POS	AMB	POS	POS	NEG
UP72 (Peru)	POS	NEG	AMB	NEG	POS	NEG	POS	AMB	NEG	NEG	NEG	NEG	POS	AMB	POS	POS	NEG
UP81 (Peru)	POS	NEG	AMB	NEG	POS	NEG	POS	AMB	NEG	NEG	NEG	NEG	POS	AMB	POS	POS	NEG
>"South American/ Mid																	
UP1073 (Peru)	POS	NEG	AMB	NEG	POS	NEG	POS	AMB	NEG	NEG	NEG	NEG	POS	AMB	POS	POS	NEG

STRAIN / ISOLATE	VIRULENCE : STAPHYLOCOCCAL SUPERANTIGEN/ENTEROTOXIN-LIKE GENES (SET/SSL)																
	ssl01												ssl02		ssl03		
	set6-var1_11	set6-var2_11	set6-var1_12	set6-var2_12	set6-var4_11	ssl01-RF122	ssl01/set6 (COL)	ssl01/set6 (Mu50+N315)	ssl01/set6 (MW2+MSSA476)	ssl01/set6 (MRSA252)	ssl01/set6 (RF122)	ssl01/set6 (other alleles)	ssl02/set7	ssl02/set7 (MRSA252)	ssl03/set8_probe 1	ssl03/set8_probe 2	ssl03/set8 (MRSA252, SAR0424)
	Staphylococcal superantigen-like protein 1												Staphylococcal superantigen-like protein 2		Staphylococcal superantigen-like protein 3		
>"South American/ Mid																	
LIT89 (Lithuania)	POS	NEG	AMB	NEG	POS	NEG	POS	AMB	NEG	NEG	NEG	NEG	POS	AMB	POS	POS	NEG
MRSA_PR1 (Malaysia)	POS	NEG	AMB	NEG	POS	NEG	POS	AMB	NEG	NEG	NEG	NEG	POS	AMB	POS	POS	NEG
Riyadh-2888905-R	POS	NEG	POS	NEG	POS	NEG	POS	AMB	NEG	NEG	NEG	NEG	POS	NEG	POS	POS	NEG
Riyadh-2888915-BC	POS	NEG	AMB	NEG	POS	NEG	POS	AMB	NEG	NEG	NEG	NEG	POS	NEG	POS	POS	NEG
Riyadh-2793706-R	POS	NEG	POS	NEG	POS	NEG	POS	AMB	NEG	NEG	NEG	NEG	POS	AMB	POS	POS	NEG
>"South American/ Mid																	
Riyadh_SSTI_18_3502925	POS	NEG	POS	NEG	POS	NEG	NEG	POS	NEG	NEG	NEG	NEG	POS	AMB	POS	POS	NEG
Riyadh-2822825-W	POS	NEG	POS	NEG	POS	NEG	POS	AMB	NEG	NEG	NEG	NEG	POS	AMB	POS	POS	NEG
>"South American/ Mid																	
UK-EMRSA-9	POS	NEG	POS	NEG	POS	NEG	POS	AMB	NEG	NEG		NEG	POS	AMB	POS	POS	NEG
>"South American/ Mid																	
Lome_HT20020815	POS	NEG	POS	NEG	POS	NEG	POS	AMB	NEG	NEG	NEG	NEG	POS	AMB	POS	POS	NEG
>"South American/ Mid																	
NA32 (Denmark)	POS	NEG	AMB	NEG	POS	NEG	POS	AMB	NEG	NEG	NEG	NEG	POS	AMB	POS	POS	NEG
H24 (Egypt)	POS	NEG	AMB	NEG	POS	NEG	POS	AMB	NEG	NEG	NEG	NEG	POS	AMB	POS	POS	NEG
ATCC BAA-39 (=HUSA304) (Hungary)	POS	NEG	AMB	NEG	POS	NEG	POS	AMB	NEG	NEG	NEG	NEG	POS	AMB	POS	POS	NEG
HUSA304 (Hungary)	POS	NEG	AMB	NEG	POS	NEG	POS	AMB	NEG	NEG	NEG	NEG	POS	AMB	POS	POS	NEG
HU106 (Hungary)	POS	NEG	AMB	NEG	POS	NEG	POS	AMB	NEG	NEG	NEG	NEG	POS	AMB	POS	POS	NEG
BSAC27 (UK)	POS	NEG	AMB	NEG	POS	NEG	POS	AMB	NEG	NEG	NEG	NEG	POS	AMB	POS	POS	NEG
NCTC13131, UK-EMRSA-4 (UK)	POS	NEG	AMB	NEG	POS	NEG	POS	AMB	NEG	NEG	NEG	NEG	POS	AMB	POS	POS	NEG
	POS	NEG	POS	NEG	POS	NEG	POS	AMB	NEG	NEG		NEG	POS	AMB	POS	POS	NEG
Algiers_HT20040080	POS	NEG	NEG	NEG	POS	NEG	POS	AMB	NEG	NEG	NEG	NEG	POS	NEG	POS	POS	NEG
Dublin-DSH_AR09_0.0066	POS	NEG	POS	NEG	POS	NEG	POS	AMB	NEG	NEG	NEG	NEG	POS	NEG	POS	POS	NEG
Dublin-DSH_AR09_0-0065	POS	NEG	NEG	NEG	POS	NEG	POS	AMB	NEG	NEG		NEG	POS	NEG	POS	POS	NEG
Dublin-DSH_Phenotype-III_84	POS	NEG	NEG	NEG	POS	NEG	POS	AMB	NEG	NEG		NEG	POS	NEG	POS	POS	NEG
Hong Kong_130	NEG	NEG	NEG	NEG	NEG	NEG	NEG	NEG	NEG	NEG	NEG	NEG	NEG	NEG	NEG	NEG	NEG
Kuwait_018	POS	NEG	AMB	NEG	POS	NEG	POS	AMB	NEG	NEG	NEG	NEG	POS	NEG	POS	POS	NEG
Perth_08-17726	POS	NEG	POS	NEG	POS	NEG	POS	AMB	NEG	NEG	NEG	NEG	POS	NEG	POS	POS	NEG
Riyadh_Alfaisal/KKKSUH_86_MRS-14-279	POS	NEG	NEG	NEG	POS	NEG	POS	AMB	NEG	NEG	NEG	NEG	POS	NEG	POS	POS	NEG
Riyadh_Alfaisal-04_Z3861_831588	POS	NEG	POS	NEG	POS	NEG	POS	AMB	NEG	NEG	NEG	NEG	POS	AMB	POS	POS	NEG
Riyadh_Alfaisal-30_S15724_1108013	POS	NEG	POS	NEG	POS	NEG	POS	AMB	NEG	NEG	NEG	NEG	POS	AMB	POS	POS	NEG
Riyadh_Alfaisal-6_22A_13_B3992_397721	POS	NEG	POS	NEG	POS	NEG	POS	AMB	NEG	NEG	NEG	NEG	POS	AMB	POS	POS	NEG
Riyadh_SSTI_52_3615482	POS	NEG	POS	NEG	POS	NEG	POS	AMB	NEG	NEG	NEG	NEG	POS	AMB	POS	POS	NEG
Riyadh-2817276-2	POS	NEG	POS	NEG	POS	NEG	POS	AMB	NEG	NEG	NEG	NEG	POS	AMB	POS	POS	NEG
Riyadh-2891670-W	POS	NEG	POS	NEG	POS	NEG	POS	AMB	NEG	NEG	NEG	NEG	POS	AMB	POS	POS	NEG
Riyadh-3006920-W	POS	NEG	POS	NEG	POS	NEG	POS	AMB	NEG	NEG	NEG	NEG	POS	AMB	POS	POS	NEG
Riyadh-R2567782	POS	NEG	POS	NEG	POS	NEG	POS	AMB	NEG	NEG	NEG	NEG	POS	AMB	POS	POS	NEG
Russia-18_0252_Krasnoyarsk_SK2	POS	NEG	POS	NEG	POS	NEG	POS	AMB	NEG	NEG	NEG	NEG	POS	NEG	POS	POS	NEG
UK-EMRSA-7	POS	NEG	AMB	NEG	POS	NEG	POS	AMB	NEG	NEG		NEG	POS	NEG	POS	POS	NEG
>"South American/ Mid																	
Dublin-DSH_AR23_0073	POS	NEG	NEG	NEG	POS	NEG	POS	AMB	NEG	NEG		NEG	POS	NEG	POS	POS	NEG
>Related to "South Ame																	
MRSA-OC3 (Russia)	POS	NEG	AMB	NEG	POS	NEG	POS	AMB	NEG	NEG	NEG	NEG	POS	AMB	POS	POS	NEG
Russia-12_0176_Krasnoyarsk	POS	NEG	POS	NEG	POS	NEG	POS	AMB	NEG	NEG	NEG	NEG	POS	NEG	POS	POS	NEG
Russia-13_0180_Krasnoyarsk	POS	NEG	POS	NEG	POS	NEG	POS	AMB	NEG	NEG	NEG	NEG	POS	AMB	POS	POS	NEG
Russia-16_0249_Krasnoyarsk	POS	NEG	POS	NEG	POS	NEG	POS	AMB	NEG	NEG	NEG	NEG	POS	AMB	POS	POS	NEG
Russia-17_0250_Krasnoyarsk	POS	NEG	AMB	NEG	POS	NEG	POS	AMB	NEG	NEG	NEG	NEG	POS	NEG	POS	POS	NEG
>Related to "South Ame																	
URU110 (Uruguay)	POS	NEG	AMB	NEG	POS	NEG	POS	AMB	NEG	NEG	NEG	NEG	POS	AMB	POS	POS	NEG
>Related to "South Ame																	
DS_014 (Thailand)	POS	NEG	AMB	NEG	POS	NEG	POS	AMB	NEG	NEG	NEG	NEG	POS	AMB	POS	POS	NEG
>Unassigned Middle Eas																	
Frankfurt_Oder_0490031797 (Libya)	POS	NEG	POS	NEG	POS	NEG	POS	AMB	NEG	NEG	NEG	NEG	POS	AMB	POS	POS	NEG
Kuwait_192	POS	NEG	POS	NEG	POS	NEG	NEG	POS	NEG	NEG	NEG	NEG	POS	AMB	POS	POS	NEG
Riyadh-9	POS	NEG	POS	NEG	POS	NEG	POS	AMB	NEG	NEG	NEG	NEG	POS	AMB	POS	POS	NEG
Russia-24_0407_Moscow	POS	NEG	POS	NEG	POS	NEG	POS	AMB	NEG	NEG	NEG	NEG	POS	NEG	POS	POS	NEG

STRAIN / ISOLATE	VIRULENCE : STAPHYLOCOCCAL SUPERANTIGEN/ENTEROTOXIN-LIKE GENES (SET/SSL)															
	ssl04		ssl05				ssl06		ssl07			ssl08		ssl09		
	ssl04/set9	ssl04/set9 (MRSA252, SAR0425)	ssl05/set3_probe 1	ssl05/set3 (RF122, probe-611)	ssl05/set3_probe 2 (612)	ssl05/set3 (MRSA252)	ssl06/set21	ssl06 (NCTC8325+MW2)	ssl07/set1	ssl07/set1 (MRSA252)	ssl07/set1 (AF188836)	ssl08/set12_probe 1	ssl08/set12_probe 2	ssl09/set5_probe 1	ssl09/set5_probe 2	ssl09/set5 (MRSA252)
	Staphylococcal superantigen-like protein 4		Staphylococcal superantigen-like protein 5				Staphylococcal superantigen-like protein 6		Staphylococcal superantigen-like protein 7			Staphylococcal superantigen-like protein 8		Staphylococcal superantigen-like protein 9		
**>"Eurasian Clade": TUR1**																
TUR1 (Turkey)	POS	NEG	POS	NEG	POS	NEG	POS	POS	POS	NEG	NEG	POS	POS	POS	POS	NEG
TUR9 (Turkey)	POS	NEG	POS	NEG	POS	NEG	POS	POS	POS	NEG	NEG	POS	POS	POS	POS	NEG
**>"Eurasian Clade" : T013**																
T0131 (China)	POS	NEG	POS	NEG	POS	NEG	POS	POS	POS	NEG	NEG	POS	POS	POS	POS	NEG
MU4 (Turkey)	POS	NEG	POS	NEG	POS	NEG	POS	POS	POS	NEG	NEG	POS	POS	POS	POS	NEG
DEU11 (Turkey)	POS	NEG	POS	NEG	POS	NEG	POS	POS	POS	NEG	NEG	POS	POS	POS	POS	NEG
DEU16 (Turkey)	POS	NEG	POS	NEG	POS	NEG	POS	POS	POS	NEG	NEG	POS	POS	POS	POS	NEG
Romania_Iasi_MRSA-07_54	POS	NEG	POS	AMB	POS	NEG	POS	POS	AMB	POS	AMB	POS	POS	POS	POS	NEG
**>"Eurasian Clade": arsC-**																
Romania_Iasi_MRSA-05_46	POS	NEG	POS	AMB	POS	NEG	POS	POS	POS	AMB	NEG	POS	POS	POS	POS	NEG
Romania_Iasi_MRSA-06_47	POS	NEG	POS	AMB	POS	NEG	POS	POS	POS	AMB	AMB	POS	POS	POS	POS	NEG
**>"Eurasian Clade": 16K/**																
CN79 (China)	POS	NEG	POS	NEG	POS	NEG	POS	POS	POS	NEG	NEG	POS	POS	POS	POS	NEG
CUHK_BJ2002 (China)	POS	NEG	POS	NEG	POS	NEG	POS	POS	POS	NEG	NEG	POS	POS	POS	POS	NEG
CUHK_BJ2007 (China)	POS	NEG	POS	NEG	POS	NEG	POS	POS	POS	NEG	NEG	POS	POS	POS	POS	NEG
3HK (Czech Republic)	POS	NEG	POS	NEG	POS	NEG	POS	POS	POS	NEG	NEG	POS	POS	POS	POS	NEG
HU109 (Hungary)	POS	NEG	POS	NEG	POS	NEG	POS	POS	POS	NEG	NEG	POS	POS	POS	POS	NEG
HUR18 (Hungary)	POS	NEG	POS	NEG	POS	NEG	POS	POS	POS	NEG	NEG	POS	POS	POS	POS	NEG
H482 (Romania)	POS	NEG	POS	NEG	POS	NEG	POS	POS	POS	NEG	NEG	POS	POS	POS	POS	NEG
16K (Russia)	POS	NEG	POS	NEG	POS	NEG	POS	POS	POS	NEG	NEG	POS	POS	POS	POS	NEG
DEU10 (Turkey)	POS	NEG	POS	NEG	POS	NEG	POS	POS	POS	NEG	NEG	POS	POS	POS	POS	NEG
DEU12 (Turkey)	POS	NEG	POS	NEG	POS	NEG	POS	POS	POS	NEG	NEG	POS	POS	POS	POS	NEG
DEU14 (Turkey)	POS	NEG	POS	NEG	POS	NEG	POS	POS	POS	NEG	NEG	POS	POS	POS	POS	NEG
DEU15 (Turkey)	POS	NEG	POS	NEG	POS	NEG	POS	POS	POS	NEG	NEG	POS	POS	POS	POS	NEG
DEU17 (Turkey)	POS	NEG	POS	NEG	POS	NEG	POS	POS	POS	NEG	NEG	POS	POS	POS	POS	NEG
DEU19 (Turkey)	POS	NEG	POS	NEG	POS	NEG	POS	POS	POS	NEG	NEG	POS	POS	POS	POS	NEG
DEU2 (Turkey)	POS	NEG	POS	NEG	POS	NEG	POS	POS	POS	NEG	NEG	POS	POS	POS	POS	NEG
DEU20 (Turkey)	POS	NEG	POS	NEG	POS	NEG	POS	POS	POS	NEG	NEG	POS	POS	POS	POS	NEG
DEU23 (Turkey)	POS	NEG	POS	NEG	POS	NEG	POS	POS	POS	NEG	NEG	POS	POS	POS	POS	NEG
DEU3 (Turkey)	POS	NEG	POS	NEG	POS	NEG	POS	POS	POS	NEG	NEG	POS	POS	POS	POS	NEG
DEU5 (Turkey)	POS	NEG	POS	NEG	POS	NEG	POS	POS	POS	NEG	NEG	POS	POS	POS	POS	NEG
DEU6 (Turkey)	POS	NEG	POS	NEG	POS	NEG	POS	POS	POS	NEG	NEG	POS	POS	POS	POS	NEG
DEU8 (Turkey)	POS	NEG	POS	NEG	POS	NEG	POS	POS	POS	NEG	NEG	POS	POS	POS	POS	NEG
HU11 (Turkey)	POS	NEG	POS	NEG	POS	NEG	POS	POS	POS	NEG	NEG	POS	POS	POS	POS	NEG
HU13 (Turkey)	POS	NEG	POS	NEG	POS	NEG	POS	POS	POS	NEG	NEG	POS	POS	POS	POS	NEG
HU14 (Turkey)	POS	NEG	POS	NEG	POS	NEG	POS	POS	POS	NEG	NEG	POS	POS	POS	POS	NEG
HU15 (Turkey)	POS	NEG	POS	NEG	POS	NEG	POS	POS	POS	NEG	NEG	POS	POS	POS	POS	NEG
HU16 (Turkey)	POS	NEG	POS	NEG	POS	NEG	POS	POS	POS	NEG	NEG	POS	POS	POS	POS	NEG
HU17 (Turkey)	POS	NEG	POS	NEG	POS	NEG	POS	POS	POS	NEG	NEG	POS	POS	POS	POS	NEG
HU21 (Turkey)	POS	NEG	POS	NEG	POS	NEG	POS	POS	POS	NEG	NEG	POS	POS	POS	POS	NEG
HU23 (Turkey)	POS	NEG	POS	NEG	POS	NEG	POS	POS	POS	NEG	NEG	POS	POS	POS	POS	NEG
HU26 (Turkey)	POS	NEG	POS	NEG	POS	NEG	POS	POS	POS	NEG	NEG	POS	POS	POS	POS	NEG
HU4 (Turkey)	POS	NEG	POS	NEG	POS	NEG	POS	POS	POS	NEG	NEG	POS	POS	POS	POS	NEG
HU5 (Turkey)	POS	NEG	POS	NEG	POS	NEG	POS	POS	POS	NEG	NEG	POS	POS	POS	POS	NEG
HU6 (Turkey)	POS	NEG	POS	NEG	POS	NEG	POS	POS	POS	NEG	NEG	POS	POS	POS	POS	NEG
HU7 (Turkey)	POS	NEG	POS	NEG	POS	NEG	POS	POS	POS	NEG	NEG	POS	POS	POS	POS	NEG
HU8 (Turkey)	POS	NEG	POS	NEG	POS	NEG	POS	POS	POS	NEG	NEG	POS	POS	POS	POS	NEG
HU9 (Turkey)	POS	NEG	POS	NEG	POS	NEG	POS	POS	POS	NEG	NEG	POS	POS	POS	POS	NEG
IU1 (Turkey)	POS	NEG	POS	NEG	POS	NEG	POS	POS	POS	NEG	NEG	POS	POS	POS	POS	NEG
IU10 (Turkey)	POS	NEG	POS	NEG	POS	NEG	POS	POS	POS	NEG	NEG	POS	POS	POS	POS	NEG
IU11 (Turkey)	POS	NEG	POS	NEG	POS	NEG	POS	POS	POS	NEG	NEG	POS	POS	POS	POS	NEG
IU12 (Turkey)	POS	NEG	POS	NEG	POS	NEG	POS	POS	POS	NEG	NEG	POS	POS	POS	POS	NEG
IU13 (Turkey)	POS	NEG	POS	NEG	POS	NEG	POS	POS	POS	NEG	NEG	POS	POS	POS	POS	NEG
IU15 (Turkey)	POS	NEG	POS	NEG	POS	NEG	POS	POS	POS	NEG	NEG	POS	POS	POS	POS	NEG
IU18 (Turkey)	POS	NEG	POS	NEG	POS	NEG	POS	POS	POS	NEG	NEG	POS	POS	POS	POS	NEG
IU19 (Turkey)	POS	NEG	POS	NEG	POS	NEG	POS	POS	POS	NEG	NEG	POS	POS	POS	POS	NEG
IU2 (Turkey)	POS	NEG	POS	NEG	POS	NEG	POS	POS	POS	NEG	NEG	POS	POS	POS	POS	NEG
IU4 (Turkey)	POS	NEG	POS	NEG	POS	NEG	POS	POS	POS	NEG	NEG	POS	POS	POS	POS	NEG
IU6 (Turkey)	POS	NEG	POS	NEG	POS	NEG	POS	POS	POS	NEG	NEG	POS	POS	POS	POS	NEG
IU7 (Turkey)	POS	NEG	POS	NEG	POS	NEG	POS	POS	POS	NEG	NEG	POS	POS	POS	POS	NEG
IU9 (Turkey)	POS	NEG	POS	NEG	POS	NEG	POS	POS	POS	NEG	NEG	POS	POS	POS	POS	NEG
MU1 (Turkey)	POS	NEG	POS	NEG	POS	NEG	POS	POS	POS	NEG	NEG	POS	POS	POS	POS	NEG
MU10 (Turkey)	POS	NEG	POS	NEG	POS	NEG	POS	POS	POS	NEG	NEG	POS	POS	POS	POS	NEG
MU20 (Turkey)	POS	NEG	POS	NEG	POS	NEG	POS	POS	POS	NEG	NEG	POS	POS	POS	POS	NEG
Mu3 (Turkey)	POS	NEG	POS	NEG	POS	NEG	POS	POS	POS	NEG	NEG	POS	POS	POS	POS	NEG
MU5 (Turkey)	POS	NEG	POS	NEG	POS	NEG	POS	POS	POS	NEG	NEG	POS	POS	POS	POS	NEG
MU6 (Turkey)	POS	NEG	POS	NEG	POS	NEG	POS	POS	POS	NEG	NEG	POS	POS	POS	POS	NEG
MU7 (Turkey)	POS	NEG	POS	NEG	POS	NEG	POS	POS	POS	NEG	NEG	POS	POS	POS	POS	NEG
TUR27 (Turkey)	POS	NEG	POS	NEG	POS	NEG	POS	POS	POS	NEG	NEG	POS	POS	POS	POS	NEG
NCTR 32S	POS	NEG	POS	NEG	POS	NEG	POS	POS	POS	NEG	NEG	POS	POS	POS	POS	NEG
SA02_A4 = Russia-06_0085_Moscow	POS	NEG	POS	NEG	POS	NEG	POS	POS	POS	NEG	NEG	POS	POS	POS	POS	NEG
	POS	NEG	POS	AMB	POS	NEG	POS	POS	POS	AMB	AMB	POS	POS	POS	POS	NEG
Dresden_08V35987 (Turkey)	POS	NEG	POS	AMB	POS	NEG	AMB	POS	POS	AMB	AMB	POS	POS	POS	AMB	NEG
Dresden_17ANR577152	POS	NEG	POS	AMB	POS	NEG	POS	POS	POS	POS	NEG	POS	POS	POS	POS	NEG
Dresden_17ANR580374 (Makedonia)	POS	NEG	POS	#ZAHL!	#ZAHL!	NEG	POS	POS	POS	NEG	NEG	POS	POS	POS	POS	NEG
Hong Kong_69-II	POS	NEG	POS	AMB	POS	NEG	POS	POS	AMB	POS	AMB	POS	POS	POS	POS	NEG
Hong Kong_93	POS	NEG	POS	POS	AMB	NEG	POS	POS	AMB	POS	AMB	POS	POS	POS	POS	NEG
Rawalpindi_Kidney Center_03_SK1	POS	NEG	POS	AMB	POS	NEG	POS	POS	POS	AMB	AMB	POS	POS	POS	POS	NEG
Rawalpindi_Kidney Center_08	POS	NEG	POS	AMB	POS	NEG	POS	POS	POS	AMB	NEG	POS	POS	POS	POS	NEG
Rawalpindi_Kidney Center_10	POS	NEG	POS	AMB	POS	NEG	POS	POS	POS	AMB	AMB	POS	POS	POS	POS	NEG
Rawalpindi_Kidney Center_19	POS	NEG	POS	AMB	POS	NEG	POS	POS	POS	AMB	AMB	POS	POS	POS	POS	NEG
Rawalpindi_Kidney Center_50_SK1	POS	NEG	POS	NEG	POS	NEG	POS	POS	POS	AMB	NEG	POS	POS	POS	POS	NEG
Romania_Iasi_BC-49_430	POS	NEG	POS	POS	AMB	NEG	POS	POS	AMB	POS	AMB	POS	POS	POS	POS	NEG
Romania_Iasi_MRSA_284	POS	NEG	POS	AMB	POS	NEG	POS	POS	POS	AMB	NEG	POS	POS	POS	POS	NEG
Romania_Iasi_MRSA-32_318	POS	NEG	POS	AMB	POS	NEG	POS	POS	POS	AMB	NEG	POS	POS	POS	POS	NEG
Romania_Iasi_MRSA-54_430	POS	NEG	POS	AMB	POS	NEG	POS	POS	AMB	POS	AMB	POS	POS	POS	POS	NEG
Romania_Iasi_SSTI-01_2	POS	NEG	POS	AMB	POS	NEG	POS	POS	POS	AMB	AMB	POS	POS	POS	POS	NEG
Romania_Iasi_SSTI-10_101	POS	NEG	POS	AMB	POS	NEG	POS	POS	POS	AMB	NEG	POS	POS	POS	POS	NEG
Romania_Iasi_SSTI-11_106	POS	NEG	POS	AMB	POS	NEG	POS	POS	POS	AMB	NEG	POS	POS	POS	POS	NEG
Russia-01_0001_SaintPetersburg	POS	NEG	POS	AMB	POS	NEG	POS	POS	POS	AMB	AMB	POS	POS	POS	POS	NEG
Russia-02_0004_SaintPetersburg	POS	NEG	POS	AMB	POS	NEG	POS	POS	POS	AMB	AMB	POS	POS	POS	POS	NEG
Russia-03_0057_SaintPetersburg	POS	NEG	POS	NEG	POS	NEG	POS	POS	POS	AMB	NEG	POS	POS	POS	POS	NEG
Russia-04_0076_Moscow	POS	NEG	POS	AMB	POS	NEG	POS	POS	POS	AMB	AMB	POS	POS	POS	POS	NEG
Russia-05_0078_Moscow	POS	NEG	POS	AMB	POS	NEG	POS	POS	POS	AMB	AMB	POS	POS	POS	POS	NEG
Russia-08_0135_Moscow	POS	NEG	POS	AMB	POS	NEG	POS	POS	POS	AMB	AMB	POS	POS	POS	POS	NEG
Russia-09_0150_Kurgan	POS	NEG	POS	AMB	POS	NEG	POS	POS	POS	AMB	AMB	POS	POS	POS	POS	NEG
Russia-10_0162_Kurgan	POS	NEG	POS	AMB	POS	NEG	POS	POS	POS	AMB	AMB	POS	POS	POS	POS	NEG
Russia-14_0184_Moscow	POS	NEG	POS	AMB	POS	NEG	POS	POS	POS	AMB	AMB	POS	POS	POS	POS	NEG
Russia-15_0232_SaintPetersburg	POS	NEG	POS	AMB	POS	NEG	POS	POS	POS	AMB	AMB	POS	POS	POS	POS	NEG
Russia-22_0391_Chelyabinsk	POS	NEG	POS	AMB	POS	NEG	POS	POS	POS	AMB	AMB	POS	POS	POS	POS	NEG
Russia-23_0392_Chelyabinsk	POS	NEG	POS	AMB	POS	NEG	POS	POS	POS	AMB	AMB	POS	POS	POS	POS	NEG
**>"Eurasian Clade" : IU17**																
IU17 (Turkey)	NEG	NEG	POS	NEG	POS	NEG	POS	POS	POS	NEG	NEG	POS	POS	POS	POS	NEG
IU20 (Turkey)	NEG	NEG	POS	NEG	POS	NEG	POS	POS	POS	NEG	NEG	POS	POS	POS	POS	NEG

STRAIN / ISOLATE	VIRULENCE : STAPHYLOCOCCAL SUPERANTIGEN/ENTEROTOXIN-LIKE GENES (SET/SSL)															
	ssl04		ssl05				ssl06		ssl07			ssl08		ssl09		
	ssl04/set9	ssl04/set9 (MRSA252, SAR0425)	ssl05/set3_probe 1	ssl05/set3 (RF122, probe-611)	ssl05/set3_probe 2 (612)	ssl05/set3 (MRSA252)	ssl06/set21	ssl06 (NCTC8325+MW2)	ssl07/set1	ssl07/set1 (MRSA252)	ssl07/set1 (AF188836)	ssl08/set12_probe 1	ssl08/set12_probe 2	ssl09/set5_probe 1	ssl09/set5_probe 2	ssl09/set5 (MRSA252)
	Staphylococcal superantigen-like protein 4		Staphylococcal superantigen-like protein 5				Staphylococcal superantigen-like protein 6		Staphylococcal superantigen-like protein 7			Staphylococcal superantigen-like protein 8		Staphylococcal superantigen-like protein 9		
**>"Eurasian Clade": Spora**																
Dresden_16ANR581769	POS	NEG	POS	NEG	POS	NEG	POS	POS	POS	NEG	NEG	POS	POS	POS	POS	NEG
Dresden_17ANR578769	POS	NEG	POS	AMB	POS	NEG	POS	POS	POS	AMB	NEG	POS	POS	POS	POS	NEG
**>"Eurasian Clade": Kuwa**																
Kuwait_306	POS	AMB	POS	POS	AMB	NEG	POS	POS	AMB	POS	AMB	POS	POS	POS	POS	NEG
Kuwait_301	POS	NEG	POS	POS	AMB	NEG	POS	POS	AMB	POS	AMB	POS	POS	POS	POS	NEG
**>"European Clade": "Gre**																
GRE18 (Greece)	POS	NEG	POS	NEG	POS	NEG	POS	POS	POS	NEG	NEG	POS	POS	POS	POS	NEG
GRE317 (Greece)	POS	NEG	POS	NEG	POS	NEG	POS	POS	POS	NEG	NEG	POS	POS	POS	POS	NEG
GRE4 (Greece)	POS	NEG	POS	NEG	POS	NEG	POS	POS	POS	NEG	NEG	POS	POS	POS	POS	NEG
R35 (USA)	POS	NEG	POS	NEG	POS	NEG	POS	POS	POS	NEG	NEG	POS	POS	POS	POS	NEG
Casablanca_HT20060548	POS	NEG	POS	AMB	POS	NEG	POS	POS	POS	AMB	AMB	POS	POS	POS	POS	NEG
Casablanca_HT20060550	POS	NEG	POS	AMB	POS	NEG	POS	POS	POS	AMB	AMB	POS	POS	POS	POS	NEG
Dresden_01V34572 (Greece)	POS	NEG	POS	NEG	POS	NEG	NEG	POS	POS	AMB	NEG	POS	POS	POS	NEG	NEG
Dresden_01V36469	POS	NEG	POS	AMB	POS	NEG	POS	POS	POS	NEG	NEG	POS	POS	POS	POS	NEG
Dresden_01V39123	POS	NEG	POS	POS	AMB	NEG	POS	POS	AMB	POS	AMB	POS	POS	POS	POS	NEG
Dresden_01V39143	POS	NEG	POS	NEG	POS	NEG	POS	POS	POS	AMB	NEG	POS	POS	POS	POS	NEG
Greece 1_3680_Harmony	POS	NEG	POS	NEG	POS	NEG	NEG	NEG	POS	NEG	NEG	POS	POS	POS	POS	NEG
**>"European Clade": UK-(**																
ANS46 (Australia)	POS	NEG	POS	NEG	POS	NEG	POS	POS	POS	NEG	NEG	POS	POS	POS	POS	NEG
LHH1 (USA)	POS	NEG	POS	NEG	POS	NEG	POS	POS	POS	NEG	NEG	POS	POS	POS	POS	NEG
Dublin-DSH_AR01_0-1085	POS	NEG	POS	AMB	POS	NEG	POS	POS	POS	AMB	NEG	POS	POS	POS	POS	NEG
Dublin-DSH_AR01_0-1118	POS	NEG	POS	AMB	POS	NEG	POS	POS	POS	POS	NEG	POS	POS	POS	POS	NEG
Dublin-DSH_AR15_0098	POS	NEG		AMB	POS	NEG	AMB	POS	POS	AMB	AMB	POS	POS	POS	POS	NEG
Dublin-DSH_AR15_0104	POS	NEG	POS	AMB	POS	NEG	POS	POS	POS	AMB	AMB	POS	POS	POS	POS	NEG
UK_NCTC11939_UK-1_Harmony	POS	NEG	POS	AMB	POS	NEG	POS	POS	POS	AMB	NEG	POS	POS	POS	POS	NEG
**>"European Clade": BK2**																
BK2421 (USA)	POS	NEG	POS	NEG	POS	NEG	POS	POS	POS	NEG	NEG	POS	POS	POS	POS	NEG
**>"Aussi/NZ Clade", JKD6**																
JKD6009 (Australia)	POS	NEG	POS	NEG	POS	NEG	POS	NEG	POS	NEG	NEG	POS	POS	POS	POS	NEG
JKD6008 (New Zealand)	POS	NEG	POS	NEG	POS	NEG	POS	POS	POS	NEG	NEG	POS	POS	POS	POS	NEG
Perth_2015-S20209	POS	NEG	POS	NEG	POS	NEG	POS	POS	POS	NEG	NEG	POS	POS	POS	POS	NEG
Perth_2015-S20305	POS	NEG	POS	NEG	POS	NEG	POS	POS	POS	NEG	NEG	POS	POS	POS	POS	NEG
Perth_2015-S20603	POS	NEG	POS	NEG	POS	NEG	POS	POS	POS	NEG	NEG	POS	POS	POS	POS	NEG
Perth_2015-S20607	POS	NEG	POS	NEG	POS	NEG	POS	POS	POS	NEG	NEG	POS	POS	POS	POS	NEG
Perth_2015-S20608	POS	NEG	POS	NEG	POS	NEG	POS	POS	POS	NEG	NEG	POS	POS	POS	POS	NEG
Perth_2015-S20610	POS	NEG	POS	NEG	POS	NEG	POS	POS	POS	NEG	NEG	POS	POS	POS	POS	NEG
Perth_2015-S20613	POS	NEG	POS	NEG	POS	NEG	POS	POS	POS	NEG	NEG	POS	POS	POS	POS	NEG
Perth_2015-S20617	POS	NEG	POS	NEG	POS	NEG	POS	POS	POS	NEG	NEG	POS	POS	POS	POS	NEG
Perth_2015-S20622	POS	NEG	POS	NEG	POS	NEG	POS	POS	POS	NEG	NEG	POS	POS	POS	POS	NEG
Perth_2015-S23619	POS	NEG	POS	NEG	POS	NEG	POS	POS	POS	NEG	NEG	POS	POS	POS	POS	NEG
Perth_2015-S41210	POS	NEG	POS	NEG	POS	NEG	POS	POS	POS	NEG	NEG	POS	POS	POS	POS	NEG
Perth_2015-S51001	POS	NEG	POS	NEG	POS	NEG	POS	POS	POS	NEG	NEG	POS	POS	POS	POS	NEG
Perth_2015-S51003	POS	NEG	POS	NEG	POS	NEG	POS	POS	POS	NEG	NEG	POS	POS	POS	POS	NEG
Perth_2015-S51006	POS	NEG	POS	NEG	POS	NEG	POS	POS	POS	NEG	NEG	POS	POS	POS	POS	NEG
Perth_2015-S51007	POS	NEG	POS	NEG	POS	NEG	POS	POS	POS	NEG	NEG	POS	POS	POS	POS	NEG
Perth_2015-S51008	POS	NEG	POS	NEG	POS	NEG	POS	POS	POS	NEG	NEG	POS	POS	POS	POS	NEG
Perth_2015-S51014	POS	NEG	POS	NEG	POS	NEG	POS	POS	POS	NEG	NEG	POS	POS	POS	POS	NEG
Perth_2015-S51408	POS	NEG	POS	NEG	POS	NEG	POS	POS	POS	NEG	NEG	POS	POS	POS	POS	NEG
Perth_2016-S20614	POS	NEG	POS	NEG	POS	NEG	POS	POS	POS	NEG	NEG	POS	POS	POS	POS	NEG
Perth_2016-S23614	POS	NEG	POS	NEG	POS	NEG	POS	POS	POS	NEG	NEG	POS	POS	POS	POS	NEG
Perth_2016-S42009	POS	NEG	POS	NEG	POS	NEG	POS	POS	POS	NEG	NEG	POS	POS	POS	POS	NEG
Perth_2016-S51001	POS	NEG	POS	NEG	POS	NEG	POS	POS	POS	NEG	NEG	POS	POS	POS	POS	NEG
Perth_2016-S51403	POS	NEG	POS	NEG	POS	NEG	POS	POS	POS	NEG	NEG	POS	POS	POS	POS	NEG
Perth_07 RPA 20	POS	NEG	POS	AMB	POS	NEG	POS	POS	POS	AMB	NEG	POS	POS	POS	POS	NEG
Perth_08-19088	POS	NEG	POS	AMB	POS	NEG	POS	POS	AMB	POS	AMB	POS	POS	POS	POS	NEG
Perth_01-15351	POS	NEG	POS	NEG	POS	NEG	POS	POS	POS	AMB	NEG	POS	POS	POS	POS	NEG
Perth_01-15357	POS	NEG	POS	NEG	POS	NEG	POS	POS	POS	AMB	NEG	POS	POS	POS	POS	NEG
Perth_01-15419	POS	NEG	POS	AMB	POS	NEG	POS	POS	POS	AMB	NEG	POS	POS	POS	POS	NEG
Perth_01-16309	POS	NEG	POS	AMB	POS	NEG	POS	POS	POS	AMB	NEG	POS	POS	POS	POS	NEG
Perth_03 SNP 95	POS	NEG	POS	AMB	POS	NEG	POS	POS	AMB	POS	AMB	POS	POS	POS	POS	NEG
Perth_2003_AH_47	POS	NEG	POS	AMB	POS	NEG	POS	POS	AMB	POS	AMB	POS	POS	POS	POS	NEG
**>"Australian/NZ Clade":**																
Perth_2015-S20623	POS	NEG	POS	NEG	POS	NEG	POS	POS	POS	NEG	NEG	POS	POS	POS	POS	NEG
Perth_2015-S20826	POS	NEG	POS	NEG	POS	NEG	POS	POS	POS	NEG	NEG	POS	POS	POS	POS	NEG
Perth_05 NH 56	POS	NEG	POS	POS	POS	NEG	POS	POS	POS	POS	NEG	POS	POS	POS	POS	NEG
**>CC5-MRSA-III LA MRSA**																
Delbrück 38 isolate from domestic turkey	POS	AMB	POS	AMB	POS	NEG	NEG	NEG	AMB	POS	AMB	POS	POS	POS	POS	NEG
**> Staph. pseudintermed**																
KM1381	NEG	NEG	NEG	NEG	NEG	NEG	NEG	NEG	NEG	NEG	NEG	NEG	NEG	NEG	NEG	NEG
**>"South-East Asian Clad**																
M92 (Canada)	POS	NEG	POS	NEG	POS	NEG	POS	POS	POS	NEG	NEG	POS	POS	POS	POS	NEG
CHI59 (China)	NEG	NEG	NEG	NEG	NEG	NEG	NEG	NEG	POS	NEG	NEG	POS	POS	POS	POS	NEG
CHI61 (China)	NEG	NEG	NEG	NEG	NEG	NEG	NEG	NEG	POS	NEG	NEG	POS	POS	POS	POS	NEG
CUHK_HK1997 (China)	POS	NEG	POS	NEG	POS	NEG	POS	POS	POS	NEG	NEG	POS	POS	POS	POS	NEG
M996 (China)	POS	NEG	POS	NEG	POS	NEG	POS	POS	POS	NEG	NEG	POS	POS	POS	POS	NEG
H211 (Denmark)	POS	NEG	POS	NEG	POS	NEG	POS	POS	POS	NEG	NEG	POS	POS	NEG	NEG	NEG
H216 (Denmark)	POS	NEG	POS	NEG	POS	NEG	POS	POS	POS	NEG	NEG	POS	POS	NEG	NEG	NEG
D71 (Germany)	POS	NEG	POS	NEG	POS	NEG	POS	POS	POS	NEG	NEG	POS	POS	POS	POS	NEG
NMR05 (India)	POS	NEG	POS	NEG	POS	NEG	POS	POS	POS	NEG	NEG	POS	POS	POS	POS	NEG
MAL11 (Malaysia)	POS	NEG	POS	NEG	POS	NEG	POS	POS	POS	NEG	NEG	POS	POS	POS	POS	NEG
MAL9 (Malaysia)	POS	NEG	POS	NEG	POS	NEG	POS	POS	POS	NEG	NEG	POS	POS	POS	POS	NEG
Na21 (Sri Lanka)	POS	NEG	POS	NEG	POS	NEG	POS	POS	POS	NEG	NEG	POS	POS	POS	POS	NEG
M592 (Syria)	POS	NEG	POS	NEG	POS	NEG	POS	POS	POS	NEG	NEG	POS	POS	POS	POS	NEG
H202 (Thailand)	POS	NEG	POS	NEG	POS	NEG	POS	POS	POS	NEG	NEG	POS	POS	NEG	NEG	NEG
S102 (Thailand)	POS	NEG	POS	NEG	POS	NEG	POS	POS	POS	NEG	NEG	POS	POS	NEG	NEG	NEG
S130 (Thailand)	POS	NEG	POS	NEG	POS	NEG	POS	POS	POS	NEG	NEG	POS	POS	NEG	NEG	NEG
S40 (Thailand)	POS	NEG	POS	NEG	POS	NEG	POS	POS	POS	NEG	NEG	POS	POS	NEG	NEG	NEG
S71 (Thailand)	POS	NEG	POS	NEG	POS	NEG	POS	POS	POS	NEG	NEG	POS	POS	NEG	NEG	NEG
S87 (Thailand)	POS	NEG	POS	NEG	POS	NEG	POS	POS	POS	NEG	NEG	POS	POS	NEG	NEG	NEG
S93 (Thailand)	POS	NEG	POS	NEG	POS	NEG	POS	POS	POS	NEG	NEG	POS	POS	NEG	NEG	NEG
US_002 (Thailand)	POS	NEG	POS	NEG	POS	NEG	POS	POS	POS	NEG	NEG	POS	POS	NEG	NEG	NEG
US_017 (Thailand)	POS	NEG	POS	NEG	POS	NEG	POS	POS	POS	NEG	NEG	POS	POS	POS	POS	NEG
WAMC6102 (Thailand)	POS	NEG	POS	NEG	POS	NEG	POS	POS	POS	NEG	NEG	POS	POS	POS	POS	NEG
NCTC_13626_TW20 (UK)	POS	NEG	POS	NEG	POS	NEG	POS	POS	POS	NEG	NEG	POS	POS	POS	POS	NEG
BSAC3021 (UK)	POS	NEG	POS	NEG	POS	NEG	POS	POS	POS	NEG	NEG	POS	POS	POS	POS	NEG
BSAC3046 (UK)	POS	NEG	POS	NEG	POS	NEG	POS	POS	POS	NEG	NEG	POS	POS	POS	POS	NEG
1193_SAUR (USA)	POS	NEG	POS	NEG	POS	NEG	POS	POS	POS	NEG	NEG	POS	POS	POS	POS	NEG
1194_SAUR (USA)	POS	NEG	POS	NEG	POS	NEG	POS	POS	POS	NEG	NEG	POS	POS	POS	POS	NEG

STRAIN / ISOLATE	VIRULENCE : STAPHYLOCOCCAL SUPERANTIGEN/ENTEROTOXIN-LIKE GENES (SET/SSL)															
	ssl04		ssl05				ssl06		ssl07			ssl08		ssl09		
	ssl04/set9	ssl04/set9 (MRSA252, SAR0425)	ssl05/set3_probe 1	ssl05/set3 (RF122, probe-611)	ssl05/set3_probe 2 (612)	ssl05/set3 (MRSA252)	ssl06/set21	ssl06 (NCTC8325+MW2)	ssl07/set1	ssl07/set1 (MRSA252)	ssl07/set1 (AF188836)	ssl08/set12_probe 1	ssl08/set12_probe 2	ssl09/set5_probe 1	ssl09/set5_probe 2	ssl09/set5 (MRSA252)
	Staphylococcal superantigen-like protein 4		Staphylococcal superantigen-like protein 5				Staphylococcal superantigen-like protein 6		Staphylococcal superantigen-like protein 7			Staphylococcal superantigen-like protein 8		Staphylococcal superantigen-like protein 9		
>"South-East Asian Clade": TW20 (Irish AR44), c																
1195_SAUR (USA)	POS	NEG	POS	NEG	POS	NEG	POS	POS	POS	NEG	NEG	POS	POS	POS	POS	NEG
FVRH6002 (USA)	POS	NEG	POS	NEG	POS	NEG	POS	POS	POS	NEG	NEG	POS	POS	POS	POS	NEG
KINW6048 (USA)	POS	NEG	POS	NEG	POS	NEG	POS	POS	POS	NEG	NEG	POS	POS	POS	POS	NEG
LAMC0011 (USA)	POS	NEG	POS	NEG	POS	NEG	POS	POS	POS	NEG	NEG	POS	POS	POS	POS	NEG
SJOS6053 (USA)	POS	NEG	POS	NEG	POS	NEG	POS	POS	POS	NEG	NEG	POS	POS	POS	POS	NEG
SJOS6072 (USA)	POS	NEG	POS	NEG	POS	NEG	POS	POS	POS	NEG	NEG	POS	POS	POS	POS	NEG
UCIM6042 (USA)	POS	NEG	POS	NEG	POS	NEG	POS	POS	POS	NEG	NEG	POS	POS	POS	POS	NEG
Perth_2015-S31902	POS	NEG	POS	NEG	POS	NEG	POS	POS	NEG	NEG	NEG	NEG	NEG	POS	POS	NEG
Perth_2015-S31904	POS	NEG	POS	NEG	POS	NEG	POS	POS	POS	NEG	NEG	POS	POS	POS	POS	NEG
Perth_2015-S31913	POS	NEG	POS	NEG	POS	NEG	POS	POS	POS	NEG	NEG	POS	POS	POS	POS	NEG
Perth_2015-S31918	POS	NEG	POS	NEG	POS	NEG	POS	POS	POS	NEG	NEG	POS	POS	POS	POS	NEG
Dublin-DSH_AR44_E1183	POS	NEG	POS	AMB	POS	NEG	POS	POS	POS	AMB	NEG	POS	POS	POS	POS	NEG
Dublin-DSH_Unfamiliar-3_E1520	POS	NEG	POS	AMB	POS	NEG	POS	POS	POS	AMB	NEG	POS	POS	POS	POS	NEG
Hong Kong_042	POS	NEG	POS	AMB	POS	NEG	POS	POS	POS	AMB	NEG	POS	POS	POS	POS	NEG
Hong Kong_101	POS	NEG	POS	AMB	POS	NEG	POS	POS	POS	AMB	AMB	POS	POS	POS	POS	NEG
Hong Kong_111	POS	NEG	POS	AMB	POS	NEG	POS	POS	AMB	POS	AMB	POS	POS	POS	POS	NEG
Hong Kong_113	POS	NEG	POS	AMB	POS	NEG	POS	POS	POS	AMB	NEG	POS	POS	POS	POS	NEG
Hong Kong_115	POS	NEG	POS	POS	POS	NEG	POS	POS	POS	POS	NEG	POS	POS	POS	POS	NEG
Hong Kong_118	POS	NEG	POS	AMB	POS	NEG	POS	POS	AMB	POS	AMB	POS	POS	POS	POS	NEG
Hong Kong_125	POS	NEG	POS	POS	AMB	NEG	POS	POS	AMB	POS	AMB	POS	POS	POS	POS	NEG
Hong Kong_129	POS	NEG	POS	AMB	POS	NEG	POS	POS	POS	AMB	NEG	POS	POS	POS	POS	NEG
Hong Kong_133	POS	NEG	POS	AMB	POS	NEG	POS	POS	POS	AMB	AMB	POS	POS	POS	POS	NEG
Kuwait_009	POS	NEG	POS	AMB	POS	NEG	POS	POS	POS	AMB	AMB	POS	POS	POS	POS	NEG
Kuwait_103	POS	NEG	POS	POS	AMB	NEG	POS	POS	AMB	POS	AMB	POS	POS	POS	POS	NEG
Kuwait_107	POS	AMB	POS	AMB	POS	NEG	POS	POS	AMB	POS	AMB	NEG	NEG	POS	POS	NEG
Perth_03 AH 88	POS	NEG	POS	AMB	POS	NEG	POS	POS	POS	AMB	NEG	POS	POS	POS	POS	NEG
Perth_08-16905	POS	NEG	POS	AMB	POS	NEG	POS	POS	POS	AMB	NEG	POS	POS	POS	POS	NEG
Riyadh_Alfaisal-10_SICU_13_B5672_1117353	POS	NEG	POS	AMB	POS	NEG	POS	POS	POS	AMB	AMB	POS	POS	POS	POS	NEG
Riyadh_SSTI_17_3508602	POS	NEG	POS	AMB	POS	NEG	POS	POS	POS	AMB	AMB	POS	POS	POS	POS	NEG
Riyadh-2822088-R	POS	NEG	POS	POS	POS	NEG	POS	POS	POS	POS	AMB	POS	POS	POS	POS	NEG
Trinidad&Tobago_2012_195 (619/12)	POS	NEG	POS	POS	AMB	NEG	POS	POS	AMB	POS	AMB	POS	POS	POS	POS	NEG
Trinidad&Tobago_2012_370 (1112-52177)	POS	AMB	POS	POS	AMB	NEG	POS	POS	AMB	POS	AMB	POS	POS	POS	POS	NEG
Trinidad&Tobago_2012_378 (4139)	POS	NEG	POS	POS	AMB	NEG	POS	POS	AMB	POS	AMB	POS	POS	POS	POS	NEG
Trinidad&Tobago_2013_PA21	POS	NEG	POS	AMB	POS	NEG	POS	POS	POS	AMB	NEG	POS	POS	POS	POS	NEG
Trinidad&Tobago_2013_PA22	POS	AMB	POS	AMB	POS	NEG	POS	POS	AMB	POS	AMB	POS	POS	POS	POS	NEG
Trinidad&Tobago_2912_340 (8523/12)	POS	NEG	POS	POS	AMB	NEG	POS	POS	AMB	POS	AMB	POS	POS	POS	POS	NEG
Trinidad&Tobago_MRSA2010_105	POS	NEG	POS	POS	NEG	NEG	POS	POS	POS	AMB	AMB	POS	POS	POS	POS	NEG
Trinidad&Tobago_MRSA2010_108	POS	AMB	POS	POS	NEG	NEG	POS	POS	AMB	POS	AMB	POS	POS	POS	POS	NEG
Trinidad&Tobago_MRSA2010_33	POS	NEG	POS	AMB	POS	NEG	POS	POS	POS	AMB	AMB	POS	POS	POS	POS	NEG
Trinidad&Tobago_MRSA2010_79	POS	NEG	POS	POS	NEG	NEG	POS	POS	POS	AMB	NEG	POS	POS	POS	POS	NEG
Trinidad&Tobago_MRSA2010_88	POS	NEG	POS	POS	NEG	NEG	POS	POS	POS	AMB	AMB	POS	POS	POS	POS	NEG
Trinidad&Tobago_MRSA2010_97	POS	NEG	POS	POS	NEG	NEG	POS	POS	POS	AMB	AMB	POS	POS	POS	POS	NEG
Trinidad&Tobago_SSTI 2012_031	POS	NEG	POS	AMB	POS	NEG	POS	POS	POS	AMB	AMB	POS	POS	POS	POS	NEG
Uganda-29_17148_250811	POS	NEG	POS	AMB	POS	NEG	POS	POS	POS	AMB	NEG	POS	POS	POS	POS	NEG
>"South-East Asian Clad																
UCIM6015	POS	NEG	POS	NEG	POS	NEG	POS	POS	POS	NEG	NEG	POS	POS	POS	POS	NEG
Hong Kong_121	POS	NEG	POS	AMB	POS	NEG	POS	POS	POS	AMB	NEG	POS	POS	POS	POS	NEG
>"South-East Asian Clad																
Z172 (Taiwan)	POS	NEG	POS	NEG	POS	NEG	POS	POS	POS	NEG	NEG	POS	POS	POS	POS	NEG
Russia-19_0342_Kurgan	POS	NEG	POS	AMB	POS	NEG	POS	POS	POS	AMB	NEG	POS	POS	POS	POS	NEG
>"South-East Asian Clad																
Perth_01-15392	POS	NEG	POS	AMB	POS	NEG	POS	POS	POS	AMB	NEG	POS	POS	POS	POS	NEG
>"South-East Asian Clad																
XN108 (China)	POS	NEG	POS	NEG	POS	NEG	POS	POS	POS	NEG	NEG	POS	POS	POS	POS	NEG
NMR09 (India)	POS	NEG	POS	NEG	POS	NEG	POS	POS	POS	NEG	NEG	POS	POS	POS	POS	NEG
346 (Malaysia)	POS	NEG	POS	NEG	POS	NEG	POS	POS	POS	NEG	NEG	POS	POS	POS	POS	NEG
DS_009 (Thailand)	POS	NEG	POS	NEG	POS	NEG	POS	POS	POS	NEG	NEG	POS	POS	NEG	NEG	NEG
18ANR581005 (Ind)	POS	NEG	POS	AMB	POS	NEG	POS	POS	POS	AMB	NEG	POS	POS	POS	POS	NEG
Bengalaru_ST20141405	POS	AMB	POS	AMB	POS	NEG	POS	POS	POS	AMB	AMB	POS	POS	POS	POS	NEG
Bengalaru_ST20141406	POS	AMB	POS	AMB	POS	NEG	POS	POS	POS	AMB	AMB	POS	POS	POS	POS	NEG
Bengalaru_ST20141417	POS	NEG	POS	AMB	POS	NEG	POS	POS	POS	AMB	AMB	POS	POS	POS	POS	NEG
Hong Kong_107	POS	NEG	POS	AMB	POS	NEG	POS	POS	POS	AMB	AMB	POS	POS	POS	POS	NEG
Hong Kong_12	POS	NEG	POS	AMB	POS	NEG	POS	POS	POS	AMB	AMB	POS	POS	POS	POS	NEG
Hong Kong_132	POS	NEG	POS	AMB	POS	NEG	POS	POS	POS	AMB	AMB	POS	POS	POS	POS	NEG
Riyadh-3028763-R	POS	NEG	POS	NEG	POS	NEG	POS	POS	POS	AMB	NEG	POS	POS	POS	POS	NEG
Trinidad&Tobago_MRSA2010_94	POS	NEG	POS	POS	NEG	NEG	POS	POS	POS	AMB	AMB	POS	POS	POS	POS	NEG
>"South-East Asian Clad																
M705 (Thailand)	POS	NEG	POS	NEG	POS	NEG	POS	POS	POS	NEG	NEG	POS	POS	NEG	NEG	NEG
S85 (Thailand)	POS	NEG	POS	NEG	POS	NEG	POS	POS	POS	NEG	NEG	POS	POS	NEG	NEG	NEG
Riyadh_Alfaisal-04_281014_S5076	POS	AMB	POS	POS	AMB	NEG	POS	POS	AMB	POS	AMB	POS	POS	POS	POS	NEG
Riyadh_Alfaisal-26_S15145_1104163	POS	NEG	POS	AMB	POS	NEG	POS	POS	POS	AMB	AMB	POS	POS	POS	POS	NEG
Riyadh-2817437-W	POS	NEG	POS	POS	AMB	NEG	POS	POS	AMB	POS	AMB	POS	POS	POS	POS	NEG
>"South-East Asian Clad																
PPUKM-775-2009 (Malaysia)	POS	NEG	POS	NEG	POS	NEG	POS	POS	POS	NEG	NEG	POS	POS	POS	POS	NEG
Hong Kong_138	POS	NEG	POS	AMB	POS	NEG	POS	POS	POS	AMB	AMB	POS	POS	POS	POS	NEG
>"South-East Asian Clad																
Kuwait_001	POS	NEG	POS	AMB	POS	NEG	POS	POS	AMB	POS	AMB	POS	POS	POS	POS	NEG
Kuwait_337	POS	AMB	POS	AMB	POS	NEG	POS	POS	AMB	POS	AMB	POS	POS	POS	POS	NEG
>"South-East Asian Clad																
CUHK_HK2007 (China)	POS	NEG	POS	NEG	POS	NEG	POS	POS	POS	NEG	NEG	POS	POS	POS	POS	NEG
NMR02 (India)	POS	NEG	POS	NEG	POS	NEG	POS	POS	POS	NEG	NEG	POS	POS	POS	POS	NEG
VB1490 (India)	POS	NEG	POS	NEG	POS	NEG	POS	POS	POS	NEG	NEG	POS	POS	POS	POS	NEG
V521 (Korea)	POS	NEG	POS	NEG	POS	NEG	POS	POS	POS	NEG	NEG	POS	POS	POS	POS	NEG
HST-077 (Lebanon)	POS	NEG	POS	NEG	POS	NEG	POS	POS	POS	NEG	NEG	POS	POS	POS	POS	NEG
MAL1 (Malaysia)	POS	NEG	POS	NEG	POS	NEG	POS	POS	POS	NEG	NEG	POS	POS	POS	POS	NEG
MAL3 (Malaysia)	POS	NEG	POS	NEG	POS	NEG	POS	POS	POS	NEG	NEG	POS	POS	POS	POS	NEG
PPUKM-261-2009 (Malaysia)	POS	NEG	POS	NEG	POS	NEG	POS	POS	POS	NEG	NEG	POS	POS	POS	POS	NEG
PPUKM-332-2009 (Malaysia)	POS	NEG	POS	NEG	POS	NEG	POS	POS	POS	NEG	NEG	POS	POS	POS	POS	NEG
S38 (Thailand)	POS	NEG	POS	NEG	POS	NEG	POS	POS	POS	NEG	NEG	POS	POS	POS	POS	NEG
S7 (Thailand)	POS	NEG	POS	NEG	POS	NEG	POS	POS	POS	NEG	NEG	POS	POS	POS	POS	NEG
US_008 (Thailand)	POS	NEG	POS	NEG	POS	NEG	POS	POS	POS	NEG	NEG	POS	POS	POS	POS	NEG
US_030 (Thailand)	POS	NEG	POS	NEG	POS	NEG	POS	POS	POS	NEG	NEG	POS	POS	NEG	NEG	NEG
M170 (Tsunami-related)	POS	NEG	POS	NEG	POS	NEG	POS	POS	POS	NEG	NEG	POS	POS	NEG	NEG	NEG
BSAC697 (UK)	POS	NEG	POS	NEG	POS	NEG	POS	POS	POS	NEG	NEG	POS	POS	POS	POS	NEG
IS-189	POS	NEG	POS	NEG	POS	NEG	POS	POS	POS	NEG	NEG	POS	POS	POS	POS	NEG
MRGR3	POS	NEG	POS	NEG	POS	NEG	POS	POS	POS	NEG	NEG	POS	POS	POS	POS	NEG
smg07	POS	NEG	POS	NEG	POS	NEG	POS	POS	POS	NEG	NEG	POS	POS	POS	POS	NEG
AH1-AUS-EMRSA-3	POS	NEG	POS	AMB	POS	NEG	POS	POS	POS	AMB	NEG	POS	POS	POS	POS	NEG
Bengalaru_ST20141403	POS	NEG	POS	AMB	POS	NEG	POS	POS	POS	AMB	AMB	POS	POS	POS	POS	NEG
Finland E24_98541_Harmony	POS	NEG	POS	POS	AMB	NEG	POS	POS	AMB	POS	AMB	POS	POS	POS	POS	NEG
Hong Kong_10	POS	NEG	POS	AMB	POS	NEG	POS	POS	POS	AMB	AMB	POS	POS	POS	POS	NEG
Hong Kong_100	POS	NEG	POS	AMB	POS	NEG	POS	POS	POS	AMB	NEG	POS	POS	POS	POS	NEG
Hong Kong_199	POS	NEG	POS	POS	AMB	NEG	POS	POS	POS	AMB	AMB	POS	POS	POS	POS	NEG
Hong Kong_34	POS	NEG	POS	AMB	POS	NEG	NEG	POS	POS	AMB	AMB	POS	POS	POS	POS	NEG
Hong Kong_88	POS	NEG	POS	POS	AMB	NEG	POS	POS	AMB	POS	AMB	POS	POS	POS	POS	NEG
Hong Kong_92	POS	NEG	POS	POS	AMB	NEG	POS	POS	POS	AMB	AMB	POS	POS	POS	POS	NEG
Hong Kong_94	POS	NEG	POS	AMB	POS	NEG	POS	POS	POS	AMB	AMB	POS	POS	POS	POS	NEG
Kuwait_257	POS	NEG	POS	AMB	POS	NEG	POS	POS	POS	AMB	AMB	POS	POS	POS	POS	NEG
Perth_01-15330	POS	NEG	POS	NEG	POS	NEG	POS	POS	POS	NEG	NEG	POS	POS	POS	POS	NEG
Perth_01-15349	AMB	NEG	POS	NEG	POS	NEG	POS	POS	POS	NEG	NEG	POS	POS	POS	POS	NEG
Perth_01-16176	POS	NEG	POS	AMB	POS	NEG	POS	POS	POS	AMB	NEG	POS	POS	POS	POS	NEG
Perth_WBG8379	POS	NEG	AMB	AMB	POS	NEG	POS	POS	POS	AMB	NEG	POS	POS	POS	POS	NEG
Rawalpindi_Kidney Center_56	POS	NEG	POS	AMB	POS	NEG	POS	POS	POS	POS	AMB	POS	POS	POS	POS	NEG
Riyadh_SSTI_05_3464614	POS	NEG	POS	AMB	POS	NEG	POS	POS	POS	AMB	NEG	POS	POS	POS	POS	NEG
Trinidad&Tobago_2012_157	POS	NEG	POS	POS	AMB	NEG	POS	POS	AMB	POS	AMB	POS	POS	POS	POS	NEG

STRAIN / ISOLATE	VIRULENCE : STAPHYLOCOCCAL SUPERANTIGEN/ENTEROTOXIN-LIKE GENES (SET/SSL)															
	ssl04		ssl05				ssl06		ssl07			ssl08		ssl09		
	ssl04/set9	ssl04/set9 (MRSA252, SAR0425)	ssl05/set3_probe 1	ssl05/set3 (RF122, probe-611)	ssl05/set3_probe 2 (612)	ssl05/set3 (MRSA252)	ssl06/set21	ssl06 (NCTC8325+MW2)	ssl07/set1	ssl07/set1 (MRSA252)	ssl07/set1 (AF188836)	ssl08/set12_probe 1	ssl08/set12_probe 2	ssl09/set5_probe 1	ssl09/set5_probe 2	ssl09/set5 (MRSA252)
	Staphylococcal superantigen-like protein 4		Staphylococcal superantigen-like protein 5				Staphylococcal superantigen-like protein 6		Staphylococcal superantigen-like protein 7			Staphylococcal superantigen-like protein 8		Staphylococcal superantigen-like protein 9		
>"South-East Asian Clad																
M418	POS	NEG	POS	NEG	POS	NEG	POS	POS	POS	NEG	NEG	POS	POS	POS	POS	NEG
>"South-East Asian Clad																
NMR07 (India)	POS	NEG	POS	NEG	POS	NEG	POS	POS	POS	NEG	NEG	POS	POS	POS	POS	NEG
NMR08 (India)	POS	NEG	POS	NEG	POS	NEG	POS	POS	POS	NEG	NEG	POS	POS	POS	POS	NEG
Bengalaru_ST20121978	POS	AMB	POS	AMB	POS	NEG	POS	POS	POS	AMB	AMB	POS	POS	POS	POS	NEG
Hong Kong_89	POS	NEG	POS	POS	AMB	NEG	POS	POS	AMB	POS	AMB	POS	POS	POS	POS	NEG
>"South-East Asian Clad																
Perth_2005_AH_5	POS	NEG	POS	NEG	POS	NEG	POS	POS	POS	AMB	NEG	POS	POS	POS	POS	NEG
>"South-East Asian Clad																
Trinidad&Tobago_MRSA2010_103	POS	NEG	POS	POS	POS	NEG	POS	POS	POS	POS	NEG	POS	POS	POS	POS	NEG
Trinidad&Tobago_2013_PA25	POS	NEG	POS	AMB	POS	NEG	POS	POS	POS	POS	AMB	POS	POS	POS	POS	NEG
Trinidad&Tobago_MRSA2010_110	POS	NEG	POS	POS	NEG	NEG	POS	POS	AMB	POS	AMB	POS	POS	POS	POS	NEG
Trinidad&Tobago_SSTI 2012_111	POS	NEG	POS	NEG	POS	NEG	POS	POS	POS	POS	NEG	POS	POS	POS	POS	NEG
Trinidad&Tobago_MRSA2010_158	POS	NEG	POS	POS	NEG	NEG	POS	POS	POS	AMB	AMB	POS	POS	POS	POS	NEG
>"South-East Asian Clad																
Kuwait_122	POS	NEG	POS	NEG	POS	NEG	POS	POS	POS	AMB	NEG	POS	POS	POS	POS	NEG
Kuwait_002	POS	NEG	POS	NEG	POS	NEG	POS	POS	POS	POS	NEG	POS	POS	POS	POS	NEG
Kuwait_196	POS	NEG	POS	AMB	POS	NEG	POS	POS	POS	AMB	NEG	POS	POS	POS	POS	NEG
Kuwait_199	POS	NEG	POS	POS	POS	NEG	POS	POS	POS	POS	NEG	POS	POS	POS	POS	NEG
Hong Kong_136	NEG	NEG	NEG	NEG	NEG	NEG	NEG	AMB	POS	AMB	AMB	POS	POS	POS	POS	NEG
>"South-East Asian Clad																
P32 (Poland)	POS	NEG	POS	NEG	POS	NEG	POS	POS	POS	NEG	NEG	POS	POS	POS	POS	NEG
>"South-East Asian Clad																
Perth_2015-S32313	POS	NEG	POS	NEG	POS	NEG	POS	POS	POS	NEG	NEG	NEG	NEG	POS	POS	NEG
>"hla-negative South-Ea																
DEN907 (Denmark)	POS	NEG	POS	NEG	POS	NEG	POS	POS	POS	NEG	NEG	POS	POS	POS	POS	NEG
>"hla-negative South-Ea																
S26 (Thailand)	POS	NEG	POS	NEG	POS	NEG	POS	POS	POS	NEG	NEG	POS	POS	POS	POS	NEG
S42 (Thailand)	POS	NEG	POS	NEG	POS	NEG	POS	POS	POS	NEG	NEG	POS	POS	POS	POS	NEG
S97 (Thailand)	POS	NEG	POS	NEG	POS	NEG	POS	POS	POS	NEG	NEG	POS	POS	POS	POS	NEG
S78 (Thailand)	POS	NEG	POS	NEG	POS	NEG	POS	POS	POS	NEG	NEG	POS	POS	POS	POS	NEG
S25 (Thailand)	POS	NEG	POS	NEG	POS	NEG	POS	POS	POS	NEG	NEG	POS	POS	POS	POS	NEG
S21 (Thailand)	POS	NEG	POS	NEG	POS	NEG	POS	POS	POS	NEG	NEG	POS	POS	POS	POS	NEG
S106 (Thailand)	POS	NEG	POS	NEG	POS	NEG	POS	POS	POS	NEG	NEG	POS	POS	POS	POS	NEG
S24 (Thailand)	POS	NEG	POS	NEG	POS	NEG	POS	POS	POS	NEG	NEG	POS	POS	POS	POS	NEG
S39 (Thailand)	POS	NEG	POS	NEG	POS	NEG	POS	POS	POS	NEG	NEG	POS	POS	POS	POS	NEG
S2 (Thailand)	POS	NEG	POS	NEG	POS	NEG	POS	POS	POS	NEG	NEG	POS	POS	POS	POS	NEG
S81 (Thailand)	POS	NEG	POS	NEG	POS	NEG	POS	POS	POS	NEG	NEG	POS	POS	POS	POS	NEG
M116 (Vietnam)	POS	NEG	POS	NEG	POS	NEG	POS	POS	POS	NEG	NEG	POS	POS	POS	POS	NEG
>"Portuguese Clade": Pc																
FFP103 (Portugal)	POS	NEG	POS	NEG	POS	NEG	POS	POS	POS	NEG	NEG	POS	POS	POS	POS	NEG
HDG2 (Portugal)	POS	NEG	POS	NEG	POS	NEG	POS	POS	POS	NEG	NEG	POS	POS	POS	POS	NEG
HSA10 (Portugal)	POS	NEG	POS	NEG	POS	NEG	POS	POS	POS	NEG	NEG	POS	POS	POS	POS	NEG
HSA11 (Portugal)	POS	NEG	POS	NEG	POS	NEG	POS	POS	POS	NEG	NEG	POS	POS	POS	POS	NEG
ICP5011 (Portugal)	POS	NEG	POS	NEG	POS	NEG	POS	POS	POS	NEG	NEG	POS	POS	POS	POS	NEG
ICP5014 (Portugal)	POS	NEG	POS	NEG	POS	NEG	POS	POS	POS	NEG	NEG	POS	POS	POS	POS	NEG
ICP5062 (Portugal)	POS	NEG	POS	NEG	POS	NEG	POS	POS	POS	NEG	NEG	POS	POS	POS	POS	NEG
Strain 1063_ATCC 33592 FDAARGOS_3 (USA)	POS	NEG	POS	NEG	POS	NEG	POS	POS	POS	NEG	NEG	POS	POS	POS	POS	NEG
	POS	NEG	POS	POS	POS	NEG	POS	POS	POS	POS	AMB	POS	POS	POS	POS	NEG
Russia-07_0110_Kurgan	POS	NEG	POS	AMB	POS	NEG	POS	POS	POS	AMB	NEG	NEG	NEG	NEG	NEG	NEG
Russia-11_0164_Kurgan	POS	NEG	POS	AMB	POS	NEG	POS	POS	POS	AMB	AMB	POS	POS	POS	POS	NEG
Russia-20_0367_Kurgan	POS	NEG	POS	AMB	POS	NEG	POS	POS	POS	AMB	AMB	POS	POS	POS	POS	NEG
Russia-21_0390_Chelyabinsk	POS	NEG	POS	AMB	POS	NEG	POS	POS	POS	AMB	AMB	POS	POS	POS	POS	NEG
>"South American/ Mid																
AGT1 (Argentina)	POS	NEG	POS	NEG	POS	NEG	POS	POS	POS	NEG	NEG	POS	POS	POS	POS	NEG
AGT120 (Argentina)	POS	NEG	POS	NEG	POS	NEG	POS	POS	POS	NEG	NEG	POS	POS	POS	POS	NEG
AGT67 (Argentina)	AMB	NEG	POS	NEG	POS	NEG	POS	POS	POS	NEG	NEG	POS	POS	POS	POS	NEG
AGT9 (Argentina)	POS	NEG	POS	NEG	POS	NEG	POS	POS	POS	NEG	NEG	POS	POS	POS	POS	NEG
RA3 (Argentina)	POS	NEG	POS	NEG	POS	NEG	POS	POS	POS	NEG	NEG	POS	POS	POS	POS	NEG
RA6 (Argentina)	POS	NEG	POS	NEG	POS	NEG	POS	POS	POS	NEG	NEG	POS	POS	POS	POS	NEG
RA7 (Argentina)	NEG	NEG	POS	NEG	POS	NEG	POS	POS	POS	NEG	NEG	POS	POS	POS	POS	NEG
Be62 (Brazil)	POS	NEG	POS	NEG	POS	NEG	POS	POS	POS	NEG	NEG	POS	POS	POS	POS	NEG
Bmb9393 (Brazil)	POS	NEG	POS	NEG	POS	NEG	POS	POS	POS	NEG	NEG	POS	POS	POS	POS	NEG
BRA36 (Brazil)	POS	NEG	POS	NEG	POS	NEG	POS	POS	POS	NEG	NEG	POS	POS	POS	POS	NEG
BZ48 (Brazil)	POS	NEG	POS	NEG	POS	NEG	POS	POS	POS	NEG	NEG	POS	POS	POS	POS	NEG
Gv51 (Brazil)	POS	NEG	POS	NEG	POS	NEG	POS	POS	POS	NEG	NEG	POS	POS	POS	POS	NEG
Gv69 (Brazil)	POS	NEG	POS	NEG	POS	NEG	POS	POS	POS	NEG	NEG	POS	POS	POS	POS	NEG
Gv88 (Brazil)	POS	NEG	POS	NEG	POS	NEG	POS	POS	POS	NEG	NEG	POS	POS	POS	POS	NEG
HC1335 (Brazil)	POS	NEG	POS	NEG	POS	NEG	POS	POS	POS	NEG	NEG	POS	POS	POS	POS	NEG
HC1340 (Brazil)	POS	NEG	POS	NEG	POS	NEG	POS	POS	POS	NEG	NEG	POS	POS	POS	POS	NEG
HC556 (Brazil)	POS	NEG	POS	NEG	POS	NEG	POS	POS	POS	NEG	NEG	POS	POS	POS	POS	NEG
HU25 (Brazil)	POS	NEG	POS	NEG	POS	NEG	POS	POS	POS	NEG	NEG	POS	POS	POS	POS	NEG
UB563 (Brazil)	POS	NEG	POS	NEG	POS	NEG	POS	POS	POS	NEG	NEG	POS	POS	POS	POS	NEG
2A8 (Czech Republic)	POS	NEG	POS	NEG	POS	NEG	POS	POS	POS	NEG	NEG	POS	POS	POS	POS	NEG
M1229 (Denmark)	POS	NEG	POS	NEG	POS	NEG	POS	POS	POS	NEG	NEG	POS	POS	NEG	NEG	NEG
FRICAR (France)	POS	NEG	POS	NEG	POS	NEG	POS	POS	POS	NEG	NEG	POS	POS	POS	POS	NEG
D90 (Germany)	POS	NEG	POS	NEG	POS	NEG	POS	POS	POS	NEG	NEG	POS	POS	POS	POS	NEG
LIT68 (Lithuania)	POS	NEG	POS	NEG	POS	NEG	POS	POS	POS	NEG	NEG	POS	POS	POS	POS	NEG
LIT76 (Lithuania)	POS	NEG	POS	NEG	POS	NEG	POS	POS	POS	NEG	NEG	POS	POS	POS	POS	NEG
HGSA142 (Portugal)	POS	NEG	POS	NEG	POS	NEG	POS	POS	POS	NEG	NEG	POS	POS	POS	POS	NEG
HGSA9 (Portugal)	POS	NEG	POS	NEG	POS	NEG	POS	POS	POS	NEG	NEG	POS	POS	POS	POS	NEG
HSJ216 (Portugal)	POS	NEG	POS	NEG	POS	NEG	POS	POS	POS	NEG	NEG	POS	POS	POS	POS	NEG
M278 (Portugal)	POS	NEG	POS	NEG	POS	NEG	POS	POS	POS	NEG	NEG	POS	POS	POS	POS	NEG
ES26 (Spain)	POS	NEG	POS	NEG	POS	NEG	POS	POS	POS	NEG	NEG	POS	POS	POS	POS	NEG
UK102 (UK)	POS	NEG	POS	NEG	POS	NEG	POS	POS	POS	NEG	NEG	POS	POS	POS	POS	NEG
URU34 (Uruguay)	POS	NEG	POS	NEG	POS	NEG	POS	POS	POS	NEG	NEG	POS	POS	POS	POS	NEG
IS-125	POS	NEG	POS	NEG	POS	NEG	POS	POS	POS	NEG	NEG	POS	POS	POS	POS	NEG
IS-157	POS	NEG	POS	NEG	POS	NEG	POS	POS	POS	NEG	NEG	POS	POS	POS	POS	NEG
Ecuador_1	POS	NEG	POS	AMB	POS	NEG	POS	POS	AMB	POS	AMB	POS	POS	POS	POS	NEG
Ecuador_2	POS	NEG	POS	AMB	POS	NEG	POS	POS	POS	AMB	AMB	POS	POS	POS	POS	NEG
Dresden_15ANR574263	POS	NEG	POS	AMB	POS	NEG	POS	POS	POS	AMB	AMB	POS	POS	POS	POS	NEG
UK-EMRSA-11	POS	NEG	POS	AMB	POS	NEG	POS	POS	POS	AMB	AMB	POS	POS	POS	POS	NEG
>"South American/ Mid																
BRA2 (Brazil)	POS	NEG	POS	NEG	POS	NEG	POS	POS	POS	NEG	NEG	POS	POS	POS	POS	NEG
CHL1 (Chile)	POS	NEG	POS	NEG	POS	NEG	POS	POS	POS	NEG	NEG	POS	POS	POS	POS	NEG
CHL151 (Chile)	POS	NEG	POS	NEG	POS	NEG	POS	POS	POS	NEG	NEG	POS	POS	POS	POS	NEG
UCL378 (Chile)	POS	NEG	POS	NEG	POS	NEG	POS	POS	POS	NEG	NEG	POS	POS	POS	POS	NEG
UP72 (Peru)	POS	NEG	POS	NEG	POS	NEG	POS	POS	POS	NEG	NEG	POS	POS	POS	POS	NEG
UP81 (Peru)	POS	NEG	POS	NEG	POS	NEG	POS	POS	POS	NEG	NEG	POS	POS	POS	POS	NEG
>"South American/ Mid																
UP1073 (Peru)	POS	NEG	POS	NEG	POS	NEG	POS	POS	POS	NEG	NEG	POS	POS	POS	POS	NEG

STRAIN / ISOLATE	VIRULENCE : STAPHYLOCOCCAL SUPERANTIGEN/ENTEROTOXIN-LIKE GENES (SET/SSL)															
	ssl04		ssl05				ssl06		ssl07			ssl08		ssl09		
	ssl04/set9	ssl04/set9 (MRSA252, SAR0425)	ssl05/set3_probe 1	ssl05/set3 (RF122, probe-611)	ssl05/set3_probe 2 (612)	ssl05/set3 (MRSA252)	ssl06/set21	ssl06 (NCTC8325+MW2)	ssl07/set1	ssl07/set1 (MRSA252)	ssl07/set1 (AF188836)	ssl08/set12_probe 1	ssl08/set12_probe 2	ssl09/set5_probe 1	ssl09/set5_probe 2	ssl09/set5 (MRSA252)
	Staphylococcal superantigen-like protein 4		Staphylococcal superantigen-like protein 5				Staphylococcal superantigen-like protein 6		Staphylococcal superantigen-like protein 7			Staphylococcal superantigen-like protein 8		Staphylococcal superantigen-like protein 9		
>"South American/ Mid																
LIT89 (Lithuania)	POS	NEG	POS	NEG	POS	NEG	POS	POS	POS	NEG	NEG	POS	POS	POS	POS	NEG
MRSA_PR1 (Malaysia)	POS	NEG	POS	NEG	POS	NEG	NEG	NEG	POS	NEG	NEG	POS	POS	POS	POS	NEG
Riyadh-2888905-R	POS	NEG	POS	AMB	POS	NEG	POS	POS	POS	POS	NEG	POS	POS	POS	POS	NEG
Riyadh-2888915-BC	POS	NEG	POS	AMB	POS	NEG	POS	POS	POS	POS	NEG	POS	POS	POS	POS	NEG
Riyadh-2793706-R	POS	NEG	POS	POS	AMB	NEG	POS	POS	AMB	POS	AMB	POS	POS	POS	POS	NEG
>"South American/ Mid																
Riyadh_SSTI_18_3502925	POS	AMB	POS	POS	AMB	NEG	POS	POS	AMB	POS	AMB	POS	POS	POS	POS	NEG
Riyadh-2822825-W	POS	NEG	POS	POS	AMB	NEG	POS	POS	AMB	POS	AMB	POS	POS	POS	POS	NEG
>"South American/ Mid																
UK-EMRSA-9	POS	NEG	POS	AMB	POS	NEG	POS	POS	POS	AMB	AMB	POS	POS	POS	POS	NEG
>"South American/ Mid																
Lome_HT20020815	POS	NEG	POS	AMB	POS	NEG	POS	POS	POS	AMB	AMB	POS	POS	POS	POS	NEG
>"South American/ Mid																
NA32 (Denmark)	POS	NEG	POS	NEG	POS	NEG	POS	POS	POS	NEG	NEG	POS	POS	POS	POS	NEG
H24 (Egypt)	POS	NEG	POS	NEG	POS	NEG	POS	POS	POS	NEG	NEG	POS	POS	POS	POS	NEG
ATCC BAA-39 (=HUSA304) (Hungary)	POS	NEG	POS	NEG	POS	NEG	POS	POS	POS	NEG	NEG	POS	POS	POS	POS	NEG
HUSA304 (Hungary)	POS	NEG	POS	NEG	POS	NEG	POS	POS	POS	NEG	NEG	POS	POS	POS	POS	NEG
HU106 (Hungary)	POS	NEG	POS	NEG	POS	NEG	POS	POS	POS	NEG	NEG	POS	POS	POS	POS	NEG
BSAC27 (UK)	POS	NEG	POS	NEG	POS	NEG	POS	POS	POS	NEG	NEG	POS	POS	POS	POS	NEG
NCTC13131, UK-EMRSA-4 (UK)	POS	NEG	POS	NEG	POS	NEG	POS	POS	POS	NEG	NEG	POS	POS	POS	POS	NEG
	POS	NEG	POS	AMB	POS	NEG	POS	POS	POS	AMB	AMB	POS	POS	POS	POS	NEG
Algiers_HT20040080	POS	NEG	POS	AMB	POS	NEG	POS	POS	POS	AMB	AMB	POS	POS	POS	POS	NEG
Dublin-DSH_AR09_0.0066	POS	NEG	POS	AMB	POS	NEG	POS	POS	POS	AMB	AMB	POS	POS	POS	POS	NEG
Dublin-DSH_AR09_0-0065	POS	NEG	POS	NEG	POS	NEG	NEG	POS	POS	AMB	NEG	POS	POS	POS	POS	NEG
Dublin-DSH_Phenotype-III_84	POS	NEG	POS	AMB	POS	NEG	POS	POS	POS	AMB	NEG	POS	POS	POS	POS	NEG
Hong Kong_130	NEG	NEG	NEG	NEG	NEG	NEG	NEG	NEG	POS	AMB	NEG	POS	POS	POS	POS	NEG
Kuwait_018	POS	NEG	POS	NEG	POS	NEG	POS	POS	POS	AMB	NEG	POS	POS	POS	POS	NEG
Perth_08-17726	POS	NEG	POS	AMB	POS	NEG	POS	POS	POS	POS	NEG	POS	POS	NEG	NEG	NEG
Riyadh_Alfaisal/KKKSUH_86_MRS-14-279	POS	NEG	POS	AMB	POS	NEG	POS	POS	POS	AMB	NEG	POS	POS	POS	POS	NEG
Riyadh_Alfaisal-04_Z3861_831588	POS	NEG	POS	AMB	POS	NEG	POS	POS	POS	AMB	AMB	POS	POS	POS	POS	NEG
Riyadh_Alfaisal-30_S15724_1108013	POS	NEG	POS	AMB	POS	NEG	POS	POS	POS	AMB	AMB	POS	POS	POS	POS	NEG
Riyadh_Alfaisal-6_22A_13_B3992_397721	POS	NEG	POS	POS	AMB	NEG	POS	POS	AMB	POS	AMB	POS	POS	POS	POS	NEG
Riyadh_SSTI_52_3615482	POS	NEG	POS	AMB	POS	NEG	POS	POS	POS	AMB	AMB	POS	POS	POS	POS	NEG
Riyadh-2817276-2	POS	NEG	POS	POS	AMB	NEG	POS	POS	AMB	POS	AMB	POS	POS	POS	POS	NEG
Riyadh-2891670-W	POS	NEG	POS	POS	AMB	NEG	POS	POS	AMB	POS	AMB	POS	POS	POS	POS	NEG
Riyadh-3006920-W	POS	NEG	POS	AMB	POS	NEG	POS	POS	AMB	POS	AMB	POS	POS	POS	POS	NEG
Riyadh-R2567782	POS	NEG	POS	AMB	POS	NEG	POS	POS	POS	AMB	AMB	POS	POS	POS	POS	NEG
Russia-18_0252_Krasnoyarsk_SK2	POS	NEG	POS	AMB	POS	NEG	POS	POS	POS	AMB	NEG	POS	POS	POS	POS	NEG
UK-EMRSA-7	POS	NEG	POS	AMB	POS	NEG	POS	POS	POS	AMB	AMB	POS	POS	POS	POS	NEG
>"South American/ Mid																
Dublin-DSH_AR23_0073	NEG	NEG	POS	AMB	POS	NEG	AMB	POS	POS	AMB	NEG	POS	POS	POS	POS	NEG
>Related to "South Ame																
MRSA-OC3 (Russia)	POS	NEG	POS	NEG	POS	NEG	POS	POS	POS	NEG	NEG	POS	POS	POS	POS	NEG
Russia-12_0176_Krasnoyarsk	POS	NEG	POS	AMB	POS	NEG	POS	POS	POS	AMB	AMB	POS	POS	POS	POS	NEG
Russia-13_0180_Krasnoyarsk	POS	NEG	POS	AMB	POS	NEG	POS	POS	POS	AMB	AMB	POS	POS	POS	POS	NEG
Russia-16_0249_Krasnoyarsk	POS	NEG	POS	AMB	POS	NEG	POS	POS	POS	AMB	AMB	POS	POS	POS	POS	NEG
Russia-17_0250_Krasnoyarsk	POS	NEG	POS	AMB	POS	NEG	POS	POS	POS	AMB	AMB	POS	POS	POS	POS	NEG
>Related to "South Ame																
URU110 (Uruguay)	NEG	NEG	POS	NEG	POS	NEG	POS	POS	POS	NEG	NEG	POS	POS	POS	POS	NEG
>Related to "South Ame																
DS_014 (Thailand)	POS	NEG	POS	NEG	POS	NEG	POS	POS	POS	NEG	NEG	POS	POS	NEG	NEG	NEG
>Unassigned Middle Eas																
Frankfurt_Oder_0490031797 (Libya)	POS	NEG	POS	POS	AMB	NEG	POS	POS	AMB	POS	AMB	POS	POS	POS	POS	NEG
Kuwait_192	POS	POS	POS	POS	AMB	NEG	POS	POS	AMB	POS	AMB	POS	POS	POS	POS	NEG
Riyadh-9	POS	NEG	POS	AMB	POS	NEG	POS	POS	POS	AMB	AMB	POS	POS	POS	POS	NEG
Russia-24_0407_Moscow	POS	NEG	POS	AMB	POS	NEG	POS	POS	POS	AMB	AMB	POS	POS	POS	POS	NEG

STRAIN / ISOLATE	VIRULENCE : STAPHYLOCOCCAL SUPERANTIGEN/ENTEROTOXIN-LIKE GENES (SET/SSL)												CAPSULE- /BIOFILM-ASSOCIATED GENES				
	ssl10			ssl11				setB3		setB2		setB1	Capsule type 1	Capsule type 5	Capsule type 8	icaA/C/D	bap
	ssl10/set4	ssl10 (RF122)	ssl10/set4 (MRSA252)	ssl11/set2 (COL)	ssl11+set2(Mu50+N315)	ssl11+set2(MW2+MSSA476)	ssl11/set2 (MRSA252)	setB3	setB3 (MRSA252)	setB2	setB2 (MRSA252)	setB1					
	Staphylococcal superantigen-like protein 10			Staphylococcal superantigene-like protein 11				Staphylococcal exotoxin-like protein, second locus					Capsule type 1	Capsule type 5	Capsule type 8	intercellular adhesion protein A	Surface protein involved in biofilm formation
>"Eurasian Clade": TUR1																	
TUR1 (Turkey)	POS	NEG	NEG	POS	NEG	NEG	NEG	POS	NEG	POS	NEG	POS	NEG	NEG	POS	POS	NEG
TUR9 (Turkey)	POS	NEG	NEG	POS	NEG	NEG	NEG	POS	NEG	POS	NEG	POS	NEG	NEG	POS	POS	NEG
>"Eurasian Clade" : T013																	
T0131 (China)	POS	NEG	NEG	POS	NEG	NEG	NEG	POS	NEG	POS	NEG	POS	NEG	NEG	POS	POS	NEG
MU4 (Turkey)	POS	NEG	NEG	POS	NEG	NEG	NEG	POS	NEG	POS	NEG	POS	NEG	NEG	POS	POS	NEG
DEU11 (Turkey)	POS	NEG	NEG	POS	NEG	NEG	NEG	POS	NEG	POS	NEG	POS	NEG	NEG	POS	POS	NEG
DEU16 (Turkey)	POS	NEG	NEG	POS	NEG	NEG	NEG	POS	NEG	POS	NEG	POS	NEG	NEG	POS	POS	NEG
Romania_Iasi_MRSA-07_54	POS	AMB	AMB	POS	NEG	NEG	NEG	POS	NEG	POS	NEG	POS	NEG	NEG	POS	POS	NEG
>"Eurasian Clade": arsC-																	
Romania_Iasi_MRSA-05_46	POS	NEG	AMB	POS	NEG	NEG	NEG	POS	NEG	POS	NEG	POS	NEG	NEG	POS	POS	NEG
Romania_Iasi_MRSA-06_47	POS	AMB	AMB	POS	NEG	NEG	NEG	POS	NEG	POS	NEG	POS	NEG	NEG	POS	POS	NEG
>"Eurasian Clade": 16K/																	
CN79 (China)	POS	NEG	NEG	POS	NEG	NEG	NEG	POS	NEG	POS	NEG	POS	NEG	NEG	POS	POS	NEG
CUHK_BJ2002 (China)	POS	NEG	NEG	POS	NEG	NEG	NEG	POS	NEG	POS	NEG	POS	NEG	NEG	POS	POS	NEG
CUHK_BJ2007 (China)	POS	NEG	NEG	POS	NEG	NEG	NEG	POS	NEG	POS	NEG	POS	NEG	NEG	POS	POS	NEG
3HK (Czech Republic)	POS	NEG	NEG	POS	NEG	NEG	NEG	POS	NEG	POS	NEG	POS	NEG	NEG	POS	POS	NEG
HU109 (Hungary)	POS	NEG	NEG	POS	NEG	NEG	NEG	POS	NEG	POS	NEG	POS	NEG	NEG	POS	POS	NEG
HUR18 (Hungary)	POS	NEG	NEG	POS	NEG	NEG	NEG	POS	NEG	POS	NEG	POS	NEG	NEG	POS	POS	NEG
H482 (Romania)	POS	NEG	NEG	POS	NEG	NEG	NEG	POS	NEG	POS	NEG	POS	NEG	NEG	POS	POS	NEG
16K (Russia)	POS	NEG	NEG	POS	NEG	NEG	NEG	POS	NEG	POS	NEG	POS	NEG	NEG	POS	POS	NEG
DEU10 (Turkey)	POS	NEG	NEG	POS	NEG	NEG	NEG	POS	NEG	POS	NEG	POS	NEG	NEG	POS	POS	NEG
DEU12 (Turkey)	POS	NEG	NEG	POS	NEG	NEG	NEG	POS	NEG	POS	NEG	POS	NEG	NEG	POS	POS	NEG
DEU14 (Turkey)	POS	NEG	NEG	POS	NEG	NEG	NEG	POS	NEG	POS	NEG	POS	NEG	NEG	POS	POS	NEG
DEU15 (Turkey)	POS	NEG	NEG	POS	NEG	NEG	NEG	POS	NEG	POS	NEG	POS	NEG	NEG	POS	POS	NEG
DEU17 (Turkey)	POS	NEG	NEG	POS	NEG	NEG	NEG	POS	NEG	POS	NEG	POS	NEG	NEG	POS	POS	NEG
DEU19 (Turkey)	POS	NEG	NEG	POS	NEG	NEG	NEG	POS	NEG	POS	NEG	POS	NEG	NEG	POS	POS	NEG
DEU2 (Turkey)	POS	NEG	NEG	POS	NEG	NEG	NEG	POS	NEG	POS	NEG	POS	NEG	NEG	POS	POS	NEG
DEU20 (Turkey)	POS	NEG	NEG	POS	NEG	NEG	NEG	POS	NEG	POS	NEG	POS	NEG	NEG	POS	POS	NEG
DEU23 (Turkey)	POS	NEG	NEG	POS	NEG	NEG	NEG	POS	NEG	POS	NEG	POS	NEG	NEG	POS	POS	NEG
DEU3 (Turkey)	POS	NEG	NEG	POS	NEG	NEG	NEG	POS	NEG	POS	NEG	POS	NEG	NEG	POS	POS	NEG
DEU5 (Turkey)	POS	NEG	NEG	POS	NEG	NEG	NEG	POS	NEG	POS	NEG	POS	NEG	NEG	POS	POS	NEG
DEU6 (Turkey)	POS	NEG	NEG	POS	NEG	NEG	NEG	POS	NEG	POS	NEG	POS	NEG	NEG	POS	POS	NEG
DEU8 (Turkey)	POS	NEG	NEG	POS	NEG	NEG	NEG	POS	NEG	POS	NEG	POS	NEG	NEG	POS	POS	NEG
HU11 (Turkey)	POS	NEG	NEG	POS	NEG	NEG	NEG	POS	NEG	POS	NEG	POS	NEG	NEG	POS	POS	NEG
HU13 (Turkey)	POS	NEG	NEG	POS	NEG	NEG	NEG	POS	NEG	POS	NEG	POS	NEG	NEG	POS	POS	NEG
HU14 (Turkey)	POS	NEG	NEG	POS	NEG	NEG	NEG	POS	NEG	POS	NEG	POS	NEG	NEG	POS	POS	NEG
HU15 (Turkey)	POS	NEG	NEG	POS	NEG	NEG	NEG	POS	NEG	POS	NEG	POS	NEG	NEG	POS	POS	NEG
HU16 (Turkey)	POS	NEG	NEG	POS	NEG	NEG	NEG	POS	NEG	POS	NEG	POS	NEG	NEG	POS	POS	NEG
HU17 (Turkey)	POS	NEG	NEG	POS	NEG	NEG	NEG	POS	NEG	POS	NEG	POS	NEG	NEG	POS	POS	NEG
HU21 (Turkey)	POS	NEG	NEG	POS	NEG	NEG	NEG	POS	NEG	POS	NEG	POS	NEG	NEG	POS	POS	NEG
HU23 (Turkey)	POS	NEG	NEG	POS	NEG	NEG	NEG	POS	NEG	POS	NEG	POS	NEG	NEG	POS	POS	NEG
HU26 (Turkey)	POS	NEG	NEG	POS	NEG	NEG	NEG	POS	NEG	POS	NEG	POS	NEG	NEG	POS	POS	NEG
HU4 (Turkey)	POS	NEG	NEG	POS	NEG	NEG	NEG	POS	NEG	POS	NEG	POS	NEG	NEG	POS	POS	NEG
HU5 (Turkey)	POS	NEG	NEG	POS	NEG	NEG	NEG	POS	NEG	POS	NEG	POS	NEG	NEG	POS	POS	NEG
HU6 (Turkey)	POS	NEG	NEG	POS	NEG	NEG	NEG	POS	NEG	POS	NEG	POS	NEG	NEG	POS	POS	NEG
HU7 (Turkey)	POS	NEG	NEG	POS	NEG	NEG	NEG	POS	NEG	POS	NEG	POS	NEG	NEG	POS	POS	NEG
HU8 (Turkey)	POS	NEG	NEG	POS	NEG	NEG	NEG	POS	NEG	POS	NEG	POS	NEG	NEG	POS	POS	NEG
HU9 (Turkey)	POS	NEG	NEG	POS	NEG	NEG	NEG	POS	NEG	POS	NEG	POS	NEG	NEG	POS	POS	NEG
IU1 (Turkey)	POS	NEG	NEG	POS	NEG	NEG	NEG	POS	NEG	POS	NEG	POS	NEG	NEG	POS	POS	NEG
IU10 (Turkey)	POS	NEG	NEG	POS	NEG	NEG	NEG	POS	NEG	POS	NEG	POS	NEG	NEG	POS	POS	NEG
IU11 (Turkey)	POS	NEG	NEG	POS	NEG	NEG	NEG	POS	NEG	POS	NEG	POS	NEG	NEG	POS	POS	NEG
IU12 (Turkey)	POS	NEG	NEG	POS	NEG	NEG	NEG	POS	NEG	POS	NEG	POS	NEG	NEG	POS	POS	NEG
IU13 (Turkey)	POS	NEG	NEG	POS	NEG	NEG	NEG	POS	NEG	POS	NEG	POS	NEG	NEG	POS	POS	NEG
IU15 (Turkey)	POS	NEG	NEG	POS	NEG	NEG	NEG	POS	NEG	POS	NEG	POS	NEG	NEG	POS	POS	NEG
IU18 (Turkey)	POS	NEG	NEG	POS	NEG	NEG	NEG	POS	NEG	POS	NEG	POS	NEG	NEG	POS	POS	NEG
IU19 (Turkey)	POS	NEG	NEG	POS	NEG	NEG	NEG	POS	NEG	POS	NEG	POS	NEG	NEG	POS	POS	NEG
IU2 (Turkey)	POS	NEG	NEG	POS	NEG	NEG	NEG	POS	NEG	POS	NEG	POS	NEG	NEG	POS	POS	NEG
IU4 (Turkey)	POS	NEG	NEG	POS	NEG	NEG	NEG	POS	NEG	POS	NEG	POS	NEG	NEG	POS	POS	NEG
IU6 (Turkey)	POS	NEG	NEG	POS	NEG	NEG	NEG	POS	NEG	POS	NEG	POS	NEG	NEG	POS	POS	NEG
IU7 (Turkey)	POS	NEG	NEG	POS	NEG	NEG	NEG	POS	NEG	POS	NEG	POS	NEG	NEG	POS	POS	NEG
IU9 (Turkey)	POS	NEG	NEG	POS	NEG	NEG	NEG	POS	NEG	POS	NEG	POS	NEG	NEG	POS	POS	NEG
MU1 (Turkey)	POS	NEG	NEG	POS	NEG	NEG	NEG	POS	NEG	POS	NEG	POS	NEG	NEG	POS	POS	NEG
MU10 (Turkey)	POS	NEG	NEG	POS	NEG	NEG	NEG	POS	NEG	POS	NEG	POS	NEG	NEG	POS	POS	NEG
MU20 (Turkey)	POS	NEG	NEG	POS	NEG	NEG	NEG	POS	NEG	POS	NEG	POS	NEG	NEG	POS	POS	NEG
Mu3 (Turkey)	POS	NEG	NEG	POS	NEG	NEG	NEG	POS	NEG	POS	NEG	POS	NEG	NEG	POS	POS	NEG
MU5 (Turkey)	POS	NEG	NEG	POS	NEG	NEG	NEG	POS	NEG	POS	NEG	POS	NEG	NEG	POS	POS	NEG
MU6 (Turkey)	POS	NEG	NEG	POS	NEG	NEG	NEG	POS	NEG	POS	NEG	POS	NEG	NEG	POS	POS	NEG
MU7 (Turkey)	POS	NEG	NEG	POS	NEG	NEG	NEG	POS	NEG	POS	NEG	POS	NEG	NEG	POS	POS	NEG
TUR27 (Turkey)	POS	NEG	NEG	POS	NEG	NEG	NEG	POS	NEG	POS	NEG	POS	NEG	NEG	POS	POS	NEG
NCTR 32S	POS	NEG	NEG	POS	NEG	NEG	NEG	POS	NEG	POS	NEG	POS	NEG	NEG	POS	POS	NEG
SA02_A4 = Russia-06_0085_Moscow	POS	NEG	NEG	POS	NEG	NEG	NEG	POS	NEG	POS	NEG	POS	NEG	NEG	POS	POS	NEG
	POS	AMB	AMB	POS	NEG	NEG	NEG	POS	NEG	POS	NEG	POS	NEG	NEG	POS	POS	NEG
Dresden_08V35987 (Turkey)	POS	NEG	NEG	POS	NEG	NEG	NEG	POS	NEG	POS	NEG	POS	NEG	NEG	POS	POS	NEG
Dresden_17ANR577152	POS	NEG	NEG	POS	NEG	NEG	NEG	POS	NEG	POS	NEG	POS	NEG	NEG	POS	POS	NEG
Dresden_17ANR580374 (Makedonia)	POS	NEG	NEG	POS	NEG	NEG	NEG	POS	NEG	POS	NEG	POS	NEG	NEG	POS	POS	NEG
Hong Kong_69-II	POS	AMB	AMB	POS	NEG	NEG	NEG	POS	NEG	POS	NEG	POS	NEG	NEG	POS	POS	NEG
Hong Kong_93	POS	AMB	AMB	POS	NEG	NEG	NEG	POS	NEG	POS	NEG	POS	NEG	NEG	POS	POS	NEG
Rawalpindi_Kidney Center_03_SK1	POS	AMB	AMB	POS	NEG	NEG	NEG	POS	NEG	POS	NEG	POS	NEG	NEG	POS	POS	NEG
Rawalpindi_Kidney Center_08	POS	NEG	AMB	POS	NEG	NEG	NEG	POS	NEG	POS	NEG	POS	NEG	NEG	POS	POS	NEG
Rawalpindi_Kidney Center_10	POS	AMB	AMB	POS	NEG	NEG	NEG	POS	NEG	POS	NEG	POS	NEG	NEG	POS	POS	NEG
Rawalpindi_Kidney Center_19	POS	AMB	AMB	POS	NEG	NEG	NEG	POS	NEG	POS	NEG	POS	NEG	NEG	POS	POS	NEG
Rawalpindi_Kidney Center_50_SK1	POS	NEG	AMB	POS	NEG	NEG	NEG	POS	NEG	POS	NEG	POS	NEG	NEG	POS	POS	NEG
Romania_Iasi_BC-49_430	POS	AMB	AMB	POS	NEG	NEG	NEG	POS	NEG	POS	NEG	POS	NEG	NEG	POS	POS	NEG
Romania_Iasi_MRSA_284	POS	AMB	AMB	POS	NEG	NEG	NEG	POS	NEG	POS	NEG	POS	NEG	NEG	POS	POS	NEG
Romania_Iasi_MRSA-32_318	POS	NEG	AMB	POS	NEG	NEG	NEG	POS	NEG	POS	NEG	POS	NEG	NEG	POS	POS	NEG
Romania_Iasi_MRSA-54_430	POS	AMB	AMB	POS	NEG	NEG	NEG	POS	NEG	POS	NEG	POS	NEG	NEG	POS	POS	NEG
Romania_Iasi_SSTI-01_2	POS	AMB	AMB	POS	NEG	NEG	NEG	POS	NEG	POS	NEG	POS	NEG	NEG	POS	POS	NEG
Romania_Iasi_SSTI-10_101	POS	NEG	AMB	POS	NEG	NEG	NEG	POS	NEG	POS	NEG	POS	NEG	NEG	POS	POS	NEG
Romania_Iasi_SSTI-11_106	POS	NEG	AMB	POS	NEG	NEG	NEG	POS	NEG	POS	NEG	POS	NEG	NEG	POS	POS	NEG
Russia-01_0001_SaintPetersburg	POS	NEG	AMB	POS	NEG	NEG	NEG	POS	NEG	POS	NEG	POS	NEG	NEG	POS	POS	NEG
Russia-02_0004_SaintPetersburg	POS	NEG	AMB	POS	NEG	NEG	NEG	POS	NEG	POS	NEG	POS	NEG	NEG	POS	POS	NEG
Russia-03_0057_SaintPetersburg	POS	NEG	NEG	POS	NEG	NEG	NEG	POS	NEG	POS	NEG	POS	NEG	NEG	POS	POS	NEG
Russia-04_0076_Moscow	POS	NEG	AMB	POS	NEG	NEG	NEG	POS	NEG	POS	NEG	POS	NEG	NEG	POS	POS	NEG
Russia-05_0078_Moscow	POS	NEG	AMB	POS	NEG	NEG	NEG	POS	NEG	POS	NEG	POS	NEG	NEG	POS	POS	NEG
Russia-08_0135_Moscow	POS	AMB	AMB	POS	NEG	NEG	NEG	POS	NEG	POS	NEG	POS	NEG	NEG	POS	POS	NEG
Russia-09_0150_Kurgan	POS	NEG	AMB	POS	NEG	NEG	NEG	POS	NEG	POS	NEG	POS	NEG	NEG	POS	POS	NEG
Russia-10_0162_Kurgan	POS	NEG	AMB	POS	NEG	NEG	NEG	POS	NEG	POS	NEG	POS	NEG	NEG	POS	POS	NEG
Russia-14_0184_Moscow	POS	NEG	AMB	POS	NEG	NEG	NEG	POS	NEG	POS	NEG	POS	NEG	NEG	POS	POS	NEG
Russia-15_0232_SaintPetersburg	POS	NEG	AMB	POS	NEG	NEG	NEG	POS	NEG	POS	NEG	POS	NEG	NEG	POS	POS	NEG
Russia-22_0391_Chelyabinsk	POS	NEG	AMB	POS	NEG	NEG	NEG	POS	NEG	POS	NEG	POS	NEG	NEG	POS	POS	NEG
Russia-23_0392_Chelyabinsk	POS	NEG	AMB	POS	NEG	NEG	NEG	POS	NEG	POS	NEG	POS	NEG	NEG	POS	POS	NEG
>"Eurasian Clade" : IU17																	
IU17 (Turkey)	POS	NEG	NEG	POS	NEG	NEG	NEG	POS	NEG	POS	NEG	POS	NEG	NEG	POS	POS	NEG
IU20 (Turkey)	POS	NEG	NEG	POS	NEG	NEG	NEG	POS	NEG	POS	NEG	POS	NEG	NEG	POS	POS	NEG

STRAIN / ISOLATE	VIRULENCE : STAPHYLOCOCCAL SUPERANTIGEN/ENTEROTOXIN-LIKE GENES (SET/SSL)												CAPSULE- /BIOFILM-ASSOCIATED GENES				
	ssl10			ssl11				setB3		setB2		setB1	Capsule type 1	Capsule type 5	Capsule type 8	icaA/C/D	bap
	ssl10/set4	ssl10 (RF122)	ssl10/set4 (MRSA252)	ssl11/set2 (COL)	ssl11+set2(Mu50+N315)	ssl11+set2(MW2+MSSA476)	ssl11/set2 (MRSA252)	setB3	setB3 (MRSA252)	setB2	setB2 (MRSA252)	setB1					
	Staphylococcal superantigen-like protein 10			Staphylococcal superantigene-like protein 11				Staphylococcal exotoxin-like protein, second locus					Capsule type 1	Capsule type 5	Capsule type 8	intercellular adhesion protein A	Surface protein involved in biofilm formation
**>"Eurasian Clade": Spora**																	
Dresden_16ANR581769	POS	NEG	NEG	POS	NEG	NEG	NEG	POS	NEG	POS	NEG	POS	NEG	NEG	POS	POS	NEG
Dresden_17ANR578769	POS	NEG	NEG	POS	NEG	NEG	NEG	POS	NEG	POS	NEG	POS	NEG	NEG	POS	POS	NEG
**>"Eurasian Clade": Kuwa**																	
Kuwait_306	POS	AMB	AMB	POS	NEG	NEG	NEG	POS	NEG	POS	NEG	POS	NEG	NEG	POS	POS	NEG
Kuwait_301	POS	AMB	AMB	POS	NEG	NEG	NEG	POS	NEG	POS	NEG	POS	NEG	NEG	POS	POS	NEG
**>"European Clade": "Gre**																	
GRE18 (Greece)	POS	NEG	NEG	POS	NEG	NEG	NEG	POS	NEG	POS	NEG	POS	NEG	NEG	POS	POS	NEG
GRE317 (Greece)	POS	NEG	NEG	POS	NEG	NEG	NEG	POS	NEG	POS	NEG	POS	NEG	NEG	POS	POS	NEG
GRE4 (Greece)	POS	NEG	NEG	POS	NEG	NEG	NEG	POS	NEG	POS	NEG	POS	NEG	NEG	POS	POS	NEG
R35 (USA)	POS	NEG	NEG	POS	NEG	NEG	NEG	POS	NEG	POS	NEG	POS	NEG	NEG	POS	POS	NEG
Casablanca_HT20060548	POS	NEG	AMB	POS	NEG	NEG	NEG	POS	NEG	POS	NEG	POS	NEG	NEG	POS	POS	NEG
Casablanca_HT20060550	POS	NEG	AMB	POS	NEG	NEG	NEG	POS	NEG	POS	NEG	POS	NEG	NEG	POS	POS	NEG
Dresden_01V34572 (Greece)	POS	NEG	NEG	POS	NEG	NEG	NEG	POS	NEG	POS	NEG	POS	NEG	NEG	POS	POS	NEG
Dresden_01V36469	POS	NEG	NEG	POS	NEG	NEG	NEG	POS	NEG	POS	NEG	POS	NEG	NEG	POS	POS	NEG
Dresden_01V39123	AMB	AMB	POS	POS	NEG	NEG	NEG	POS	NEG	POS	NEG	POS	NEG	NEG	POS	POS	NEG
Dresden_01V39143	POS	NEG	NEG	POS	NEG	NEG	NEG	POS	NEG	POS	NEG	POS	NEG	NEG	POS	POS	NEG
Greece 1_3680_Harmony	POS	NEG	NEG	POS	NEG	NEG	NEG	POS	NEG	POS	NEG	POS	NEG	NEG	POS	POS	NEG
**>"European Clade": UK-(**																	
ANS46 (Australia)	POS	NEG	NEG	POS	NEG	NEG	NEG	POS	NEG	POS	NEG	POS	NEG	NEG	POS	POS	NEG
LHH1 (USA)	POS	NEG	NEG	POS	NEG	NEG	NEG	POS	NEG	POS	NEG	POS	NEG	NEG	POS	POS	NEG
Dublin-DSH_AR01_0-1085	POS	NEG	AMB	POS	NEG	NEG	NEG	POS	NEG	POS	NEG	POS	NEG	NEG	POS	POS	NEG
Dublin-DSH_AR01_0-1118	POS	NEG	NEG	POS	NEG	NEG	NEG	POS	NEG	POS	NEG	POS	NEG	NEG	POS	POS	NEG
Dublin-DSH_AR15_0098	POS	NEG	NEG	POS	NEG	NEG	NEG	POS	NEG	POS	NEG	POS	NEG	NEG	POS	POS	NEG
Dublin-DSH_AR15_0104	POS	NEG	NEG	POS	NEG	NEG	NEG	POS	NEG	POS	NEG	POS	NEG	NEG	POS	POS	NEG
UK_NCTC11939_UK-1_Harmony	POS	NEG	AMB	POS	NEG	NEG	NEG	POS	NEG	POS	NEG	POS	NEG	NEG	POS	POS	NEG
**>"European Clade": BK2**																	
BK2421 (USA)	POS	NEG	NEG	POS	NEG	NEG	NEG	POS	NEG	POS	NEG	POS	NEG	NEG	POS	POS	NEG
**>"Aussi/NZ Clade", JKD6**																	
JKD6009 (Australia)	POS	NEG	NEG	POS	NEG	NEG	NEG	POS	NEG	POS	NEG	POS	NEG	NEG	POS	POS	NEG
JKD6008 (New Zealand)	POS	NEG	NEG	POS	NEG	NEG	NEG	POS	NEG	POS	NEG	POS	NEG	NEG	POS	POS	NEG
Perth_2015-S20209	POS	NEG	NEG	POS	NEG	NEG	NEG	NEG	NEG	NEG	NEG	POS	NEG	NEG	POS	POS	NEG
Perth_2015-S20305	POS	NEG	NEG	POS	NEG	NEG	NEG	POS	NEG	POS	NEG	POS	NEG	NEG	POS	POS	NEG
Perth_2015-S20603	POS	NEG	NEG	POS	NEG	NEG	NEG	POS	NEG	POS	NEG	POS	NEG	NEG	POS	POS	NEG
Perth_2015-S20607	POS	NEG	NEG	POS	NEG	NEG	NEG	POS	NEG	POS	NEG	POS	NEG	NEG	POS	POS	NEG
Perth_2015-S20608	POS	NEG	NEG	POS	NEG	NEG	NEG	POS	NEG	POS	NEG	POS	NEG	NEG	POS	POS	NEG
Perth_2015-S20610	POS	NEG	NEG	POS	NEG	NEG	NEG	POS	NEG	POS	NEG	POS	NEG	NEG	POS	POS	NEG
Perth_2015-S20613	POS	NEG	NEG	POS	NEG	NEG	NEG	POS	NEG	POS	NEG	POS	NEG	NEG	POS	POS	NEG
Perth_2015-S20617	POS	NEG	NEG	POS	NEG	NEG	NEG	POS	NEG	POS	NEG	POS	NEG	NEG	POS	POS	NEG
Perth_2015-S20622	POS	NEG	NEG	POS	NEG	NEG	NEG	POS	NEG	POS	NEG	POS	NEG	NEG	POS	POS	NEG
Perth_2015-S23619	POS	NEG	NEG	POS	NEG	NEG	NEG	POS	NEG	POS	NEG	POS	NEG	NEG	POS	POS	NEG
Perth_2015-S41210	POS	NEG	NEG	POS	NEG	NEG	NEG	POS	NEG	POS	NEG	POS	NEG	NEG	POS	POS	NEG
Perth_2015-S51001	POS	NEG	NEG	POS	NEG	NEG	NEG	POS	NEG	POS	NEG	POS	NEG	NEG	POS	POS	NEG
Perth_2015-S51003	POS	NEG	NEG	POS	NEG	NEG	NEG	POS	NEG	POS	NEG	POS	NEG	NEG	POS	POS	NEG
Perth_2015-S51006	POS	NEG	NEG	POS	NEG	NEG	NEG	POS	NEG	POS	NEG	POS	NEG	NEG	POS	POS	NEG
Perth_2015-S51007	POS	NEG	NEG	POS	NEG	NEG	NEG	POS	NEG	POS	NEG	POS	NEG	NEG	POS	POS	NEG
Perth_2015-S51008	POS	NEG	NEG	POS	NEG	NEG	NEG	POS	NEG	POS	NEG	POS	NEG	NEG	POS	POS	NEG
Perth_2015-S51014	POS	NEG	NEG	POS	NEG	NEG	NEG	NEG	NEG	NEG	NEG	NEG	NEG	NEG	POS	POS	NEG
Perth_2015-S51408	POS	NEG	NEG	POS	NEG	NEG	NEG	POS	NEG	POS	NEG	POS	NEG	NEG	POS	POS	NEG
Perth_2016-S20614	POS	NEG	NEG	POS	NEG	NEG	NEG	POS	NEG	POS	NEG	POS	NEG	NEG	POS	POS	NEG
Perth_2016-S23614	POS	NEG	NEG	POS	NEG	NEG	NEG	POS	NEG	POS	NEG	POS	NEG	NEG	POS	POS	NEG
Perth_2016-S42009	POS	NEG	NEG	POS	NEG	NEG	NEG	POS	NEG	POS	NEG	POS	NEG	NEG	POS	POS	NEG
Perth_2016-S51001	POS	NEG	NEG	POS	NEG	NEG	NEG	POS	NEG	POS	NEG	POS	NEG	NEG	POS	POS	NEG
Perth_2016-S51403	POS	NEG	NEG	POS	NEG	NEG	NEG	POS	NEG	POS	NEG	POS	NEG	NEG	POS	POS	NEG
Perth_07 RPA 20	POS	NEG	AMB	POS	NEG	NEG	NEG	POS	NEG	POS	NEG	POS	NEG	NEG	POS	POS	NEG
Perth_08-19088	POS	AMB	AMB	POS	NEG	NEG	NEG	POS	NEG	POS	NEG	POS	NEG	NEG	POS	POS	NEG
Perth_01-15351	POS	NEG	NEG	POS	NEG	NEG	NEG	POS	NEG	POS	NEG	POS	NEG	NEG	POS	POS	NEG
Perth_01-15357	POS	NEG	NEG	POS	NEG	NEG	NEG	POS	NEG	POS	NEG	POS	NEG	NEG	POS	POS	NEG
Perth_01-15419	POS	NEG	NEG	POS	NEG	NEG	NEG	POS	NEG	POS	NEG	POS	NEG	NEG	POS	POS	NEG
Perth_01-16309	POS	NEG	NEG	POS	NEG	NEG	NEG	POS	NEG	POS	NEG	POS	NEG	NEG	POS	POS	NEG
Perth_03 SNP 95	POS	AMB	AMB	POS	NEG	NEG	NEG	POS	NEG	POS	NEG	POS	NEG	NEG	POS	POS	NEG
Perth_2003_AH_47	POS	NEG	AMB	POS	NEG	NEG	NEG	POS	NEG	POS	NEG	POS	NEG	NEG	POS	POS	NEG
**>"Australian/NZ Clade":**																	
Perth_2015-S20623	POS	NEG	NEG	POS	NEG	NEG	NEG	POS	NEG	POS	NEG	POS	NEG	NEG	POS	POS	NEG
Perth_2015-S20826	POS	NEG	NEG	POS	NEG	NEG	NEG	POS	NEG	POS	NEG	POS	NEG	NEG	POS	POS	NEG
Perth_05 NH 56	POS	NEG	NEG	POS	NEG	NEG	NEG	POS	NEG	POS	NEG	POS	NEG	NEG	POS	POS	NEG
**>CC5-MRSA-III LA MRSA**																	
Delbrück 38 isolate from domestic turkey	POS	AMB	AMB	NEG	POS	NEG	NEG	POS	NEG	POS	NEG	POS	NEG	POS	NEG	POS	NEG
**> Staph. pseudintermed**																	
KM1381	NEG	NEG	NEG	NEG	NEG	NEG	NEG	NEG	NEG	NEG	NEG	NEG	NEG	NEG	NEG	NEG	NEG
**>"South-East Asian Clad**																	
M92 (Canada)	POS	NEG	NEG	POS	NEG	NEG	NEG	POS	NEG	POS	NEG	POS	NEG	NEG	POS	POS	NEG
CHI59 (China)	POS	NEG	NEG	POS	NEG	NEG	NEG	POS	NEG	POS	NEG	POS	NEG	NEG	POS	POS	NEG
CHI61 (China)	POS	NEG	NEG	POS	NEG	NEG	NEG	POS	NEG	POS	NEG	POS	NEG	NEG	POS	POS	NEG
CUHK_HK1997 (China)	POS	NEG	NEG	POS	NEG	NEG	NEG	POS	NEG	POS	NEG	POS	NEG	NEG	POS	POS	NEG
M996 (China)	POS	NEG	NEG	POS	NEG	NEG	NEG	POS	NEG	POS	NEG	POS	NEG	NEG	POS	POS	NEG
H211 (Denmark)	POS	NEG	NEG	POS	NEG	NEG	NEG	POS	NEG	POS	NEG	POS	NEG	NEG	POS	POS	NEG
H216 (Denmark)	POS	NEG	NEG	POS	NEG	NEG	NEG	POS	NEG	POS	NEG	POS	NEG	NEG	POS	POS	NEG
D71 (Germany)	POS	NEG	NEG	POS	NEG	NEG	NEG	POS	NEG	POS	NEG	POS	NEG	NEG	POS	POS	NEG
NMR05 (India)	POS	NEG	NEG	POS	NEG	NEG	NEG	POS	NEG	POS	NEG	POS	NEG	NEG	POS	POS	NEG
MAL11 (Malaysia)	POS	NEG	NEG	POS	NEG	NEG	NEG	POS	NEG	POS	NEG	POS	NEG	NEG	POS	POS	NEG
MAL9 (Malaysia)	POS	NEG	NEG	POS	NEG	NEG	NEG	POS	NEG	POS	NEG	POS	NEG	NEG	POS	POS	NEG
Na21 (Sri Lanka)	POS	NEG	NEG	POS	NEG	NEG	NEG	POS	NEG	POS	NEG	POS	NEG	NEG	POS	POS	NEG
M592 (Syria)	POS	NEG	NEG	POS	NEG	NEG	NEG	POS	NEG	POS	NEG	POS	NEG	NEG	POS	POS	NEG
H202 (Thailand)	POS	NEG	NEG	POS	NEG	NEG	NEG	POS	NEG	POS	NEG	POS	NEG	NEG	POS	POS	NEG
S102 (Thailand)	POS	NEG	NEG	POS	NEG	NEG	NEG	POS	NEG	POS	NEG	POS	NEG	NEG	POS	POS	NEG
S130 (Thailand)	POS	NEG	NEG	POS	NEG	NEG	NEG	POS	NEG	POS	NEG	POS	NEG	NEG	POS	POS	NEG
S40 (Thailand)	POS	NEG	NEG	POS	NEG	NEG	NEG	POS	NEG	POS	NEG	POS	NEG	NEG	POS	POS	NEG
S71 (Thailand)	POS	NEG	NEG	POS	NEG	NEG	NEG	POS	NEG	POS	NEG	POS	NEG	NEG	POS	POS	NEG
S87 (Thailand)	POS	NEG	NEG	POS	NEG	NEG	NEG	POS	NEG	POS	NEG	POS	NEG	NEG	POS	POS	NEG
S93 (Thailand)	POS	NEG	NEG	POS	NEG	NEG	NEG	POS	NEG	POS	NEG	POS	NEG	NEG	POS	POS	NEG
US_002 (Thailand)	POS	NEG	NEG	POS	NEG	NEG	NEG	POS	NEG	POS	NEG	POS	NEG	NEG	POS	POS	NEG
US_017 (Thailand)	POS	NEG	NEG	POS	NEG	NEG	NEG	POS	NEG	POS	NEG	POS	NEG	NEG	POS	POS	NEG
WAMC6102 (Thailand)	POS	NEG	NEG	POS	NEG	NEG	NEG	POS	NEG	POS	NEG	POS	NEG	NEG	POS	POS	NEG
NCTC_13626_TW20 (UK)	POS	NEG	NEG	POS	NEG	NEG	NEG	POS	NEG	POS	NEG	POS	NEG	NEG	POS	POS	NEG
BSAC3021 (UK)	POS	NEG	NEG	POS	NEG	NEG	NEG	POS	NEG	POS	NEG	POS	NEG	NEG	POS	POS	NEG
BSAC3046 (UK)	POS	NEG	NEG	POS	NEG	NEG	NEG	POS	NEG	POS	NEG	POS	NEG	NEG	POS	POS	NEG
1193_SAUR (USA)	POS	NEG	NEG	POS	NEG	NEG	NEG	POS	NEG	POS	NEG	POS	NEG	NEG	POS	POS	NEG
1194_SAUR (USA)	POS	NEG	NEG	POS	NEG	NEG	NEG	POS	NEG	POS	NEG	POS	NEG	NEG	POS	POS	NEG

STRAIN / ISOLATE	VIRULENCE : STAPHYLOCOCCAL SUPERANTIGEN/ENTEROTOXIN-LIKE GENES (SET/SSL)												CAPSULE- /BIOFILM-ASSOCIATED GENES				
	ssl10			ssl11				setB3		setB2		setB1	Capsule type 1	Capsule type 5	Capsule type 8	icaA/C/D	bap
	ssl10/set4	ssl10 (RF122)	ssl10/set4 (MRSA252)	ssl11/set2 (COL)	ssl11+set2(Mu50+N315)	ssl11+set2(MW2+MSSA476)	ssl11/set2 (MRSA252)	setB3	setB3 (MRSA252)	setB2	setB2 (MRSA252)	setB1					
	Staphylococcal superantigen-like protein 10			Staphylococcal superantigene-like protein 11				Staphylococcal exotoxin-like protein, second locus					Capsule type 1	Capsule type 5	Capsule type 8	intercellular adhesion protein A	Surface protein involved in biofilm formation
>"South-East Asian Clade": TW20 (Irish AR44), c[illegible]																	
1195_SAUR (USA)	POS	NEG	NEG	POS	NEG	NEG	NEG	POS	NEG	POS	NEG	POS	NEG	NEG	POS	POS	NEG
FVRH6002 (USA)	POS	NEG	NEG	POS	NEG	NEG	NEG	POS	NEG	POS	NEG	POS	NEG	NEG	POS	POS	NEG
KINW6048 (USA)	POS	NEG	NEG	POS	NEG	NEG	NEG	POS	NEG	POS	NEG	POS	NEG	NEG	POS	POS	NEG
LAMC0011 (USA)	POS	NEG	NEG	POS	NEG	NEG	NEG	POS	NEG	POS	NEG	POS	NEG	NEG	POS	POS	NEG
SJOS6053 (USA)	POS	NEG	NEG	POS	NEG	NEG	NEG	POS	NEG	POS	NEG	POS	NEG	NEG	POS	POS	NEG
SJOS6072 (USA)	POS	NEG	NEG	POS	NEG	NEG	NEG	POS	NEG	POS	NEG	POS	NEG	NEG	POS	POS	NEG
UCIM6042 (USA)	POS	NEG	NEG	POS	NEG	NEG	NEG	POS	NEG	POS	NEG	POS	NEG	NEG	POS	POS	NEG
Perth_2015-S31902	POS	NEG	NEG	NEG	NEG	NEG	NEG	POS	NEG	POS	NEG	POS	NEG	NEG	POS	POS	NEG
Perth_2015-S31904	POS	NEG	NEG	POS	NEG	NEG	NEG	POS	NEG	POS	NEG	POS	NEG	NEG	POS	POS	NEG
Perth_2015-S31913	POS	NEG	NEG	POS	NEG	NEG	NEG	POS	NEG	POS	NEG	POS	NEG	NEG	POS	POS	NEG
Perth_2015-S31918	POS	NEG	NEG	NEG	NEG	NEG	NEG	POS	NEG	POS	NEG	POS	NEG	NEG	POS	POS	NEG
Dublin-DSH_AR44_E1183	POS	NEG	NEG	POS	NEG	NEG	NEG	POS	NEG	POS	NEG	POS	NEG	NEG	POS	POS	NEG
Dublin-DSH_Unfamiliar-3_E1520	POS	NEG	NEG	POS	NEG	NEG	NEG	POS	NEG	POS	NEG	POS	NEG	NEG	POS	POS	NEG
Hong Kong_042	POS	NEG	AMB	POS	NEG	NEG	NEG	POS	NEG	POS	NEG	POS	NEG	NEG	POS	POS	NEG
Hong Kong_101	POS	NEG	AMB	POS	NEG	NEG	NEG	POS	NEG	POS	NEG	POS	NEG	NEG	POS	POS	NEG
Hong Kong_111	POS	AMB	AMB	POS	NEG	NEG	NEG	NEG	NEG	NEG	NEG	NEG	AMB	NEG	POS	POS	NEG
Hong Kong_113	POS	NEG	NEG	POS	NEG	NEG	NEG	POS	NEG	POS	NEG	POS	NEG	NEG	POS	POS	NEG
Hong Kong_115	POS	NEG	AMB	POS	NEG	NEG	NEG	POS	NEG	POS	NEG	POS	NEG	NEG	POS	POS	NEG
Hong Kong_118	POS	AMB	AMB	POS	NEG	NEG	NEG	POS	NEG	POS	NEG	POS	NEG	NEG	POS	POS	NEG
Hong Kong_125	POS	AMB	AMB	POS	NEG	NEG	NEG	POS	NEG	POS	NEG	POS	NEG	NEG	POS	POS	NEG
Hong Kong_129	POS	NEG	NEG	POS	NEG	NEG	NEG	POS	NEG	POS	NEG	POS	NEG	NEG	POS	POS	NEG
Hong Kong_133	POS	AMB	AMB	POS	NEG	NEG	NEG	NEG	NEG	NEG	NEG	NEG	NEG	NEG	NEG	POS	NEG
Kuwait_009	POS	NEG	AMB	POS	NEG	NEG	NEG	POS	NEG	POS	NEG	POS	NEG	NEG	POS	POS	NEG
Kuwait_103	POS	AMB	AMB	POS	NEG	NEG	NEG	POS	NEG	POS	NEG	POS	NEG	NEG	POS	POS	NEG
Kuwait_107	POS	AMB	AMB	POS	NEG	NEG	NEG	POS	NEG	POS	NEG	POS	NEG	NEG	POS	POS	NEG
Perth_03 AH 88	POS	NEG	NEG	POS	NEG	NEG	NEG	POS	NEG	POS	NEG	POS	NEG	NEG	POS	POS	NEG
Perth_08-16905	POS	NEG	AMB	POS	NEG	NEG	NEG	POS	NEG	POS	NEG	POS	NEG	NEG	POS	POS	NEG
Riyadh_Alfaisal-10_SICU_13_B5672_1117353	POS	AMB	AMB	POS	NEG	NEG	NEG	POS	NEG	POS	NEG	POS	NEG	NEG	POS	POS	NEG
Riyadh_SSTI_17_3508602	POS	NEG	AMB	POS	NEG	NEG	NEG	POS	NEG	POS	NEG	POS	NEG	NEG	POS	POS	NEG
Riyadh-2822088-R	POS	NEG	AMB	POS	NEG	NEG	NEG	POS	NEG	POS	NEG	POS	NEG	NEG	POS	POS	NEG
Trinidad&Tobago_2012_195 (619/12)	AMB	AMB	POS	POS	NEG	NEG	NEG	POS	NEG	POS	NEG	POS	NEG	NEG	POS	POS	NEG
Trinidad&Tobago_2012_370 (1112-52177)	AMB	AMB	POS	POS	NEG	NEG	NEG	POS	NEG	POS	NEG	POS	NEG	NEG	POS	POS	NEG
Trinidad&Tobago_2012_378 (4139)	POS	AMB	AMB	POS	NEG	NEG	NEG	POS	NEG	POS	NEG	POS	NEG	NEG	POS	POS	NEG
Trinidad&Tobago_2013_PA21	POS	NEG	AMB	POS	NEG	NEG	NEG	POS	NEG	POS	NEG	POS	NEG	NEG	POS	POS	NEG
Trinidad&Tobago_2013_PA22	POS	AMB	AMB	POS	NEG	NEG	NEG	POS	NEG	POS	NEG	POS	NEG	NEG	POS	POS	NEG
Trinidad&Tobago_2912_340 (8523/12)	POS	AMB	AMB	POS	NEG	NEG	NEG	POS	NEG	POS	NEG	POS	NEG	NEG	POS	POS	NEG
Trinidad&Tobago_MRSA2010_105	POS	AMB	AMB	POS	NEG	NEG	NEG	POS	NEG	POS	NEG	POS	NEG	NEG	POS	POS	NEG
Trinidad&Tobago_MRSA2010_108	POS	AMB	AMB	POS	NEG	NEG	NEG	POS	NEG	POS	NEG	POS	NEG	NEG	POS	POS	NEG
Trinidad&Tobago_MRSA2010_33	POS	NEG	AMB	POS	NEG	NEG	NEG	POS	NEG	POS	NEG	POS	NEG	NEG	POS	POS	NEG
Trinidad&Tobago_MRSA2010_79	POS	NEG	AMB	POS	NEG	NEG	NEG	POS	NEG	POS	NEG	POS	NEG	NEG	POS	POS	NEG
Trinidad&Tobago_MRSA2010_88	POS	NEG	AMB	POS	NEG	NEG	NEG	POS	NEG	POS	NEG	POS	NEG	NEG	POS	POS	NEG
Trinidad&Tobago_MRSA2010_97	POS	AMB	AMB	POS	NEG	NEG	NEG	POS	NEG	POS	NEG	POS	NEG	NEG	POS	POS	NEG
Trinidad&Tobago_SSTI 2012_031	POS	AMB	AMB	POS	NEG	NEG	NEG	POS	NEG	POS	NEG	POS	NEG	NEG	POS	POS	NEG
Uganda-29_17148_250811	POS	NEG	AMB	POS	NEG	NEG	NEG	POS	NEG	POS	NEG	POS	NEG	NEG	POS	POS	NEG
>"South-East Asian Clad[illegible]																	
UCIM6015	POS	NEG	NEG	POS	NEG	NEG	NEG	POS	NEG	POS	NEG	POS	NEG	NEG	POS	POS	NEG
Hong Kong_121	POS	NEG	NEG	POS	NEG	NEG	NEG	POS	NEG	POS	NEG	POS	NEG	NEG	POS	POS	NEG
>"South-East Asian Clad[illegible]																	
Z172 (Taiwan)	POS	NEG	NEG	POS	NEG	NEG	NEG	POS	NEG	POS	NEG	POS	NEG	NEG	POS	POS	NEG
Russia-19_0342_Kurgan	POS	NEG	AMB	POS	NEG	NEG	NEG	POS	NEG	POS	NEG	POS	NEG	NEG	POS	POS	NEG
>"South-East Asian Clad[illegible]																	
Perth_01-15392	POS	NEG	NEG	POS	NEG	NEG	NEG	POS	NEG	POS	NEG	POS	NEG	NEG	POS	POS	NEG
>"South-East Asian Clad[illegible]																	
XN108 (China)	POS	NEG	NEG	POS	NEG	NEG	NEG	POS	NEG	POS	NEG	POS	NEG	NEG	POS	POS	NEG
NMR09 (India)	POS	NEG	NEG	POS	NEG	NEG	NEG	POS	NEG	POS	NEG	POS	NEG	NEG	POS	POS	NEG
346 (Malaysia)	POS	NEG	NEG	POS	NEG	NEG	NEG	POS	NEG	POS	NEG	POS	NEG	NEG	POS	POS	NEG
DS_009 (Thailand)	POS	NEG	NEG	POS	NEG	NEG	NEG	POS	NEG	POS	NEG	POS	NEG	NEG	POS	POS	NEG
18ANR581005 (Ind)	POS	NEG	AMB	POS	NEG	NEG	NEG	POS	NEG	POS	NEG	POS	NEG	NEG	POS	POS	NEG
Bengalaru_ST20141405	POS	AMB	AMB	POS	NEG	NEG	NEG	POS	NEG	POS	NEG	POS	NEG	NEG	POS	POS	NEG
Bengalaru_ST20141406	POS	AMB	AMB	POS	NEG	NEG	NEG	POS	NEG	POS	NEG	POS	NEG	NEG	POS	POS	NEG
Bengalaru_ST20141417	POS	NEG	AMB	POS	NEG	NEG	NEG	POS	NEG	POS	NEG	POS	NEG	NEG	POS	POS	NEG
Hong Kong_107	POS	NEG	AMB	POS	NEG	NEG	NEG	POS	NEG	POS	NEG	POS	NEG	NEG	POS	POS	NEG
Hong Kong_12	POS	AMB	AMB	POS	NEG	NEG	NEG	POS	NEG	POS	NEG	POS	NEG	NEG	POS	POS	NEG
Hong Kong_132	POS	NEG	AMB	POS	NEG	NEG	NEG	POS	NEG	POS	NEG	POS	NEG	NEG	POS	POS	NEG
Riyadh-3028763-R	POS	NEG	AMB	POS	NEG	NEG	NEG	POS	NEG	POS	NEG	POS	NEG	NEG	POS	POS	NEG
Trinidad&Tobago_MRSA2010_94	POS	NEG	AMB	POS	NEG	NEG	NEG	POS	NEG	POS	NEG	POS	NEG	NEG	POS	POS	NEG
>"South-East Asian Clad[illegible]																	
M705 (Thailand)	POS	NEG	NEG	POS	NEG	NEG	NEG	POS	NEG	POS	NEG	POS	NEG	NEG	POS	POS	NEG
S85 (Thailand)	POS	NEG	NEG	POS	NEG	NEG	NEG	POS	NEG	POS	NEG	POS	NEG	NEG	POS	POS	NEG
Riyadh_Alfaisal-04_281014_S5076	POS	AMB	AMB	POS	NEG	NEG	NEG	POS	POS	POS	NEG	POS	NEG	NEG	POS	POS	NEG
Riyadh_Alfaisal-26_S15145_1104163	POS	AMB	AMB	POS	NEG	NEG	NEG	POS	NEG	POS	NEG	POS	NEG	NEG	POS	POS	NEG
Riyadh-2817437-W	POS	AMB	AMB	POS	NEG	NEG	NEG	POS	NEG	POS	NEG	POS	NEG	NEG	POS	POS	NEG
>"South-East Asian Clad[illegible]																	
PPUKM-775-2009 (Malaysia)	POS	NEG	NEG	POS	NEG	NEG	NEG	POS	NEG	POS	NEG	POS	NEG	NEG	POS	POS	NEG
Hong Kong_138	POS	AMB	AMB	POS	NEG	NEG	NEG	POS	NEG	POS	NEG	POS	NEG	NEG	POS	POS	NEG
>"South-East Asian Clad[illegible]																	
Kuwait_001	POS	NEG	AMB	POS	NEG	NEG	NEG	POS	NEG	POS	NEG	POS	NEG	NEG	POS	POS	NEG
Kuwait_337	POS	AMB	AMB	POS	NEG	NEG	NEG	POS	NEG	POS	NEG	POS	NEG	NEG	POS	POS	NEG
>"South-East Asian Clad[illegible]																	
CUHK_HK2007 (China)	POS	NEG	NEG	POS	NEG	NEG	NEG	NEG	NEG	NEG	NEG	NEG	NEG	NEG	POS	POS	NEG
NMR02 (India)	POS	NEG	NEG	POS	NEG	NEG	NEG	POS	NEG	POS	NEG	POS	NEG	NEG	POS	POS	NEG
VB1490 (India)	POS	NEG	NEG	POS	NEG	NEG	NEG	POS	NEG	POS	NEG	POS	NEG	NEG	POS	POS	NEG
V521 (Korea)	POS	NEG	NEG	POS	NEG	NEG	NEG	POS	NEG	POS	NEG	POS	NEG	NEG	POS	POS	NEG
HST-077 (Lebanon)	POS	NEG	NEG	POS	NEG	NEG	NEG	POS	NEG	POS	NEG	POS	NEG	NEG	POS	POS	NEG
MAL1 (Malaysia)	POS	NEG	NEG	POS	NEG	NEG	NEG	POS	NEG	POS	NEG	POS	NEG	NEG	POS	POS	NEG
MAL3 (Malaysia)	POS	NEG	NEG	POS	NEG	NEG	NEG	POS	NEG	POS	NEG	POS	NEG	NEG	POS	POS	NEG
PPUKM-261-2009 (Malaysia)	POS	NEG	NEG	POS	NEG	NEG	NEG	POS	NEG	POS	NEG	POS	NEG	NEG	POS	POS	NEG
PPUKM-332-2009 (Malaysia)	POS	NEG	NEG	POS	NEG	NEG	NEG	POS	NEG	POS	NEG	POS	NEG	NEG	POS	POS	NEG
S38 (Thailand)	POS	NEG	NEG	POS	NEG	NEG	NEG	POS	NEG	POS	NEG	POS	NEG	NEG	POS	POS	NEG
S7 (Thailand)	POS	NEG	NEG	POS	NEG	NEG	NEG	POS	NEG	POS	NEG	POS	NEG	NEG	POS	POS	NEG
US_008 (Thailand)	POS	NEG	NEG	POS	NEG	NEG	NEG	POS	NEG	POS	NEG	POS	NEG	NEG	POS	POS	NEG
US_030 (Thailand)	POS	NEG	NEG	POS	NEG	NEG	NEG	POS	NEG	POS	NEG	POS	NEG	NEG	POS	POS	NEG
M170 (Tsunami-related)	POS	NEG	NEG	POS	NEG	NEG	NEG	POS	NEG	POS	NEG	POS	NEG	NEG	POS	POS	NEG
BSAC697 (UK)	POS	NEG	NEG	POS	NEG	NEG	NEG	POS	NEG	POS	NEG	POS	NEG	NEG	POS	POS	NEG
IS-189	POS	NEG	NEG	POS	NEG	NEG	NEG	POS	NEG	POS	NEG	POS	NEG	NEG	POS	POS	NEG
MRGR3	POS	NEG	NEG	POS	NEG	NEG	NEG	POS	NEG	POS	NEG	POS	NEG	NEG	POS	POS	NEG
smg07	POS	NEG	NEG	POS	NEG	NEG	NEG	POS	NEG	POS	NEG	POS	NEG	NEG	POS	POS	NEG
AH1-AUS-EMRSA-3	POS	NEG	NEG	POS	NEG	NEG	NEG	POS	NEG	POS	NEG	POS	NEG	NEG	POS	POS	NEG
Bengalaru_ST20141403	POS	NEG	AMB	POS	NEG	NEG	NEG	POS	NEG	POS	NEG	POS	NEG	NEG	POS	POS	NEG
Finland E24_98541_Harmony	POS	AMB	AMB	POS	NEG	NEG	NEG	POS	NEG	POS	NEG	POS	NEG	NEG	POS	POS	NEG
Hong Kong_10	POS	AMB	AMB	POS	NEG	NEG	NEG	POS	NEG	POS	NEG	POS	NEG	NEG	POS	POS	NEG
Hong Kong_100	POS	NEG	AMB	POS	NEG	NEG	NEG	POS	NEG	POS	NEG	POS	NEG	NEG	POS	POS	NEG
Hong Kong_199	POS	AMB	AMB	POS	NEG	NEG	NEG	POS	NEG	POS	NEG	POS	NEG	NEG	POS	POS	NEG
Hong Kong_34	POS	AMB	AMB	POS	NEG	NEG	NEG	POS	NEG	POS	NEG	POS	NEG	NEG	POS	POS	NEG
Hong Kong_88	POS	AMB	AMB	POS	NEG	NEG	NEG	POS	NEG	POS	NEG	POS	NEG	NEG	POS	POS	NEG
Hong Kong_92	POS	AMB	AMB	POS	NEG	NEG	NEG	NEG	NEG	NEG	NEG	NEG	NEG	NEG	POS	POS	NEG
Hong Kong_94	POS	NEG	AMB	POS	NEG	NEG	NEG	POS	NEG	POS	NEG	POS	NEG	NEG	POS	POS	NEG
Kuwait_257	POS	AMB	AMB	POS	NEG	NEG	NEG	POS	NEG	POS	NEG	POS	NEG	NEG	POS	POS	NEG
Perth_01-15330	POS	NEG	NEG	POS	NEG	NEG	NEG	POS	NEG	POS	NEG	POS	NEG	NEG	POS	POS	NEG
Perth_01-15349	POS	NEG	NEG	POS	NEG	NEG	NEG	POS	NEG	POS	NEG	POS	NEG	NEG	POS	POS	NEG
Perth_01-16176	POS	NEG	NEG	POS	NEG	NEG	NEG	POS	NEG	POS	NEG	POS	NEG	NEG	POS	POS	NEG
Perth_WBG8379	POS	NEG	NEG	POS	NEG	NEG	NEG	POS	NEG	POS	NEG	POS	NEG	NEG	POS	POS	NEG
Rawalpindi_Kidney Center_56	POS	NEG	AMB	POS	NEG	NEG	NEG	POS	NEG	POS	NEG	POS	NEG	AMB	POS	POS	NEG
Riyadh_SSTI_05_3464614	POS	NEG	AMB	POS	NEG	NEG	NEG	POS	NEG	POS	NEG	POS	NEG	NEG	POS	POS	NEG
Trinidad&Tobago_2012_157	POS	AMB	AMB	POS	NEG	NEG	NEG	POS	NEG	POS	NEG	POS	NEG	NEG	POS	POS	NEG

STRAIN / ISOLATE	VIRULENCE : STAPHYLOCOCCAL SUPERANTIGEN/ENTEROTOXIN-LIKE GENES (SET/SSL)												CAPSULE- /BIOFILM-ASSOCIATED GENES				
	ssl10			ssl11				setB3		setB2		setB1	Capsule type 1	Capsule type 5	Capsule type 8	icaA/C/D	bap
	ssl10/set4	ssl10 (RF122)	ssl10/set4 (MRSA252)	ssl11/set2 (COL)	ssl11+set2(Mu50+N315)	ssl11+set2(MW2+MSSA476)	ssl11/set2 (MRSA252)	setB3	setB3 (MRSA252)	setB2	setB2 (MRSA252)	setB1					
	Staphylococcal superantigen-like protein 10			Staphylococcal superantigene-like protein 11				Staphylococcal exotoxin-like protein, second locus					Capsule type 1	Capsule type 5	Capsule type 8	intercellular adhesion protein A	Surface protein involved in biofilm formation
>"South-East Asian Clad																	
M418	POS	NEG	NEG	POS	NEG	NEG	NEG	POS	NEG	POS	NEG	POS	NEG	NEG	POS	POS	NEG
>"South-East Asian Clad																	
NMR07 (India)	POS	NEG	NEG	POS	NEG	NEG	NEG	POS	NEG	POS	NEG	POS	NEG	NEG	POS	POS	NEG
NMR08 (India)	POS	NEG	NEG	POS	NEG	NEG	NEG	POS	NEG	POS	NEG	POS	NEG	NEG	POS	POS	NEG
Bengalaru_ST20121978	POS	AMB	AMB	POS	NEG	NEG	NEG	POS	NEG	POS	NEG	POS	NEG	NEG	POS	POS	NEG
Hong Kong_89	POS	AMB	AMB	POS	NEG	NEG	NEG	POS	NEG	POS	NEG	POS	NEG	NEG	POS	POS	NEG
>"South-East Asian Clad																	
Perth_2005_AH_5	POS	NEG	AMB	POS	NEG	NEG	NEG	POS	NEG	POS	NEG	POS	NEG	NEG	POS	POS	NEG
>"South-East Asian Clad																	
Trinidad&Tobago_MRSA2010_103	POS	NEG	NEG	POS	NEG	NEG	NEG	POS	NEG	POS	NEG	POS	NEG	NEG	POS	POS	NEG
Trinidad&Tobago_2013_PA25	POS	AMB	AMB	POS	NEG	NEG	NEG	POS	NEG	POS	NEG	POS	NEG	NEG	POS	POS	NEG
Trinidad&Tobago_MRSA2010_110	POS	AMB	AMB	POS	NEG	NEG	NEG	POS	NEG	POS	NEG	POS	NEG	NEG	POS	POS	NEG
Trinidad&Tobago_SSTI 2012_111	POS	NEG	NEG	POS	NEG	NEG	NEG	POS	NEG	POS	NEG	POS	NEG	NEG	POS	POS	NEG
Trinidad&Tobago_MRSA2010_158	POS	NEG	AMB	POS	NEG	NEG	NEG	POS	NEG	POS	NEG	POS	NEG	NEG	POS	POS	NEG
>"South-East Asian Clad																	
Kuwait_122	POS	NEG	NEG	POS	NEG	NEG	NEG	POS	NEG	POS	NEG	POS	NEG	NEG	POS	POS	NEG
Kuwait_002	POS	NEG	NEG	POS	NEG	NEG	NEG	POS	NEG	POS	NEG	POS	NEG	NEG	POS	POS	NEG
Kuwait_196	POS	NEG	AMB	POS	NEG	NEG	NEG	POS	NEG	POS	NEG	POS	NEG	NEG	POS	POS	NEG
Kuwait_199	POS	NEG	AMB	POS	NEG	NEG	NEG	POS	NEG	POS	NEG	POS	NEG	NEG	POS	POS	NEG
Hong Kong_136	POS	NEG	AMB	POS	NEG	NEG	NEG	POS	NEG	POS	NEG	POS	NEG	NEG	POS	POS	NEG
>"South-East Asian Clad																	
P32 (Poland)	POS	NEG	NEG	POS	NEG	NEG	NEG	POS	NEG	POS	NEG	POS	NEG	NEG	POS	POS	NEG
>"South-East Asian Clad																	
Perth_2015-S32313	POS	NEG	NEG	POS	NEG	NEG	NEG	POS	NEG	POS	NEG	POS	NEG	NEG	POS	POS	NEG
>"hla-negative South-Ea																	
DEN907 (Denmark)	POS	NEG	NEG	POS	NEG	NEG	NEG	POS	NEG	POS	NEG	POS	NEG	NEG	POS	POS	NEG
>"hla-negative South-Ea																	
S26 (Thailand)	POS	NEG	NEG	POS	NEG	NEG	NEG	POS	NEG	POS	NEG	POS	NEG	NEG	POS	POS	NEG
S42 (Thailand)	POS	NEG	NEG	POS	NEG	NEG	NEG	POS	NEG	POS	NEG	POS	NEG	NEG	POS	POS	NEG
S97 (Thailand)	POS	NEG	NEG	POS	NEG	NEG	NEG	POS	NEG	POS	NEG	POS	NEG	NEG	POS	POS	NEG
S78 (Thailand)	POS	NEG	NEG	POS	NEG	NEG	NEG	POS	NEG	POS	NEG	POS	NEG	NEG	POS	POS	NEG
S25 (Thailand)	POS	NEG	NEG	POS	NEG	NEG	NEG	POS	NEG	POS	NEG	POS	NEG	NEG	POS	POS	NEG
S21 (Thailand)	POS	NEG	NEG	POS	NEG	NEG	NEG	POS	NEG	POS	NEG	POS	NEG	NEG	POS	POS	NEG
S106 (Thailand)	POS	NEG	NEG	POS	NEG	NEG	NEG	POS	NEG	POS	NEG	POS	NEG	NEG	POS	POS	NEG
S24 (Thailand)	POS	NEG	NEG	POS	NEG	NEG	NEG	POS	NEG	POS	NEG	POS	NEG	NEG	POS	POS	NEG
S39 (Thailand)	POS	NEG	NEG	POS	NEG	NEG	NEG	POS	NEG	POS	NEG	POS	NEG	NEG	POS	POS	NEG
S2 (Thailand)	POS	NEG	NEG	POS	NEG	NEG	NEG	POS	NEG	POS	NEG	POS	NEG	NEG	POS	POS	NEG
S81 (Thailand)	POS	NEG	NEG	POS	NEG	NEG	NEG	POS	NEG	POS	NEG	POS	NEG	NEG	POS	POS	NEG
M116 (Vietnam)	POS	NEG	NEG	POS	NEG	NEG	NEG	POS	NEG	POS	NEG	POS	NEG	NEG	POS	POS	NEG
>"Portuguese Clade": Pc																	
FFP103 (Portugal)	POS	NEG	NEG	POS	NEG	NEG	NEG	POS	NEG	POS	NEG	POS	NEG	NEG	POS	POS	NEG
HDG2 (Portugal)	POS	NEG	NEG	POS	NEG	NEG	NEG	POS	NEG	POS	NEG	POS	NEG	NEG	POS	POS	NEG
HSA10 (Portugal)	POS	NEG	NEG	POS	NEG	NEG	NEG	POS	NEG	POS	NEG	POS	NEG	NEG	POS	POS	NEG
HSA11 (Portugal)	POS	NEG	NEG	POS	NEG	NEG	NEG	POS	NEG	POS	NEG	POS	NEG	NEG	POS	POS	NEG
ICP5011 (Portugal)	POS	NEG	NEG	POS	NEG	NEG	NEG	POS	NEG	POS	NEG	POS	NEG	NEG	POS	POS	NEG
ICP5014 (Portugal)	POS	NEG	NEG	POS	NEG	NEG	NEG	POS	NEG	POS	NEG	POS	NEG	NEG	POS	POS	NEG
ICP5062 (Portugal)	POS	NEG	NEG	POS	NEG	NEG	NEG	POS	NEG	POS	NEG	POS	NEG	NEG	POS	POS	NEG
Strain 1063_ATCC 33592 FDAARGOS_3 (USA)	POS	NEG	NEG	POS	NEG	NEG	NEG	POS	NEG	POS	NEG	POS	NEG	NEG	POS	POS	NEG
	POS	NEG	AMB	POS	NEG	NEG	NEG	POS	NEG	POS	NEG	POS	NEG	NEG	POS	POS	NEG
Russia-07_0110_Kurgan	POS	NEG	NEG	POS	NEG	NEG	NEG	POS	NEG	POS	NEG	POS	NEG	NEG	POS	POS	NEG
Russia-11_0164_Kurgan	POS	NEG	AMB	POS	NEG	NEG	NEG	POS	NEG	POS	NEG	POS	NEG	NEG	POS	POS	NEG
Russia-20_0367_Kurgan	POS	NEG	AMB	POS	NEG	NEG	NEG	POS	NEG	POS	NEG	POS	NEG	NEG	POS	POS	NEG
Russia-21_0390_Chelyabinsk	POS	NEG	AMB	POS	NEG	NEG	NEG	POS	NEG	POS	NEG	POS	NEG	NEG	POS	POS	NEG
>"South American/ Mid																	
AGT1 (Argentina)	POS	NEG	NEG	POS	NEG	NEG	NEG	POS	NEG	POS	NEG	POS	NEG	NEG	POS	POS	NEG
AGT120 (Argentina)	POS	NEG	NEG	POS	NEG	NEG	NEG	POS	NEG	POS	NEG	POS	NEG	NEG	POS	POS	NEG
AGT67 (Argentina)	POS	NEG	NEG	POS	NEG	NEG	NEG	POS	NEG	POS	NEG	POS	NEG	NEG	POS	POS	NEG
AGT9 (Argentina)	POS	NEG	NEG	POS	NEG	NEG	NEG	POS	NEG	POS	NEG	POS	NEG	NEG	POS	POS	NEG
RA3 (Argentina)	POS	NEG	NEG	POS	NEG	NEG	NEG	POS	NEG	POS	NEG	POS	NEG	NEG	POS	POS	NEG
RA6 (Argentina)	POS	NEG	NEG	POS	NEG	NEG	NEG	POS	NEG	POS	NEG	POS	NEG	NEG	POS	POS	NEG
RA7 (Argentina)	POS	NEG	NEG	POS	NEG	NEG	NEG	POS	NEG	POS	NEG	POS	NEG	NEG	POS	POS	NEG
Be62 (Brazil)	POS	NEG	NEG	POS	NEG	NEG	NEG	POS	NEG	POS	NEG	POS	NEG	NEG	POS	POS	NEG
Bmb9393 (Brazil)	POS	NEG	NEG	POS	NEG	NEG	NEG	POS	NEG	POS	NEG	POS	NEG	NEG	POS	POS	NEG
BRA36 (Brazil)	POS	NEG	NEG	POS	NEG	NEG	NEG	POS	NEG	POS	NEG	POS	NEG	NEG	POS	POS	NEG
BZ48 (Brazil)	POS	NEG	NEG	POS	NEG	NEG	NEG	POS	NEG	POS	NEG	POS	NEG	NEG	POS	POS	NEG
Gv51 (Brazil)	POS	NEG	NEG	POS	NEG	NEG	NEG	POS	NEG	POS	NEG	POS	NEG	NEG	POS	POS	NEG
Gv69 (Brazil)	POS	NEG	NEG	POS	NEG	NEG	NEG	POS	NEG	POS	NEG	POS	NEG	NEG	POS	POS	NEG
Gv88 (Brazil)	POS	NEG	NEG	POS	NEG	NEG	NEG	POS	NEG	POS	NEG	POS	NEG	NEG	POS	POS	NEG
HC1335 (Brazil)	POS	NEG	NEG	POS	NEG	NEG	NEG	POS	NEG	POS	NEG	POS	NEG	NEG	POS	POS	NEG
HC1340 (Brazil)	POS	NEG	NEG	POS	NEG	NEG	NEG	POS	NEG	POS	NEG	POS	NEG	NEG	POS	POS	NEG
HC556 (Brazil)	POS	NEG	NEG	POS	NEG	NEG	NEG	POS	NEG	POS	NEG	POS	NEG	NEG	POS	POS	NEG
HU25 (Brazil)	POS	NEG	NEG	POS	NEG	NEG	NEG	POS	NEG	POS	NEG	POS	NEG	NEG	POS	POS	NEG
UB563 (Brazil)	POS	NEG	NEG	POS	NEG	NEG	NEG	POS	NEG	POS	NEG	POS	NEG	NEG	POS	POS	NEG
2A8 (Czech Republic)	POS	NEG	NEG	POS	NEG	NEG	NEG	POS	NEG	POS	NEG	POS	NEG	NEG	POS	POS	NEG
M1229 (Denmark)	POS	NEG	NEG	POS	NEG	NEG	NEG	POS	NEG	POS	NEG	POS	NEG	NEG	POS	POS	NEG
FRICAR (France)	POS	NEG	NEG	POS	NEG	NEG	NEG	POS	NEG	POS	NEG	POS	NEG	NEG	POS	POS	NEG
D90 (Germany)	POS	NEG	NEG	POS	NEG	NEG	NEG	POS	NEG	POS	NEG	POS	NEG	NEG	POS	POS	NEG
LIT68 (Lithuania)	POS	NEG	NEG	POS	NEG	NEG	NEG	POS	NEG	POS	NEG	POS	NEG	NEG	POS	POS	NEG
LIT76 (Lithuania)	POS	NEG	NEG	POS	NEG	NEG	NEG	POS	NEG	POS	NEG	POS	NEG	NEG	POS	POS	NEG
HGSA142 (Portugal)	POS	NEG	NEG	POS	NEG	NEG	NEG	POS	NEG	POS	NEG	POS	NEG	NEG	POS	POS	NEG
HGSA9 (Portugal)	POS	NEG	NEG	POS	NEG	NEG	NEG	POS	NEG	POS	NEG	POS	NEG	NEG	POS	POS	NEG
HSJ216 (Portugal)	POS	NEG	NEG	POS	NEG	NEG	NEG	POS	NEG	POS	NEG	POS	NEG	NEG	POS	POS	NEG
M278 (Portugal)	POS	NEG	NEG	POS	NEG	NEG	NEG	POS	NEG	POS	NEG	POS	NEG	NEG	POS	POS	NEG
ES26 (Spain)	POS	NEG	NEG	POS	NEG	NEG	NEG	POS	NEG	POS	NEG	POS	NEG	NEG	POS	POS	NEG
UK102 (UK)	POS	NEG	NEG	POS	NEG	NEG	NEG	POS	NEG	POS	NEG	POS	NEG	NEG	POS	POS	NEG
URU34 (Uruguay)	POS	NEG	NEG	POS	NEG	NEG	NEG	POS	NEG	POS	NEG	POS	NEG	NEG	POS	POS	NEG
IS-125	POS	NEG	NEG	POS	NEG	NEG	NEG	POS	NEG	POS	NEG	POS	NEG	NEG	POS	POS	NEG
IS-157	POS	NEG	NEG	POS	NEG	NEG	NEG	POS	NEG	POS	NEG	POS	NEG	NEG	POS	POS	NEG
Ecuador_1	POS	NEG	AMB	POS	NEG	NEG	NEG	NEG	NEG	NEG	NEG	NEG	NEG	NEG	POS	POS	NEG
Ecuador_2	POS	NEG	AMB	POS	NEG	NEG	NEG	POS	NEG	POS	NEG	POS	NEG	NEG	POS	POS	NEG
Dresden_15ANR574263	POS	AMB	AMB	POS	NEG	NEG	NEG	POS	NEG	POS	NEG	POS	NEG	NEG	POS	POS	NEG
UK-EMRSA-11	POS	AMB	AMB	POS	NEG	NEG	NEG	POS	NEG	POS	NEG	POS	NEG	NEG	POS	POS	NEG
>"South American/ Mid																	
BRA2 (Brazil)	POS	NEG	NEG	POS	NEG	NEG	NEG	POS	NEG	POS	NEG	POS	NEG	NEG	POS	POS	NEG
CHL1 (Chile)	POS	NEG	NEG	POS	NEG	NEG	NEG	POS	NEG	POS	NEG	POS	NEG	NEG	POS	POS	NEG
CHL151 (Chile)	POS	NEG	NEG	POS	NEG	NEG	NEG	POS	NEG	POS	NEG	POS	NEG	NEG	POS	POS	NEG
UCL378 (Chile)	POS	NEG	NEG	POS	NEG	NEG	NEG	POS	NEG	POS	NEG	POS	NEG	NEG	POS	POS	NEG
UP72 (Peru)	POS	NEG	NEG	POS	NEG	NEG	NEG	POS	NEG	POS	NEG	POS	NEG	NEG	POS	POS	NEG
UP81 (Peru)	POS	NEG	NEG	POS	NEG	NEG	NEG	POS	NEG	POS	NEG	POS	NEG	NEG	POS	POS	NEG
>"South American/ Mid																	
UP1073 (Peru)	POS	NEG	NEG	POS	NEG	NEG	NEG	POS	NEG	POS	NEG	POS	NEG	NEG	POS	POS	NEG

STRAIN / ISOLATE	VIRULENCE : STAPHYLOCOCCAL SUPERANTIGEN/ENTEROTOXIN-LIKE GENES (SET/SSL)												CAPSULE- /BIOFILM-ASSOCIATED GENES				
	ssl10			ssl11				setB3		setB2		setB1	Capsule type 1	Capsule type 5	Capsule type 8	icaA/C/D	bap
	ssl10/set4	ssl10 (RF122)	ssl10/set4 (MRSA252)	ssl11/set2 (COL)	ssl11+set2(Mu50+N315)	ssl11+set2(MW2+MSSA476)	ssl11/set2 (MRSA252)	setB3	setB3 (MRSA252)	setB2	setB2 (MRSA252)	setB1					
	Staphylococcal superantigen-like protein 10			Staphylococcal superantigene-like protein 11				Staphylococcal exotoxin-like protein, second locus					Capsule type 1	Capsule type 5	Capsule type 8	intercellular adhesion protein A	Surface protein involved in biofilm formation
>"South American/ Mid																	
LIT89 (Lithuania)	POS	NEG	NEG	POS	NEG	NEG	NEG	POS	NEG	POS	NEG	POS	NEG	NEG	POS	POS	NEG
MRSA_PR1 (Malaysia)	POS	NEG	NEG	POS	NEG	NEG	NEG	POS	NEG	POS	NEG	POS	NEG	NEG	POS	POS	NEG
Riyadh-2888905-R	POS	NEG	AMB	POS	NEG	NEG	NEG	POS	NEG	POS	NEG	POS	NEG	NEG	POS	POS	NEG
Riyadh-2888915-BC	POS	NEG	NEG	POS	NEG	NEG	NEG	POS	NEG	POS	NEG	POS	NEG	NEG	POS	POS	NEG
Riyadh-2793706-R	POS	NEG	AMB	POS	NEG	NEG	NEG	POS	NEG	POS	NEG	POS	NEG	NEG	POS	POS	NEG
>"South American/ Mid																	
Riyadh_SSTI_18_3502925	POS	AMB	AMB	POS	NEG	NEG	NEG	POS	NEG	POS	NEG	POS	NEG	NEG	POS	POS	NEG
Riyadh-2822825-W	POS	AMB	AMB	POS	NEG	AMB	NEG	POS	NEG	POS	NEG	POS	NEG	NEG	POS	POS	NEG
>"South American/ Mid																	
UK-EMRSA-9	POS	NEG	NEG	POS	NEG	NEG	NEG	POS	NEG	POS	NEG	POS	NEG	NEG	POS	POS	NEG
>"South American/ Mid																	
Lome_HT20020815	POS	NEG	AMB	POS	NEG	NEG	NEG	POS	NEG	POS	NEG	POS	NEG	NEG	POS	POS	NEG
>"South American/ Mid																	
NA32 (Denmark)	POS	NEG	NEG	POS	NEG	NEG	NEG	POS	NEG	POS	NEG	POS	NEG	NEG	POS	POS	NEG
H24 (Egypt)	POS	NEG	NEG	POS	NEG	NEG	NEG	POS	NEG	POS	NEG	POS	NEG	NEG	POS	POS	NEG
ATCC BAA-39 (=HUSA304) (Hungary)	POS	NEG	NEG	POS	NEG	NEG	NEG	POS	NEG	POS	NEG	POS	NEG	NEG	POS	POS	NEG
HUSA304 (Hungary)	POS	NEG	NEG	POS	NEG	NEG	NEG	POS	NEG	POS	NEG	POS	NEG	NEG	POS	POS	NEG
HU106 (Hungary)	POS	NEG	NEG	POS	NEG	NEG	NEG	POS	NEG	POS	NEG	POS	NEG	NEG	POS	POS	NEG
BSAC27 (UK)	POS	NEG	NEG	POS	NEG	NEG	NEG	POS	NEG	POS	NEG	POS	NEG	NEG	POS	POS	NEG
NCTC13131, UK-EMRSA-4 (UK)	POS	NEG	NEG	POS	NEG	NEG	NEG	POS	NEG	POS	NEG	POS	NEG	NEG	POS	POS	NEG
	POS	AMB	AMB	POS	NEG	NEG	NEG	POS	NEG	POS	NEG	POS	NEG	NEG	POS	POS	NEG
Algiers_HT20040080	POS	NEG	AMB	POS	NEG	NEG	NEG	POS	NEG	POS	NEG	POS	NEG	NEG	POS	POS	NEG
Dublin-DSH_AR09_0.0066	POS	NEG	AMB	POS	NEG	NEG	NEG	POS	NEG	POS	NEG	POS	NEG	NEG	POS	POS	NEG
Dublin-DSH_AR09_0-0065	POS	NEG	NEG	POS	NEG	NEG	NEG	POS	NEG	POS	NEG	POS	NEG	NEG	POS	POS	NEG
Dublin-DSH_Phenotype-III_84	POS	NEG	NEG	POS	NEG	NEG	NEG	POS	NEG	POS	NEG	POS	NEG	NEG	POS	POS	NEG
Hong Kong_130	POS	NEG	NEG	POS	NEG	NEG	NEG	POS	NEG	POS	NEG	POS	NEG	NEG	POS	POS	NEG
Kuwait_018	POS	NEG	NEG	POS	NEG	NEG	NEG	POS	NEG	POS	NEG	POS	NEG	NEG	POS	POS	NEG
Perth_08-17726	POS	NEG	AMB	POS	NEG	NEG	NEG	POS	NEG	POS	NEG	POS	NEG	NEG	POS	POS	NEG
Riyadh_Alfaisal/KKKSUH_86_MRS-14-279	POS	NEG	NEG	POS	NEG	NEG	NEG	POS	NEG	POS	NEG	POS	NEG	NEG	POS	POS	NEG
Riyadh_Alfaisal-04_Z3861_831588	POS	AMB	AMB	POS	NEG	NEG	NEG	POS	NEG	POS	NEG	POS	NEG	NEG	POS	POS	NEG
Riyadh_Alfaisal-30_S15724_1108013	POS	NEG	AMB	POS	NEG	NEG	NEG	POS	NEG	POS	NEG	POS	NEG	NEG	POS	POS	NEG
Riyadh_Alfaisal-6_22A_13_B3992_397721	POS	AMB	AMB	POS	NEG	NEG	NEG	POS	NEG	POS	NEG	POS	NEG	NEG	POS	POS	NEG
Riyadh_SSTI_52_3615482	POS	NEG	AMB	POS	NEG	NEG	NEG	POS	NEG	POS	NEG	POS	NEG	NEG	POS	POS	NEG
Riyadh-2817276-2	POS	AMB	AMB	POS	NEG	NEG	NEG	POS	NEG	POS	NEG	POS	NEG	NEG	POS	POS	NEG
Riyadh-2891670-W	POS	AMB	AMB	POS	NEG	NEG	NEG	POS	NEG	POS	NEG	POS	NEG	NEG	POS	POS	NEG
Riyadh-3006920-W	POS	NEG	AMB	POS	NEG	NEG	NEG	POS	NEG	POS	NEG	POS	NEG	NEG	POS	POS	NEG
Riyadh-R2567782	POS	NEG	AMB	POS	NEG	NEG	NEG	POS	NEG	POS	NEG	POS	NEG	NEG	POS	POS	NEG
Russia-18_0252_Krasnoyarsk_SK2	POS	AMB	AMB	POS	NEG	NEG	NEG	POS	NEG	POS	NEG	POS	NEG	NEG	POS	POS	NEG
UK-EMRSA-7	POS	NEG	AMB	POS	NEG	NEG	NEG	POS	NEG	POS	NEG	POS	NEG	NEG	POS	POS	NEG
>"South American/ Mid																	
Dublin-DSH_AR23_0073	POS	NEG	NEG	POS	NEG	NEG	NEG	POS	NEG	POS	NEG	POS	NEG	NEG	POS	POS	NEG
>Related to "South Ame																	
MRSA-OC3 (Russia)	POS	NEG	NEG	POS	NEG	NEG	NEG	POS	NEG	POS	NEG	POS	NEG	NEG	POS	POS	NEG
Russia-12_0176_Krasnoyarsk	POS	NEG	AMB	POS	NEG	NEG	NEG	POS	NEG	POS	NEG	POS	NEG	NEG	POS	POS	NEG
Russia-13_0180_Krasnoyarsk	POS	AMB	AMB	POS	NEG	NEG	NEG	POS	NEG	POS	NEG	POS	NEG	NEG	POS	POS	NEG
Russia-16_0249_Krasnoyarsk	POS	AMB	AMB	POS	NEG	NEG	NEG	POS	NEG	POS	NEG	POS	NEG	NEG	POS	POS	NEG
Russia-17_0250_Krasnoyarsk	POS	NEG	AMB	POS	NEG	NEG	NEG	POS	NEG	POS	NEG	POS	NEG	NEG	POS	POS	NEG
>Related to "South Ame																	
URU110 (Uruguay)	POS	NEG	NEG	POS	NEG	NEG	NEG	POS	NEG	POS	NEG	POS	NEG	NEG	POS	POS	NEG
>Related to "South Ame																	
DS_014 (Thailand)	POS	NEG	NEG	POS	NEG	NEG	NEG	POS	NEG	POS	NEG	POS	NEG	NEG	POS	POS	NEG
>Unassigned Middle Eas																	
Frankfurt_Oder_0490031797 (Libya)	POS	AMB	AMB	POS	NEG	NEG	NEG	POS	NEG	POS	NEG	POS	NEG	NEG	POS	POS	NEG
Kuwait_192	POS	AMB	AMB	POS	NEG	NEG	NEG	POS	NEG	POS	NEG	POS	NEG	NEG	POS	POS	NEG
Riyadh-9	POS	AMB	AMB	POS	NEG	NEG	NEG	POS	NEG	POS	NEG	POS	NEG	NEG	POS	POS	NEG
Russia-24_0407_Moscow	POS	NEG	NEG	POS	NEG	NEG	NEG	POS	NEG	POS	NEG	POS	NEG	NEG	POS	POS	NEG

STRAIN / ISOLATE	ADHAESION FACTORS / GENES ENCODING MICROBIAL SURFACE COMPONENTS RECOGNIZING ADHESIVE MATRIX MOLECULES (MSCRAMM GENES)																	
	bbp							clfA					clfB					cna
	bbp	bbp (cons)	bbp (COL+MW2)	bbp (MRSA252)	bbp (Mu50)	bbp (RF122)	bbp (ST45)	clfA	clfA (cons)	clfA (COL+RF122)	clfA (MRSA252)	clfA (Mu50+MW2)	clfB	clfB (cons)	clfB (COL+Mu50)	clfB (MW2)	clfB (RF122)	cna
	Bone sialoprotein-binding protein							Clumping factor A					Clumping factor B					Collagen-binding adhesin
**>"Eurasian Clade": TUR1**																		
TUR1 (Turkey)	POS	POS	POS	NEG	NEG	NEG	NEG	POS	POS	POS	NEG	AMB	POS	POS	NEG	NEG	NEG	POS
TUR9 (Turkey)	POS	POS	POS	NEG	NEG	NEG	NEG	POS	POS	POS	NEG	AMB	POS	POS	NEG	NEG	NEG	POS
**>"Eurasian Clade" : T013**																		
T0131 (China)	POS	POS	POS	NEG	NEG	NEG	NEG	POS	POS	POS	NEG	AMB	POS	POS	NEG	NEG	NEG	POS
MU4 (Turkey)	POS	POS	POS	NEG	NEG	NEG	NEG	POS	POS	POS	NEG	AMB	POS	POS	NEG	NEG	NEG	POS
DEU11 (Turkey)	POS	POS	POS	NEG	NEG	NEG	NEG	POS	POS	POS	NEG	AMB	POS	POS	NEG	NEG	NEG	POS
DEU16 (Turkey)	POS	POS	POS	NEG	NEG	NEG	NEG	POS	POS	POS	NEG	AMB	POS	POS	NEG	NEG	NEG	POS
Romania_Iasi_MRSA-07_54	POS	POS	POS	NEG	NEG	NEG	AMB	POS	POS	POS	AMB	AMB	POS	POS	NEG	NEG	POS	POS
**>"Eurasian Clade": arsC-**																		
Romania_Iasi_MRSA-05_46	POS	POS	POS	NEG	NEG	NEG	NEG	POS	POS	POS	AMB	AMB	POS	POS	NEG	NEG	NEG	POS
Romania_Iasi_MRSA-06_47	POS	POS	POS	NEG	NEG	NEG	NEG	POS	POS	POS	AMB	AMB	POS	POS	NEG	NEG	POS	POS
**>"Eurasian Clade": 16K/**																		
CN79 (China)	POS	POS	POS	NEG	NEG	NEG	NEG	POS	POS	POS	NEG	AMB	POS	POS	NEG	NEG	NEG	POS
CUHK_BJ2002 (China)	POS	POS	POS	NEG	NEG	NEG	NEG	POS	POS	POS	NEG	AMB	POS	POS	NEG	NEG	NEG	POS
CUHK_BJ2007 (China)	POS	POS	POS	NEG	NEG	NEG	NEG	POS	POS	POS	NEG	AMB	POS	POS	NEG	NEG	NEG	POS
3HK (Czech Republic)	POS	POS	POS	NEG	NEG	NEG	NEG	POS	POS	POS	NEG	AMB	POS	POS	NEG	NEG	NEG	POS
HU109 (Hungary)	POS	POS	POS	NEG	NEG	NEG	NEG	POS	POS	POS	NEG	AMB	POS	POS	NEG	NEG	NEG	POS
HUR18 (Hungary)	POS	POS	POS	NEG	NEG	NEG	NEG	POS	POS	POS	NEG	AMB	POS	POS	NEG	NEG	NEG	POS
H482 (Romania)	POS	POS	POS	NEG	NEG	NEG	NEG	POS	POS	POS	NEG	AMB	POS	POS	NEG	NEG	NEG	POS
16K (Russia)	POS	POS	POS	NEG	NEG	NEG	NEG	POS	POS	POS	NEG	AMB	POS	POS	NEG	NEG	NEG	POS
DEU10 (Turkey)	POS	POS	POS	NEG	NEG	NEG	NEG	POS	POS	POS	NEG	AMB	POS	POS	NEG	NEG	NEG	POS
DEU12 (Turkey)	POS	POS	POS	NEG	NEG	NEG	NEG	POS	POS	POS	NEG	AMB	POS	POS	NEG	NEG	NEG	POS
DEU14 (Turkey)	POS	POS	POS	NEG	NEG	NEG	NEG	POS	POS	POS	NEG	AMB	POS	POS	NEG	NEG	NEG	POS
DEU15 (Turkey)	POS	POS	POS	NEG	NEG	NEG	NEG	POS	POS	POS	NEG	AMB	POS	POS	NEG	NEG	NEG	POS
DEU17 (Turkey)	POS	POS	POS	NEG	NEG	NEG	NEG	POS	POS	POS	NEG	AMB	POS	POS	NEG	NEG	NEG	POS
DEU19 (Turkey)	POS	POS	POS	NEG	NEG	NEG	NEG	POS	POS	POS	NEG	AMB	POS	POS	NEG	NEG	NEG	POS
DEU2 (Turkey)	POS	POS	POS	NEG	NEG	NEG	NEG	POS	POS	POS	NEG	AMB	POS	POS	NEG	NEG	NEG	POS
DEU20 (Turkey)	POS	POS	POS	NEG	NEG	NEG	NEG	POS	POS	POS	NEG	AMB	POS	POS	NEG	NEG	NEG	POS
DEU23 (Turkey)	POS	POS	POS	NEG	NEG	NEG	NEG	POS	POS	POS	NEG	AMB	POS	POS	NEG	NEG	NEG	POS
DEU3 (Turkey)	POS	POS	POS	NEG	NEG	NEG	NEG	POS	POS	POS	NEG	AMB	POS	POS	NEG	NEG	NEG	POS
DEU5 (Turkey)	POS	POS	POS	NEG	NEG	NEG	NEG	POS	POS	POS	NEG	AMB	POS	POS	NEG	NEG	NEG	POS
DEU6 (Turkey)	POS	POS	POS	NEG	NEG	NEG	NEG	POS	POS	POS	NEG	AMB	POS	POS	NEG	NEG	NEG	POS
DEU8 (Turkey)	POS	POS	POS	NEG	NEG	NEG	NEG	POS	POS	POS	NEG	AMB	POS	POS	NEG	NEG	NEG	POS
HU11 (Turkey)	POS	POS	POS	NEG	NEG	NEG	NEG	POS	POS	POS	NEG	AMB	POS	POS	NEG	NEG	NEG	POS
HU13 (Turkey)	POS	POS	POS	NEG	NEG	NEG	NEG	POS	POS	POS	NEG	AMB	POS	POS	NEG	NEG	NEG	POS
HU14 (Turkey)	POS	POS	POS	NEG	NEG	NEG	NEG	POS	POS	POS	NEG	AMB	POS	POS	NEG	NEG	NEG	POS
HU15 (Turkey)	POS	POS	POS	NEG	NEG	NEG	NEG	POS	POS	POS	NEG	AMB	POS	POS	NEG	NEG	NEG	POS
HU16 (Turkey)	POS	POS	POS	NEG	NEG	NEG	NEG	POS	POS	POS	NEG	AMB	POS	POS	NEG	NEG	NEG	POS
HU17 (Turkey)	POS	POS	POS	NEG	NEG	NEG	NEG	POS	POS	POS	NEG	AMB	POS	POS	NEG	NEG	NEG	POS
HU21 (Turkey)	POS	POS	POS	NEG	NEG	NEG	NEG	POS	POS	POS	NEG	AMB	POS	POS	NEG	NEG	NEG	POS
HU23 (Turkey)	POS	POS	POS	NEG	NEG	NEG	NEG	POS	POS	POS	NEG	AMB	POS	POS	NEG	NEG	NEG	POS
HU26 (Turkey)	POS	POS	POS	NEG	NEG	NEG	NEG	POS	POS	POS	NEG	AMB	POS	POS	NEG	NEG	NEG	POS
HU4 (Turkey)	POS	POS	POS	NEG	NEG	NEG	NEG	POS	POS	POS	NEG	AMB	POS	POS	NEG	NEG	NEG	POS
HU5 (Turkey)	POS	POS	POS	NEG	NEG	NEG	NEG	POS	POS	POS	NEG	AMB	POS	POS	NEG	NEG	NEG	POS
HU6 (Turkey)	POS	POS	POS	NEG	NEG	NEG	NEG	POS	POS	POS	NEG	AMB	POS	POS	NEG	NEG	NEG	POS
HU7 (Turkey)	POS	POS	POS	NEG	NEG	NEG	NEG	POS	POS	POS	NEG	AMB	POS	POS	NEG	NEG	NEG	POS
HU8 (Turkey)	POS	POS	POS	NEG	NEG	NEG	NEG	POS	POS	POS	NEG	AMB	POS	POS	NEG	NEG	NEG	POS
HU9 (Turkey)	POS	POS	POS	NEG	NEG	NEG	NEG	POS	POS	POS	NEG	AMB	POS	POS	NEG	NEG	NEG	POS
IU1 (Turkey)	POS	POS	POS	NEG	NEG	NEG	NEG	POS	POS	POS	NEG	AMB	POS	POS	NEG	NEG	NEG	POS
IU10 (Turkey)	POS	POS	POS	NEG	NEG	NEG	NEG	POS	POS	POS	NEG	AMB	POS	POS	NEG	NEG	NEG	POS
IU11 (Turkey)	POS	POS	POS	NEG	NEG	NEG	NEG	POS	POS	POS	NEG	AMB	POS	POS	NEG	NEG	NEG	POS
IU12 (Turkey)	POS	POS	POS	NEG	NEG	NEG	NEG	POS	POS	POS	NEG	AMB	POS	POS	NEG	NEG	NEG	POS
IU13 (Turkey)	POS	POS	POS	NEG	NEG	NEG	NEG	POS	POS	POS	NEG	AMB	POS	POS	NEG	NEG	NEG	POS
IU15 (Turkey)	POS	POS	POS	NEG	NEG	NEG	NEG	POS	POS	POS	NEG	AMB	POS	POS	NEG	NEG	NEG	POS
IU18 (Turkey)	POS	POS	POS	NEG	NEG	NEG	NEG	POS	POS	POS	NEG	AMB	POS	POS	NEG	NEG	NEG	POS
IU19 (Turkey)	POS	POS	POS	NEG	NEG	NEG	NEG	POS	POS	POS	NEG	AMB	POS	POS	NEG	NEG	NEG	POS
IU2 (Turkey)	POS	POS	POS	NEG	NEG	NEG	NEG	POS	POS	POS	NEG	AMB	POS	POS	NEG	NEG	NEG	POS
IU4 (Turkey)	POS	POS	POS	NEG	NEG	NEG	NEG	POS	POS	POS	NEG	AMB	POS	POS	NEG	NEG	NEG	POS
IU6 (Turkey)	POS	POS	POS	NEG	NEG	NEG	NEG	POS	POS	POS	NEG	AMB	POS	POS	NEG	NEG	NEG	POS
IU7 (Turkey)	POS	POS	POS	NEG	NEG	NEG	NEG	POS	POS	POS	NEG	AMB	POS	POS	NEG	NEG	NEG	POS
IU9 (Turkey)	POS	POS	POS	NEG	NEG	NEG	NEG	POS	POS	POS	NEG	AMB	POS	POS	NEG	NEG	NEG	POS
MU1 (Turkey)	POS	POS	POS	NEG	NEG	NEG	NEG	POS	POS	POS	NEG	AMB	POS	POS	NEG	NEG	NEG	POS
MU10 (Turkey)	POS	POS	POS	NEG	NEG	NEG	NEG	POS	POS	POS	NEG	AMB	POS	POS	NEG	NEG	NEG	POS
MU20 (Turkey)	POS	POS	POS	NEG	NEG	NEG	NEG	POS	POS	POS	NEG	AMB	POS	POS	NEG	NEG	NEG	POS
Mu3 (Turkey)	POS	POS	POS	NEG	NEG	NEG	NEG	POS	POS	POS	NEG	AMB	POS	POS	NEG	NEG	NEG	POS
MU5 (Turkey)	POS	POS	POS	NEG	NEG	NEG	NEG	POS	POS	POS	NEG	AMB	POS	POS	NEG	NEG	NEG	POS
MU6 (Turkey)	POS	POS	POS	NEG	NEG	NEG	NEG	POS	POS	POS	NEG	AMB	POS	POS	NEG	NEG	NEG	POS
MU7 (Turkey)	POS	POS	POS	NEG	NEG	NEG	NEG	POS	POS	POS	NEG	AMB	POS	POS	NEG	NEG	NEG	POS
TUR27 (Turkey)	POS	POS	POS	NEG	NEG	NEG	NEG	POS	POS	POS	NEG	AMB	POS	POS	NEG	NEG	NEG	POS
NCTR 32S	POS	POS	POS	NEG	NEG	NEG	NEG	POS	POS	POS	NEG	AMB	POS	POS	NEG	NEG	NEG	POS
SA02_A4 = Russia-06_0085_Moscow	POS	POS	POS	NEG	NEG	NEG	NEG	POS	POS	POS	NEG	AMB	POS	POS	NEG	NEG	NEG	POS
	POS	POS	POS	NEG	AMB	NEG	NEG	POS	POS	POS	AMB	AMB	POS	POS	NEG	NEG	POS	POS
Dresden_08V35987 (Turkey)	POS	POS	POS	NEG	NEG	NEG	NEG	POS	POS	POS	AMB	AMB	POS	POS	NEG	NEG	AMB	POS
Dresden_17ANR577152	POS	POS	POS	NEG	NEG	NEG	NEG	POS	POS	POS	AMB	POS	POS	POS	NEG	NEG	AMB	POS
Dresden_17ANR580374 (Makedonia)	POS	POS	POS	NEG	NEG	NEG	NEG	POS	POS	POS	NEG	POS	POS	POS	NEG	NEG	AMB	POS
Hong Kong_69-II	POS	POS	POS	NEG	AMB	NEG	NEG	AMB	POS	POS	AMB	AMB	AMB	POS	NEG	NEG	POS	POS
Hong Kong_93	POS	POS	POS	NEG	NEG	NEG	AMB	AMB	POS	POS	AMB	AMB	AMB	POS	NEG	NEG	POS	POS
Rawalpindi_Kidney Center_03_SK1	POS	POS	POS	NEG	AMB	NEG	NEG	POS	POS	POS	AMB	AMB	POS	POS	NEG	NEG	POS	POS
Rawalpindi_Kidney Center_08	POS	POS	POS	NEG	AMB	NEG	NEG	POS	POS	POS	AMB	AMB	POS	POS	NEG	NEG	POS	POS
Rawalpindi_Kidney Center_10	POS	POS	POS	NEG	AMB	NEG	AMB	POS	POS	POS	AMB	AMB	POS	POS	NEG	NEG	POS	POS
Rawalpindi_Kidney Center_19	POS	POS	POS	NEG	AMB	AMB	AMB	POS	POS	AMB	AMB	POS	POS	POS	NEG	NEG	POS	POS
Rawalpindi_Kidney Center_50_SK1	POS	POS	POS	NEG	AMB	NEG	NEG	POS	POS	POS	AMB	AMB	POS	POS	NEG	NEG	POS	POS
Romania_Iasi_BC-49_430	POS	POS	POS	NEG	NEG	NEG	NEG	POS	POS	AMB	AMB	POS	POS	POS	NEG	NEG	POS	POS
Romania_Iasi_MRSA_284	POS	POS	POS	NEG	NEG	NEG	NEG	POS	POS	POS	AMB	AMB	POS	POS	NEG	NEG	POS	POS
Romania_Iasi_MRSA-32_318	POS	POS	POS	NEG	NEG	NEG	NEG	POS	POS	POS	AMB	AMB	POS	POS	NEG	NEG	AMB	POS
Romania_Iasi_MRSA-54_430	POS	POS	POS	NEG	AMB	NEG	NEG	POS	POS	POS	AMB	AMB	POS	POS	NEG	NEG	POS	POS
Romania_Iasi_SSTI-01_2	POS	POS	POS	NEG	AMB	NEG	NEG	POS	POS	POS	AMB	AMB	POS	POS	NEG	NEG	POS	POS
Romania_Iasi_SSTI-10_101	POS	POS	POS	NEG	NEG	NEG	NEG	POS	POS	POS	AMB	AMB	POS	POS	NEG	NEG	NEG	POS
Romania_Iasi_SSTI-11_106	POS	POS	POS	NEG	AMB	NEG	NEG	POS	POS	POS	AMB	POS	POS	POS	NEG	NEG	POS	POS
Russia-01_0001_SaintPetersburg	POS	POS	POS	NEG	AMB	NEG	NEG	POS	POS	POS	AMB	AMB	POS	POS	NEG	NEG	POS	POS
Russia-02_0004_SaintPetersburg	POS	POS	POS	NEG	AMB	NEG	NEG	POS	POS	POS	AMB	AMB	POS	POS	NEG	NEG	POS	POS
Russia-03_0057_SaintPetersburg	POS	POS	POS	NEG	NEG	NEG	NEG	POS	POS	POS	NEG	AMB	POS	POS	NEG	NEG	NEG	POS
Russia-04_0076_Moscow	POS	POS	POS	NEG	AMB	NEG	NEG	POS	POS	POS	AMB	AMB	POS	POS	NEG	NEG	POS	POS
Russia-05_0078_Moscow	POS	POS	POS	NEG	AMB	NEG	NEG	POS	POS	POS	AMB	AMB	POS	POS	NEG	NEG	POS	POS
Russia-08_0135_Moscow	POS	POS	POS	NEG	AMB	AMB	NEG	POS	POS	POS	AMB	AMB	POS	POS	NEG	NEG	POS	POS
Russia-09_0150_Kurgan	POS	POS	POS	NEG	AMB	NEG	NEG	POS	POS	POS	AMB	AMB	POS	POS	NEG	NEG	POS	POS
Russia-10_0162_Kurgan	POS	POS	POS	NEG	AMB	NEG	NEG	POS	POS	POS	AMB	AMB	POS	POS	NEG	NEG	POS	POS
Russia-14_0184_Moscow	POS	POS	POS	NEG	NEG	NEG	NEG	POS	POS	POS	AMB	AMB	POS	POS	NEG	NEG	POS	POS
Russia-15_0232_SaintPetersburg	POS	POS	POS	NEG	AMB	NEG	NEG	POS	POS	POS	AMB	AMB	POS	POS	NEG	NEG	POS	POS
Russia-22_0391_Chelyabinsk	POS	POS	POS	NEG	AMB	NEG	NEG	POS	POS	POS	AMB	AMB	POS	POS	NEG	NEG	POS	POS
Russia-23_0392_Chelyabinsk	POS	POS	POS	NEG	AMB	NEG	NEG	POS	POS	POS	AMB	AMB	POS	POS	NEG	NEG	POS	POS
**>"Eurasian Clade" : IU17**																		
IU17 (Turkey)	POS	POS	POS	NEG	NEG	NEG	NEG	POS	POS	POS	NEG	AMB	POS	POS	NEG	NEG	NEG	POS
IU20 (Turkey)	POS	POS	POS	NEG	NEG	NEG	NEG	POS	POS	POS	NEG	AMB	POS	POS	NEG	NEG	NEG	POS

STRAIN / ISOLATE	ADHAESION FACTORS / GENES ENCODING MICROBIAL SURFACE COMPONENTS RECOGNIZING ADHESIVE MATRIX MOLECULES (MSCRAMM GENES)																	
	bbp							clfA					clfB					cna
	bbp	bbp (cons)	bbp (COL+MW2)	bbp (MRSA252)	bbp (Mu50)	bbp (RF122)	bbp (ST45)	clfA	clfA (cons)	clfA (COL+RF122)	clfA (MRSA252)	clfA (Mu50+MW2)	clfB	clfB (cons)	clfB (COL+Mu50)	clfB (MW2)	clfB (RF122)	cna
	Bone sialoprotein-binding protein							Clumping factor A					Clumping factor B					Collagen-binding adhesin
**>"Eurasian Clade": Spora**																		
Dresden_16ANR581769	POS	POS	POS	NEG	NEG	NEG	NEG	POS	POS	POS	NEG	NEG	POS	POS	NEG	NEG	NEG	POS
Dresden_17ANR578769	POS	POS	POS	NEG	NEG	NEG	NEG	POS	POS	POS	AMB	AMB	POS	POS	NEG	NEG	AMB	POS
**>"Eurasian Clade": Kuwa**																		
Kuwait_306	POS	POS	POS	NEG	AMB	NEG	NEG	POS	POS	POS	AMB	AMB	POS	POS	NEG	NEG	POS	POS
Kuwait_301	POS	POS	POS	NEG	AMB	NEG	NEG	POS	POS	AMB	AMB	POS	POS	POS	NEG	NEG	POS	POS
**>"European Clade": "Gre**																		
GRE18 (Greece)	POS	POS	POS	NEG	NEG	NEG	NEG	POS	POS	POS	NEG	AMB	POS	POS	NEG	NEG	NEG	POS
GRE317 (Greece)	POS	POS	POS	NEG	NEG	NEG	NEG	POS	POS	POS	NEG	AMB	POS	POS	NEG	NEG	NEG	POS
GRE4 (Greece)	POS	POS	POS	NEG	NEG	NEG	NEG	POS	POS	POS	NEG	AMB	POS	POS	NEG	NEG	NEG	POS
R35 (USA)	POS	POS	POS	NEG	NEG	NEG	NEG	POS	POS	POS	NEG	AMB	POS	POS	NEG	NEG	NEG	POS
Casablanca_HT20060548	POS	POS	POS	NEG	AMB	NEG	NEG	POS	POS	POS	AMB	AMB	POS	POS	NEG	NEG	POS	POS
Casablanca_HT20060550	POS	POS	POS	NEG	AMB	NEG	NEG	POS	POS	POS	AMB	AMB	POS	POS	NEG	NEG	POS	POS
Dresden_01V34572 (Greece)	POS	POS	POS	NEG	NEG	NEG	NEG	POS	POS	POS	AMB	POS	POS	POS	NEG	NEG	NEG	POS
Dresden_01V36469	POS	POS	POS	NEG	NEG	NEG	NEG	POS	POS	POS	NEG	AMB	POS	POS	NEG	NEG	AMB	POS
Dresden_01V39123	POS	POS	POS	NEG	AMB	NEG	AMB	POS	POS	POS	AMB	AMB	POS	POS	NEG	NEG	POS	POS
Dresden_01V39143	POS	POS	POS	NEG	NEG	NEG	NEG	POS	POS	POS	NEG	AMB	POS	POS	NEG	NEG	AMB	POS
Greece 1_3680_Harmony	POS	POS	POS	NEG	NEG	NEG	NEG	POS	POS	POS	AMB	AMB	POS	POS	NEG	NEG	NEG	POS
**>"European Clade": UK-(**																		
ANS46 (Australia)	POS	POS	POS	NEG	NEG	NEG	NEG	POS	POS	POS	NEG	AMB	POS	POS	NEG	NEG	NEG	POS
LHH1 (USA)	POS	POS	POS	NEG	NEG	NEG	NEG	POS	POS	POS	NEG	AMB	POS	POS	NEG	NEG	NEG	POS
Dublin-DSH_AR01_0-1085	NEG	NEG	NEG	NEG	NEG	NEG		POS	POS	POS	AMB	AMB	POS	POS	NEG	NEG	POS	POS
Dublin-DSH_AR01_0-1118	NEG	NEG	NEG	NEG	NEG	NEG	NEG	POS	POS	POS	NEG	NEG	POS	POS	NEG	NEG	AMB	POS
Dublin-DSH_AR15_0098	NEG	NEG	NEG	NEG	NEG	NEG		POS	POS	POS	AMB	AMB	POS	POS	NEG	NEG	POS	POS
Dublin-DSH_AR15_0104	NEG	NEG	NEG	NEG	NEG	NEG	NEG	POS	POS	POS	AMB	AMB	POS	POS	NEG	NEG	POS	POS
UK_NCTC11939_UK-1_Harmony	NEG	NEG	NEG	NEG	NEG	NEG	NEG	POS	POS	POS	AMB	AMB	POS	POS	NEG	NEG	NEG	POS
**>"European Clade": BK2**																		
BK2421 (USA)	POS	POS	POS	NEG	NEG	NEG	NEG	POS	POS	POS	NEG	AMB	POS	POS	NEG	NEG	NEG	POS
**>"Aussi/NZ Clade", JKD6**																		
JKD6009 (Australia)	POS	POS	POS	NEG	NEG	NEG	NEG	POS	POS	POS	NEG	AMB	POS	POS	NEG	NEG	NEG	POS
JKD6008 (New Zealand)	POS	POS	POS	NEG	NEG	NEG	NEG	POS	POS	POS	NEG	AMB	POS	POS	NEG	NEG	NEG	POS
Perth_2015-S20209	POS	POS	POS	NEG	NEG	NEG	NEG	POS	POS	POS	NEG	AMB	POS	POS	NEG	NEG	NEG	POS
Perth_2015-S20305	POS	POS	POS	NEG	NEG	NEG	NEG	POS	POS	POS	NEG	AMB	POS	POS	NEG	NEG	NEG	POS
Perth_2015-S20603	POS	POS	POS	NEG	NEG	NEG	NEG	POS	POS	POS	NEG	AMB	POS	POS	NEG	NEG	NEG	POS
Perth_2015-S20607	POS	POS	POS	NEG	NEG	NEG	NEG	POS	POS	POS	NEG	AMB	POS	POS	NEG	NEG	NEG	POS
Perth_2015-S20608	POS	POS	POS	NEG	NEG	NEG	NEG	POS	POS	POS	NEG	AMB	POS	POS	NEG	NEG	NEG	POS
Perth_2015-S20610	POS	POS	POS	NEG	NEG	NEG	NEG	POS	POS	POS	NEG	AMB	POS	POS	NEG	NEG	NEG	POS
Perth_2015-S20613	POS	POS	POS	NEG	NEG	NEG	NEG	POS	POS	POS	NEG	AMB	POS	POS	NEG	NEG	NEG	POS
Perth_2015-S20617	POS	POS	POS	NEG	NEG	NEG	NEG	POS	POS	POS	NEG	AMB	POS	POS	NEG	NEG	NEG	POS
Perth_2015-S20622	POS	POS	POS	NEG	NEG	NEG	NEG	POS	POS	POS	NEG	AMB	POS	POS	NEG	NEG	NEG	POS
Perth_2015-S23619	POS	POS	POS	NEG	NEG	NEG	NEG	POS	POS	POS	NEG	AMB	POS	POS	NEG	NEG	NEG	POS
Perth_2015-S41210	POS	POS	POS	NEG	NEG	NEG	NEG	POS	POS	POS	NEG	AMB	POS	POS	NEG	NEG	NEG	POS
Perth_2015-S51001	POS	POS	POS	NEG	NEG	NEG	NEG	POS	POS	POS	NEG	AMB	POS	POS	NEG	NEG	NEG	POS
Perth_2015-S51003	POS	POS	POS	NEG	NEG	NEG	NEG	POS	POS	POS	NEG	AMB	POS	POS	NEG	NEG	NEG	POS
Perth_2015-S51006	POS	POS	POS	NEG	NEG	NEG	NEG	POS	POS	POS	NEG	AMB	POS	POS	NEG	NEG	NEG	POS
Perth_2015-S51007	POS	POS	POS	NEG	NEG	NEG	NEG	POS	POS	POS	NEG	AMB	POS	POS	NEG	NEG	NEG	POS
Perth_2015-S51008	POS	POS	POS	NEG	NEG	NEG	NEG	POS	POS	POS	NEG	AMB	POS	POS	NEG	NEG	NEG	POS
Perth_2015-S51014	POS	POS	POS	NEG	NEG	NEG	NEG	POS	POS	POS	NEG	AMB	POS	POS	NEG	NEG	NEG	POS
Perth_2015-S51408	POS	POS	POS	NEG	NEG	NEG	NEG	POS	POS	POS	NEG	AMB	POS	POS	NEG	NEG	NEG	POS
Perth_2016-S20614	POS	POS	POS	NEG	NEG	NEG	NEG	POS	POS	POS	NEG	AMB	POS	POS	NEG	NEG	NEG	POS
Perth_2016-S23614	POS	POS	POS	NEG	NEG	NEG	NEG	POS	POS	POS	NEG	AMB	POS	POS	NEG	NEG	NEG	POS
Perth_2016-S42009	POS	POS	POS	NEG	NEG	NEG	NEG	NEG	NEG	NEG	NEG	NEG	POS	POS	NEG	NEG	NEG	POS
Perth_2016-S51001	POS	POS	POS	NEG	NEG	NEG	NEG	POS	POS	POS	NEG	AMB	POS	POS	NEG	NEG	NEG	POS
Perth_2016-S51403	POS	POS	POS	NEG	NEG	NEG	NEG	POS	POS	POS	NEG	AMB	POS	POS	NEG	NEG	NEG	POS
Perth_07 RPA 20	POS	POS	POS	NEG	NEG	NEG	NEG	POS	POS	POS	NEG	AMB	POS	POS	NEG	NEG	NEG	POS
Perth_08-19088	POS	POS	POS	NEG	NEG	NEG	NEG	POS	POS	POS	AMB	AMB	POS	POS	NEG	NEG	POS	POS
Perth_01-15351	POS	POS	POS	NEG	NEG	NEG	NEG	POS	POS	POS	NEG	AMB	POS	POS	NEG	NEG	AMB	POS
Perth_01-15357	POS	POS	POS	NEG	NEG	NEG	NEG	POS	POS	POS	NEG	AMB	POS	POS	NEG	NEG	NEG	POS
Perth_01-15419	POS	POS	POS	NEG	NEG	NEG	NEG	POS	POS	POS	NEG	AMB	POS	POS	NEG	NEG	NEG	POS
Perth_01-16309	POS	POS	POS	NEG	NEG	NEG	NEG	POS	POS	POS	NEG	AMB	POS	POS	NEG	NEG	NEG	POS
Perth_03 SNP 95	POS	POS	POS	NEG	AMB	NEG	NEG	POS	POS	AMB	AMB	POS	POS	POS	NEG	NEG	POS	POS
Perth_2003_AH_47	POS	POS	POS	NEG	AMB	NEG	NEG	POS	POS	POS	AMB	AMB	POS	POS	NEG	NEG	POS	POS
**>"Australian/NZ Clade":**																		
Perth_2015-S20623	POS	POS	POS	NEG	NEG	NEG	NEG	POS	POS	POS	NEG	AMB	POS	POS	NEG	NEG	NEG	POS
Perth_2015-S20826	POS	POS	POS	NEG	NEG	NEG	NEG	POS	POS	POS	NEG	AMB	POS	POS	NEG	NEG	NEG	POS
Perth_05 NH 56	POS	POS	POS	NEG	AMB	NEG	NEG	POS	POS	POS	AMB	POS	POS	POS	NEG	NEG	AMB	POS
**>CC5-MRSA-III LA MRSA**																		
Delbrück 38 isolate from domestic turkey	POS	POS	AMB	NEG	POS	NEG	NEG	POS	POS	NEG	NEG	POS	POS	POS	POS	NEG	NEG	NEG
**> Staph. pseudintermed**																		
KM1381	NEG	NEG	NEG	NEG	NEG	NEG	NEG	NEG	NEG	NEG	NEG	NEG	NEG	NEG	NEG	NEG	NEG	NEG
**>"South-East Asian Clad**																		
M92 (Canada)	POS	POS	POS	NEG	NEG	NEG	NEG	POS	POS	POS	NEG	AMB	POS	POS	NEG	NEG	NEG	POS
CHI59 (China)	POS	POS	POS	NEG	NEG	NEG	NEG	POS	POS	POS	NEG	AMB	POS	POS	NEG	NEG	NEG	POS
CHI61 (China)	POS	POS	POS	NEG	NEG	NEG	NEG	POS	POS	POS	NEG	AMB	POS	POS	NEG	NEG	NEG	POS
CUHK_HK1997 (China)	POS	POS	POS	NEG	NEG	NEG	NEG	POS	POS	POS	NEG	AMB	POS	POS	NEG	NEG	NEG	POS
M996 (China)	POS	POS	POS	NEG	NEG	NEG	NEG	POS	POS	POS	NEG	AMB	POS	POS	NEG	NEG	NEG	POS
H211 (Denmark)	POS	POS	POS	NEG	NEG	NEG	NEG	POS	POS	POS	NEG	AMB	POS	POS	NEG	NEG	NEG	POS
H216 (Denmark)	POS	POS	POS	NEG	NEG	NEG	NEG	POS	POS	POS	NEG	AMB	POS	POS	NEG	NEG	NEG	POS
D71 (Germany)	POS	POS	POS	NEG	NEG	NEG	NEG	POS	POS	POS	NEG	AMB	POS	POS	NEG	NEG	NEG	POS
NMR05 (India)	POS	POS	POS	NEG	NEG	NEG	NEG	POS	POS	POS	NEG	AMB	POS	POS	NEG	NEG	NEG	POS
MAL11 (Malaysia)	POS	POS	POS	NEG	NEG	NEG	NEG	POS	POS	POS	NEG	AMB	POS	POS	NEG	NEG	NEG	POS
MAL9 (Malaysia)	POS	POS	POS	NEG	NEG	NEG	NEG	POS	POS	POS	NEG	AMB	POS	POS	NEG	NEG	NEG	POS
Na21 (Sri Lanka)	POS	POS	POS	NEG	NEG	NEG	NEG	POS	POS	POS	NEG	AMB	POS	POS	NEG	NEG	NEG	POS
M592 (Syria)	POS	POS	POS	NEG	NEG	NEG	NEG	POS	POS	POS	NEG	AMB	POS	POS	NEG	NEG	NEG	POS
H202 (Thailand)	POS	POS	POS	NEG	NEG	NEG	NEG	POS	POS	POS	NEG	AMB	POS	POS	NEG	NEG	NEG	POS
S102 (Thailand)	POS	POS	POS	NEG	NEG	NEG	NEG	POS	POS	POS	NEG	AMB	POS	POS	NEG	NEG	NEG	POS
S130 (Thailand)	POS	POS	POS	NEG	NEG	NEG	NEG	POS	POS	POS	NEG	AMB	POS	POS	NEG	NEG	NEG	POS
S40 (Thailand)	POS	POS	POS	NEG	NEG	NEG	NEG	POS	POS	POS	NEG	AMB	POS	POS	NEG	NEG	NEG	POS
S71 (Thailand)	POS	POS	POS	NEG	NEG	NEG	NEG	POS	POS	POS	NEG	AMB	POS	POS	NEG	NEG	NEG	POS
S87 (Thailand)	POS	POS	POS	NEG	NEG	NEG	NEG	POS	POS	POS	NEG	AMB	POS	POS	NEG	NEG	NEG	POS
S93 (Thailand)	POS	POS	POS	NEG	NEG	NEG	NEG	POS	POS	POS	NEG	AMB	POS	POS	NEG	NEG	NEG	POS
US_002 (Thailand)	POS	POS	POS	NEG	NEG	NEG	NEG	POS	POS	POS	NEG	AMB	POS	POS	NEG	NEG	NEG	POS
US_017 (Thailand)	POS	POS	POS	NEG	NEG	NEG	NEG	POS	POS	POS	NEG	AMB	POS	POS	NEG	NEG	NEG	POS
WAMC6102 (Thailand)	POS	POS	POS	NEG	NEG	NEG	NEG	POS	POS	POS	NEG	AMB	POS	POS	NEG	NEG	NEG	POS
NCTC_13626_TW20 (UK)	POS	POS	POS	NEG	NEG	NEG	NEG	POS	POS	POS	NEG	AMB	POS	POS	NEG	NEG	NEG	POS
BSAC3021 (UK)	POS	POS	POS	NEG	NEG	NEG	NEG	POS	POS	POS	NEG	AMB	POS	POS	NEG	NEG	NEG	POS
BSAC3046 (UK)	POS	POS	POS	NEG	NEG	NEG	NEG	POS	POS	POS	NEG	AMB	POS	POS	NEG	NEG	NEG	POS
1193_SAUR (USA)	POS	POS	POS	NEG	NEG	NEG	NEG	POS	POS	POS	NEG	AMB	POS	POS	NEG	NEG	NEG	POS
1194_SAUR (USA)	POS	POS	POS	NEG	NEG	NEG	NEG	POS	POS	POS	NEG	AMB	POS	POS	NEG	NEG	NEG	POS

STRAIN / ISOLATE	ADHAESION FACTORS / GENES ENCODING MICROBIAL SURFACE COMPONENTS RECOGNIZING ADHESIVE MATRIX MOLECULES (MSCRAMM GENES)																	
	bbp							clfA					clfB					cna
	bbp	bbp (cons)	bbp (COL+MW2)	bbp (MRSA252)	bbp (Mu50)	bbp (RF122)	bbp (ST45)	clfA	clfA (cons)	clfA (COL+RF122)	clfA (MRSA252)	clfA (Mu50+MW2)	clfB	clfB (cons)	clfB (COL+Mu50)	clfB (MW2)	clfB (RF122)	cna
	Bone sialoprotein-binding protein							Clumping factor A					Clumping factor B					Collagen-binding adhesin
>"South-East Asian Clade": TW20 (Irish AR44), c																		
1195_SAUR (USA)	POS	POS	POS	NEG	NEG	NEG	NEG	POS	POS	POS	NEG	AMB	POS	POS	NEG	NEG	NEG	POS
FVRH6002 (USA)	POS	POS	POS	NEG	NEG	NEG	NEG	POS	POS	POS	NEG	AMB	POS	POS	NEG	NEG	NEG	POS
KINW6048 (USA)	POS	POS	POS	NEG	NEG	NEG	NEG	POS	POS	POS	NEG	AMB	POS	POS	NEG	NEG	NEG	POS
LAMC0011 (USA)	POS	POS	POS	NEG	NEG	NEG	NEG	POS	POS	POS	NEG	AMB	POS	POS	NEG	NEG	NEG	POS
SJOS6053 (USA)	POS	POS	POS	NEG	NEG	NEG	NEG	POS	POS	POS	NEG	AMB	POS	POS	NEG	NEG	NEG	POS
SJOS6072 (USA)	POS	POS	POS	NEG	NEG	NEG	NEG	POS	POS	POS	NEG	AMB	POS	POS	NEG	NEG	NEG	POS
UCIM6042 (USA)	POS	POS	POS	NEG	NEG	NEG	NEG	POS	POS	POS	NEG	AMB	POS	POS	NEG	NEG	NEG	POS
Perth_2015-S31902	POS	POS	POS	NEG	NEG	NEG	NEG	POS	POS	POS	NEG	AMB	POS	POS	NEG	NEG	NEG	POS
Perth_2015-S31904	POS	POS	POS	NEG	NEG	NEG	NEG	POS	POS	POS	NEG	AMB	POS	POS	NEG	NEG	NEG	POS
Perth_2015-S31913	POS	POS	POS	NEG	NEG	NEG	NEG	POS	POS	POS	NEG	AMB	POS	POS	NEG	NEG	NEG	POS
Perth_2015-S31918	POS	POS	POS	NEG	NEG	NEG	NEG	POS	POS	POS	NEG	AMB	POS	POS	NEG	NEG	NEG	POS
Dublin-DSH_AR44_E1183	POS	POS	POS	NEG	NEG	NEG	NEG	POS	POS	POS	AMB	AMB	POS	POS	NEG	NEG	POS	POS
Dublin-DSH_Unfamiliar-3_E1520	POS	POS	POS	NEG	AMB	NEG	NEG	POS	POS	POS	AMB	AMB	POS	POS	NEG	NEG	NEG	POS
Hong Kong_042	POS	POS	POS	NEG	NEG	NEG	NEG	POS	POS	POS	AMB	AMB	POS	POS	NEG	NEG	POS	POS
Hong Kong_101	POS	POS	POS	NEG	NEG	NEG	NEG	POS	POS	POS	AMB	AMB	POS	POS	NEG	NEG	POS	POS
Hong Kong_111	POS	POS	POS	NEG	NEG	NEG	AMB	AMB	POS	AMB	AMB	POS	AMB	POS	NEG	NEG	POS	POS
Hong Kong_113	POS	POS	POS	NEG	NEG	NEG	NEG	POS	POS	POS	AMB	AMB	POS	POS	NEG	NEG	NEG	POS
Hong Kong_115	POS	POS	POS	NEG	AMB	NEG	NEG	POS	POS	POS	AMB	AMB	POS	POS	NEG	NEG	POS	POS
Hong Kong_118	POS	POS	POS	NEG	AMB	NEG	NEG	POS	POS	POS	AMB	AMB	POS	POS	NEG	NEG	POS	POS
Hong Kong_125	POS	POS	POS	NEG	NEG	NEG	NEG	POS	POS	AMB	AMB	POS	POS	POS	NEG	NEG	POS	POS
Hong Kong_129	POS	POS	POS	NEG	NEG	NEG	NEG	POS	POS	POS	AMB	AMB	POS	POS	NEG	NEG	AMB	POS
Hong Kong_133	POS	POS	POS	NEG	NEG	NEG	NEG	POS	POS	POS	AMB	AMB	POS	POS	NEG	NEG	POS	POS
Kuwait_009	NEG	NEG	NEG	NEG	NEG	NEG	NEG	POS	POS	POS	AMB	AMB	POS	POS	NEG	NEG	POS	POS
Kuwait_103	POS	POS	POS	NEG	AMB	NEG	AMB	POS	POS	POS	AMB	AMB	POS	POS	NEG	NEG	POS	POS
Kuwait_107	POS	POS	POS	NEG	AMB	NEG	NEG	POS	POS	AMB	AMB	POS	POS	POS	NEG	NEG	POS	POS
Perth_03 AH 88	POS	POS	POS	NEG	NEG	NEG	NEG	POS	POS	POS	NEG	AMB	POS	POS	NEG	NEG	NEG	POS
Perth_08-16905	POS	POS	POS	NEG	NEG	NEG	NEG	POS	POS	POS	AMB	AMB	POS	POS	NEG	NEG	AMB	POS
Riyadh_Alfaisal-10_SICU_13_B5672_1117353	POS	POS	POS	NEG	AMB	NEG	NEG	POS	POS	POS	AMB	AMB	POS	POS	NEG	NEG	POS	POS
Riyadh_SSTI_17_3508602	POS	POS	POS	NEG	AMB	NEG	NEG	POS	POS	POS	AMB	AMB	POS	POS	NEG	NEG	POS	POS
Riyadh-2822088-R	POS	POS	POS	NEG	AMB	NEG	NEG	POS	POS	POS	AMB	AMB	POS	POS	NEG	NEG	POS	POS
Trinidad&Tobago_2012_195 (619/12)	POS	POS	POS	NEG	NEG	NEG	NEG	POS	POS	AMB	AMB	POS	POS	POS	NEG	NEG	POS	POS
Trinidad&Tobago_2012_370 (1112-52177)	POS	POS	POS	NEG	AMB	NEG	NEG	POS	POS	AMB	POS	AMB	POS	POS	NEG	NEG	POS	POS
Trinidad&Tobago_2012_378 (4139)	POS	POS	POS	NEG	NEG	NEG	NEG	POS	POS	AMB	POS	AMB	POS	POS	NEG	NEG	POS	POS
Trinidad&Tobago_2013_PA21	POS	POS	POS	NEG	NEG	NEG	NEG	POS	POS	POS	AMB	AMB	POS	POS	NEG	NEG	NEG	POS
Trinidad&Tobago_2013_PA22	POS	POS	POS	NEG	AMB	NEG	NEG	POS	POS	AMB	AMB	POS	POS	POS	NEG	NEG	POS	POS
Trinidad&Tobago_2912_340 (8523/12)	POS	POS	POS	NEG	NEG	NEG	NEG	POS	POS	AMB	POS	AMB	POS	POS	NEG	NEG	POS	POS
Trinidad&Tobago_MRSA2010_105	POS	POS	POS	NEG	AMB	NEG	NEG	POS	POS	POS	AMB	AMB	POS	POS	NEG	NEG	POS	POS
Trinidad&Tobago_MRSA2010_108	POS	POS	POS	NEG	AMB	NEG	NEG	POS	POS	POS	AMB	AMB	POS	POS	NEG	NEG	POS	POS
Trinidad&Tobago_MRSA2010_33	POS	POS	POS	NEG	NEG	NEG	NEG	POS	POS	POS	AMB	AMB	POS	POS	NEG	NEG	POS	POS
Trinidad&Tobago_MRSA2010_79	POS	POS	POS	NEG	NEG	NEG	NEG	POS	POS	POS	AMB	AMB	POS	POS	NEG	NEG	NEG	POS
Trinidad&Tobago_MRSA2010_88	POS	POS	POS	NEG	NEG	NEG	NEG	POS	POS	POS	AMB	AMB	POS	POS	NEG	NEG	POS	POS
Trinidad&Tobago_MRSA2010_97	POS	POS	POS	NEG	NEG	NEG	NEG	POS	POS	POS	AMB	AMB	POS	POS	NEG	NEG	POS	POS
Trinidad&Tobago_SSTI 2012_031	POS	POS	POS	NEG	AMB	NEG	NEG	POS	POS	AMB	AMB	POS	POS	POS	NEG	NEG	POS	POS
Uganda-29_17148_250811	POS	POS	POS	NEG	NEG	NEG	NEG	POS	POS	POS	AMB	AMB	POS	POS	NEG	NEG	POS	POS
>"South-East Asian Clad																		
UCIM6015	POS	POS	POS	NEG	NEG	NEG	NEG	POS	POS	POS	NEG	AMB	POS	POS	NEG	NEG	NEG	POS
Hong Kong_121	POS	POS	POS	NEG	NEG	NEG	NEG	POS	POS	POS	AMB	AMB	POS	POS	NEG	NEG	NEG	POS
>"South-East Asian Clad																		
Z172 (Taiwan)	POS	POS	POS	NEG	NEG	NEG	NEG	POS	POS	POS	NEG	AMB	POS	POS	NEG	NEG	NEG	POS
Russia-19_0342_Kurgan	POS	POS	POS	NEG	NEG	NEG	NEG	POS	POS	POS	AMB	AMB	POS	POS	NEG	NEG	POS	POS
>"South-East Asian Clad																		
Perth_01-15392	POS	POS	POS	NEG	NEG	NEG	NEG	POS	POS	POS	NEG	AMB	POS	POS	NEG	NEG	AMB	POS
>"South-East Asian Clad																		
XN108 (China)	POS	POS	POS	NEG	NEG	NEG	NEG	POS	POS	POS	NEG	AMB	POS	POS	NEG	NEG	NEG	POS
NMR09 (India)	POS	POS	POS	NEG	NEG	NEG	NEG	POS	POS	POS	NEG	AMB	POS	POS	NEG	NEG	NEG	POS
346 (Malaysia)	POS	POS	POS	NEG	NEG	NEG	NEG	POS	POS	POS	NEG	AMB	POS	POS	NEG	NEG	NEG	POS
DS_009 (Thailand)	POS	POS	POS	NEG	NEG	NEG	NEG	POS	POS	POS	NEG	AMB	POS	POS	NEG	NEG	NEG	POS
18ANR581005 (Ind)	POS	POS	POS	NEG	AMB	NEG	NEG	POS	POS	POS	AMB	AMB	POS	POS	NEG	NEG	POS	POS
Bengalaru_ST20141405	POS	POS	POS	NEG	AMB	NEG	NEG	POS	POS	POS	AMB	AMB	POS	POS	NEG	NEG	POS	POS
Bengalaru_ST20141406	POS	POS	POS	NEG	AMB	NEG	NEG	POS	POS	POS	AMB	AMB	POS	POS	NEG	NEG	POS	POS
Bengalaru_ST20141417	POS	POS	POS	NEG	AMB	NEG	NEG	POS	POS	POS	AMB	AMB	POS	POS	NEG	NEG	POS	POS
Hong Kong_107	POS	POS	POS	NEG	NEG	NEG	NEG	AMB	POS	POS	AMB	AMB	AMB	POS	NEG	NEG	POS	POS
Hong Kong_12	POS	POS	POS	NEG	NEG	NEG	NEG	POS	POS	POS	AMB	AMB	POS	POS	NEG	NEG	POS	POS
Hong Kong_132	POS	POS	POS	NEG	NEG	NEG	NEG	AMB	POS	POS	AMB	AMB	AMB	POS	NEG	NEG	POS	POS
Riyadh-3028763-R	POS	POS	POS	NEG	AMB	NEG	NEG	POS	POS	POS	AMB	POS	POS	POS	NEG	NEG	AMB	POS
Trinidad&Tobago_MRSA2010_94	POS	POS	POS	NEG	NEG	NEG	NEG	POS	POS	POS	AMB	AMB	POS	POS	NEG	NEG	POS	POS
>"South-East Asian Clad																		
M705 (Thailand)	POS	POS	POS	NEG	NEG	NEG	NEG	POS	POS	POS	NEG	AMB	POS	POS	NEG	NEG	NEG	POS
S85 (Thailand)	POS	POS	POS	NEG	NEG	NEG	NEG	POS	POS	POS	NEG	AMB	POS	POS	NEG	NEG	NEG	POS
Riyadh_Alfaisal-04_281014_S5076	POS	POS	POS	NEG	AMB	AMB	AMB	POS	POS	POS	AMB	AMB	POS	POS	NEG	NEG	POS	POS
Riyadh_Alfaisal-26_S15145_1104163	POS	POS	POS	NEG	AMB	NEG	NEG	POS	POS	POS	AMB	AMB	POS	POS	NEG	NEG	POS	POS
Riyadh-2817437-W	POS	POS	POS	NEG	AMB	NEG	NEG	POS	POS	AMB	AMB	POS	POS	POS	NEG	NEG	POS	POS
>"South-East Asian Clad																		
PPUKM-775-2009 (Malaysia)	POS	POS	POS	NEG	NEG	NEG	NEG	POS	POS	POS	NEG	AMB	POS	POS	NEG	NEG	NEG	POS
Hong Kong_138	POS	POS	POS	NEG	NEG	NEG	NEG	POS	POS	POS	AMB	AMB	POS	POS	NEG	NEG	POS	POS
>"South-East Asian Clad																		
Kuwait_001	POS	POS	POS	NEG	AMB	NEG	NEG	POS	POS	POS	AMB	AMB	POS	POS	NEG	NEG	POS	POS
Kuwait_337	POS	POS	POS	NEG	AMB	NEG	NEG	POS	POS	POS	AMB	AMB	POS	POS	NEG	NEG	POS	POS
>"South-East Asian Clad																		
CUHK_HK2007 (China)	POS	POS	POS	NEG	NEG	NEG	NEG	POS	POS	POS	NEG	AMB	POS	POS	NEG	NEG	NEG	POS
NMR02 (India)	POS	POS	POS	NEG	NEG	NEG	NEG	POS	POS	POS	NEG	AMB	POS	POS	NEG	NEG	NEG	POS
VB1490 (India)	NEG	NEG	NEG	NEG	NEG	NEG	NEG	POS	POS	POS	NEG	AMB	POS	POS	NEG	NEG	NEG	POS
V521 (Korea)	POS	POS	POS	NEG	NEG	NEG	NEG	POS	POS	POS	NEG	AMB	POS	POS	NEG	NEG	NEG	POS
HST-077 (Lebanon)	POS	POS	POS	NEG	NEG	NEG	NEG	POS	POS	POS	NEG	AMB	POS	POS	NEG	NEG	NEG	POS
MAL1 (Malaysia)	POS	POS	POS	NEG	NEG	NEG	NEG	POS	POS	POS	NEG	AMB	POS	POS	NEG	NEG	NEG	POS
MAL3 (Malaysia)	POS	POS	POS	NEG	NEG	NEG	NEG	POS	POS	POS	NEG	AMB	POS	POS	NEG	NEG	NEG	POS
PPUKM-261-2009 (Malaysia)	POS	POS	POS	NEG	NEG	NEG	NEG	POS	POS	POS	NEG	AMB	POS	POS	NEG	NEG	NEG	POS
PPUKM-332-2009 (Malaysia)	POS	POS	POS	NEG	NEG	NEG	NEG	POS	POS	POS	NEG	AMB	POS	POS	NEG	NEG	NEG	POS
S38 (Thailand)	POS	POS	POS	NEG	NEG	NEG	NEG	POS	POS	POS	NEG	AMB	POS	POS	NEG	NEG	NEG	POS
S7 (Thailand)	POS	POS	POS	NEG	NEG	NEG	NEG	POS	POS	POS	NEG	AMB	POS	POS	NEG	NEG	NEG	POS
US_008 (Thailand)	POS	POS	POS	NEG	NEG	NEG	NEG	POS	POS	POS	NEG	AMB	POS	POS	NEG	NEG	NEG	POS
US_030 (Thailand)	POS	POS	POS	NEG	NEG	NEG	NEG	POS	POS	POS	NEG	AMB	POS	POS	NEG	NEG	NEG	POS
M170 (Tsunami-related)	POS	POS	POS	NEG	NEG	NEG	NEG	POS	POS	POS	NEG	AMB	POS	POS	NEG	NEG	NEG	POS
BSAC697 (UK)	POS	POS	POS	NEG	NEG	NEG	NEG	POS	POS	POS	NEG	AMB	POS	POS	NEG	NEG	NEG	POS
IS-189	POS	POS	POS	NEG	NEG	NEG	NEG	POS	POS	POS	NEG	AMB	POS	POS	NEG	NEG	NEG	POS
MRGR3	POS	POS	POS	NEG	NEG	NEG	NEG	POS	POS	POS	NEG	AMB	POS	POS	NEG	NEG	NEG	POS
smg07	POS	POS	POS	NEG	NEG	NEG	NEG	POS	POS	POS	NEG	AMB	POS	POS	NEG	NEG	NEG	POS
AH1-AUS-EMRSA-3	AMB	POS	POS	NEG	NEG	NEG	NEG	AMB	POS	POS	AMB	AMB	AMB	POS	NEG	NEG	POS	POS
Bengalaru_ST20141403	POS	POS	POS	NEG	AMB	NEG	NEG	POS	POS	POS	AMB	AMB	POS	POS	NEG	NEG	POS	POS
Finland E24_98541_Harmony	POS	POS	POS	NEG	NEG	NEG	NEG	POS	POS	AMB	POS	AMB	POS	POS	NEG	NEG	POS	POS
Hong Kong_10	POS	POS	POS	NEG	NEG	NEG	NEG	POS	POS	POS	AMB	AMB	POS	POS	NEG	NEG	POS	POS
Hong Kong_100	POS	POS	POS	NEG	NEG	NEG	NEG	POS	POS	POS	AMB	AMB	POS	POS	NEG	NEG	POS	POS
Hong Kong_199	POS	POS	POS	NEG	AMB	NEG	AMB	POS	POS	POS	AMB	AMB	POS	POS	NEG	NEG	POS	POS
Hong Kong_34	POS	POS	POS	NEG	AMB	NEG	AMB	POS	POS	POS	AMB	AMB	POS	POS	NEG	NEG	POS	POS
Hong Kong_88	POS	POS	POS	NEG	AMB	AMB	AMB	POS	POS	AMB	POS	AMB	POS	POS	NEG	NEG	POS	POS
Hong Kong_92	POS	POS	POS	NEG	NEG	NEG	AMB	POS	POS	POS	AMB	AMB	POS	POS	NEG	NEG	POS	POS
Hong Kong_94	POS	POS	POS	NEG	NEG	NEG	NEG	POS	POS	POS	AMB	AMB	POS	POS	NEG	NEG	POS	POS
Kuwait_257	POS	POS	POS	NEG	NEG	NEG	NEG	POS	POS	AMB	AMB	POS	POS	POS	NEG	NEG	POS	POS
Perth_01-15330	POS	POS	POS	NEG	NEG	NEG	NEG	POS	POS	POS	NEG	AMB	POS	POS	NEG	NEG	NEG	POS
Perth_01-15349	POS	POS	POS	NEG	NEG	NEG	NEG	POS	POS	POS	NEG	AMB	POS	POS	NEG	NEG	AMB	POS
Perth_01-16176	NEG	NEG	NEG	NEG	NEG	NEG	NEG	POS	POS	POS	NEG	AMB	POS	POS	NEG	NEG	NEG	POS
Perth_WBG8379	NEG	NEG	NEG	NEG	NEG	NEG	NEG	POS	POS	POS	AMB	AMB	POS	POS	NEG	NEG	POS	POS
Rawalpindi_Kidney Center_56	POS	POS	POS	NEG	AMB	NEG	NEG	POS	POS	AMB	AMB	POS	POS	POS	NEG	NEG	POS	POS
Riyadh_SSTI_05_3464614	POS	POS	POS	NEG	NEG	NEG	NEG	POS	POS	POS	AMB	AMB	POS	POS	NEG	NEG	NEG	POS
Trinidad&Tobago_2012_157	POS	POS	POS	NEG	AMB	NEG	NEG	POS	POS	AMB	POS	AMB	POS	POS	NEG	NEG	POS	POS

STRAIN / ISOLATE	ADHAESION FACTORS / GENES ENCODING MICROBIAL SURFACE COMPONENTS RECOGNIZING ADHESIVE MATRIX MOLECULES (MSCRAMM GENES)																	
	bbp							clfA					clfB					cna
	bbp	bbp (cons)	bbp (COL+MW2)	bbp (MRSA252)	bbp (Mu50)	bbp (RF122)	bbp (ST45)	clfA	clfA (cons)	clfA (COL+RF122)	clfA (MRSA252)	clfA (Mu50+MW2)	clfB	clfB (cons)	clfB (COL+Mu50)	clfB (MW2)	clfB (RF122)	cna
	Bone sialoprotein-binding protein							Clumping factor A					Clumping factor B					Collagen-binding adhesin
>"South-East Asian Clad																		
M418	POS	POS	POS	NEG	NEG	NEG	NEG	POS	POS	POS	NEG	AMB	POS	POS	NEG	NEG	NEG	POS
>"South-East Asian Clad																		
NMR07 (India)	POS	POS	POS	NEG	NEG	NEG	NEG	POS	POS	POS	NEG	AMB	POS	POS	NEG	NEG	NEG	POS
NMR08 (India)	POS	POS	POS	NEG	NEG	NEG	NEG	POS	POS	POS	NEG	AMB	POS	POS	NEG	NEG	NEG	POS
Bengalaru_ST20121978	POS	POS	POS	NEG	AMB	NEG	NEG	POS	POS	POS	AMB	AMB	POS	POS	NEG	NEG	POS	POS
Hong Kong_89	POS	POS	POS	NEG	AMB	AMB	AMB	POS	POS	AMB	AMB	POS	POS	POS	NEG	NEG	POS	POS
>"South-East Asian Clad																		
Perth_2005_AH_5	POS	POS	POS	NEG	NEG	NEG	NEG	POS	POS	POS	AMB	AMB	POS	POS	NEG	NEG	NEG	POS
>"South-East Asian Clad																		
Trinidad&Tobago_MRSA2010_103	POS	POS	POS	NEG	NEG	NEG	NEG	POS	POS	POS	AMB	AMB	POS	POS	NEG	NEG	AMB	POS
Trinidad&Tobago_2013_PA25	POS	POS	POS	NEG	AMB	NEG	NEG	POS	POS	POS	AMB	AMB	POS	POS	NEG	NEG	POS	POS
Trinidad&Tobago_MRSA2010_110	POS	POS	POS	NEG	NEG	NEG	NEG	POS	POS	POS	AMB	AMB	POS	POS	NEG	NEG	POS	POS
Trinidad&Tobago_SSTI 2012_111	POS	POS	POS	NEG	AMB	NEG	NEG	POS	POS	POS	AMB	AMB	POS	POS	NEG	NEG	AMB	POS
Trinidad&Tobago_MRSA2010_158	POS	POS	POS	NEG	NEG	NEG	NEG	POS	POS	POS	AMB	AMB	POS	POS	NEG	NEG	POS	POS
>"South-East Asian Clad																		
Kuwait_122	POS	POS	POS	NEG	NEG	NEG	NEG	POS	POS	POS	AMB	AMB	POS	POS	NEG	NEG	AMB	POS
Kuwait_002	POS	POS	POS	NEG	NEG	NEG	NEG	POS	POS	POS	AMB	POS	POS	POS	NEG	NEG	AMB	POS
Kuwait_196	POS	POS	POS	NEG	AMB	NEG	NEG	POS	POS	POS	AMB	AMB	POS	POS	NEG	NEG	POS	POS
Kuwait_199	POS	POS	POS	NEG	AMB	NEG	NEG	POS	POS	POS	POS	AMB	POS	POS	NEG	NEG	POS	POS
Hong Kong_136	POS	POS	POS	NEG	AMB	NEG	NEG	POS	POS	POS	AMB	AMB	POS	POS	NEG	NEG	POS	POS
>"South-East Asian Clad																		
P32 (Poland)	POS	POS	POS	NEG	NEG	NEG	NEG	POS	POS	POS	NEG	AMB	POS	POS	NEG	NEG	NEG	POS
>"South-East Asian Clad																		
Perth_2015-S32313	POS	POS	POS	NEG	NEG	NEG	NEG	POS	POS	POS	NEG	AMB	POS	POS	NEG	NEG	NEG	POS
>"hla-negative South-Ea																		
DEN907 (Denmark)	POS	POS	POS	NEG	NEG	NEG	NEG	POS	POS	POS	NEG	AMB	POS	POS	NEG	NEG	NEG	POS
>"hla-negative South-Ea																		
S26 (Thailand)	POS	POS	POS	NEG	NEG	NEG	NEG	POS	POS	POS	NEG	AMB	POS	POS	NEG	NEG	NEG	POS
S42 (Thailand)	POS	POS	POS	NEG	NEG	NEG	NEG	POS	POS	POS	NEG	AMB	POS	POS	NEG	NEG	NEG	POS
S97 (Thailand)	POS	POS	POS	NEG	NEG	NEG	NEG	POS	POS	POS	NEG	AMB	POS	POS	NEG	NEG	NEG	POS
S78 (Thailand)	POS	POS	POS	NEG	NEG	NEG	NEG	POS	POS	POS	NEG	AMB	POS	POS	NEG	NEG	NEG	POS
S25 (Thailand)	POS	POS	POS	NEG	NEG	NEG	NEG	POS	POS	POS	NEG	AMB	POS	POS	NEG	NEG	NEG	POS
S21 (Thailand)	POS	POS	POS	NEG	NEG	NEG	NEG	POS	POS	POS	NEG	AMB	POS	POS	NEG	NEG	NEG	POS
S106 (Thailand)	POS	POS	POS	NEG	NEG	NEG	NEG	POS	POS	POS	NEG	AMB	POS	POS	NEG	NEG	NEG	POS
S24 (Thailand)	POS	POS	POS	NEG	NEG	NEG	NEG	POS	POS	POS	NEG	AMB	POS	POS	NEG	NEG	NEG	POS
S39 (Thailand)	POS	POS	POS	NEG	NEG	NEG	NEG	POS	POS	POS	NEG	AMB	POS	POS	NEG	NEG	NEG	POS
S2 (Thailand)	POS	POS	POS	NEG	NEG	NEG	NEG	POS	POS	POS	NEG	AMB	POS	POS	NEG	NEG	NEG	POS
S81 (Thailand)	POS	POS	POS	NEG	NEG	NEG	NEG	POS	POS	POS	NEG	AMB	POS	POS	NEG	NEG	NEG	POS
M116 (Vietnam)	POS	POS	POS	NEG	NEG	NEG	NEG	POS	POS	POS	NEG	AMB	POS	POS	NEG	NEG	NEG	POS
>"Portuguese Clade": P																		
FFP103 (Portugal)	POS	POS	POS	NEG	NEG	NEG	NEG	POS	POS	POS	NEG	AMB	POS	POS	NEG	NEG	NEG	POS
HDG2 (Portugal)	POS	POS	POS	NEG	NEG	NEG	NEG	POS	POS	POS	NEG	AMB	POS	POS	NEG	NEG	NEG	POS
HSA10 (Portugal)	NEG	NEG	NEG	NEG	NEG	NEG	NEG	POS	POS	POS	NEG	AMB	POS	POS	NEG	NEG	NEG	POS
HSA11 (Portugal)	NEG	NEG	NEG	NEG	NEG	NEG	NEG	POS	POS	POS	NEG	AMB	POS	POS	NEG	NEG	NEG	POS
ICP5011 (Portugal)	POS	POS	POS	NEG	NEG	NEG	NEG	POS	POS	POS	NEG	AMB	POS	POS	NEG	NEG	NEG	POS
ICP5014 (Portugal)	POS	POS	POS	NEG	NEG	NEG	NEG	POS	POS	POS	NEG	AMB	POS	POS	NEG	NEG	NEG	POS
ICP5062 (Portugal)	POS	POS	POS	NEG	NEG	NEG	NEG	POS	POS	POS	NEG	AMB	POS	POS	NEG	NEG	NEG	POS
Strain 1063_ATCC 33592 FDAARGOS_3 (USA)	POS	POS	POS	NEG	NEG	NEG	NEG	POS	POS	POS	NEG	AMB	POS	POS	NEG	NEG	NEG	POS
	POS	POS	POS	NEG	AMB	NEG	NEG	POS	POS	POS	AMB	AMB	POS	POS	NEG	NEG	POS	POS
Russia-07_0110_Kurgan	POS	POS	POS	NEG	NEG	NEG	NEG	POS	POS	POS	AMB	AMB	POS	POS	NEG	NEG	POS	POS
Russia-11_0164_Kurgan	POS	POS	POS	NEG	AMB	NEG	NEG	POS	POS	POS	AMB	AMB	POS	POS	NEG	NEG	POS	POS
Russia-20_0367_Kurgan	POS	POS	POS	NEG	AMB	NEG	NEG	POS	POS	POS	AMB	AMB	POS	POS	NEG	NEG	POS	POS
Russia-21_0390_Chelyabinsk	POS	POS	POS	NEG	AMB	NEG	NEG	POS	POS	POS	AMB	AMB	POS	POS	NEG	NEG	POS	POS
>"South American/ Mid																		
AGT1 (Argentina)	POS	POS	POS	NEG	NEG	NEG	NEG	POS	POS	POS	NEG	AMB	POS	POS	NEG	NEG	NEG	POS
AGT120 (Argentina)	POS	POS	POS	NEG	NEG	NEG	NEG	POS	POS	POS	NEG	AMB	POS	POS	NEG	NEG	NEG	POS
AGT67 (Argentina)	POS	POS	NEG	NEG	NEG	NEG	NEG	POS	POS	POS	NEG	AMB	POS	POS	NEG	NEG	NEG	POS
AGT9 (Argentina)	POS	POS	POS	NEG	NEG	NEG	NEG	POS	POS	POS	NEG	AMB	POS	POS	NEG	NEG	NEG	POS
RA3 (Argentina)	POS	POS	POS	NEG	NEG	NEG	NEG	POS	POS	POS	NEG	AMB	POS	POS	NEG	NEG	NEG	POS
RA6 (Argentina)	POS	POS	POS	NEG	NEG	NEG	NEG	POS	POS	POS	NEG	AMB	POS	POS	NEG	NEG	NEG	POS
RA7 (Argentina)	POS	POS	POS	NEG	NEG	NEG	NEG	POS	POS	POS	NEG	AMB	POS	POS	NEG	NEG	NEG	POS
Be62 (Brazil)	POS	POS	POS	NEG	NEG	NEG	NEG	POS	POS	POS	NEG	AMB	POS	POS	NEG	NEG	NEG	POS
Bmb9393 (Brazil)	POS	POS	POS	NEG	NEG	NEG	NEG	POS	POS	POS	NEG	AMB	POS	POS	NEG	NEG	NEG	POS
BRA36 (Brazil)	POS	POS	POS	NEG	NEG	NEG	NEG	POS	POS	POS	NEG	AMB	POS	POS	NEG	NEG	NEG	POS
BZ48 (Brazil)	POS	POS	POS	NEG	NEG	NEG	NEG	POS	POS	POS	NEG	AMB	POS	POS	NEG	NEG	NEG	POS
Gv51 (Brazil)	POS	POS	POS	NEG	NEG	NEG	NEG	POS	POS	POS	NEG	AMB	POS	POS	NEG	NEG	NEG	POS
Gv69 (Brazil)	POS	POS	POS	NEG	NEG	NEG	NEG	POS	POS	POS	NEG	AMB	POS	POS	NEG	NEG	NEG	POS
Gv88 (Brazil)	POS	POS	POS	NEG	NEG	NEG	NEG	POS	POS	POS	NEG	AMB	POS	POS	NEG	NEG	NEG	POS
HC1335 (Brazil)	POS	POS	POS	NEG	NEG	NEG	NEG	POS	POS	POS	NEG	AMB	POS	POS	NEG	NEG	NEG	POS
HC1340 (Brazil)	POS	POS	POS	NEG	NEG	NEG	NEG	POS	POS	POS	NEG	AMB	POS	POS	NEG	NEG	NEG	POS
HC556 (Brazil)	POS	POS	POS	NEG	NEG	NEG	NEG	POS	POS	POS	NEG	AMB	POS	POS	NEG	NEG	NEG	POS
HU25 (Brazil)	POS	POS	POS	NEG	NEG	NEG	NEG	POS	POS	POS	NEG	AMB	POS	POS	NEG	NEG	NEG	POS
UB563 (Brazil)	POS	POS	POS	NEG	NEG	NEG	NEG	POS	POS	POS	NEG	AMB	POS	POS	NEG	NEG	NEG	POS
2A8 (Czech Republic)	POS	POS	POS	NEG	NEG	NEG	NEG	POS	POS	POS	NEG	AMB	POS	POS	NEG	NEG	NEG	POS
M1229 (Denmark)	POS	POS	POS	NEG	NEG	NEG	NEG	POS	POS	POS	NEG	AMB	POS	POS	NEG	NEG	NEG	POS
FRICAR (France)	POS	POS	POS	NEG	NEG	NEG	NEG	POS	POS	POS	NEG	AMB	POS	POS	NEG	NEG	NEG	POS
D90 (Germany)	POS	POS	POS	NEG	NEG	NEG	NEG	POS	POS	POS	NEG	AMB	POS	POS	NEG	NEG	NEG	POS
LIT68 (Lithuania)	POS	POS	POS	NEG	NEG	NEG	NEG	POS	POS	POS	NEG	AMB	POS	POS	NEG	NEG	NEG	POS
LIT76 (Lithuania)	POS	POS	POS	NEG	NEG	NEG	NEG	POS	POS	POS	NEG	AMB	POS	POS	NEG	NEG	NEG	POS
HGSA142 (Portugal)	NEG	NEG	NEG	NEG	NEG	NEG	NEG	POS	POS	POS	NEG	AMB	POS	POS	NEG	NEG	NEG	POS
HGSA9 (Portugal)	POS	POS	POS	NEG	NEG	NEG	NEG	POS	POS	POS	NEG	AMB	POS	POS	NEG	NEG	NEG	POS
HSJ216 (Portugal)	POS	POS	POS	NEG	NEG	NEG	NEG	POS	POS	POS	NEG	AMB	POS	POS	NEG	NEG	NEG	POS
M278 (Portugal)	POS	POS	POS	NEG	NEG	NEG	NEG	POS	POS	POS	NEG	AMB	POS	POS	NEG	NEG	NEG	POS
ES26 (Spain)	POS	POS	POS	NEG	NEG	NEG	NEG	POS	POS	POS	NEG	AMB	POS	POS	NEG	NEG	NEG	POS
UK102 (UK)	POS	POS	POS	NEG	NEG	NEG	NEG	POS	POS	POS	NEG	AMB	POS	POS	NEG	NEG	NEG	POS
URU34 (Uruguay)	POS	POS	POS	NEG	NEG	NEG	NEG	POS	POS	POS	NEG	AMB	POS	POS	NEG	NEG	NEG	POS
IS-125	POS	POS	POS	NEG	NEG	NEG	NEG	POS	POS	POS	NEG	AMB	POS	POS	NEG	NEG	NEG	POS
IS-157	POS	POS	POS	NEG	NEG	NEG	NEG	POS	POS	POS	NEG	AMB	POS	POS	NEG	NEG	NEG	POS
Ecuador_1	POS	POS	POS	NEG	AMB	NEG	NEG	POS	POS	POS	AMB	AMB	POS	POS	NEG	NEG	POS	POS
Ecuador_2	POS	POS	POS	NEG	NEG	NEG	NEG	POS	POS	POS	AMB	AMB	POS	POS	NEG	NEG	POS	POS
Dresden_15ANR574263	POS	POS	POS	NEG	NEG	NEG	NEG	POS	POS	POS	AMB	AMB	POS	POS	NEG	NEG	POS	POS
UK-EMRSA-11	POS	POS	POS	NEG	AMB	NEG	NEG	POS	POS	POS	AMB	AMB	POS	POS	NEG	NEG	POS	POS
>"South American/ Mid																		
BRA2 (Brazil)	POS	POS	POS	NEG	NEG	NEG	NEG	POS	POS	POS	NEG	AMB	POS	POS	NEG	NEG	NEG	POS
CHL1 (Chile)	POS	POS	POS	NEG	NEG	NEG	NEG	POS	POS	POS	NEG	AMB	POS	POS	NEG	NEG	NEG	POS
CHL151 (Chile)	POS	POS	POS	NEG	NEG	NEG	NEG	POS	POS	POS	NEG	AMB	POS	POS	NEG	NEG	NEG	POS
UCL378 (Chile)	POS	POS	POS	NEG	NEG	NEG	NEG	POS	POS	POS	NEG	AMB	POS	POS	NEG	NEG	NEG	POS
UP72 (Peru)	POS	POS	POS	NEG	NEG	NEG	NEG	POS	POS	POS	NEG	AMB	POS	POS	NEG	NEG	NEG	POS
UP81 (Peru)	POS	POS	POS	NEG	NEG	NEG	NEG	POS	POS	POS	NEG	AMB	POS	POS	NEG	NEG	NEG	POS
>"South American/ Mid																		
UP1073 (Peru)	POS	POS	POS	NEG	NEG	NEG	NEG	POS	POS	POS	NEG	AMB	POS	POS	NEG	NEG	NEG	POS

STRAIN / ISOLATE	ADHAESION FACTORS / GENES ENCODING MICROBIAL SURFACE COMPONENTS RECOGNIZING ADHESIVE MATRIX MOLECULES (MSCRAMM GENES)																	
	bbp							clfA					clfB					cna
	bbp	bbp (cons)	bbp (COL+MW2)	bbp (MRSA252)	bbp (Mu50)	bbp (RF122)	bbp (ST45)	clfA	clfA (cons)	clfA (COL+RF122)	clfA (MRSA252)	clfA (Mu50+MW2)	clfB	clfB (cons)	clfB (COL+Mu50)	clfB (MW2)	clfB (RF122)	cna
	Bone sialoprotein-binding protein							Clumping factor A					Clumping factor B					Collagen-binding adhesin
>"South American/ Mid																		
LIT89 (Lithuania)	POS	POS	POS	NEG	NEG	NEG	NEG	POS	POS	POS	NEG	AMB	POS	POS	NEG	NEG	NEG	POS
MRSA_PR1 (Malaysia)	NEG	NEG	NEG	NEG	NEG	NEG	NEG	POS	POS	POS	NEG	AMB	POS	POS	NEG	NEG	NEG	POS
Riyadh-2888905-R	POS	POS	POS	NEG	AMB	NEG	NEG	POS	POS	POS	AMB	AMB	POS	POS	NEG	NEG	AMB	POS
Riyadh-2888915-BC	POS	POS	POS	NEG	NEG	NEG	NEG	POS	POS	POS	AMB	AMB	POS	POS	NEG	NEG	AMB	POS
Riyadh-2793706-R	POS	POS	POS	NEG	NEG	NEG	NEG	POS	POS	AMB	AMB	POS	POS	POS	NEG	NEG	POS	POS
>"South American/ Mid																		
Riyadh_SSTI_18_3502925	POS	POS	POS	NEG	AMB	NEG	NEG	POS	POS	AMB	POS	AMB	POS	POS	NEG	NEG	POS	POS
Riyadh-2822825-W	POS	POS	POS	NEG	AMB	NEG	NEG	POS	POS	POS	AMB	AMB	POS	POS	AMB	NEG	POS	POS
>"South American/ Mid																		
UK-EMRSA-9	POS	POS	POS	NEG	AMB	NEG	NEG	POS	POS	POS	AMB	AMB	POS	POS	NEG	NEG	POS	POS
>"South American/ Mid																		
Lome_HT20020815	POS	POS	POS	NEG	AMB	NEG	NEG	POS	POS	POS	AMB	AMB	POS	POS	NEG	NEG	POS	POS
>"South American/ Mid																		
NA32 (Denmark)	POS	POS	POS	NEG	NEG	NEG	NEG	POS	POS	POS	NEG	AMB	POS	POS	NEG	NEG	NEG	POS
H24 (Egypt)	POS	POS	POS	NEG	NEG	NEG	NEG	POS	POS	POS	NEG	AMB	POS	POS	NEG	NEG	NEG	POS
ATCC BAA-39 (=HUSA304) (Hungary)	POS	POS	POS	NEG	NEG	NEG	NEG	POS	POS	POS	NEG	AMB	POS	POS	NEG	NEG	NEG	POS
HUSA304 (Hungary)	POS	POS	POS	NEG	NEG	NEG	NEG	POS	POS	POS	NEG	AMB	POS	POS	NEG	NEG	NEG	POS
HU106 (Hungary)	POS	POS	POS	NEG	NEG	NEG	NEG	POS	POS	POS	NEG	AMB	POS	POS	NEG	NEG	NEG	POS
BSAC27 (UK)	POS	POS	POS	NEG	NEG	NEG	NEG	POS	POS	POS	NEG	AMB	POS	POS	NEG	NEG	NEG	POS
NCTC13131, UK-EMRSA-4 (UK)	POS	POS	POS	NEG	NEG	NEG	NEG	POS	POS	POS	NEG	AMB	POS	POS	NEG	NEG	NEG	POS
	POS	POS	POS	NEG	AMB	NEG	NEG	POS	POS	POS	AMB	AMB	POS	POS	NEG	NEG	POS	POS
Algiers_HT20040080	POS	POS	POS	NEG	AMB	NEG	NEG	POS	POS	POS	AMB	AMB	POS	POS	NEG	NEG	POS	POS
Dublin-DSH_AR09_0.0066	POS	POS	POS	NEG	AMB	NEG	NEG	POS	POS	POS	AMB	AMB	POS	POS	NEG	NEG	POS	POS
Dublin-DSH_AR09_0-0065	POS	POS	POS	NEG	NEG	NEG	NEG	POS	POS	POS	NEG	AMB	POS	POS	NEG	NEG	NEG	POS
Dublin-DSH_Phenotype-III_84	POS	POS	POS	NEG	NEG	NEG	NEG	POS	POS	POS	AMB	AMB	POS	POS	NEG	NEG	NEG	POS
Hong Kong_130	POS	POS	POS	NEG	NEG	NEG	NEG	POS	POS	POS	AMB	POS	POS	POS	NEG	NEG	AMB	POS
Kuwait_018	POS	POS	POS	NEG	NEG	NEG	NEG	POS	POS	POS	AMB	AMB	POS	POS	NEG	NEG	AMB	POS
Perth_08-17726	POS	POS	POS	NEG	AMB	NEG	NEG	POS	POS	POS	AMB	AMB	POS	POS	NEG	NEG	POS	POS
Riyadh_Alfaisal/KKKSUH_86_MRS-14-279	POS	POS	POS	NEG	NEG	NEG	NEG	POS	POS	POS	AMB	AMB	POS	POS	NEG	NEG	NEG	POS
Riyadh_Alfaisal-04_Z3861_831588	POS	POS	POS	NEG	AMB	NEG	NEG	POS	POS	POS	AMB	AMB	POS	POS	NEG	NEG	POS	POS
Riyadh_Alfaisal-30_S15724_1108013	POS	POS	POS	NEG	AMB	NEG	NEG	POS	POS	POS	AMB	AMB	POS	POS	NEG	NEG	POS	POS
Riyadh_Alfaisal-6_22A_13_B3992_397721	POS	POS	POS	NEG	AMB	NEG	NEG	POS	POS	POS	AMB	AMB	POS	POS	NEG	NEG	POS	POS
Riyadh_SSTI_52_3615482	POS	POS	POS	NEG	AMB	NEG	NEG	POS	POS	POS	AMB	AMB	POS	POS	NEG	NEG	POS	POS
Riyadh-2817276-2	POS	POS	POS	NEG	AMB	NEG	NEG	POS	POS	AMB	POS	AMB	POS	POS	NEG	NEG	POS	POS
Riyadh-2891670-W	POS	POS	POS	NEG	AMB	NEG	AMB	POS	POS	POS	AMB	AMB	POS	POS	NEG	NEG	POS	POS
Riyadh-3006920-W	POS	POS	POS	NEG	NEG	NEG	NEG	POS	POS	POS	AMB	AMB	POS	POS	NEG	NEG	POS	POS
Riyadh-R2567782	POS	POS	POS	NEG	NEG	NEG	NEG	POS	POS	POS	AMB	AMB	POS	POS	NEG	NEG	POS	POS
Russia-18_0252_Krasnoyarsk_SK2	POS	POS	POS	NEG	NEG	NEG	NEG	POS	POS	POS	AMB	AMB	POS	POS	NEG	NEG	NEG	POS
UK-EMRSA-7	POS	POS	POS	NEG	AMB	NEG	NEG	POS	POS	POS	AMB	AMB	POS	POS	NEG	NEG	POS	POS
>"South American/ Mid																		
Dublin-DSH_AR23_0073	POS	POS	POS	NEG	NEG	NEG	NEG	POS	POS	POS	AMB	AMB	POS	POS	NEG	NEG	NEG	POS
>Related to "South Ame																		
MRSA-OC3 (Russia)	POS	POS	POS	NEG	NEG	NEG	NEG	POS	POS	POS	NEG	AMB	POS	POS	NEG	NEG	NEG	POS
Russia-12_0176_Krasnoyarsk	POS	POS	POS	NEG	AMB	NEG	NEG	POS	POS	POS	AMB	AMB	POS	POS	NEG	NEG	POS	POS
Russia-13_0180_Krasnoyarsk	POS	POS	POS	NEG	AMB	NEG	NEG	POS	POS	POS	AMB	AMB	POS	POS	NEG	NEG	POS	POS
Russia-16_0249_Krasnoyarsk	POS	POS	POS	NEG	AMB	NEG	NEG	POS	POS	POS	AMB	AMB	POS	POS	NEG	NEG	POS	POS
Russia-17_0250_Krasnoyarsk	POS	POS	POS	NEG	AMB	NEG	NEG	POS	POS	POS	AMB	AMB	POS	POS	NEG	NEG	POS	POS
>Related to "South Ame																		
URU110 (Uruguay)	POS	POS	POS	NEG	NEG	NEG	NEG	POS	POS	POS	NEG	AMB	POS	POS	NEG	NEG	NEG	POS
>Related to "South Ame																		
DS_014 (Thailand)	POS	POS	POS	NEG	NEG	NEG	NEG	POS	POS	POS	NEG	AMB	POS	POS	NEG	NEG	NEG	POS
>Unassigned Middle Eas																		
Frankfurt_Oder_0490031797 (Libya)	POS	POS	POS	NEG	NEG	NEG	NEG	POS	POS	AMB	POS	AMB	POS	POS	NEG	NEG	POS	POS
Kuwait_192	POS	POS	POS	NEG	AMB	NEG	NEG	POS	POS	AMB	AMB	POS	POS	POS	NEG	NEG	POS	POS
Riyadh-9	POS	POS	POS	NEG	AMB	NEG	NEG	POS	POS	POS	AMB	AMB	POS	POS	NEG	NEG	POS	POS
Russia-24_0407_Moscow	POS	POS	POS	NEG	AMB	NEG	NEG	POS	POS	POS	AMB	AMB	POS	POS	NEG	NEG	POS	POS

STRAIN / ISOLATE	ADHAESION FACTORS / GENES ENCODING MICROBIAL SURFACE COMPONENTS RECOGNIZING ADHESIVE MATRIX MOLECULES														
	ebh	ebpS					eno	efb		fnbA					
	ebh (cons)	ebpS	ebpS_probe 612	ebpS_probe 614	ebpS (01-1111)	ebpS (COL)	eno	efb / fib	efb / fib (MRSA252)	fnbA	fnbA (cons)	fnbA (COL)	fnbA (MRSA252)	fnbA (Mu50+MW2)	fnbA (RF122)
	Cell wall associated fibronectin-binding protein	cell surface elastin binding protein					enolase	fibrinogen binding protein (19 kDa)		fibronectin-binding protein A					
>"Eurasian Clade": TUR1															
TUR1 (Turkey)	POS	POS	POS	POS	NEG	POS	POS	POS	NEG	POS	POS	POS	NEG	NEG	NEG
TUR9 (Turkey)	POS	POS	POS	POS	NEG	POS	POS	POS	NEG	POS	POS	POS	NEG	NEG	NEG
>"Eurasian Clade" : T013															
T0131 (China)	POS	POS	POS	POS	NEG	POS	POS	POS	NEG	POS	POS	POS	NEG	NEG	NEG
MU4 (Turkey)	POS	POS	POS	POS	NEG	POS	POS	POS	NEG	POS	POS	POS	NEG	NEG	NEG
DEU11 (Turkey)	POS	POS	POS	POS	NEG	POS	POS	POS	NEG	POS	POS	POS	NEG	NEG	NEG
DEU16 (Turkey)	POS	POS	POS	POS	NEG	POS	POS	POS	NEG	POS	POS	POS	NEG	NEG	NEG
Romania_Iasi_MRSA-07_54	POS	POS	POS	POS	NEG	POS	POS	POS	NEG	POS	POS	POS	NEG	NEG	NEG
>"Eurasian Clade": arsC-															
Romania_Iasi_MRSA-05_46	POS	POS	POS	POS	NEG	POS	POS	POS	NEG	POS	POS	POS	NEG	NEG	NEG
Romania_Iasi_MRSA-06_47	POS	POS	POS	POS	NEG	POS	POS	POS	AMB	POS	POS	POS	NEG	NEG	NEG
>"Eurasian Clade": 16K/															
CN79 (China)	POS	POS	POS	POS	NEG	POS	POS	POS	NEG	POS	POS	POS	NEG	NEG	NEG
CUHK_BJ2002 (China)	POS	POS	POS	POS	NEG	POS	POS	POS	NEG	POS	POS	POS	NEG	NEG	NEG
CUHK_BJ2007 (China)	POS	POS	POS	POS	NEG	POS	POS	POS	NEG	POS	POS	POS	NEG	NEG	NEG
3HK (Czech Republic)	POS	POS	POS	POS	NEG	POS	POS	POS	NEG	POS	POS	POS	NEG	NEG	NEG
HU109 (Hungary)	POS	POS	POS	POS	NEG	POS	POS	POS	NEG	POS	POS	POS	NEG	NEG	NEG
HUR18 (Hungary)	POS	POS	POS	POS	NEG	POS	POS	POS	NEG	POS	POS	POS	NEG	NEG	NEG
H482 (Romania)	POS	POS	POS	POS	NEG	POS	POS	POS	NEG	POS	POS	POS	NEG	NEG	NEG
16K (Russia)	POS	POS	POS	POS	NEG	POS	POS	POS	NEG	POS	POS	POS	NEG	NEG	NEG
DEU10 (Turkey)	POS	POS	POS	POS	NEG	POS	POS	POS	NEG	POS	POS	POS	NEG	NEG	NEG
DEU12 (Turkey)	POS	POS	POS	POS	NEG	POS	POS	POS	NEG	POS	POS	POS	NEG	NEG	NEG
DEU14 (Turkey)	POS	POS	POS	POS	NEG	POS	POS	POS	NEG	POS	POS	POS	NEG	NEG	NEG
DEU15 (Turkey)	POS	POS	POS	POS	NEG	POS	POS	POS	NEG	POS	POS	POS	NEG	NEG	NEG
DEU17 (Turkey)	POS	POS	POS	POS	NEG	POS	POS	POS	NEG	POS	POS	POS	NEG	NEG	NEG
DEU19 (Turkey)	POS	POS	POS	POS	NEG	POS	POS	POS	NEG	POS	POS	POS	NEG	NEG	NEG
DEU2 (Turkey)	POS	POS	POS	POS	NEG	POS	POS	POS	NEG	POS	POS	POS	NEG	NEG	NEG
DEU20 (Turkey)	POS	POS	POS	POS	NEG	POS	POS	POS	NEG	POS	POS	POS	NEG	NEG	NEG
DEU23 (Turkey)	POS	POS	POS	POS	NEG	POS	POS	POS	NEG	POS	POS	POS	NEG	NEG	NEG
DEU3 (Turkey)	POS	POS	POS	POS	NEG	POS	POS	POS	NEG	POS	POS	POS	NEG	NEG	NEG
DEU5 (Turkey)	POS	POS	POS	POS	NEG	POS	POS	POS	NEG	POS	POS	POS	NEG	NEG	NEG
DEU6 (Turkey)	POS	POS	POS	POS	NEG	POS	POS	POS	NEG	POS	POS	POS	NEG	NEG	NEG
DEU8 (Turkey)	POS	POS	POS	POS	NEG	POS	POS	POS	NEG	POS	POS	POS	NEG	NEG	NEG
HU11 (Turkey)	POS	POS	POS	POS	NEG	POS	POS	POS	NEG	POS	POS	POS	NEG	NEG	NEG
HU13 (Turkey)	POS	POS	POS	POS	NEG	POS	POS	POS	NEG	POS	POS	POS	NEG	NEG	NEG
HU14 (Turkey)	POS	POS	POS	POS	NEG	POS	POS	POS	NEG	POS	POS	POS	NEG	NEG	NEG
HU15 (Turkey)	POS	POS	POS	POS	NEG	POS	POS	POS	NEG	POS	POS	POS	NEG	NEG	NEG
HU16 (Turkey)	POS	POS	POS	POS	NEG	POS	POS	POS	NEG	POS	POS	POS	NEG	NEG	NEG
HU17 (Turkey)	POS	POS	POS	POS	NEG	POS	POS	POS	NEG	POS	POS	POS	NEG	NEG	NEG
HU21 (Turkey)	POS	POS	POS	POS	NEG	POS	POS	POS	NEG	POS	POS	POS	NEG	NEG	NEG
HU23 (Turkey)	POS	POS	POS	POS	NEG	POS	POS	POS	NEG	POS	POS	POS	NEG	NEG	NEG
HU26 (Turkey)	POS	POS	POS	POS	NEG	POS	POS	POS	NEG	POS	POS	POS	NEG	NEG	NEG
HU4 (Turkey)	POS	POS	POS	POS	NEG	POS	POS	POS	NEG	POS	POS	POS	NEG	NEG	NEG
HU5 (Turkey)	POS	POS	POS	POS	NEG	POS	POS	POS	NEG	POS	POS	POS	NEG	NEG	NEG
HU6 (Turkey)	POS	POS	POS	POS	NEG	POS	POS	POS	NEG	POS	POS	POS	NEG	NEG	NEG
HU7 (Turkey)	POS	POS	POS	POS	NEG	POS	POS	POS	NEG	POS	POS	POS	NEG	NEG	NEG
HU8 (Turkey)	POS	POS	POS	POS	NEG	POS	POS	POS	NEG	POS	POS	POS	NEG	NEG	NEG
HU9 (Turkey)	POS	POS	POS	POS	NEG	POS	POS	POS	NEG	POS	POS	POS	NEG	NEG	NEG
IU1 (Turkey)	POS	POS	POS	POS	NEG	POS	POS	POS	NEG	POS	POS	POS	NEG	NEG	NEG
IU10 (Turkey)	POS	POS	POS	POS	NEG	POS	POS	POS	NEG	POS	POS	POS	NEG	NEG	NEG
IU11 (Turkey)	POS	POS	POS	POS	NEG	POS	POS	POS	NEG	POS	POS	POS	NEG	NEG	NEG
IU12 (Turkey)	POS	POS	POS	POS	NEG	POS	POS	POS	NEG	POS	POS	POS	NEG	NEG	NEG
IU13 (Turkey)	POS	POS	POS	POS	NEG	POS	POS	POS	NEG	POS	POS	POS	NEG	NEG	NEG
IU15 (Turkey)	POS	POS	POS	POS	NEG	POS	POS	POS	NEG	POS	POS	POS	NEG	NEG	NEG
IU18 (Turkey)	POS	POS	POS	POS	NEG	POS	POS	POS	NEG	POS	POS	POS	NEG	NEG	NEG
IU19 (Turkey)	POS	POS	POS	POS	NEG	POS	POS	POS	NEG	POS	POS	POS	NEG	NEG	NEG
IU2 (Turkey)	POS	POS	POS	POS	NEG	POS	POS	POS	NEG	POS	POS	POS	NEG	NEG	NEG
IU4 (Turkey)	POS	POS	POS	POS	NEG	POS	POS	POS	NEG	POS	POS	POS	NEG	NEG	NEG
IU6 (Turkey)	POS	POS	POS	POS	NEG	POS	POS	POS	NEG	POS	POS	POS	NEG	NEG	NEG
IU7 (Turkey)	POS	POS	POS	POS	NEG	POS	POS	POS	NEG	POS	POS	POS	NEG	NEG	NEG
IU9 (Turkey)	POS	POS	POS	POS	NEG	POS	POS	POS	NEG	POS	POS	POS	NEG	NEG	NEG
MU1 (Turkey)	POS	POS	POS	POS	NEG	POS	POS	POS	NEG	POS	POS	POS	NEG	NEG	NEG
MU10 (Turkey)	POS	POS	POS	POS	NEG	POS	POS	POS	NEG	POS	POS	POS	NEG	NEG	NEG
MU20 (Turkey)	POS	POS	POS	POS	NEG	POS	POS	POS	NEG	POS	POS	POS	NEG	NEG	NEG
Mu3 (Turkey)	POS	POS	POS	POS	NEG	POS	POS	POS	NEG	POS	POS	POS	NEG	NEG	NEG
MU5 (Turkey)	POS	POS	POS	POS	NEG	POS	POS	POS	NEG	POS	POS	POS	NEG	NEG	NEG
MU6 (Turkey)	POS	POS	POS	POS	NEG	POS	POS	POS	NEG	POS	POS	POS	NEG	NEG	NEG
MU7 (Turkey)	POS	POS	POS	POS	NEG	POS	POS	POS	NEG	POS	POS	POS	NEG	NEG	NEG
TUR27 (Turkey)	POS	POS	POS	POS	NEG	POS	POS	POS	NEG	POS	POS	POS	NEG	NEG	NEG
NCTR 32S	POS	POS	POS	POS	NEG	POS	POS	POS	NEG	POS	POS	POS	NEG	NEG	NEG
SA02_A4 = Russia-06_0085_Moscow	POS	POS	POS	POS	NEG	POS	POS	POS	NEG	POS	POS	POS	NEG	NEG	NEG
	POS	POS	POS	POS	NEG	POS	POS	POS	NEG	POS	POS	POS	NEG	NEG	NEG
Dresden_08V35987 (Turkey)	POS	POS	POS	POS	NEG	POS	POS	POS	NEG	POS	POS	POS	NEG	NEG	NEG
Dresden_17ANR577152	POS	POS	POS	POS	NEG	POS	POS	POS	NEG	POS	POS	POS	NEG	NEG	NEG
Dresden_17ANR580374 (Makedonia)	POS	POS	POS	POS	NEG	POS	POS	POS	NEG	POS	POS	POS	NEG	NEG	NEG
Hong Kong_69-II	POS	AMB	POS	POS	NEG	POS	POS	POS	NEG	POS	POS	POS	NEG	NEG	NEG
Hong Kong_93	POS	AMB	POS	POS	NEG	POS	POS	POS	NEG	POS	POS	POS	NEG	NEG	NEG
Rawalpindi_Kidney Center_03_SK1	POS	POS	POS	POS	NEG	POS	POS	POS	NEG	POS	POS	POS	NEG	NEG	NEG
Rawalpindi_Kidney Center_08	POS	POS	POS	POS	NEG	POS	POS	POS	NEG	POS	POS	POS	NEG	NEG	NEG
Rawalpindi_Kidney Center_10	POS	POS	POS	POS	NEG	POS	POS	POS	NEG	POS	POS	POS	NEG	NEG	NEG
Rawalpindi_Kidney Center_19	POS	POS	POS	POS	NEG	POS	POS	POS	AMB	POS	POS	POS	NEG	NEG	NEG
Rawalpindi_Kidney Center_50_SK1	POS	POS	POS	POS	NEG	POS	POS	POS	NEG	POS	POS	POS	NEG	NEG	NEG
Romania_Iasi_BC-49_430	POS	POS	POS	POS	NEG	POS	POS	POS	AMB	POS	POS	POS	NEG	NEG	NEG
Romania_Iasi_MRSA_284	POS	POS	POS	POS	NEG	POS	POS	POS	NEG	POS	POS	POS	NEG	NEG	NEG
Romania_Iasi_MRSA-32_318	POS	POS	POS	POS	NEG	POS	POS	POS	NEG	POS	POS	POS	NEG	NEG	NEG
Romania_Iasi_MRSA-54_430	POS	POS	POS	POS	NEG	POS	POS	POS	NEG	POS	POS	POS	NEG	NEG	NEG
Romania_Iasi_SSTI-01_2	POS	POS	POS	POS	NEG	POS	POS	POS	NEG	POS	POS	POS	NEG	NEG	NEG
Romania_Iasi_SSTI-10_101	POS	POS	POS	POS	NEG	POS	POS	POS	NEG	POS	POS	POS	NEG	NEG	NEG
Romania_Iasi_SSTI-11_106	POS	POS	POS	POS	NEG	POS	POS	POS	NEG	POS	POS	POS	NEG	NEG	NEG
Russia-01_0001_SaintPetersburg	POS	POS	POS	POS	NEG	POS	POS	POS	NEG	POS	POS	POS	NEG	NEG	NEG
Russia-02_0004_SaintPetersburg	POS	POS	POS	POS	NEG	POS	POS	POS	NEG	POS	POS	POS	NEG	NEG	NEG
Russia-03_0057_SaintPetersburg	POS	POS	POS	POS	NEG	POS	POS	POS	NEG	POS	POS	POS	NEG	NEG	NEG
Russia-04_0076_Moscow	POS	POS	POS	POS	NEG	POS	POS	POS	NEG	POS	POS	POS	NEG	NEG	NEG
Russia-05_0078_Moscow	POS	POS	POS	POS	NEG	POS	POS	POS	NEG	POS	POS	POS	NEG	NEG	NEG
Russia-08_0135_Moscow	POS	POS	POS	POS	NEG	POS	POS	POS	NEG	POS	POS	POS	NEG	NEG	NEG
Russia-09_0150_Kurgan	POS	POS	POS	POS	NEG	POS	POS	POS	NEG	POS	POS	POS	NEG	NEG	NEG
Russia-10_0162_Kurgan	POS	POS	POS	POS	NEG	POS	POS	POS	NEG	POS	POS	POS	NEG	NEG	NEG
Russia-14_0184_Moscow	POS	POS	POS	POS	NEG	POS	POS	POS	NEG	POS	POS	POS	NEG	NEG	NEG
Russia-15_0232_SaintPetersburg	POS	POS	POS	POS	NEG	POS	POS	POS	NEG	POS	POS	POS	NEG	NEG	NEG
Russia-22_0391_Chelyabinsk	POS	POS	POS	POS	NEG	POS	POS	POS	NEG	POS	POS	POS	NEG	NEG	NEG
Russia-23_0392_Chelyabinsk	POS	POS	POS	POS	NEG	POS	POS	POS	NEG	POS	POS	POS	NEG	NEG	NEG
>"Eurasian Clade" : IU17															
IU17 (Turkey)	POS	POS	POS	POS	NEG	POS	POS	POS	NEG	POS	POS	POS	NEG	NEG	NEG
IU20 (Turkey)	POS	POS	POS	POS	NEG	POS	POS	POS	NEG	POS	POS	POS	NEG	NEG	NEG

STRAIN / ISOLATE	ADHAESION FACTORS / GENES ENCODING MICROBIAL SURFACE COMPONENTS RECOGNIZING ADHESIVE MATRIX MOLECULES														
	ebh	ebpS					eno	efb		fnbA					
	ebh (cons)	ebpS	ebpS_probe 612	ebpS_probe 614	ebpS (01-1111)	ebpS (COL)	eno	efb / fib	efb / fib (MRSA252)	fnbA	fnbA (cons)	fnbA (COL)	fnbA (MRSA252)	fnbA (Mu50+MW2)	fnbA (RF122)
	Cell wall associated fibronectin-binding protein	cell surface elastin binding protein					enolase	fibrinogen binding protein (19 kDa)		fibronectin-binding protein A					
**>"Eurasian Clade": Spora**															
Dresden_16ANR581769	POS	POS	POS	POS	NEG	POS	POS	POS	NEG	POS	POS	POS	NEG	NEG	NEG
Dresden_17ANR578769	POS	POS	POS	POS	NEG	POS	POS	POS	NEG	POS	POS	POS	NEG	NEG	NEG
**>"Eurasian Clade": Kuwa**															
Kuwait_306	POS	POS	POS	POS	NEG	POS	POS	POS	NEG	POS	POS	POS	NEG	NEG	NEG
Kuwait_301	POS	POS	POS	POS	NEG	POS	POS	POS	AMB	POS	POS	POS	NEG	NEG	NEG
**>"European Clade": "Gre**															
GRE18 (Greece)	POS	POS	POS	POS	NEG	POS	POS	POS	NEG	NEG	NEG	NEG	NEG	NEG	NEG
GRE317 (Greece)	POS	POS	POS	POS	NEG	POS	POS	POS	NEG	NEG	NEG	NEG	NEG	NEG	NEG
GRE4 (Greece)	POS	POS	POS	POS	NEG	POS	POS	POS	NEG	NEG	NEG	NEG	NEG	NEG	NEG
R35 (USA)	POS	POS	POS	POS	NEG	POS	POS	POS	NEG	POS	POS	POS	NEG	NEG	NEG
Casablanca HT20060548	POS	POS	POS	POS	NEG	POS	POS	POS	NEG	NEG	NEG	NEG	NEG	NEG	NEG
Casablanca HT20060550	POS	POS	POS	POS	NEG	POS	POS	POS	NEG	NEG	NEG	NEG	NEG	NEG	NEG
Dresden_01V34572 (Greece)	POS	AMB	AMB	POS	NEG	POS	POS	POS	NEG	NEG	NEG	NEG	NEG	NEG	NEG
Dresden_01V36469	POS	POS	POS	POS	NEG	POS	POS	POS	NEG	NEG	NEG	NEG	NEG	NEG	NEG
Dresden 01V39123	POS	POS	POS	POS	NEG	POS	POS	POS	NEG	NEG	NEG	NEG	NEG	NEG	NEG
Dresden 01V39143	POS	POS	POS	POS	NEG	POS	POS	POS	NEG	NEG	NEG	NEG	NEG	NEG	NEG
Greece 1 3680 Harmony	POS	POS	NEG	POS	NEG	POS	POS	POS	NEG	NEG	NEG	NEG	NEG	NEG	NEG
**>"European Clade": UK-**															
ANS46 (Australia)	POS	POS	POS	POS	NEG	POS	POS	POS	NEG	POS	POS	POS	NEG	NEG	NEG
LHH1 (USA)	POS	POS	POS	POS	NEG	POS	POS	POS	NEG	POS	POS	POS	NEG	NEG	NEG
Dublin-DSH_AR01_0-1085	POS	POS	POS	POS	NEG	POS	POS	POS	NEG	POS	POS	POS	NEG	NEG	NEG
Dublin-DSH_AR01_0-1118	POS	POS	POS	POS	NEG	POS	POS	POS	NEG	POS	POS	POS	NEG	NEG	NEG
Dublin-DSH_AR15_0098	POS	POS	POS	POS	NEG	POS	POS	POS	NEG	POS	POS	POS	NEG	NEG	NEG
Dublin-DSH_AR15_0104	POS	AMB	POS	POS	NEG	POS	POS	POS	NEG	POS	POS	POS	NEG	NEG	NEG
UK_NCTC11939_UK-1_Harmony	POS	POS	POS	POS	NEG	POS	POS	POS	NEG	POS	POS	POS	NEG	NEG	NEG
**>"European Clade": BK2**															
BK2421 (USA)	POS	POS	POS	POS	NEG	POS	POS	POS	NEG	POS	POS	POS	NEG	NEG	NEG
**>"Aussi/NZ Clade", JKD6**															
JKD6009 (Australia)	POS	POS	POS	POS	NEG	POS	POS	POS	NEG	POS	POS	POS	NEG	NEG	NEG
JKD6008 (New Zealand)	POS	POS	POS	POS	NEG	POS	POS	POS	NEG	POS	POS	POS	NEG	NEG	NEG
Perth_2015-S20209	POS	POS	POS	POS	NEG	POS	POS	POS	NEG	POS	POS	POS	NEG	NEG	NEG
Perth_2015-S20305	POS	POS	POS	POS	NEG	POS	POS	POS	NEG	POS	POS	POS	NEG	NEG	NEG
Perth_2015-S20603	POS	POS	POS	POS	NEG	POS	POS	POS	NEG	POS	POS	POS	NEG	NEG	NEG
Perth_2015-S20607	POS	POS	POS	POS	NEG	POS	POS	POS	NEG	POS	POS	POS	NEG	NEG	NEG
Perth_2015-S20608	POS	POS	POS	POS	NEG	POS	POS	POS	NEG	POS	POS	POS	NEG	NEG	NEG
Perth_2015-S20610	POS	POS	POS	POS	NEG	POS	POS	POS	NEG	POS	POS	POS	NEG	NEG	NEG
Perth_2015-S20613	POS	POS	POS	POS	NEG	POS	POS	POS	NEG	POS	POS	POS	NEG	NEG	NEG
Perth_2015-S20617	POS	POS	POS	POS	NEG	POS	POS	POS	NEG	POS	POS	POS	NEG	NEG	NEG
Perth_2015-S20622	POS	POS	POS	POS	NEG	POS	POS	POS	NEG	POS	POS	POS	NEG	NEG	NEG
Perth_2015-S23619	POS	POS	POS	POS	NEG	POS	POS	POS	NEG	POS	POS	POS	NEG	NEG	NEG
Perth_2015-S41210	POS	POS	POS	POS	NEG	POS	POS	POS	NEG	POS	POS	POS	NEG	NEG	NEG
Perth_2015-S51001	POS	POS	POS	POS	NEG	POS	POS	POS	NEG	POS	POS	POS	NEG	NEG	NEG
Perth_2015-S51003	POS	POS	POS	POS	NEG	POS	POS	POS	NEG	POS	POS	POS	NEG	NEG	NEG
Perth_2015-S51006	POS	POS	POS	POS	NEG	POS	POS	POS	NEG	POS	POS	POS	NEG	NEG	NEG
Perth_2015-S51007	POS	POS	POS	POS	NEG	POS	POS	POS	NEG	POS	POS	POS	NEG	NEG	NEG
Perth_2015-S51008	POS	POS	POS	POS	NEG	POS	POS	POS	NEG	POS	POS	POS	NEG	NEG	NEG
Perth_2015-S51014	POS	POS	POS	POS	NEG	POS	POS	NEG	NEG	POS	POS	POS	NEG	NEG	NEG
Perth_2015-S51408	POS	POS	POS	POS	NEG	POS	POS	POS	NEG	POS	POS	POS	NEG	NEG	NEG
Perth_2016-S20614	POS	POS	POS	POS	NEG	POS	POS	POS	NEG	POS	POS	POS	NEG	NEG	NEG
Perth_2016-S23614	POS	POS	POS	POS	NEG	POS	POS	POS	NEG	POS	POS	POS	NEG	NEG	NEG
Perth_2016-S42009	POS	POS	POS	POS	NEG	POS	POS	POS	NEG	POS	POS	POS	NEG	NEG	NEG
Perth_2016-S51001	POS	POS	POS	POS	NEG	POS	POS	POS	NEG	POS	POS	POS	NEG	NEG	NEG
Perth_2016-S51403	POS	POS	POS	POS	NEG	POS	POS	POS	NEG	POS	POS	POS	NEG	NEG	NEG
Perth_07 RPA 20	POS	POS	POS	POS	NEG	POS	POS	POS	NEG	POS	POS	POS	NEG	NEG	NEG
Perth_08-19088	POS	POS	POS	POS	NEG	POS	POS	POS	NEG	POS	POS	POS	NEG	NEG	NEG
Perth_01-15351	POS	POS	POS	POS	NEG	POS	POS	POS	NEG	POS	POS	POS	NEG	NEG	NEG
Perth_01-15357	POS	POS	AMB	POS	NEG	POS	POS	POS	NEG	POS	POS	POS	NEG	NEG	NEG
Perth_01-15419	POS	AMB	POS	POS	NEG	POS	POS	POS	NEG	POS	POS	POS	NEG	NEG	NEG
Perth_01-16309	POS	POS	POS	POS	NEG	POS	POS	POS	NEG	POS	POS	POS	NEG	NEG	NEG
Perth_03 SNP 95	POS	POS	POS	POS	NEG	POS	POS	POS	POS	POS	POS	POS	NEG	NEG	NEG
Perth_2003_AH_47	POS	POS	POS	POS	NEG	POS	POS	POS	NEG	POS	POS	POS	NEG	NEG	NEG
**>"Australian/NZ Clade":**															
Perth_2015-S20623	POS	POS	POS	POS	NEG	POS	POS	POS	NEG	POS	POS	POS	NEG	NEG	NEG
Perth_2015-S20826	POS	POS	POS	POS	NEG	POS	POS	POS	NEG	POS	POS	POS	NEG	NEG	NEG
Perth_05 NH 56	POS	POS	POS	POS	NEG	POS	POS	POS	AMB	POS	POS	POS	NEG	NEG	NEG
**>CC5-MRSA-III LA MRSA**															
Delbrück 38 isolate from domestic turkey	POS	POS	POS	POS	NEG	NEG	POS	POS	NEG	POS	POS	NEG	NEG	POS	NEG
**> Staph. pseudintermed**															
KM1381	NEG	NEG	NEG	NEG	NEG	NEG	NEG	NEG	NEG	NEG	NEG	NEG	NEG	NEG	NEG
**>"South-East Asian Clad**															
M92 (Canada)	POS	POS	POS	POS	NEG	POS	POS	POS	NEG	POS	POS	POS	NEG	NEG	NEG
CHI59 (China)	POS	POS	POS	POS	NEG	POS	POS	POS	NEG	POS	POS	POS	NEG	NEG	NEG
CHI61 (China)	POS	POS	POS	POS	NEG	POS	POS	POS	NEG	POS	POS	POS	NEG	NEG	NEG
CUHK_HK1997 (China)	POS	POS	POS	POS	NEG	POS	POS	NEG	NEG	POS	POS	POS	NEG	NEG	NEG
M996 (China)	POS	POS	POS	POS	NEG	POS	POS	POS	NEG	POS	POS	POS	NEG	NEG	NEG
H211 (Denmark)	POS	POS	POS	POS	NEG	POS	POS	POS	NEG	POS	POS	POS	NEG	NEG	NEG
H216 (Denmark)	POS	POS	POS	POS	NEG	POS	POS	POS	NEG	POS	POS	POS	NEG	NEG	NEG
D71 (Germany)	POS	POS	POS	POS	NEG	POS	POS	POS	NEG	POS	POS	POS	NEG	NEG	NEG
NMR05 (India)	POS	POS	POS	POS	NEG	POS	POS	POS	NEG	POS	POS	POS	NEG	NEG	NEG
MAL11 (Malaysia)	POS	POS	POS	POS	NEG	POS	POS	POS	NEG	POS	POS	POS	NEG	NEG	NEG
MAL9 (Malaysia)	POS	POS	POS	POS	NEG	POS	POS	POS	NEG	POS	POS	POS	NEG	NEG	NEG
Na21 (Sri Lanka)	POS	POS	POS	POS	NEG	POS	POS	POS	NEG	POS	POS	POS	NEG	NEG	NEG
M592 (Syria)	POS	POS	POS	POS	NEG	POS	POS	POS	NEG	POS	POS	POS	NEG	NEG	NEG
H202 (Thailand)	POS	POS	POS	POS	NEG	POS	POS	POS	NEG	POS	POS	POS	NEG	NEG	NEG
S102 (Thailand)	POS	POS	POS	POS	NEG	POS	POS	POS	NEG	POS	POS	POS	NEG	NEG	NEG
S130 (Thailand)	POS	POS	POS	POS	NEG	POS	POS	POS	NEG	POS	POS	POS	NEG	NEG	NEG
S40 (Thailand)	POS	POS	POS	POS	NEG	POS	POS	POS	NEG	POS	POS	POS	NEG	NEG	NEG
S71 (Thailand)	POS	POS	POS	POS	NEG	POS	POS	POS	NEG	POS	POS	POS	NEG	NEG	NEG
S87 (Thailand)	POS	POS	POS	POS	NEG	POS	POS	POS	NEG	POS	POS	POS	NEG	NEG	NEG
S93 (Thailand)	POS	POS	POS	POS	NEG	POS	POS	POS	NEG	POS	POS	POS	NEG	NEG	NEG
US_002 (Thailand)	POS	POS	POS	POS	NEG	POS	POS	POS	NEG	POS	POS	POS	NEG	NEG	NEG
US_017 (Thailand)	POS	POS	POS	POS	NEG	POS	POS	POS	NEG	POS	POS	POS	NEG	NEG	NEG
WAMC6102 (Thailand)	POS	POS	POS	POS	NEG	POS	POS	POS	NEG	POS	POS	POS	NEG	NEG	NEG
NCTC_13626_TW20 (UK)	POS	POS	POS	POS	NEG	POS	POS	POS	NEG	POS	POS	POS	NEG	NEG	NEG
BSAC3021 (UK)	POS	POS	POS	POS	NEG	POS	POS	POS	NEG	POS	POS	POS	NEG	NEG	NEG
BSAC3046 (UK)	POS	POS	POS	POS	NEG	POS	POS	POS	NEG	POS	POS	POS	NEG	NEG	NEG
1193_SAUR (USA)	POS	POS	POS	POS	NEG	POS	POS	POS	NEG	POS	POS	POS	NEG	NEG	NEG
1194_SAUR (USA)	POS	POS	POS	POS	NEG	POS	POS	POS	NEG	POS	POS	POS	NEG	NEG	NEG

STRAIN / ISOLATE	ADHAESION FACTORS / GENES ENCODING MICROBIAL SURFACE COMPONENTS RECOGNIZING ADHESIVE MATRIX MOLECULES														
	ebh	ebpS					eno	efb		fnbA					
	ebh (cons)	ebpS	ebpS_probe 612	ebpS_probe 614	ebpS (01-1111)	ebpS (COL)	eno	efb / fib	efb / fib (MRSA252)	fnbA	fnbA (cons)	fnbA (COL)	fnbA (MRSA252)	fnbA (Mu50+MW2)	fnbA (RF122)
	Cell wall associated fibronectin-binding protein	cell surface elastin binding protein					enolase	fibrinogen binding protein (19 kDa)		fibronectin-binding protein A					
>"South-East Asian Clade": TW20 (Irish AR44), c															
1195_SAUR (USA)	POS	POS	POS	POS	NEG	POS	POS	POS	NEG	POS	POS	POS	NEG	NEG	NEG
FVRH6002 (USA)	POS	POS	POS	POS	NEG	POS	POS	POS	NEG	POS	POS	POS	NEG	NEG	NEG
KINW6048 (USA)	POS	POS	POS	POS	NEG	POS	POS	POS	NEG	POS	POS	POS	NEG	NEG	NEG
LAMC0011 (USA)	POS	POS	POS	POS	NEG	POS	POS	POS	NEG	POS	POS	POS	NEG	NEG	NEG
SJOS6053 (USA)	POS	POS	POS	POS	NEG	POS	POS	POS	NEG	POS	POS	POS	NEG	NEG	NEG
SJOS6072 (USA)	POS	POS	POS	POS	NEG	POS	POS	POS	NEG	POS	POS	POS	NEG	NEG	NEG
UCIM6042 (USA)	POS	POS	POS	POS	NEG	POS	POS	POS	NEG	POS	POS	POS	NEG	NEG	NEG
Perth_2015-S31902	POS	POS	POS	POS	NEG	POS	POS	NEG	NEG	POS	POS	POS	NEG	NEG	NEG
Perth_2015-S31904	POS	POS	POS	POS	NEG	POS	POS	POS	NEG	POS	POS	POS	NEG	NEG	NEG
Perth_2015-S31913	POS	POS	POS	POS	NEG	POS	POS	POS	NEG	POS	POS	POS	NEG	NEG	NEG
Perth_2015-S31918	POS	POS	POS	POS	NEG	POS	POS	POS	NEG	POS	POS	POS	NEG	NEG	NEG
Dublin-DSH_AR44_E1183	POS	POS	POS	POS	NEG	POS	POS	POS	NEG	POS	POS	POS	NEG	NEG	NEG
Dublin-DSH_Unfamiliar-3_E1520	POS	POS	POS	POS	NEG	POS	POS	POS	NEG	POS	POS	POS	NEG	NEG	NEG
Hong Kong_042	POS	POS	POS	POS	NEG	POS	POS	POS	NEG	POS	POS	POS	NEG	NEG	NEG
Hong Kong_101	POS	POS	POS	POS	NEG	POS	POS	POS	NEG	POS	POS	POS	NEG	NEG	NEG
Hong Kong_111	POS	AMB	POS	POS	NEG	POS	POS	POS	NEG	POS	POS	POS	NEG	NEG	NEG
Hong Kong_113	POS	POS	POS	POS	NEG	POS	POS	POS	NEG	POS	POS	POS	NEG	NEG	NEG
Hong Kong_115	POS	POS	POS	POS	NEG	POS	POS	POS	NEG	POS	POS	POS	NEG	NEG	NEG
Hong Kong_118	POS	POS	POS	POS	NEG	POS	POS	POS	NEG	POS	POS	POS	NEG	NEG	NEG
Hong Kong_125	POS	POS	POS	POS	NEG	POS	POS	POS	NEG	POS	POS	POS	NEG	NEG	NEG
Hong Kong_129	POS	POS	POS	POS	NEG	POS	POS	POS	NEG	POS	POS	POS	NEG	NEG	NEG
Hong Kong_133	POS	POS	POS	POS	NEG	POS	POS	POS	NEG	POS	POS	POS	NEG	NEG	NEG
Kuwait_009	POS	POS	POS	POS	NEG	POS	POS	POS	NEG	POS	POS	POS	NEG	NEG	NEG
Kuwait_103	POS	POS	POS	POS	NEG	POS	POS	POS	NEG	POS	POS	POS	NEG	NEG	NEG
Kuwait_107	POS	POS	POS	POS	NEG	POS	POS	POS	NEG	POS	POS	POS	NEG	NEG	NEG
Perth_03 AH 88	POS	POS	POS	POS	NEG	POS	POS	POS	NEG	POS	POS	POS	NEG	NEG	NEG
Perth_08-16905	POS	POS	POS	POS	NEG	POS	POS	POS	NEG	POS	POS	POS	NEG	NEG	NEG
Riyadh_Alfaisal-10_SICU_13_B5672_1117353	NEG	POS	POS	POS	NEG	POS	POS	POS	NEG	POS	POS	POS	NEG	NEG	NEG
Riyadh_SSTI_17_3508602	POS	POS	POS	POS	NEG	POS	POS	POS	NEG	POS	POS	POS	NEG	NEG	NEG
Riyadh-2822088-R	POS	POS	POS	POS	NEG	POS	POS	POS	NEG	POS	POS	POS	NEG	NEG	NEG
Trinidad&Tobago_2012_195 (619/12)	POS	POS	POS	POS	NEG	POS	POS	POS	AMB	POS	POS	POS	NEG	NEG	NEG
Trinidad&Tobago_2012_370 (1112-52177)	POS	POS	POS	POS	NEG	POS	POS	POS	NEG	POS	POS	POS	NEG	NEG	NEG
Trinidad&Tobago_2012_378 (4139)	POS	POS	POS	POS	NEG	POS	POS	POS	AMB	POS	POS	POS	NEG	NEG	NEG
Trinidad&Tobago_2013_PA21	POS	POS	POS	POS	NEG	POS	POS	POS	NEG	POS	POS	POS	NEG	NEG	NEG
Trinidad&Tobago_2013_PA22	POS	POS	POS	POS	NEG	POS	POS	POS	NEG	POS	POS	POS	NEG	NEG	NEG
Trinidad&Tobago_2912_340 (8523/12)	POS	POS	POS	POS	NEG	POS	POS	POS	NEG	POS	POS	POS	NEG	NEG	NEG
Trinidad&Tobago_MRSA2010_105	POS	POS	POS	POS	NEG	POS	POS	POS	NEG	POS	POS	POS	NEG	NEG	NEG
Trinidad&Tobago_MRSA2010_108	POS	POS	POS	POS	NEG	POS	POS	POS	NEG	POS	POS	POS	NEG	NEG	NEG
Trinidad&Tobago_MRSA2010_33	POS	AMB	POS	POS	NEG	POS	POS	POS	NEG	POS	POS	POS	NEG	NEG	NEG
Trinidad&Tobago_MRSA2010_79	POS	POS	POS	POS	NEG	POS	POS	POS	NEG	POS	POS	POS	NEG	NEG	NEG
Trinidad&Tobago_MRSA2010_88	POS	POS	POS	POS	NEG	POS	POS	POS	NEG	POS	POS	POS	NEG	NEG	NEG
Trinidad&Tobago_MRSA2010_97	POS	POS	POS	POS	NEG	POS	POS	POS	NEG	POS	POS	POS	NEG	NEG	NEG
Trinidad&Tobago_SSTI 2012_031	POS	POS	POS	POS	NEG	POS	POS	POS	NEG	POS	POS	POS	NEG	NEG	NEG
Uganda-29_17148_250811	POS	POS	POS	POS	NEG	POS	POS	POS	NEG	POS	POS	POS	NEG	NEG	NEG
>"South-East Asian Clad															
UCIM6015	POS	POS	POS	POS	NEG	POS	POS	POS	NEG	POS	POS	POS	NEG	NEG	NEG
Hong Kong_121	POS	POS	POS	POS	NEG	POS	POS	POS	NEG	POS	POS	POS	NEG	NEG	NEG
>"South-East Asian Clad															
Z172 (Taiwan)	POS	POS	POS	POS	NEG	POS	POS	POS	NEG	POS	POS	POS	NEG	NEG	NEG
Russia-19_0342_Kurgan	POS	POS	POS	POS	NEG	POS	POS	POS	NEG	POS	POS	POS	NEG	NEG	NEG
>"South-East Asian Clad															
Perth_01-15392	POS	POS	POS	POS	NEG	POS	POS	POS	NEG	POS	POS	POS	NEG	NEG	NEG
>"South-East Asian Clad															
XN108 (China)	POS	POS	POS	POS	NEG	POS	POS	POS	NEG	POS	POS	POS	NEG	NEG	NEG
NMR09 (India)	POS	POS	POS	POS	NEG	POS	POS	POS	NEG	POS	POS	POS	NEG	NEG	NEG
346 (Malaysia)	POS	POS	POS	POS	NEG	POS	POS	POS	NEG	POS	POS	POS	NEG	NEG	NEG
DS_009 (Thailand)	POS	POS	POS	POS	NEG	POS	POS	POS	NEG	POS	POS	POS	NEG	NEG	NEG
18ANR581005 (Ind)	POS	POS	POS	POS	NEG	POS	POS	POS	NEG	POS	POS	POS	NEG	NEG	NEG
Bengalaru_ST20141405	POS	POS	POS	POS	NEG	POS	POS	POS	NEG	POS	POS	POS	NEG	NEG	NEG
Bengalaru_ST20141406	POS	POS	POS	POS	NEG	POS	POS	POS	NEG	POS	POS	POS	NEG	NEG	NEG
Bengalaru_ST20141417	POS	POS	POS	POS	NEG	POS	POS	POS	NEG	POS	POS	POS	NEG	NEG	NEG
Hong Kong_107	POS	AMB	POS	POS	NEG	POS	POS	POS	NEG	POS	POS	POS	NEG	NEG	NEG
Hong Kong_12	POS	POS	POS	POS	NEG	POS	POS	POS	NEG	POS	POS	POS	NEG	NEG	NEG
Hong Kong_132	POS	AMB	POS	POS	NEG	POS	POS	POS	NEG	POS	POS	POS	NEG	NEG	NEG
Riyadh-3028763-R	POS	POS	POS	POS	NEG	POS	POS	POS	NEG	POS	POS	POS	NEG	NEG	NEG
Trinidad&Tobago_MRSA2010_94	POS	POS	POS	POS	NEG	POS	POS	POS	NEG	POS	POS	POS	NEG	NEG	NEG
>"South-East Asian Clad															
M705 (Thailand)	POS	POS	POS	POS	NEG	POS	POS	POS	NEG	POS	POS	POS	NEG	NEG	NEG
S85 (Thailand)	POS	POS	POS	POS	NEG	POS	POS	POS	NEG	POS	POS	POS	NEG	NEG	NEG
Riyadh_Alfaisal-04_281014_S5076	POS	POS	POS	POS	NEG	POS	POS	POS	NEG	POS	POS	POS	NEG	NEG	NEG
Riyadh_Alfaisal-26_S15145_1104163	POS	POS	POS	POS	NEG	POS	POS	POS	NEG	POS	POS	POS	NEG	NEG	NEG
Riyadh-2817437-W	POS	POS	POS	POS	NEG	POS	POS	POS	NEG	POS	POS	POS	NEG	NEG	NEG
>"South-East Asian Clad															
PPUKM-775-2009 (Malaysia)	POS	POS	POS	POS	NEG	POS	POS	POS	NEG	POS	POS	POS	NEG	NEG	NEG
Hong Kong_138	POS	POS	POS	POS	NEG	POS	POS	POS	NEG	POS	POS	POS	NEG	NEG	NEG
>"South-East Asian Clad															
Kuwait_001	POS	POS	POS	POS	NEG	POS	POS	POS	NEG	POS	POS	POS	NEG	NEG	NEG
Kuwait_337	POS	POS	POS	POS	NEG	POS	POS	POS	NEG	POS	POS	POS	NEG	NEG	NEG
>"South-East Asian Clad															
CUHK_HK2007 (China)	POS	POS	POS	POS	NEG	POS	POS	POS	NEG	POS	POS	POS	NEG	NEG	NEG
NMR02 (India)	POS	POS	POS	POS	NEG	POS	POS	POS	NEG	POS	POS	POS	NEG	NEG	NEG
VB1490 (India)	POS	POS	POS	POS	NEG	POS	POS	NEG	NEG	POS	POS	POS	NEG	NEG	NEG
V521 (Korea)	POS	POS	POS	POS	NEG	POS	POS	POS	NEG	POS	POS	POS	NEG	NEG	NEG
HST-077 (Lebanon)	POS	POS	POS	POS	NEG	POS	POS	POS	NEG	POS	POS	POS	NEG	NEG	NEG
MAL1 (Malaysia)	POS	POS	POS	POS	NEG	POS	POS	POS	NEG	POS	POS	POS	NEG	NEG	NEG
MAL3 (Malaysia)	POS	POS	POS	POS	NEG	POS	POS	POS	NEG	POS	POS	POS	NEG	NEG	NEG
PPUKM-261-2009 (Malaysia)	POS	POS	POS	POS	NEG	POS	POS	POS	NEG	POS	POS	POS	NEG	NEG	NEG
PPUKM-332-2009 (Malaysia)	POS	POS	POS	POS	NEG	POS	POS	POS	NEG	POS	POS	POS	NEG	NEG	NEG
S38 (Thailand)	POS	POS	POS	POS	NEG	POS	POS	POS	NEG	POS	POS	POS	NEG	NEG	NEG
S7 (Thailand)	POS	POS	POS	POS	NEG	POS	POS	POS	NEG	POS	POS	POS	NEG	NEG	NEG
US_008 (Thailand)	POS	POS	POS	POS	NEG	POS	POS	POS	NEG	POS	POS	POS	NEG	NEG	NEG
US_030 (Thailand)	POS	POS	POS	POS	NEG	POS	POS	POS	NEG	POS	POS	POS	NEG	NEG	NEG
M170 (Tsunami-related)	POS	POS	POS	POS	NEG	POS	POS	POS	NEG	POS	POS	POS	NEG	NEG	NEG
BSAC697 (UK)	POS	POS	POS	POS	NEG	POS	POS	POS	NEG	POS	POS	POS	NEG	NEG	NEG
IS-189	POS	POS	POS	POS	NEG	POS	POS	POS	NEG	POS	POS	POS	NEG	NEG	NEG
MRGR3	POS	POS	POS	POS	NEG	POS	POS	POS	NEG	POS	POS	POS	NEG	NEG	NEG
smg07	POS	POS	POS	POS	NEG	POS	POS	POS	NEG	POS	POS	POS	NEG	NEG	NEG
AH1-AUS-EMRSA-3	POS	AMB	POS	POS	NEG	POS	POS	POS	NEG	POS	POS	POS	NEG	NEG	NEG
Bengalaru_ST20141403	POS	POS	POS	POS	NEG	POS	POS	POS	NEG	POS	POS	POS	NEG	NEG	NEG
Finland E24_98541_Harmony	POS	POS	POS	POS	NEG	POS	POS	POS	NEG	POS	POS	POS	NEG	NEG	NEG
Hong Kong_10	POS	POS	POS	POS	NEG	POS	POS	POS	AMB	POS	POS	POS	NEG	NEG	NEG
Hong Kong_100	POS	POS	POS	POS	NEG	POS	POS	POS	NEG	POS	POS	POS	NEG	NEG	NEG
Hong Kong_199	POS	POS	POS	POS	NEG	POS	POS	POS	NEG	POS	POS	POS	NEG	NEG	NEG
Hong Kong_34	POS	AMB	POS	POS	NEG	POS	POS	POS	NEG	POS	POS	POS	NEG	NEG	NEG
Hong Kong_88	POS	POS	POS	POS	NEG	POS	POS	POS	AMB	POS	POS	POS	NEG	NEG	NEG
Hong Kong_92	POS	POS	POS	POS	NEG	POS	POS	POS	NEG	POS	POS	POS	NEG	NEG	NEG
Hong Kong_94	POS	POS	POS	POS	NEG	POS	POS	POS	NEG	POS	POS	POS	NEG	NEG	NEG
Kuwait_257	POS	POS	POS	POS	NEG	POS	POS	POS	NEG	POS	POS	POS	NEG	NEG	NEG
Perth_01-15330	POS	POS	POS	POS	NEG	POS	POS	POS	NEG	POS	POS	POS	NEG	NEG	NEG
Perth_01-15349	POS	POS	POS	POS	NEG	POS	POS	POS	NEG	POS	POS	POS	NEG	NEG	NEG
Perth_01-16176	POS	POS	POS	POS	NEG	POS	POS	POS	NEG	POS	POS	POS	NEG	NEG	NEG
Perth_WBG8379	POS	POS	POS	POS	NEG	POS	POS	POS	NEG	POS	POS	POS	NEG	NEG	NEG
Rawalpindi_Kidney Center_56	POS	POS	POS	POS	NEG	POS	POS	POS	NEG	POS	POS	POS	NEG	NEG	NEG
Riyadh_SSTI_05_3464614	POS	POS	POS	POS	NEG	POS	POS	POS	NEG	POS	POS	POS	NEG	NEG	NEG
Trinidad&Tobago_2012_157	POS	POS	POS	POS	NEG	POS	POS	POS	NEG	POS	POS	POS	NEG	NEG	NEG

STRAIN / ISOLATE	ADHESION FACTORS / GENES ENCODING MICROBIAL SURFACE COMPONENTS RECOGNIZING ADHESIVE MATRIX MOLECULES														
	ebh	ebpS					eno	efb		fnbA					
	ebh (cons)	ebpS	ebpS_probe 612	ebpS_probe 614	ebpS (01-1111)	ebpS (COL)	eno	efb / fib	efb / fib (MRSA252)	fnbA	fnbA (cons)	fnbA (COL)	fnbA (MRSA252)	fnbA (Mu50+MW2)	fnbA (RF122)
	Cell wall associated fibronectin-binding protein	cell surface elastin binding protein					enolase	fibrinogen binding protein (19 kDa)		fibronectin-binding protein A					
>"South-East Asian Clad															
M418	POS	POS	POS	POS	NEG	POS	POS	POS	NEG	NEG	NEG	NEG	NEG	NEG	NEG
>"South-East Asian Clad															
NMR07 (India)	POS	POS	POS	POS	NEG	POS	POS	POS	NEG	POS	POS	POS	NEG	NEG	NEG
NMR08 (India)	POS	POS	POS	POS	NEG	POS	POS	POS	NEG	POS	POS	POS	NEG	NEG	NEG
Bengalaru_ST20121978	POS	POS	POS	POS	NEG	POS	POS	POS	NEG	POS	POS	POS	NEG	NEG	NEG
Hong Kong_89	POS	POS	POS	POS	NEG	POS	POS	POS	AMB	POS	POS	POS	NEG	NEG	NEG
>"South-East Asian Clad															
Perth_2005_AH_5	POS	AMB	AMB	POS	NEG	POS	POS	POS	NEG	POS	POS	POS	NEG	NEG	NEG
>"South-East Asian Clad															
Trinidad&Tobago_MRSA2010_103	POS	POS	POS	POS	NEG	POS	POS	POS	NEG	POS	POS	POS	NEG	NEG	NEG
Trinidad&Tobago_2013_PA25	POS	POS	POS	POS	NEG	POS	POS	POS	NEG	POS	POS	POS	NEG	NEG	NEG
Trinidad&Tobago_MRSA2010_110	POS	AMB	POS	POS	NEG	POS	POS	POS	NEG	POS	POS	POS	NEG	NEG	NEG
Trinidad&Tobago_SSTI 2012_111	POS	POS	POS	POS	NEG	POS	POS	POS	NEG	POS	POS	POS	NEG	NEG	NEG
Trinidad&Tobago_MRSA2010_158	POS	POS	POS	POS	NEG	POS	POS	POS	NEG	POS	POS	POS	NEG	NEG	NEG
>"South-East Asian Clad															
Kuwait_122	POS	POS	POS	POS	NEG	POS	POS	POS	NEG	POS	POS	POS	NEG	NEG	NEG
Kuwait_002	POS	POS	POS	POS	NEG	POS	POS	POS	NEG	POS	POS	POS	NEG	NEG	NEG
Kuwait_196	POS	POS	POS	POS	NEG	POS	POS	POS	NEG	POS	POS	POS	NEG	NEG	NEG
Kuwait_199	POS	POS	POS	POS	NEG	POS	POS	POS	NEG	POS	POS	POS	NEG	NEG	NEG
Hong Kong_136	POS	POS	POS	POS	NEG	POS	POS	POS	NEG	POS	POS	POS	NEG	NEG	NEG
>"South-East Asian Clad															
P32 (Poland)	POS	POS	POS	POS	NEG	POS	POS	POS	NEG	POS	POS	POS	NEG	NEG	NEG
>"South-East Asian Clad															
Perth_2015-S32313	POS	POS	POS	POS	NEG	POS	POS	POS	NEG	POS	POS	POS	NEG	NEG	NEG
>"hla-negative South-Ea															
DEN907 (Denmark)	POS	POS	POS	POS	NEG	POS	POS	POS	NEG	POS	POS	POS	NEG	NEG	NEG
>"hla-negative South-Ea															
S26 (Thailand)	POS	POS	POS	POS	NEG	POS	POS	POS	NEG	POS	POS	POS	NEG	NEG	NEG
S42 (Thailand)	POS	POS	POS	POS	NEG	POS	POS	POS	NEG	POS	POS	POS	NEG	NEG	NEG
S97 (Thailand)	POS	POS	POS	POS	NEG	POS	POS	POS	NEG	POS	POS	POS	NEG	NEG	NEG
S78 (Thailand)	POS	POS	POS	POS	NEG	POS	POS	POS	NEG	POS	POS	POS	NEG	NEG	NEG
S25 (Thailand)	POS	POS	POS	POS	NEG	POS	POS	POS	NEG	POS	POS	POS	NEG	NEG	NEG
S21 (Thailand)	POS	POS	POS	POS	NEG	POS	POS	POS	NEG	POS	POS	POS	NEG	NEG	NEG
S106 (Thailand)	POS	POS	POS	POS	NEG	POS	POS	POS	NEG	POS	POS	POS	NEG	NEG	NEG
S24 (Thailand)	POS	POS	POS	POS	NEG	POS	POS	POS	NEG	POS	POS	POS	NEG	NEG	NEG
S39 (Thailand)	POS	POS	POS	POS	NEG	POS	POS	POS	NEG	POS	POS	POS	NEG	NEG	NEG
S2 (Thailand)	POS	POS	POS	POS	NEG	POS	POS	POS	NEG	POS	POS	POS	NEG	NEG	NEG
S81 (Thailand)	POS	POS	POS	POS	NEG	POS	POS	POS	NEG	POS	POS	POS	NEG	NEG	NEG
M116 (Vietnam)	POS	POS	POS	POS	NEG	POS	POS	POS	NEG	POS	POS	POS	NEG	NEG	NEG
>"Portuguese Clade": Pc															
FFP103 (Portugal)	POS	POS	POS	POS	NEG	POS	POS	POS	NEG	POS	POS	POS	NEG	NEG	NEG
HDG2 (Portugal)	POS	POS	POS	POS	NEG	POS	POS	POS	NEG	POS	POS	POS	NEG	NEG	NEG
HSA10 (Portugal)	POS	POS	POS	POS	NEG	POS	POS	POS	NEG	POS	POS	POS	NEG	NEG	NEG
HSA11 (Portugal)	POS	POS	POS	POS	NEG	POS	POS	POS	NEG	POS	POS	POS	NEG	NEG	NEG
ICP5011 (Portugal)	POS	POS	POS	POS	NEG	POS	POS	POS	NEG	POS	POS	POS	NEG	NEG	NEG
ICP5014 (Portugal)	POS	POS	POS	POS	NEG	POS	POS	POS	NEG	POS	POS	POS	NEG	NEG	NEG
ICP5062 (Portugal)	POS	POS	POS	POS	NEG	POS	POS	POS	NEG	POS	POS	POS	NEG	NEG	NEG
Strain 1063_ATCC 33592 FDAARGOS_3 (USA)	POS	POS	POS	POS	NEG	POS	POS	POS	NEG	POS	POS	POS	NEG	NEG	NEG
	POS	AMB	POS	POS	NEG	POS	POS	POS	NEG	POS	POS	POS	NEG	NEG	NEG
Russia-07_0110_Kurgan	POS	POS	POS	POS	NEG	POS	POS	POS	NEG	POS	POS	POS	NEG	NEG	NEG
Russia-11_0164_Kurgan	POS	POS	POS	POS	NEG	POS	POS	POS	NEG	POS	POS	POS	NEG	NEG	NEG
Russia-20_0367_Kurgan	POS	POS	POS	POS	NEG	POS	POS	POS	NEG	POS	POS	POS	NEG	NEG	NEG
Russia-21_0390_Chelyabinsk	POS	POS	POS	POS	NEG	POS	POS	POS	NEG	POS	POS	POS	NEG	NEG	NEG
>"South American/ Mid															
AGT1 (Argentina)	POS	POS	POS	POS	NEG	POS	POS	POS	NEG	POS	POS	POS	NEG	NEG	NEG
AGT120 (Argentina)	POS	POS	POS	POS	NEG	POS	POS	POS	NEG	POS	POS	POS	NEG	NEG	NEG
AGT67 (Argentina)	POS	POS	POS	POS	NEG	POS	POS	POS	NEG	POS	POS	POS	NEG	NEG	NEG
AGT9 (Argentina)	POS	POS	POS	POS	NEG	POS	POS	POS	NEG	POS	POS	POS	NEG	NEG	NEG
RA3 (Argentina)	POS	POS	POS	POS	NEG	POS	POS	POS	NEG	POS	POS	POS	NEG	NEG	NEG
RA6 (Argentina)	POS	POS	POS	POS	NEG	POS	POS	POS	NEG	POS	POS	POS	NEG	NEG	NEG
RA7 (Argentina)	POS	POS	POS	POS	NEG	POS	POS	POS	NEG	POS	POS	POS	NEG	NEG	NEG
Be62 (Brazil)	POS	POS	POS	POS	NEG	POS	POS	POS	NEG	POS	POS	POS	NEG	NEG	NEG
Bmb9393 (Brazil)	POS	POS	POS	POS	NEG	POS	POS	POS	NEG	POS	POS	POS	NEG	NEG	NEG
BRA36 (Brazil)	POS	POS	POS	POS	NEG	POS	POS	POS	NEG	POS	POS	POS	NEG	NEG	NEG
BZ48 (Brazil)	POS	POS	POS	POS	NEG	POS	POS	POS	NEG	POS	POS	POS	NEG	NEG	NEG
Gv51 (Brazil)	POS	POS	POS	POS	NEG	POS	POS	POS	NEG	POS	POS	POS	NEG	NEG	NEG
Gv69 (Brazil)	POS	POS	POS	POS	NEG	POS	POS	POS	NEG	POS	POS	POS	NEG	NEG	NEG
Gv88 (Brazil)	POS	POS	POS	POS	NEG	POS	POS	POS	NEG	POS	POS	POS	NEG	NEG	NEG
HC1335 (Brazil)	POS	POS	POS	POS	NEG	POS	POS	POS	NEG	POS	POS	POS	NEG	NEG	NEG
HC1340 (Brazil)	POS	POS	POS	POS	NEG	POS	POS	POS	NEG	POS	POS	POS	NEG	NEG	NEG
HC556 (Brazil)	POS	POS	POS	POS	NEG	POS	POS	POS	NEG	POS	POS	POS	NEG	NEG	NEG
HU25 (Brazil)	POS	POS	POS	POS	NEG	POS	POS	POS	NEG	POS	POS	POS	NEG	NEG	NEG
UB563 (Brazil)	POS	POS	POS	POS	NEG	POS	POS	POS	NEG	POS	POS	POS	NEG	NEG	NEG
2A8 (Czech Republic)	POS	POS	POS	POS	NEG	POS	POS	POS	NEG	POS	POS	POS	NEG	NEG	NEG
M1229 (Denmark)	POS	POS	POS	POS	NEG	POS	POS	POS	NEG	POS	POS	POS	NEG	NEG	NEG
FRICAR (France)	POS	POS	POS	POS	NEG	POS	POS	POS	NEG	POS	POS	POS	NEG	NEG	NEG
D90 (Germany)	POS	POS	POS	POS	NEG	POS	POS	POS	NEG	POS	POS	POS	NEG	NEG	NEG
LIT68 (Lithuania)	POS	POS	POS	POS	NEG	POS	POS	POS	NEG	POS	POS	POS	NEG	NEG	NEG
LIT76 (Lithuania)	POS	POS	POS	POS	NEG	POS	POS	POS	NEG	POS	POS	POS	NEG	NEG	NEG
HGSA142 (Portugal)	POS	POS	POS	POS	NEG	POS	POS	POS	NEG	POS	POS	POS	NEG	NEG	NEG
HGSA9 (Portugal)	POS	POS	POS	POS	NEG	POS	POS	POS	NEG	POS	POS	POS	NEG	NEG	NEG
HSJ216 (Portugal)	POS	POS	POS	POS	NEG	POS	POS	POS	NEG	POS	POS	POS	NEG	NEG	NEG
M278 (Portugal)	POS	POS	POS	POS	NEG	POS	POS	POS	NEG	POS	POS	POS	NEG	NEG	NEG
ES26 (Spain)	POS	POS	POS	POS	NEG	POS	POS	POS	NEG	POS	POS	POS	NEG	NEG	NEG
UK102 (UK)	POS	POS	POS	POS	NEG	POS	POS	POS	NEG	POS	POS	POS	NEG	NEG	NEG
URU34 (Uruguay)	POS	POS	POS	POS	NEG	POS	POS	POS	NEG	POS	POS	POS	NEG	NEG	NEG
IS-125	POS	POS	POS	POS	NEG	POS	POS	POS	NEG	POS	POS	POS	NEG	NEG	NEG
IS-157	POS	POS	POS	POS	NEG	POS	POS	POS	NEG	POS	POS	POS	NEG	NEG	NEG
Ecuador_1	POS	POS	POS	POS	NEG	POS	POS	POS	NEG	POS	POS	POS	NEG	NEG	NEG
Ecuador_2	POS	POS	POS	POS	NEG	POS	POS	POS	NEG	POS	POS	POS	NEG	NEG	NEG
Dresden_15ANR574263	POS	POS	POS	POS	NEG	POS	POS	POS	NEG	POS	POS	POS	NEG	NEG	NEG
UK-EMRSA-11	POS	POS	POS	POS	NEG	POS	POS	POS	NEG	POS	POS	POS	NEG	NEG	NEG
>"South American/ Mid															
BRA2 (Brazil)	POS	POS	POS	POS	NEG	POS	POS	POS	NEG	POS	POS	POS	NEG	NEG	NEG
CHL1 (Chile)	POS	POS	POS	POS	NEG	POS	POS	POS	NEG	POS	POS	POS	NEG	NEG	NEG
CHL151 (Chile)	POS	POS	POS	POS	NEG	POS	POS	POS	NEG	POS	POS	POS	NEG	NEG	NEG
UCL378 (Chile)	POS	POS	POS	POS	NEG	POS	POS	POS	NEG	POS	POS	POS	NEG	NEG	NEG
UP72 (Peru)	POS	POS	POS	POS	NEG	POS	POS	POS	NEG	POS	POS	POS	NEG	NEG	NEG
UP81 (Peru)	POS	POS	POS	POS	NEG	POS	POS	POS	NEG	POS	POS	POS	NEG	NEG	NEG
>"South American/ Mid															
UP1073 (Peru)	POS	POS	POS	POS	NEG	POS	POS	POS	NEG	POS	POS	POS	NEG	NEG	NEG

STRAIN / ISOLATE	ADHAESION FACTORS / GENES ENCODING MICROBIAL SURFACE COMPONENTS RECOGNIZING ADHESIVE MATRIX MOLECULES														
	ebh	ebpS					eno	efb		fnbA					
	ebh (cons)	ebpS	ebpS_probe 612	ebpS_probe 614	ebpS (01-1111)	ebpS (COL)	eno	efb / fib	efb / fib (MRSA252)	fnbA	fnbA (cons)	fnbA (COL)	fnbA (MRSA252)	fnbA (Mu50+MW2)	fnbA (RF122)
	Cell wall associated fibronectin-binding protein	cell surface elastin binding protein					enolase	fibrinogen binding protein (19 kDa)		fibronectin-binding protein A					
>"South American/ Mid															
LIT89 (Lithuania)	POS	POS	POS	POS	NEG	POS	POS	POS	NEG	POS	POS	POS	NEG	NEG	NEG
MRSA_PR1 (Malaysia)	POS	POS	POS	POS	NEG	POS	POS	POS	NEG	POS	POS	POS	NEG	NEG	NEG
Riyadh-2888905-R	POS	POS	POS	POS	NEG	POS	POS	POS	NEG	POS	POS	POS	NEG	NEG	NEG
Riyadh-2888915-BC	POS	POS	POS	POS	NEG	POS	POS	POS	NEG	POS	POS	POS	NEG	NEG	NEG
Riyadh-2793706-R	POS	POS	POS	POS	NEG	POS	POS	POS	NEG	POS	POS	POS	NEG	NEG	NEG
>"South American/ Mid															
Riyadh_SSTI_18_3502925	POS	POS	POS	POS	NEG	POS	POS	POS	AMB	POS	POS	POS	NEG	NEG	NEG
Riyadh-2822825-W	POS	POS	POS	POS	NEG	POS	POS	POS	AMB	POS	POS	POS	AMB	NEG	NEG
>"South American/ Mid															
UK-EMRSA-9	POS	AMB	POS	POS	NEG	POS	POS	POS	NEG	POS	POS	POS	NEG	NEG	NEG
>"South American/ Mid															
Lome_HT20020815	POS	POS	POS	POS	NEG	POS	POS	POS	NEG	POS	POS	POS	NEG	NEG	NEG
>"South American/ Mid															
NA32 (Denmark)	POS	POS	POS	POS	NEG	POS	POS	POS	NEG	POS	POS	POS	NEG	NEG	NEG
H24 (Egypt)	POS	POS	POS	POS	NEG	POS	POS	POS	NEG	POS	POS	POS	NEG	NEG	NEG
ATCC BAA-39 (=HUSA304) (Hungary)	POS	POS	POS	POS	NEG	POS	POS	POS	NEG	POS	POS	POS	NEG	NEG	NEG
HUSA304 (Hungary)	POS	POS	POS	POS	NEG	POS	POS	POS	NEG	POS	POS	POS	NEG	NEG	NEG
HU106 (Hungary)	POS	POS	POS	POS	NEG	POS	POS	POS	NEG	POS	POS	POS	NEG	NEG	NEG
BSAC27 (UK)	POS	POS	POS	POS	NEG	POS	POS	POS	NEG	POS	POS	POS	NEG	NEG	NEG
NCTC13131, UK-EMRSA-4 (UK)	POS	POS	POS	POS	NEG	POS	POS	POS	NEG	POS	POS	POS	NEG	NEG	NEG
	POS	POS	POS	POS	NEG	POS	POS	POS	NEG	POS	POS	POS	NEG	NEG	NEG
Algiers_HT20040080	POS	POS	POS	POS	NEG	POS	POS	POS	NEG	POS	POS	POS	NEG	NEG	NEG
Dublin-DSH_AR09_0.0066	POS	AMB	POS	POS	NEG	POS	POS	POS	NEG	POS	POS	POS	NEG	NEG	NEG
Dublin-DSH_AR09_0-0065	POS	POS	NEG	POS	NEG	POS	POS	POS	NEG	POS	POS	POS	NEG	NEG	NEG
Dublin-DSH_Phenotype-III_84	POS	POS	POS	POS	NEG	POS	POS	POS	NEG	POS	POS	POS	NEG	NEG	NEG
Hong Kong_130	POS	POS	POS	POS	NEG	POS	POS	POS	NEG	POS	POS	POS	NEG	NEG	NEG
Kuwait_018	POS	POS	POS	POS	NEG	POS	POS	POS	NEG	POS	POS	POS	NEG	NEG	NEG
Perth_08-17726	POS	POS	POS	POS	NEG	POS	POS	POS	NEG	POS	POS	POS	NEG	NEG	NEG
Riyadh_Alfaisal/KKKSUH_86_MRS-14-279	POS	POS	POS	POS	NEG	POS	POS	POS	NEG	POS	POS	POS	NEG	NEG	NEG
Riyadh_Alfaisal-04_Z3861_831588	POS	POS	POS	POS	NEG	POS	POS	POS	NEG	POS	POS	POS	NEG	NEG	NEG
Riyadh_Alfaisal-30_S15724_1108013	POS	POS	POS	POS	NEG	POS	POS	POS	NEG	POS	POS	POS	NEG	NEG	NEG
Riyadh_Alfaisal-6_22A_13_B3992_397721	POS	POS	POS	POS	NEG	POS	POS	POS	NEG	POS	POS	POS	NEG	NEG	NEG
Riyadh_SSTI_52_3615482	POS	POS	POS	POS	NEG	POS	POS	POS	NEG	POS	POS	POS	NEG	NEG	NEG
Riyadh-2817276-2	POS	POS	POS	POS	NEG	POS	POS	POS	NEG	POS	POS	POS	AMB	NEG	NEG
Riyadh-2891670-W	POS	POS	POS	POS	NEG	POS	POS	POS	NEG	POS	POS	POS	AMB	NEG	NEG
Riyadh-3006920-W	POS	POS	POS	POS	NEG	POS	POS	POS	NEG	POS	POS	POS	NEG	NEG	NEG
Riyadh-R2567782	NEG	POS	POS	POS	NEG	POS	POS	POS	NEG	POS	POS	POS	NEG	NEG	NEG
Russia-18_0252_Krasnoyarsk_SK2	POS	POS	POS	POS	NEG	POS	POS	POS	NEG	POS	POS	POS	NEG	NEG	NEG
UK-EMRSA-7	POS	POS	POS	POS	NEG	POS	POS	POS	NEG	POS	POS	POS	NEG	NEG	NEG
>"South American/ Mid															
Dublin-DSH_AR23_0073	POS	POS	AMB	POS	NEG	POS	POS	POS	NEG	POS	POS	POS	NEG	NEG	NEG
>Related to "South Ame															
MRSA-OC3 (Russia)	POS	POS	POS	POS	NEG	POS	POS	POS	NEG	POS	POS	POS	NEG	NEG	NEG
Russia-12_0176_Krasnoyarsk	POS	POS	POS	POS	NEG	POS	POS	POS	NEG	POS	POS	POS	NEG	NEG	NEG
Russia-13_0180_Krasnoyarsk	POS	POS	POS	POS	NEG	POS	POS	POS	NEG	POS	POS	POS	NEG	NEG	NEG
Russia-16_0249_Krasnoyarsk	POS	POS	POS	POS	NEG	POS	POS	POS	NEG	POS	POS	POS	NEG	NEG	NEG
Russia-17_0250_Krasnoyarsk	POS	POS	POS	POS	NEG	POS	POS	POS	NEG	POS	POS	POS	NEG	NEG	NEG
>Related to "South Ame															
URU110 (Uruguay)	POS	POS	POS	POS	NEG	POS	POS	POS	NEG	POS	POS	POS	NEG	NEG	NEG
>Related to "South Ame															
DS_014 (Thailand)	POS	POS	POS	POS	NEG	POS	POS	POS	NEG	POS	POS	POS	NEG	NEG	NEG
>Unassigned Middle Eas															
Frankfurt_Oder_0490031797 (Libya)	POS	POS	POS	POS	NEG	POS	POS	POS	NEG	POS	POS	POS	NEG	NEG	NEG
Kuwait_192	POS	POS	POS	POS	NEG	POS	POS	POS	NEG	POS	POS	POS	NEG	NEG	NEG
Riyadh-9	POS	POS	POS	POS	NEG	POS	POS	POS	NEG	POS	POS	POS	NEG	NEG	NEG
Russia-24_0407_Moscow	POS	POS	POS	POS	NEG	POS	POS	POS	NEG	POS	POS	POS	NEG	NEG	NEG

STRAIN / ISOLATE	ADHESION FACTORS / GENES ENCODING MICROBIAL SURFACE COMPONENTS RECOGNIZING ADHESIVE MATRIX MOLECULES															
	fnbB							map				sasG				sasX / sesI
	fnbB	fnbB (COL)	fnbB (COL+Mu50+MW2)	fnbB (Mu50)	fnbB (MW2)	fnbB (ST15)	fnbB (ST45-2)	map	map (COL)	map (MRSA252)	map (Mu50+MW2)	sasG	sasG (COL+Mu50)	sasG (MW2)	sasG (OtherThan252+122)	
	Fibronectin-binding protein B							Major histocompatibility complex class II analog protein (=Extracellular adherence protein, eap)				Staphylococcus aureus surface protein G				
**>"Eurasian Clade": TUR1**																**NEG**
TUR1 (Turkey)	NEG	NEG	NEG	NEG	NEG	NEG	NEG	POS	POS	NEG	NEG	POS	POS	NEG	POS	NEG
TUR9 (Turkey)	NEG	NEG	NEG	NEG	NEG	NEG	NEG	POS	POS	NEG	NEG	POS	POS	NEG	POS	NEG
**>"Eurasian Clade" : T013**																**NEG**
T0131 (China)	NEG	NEG	NEG	NEG	NEG	NEG	NEG	POS	POS	NEG	NEG	POS	POS	NEG	POS	NEG
MU4 (Turkey)	NEG	NEG	NEG	NEG	NEG	NEG	NEG	POS	POS	NEG	NEG	POS	POS	NEG	POS	NEG
DEU11 (Turkey)	NEG	NEG	NEG	NEG	NEG	NEG	NEG	POS	POS	NEG	NEG	POS	POS	NEG	POS	NEG
DEU16 (Turkey)	NEG	NEG	NEG	NEG	NEG	NEG	NEG	POS	POS	NEG	NEG	POS	POS	NEG	POS	NEG
Romania_Iasi_MRSA-07_54	NEG	NEG	NEG	NEG	NEG	NEG	NEG	POS	POS	NEG	AMB	POS	POS	NEG	POS	NEG
**>"Eurasian Clade": arsC-**																**NEG**
Romania_Iasi_MRSA-05_46	NEG	NEG	NEG	NEG	NEG	NEG	NEG	POS	POS	NEG	AMB	POS	POS	NEG	POS	NEG
Romania_Iasi_MRSA-06_47	NEG	NEG	NEG	NEG	NEG	NEG	NEG	POS	POS	NEG	AMB	POS	POS	NEG	POS	NEG
**>"Eurasian Clade": 16K/**																**NEG**
CN79 (China)	NEG	NEG	NEG	NEG	NEG	NEG	NEG	POS	POS	NEG	NEG	POS	POS	NEG	POS	NEG
CUHK_BJ2002 (China)	NEG	NEG	NEG	NEG	NEG	NEG	NEG	POS	POS	NEG	NEG	POS	POS	NEG	POS	NEG
CUHK_BJ2007 (China)	NEG	NEG	NEG	NEG	NEG	NEG	NEG	POS	POS	NEG	NEG	POS	POS	NEG	POS	NEG
3HK (Czech Republic)	NEG	NEG	NEG	NEG	NEG	NEG	NEG	POS	POS	NEG	NEG	POS	POS	NEG	POS	NEG
HU109 (Hungary)	NEG	NEG	NEG	NEG	NEG	NEG	NEG	POS	POS	NEG	NEG	POS	POS	NEG	POS	NEG
HUR18 (Hungary)	NEG	NEG	NEG	NEG	NEG	NEG	NEG	POS	POS	NEG	NEG	POS	POS	NEG	POS	NEG
H482 (Romania)	NEG	NEG	NEG	NEG	NEG	NEG	NEG	POS	POS	NEG	NEG	POS	POS	NEG	POS	NEG
16K (Russia)	NEG	NEG	NEG	NEG	NEG	NEG	NEG	POS	POS	NEG	NEG	POS	POS	NEG	POS	NEG
DEU10 (Turkey)	NEG	NEG	NEG	NEG	NEG	NEG	NEG	POS	POS	NEG	NEG	POS	POS	NEG	POS	NEG
DEU12 (Turkey)	NEG	NEG	NEG	NEG	NEG	NEG	NEG	POS	POS	NEG	NEG	POS	POS	NEG	POS	NEG
DEU14 (Turkey)	NEG	NEG	NEG	NEG	NEG	NEG	NEG	POS	POS	NEG	NEG	POS	POS	NEG	POS	NEG
DEU15 (Turkey)	NEG	NEG	NEG	NEG	NEG	NEG	NEG	POS	POS	NEG	NEG	POS	POS	NEG	POS	NEG
DEU17 (Turkey)	NEG	NEG	NEG	NEG	NEG	NEG	NEG	POS	POS	NEG	NEG	POS	POS	NEG	POS	NEG
DEU19 (Turkey)	NEG	NEG	NEG	NEG	NEG	NEG	NEG	POS	POS	NEG	NEG	POS	POS	NEG	POS	NEG
DEU2 (Turkey)	NEG	NEG	NEG	NEG	NEG	NEG	NEG	POS	POS	NEG	NEG	POS	POS	NEG	POS	NEG
DEU20 (Turkey)	NEG	NEG	NEG	NEG	NEG	NEG	NEG	POS	POS	NEG	NEG	POS	POS	NEG	POS	NEG
DEU23 (Turkey)	NEG	NEG	NEG	NEG	NEG	NEG	NEG	POS	POS	NEG	NEG	POS	POS	NEG	POS	NEG
DEU3 (Turkey)	NEG	NEG	NEG	NEG	NEG	NEG	NEG	POS	POS	NEG	NEG	POS	POS	NEG	POS	NEG
DEU5 (Turkey)	NEG	NEG	NEG	NEG	NEG	NEG	NEG	POS	POS	NEG	NEG	POS	POS	NEG	POS	NEG
DEU6 (Turkey)	NEG	NEG	NEG	NEG	NEG	NEG	NEG	POS	POS	NEG	NEG	POS	POS	NEG	POS	NEG
DEU8 (Turkey)	NEG	NEG	NEG	NEG	NEG	NEG	NEG	POS	POS	NEG	NEG	POS	POS	NEG	POS	NEG
HU11 (Turkey)	NEG	NEG	NEG	NEG	NEG	NEG	NEG	POS	POS	NEG	NEG	POS	POS	NEG	POS	NEG
HU13 (Turkey)	NEG	NEG	NEG	NEG	NEG	NEG	NEG	POS	POS	NEG	NEG	POS	POS	NEG	POS	NEG
HU14 (Turkey)	NEG	NEG	NEG	NEG	NEG	NEG	NEG	POS	POS	NEG	NEG	POS	POS	NEG	POS	NEG
HU15 (Turkey)	NEG	NEG	NEG	NEG	NEG	NEG	NEG	POS	POS	NEG	NEG	POS	POS	NEG	POS	NEG
HU16 (Turkey)	NEG	NEG	NEG	NEG	NEG	NEG	NEG	POS	POS	NEG	NEG	POS	POS	NEG	POS	NEG
HU17 (Turkey)	NEG	NEG	NEG	NEG	NEG	NEG	NEG	POS	POS	NEG	NEG	POS	POS	NEG	POS	NEG
HU21 (Turkey)	NEG	NEG	NEG	NEG	NEG	NEG	NEG	POS	POS	NEG	NEG	POS	POS	NEG	POS	NEG
HU23 (Turkey)	NEG	NEG	NEG	NEG	NEG	NEG	NEG	POS	POS	NEG	NEG	POS	POS	NEG	POS	NEG
HU26 (Turkey)	NEG	NEG	NEG	NEG	NEG	NEG	NEG	POS	POS	NEG	NEG	POS	POS	NEG	POS	NEG
HU4 (Turkey)	NEG	NEG	NEG	NEG	NEG	NEG	NEG	POS	POS	NEG	NEG	POS	POS	NEG	POS	NEG
HU5 (Turkey)	NEG	NEG	NEG	NEG	NEG	NEG	NEG	POS	POS	NEG	NEG	POS	POS	NEG	POS	NEG
HU6 (Turkey)	NEG	NEG	NEG	NEG	NEG	NEG	NEG	POS	POS	NEG	NEG	POS	POS	NEG	POS	NEG
HU7 (Turkey)	NEG	NEG	NEG	NEG	NEG	NEG	NEG	POS	POS	NEG	NEG	POS	POS	NEG	POS	NEG
HU8 (Turkey)	NEG	NEG	NEG	NEG	NEG	NEG	NEG	POS	POS	NEG	NEG	POS	POS	NEG	POS	NEG
HU9 (Turkey)	NEG	NEG	NEG	NEG	NEG	NEG	NEG	POS	POS	NEG	NEG	POS	POS	NEG	POS	NEG
IU1 (Turkey)	NEG	NEG	NEG	NEG	NEG	NEG	NEG	POS	POS	NEG	NEG	POS	POS	NEG	POS	NEG
IU10 (Turkey)	NEG	NEG	NEG	NEG	NEG	NEG	NEG	POS	POS	NEG	NEG	POS	POS	NEG	POS	NEG
IU11 (Turkey)	NEG	NEG	NEG	NEG	NEG	NEG	NEG	POS	POS	NEG	NEG	POS	POS	NEG	POS	NEG
IU12 (Turkey)	NEG	NEG	NEG	NEG	NEG	NEG	NEG	POS	POS	NEG	NEG	POS	POS	NEG	POS	NEG
IU13 (Turkey)	NEG	NEG	NEG	NEG	NEG	NEG	NEG	POS	POS	NEG	NEG	POS	POS	NEG	POS	NEG
IU15 (Turkey)	NEG	NEG	NEG	NEG	NEG	NEG	NEG	POS	POS	NEG	NEG	POS	POS	NEG	POS	NEG
IU18 (Turkey)	NEG	NEG	NEG	NEG	NEG	NEG	NEG	POS	POS	NEG	NEG	POS	POS	NEG	POS	NEG
IU19 (Turkey)	NEG	NEG	NEG	NEG	NEG	NEG	NEG	POS	POS	NEG	NEG	POS	POS	NEG	POS	NEG
IU2 (Turkey)	NEG	NEG	NEG	NEG	NEG	NEG	NEG	POS	POS	NEG	NEG	POS	POS	NEG	POS	NEG
IU4 (Turkey)	NEG	NEG	NEG	NEG	NEG	NEG	NEG	POS	POS	NEG	NEG	POS	POS	NEG	POS	NEG
IU6 (Turkey)	NEG	NEG	NEG	NEG	NEG	NEG	NEG	POS	POS	NEG	NEG	POS	POS	NEG	POS	NEG
IU7 (Turkey)	NEG	NEG	NEG	NEG	NEG	NEG	NEG	POS	POS	NEG	NEG	POS	POS	NEG	POS	NEG
IU9 (Turkey)	NEG	NEG	NEG	NEG	NEG	NEG	NEG	POS	POS	NEG	NEG	POS	POS	NEG	POS	NEG
MU1 (Turkey)	NEG	NEG	NEG	NEG	NEG	NEG	NEG	POS	POS	NEG	NEG	POS	POS	NEG	POS	NEG
MU10 (Turkey)	NEG	NEG	NEG	NEG	NEG	NEG	NEG	POS	POS	NEG	NEG	POS	POS	NEG	POS	NEG
MU20 (Turkey)	NEG	NEG	NEG	NEG	NEG	NEG	NEG	POS	POS	NEG	NEG	POS	POS	NEG	POS	NEG
Mu3 (Turkey)	NEG	NEG	NEG	NEG	NEG	NEG	NEG	POS	POS	NEG	NEG	POS	POS	NEG	POS	NEG
MU5 (Turkey)	NEG	NEG	NEG	NEG	NEG	NEG	NEG	POS	POS	NEG	NEG	POS	POS	NEG	POS	NEG
MU6 (Turkey)	NEG	NEG	NEG	NEG	NEG	NEG	NEG	POS	POS	NEG	NEG	POS	POS	NEG	POS	NEG
MU7 (Turkey)	NEG	NEG	NEG	NEG	NEG	NEG	NEG	POS	POS	NEG	NEG	POS	POS	NEG	POS	NEG
TUR27 (Turkey)	NEG	NEG	NEG	NEG	NEG	NEG	NEG	POS	POS	NEG	NEG	POS	POS	NEG	POS	NEG
NCTR 32S	NEG	NEG	NEG	NEG	NEG	NEG	NEG	POS	POS	NEG	NEG	POS	POS	NEG	POS	NEG
SA02_A4 = Russia-06_0085_Moscow	NEG	NEG	NEG	NEG	NEG	NEG	NEG	POS	POS	NEG	NEG	POS	POS	NEG	POS	NEG
	NEG	NEG	NEG	NEG	NEG	NEG	NEG	POS	POS	NEG	AMB	POS	POS	NEG	POS	NEG
Dresden_08V35987 (Turkey)	NEG	NEG	NEG	NEG	NEG	NEG	NEG	POS	POS	NEG	NEG	POS	POS	NEG	POS	NEG
Dresden_17ANR577152	NEG	NEG	NEG	NEG	NEG	NEG	NEG	POS	POS	NEG	NEG	POS	POS	NEG	POS	NEG
Dresden_17ANR580374 (Makedonia)	NEG	NEG	NEG	NEG	NEG	NEG	NEG	POS	POS	NEG	AMB	POS	POS	NEG	POS	NEG
Hong Kong_69-II	NEG	NEG	NEG	NEG	NEG	NEG	NEG	AMB	POS	NEG	NEG	AMB	POS	NEG	POS	NEG
Hong Kong_93	NEG	NEG	NEG	NEG	NEG	NEG	NEG	AMB	POS	NEG	NEG	AMB	POS	NEG	POS	NEG
Rawalpindi_Kidney Center_03_SK1	NEG	NEG	NEG	NEG	NEG	NEG	NEG	POS	POS	NEG	AMB	NEG	NEG	NEG	NEG	NEG
Rawalpindi_Kidney Center_08	NEG	NEG	NEG	NEG	NEG	NEG	NEG	POS	POS	NEG	NEG	POS	POS	NEG	POS	NEG
Rawalpindi_Kidney Center_10	NEG	NEG	NEG	NEG	NEG	NEG	NEG	POS	POS	NEG	POS	POS	POS	NEG	POS	NEG
Rawalpindi_Kidney Center_19	NEG	NEG	NEG	NEG	NEG	NEG	NEG	POS	POS	NEG	POS	POS	POS	NEG	POS	NEG
Rawalpindi_Kidney Center_50_SK1	NEG	NEG	NEG	NEG	NEG	NEG	NEG	POS	POS	NEG	NEG	POS	POS	NEG	POS	NEG
Romania_Iasi_BC-49_430	NEG	NEG	NEG	NEG	NEG	NEG	NEG	POS	POS	NEG	AMB	POS	POS	AMB	POS	NEG
Romania_Iasi_MRSA_284	NEG	NEG	NEG	NEG	NEG	NEG	NEG	POS	POS	NEG	AMB	POS	POS	NEG	POS	NEG
Romania_Iasi_MRSA-32_318	NEG	NEG	NEG	NEG	NEG	NEG	NEG	POS	POS	NEG	NEG	POS	POS	NEG	POS	NEG
Romania_Iasi_MRSA-54_430	POS	NEG	NEG	NEG	POS	NEG	NEG	POS	POS	NEG	POS	POS	POS	AMB	POS	NEG
Romania_Iasi_SSTI-01_2	NEG	NEG	NEG	NEG	NEG	NEG	NEG	NEG	NEG	NEG	NEG	POS	POS	NEG	POS	NEG
Romania_Iasi_SSTI-10_101	NEG	NEG	NEG	NEG	NEG	NEG	NEG	POS	POS	NEG	NEG	POS	POS	NEG	POS	NEG
Romania_Iasi_SSTI-11_106	NEG	NEG	NEG	NEG	NEG	NEG	NEG	POS	POS	NEG	NEG	POS	POS	NEG	POS	NEG
Russia-01_0001_SaintPetersburg	NEG	NEG	NEG	NEG	NEG	NEG	NEG	POS	POS	NEG	NEG	POS	POS	NEG	POS	NEG
Russia-02_0004_SaintPetersburg	NEG	NEG	NEG	NEG	NEG	NEG	NEG	POS	POS	NEG	NEG	POS	POS	NEG	POS	NEG
Russia-03_0057_SaintPetersburg	NEG	NEG	NEG	NEG	NEG	NEG	NEG	POS	POS	NEG	NEG	POS	POS	NEG	POS	NEG
Russia-04_0076_Moscow	NEG	NEG	NEG	NEG	NEG	NEG	NEG	POS	POS	NEG	NEG	POS	POS	NEG	POS	NEG
Russia-05_0078_Moscow	NEG	NEG	NEG	NEG	NEG	NEG	NEG	POS	POS	NEG	NEG	POS	POS	NEG	POS	NEG
Russia-08_0135_Moscow	NEG	NEG	NEG	NEG	NEG	NEG	NEG	POS	POS	NEG	NEG	POS	POS	NEG	POS	NEG
Russia-09_0150_Kurgan	NEG	NEG	NEG	NEG	NEG	NEG	NEG	POS	POS	NEG	NEG	POS	POS	NEG	POS	NEG
Russia-10_0162_Kurgan	NEG	NEG	NEG	NEG	NEG	NEG	NEG	POS	POS	NEG	NEG	POS	POS	NEG	POS	NEG
Russia-14_0184_Moscow	NEG	NEG	NEG	NEG	NEG	NEG	NEG	POS	POS	NEG	NEG	POS	POS	NEG	POS	NEG
Russia-15_0232_SaintPetersburg	NEG	NEG	NEG	NEG	NEG	NEG	NEG	POS	POS	NEG	NEG	POS	POS	NEG	POS	NEG
Russia-22_0391_Chelyabinsk	NEG	NEG	NEG	NEG	NEG	NEG	NEG	POS	POS	NEG	NEG	POS	POS	NEG	POS	NEG
Russia-23_0392_Chelyabinsk	NEG	NEG	NEG	NEG	NEG	NEG	NEG	POS	POS	NEG	NEG	POS	POS	NEG	POS	NEG
**>"Eurasian Clade" : IU17**																**NEG**
IU17 (Turkey)	NEG	NEG	NEG	NEG	NEG	NEG	NEG	POS	POS	NEG	NEG	POS	POS	NEG	POS	NEG
IU20 (Turkey)	NEG	NEG	NEG	NEG	NEG	NEG	NEG	POS	POS	NEG	NEG	POS	POS	NEG	POS	NEG

STRAIN / ISOLATE	ADHAESION FACTORS / GENES ENCODING MICROBIAL SURFACE COMPONENTS RECOGNIZING ADHESIVE MATRIX MOLECULES															
	fnbB							map				sasG				sasX / sesI
	fnbB	fnbB (COL)	fnbB (COL+Mu50+MW2)	fnbB (Mu50)	fnbB (MW2)	fnbB (ST15)	fnbB (ST45-2)	map	map (COL)	map (MRSA252)	map (Mu50+MW2)	sasG	sasG (COL+Mu50)	sasG (MW2)	sasG (OtherThan252+122)	
	Fibronectin-binding protein B							Major histocompatibility complex class II analog protein (=Extracellular adherence protein, eap)				Staphylococcus aureus surface protein G				
**>"Eurasian Clade": Spor**																**NEG**
Dresden_16ANR581769	NEG	NEG	NEG	NEG	NEG	NEG	NEG	POS	POS	NEG	NEG	POS	POS	NEG	POS	NEG
Dresden_17ANR578769	NEG	NEG	NEG	NEG	NEG	NEG	NEG	POS	POS	NEG	NEG	POS	POS	NEG	POS	NEG
**>"Eurasian Clade": Kuwa**																**NEG**
Kuwait_306	NEG	NEG	NEG	NEG	NEG	NEG	NEG	POS	POS	NEG	POS	POS	POS	NEG	POS	NEG
Kuwait_301	NEG	NEG	NEG	NEG	NEG	NEG	NEG	POS	POS	NEG	POS	POS	POS	NEG	POS	NEG
**>"European Clade": "Gr**																**NEG**
GRE18 (Greece)	POS	AMB	AMB	NEG	NEG	NEG	NEG	POS	POS	NEG	NEG	POS	POS	NEG	POS	NEG
GRE317 (Greece)	POS	AMB	AMB	NEG	NEG	NEG	NEG	POS	POS	NEG	NEG	POS	POS	NEG	POS	NEG
GRE4 (Greece)	POS	AMB	AMB	NEG	NEG	NEG	NEG	POS	POS	NEG	NEG	POS	POS	NEG	POS	NEG
R35 (USA)	POS	AMB	AMB	NEG	NEG	NEG	NEG	POS	POS	NEG	NEG	POS	POS	NEG	POS	NEG
Casablanca_HT20060548	POS	POS	AMB	AMB	NEG	NEG	NEG	POS	POS	NEG	AMB	POS	POS	NEG	POS	NEG
Casablanca_HT20060550	POS	POS	AMB	AMB	NEG	NEG	NEG	POS	POS	NEG	NEG	POS	POS	NEG	POS	NEG
Dresden_01V34572 (Greece)	POS	POS	POS	NEG	NEG	NEG	NEG	POS	POS	NEG	NEG	POS	POS	NEG	POS	NEG
Dresden_01V36469	POS	POS	POS	NEG	NEG	NEG	NEG	POS	POS	NEG	NEG	POS	POS	NEG	POS	NEG
Dresden_01V39123	POS	POS	AMB	AMB	NEG	NEG	NEG	POS	POS	NEG	AMB	POS	POS	POS	POS	NEG
Dresden_01V39143	POS	POS	AMB	NEG	NEG	NEG	NEG	POS	POS	NEG	NEG	POS	POS	NEG	POS	NEG
Greece 1_3680_Harmony	POS	AMB	POS	NEG	NEG	NEG	NEG	POS	POS	NEG	NEG	POS	POS	NEG	POS	NEG
**>"European Clade": UK-**																**NEG**
ANS46 (Australia)	POS	AMB	AMB	NEG	NEG	NEG	NEG	POS	POS	NEG	NEG	POS	POS	NEG	POS	NEG
LHH1 (USA)	POS	AMB	AMB	NEG	NEG	NEG	NEG	POS	POS	NEG	NEG	POS	POS	NEG	POS	NEG
Dublin-DSH_AR01_0-1085	POS	POS	AMB	AMB	NEG	NEG	NEG	POS	POS	NEG	NEG	POS	POS	NEG	POS	NEG
Dublin-DSH_AR01_0-1118	POS	POS	POS	NEG	NEG	NEG	NEG	POS	POS	NEG	NEG	POS	POS	NEG	POS	NEG
Dublin-DSH_AR15_0098	POS	POS	NEG	AMB	NEG	NEG	NEG	POS	POS	NEG	NEG	POS	POS	NEG	POS	NEG
Dublin-DSH_AR15_0104	POS	POS	POS	AMB	NEG	NEG	NEG	POS	POS	NEG	NEG	POS	POS	NEG	POS	NEG
UK_NCTC11939_UK-1_Harmony	POS	POS	AMB	NEG	NEG	NEG	NEG	POS	POS	NEG	NEG	POS	POS	NEG	POS	NEG
**>"European Clade": BK2**																**NEG**
BK2421 (USA)	POS	AMB	AMB	NEG	NEG	NEG	NEG	POS	POS	NEG	NEG	POS	POS	NEG	POS	NEG
**>"Aussi/NZ Clade", JKD6**																**NEG**
JKD6009 (Australia)	POS	AMB	AMB	NEG	NEG	NEG	NEG	POS	POS	NEG	NEG	POS	POS	NEG	POS	NEG
JKD6008 (New Zealand)	POS	AMB	AMB	NEG	NEG	NEG	NEG	POS	POS	NEG	NEG	POS	POS	NEG	POS	NEG
Perth_2015-S20209	POS	AMB	AMB	NEG	NEG	NEG	NEG	POS	POS	NEG	NEG	POS	POS	NEG	POS	NEG
Perth_2015-S20305	POS	AMB	AMB	NEG	NEG	NEG	NEG	POS	POS	NEG	NEG	POS	POS	NEG	POS	NEG
Perth_2015-S20603	POS	AMB	AMB	NEG	NEG	NEG	NEG	POS	POS	NEG	NEG	POS	POS	NEG	POS	NEG
Perth_2015-S20607	POS	AMB	AMB	NEG	NEG	NEG	NEG	POS	POS	NEG	NEG	POS	POS	NEG	POS	NEG
Perth_2015-S20608	POS	AMB	AMB	NEG	NEG	NEG	NEG	POS	POS	NEG	NEG	POS	POS	NEG	POS	NEG
Perth_2015-S20610	POS	AMB	AMB	NEG	NEG	NEG	NEG	POS	POS	NEG	NEG	POS	POS	NEG	POS	NEG
Perth_2015-S20613	POS	AMB	AMB	NEG	NEG	NEG	NEG	POS	POS	NEG	NEG	POS	POS	NEG	POS	NEG
Perth_2015-S20617	POS	AMB	AMB	NEG	NEG	NEG	NEG	POS	POS	NEG	NEG	POS	POS	NEG	POS	NEG
Perth_2015-S20622	POS	AMB	AMB	NEG	NEG	NEG	NEG	POS	POS	NEG	NEG	POS	POS	NEG	POS	NEG
Perth_2015-S23619	POS	AMB	AMB	NEG	NEG	NEG	NEG	POS	POS	NEG	NEG	POS	POS	NEG	POS	NEG
Perth_2015-S41210	POS	AMB	AMB	NEG	NEG	NEG	NEG	POS	POS	NEG	NEG	POS	POS	NEG	POS	NEG
Perth_2015-S51001	POS	AMB	AMB	NEG	NEG	NEG	NEG	POS	POS	NEG	NEG	POS	POS	NEG	POS	NEG
Perth_2015-S51003	POS	AMB	AMB	NEG	NEG	NEG	NEG	POS	POS	NEG	NEG	POS	POS	NEG	POS	NEG
Perth_2015-S51006	POS	AMB	AMB	NEG	NEG	NEG	NEG	POS	POS	NEG	NEG	POS	POS	NEG	POS	NEG
Perth_2015-S51007	POS	AMB	AMB	NEG	NEG	NEG	NEG	POS	POS	NEG	NEG	POS	POS	NEG	POS	NEG
Perth_2015-S51008	POS	AMB	AMB	NEG	NEG	NEG	NEG	POS	POS	NEG	NEG	POS	POS	NEG	POS	NEG
Perth_2015-S51014	POS	AMB	AMB	NEG	NEG	NEG	NEG	POS	POS	NEG	NEG	POS	POS	NEG	POS	NEG
Perth_2015-S51408	POS	AMB	AMB	NEG	NEG	NEG	NEG	POS	POS	NEG	NEG	POS	POS	NEG	POS	NEG
Perth_2016-S20614	POS	AMB	AMB	NEG	NEG	NEG	NEG	POS	POS	NEG	NEG	POS	POS	NEG	POS	NEG
Perth_2016-S23614	POS	AMB	AMB	NEG	NEG	NEG	NEG	POS	POS	NEG	NEG	POS	POS	NEG	POS	NEG
Perth_2016-S42009	POS	AMB	AMB	NEG	NEG	NEG	NEG	POS	POS	NEG	NEG	POS	POS	NEG	POS	NEG
Perth_2016-S51001	POS	AMB	AMB	NEG	NEG	NEG	NEG	POS	POS	NEG	NEG	POS	POS	NEG	POS	NEG
Perth_2016-S51403	POS	AMB	AMB	NEG	NEG	NEG	NEG	POS	POS	NEG	NEG	POS	POS	NEG	POS	NEG
Perth_07 RPA 20	POS	POS	AMB	NEG	NEG	NEG	NEG	POS	POS	NEG	NEG	POS	POS	NEG	POS	NEG
Perth_08-19088	POS	POS	AMB	AMB	NEG	NEG	NEG	POS	POS	NEG	AMB	POS	POS	NEG	POS	NEG
Perth_01-15351	POS	POS	AMB	NEG	NEG	NEG	NEG	POS	POS	NEG	NEG	POS	POS	NEG	POS	NEG
Perth_01-15357	POS	POS	POS	NEG	NEG	NEG	NEG	POS	POS	NEG	NEG	POS	POS	NEG	POS	NEG
Perth_01-15419	POS	AMB	POS	NEG	NEG	NEG	NEG	POS	POS	NEG	NEG	POS	POS	NEG	POS	NEG
Perth_01-16309	POS	POS	AMB	NEG	NEG	NEG	NEG	POS	POS	NEG	NEG	POS	POS	NEG	POS	NEG
Perth_03 SNP 95	POS	POS	AMB	AMB	NEG	NEG	NEG	POS	POS	NEG	POS	POS	POS	NEG	POS	NEG
Perth_2003_AH_47	POS	POS	AMB	AMB	NEG	NEG	NEG	POS	POS	NEG	POS	POS	POS	NEG	POS	
**>"Australian/NZ Clade":**																**NEG**
Perth_2015-S20623	POS	AMB	AMB	NEG	NEG	NEG	NEG	POS	POS	NEG	NEG	POS	POS	NEG	POS	NEG
Perth_2015-S20826	POS	AMB	AMB	NEG	NEG	NEG	NEG	POS	POS	NEG	NEG	POS	POS	NEG	POS	NEG
Perth_05 NH 56	POS	POS	AMB	AMB	NEG	NEG	NEG	POS	POS	NEG	AMB	POS	POS	NEG	POS	NEG
**>CC5-MRSA-III LA MRSA**																**NEG**
Delbrück 38 isolate from domestic turkey	POS	NEG	POS	AMB	NEG	AMB	NEG	POS	POS	NEG	POS	POS	POS	NEG	POS	NEG
**> Staph. pseudintermed**																**NEG**
KM1381	NEG	NEG	NEG	NEG	NEG	NEG	NEG	NEG	NEG	NEG	NEG	NEG	NEG	NEG	NEG	NEG
**>"South-East Asian Clad**																**POS**
M92 (Canada)	POS	AMB	AMB	NEG	NEG	NEG	NEG	POS	POS	NEG	NEG	POS	POS	NEG	POS	POS
CHI59 (China)	POS	AMB	AMB	NEG	NEG	NEG	NEG	POS	POS	NEG	NEG	POS	POS	NEG	POS	POS
CHI61 (China)	POS	AMB	AMB	NEG	NEG	NEG	NEG	POS	POS	NEG	NEG	POS	POS	NEG	POS	POS
CUHK_HK1997 (China)	POS	AMB	AMB	NEG	NEG	NEG	NEG	POS	POS	NEG	NEG	POS	POS	NEG	POS	POS
M996 (China)	POS	AMB	AMB	NEG	NEG	NEG	NEG	POS	POS	NEG	NEG	POS	POS	NEG	POS	POS
H211 (Denmark)	POS	AMB	AMB	NEG	NEG	NEG	NEG	POS	POS	NEG	NEG	POS	POS	NEG	POS	POS
H216 (Denmark)	POS	AMB	AMB	NEG	NEG	NEG	NEG	POS	POS	NEG	NEG	POS	POS	NEG	POS	POS
D71 (Germany)	POS	AMB	AMB	NEG	NEG	NEG	NEG	POS	POS	NEG	NEG	POS	POS	NEG	POS	POS
NMR05 (India)	POS	AMB	AMB	NEG	NEG	NEG	NEG	POS	POS	NEG	NEG	POS	POS	NEG	POS	POS
MAL11 (Malaysia)	POS	AMB	AMB	NEG	NEG	NEG	NEG	POS	POS	NEG	NEG	POS	POS	NEG	POS	POS
MAL9 (Malaysia)	POS	AMB	AMB	NEG	NEG	NEG	NEG	POS	POS	NEG	NEG	POS	POS	NEG	POS	POS
Na21 (Sri Lanka)	POS	AMB	AMB	NEG	NEG	NEG	NEG	POS	POS	NEG	NEG	POS	POS	NEG	POS	POS
M592 (Syria)	POS	AMB	AMB	NEG	NEG	NEG	NEG	POS	POS	NEG	NEG	POS	POS	NEG	POS	POS
H202 (Thailand)	POS	AMB	AMB	NEG	NEG	NEG	NEG	POS	POS	NEG	NEG	POS	POS	NEG	POS	POS
S102 (Thailand)	POS	AMB	AMB	NEG	NEG	NEG	NEG	POS	POS	NEG	NEG	POS	POS	NEG	POS	POS
S130 (Thailand)	POS	AMB	AMB	NEG	NEG	NEG	NEG	POS	POS	NEG	NEG	POS	POS	NEG	POS	POS
S40 (Thailand)	POS	AMB	AMB	NEG	NEG	NEG	NEG	POS	POS	NEG	NEG	POS	POS	NEG	POS	POS
S71 (Thailand)	POS	AMB	AMB	NEG	NEG	NEG	NEG	POS	POS	NEG	NEG	POS	POS	NEG	POS	POS
S87 (Thailand)	POS	AMB	AMB	NEG	NEG	NEG	NEG	POS	POS	NEG	NEG	POS	POS	NEG	POS	POS
S93 (Thailand)	POS	AMB	AMB	NEG	NEG	NEG	NEG	POS	POS	NEG	NEG	POS	POS	NEG	POS	POS
US_002 (Thailand)	POS	AMB	AMB	NEG	NEG	NEG	NEG	POS	POS	NEG	NEG	POS	POS	NEG	POS	POS
US_017 (Thailand)	POS	AMB	AMB	NEG	NEG	NEG	NEG	POS	POS	NEG	NEG	POS	POS	NEG	POS	POS
WAMC6102 (Thailand)	POS	AMB	AMB	NEG	NEG	NEG	NEG	POS	POS	NEG	NEG	POS	POS	NEG	POS	POS
NCTC_13626_TW20 (UK)	POS	AMB	AMB	NEG	NEG	NEG	NEG	POS	POS	NEG	NEG	POS	POS	NEG	POS	POS
BSAC3021 (UK)	POS	AMB	AMB	NEG	NEG	NEG	NEG	POS	POS	NEG	NEG	POS	POS	NEG	POS	POS
BSAC3046 (UK)	POS	AMB	AMB	NEG	NEG	NEG	NEG	POS	POS	NEG	NEG	POS	POS	NEG	POS	POS
1193_SAUR (USA)	POS	AMB	AMB	NEG	NEG	NEG	NEG	POS	POS	NEG	NEG	POS	POS	NEG	POS	POS
1194_SAUR (USA)	POS	AMB	AMB	NEG	NEG	NEG	NEG	POS	POS	NEG	NEG	POS	POS	NEG	POS	POS

STRAIN / ISOLATE	ADHAESION FACTORS / GENES ENCODING MICROBIAL SURFACE COMPONENTS RECOGNIZING ADHESIVE MATRIX MOLECULES															
	fnbB							map				sasG				sasX / sesI
	fnbB	fnbB (COL)	fnbB (COL+Mu50+MW2)	fnbB (Mu50)	fnbB (MW2)	fnbB (ST15)	fnbB (ST45-2)	map	map (COL)	map (MRSA252)	map (Mu50+MW2)	sasG	sasG (COL+Mu50)	sasG (MW2)	sasG (OtherThan252+122)	
	Fibronectin-binding protein B							Major histocompatibility complex class II analog protein (=Extracellular adherence protein, eap)				Staphylococcus aureus surface protein G				
>"South-East Asian Clade": TW20 (Irish AR44), c																
1195_SAUR (USA)	POS	AMB	AMB	NEG	NEG	NEG	NEG	POS	POS	NEG	NEG	POS	POS	NEG	POS	POS
FVRH6002 (USA)	POS	AMB	AMB	NEG	NEG	NEG	NEG	POS	POS	NEG	NEG	POS	POS	NEG	POS	POS
KINW6048 (USA)	POS	AMB	AMB	NEG	NEG	NEG	NEG	POS	POS	NEG	NEG	POS	POS	NEG	POS	POS
LAMC0011 (USA)	POS	AMB	AMB	NEG	NEG	NEG	NEG	POS	POS	NEG	NEG	POS	POS	NEG	POS	POS
SJOS6053 (USA)	POS	AMB	AMB	NEG	NEG	NEG	NEG	POS	POS	NEG	NEG	POS	POS	NEG	POS	POS
SJOS6072 (USA)	POS	AMB	AMB	NEG	NEG	NEG	NEG	POS	POS	NEG	NEG	POS	POS	NEG	POS	POS
UCIM6042 (USA)	POS	AMB	AMB	NEG	NEG	NEG	NEG	POS	POS	NEG	NEG	POS	POS	NEG	POS	POS
Perth_2015-S31902	POS	AMB	AMB	NEG	NEG	NEG	NEG	NEG	NEG	NEG	NEG	POS	POS	NEG	POS	POS
Perth_2015-S31904	POS	AMB	AMB	NEG	NEG	NEG	NEG	POS	POS	NEG	NEG	POS	POS	NEG	POS	POS
Perth_2015-S31913	POS	AMB	AMB	NEG	NEG	NEG	NEG	POS	POS	NEG	NEG	POS	POS	NEG	POS	POS
Perth_2015-S31918	POS	AMB	AMB	NEG	NEG	NEG	NEG	NEG	NEG	NEG	NEG	POS	POS	NEG	POS	POS
Dublin-DSH_AR44_E1183	POS	POS	AMB	AMB	NEG	NEG	NEG	POS	POS	NEG	NEG	POS	POS	NEG	POS	POS
Dublin-DSH_Unfamiliar-3_E1520	POS	POS	AMB	NEG	NEG	NEG	NEG	POS	POS	NEG	NEG	POS	POS	NEG	POS	POS
Hong Kong_042	POS	POS	AMB	AMB	NEG	NEG	NEG	POS	POS	NEG	NEG	POS	POS	NEG	POS	POS
Hong Kong_101	POS	POS	AMB	AMB	NEG	NEG	NEG	POS	POS	NEG	NEG	POS	POS	NEG	POS	POS
Hong Kong_111	AMB	POS	AMB	AMB	NEG	NEG	NEG	AMB	POS	NEG	POS	AMB	POS	NEG	POS	POS
Hong Kong_113	POS	POS	AMB	NEG	NEG	NEG	NEG	POS	POS	NEG	NEG	POS	POS	NEG	POS	POS
Hong Kong_115	POS	POS	AMB	AMB	NEG	NEG	NEG	POS	POS	NEG	NEG	POS	POS	NEG	POS	POS
Hong Kong_118	POS	POS	AMB	AMB	NEG	NEG	NEG	POS	POS	NEG	AMB	POS	POS	NEG	POS	POS
Hong Kong_125	POS	POS	AMB	AMB	NEG	NEG	NEG	POS	POS	NEG	AMB	POS	POS	NEG	POS	POS
Hong Kong_129	POS	POS	POS	NEG	NEG	NEG	NEG	POS	POS	NEG	NEG	POS	POS	NEG	POS	POS
Hong Kong_133	POS	POS	AMB	AMB	NEG	NEG	NEG	POS	POS	NEG	AMB	POS	POS	NEG	POS	POS
Kuwait_009	POS	POS	AMB	AMB	NEG	NEG	NEG	POS	POS	NEG	NEG	POS	POS	NEG	POS	POS
Kuwait_103	POS	POS	AMB	AMB	NEG	NEG	NEG	POS	POS	NEG	POS	POS	POS	NEG	POS	POS
Kuwait_107	POS	POS	AMB	AMB	NEG	NEG	NEG	POS	POS	NEG	POS	POS	POS	NEG	POS	POS
Perth_03 AH 88	POS	POS	AMB	NEG	NEG	NEG	NEG	POS	POS	NEG	NEG	POS	POS	NEG	POS	POS
Perth_08-16905	POS	POS	AMB	NEG	NEG	NEG	NEG	POS	POS	NEG	NEG	POS	POS	NEG	POS	POS
Riyadh_Alfaisal-10_SICU_13_B5672_1117353	POS	POS	AMB	AMB	NEG	NEG	NEG	POS	POS	NEG	NEG	POS	POS	NEG	POS	POS
Riyadh_SSTI_17_3508602	POS	POS	AMB	AMB	NEG	NEG	NEG	POS	POS	NEG	POS	POS	POS	NEG	POS	POS
Riyadh-2822088-R	POS	POS	AMB	AMB	NEG	NEG	NEG	POS	POS	NEG	POS	POS	POS	NEG	POS	POS
Trinidad&Tobago_2012_195 (619/12)	POS	POS	AMB	AMB	NEG	NEG	NEG	POS	POS	NEG	AMB	POS	POS	NEG	POS	POS
Trinidad&Tobago_2012_370 (1112-52177)	POS	AMB	AMB	POS	NEG	NEG	NEG	POS	POS	NEG	POS	POS	POS	NEG	POS	POS
Trinidad&Tobago_2012_378 (4139)	POS	AMB	AMB	POS	NEG	NEG	NEG	POS	POS	NEG	AMB	POS	POS	NEG	POS	POS
Trinidad&Tobago_2013_PA21	POS	POS	AMB	NEG	NEG	NEG	NEG	POS	POS	NEG	NEG	POS	POS	NEG	POS	POS
Trinidad&Tobago_2013_PA22	POS	POS	AMB	AMB	NEG	NEG	NEG	POS	POS	NEG	POS	POS	POS	NEG	POS	POS
Trinidad&Tobago_2912_340 (8523/12)	POS	AMB	AMB	POS	NEG	NEG	NEG	POS	POS	NEG	AMB	POS	POS	NEG	POS	POS
Trinidad&Tobago_MRSA2010_105	POS	POS	AMB	AMB	NEG	NEG	NEG	POS	POS	NEG	AMB	POS	POS	NEG	POS	
Trinidad&Tobago_MRSA2010_108	POS	POS	AMB	AMB	NEG	NEG	NEG	POS	POS	NEG	POS	POS	POS	AMB	POS	POS
Trinidad&Tobago_MRSA2010_33	POS	POS	AMB	AMB	NEG	NEG	NEG	POS	POS	NEG	NEG	POS	POS	NEG	POS	POS
Trinidad&Tobago_MRSA2010_79	POS	POS	AMB	AMB	NEG	NEG	NEG	POS	POS	NEG	NEG	POS	POS	NEG	POS	POS
Trinidad&Tobago_MRSA2010_88	POS	POS	AMB	AMB	NEG	NEG	NEG	POS	POS	NEG	NEG	POS	POS	NEG	POS	POS
Trinidad&Tobago_MRSA2010_97	POS	POS	AMB	AMB	NEG	NEG	NEG	POS	POS	NEG	AMB	POS	POS	AMB	POS	POS
Trinidad&Tobago_SSTI 2012_031	POS	POS	AMB	AMB	NEG	NEG	NEG	POS	POS	NEG	AMB	POS	POS	NEG	POS	POS
Uganda-29_17148_250811	POS	POS	AMB	AMB	NEG	NEG	NEG	POS	POS	NEG	NEG	POS	POS	NEG	POS	POS
>"South-East Asian Clad																POS
UCIM6015	POS	AMB	AMB	NEG	NEG	NEG	NEG	POS	POS	NEG	NEG	POS	POS	NEG	POS	POS
Hong Kong_121	POS	POS	AMB	NEG	NEG	NEG	NEG	POS	POS	NEG	NEG	POS	POS	NEG	POS	POS
>"South-East Asian Clad																POS
Z172 (Taiwan)	POS	AMB	AMB	NEG	NEG	NEG	NEG	POS	POS	NEG	NEG	POS	POS	NEG	POS	POS
Russia-19_0342_Kurgan	POS	POS	AMB	NEG	NEG	NEG	NEG	POS	POS	NEG	NEG	POS	POS	NEG	POS	POS
>"South-East Asian Clad																POS
Perth_01-15392	POS	POS	POS	NEG	NEG	NEG	NEG	POS	POS	NEG	NEG	POS	POS	NEG	POS	POS
>"South-East Asian Clad																POS
XN108 (China)	POS	AMB	AMB	NEG	NEG	NEG	NEG	POS	POS	NEG	NEG	POS	POS	NEG	POS	POS
NMR09 (India)	POS	AMB	AMB	NEG	NEG	NEG	NEG	POS	POS	NEG	NEG	POS	POS	NEG	POS	POS
346 (Malaysia)	POS	AMB	AMB	NEG	NEG	NEG	NEG	POS	POS	NEG	NEG	POS	POS	NEG	POS	POS
DS_009 (Thailand)	POS	AMB	AMB	NEG	NEG	NEG	NEG	POS	POS	NEG	NEG	POS	POS	NEG	POS	POS
18ANR581005 (Ind)	POS	POS	AMB	AMB	NEG	NEG	NEG	POS	POS	NEG	NEG	POS	POS	NEG	POS	POS
Bengalaru_ST20141405	POS	POS	AMB	AMB	NEG	NEG	NEG	POS	POS	NEG	POS	POS	POS	NEG	POS	POS
Bengalaru_ST20141406	POS	POS	AMB	AMB	NEG	NEG	NEG	POS	POS	NEG	POS	POS	POS	NEG	POS	POS
Bengalaru_ST20141417	POS	POS	AMB	AMB	NEG	NEG	NEG	POS	POS	NEG	NEG	POS	POS	NEG	POS	POS
Hong Kong_107	AMB	POS	AMB	AMB	NEG	NEG	NEG	AMB	POS	NEG	NEG	AMB	POS	NEG	POS	POS
Hong Kong_12	POS	POS	AMB	NEG	NEG	NEG	NEG	POS	POS	NEG	AMB	POS	POS	NEG	POS	POS
Hong Kong_132	AMB	POS	AMB	AMB	NEG	NEG	NEG	AMB	POS	NEG	AMB	AMB	POS	NEG	POS	POS
Riyadh-3028763-R	POS	POS	AMB	AMB	NEG	NEG	NEG	POS	POS	NEG	AMB	POS	POS	NEG	POS	POS
Trinidad&Tobago_MRSA2010_94	POS	POS	AMB	AMB	NEG	NEG	NEG	POS	POS	NEG	NEG	POS	POS	NEG	POS	pos
>"South-East Asian Clad																POS
M705 (Thailand)	POS	AMB	AMB	NEG	NEG	NEG	NEG	POS	POS	NEG	NEG	POS	POS	NEG	POS	POS
S85 (Thailand)	POS	AMB	AMB	NEG	NEG	NEG	NEG	POS	POS	NEG	NEG	POS	POS	NEG	POS	POS
Riyadh_Alfaisal-04_281014_S5076	POS	POS	AMB	AMB	AMB	NEG	NEG	POS	POS	NEG	POS	POS	POS	AMB	POS	POS
Riyadh_Alfaisal-26_S15145_1104163	POS	POS	AMB	AMB	NEG	NEG	NEG	POS	POS	NEG	POS	POS	POS	NEG	POS	POS
Riyadh-2817437-W	POS	POS	AMB	AMB	NEG	NEG	NEG	POS	POS	NEG	POS	POS	POS	NEG	POS	POS
>"South-East Asian Clad																POS
PPUKM-775-2009 (Malaysia)	POS	AMB	AMB	NEG	NEG	NEG	NEG	POS	POS	NEG	NEG	POS	POS	NEG	POS	POS
Hong Kong_138	POS	POS	AMB	AMB	NEG	NEG	NEG	POS	POS	NEG	AMB	POS	POS	NEG	POS	POS
>"South-East Asian Clad																POS
Kuwait_001	POS	POS	AMB	AMB	NEG	NEG	NEG	POS	POS	NEG	AMB	POS	POS	NEG	POS	POS
Kuwait_337	POS	POS	AMB	AMB	NEG	NEG	NEG	POS	POS	NEG	POS	POS	POS	NEG	POS	POS
>"South-East Asian Clad																POS
CUHK_HK2007 (China)	POS	AMB	AMB	NEG	NEG	NEG	NEG	POS	POS	NEG	NEG	POS	POS	NEG	POS	POS
NMR02 (India)	POS	AMB	AMB	NEG	NEG	NEG	NEG	POS	POS	NEG	NEG	POS	POS	NEG	POS	POS
VB1490 (India)	POS	AMB	AMB	NEG	NEG	NEG	NEG	POS	POS	NEG	NEG	POS	POS	NEG	POS	POS
V521 (Korea)	POS	AMB	AMB	NEG	NEG	NEG	NEG	POS	POS	NEG	NEG	POS	POS	NEG	POS	POS
HST-077 (Lebanon)	NEG	NEG	NEG	NEG	NEG	NEG	NEG	POS	POS	NEG	NEG	POS	POS	NEG	POS	POS
MAL1 (Malaysia)	POS	AMB	AMB	NEG	NEG	NEG	NEG	POS	POS	NEG	NEG	POS	POS	NEG	POS	POS
MAL3 (Malaysia)	POS	AMB	AMB	NEG	NEG	NEG	NEG	POS	POS	NEG	NEG	POS	POS	NEG	POS	POS
PPUKM-261-2009 (Malaysia)	POS	AMB	AMB	NEG	NEG	NEG	NEG	POS	POS	NEG	NEG	POS	POS	NEG	POS	POS
PPUKM-332-2009 (Malaysia)	POS	AMB	AMB	NEG	NEG	NEG	NEG	POS	POS	NEG	NEG	POS	POS	NEG	POS	POS
S38 (Thailand)	POS	AMB	AMB	NEG	NEG	NEG	NEG	POS	POS	NEG	NEG	POS	POS	NEG	POS	POS
S7 (Thailand)	POS	AMB	AMB	NEG	NEG	NEG	NEG	POS	POS	NEG	NEG	POS	POS	NEG	POS	POS
US_008 (Thailand)	POS	AMB	AMB	NEG	NEG	NEG	NEG	POS	POS	NEG	NEG	POS	POS	NEG	POS	POS
US_030 (Thailand)	POS	AMB	AMB	NEG	NEG	NEG	NEG	POS	POS	NEG	NEG	POS	POS	NEG	POS	POS
M170 (Tsunami-related)	POS	AMB	AMB	NEG	NEG	NEG	NEG	POS	POS	NEG	NEG	POS	POS	NEG	POS	POS
BSAC697 (UK)	POS	AMB	AMB	NEG	NEG	NEG	NEG	POS	POS	NEG	NEG	POS	POS	NEG	POS	POS
IS-189	NEG	NEG	NEG	NEG	NEG	NEG	NEG	POS	POS	NEG	NEG	POS	POS	NEG	POS	POS
MRGR3	POS	AMB	AMB	NEG	NEG	NEG	NEG	POS	POS	NEG	NEG	POS	POS	NEG	POS	POS
smg07	POS	AMB	AMB	NEG	NEG	NEG	NEG	POS	POS	NEG	NEG	POS	POS	NEG	POS	POS
AH1-AUS-EMRSA-3	AMB	POS	AMB	AMB	NEG	NEG	NEG	AMB	POS	NEG	NEG	AMB	POS	NEG	POS	POS
Bengalaru_ST20141403	POS	POS	AMB	AMB	NEG	NEG	NEG	POS	POS	NEG	POS	POS	POS	NEG	POS	POS
Finland E24_98541_Harmony	POS	POS	AMB	AMB	NEG	NEG	NEG	POS	POS	NEG	AMB	POS	POS	NEG	POS	POS
Hong Kong_10	POS	POS	AMB	AMB	NEG	NEG	NEG	POS	POS	NEG	AMB	POS	POS	NEG	POS	POS
Hong Kong_100	POS	POS	AMB	AMB	NEG	NEG	NEG	POS	POS	NEG	AMB	POS	POS	NEG	POS	POS
Hong Kong_199	POS	POS	AMB	AMB	NEG	NEG	NEG	POS	POS	NEG	POS	POS	POS	NEG	POS	POS
Hong Kong_34	POS	POS	AMB	AMB	NEG	NEG	NEG	POS	POS	NEG	POS	POS	POS	NEG	POS	POS
Hong Kong_88	POS	POS	AMB	AMB	NEG	NEG	NEG	POS	POS	NEG	POS	POS	POS	AMB	POS	POS
Hong Kong_92	POS	POS	AMB	AMB	NEG	NEG	NEG	POS	POS	NEG	AMB	POS	POS	NEG	POS	POS
Hong Kong_94	POS	POS	AMB	NEG	NEG	NEG	NEG	POS	POS	NEG	NEG	POS	POS	NEG	POS	POS
Kuwait_257	NEG	NEG	NEG	NEG	NEG	NEG	NEG	POS	POS	NEG	NEG	POS	POS	NEG	POS	POS
Perth_01-15330	POS	POS	POS	NEG	NEG	NEG	NEG	POS	POS	NEG	NEG	POS	POS	NEG	POS	POS
Perth_01-15349	POS	POS	POS	NEG	NEG	NEG	NEG	POS	POS	NEG	NEG	POS	POS	NEG	POS	POS
Perth_01-16176	POS	POS	AMB	NEG	NEG	NEG	NEG	POS	POS	NEG	NEG	POS	POS	NEG	POS	POS
Perth_WBG8379	POS	POS	AMB	NEG	NEG	NEG	NEG	POS	POS	NEG	NEG	POS	POS	NEG	POS	POS
Rawalpindi_Kidney Center_56	POS	POS	AMB	NEG	NEG	NEG	NEG	POS	POS	NEG	POS	POS	POS	NEG	POS	POS
Riyadh_SSTI_05_3464614	POS	POS	AMB	NEG	NEG	NEG	NEG	POS	POS	NEG	NEG	POS	POS	NEG	POS	POS
Trinidad&Tobago_2012_157	POS	AMB	AMB	POS	NEG	NEG	NEG	POS	POS	NEG	AMB	POS	POS	NEG	POS	POS

STRAIN / ISOLATE	ADHAESION FACTORS / GENES ENCODING MICROBIAL SURFACE COMPONENTS RECOGNIZING ADHESIVE MATRIX MOLECULES															
	fnbB							map				sasG				sasX / sesI
	fnbB	fnbB (COL)	fnbB (COL+Mu50+MW2)	fnbB (Mu50)	fnbB (MW2)	fnbB (ST15)	fnbB (ST45-2)	map	map (COL)	map (MRSA252)	map (Mu50+MW2)	sasG	sasG (COL+Mu50)	sasG (MW2)	sasG (OtherThan252+122	
	Fibronectin-binding protein B							Major histocompatibility complex class II analog protein (=Extracellular adherence protein, eap)				Staphylococcus aureus surface protein G				
>"South-East Asian Clad																POS
M418	POS	AMB	AMB	NEG	NEG	NEG	NEG	POS	POS	NEG	NEG	POS	POS	NEG	POS	POS
>"South-East Asian Clad																POS
NMR07 (India)	POS	AMB	AMB	NEG	NEG	NEG	NEG	POS	POS	NEG	NEG	POS	POS	NEG	POS	POS
NMR08 (India)	POS	AMB	AMB	NEG	NEG	NEG	NEG	POS	POS	NEG	NEG	POS	POS	NEG	POS	POS
Bengalaru_ST20121978	POS	POS	AMB	AMB	NEG	NEG	NEG	POS	POS	NEG	POS	POS	POS	NEG	POS	POS
Hong Kong_89	POS	AMB	AMB	POS	NEG	NEG	NEG	POS	POS	NEG	POS	POS	POS	AMB	POS	POS
>"South-East Asian Clad																POS
Perth_2005_AH_5	POS	POS	AMB	NEG	NEG	NEG	NEG	POS	POS	NEG	NEG	POS	POS	NEG	POS	POS
>"South-East Asian Clad																POS
Trinidad&Tobago_MRSA2010_103	POS	POS	AMB	NEG	NEG	NEG	NEG	POS	POS	NEG	AMB	POS	POS	NEG	POS	POS
Trinidad&Tobago_2013_PA25	POS	POS	AMB	AMB	NEG	NEG	NEG	POS	POS	NEG	POS	POS	POS	NEG	POS	POS
Trinidad&Tobago_MRSA2010_110	POS	POS	AMB	AMB	NEG	NEG	NEG	POS	POS	NEG	AMB	POS	POS	AMB	POS	POS
Trinidad&Tobago_SSTI 2012_111	POS	POS	AMB	POS	NEG	NEG	NEG	POS	POS	NEG	AMB	POS	POS	NEG	POS	POS
Trinidad&Tobago_MRSA2010_158	POS	POS	AMB	AMB	NEG	NEG	NEG	POS	POS	NEG	NEG	POS	POS	NEG	POS	POS
>"South-East Asian Clad																POS
Kuwait_122	POS	POS	AMB	AMB	NEG	NEG	NEG	POS	POS	NEG	NEG	POS	POS	NEG	POS	POS
Kuwait_002	POS	POS	AMB	NEG	NEG	NEG	NEG	POS	POS	NEG	NEG	POS	POS	NEG	POS	POS
Kuwait_196	POS	POS	AMB	AMB	NEG	NEG	NEG	POS	POS	NEG	NEG	POS	POS	NEG	POS	POS
Kuwait_199	POS	POS	AMB	POS	NEG	NEG	NEG	POS	POS	NEG	AMB	POS	POS	NEG	POS	POS
Hong Kong_136	POS	POS	AMB	NEG	NEG	NEG	NEG	POS	POS	NEG	NEG	POS	POS	NEG	POS	POS
>"South-East Asian Clad																POS
P32 (Poland)	POS	AMB	AMB	NEG	NEG	NEG	NEG	POS	POS	NEG	NEG	POS	POS	NEG	POS	POS
>"South-East Asian Clad																POS
Perth_2015-S32313	POS	AMB	AMB	NEG	NEG	NEG	NEG	POS	POS	NEG	NEG	POS	POS	NEG	POS	POS
>"hla-negative South-Ea																POS
DEN907 (Denmark)	POS	AMB	AMB	NEG	NEG	NEG	NEG	POS	POS	NEG	NEG	POS	POS	NEG	POS	POS
>"hla-negative South-Ea																POS
S26 (Thailand)	POS	AMB	AMB	NEG	NEG	NEG	NEG	POS	POS	NEG	NEG	POS	POS	NEG	POS	POS
S42 (Thailand)	POS	AMB	AMB	NEG	NEG	NEG	NEG	POS	POS	NEG	NEG	POS	POS	NEG	POS	POS
S97 (Thailand)	POS	AMB	AMB	NEG	NEG	NEG	NEG	POS	POS	NEG	NEG	POS	POS	NEG	POS	POS
S78 (Thailand)	POS	AMB	AMB	NEG	NEG	NEG	NEG	POS	POS	NEG	NEG	POS	POS	NEG	POS	POS
S25 (Thailand)	POS	AMB	AMB	NEG	NEG	NEG	NEG	POS	POS	NEG	NEG	POS	POS	NEG	POS	POS
S21 (Thailand)	POS	AMB	AMB	NEG	NEG	NEG	NEG	POS	POS	NEG	NEG	POS	POS	NEG	POS	POS
S106 (Thailand)	POS	AMB	AMB	NEG	NEG	NEG	NEG	POS	POS	NEG	NEG	POS	POS	NEG	POS	POS
S24 (Thailand)	POS	AMB	AMB	NEG	NEG	NEG	NEG	POS	POS	NEG	NEG	POS	POS	NEG	POS	POS
S39 (Thailand)	POS	AMB	AMB	NEG	NEG	NEG	NEG	POS	POS	NEG	NEG	POS	POS	NEG	POS	POS
S2 (Thailand)	POS	AMB	AMB	NEG	NEG	NEG	NEG	POS	POS	NEG	NEG	POS	POS	NEG	POS	POS
S81 (Thailand)	POS	AMB	AMB	NEG	NEG	NEG	NEG	POS	POS	NEG	NEG	POS	POS	NEG	POS	POS
M116 (Vietnam)	POS	AMB	AMB	NEG	NEG	NEG	NEG	POS	POS	NEG	NEG	POS	POS	NEG	POS	POS
>"Portuguese Clade": Pc																NEG
FFP103 (Portugal)	POS	AMB	AMB	NEG	NEG	NEG	NEG	POS	POS	NEG	NEG	POS	POS	NEG	POS	NEG
HDG2 (Portugal)	POS	AMB	AMB	NEG	NEG	NEG	NEG	POS	POS	NEG	NEG	POS	POS	NEG	POS	NEG
HSA10 (Portugal)	POS	AMB	AMB	NEG	NEG	NEG	NEG	POS	POS	NEG	NEG	POS	POS	NEG	POS	NEG
HSA11 (Portugal)	POS	AMB	AMB	NEG	NEG	NEG	NEG	POS	POS	NEG	NEG	POS	POS	NEG	POS	NEG
ICP5011 (Portugal)	POS	AMB	AMB	NEG	NEG	NEG	NEG	POS	POS	NEG	NEG	POS	POS	NEG	POS	NEG
ICP5014 (Portugal)	POS	AMB	AMB	NEG	NEG	NEG	NEG	POS	POS	NEG	NEG	POS	POS	NEG	POS	NEG
ICP5062 (Portugal)	POS	AMB	AMB	NEG	NEG	NEG	NEG	POS	POS	NEG	NEG	POS	POS	NEG	POS	NEG
Strain 1063_ATCC 33592 FDAARGOS_3 (USA)	POS	AMB	AMB	NEG	NEG	NEG	NEG	POS	POS	NEG	NEG	POS	POS	NEG	POS	NEG
	POS	POS	AMB	AMB	NEG	NEG	NEG	POS	POS	NEG	NEG	POS	POS	NEG	POS	NEG
Russia-07_0110_Kurgan	POS	POS	AMB	NEG	NEG	NEG	NEG	POS	POS	NEG	NEG	POS	POS	NEG	POS	NEG
Russia-11_0164_Kurgan	POS	POS	AMB	AMB	NEG	NEG	NEG	POS	POS	NEG	NEG	POS	POS	NEG	POS	NEG
Russia-20_0367_Kurgan	POS	POS	AMB	AMB	NEG	NEG	NEG	POS	POS	NEG	NEG	POS	POS	NEG	POS	NEG
Russia-21_0390_Chelyabinsk	POS	POS	AMB	AMB	NEG	NEG	NEG	POS	POS	NEG	NEG	POS	POS	NEG	POS	NEG
>"South American/ Mid																NEG
AGT1 (Argentina)	POS	AMB	AMB	NEG	NEG	NEG	NEG	POS	POS	NEG	NEG	POS	POS	NEG	POS	NEG
AGT120 (Argentina)	POS	AMB	AMB	NEG	NEG	NEG	NEG	POS	POS	NEG	NEG	POS	POS	NEG	POS	NEG
AGT67 (Argentina)	NEG	NEG	NEG	NEG	NEG	NEG	NEG	NEG	AMB	NEG	NEG	POS	POS	NEG	POS	NEG
AGT9 (Argentina)	POS	AMB	AMB	NEG	NEG	NEG	NEG	POS	POS	NEG	NEG	POS	POS	NEG	POS	NEG
RA3 (Argentina)	POS	AMB	AMB	NEG	NEG	NEG	NEG	POS	POS	NEG	NEG	POS	POS	NEG	POS	NEG
RA6 (Argentina)	POS	AMB	AMB	NEG	NEG	NEG	NEG	POS	POS	NEG	NEG	POS	POS	NEG	POS	NEG
RA7 (Argentina)	POS	AMB	AMB	NEG	NEG	NEG	NEG	POS	POS	NEG	NEG	POS	POS	NEG	POS	NEG
Be62 (Brazil)	POS	AMB	AMB	NEG	NEG	NEG	NEG	POS	POS	NEG	NEG	POS	POS	NEG	POS	NEG
Bmb9393 (Brazil)	POS	AMB	AMB	NEG	NEG	NEG	NEG	POS	POS	NEG	NEG	POS	POS	NEG	POS	NEG
BRA36 (Brazil)	POS	AMB	AMB	NEG	NEG	NEG	NEG	POS	POS	NEG	NEG	POS	POS	NEG	POS	NEG
BZ48 (Brazil)	POS	AMB	AMB	NEG	NEG	NEG	NEG	POS	POS	NEG	NEG	POS	POS	NEG	POS	NEG
Gv51 (Brazil)	POS	AMB	AMB	NEG	NEG	NEG	NEG	POS	POS	NEG	NEG	POS	POS	NEG	POS	NEG
Gv69 (Brazil)	POS	AMB	AMB	NEG	NEG	NEG	NEG	POS	POS	NEG	NEG	POS	POS	NEG	POS	NEG
Gv88 (Brazil)	POS	AMB	AMB	NEG	NEG	NEG	NEG	POS	POS	NEG	NEG	POS	POS	NEG	POS	NEG
HC1335 (Brazil)	POS	AMB	AMB	NEG	NEG	NEG	NEG	POS	POS	NEG	NEG	POS	POS	NEG	POS	NEG
HC1340 (Brazil)	POS	AMB	AMB	NEG	NEG	NEG	NEG	POS	POS	NEG	NEG	POS	POS	NEG	POS	NEG
HC556 (Brazil)	POS	AMB	AMB	NEG	NEG	NEG	NEG	POS	POS	NEG	NEG	POS	POS	NEG	POS	NEG
HU25 (Brazil)	POS	AMB	AMB	NEG	NEG	NEG	NEG	POS	POS	NEG	NEG	POS	POS	NEG	POS	NEG
UB563 (Brazil)	POS	AMB	AMB	NEG	NEG	NEG	NEG	POS	POS	NEG	NEG	POS	POS	NEG	POS	NEG
2A8 (Czech Republic)	POS	AMB	AMB	NEG	NEG	NEG	NEG	POS	POS	NEG	NEG	POS	POS	NEG	POS	NEG
M1229 (Denmark)	POS	AMB	AMB	NEG	NEG	NEG	NEG	POS	POS	NEG	NEG	POS	POS	NEG	POS	NEG
FRICAR (France)	POS	AMB	AMB	NEG	NEG	NEG	NEG	POS	POS	NEG	NEG	POS	POS	NEG	POS	NEG
D90 (Germany)	POS	AMB	AMB	NEG	NEG	NEG	NEG	POS	POS	NEG	NEG	POS	POS	NEG	POS	NEG
LIT68 (Lithuania)	POS	AMB	AMB	NEG	NEG	NEG	NEG	POS	POS	NEG	NEG	POS	POS	NEG	POS	NEG
LIT76 (Lithuania)	POS	AMB	AMB	NEG	NEG	NEG	NEG	POS	POS	NEG	NEG	POS	POS	NEG	POS	NEG
HGSA142 (Portugal)	POS	AMB	AMB	NEG	NEG	NEG	NEG	POS	POS	NEG	NEG	POS	POS	NEG	POS	NEG
HGSA9 (Portugal)	POS	AMB	AMB	NEG	NEG	NEG	NEG	POS	POS	NEG	NEG	POS	POS	NEG	POS	NEG
HSJ216 (Portugal)	POS	AMB	AMB	NEG	NEG	NEG	NEG	POS	POS	NEG	NEG	POS	POS	NEG	POS	NEG
M278 (Portugal)	POS	AMB	AMB	NEG	NEG	NEG	NEG	POS	POS	NEG	NEG	POS	POS	NEG	POS	NEG
ES26 (Spain)	POS	AMB	AMB	NEG	NEG	NEG	NEG	POS	POS	NEG	NEG	POS	POS	NEG	POS	NEG
UK102 (UK)	POS	AMB	AMB	NEG	NEG	NEG	NEG	POS	POS	NEG	NEG	POS	POS	NEG	POS	NEG
URU34 (Uruguay)	POS	AMB	AMB	NEG	NEG	NEG	NEG	POS	POS	NEG	NEG	POS	POS	NEG	POS	NEG
IS-125	POS	AMB	AMB	NEG	NEG	NEG	NEG	POS	POS	NEG	NEG	POS	POS	NEG	POS	NEG
IS-157	POS	AMB	AMB	NEG	NEG	NEG	NEG	NEG	AMB	NEG	NEG	POS	POS	NEG	POS	NEG
Ecuador_1	POS	POS	AMB	AMB	NEG	NEG	NEG	POS	POS	NEG	NEG	POS	POS	NEG	POS	NEG
Ecuador_2	POS	POS	AMB	AMB	NEG	NEG	NEG	POS	POS	NEG	NEG	POS	POS	NEG	POS	NEG
Dresden_15ANR574263	POS	POS	AMB	AMB	NEG	NEG	NEG	POS	POS	NEG	AMB	POS	POS	NEG	POS	NEG
UK-EMRSA-11	POS	POS	AMB	AMB	NEG	NEG	NEG	POS	POS	NEG	AMB	POS	POS	NEG	POS	NEG
>"South American/ Mid																NEG
BRA2 (Brazil)	POS	AMB	AMB	NEG	NEG	NEG	NEG	POS	POS	NEG	NEG	POS	POS	NEG	POS	NEG
CHL1 (Chile)	POS	AMB	AMB	NEG	NEG	NEG	NEG	POS	POS	NEG	NEG	POS	POS	NEG	POS	NEG
CHL151 (Chile)	POS	AMB	AMB	NEG	NEG	NEG	NEG	POS	POS	NEG	NEG	POS	POS	NEG	POS	NEG
UCL378 (Chile)	POS	AMB	AMB	NEG	NEG	NEG	NEG	POS	POS	NEG	NEG	POS	POS	NEG	POS	NEG
UP72 (Peru)	POS	AMB	AMB	NEG	NEG	NEG	NEG	POS	POS	NEG	NEG	POS	POS	NEG	POS	NEG
UP81 (Peru)	POS	AMB	AMB	NEG	NEG	NEG	NEG	POS	POS	NEG	NEG	POS	POS	NEG	POS	NEG
>"South American/ Mid																NEG
UP1073 (Peru)	POS	AMB	AMB	NEG	NEG	NEG	NEG	POS	POS	NEG	NEG	POS	POS	NEG	POS	NEG

STRAIN / ISOLATE	ADHAESION FACTORS / GENES ENCODING MICROBIAL SURFACE COMPONENTS RECOGNIZING ADHESIVE MATRIX MOLECULES															
	fnbB							map				sasG				sasX / sesI
	fnbB	fnbB (COL)	fnbB (COL+Mu50+MW2)	fnbB (Mu50)	fnbB (MW2)	fnbB (ST15)	fnbB (ST45-2)	map	map (COL)	map (MRSA252)	map (Mu50+MW2)	sasG	sasG (COL+Mu50)	sasG (MW2)	sasG (OtherThan252+122)	
	Fibronectin-binding protein B							Major histocompatibility complex class II analog protein (=Extracellular adherence protein, eap)				Staphylococcus aureus surface protein G				
**>"South American/ Mid**																**NEG**
LIT89 (Lithuania)	POS	AMB	AMB	NEG	NEG	NEG	NEG	POS	POS	NEG	NEG	POS	POS	NEG	POS	NEG
MRSA_PR1 (Malaysia)	POS	AMB	AMB	NEG	NEG	NEG	NEG	POS	POS	NEG	NEG	POS	POS	NEG	POS	NEG
Riyadh-2888905-R	POS	POS	AMB	AMB	NEG	NEG	NEG	POS	POS	NEG	AMB	POS	POS	NEG	POS	NEG
Riyadh-2888915-BC	POS	POS	AMB	NEG	NEG	NEG	NEG	NEG	NEG	NEG	NEG	POS	POS	NEG	POS	NEG
Riyadh-2793706-R	POS	POS	AMB	AMB	NEG	NEG	NEG	POS	POS	NEG	POS	POS	POS	NEG	POS	NEG
**>"South American/ Mid**																**NEG**
Riyadh_SSTI_18_3502925	POS	AMB	AMB	POS	NEG	NEG	NEG	POS	POS	NEG	POS	POS	POS	NEG	POS	NEG
Riyadh-2822825-W	POS	POS	AMB	AMB	NEG	NEG	NEG	POS	POS	NEG	POS	POS	POS	NEG	POS	NEG
**>"South American/ Mid**																**NEG**
UK-EMRSA-9	POS	POS	AMB	AMB	NEG	NEG	NEG	POS	POS	NEG	AMB	POS	POS	NEG	POS	NEG
**>"South American/ Mid**																**NEG**
Lome_HT20020815	POS	POS	AMB	AMB	NEG	NEG	NEG	POS	POS	NEG	NEG	POS	POS	NEG	POS	NEG
**>"South American/ Mid**																**NEG**
NA32 (Denmark)	POS	AMB	AMB	NEG	NEG	NEG	NEG	POS	POS	NEG	NEG	POS	POS	NEG	POS	NEG
H24 (Egypt)	POS	AMB	AMB	NEG	NEG	NEG	NEG	POS	POS	NEG	NEG	POS	POS	NEG	POS	NEG
ATCC BAA-39 (=HUSA304) (Hungary)	POS	AMB	AMB	NEG	NEG	NEG	NEG	POS	POS	NEG	NEG	POS	POS	NEG	POS	NEG
HUSA304 (Hungary)	POS	AMB	AMB	NEG	NEG	NEG	NEG	POS	POS	NEG	NEG	POS	POS	NEG	POS	NEG
HU106 (Hungary)	NEG	NEG	NEG	NEG	NEG	NEG	NEG	POS	POS	NEG	NEG	POS	POS	NEG	POS	NEG
BSAC27 (UK)	POS	AMB	AMB	NEG	NEG	NEG	NEG	POS	POS	NEG	NEG	POS	POS	NEG	POS	NEG
NCTC13131, UK-EMRSA-4 (UK)	POS	AMB	AMB	NEG	NEG	NEG	NEG	POS	POS	NEG	NEG	POS	POS	NEG	POS	NEG
	POS	POS	AMB	AMB	NEG	NEG	NEG	POS	POS	NEG	AMB	POS	POS	NEG	POS	NEG
Algiers_HT20040080	NEG	NEG	NEG	NEG	NEG	NEG	NEG	POS	POS	NEG	NEG	POS	POS	NEG	POS	NEG
Dublin-DSH_AR09_0.0066	POS	POS	AMB	AMB	NEG	NEG	NEG	POS	POS	NEG	NEG	POS	POS	NEG	POS	NEG
Dublin-DSH_AR09_0-0065	POS	POS	AMB	NEG	NEG	NEG	NEG	POS	POS	NEG	NEG	POS	POS	NEG	POS	NEG
Dublin-DSH_Phenotype-III_84	POS	AMB	POS	NEG	NEG	NEG	NEG	POS	POS	NEG	NEG	POS	POS	NEG	POS	NEG
Hong Kong_130	POS	POS	AMB	NEG	NEG	NEG	NEG	POS	POS	NEG	NEG	POS	POS	NEG	POS	NEG
Kuwait_018	POS	POS	AMB	NEG	NEG	NEG	NEG	POS	POS	NEG	NEG	POS	POS	NEG	POS	NEG
Perth_08-17726	POS	POS	AMB	AMB	NEG	NEG	NEG	POS	POS	NEG	NEG	POS	POS	NEG	POS	NEG
Riyadh_Alfaisal/KKKSUH_86_MRS-14-279	POS	POS	AMB	NEG	NEG	NEG	NEG	POS	POS	NEG	NEG	POS	POS	NEG	POS	NEG
Riyadh_Alfaisal-04_Z3861_831588	POS	POS	AMB	AMB	NEG	NEG	NEG	POS	POS	NEG	AMB	POS	POS	NEG	POS	NEG
Riyadh_Alfaisal-30_S15724_1108013	POS	POS	AMB	AMB	NEG	NEG	NEG	POS	POS	NEG	AMB	POS	POS	NEG	POS	NEG
Riyadh_Alfaisal-6_22A_13_B3992_397721	POS	POS	AMB	AMB	NEG	NEG	NEG	POS	POS	NEG	AMB	POS	POS	NEG	POS	NEG
Riyadh_SSTI_52_3615482	POS	POS	AMB	AMB	NEG	NEG	NEG	POS	POS	NEG	POS	POS	POS	NEG	POS	NEG
Riyadh-2817276-2	POS	POS	AMB	AMB	NEG	NEG	NEG	POS	POS	NEG	POS	POS	POS	NEG	POS	NEG
Riyadh-2891670-W	POS	POS	AMB	AMB	NEG	NEG	NEG	POS	POS	NEG	POS	POS	POS	NEG	POS	NEG
Riyadh-3006920-W	POS	POS	AMB	AMB	NEG	NEG	NEG	POS	POS	NEG	AMB	POS	POS	NEG	POS	NEG
Riyadh-R2567782	POS	POS	AMB	AMB	NEG	NEG	NEG	POS	POS	NEG	AMB	POS	POS	NEG	POS	NEG
Russia-18_0252_Krasnoyarsk_SK2	POS	POS	AMB	AMB	NEG	NEG	NEG	POS	POS	NEG	NEG	POS	POS	NEG	POS	NEG
UK-EMRSA-7	POS	POS	AMB	NEG	NEG	NEG	NEG	POS	POS	NEG	NEG	POS	POS	NEG	POS	NEG
**>"South American/ Mid**																**NEG**
Dublin-DSH_AR23_0073	POS	POS	AMB	NEG	NEG	NEG	NEG	POS	POS	NEG	NEG	POS	POS	NEG	POS	NEG
**>Related to "South Ame**																**NEG**
MRSA-OC3 (Russia)	POS	AMB	AMB	NEG	NEG	NEG	NEG	POS	POS	NEG	NEG	POS	POS	NEG	POS	NEG
Russia-12_0176_Krasnoyarsk	POS	POS	AMB	AMB	NEG	NEG	NEG	POS	POS	NEG	NEG	POS	POS	NEG	POS	NEG
Russia-13_0180_Krasnoyarsk	POS	POS	AMB	AMB	NEG	NEG	NEG	POS	POS	NEG	NEG	POS	POS	NEG	POS	NEG
Russia-16_0249_Krasnoyarsk	POS	POS	AMB	AMB	NEG	NEG	NEG	POS	POS	NEG	NEG	POS	POS	NEG	POS	NEG
Russia-17_0250_Krasnoyarsk	POS	POS	AMB	AMB	NEG	NEG	NEG	POS	POS	NEG	NEG	POS	POS	NEG	POS	NEG
**>Related to "South Ame**																**NEG**
URU110 (Uruguay)	POS	AMB	AMB	NEG	NEG	NEG	NEG	POS	POS	NEG	NEG	POS	POS	NEG	POS	NEG
**>Related to "South Ame**																**NEG**
DS_014 (Thailand)	POS	AMB	AMB	NEG	NEG	NEG	NEG	POS	POS	NEG	NEG	POS	POS	NEG	POS	NEG
**>Unassigned Middle Eas**																**NEG**
Frankfurt_Oder_0490031797 (Libya)	POS	AMB	AMB	POS	NEG	NEG	NEG	POS	POS	NEG	AMB	POS	POS	NEG	POS	NEG
Kuwait_192	POS	POS	AMB	AMB	NEG	NEG	NEG	POS	POS	NEG	POS	POS	POS	NEG	POS	NEG
Riyadh-9	POS	POS	AMB	AMB	NEG	NEG	NEG	POS	POS	NEG	AMB	POS	POS	NEG	POS	NEG
Russia-24_0407_Moscow	POS	POS	AMB	AMB	NEG	NEG	NEG	POS	POS	NEG	NEG	POS	POS	NEG	POS	NEG

STRAIN / ISOLATE	ADHAESION FACTORS / GENES ENCODING MICROBIAL SURFACE COMPONENTS RECOGNIZING ADHESIVE MATRIX MOLECULES																	
	sdrC							sdrD					vwb					
	sdrC	sdrC (cons)	sdrC (B1)	sdrC (COL)	sdrC (Mu50)	sdrC (MW2+MRSA252+RF122)	sdrC (OtherThan252+RF122)	sdrD	sdrD (cons)	sdrD (COL+MW2)	sdrD (Mu50)	sdrD (other)	vwb	vwb (cons)	vwb (COL+MW2)	vwb (MRSA252)	vwb (Mu50)	vwb (RF122)
	Ser-Asp rich fibrinogen-/bone sialoprotein-binding protein C							Ser-Asp rich fibrinogen-/bone sialoprotein-binding protein D					van Willebrand factor binding protein					
**>"Eurasian Clade": TUR1**																		
TUR1 (Turkey)	POS	POS	NEG	POS	NEG	NEG	POS	POS	POS	POS	NEG	NEG	POS	POS	POS	NEG	NEG	NEG
TUR9 (Turkey)	POS	POS	NEG	POS	NEG	NEG	POS	POS	POS	POS	NEG	NEG	POS	POS	POS	NEG	NEG	NEG
**>"Eurasian Clade" : T013**																		
T0131 (China)	POS	POS	NEG	POS	NEG	NEG	POS	POS	POS	POS	NEG	NEG	POS	POS	POS	NEG	NEG	NEG
MU4 (Turkey)	POS	POS	NEG	POS	NEG	NEG	POS	POS	POS	POS	NEG	NEG	POS	POS	POS	NEG	NEG	NEG
DEU11 (Turkey)	POS	POS	NEG	POS	NEG	NEG	POS	POS	POS	POS	NEG	NEG	POS	POS	POS	NEG	NEG	NEG
DEU16 (Turkey)	POS	POS	NEG	POS	NEG	NEG	POS	POS	POS	POS	NEG	NEG	POS	POS	POS	NEG	NEG	NEG
Romania_Iasi_MRSA-07_54	POS	POS	NEG	POS	NEG	NEG	POS	POS	POS	POS	NEG	NEG	POS	POS	POS	NEG	NEG	NEG
**>"Eurasian Clade": arsC-**																		
Romania_Iasi_MRSA-05_46	POS	POS	NEG	POS	NEG	NEG	POS	POS	POS	POS	NEG	NEG	POS	POS	POS	NEG	NEG	NEG
Romania_Iasi_MRSA-06_47	POS	POS	NEG	POS	NEG	NEG	POS	POS	POS	POS	NEG	NEG	POS	POS	POS	NEG	NEG	NEG
**>"Eurasian Clade": 16K/**																		
CN79 (China)	POS	POS	NEG	POS	NEG	NEG	POS	POS	POS	POS	NEG	NEG	POS	POS	POS	NEG	NEG	NEG
CUHK_BJ2002 (China)	POS	POS	NEG	POS	NEG	NEG	POS	POS	POS	POS	NEG	NEG	POS	POS	POS	NEG	NEG	NEG
CUHK_BJ2007 (China)	POS	POS	NEG	POS	NEG	NEG	POS	POS	POS	POS	NEG	NEG	POS	POS	POS	NEG	NEG	NEG
3HK (Czech Republic)	POS	POS	NEG	POS	NEG	NEG	POS	POS	POS	POS	NEG	NEG	POS	POS	POS	NEG	NEG	NEG
HU109 (Hungary)	POS	POS	NEG	POS	NEG	NEG	POS	POS	POS	POS	NEG	NEG	POS	POS	POS	NEG	NEG	NEG
HUR18 (Hungary)	POS	POS	NEG	POS	NEG	NEG	POS	POS	POS	POS	NEG	NEG	POS	POS	POS	NEG	NEG	NEG
H482 (Romania)	POS	POS	NEG	POS	NEG	NEG	POS	POS	POS	POS	NEG	NEG	POS	POS	POS	NEG	NEG	NEG
16K (Russia)	POS	POS	NEG	POS	NEG	NEG	POS	POS	POS	POS	NEG	NEG	POS	POS	POS	NEG	NEG	NEG
DEU10 (Turkey)	POS	POS	NEG	POS	NEG	NEG	POS	POS	POS	POS	NEG	NEG	POS	POS	POS	NEG	NEG	NEG
DEU12 (Turkey)	POS	POS	NEG	POS	NEG	NEG	POS	POS	POS	POS	NEG	NEG	POS	POS	POS	NEG	NEG	NEG
DEU14 (Turkey)	POS	POS	NEG	POS	NEG	NEG	POS	POS	POS	POS	NEG	NEG	POS	POS	POS	NEG	NEG	NEG
DEU15 (Turkey)	POS	POS	NEG	POS	NEG	NEG	POS	POS	POS	POS	NEG	NEG	POS	POS	POS	NEG	NEG	NEG
DEU17 (Turkey)	POS	POS	NEG	POS	NEG	NEG	POS	POS	POS	POS	NEG	NEG	POS	POS	POS	NEG	NEG	NEG
DEU19 (Turkey)	POS	POS	NEG	POS	NEG	NEG	POS	POS	POS	POS	NEG	NEG	POS	POS	POS	NEG	NEG	NEG
DEU2 (Turkey)	POS	POS	NEG	POS	NEG	NEG	POS	POS	POS	POS	NEG	NEG	POS	POS	POS	NEG	NEG	NEG
DEU20 (Turkey)	POS	POS	NEG	POS	NEG	NEG	POS	POS	POS	POS	NEG	NEG	POS	POS	POS	NEG	NEG	NEG
DEU23 (Turkey)	POS	POS	NEG	POS	NEG	NEG	POS	POS	POS	POS	NEG	NEG	POS	POS	POS	NEG	NEG	NEG
DEU3 (Turkey)	POS	POS	NEG	POS	NEG	NEG	POS	POS	POS	POS	NEG	NEG	POS	POS	POS	NEG	NEG	NEG
DEU5 (Turkey)	POS	POS	NEG	POS	NEG	NEG	POS	POS	POS	POS	NEG	NEG	POS	POS	POS	NEG	NEG	NEG
DEU6 (Turkey)	POS	POS	NEG	POS	NEG	NEG	POS	POS	POS	POS	NEG	NEG	POS	POS	POS	NEG	NEG	NEG
DEU8 (Turkey)	POS	POS	NEG	POS	NEG	NEG	POS	POS	POS	POS	NEG	NEG	POS	POS	POS	NEG	NEG	NEG
HU11 (Turkey)	POS	POS	NEG	POS	NEG	NEG	POS	POS	POS	POS	NEG	NEG	POS	POS	POS	NEG	NEG	NEG
HU13 (Turkey)	POS	POS	NEG	POS	NEG	NEG	POS	POS	POS	POS	NEG	NEG	POS	POS	POS	NEG	NEG	NEG
HU14 (Turkey)	POS	POS	NEG	POS	NEG	NEG	POS	POS	POS	POS	NEG	NEG	POS	POS	POS	NEG	NEG	NEG
HU15 (Turkey)	POS	POS	NEG	POS	NEG	NEG	POS	POS	POS	POS	NEG	NEG	POS	POS	POS	NEG	NEG	NEG
HU16 (Turkey)	POS	POS	NEG	POS	NEG	NEG	POS	POS	POS	POS	NEG	NEG	POS	POS	POS	NEG	NEG	NEG
HU17 (Turkey)	POS	POS	NEG	POS	NEG	NEG	POS	POS	POS	POS	NEG	NEG	POS	POS	POS	NEG	NEG	NEG
HU21 (Turkey)	POS	POS	NEG	POS	NEG	NEG	POS	POS	POS	POS	NEG	NEG	POS	POS	POS	NEG	NEG	NEG
HU23 (Turkey)	POS	POS	NEG	POS	NEG	NEG	POS	POS	POS	POS	NEG	NEG	POS	POS	POS	NEG	NEG	NEG
HU26 (Turkey)	POS	POS	NEG	POS	NEG	NEG	POS	POS	POS	POS	NEG	NEG	POS	POS	POS	NEG	NEG	NEG
HU4 (Turkey)	POS	POS	NEG	POS	NEG	NEG	POS	POS	POS	POS	NEG	NEG	POS	POS	POS	NEG	NEG	NEG
HU5 (Turkey)	POS	POS	NEG	POS	NEG	NEG	POS	POS	POS	POS	NEG	NEG	POS	POS	POS	NEG	NEG	NEG
HU6 (Turkey)	POS	POS	NEG	POS	NEG	NEG	POS	POS	POS	POS	NEG	NEG	POS	POS	POS	NEG	NEG	NEG
HU7 (Turkey)	POS	POS	NEG	POS	NEG	NEG	POS	POS	POS	POS	NEG	NEG	POS	POS	POS	NEG	NEG	NEG
HU8 (Turkey)	POS	POS	NEG	POS	NEG	NEG	POS	POS	POS	POS	NEG	NEG	POS	POS	POS	NEG	NEG	NEG
HU9 (Turkey)	POS	POS	NEG	POS	NEG	NEG	POS	POS	POS	POS	NEG	NEG	POS	POS	POS	NEG	NEG	NEG
IU1 (Turkey)	POS	POS	NEG	POS	NEG	NEG	POS	POS	POS	POS	NEG	NEG	POS	POS	POS	NEG	NEG	NEG
IU10 (Turkey)	POS	POS	NEG	POS	NEG	NEG	POS	POS	POS	POS	NEG	NEG	POS	POS	POS	NEG	NEG	NEG
IU11 (Turkey)	POS	POS	NEG	POS	NEG	NEG	POS	POS	POS	POS	NEG	NEG	POS	POS	POS	NEG	NEG	NEG
IU12 (Turkey)	POS	POS	NEG	POS	NEG	NEG	POS	POS	POS	POS	NEG	NEG	POS	POS	POS	NEG	NEG	NEG
IU13 (Turkey)	POS	POS	NEG	POS	NEG	NEG	POS	POS	POS	POS	NEG	NEG	POS	POS	POS	NEG	NEG	NEG
IU15 (Turkey)	POS	POS	NEG	POS	NEG	NEG	POS	POS	POS	POS	NEG	NEG	POS	POS	POS	NEG	NEG	NEG
IU18 (Turkey)	POS	POS	NEG	POS	NEG	NEG	POS	POS	POS	POS	NEG	NEG	POS	POS	POS	NEG	NEG	NEG
IU19 (Turkey)	POS	POS	NEG	POS	NEG	NEG	POS	POS	POS	POS	NEG	NEG	POS	POS	POS	NEG	NEG	NEG
IU2 (Turkey)	POS	POS	NEG	POS	NEG	NEG	POS	POS	POS	POS	NEG	NEG	POS	POS	POS	NEG	NEG	NEG
IU4 (Turkey)	POS	POS	NEG	POS	NEG	NEG	POS	POS	POS	POS	NEG	NEG	POS	POS	POS	NEG	NEG	NEG
IU6 (Turkey)	POS	POS	NEG	POS	NEG	NEG	POS	POS	POS	POS	NEG	NEG	POS	POS	POS	NEG	NEG	NEG
IU7 (Turkey)	POS	POS	NEG	POS	NEG	NEG	POS	POS	POS	POS	NEG	NEG	POS	POS	POS	NEG	NEG	NEG
IU9 (Turkey)	POS	POS	NEG	POS	NEG	NEG	POS	POS	POS	POS	NEG	NEG	POS	POS	POS	NEG	NEG	NEG
MU1 (Turkey)	POS	POS	NEG	POS	NEG	NEG	POS	POS	POS	POS	NEG	NEG	POS	POS	POS	NEG	NEG	NEG
MU10 (Turkey)	POS	POS	NEG	POS	NEG	NEG	POS	POS	POS	POS	NEG	NEG	POS	POS	POS	NEG	NEG	NEG
MU20 (Turkey)	POS	POS	NEG	POS	NEG	NEG	POS	POS	POS	POS	NEG	NEG	POS	POS	POS	NEG	NEG	NEG
Mu3 (Turkey)	POS	POS	NEG	POS	NEG	NEG	POS	POS	POS	POS	NEG	NEG	POS	POS	POS	NEG	NEG	NEG
MU5 (Turkey)	POS	POS	NEG	POS	NEG	NEG	POS	POS	POS	POS	NEG	NEG	POS	POS	POS	NEG	NEG	NEG
MU6 (Turkey)	POS	POS	NEG	POS	NEG	NEG	POS	POS	POS	POS	NEG	NEG	POS	POS	POS	NEG	NEG	NEG
MU7 (Turkey)	POS	POS	NEG	POS	NEG	NEG	POS	POS	POS	POS	NEG	NEG	POS	POS	POS	NEG	NEG	NEG
TUR27 (Turkey)	POS	POS	NEG	POS	NEG	NEG	POS	POS	POS	POS	NEG	NEG	POS	POS	POS	NEG	NEG	NEG
NCTR 32S	POS	POS	NEG	POS	NEG	NEG	POS	POS	POS	POS	NEG	NEG	POS	POS	POS	NEG	NEG	NEG
SA02_A4 = Russia-06_0085_Moscow	POS	POS	NEG	POS	NEG	NEG	POS	POS	POS	POS	NEG	NEG	POS	POS	POS	NEG	NEG	NEG
	POS	POS	NEG	POS	NEG	NEG	POS	POS	POS	POS	NEG	NEG	POS	POS	POS	NEG	NEG	NEG
Dresden_08V35987 (Turkey)	POS	POS	NEG	POS	NEG	NEG	POS	POS	POS	POS	NEG	NEG	POS	POS	POS	NEG	NEG	NEG
Dresden_17ANR577152	POS	POS	NEG	POS	NEG	NEG	POS	POS	POS	POS	NEG	NEG	POS	POS	POS	NEG	NEG	NEG
Dresden_17ANR580374 (Makedonia)	POS	POS	NEG	POS	NEG	NEG	POS	POS	POS	POS	NEG	NEG	POS	POS	POS	NEG	NEG	NEG
Hong Kong_69-II	AMB	POS	NEG	POS	NEG	NEG	POS	AMB	POS	POS	NEG	NEG	AMB	POS	POS	NEG	NEG	NEG
Hong Kong_93	AMB	POS	NEG	POS	NEG	AMB	POS	AMB	POS	POS	NEG	NEG	AMB	POS	POS	NEG	NEG	NEG
Rawalpindi_Kidney Center_03_SK1	POS	POS	NEG	POS	NEG	NEG	POS	POS	POS	POS	NEG	NEG	POS	POS	POS	NEG	NEG	NEG
Rawalpindi_Kidney Center_08	POS	POS	NEG	POS	NEG	NEG	POS	POS	POS	POS	NEG	NEG	POS	POS	POS	NEG	NEG	NEG
Rawalpindi_Kidney Center_10	POS	POS	NEG	POS	NEG	NEG	POS	POS	POS	POS	NEG	NEG	POS	POS	POS	NEG	NEG	NEG
Rawalpindi_Kidney Center_19	POS	POS	NEG	POS	NEG	AMB	POS	POS	POS	POS	NEG	NEG	POS	POS	POS	NEG	NEG	NEG
Rawalpindi_Kidney Center_50_SK1	POS	POS	NEG	POS	NEG	NEG	POS	POS	POS	POS	NEG	NEG	POS	POS	POS	NEG	NEG	NEG
Romania_Iasi_BC-49_430	POS	POS	NEG	POS	NEG	NEG	POS	POS	POS	POS	NEG	NEG	POS	POS	POS	NEG	NEG	NEG
Romania_Iasi_MRSA_284	POS	POS	NEG	POS	NEG	NEG	POS	POS	POS	POS	NEG	NEG	POS	POS	POS	NEG	NEG	NEG
Romania_Iasi_MRSA-32_318	POS	POS	NEG	POS	NEG	NEG	POS	POS	POS	POS	NEG	NEG	POS	POS	POS	NEG	NEG	NEG
Romania_Iasi_MRSA-54_430	AMB	POS	AMB	POS	NEG	AMB	POS	POS	POS	POS	NEG	NEG	POS	POS	POS	NEG	NEG	NEG
Romania_Iasi_SSTI-01_2	AMB	POS	NEG	POS	NEG	NEG	POS	POS	POS	POS	NEG	NEG	POS	POS	POS	NEG	NEG	NEG
Romania_Iasi_SSTI-10_101	AMB	POS	NEG	POS	NEG	NEG	POS	POS	POS	POS	NEG	NEG	POS	POS	POS	NEG	NEG	NEG
Romania_Iasi_SSTI-11_106	POS	POS	NEG	POS	NEG	NEG	POS	POS	POS	POS	NEG	NEG	POS	POS	POS	NEG	NEG	NEG
Russia-01_0001_SaintPetersburg	POS	POS	NEG	POS	NEG	NEG	POS	POS	POS	POS	NEG	NEG	POS	POS	POS	NEG	NEG	NEG
Russia-02_0004_SaintPetersburg	POS	POS	NEG	POS	NEG	NEG	POS	POS	POS	POS	NEG	NEG	POS	POS	POS	NEG	NEG	NEG
Russia-03_0057_SaintPetersburg	POS	POS	NEG	POS	NEG	NEG	POS	POS	POS	POS	NEG	NEG	POS	POS	POS	NEG	NEG	NEG
Russia-04_0076_Moscow	POS	POS	NEG	POS	NEG	NEG	POS	POS	POS	POS	NEG	NEG	POS	POS	POS	NEG	NEG	NEG
Russia-05_0078_Moscow	POS	POS	NEG	POS	NEG	NEG	POS	POS	POS	POS	NEG	NEG	POS	POS	POS	NEG	NEG	NEG
Russia-08_0135_Moscow	POS	POS	NEG	POS	NEG	NEG	POS	POS	POS	POS	NEG	NEG	POS	POS	POS	NEG	NEG	NEG
Russia-09_0150_Kurgan	POS	POS	NEG	POS	NEG	NEG	POS	POS	POS	POS	NEG	NEG	POS	POS	POS	NEG	NEG	NEG
Russia-10_0162_Kurgan	POS	POS	NEG	POS	NEG	NEG	POS	POS	POS	POS	NEG	NEG	POS	POS	POS	NEG	NEG	NEG
Russia-14_0184_Moscow	POS	POS	NEG	POS	NEG	NEG	POS	POS	POS	POS	NEG	NEG	POS	POS	POS	NEG	NEG	NEG
Russia-15_0232_SaintPetersburg	POS	POS	NEG	POS	NEG	NEG	POS	POS	POS	POS	NEG	NEG	POS	POS	POS	NEG	NEG	NEG
Russia-22_0391_Chelyabinsk	POS	POS	NEG	POS	NEG	NEG	POS	POS	POS	POS	NEG	NEG	POS	POS	POS	NEG	NEG	NEG
Russia-23_0392_Chelyabinsk	POS	POS	NEG	POS	NEG	NEG	POS	POS	POS	POS	NEG	NEG	POS	POS	POS	NEG	NEG	NEG
**>"Eurasian Clade" : IU17**																		
IU17 (Turkey)	POS	POS	NEG	POS	NEG	NEG	POS	POS	POS	POS	NEG	NEG	POS	POS	POS	NEG	NEG	NEG
IU20 (Turkey)	POS	POS	NEG	POS	NEG	NEG	POS	POS	POS	POS	NEG	NEG	POS	POS	POS	NEG	NEG	NEG

STRAIN / ISOLATE	ADHAESION FACTORS / GENES ENCODING MICROBIAL SURFACE COMPONENTS RECOGNIZING ADHESIVE MATRIX MOLECULES																	
	sdrC							sdrD					vwb					
	sdrC	sdrC (cons)	sdrC (B1)	sdrC (COL)	sdrC (Mu50)	sdrC (MW2+MRSA252+RF122)	sdrC (OtherThan252+RF122)	sdrD	sdrD (cons)	sdrD (COL+MW2)	sdrD (Mu50)	sdrD (other)	vwb	vwb (cons)	vwb (COL+MW2)	vwb (MRSA252)	vwb (Mu50)	vwb (RF122)
	Ser-Asp rich fibrinogen-/bone sialoprotein-binding protein C							Ser-Asp rich fibrinogen-/bone sialoprotein-binding protein D					van Willebrand factor binding protein					
>"Eurasian Clade": Spora																		
Dresden_16ANR581769	POS	POS	NEG	POS	NEG	NEG	POS	POS	POS	POS	NEG	NEG	POS	POS	POS	NEG	NEG	NEG
Dresden_17ANR578769	POS	POS	NEG	POS	NEG	NEG	POS	POS	POS	POS	NEG	NEG	POS	POS	POS	NEG	NEG	NEG
>"Eurasian Clade": Kuwa																		
Kuwait_306	POS	POS	NEG	POS	NEG	AMB	POS	POS	POS	POS	NEG	NEG	POS	POS	POS	NEG	NEG	NEG
Kuwait_301	POS	POS	NEG	POS	NEG	NEG	POS	POS	POS	POS	NEG	NEG	POS	POS	POS	NEG	NEG	NEG
>"European Clade": "Gre																		
GRE18 (Greece)	POS	POS	NEG	POS	NEG	NEG	POS	POS	POS	POS	NEG	NEG	POS	POS	POS	NEG	NEG	NEG
GRE317 (Greece)	POS	POS	NEG	POS	NEG	NEG	POS	POS	POS	POS	NEG	NEG	POS	POS	POS	NEG	NEG	NEG
GRE4 (Greece)	POS	POS	NEG	POS	NEG	NEG	POS	POS	POS	POS	NEG	NEG	POS	POS	POS	NEG	NEG	NEG
R35 (USA)	POS	POS	NEG	POS	NEG	NEG	POS	POS	POS	POS	NEG	NEG	POS	POS	POS	NEG	NEG	NEG
Casablanca HT20060548	POS	POS	NEG	POS	NEG	NEG	POS	POS	POS	POS	NEG	NEG	POS	POS	POS	NEG	NEG	NEG
Casablanca HT20060550	POS	POS	NEG	POS	NEG	NEG	POS	POS	POS	POS	NEG	NEG	POS	POS	POS	NEG	NEG	NEG
Dresden_01V34572 (Greece)	POS	POS	NEG	POS	NEG	NEG	POS	POS	POS	AMB	NEG	NEG	POS	POS	POS	NEG	NEG	NEG
Dresden_01V36469	POS	POS	NEG	POS	NEG	NEG	POS	POS	POS	POS	NEG	NEG	POS	POS	POS	NEG	NEG	NEG
Dresden 01V39123	POS	POS	AMB	POS	AMB	NEG	POS	POS	POS	POS	NEG	NEG	POS	POS	POS	NEG	NEG	NEG
Dresden 01V39143	POS	POS	NEG	POS	NEG	NEG	POS	POS	POS	POS	NEG	NEG	POS	POS	POS	NEG	NEG	NEG
Greece 1 3680 Harmony	POS	AMB	NEG	POS	NEG	NEG	POS	POS	POS	NEG	NEG	NEG	POS	POS	POS	NEG	NEG	NEG
>"European Clade": UK-(																		
ANS46 (Australia)	POS	POS	NEG	POS	NEG	NEG	POS	POS	POS	POS	NEG	NEG	POS	POS	POS	NEG	NEG	NEG
LHH1 (USA)	POS	POS	NEG	POS	NEG	NEG	POS	POS	POS	POS	NEG	NEG	POS	POS	POS	NEG	NEG	NEG
Dublin-DSH_AR01_0-1085	POS	POS	NEG	POS	NEG	NEG	POS	POS	POS	POS	NEG	NEG	POS	POS	POS	NEG	NEG	NEG
Dublin-DSH_AR01_0-1118	POS	POS	NEG	POS	NEG	NEG	POS	POS	POS	POS	NEG	NEG	POS	POS	POS	NEG	NEG	NEG
Dublin-DSH_AR15_0098	POS	POS	NEG	POS	NEG	NEG	POS	POS	POS	POS	NEG	NEG	POS	POS	POS	NEG	NEG	NEG
Dublin-DSH_AR15_0104	POS	POS	NEG	POS	NEG	NEG	POS	POS	POS	POS	NEG	NEG	POS	POS	POS	NEG	NEG	NEG
UK_NCTC11939_UK-1_Harmony	POS	POS	NEG	POS	NEG	NEG	POS	POS	POS	POS	NEG	NEG	POS	POS	POS	NEG	NEG	NEG
>"European Clade": BK2																		
BK2421 (USA)	POS	POS	NEG	POS	NEG	NEG	POS	POS	POS	POS	NEG	NEG	POS	POS	POS	NEG	NEG	NEG
>"Aussi/NZ Clade", JKD6																		
JKD6009 (Australia)	POS	POS	NEG	POS	NEG	NEG	POS	POS	POS	POS	NEG	NEG	POS	POS	POS	NEG	NEG	NEG
JKD6008 (New Zealand)	POS	POS	NEG	POS	NEG	NEG	POS	POS	POS	POS	NEG	NEG	POS	POS	POS	NEG	NEG	NEG
Perth_2015-S20209	POS	POS	NEG	POS	NEG	NEG	POS	POS	POS	POS	NEG	NEG	POS	POS	POS	NEG	NEG	NEG
Perth_2015-S20305	POS	POS	NEG	POS	NEG	NEG	POS	POS	POS	POS	NEG	NEG	POS	POS	POS	NEG	NEG	NEG
Perth_2015-S20603	POS	POS	NEG	POS	NEG	NEG	POS	POS	POS	POS	NEG	NEG	POS	POS	POS	NEG	NEG	NEG
Perth_2015-S20607	POS	POS	NEG	POS	NEG	NEG	POS	POS	POS	POS	NEG	NEG	POS	POS	POS	NEG	NEG	NEG
Perth_2015-S20608	POS	POS	NEG	POS	NEG	NEG	POS	POS	POS	POS	NEG	NEG	POS	POS	POS	NEG	NEG	NEG
Perth_2015-S20610	POS	POS	NEG	POS	NEG	NEG	POS	POS	POS	POS	NEG	NEG	POS	POS	POS	NEG	NEG	NEG
Perth_2015-S20613	POS	POS	NEG	POS	NEG	NEG	POS	POS	POS	POS	NEG	NEG	POS	POS	POS	NEG	NEG	NEG
Perth_2015-S20617	POS	POS	NEG	POS	NEG	NEG	POS	POS	POS	POS	NEG	NEG	POS	POS	POS	NEG	NEG	NEG
Perth_2015-S20622	POS	POS	NEG	POS	NEG	NEG	POS	POS	POS	POS	NEG	NEG	POS	POS	POS	NEG	NEG	NEG
Perth_2015-S23619	POS	POS	NEG	POS	NEG	NEG	POS	POS	POS	POS	NEG	NEG	POS	POS	POS	NEG	NEG	NEG
Perth_2015-S41210	POS	POS	NEG	POS	NEG	NEG	POS	POS	POS	POS	NEG	NEG	POS	POS	POS	NEG	NEG	NEG
Perth_2015-S51001	POS	POS	NEG	POS	NEG	NEG	POS	POS	POS	POS	NEG	NEG	POS	POS	POS	NEG	NEG	NEG
Perth_2015-S51003	POS	POS	NEG	POS	NEG	NEG	POS	POS	POS	POS	NEG	NEG	POS	POS	POS	NEG	NEG	NEG
Perth_2015-S51006	POS	POS	NEG	POS	NEG	NEG	POS	POS	POS	POS	NEG	NEG	POS	POS	POS	NEG	NEG	NEG
Perth_2015-S51007	POS	POS	NEG	POS	NEG	NEG	POS	POS	POS	POS	NEG	NEG	POS	POS	POS	NEG	NEG	NEG
Perth_2015-S51008	POS	POS	NEG	POS	NEG	NEG	POS	POS	POS	POS	NEG	NEG	POS	POS	POS	NEG	NEG	NEG
Perth_2015-S51014	POS	POS	NEG	POS	NEG	NEG	POS	POS	POS	POS	NEG	NEG	POS	POS	POS	NEG	NEG	NEG
Perth_2015-S51408	POS	POS	NEG	POS	NEG	NEG	POS	POS	POS	POS	NEG	NEG	POS	POS	POS	NEG	NEG	NEG
Perth_2016-S20614	POS	POS	NEG	POS	NEG	NEG	POS	POS	POS	POS	NEG	NEG	POS	POS	POS	NEG	NEG	NEG
Perth_2016-S23614	POS	POS	NEG	POS	NEG	NEG	POS	POS	POS	POS	NEG	NEG	POS	POS	POS	NEG	NEG	NEG
Perth_2016-S42009	POS	POS	NEG	POS	NEG	NEG	POS	POS	POS	POS	NEG	NEG	NEG	NEG	NEG	NEG	NEG	NEG
Perth_2016-S51001	POS	POS	NEG	POS	NEG	NEG	POS	POS	POS	POS	NEG	NEG	POS	POS	POS	NEG	NEG	NEG
Perth_2016-S51403	POS	POS	NEG	POS	NEG	NEG	POS	POS	POS	POS	NEG	NEG	POS	POS	POS	NEG	NEG	NEG
Perth_07 RPA 20	POS	POS	NEG	POS	NEG	NEG	POS	POS	POS	POS	NEG	NEG	POS	POS	POS	NEG	NEG	NEG
Perth_08-19088	POS	POS	NEG	POS	NEG	NEG	POS	POS	POS	POS	NEG	NEG	POS	POS	POS	NEG	NEG	NEG
Perth_01-15351	POS	POS	NEG	POS	NEG	NEG	POS	POS	POS	POS	NEG	NEG	POS	POS	POS	NEG	NEG	NEG
Perth_01-15357	POS	POS	NEG	POS	NEG	NEG	POS	POS	POS	POS	NEG	NEG	POS	POS	POS	NEG	NEG	NEG
Perth_01-15419	POS	POS	NEG	POS	NEG	NEG	POS	POS	POS	POS	NEG	NEG	POS	POS	POS	NEG	NEG	NEG
Perth_01-16309	POS	POS	NEG	POS	NEG	NEG	POS	POS	POS	POS	NEG	NEG	POS	POS	POS	NEG	NEG	NEG
Perth_03 SNP 95	POS	POS	NEG	POS	NEG	AMB	POS	POS	POS	POS	NEG	NEG	POS	POS	POS	NEG	NEG	NEG
Perth_2003_AH_47	POS	POS	NEG	POS	NEG	NEG	POS	POS	POS	POS	NEG	NEG	POS	POS	POS	NEG	NEG	NEG
>"Australian/NZ Clade":																		
Perth_2015-S20623	POS	POS	NEG	POS	NEG	NEG	POS	POS	POS	POS	NEG	NEG	POS	POS	POS	NEG	NEG	NEG
Perth_2015-S20826	POS	POS	NEG	POS	NEG	NEG	POS	POS	POS	POS	NEG	NEG	POS	POS	POS	NEG	NEG	NEG
Perth_05 NH 56	POS	POS	NEG	POS	NEG	NEG	POS	POS	POS	POS	NEG	NEG	POS	POS	POS	NEG	NEG	NEG
>CC5-MRSA-III LA MRSA																		
Delbrück 38 isolate from domestic turkey	POS	POS	NEG	NEG	POS	NEG	POS	POS	POS	NEG	POS	NEG	POS	POS	NEG	NEG	POS	NEG
> Staph. pseudintermed																		
KM1381	NEG	NEG	NEG	NEG	NEG	NEG	NEG	NEG	NEG	NEG	NEG	NEG	NEG	NEG	NEG	NEG	NEG	NEG
>"South-East Asian Clad																		
M92 (Canada)	POS	POS	NEG	POS	NEG	NEG	POS	POS	POS	POS	NEG	NEG	POS	POS	POS	NEG	NEG	NEG
CHI59 (China)	POS	POS	NEG	POS	NEG	NEG	POS	POS	POS	POS	NEG	NEG	POS	POS	POS	NEG	NEG	NEG
CHI61 (China)	POS	POS	NEG	POS	NEG	NEG	POS	POS	POS	POS	NEG	NEG	POS	POS	POS	NEG	NEG	NEG
CUHK_HK1997 (China)	POS	POS	NEG	POS	NEG	NEG	POS	POS	POS	POS	NEG	NEG	POS	POS	POS	NEG	NEG	NEG
M996 (China)	POS	POS	NEG	POS	NEG	NEG	POS	POS	POS	POS	NEG	NEG	POS	POS	POS	NEG	NEG	NEG
H211 (Denmark)	POS	POS	NEG	POS	NEG	NEG	POS	POS	POS	POS	NEG	NEG	POS	POS	POS	NEG	NEG	NEG
H216 (Denmark)	POS	POS	NEG	POS	NEG	NEG	POS	POS	POS	POS	NEG	NEG	POS	POS	POS	NEG	NEG	NEG
D71 (Germany)	POS	POS	NEG	POS	NEG	NEG	POS	POS	POS	POS	NEG	NEG	POS	POS	POS	NEG	NEG	NEG
NMR05 (India)	POS	POS	NEG	POS	NEG	NEG	POS	NEG	NEG	NEG	NEG	NEG	POS	POS	POS	NEG	NEG	NEG
MAL11 (Malaysia)	POS	POS	NEG	POS	NEG	NEG	POS	POS	POS	POS	NEG	NEG	POS	POS	POS	NEG	NEG	NEG
MAL9 (Malaysia)	POS	POS	NEG	POS	NEG	NEG	POS	POS	POS	POS	NEG	NEG	POS	POS	POS	NEG	NEG	NEG
Na21 (Sri Lanka)	POS	POS	NEG	POS	NEG	NEG	POS	NEG	NEG	NEG	NEG	NEG	POS	POS	POS	NEG	NEG	NEG
M592 (Syria)	POS	POS	NEG	POS	NEG	NEG	POS	POS	POS	POS	NEG	NEG	POS	POS	POS	NEG	NEG	NEG
H202 (Thailand)	POS	POS	NEG	POS	NEG	NEG	POS	POS	POS	POS	NEG	NEG	POS	POS	POS	NEG	NEG	NEG
S102 (Thailand)	POS	POS	NEG	POS	NEG	NEG	POS	POS	POS	POS	NEG	NEG	POS	POS	POS	NEG	NEG	NEG
S130 (Thailand)	POS	POS	NEG	POS	NEG	NEG	POS	POS	POS	POS	NEG	NEG	POS	POS	POS	NEG	NEG	NEG
S40 (Thailand)	POS	POS	NEG	POS	NEG	NEG	POS	POS	POS	POS	NEG	NEG	POS	POS	POS	NEG	NEG	NEG
S71 (Thailand)	POS	POS	NEG	POS	NEG	NEG	POS	POS	POS	POS	NEG	NEG	POS	POS	POS	NEG	NEG	NEG
S87 (Thailand)	POS	POS	NEG	POS	NEG	NEG	POS	POS	POS	POS	NEG	NEG	POS	POS	POS	NEG	NEG	NEG
S93 (Thailand)	POS	POS	NEG	POS	NEG	NEG	POS	POS	POS	POS	NEG	NEG	POS	POS	POS	NEG	NEG	NEG
US_002 (Thailand)	POS	POS	NEG	POS	NEG	NEG	POS	POS	POS	POS	NEG	NEG	POS	POS	POS	NEG	NEG	NEG
US_017 (Thailand)	POS	POS	NEG	POS	NEG	NEG	POS	POS	POS	POS	NEG	NEG	POS	POS	POS	NEG	NEG	NEG
WAMC6102 (Thailand)	POS	POS	NEG	POS	NEG	NEG	POS	POS	POS	POS	NEG	NEG	POS	POS	POS	NEG	NEG	NEG
NCTC_13626_TW20 (UK)	POS	POS	NEG	POS	NEG	NEG	POS	POS	POS	POS	NEG	NEG	POS	POS	POS	NEG	NEG	NEG
BSAC3021 (UK)	POS	POS	NEG	POS	NEG	NEG	POS	POS	POS	POS	NEG	NEG	POS	POS	POS	NEG	NEG	NEG
BSAC3046 (UK)	POS	POS	NEG	POS	NEG	NEG	POS	POS	POS	POS	NEG	NEG	POS	POS	POS	NEG	NEG	NEG
1193_SAUR (USA)	POS	POS	NEG	POS	NEG	NEG	POS	POS	POS	POS	NEG	NEG	POS	POS	POS	NEG	NEG	NEG
1194_SAUR (USA)	POS	POS	NEG	POS	NEG	NEG	POS	POS	POS	POS	NEG	NEG	POS	POS	POS	NEG	NEG	NEG

STRAIN / ISOLATE	ADHESION FACTORS / GENES ENCODING MICROBIAL SURFACE COMPONENTS RECOGNIZING ADHESIVE MATRIX MOLECULES																	
	sdrC							sdrD					vwb					
	sdrC	sdrC (cons)	sdrC (B1)	sdrC (COL)	sdrC (Mu50)	sdrC (MW2+MRSA252+RF122)	sdrC (OtherThan252+RF122)	sdrD	sdrD (cons)	sdrD (COL+MW2)	sdrD (Mu50)	sdrD (other)	vwb	vwb (cons)	vwb (COL+MW2)	vwb (MRSA252)	vwb (Mu50)	vwb (RF122)
	Ser-Asp rich fibrinogen-/bone sialoprotein-binding protein C							Ser-Asp rich fibrinogen-/bone sialoprotein-binding protein D					van Willebrand factor binding protein					
>"South-East Asian Clade": TW20 (Irish AR44), c																		
1195_SAUR (USA)	POS	POS	NEG	POS	NEG	NEG	POS	POS	POS	POS	NEG	NEG	POS	POS	POS	NEG	NEG	NEG
FVRH6002 (USA)	POS	POS	NEG	POS	NEG	NEG	POS	POS	POS	POS	NEG	NEG	POS	POS	POS	NEG	NEG	NEG
KINW6048 (USA)	POS	POS	NEG	POS	NEG	NEG	POS	POS	POS	POS	NEG	NEG	POS	POS	POS	NEG	NEG	NEG
LAMC0011 (USA)	POS	POS	NEG	POS	NEG	NEG	POS	POS	POS	POS	NEG	NEG	POS	POS	POS	NEG	NEG	NEG
SJOS6053 (USA)	POS	POS	NEG	POS	NEG	NEG	POS	POS	POS	POS	NEG	NEG	POS	POS	POS	NEG	NEG	NEG
SJOS6072 (USA)	POS	POS	NEG	POS	NEG	NEG	POS	POS	POS	POS	NEG	NEG	POS	POS	POS	NEG	NEG	NEG
UCIM6042 (USA)	POS	POS	NEG	POS	NEG	NEG	POS	POS	POS	POS	NEG	NEG	POS	POS	POS	NEG	NEG	NEG
Perth_2015-S31902	POS	POS	NEG	NEG	NEG	NEG	POS	POS	POS	POS	NEG	NEG	POS	POS	POS	NEG	NEG	NEG
Perth_2015-S31904	POS	POS	NEG	POS	NEG	NEG	POS	POS	POS	POS	NEG	NEG	POS	POS	POS	NEG	NEG	NEG
Perth_2015-S31913	POS	POS	NEG	POS	NEG	NEG	POS	POS	POS	POS	NEG	NEG	POS	POS	POS	NEG	NEG	NEG
Perth_2015-S31918	POS	POS	NEG	POS	NEG	NEG	POS	POS	POS	POS	NEG	NEG	POS	POS	POS	NEG	NEG	NEG
Dublin-DSH_AR44_E1183	AMB	POS	NEG	POS	NEG	NEG	POS	POS	POS	POS	NEG	NEG	POS	POS	POS	NEG	NEG	NEG
Dublin-DSH_Unfamiliar-3_E1520	AMB	POS	NEG	POS	NEG	NEG	POS	POS	POS	POS	NEG	NEG	POS	POS	POS	NEG	NEG	NEG
Hong Kong_042	POS	POS	NEG	POS	NEG	NEG	POS	POS	POS	POS	NEG	NEG	POS	POS	POS	NEG	NEG	NEG
Hong Kong_101	POS	POS	NEG	POS	NEG	NEG	POS	POS	POS	POS	NEG	NEG	POS	POS	POS	NEG	NEG	NEG
Hong Kong_111	AMB	POS	AMB	POS	AMB	AMB	POS	AMB	POS	POS	NEG	NEG	AMB	POS	POS	NEG	NEG	NEG
Hong Kong_113	POS	POS	NEG	POS	NEG	NEG	POS	POS	POS	POS	NEG	NEG	POS	POS	POS	NEG	NEG	NEG
Hong Kong_115	POS	POS	NEG	POS	NEG	NEG	POS	POS	POS	POS	NEG	NEG	POS	POS	POS	NEG	NEG	NEG
Hong Kong_118	POS	POS	NEG	POS	NEG	NEG	POS	POS	POS	POS	NEG	NEG	POS	POS	POS	NEG	NEG	NEG
Hong Kong_125	POS	POS	NEG	POS	NEG	NEG	POS	POS	POS	POS	NEG	NEG	POS	POS	POS	NEG	NEG	NEG
Hong Kong_129	POS	POS	NEG	POS	NEG	NEG	POS	POS	POS	POS	NEG	NEG	POS	POS	POS	NEG	NEG	NEG
Hong Kong_133	POS	POS	NEG	POS	NEG	NEG	POS	POS	POS	POS	NEG	NEG	POS	POS	POS	NEG	NEG	NEG
Kuwait_009	POS	POS	NEG	POS	NEG	NEG	POS	POS	POS	POS	NEG	NEG	POS	POS	POS	NEG	NEG	NEG
Kuwait_103	POS	POS	NEG	POS	NEG	NEG	POS	POS	POS	POS	NEG	NEG	POS	POS	POS	NEG	NEG	NEG
Kuwait_107	POS	POS	NEG	POS	NEG	NEG	POS	POS	POS	POS	NEG	NEG	POS	POS	POS	NEG	NEG	NEG
Perth_03 AH 88	POS	POS	NEG	POS	NEG	NEG	POS	POS	POS	POS	NEG	NEG	POS	POS	POS	NEG	NEG	NEG
Perth_08-16905	POS	POS	NEG	POS	NEG	NEG	POS	POS	POS	POS	NEG	NEG	POS	POS	POS	NEG	NEG	NEG
Riyadh_Alfaisal-10_SICU_13_B5672_1117353	POS	POS	NEG	POS	NEG	NEG	POS	POS	POS	POS	NEG	NEG	POS	POS	POS	NEG	NEG	NEG
Riyadh_SSTI_17_3508602	POS	POS	NEG	POS	NEG	NEG	POS	POS	POS	POS	NEG	NEG	POS	POS	POS	NEG	NEG	NEG
Riyadh-2822088-R	POS	POS	AMB	POS	AMB	NEG	POS	POS	POS	POS	NEG	NEG	POS	POS	POS	NEG	NEG	NEG
Trinidad&Tobago_2012_195 (619/12)	POS	POS	NEG	POS	NEG	NEG	POS	POS	POS	POS	NEG	NEG	POS	POS	POS	NEG	NEG	NEG
Trinidad&Tobago_2012_370 (1112-52177)	POS	POS	NEG	POS	NEG	AMB	POS	POS	POS	POS	NEG	NEG	POS	POS	POS	NEG	NEG	NEG
Trinidad&Tobago_2012_378 (4139)	POS	POS	NEG	POS	NEG	NEG	POS	POS	POS	POS	NEG	NEG	POS	POS	POS	NEG	NEG	NEG
Trinidad&Tobago_2013_PA21	POS	POS	NEG	POS	NEG	NEG	POS	POS	POS	POS	NEG	NEG	POS	POS	POS	NEG	NEG	NEG
Trinidad&Tobago_2013_PA22	POS	POS	NEG	POS	NEG	NEG	POS	POS	POS	POS	NEG	NEG	POS	POS	POS	NEG	NEG	NEG
Trinidad&Tobago_2912_340 (8523/12)	POS	POS	NEG	POS	NEG	NEG	POS	POS	POS	POS	NEG	NEG	POS	POS	POS	NEG	NEG	NEG
Trinidad&Tobago_MRSA2010_105	POS	POS	NEG	POS	NEG	NEG	POS	POS	POS	POS	NEG	NEG	POS	POS	POS	NEG	NEG	NEG
Trinidad&Tobago_MRSA2010_108	POS	POS	NEG	POS	NEG	POS	POS	POS	POS	POS	NEG	NEG	POS	POS	POS	NEG	NEG	NEG
Trinidad&Tobago_MRSA2010_33	POS	POS	NEG	POS	NEG	NEG	POS	POS	POS	POS	NEG	NEG	POS	POS	POS	NEG	NEG	NEG
Trinidad&Tobago_MRSA2010_79	POS	POS	NEG	POS	NEG	NEG	POS	POS	POS	POS	NEG	NEG	POS	POS	POS	NEG	NEG	NEG
Trinidad&Tobago_MRSA2010_88	POS	POS	NEG	POS	NEG	NEG	POS	POS	POS	POS	NEG	NEG	POS	POS	POS	NEG	NEG	NEG
Trinidad&Tobago_MRSA2010_97	POS	POS	NEG	POS	NEG	NEG	POS	POS	POS	POS	NEG	NEG	POS	POS	POS	NEG	NEG	NEG
Trinidad&Tobago_SSTI 2012_031	POS	POS	NEG	POS	NEG	NEG	POS	POS	POS	POS	NEG	NEG	POS	POS	POS	NEG	NEG	NEG
Uganda-29_17148_250811	POS	POS	NEG	POS	NEG	NEG	POS	POS	POS	POS	NEG	NEG	POS	POS	POS	NEG	NEG	NEG
>"South-East Asian Clad																		
UCIM6015	POS	POS	NEG	POS	NEG	NEG	POS	POS	POS	POS	NEG	NEG	POS	POS	POS	NEG	NEG	NEG
Hong Kong_121	POS	POS	NEG	POS	NEG	NEG	POS	POS	POS	POS	NEG	NEG	POS	POS	POS	NEG	NEG	NEG
>"South-East Asian Clad																		
Z172 (Taiwan)	POS	POS	NEG	POS	NEG	NEG	POS	POS	POS	POS	NEG	NEG	POS	POS	POS	NEG	NEG	NEG
Russia-19_0342_Kurgan	POS	POS	NEG	POS	NEG	NEG	POS	POS	POS	POS	NEG	NEG	POS	POS	POS	NEG	NEG	NEG
>"South-East Asian Clad																		
Perth_01-15392	POS	POS	NEG	POS	NEG	NEG	POS	POS	POS	POS	NEG	NEG	POS	POS	POS	NEG	NEG	NEG
>"South-East Asian Clad																		
XN108 (China)	POS	POS	NEG	POS	NEG	NEG	POS	POS	POS	POS	NEG	NEG	POS	POS	POS	NEG	NEG	NEG
NMR09 (India)	POS	POS	NEG	POS	NEG	NEG	POS	POS	POS	POS	NEG	NEG	POS	POS	POS	NEG	NEG	NEG
346 (Malaysia)	POS	POS	NEG	POS	NEG	NEG	POS	POS	POS	POS	NEG	NEG	POS	POS	POS	NEG	NEG	NEG
DS_009 (Thailand)	POS	POS	NEG	POS	NEG	NEG	POS	POS	POS	POS	NEG	NEG	POS	POS	POS	NEG	NEG	NEG
18ANR581005 (Ind)	POS	POS	NEG	POS	NEG	NEG	POS	POS	POS	POS	NEG	NEG	POS	POS	POS	NEG	NEG	NEG
Bengalaru_ST20141405	POS	POS	NEG	POS	NEG	AMB	POS	POS	POS	POS	NEG	NEG	POS	POS	POS	NEG	NEG	NEG
Bengalaru_ST20141406	POS	POS	NEG	POS	NEG	NEG	POS	POS	POS	POS	NEG	NEG	POS	POS	POS	NEG	NEG	NEG
Bengalaru_ST20141417	POS	POS	NEG	POS	NEG	NEG	POS	POS	POS	POS	NEG	NEG	POS	POS	POS	NEG	NEG	NEG
Hong Kong_107	AMB	POS	NEG	POS	NEG	NEG	POS	AMB	POS	POS	NEG	NEG	AMB	POS	POS	NEG	NEG	NEG
Hong Kong_12	POS	POS	NEG	POS	NEG	NEG	POS	POS	POS	POS	NEG	NEG	POS	POS	POS	NEG	NEG	NEG
Hong Kong_132	AMB	POS	NEG	POS	NEG	NEG	POS	AMB	POS	POS	NEG	NEG	AMB	POS	POS	NEG	NEG	NEG
Riyadh-3028763-R	POS	POS	NEG	POS	NEG	NEG	POS	POS	POS	POS	NEG	NEG	POS	POS	POS	NEG	NEG	NEG
Trinidad&Tobago_MRSA2010_94	POS	POS	NEG	POS	NEG	NEG	POS	POS	POS	POS	NEG	NEG	POS	POS	POS	NEG	NEG	NEG
>"South-East Asian Clad																		
M705 (Thailand)	POS	POS	NEG	POS	NEG	NEG	POS	POS	POS	POS	NEG	NEG	POS	POS	POS	NEG	NEG	NEG
S85 (Thailand)	POS	POS	NEG	POS	NEG	NEG	POS	POS	POS	POS	NEG	NEG	POS	POS	POS	NEG	NEG	NEG
Riyadh_Alfaisal-04_281014_S5076	POS	POS	NEG	POS	AMB	POS	POS	POS	POS	POS	NEG	NEG	POS	POS	POS	NEG	NEG	NEG
Riyadh_Alfaisal-26_S15145_1104163	POS	POS	NEG	POS	NEG	NEG	POS	POS	POS	POS	NEG	NEG	POS	POS	POS	NEG	NEG	NEG
Riyadh-2817437-W	POS	POS	AMB	POS	AMB	NEG	POS	POS	POS	POS	NEG	NEG	POS	POS	POS	NEG	NEG	NEG
>"South-East Asian Clad																		
PPUKM-775-2009 (Malaysia)	POS	POS	NEG	POS	NEG	NEG	POS	POS	POS	POS	NEG	NEG	POS	POS	POS	NEG	NEG	NEG
Hong Kong_138	POS	POS	NEG	POS	NEG	NEG	POS	POS	POS	POS	NEG	NEG	POS	POS	POS	NEG	NEG	NEG
>"South-East Asian Clad																		
Kuwait_001	POS	POS	NEG	POS	NEG	NEG	POS	POS	POS	POS	NEG	NEG	POS	POS	POS	NEG	NEG	NEG
Kuwait_337	POS	POS	NEG	POS	NEG	NEG	POS	POS	POS	POS	NEG	NEG	POS	POS	POS	NEG	NEG	NEG
>"South-East Asian Clad																		
CUHK_HK2007 (China)	POS	POS	NEG	POS	NEG	NEG	POS	POS	POS	POS	NEG	NEG	POS	POS	POS	NEG	NEG	NEG
NMR02 (India)	POS	POS	NEG	POS	NEG	NEG	POS	NEG	NEG	NEG	NEG	NEG	POS	POS	POS	NEG	NEG	NEG
VB1490 (India)	POS	POS	NEG	POS	NEG	NEG	POS	NEG	NEG	NEG	NEG	NEG	POS	POS	POS	NEG	NEG	NEG
V521 (Korea)	POS	POS	NEG	POS	NEG	NEG	POS	POS	POS	POS	NEG	NEG	POS	POS	POS	NEG	NEG	NEG
HST-077 (Lebanon)	POS	POS	NEG	POS	NEG	NEG	POS	POS	POS	POS	NEG	NEG	POS	POS	POS	NEG	NEG	NEG
MAL1 (Malaysia)	POS	POS	NEG	POS	NEG	NEG	POS	POS	POS	POS	NEG	NEG	POS	POS	POS	NEG	NEG	NEG
MAL3 (Malaysia)	POS	POS	NEG	POS	NEG	NEG	POS	POS	POS	POS	NEG	NEG	POS	POS	POS	NEG	NEG	NEG
PPUKM-261-2009 (Malaysia)	POS	POS	NEG	POS	NEG	NEG	POS	POS	POS	POS	NEG	NEG	POS	POS	POS	NEG	NEG	NEG
PPUKM-332-2009 (Malaysia)	POS	POS	NEG	POS	NEG	NEG	POS	POS	POS	POS	NEG	NEG	POS	POS	POS	NEG	NEG	NEG
S38 (Thailand)	POS	POS	NEG	POS	NEG	NEG	POS	POS	POS	POS	NEG	NEG	POS	POS	POS	NEG	NEG	NEG
S7 (Thailand)	POS	POS	NEG	POS	NEG	NEG	POS	POS	POS	POS	NEG	NEG	POS	POS	POS	NEG	NEG	NEG
US_008 (Thailand)	POS	POS	NEG	POS	NEG	NEG	POS	POS	POS	POS	NEG	NEG	POS	POS	POS	NEG	NEG	NEG
US_030 (Thailand)	POS	POS	NEG	POS	NEG	NEG	POS	POS	POS	POS	NEG	NEG	POS	POS	POS	NEG	NEG	NEG
M170 (Tsunami-related)	POS	POS	NEG	POS	NEG	NEG	POS	POS	POS	POS	NEG	NEG	POS	POS	POS	NEG	NEG	NEG
BSAC697 (UK)	POS	POS	NEG	POS	NEG	NEG	POS	POS	POS	POS	NEG	NEG	POS	POS	POS	NEG	NEG	NEG
IS-189	POS	POS	NEG	POS	NEG	NEG	POS	POS	POS	POS	NEG	NEG	POS	POS	POS	NEG	NEG	NEG
MRGR3	POS	POS	NEG	POS	NEG	NEG	POS	POS	POS	POS	NEG	NEG	POS	POS	POS	NEG	NEG	NEG
smg07	POS	POS	NEG	POS	NEG	NEG	POS	POS	POS	POS	NEG	NEG	POS	POS	POS	NEG	NEG	NEG
AH1-AUS-EMRSA-3	AMB	POS	NEG	POS	NEG	NEG	POS	AMB	POS	POS	NEG	NEG	AMB	POS	POS	NEG	NEG	NEG
Bengalaru_ST20141403	POS	POS	NEG	POS	NEG	NEG	POS	POS	POS	POS	NEG	NEG	POS	POS	POS	NEG	NEG	NEG
Finland E24_98541_Harmony	POS	POS	NEG	POS	NEG	NEG	POS	POS	POS	POS	NEG	NEG	POS	POS	POS	NEG	NEG	NEG
Hong Kong_10	AMB	POS	NEG	POS	NEG	NEG	POS	POS	POS	POS	NEG	NEG	POS	POS	POS	NEG	NEG	NEG
Hong Kong_100	POS	POS	NEG	POS	NEG	NEG	POS	POS	POS	POS	NEG	NEG	POS	POS	POS	NEG	NEG	NEG
Hong Kong_199	POS	POS	NEG	POS	NEG	AMB	POS	POS	POS	POS	NEG	NEG	POS	POS	POS	NEG	NEG	NEG
Hong Kong_34	POS	POS	NEG	POS	NEG	NEG	POS	POS	POS	POS	NEG	NEG	POS	POS	POS	NEG	NEG	NEG
Hong Kong_88	AMB	POS	NEG	POS	AMB	POS	POS	POS	POS	POS	NEG	NEG	POS	POS	POS	NEG	NEG	NEG
Hong Kong_92	POS	POS	NEG	POS	NEG	NEG	POS	POS	POS	POS	NEG	NEG	POS	POS	POS	NEG	NEG	NEG
Hong Kong_94	POS	POS	NEG	POS	NEG	NEG	POS	POS	POS	POS	NEG	NEG	POS	POS	POS	NEG	NEG	NEG
Kuwait_257	POS	POS	NEG	POS	NEG	NEG	POS	POS	POS	POS	NEG	NEG	POS	POS	POS	NEG	NEG	NEG
Perth_01-15330	POS	POS	NEG	POS	NEG	NEG	POS	POS	POS	POS	NEG	NEG	POS	POS	POS	NEG	NEG	NEG
Perth_01-15349	POS	POS	NEG	POS	NEG	NEG	POS	POS	POS	POS	NEG	NEG	POS	POS	POS	NEG	NEG	NEG
Perth_01-16176	POS	POS	NEG	POS	NEG	NEG	POS	POS	POS	POS	NEG	NEG	POS	POS	POS	NEG	NEG	NEG
Perth_WBG8379	POS	POS	NEG	POS	NEG	NEG	POS	POS	POS	POS	NEG	NEG	POS	POS	POS	NEG	NEG	NEG
Rawalpindi_Kidney Center_56	POS	POS	NEG	POS	NEG	NEG	POS	POS	POS	POS	NEG	NEG	POS	POS	POS	NEG	NEG	NEG
Riyadh_SSTI_05_3464614	POS	POS	NEG	POS	NEG	NEG	POS	POS	POS	POS	NEG	NEG	POS	POS	POS	NEG	NEG	NEG
Trinidad&Tobago_2012_157	POS	POS	NEG	POS	NEG	AMB	POS	POS	POS	POS	NEG	NEG	POS	POS	POS	NEG	NEG	NEG

STRAIN / ISOLATE	ADHAESION FACTORS / GENES ENCODING MICROBIAL SURFACE COMPONENTS RECOGNIZING ADHESIVE MATRIX MOLECULES																	
	sdrC							sdrD					vwb					
	sdrC	sdrC (cons)	sdrC (B1)	sdrC (COL)	sdrC (Mu50)	sdrC (MW2+MRSA252+RF122)	sdrC (OtherThan252+RF122)	sdrD	sdrD (cons)	sdrD (COL+MW2)	sdrD (Mu50)	sdrD (other)	vwb	vwb (cons)	vwb (COL+MW2)	vwb (MRSA252)	vwb (Mu50)	vwb (RF122)
	Ser-Asp rich fibrinogen-/bone sialoprotein-binding protein C							Ser-Asp rich fibrinogen-/bone sialoprotein-binding protein D					van Willebrand factor binding protein					
>"South-East Asian Clad																		
M418	POS	POS	NEG	POS	NEG	NEG	POS	POS	POS	POS	NEG	NEG	POS	POS	POS	NEG	NEG	NEG
>"South-East Asian Clad																		
NMR07 (India)	POS	POS	NEG	POS	NEG	NEG	POS	POS	POS	POS	NEG	NEG	POS	POS	POS	NEG	NEG	NEG
NMR08 (India)	POS	POS	NEG	POS	NEG	NEG	POS	POS	POS	POS	NEG	NEG	POS	POS	POS	NEG	NEG	NEG
Bengalaru_ST20121978	POS	POS	NEG	POS	NEG	NEG	POS	POS	POS	POS	NEG	NEG	POS	POS	POS	NEG	NEG	NEG
Hong Kong_89	POS	POS	AMB	POS	AMB	AMB	POS	POS	POS	POS	NEG	NEG	POS	POS	POS	NEG	NEG	NEG
>"South-East Asian Clad																		
Perth_2005_AH_5	POS	POS	NEG	POS	NEG	NEG	POS	POS	POS	POS	NEG	NEG	POS	POS	POS	NEG	NEG	NEG
>"South-East Asian Clad																		
Trinidad&Tobago_MRSA2010_103	POS	POS	NEG	POS	NEG	NEG	POS	POS	POS	POS	NEG	NEG	POS	POS	POS	NEG	NEG	NEG
Trinidad&Tobago_2013_PA25	POS	POS	NEG	POS	NEG	NEG	POS	POS	POS	POS	NEG	NEG	POS	POS	POS	NEG	NEG	NEG
Trinidad&Tobago_MRSA2010_110	POS	POS	NEG	POS	NEG	NEG	POS	POS	POS	POS	NEG	NEG	POS	POS	POS	NEG	NEG	NEG
Trinidad&Tobago_SSTI 2012_111	POS	POS	NEG	POS	NEG	NEG	POS	POS	POS	POS	NEG	NEG	POS	POS	POS	NEG	NEG	NEG
Trinidad&Tobago_MRSA2010_158	AMB	POS	NEG	POS	NEG	NEG	POS	POS	POS	POS	NEG	NEG	POS	POS	POS	NEG	NEG	NEG
>"South-East Asian Clad																		
Kuwait_122	POS	POS	NEG	POS	NEG	NEG	POS	POS	POS	POS	NEG	NEG	POS	POS	POS	NEG	NEG	NEG
Kuwait_002	POS	POS	NEG	POS	NEG	NEG	POS	POS	POS	POS	NEG	NEG	POS	POS	POS	NEG	NEG	NEG
Kuwait_196	POS	POS	NEG	POS	NEG	NEG	POS	POS	POS	POS	NEG	NEG	POS	POS	POS	NEG	NEG	NEG
Kuwait_199	POS	POS	NEG	POS	NEG	NEG	POS	POS	POS	POS	NEG	NEG	POS	POS	POS	NEG	NEG	NEG
Hong Kong_136	POS	POS	NEG	POS	NEG	NEG	POS	POS	POS	POS	NEG	NEG	POS	POS	POS	NEG	NEG	NEG
>"South-East Asian Clad																		
P32 (Poland)	POS	POS	NEG	POS	NEG	NEG	POS	NEG	NEG	NEG	NEG	NEG	POS	POS	POS	NEG	NEG	NEG
>"South-East Asian Clad																		
Perth_2015-S32313	POS	POS	NEG	POS	NEG	NEG	POS	POS	POS	POS	NEG	NEG	POS	POS	POS	NEG	NEG	NEG
>"hla-negative South-Ea																		
DEN907 (Denmark)	POS	POS	NEG	POS	NEG	NEG	POS	POS	POS	POS	NEG	NEG	POS	POS	POS	NEG	NEG	NEG
>"hla-negative South-Ea																		
S26 (Thailand)	POS	POS	NEG	POS	NEG	NEG	POS	POS	POS	POS	NEG	NEG	POS	POS	POS	NEG	NEG	NEG
S42 (Thailand)	POS	POS	NEG	POS	NEG	NEG	POS	POS	POS	POS	NEG	NEG	POS	POS	POS	NEG	NEG	NEG
S97 (Thailand)	POS	POS	NEG	POS	NEG	NEG	POS	POS	POS	POS	NEG	NEG	POS	POS	POS	NEG	NEG	NEG
S78 (Thailand)	POS	POS	NEG	POS	NEG	NEG	POS	POS	POS	POS	NEG	NEG	POS	POS	POS	NEG	NEG	NEG
S25 (Thailand)	POS	POS	NEG	POS	NEG	NEG	POS	POS	POS	POS	NEG	NEG	POS	POS	POS	NEG	NEG	NEG
S21 (Thailand)	POS	POS	NEG	POS	NEG	NEG	POS	POS	POS	POS	NEG	NEG	POS	POS	POS	NEG	NEG	NEG
S106 (Thailand)	POS	POS	NEG	POS	NEG	NEG	POS	POS	POS	POS	NEG	NEG	POS	POS	POS	NEG	NEG	NEG
S24 (Thailand)	POS	POS	NEG	POS	NEG	NEG	POS	POS	POS	POS	NEG	NEG	POS	POS	POS	NEG	NEG	NEG
S39 (Thailand)	POS	POS	NEG	POS	NEG	NEG	POS	POS	POS	POS	NEG	NEG	POS	POS	POS	NEG	NEG	NEG
S2 (Thailand)	POS	POS	NEG	POS	NEG	NEG	POS	POS	POS	POS	NEG	NEG	POS	POS	POS	NEG	NEG	NEG
S81 (Thailand)	POS	POS	NEG	POS	NEG	NEG	POS	POS	POS	POS	NEG	NEG	POS	POS	POS	NEG	NEG	NEG
M116 (Vietnam)	POS	POS	NEG	POS	NEG	NEG	POS	POS	POS	POS	NEG	NEG	POS	POS	POS	NEG	NEG	NEG
>"Portuguese Clade": Pc																		
FFP103 (Portugal)	POS	POS	NEG	POS	NEG	NEG	POS	POS	POS	POS	NEG	NEG	POS	POS	POS	NEG	NEG	NEG
HDG2 (Portugal)	POS	POS	NEG	POS	NEG	NEG	POS	POS	POS	POS	NEG	NEG	POS	POS	POS	NEG	NEG	NEG
HSA10 (Portugal)	POS	POS	NEG	POS	NEG	NEG	POS	NEG	NEG	NEG	NEG	NEG	POS	POS	POS	NEG	NEG	NEG
HSA11 (Portugal)	POS	POS	NEG	POS	NEG	NEG	POS	POS	POS	POS	NEG	NEG	POS	POS	POS	NEG	NEG	NEG
ICP5011 (Portugal)	POS	POS	NEG	POS	NEG	NEG	POS	NEG	NEG	NEG	NEG	NEG	POS	POS	POS	NEG	NEG	NEG
ICP5014 (Portugal)	POS	POS	NEG	POS	NEG	NEG	POS	POS	POS	POS	NEG	NEG	POS	POS	POS	NEG	NEG	NEG
ICP5062 (Portugal)	POS	POS	NEG	POS	NEG	NEG	POS	POS	POS	POS	NEG	NEG	POS	POS	POS	NEG	NEG	NEG
Strain 1063_ATCC 33592 FDAARGOS_3 (USA)	POS	POS	NEG	POS	NEG	NEG	POS	POS	POS	POS	NEG	NEG	POS	POS	POS	NEG	NEG	NEG
	POS	POS	NEG	POS	NEG	NEG	POS	POS	POS	POS	NEG	NEG	POS	POS	POS	NEG	NEG	NEG
Russia-07_0110_Kurgan	POS	POS	NEG	POS	NEG	NEG	POS	NEG	NEG	NEG	NEG	NEG	POS	POS	POS	NEG	NEG	NEG
Russia-11_0164_Kurgan	POS	POS	NEG	POS	NEG	NEG	POS	POS	POS	POS	NEG	NEG	POS	POS	POS	NEG	NEG	NEG
Russia-20_0367_Kurgan	POS	POS	NEG	POS	NEG	NEG	POS	POS	POS	POS	NEG	NEG	POS	POS	POS	NEG	NEG	NEG
Russia-21_0390_Chelyabinsk	POS	POS	NEG	POS	NEG	NEG	POS	POS	POS	POS	NEG	NEG	POS	POS	POS	NEG	NEG	NEG
>"South American/ Mid																		
AGT1 (Argentina)	POS	POS	NEG	POS	NEG	NEG	POS	POS	POS	POS	NEG	NEG	POS	POS	POS	NEG	NEG	NEG
AGT120 (Argentina)	POS	POS	NEG	POS	NEG	NEG	POS	POS	POS	POS	NEG	NEG	POS	POS	POS	NEG	NEG	NEG
AGT67 (Argentina)	POS	POS	NEG	POS	NEG	NEG	POS	POS	POS	POS	NEG	NEG	POS	POS	POS	NEG	NEG	NEG
AGT9 (Argentina)	POS	POS	NEG	POS	NEG	NEG	POS	POS	POS	POS	NEG	NEG	POS	POS	POS	NEG	NEG	NEG
RA3 (Argentina)	POS	POS	NEG	POS	NEG	NEG	POS	POS	POS	POS	NEG	NEG	POS	POS	POS	NEG	NEG	NEG
RA6 (Argentina)	POS	POS	NEG	POS	NEG	NEG	POS	POS	POS	POS	NEG	NEG	POS	POS	POS	NEG	NEG	NEG
RA7 (Argentina)	POS	POS	NEG	POS	NEG	NEG	POS	POS	POS	POS	NEG	NEG	POS	POS	POS	NEG	NEG	NEG
Be62 (Brazil)	POS	POS	NEG	POS	NEG	NEG	POS	POS	POS	POS	NEG	NEG	POS	POS	POS	NEG	NEG	NEG
Bmb9393 (Brazil)	POS	POS	NEG	POS	NEG	NEG	POS	POS	POS	POS	NEG	NEG	POS	POS	POS	NEG	NEG	NEG
BRA36 (Brazil)	POS	POS	NEG	POS	NEG	NEG	POS	POS	POS	POS	NEG	NEG	POS	POS	POS	NEG	NEG	NEG
BZ48 (Brazil)	POS	POS	NEG	POS	NEG	NEG	POS	POS	POS	POS	NEG	NEG	POS	POS	POS	NEG	NEG	NEG
Gv51 (Brazil)	POS	POS	NEG	POS	NEG	NEG	POS	POS	POS	POS	NEG	NEG	POS	POS	POS	NEG	NEG	NEG
Gv69 (Brazil)	POS	POS	NEG	POS	NEG	NEG	POS	POS	POS	POS	NEG	NEG	POS	POS	POS	NEG	NEG	NEG
Gv88 (Brazil)	POS	POS	NEG	POS	NEG	NEG	POS	POS	POS	POS	NEG	NEG	POS	POS	POS	NEG	NEG	NEG
HC1335 (Brazil)	POS	POS	NEG	POS	NEG	NEG	POS	POS	POS	POS	NEG	NEG	POS	POS	POS	NEG	NEG	NEG
HC1340 (Brazil)	POS	POS	NEG	POS	NEG	NEG	POS	POS	POS	POS	NEG	NEG	POS	POS	POS	NEG	NEG	NEG
HC556 (Brazil)	POS	POS	NEG	POS	NEG	NEG	POS	POS	POS	POS	NEG	NEG	POS	POS	POS	NEG	NEG	NEG
HU25 (Brazil)	POS	POS	NEG	POS	NEG	NEG	POS	POS	POS	POS	NEG	NEG	POS	POS	POS	NEG	NEG	NEG
UB563 (Brazil)	POS	POS	NEG	POS	NEG	NEG	POS	POS	POS	POS	NEG	NEG	POS	POS	POS	NEG	NEG	NEG
2A8 (Czech Republic)	POS	POS	NEG	POS	NEG	NEG	POS	POS	POS	POS	NEG	NEG	POS	POS	POS	NEG	NEG	NEG
M1229 (Denmark)	POS	POS	NEG	POS	NEG	NEG	POS	POS	POS	POS	NEG	NEG	POS	POS	POS	NEG	NEG	NEG
FRICAR (France)	POS	POS	NEG	POS	NEG	NEG	POS	POS	POS	POS	NEG	NEG	POS	POS	POS	NEG	NEG	NEG
D90 (Germany)	POS	POS	NEG	POS	NEG	NEG	POS	POS	POS	POS	NEG	NEG	POS	POS	POS	NEG	NEG	NEG
LIT68 (Lithuania)	POS	POS	NEG	POS	NEG	NEG	POS	POS	POS	POS	NEG	NEG	POS	POS	POS	NEG	NEG	NEG
LIT76 (Lithuania)	POS	POS	NEG	POS	NEG	NEG	POS	POS	POS	POS	NEG	NEG	POS	POS	POS	NEG	NEG	NEG
HGSA142 (Portugal)	POS	POS	NEG	POS	NEG	NEG	POS	POS	POS	POS	NEG	NEG	POS	POS	POS	NEG	NEG	NEG
HGSA9 (Portugal)	POS	POS	NEG	POS	NEG	NEG	POS	POS	POS	POS	NEG	NEG	POS	POS	POS	NEG	NEG	NEG
HSJ216 (Portugal)	POS	POS	NEG	POS	NEG	NEG	POS	POS	POS	POS	NEG	NEG	POS	POS	POS	NEG	NEG	NEG
M278 (Portugal)	POS	POS	NEG	POS	NEG	NEG	POS	POS	POS	POS	NEG	NEG	POS	POS	POS	NEG	NEG	NEG
ES26 (Spain)	POS	POS	NEG	POS	NEG	NEG	POS	POS	POS	POS	NEG	NEG	POS	POS	POS	NEG	NEG	NEG
UK102 (UK)	POS	POS	NEG	POS	NEG	NEG	POS	POS	POS	POS	NEG	NEG	POS	POS	POS	NEG	NEG	NEG
URU34 (Uruguay)	POS	POS	NEG	POS	NEG	NEG	POS	POS	POS	POS	NEG	NEG	POS	POS	POS	NEG	NEG	NEG
IS-125	POS	POS	NEG	POS	NEG	NEG	POS	POS	POS	POS	NEG	NEG	POS	POS	POS	NEG	NEG	NEG
IS-157	POS	POS	NEG	POS	NEG	NEG	POS	POS	POS	POS	NEG	NEG	POS	POS	POS	NEG	NEG	NEG
Ecuador_1	POS	POS	NEG	POS	NEG	NEG	POS	POS	POS	POS	NEG	NEG	POS	POS	POS	NEG	NEG	NEG
Ecuador_2	POS	POS	NEG	POS	NEG	NEG	POS	POS	POS	POS	NEG	NEG	POS	POS	POS	NEG	NEG	NEG
Dresden_15ANR574263	POS	POS	NEG	POS	NEG	NEG	POS	POS	POS	POS	NEG	NEG	POS	POS	POS	NEG	NEG	NEG
UK-EMRSA-11	POS	POS	NEG	POS	NEG	NEG	POS	POS	POS	POS	NEG	NEG	POS	POS	POS	NEG	NEG	NEG
>"South American/ Mid																		
BRA2 (Brazil)	POS	POS	NEG	POS	NEG	NEG	POS	POS	POS	POS	NEG	NEG	POS	POS	POS	NEG	NEG	NEG
CHL1 (Chile)	POS	POS	NEG	POS	NEG	NEG	POS	POS	POS	POS	NEG	NEG	POS	POS	POS	NEG	NEG	NEG
CHL151 (Chile)	POS	POS	NEG	POS	NEG	NEG	POS	POS	POS	POS	NEG	NEG	POS	POS	POS	NEG	NEG	NEG
UCL378 (Chile)	POS	POS	NEG	POS	NEG	NEG	POS	POS	POS	POS	NEG	NEG	POS	POS	POS	NEG	NEG	NEG
UP72 (Peru)	POS	POS	NEG	POS	NEG	NEG	POS	POS	POS	POS	NEG	NEG	POS	POS	POS	NEG	NEG	NEG
UP81 (Peru)	POS	POS	NEG	POS	NEG	NEG	POS	POS	POS	POS	NEG	NEG	POS	POS	POS	NEG	NEG	NEG
>"South American/ Mid																		
UP1073 (Peru)	POS	POS	NEG	POS	NEG	NEG	POS	POS	POS	POS	NEG	NEG	POS	POS	POS	NEG	NEG	NEG

STRAIN / ISOLATE	ADHAESION FACTORS / GENES ENCODING MICROBIAL SURFACE COMPONENTS RECOGNIZING ADHESIVE MATRIX MOLECULES																	
	sdrC							sdrD					vwb					
	sdrC	sdrC (cons)	sdrC (B1)	sdrC (COL)	sdrC (Mu50)	sdrC (MW2+MRSA252+RF122)	sdrC (OtherThan252+RF122)	sdrD	sdrD (cons)	sdrD (COL+MW2)	sdrD (Mu50)	sdrD (other)	vwb	vwb (cons)	vwb (COL+MW2)	vwb (MRSA252)	vwb (Mu50)	vwb (RF122)
	Ser-Asp rich fibrinogen-/bone sialoprotein-binding protein C							Ser-Asp rich fibrinogen-/bone sialoprotein-binding protein D					van Willebrand factor binding protein					
>"South American/ Mid																		
LIT89 (Lithuania)	POS	POS	NEG	POS	NEG	NEG	POS	POS	POS	POS	NEG	NEG	POS	POS	POS	NEG	NEG	NEG
MRSA_PR1 (Malaysia)	POS	POS	NEG	POS	NEG	NEG	POS	POS	POS	POS	NEG	NEG	POS	POS	POS	NEG	NEG	NEG
Riyadh-2888905-R	POS	POS	NEG	POS	NEG	NEG	POS	POS	POS	POS	NEG	NEG	POS	POS	POS	NEG	NEG	NEG
Riyadh-2888915-BC	POS	POS	NEG	POS	NEG	NEG	POS	POS	POS	POS	NEG	NEG	POS	POS	POS	NEG	NEG	NEG
Riyadh-2793706-R	POS	POS	AMB	POS	AMB	NEG	POS	POS	POS	POS	NEG	NEG	POS	POS	POS	NEG	NEG	NEG
>"South American/ Mid																		
Riyadh_SSTI_18_3502925	POS	POS	AMB	POS	NEG	POS	POS	POS	POS	POS	NEG	NEG	POS	POS	POS	NEG	NEG	NEG
Riyadh-2822825-W	POS	POS	POS	POS	AMB	AMB	POS	POS	POS	POS	NEG	NEG	POS	POS	POS	NEG	NEG	NEG
>"South American/ Mid																		
UK-EMRSA-9	POS	POS	NEG	POS	NEG	NEG	POS	POS	POS	POS	NEG	NEG	POS	POS	POS	NEG	NEG	NEG
>"South American/ Mid																		
Lome_HT20020815	POS	POS	NEG	POS	NEG	NEG	POS	POS	POS	POS	NEG	NEG	POS	POS	POS	NEG	NEG	NEG
>"South American/ Mid																		
NA32 (Denmark)	POS	POS	NEG	POS	NEG	NEG	POS	NEG	NEG	NEG	NEG	NEG	POS	POS	POS	NEG	NEG	NEG
H24 (Egypt)	POS	POS	NEG	POS	NEG	NEG	POS	POS	POS	POS	NEG	NEG	POS	POS	POS	NEG	NEG	NEG
ATCC BAA-39 (=HUSA304) (Hungary)	POS	POS	NEG	POS	NEG	NEG	POS	POS	POS	POS	NEG	NEG	POS	POS	POS	NEG	NEG	NEG
HUSA304 (Hungary)	POS	POS	NEG	POS	NEG	NEG	POS	POS	POS	POS	NEG	NEG	POS	POS	POS	NEG	NEG	NEG
HU106 (Hungary)	POS	POS	NEG	POS	NEG	NEG	POS	POS	POS	POS	NEG	NEG	POS	POS	POS	NEG	NEG	NEG
BSAC27 (UK)	POS	POS	NEG	POS	NEG	NEG	POS	POS	POS	POS	NEG	NEG	POS	POS	POS	NEG	NEG	NEG
NCTC13131, UK-EMRSA-4 (UK)	POS	POS	NEG	POS	NEG	NEG	POS	POS	POS	POS	NEG	NEG	POS	POS	POS	NEG	NEG	NEG
	POS	POS	NEG	POS	NEG	NEG	POS	POS	POS	POS	NEG	NEG	POS	POS	POS	NEG	NEG	NEG
Algiers_HT20040080	POS	POS	NEG	POS	NEG	NEG	POS	POS	POS	POS	NEG	NEG	POS	POS	POS	NEG	NEG	NEG
Dublin-DSH_AR09_0.0066	POS	POS	NEG	POS	NEG	NEG	POS	POS	POS	POS	NEG	NEG	POS	POS	POS	NEG	NEG	NEG
Dublin-DSH_AR09_0-0065	AMB	AMB	NEG	POS	NEG	NEG	POS	POS	POS	AMB	NEG	NEG	POS	POS	POS	NEG	NEG	NEG
Dublin-DSH_Phenotype-III_84	AMB	POS	NEG	POS	NEG	NEG	POS	POS	POS	POS	NEG	NEG	POS	POS	POS	NEG	NEG	NEG
Hong Kong_130	POS	POS	NEG	POS	NEG	NEG	POS	POS	POS	POS	NEG	NEG	POS	POS	POS	NEG	NEG	NEG
Kuwait_018	POS	POS	NEG	POS	NEG	NEG	POS	POS	POS	POS	NEG	NEG	POS	POS	POS	NEG	NEG	NEG
Perth_08-17726	POS	POS	NEG	POS	NEG	NEG	POS	POS	POS	POS	NEG	NEG	POS	POS	POS	NEG	NEG	NEG
Riyadh_Alfaisal/KKKSUH_86_MRS-14-279	POS	POS	NEG	POS	NEG	NEG	POS	POS	POS	POS	NEG	NEG	POS	POS	POS	NEG	NEG	NEG
Riyadh_Alfaisal-04_Z3861_831588	POS	POS	NEG	POS	NEG	NEG	POS	POS	POS	POS	NEG	NEG	POS	POS	POS	NEG	NEG	NEG
Riyadh_Alfaisal-30_S15724_1108013	POS	POS	NEG	POS	NEG	NEG	POS	POS	POS	POS	NEG	NEG	POS	POS	POS	NEG	NEG	NEG
Riyadh_Alfaisal-6_22A_13_B3992_397721	POS	POS	NEG	POS	NEG	NEG	POS	POS	POS	POS	NEG	NEG	POS	POS	POS	NEG	NEG	NEG
Riyadh_SSTI_52_3615482	POS	POS	NEG	POS	NEG	NEG	POS	POS	POS	POS	NEG	NEG	POS	POS	POS	NEG	NEG	NEG
Riyadh-2817276-2	POS	POS	AMB	POS	AMB	NEG	POS	POS	POS	POS	NEG	NEG	POS	POS	POS	NEG	NEG	NEG
Riyadh-2891670-W	POS	POS	AMB	POS	AMB	NEG	POS	POS	POS	POS	NEG	NEG	POS	POS	POS	NEG	NEG	NEG
Riyadh-3006920-W	POS	POS	AMB	POS	AMB	NEG	POS	POS	POS	POS	NEG	NEG	POS	POS	POS	NEG	NEG	NEG
Riyadh-R2567782	POS	POS	NEG	POS	NEG	NEG	POS	POS	POS	POS	NEG	NEG	POS	POS	POS	NEG	NEG	NEG
Russia-18_0252_Krasnoyarsk_SK2	POS	POS	NEG	POS	NEG	NEG	POS	POS	POS	POS	NEG	NEG	POS	POS	POS	NEG	NEG	NEG
UK-EMRSA-7	POS	POS	NEG	POS	NEG	NEG	POS	POS	POS	POS	NEG	NEG	POS	POS	POS	NEG	NEG	NEG
>"South American/ Mid																		
Dublin-DSH_AR23_0073	POS	POS	NEG	POS	NEG	NEG	POS	POS	POS	POS	NEG	NEG	POS	POS	POS	NEG	NEG	NEG
>Related to "South Ame																		
MRSA-OC3 (Russia)	POS	POS	NEG	POS	NEG	NEG	POS	POS	POS	POS	NEG	NEG	POS	POS	POS	NEG	NEG	NEG
Russia-12_0176_Krasnoyarsk	POS	POS	NEG	POS	NEG	NEG	POS	POS	POS	POS	NEG	NEG	POS	POS	POS	NEG	NEG	NEG
Russia-13_0180_Krasnoyarsk	POS	POS	NEG	POS	NEG	NEG	POS	POS	POS	POS	NEG	NEG	POS	POS	POS	NEG	NEG	NEG
Russia-16_0249_Krasnoyarsk	POS	POS	NEG	POS	NEG	NEG	POS	POS	POS	POS	NEG	NEG	POS	POS	POS	NEG	NEG	NEG
Russia-17_0250_Krasnoyarsk	POS	POS	NEG	POS	NEG	NEG	POS	POS	POS	POS	NEG	NEG	POS	POS	POS	NEG	NEG	NEG
>Related to "South Ame																		
URU110 (Uruguay)	POS	POS	NEG	POS	NEG	NEG	POS	POS	POS	POS	NEG	NEG	POS	POS	POS	NEG	NEG	NEG
>Related to "South Ame																		
DS_014 (Thailand)	POS	POS	NEG	POS	NEG	NEG	POS	POS	POS	POS	NEG	NEG	POS	POS	POS	NEG	NEG	NEG
>Unassigned Middle Eas																		
Frankfurt_Oder_0490031797 (Libya)	POS	POS	NEG	POS	NEG	NEG	POS	POS	POS	POS	NEG	NEG	POS	POS	POS	NEG	NEG	NEG
Kuwait_192	POS	POS	NEG	POS	NEG	AMB	POS	POS	POS	POS	NEG	NEG	POS	POS	POS	NEG	NEG	NEG
Riyadh-9	POS	POS	NEG	POS	NEG	NEG	POS	POS	POS	POS	NEG	NEG	POS	POS	POS	NEG	NEG	NEG
Russia-24_0407_Moscow	POS	POS	NEG	POS	NEG	NEG	POS	POS	POS	POS	NEG	NEG	POS	POS	POS	NEG	NEG	NEG

STRAIN / ISOLATE	IMMUNOD.AG.B		DEFENSIN RESIST.		TRANSFERRIN BINDING PROT			PUTATIVE TRANSPORTER		TYPE I RESTR:-MODIFICATION SYSTEM				
	isaB		mprF		isdA			lmrP		hsdS1	hsdS2			
	isaB	isaB (MRSA252)	mprF (COL+MW2)	mprF (Mu50+252)	isdA (cons)	isdA (MRSA252)	isdA (Other Than MRSA252)	lmrP (OtherThanRF122)	lmrP (RF122)	hsdS1-RF122	hsdS2-ST5+ST8	hsdS2-MW2+476	hsdS2-RF122	hsdS2-MRSA252
	immunodominant antigen B		defensin resistance protein		transferrin-binding protein			hypothetical protein, similar to integral membrane protein LmrP		type I site-specific deoxyribo-nuclease subunit, 1st locus	type I site-specific deoxyribonuclease subunit, 2nd locus			
**>"Eurasian Clade": TUR1**														
TUR1 (Turkey)	NEG	POS	POS	NEG	POS	NEG	POS	POS	NEG	NEG	POS	NEG	NEG	NEG
TUR9 (Turkey)	NEG	POS	POS	NEG	POS	NEG	POS	POS	NEG	NEG	POS	NEG	NEG	NEG
**>"Eurasian Clade" : T013**														
T0131 (China)	NEG	POS	POS	NEG	POS	NEG	POS	POS	NEG	NEG	POS	NEG	NEG	NEG
MU4 (Turkey)	NEG	POS	POS	NEG	POS	NEG	POS	POS	NEG	NEG	POS	NEG	NEG	NEG
DEU11 (Turkey)	NEG	POS	POS	NEG	POS	NEG	POS	POS	NEG	NEG	POS	NEG	NEG	NEG
DEU16 (Turkey)	NEG	POS	POS	NEG	POS	NEG	POS	POS	NEG	NEG	POS	NEG	NEG	NEG
Romania_Iasi_MRSA-07_54	NEG	POS	POS	AMB	POS	AMB	POS	POS	NEG	NEG	POS		NEG	NEG
**>"Eurasian Clade": arsC-**														
Romania_Iasi_MRSA-05_46	NEG	POS	POS	AMB	POS	NEG	POS	POS	NEG	NEG	POS	NEG	NEG	NEG
Romania_Iasi_MRSA-06_47	NEG	POS	POS	AMB	POS	AMB	POS	POS	NEG	NEG	POS	NEG	NEG	NEG
**>"Eurasian Clade": 16K/**														
CN79 (China)	NEG	POS	POS	NEG	POS	NEG	POS	POS	NEG	NEG	POS	NEG	NEG	NEG
CUHK_BJ2002 (China)	NEG	POS	POS	NEG	POS	NEG	POS	POS	NEG	NEG	POS	NEG	NEG	NEG
CUHK_BJ2007 (China)	NEG	POS	POS	NEG	POS	NEG	POS	POS	NEG	NEG	POS	NEG	NEG	NEG
3HK (Czech Republic)	NEG	POS	POS	NEG	POS	NEG	POS	POS	NEG	NEG	POS	NEG	NEG	NEG
HU109 (Hungary)	NEG	POS	POS	NEG	POS	NEG	POS	POS	NEG	NEG	POS	NEG	NEG	NEG
HUR18 (Hungary)	NEG	POS	POS	NEG	POS	NEG	POS	POS	NEG	NEG	POS	NEG	NEG	NEG
H482 (Romania)	NEG	POS	POS	NEG	POS	NEG	POS	POS	NEG	NEG	POS	NEG	NEG	NEG
16K (Russia)	NEG	POS	POS	NEG	POS	NEG	POS	POS	NEG	NEG	POS	NEG	NEG	NEG
DEU10 (Turkey)	NEG	POS	POS	NEG	POS	NEG	POS	POS	NEG	NEG	POS	NEG	NEG	NEG
DEU12 (Turkey)	NEG	POS	POS	NEG	POS	NEG	POS	POS	NEG	NEG	POS	NEG	NEG	NEG
DEU14 (Turkey)	NEG	POS	POS	NEG	POS	NEG	POS	POS	NEG	NEG	POS	NEG	NEG	NEG
DEU15 (Turkey)	NEG	POS	POS	NEG	POS	NEG	POS	POS	NEG	NEG	POS	NEG	NEG	NEG
DEU17 (Turkey)	NEG	POS	POS	NEG	POS	NEG	POS	POS	NEG	NEG	POS	NEG	NEG	NEG
DEU19 (Turkey)	NEG	POS	POS	NEG	POS	NEG	POS	POS	NEG	NEG	POS	NEG	NEG	NEG
DEU2 (Turkey)	NEG	POS	POS	NEG	POS	NEG	POS	POS	NEG	NEG	POS	NEG	NEG	NEG
DEU20 (Turkey)	NEG	POS	POS	NEG	POS	NEG	POS	POS	NEG	NEG	POS	NEG	NEG	NEG
DEU23 (Turkey)	NEG	POS	POS	NEG	POS	NEG	POS	POS	NEG	NEG	POS	NEG	NEG	NEG
DEU3 (Turkey)	NEG	POS	POS	NEG	POS	NEG	POS	POS	NEG	NEG	POS	NEG	NEG	NEG
DEU5 (Turkey)	NEG	POS	POS	NEG	POS	NEG	POS	POS	NEG	NEG	POS	NEG	NEG	NEG
DEU6 (Turkey)	NEG	POS	POS	NEG	POS	NEG	POS	POS	NEG	NEG	POS	NEG	NEG	NEG
DEU8 (Turkey)	NEG	POS	POS	NEG	POS	NEG	POS	POS	NEG	NEG	POS	NEG	NEG	NEG
HU11 (Turkey)	NEG	POS	POS	NEG	POS	NEG	POS	POS	NEG	NEG	POS	NEG	NEG	NEG
HU13 (Turkey)	NEG	POS	POS	NEG	POS	NEG	POS	POS	NEG	NEG	POS	NEG	NEG	NEG
HU14 (Turkey)	NEG	POS	POS	NEG	POS	NEG	POS	POS	NEG	NEG	POS	NEG	NEG	NEG
HU15 (Turkey)	NEG	POS	POS	NEG	POS	NEG	POS	POS	NEG	NEG	POS	NEG	NEG	NEG
HU16 (Turkey)	NEG	POS	POS	NEG	POS	NEG	POS	POS	NEG	NEG	POS	NEG	NEG	NEG
HU17 (Turkey)	NEG	POS	POS	NEG	POS	NEG	POS	POS	NEG	NEG	POS	NEG	NEG	NEG
HU21 (Turkey)	NEG	POS	POS	NEG	POS	NEG	POS	POS	NEG	NEG	POS	NEG	NEG	NEG
HU23 (Turkey)	NEG	POS	POS	NEG	POS	NEG	POS	POS	NEG	NEG	POS	NEG	NEG	NEG
HU26 (Turkey)	NEG	POS	POS	NEG	POS	NEG	POS	POS	NEG	NEG	POS	NEG	NEG	NEG
HU4 (Turkey)	NEG	POS	POS	NEG	POS	NEG	POS	POS	NEG	NEG	POS	NEG	NEG	NEG
HU5 (Turkey)	NEG	POS	POS	NEG	POS	NEG	POS	POS	NEG	NEG	POS	NEG	NEG	NEG
HU6 (Turkey)	NEG	POS	POS	NEG	POS	NEG	POS	POS	NEG	NEG	POS	NEG	NEG	NEG
HU7 (Turkey)	NEG	POS	POS	NEG	POS	NEG	POS	POS	NEG	NEG	POS	NEG	NEG	NEG
HU8 (Turkey)	NEG	POS	POS	NEG	POS	NEG	POS	POS	NEG	NEG	POS	NEG	NEG	NEG
HU9 (Turkey)	NEG	POS	POS	NEG	POS	NEG	POS	POS	NEG	NEG	POS	NEG	NEG	NEG
IU1 (Turkey)	NEG	POS	POS	NEG	POS	NEG	POS	POS	NEG	NEG	POS	NEG	NEG	NEG
IU10 (Turkey)	NEG	POS	POS	NEG	POS	NEG	POS	POS	NEG	NEG	POS	NEG	NEG	NEG
IU11 (Turkey)	NEG	POS	POS	NEG	POS	NEG	POS	POS	NEG	NEG	POS	NEG	NEG	NEG
IU12 (Turkey)	NEG	POS	POS	NEG	POS	NEG	POS	POS	NEG	NEG	POS	NEG	NEG	NEG
IU13 (Turkey)	NEG	POS	POS	NEG	POS	NEG	POS	POS	NEG	NEG	POS	NEG	NEG	NEG
IU15 (Turkey)	NEG	POS	POS	NEG	POS	NEG	POS	POS	NEG	NEG	POS	NEG	NEG	NEG
IU18 (Turkey)	NEG	POS	POS	NEG	POS	NEG	POS	POS	NEG	NEG	POS	NEG	NEG	NEG
IU19 (Turkey)	NEG	POS	POS	NEG	POS	NEG	POS	POS	NEG	NEG	POS	NEG	NEG	NEG
IU2 (Turkey)	NEG	POS	POS	NEG	POS	NEG	POS	POS	NEG	NEG	POS	NEG	NEG	NEG
IU4 (Turkey)	NEG	POS	POS	NEG	POS	NEG	POS	POS	NEG	NEG	POS	NEG	NEG	NEG
IU6 (Turkey)	NEG	POS	POS	NEG	POS	NEG	POS	POS	NEG	NEG	POS	NEG	NEG	NEG
IU7 (Turkey)	NEG	POS	POS	NEG	POS	NEG	POS	POS	NEG	NEG	POS	NEG	NEG	NEG
IU9 (Turkey)	NEG	POS	POS	NEG	POS	NEG	POS	POS	NEG	NEG	POS	NEG	NEG	NEG
MU1 (Turkey)	NEG	POS	POS	NEG	POS	NEG	POS	POS	NEG	NEG	POS	NEG	NEG	NEG
MU10 (Turkey)	NEG	POS	POS	NEG	POS	NEG	POS	POS	NEG	NEG	POS	NEG	NEG	NEG
MU20 (Turkey)	NEG	POS	POS	NEG	POS	NEG	POS	POS	NEG	NEG	POS	NEG	NEG	NEG
Mu3 (Turkey)	NEG	POS	POS	NEG	POS	NEG	POS	POS	NEG	NEG	POS	NEG	NEG	NEG
MU5 (Turkey)	NEG	POS	POS	NEG	POS	NEG	POS	POS	NEG	NEG	POS	NEG	NEG	NEG
MU6 (Turkey)	NEG	POS	POS	NEG	POS	NEG	POS	POS	NEG	NEG	POS	NEG	NEG	NEG
MU7 (Turkey)	NEG	POS	POS	NEG	POS	NEG	POS	POS	NEG	NEG	POS	NEG	NEG	NEG
TUR27 (Turkey)	NEG	POS	POS	NEG	POS	NEG	POS	POS	NEG	NEG	POS	NEG	NEG	NEG
NCTR 32S	NEG	POS	POS	NEG	POS	NEG	POS	POS	NEG	NEG	POS	NEG	NEG	NEG
SA02_A4 = Russia-06_0085_Moscow	NEG	POS	POS	NEG	POS	NEG	POS	POS	NEG	NEG	POS	NEG	NEG	NEG
	NEG	POS	POS	AMB	POS	AMB	POS	POS	NEG	NEG	POS	NEG	NEG	NEG
Dresden_08V35987 (Turkey)	NEG	POS	POS	AMB	POS	NEG	POS	POS	NEG	NEG	POS	NEG	NEG	NEG
Dresden_17ANR577152	NEG	POS	POS	AMB	POS	NEG	POS	POS	NEG	NEG	POS	NEG	NEG	NEG
Dresden_17ANR580374 (Makedonia)	NEG	POS	POS	NEG	POS	NEG	POS	POS	NEG	NEG	POS	NEG	NEG	NEG
Hong Kong_69-II	NEG	POS	POS	AMB	POS	AMB	POS	POS	NEG	NEG	POS	NEG	NEG	NEG
Hong Kong_93	NEG	POS	POS	AMB	POS	AMB	POS	POS	NEG	NEG	POS	AMB	NEG	NEG
Rawalpindi_Kidney Center_03_SK1	NEG	POS	POS	AMB	POS	AMB	POS	POS	NEG	NEG	POS	NEG	NEG	NEG
Rawalpindi_Kidney Center_08	NEG	POS	POS	AMB	POS	NEG	POS	POS	NEG	NEG	POS	NEG	NEG	NEG
Rawalpindi_Kidney Center_10	NEG	POS	POS	AMB	POS	AMB	POS	POS	NEG	NEG	POS	NEG	NEG	NEG
Rawalpindi_Kidney Center_19	AMB	POS	POS	AMB	POS	AMB	POS	POS	NEG	NEG	POS	NEG	NEG	NEG
Rawalpindi_Kidney Center_50_SK1	NEG	POS	POS	AMB	POS	NEG	POS	POS	NEG	NEG	POS	NEG	NEG	NEG
Romania_Iasi_BC-49_430	NEG	POS	POS	AMB	POS	AMB	POS	POS	NEG	NEG	POS	AMB	NEG	NEG
Romania_Iasi_MRSA_284	NEG	POS	POS	AMB	POS	AMB	POS	POS	NEG	NEG	POS	AMB	NEG	NEG
Romania_Iasi_MRSA-32_318	NEG	POS	POS	AMB	POS	NEG	POS	POS	NEG	NEG	POS	NEG	NEG	NEG
Romania_Iasi_MRSA-54_430	AMB	POS	AMB	POS	POS	AMB	POS	POS	NEG	NEG	POS	POS	NEG	NEG
Romania_Iasi_SSTI-01_2	NEG	POS	POS	AMB	POS	AMB	POS	POS	NEG	NEG	POS	AMB	NEG	NEG
Romania_Iasi_SSTI-10_101	NEG	POS	POS	AMB	POS	NEG	POS	POS	NEG	NEG	POS	NEG	NEG	NEG
Romania_Iasi_SSTI-11_106	NEG	POS	POS	AMB	POS	NEG	POS	POS	NEG	NEG	POS	NEG	NEG	NEG
Russia-01_0001_SaintPetersburg	NEG	POS	POS	AMB	POS	NEG	POS	POS	NEG	NEG	POS	NEG	NEG	NEG
Russia-02_0004_SaintPetersburg	NEG	POS	POS	AMB	POS	AMB	POS	POS	NEG	NEG	POS	NEG	NEG	NEG
Russia-03_0057_SaintPetersburg	NEG	POS	POS	NEG	POS	NEG	POS	POS	NEG	NEG	POS	NEG	NEG	NEG
Russia-04_0076_Moscow	NEG	POS	POS	AMB	POS	NEG	POS	POS	NEG	NEG	POS	NEG	NEG	NEG
Russia-05_0078_Moscow	NEG	POS	POS	AMB	POS	AMB	POS	POS	NEG	NEG	POS	NEG	NEG	NEG
Russia-08_0135_Moscow	NEG	POS	POS	AMB	POS	AMB	POS	POS	NEG	NEG	POS	NEG	NEG	NEG
Russia-09_0150_Kurgan	NEG	POS	POS	AMB	POS	NEG	POS	POS	NEG	NEG	POS	NEG	NEG	NEG
Russia-10_0162_Kurgan	NEG	POS	POS	AMB	POS	NEG	POS	POS	NEG	NEG	POS	NEG	NEG	NEG
Russia-14_0184_Moscow	NEG	POS	POS	AMB	POS	NEG	POS	POS	NEG	NEG	POS	NEG	NEG	NEG
Russia-15_0232_SaintPetersburg	NEG	POS	POS	AMB	POS	NEG	POS	POS	NEG	NEG	POS	NEG	NEG	NEG
Russia-22_0391_Chelyabinsk	NEG	POS	POS	AMB	POS	NEG	POS	POS	NEG	NEG	POS	NEG	NEG	NEG
Russia-23_0392_Chelyabinsk	NEG	POS	POS	AMB	POS	NEG	POS	POS	NEG	NEG	POS	NEG	NEG	NEG
**>"Eurasian Clade" : IU17**														
IU17 (Turkey)	NEG	POS	POS	NEG	POS	NEG	POS	POS	NEG	NEG	POS	NEG	NEG	NEG
IU20 (Turkey)	NEG	POS	POS	NEG	POS	NEG	POS	POS	NEG	NEG	POS	NEG	NEG	NEG

STRAIN / ISOLATE	IMMUNOD.AG.B		DEFENSIN RESIST.		TRANSFERRIN BINDING PROT			PUTATIVE TRANSPORTER		TYPE I RESTR:-MODIFICATION SYSTEM				
	isaB		mprF		isdA			lmrP		hsdS1	hsdS2			
	isaB	isaB (MRSA252)	mprF (COL+MW2)	mprF (Mu50+252)	isdA (cons)	isdA (MRSA252)	isdA (Other Than MRSA252)	lmrP (OtherThanRF122)	lmrP (RF122)	hsdS1-RF122	hsdS2-ST5+ST8	hsdS2-MW2+476	hsdS2-RF122	hsdS2-MRSA252
	immunodominant antigen B		defensin resistance protein		transferrin-binding protein			hypothetical protein, similar to integral membrane protein LmrP		type I site-specific deoxyribo-nuclease subunit, 1st locus	type I site-specific deoxyribonuclease subunit, 2nd locus			
>"Eurasian Clade": Spora														
Dresden_16ANR581769	NEG	POS	POS	NEG	POS	NEG	POS	POS	NEG	NEG	POS	NEG	NEG	NEG
Dresden_17ANR578769	NEG	POS	POS	AMB	POS	NEG	POS	POS	NEG	NEG	POS	NEG	NEG	NEG
>"Eurasian Clade": Kuwa														
Kuwait_306	AMB	POS	POS	AMB	POS	AMB	POS	POS	NEG	NEG	POS	AMB	NEG	NEG
Kuwait_301	AMB	POS	POS	AMB	POS	AMB	POS	POS	NEG	NEG	POS	NEG	NEG	NEG
>"European Clade": "Gre														
GRE18 (Greece)	NEG	POS	POS	NEG	POS	NEG	POS	POS	NEG	NEG	NEG	NEG	NEG	NEG
GRE317 (Greece)	NEG	POS	POS	NEG	POS	NEG	POS	POS	NEG	NEG	NEG	NEG	NEG	NEG
GRE4 (Greece)	NEG	POS	POS	NEG	POS	NEG	POS	POS	NEG	NEG	NEG	NEG	NEG	NEG
R35 (USA)	NEG	POS	POS	NEG	POS	NEG	POS	POS	NEG	NEG	NEG	NEG	NEG	NEG
Casablanca_HT20060548	NEG	POS	POS	AMB	POS	AMB	POS	POS	NEG	NEG	POS	NEG	NEG	NEG
Casablanca_HT20060550	NEG	POS	POS	AMB	POS	AMB	POS	POS	NEG	NEG	POS	NEG	NEG	NEG
Dresden_01V34572 (Greece)	NEG	POS	POS	NEG	POS	NEG	POS	POS	NEG	NEG	NEG	NEG	NEG	NEG
Dresden_01V36469	NEG	POS	POS	NEG	POS	NEG	POS	POS	NEG	NEG	NEG	NEG	NEG	NEG
Dresden_01V39123	NEG	POS	POS	AMB	POS	AMB	POS	POS	NEG	NEG	NEG	POS	NEG	NEG
Dresden_01V39143	NEG	POS	POS	AMB	POS	NEG	POS	POS	NEG	NEG	NEG	NEG	NEG	NEG
Greece 1_3680_Harmony	NEG	POS	POS	AMB	POS	NEG	POS	POS	NEG	NEG	NEG	AMB	NEG	NEG
>"European Clade": UK-														
ANS46 (Australia)	NEG	POS	POS	NEG	POS	NEG	POS	POS	NEG	NEG	POS	NEG	NEG	NEG
LHH1 (USA)	NEG	POS	POS	NEG	POS	NEG	POS	POS	NEG	NEG	POS	NEG	NEG	NEG
Dublin-DSH_AR01_0-1085	NEG	POS	POS	AMB	POS	NEG	POS	POS	NEG	NEG	POS	NEG	NEG	NEG
Dublin-DSH_AR01_0-1118	NEG	POS	POS	NEG	POS	NEG	POS	POS	NEG	NEG	POS	NEG	NEG	NEG
Dublin-DSH_AR15_0098	NEG	POS	POS	AMB	POS	NEG	POS	POS	NEG	NEG	POS	NEG	NEG	NEG
Dublin-DSH_AR15_0104	NEG	POS	POS	AMB	POS	NEG	POS	POS	NEG	NEG	POS	NEG	NEG	NEG
UK_NCTC11939_UK-1_Harmony	NEG	POS	POS	AMB	POS	NEG	POS	POS	NEG	NEG	POS	NEG	NEG	NEG
>"European Clade": BK2														
BK2421 (USA)	NEG	POS	POS	NEG	POS	NEG	POS	POS	NEG	NEG	POS	NEG	NEG	NEG
>"Aussi/NZ Clade", JKD6														
JKD6009 (Australia)	NEG	POS	POS	NEG	POS	NEG	POS	POS	NEG	NEG	POS	NEG	NEG	NEG
JKD6008 (New Zealand)	NEG	POS	POS	NEG	POS	NEG	POS	POS	NEG	NEG	POS	NEG	NEG	NEG
Perth_2015-S20209	NEG	POS	POS	NEG	POS	NEG	POS	POS	NEG	NEG	POS	NEG	NEG	NEG
Perth_2015-S20305	NEG	POS	POS	NEG	POS	NEG	POS	POS	NEG	NEG	POS	NEG	NEG	NEG
Perth_2015-S20603	NEG	POS	POS	NEG	POS	NEG	POS	POS	NEG	NEG	POS	NEG	NEG	NEG
Perth_2015-S20607	NEG	POS	POS	NEG	POS	NEG	POS	POS	NEG	NEG	POS	NEG	NEG	NEG
Perth_2015-S20608	NEG	POS	POS	NEG	POS	NEG	POS	POS	NEG	NEG	POS	NEG	NEG	NEG
Perth_2015-S20610	NEG	POS	POS	NEG	POS	NEG	POS	POS	NEG	NEG	POS	NEG	NEG	NEG
Perth_2015-S20613	NEG	POS	POS	NEG	POS	NEG	POS	POS	NEG	NEG	POS	NEG	NEG	NEG
Perth_2015-S20617	NEG	POS	POS	NEG	POS	NEG	POS	POS	NEG	NEG	POS	NEG	NEG	NEG
Perth_2015-S20622	NEG	POS	POS	NEG	POS	NEG	POS	POS	NEG	NEG	POS	NEG	NEG	NEG
Perth_2015-S23619	NEG	POS	POS	NEG	POS	NEG	POS	POS	NEG	NEG	POS	NEG	NEG	NEG
Perth_2015-S41210	NEG	POS	POS	NEG	POS	NEG	POS	POS	NEG	NEG	POS	NEG	NEG	NEG
Perth_2015-S51001	NEG	POS	POS	NEG	POS	NEG	POS	POS	NEG	NEG	POS	NEG	NEG	NEG
Perth_2015-S51003	NEG	POS	POS	NEG	POS	NEG	POS	POS	NEG	NEG	POS	NEG	NEG	NEG
Perth_2015-S51006	NEG	POS	POS	NEG	POS	NEG	POS	POS	NEG	NEG	POS	NEG	NEG	NEG
Perth_2015-S51007	NEG	POS	POS	NEG	POS	NEG	POS	POS	NEG	NEG	POS	NEG	NEG	NEG
Perth_2015-S51008	NEG	POS	POS	NEG	POS	NEG	POS	POS	NEG	NEG	POS	NEG	NEG	NEG
Perth_2015-S51014	NEG	POS	POS	NEG	POS	NEG	POS	POS	NEG	NEG	POS	NEG	NEG	NEG
Perth_2015-S51408	NEG	POS	POS	NEG	POS	NEG	POS	POS	NEG	NEG	POS	NEG	NEG	NEG
Perth_2016-S20614	NEG	POS	POS	NEG	POS	NEG	POS	POS	NEG	NEG	POS	NEG	NEG	NEG
Perth_2016-S23614	NEG	POS	POS	NEG	POS	NEG	POS	POS	NEG	NEG	POS	NEG	NEG	NEG
Perth_2016-S42009	NEG	POS	POS	NEG	POS	NEG	POS	POS	NEG	NEG	POS	NEG	NEG	NEG
Perth_2016-S51001	NEG	POS	POS	NEG	POS	NEG	POS	POS	NEG	NEG	POS	NEG	NEG	NEG
Perth_2016-S51403	NEG	POS	POS	NEG	POS	NEG	POS	POS	NEG	NEG	POS	NEG	NEG	NEG
Perth_07 RPA 20	NEG	POS	POS	AMB	POS	NEG	POS	POS	NEG	NEG	POS	NEG	NEG	NEG
Perth_08-19088	NEG	POS	POS	AMB	POS	NEG	POS	POS	NEG	NEG	POS	POS	NEG	NEG
Perth_01-15351	NEG	POS	POS	AMB	POS	NEG	POS	POS	NEG	NEG	POS	NEG	NEG	NEG
Perth_01-15357	NEG	POS	POS	NEG	POS	NEG	POS	POS	NEG	NEG	POS	NEG	NEG	NEG
Perth_01-15419	NEG	POS	POS	AMB	POS	NEG	POS	POS	NEG	NEG	POS	NEG	NEG	NEG
Perth_01-16309	NEG	POS	POS	AMB	POS	NEG	POS	POS	NEG	NEG	POS	NEG	NEG	NEG
Perth_03 SNP 95	NEG	POS	POS	AMB	POS	AMB	POS	POS	NEG	NEG	POS	POS	NEG	NEG
Perth_2003_AH_47	NEG	POS	POS	AMB	POS	AMB	POS	POS	NEG	NEG	POS	NEG	NEG	NEG
>"Australian/NZ Clade":														
Perth_2015-S20623	NEG	POS	POS	NEG	POS	NEG	POS	POS	NEG	NEG	POS	NEG	NEG	NEG
Perth_2015-S20826	NEG	POS	POS	NEG	POS	NEG	POS	POS	NEG	NEG	POS	NEG	NEG	NEG
Perth_05 NH 56	NEG	POS	POS	AMB	POS	NEG	POS	POS	NEG	NEG	POS	AMB	NEG	NEG
>CC5-MRSA-III LA MRSA														
Delbrück 38 isolate from domestic turkey	POS	AMB	POS	AMB	POS	NEG	POS	POS	NEG	NEG	POS	NEG	NEG	NEG
> Staph. pseudintermed														
KM1381	NEG	NEG	NEG	NEG	NEG	NEG	NEG	NEG	NEG	NEG	NEG	NEG	NEG	NEG
>"South-East Asian Clad														
M92 (Canada)	NEG	POS	POS	NEG	POS	NEG	POS	POS	NEG	NEG	POS	NEG	NEG	NEG
CHI59 (China)	NEG	POS	POS	NEG	POS	NEG	POS	POS	NEG	NEG	POS	NEG	NEG	NEG
CHI61 (China)	NEG	POS	POS	NEG	POS	NEG	POS	POS	NEG	NEG	POS	NEG	NEG	NEG
CUHK_HK1997 (China)	NEG	POS	POS	NEG	POS	NEG	POS	POS	NEG	NEG	POS	NEG	NEG	NEG
M996 (China)	NEG	POS	POS	NEG	POS	NEG	POS	POS	NEG	NEG	POS	NEG	NEG	NEG
H211 (Denmark)	NEG	POS	POS	NEG	POS	NEG	POS	POS	NEG	NEG	POS	NEG	NEG	NEG
H216 (Denmark)	NEG	POS	POS	NEG	POS	NEG	POS	POS	NEG	NEG	POS	NEG	NEG	NEG
D71 (Germany)	NEG	POS	POS	NEG	POS	NEG	POS	POS	NEG	NEG	POS	NEG	NEG	NEG
NMR05 (India)	NEG	POS	POS	NEG	POS	NEG	POS	POS	NEG	NEG	POS	NEG	NEG	NEG
MAL11 (Malaysia)	NEG	POS	POS	NEG	POS	NEG	POS	POS	NEG	NEG	POS	NEG	NEG	NEG
MAL9 (Malaysia)	NEG	POS	POS	NEG	POS	NEG	POS	POS	NEG	NEG	POS	NEG	NEG	NEG
Na21 (Sri Lanka)	NEG	POS	POS	NEG	POS	NEG	POS	POS	NEG	NEG	POS	NEG	NEG	NEG
M592 (Syria)	NEG	POS	POS	NEG	POS	NEG	POS	POS	NEG	NEG	POS	NEG	NEG	NEG
H202 (Thailand)	NEG	POS	POS	NEG	POS	NEG	POS	POS	NEG	NEG	POS	NEG	NEG	NEG
S102 (Thailand)	NEG	POS	POS	NEG	POS	NEG	POS	POS	NEG	NEG	POS	NEG	NEG	NEG
S130 (Thailand)	NEG	POS	POS	NEG	POS	NEG	POS	POS	NEG	NEG	POS	NEG	NEG	NEG
S40 (Thailand)	NEG	POS	POS	NEG	POS	NEG	POS	POS	NEG	NEG	POS	NEG	NEG	NEG
S71 (Thailand)	NEG	POS	POS	NEG	POS	NEG	POS	POS	NEG	NEG	POS	NEG	NEG	NEG
S87 (Thailand)	NEG	POS	POS	NEG	POS	NEG	POS	POS	NEG	NEG	POS	NEG	NEG	NEG
S93 (Thailand)	NEG	POS	POS	NEG	POS	NEG	POS	POS	NEG	NEG	POS	NEG	NEG	NEG
US_002 (Thailand)	NEG	POS	POS	NEG	POS	NEG	POS	POS	NEG	NEG	POS	NEG	NEG	NEG
US_017 (Thailand)	NEG	POS	POS	NEG	POS	NEG	POS	POS	NEG	NEG	POS	NEG	NEG	NEG
WAMC6102 (Thailand)	NEG	POS	POS	NEG	POS	NEG	POS	POS	NEG	NEG	POS	NEG	NEG	NEG
NCTC_13626_TW20 (UK)	NEG	POS	POS	NEG	POS	NEG	POS	POS	NEG	NEG	POS	NEG	NEG	NEG
BSAC3021 (UK)	NEG	POS	POS	NEG	POS	NEG	POS	POS	NEG	NEG	POS	NEG	NEG	NEG
BSAC3046 (UK)	NEG	POS	POS	NEG	POS	NEG	POS	POS	NEG	NEG	POS	NEG	NEG	NEG
1193_SAUR (USA)	NEG	POS	POS	NEG	POS	NEG	POS	POS	NEG	NEG	POS	NEG	NEG	NEG
1194_SAUR (USA)	NEG	POS	POS	NEG	POS	NEG	POS	POS	NEG	NEG	POS	NEG	NEG	NEG

STRAIN / ISOLATE	IMMUNOD.AG.B		DEFENSIN RESIST.		TRANSFERRIN BINDING PROT			PUTATIVE TRANSPORTER		TYPE I RESTR:-MODIFICATION SYSTEM				
	isaB		mprF		isdA			lmrP		hsdS1	hsdS2			
	isaB	isaB (MRSA252)	mprF (COL+MW2)	mprF (Mu50+252)	isdA (cons)	isdA (MRSA252)	isdA (Other Than MRSA252)	lmrP (OtherThanRF122)	lmrP (RF122)	hsdS1-RF122	hsdS2-ST5+ST8	hsdS2-MW2+476	hsdS2-RF122	hsdS2-MRSA252
	immunodominant antigen B		defensin resistance protein		transferrin-binding protein			hypothetical protein, similar to integral membrane protein LmrP		type I site-specific deoxyribo-nuclease subunit, 1st locus	type I site-specific deoxyribonuclease subunit, 2nd locus			
>"South-East Asian Clade": TW20 (Irish AR44), c														
1195_SAUR (USA)	NEG	POS	POS	NEG	POS	NEG	POS	POS	NEG	NEG	POS	NEG	NEG	NEG
FVRH6002 (USA)	NEG	POS	POS	NEG	POS	NEG	POS	POS	NEG	NEG	POS	NEG	NEG	NEG
KINW6048 (USA)	NEG	POS	POS	NEG	POS	NEG	POS	POS	NEG	NEG	POS	NEG	NEG	NEG
LAMC0011 (USA)	NEG	POS	POS	NEG	POS	NEG	POS	POS	NEG	NEG	POS	NEG	NEG	NEG
SJOS6053 (USA)	NEG	POS	POS	NEG	POS	NEG	POS	POS	NEG	NEG	POS	NEG	NEG	NEG
SJOS6072 (USA)	NEG	POS	POS	NEG	POS	NEG	POS	POS	NEG	NEG	POS	NEG	NEG	NEG
UCIM6042 (USA)	NEG	POS	POS	NEG	POS	NEG	POS	POS	NEG	NEG	POS	NEG	NEG	NEG
Perth_2015-S31902	NEG	POS	NEG	NEG	POS	NEG	POS	POS	NEG	NEG	POS	NEG	NEG	NEG
Perth_2015-S31904	NEG	POS	POS	NEG	POS	NEG	POS	POS	NEG	NEG	POS	NEG	NEG	NEG
Perth_2015-S31913	NEG	POS	POS	NEG	POS	NEG	POS	POS	NEG	NEG	POS	NEG	NEG	NEG
Perth_2015-S31918	NEG	POS	POS	NEG	POS	NEG	POS	POS	NEG	NEG	NEG	NEG	NEG	NEG
Dublin-DSH_AR44_E1183	NEG	POS	POS	AMB	POS	NEG	POS	POS	NEG	NEG	POS	NEG	NEG	NEG
Dublin-DSH_Unfamiliar-3_E1520	NEG	POS	POS	AMB	POS	NEG	POS	POS	NEG	NEG	POS	NEG	NEG	NEG
Hong Kong_042	NEG	POS	POS	AMB	POS	NEG	POS	POS	NEG	NEG	POS	NEG	NEG	NEG
Hong Kong_101	NEG	POS	POS	AMB	POS	NEG	POS	POS	NEG	NEG	POS	NEG	NEG	NEG
Hong Kong_111	AMB	POS	POS	AMB	POS	AMB	POS	POS	NEG	NEG	POS	POS	NEG	NEG
Hong Kong_113	NEG	POS	POS	AMB	POS	NEG	POS	POS	NEG	NEG	POS	NEG	NEG	NEG
Hong Kong_115	NEG	POS	POS	AMB	POS	NEG	POS	POS	NEG	NEG	POS	NEG	NEG	NEG
Hong Kong_118	NEG	POS	POS	AMB	POS	NEG	POS	POS	NEG	NEG	POS	NEG	NEG	NEG
Hong Kong_125	NEG	POS	POS	AMB	POS	NEG	POS	POS	NEG	NEG	POS	NEG	NEG	NEG
Hong Kong_129	NEG	POS	POS	AMB	POS	NEG	POS	POS	NEG	NEG	POS	NEG	NEG	NEG
Hong Kong_133	NEG	POS	POS	AMB	POS	NEG	POS	NEG	NEG	NEG	POS	NEG	NEG	NEG
Kuwait_009	NEG	POS	POS	AMB	POS	NEG	POS	POS	NEG	NEG	POS	NEG	NEG	NEG
Kuwait_103	AMB	POS	POS	AMB	POS	AMB	POS	POS	NEG	NEG	POS	NEG	NEG	NEG
Kuwait_107	AMB	POS	POS	AMB	POS	AMB	POS	POS	NEG	NEG	POS	NEG	NEG	NEG
Perth_03 AH 88	NEG	POS	POS	AMB	POS	NEG	POS	POS	NEG	NEG	POS	NEG	NEG	NEG
Perth_08-16905	NEG	POS	POS	AMB	POS	NEG	POS	POS	NEG	NEG	POS	NEG	NEG	NEG
Riyadh_Alfaisal-10_SICU_13_B5672_1117353	NEG	POS	POS	AMB	POS	AMB	POS	POS	NEG	NEG	POS	NEG	NEG	NEG
Riyadh_SSTI_17_3508602	NEG	POS	POS	AMB	POS	NEG	POS	POS	NEG	NEG	POS	AMB	NEG	NEG
Riyadh-2822088-R	NEG	POS	POS	AMB	POS	AMB	POS	POS	NEG	NEG	POS	AMB	NEG	NEG
Trinidad&Tobago_2012_195 (619/12)	NEG	POS	AMB	POS	POS	AMB	POS	POS	NEG	NEG	POS	NEG	NEG	NEG
Trinidad&Tobago_2012_370 (1112-52177)	NEG	POS	AMB	POS	POS	AMB	POS	POS	NEG	NEG	POS	NEG	NEG	NEG
Trinidad&Tobago_2012_378 (4139)	NEG	POS	AMB	POS	POS	AMB	POS	POS	NEG	NEG	POS	NEG	NEG	NEG
Trinidad&Tobago_2013_PA21	NEG	POS	POS	AMB	POS	NEG	POS	POS	NEG	NEG	POS	NEG	NEG	NEG
Trinidad&Tobago_2013_PA22	AMB	POS	AMB	POS	POS	AMB	POS	POS	NEG	NEG	POS	NEG	NEG	NEG
Trinidad&Tobago_2912_340 (8523/12)	NEG	POS	POS	AMB	POS	AMB	POS	POS	NEG	NEG	POS	NEG	NEG	NEG
Trinidad&Tobago_MRSA2010_105	NEG	POS	POS	AMB	POS	NEG	POS	POS	NEG	NEG	POS	NEG	NEG	NEG
Trinidad&Tobago_MRSA2010_108	AMB	POS	POS	AMB	POS	AMB	POS	POS	NEG	NEG	POS	NEG	NEG	NEG
Trinidad&Tobago_MRSA2010_33	NEG	POS	POS	AMB	POS	NEG	POS	POS	NEG	NEG	POS	NEG	NEG	NEG
Trinidad&Tobago_MRSA2010_79	NEG	POS	POS	AMB	POS	NEG	POS	POS	NEG	NEG	POS	NEG	NEG	NEG
Trinidad&Tobago_MRSA2010_88	NEG	POS	POS	AMB	POS	NEG	POS	POS	NEG	NEG	POS	NEG	NEG	NEG
Trinidad&Tobago_MRSA2010_97	NEG	POS	POS	AMB	POS	NEG	POS	POS	NEG	NEG	POS	NEG	NEG	NEG
Trinidad&Tobago_SSTI 2012_031	NEG	POS	POS	AMB	POS	AMB	POS	POS	NEG	NEG	POS	AMB	NEG	NEG
Uganda-29_17148_250811	NEG	POS	POS	AMB	POS	NEG	POS	POS	NEG	NEG	POS	AMB	NEG	NEG
>"South-East Asian Clad														
UCIM6015	NEG	POS	POS	NEG	POS	NEG	POS	POS	NEG	NEG	POS	NEG	NEG	NEG
Hong Kong_121	NEG	POS	POS	AMB	POS	NEG	POS	POS	NEG	NEG	POS	NEG	NEG	NEG
>"South-East Asian Clad														
Z172 (Taiwan)	NEG	POS	POS	NEG	POS	NEG	POS	POS	NEG	NEG	POS	NEG	NEG	NEG
Russia-19_0342_Kurgan	NEG	POS	POS	AMB	POS	NEG	POS	POS	NEG	NEG	POS	NEG	NEG	NEG
>"South-East Asian Clad														
Perth_01-15392	NEG	POS	POS	AMB	POS	NEG	POS	POS	NEG	NEG	POS	NEG	NEG	NEG
>"South-East Asian Clad														
XN108 (China)	NEG	POS	POS	NEG	POS	NEG	POS	POS	NEG	NEG	POS	NEG	NEG	NEG
NMR09 (India)	NEG	POS	POS	NEG	POS	NEG	POS	POS	NEG	NEG	POS	NEG	NEG	NEG
346 (Malaysia)	NEG	POS	POS	NEG	POS	NEG	POS	POS	NEG	NEG	POS	NEG	NEG	NEG
DS_009 (Thailand)	NEG	POS	POS	NEG	POS	NEG	POS	POS	NEG	NEG	POS	NEG	NEG	NEG
18ANR581005 (Ind)	NEG	POS	POS	AMB	POS	NEG	POS	POS	NEG	NEG	POS	NEG	NEG	NEG
Bengalaru_ST20141405	NEG	POS	POS	AMB	POS	AMB	POS	POS	NEG	NEG	POS	NEG	NEG	NEG
Bengalaru_ST20141406	NEG	POS	POS	AMB	POS	AMB	POS	POS	NEG	NEG	POS	NEG	NEG	NEG
Bengalaru_ST20141417	NEG	POS	POS	AMB	POS	NEG	POS	POS	NEG	NEG	POS	NEG	NEG	NEG
Hong Kong_107			POS	AMB	POS	NEG	POS	POS	NEG	NEG	POS	NEG	NEG	NEG
Hong Kong_12	NEG	POS	POS	AMB	POS	AMB	POS	POS	NEG	NEG	POS	AMB	NEG	NEG
Hong Kong_132	NEG	POS	POS	AMB	POS	NEG	POS	POS	NEG	NEG	POS	NEG	NEG	NEG
Riyadh-3028763-R	NEG	POS	POS	AMB	POS	NEG	POS	POS	NEG	NEG	POS	AMB	NEG	NEG
Trinidad&Tobago_MRSA2010_94	NEG	POS	POS	AMB	POS	NEG	POS	POS	NEG	NEG	POS	NEG	NEG	NEG
>"South-East Asian Clad														
M705 (Thailand)	NEG	POS	POS	NEG	POS	NEG	POS	POS	NEG	NEG	POS	NEG	NEG	NEG
S85 (Thailand)	NEG	POS	POS	NEG	POS	NEG	POS	POS	NEG	NEG	POS	NEG	NEG	NEG
Riyadh_Alfaisal-04_281014_S5076	AMB	POS	POS	AMB	POS	AMB	POS	POS	NEG	NEG	POS	POS	NEG	NEG
Riyadh_Alfaisal-26_S15145_1104163	NEG	POS	POS	AMB	POS	AMB	POS	POS	NEG	NEG	POS	NEG	NEG	NEG
Riyadh-2817437-W	AMB	POS	POS	AMB	POS	AMB	POS	POS	NEG	NEG	POS	POS	NEG	NEG
>"South-East Asian Clad														
PPUKM-775-2009 (Malaysia)	NEG	POS	POS	NEG	POS	NEG	POS	POS	NEG	NEG	POS	NEG	NEG	NEG
Hong Kong_138	NEG	POS	POS	AMB	POS	NEG	POS	POS	NEG	NEG	POS	NEG	NEG	NEG
>"South-East Asian Clad														
Kuwait_001	NEG	POS	POS	AMB	POS	NEG	POS	POS	NEG	NEG	POS	NEG	NEG	NEG
Kuwait_337	NEG	POS	POS	AMB	POS	AMB	POS	POS	NEG	NEG	POS	NEG	NEG	NEG
>"South-East Asian Clad														
CUHK_HK2007 (China)	NEG	POS	POS	NEG	POS	NEG	POS	POS	NEG	NEG	POS	NEG	NEG	NEG
NMR02 (India)	NEG	POS	POS	NEG	POS	NEG	POS	POS	NEG	NEG	POS	NEG	NEG	NEG
VB1490 (India)	NEG	POS	POS	NEG	POS	NEG	POS	POS	NEG	NEG	NEG	NEG	NEG	NEG
V521 (Korea)	NEG	POS	POS	NEG	POS	NEG	POS	POS	NEG	NEG	POS	NEG	NEG	NEG
HST-077 (Lebanon)	NEG	POS	POS	NEG	POS	NEG	POS	POS	NEG	NEG	POS	NEG	NEG	NEG
MAL1 (Malaysia)	NEG	POS	POS	NEG	POS	NEG	POS	POS	NEG	NEG	POS	NEG	NEG	NEG
MAL3 (Malaysia)	NEG	POS	POS	NEG	POS	NEG	POS	POS	NEG	NEG	POS	NEG	NEG	NEG
PPUKM-261-2009 (Malaysia)	NEG	POS	POS	NEG	POS	NEG	POS	POS	NEG	NEG	POS	NEG	NEG	NEG
PPUKM-332-2009 (Malaysia)	NEG	POS	POS	NEG	POS	NEG	POS	POS	NEG	NEG	POS	NEG	NEG	NEG
S38 (Thailand)	NEG	POS	POS	NEG	POS	NEG	POS	POS	NEG	NEG	POS	NEG	NEG	NEG
S7 (Thailand)	NEG	POS	POS	NEG	POS	NEG	POS	POS	NEG	NEG	POS	NEG	NEG	NEG
US_008 (Thailand)	NEG	POS	POS	NEG	POS	NEG	POS	POS	NEG	NEG	POS	NEG	NEG	NEG
US_030 (Thailand)	NEG	POS	POS	NEG	POS	NEG	POS	POS	NEG	NEG	POS	NEG	NEG	NEG
M170 (Tsunami-related)	NEG	POS	POS	NEG	POS	NEG	POS	POS	NEG	NEG	POS	NEG	NEG	NEG
BSAC697 (UK)	NEG	POS	POS	NEG	POS	NEG	POS	POS	NEG	NEG	POS	NEG	NEG	NEG
IS-189	NEG	POS	POS	NEG	POS	NEG	POS	POS	NEG	NEG	POS	NEG	NEG	NEG
MRGR3	NEG	POS	POS	NEG	POS	NEG	POS	POS	NEG	NEG	NEG	NEG	NEG	NEG
smg07	NEG	POS	POS	NEG	POS	NEG	POS	POS	NEG	NEG	POS	NEG	NEG	NEG
AH1-AUS-EMRSA-3	NEG	POS	POS	AMB	POS	NEG	POS	POS	NEG	NEG	POS	NEG	NEG	NEG
Bengalaru_ST20141403	NEG	POS	POS	AMB	POS	AMB	POS	POS	NEG	NEG	POS	NEG	NEG	NEG
Finland E24_98541_Harmony	NEG	POS	AMB	POS	POS	NEG	POS	POS	NEG	NEG	POS	NEG	NEG	NEG
Hong Kong_10	AMB	POS	POS	AMB	POS	AMB	POS	POS	NEG	NEG	POS	POS	NEG	NEG
Hong Kong_100	NEG	POS	POS	AMB	POS	NEG	POS	POS	NEG	NEG	POS		NEG	NEG
Hong Kong_199	AMB	POS	POS	AMB	POS	AMB	POS	POS	NEG	NEG	POS	POS	NEG	NEG
Hong Kong_34	AMB	POS	POS	AMB	POS	AMB	POS	POS	NEG	NEG	POS	POS	NEG	NEG
Hong Kong_88	AMB	POS	POS	AMB	POS	AMB	POS	POS	NEG	NEG	POS	POS	NEG	NEG
Hong Kong_92	NEG	POS	POS	AMB	POS	AMB	POS	POS	NEG	NEG	POS	AMB	NEG	NEG
Hong Kong_94	NEG	POS	POS	AMB	POS	NEG	POS	POS	NEG	NEG	POS	NEG	NEG	NEG
Kuwait_257	NEG	POS	POS	AMB	POS	NEG	POS	POS	NEG	NEG	POS	NEG	NEG	NEG
Perth_01-15330	NEG	POS	POS	NEG	POS	NEG	POS	POS	NEG	NEG	POS	NEG	NEG	NEG
Perth_01-15349	NEG	POS	POS	AMB	POS	NEG	POS	POS	NEG	NEG	POS	NEG	NEG	NEG
Perth_01-16176	NEG	POS	POS	AMB	POS	NEG	POS	POS	NEG	NEG	POS	NEG	NEG	NEG
Perth_WBG8379	NEG	POS	POS	AMB	POS	NEG	POS	POS	NEG	NEG	POS	NEG	NEG	NEG
Rawalpindi_Kidney Center_56	NEG	POS	POS	POS	POS	AMB	POS	POS	NEG	AMB	POS	NEG	NEG	NEG
Riyadh_SSTI_05_3464614	NEG	POS	POS	AMB	POS	NEG	POS	POS	NEG	NEG	POS	NEG	NEG	NEG
Trinidad&Tobago_2012_157	NEG	POS	POS	AMB	POS	AMB	POS	POS	NEG	NEG	POS	NEG	NEG	NEG

STRAIN / ISOLATE	IMMUNOD.AG.B		DEFENSIN RESIST.		TRANSFERRIN BINDING PROT			PUTATIVE TRANSPORTER		TYPE I RESTR:-MODIFICATION SYSTEM				
	isaB		mprF		isdA			lmrP		hsdS1	hsdS2			
	isaB	isaB (MRSA252)	mprF (COL+MW2)	mprF (Mu50+252)	isdA (cons)	isdA (MRSA252)	isdA (Other Than MRSA252)	lmrP (OtherThanRF122)	lmrP (RF122)	hsdS1-RF122	hsdS2-ST5+ST8	hsdS2-MW2+476	hsdS2-RF122	hsdS2-MRSA252
	immunodominant antigen B		defensin resistance protein		transferrin-binding protein			hypothetical protein, similar to integral membrane protein LmrP		type I site-specific deoxyribo-nuclease subunit, 1st locus	type I site-specific deoxyribonuclease subunit, 2nd locus			
>"South-East Asian Clad														
M418	NEG	POS	POS	NEG	POS	NEG	POS	POS	NEG	NEG	POS	NEG	NEG	NEG
>"South-East Asian Clad														
NMR07 (India)	NEG	POS	POS	NEG	POS	NEG	POS	POS	NEG	NEG	POS	NEG	NEG	NEG
NMR08 (India)	NEG	POS	POS	NEG	POS	NEG	POS	POS	NEG	NEG	POS	NEG	NEG	NEG
Bengalaru_ST20121978	AMB	POS	POS	AMB	POS	AMB	POS	POS	NEG	NEG	POS	NEG	NEG	NEG
Hong Kong_89	AMB	POS	POS	AMB	POS	AMB	POS	POS	NEG	NEG	POS	POS	NEG	NEG
>"South-East Asian Clad														
Perth_2005_AH_5	NEG	POS	POS	AMB	POS	AMB	POS	POS	NEG	NEG	POS	NEG	NEG	NEG
>"South-East Asian Clad														
Trinidad&Tobago_MRSA2010_103	NEG	POS	POS	AMB	POS	NEG	POS	POS	NEG	NEG	POS	NEG	NEG	NEG
Trinidad&Tobago_2013_PA25	NEG	POS	POS	AMB	POS	NEG	POS	POS	NEG	NEG	POS	NEG	NEG	NEG
Trinidad&Tobago_MRSA2010_110	NEG	POS	POS	AMB	POS	NEG	POS	POS	NEG	NEG	POS	NEG	NEG	NEG
Trinidad&Tobago_SSTI 2012_111	NEG	POS	POS	POS	POS	NEG	POS	POS	NEG	NEG	POS	NEG	NEG	NEG
Trinidad&Tobago_MRSA2010_158	NEG	POS	POS	AMB	POS	NEG	POS	POS	NEG	NEG	POS	NEG	NEG	NEG
>"South-East Asian Clad														
Kuwait_122	NEG	POS	POS	AMB	POS	NEG	POS	POS	NEG	NEG	POS	NEG	NEG	NEG
Kuwait_002	NEG	POS	POS	AMB	POS	NEG	POS	POS	NEG	NEG	POS	NEG	NEG	NEG
Kuwait_196	NEG	POS	POS	AMB	POS	NEG	POS	POS	NEG	NEG	POS	NEG	NEG	NEG
Kuwait_199	NEG	POS	POS	POS	POS	NEG	POS	POS	NEG	NEG	POS	NEG	NEG	NEG
Hong Kong_136	NEG	POS	POS	AMB	POS	NEG	POS	POS	NEG	NEG	POS	NEG	NEG	NEG
>"South-East Asian Clad														
P32 (Poland)	NEG	POS	POS	NEG	POS	NEG	POS	POS	NEG	NEG	POS	NEG	NEG	NEG
>"South-East Asian Clad														
Perth_2015-S32313	NEG	POS	POS	NEG	POS	NEG	POS	POS	NEG	NEG	POS	NEG	NEG	NEG
>"hla-negative South-Ea														
DEN907 (Denmark)	NEG	POS	POS	NEG	POS	NEG	POS	POS	NEG	NEG	POS	NEG	NEG	NEG
>"hla-negative South-Ea														
S26 (Thailand)	NEG	POS	POS	NEG	POS	NEG	POS	POS	NEG	NEG	POS	NEG	NEG	NEG
S42 (Thailand)	NEG	POS	POS	NEG	POS	NEG	POS	POS	NEG	NEG	POS	NEG	NEG	NEG
S97 (Thailand)	NEG	POS	POS	NEG	POS	NEG	POS	POS	NEG	NEG	POS	NEG	NEG	NEG
S78 (Thailand)	NEG	POS	POS	NEG	POS	NEG	POS	POS	NEG	NEG	POS	NEG	NEG	NEG
S25 (Thailand)	NEG	POS	POS	NEG	POS	NEG	POS	POS	NEG	NEG	POS	NEG	NEG	NEG
S21 (Thailand)	NEG	POS	POS	NEG	POS	NEG	POS	POS	NEG	NEG	POS	NEG	NEG	NEG
S106 (Thailand)	NEG	POS	POS	NEG	POS	NEG	POS	POS	NEG	NEG	POS	NEG	NEG	NEG
S24 (Thailand)	NEG	POS	POS	NEG	POS	NEG	POS	POS	NEG	NEG	POS	NEG	NEG	NEG
S39 (Thailand)	NEG	POS	POS	NEG	POS	NEG	POS	POS	NEG	NEG	POS	NEG	NEG	NEG
S2 (Thailand)	NEG	POS	POS	NEG	POS	NEG	POS	POS	NEG	NEG	POS	NEG	NEG	NEG
S81 (Thailand)	NEG	POS	POS	NEG	POS	NEG	POS	POS	NEG	NEG	POS	NEG	NEG	NEG
M116 (Vietnam)	NEG	POS	POS	NEG	POS	NEG	POS	POS	NEG	NEG	POS	NEG	NEG	NEG
>"Portuguese Clade": Pc														
FFP103 (Portugal)	NEG	POS	POS	NEG	POS	NEG	POS	POS	NEG	NEG	POS	NEG	NEG	NEG
HDG2 (Portugal)	NEG	POS	POS	NEG	POS	NEG	POS	POS	NEG	NEG	POS	NEG	NEG	NEG
HSA10 (Portugal)	NEG	POS	POS	NEG	POS	NEG	POS	POS	NEG	NEG	POS	NEG	NEG	NEG
HSA11 (Portugal)	NEG	POS	POS	NEG	POS	NEG	POS	POS	NEG	NEG	POS	NEG	NEG	NEG
ICP5011 (Portugal)	NEG	POS	POS	NEG	POS	NEG	POS	POS	NEG	NEG	POS	NEG	NEG	NEG
ICP5014 (Portugal)	NEG	POS	POS	NEG	POS	NEG	POS	POS	NEG	NEG	POS	NEG	NEG	NEG
ICP5062 (Portugal)	NEG	POS	POS	NEG	POS	NEG	POS	POS	NEG	NEG	POS	NEG	NEG	NEG
Strain 1063_ATCC 33592 FDAARGOS_3 (USA)	NEG	POS	POS	NEG	POS	NEG	POS	POS	NEG	NEG	POS	NEG	NEG	NEG
	NEG	POS	POS	AMB	POS	NEG	POS	POS	NEG	NEG	POS	NEG	NEG	NEG
Russia-07_0110_Kurgan	NEG	POS	POS	AMB	POS	NEG	POS	POS	NEG	NEG	POS	NEG	NEG	NEG
Russia-11_0164_Kurgan	NEG	POS	POS	AMB	POS	NEG	POS	POS	NEG	NEG	POS	NEG	NEG	NEG
Russia-20_0367_Kurgan	NEG	POS	POS	AMB	POS	NEG	POS	POS	NEG	NEG	POS	NEG	NEG	NEG
Russia-21_0390_Chelyabinsk	NEG	POS	POS	AMB	POS	NEG	POS	POS	NEG	NEG	POS	NEG	NEG	NEG
>"South American/ Mid														
AGT1 (Argentina)	NEG	POS	POS	NEG	POS	NEG	POS	POS	NEG	NEG	POS	NEG	NEG	NEG
AGT120 (Argentina)	NEG	POS	POS	NEG	POS	NEG	POS	POS	NEG	NEG	POS	NEG	NEG	NEG
AGT67 (Argentina)	NEG	POS	POS	NEG	POS	NEG	POS	POS	NEG	NEG	NEG	NEG	NEG	NEG
AGT9 (Argentina)	NEG	POS	POS	NEG	POS	NEG	POS	POS	NEG	NEG	POS	NEG	NEG	NEG
RA3 (Argentina)	NEG	POS	POS	NEG	POS	NEG	POS	POS	NEG	NEG	POS	NEG	NEG	NEG
RA6 (Argentina)	NEG	POS	POS	NEG	POS	NEG	POS	POS	NEG	NEG	POS	NEG	NEG	NEG
RA7 (Argentina)	NEG	POS	POS	NEG	POS	NEG	POS	POS	NEG	NEG	POS	NEG	NEG	NEG
Be62 (Brazil)	NEG	POS	POS	NEG	POS	NEG	POS	POS	NEG	NEG	POS	NEG	NEG	NEG
Bmb9393 (Brazil)	NEG	POS	POS	NEG	POS	NEG	POS	POS	NEG	NEG	POS	NEG	NEG	NEG
BRA36 (Brazil)	NEG	POS	POS	NEG	POS	NEG	POS	POS	NEG	NEG	POS	NEG	NEG	NEG
BZ48 (Brazil)	NEG	POS	POS	NEG	POS	NEG	POS	POS	NEG	NEG	POS	NEG	NEG	NEG
Gv51 (Brazil)	NEG	POS	POS	NEG	POS	NEG	POS	POS	NEG	NEG	POS	NEG	NEG	NEG
Gv69 (Brazil)	NEG	POS	POS	NEG	POS	NEG	POS	POS	NEG	NEG	POS	NEG	NEG	NEG
Gv88 (Brazil)	NEG	POS	POS	NEG	POS	NEG	POS	POS	NEG	NEG	POS	NEG	NEG	NEG
HC1335 (Brazil)	NEG	POS	POS	NEG	POS	NEG	POS	POS	NEG	NEG	POS	NEG	NEG	NEG
HC1340 (Brazil)	NEG	POS	POS	NEG	POS	NEG	POS	POS	NEG	NEG	POS	NEG	NEG	NEG
HC556 (Brazil)	NEG	POS	POS	NEG	POS	NEG	POS	POS	NEG	NEG	POS	NEG	NEG	NEG
HU25 (Brazil)	NEG	POS	POS	NEG	POS	NEG	POS	POS	NEG	NEG	POS	NEG	NEG	NEG
UB563 (Brazil)	NEG	POS	POS	NEG	POS	NEG	POS	POS	NEG	NEG	POS	NEG	NEG	NEG
2A8 (Czech Republic)	NEG	POS	POS	NEG	POS	NEG	POS	POS	NEG	NEG	POS	NEG	NEG	NEG
M1229 (Denmark)	NEG	POS	POS	NEG	POS	NEG	POS	POS	NEG	NEG	POS	NEG	NEG	NEG
FRICAR (France)	NEG	POS	POS	NEG	POS	NEG	POS	POS	NEG	NEG	POS	NEG	NEG	NEG
D90 (Germany)	NEG	POS	POS	NEG	POS	NEG	POS	POS	NEG	NEG	POS	NEG	NEG	NEG
LIT68 (Lithuania)	NEG	POS	POS	NEG	POS	NEG	POS	POS	NEG	NEG	POS	NEG	NEG	NEG
LIT76 (Lithuania)	NEG	POS	POS	NEG	POS	NEG	POS	POS	NEG	NEG	POS	NEG	NEG	NEG
HGSA142 (Portugal)	NEG	POS	POS	NEG	POS	NEG	POS	POS	NEG	NEG	POS	NEG	NEG	NEG
HGSA9 (Portugal)	NEG	POS	POS	NEG	POS	NEG	POS	POS	NEG	NEG	POS	NEG	NEG	NEG
HSJ216 (Portugal)	NEG	POS	POS	NEG	POS	NEG	POS	POS	NEG	NEG	POS	NEG	NEG	NEG
M278 (Portugal)	NEG	POS	POS	NEG	POS	NEG	POS	POS	NEG	NEG	POS	NEG	NEG	NEG
ES26 (Spain)	NEG	POS	POS	NEG	POS	NEG	POS	POS	NEG	NEG	POS	NEG	NEG	NEG
UK102 (UK)	NEG	POS	POS	NEG	POS	NEG	POS	POS	NEG	NEG	POS	NEG	NEG	NEG
URU34 (Uruguay)	NEG	POS	POS	NEG	POS	NEG	POS	POS	NEG	NEG	POS	NEG	NEG	NEG
IS-125	NEG	POS	POS	NEG	POS	NEG	POS	POS	NEG	NEG	POS	NEG	NEG	NEG
IS-157	NEG	POS	POS	NEG	POS	NEG	POS	POS	NEG	NEG	POS	NEG	NEG	NEG
Ecuador_1	NEG	POS	POS	AMB	POS	NEG	POS	POS	NEG	NEG	POS	POS	NEG	NEG
Ecuador_2	NEG	POS	POS	AMB	POS	NEG	POS	POS	NEG	NEG	POS	NEG	NEG	NEG
Dresden_15ANR574263	NEG	POS	POS	AMB	POS	AMB	POS	POS	NEG	NEG	POS	NEG	NEG	NEG
UK-EMRSA-11	NEG	POS	POS	AMB	POS	NEG	POS	POS	NEG	NEG	POS	NEG	NEG	NEG
>"South American/ Mid														
BRA2 (Brazil)	NEG	POS	POS	NEG	POS	NEG	POS	POS	NEG	NEG	POS	NEG	NEG	NEG
CHL1 (Chile)	NEG	POS	POS	NEG	POS	NEG	POS	POS	NEG	NEG	POS	NEG	NEG	NEG
CHL151 (Chile)	NEG	POS	POS	NEG	POS	NEG	POS	POS	NEG	NEG	POS	NEG	NEG	NEG
UCL378 (Chile)	NEG	POS	POS	NEG	POS	NEG	POS	POS	NEG	NEG	POS	NEG	NEG	NEG
UP72 (Peru)	NEG	POS	POS	NEG	POS	NEG	POS	POS	NEG	NEG	POS	NEG	NEG	NEG
UP81 (Peru)	NEG	POS	POS	NEG	POS	NEG	POS	POS	NEG	NEG	POS	NEG	NEG	NEG
>"South American/ Mid														
UP1073 (Peru)	NEG	POS	POS	NEG	POS	NEG	POS	POS	NEG	NEG	POS	NEG	NEG	NEG

STRAIN / ISOLATE	IMMUNOD.AG.B		DEFENSIN RESIST.		TRANSFERRIN BINDING PROT			PUTATIVE TRANSPORTER		TYPE I RESTR:-MODIFICATION SYSTEM				
	isaB		mprF		isdA			lmrP		hsdS1	hsdS2			
	isaB	isaB (MRSA252)	mprF (COL+MW2)	mprF (Mu50+252)	isdA (cons)	isdA (MRSA252)	isdA (Other Than MRSA252)	lmrP (OtherThanRF122)	lmrP (RF122)	hsdS1-RF122	hsdS2-ST5+ST8	hsdS2-MW2+476	hsdS2-RF122	hsdS2-MRSA252
	immunodominant antigen B		defensin resistance protein		transferrin-binding protein			hypothetical protein, similar to integral membrane protein LmrP		type I site-specific deoxyribo-nuclease subunit, 1st locus	type I site-specific deoxyribonuclease subunit, 2nd locus			
>"South American/ Mid														
LIT89 (Lithuania)	NEG	POS	POS	NEG	POS	NEG	POS	POS	NEG	NEG	POS	NEG	NEG	NEG
MRSA_PR1 (Malaysia)	NEG	POS	POS	NEG	POS	NEG	POS	POS	NEG	NEG	POS	NEG	NEG	NEG
Riyadh-2888905-R	NEG	POS	POS	AMB	POS	NEG	POS	POS	NEG	NEG	POS	NEG	NEG	NEG
Riyadh-2888915-BC	NEG	POS	POS	AMB	POS	NEG	POS	POS	NEG	NEG	POS	AMB	NEG	NEG
Riyadh-2793706-R	NEG	POS	POS	AMB	POS	AMB	POS	POS	NEG	NEG	POS	AMB	NEG	NEG
>"South American/ Mid														
Riyadh_SSTI_18_3502925	AMB	POS	POS	AMB	POS	AMB	POS	POS	NEG	NEG	POS	AMB	NEG	NEG
Riyadh-2822825-W	AMB	POS	POS	AMB	POS	AMB	POS	POS	NEG	NEG	POS	AMB	NEG	NEG
>"South American/ Mid														
UK-EMRSA-9	NEG	POS	POS	AMB	POS	NEG	POS	POS	NEG	NEG	POS	NEG	NEG	NEG
>"South American/ Mid														
Lome_HT20020815	NEG	POS	POS	AMB	POS	AMB	POS	POS	NEG	NEG	POS	NEG	NEG	NEG
>"South American/ Mid														
NA32 (Denmark)	NEG	POS	POS	NEG	POS	NEG	POS	POS	NEG	NEG	POS	NEG	NEG	NEG
H24 (Egypt)	NEG	POS	POS	NEG	POS	NEG	POS	POS	NEG	NEG	POS	NEG	NEG	NEG
ATCC BAA-39 (=HUSA304) (Hungary)	NEG	POS	POS	NEG	POS	NEG	POS	POS	NEG	NEG	POS	NEG	NEG	NEG
HUSA304 (Hungary)	NEG	POS	POS	NEG	POS	NEG	POS	POS	NEG	NEG	POS	NEG	NEG	NEG
HU106 (Hungary)	NEG	POS	POS	NEG	POS	NEG	POS	POS	NEG	NEG	POS	NEG	NEG	NEG
BSAC27 (UK)	NEG	POS	POS	NEG	POS	NEG	POS	POS	NEG	NEG	POS	NEG	NEG	NEG
NCTC13131, UK-EMRSA-4 (UK)	NEG	POS	POS	NEG	POS	NEG	POS	POS	NEG	NEG	POS	NEG	NEG	NEG
	NEG	POS	POS	AMB	POS	NEG	POS	POS	NEG	NEG	POS	NEG	NEG	NEG
Algiers_HT20040080	NEG	POS	POS	AMB	POS	NEG	POS	POS	NEG	NEG	POS	NEG	NEG	NEG
Dublin-DSH_AR09_0.0066	NEG	POS	POS	AMB	POS	NEG	POS	POS	NEG	NEG	POS	NEG	NEG	NEG
Dublin-DSH_AR09_0-0065	NEG	POS	POS	AMB	POS	NEG	POS	POS	NEG	NEG	POS	NEG	NEG	NEG
Dublin-DSH_Phenotype-III_84	NEG	POS	POS	AMB	POS	NEG	POS	POS	NEG	NEG	POS	NEG	NEG	NEG
Hong Kong_130	NEG	POS	POS	AMB	POS	NEG	POS	POS	NEG	NEG	POS	NEG	NEG	NEG
Kuwait_018	NEG	POS	POS	AMB	POS	NEG	POS	POS	NEG	NEG	POS	NEG	NEG	NEG
Perth_08-17726	NEG	POS	POS	AMB	POS	NEG	POS	POS	NEG	NEG	POS	AMB	NEG	NEG
Riyadh_Alfaisal/KKKSUH_86_MRS-14-279	NEG	POS	POS	AMB	POS	NEG	POS	POS	NEG	NEG	POS	NEG	NEG	NEG
Riyadh_Alfaisal-04_Z3861_831588	NEG	POS	POS	AMB	POS	AMB	POS	POS	NEG	NEG	POS	NEG	NEG	NEG
Riyadh_Alfaisal-30_S15724_1108013	NEG	POS	POS	AMB	POS	NEG	POS	POS	NEG	NEG	POS	NEG	NEG	NEG
Riyadh_Alfaisal-6_22A_13_B3992_397721	NEG	POS	POS	AMB	POS	AMB	POS	POS	NEG	NEG	POS	NEG	NEG	NEG
Riyadh_SSTI_52_3615482	NEG	POS	POS	AMB	POS	NEG	POS	POS	NEG	NEG	POS	NEG	NEG	NEG
Riyadh-2817276-2	AMB	POS	POS	AMB	POS	AMB	POS	POS	NEG	NEG	POS	POS	NEG	NEG
Riyadh-2891670-W	AMB	POS	POS	AMB	POS	AMB	POS	POS	NEG	NEG	POS	POS	NEG	NEG
Riyadh-3006920-W	NEG	POS	POS	AMB	POS	AMB	POS	POS	NEG	NEG	POS	AMB	NEG	NEG
Riyadh-R2567782	NEG	POS	POS	AMB	POS	AMB	POS	POS	NEG	NEG	POS	AMB	NEG	NEG
Russia-18_0252_Krasnoyarsk_SK2	NEG	POS	POS	AMB	POS	NEG	POS	POS	NEG	NEG	POS	NEG	NEG	NEG
UK-EMRSA-7	NEG	POS	POS	AMB	POS	NEG	POS	POS	NEG	NEG	POS	NEG	NEG	NEG
>"South American/ Mid														
Dublin-DSH_AR23_0073	NEG	POS	POS	AMB	POS	NEG	POS	POS	NEG	NEG	POS	NEG	NEG	NEG
>Related to "South Ame														
MRSA-OC3 (Russia)	NEG	POS	POS	NEG	POS	NEG	POS	POS	NEG	NEG	POS	NEG	NEG	NEG
Russia-12_0176_Krasnoyarsk	NEG	POS	POS	AMB	POS	NEG	POS	POS	NEG	NEG	POS	NEG	NEG	NEG
Russia-13_0180_Krasnoyarsk	NEG	POS	POS	AMB	POS	NEG	POS	POS	NEG	NEG	POS	NEG	NEG	NEG
Russia-16_0249_Krasnoyarsk	NEG	POS	POS	AMB	POS	NEG	POS	POS	NEG	NEG	POS	NEG	NEG	NEG
Russia-17_0250_Krasnoyarsk	NEG	POS	POS	AMB	POS	NEG	POS	POS	NEG	NEG	POS	NEG	NEG	NEG
>Related to "South Ame														
URU110 (Uruguay)	NEG	POS	POS	NEG	POS	NEG	POS	POS	NEG	NEG	POS	NEG	NEG	NEG
>Related to "South Ame														
DS_014 (Thailand)	NEG	POS	POS	NEG	POS	NEG	POS	POS	NEG	NEG	POS	NEG	NEG	NEG
>Unassigned Middle Eas														
Frankfurt_Oder_0490031797 (Libya)	NEG	POS	AMB	POS	POS	NEG	POS	POS	NEG	NEG	NEG	AMB	NEG	NEG
Kuwait_192	AMB	POS	AMB	POS	POS	AMB	POS	POS	NEG	NEG	NEG	AMB	NEG	NEG
Riyadh-9	AMB	POS	POS	AMB	POS	AMB	POS	POS	NEG	NEG	NEG	AMB	NEG	NEG
Russia-24_0407_Moscow	NEG	POS	POS	AMB	POS	NEG	POS	POS	NEG	NEG	NEG	NEG	NEG	NEG

STRAIN / ISOLATE	TYPE I RESTR:-MODIFICATION SYSTEM								MISCELLANEOUS GENES							
	hsdS3					hsdSx			ear2	Q2YUB3	Q7A4X2	Q931R4	Q9RL82			cstB
	hsdS3-AllOtherThanRF122+252	hsdS3-ST8+ST1+RF122	hsdS3-Mu50+N315	hsdS3-CC51+252	hsdS3-MRSA252	hsdSx-CC25	hsdSx-CC15	hsdSx-etd	ear2 = Q2FXC0	Q2YUB3	Q7A4X2	Q931R4 (CC5, CC15, CC30, CC97, CC188, ST1850)	Q9RL82 (consensus)	Q9RL82 (CC8)	Q9RL82-CC10/361	Q2G1R6-genomic island / cstB
	type I site-specific deoxyribonuclease subunit, 3rd locus					type I site-specific deoxyribonuclease subunit, unknown locus			Putative protein	Multidrug resistance transporter	Putative protein	major facilitator superfamily transporter	Putative protein			
**>"Eurasian Clade": TUR1**																
TUR1 (Turkey)	POS	POS	NEG	NEG	NEG	POS	NEG	NEG	POS	NEG	NEG	NEG	NEG	NEG	NEG	NEG
TUR9 (Turkey)	POS	POS	NEG	NEG	NEG	POS	NEG	NEG	POS	NEG	NEG	NEG	NEG	NEG	NEG	NEG
**>"Eurasian Clade" : T013**																
T0131 (China)	POS	POS	NEG	NEG	NEG	POS	NEG	NEG	POS	NEG	NEG	NEG	NEG	NEG	NEG	NEG
MU4 (Turkey)	POS	POS	NEG	NEG	NEG	POS	NEG	NEG	POS	NEG	NEG	NEG	NEG	NEG	NEG	NEG
DEU11 (Turkey)	POS	POS	NEG	NEG	NEG	POS	NEG	NEG	POS	NEG	NEG	NEG	NEG	NEG	NEG	NEG
DEU16 (Turkey)	POS	POS	NEG	NEG	NEG	POS	NEG	NEG	POS	NEG	NEG	NEG	NEG	NEG	NEG	NEG
Romania_Iasi_MRSA-07_54	POS	POS	NEG	NEG	NEG	POS	NEG	NEG	POS	AMB	NEG	NEG	NEG	NEG	NEG	NEG
**>"Eurasian Clade": arsC-**																
Romania_Iasi_MRSA-05_46	POS	POS	NEG	NEG	NEG	POS	NEG	NEG	POS	NEG	NEG	NEG	NEG	NEG	NEG	NEG
Romania_Iasi_MRSA-06_47	POS	POS	NEG	NEG	NEG	POS	NEG	NEG	POS	AMB	NEG	NEG	NEG	NEG	NEG	NEG
**>"Eurasian Clade": 16K/**																
CN79 (China)	POS	POS	NEG	NEG	NEG	POS	NEG	NEG	POS	NEG	NEG	NEG	NEG	NEG	NEG	NEG
CUHK_BJ2002 (China)	POS	POS	NEG	NEG	NEG	POS	NEG	NEG	POS	NEG	NEG	NEG	NEG	NEG	NEG	NEG
CUHK_BJ2007 (China)	POS	POS	NEG	NEG	NEG	POS	NEG	NEG	POS	NEG	NEG	NEG	NEG	NEG	NEG	NEG
3HK (Czech Republic)	POS	POS	NEG	NEG	NEG	POS	NEG	NEG	POS	NEG	NEG	NEG	NEG	NEG	NEG	NEG
HU109 (Hungary)	POS	POS	NEG	NEG	NEG	POS	NEG	NEG	POS	NEG	NEG	NEG	NEG	NEG	NEG	NEG
HUR18 (Hungary)	POS	POS	NEG	NEG	NEG	POS	NEG	NEG	POS	NEG	NEG	NEG	NEG	NEG	NEG	NEG
H482 (Romania)	POS	POS	NEG	NEG	NEG	POS	NEG	NEG	POS	NEG	NEG	NEG	NEG	NEG	NEG	NEG
16K (Russia)	POS	POS	NEG	NEG	NEG	POS	NEG	NEG	POS	NEG	NEG	NEG	NEG	NEG	NEG	NEG
DEU10 (Turkey)	POS	POS	NEG	NEG	NEG	POS	NEG	NEG	POS	NEG	NEG	NEG	NEG	NEG	NEG	NEG
DEU12 (Turkey)	POS	POS	NEG	NEG	NEG	POS	NEG	NEG	POS	NEG	NEG	NEG	NEG	NEG	NEG	NEG
DEU14 (Turkey)	POS	POS	NEG	NEG	NEG	POS	NEG	NEG	POS	NEG	NEG	NEG	NEG	NEG	NEG	NEG
DEU15 (Turkey)	POS	POS	NEG	NEG	NEG	POS	NEG	NEG	POS	NEG	NEG	NEG	NEG	NEG	NEG	NEG
DEU17 (Turkey)	POS	POS	NEG	NEG	NEG	POS	NEG	NEG	POS	NEG	NEG	NEG	NEG	NEG	NEG	NEG
DEU19 (Turkey)	POS	POS	NEG	NEG	NEG	POS	NEG	NEG	POS	NEG	NEG	NEG	NEG	NEG	NEG	NEG
DEU2 (Turkey)	POS	POS	NEG	NEG	NEG	POS	NEG	NEG	POS	NEG	NEG	NEG	NEG	NEG	NEG	NEG
DEU20 (Turkey)	POS	POS	NEG	NEG	NEG	POS	NEG	NEG	POS	NEG	NEG	NEG	NEG	NEG	NEG	NEG
DEU23 (Turkey)	POS	POS	NEG	NEG	NEG	POS	NEG	NEG	POS	NEG	NEG	NEG	NEG	NEG	NEG	NEG
DEU3 (Turkey)	POS	POS	NEG	NEG	NEG	POS	NEG	NEG	POS	NEG	NEG	NEG	NEG	NEG	NEG	NEG
DEU5 (Turkey)	POS	POS	NEG	NEG	NEG	POS	NEG	NEG	POS	NEG	NEG	NEG	NEG	NEG	NEG	NEG
DEU6 (Turkey)	POS	POS	NEG	NEG	NEG	POS	NEG	NEG	POS	NEG	NEG	NEG	NEG	NEG	NEG	NEG
DEU8 (Turkey)	POS	POS	NEG	NEG	NEG	POS	NEG	NEG	POS	NEG	NEG	NEG	NEG	NEG	NEG	NEG
HU11 (Turkey)	POS	POS	NEG	NEG	NEG	POS	NEG	NEG	POS	NEG	NEG	NEG	NEG	NEG	NEG	NEG
HU13 (Turkey)	POS	POS	NEG	NEG	NEG	POS	NEG	NEG	POS	NEG	NEG	NEG	NEG	NEG	NEG	NEG
HU14 (Turkey)	POS	POS	NEG	NEG	NEG	POS	NEG	NEG	POS	NEG	NEG	NEG	NEG	NEG	NEG	NEG
HU15 (Turkey)	POS	POS	NEG	NEG	NEG	POS	NEG	NEG	POS	NEG	NEG	NEG	NEG	NEG	NEG	NEG
HU16 (Turkey)	POS	POS	NEG	NEG	NEG	POS	NEG	NEG	POS	NEG	NEG	NEG	NEG	NEG	NEG	NEG
HU17 (Turkey)	POS	POS	NEG	NEG	NEG	POS	NEG	NEG	POS	NEG	NEG	NEG	NEG	NEG	NEG	NEG
HU21 (Turkey)	POS	POS	NEG	NEG	NEG	POS	NEG	NEG	POS	NEG	NEG	NEG	NEG	NEG	NEG	NEG
HU23 (Turkey)	POS	POS	NEG	NEG	NEG	POS	NEG	NEG	POS	NEG	NEG	NEG	NEG	NEG	NEG	NEG
HU26 (Turkey)	POS	POS	NEG	NEG	NEG	POS	NEG	NEG	POS	NEG	NEG	NEG	NEG	NEG	NEG	NEG
HU4 (Turkey)	POS	POS	NEG	NEG	NEG	POS	NEG	NEG	POS	NEG	NEG	NEG	NEG	NEG	NEG	NEG
HU5 (Turkey)	POS	POS	NEG	NEG	NEG	POS	NEG	NEG	POS	NEG	NEG	NEG	NEG	NEG	NEG	NEG
HU6 (Turkey)	POS	POS	NEG	NEG	NEG	POS	NEG	NEG	POS	NEG	NEG	NEG	NEG	NEG	NEG	NEG
HU7 (Turkey)	POS	POS	NEG	NEG	NEG	POS	NEG	NEG	POS	NEG	NEG	NEG	NEG	NEG	NEG	NEG
HU8 (Turkey)	POS	POS	NEG	NEG	NEG	POS	NEG	NEG	POS	NEG	NEG	NEG	NEG	NEG	NEG	NEG
HU9 (Turkey)	POS	POS	NEG	NEG	NEG	POS	NEG	NEG	POS	NEG	NEG	NEG	NEG	NEG	NEG	NEG
IU1 (Turkey)	POS	POS	NEG	NEG	NEG	POS	NEG	NEG	POS	NEG	NEG	NEG	NEG	NEG	NEG	NEG
IU10 (Turkey)	POS	POS	NEG	NEG	NEG	POS	NEG	NEG	POS	NEG	NEG	NEG	NEG	NEG	NEG	NEG
IU11 (Turkey)	POS	POS	NEG	NEG	NEG	POS	NEG	NEG	POS	NEG	NEG	NEG	NEG	NEG	NEG	NEG
IU12 (Turkey)	POS	POS	NEG	NEG	NEG	POS	NEG	NEG	POS	NEG	NEG	NEG	NEG	NEG	NEG	NEG
IU13 (Turkey)	POS	POS	NEG	NEG	NEG	POS	NEG	NEG	POS	NEG	NEG	NEG	NEG	NEG	NEG	NEG
IU15 (Turkey)	POS	POS	NEG	NEG	NEG	POS	NEG	NEG	POS	NEG	NEG	NEG	NEG	NEG	NEG	NEG
IU18 (Turkey)	POS	POS	NEG	NEG	NEG	POS	NEG	NEG	POS	NEG	NEG	NEG	NEG	NEG	NEG	NEG
IU19 (Turkey)	POS	POS	NEG	NEG	NEG	POS	NEG	NEG	POS	NEG	NEG	NEG	NEG	NEG	NEG	NEG
IU2 (Turkey)	POS	POS	NEG	NEG	NEG	POS	NEG	NEG	POS	NEG	NEG	NEG	NEG	NEG	NEG	NEG
IU4 (Turkey)	POS	POS	NEG	NEG	NEG	POS	NEG	NEG	POS	NEG	NEG	NEG	NEG	NEG	NEG	NEG
IU6 (Turkey)	POS	POS	NEG	NEG	NEG	POS	NEG	NEG	POS	NEG	NEG	NEG	NEG	NEG	NEG	NEG
IU7 (Turkey)	POS	POS	NEG	NEG	NEG	POS	NEG	NEG	POS	NEG	NEG	NEG	NEG	NEG	NEG	NEG
IU9 (Turkey)	POS	POS	NEG	NEG	NEG	POS	NEG	NEG	POS	NEG	NEG	NEG	NEG	NEG	NEG	NEG
MU1 (Turkey)	POS	POS	NEG	NEG	NEG	POS	NEG	NEG	POS	NEG	NEG	NEG	NEG	NEG	NEG	NEG
MU10 (Turkey)	POS	POS	NEG	NEG	NEG	POS	NEG	NEG	POS	NEG	NEG	NEG	NEG	NEG	NEG	NEG
MU20 (Turkey)	POS	POS	NEG	NEG	NEG	POS	NEG	NEG	POS	NEG	NEG	NEG	NEG	NEG	NEG	NEG
Mu3 (Turkey)	NEG	POS	NEG	NEG	NEG	AMB	NEG	NEG	POS	NEG	NEG	NEG	NEG	NEG	NEG	NEG
MU5 (Turkey)	POS	POS	NEG	NEG	NEG	POS	NEG	NEG	POS	NEG	NEG	NEG	NEG	NEG	NEG	NEG
MU6 (Turkey)	POS	POS	NEG	NEG	NEG	POS	NEG	NEG	POS	NEG	NEG	NEG	NEG	NEG	NEG	NEG
MU7 (Turkey)	POS	POS	NEG	NEG	NEG	POS	NEG	NEG	POS	NEG	NEG	NEG	NEG	NEG	NEG	NEG
TUR27 (Turkey)	POS	POS	NEG	NEG	NEG	POS	NEG	NEG	POS	NEG	NEG	NEG	NEG	NEG	NEG	NEG
NCTR 32S	POS	POS	NEG	NEG	NEG	POS	NEG	NEG	POS	NEG	NEG	NEG	NEG	NEG	NEG	NEG
SA02_A4 = Russia-06_0085_Moscow	POS	POS	NEG	NEG	NEG	POS	NEG	NEG	POS	NEG	NEG	NEG	NEG	NEG	NEG	NEG
	POS	POS	NEG	NEG	NEG	POS	NEG	NEG	POS	NEG	NEG	NEG	NEG	NEG	NEG	NEG
Dresden_08V35987 (Turkey)	POS	POS	NEG	NEG	NEG	POS	NEG	NEG	POS	NEG	NEG	NEG	NEG	NEG	NEG	NEG
Dresden_17ANR577152	POS	POS	NEG	NEG	NEG	POS	NEG	NEG	POS	NEG	NEG	NEG	NEG	NEG	NEG	NEG
Dresden_17ANR580374 (Makedonia)	POS	POS	NEG	NEG	NEG	POS	NEG	NEG	POS	NEG	NEG	NEG	NEG	NEG	NEG	NEG
Hong Kong_69-II	POS	POS	NEG	NEG	NEG	POS	NEG	NEG	POS	NEG	NEG	NEG	NEG	NEG	NEG	NEG
Hong Kong_93	POS	POS	NEG	NEG	NEG	POS	AMB	NEG	POS	POS	NEG	NEG	NEG	NEG	NEG	NEG
Rawalpindi_Kidney Center_03_SK1	POS	POS	NEG	NEG	NEG	POS	NEG	NEG	POS	NEG	NEG	NEG	NEG	NEG	NEG	NEG
Rawalpindi_Kidney Center_08	POS	POS	NEG	NEG	NEG	POS	NEG	NEG	POS	NEG	NEG	NEG	NEG	NEG	NEG	NEG
Rawalpindi_Kidney Center_10	POS	POS	NEG	NEG	NEG	POS	NEG	NEG	POS	AMB	NEG	NEG	NEG	NEG	NEG	NEG
Rawalpindi_Kidney Center_19	POS	POS	NEG	NEG	NEG	POS	NEG	NEG	POS	AMB	NEG	NEG	NEG	NEG	NEG	NEG
Rawalpindi_Kidney Center_50_SK1	POS	POS	NEG	NEG	NEG	POS	NEG	NEG	POS	NEG	NEG	NEG	NEG	NEG	NEG	NEG
Romania_Iasi_BC-49_430	POS	POS	NEG	NEG	NEG	POS	NEG	NEG	POS	AMB	NEG	NEG	NEG	NEG	NEG	NEG
Romania_Iasi_MRSA_284	POS	POS	NEG	NEG	NEG	POS	NEG	NEG	POS	NEG	NEG	NEG	NEG	NEG	NEG	NEG
Romania_Iasi_MRSA-32_318	POS	POS	NEG	NEG	NEG	POS	NEG	NEG	POS	NEG	NEG	NEG	NEG	NEG	NEG	NEG
Romania_Iasi_MRSA-54_430	POS	POS	NEG	NEG	NEG	POS	AMB	NEG	POS	POS	NEG	NEG	NEG	NEG	NEG	NEG
Romania_Iasi_SSTI-01_2	POS	POS	NEG	NEG	NEG	POS	NEG	NEG	POS	NEG	NEG	NEG	NEG	NEG	NEG	NEG
Romania_Iasi_SSTI-10_101	POS	POS	NEG	NEG	NEG	POS	NEG	NEG	POS	NEG	NEG	NEG	NEG	NEG	NEG	NEG
Romania_Iasi_SSTI-11_106	POS	POS	NEG	NEG	NEG	POS	NEG	NEG	POS	NEG	NEG	NEG	NEG	NEG	NEG	NEG
Russia-01_0001_SaintPetersburg	POS	POS	NEG	NEG	NEG	POS	NEG	NEG	POS	NEG	NEG	NEG	NEG	NEG	NEG	NEG
Russia-02_0004_SaintPetersburg	POS	POS	NEG	NEG	NEG	POS	NEG	NEG	POS	NEG	NEG	NEG	NEG	NEG	NEG	NEG
Russia-03_0057_SaintPetersburg	POS	POS	NEG	NEG	NEG	POS	NEG	NEG	POS	NEG	NEG	NEG	NEG	NEG	NEG	NEG
Russia-04_0076_Moscow	POS	POS	NEG	NEG	NEG	POS	NEG	NEG	POS	NEG	NEG	NEG	NEG	NEG	NEG	NEG
Russia-05_0078_Moscow	POS	POS	NEG	NEG	NEG	POS	NEG	NEG	POS	NEG	NEG	NEG	NEG	NEG	NEG	NEG
Russia-08_0135_Moscow	POS	POS	NEG	NEG	NEG	POS	NEG	NEG	POS	NEG	NEG	NEG	NEG	NEG	NEG	NEG
Russia-09_0150_Kurgan	POS	POS	NEG	NEG	NEG	POS	NEG	NEG	POS	NEG	NEG	NEG	NEG	NEG	NEG	NEG
Russia-10_0162_Kurgan	POS	POS	NEG	NEG	NEG	POS	NEG	NEG	POS	NEG	NEG	NEG	NEG	NEG	NEG	NEG
Russia-14_0184_Moscow	POS	POS	NEG	NEG	NEG	POS	NEG	NEG	POS	NEG	NEG	NEG	NEG	NEG	NEG	NEG
Russia-15_0232_SaintPetersburg	POS	POS	NEG	NEG	NEG	POS	NEG	NEG	POS	NEG	NEG	NEG	NEG	NEG	NEG	NEG
Russia-22_0391_Chelyabinsk	POS	POS	NEG	NEG	NEG	POS	NEG	NEG	POS	NEG	NEG	NEG	NEG	NEG	NEG	NEG
Russia-23_0392_Chelyabinsk	POS	POS	NEG	NEG	NEG	POS	NEG	NEG	POS	NEG	NEG	NEG	NEG	NEG	NEG	NEG
**>"Eurasian Clade" : IU17**																
IU17 (Turkey)	POS	POS	NEG	NEG	NEG	POS	NEG	NEG	POS	NEG	NEG	NEG	NEG	NEG	NEG	NEG
IU20 (Turkey)	POS	POS	NEG	NEG	NEG	POS	NEG	NEG	POS	NEG	NEG	NEG	NEG	NEG	NEG	NEG

STRAIN / ISOLATE	TYPE I RESTR:-MODIFICATION SYSTEM								MISCELLANEOUS GENES							
	hsdS3					hsdSx			ear2	Q2YUB3	Q7A4X2	Q931R4	Q9RL82			cstB
	hsdS3-AllOtherThanRF12 2+252	hsdS3-ST8+ST1+RF122	hsdS3-Mu50+N315	hsdS3-CC51+252	hsdS3-MRSA252	hsdSx-CC25	hsdSx-CC15	hsdSx-etd	ear2 = Q2FXC0	Q2YUB3	Q7A4X2	Q931R4 (CC5, CC15, CC30, CC97, CC188, ST1850)	Q9RL82 (consensus)	Q9RL82 (CC8)	Q9RL82-CC10/361	Q2G1R6-genomic island / cstB
	type I site-specific deoxyribonuclease subunit, 3rd locus					type I site-specific deoxyribonuclease subunit, unknown locus			Putative protein	Multidrug resistance transporter	Putative protein	major facilitator superfamily transporter	Putative protein			
**>"Eurasian Clade": Spor[illegible]**																
Dresden_16ANR581769	POS	POS	NEG	NEG	NEG	POS	NEG	NEG	POS	NEG	NEG	NEG	NEG	NEG	NEG	NEG
Dresden_17ANR578769	POS	POS	NEG	NEG	NEG	POS	NEG	NEG	POS	NEG	NEG	NEG	NEG	NEG	NEG	NEG
**>"Eurasian Clade": Kuw[illegible]**																
Kuwait_306	POS	POS	NEG	NEG	NEG	POS	NEG	NEG	POS	POS	NEG	NEG	NEG	NEG	NEG	NEG
Kuwait_301	POS	POS	NEG	NEG	NEG	POS	NEG	NEG	POS	POS	NEG	NEG	NEG	NEG	NEG	NEG
**>"European Clade": "Gr[illegible]**																
GRE18 (Greece)	POS	POS	NEG	NEG	NEG	POS	NEG	NEG	POS	NEG	NEG	NEG	NEG	NEG	NEG	NEG
GRE317 (Greece)	POS	POS	NEG	NEG	NEG	POS	NEG	NEG	POS	NEG	NEG	NEG	NEG	NEG	NEG	NEG
GRE4 (Greece)	POS	NEG	NEG	NEG	NEG	POS	NEG	NEG	NEG	NEG	NEG	NEG	NEG	NEG	NEG	NEG
R35 (USA)	POS	POS	NEG	NEG	NEG	POS	NEG	NEG	POS	NEG	NEG	NEG	NEG	NEG	NEG	NEG
Casablanca_HT20060548	POS	POS	NEG	NEG	NEG	POS	NEG	NEG	POS	NEG	NEG	NEG	NEG	NEG	NEG	NEG
Casablanca_HT20060550	POS	POS	NEG	NEG	NEG	POS	NEG	NEG	POS	NEG	POS	NEG	NEG	NEG	NEG	NEG
Dresden_01V34572 (Greece)	POS	POS	NEG	NEG	NEG	POS	NEG	NEG	POS	NEG	NEG	NEG	NEG	NEG	NEG	NEG
Dresden_01V36469	POS	POS	NEG	NEG	NEG	POS	NEG	NEG	POS	NEG	NEG	NEG	NEG	NEG	NEG	NEG
Dresden_01V39123	POS	POS	NEG	NEG	NEG	POS	NEG	NEG	POS	NEG	NEG	NEG	NEG	NEG	NEG	NEG
Dresden_01V39143	POS	POS	NEG	NEG	NEG	POS	NEG	NEG	POS	NEG	NEG	NEG	NEG	NEG	NEG	NEG
Greece 1_3680_Harmony	POS	POS	NEG	NEG	NEG	POS	NEG	NEG	POS	NEG	NEG	NEG	NEG	NEG	NEG	NEG
**>"European Clade": UK-[illegible]**																
ANS46 (Australia)	POS	POS	NEG	NEG	NEG	POS	NEG	NEG	POS	NEG	NEG	NEG	NEG	NEG	NEG	NEG
LHH1 (USA)	POS	POS	NEG	NEG	NEG	POS	NEG	NEG	POS	NEG	NEG	NEG	NEG	NEG	NEG	NEG
Dublin-DSH_AR01_0-1085	POS	POS	NEG	NEG	NEG	POS	NEG	NEG	POS	NEG	NEG	NEG	NEG	NEG	NEG	NEG
Dublin-DSH_AR01_0-1118	POS	POS	NEG	NEG	NEG	POS	NEG	NEG	POS	NEG	NEG	NEG	NEG	NEG	NEG	NEG
Dublin-DSH_AR15_0098	POS	POS	NEG	NEG	NEG	POS	NEG	NEG	POS	NEG	NEG	NEG	NEG	NEG	NEG	NEG
Dublin-DSH_AR15_0104	POS	POS	NEG	NEG	NEG	POS	NEG	NEG	POS	NEG	NEG	NEG	NEG	NEG	NEG	NEG
UK_NCTC11939_UK-1_Harmony	POS	POS	NEG	NEG	NEG	POS	NEG	NEG	POS	NEG	NEG	NEG	NEG	NEG	NEG	NEG
**>"European Clade": BK2**																
BK2421 (USA)	POS	POS	NEG	NEG	NEG	POS	NEG	NEG	POS	NEG	NEG	NEG	NEG	NEG	NEG	NEG
**>"Aussi/NZ Clade", JKD[illegible]**																
JKD6009 (Australia)	NEG	NEG	NEG	NEG	NEG	AMB	NEG	NEG	NEG	NEG	NEG	NEG	NEG	NEG	NEG	NEG
JKD6008 (New Zealand)	NEG	NEG	NEG	NEG	NEG	AMB	NEG	NEG	NEG	NEG	NEG	NEG	NEG	NEG	NEG	NEG
Perth_2015-S20209	POS	POS	NEG	NEG	NEG	POS	NEG	NEG	POS	NEG	NEG	NEG	NEG	NEG	NEG	NEG
Perth_2015-S20305	NEG	NEG	NEG	NEG	NEG	AMB	NEG	NEG	POS	NEG	NEG	NEG	NEG	NEG	NEG	NEG
Perth_2015-S20603	NEG	NEG	NEG	NEG	NEG	AMB	NEG	NEG	POS	NEG	NEG	NEG	NEG	NEG	NEG	NEG
Perth_2015-S20607	POS	POS	NEG	NEG	NEG	POS	NEG	NEG	POS	NEG	NEG	NEG	NEG	NEG	NEG	NEG
Perth_2015-S20608	NEG	NEG	NEG	NEG	NEG	AMB	NEG	NEG	POS	NEG	NEG	NEG	NEG	NEG	NEG	NEG
Perth_2015-S20610	NEG	NEG	NEG	NEG	NEG	AMB	NEG	NEG	POS	NEG	NEG	NEG	NEG	NEG	NEG	NEG
Perth_2015-S20613	NEG	NEG	NEG	NEG	NEG	AMB	NEG	NEG	POS	NEG	NEG	NEG	NEG	NEG	NEG	NEG
Perth_2015-S20617	NEG	NEG	NEG	NEG	NEG	AMB	NEG	NEG	POS	NEG	NEG	NEG	NEG	NEG	NEG	NEG
Perth_2015-S20622	NEG	NEG	NEG	NEG	NEG	AMB	NEG	NEG	POS	NEG	NEG	NEG	NEG	NEG	NEG	NEG
Perth_2015-S23619	NEG	NEG	NEG	NEG	NEG	AMB	NEG	NEG	NEG	NEG	NEG	NEG	NEG	NEG	NEG	NEG
Perth_2015-S41210	POS	POS	NEG	NEG	NEG	POS	NEG	NEG	POS	NEG	NEG	NEG	NEG	NEG	NEG	NEG
Perth_2015-S51001	POS	POS	NEG	NEG	NEG	POS	NEG	NEG	POS	NEG	NEG	NEG	NEG	NEG	NEG	NEG
Perth_2015-S51003	POS	POS	NEG	NEG	NEG	POS	NEG	NEG	POS	NEG	NEG	NEG	NEG	NEG	NEG	NEG
Perth_2015-S51006	POS	POS	NEG	NEG	NEG	POS	NEG	NEG	POS	NEG	NEG	NEG	NEG	NEG	NEG	NEG
Perth_2015-S51007	POS	POS	NEG	NEG	NEG	POS	NEG	NEG	POS	NEG	NEG	NEG	NEG	NEG	NEG	NEG
Perth_2015-S51008	POS	POS	NEG	NEG	NEG	POS	NEG	NEG	POS	NEG	NEG	NEG	NEG	NEG	NEG	NEG
Perth_2015-S51014	POS	POS	NEG	NEG	NEG	POS	NEG	NEG	POS	NEG	NEG	NEG	NEG	NEG	NEG	NEG
Perth_2015-S51408	POS	POS	NEG	NEG	NEG	POS	NEG	NEG	POS	NEG	NEG	NEG	NEG	NEG	NEG	NEG
Perth_2016-S20614	NEG	NEG	NEG	NEG	NEG	AMB	NEG	NEG	POS	NEG	NEG	NEG	NEG	NEG	NEG	NEG
Perth_2016-S23614	NEG	NEG	NEG	NEG	NEG	AMB	NEG	NEG	NEG	NEG	NEG	NEG	NEG	NEG	NEG	NEG
Perth_2016-S42009	POS	POS	NEG	NEG	NEG	POS	NEG	NEG	POS	NEG	NEG	NEG	NEG	NEG	NEG	NEG
Perth_2016-S51001	POS	POS	NEG	NEG	NEG	POS	NEG	NEG	POS	NEG	NEG	NEG	NEG	NEG	NEG	NEG
Perth_2016-S51403	POS	POS	NEG	NEG	NEG	POS	NEG	NEG	POS	NEG	NEG	NEG	NEG	NEG	NEG	NEG
Perth_07 RPA 20	NEG	NEG	NEG	NEG	NEG	POS	NEG	NEG	NEG	NEG	NEG	NEG	NEG	NEG	NEG	NEG
Perth_08-19088	POS	POS	NEG	NEG	NEG	POS	NEG	NEG	POS	NEG	NEG	NEG	NEG	NEG	NEG	NEG
Perth_01-15351	POS	POS	NEG	NEG	NEG	POS	NEG	NEG	POS	NEG	NEG	NEG	NEG	NEG	NEG	NEG
Perth_01-15357	POS	POS	NEG	NEG	NEG	POS	NEG	NEG	POS	NEG	NEG	NEG	NEG	NEG	NEG	NEG
Perth_01-15419	POS	POS	NEG	NEG	NEG	POS	NEG	NEG	POS	NEG	NEG	NEG	NEG	NEG	NEG	NEG
Perth_01-16309	POS	POS	NEG	NEG	NEG	POS	NEG	NEG	POS	NEG	NEG	NEG	NEG	NEG	NEG	NEG
Perth_03 SNP 95	POS	POS	NEG	NEG	NEG	POS	NEG	NEG	POS	POS	NEG	NEG	NEG	NEG	NEG	NEG
Perth_2003_AH_47	POS	POS	NEG	NEG	NEG	POS	NEG	NEG	POS	NEG	NEG	NEG	NEG	NEG	NEG	NEG
**>"Australian/NZ Clade":**																
Perth_2015-S20623	NEG	NEG	NEG	NEG	NEG	AMB	NEG	NEG	POS	NEG	NEG	NEG	NEG	NEG	NEG	NEG
Perth_2015-S20826	POS	POS	NEG	NEG	NEG	POS	NEG	NEG	POS	NEG	NEG	NEG	NEG	NEG	NEG	NEG
Perth_05 NH 56	POS	POS	NEG	NEG	NEG	POS	NEG	NEG	POS	AMB	NEG	NEG	NEG	NEG	NEG	NEG
**>CC5-MRSA-III LA MRSA**																
Delbrück 38 isolate from domestic turkey	POS	NEG	POS	NEG	NEG	POS	AMB	NEG	NEG	AMB	POS	POS	NEG	NEG	NEG	POS
**> Staph. pseudintermed**																
KM1381	NEG	NEG	NEG	NEG	NEG	NEG	NEG	NEG	NEG	NEG	NEG	NEG	NEG	NEG	NEG	NEG
**>"South-East Asian Clad[illegible]**																
M92 (Canada)	POS	POS	NEG	NEG	NEG	POS	NEG	NEG	POS	NEG	NEG	NEG	NEG	NEG	NEG	NEG
CHI59 (China)	POS	POS	NEG	NEG	NEG	POS	NEG	NEG	POS	NEG	NEG	NEG	NEG	NEG	NEG	NEG
CHI61 (China)	POS	POS	NEG	NEG	NEG	POS	NEG	NEG	POS	NEG	NEG	NEG	NEG	NEG	NEG	NEG
CUHK_HK1997 (China)	POS	POS	NEG	NEG	NEG	POS	NEG	NEG	POS	NEG	NEG	NEG	NEG	NEG	NEG	NEG
M996 (China)	POS	POS	NEG	NEG	NEG	POS	NEG	NEG	POS	NEG	NEG	NEG	NEG	NEG	NEG	NEG
H211 (Denmark)	POS	POS	NEG	NEG	NEG	POS	NEG	NEG	POS	NEG	NEG	NEG	NEG	NEG	NEG	NEG
H216 (Denmark)	POS	POS	NEG	NEG	NEG	POS	NEG	NEG	POS	NEG	NEG	NEG	NEG	NEG	NEG	NEG
D71 (Germany)	POS	POS	NEG	NEG	NEG	POS	NEG	NEG	POS	NEG	NEG	NEG	NEG	NEG	NEG	NEG
NMR05 (India)	POS	POS	NEG	NEG	NEG	POS	NEG	NEG	POS	NEG	NEG	NEG	NEG	NEG	NEG	NEG
MAL11 (Malaysia)	POS	POS	NEG	NEG	NEG	POS	NEG	NEG	POS	NEG	NEG	NEG	NEG	NEG	NEG	NEG
MAL9 (Malaysia)	POS	POS	NEG	NEG	NEG	POS	NEG	NEG	POS	NEG	NEG	NEG	NEG	NEG	NEG	NEG
Na21 (Sri Lanka)	POS	POS	NEG	NEG	NEG	POS	NEG	NEG	POS	NEG	NEG	NEG	NEG	NEG	NEG	NEG
M592 (Syria)	POS	POS	NEG	NEG	NEG	POS	NEG	NEG	POS	NEG	NEG	NEG	NEG	NEG	NEG	NEG
H202 (Thailand)	POS	POS	NEG	NEG	NEG	POS	NEG	NEG	POS	NEG	NEG	NEG	NEG	NEG	NEG	NEG
S102 (Thailand)	POS	POS	NEG	NEG	NEG	POS	NEG	NEG	POS	NEG	NEG	NEG	NEG	NEG	NEG	NEG
S130 (Thailand)	POS	POS	NEG	NEG	NEG	POS	NEG	NEG	POS	NEG	NEG	NEG	NEG	NEG	NEG	NEG
S40 (Thailand)	POS	POS	NEG	NEG	NEG	POS	NEG	NEG	POS	NEG	NEG	NEG	NEG	NEG	NEG	NEG
S71 (Thailand)	POS	POS	NEG	NEG	NEG	POS	NEG	NEG	POS	NEG	NEG	NEG	NEG	NEG	NEG	NEG
S87 (Thailand)	POS	POS	NEG	NEG	NEG	POS	NEG	NEG	POS	NEG	NEG	NEG	NEG	NEG	NEG	NEG
S93 (Thailand)	POS	POS	NEG	NEG	NEG	POS	NEG	NEG	POS	NEG	NEG	NEG	NEG	NEG	NEG	NEG
US_002 (Thailand)	POS	POS	NEG	NEG	NEG	POS	NEG	NEG	POS	NEG	NEG	NEG	NEG	NEG	NEG	NEG
US_017 (Thailand)	POS	POS	NEG	NEG	NEG	POS	NEG	NEG	POS	NEG	NEG	NEG	NEG	NEG	NEG	NEG
WAMC6102 (Thailand)	POS	POS	NEG	NEG	NEG	POS	NEG	NEG	POS	NEG	NEG	NEG	NEG	NEG	NEG	NEG
NCTC_13626_TW20 (UK)	POS	POS	NEG	NEG	NEG	POS	NEG	NEG	POS	NEG	NEG	NEG	NEG	NEG	NEG	NEG
BSAC3021 (UK)	POS	POS	NEG	NEG	NEG	POS	NEG	NEG	POS	NEG	NEG	NEG	NEG	NEG	NEG	NEG
BSAC3046 (UK)	POS	POS	NEG	NEG	NEG	POS	NEG	NEG	POS	NEG	NEG	NEG	NEG	NEG	NEG	NEG
1193_SAUR (USA)	POS	POS	NEG	NEG	NEG	POS	NEG	NEG	POS	NEG	NEG	NEG	NEG	NEG	NEG	NEG
1194_SAUR (USA)	POS	POS	NEG	NEG	NEG	POS	NEG	NEG	POS	NEG	NEG	NEG	NEG	NEG	NEG	NEG

STRAIN / ISOLATE	TYPE I RESTR:-MODIFICATION SYSTEM								MISCELLANEOUS GENES							
	hsdS3					hsdSx			ear2	Q2YUB3	Q7A4X2	Q931R4	Q9RL82			cstB
	hsdS3-AllOtherThanRF12 2+252	hsdS3-ST8+ST1+RF122	hsdS3-Mu50+N315	hsdS3-CC51+252	hsdS3-MRSA252	hsdSx-CC25	hsdSx-CC15	hsdSx-etd	ear2 = Q2FXC0	Q2YUB3	Q7A4X2	Q931R4 (CC5, CC15, CC30, CC97, CC188, ST1850)	Q9RL82 (consensus)	Q9RL82 (CC8)	Q9RL82-CC10/361	Q2G1R6-genomic island / cstB
	type I site-specific deoxyribonuclease subunit, 3rd locus					type I site-specific deoxyribonuclease subunit, unknown locus			Putative protein	Multidrug resistance transporter	Putative protein	major facilitator superfamily transporter	Putative protein			
>"South-East Asian Clade": TW20 (Irish AR44), c[illegible]																
1195_SAUR (USA)	POS	POS	NEG	NEG	NEG	POS	NEG	NEG	POS	NEG	NEG	NEG	NEG	NEG	NEG	NEG
FVRH6002 (USA)	POS	POS	NEG	NEG	NEG	POS	NEG	NEG	POS	NEG	NEG	NEG	NEG	NEG	NEG	NEG
KINW6048 (USA)	POS	POS	NEG	NEG	NEG	POS	NEG	NEG	POS	NEG	NEG	NEG	NEG	NEG	NEG	NEG
LAMC0011 (USA)	POS	POS	NEG	NEG	NEG	POS	NEG	NEG	POS	NEG	NEG	NEG	NEG	NEG	NEG	NEG
SJOS6053 (USA)	POS	POS	NEG	NEG	NEG	POS	NEG	NEG	POS	NEG	NEG	NEG	NEG	NEG	NEG	NEG
SJOS6072 (USA)	POS	POS	NEG	NEG	NEG	POS	NEG	NEG	POS	NEG	NEG	NEG	NEG	NEG	NEG	NEG
UCIM6042 (USA)	POS	POS	NEG	NEG	NEG	POS	NEG	NEG	POS	NEG	NEG	NEG	NEG	NEG	NEG	NEG
Perth_2015-S31902	NEG	NEG	NEG	NEG	NEG	AMB	NEG	NEG	POS	NEG	NEG	NEG	NEG	NEG	NEG	NEG
Perth_2015-S31904	POS	POS	NEG	NEG	NEG	POS	NEG	NEG	POS	NEG	NEG	NEG	NEG	NEG	NEG	NEG
Perth_2015-S31913	POS	POS	NEG	NEG	NEG	POS	NEG	NEG	POS	NEG	NEG	NEG	NEG	NEG	NEG	NEG
Perth_2015-S31918	POS	POS	NEG	NEG	NEG	POS	NEG	NEG	POS	NEG	NEG	NEG	NEG	NEG	NEG	NEG
Dublin-DSH_AR44_E1183	POS	POS	NEG	NEG	NEG	POS	NEG	NEG	POS	NEG	NEG	NEG	NEG	NEG	NEG	NEG
Dublin-DSH_Unfamiliar-3_E1520	POS	POS	NEG	NEG	NEG	POS	NEG	NEG	POS	NEG	NEG	NEG	NEG	NEG	NEG	NEG
Hong Kong_042	POS	POS	NEG	NEG	NEG	POS	NEG	NEG	POS	NEG	NEG	NEG	NEG	NEG	NEG	NEG
Hong Kong_101	POS	POS	NEG	NEG	NEG	POS	NEG	NEG	POS	NEG	NEG	NEG	NEG	NEG	NEG	NEG
Hong Kong_111	POS	POS	NEG	NEG	NEG	POS	AMB	NEG	POS	POS	NEG	NEG	NEG	NEG	NEG	NEG
Hong Kong_113	POS	POS	NEG	NEG	NEG	POS	NEG	NEG	POS	NEG	NEG	NEG	NEG	NEG	NEG	NEG
Hong Kong_115	POS	POS	NEG	NEG	NEG	POS	NEG	NEG	POS	NEG	NEG	NEG	NEG	NEG	NEG	NEG
Hong Kong_118	POS	POS	NEG	NEG	NEG	POS	NEG	NEG	POS	NEG	NEG	NEG	NEG	NEG	NEG	NEG
Hong Kong_125	POS	POS	NEG	NEG	NEG	POS	NEG	NEG	POS	NEG	NEG	NEG	NEG	NEG	NEG	NEG
Hong Kong_129	POS	POS	NEG	NEG	NEG	POS	NEG	NEG	POS	NEG	NEG	NEG	NEG	NEG	NEG	NEG
Hong Kong_133	POS	POS	NEG	NEG	NEG	POS	NEG	NEG	POS	NEG	NEG	NEG	NEG	NEG	NEG	NEG
Kuwait_009	POS	POS	NEG	NEG	NEG	POS	NEG	NEG	POS	NEG	NEG	NEG	NEG	NEG	NEG	NEG
Kuwait_103	POS	POS	NEG	NEG	NEG	POS	AMB	NEG	POS	POS	NEG	NEG	NEG	NEG	NEG	NEG
Kuwait_107	POS	POS	NEG	NEG	NEG	POS	NEG	NEG	POS	POS	NEG	NEG	NEG	NEG	NEG	NEG
Perth_03 AH 88	POS	POS	NEG	NEG	NEG	POS	NEG	NEG	POS	NEG	NEG	NEG	NEG	NEG	NEG	NEG
Perth_08-16905	POS	POS	NEG	NEG	NEG	POS	NEG	NEG	POS	NEG	NEG	NEG	NEG	NEG	NEG	NEG
Riyadh_Alfaisal-10_SICU_13_B5672_1117353	POS	POS	NEG	NEG	NEG	POS	NEG	NEG	POS	NEG	NEG	NEG	NEG	NEG	NEG	NEG
Riyadh_SSTI_17_3508602	POS	POS	NEG	NEG	NEG	POS	NEG	NEG	POS	NEG	NEG	NEG	NEG	NEG	NEG	NEG
Riyadh-2822088-R	POS	POS	NEG	NEG	NEG	POS	NEG	NEG	POS	AMB	NEG	NEG	NEG	NEG	NEG	NEG
Trinidad&Tobago_2012_195 (619/12)	POS	POS	NEG	NEG	NEG	POS	NEG	NEG	POS	AMB	NEG	NEG	NEG	NEG	NEG	NEG
Trinidad&Tobago_2012_370 (1112-52177)	POS	POS	NEG	NEG	NEG	POS	NEG	NEG	POS	AMB	NEG	NEG	NEG	NEG	NEG	NEG
Trinidad&Tobago_2012_378 (4139)	POS	POS	NEG	NEG	NEG	POS	NEG	NEG	POS	AMB	NEG	NEG	NEG	NEG	NEG	NEG
Trinidad&Tobago_2013_PA21	POS	POS	NEG	NEG	NEG	POS	NEG	NEG	POS	NEG	NEG	NEG	NEG	NEG	NEG	NEG
Trinidad&Tobago_2013_PA22	POS	POS	NEG	NEG	NEG	POS	NEG	NEG	POS	POS	NEG	NEG	NEG	NEG	NEG	NEG
Trinidad&Tobago_2912_340 (8523/12)	POS	POS	NEG	NEG	NEG	POS	NEG	NEG	POS	AMB	NEG	NEG	NEG	NEG	NEG	NEG
Trinidad&Tobago_MRSA2010_105	POS	POS	NEG	NEG	NEG	POS	NEG	NEG	POS	AMB	NEG	NEG	NEG	NEG	NEG	NEG
Trinidad&Tobago_MRSA2010_108	POS	POS	NEG	NEG	NEG	POS	NEG	NEG	POS	POS	NEG	NEG	NEG	NEG	NEG	NEG
Trinidad&Tobago_MRSA2010_33	POS	POS	NEG	NEG	NEG	POS	NEG	NEG	POS	NEG	NEG	NEG	NEG	NEG	NEG	AMB
Trinidad&Tobago_MRSA2010_79	POS	POS	NEG	NEG	NEG	POS	NEG	NEG	POS	NEG	NEG	NEG	NEG	NEG	NEG	NEG
Trinidad&Tobago_MRSA2010_88	POS	POS	NEG	NEG	NEG	POS	NEG	NEG	POS	AMB	NEG	NEG	NEG	NEG	NEG	NEG
Trinidad&Tobago_MRSA2010_97	POS	POS	NEG	NEG	NEG	POS	NEG	NEG	POS	AMB	NEG	NEG	NEG	NEG	NEG	NEG
Trinidad&Tobago_SSTI 2012_031	POS	POS	NEG	NEG	NEG	POS	NEG	NEG	POS	AMB	NEG	NEG	NEG	NEG	NEG	NEG
Uganda-29_17148_250811	POS	POS	NEG	NEG	NEG	POS	NEG	NEG	POS	NEG	NEG	NEG	NEG	NEG	NEG	NEG
>"South-East Asian Clad[illegible]																
UCIM6015	POS	POS	NEG	NEG	NEG	POS	NEG	NEG	POS	NEG	NEG	NEG	NEG	NEG	NEG	NEG
Hong Kong_121	POS	POS	NEG	NEG	NEG	POS	NEG	NEG	POS	NEG	NEG	NEG	NEG	NEG	NEG	NEG
>"South-East Asian Clad[illegible]																
Z172 (Taiwan)	POS	POS	NEG	NEG	NEG	POS	NEG	NEG	POS	NEG	NEG	NEG	NEG	NEG	NEG	NEG
Russia-19_0342_Kurgan	POS	POS	NEG	NEG	NEG	POS	NEG	NEG	POS	NEG	NEG	NEG	NEG	NEG	NEG	NEG
>"South-East Asian Clad[illegible]																
Perth_01-15392	POS	POS	NEG	NEG	NEG	POS	NEG	NEG	POS	NEG	NEG	NEG	NEG	NEG	NEG	NEG
>"South-East Asian Clad[illegible]																
XN108 (China)	POS	POS	NEG	NEG	NEG	POS	NEG	NEG	POS	NEG	NEG	NEG	NEG	NEG	NEG	NEG
NMR09 (India)	POS	POS	NEG	NEG	NEG	POS	NEG	NEG	POS	NEG	NEG	NEG	NEG	NEG	NEG	NEG
346 (Malaysia)	POS	POS	NEG	NEG	NEG	POS	NEG	NEG	POS	NEG	NEG	NEG	NEG	NEG	NEG	NEG
DS_009 (Thailand)	POS	POS	NEG	NEG	NEG	POS	NEG	NEG	POS	NEG	NEG	NEG	NEG	NEG	NEG	NEG
18ANR581005 (Ind)	POS	POS	NEG	NEG	NEG	POS	NEG	NEG	NEG	NEG	NEG	NEG	NEG	NEG	NEG	NEG
Bengalaru_ST20141405	POS	POS	NEG	NEG	NEG	POS	NEG	NEG	POS	NEG	NEG	NEG	NEG	NEG	NEG	NEG
Bengalaru_ST20141406	POS	POS	NEG	NEG	NEG	POS	NEG	NEG	POS	NEG	NEG	NEG	NEG	NEG	NEG	NEG
Bengalaru_ST20141417	POS	POS	NEG	NEG	NEG	POS	NEG	NEG	POS	NEG	NEG	NEG	NEG	NEG	NEG	NEG
Hong Kong_107	POS	POS	NEG	NEG	NEG	POS	NEG	NEG	POS	NEG	NEG	NEG	NEG	NEG	NEG	NEG
Hong Kong_12	POS	POS	NEG	NEG	NEG	POS	NEG	NEG	POS	NEG	NEG	NEG	NEG	NEG	NEG	NEG
Hong Kong_132	POS	POS	NEG	NEG	NEG	POS	NEG	NEG	POS	NEG	NEG	NEG	NEG	NEG	NEG	NEG
Riyadh-3028763-R	POS	POS	NEG	NEG	NEG	POS	NEG	NEG	POS	NEG	NEG	NEG	NEG	NEG	NEG	NEG
Trinidad&Tobago_MRSA2010_94	POS	POS	NEG	NEG	NEG	POS	NEG	NEG	POS	NEG	NEG	NEG	NEG	NEG	NEG	NEG
>"South-East Asian Clad[illegible]																
M705 (Thailand)	POS	POS	NEG	NEG	NEG	POS	NEG	NEG	POS	NEG	NEG	NEG	NEG	NEG	NEG	NEG
S85 (Thailand)	POS	POS	NEG	NEG	NEG	POS	NEG	NEG	POS	NEG	NEG	NEG	NEG	NEG	NEG	NEG
Riyadh_Alfaisal-04_281014_S5076	POS	POS	NEG	NEG	NEG	POS	POS	NEG	POS	POS	NEG	NEG	NEG	NEG	NEG	NEG
Riyadh_Alfaisal-26_S15145_1104163	POS	POS	NEG	NEG	NEG	POS	NEG	NEG	POS	NEG	NEG	NEG	NEG	NEG	NEG	NEG
Riyadh-2817437-W	POS	POS	NEG	NEG	NEG	POS	NEG	NEG	POS	AMB	NEG	NEG	NEG	NEG	NEG	NEG
>"South-East Asian Clad[illegible]																
PPUKM-775-2009 (Malaysia)	POS	POS	NEG	NEG	NEG	POS	NEG	NEG	POS	NEG	NEG	NEG	NEG	NEG	NEG	NEG
Hong Kong_138	POS	POS	NEG	NEG	NEG	POS	NEG	NEG	POS	NEG	NEG	NEG	NEG	NEG	NEG	NEG
>"South-East Asian Clad[illegible]																
Kuwait_001	POS	POS	NEG	NEG	NEG	POS	NEG	NEG	POS	NEG	NEG	NEG	NEG	NEG	NEG	NEG
Kuwait_337	POS	POS	NEG	NEG	NEG	POS	NEG	NEG	POS	POS	NEG	POS	POS	POS	NEG	NEG
>"South-East Asian Clad[illegible]																
CUHK_HK2007 (China)	POS	POS	NEG	NEG	NEG	POS	NEG	NEG	POS	NEG	NEG	NEG	NEG	NEG	NEG	NEG
NMR02 (India)	NEG	POS	NEG	NEG	NEG	AMB	NEG	NEG	POS	NEG	NEG	NEG	NEG	NEG	NEG	NEG
VB1490 (India)	AMB	POS	NEG	NEG	NEG	POS	NEG	NEG	POS	NEG	NEG	NEG	NEG	NEG	NEG	NEG
V521 (Korea)	POS	POS	NEG	NEG	NEG	POS	NEG	NEG	POS	NEG	NEG	NEG	NEG	NEG	NEG	NEG
HST-077 (Lebanon)	POS	POS	NEG	NEG	NEG	POS	NEG	NEG	POS	NEG	NEG	NEG	NEG	NEG	NEG	NEG
MAL1 (Malaysia)	POS	POS	NEG	NEG	NEG	POS	NEG	NEG	POS	NEG	NEG	NEG	NEG	NEG	NEG	NEG
MAL3 (Malaysia)	POS	POS	NEG	NEG	NEG	POS	NEG	NEG	POS	NEG	NEG	NEG	NEG	NEG	NEG	NEG
PPUKM-261-2009 (Malaysia)	POS	POS	NEG	NEG	NEG	POS	NEG	NEG	POS	NEG	NEG	NEG	NEG	NEG	NEG	NEG
PPUKM-332-2009 (Malaysia)	POS	POS	NEG	NEG	NEG	POS	NEG	NEG	POS	NEG	NEG	NEG	NEG	NEG	NEG	NEG
S38 (Thailand)	POS	POS	NEG	NEG	NEG	POS	NEG	NEG	POS	NEG	NEG	NEG	NEG	NEG	NEG	NEG
S7 (Thailand)	POS	POS	NEG	NEG	NEG	POS	NEG	NEG	POS	NEG	NEG	NEG	NEG	NEG	NEG	NEG
US_008 (Thailand)	POS	POS	NEG	NEG	NEG	POS	NEG	NEG	POS	NEG	NEG	NEG	NEG	NEG	NEG	NEG
US_030 (Thailand)	POS	POS	NEG	NEG	NEG	POS	NEG	NEG	POS	NEG	NEG	NEG	NEG	NEG	NEG	NEG
M170 (Tsunami-related)	POS	POS	NEG	NEG	NEG	POS	NEG	NEG	POS	NEG	NEG	NEG	NEG	NEG	NEG	NEG
BSAC697 (UK)	POS	POS	NEG	NEG	NEG	POS	NEG	NEG	POS	NEG	NEG	NEG	NEG	NEG	NEG	NEG
IS-189	POS	POS	NEG	NEG	NEG	POS	NEG	NEG	POS	NEG	NEG	NEG	NEG	NEG	NEG	NEG
MRGR3	POS	POS	NEG	NEG	NEG	POS	NEG	NEG	POS	NEG	NEG	NEG	NEG	NEG	NEG	NEG
smg07	NEG	POS	NEG	NEG	NEG	AMB	NEG	NEG	POS	NEG	NEG	NEG	NEG	NEG	NEG	NEG
AH1-AUS-EMRSA-3	POS	POS	NEG	NEG	NEG	POS	NEG	NEG	POS	NEG	NEG	NEG	NEG	NEG	NEG	NEG
Bengalaru_ST20141403	POS	POS	NEG	NEG	NEG	POS	NEG	NEG	POS	NEG	NEG	NEG	NEG	NEG	NEG	NEG
Finland E24_98541_Harmony	POS	POS	NEG	NEG	NEG	POS	NEG	NEG	POS	AMB	NEG	NEG	NEG	NEG	NEG	NEG
Hong Kong_10	POS	POS	NEG	NEG	NEG	POS	NEG	NEG	POS	AMB	NEG	NEG	NEG	NEG	NEG	NEG
Hong Kong_100	POS	POS	NEG	NEG	NEG	POS	NEG	NEG	POS	NEG	NEG	NEG	NEG	NEG	NEG	NEG
Hong Kong_199	POS	POS	NEG	NEG	NEG	POS	POS	NEG	POS	AMB	NEG	NEG	NEG	NEG	NEG	NEG
Hong Kong_34	POS	POS	NEG	NEG	NEG	POS	AMB	NEG	POS	AMB	NEG	NEG	NEG	NEG	NEG	NEG
Hong Kong_88	POS	POS	NEG	NEG	NEG	POS	POS	NEG	POS	POS	NEG	NEG	NEG	NEG	NEG	NEG
Hong Kong_92	POS	POS	NEG	NEG	NEG	POS	AMB	NEG	POS	AMB	NEG	NEG	NEG	NEG	NEG	NEG
Hong Kong_94	POS	POS	NEG	NEG	NEG	POS	NEG	NEG	POS	NEG	NEG	NEG	NEG	NEG	NEG	NEG
Kuwait_257	POS	POS	NEG	NEG	NEG	POS	NEG	NEG	POS	NEG	NEG	NEG	NEG	NEG	NEG	NEG
Perth_01-15330	POS	POS	NEG	NEG	NEG	POS	NEG	NEG	POS	NEG	NEG	NEG	NEG	NEG	NEG	NEG
Perth_01-15349	POS	POS	NEG	NEG	NEG	POS	NEG	NEG	POS	NEG	NEG	NEG	NEG	NEG	NEG	NEG
Perth_01-16176	POS	POS	NEG	NEG	NEG	POS	NEG	NEG	POS	NEG	NEG	NEG	NEG	NEG	NEG	NEG
Perth_WBG8379	POS	POS	NEG	NEG	NEG	POS	NEG	NEG	POS	NEG	NEG	NEG	NEG	NEG	NEG	NEG
Rawalpindi_Kidney Center_56	AMB	POS	NEG	NEG	NEG	POS	AMB	NEG	POS	AMB	AMB	NEG	NEG	NEG	NEG	NEG
Riyadh_SSTI_05_3464614	POS	POS	NEG	NEG	NEG	POS	NEG	NEG	POS	NEG	NEG	NEG	NEG	NEG	NEG	NEG
Trinidad&Tobago_2012_157	POS	POS	NEG	NEG	NEG	POS	NEG	NEG	POS	AMB	NEG	NEG	NEG	NEG	NEG	NEG

STRAIN / ISOLATE	TYPE I RESTR:-MODIFICATION SYSTEM								MISCELLANEOUS GENES							
	hsdS3					hsdSx			ear2	Q2YUB3	Q7A4X2	Q931R4	Q9RL82			cstB
	hsdS3-AllOtherThanRF122+252	hsdS3-ST8+ST1+RF122	hsdS3-Mu50+N315	hsdS3-CC51+252	hsdS3-MRSA252	hsdSx-CC25	hsdSx-CC15	hsdSx-etd	ear2 = Q2FXC0	Q2YUB3	Q7A4X2	Q931R4 (CC5, CC15, CC30, CC97, CC188, ST1850)	Q9RL82 (consensus)	Q9RL82 (CC8)	Q9RL82-CC10/361	Q2G1R6-genomic island / cstB
	type I site-specific deoxyribonuclease subunit, 3rd locus					type I site-specific deoxyribonuclease subunit, unknown locus			Putative protein	Multidrug resistance transporter	Putative protein	major facilitator superfamily transporter	Putative protein			
>"South-East Asian Clad																
M418	POS	POS	NEG	NEG	NEG	POS	NEG	NEG	POS	NEG	NEG	NEG	NEG	NEG	NEG	NEG
>"South-East Asian Clad																
NMR07 (India)	POS	POS	NEG	NEG	NEG	POS	NEG	NEG	POS	NEG	NEG	NEG	NEG	NEG	NEG	NEG
NMR08 (India)	POS	POS	NEG	NEG	NEG	POS	NEG	NEG	POS	NEG	NEG	NEG	NEG	NEG	NEG	NEG
Bengalaru_ST20121978	POS	POS	NEG	NEG	NEG	POS	NEG	NEG	POS	NEG	NEG	NEG	NEG	NEG	NEG	NEG
Hong Kong_89	POS	POS	NEG	NEG	NEG	POS	POS	NEG	POS	POS	NEG	NEG	NEG	NEG	NEG	NEG
>"South-East Asian Clad																
Perth_2005_AH_5	POS	POS	NEG	NEG	NEG	POS	NEG	NEG	POS	NEG	NEG	NEG	NEG	NEG	NEG	NEG
>"South-East Asian Clad																
Trinidad&Tobago_MRSA2010_103	POS	POS	NEG	NEG	NEG	POS	NEG	NEG	POS	AMB	NEG	NEG	NEG	NEG	NEG	NEG
Trinidad&Tobago_2013_PA25	POS	POS	NEG	NEG	NEG	POS	NEG	NEG	POS	NEG	NEG	NEG	NEG	NEG	NEG	NEG
Trinidad&Tobago_MRSA2010_110	POS	POS	NEG	NEG	NEG	POS	NEG	NEG	POS	AMB	NEG	NEG	NEG	NEG	NEG	NEG
Trinidad&Tobago_SSTI 2012_111	POS	POS	NEG	NEG	NEG	POS	NEG	NEG	POS	AMB	NEG	NEG	NEG	NEG	NEG	NEG
Trinidad&Tobago_MRSA2010_158	POS	POS	NEG	NEG	NEG	POS	NEG	NEG	POS	NEG	NEG	NEG	NEG	NEG	NEG	NEG
>"South-East Asian Clad																
Kuwait_122	POS	POS	NEG	NEG	NEG	POS	NEG	NEG	POS	NEG	NEG	NEG	NEG	NEG	NEG	NEG
Kuwait_002	POS	POS	NEG	NEG	NEG	POS	NEG	NEG	POS	NEG	NEG	NEG	NEG	NEG	NEG	NEG
Kuwait_196	POS	POS	NEG	NEG	NEG	POS	NEG	NEG	POS	NEG	NEG	NEG	NEG	NEG	NEG	NEG
Kuwait_199	POS	POS	NEG	NEG	NEG	POS	NEG	NEG	POS	AMB	NEG	NEG	NEG	NEG	NEG	NEG
Hong Kong_136	POS	POS	NEG	NEG	NEG	POS	NEG	NEG	POS	NEG	NEG	NEG	NEG	NEG	NEG	NEG
>"South-East Asian Clad																
P32 (Poland)	POS	POS	NEG	NEG	NEG	POS	NEG	NEG	POS	NEG	NEG	NEG	NEG	NEG	NEG	NEG
>"South-East Asian Clad																
Perth_2015-S32313	POS	POS	NEG	NEG	NEG	POS	NEG	NEG	POS	NEG	NEG	NEG	NEG	NEG	NEG	NEG
>"hla-negative South-Ea																
DEN907 (Denmark)	POS	POS	NEG	NEG	NEG	POS	NEG	NEG	POS	NEG	NEG	NEG	NEG	NEG	NEG	NEG
>"hla-negative South-Ea																
S26 (Thailand)	POS	POS	NEG	NEG	NEG	POS	NEG	NEG	POS	NEG	NEG	NEG	NEG	NEG	NEG	NEG
S42 (Thailand)	POS	POS	NEG	NEG	NEG	POS	NEG	NEG	POS	NEG	NEG	NEG	NEG	NEG	NEG	NEG
S97 (Thailand)	POS	POS	NEG	NEG	NEG	POS	NEG	NEG	POS	NEG	NEG	NEG	NEG	NEG	NEG	NEG
S78 (Thailand)	POS	POS	NEG	NEG	NEG	POS	NEG	NEG	POS	NEG	NEG	NEG	NEG	NEG	NEG	NEG
S25 (Thailand)	POS	POS	NEG	NEG	NEG	POS	NEG	NEG	POS	NEG	NEG	NEG	NEG	NEG	NEG	NEG
S21 (Thailand)	POS	POS	NEG	NEG	NEG	POS	NEG	NEG	POS	NEG	NEG	NEG	NEG	NEG	NEG	NEG
S106 (Thailand)	POS	POS	NEG	NEG	NEG	POS	NEG	NEG	POS	NEG	NEG	NEG	NEG	NEG	NEG	NEG
S24 (Thailand)	POS	POS	NEG	NEG	NEG	POS	NEG	NEG	POS	NEG	NEG	NEG	NEG	NEG	NEG	NEG
S39 (Thailand)	POS	POS	NEG	NEG	NEG	POS	NEG	NEG	POS	NEG	NEG	NEG	NEG	NEG	NEG	NEG
S2 (Thailand)	POS	POS	NEG	NEG	NEG	POS	NEG	NEG	POS	NEG	NEG	NEG	NEG	NEG	NEG	NEG
S81 (Thailand)	POS	POS	NEG	NEG	NEG	POS	NEG	NEG	POS	NEG	NEG	NEG	NEG	NEG	NEG	NEG
M116 (Vietnam)	POS	POS	NEG	NEG	NEG	POS	NEG	NEG	POS	NEG	NEG	NEG	NEG	NEG	NEG	NEG
>"Portuguese Clade": Pc																
FFP103 (Portugal)	POS	POS	NEG	NEG	NEG	POS	NEG	NEG	POS	NEG	NEG	NEG	NEG	NEG	NEG	NEG
HDG2 (Portugal)	POS	POS	NEG	NEG	NEG	POS	NEG	NEG	POS	NEG	NEG	NEG	NEG	NEG	NEG	NEG
HSA10 (Portugal)	POS	POS	NEG	NEG	NEG	POS	NEG	NEG	POS	NEG	NEG	NEG	NEG	NEG	NEG	NEG
HSA11 (Portugal)	POS	POS	NEG	NEG	NEG	POS	NEG	NEG	POS	NEG	NEG	NEG	NEG	NEG	NEG	NEG
ICP5011 (Portugal)	NEG	NEG	NEG	NEG	NEG	AMB	NEG	NEG	NEG	NEG	NEG	NEG	NEG	NEG	NEG	NEG
ICP5014 (Portugal)	POS	POS	NEG	NEG	NEG	POS	NEG	NEG	POS	NEG	NEG	NEG	NEG	NEG	NEG	NEG
ICP5062 (Portugal)	POS	POS	NEG	NEG	NEG	POS	NEG	NEG	POS	NEG	NEG	NEG	NEG	NEG	NEG	NEG
Strain 1063_ATCC 33592 FDAARGOS_3 (USA)	POS	POS	NEG	NEG	NEG	POS	NEG	NEG	POS	NEG	NEG	NEG	NEG	NEG	NEG	NEG
	POS	POS	NEG	NEG	NEG	POS	NEG	NEG	POS	NEG	NEG	NEG	NEG	NEG	NEG	NEG
Russia-07_0110_Kurgan	POS	POS	NEG	NEG	NEG	POS	NEG	NEG	POS	NEG	NEG	NEG	NEG	NEG	NEG	NEG
Russia-11_0164_Kurgan	POS	POS	NEG	NEG	NEG	POS	NEG	NEG	POS	NEG	NEG	NEG	NEG	NEG	NEG	NEG
Russia-20_0367_Kurgan	POS	POS	NEG	NEG	NEG	POS	NEG	NEG	POS	NEG	NEG	NEG	NEG	NEG	NEG	NEG
Russia-21_0390_Chelyabinsk	POS	POS	NEG	NEG	NEG	POS	NEG	NEG	POS	NEG	NEG	NEG	NEG	NEG	NEG	NEG
>"South American/ Mid																
AGT1 (Argentina)	POS	POS	NEG	NEG	NEG	POS	NEG	NEG	POS	NEG	NEG	NEG	NEG	NEG	NEG	NEG
AGT120 (Argentina)	POS	POS	NEG	NEG	NEG	POS	NEG	NEG	POS	NEG	NEG	NEG	NEG	NEG	NEG	NEG
AGT67 (Argentina)	POS	POS	NEG	NEG	NEG	POS	NEG	NEG	POS	NEG	NEG	NEG	NEG	NEG	NEG	NEG
AGT9 (Argentina)	POS	POS	NEG	NEG	NEG	POS	NEG	NEG	POS	NEG	NEG	NEG	NEG	NEG	NEG	NEG
RA3 (Argentina)	POS	POS	NEG	NEG	NEG	POS	NEG	NEG	POS	NEG	NEG	NEG	NEG	NEG	NEG	NEG
RA6 (Argentina)	POS	POS	NEG	NEG	NEG	POS	NEG	NEG	POS	NEG	NEG	NEG	NEG	NEG	NEG	NEG
RA7 (Argentina)	POS	POS	NEG	NEG	NEG	POS	NEG	NEG	POS	NEG	NEG	NEG	NEG	NEG	NEG	NEG
Be62 (Brazil)	POS	POS	NEG	NEG	NEG	POS	NEG	NEG	POS	NEG	NEG	NEG	NEG	NEG	NEG	NEG
Bmb9393 (Brazil)	POS	POS	NEG	NEG	NEG	POS	NEG	NEG	POS	NEG	NEG	NEG	NEG	NEG	NEG	NEG
BRA36 (Brazil)	POS	POS	NEG	NEG	NEG	POS	NEG	NEG	POS	NEG	NEG	NEG	NEG	NEG	NEG	NEG
BZ48 (Brazil)	POS	POS	NEG	NEG	NEG	POS	NEG	NEG	POS	NEG	NEG	NEG	NEG	NEG	NEG	NEG
Gv51 (Brazil)	POS	POS	NEG	NEG	NEG	POS	NEG	NEG	POS	NEG	NEG	NEG	NEG	NEG	NEG	NEG
Gv69 (Brazil)	POS	POS	NEG	NEG	NEG	POS	NEG	NEG	POS	NEG	NEG	NEG	NEG	NEG	NEG	NEG
Gv88 (Brazil)	POS	POS	NEG	NEG	NEG	POS	NEG	NEG	POS	NEG	NEG	NEG	NEG	NEG	NEG	NEG
HC1335 (Brazil)	POS	POS	NEG	NEG	NEG	POS	NEG	NEG	POS	NEG	NEG	NEG	NEG	NEG	NEG	NEG
HC1340 (Brazil)	POS	POS	NEG	NEG	NEG	POS	NEG	NEG	POS	NEG	NEG	NEG	NEG	NEG	NEG	NEG
HC556 (Brazil)	POS	POS	NEG	NEG	NEG	POS	NEG	NEG	POS	NEG	NEG	NEG	NEG	NEG	NEG	NEG
HU25 (Brazil)	POS	POS	NEG	NEG	NEG	POS	NEG	NEG	POS	NEG	NEG	NEG	NEG	NEG	NEG	NEG
UB563 (Brazil)	POS	POS	NEG	NEG	NEG	POS	NEG	NEG	POS	NEG	NEG	NEG	NEG	NEG	NEG	NEG
2A8 (Czech Republic)	POS	POS	NEG	NEG	NEG	POS	NEG	NEG	POS	NEG	NEG	NEG	NEG	NEG	NEG	NEG
M1229 (Denmark)	POS	POS	NEG	NEG	NEG	POS	NEG	NEG	POS	NEG	NEG	NEG	NEG	NEG	NEG	NEG
FRICAR (France)	POS	POS	NEG	NEG	NEG	POS	NEG	NEG	POS	NEG	NEG	NEG	NEG	NEG	NEG	NEG
D90 (Germany)	POS	POS	NEG	NEG	NEG	POS	NEG	NEG	POS	NEG	NEG	NEG	NEG	NEG	NEG	NEG
LIT68 (Lithuania)	POS	POS	NEG	NEG	NEG	POS	NEG	NEG	POS	NEG	NEG	NEG	NEG	NEG	NEG	NEG
LIT76 (Lithuania)	POS	POS	NEG	NEG	NEG	POS	NEG	NEG	POS	NEG	NEG	NEG	NEG	NEG	NEG	NEG
HGSA142 (Portugal)	POS	POS	NEG	NEG	NEG	POS	NEG	NEG	POS	NEG	NEG	NEG	NEG	NEG	NEG	NEG
HGSA9 (Portugal)	POS	POS	NEG	NEG	NEG	POS	NEG	NEG	POS	NEG	NEG	NEG	NEG	NEG	NEG	NEG
HSJ216 (Portugal)	POS	POS	NEG	NEG	NEG	POS	NEG	NEG	POS	NEG	NEG	NEG	NEG	NEG	NEG	NEG
M278 (Portugal)	POS	POS	NEG	NEG	NEG	POS	NEG	NEG	POS	NEG	NEG	NEG	NEG	NEG	NEG	NEG
ES26 (Spain)	POS	POS	NEG	NEG	NEG	POS	NEG	NEG	POS	NEG	NEG	NEG	NEG	NEG	NEG	NEG
UK102 (UK)	POS	POS	NEG	NEG	NEG	POS	NEG	NEG	POS	NEG	NEG	NEG	NEG	NEG	NEG	NEG
URU34 (Uruguay)	POS	POS	NEG	NEG	NEG	POS	NEG	NEG	POS	NEG	NEG	NEG	NEG	NEG	NEG	NEG
IS-125	POS	POS	NEG	NEG	NEG	POS	NEG	NEG	POS	NEG	NEG	NEG	NEG	NEG	NEG	NEG
IS-157	POS	POS	NEG	NEG	NEG	POS	NEG	NEG	POS	NEG	NEG	NEG	NEG	NEG	NEG	NEG
Ecuador_1	POS	POS	NEG	NEG	NEG	POS	NEG	NEG	POS	AMB	NEG	NEG	NEG	NEG	NEG	NEG
Ecuador_2	POS	POS	NEG	NEG	NEG	POS	NEG	NEG	POS	NEG	NEG	NEG	NEG	NEG	NEG	NEG
Dresden_15ANR574263	POS	POS	NEG	NEG	NEG	POS	NEG	NEG	POS	NEG	NEG	NEG	NEG	NEG	NEG	NEG
UK-EMRSA-11	POS	POS	NEG	NEG	NEG	POS	NEG	NEG	POS	NEG	NEG	NEG	NEG	NEG	NEG	NEG
>"South American/ Mid																
BRA2 (Brazil)	POS	POS	NEG	NEG	NEG	POS	NEG	NEG	POS	NEG	NEG	NEG	NEG	NEG	NEG	NEG
CHL1 (Chile)	POS	POS	NEG	NEG	NEG	POS	NEG	NEG	POS	NEG	NEG	NEG	NEG	NEG	NEG	NEG
CHL151 (Chile)	POS	POS	NEG	NEG	NEG	POS	NEG	NEG	POS	NEG	NEG	NEG	NEG	NEG	NEG	NEG
UCL378 (Chile)	POS	POS	NEG	NEG	NEG	POS	NEG	NEG	POS	NEG	NEG	NEG	NEG	NEG	NEG	NEG
UP72 (Peru)	POS	POS	NEG	NEG	NEG	POS	NEG	NEG	POS	NEG	NEG	NEG	NEG	NEG	NEG	NEG
UP81 (Peru)	POS	POS	NEG	NEG	NEG	POS	NEG	NEG	POS	NEG	NEG	NEG	NEG	NEG	NEG	NEG
>"South American/ Mid																
UP1073 (Peru)	POS	POS	NEG	NEG	NEG	POS	NEG	NEG	POS	NEG	NEG	NEG	NEG	NEG	NEG	NEG

STRAIN / ISOLATE	TYPE I RESTR:-MODIFICATION SYSTEM								MISCELLANEOUS GENES							
	hsdS3					hsdSx			ear2	Q2YUB3	Q7A4X2	Q931R4	Q9RL82			cstB
	hsdS3-AllOtherThanRF122+252	hsdS3-ST8+ST1+RF122	hsdS3-Mu50+N315	hsdS3-CC51+252	hsdS3-MRSA252	hsdSx-CC25	hsdSx-CC15	hsdSx-etd	ear2 = Q2FXC0	Q2YUB3	Q7A4X2	Q931R4 (CC5, CC15, CC30, CC97, CC188, ST1850)	Q9RL82 (consensus)	Q9RL82 (CC8)	Q9RL82-CC10/361	Q2G1R6-genomic island / cstB
	type I site-specific deoxyribonuclease subunit, 3rd locus					type I site-specific deoxyribonuclease subunit, unknown locus			Putative protein	Multidrug resistance transporter	Putative protein	major facilitator superfamily transporter	Putative protein			
>"South American/ Mid																
LIT89 (Lithuania)	POS	POS	NEG	NEG	NEG	POS	NEG	NEG	POS	NEG	NEG	NEG	NEG	NEG	NEG	NEG
MRSA_PR1 (Malaysia)	POS	POS	NEG	NEG	NEG	POS	NEG	NEG	POS	NEG	NEG	NEG	NEG	NEG	NEG	NEG
Riyadh-2888905-R	POS	POS	NEG	NEG	NEG	POS	NEG	NEG	POS	NEG	NEG	NEG	NEG	NEG	NEG	NEG
Riyadh-2888915-BC	POS	POS	NEG	NEG	NEG	POS	NEG	NEG	POS	NEG	NEG	NEG	NEG	NEG	NEG	NEG
Riyadh-2793706-R	POS	POS	NEG	NEG	NEG	POS	NEG	NEG	POS	NEG	NEG	NEG	NEG	NEG	NEG	NEG
>"South American/ Mid																
Riyadh_SSTI_18_3502925	POS	POS	NEG	NEG	NEG	POS	NEG	NEG	POS	POS	NEG	NEG	NEG	NEG	NEG	NEG
Riyadh-2822825-W	POS	POS	NEG	NEG	NEG	POS	NEG	NEG	POS	POS	NEG	NEG	NEG	NEG	NEG	NEG
>"South American/ Mid																
UK-EMRSA-9	POS	POS	NEG	NEG	NEG	POS	NEG	NEG	POS	NEG	NEG	NEG	NEG	NEG	NEG	NEG
>"South American/ Mid																
Lome_HT20020815	POS	POS	NEG	NEG	NEG	POS	NEG	NEG	POS	NEG	NEG	NEG	NEG	NEG	NEG	NEG
>"South American/ Mid																
NA32 (Denmark)	POS	POS	NEG	NEG	NEG	POS	NEG	NEG	POS	NEG	NEG	NEG	NEG	NEG	NEG	NEG
H24 (Egypt)	POS	POS	NEG	NEG	NEG	POS	NEG	NEG	POS	NEG	NEG	NEG	NEG	NEG	NEG	NEG
ATCC BAA-39 (=HUSA304) (Hungary)	POS	POS	NEG	NEG	NEG	POS	NEG	NEG	POS	NEG	NEG	NEG	NEG	NEG	NEG	NEG
HUSA304 (Hungary)	POS	POS	NEG	NEG	NEG	POS	NEG	NEG	POS	NEG	NEG	NEG	NEG	NEG	NEG	NEG
HU106 (Hungary)	POS	POS	NEG	NEG	NEG	POS	NEG	NEG	POS	NEG	NEG	NEG	NEG	NEG	NEG	NEG
BSAC27 (UK)	POS	POS	NEG	NEG	NEG	POS	NEG	NEG	POS	NEG	NEG	NEG	NEG	NEG	NEG	NEG
NCTC13131, UK-EMRSA-4 (UK)	POS	POS	NEG	NEG	NEG	POS	NEG	NEG	POS	NEG	NEG	NEG	NEG	NEG	NEG	NEG
	POS	POS	NEG	NEG	NEG	POS	NEG	NEG	POS	NEG	NEG	NEG	AMB	NEG	NEG	NEG
Algiers_HT20040080	POS	POS	NEG	NEG	NEG	POS	NEG	NEG	POS	NEG	NEG	NEG	NEG	NEG	NEG	NEG
Dublin-DSH_AR09_0.0066	POS	POS	NEG	NEG	NEG	POS	NEG	NEG	POS	NEG	NEG	NEG	NEG	NEG	NEG	NEG
Dublin-DSH_AR09_0-0065	POS	POS	NEG	NEG	NEG	POS	NEG	NEG	POS	NEG	NEG	NEG	NEG	NEG	NEG	NEG
Dublin-DSH_Phenotype-III_84	POS	POS	NEG	NEG	NEG	POS	NEG	NEG	POS	NEG	NEG	NEG	NEG	NEG	NEG	NEG
Hong Kong_130	POS	POS	NEG	NEG	NEG	POS	NEG	NEG	POS	NEG	NEG	NEG	NEG	NEG	NEG	NEG
Kuwait_018	POS	POS	NEG	NEG	NEG	POS	NEG	NEG	POS	NEG	NEG	NEG	NEG	NEG	NEG	NEG
Perth_08-17726	POS	POS	NEG	NEG	NEG	POS	NEG	NEG	POS	NEG	NEG	NEG	NEG	NEG	NEG	NEG
Riyadh_Alfaisal/KKKSUH_86_MRS-14-279	POS	POS	NEG	NEG	NEG	POS	NEG	NEG	POS	NEG	NEG	NEG	NEG	NEG	NEG	NEG
Riyadh_Alfaisal-04_Z3861_831588	POS	POS	NEG	NEG	NEG	POS	NEG	NEG	POS	NEG	NEG	NEG	NEG	NEG	NEG	NEG
Riyadh_Alfaisal-30_S15724_1108013	POS	POS	NEG	NEG	NEG	POS	NEG	NEG	POS	NEG	NEG	NEG	NEG	NEG	NEG	NEG
Riyadh_Alfaisal-6_22A_13_B3992_397721	POS	POS	NEG	NEG	NEG	POS	NEG	NEG	POS	NEG	NEG	NEG	NEG	NEG	NEG	NEG
Riyadh_SSTI_52_3615482	POS	POS	NEG	NEG	NEG	POS	NEG	NEG	POS	NEG	NEG	NEG	NEG	NEG	NEG	NEG
Riyadh-2817276-2	POS	POS	NEG	NEG	NEG	POS	NEG	NEG	POS	AMB	NEG	NEG	NEG	NEG	NEG	NEG
Riyadh-2891670-W	POS	POS	NEG	NEG	NEG	POS	NEG	NEG	POS	AMB	NEG	NEG	NEG	NEG	NEG	NEG
Riyadh-3006920-W	POS	POS	NEG	NEG	NEG	POS	NEG	NEG	POS	AMB	NEG	NEG	NEG	NEG	NEG	NEG
Riyadh-R2567782	POS	POS	NEG	NEG	NEG	POS	NEG	NEG	POS	NEG	NEG	NEG	AMB	NEG	NEG	NEG
Russia-18_0252_Krasnoyarsk_SK2	POS	POS	NEG	NEG	NEG	POS	NEG	NEG	POS	NEG	NEG	NEG	NEG	NEG	NEG	NEG
UK-EMRSA-7	POS	POS	NEG	NEG	NEG	POS	NEG	NEG	POS	NEG	NEG	NEG	AMB	NEG	NEG	NEG
>"South American/ Mid																
Dublin-DSH_AR23_0073	POS	POS	NEG	NEG	NEG	POS	NEG	NEG	POS	NEG	NEG	NEG	NEG	NEG	NEG	NEG
>Related to "South Ame																
MRSA-OC3 (Russia)	POS	POS	NEG	NEG	NEG	POS	NEG	NEG	POS	NEG	NEG	NEG	NEG	NEG	NEG	NEG
Russia-12_0176_Krasnoyarsk	POS	POS	NEG	NEG	NEG	POS	NEG	NEG	POS	NEG	NEG	NEG	NEG	NEG	NEG	NEG
Russia-13_0180_Krasnoyarsk	POS	POS	NEG	NEG	NEG	POS	NEG	NEG	POS	NEG	NEG	NEG	NEG	NEG	NEG	NEG
Russia-16_0249_Krasnoyarsk	POS	POS	NEG	NEG	NEG	POS	NEG	NEG	POS	NEG	NEG	NEG	NEG	NEG	NEG	NEG
Russia-17_0250_Krasnoyarsk	POS	POS	NEG	NEG	NEG	POS	NEG	NEG	POS	NEG	NEG	NEG	NEG	NEG	NEG	NEG
>Related to "South Ame																
URU110 (Uruguay)	POS	POS	NEG	NEG	NEG	POS	NEG	NEG	POS	NEG	NEG	NEG	NEG	NEG	NEG	NEG
>Related to "South Ame																
DS_014 (Thailand)	POS	POS	NEG	NEG	NEG	POS	NEG	NEG	POS	NEG	NEG	NEG	NEG	NEG	NEG	NEG
>Unassigned Middle Eas																
Frankfurt_Oder_0490031797 (Libya)	POS	POS	NEG	NEG	NEG	POS	NEG	NEG	POS	NEG	NEG	NEG	NEG	NEG	NEG	NEG
Kuwait_192	POS	POS	NEG	NEG	NEG	POS	NEG	NEG	POS	AMB	NEG	NEG	NEG	NEG	NEG	NEG
Riyadh-9	POS	POS	NEG	NEG	NEG	POS	NEG	NEG	POS	NEG	NEG	NEG	NEG	NEG	NEG	NEG
Russia-24_0407_Moscow	POS	POS	NEG	NEG	NEG	POS	NEG	NEG	POS	NEG	NEG	NEG	NEG	NEG	NEG	NEG

STRAIN / ISOLATE	MISCELLANEOUS GENES															
	sau				sau96I	G7ZRU6	ycjY	sagD	G7ZTC1		sdrM					
	sau3AI	sauUSI	sauRF122	sauSO385	sau96I	G7ZRU6	ycjY = C5Q1F1 ("Argenteus/ST1850-like", CC12, CC361, CC398)	sagD	G7ZTC1	G7ZTC1-argenteus	sdrM / tetEfflux	sdrM (cons)	hp_sdrM-801	hp_sdrM-802	sdrM (CC30)	sdrM (argen-teus)
	type II restriction-modification system endonuclease					acetyltransferase, GNAT family, "Argenteus/ST1850-like"		Putative bacteriocin biosynthesis associated protein	TetR family regulator		Multidrug resistance transporter					
**>"Eurasian Clade": TUR1**																
TUR1 (Turkey)	NEG	POS	NEG	NEG	NEG	NEG	NEG	NEG	NEG	NEG	POS	NEG	POS	NEG	NEG	NEG
TUR9 (Turkey)	NEG	POS	NEG	NEG	NEG	NEG	NEG	NEG	NEG	NEG	POS	NEG	POS	NEG	NEG	NEG
**>"Eurasian Clade" : T013**																
T0131 (China)	NEG	POS	NEG	NEG	NEG	NEG	NEG	NEG	NEG	NEG	POS	NEG	POS	NEG	NEG	NEG
MU4 (Turkey)	NEG	POS	NEG	NEG	NEG	NEG	NEG	NEG	NEG	NEG	POS	NEG	POS	NEG	NEG	NEG
DEU11 (Turkey)	NEG	POS	NEG	NEG	NEG	NEG	NEG	NEG	NEG	NEG	POS	NEG	POS	NEG	NEG	NEG
DEU16 (Turkey)	NEG	POS	NEG	NEG	NEG	NEG	NEG	NEG	NEG	NEG	POS	NEG	POS	NEG	NEG	NEG
Romania_Iasi_MRSA-07_54	NEG	POS	NEG	NEG	NEG	NEG	NEG	NEG	NEG	NEG	POS	POS	POS	POS	NEG	NEG
**>"Eurasian Clade": arsC-**																
Romania_Iasi_MRSA-05_46	NEG	POS	NEG	NEG	NEG	NEG	NEG	NEG	NEG	NEG	POS	POS	POS	POS	NEG	NEG
Romania_Iasi_MRSA-06_47	NEG	POS	NEG	NEG	NEG	NEG	NEG	NEG	NEG	NEG	POS	POS	POS	POS	NEG	NEG
**>"Eurasian Clade": 16K/**																
CN79 (China)	NEG	POS	NEG	NEG	NEG	NEG	NEG	NEG	NEG	NEG	POS	NEG	POS	NEG	NEG	NEG
CUHK_BJ2002 (China)	NEG	POS	NEG	NEG	NEG	NEG	NEG	NEG	NEG	NEG	POS	NEG	POS	NEG	NEG	NEG
CUHK_BJ2007 (China)	NEG	POS	NEG	NEG	NEG	NEG	NEG	NEG	NEG	NEG	POS	NEG	POS	NEG	NEG	NEG
3HK (Czech Republic)	NEG	POS	NEG	NEG	NEG	NEG	NEG	NEG	NEG	NEG	POS	NEG	POS	NEG	NEG	NEG
HU109 (Hungary)	NEG	POS	NEG	NEG	NEG	NEG	NEG	NEG	NEG	NEG	POS	NEG	POS	NEG	NEG	NEG
HUR18 (Hungary)	NEG	POS	NEG	NEG	NEG	NEG	NEG	NEG	NEG	NEG	POS	NEG	POS	NEG	NEG	NEG
H482 (Romania)	NEG	POS	NEG	NEG	NEG	NEG	NEG	NEG	NEG	NEG	POS	NEG	POS	NEG	NEG	NEG
16K (Russia)	NEG	POS	NEG	NEG	NEG	NEG	NEG	NEG	NEG	NEG	POS	NEG	POS	NEG	NEG	NEG
DEU10 (Turkey)	NEG	POS	NEG	NEG	NEG	NEG	NEG	NEG	NEG	NEG	POS	NEG	POS	NEG	NEG	NEG
DEU12 (Turkey)	NEG	POS	NEG	NEG	NEG	NEG	NEG	NEG	NEG	NEG	POS	NEG	POS	NEG	NEG	NEG
DEU14 (Turkey)	NEG	POS	NEG	NEG	NEG	NEG	NEG	NEG	NEG	NEG	POS	NEG	POS	NEG	NEG	NEG
DEU15 (Turkey)	NEG	POS	NEG	NEG	NEG	NEG	NEG	NEG	NEG	NEG	POS	NEG	POS	NEG	NEG	NEG
DEU17 (Turkey)	NEG	POS	NEG	NEG	NEG	NEG	NEG	NEG	NEG	NEG	POS	NEG	POS	NEG	NEG	NEG
DEU19 (Turkey)	NEG	POS	NEG	NEG	NEG	NEG	NEG	NEG	NEG	NEG	POS	NEG	POS	NEG	NEG	NEG
DEU2 (Turkey)	NEG	POS	NEG	NEG	NEG	NEG	NEG	NEG	NEG	NEG	POS	NEG	POS	NEG	NEG	NEG
DEU20 (Turkey)	NEG	POS	NEG	NEG	NEG	NEG	NEG	NEG	NEG	NEG	POS	NEG	POS	NEG	NEG	NEG
DEU23 (Turkey)	NEG	POS	NEG	NEG	NEG	NEG	NEG	NEG	NEG	NEG	POS	NEG	POS	NEG	NEG	NEG
DEU3 (Turkey)	NEG	POS	NEG	NEG	NEG	NEG	NEG	NEG	NEG	NEG	POS	NEG	POS	NEG	NEG	NEG
DEU5 (Turkey)	NEG	POS	NEG	NEG	NEG	NEG	NEG	NEG	NEG	NEG	POS	NEG	POS	NEG	NEG	NEG
DEU6 (Turkey)	NEG	POS	NEG	NEG	NEG	NEG	NEG	NEG	NEG	NEG	POS	NEG	POS	NEG	NEG	NEG
DEU8 (Turkey)	NEG	POS	NEG	NEG	NEG	NEG	NEG	NEG	NEG	NEG	POS	NEG	POS	NEG	NEG	NEG
HU11 (Turkey)	NEG	POS	NEG	NEG	NEG	NEG	NEG	NEG	NEG	NEG	POS	NEG	POS	NEG	NEG	NEG
HU13 (Turkey)	NEG	POS	NEG	NEG	NEG	NEG	NEG	NEG	NEG	NEG	POS	NEG	POS	NEG	NEG	NEG
HU14 (Turkey)	NEG	POS	NEG	NEG	NEG	NEG	NEG	NEG	NEG	NEG	POS	NEG	POS	NEG	NEG	NEG
HU15 (Turkey)	NEG	POS	NEG	NEG	NEG	NEG	NEG	NEG	NEG	NEG	POS	NEG	POS	NEG	NEG	NEG
HU16 (Turkey)	NEG	POS	NEG	NEG	NEG	NEG	NEG	NEG	NEG	NEG	POS	NEG	POS	NEG	NEG	NEG
HU17 (Turkey)	NEG	POS	NEG	NEG	NEG	NEG	NEG	NEG	NEG	NEG	POS	NEG	POS	NEG	NEG	NEG
HU21 (Turkey)	NEG	POS	NEG	NEG	NEG	NEG	NEG	NEG	NEG	NEG	POS	NEG	POS	NEG	NEG	NEG
HU23 (Turkey)	NEG	POS	NEG	NEG	NEG	NEG	NEG	NEG	NEG	NEG	POS	NEG	POS	NEG	NEG	NEG
HU26 (Turkey)	NEG	POS	NEG	NEG	NEG	NEG	NEG	NEG	NEG	NEG	POS	NEG	POS	NEG	NEG	NEG
HU4 (Turkey)	NEG	POS	NEG	NEG	NEG	NEG	NEG	NEG	NEG	NEG	POS	NEG	POS	NEG	NEG	NEG
HU5 (Turkey)	NEG	POS	NEG	NEG	NEG	NEG	NEG	NEG	NEG	NEG	POS	NEG	POS	NEG	NEG	NEG
HU6 (Turkey)	NEG	POS	NEG	NEG	NEG	NEG	NEG	NEG	NEG	NEG	POS	NEG	POS	NEG	NEG	NEG
HU7 (Turkey)	NEG	POS	NEG	NEG	NEG	NEG	NEG	NEG	NEG	NEG	POS	NEG	POS	NEG	NEG	NEG
HU8 (Turkey)	NEG	POS	NEG	NEG	NEG	NEG	NEG	NEG	NEG	NEG	POS	NEG	POS	NEG	NEG	NEG
HU9 (Turkey)	NEG	POS	NEG	NEG	NEG	NEG	NEG	NEG	NEG	NEG	POS	NEG	POS	NEG	NEG	NEG
IU1 (Turkey)	NEG	POS	NEG	NEG	NEG	NEG	NEG	NEG	NEG	NEG	POS	NEG	POS	NEG	NEG	NEG
IU10 (Turkey)	NEG	POS	NEG	NEG	NEG	NEG	NEG	NEG	NEG	NEG	POS	NEG	POS	NEG	NEG	NEG
IU11 (Turkey)	NEG	POS	NEG	NEG	NEG	NEG	NEG	NEG	NEG	NEG	POS	NEG	POS	NEG	NEG	NEG
IU12 (Turkey)	NEG	POS	NEG	NEG	NEG	NEG	NEG	NEG	NEG	NEG	POS	NEG	POS	NEG	NEG	NEG
IU13 (Turkey)	NEG	POS	NEG	NEG	NEG	NEG	NEG	NEG	NEG	NEG	POS	NEG	POS	NEG	NEG	NEG
IU15 (Turkey)	NEG	POS	NEG	NEG	NEG	NEG	NEG	NEG	NEG	NEG	POS	NEG	POS	NEG	NEG	NEG
IU18 (Turkey)	NEG	POS	NEG	NEG	NEG	NEG	NEG	NEG	NEG	NEG	POS	NEG	POS	NEG	NEG	NEG
IU19 (Turkey)	NEG	POS	NEG	NEG	NEG	NEG	NEG	NEG	NEG	NEG	POS	NEG	POS	NEG	NEG	NEG
IU2 (Turkey)	NEG	POS	NEG	NEG	NEG	NEG	NEG	NEG	NEG	NEG	POS	NEG	POS	NEG	NEG	NEG
IU4 (Turkey)	NEG	POS	NEG	NEG	NEG	NEG	NEG	NEG	NEG	NEG	POS	NEG	POS	NEG	NEG	NEG
IU6 (Turkey)	NEG	POS	NEG	NEG	NEG	NEG	NEG	NEG	NEG	NEG	POS	NEG	POS	NEG	NEG	NEG
IU7 (Turkey)	NEG	POS	NEG	NEG	NEG	NEG	NEG	NEG	NEG	NEG	POS	NEG	POS	NEG	NEG	NEG
IU9 (Turkey)	NEG	POS	NEG	NEG	NEG	NEG	NEG	NEG	NEG	NEG	POS	NEG	POS	NEG	NEG	NEG
MU1 (Turkey)	NEG	POS	NEG	NEG	NEG	NEG	NEG	NEG	NEG	NEG	POS	NEG	POS	NEG	NEG	NEG
MU10 (Turkey)	NEG	POS	NEG	NEG	NEG	NEG	NEG	NEG	NEG	NEG	POS	NEG	POS	NEG	NEG	NEG
MU20 (Turkey)	NEG	POS	NEG	NEG	NEG	NEG	NEG	NEG	NEG	NEG	POS	NEG	POS	NEG	NEG	NEG
Mu3 (Turkey)	NEG	POS	NEG	NEG	NEG	NEG	NEG	NEG	NEG	NEG	POS	NEG	POS	NEG	NEG	NEG
MU5 (Turkey)	NEG	POS	NEG	NEG	NEG	NEG	NEG	NEG	NEG	NEG	POS	NEG	POS	NEG	NEG	NEG
MU6 (Turkey)	NEG	POS	NEG	NEG	NEG	NEG	NEG	NEG	NEG	NEG	POS	NEG	POS	NEG	NEG	NEG
MU7 (Turkey)	NEG	POS	NEG	NEG	NEG	NEG	NEG	NEG	NEG	NEG	POS	NEG	POS	NEG	NEG	NEG
TUR27 (Turkey)	NEG	POS	NEG	NEG	NEG	NEG	NEG	NEG	NEG	NEG	POS	NEG	POS	NEG	NEG	NEG
NCTR 32S	NEG	POS	NEG	NEG	NEG	NEG	NEG	NEG	NEG	NEG	POS	NEG	POS	NEG	NEG	NEG
SA02_A4 = Russia-06_0085_Moscow	NEG	POS	NEG	NEG	NEG	NEG	NEG	NEG	NEG	NEG	POS	NEG	POS	NEG	NEG	NEG
	NEG	POS	NEG	NEG	NEG	NEG	NEG	NEG	NEG	NEG	POS	POS	POS	POS	NEG	NEG
Dresden_08V35987 (Turkey)	NEG	POS	NEG	NEG	NEG	NEG	NEG	NEG	NEG	NEG	POS	POS	POS	POS	NEG	NEG
Dresden_17ANR577152	NEG	POS	NEG	NEG	NEG	NEG	NEG	NEG	NEG	NEG	POS	POS	POS	POS	NEG	NEG
Dresden_17ANR580374 (Makedonia)	NEG	POS	NEG	NEG	NEG	NEG	NEG	NEG	NEG	NEG	POS	POS	POS	POS	NEG	NEG
Hong Kong_69-II	NEG	POS	NEG	NEG	NEG	NEG	AMB	NEG	NEG	NEG	POS	POS	POS	AMB	NEG	NEG
Hong Kong_93	NEG	POS	NEG	NEG	NEG	NEG	NEG	NEG	NEG	NEG	POS	POS	POS	POS	NEG	NEG
Rawalpindi_Kidney Center_03_SK1	NEG	POS	NEG	NEG	NEG	NEG	NEG	NEG	NEG	NEG	POS	POS	POS	POS	NEG	NEG
Rawalpindi_Kidney Center_08	NEG	POS	NEG	NEG	NEG	NEG	NEG	NEG	NEG	NEG	POS	POS	POS	POS	NEG	NEG
Rawalpindi_Kidney Center_10	NEG	POS	NEG	NEG	NEG	NEG	NEG	NEG	NEG	NEG	POS	POS	POS	POS	NEG	NEG
Rawalpindi_Kidney Center_19	NEG	POS	NEG	NEG	NEG	NEG	NEG	NEG	NEG	NEG	POS	POS	POS	POS	NEG	NEG
Rawalpindi_Kidney Center_50_SK1	NEG	POS	NEG	NEG	NEG	NEG	NEG	NEG	NEG	NEG	POS	POS	POS	POS	NEG	NEG
Romania_Iasi_BC-49_430	NEG	POS	NEG	NEG	NEG	NEG	NEG	NEG	NEG	NEG	POS	POS	POS	NEG	NEG	NEG
Romania_Iasi_MRSA_284	NEG	NEG	NEG	NEG	NEG	NEG	NEG	NEG	NEG	NEG	POS	NEG	NEG	NEG	NEG	NEG
Romania_Iasi_MRSA-32_318	NEG	POS	#ZAHL!	#ZAHL!	NEG	NEG	NEG	NEG	NEG	NEG	POS	POS	POS	POS	NEG	NEG
Romania_Iasi_MRSA-54_430	NEG	POS	NEG	NEG	NEG	NEG	NEG	NEG	NEG	NEG	POS	POS	POS	POS	NEG	NEG
Romania_Iasi_SSTI-01_2	NEG	POS	NEG	NEG	NEG	NEG	NEG	NEG	NEG	NEG	POS	POS	POS	POS	NEG	NEG
Romania_Iasi_SSTI-10_101	NEG	POS	NEG	NEG	NEG	NEG	NEG	NEG	NEG	NEG	POS	POS	POS	POS	NEG	NEG
Romania_Iasi_SSTI-11_106	NEG	POS	NEG	NEG	NEG	NEG	NEG	NEG	NEG	NEG	POS	POS	POS	POS	NEG	NEG
Russia-01_0001_SaintPetersburg	NEG	POS	NEG	NEG	NEG	NEG	NEG	NEG	NEG	NEG	POS	POS	POS	AMB	NEG	NEG
Russia-02_0004_SaintPetersburg	NEG	POS	NEG	NEG	NEG	NEG	NEG	NEG	NEG	NEG	POS	POS	POS	POS	NEG	NEG
Russia-03_0057_SaintPetersburg	NEG	POS	NEG	NEG	NEG	NEG	NEG	NEG	NEG	NEG	POS	POS	POS	NEG	NEG	NEG
Russia-04_0076_Moscow	NEG	POS	NEG	NEG	NEG	NEG	NEG	NEG	NEG	NEG	POS	POS	POS	NEG	NEG	NEG
Russia-05_0078_Moscow	NEG	POS	NEG	NEG	NEG	NEG	NEG	NEG	NEG	NEG	POS	POS	POS	AMB	NEG	NEG
Russia-08_0135_Moscow	NEG	POS	NEG	NEG	NEG	NEG	NEG	NEG	NEG	NEG	POS	POS	POS	AMB	NEG	NEG
Russia-09_0150_Kurgan	NEG	POS	NEG	NEG	NEG	NEG	NEG	NEG	NEG	NEG	POS	POS	POS	AMB	NEG	NEG
Russia-10_0162_Kurgan	NEG	POS	NEG	NEG	NEG	NEG	NEG	NEG	NEG	NEG	POS	POS	POS	AMB	NEG	NEG
Russia-14_0184_Moscow	NEG	AMB	NEG	NEG	NEG	NEG	NEG	NEG	NEG	NEG	POS	NEG	POS	NEG	NEG	NEG
Russia-15_0232_SaintPetersburg	NEG	POS	NEG	NEG	NEG	NEG	NEG	NEG	NEG	NEG	POS	AMB	POS	NEG	NEG	NEG
Russia-22_0391_Chelyabinsk	NEG	POS	NEG	NEG	NEG	NEG	NEG	NEG	NEG	NEG	POS	POS	POS	AMB	NEG	NEG
Russia-23_0392_Chelyabinsk	NEG	POS	NEG	NEG	NEG	NEG	NEG	NEG	NEG	NEG	POS	POS	POS	NEG	NEG	NEG
**>"Eurasian Clade" : IU17**																
IU17 (Turkey)	NEG	POS	NEG	NEG	NEG	NEG	NEG	NEG	NEG	NEG	POS	NEG	POS	NEG	NEG	NEG
IU20 (Turkey)	NEG	POS	NEG	NEG	NEG	NEG	NEG	NEG	NEG	NEG	POS	NEG	POS	NEG	NEG	NEG

STRAIN / ISOLATE	MISCELLANEOUS GENES															
	sau				sau96I	G7ZRU6	ycjY	sagD	G7ZTC1		sdrM					
	sau3AI	sauUSI	sauRF122	sauSO385	sau96I	G7ZRU6	ycjY = C5Q1F1 ("Argenteus/ST1850-like", CC12, CC361, CC398)	sagD	G7ZTC1	G7ZTC1-argenteus	sdrM / tetEfflux	sdrM (cons)	hp_sdrM-801	hp_sdrM-802	sdrM (CC30)	sdrM (argen-teus)
	type II restriction-modification system endonuclease					acetyltransferase, GNAT family, "Argenteus/ST1850-like"		Putative bacteriocin biosynthesis associated protein	TetR family regulator		Multidrug resistance transporter					
**>"Eurasian Clade": Spor[illegible]**																
Dresden_16ANR581769	NEG	POS	NEG	NEG	NEG	NEG	NEG	NEG	NEG	NEG	POS	POS	POS	POS	NEG	NEG
Dresden_17ANR578769	NEG	POS	NEG	NEG	NEG	NEG	NEG	NEG	NEG	NEG	POS	POS	POS	NEG	NEG	NEG
**>"Eurasian Clade": Kuw[illegible]**																
Kuwait_306	NEG	POS	NEG	NEG	NEG	NEG	NEG	NEG	NEG	NEG	POS	POS	POS	NEG	NEG	NEG
Kuwait_301	NEG	POS	NEG	NEG	NEG	NEG	NEG	NEG	NEG	NEG	POS	POS	POS	POS	NEG	NEG
**>"European Clade": "Gr[illegible]**																
GRE18 (Greece)	NEG	POS	NEG	NEG	NEG	NEG	NEG	NEG	NEG	NEG	POS	NEG	POS	NEG	NEG	NEG
GRE317 (Greece)	NEG	POS	NEG	NEG	NEG	NEG	NEG	NEG	NEG	NEG	POS	NEG	POS	NEG	NEG	NEG
GRE4 (Greece)	NEG	POS	NEG	NEG	NEG	NEG	NEG	NEG	NEG	NEG	POS	NEG	POS	NEG	NEG	NEG
R35 (USA)	NEG	POS	NEG	NEG	NEG	NEG	NEG	NEG	NEG	NEG	POS	NEG	POS	NEG	NEG	NEG
Casablanca_HT20060548	NEG	POS	NEG	NEG	NEG	NEG	NEG	NEG	NEG	NEG	POS	POS	POS	POS	NEG	NEG
Casablanca_HT20060550	NEG	POS	#ZAHL!	#ZAHL!	NEG	NEG	NEG	NEG	NEG	NEG	POS	POS	POS	POS	NEG	NEG
Dresden_01V34572 (Greece)	NEG	POS	NEG	NEG	NEG	NEG	NEG	NEG	NEG	NEG	POS	POS	POS	POS	NEG	NEG
Dresden_01V36469	NEG	POS	NEG	NEG	NEG	NEG	NEG	NEG	NEG	NEG	POS	POS	POS	POS	NEG	NEG
Dresden_01V39123	NEG	POS	NEG	NEG	NEG	NEG	NEG	NEG	NEG	NEG	POS	POS	POS	POS	NEG	NEG
Dresden_01V39143	NEG	POS	NEG	NEG	NEG	NEG	NEG	NEG	NEG	NEG	POS	POS	POS	POS	NEG	NEG
Greece 1_3680_Harmony	NEG	POS	NEG	NEG	NEG	NEG	NEG	NEG	NEG	NEG	POS	POS	POS	POS	NEG	NEG
**>"European Clade": UK-[illegible]**																
ANS46 (Australia)	NEG	POS	NEG	NEG	NEG	NEG	NEG	NEG	NEG	NEG	POS	NEG	POS	NEG	NEG	NEG
LHH1 (USA)	NEG	POS	NEG	NEG	NEG	NEG	NEG	NEG	NEG	NEG	POS	NEG	POS	NEG	NEG	NEG
Dublin-DSH_AR01_0-1085	NEG	POS	NEG	NEG	NEG	NEG	NEG	NEG	NEG	NEG	POS	POS	POS	POS	NEG	NEG
Dublin-DSH_AR01_0-1118	NEG	POS	NEG	NEG	NEG	NEG	NEG	NEG	NEG	NEG	POS	POS	POS	POS	NEG	NEG
Dublin-DSH_AR15_0098	NEG	POS	NEG	NEG	NEG	NEG	NEG	NEG	NEG	NEG	POS	POS	POS	POS	NEG	NEG
Dublin-DSH_AR15_0104	NEG	POS	NEG	NEG	NEG	NEG	NEG	NEG	NEG	NEG	POS	POS	POS	POS	NEG	NEG
UK_NCTC11939_UK-1_Harmony	NEG	POS	NEG	NEG	NEG	NEG	NEG	NEG	NEG	NEG	POS	POS	POS	AMB	NEG	NEG
**>"European Clade": BK2**																
BK2421 (USA)	NEG	POS	NEG	NEG	NEG	NEG	NEG	NEG	NEG	NEG	POS	NEG	POS	NEG	NEG	NEG
**>"Aussi/NZ Clade", JKD[illegible]**																
JKD6009 (Australia)	NEG	POS	NEG	NEG	NEG	NEG	NEG	NEG	NEG	NEG	POS	NEG	POS	NEG	NEG	NEG
JKD6008 (New Zealand)	NEG	POS	NEG	NEG	NEG	NEG	NEG	NEG	NEG	NEG	POS	NEG	POS	NEG	NEG	NEG
Perth_2015-S20209	NEG	NEG	NEG	NEG	NEG	NEG	NEG	NEG	NEG	NEG	POS	NEG	POS	NEG	NEG	NEG
Perth_2015-S20305	NEG	NEG	NEG	NEG	NEG	NEG	NEG	NEG	NEG	NEG	POS	NEG	POS	NEG	NEG	NEG
Perth_2015-S20603	NEG	POS	NEG	NEG	NEG	NEG	NEG	NEG	NEG	NEG	POS	NEG	POS	NEG	NEG	NEG
Perth_2015-S20607	NEG	POS	NEG	NEG	NEG	NEG	NEG	NEG	NEG	NEG	POS	NEG	POS	NEG	NEG	NEG
Perth_2015-S20608	NEG	POS	NEG	NEG	NEG	NEG	NEG	NEG	NEG	NEG	POS	NEG	POS	NEG	NEG	NEG
Perth_2015-S20610	NEG	POS	NEG	NEG	NEG	NEG	NEG	NEG	NEG	NEG	POS	NEG	POS	NEG	NEG	NEG
Perth_2015-S20613	NEG	POS	NEG	NEG	NEG	NEG	NEG	NEG	NEG	NEG	POS	NEG	POS	NEG	NEG	NEG
Perth_2015-S20617	NEG	NEG	NEG	NEG	NEG	NEG	NEG	NEG	NEG	NEG	POS	NEG	POS	NEG	NEG	NEG
Perth_2015-S20622	NEG	POS	NEG	NEG	NEG	NEG	NEG	NEG	NEG	NEG	POS	NEG	AMB	NEG	NEG	NEG
Perth_2015-S23619	NEG	POS	NEG	NEG	NEG	NEG	NEG	NEG	NEG	NEG	POS	NEG	POS	NEG	NEG	NEG
Perth_2015-S41210	NEG	POS	NEG	NEG	NEG	NEG	NEG	NEG	NEG	NEG	POS	NEG	POS	NEG	NEG	NEG
Perth_2015-S51001	NEG	POS	NEG	NEG	NEG	NEG	NEG	NEG	NEG	NEG	POS	NEG	POS	NEG	NEG	NEG
Perth_2015-S51003	NEG	POS	NEG	NEG	NEG	NEG	NEG	NEG	NEG	NEG	POS	NEG	POS	NEG	NEG	NEG
Perth_2015-S51006	NEG	POS	NEG	NEG	NEG	NEG	NEG	NEG	NEG	NEG	POS	NEG	POS	NEG	NEG	NEG
Perth_2015-S51007	NEG	POS	NEG	NEG	NEG	NEG	NEG	NEG	NEG	NEG	POS	NEG	POS	NEG	NEG	NEG
Perth_2015-S51008	NEG	POS	NEG	NEG	NEG	NEG	NEG	NEG	NEG	NEG	POS	NEG	POS	NEG	NEG	NEG
Perth_2015-S51014	NEG	POS	NEG	NEG	NEG	NEG	NEG	NEG	NEG	NEG	POS	NEG	POS	NEG	NEG	NEG
Perth_2015-S51408	NEG	POS	NEG	NEG	NEG	NEG	NEG	NEG	NEG	NEG	POS	NEG	POS	NEG	NEG	NEG
Perth_2016-S20614	NEG	POS	NEG	NEG	NEG	NEG	NEG	NEG	NEG	NEG	POS	NEG	POS	NEG	NEG	NEG
Perth_2016-S23614	NEG	POS	NEG	NEG	NEG	NEG	NEG	NEG	NEG	NEG	POS	NEG	POS	NEG	NEG	NEG
Perth_2016-S42009	NEG	POS	NEG	NEG	NEG	NEG	NEG	NEG	NEG	NEG	POS	NEG	POS	NEG	NEG	NEG
Perth_2016-S51001	NEG	POS	NEG	NEG	NEG	NEG	NEG	NEG	NEG	NEG	POS	NEG	POS	NEG	NEG	NEG
Perth_2016-S51403	NEG	POS	NEG	NEG	NEG	NEG	NEG	NEG	NEG	NEG	POS	NEG	POS	NEG	NEG	NEG
Perth_07 RPA 20	NEG	POS	NEG	NEG	NEG	NEG	NEG	NEG	NEG	NEG	POS	POS	POS	POS	NEG	NEG
Perth_08-19088	NEG	POS	NEG	NEG	NEG	NEG	NEG	NEG	NEG	NEG	POS	POS	POS	POS	NEG	NEG
Perth_01-15351	NEG	POS	NEG	NEG	NEG	NEG	NEG	NEG	NEG	NEG	POS	POS	POS	POS	NEG	NEG
Perth_01-15357	NEG	POS	NEG	NEG	NEG	NEG	NEG	NEG	NEG	NEG	POS	POS	POS	POS	NEG	NEG
Perth_01-15419	NEG	POS	NEG	NEG	NEG	NEG	NEG	NEG	NEG	NEG	POS	POS	POS	POS	NEG	NEG
Perth_01-16309	NEG	POS	NEG	NEG	NEG	NEG	NEG	NEG	NEG	NEG	POS	POS	POS	POS	NEG	NEG
Perth_03 SNP 95	NEG	POS	NEG	NEG	NEG	NEG	NEG	NEG	NEG	NEG	POS	POS	POS	POS	NEG	NEG
Perth_2003_AH_47	NEG	POS	NEG	NEG	NEG	NEG	NEG	NEG	NEG	NEG	POS	POS	POS	AMB	NEG	NEG
**>"Australian/NZ Clade":**																
Perth_2015-S20623	NEG	POS	NEG	NEG	NEG	NEG	NEG	NEG	NEG	NEG	POS	NEG	POS	NEG	NEG	NEG
Perth_2015-S20826	NEG	POS	NEG	NEG	NEG	NEG	NEG	NEG	NEG	NEG	POS	NEG	POS	NEG	NEG	NEG
Perth_05 NH 56	NEG	POS	NEG	NEG	NEG	NEG	NEG	NEG	NEG	NEG	POS	POS	POS	POS	NEG	NEG
**>CC5-MRSA-III LA MRSA**																
Delbrück 38 isolate from domestic turkey	NEG	POS	NEG	NEG	NEG	NEG	NEG	NEG	NEG	NEG	POS	POS	POS	POS	NEG	NEG
**> Staph. pseudintermed**																
KM1381	NEG	NEG	NEG	NEG	NEG	NEG	NEG	NEG	NEG	NEG	NEG	NEG	NEG	NEG	NEG	NEG
**>"South-East Asian Clad[illegible]**																
M92 (Canada)	NEG	POS	NEG	NEG	NEG	NEG	NEG	NEG	NEG	NEG	POS	NEG	POS	NEG	NEG	NEG
CHI59 (China)	NEG	POS	NEG	NEG	NEG	NEG	NEG	NEG	NEG	NEG	POS	NEG	POS	NEG	NEG	NEG
CHI61 (China)	NEG	POS	NEG	NEG	NEG	NEG	NEG	NEG	NEG	NEG	POS	NEG	POS	NEG	NEG	NEG
CUHK_HK1997 (China)	NEG	POS	NEG	NEG	NEG	NEG	NEG	NEG	NEG	NEG	POS	NEG	POS	NEG	NEG	NEG
M996 (China)	NEG	POS	NEG	NEG	NEG	NEG	NEG	NEG	NEG	NEG	POS	NEG	POS	NEG	NEG	NEG
H211 (Denmark)	NEG	POS	NEG	NEG	NEG	NEG	NEG	NEG	NEG	NEG	POS	NEG	POS	NEG	NEG	NEG
H216 (Denmark)	NEG	POS	NEG	NEG	NEG	NEG	NEG	NEG	NEG	NEG	POS	NEG	POS	NEG	NEG	NEG
D71 (Germany)	NEG	POS	NEG	NEG	NEG	NEG	NEG	NEG	NEG	NEG	POS	NEG	POS	NEG	NEG	NEG
NMR05 (India)	NEG	POS	NEG	NEG	NEG	NEG	NEG	NEG	NEG	NEG	POS	NEG	POS	NEG	NEG	NEG
MAL11 (Malaysia)	NEG	POS	NEG	NEG	NEG	NEG	NEG	NEG	NEG	NEG	POS	NEG	POS	NEG	NEG	NEG
MAL9 (Malaysia)	NEG	POS	NEG	NEG	NEG	NEG	NEG	NEG	NEG	NEG	POS	NEG	POS	NEG	NEG	NEG
Na21 (Sri Lanka)	NEG	POS	NEG	NEG	NEG	NEG	NEG	NEG	NEG	NEG	POS	NEG	POS	NEG	NEG	NEG
M592 (Syria)	NEG	POS	NEG	NEG	NEG	NEG	NEG	NEG	NEG	NEG	POS	NEG	POS	NEG	NEG	NEG
H202 (Thailand)	NEG	POS	NEG	NEG	NEG	NEG	NEG	NEG	NEG	NEG	POS	NEG	POS	NEG	NEG	NEG
S102 (Thailand)	NEG	POS	NEG	NEG	NEG	NEG	NEG	NEG	NEG	NEG	POS	NEG	POS	NEG	NEG	NEG
S130 (Thailand)	NEG	POS	NEG	NEG	NEG	NEG	NEG	NEG	NEG	NEG	POS	NEG	POS	NEG	NEG	NEG
S40 (Thailand)	NEG	POS	NEG	NEG	NEG	NEG	NEG	NEG	NEG	NEG	POS	NEG	POS	NEG	NEG	NEG
S71 (Thailand)	NEG	POS	NEG	NEG	NEG	NEG	NEG	NEG	NEG	NEG	POS	NEG	POS	NEG	NEG	NEG
S87 (Thailand)	NEG	POS	NEG	NEG	NEG	NEG	NEG	NEG	NEG	NEG	POS	NEG	POS	NEG	NEG	NEG
S93 (Thailand)	NEG	POS	NEG	NEG	NEG	NEG	NEG	NEG	NEG	NEG	POS	NEG	POS	NEG	NEG	NEG
US_002 (Thailand)	NEG	POS	NEG	NEG	NEG	NEG	NEG	NEG	NEG	NEG	POS	NEG	POS	NEG	NEG	NEG
US_017 (Thailand)	NEG	POS	NEG	NEG	NEG	NEG	NEG	NEG	NEG	NEG	POS	NEG	POS	NEG	NEG	NEG
WAMC6102 (Thailand)	NEG	POS	NEG	NEG	NEG	NEG	NEG	NEG	NEG	NEG	POS	NEG	POS	NEG	NEG	NEG
NCTC_13626_TW20 (UK)	NEG	POS	NEG	NEG	NEG	NEG	NEG	NEG	NEG	NEG	POS	NEG	POS	NEG	NEG	NEG
BSAC3021 (UK)	NEG	POS	NEG	NEG	NEG	NEG	NEG	NEG	NEG	NEG	POS	NEG	POS	NEG	NEG	NEG
BSAC3046 (UK)	NEG	POS	NEG	NEG	NEG	NEG	NEG	NEG	NEG	NEG	POS	NEG	POS	NEG	NEG	NEG
1193_SAUR (USA)	NEG	POS	NEG	NEG	NEG	NEG	NEG	NEG	NEG	NEG	POS	NEG	POS	NEG	NEG	NEG
1194_SAUR (USA)	NEG	POS	NEG	NEG	NEG	NEG	NEG	NEG	NEG	NEG	POS	NEG	POS	NEG	NEG	NEG

STRAIN / ISOLATE	MISCELLANEOUS GENES															
	sau				sau96I	G7ZRU6	ycjY	sagD	G7ZTC1		sdrM					
	sau3AI	sauUSI	sauRF122	sauSO385	sau96I	G7ZRU6	ycjY = C5Q1F1 ("Argenteus/ST1850-like", CC12, CC361, CC398)	sagD	G7ZTC1	G7ZTC1-argenteus	sdrM / tetEfflux	sdrM (cons)	hp_sdrM-801	hp_sdrM-802	sdrM (CC30)	sdrM (argen-teus)
	type II restriction-modification system endonuclease					acetyltransferase, GNAT family, "Argenteus/ST1850-like"		Putative bacteriocin biosynthesis associated protein	TetR family regulator		Multidrug resistance transporter					
>"South-East Asian Clade": TW20 (Irish AR44), c																
1195_SAUR (USA)	NEG	POS	NEG	NEG	NEG	NEG	NEG	NEG	NEG	NEG	POS	NEG	POS	NEG	NEG	NEG
FVRH6002 (USA)	NEG	POS	NEG	NEG	NEG	NEG	NEG	NEG	NEG	NEG	POS	NEG	POS	NEG	NEG	NEG
KINW6048 (USA)	NEG	POS	NEG	NEG	NEG	NEG	NEG	NEG	NEG	NEG	POS	NEG	POS	NEG	NEG	NEG
LAMC0011 (USA)	NEG	POS	NEG	NEG	NEG	NEG	NEG	NEG	NEG	NEG	POS	NEG	POS	NEG	NEG	NEG
SJOS6053 (USA)	NEG	POS	NEG	NEG	NEG	NEG	NEG	NEG	NEG	NEG	POS	NEG	POS	NEG	NEG	NEG
SJOS6072 (USA)	NEG	POS	NEG	NEG	NEG	NEG	NEG	NEG	NEG	NEG	POS	NEG	POS	NEG	NEG	NEG
UCIM6042 (USA)	NEG	POS	NEG	NEG	NEG	NEG	NEG	NEG	NEG	NEG	POS	NEG	POS	NEG	NEG	NEG
Perth_2015-S31902	NEG	NEG	NEG	NEG	NEG	NEG	NEG	NEG	NEG	NEG	POS	NEG	POS	NEG	NEG	NEG
Perth_2015-S31904	NEG	POS	NEG	NEG	NEG	NEG	NEG	NEG	NEG	NEG	POS	NEG	POS	NEG	NEG	NEG
Perth_2015-S31913	NEG	POS	NEG	NEG	NEG	NEG	NEG	NEG	NEG	NEG	POS	NEG	POS	NEG	NEG	NEG
Perth_2015-S31918	NEG	NEG	NEG	NEG	NEG	NEG	NEG	NEG	NEG	NEG	POS	NEG	POS	NEG	NEG	NEG
Dublin-DSH_AR44_E1183	NEG	POS	NEG	NEG	NEG	NEG	NEG	NEG	NEG	NEG	POS	POS	POS	POS	NEG	NEG
Dublin-DSH_Unfamiliar-3_E1520	NEG	POS	NEG	NEG	NEG	NEG	NEG	NEG	NEG	NEG	POS	POS	POS	POS	NEG	NEG
Hong Kong_042	NEG	POS	NEG	NEG	NEG	NEG	NEG	NEG	NEG	NEG	POS	POS	POS	POS	NEG	NEG
Hong Kong_101	NEG	POS	NEG	NEG	NEG	NEG	NEG	NEG	NEG	NEG	POS	POS	POS	POS	NEG	NEG
Hong Kong_111	NEG	POS	NEG	NEG	NEG	NEG	NEG	NEG	NEG	NEG	POS	POS	POS	POS	NEG	NEG
Hong Kong_113	NEG	POS	NEG	NEG	NEG	NEG	NEG	NEG	NEG	NEG	POS	POS	POS	POS	NEG	NEG
Hong Kong_115	NEG	POS	NEG	NEG	NEG	NEG	NEG	NEG	NEG	NEG	POS	POS	POS	POS	NEG	NEG
Hong Kong_118	NEG	POS	NEG	NEG	NEG	NEG	NEG	NEG	NEG	NEG	POS	POS	POS	AMB	NEG	NEG
Hong Kong_125	NEG	POS	NEG	NEG	NEG	NEG	NEG	NEG	NEG	NEG	POS	POS	POS	POS	NEG	NEG
Hong Kong_129	NEG	POS	NEG	NEG	NEG	NEG	NEG	NEG	NEG	NEG	POS	POS	POS	POS	NEG	NEG
Hong Kong_133	NEG	POS	NEG	NEG	NEG	NEG	NEG	NEG	NEG	NEG	POS	POS	POS	POS	NEG	NEG
Kuwait_009	NEG	POS	NEG	NEG	NEG	NEG	NEG	NEG	NEG	NEG	POS	POS	POS	POS	NEG	NEG
Kuwait_103	NEG	POS	NEG	NEG	NEG	NEG	NEG	NEG	NEG	NEG	POS	POS	POS	POS	NEG	NEG
Kuwait_107	NEG	POS	NEG	NEG	NEG	NEG	NEG	NEG	NEG	NEG	POS	POS	POS	POS	NEG	NEG
Perth_03 AH 88	NEG	POS	NEG	NEG	NEG	NEG	NEG	NEG	NEG	NEG	POS	POS	POS	POS	NEG	NEG
Perth_08-16905	NEG	POS	NEG	NEG	NEG	NEG	NEG	NEG	NEG	NEG	POS	POS	POS	POS	NEG	NEG
Riyadh_Alfaisal-10_SICU_13_B5672_1117353	NEG	POS	NEG	NEG	NEG	NEG	NEG	NEG	NEG	NEG	POS	POS	POS	POS	NEG	NEG
Riyadh_SSTI_17_3508602	NEG	POS	NEG	NEG	NEG	NEG	NEG	NEG	NEG	NEG	POS	POS	POS	NEG	NEG	NEG
Riyadh-2822088-R	NEG	POS	NEG	NEG	NEG	NEG	NEG	NEG	NEG	NEG	POS	POS	POS	POS	NEG	NEG
Trinidad&Tobago_2012_195 (619/12)	NEG	POS	NEG	NEG	NEG	NEG	NEG	NEG	NEG	NEG	POS	POS	POS	AMB	NEG	NEG
Trinidad&Tobago_2012_370 (1112-52177)	NEG	POS	NEG	NEG	NEG	NEG	NEG	NEG	NEG	NEG	POS	POS	POS	NEG	NEG	NEG
Trinidad&Tobago_2012_378 (4139)	NEG	POS	NEG	NEG	NEG	NEG	NEG	NEG	NEG	NEG	POS	POS	POS	POS	NEG	NEG
Trinidad&Tobago_2013_PA21	NEG	POS	NEG	NEG	NEG	NEG	NEG	NEG	NEG	NEG	POS	POS	POS	POS	NEG	AMB
Trinidad&Tobago_2013_PA22	NEG	POS	NEG	NEG	NEG	NEG	NEG	NEG	NEG	NEG	POS	POS	POS	POS	NEG	NEG
Trinidad&Tobago_2912_340 (8523/12)	NEG	POS	NEG	NEG	NEG	NEG	NEG	NEG	NEG	NEG	POS	NEG	POS	NEG	NEG	NEG
Trinidad&Tobago_MRSA2010_105	NEG	POS	NEG	NEG	NEG	NEG	AMB	NEG	NEG	NEG	POS	POS	POS	POS	NEG	NEG
Trinidad&Tobago_MRSA2010_108	NEG	POS	NEG	NEG	NEG	NEG	NEG	NEG	NEG	NEG	POS	POS	POS	POS	NEG	NEG
Trinidad&Tobago_MRSA2010_33	NEG	POS	NEG	NEG	NEG	NEG	NEG	NEG	NEG	NEG	POS	POS	POS	POS	NEG	NEG
Trinidad&Tobago_MRSA2010_79	NEG	POS	NEG	NEG	NEG	NEG	NEG	NEG	NEG	NEG	POS	POS	POS	POS	NEG	NEG
Trinidad&Tobago_MRSA2010_88	NEG	POS	NEG	NEG	NEG	NEG	NEG	NEG	NEG	NEG	POS	POS	POS	POS	NEG	NEG
Trinidad&Tobago_MRSA2010_97	NEG	POS	NEG	NEG	NEG	NEG	NEG	NEG	NEG	NEG	POS	POS	POS	POS	NEG	NEG
Trinidad&Tobago_SSTI 2012_031	NEG	POS	NEG	NEG	NEG	NEG	AMB	NEG	NEG	NEG	POS	POS	POS	POS	NEG	AMB
Uganda-29_17148_250811	NEG	POS	NEG	NEG	NEG	NEG	NEG	NEG	NEG	NEG	POS	POS	POS	AMB	NEG	NEG
>"South-East Asian Clad																
UCIM6015	NEG	POS	NEG	NEG	NEG	NEG	NEG	NEG	NEG	NEG	POS	NEG	POS	NEG	NEG	NEG
Hong Kong_121	NEG	POS	NEG	NEG	NEG	NEG	NEG	NEG	NEG	NEG	POS	POS	POS	POS	NEG	NEG
>"South-East Asian Clad																
Z172 (Taiwan)	NEG	POS	NEG	NEG	NEG	NEG	NEG	NEG	NEG	NEG	POS	NEG	POS	NEG	NEG	NEG
Russia-19_0342_Kurgan	NEG	AMB	NEG	NEG	NEG	NEG	NEG	NEG	NEG	NEG	POS	AMB	POS	NEG	NEG	NEG
>"South-East Asian Clad																
Perth_01-15392	NEG	POS	NEG	NEG	NEG	NEG	NEG	NEG		NEG	POS	POS	POS	POS	NEG	NEG
>"South-East Asian Clad																
XN108 (China)	NEG	POS	NEG	NEG	NEG	NEG	NEG	NEG	NEG	NEG	POS	NEG	POS	NEG	NEG	NEG
NMR09 (India)	NEG	POS	NEG	NEG	NEG	NEG	NEG	NEG	NEG	NEG	POS	NEG	POS	NEG	NEG	NEG
346 (Malaysia)	NEG	POS	NEG	NEG	NEG	NEG	NEG	NEG	NEG	NEG	POS	NEG	POS	NEG	NEG	NEG
DS_009 (Thailand)	NEG	POS	NEG	NEG	NEG	NEG	NEG	NEG	NEG	NEG	POS	NEG	POS	NEG	NEG	NEG
18ANR581005 (Ind)	NEG	POS	NEG	NEG	NEG	#ZAHL!	NEG	NEG	NEG	NEG	POS	POS	POS	AMB	NEG	NEG
Bengalaru_ST20141405	NEG	POS	NEG	NEG	NEG	NEG	NEG	NEG	NEG	NEG	POS	POS	POS	POS	NEG	NEG
Bengalaru_ST20141406	NEG	POS	NEG	NEG	NEG	NEG	NEG	NEG	NEG	NEG	POS	POS	POS	POS	NEG	NEG
Bengalaru_ST20141417	NEG	POS	NEG	NEG	NEG	NEG	NEG	NEG	NEG	NEG	POS	POS	POS	POS	NEG	NEG
Hong Kong_107	NEG	POS	NEG	NEG	NEG	NEG	NEG	NEG	NEG	NEG	POS	POS	POS	POS	NEG	NEG
Hong Kong_12	NEG	POS	NEG	NEG	NEG	NEG	NEG	NEG	NEG	NEG	POS	AMB	POS	AMB	NEG	NEG
Hong Kong_132	NEG	POS	NEG	NEG	NEG	NEG	NEG	NEG	NEG	NEG	POS	POS	POS	POS	NEG	NEG
Riyadh-3028763-R	NEG	POS	NEG	NEG	NEG	NEG	NEG	NEG	NEG	NEG	POS	POS	POS	POS	NEG	NEG
Trinidad&Tobago_MRSA2010_94	NEG	POS	NEG	NEG	NEG	NEG	NEG	NEG	NEG	NEG	POS	POS	POS	POS	NEG	NEG
>"South-East Asian Clad																
M705 (Thailand)	NEG	POS	NEG	NEG	NEG	NEG	NEG	NEG	NEG	NEG	POS	NEG	POS	NEG	NEG	NEG
S85 (Thailand)	NEG	POS	NEG	NEG	NEG	NEG	NEG	NEG	NEG	NEG	POS	NEG	POS	NEG	NEG	NEG
Riyadh_Alfaisal-04_281014_S5076	NEG	POS	NEG	NEG	NEG	NEG	NEG	NEG	NEG	NEG	POS	NEG	POS	NEG	NEG	NEG
Riyadh_Alfaisal-26_S15145_1104163	NEG	POS	NEG	NEG	NEG	NEG	NEG	NEG	NEG	NEG	POS	POS	POS	POS	NEG	NEG
Riyadh-2817437-W	NEG	POS	NEG	NEG	NEG	NEG	NEG	NEG	NEG	NEG	POS	POS	POS	POS	NEG	NEG
>"South-East Asian Clad																
PPUKM-775-2009 (Malaysia)	NEG	POS	NEG	NEG	NEG	NEG	NEG	NEG	NEG	NEG	POS	NEG	POS	NEG	NEG	NEG
Hong Kong_138	NEG	POS	NEG	NEG	NEG	NEG	NEG	NEG	NEG	NEG	POS	POS	POS	AMB	NEG	NEG
>"South-East Asian Clad																
Kuwait_001	NEG	POS	NEG	NEG	NEG	NEG	NEG	NEG	NEG	NEG	POS	POS	POS	POS	NEG	NEG
Kuwait_337	NEG	POS	NEG	NEG	NEG	NEG	POS	NEG	POS	NEG	POS	POS	POS	NEG	NEG	NEG
>"South-East Asian Clad																
CUHK_HK2007 (China)	NEG	POS	NEG	NEG	NEG	NEG	NEG	NEG	NEG	NEG	POS	NEG	POS	NEG	NEG	NEG
NMR02 (India)	NEG	POS	NEG	NEG	NEG	NEG	NEG	NEG	NEG	NEG	POS	NEG	POS	NEG	NEG	NEG
VB1490 (India)	NEG	POS	NEG	NEG	NEG	NEG	NEG	NEG	NEG	NEG	POS	NEG	POS	NEG	NEG	NEG
V521 (Korea)	NEG	POS	NEG	NEG	NEG	NEG	NEG	NEG	NEG	NEG	POS	NEG	POS	NEG	NEG	NEG
HST-077 (Lebanon)	NEG	POS	NEG	NEG	NEG	NEG	NEG	NEG	NEG	NEG	POS	NEG	POS	NEG	NEG	NEG
MAL1 (Malaysia)	NEG	POS	NEG	NEG	NEG	NEG	NEG	NEG	NEG	NEG	POS	NEG	POS	NEG	NEG	NEG
MAL3 (Malaysia)	NEG	POS	NEG	NEG	NEG	NEG	NEG	NEG	NEG	NEG	POS	NEG	POS	NEG	NEG	NEG
PPUKM-261-2009 (Malaysia)	NEG	POS	NEG	NEG	NEG	NEG	NEG	NEG	NEG	NEG	POS	NEG	POS	NEG	NEG	NEG
PPUKM-332-2009 (Malaysia)	NEG	POS	NEG	NEG	NEG	NEG	NEG	NEG	NEG	NEG	POS	NEG	POS	NEG	NEG	NEG
S38 (Thailand)	NEG	POS	NEG	NEG	NEG	NEG	NEG	NEG	NEG	NEG	POS	NEG	POS	NEG	NEG	NEG
S7 (Thailand)	NEG	POS	NEG	NEG	NEG	NEG	NEG	NEG	NEG	NEG	POS	NEG	POS	NEG	NEG	NEG
US_008 (Thailand)	NEG	POS	NEG	NEG	NEG	NEG	NEG	NEG	NEG	NEG	POS	NEG	POS	NEG	NEG	NEG
US_030 (Thailand)	NEG	POS	NEG	NEG	NEG	NEG	NEG	NEG	NEG	NEG	POS	NEG	POS	NEG	NEG	NEG
M170 (Tsunami-related)	NEG	POS	NEG	NEG	NEG	NEG	NEG	NEG	NEG	NEG	POS	NEG	POS	NEG	NEG	NEG
BSAC697 (UK)	NEG	POS	NEG	NEG	NEG	NEG	NEG	NEG	NEG	NEG	POS	NEG	POS	NEG	NEG	NEG
IS-189	NEG	POS	NEG	NEG	NEG	NEG	NEG	NEG	NEG	NEG	POS	NEG	POS	NEG	NEG	NEG
MRGR3	NEG	POS	NEG	NEG	NEG	NEG	NEG	NEG	NEG	NEG	POS	NEG	POS	NEG	NEG	NEG
smg07	NEG	POS	NEG	NEG	NEG	NEG	NEG	NEG	NEG	NEG	POS	NEG	POS	NEG	NEG	NEG
AH1-AUS-EMRSA-3	NEG	POS	NEG	NEG	NEG	NEG	NEG	NEG	NEG	NEG	POS	POS	POS	POS	NEG	NEG
Bengalaru_ST20141403	NEG	POS	NEG	NEG	NEG	NEG	NEG	NEG	NEG	NEG	POS	POS	POS	POS	NEG	NEG
Finland E24_98541_Harmony	NEG	POS	NEG	NEG	NEG	NEG	NEG	NEG	NEG	NEG	POS	POS	POS	POS	NEG	NEG
Hong Kong_10	NEG	POS	NEG	NEG	NEG	NEG	NEG	NEG	NEG	NEG	POS	POS	POS	POS	NEG	NEG
Hong Kong_100	NEG	POS	NEG	NEG	NEG	NEG	NEG	NEG	NEG	NEG	POS	POS	POS	POS	NEG	NEG
Hong Kong_199	NEG	POS	NEG	NEG	NEG	NEG	NEG	NEG	NEG	NEG	POS	POS	POS	POS	NEG	NEG
Hong Kong_34	NEG	POS	NEG	NEG	NEG	NEG	NEG	NEG	NEG	NEG	POS	POS	POS	POS	NEG	NEG
Hong Kong_88	NEG	POS	NEG	NEG	NEG	NEG	NEG	NEG	NEG	NEG	POS	POS	POS	POS	NEG	NEG
Hong Kong_92	NEG	POS	NEG	NEG	NEG	NEG	NEG	NEG	NEG	NEG	POS	POS	POS	POS	NEG	NEG
Hong Kong_94	NEG	POS	NEG	NEG	NEG	NEG	NEG	NEG	NEG	NEG	POS	POS	POS	POS	NEG	NEG
Kuwait_257	NEG	POS	NEG	NEG	NEG	NEG	NEG	NEG	NEG	NEG	POS	POS	POS	POS	NEG	NEG
Perth_01-15330	NEG	POS	NEG	NEG	NEG	NEG	NEG	NEG	NEG	NEG	POS	POS	POS	POS	NEG	NEG
Perth_01-15349	NEG	POS	NEG	NEG	NEG	NEG	NEG	NEG	NEG	NEG	POS	POS	POS	POS	NEG	NEG
Perth_01-16176	NEG	POS	NEG	NEG	NEG	NEG	NEG	NEG	NEG	NEG	POS	POS	POS	POS	NEG	NEG
Perth_WBG8379	NEG	POS	NEG	NEG	NEG	NEG	NEG	NEG	NEG	NEG	POS	POS	POS	POS	NEG	NEG
Rawalpindi_Kidney Center_56	NEG	POS	NEG	NEG	NEG	NEG	NEG	NEG	NEG	NEG	POS	POS	POS	POS	NEG	NEG
Riyadh_SSTI_05_3464614	NEG	POS	NEG	NEG	NEG	NEG	NEG	NEG	NEG	NEG	POS	POS	POS	AMB	NEG	NEG
Trinidad&Tobago_2012_157	NEG	POS	NEG	NEG	NEG	NEG	AMB	NEG	NEG	NEG	POS	POS	POS	POS	NEG	NEG

STRAIN / ISOLATE	MISCELLANEOUS GENES															
	sau				sau96I	G7ZRU6	ycjY	sagD	G7ZTC1		sdrM					
	sau3AI	sauUSI	sauRF122	sauSO385	sau96I	G7ZRU6	ycjY = C5Q1F1 ("Argenteus/ST1850-like", CC12, CC361, CC398)	sagD	G7ZTC1	G7ZTC1-argenteus	sdrM / tetEfflux	sdrM (cons)	hp_sdrM-801	hp_sdrM-802	sdrM (CC30)	sdrM (argen-teus)
	type II restriction-modification system endonuclease					acetyltransferase, GNAT family, "Argenteus/ST1850-like"		Putative bacteriocin biosynthesis associated protein	TetR family regulator		Multidrug resistance transporter					
>"South-East Asian Clad																
M418	NEG	POS	NEG	NEG	NEG	NEG	NEG	NEG	NEG	NEG	POS	NEG	POS	NEG	NEG	NEG
>"South-East Asian Clad																
NMR07 (India)	NEG	POS	NEG	NEG	NEG	NEG	NEG	NEG	NEG	NEG	POS	NEG	POS	NEG	NEG	NEG
NMR08 (India)	NEG	POS	NEG	NEG	NEG	NEG	NEG	NEG	NEG	NEG	POS	NEG	POS	NEG	NEG	NEG
Bengalaru_ST20121978	NEG	POS	NEG	NEG	NEG	NEG	NEG	NEG	NEG	NEG	POS	POS	POS	POS	NEG	NEG
Hong Kong_89	NEG	POS	NEG	NEG	NEG	NEG	NEG	NEG	NEG	NEG	POS	POS	POS	POS	NEG	NEG
>"South-East Asian Clad																
Perth_2005_AH_5	NEG	POS	NEG	NEG	NEG	NEG	NEG	NEG	NEG	NEG	POS	POS	POS	POS	NEG	NEG
>"South-East Asian Clad																
Trinidad&Tobago_MRSA2010_103	NEG	POS	NEG	NEG	NEG	NEG	NEG	NEG	NEG	NEG	POS	POS	POS	POS	NEG	NEG
Trinidad&Tobago_2013_PA25	NEG	POS	NEG	NEG	NEG	NEG	NEG	NEG	NEG	NEG	POS	POS	POS	POS	NEG	NEG
Trinidad&Tobago_MRSA2010_110	NEG	POS	NEG	NEG	NEG	NEG	NEG	NEG	NEG	NEG	POS	POS	POS	POS	NEG	NEG
Trinidad&Tobago_SSTI 2012_111	NEG	POS	NEG	NEG	NEG	NEG	NEG	NEG	NEG	NEG	POS	POS	POS	POS	NEG	NEG
Trinidad&Tobago_MRSA2010_158	NEG	POS	NEG	NEG	NEG	NEG	AMB	NEG	NEG	NEG	POS	POS	POS	POS	NEG	NEG
>"South-East Asian Clad																
Kuwait_122	NEG	POS	NEG	NEG	NEG	NEG	NEG	NEG	NEG	NEG	POS	POS	POS	AMB	NEG	NEG
Kuwait_002	NEG	POS	NEG	NEG	NEG	NEG	NEG	NEG	NEG	NEG	POS	POS	POS	POS	NEG	NEG
Kuwait_196	NEG	POS	NEG	NEG	NEG	NEG	NEG	NEG	NEG	NEG	POS	POS	POS	POS	NEG	NEG
Kuwait_199	NEG	POS	NEG	NEG	NEG	NEG	NEG	NEG	NEG	NEG	POS	POS	POS	POS	NEG	NEG
Hong Kong_136	NEG	POS	NEG	NEG	NEG	NEG	NEG	NEG	NEG	NEG	POS	POS	POS	POS	NEG	NEG
>"South-East Asian Clad																
P32 (Poland)	NEG	POS	NEG	NEG	NEG	NEG	NEG	NEG	NEG	NEG	POS	NEG	POS	NEG	NEG	NEG
>"South-East Asian Clad																
Perth_2015-S32313	NEG	POS	NEG	NEG	NEG	NEG	NEG	NEG	NEG	NEG	POS	NEG	POS	NEG	NEG	NEG
>"hla-negative South-Ea																
DEN907 (Denmark)	NEG	POS	NEG	NEG	NEG	NEG	NEG	NEG	NEG	NEG	POS	NEG	POS	NEG	NEG	NEG
>"hla-negative South-Ea																
S26 (Thailand)	NEG	POS	NEG	NEG	NEG	NEG	NEG	NEG	NEG	NEG	POS	NEG	POS	NEG	NEG	NEG
S42 (Thailand)	NEG	POS	NEG	NEG	NEG	NEG	NEG	NEG	NEG	NEG	POS	NEG	POS	NEG	NEG	NEG
S97 (Thailand)	NEG	POS	NEG	NEG	NEG	NEG	NEG	NEG	NEG	NEG	POS	NEG	POS	NEG	NEG	NEG
S78 (Thailand)	NEG	POS	NEG	NEG	NEG	NEG	NEG	NEG	NEG	NEG	POS	NEG	POS	NEG	NEG	NEG
S25 (Thailand)	NEG	POS	NEG	NEG	NEG	NEG	NEG	NEG	NEG	NEG	POS	NEG	POS	NEG	NEG	NEG
S21 (Thailand)	NEG	POS	NEG	NEG	NEG	NEG	NEG	NEG	NEG	NEG	POS	NEG	POS	NEG	NEG	NEG
S106 (Thailand)	NEG	POS	NEG	NEG	NEG	NEG	NEG	NEG	NEG	NEG	POS	NEG	POS	NEG	NEG	NEG
S24 (Thailand)	NEG	POS	NEG	NEG	NEG	NEG	NEG	NEG	NEG	NEG	POS	NEG	POS	NEG	NEG	NEG
S39 (Thailand)	NEG	POS	NEG	NEG	NEG	NEG	NEG	NEG	NEG	NEG	POS	NEG	POS	NEG	NEG	NEG
S2 (Thailand)	NEG	POS	NEG	NEG	NEG	NEG	NEG	NEG	NEG	NEG	POS	NEG	POS	NEG	NEG	NEG
S81 (Thailand)	NEG	POS	NEG	NEG	NEG	NEG	NEG	NEG	NEG	NEG	POS	NEG	POS	NEG	NEG	NEG
M116 (Vietnam)	NEG	POS	NEG	NEG	NEG	NEG	NEG	NEG	NEG	NEG	POS	NEG	POS	NEG	NEG	NEG
>"Portuguese Clade": Pc																
FFP103 (Portugal)	NEG	POS	NEG	NEG	NEG	NEG	NEG	NEG	NEG	NEG	POS	NEG	POS	NEG	NEG	NEG
HDG2 (Portugal)	NEG	POS	NEG	NEG	NEG	NEG	NEG	NEG	NEG	NEG	POS	NEG	POS	NEG	NEG	NEG
HSA10 (Portugal)	NEG	POS	NEG	NEG	NEG	NEG	NEG	NEG	NEG	NEG	POS	NEG	POS	NEG	NEG	NEG
HSA11 (Portugal)	NEG	POS	NEG	NEG	NEG	NEG	NEG	NEG	NEG	NEG	POS	NEG	POS	NEG	NEG	NEG
ICP5011 (Portugal)	NEG	POS	NEG	NEG	NEG	NEG	NEG	NEG	NEG	NEG	POS	NEG	POS	NEG	NEG	NEG
ICP5014 (Portugal)	NEG	POS	NEG	NEG	NEG	NEG	NEG	NEG	NEG	NEG	POS	NEG	POS	NEG	NEG	NEG
ICP5062 (Portugal)	NEG	POS	NEG	NEG	NEG	NEG	NEG	NEG	NEG	NEG	POS	NEG	POS	NEG	NEG	NEG
Strain 1063_ATCC 33592 FDAARGOS_3 (USA)	NEG	POS	NEG	NEG	NEG	NEG	NEG	NEG	NEG	NEG	POS	NEG	POS	NEG	NEG	NEG
	NEG	POS	NEG	NEG	NEG	NEG	NEG	NEG	NEG	NEG	POS	POS	POS	POS	NEG	NEG
Russia-07_0110_Kurgan	NEG	NEG	NEG	NEG	NEG	NEG	NEG	NEG	NEG	NEG	POS	NEG	POS	NEG	NEG	NEG
Russia-11_0164_Kurgan	NEG	POS	NEG	NEG	NEG	NEG	NEG	NEG	NEG	NEG	POS	POS	POS	POS	NEG	NEG
Russia-20_0367_Kurgan	NEG	POS	NEG	NEG	NEG	NEG	NEG	NEG	NEG	NEG	POS	POS	POS	AMB	NEG	NEG
Russia-21_0390_Chelyabinsk	NEG	POS	NEG	NEG	NEG	NEG	NEG	NEG	NEG	NEG	POS	POS	POS	AMB	NEG	NEG
>"South American/ Mid																
AGT1 (Argentina)	NEG	POS	NEG	NEG	NEG	NEG	NEG	NEG	NEG	NEG	POS	NEG	POS	NEG	NEG	NEG
AGT120 (Argentina)	NEG	POS	NEG	NEG	NEG	NEG	NEG	NEG	NEG	NEG	POS	NEG	POS	NEG	NEG	NEG
AGT67 (Argentina)	NEG	POS	NEG	NEG	NEG	NEG	NEG	NEG	NEG	NEG	POS	NEG	POS	NEG	NEG	NEG
AGT9 (Argentina)	NEG	POS	NEG	NEG	NEG	NEG	NEG	NEG	NEG	NEG	POS	NEG	POS	NEG	NEG	NEG
RA3 (Argentina)	NEG	POS	NEG	NEG	NEG	NEG	NEG	NEG	NEG	NEG	POS	NEG	POS	NEG	NEG	NEG
RA6 (Argentina)	NEG	POS	NEG	NEG	NEG	NEG	NEG	NEG	NEG	NEG	POS	NEG	POS	NEG	NEG	NEG
RA7 (Argentina)	NEG	POS	NEG	NEG	NEG	NEG	NEG	NEG	NEG	NEG	POS	NEG	POS	NEG	NEG	NEG
Be62 (Brazil)	NEG	POS	NEG	NEG	NEG	NEG	NEG	NEG	NEG	NEG	POS	NEG	POS	NEG	NEG	NEG
Bmb9393 (Brazil)	NEG	POS	NEG	NEG	NEG	NEG	NEG	NEG	NEG	NEG	POS	NEG	POS	NEG	NEG	NEG
BRA36 (Brazil)	NEG	POS	NEG	NEG	NEG	NEG	NEG	NEG	NEG	NEG	POS	NEG	POS	NEG	NEG	NEG
BZ48 (Brazil)	NEG	POS	NEG	NEG	NEG	NEG	NEG	NEG	NEG	NEG	POS	NEG	POS	NEG	NEG	NEG
Gv51 (Brazil)	NEG	POS	NEG	NEG	NEG	NEG	NEG	NEG	NEG	NEG	POS	NEG	POS	NEG	NEG	NEG
Gv69 (Brazil)	NEG	POS	NEG	NEG	NEG	NEG	NEG	NEG	NEG	NEG	POS	NEG	POS	NEG	NEG	NEG
Gv88 (Brazil)	NEG	POS	NEG	NEG	NEG	NEG	NEG	NEG	NEG	NEG	POS	NEG	POS	NEG	NEG	NEG
HC1335 (Brazil)	NEG	POS	NEG	NEG	NEG	NEG	NEG	NEG	NEG	NEG	POS	NEG	POS	NEG	NEG	NEG
HC1340 (Brazil)	NEG	POS	NEG	NEG	NEG	NEG	NEG	NEG	NEG	NEG	POS	NEG	POS	NEG	NEG	NEG
HC556 (Brazil)	NEG	POS	NEG	NEG	NEG	NEG	NEG	NEG	NEG	NEG	POS	NEG	POS	NEG	NEG	NEG
HU25 (Brazil)	NEG	POS	NEG	NEG	NEG	NEG	NEG	NEG	NEG	NEG	POS	NEG	POS	NEG	NEG	NEG
UB563 (Brazil)	NEG	POS	NEG	NEG	NEG	NEG	NEG	NEG	NEG	NEG	POS	NEG	POS	NEG	NEG	NEG
2A8 (Czech Republic)	NEG	POS	NEG	NEG	NEG	NEG	NEG	NEG	NEG	NEG	POS	NEG	POS	NEG	NEG	NEG
M1229 (Denmark)	NEG	POS	NEG	NEG	NEG	NEG	NEG	NEG	NEG	NEG	POS	NEG	POS	NEG	NEG	NEG
FRICAR (France)	NEG	POS	NEG	NEG	NEG	NEG	NEG	NEG	NEG	NEG	POS	NEG	POS	NEG	NEG	NEG
D90 (Germany)	NEG	POS	NEG	NEG	NEG	NEG	NEG	NEG	NEG	NEG	POS	NEG	POS	NEG	NEG	NEG
LIT68 (Lithuania)	NEG	POS	NEG	NEG	NEG	NEG	NEG	NEG	NEG	NEG	POS	NEG	POS	NEG	NEG	NEG
LIT76 (Lithuania)	NEG	POS	NEG	NEG	NEG	NEG	NEG	NEG	NEG	NEG	POS	NEG	POS	NEG	NEG	NEG
HGSA142 (Portugal)	NEG	POS	NEG	NEG	NEG	NEG	NEG	NEG	NEG	NEG	POS	NEG	POS	NEG	NEG	NEG
HGSA9 (Portugal)	NEG	POS	NEG	NEG	NEG	NEG	NEG	NEG	NEG	NEG	POS	NEG	POS	NEG	NEG	NEG
HSJ216 (Portugal)	NEG	POS	NEG	NEG	NEG	NEG	NEG	NEG	NEG	NEG	POS	NEG	POS	NEG	NEG	NEG
M278 (Portugal)	NEG	POS	NEG	NEG	NEG	NEG	NEG	NEG	NEG	NEG	POS	NEG	POS	NEG	NEG	NEG
ES26 (Spain)	NEG	POS	NEG	NEG	NEG	NEG	NEG	NEG	NEG	NEG	POS	NEG	POS	NEG	NEG	NEG
UK102 (UK)	NEG	POS	NEG	NEG	NEG	NEG	NEG	NEG	NEG	NEG	POS	NEG	POS	NEG	NEG	NEG
URU34 (Uruguay)	NEG	POS	NEG	NEG	NEG	NEG	NEG	NEG	NEG	NEG	POS	NEG	POS	NEG	NEG	NEG
IS-125	NEG	POS	NEG	NEG	NEG	NEG	NEG	NEG	NEG	NEG	POS	NEG	POS	NEG	NEG	NEG
IS-157	NEG	POS	NEG	NEG	NEG	NEG	NEG	NEG	NEG	NEG	POS	NEG	POS	NEG	NEG	NEG
Ecuador_1	NEG	POS	NEG	NEG	NEG	NEG	NEG	NEG	NEG	NEG	POS	POS	POS	AMB	NEG	NEG
Ecuador_2	NEG	POS	NEG	NEG	NEG	NEG	NEG	NEG	NEG	NEG	POS	POS	POS	AMB	NEG	NEG
Dresden_15ANR574263	NEG	POS	NEG	NEG	NEG	AMB	NEG	NEG	NEG	NEG	POS	POS	POS	POS	NEG	AMB
UK-EMRSA-11	NEG	POS	NEG	NEG	NEG	NEG	NEG	NEG	NEG	NEG	POS	POS	POS	POS	NEG	NEG
>"South American/ Mid																
BRA2 (Brazil)	NEG	POS	NEG	NEG	NEG	NEG	NEG	NEG	NEG	NEG	POS	NEG	POS	NEG	NEG	NEG
CHL1 (Chile)	NEG	POS	NEG	NEG	NEG	NEG	NEG	NEG	NEG	NEG	POS	NEG	POS	NEG	NEG	NEG
CHL151 (Chile)	NEG	POS	NEG	NEG	NEG	NEG	NEG	NEG	NEG	NEG	POS	NEG	POS	NEG	NEG	NEG
UCL378 (Chile)	NEG	POS	NEG	NEG	NEG	NEG	NEG	NEG	NEG	NEG	POS	NEG	POS	NEG	NEG	NEG
UP72 (Peru)	NEG	POS	NEG	NEG	NEG	NEG	NEG	NEG	NEG	NEG	POS	NEG	POS	NEG	NEG	NEG
UP81 (Peru)	NEG	POS	NEG	NEG	NEG	NEG	NEG	NEG	NEG	NEG	POS	NEG	POS	NEG	NEG	NEG
>"South American/ Mid																
UP1073 (Peru)	NEG	POS	NEG	NEG	NEG	NEG	NEG	NEG	NEG	NEG	POS	NEG	POS	NEG	NEG	NEG

STRAIN / ISOLATE	MISCELLANEOUS GENES															
	sau				sau96I	G7ZRU6	ycjY	sagD	G7ZTC1		sdrM					
	sau3AI	sauUSI	sauRF122	sauSO385	sau96I	G7ZRU6	ycjY = C5Q1F1 ("Argenteus/ST1850-like", CC12, CC361, CC398)	sagD	G7ZTC1	G7ZTC1-argenteus	sdrM / tetEfflux	sdrM (cons)	hp_sdrM-801	hp_sdrM-802	sdrM (CC30)	sdrM (argen-teus)
	type II restriction-modification system endonuclease					acetyltransferase, GNAT family, "Argenteus/ST1850-like"		Putative bacteriocin biosynthesis associated protein	TetR family regulator		Multidrug resistance transporter					
>"South American/ Mid																
LIT89 (Lithuania)	NEG	POS	NEG	NEG	NEG	NEG	NEG	NEG	NEG	NEG	POS	NEG	POS	NEG	NEG	NEG
MRSA_PR1 (Malaysia)	NEG	POS	NEG	NEG	NEG	NEG	NEG	NEG	NEG	NEG	POS	NEG	POS	NEG	NEG	NEG
Riyadh-2888905-R	NEG	POS	NEG	NEG	NEG	NEG	NEG	NEG	NEG	NEG	POS	POS	POS	POS	NEG	NEG
Riyadh-2888915-BC	NEG	POS	NEG	NEG	NEG	NEG	NEG	NEG	NEG	NEG	POS	POS	POS	POS	NEG	NEG
Riyadh-2793706-R	NEG	POS	NEG	NEG	NEG	NEG	NEG	NEG	NEG	NEG	POS	POS	POS	POS	NEG	NEG
>"South American/ Mid																
Riyadh_SSTI_18_3502925	NEG	POS	NEG	NEG	NEG	NEG	NEG	NEG	NEG	NEG	POS	POS	POS	POS	NEG	NEG
Riyadh-2822825-W	NEG	POS	NEG	NEG	NEG	NEG	NEG	NEG	NEG	NEG	POS	POS	POS	POS	NEG	NEG
>"South American/ Mid																
UK-EMRSA-9	NEG	POS	NEG	NEG	NEG	NEG	NEG	NEG	NEG	NEG	POS	POS	POS	POS	NEG	NEG
>"South American/ Mid																
Lome_HT20020815	NEG	POS	NEG	NEG	NEG	NEG	NEG	NEG	NEG	NEG	POS	POS	POS	POS	NEG	NEG
>"South American/ Mid																
NA32 (Denmark)	NEG	POS	NEG	NEG	NEG	NEG	NEG	NEG	NEG	NEG	POS	NEG	POS	NEG	NEG	NEG
H24 (Egypt)	NEG	POS	NEG	NEG	NEG	NEG	NEG	NEG	NEG	NEG	POS	NEG	POS	NEG	NEG	NEG
ATCC BAA-39 (=HUSA304) (Hungary)	NEG	POS	NEG	NEG	NEG	NEG	NEG	NEG	NEG	NEG	POS	NEG	POS	NEG	NEG	NEG
HUSA304 (Hungary)	NEG	POS	NEG	NEG	NEG	NEG	NEG	NEG	NEG	NEG	POS	NEG	POS	NEG	NEG	NEG
HU106 (Hungary)	NEG	POS	NEG	NEG	NEG	NEG	NEG	NEG	NEG	NEG	POS	NEG	POS	NEG	NEG	NEG
BSAC27 (UK)	NEG	POS	NEG	NEG	NEG	NEG	NEG	NEG	NEG	NEG	POS	NEG	POS	NEG	NEG	NEG
NCTC13131, UK-EMRSA-4 (UK)	NEG	POS	NEG	NEG	NEG	NEG	NEG	NEG	NEG	NEG	POS	NEG	POS	NEG	NEG	NEG
	NEG	POS	NEG	NEG	NEG	NEG	NEG	NEG	NEG	NEG	POS	POS	POS	AMB	NEG	AMB
Algiers_HT20040080	NEG	POS	NEG	NEG	NEG	NEG	NEG	NEG	NEG	NEG	POS	POS	POS	POS	NEG	NEG
Dublin-DSH_AR09_0.0066	NEG	POS	NEG	NEG	NEG	NEG	NEG	NEG	NEG	NEG	POS	POS	POS	POS	NEG	NEG
Dublin-DSH_AR09_0-0065	NEG	NEG	NEG	NEG	NEG	NEG	NEG	NEG	NEG	NEG	POS	POS	POS	POS	NEG	NEG
Dublin-DSH_Phenotype-III_84	NEG	POS	NEG	NEG	NEG	NEG	NEG	NEG	NEG	NEG	POS	POS	POS	POS	NEG	NEG
Hong Kong_130	NEG	POS	NEG	NEG	NEG	NEG	NEG	NEG	NEG	NEG	POS	POS	POS	POS	NEG	NEG
Kuwait_018	NEG	POS	NEG	NEG	NEG	NEG	NEG	NEG	NEG	NEG	POS	POS	POS	POS	NEG	NEG
Perth_08-17726	NEG	POS	NEG	NEG	NEG	NEG	NEG	NEG	NEG	NEG	POS	POS	POS	POS	NEG	NEG
Riyadh_Alfaisal/KKKSUH_86_MRS-14-279	NEG	POS	NEG	NEG	NEG	NEG	NEG	NEG	NEG	NEG	POS	POS	POS	POS	NEG	NEG
Riyadh_Alfaisal-04_Z3861_831588	NEG	POS	NEG	NEG	NEG	NEG	NEG	NEG	NEG	NEG	POS	POS	POS	POS	NEG	NEG
Riyadh_Alfaisal-30_S15724_1108013	NEG	POS	NEG	NEG	NEG	NEG	NEG	NEG	NEG	NEG	POS	POS	POS	POS	NEG	NEG
Riyadh_Alfaisal-6_22A_13_B3992_397721	NEG	POS	NEG	NEG	NEG	NEG	NEG	NEG	NEG	NEG	POS	AMB	POS	NEG	NEG	NEG
Riyadh_SSTI_52_3615482	NEG	POS	NEG	NEG	NEG	NEG	NEG	NEG	NEG	NEG	POS	POS	POS	POS	NEG	NEG
Riyadh-2817276-2	NEG	POS	NEG	NEG	NEG	NEG	NEG	NEG	NEG	NEG	POS	POS	POS	POS	NEG	NEG
Riyadh-2891670-W	NEG	POS	NEG	NEG	NEG	NEG	NEG	NEG	NEG	NEG	POS	POS	POS	POS	NEG	NEG
Riyadh-3006920-W	NEG	POS	NEG	NEG	NEG	NEG	NEG	NEG	NEG	NEG	POS	POS	POS	POS	NEG	NEG
Riyadh-R2567782	NEG	POS	NEG	NEG	NEG	NEG	NEG	NEG	NEG	NEG	POS	POS	POS	POS	NEG	NEG
Russia-18_0252_Krasnoyarsk_SK2	NEG	POS	NEG	NEG	NEG	NEG	NEG	NEG	NEG	NEG	POS	POS	POS	POS	NEG	NEG
UK-EMRSA-7	NEG	POS	NEG	NEG	NEG	NEG	NEG	NEG	NEG	NEG	POS	POS	POS	POS	NEG	NEG
>"South American/ Mid																
Dublin-DSH_AR23_0073	NEG	POS	NEG	NEG	NEG	NEG	NEG	NEG	NEG	NEG	POS	POS	POS	POS	NEG	NEG
>Related to "South Ame																
MRSA-OC3 (Russia)	NEG	POS	NEG	NEG	NEG	NEG	NEG	NEG	NEG	NEG	POS	NEG	POS	NEG	NEG	NEG
Russia-12_0176_Krasnoyarsk	NEG	POS	NEG	NEG	NEG	NEG	NEG	NEG	NEG	NEG	POS	POS	POS	AMB	NEG	NEG
Russia-13_0180_Krasnoyarsk	NEG	POS	NEG	NEG	NEG	NEG	NEG	NEG	NEG	NEG	POS	POS	POS	POS	NEG	NEG
Russia-16_0249_Krasnoyarsk	NEG	POS	NEG	NEG	NEG	NEG	NEG	NEG	NEG	NEG	POS	POS	POS	AMB	NEG	NEG
Russia-17_0250_Krasnoyarsk	NEG	AMB	NEG	NEG	NEG	NEG	NEG	NEG	NEG	NEG	POS	AMB	POS	NEG	NEG	NEG
>Related to "South Ame																
URU110 (Uruguay)	NEG	POS	NEG	NEG	NEG	NEG	NEG	NEG	NEG	NEG	POS	NEG	POS	NEG	NEG	NEG
>Related to "South Ame																
DS_014 (Thailand)	NEG	POS	NEG	NEG	NEG	NEG	NEG	NEG	NEG	NEG	POS	NEG	POS	NEG	NEG	NEG
>Unassigned Middle Eas																
Frankfurt_Oder_0490031797 (Libya)	NEG	POS	NEG	NEG	NEG	NEG	NEG	NEG	NEG	NEG	POS	POS	POS	POS	NEG	NEG
Kuwait_192	NEG	POS	NEG	NEG	NEG	NEG	NEG	NEG	NEG	NEG	POS	POS	POS	POS	NEG	NEG
Riyadh-9	NEG	POS	NEG	NEG	NEG	NEG	NEG	NEG	NEG	NEG	POS	POS	POS	POS	NEG	NEG
Russia-24_0407_Moscow	NEG	AMB	NEG	NEG	NEG	NEG	NEG	NEG	NEG	NEG	POS	POS	POS	NEG	NEG	NEG

STRAIN / ISOLATE	MISCELLANEOUS GENES			HYALURONATE LYASE							
	Q2YUB3			hysA1			hysA2				
	Q2YUB3 (RF122)	Q2YUB3 (Swar)	Q2YUB3 (Sepi)	hysA1 (MRSA252)	hysA1 (MRSA252+RF122) and/or hysA2 (cons)	hysA1 (MRSA252+RF122) and/or hysA2 (COL+USA300)	hysA2 (All Other Than MRSA252)	hysA2 (COL+USA300+NCTC8325)	hysA2 (All Other Than COL+USA300+NCTC8325)	hysA2 (All Other Than COL+USA300+NCTC8325)	hysA2 (MRSA252)
	Multidrug resistance transporter			Hyaluronate lyase, variable first / second locus			Hyaluronate lyase, second locus				
**>"Eurasian Clade": TUR1**											
TUR1 (Turkey)	NEG	NEG	NEG	NEG	POS	NEG	POS	POS	NEG	NEG	NEG
TUR9 (Turkey)	NEG	NEG	NEG	NEG	POS	NEG	POS	POS	NEG	NEG	NEG
**>"Eurasian Clade" : T013**											
T0131 (China)	NEG	NEG	NEG	NEG	POS	NEG	POS	POS	NEG	NEG	NEG
MU4 (Turkey)	NEG	NEG	NEG	NEG	POS	NEG	POS	POS	NEG	NEG	NEG
DEU11 (Turkey)	NEG	NEG	NEG	NEG	POS	NEG	POS	POS	NEG	NEG	NEG
DEU16 (Turkey)	NEG	NEG	NEG	NEG	POS	NEG	POS	POS	NEG	NEG	NEG
Romania_Iasi_MRSA-07_54	NEG	NEG	NEG	NEG	POS	POS	POS	POS	NEG	NEG	NEG
**>"Eurasian Clade": arsC-**											
Romania_Iasi_MRSA-05_46	NEG	NEG	NEG	NEG	POS	POS	POS	POS	NEG	NEG	NEG
Romania_Iasi_MRSA-06_47	NEG	NEG	NEG	NEG	POS	POS	POS	POS	NEG	NEG	NEG
**>"Eurasian Clade": 16K/**											
CN79 (China)	NEG	NEG	NEG	NEG	POS	NEG	POS	POS	NEG	NEG	NEG
CUHK_BJ2002 (China)	NEG	NEG	NEG	NEG	POS	NEG	POS	POS	NEG	NEG	NEG
CUHK_BJ2007 (China)	NEG	NEG	NEG	NEG	POS	NEG	POS	POS	NEG	NEG	NEG
3HK (Czech Republic)	NEG	NEG	NEG	NEG	POS	NEG	POS	POS	NEG	NEG	NEG
HU109 (Hungary)	NEG	NEG	NEG	NEG	POS	NEG	POS	POS	NEG	NEG	NEG
HUR18 (Hungary)	NEG	NEG	NEG	NEG	POS	NEG	POS	POS	NEG	NEG	NEG
H482 (Romania)	NEG	NEG	NEG	NEG	POS	NEG	POS	POS	NEG	NEG	NEG
16K (Russia)	NEG	NEG	NEG	NEG	POS	NEG	POS	POS	NEG	NEG	NEG
DEU10 (Turkey)	NEG	NEG	NEG	NEG	POS	NEG	POS	POS	NEG	NEG	NEG
DEU12 (Turkey)	NEG	NEG	NEG	NEG	POS	NEG	POS	POS	NEG	NEG	NEG
DEU14 (Turkey)	NEG	NEG	NEG	NEG	POS	NEG	POS	POS	NEG	NEG	NEG
DEU15 (Turkey)	NEG	NEG	NEG	NEG	POS	NEG	POS	POS	NEG	NEG	NEG
DEU17 (Turkey)	NEG	NEG	NEG	NEG	POS	NEG	POS	POS	NEG	NEG	NEG
DEU19 (Turkey)	NEG	NEG	NEG	NEG	POS	NEG	POS	POS	NEG	NEG	NEG
DEU2 (Turkey)	NEG	NEG	NEG	NEG	POS	NEG	POS	POS	NEG	NEG	NEG
DEU20 (Turkey)	NEG	NEG	NEG	NEG	POS	NEG	POS	POS	NEG	NEG	NEG
DEU23 (Turkey)	NEG	NEG	NEG	NEG	POS	NEG	POS	POS	NEG	NEG	NEG
DEU3 (Turkey)	NEG	NEG	NEG	NEG	POS	NEG	POS	POS	NEG	NEG	NEG
DEU5 (Turkey)	NEG	NEG	NEG	NEG	POS	NEG	POS	POS	NEG	NEG	NEG
DEU6 (Turkey)	NEG	NEG	NEG	NEG	POS	NEG	POS	POS	NEG	NEG	NEG
DEU8 (Turkey)	NEG	NEG	NEG	NEG	POS	NEG	POS	POS	NEG	NEG	NEG
HU11 (Turkey)	NEG	NEG	NEG	NEG	POS	NEG	POS	POS	NEG	NEG	NEG
HU13 (Turkey)	NEG	NEG	NEG	NEG	POS	NEG	POS	POS	NEG	NEG	NEG
HU14 (Turkey)	NEG	NEG	NEG	NEG	POS	NEG	POS	POS	NEG	NEG	NEG
HU15 (Turkey)	NEG	NEG	NEG	NEG	POS	NEG	POS	POS	NEG	NEG	NEG
HU16 (Turkey)	NEG	NEG	NEG	NEG	POS	NEG	POS	POS	NEG	NEG	NEG
HU17 (Turkey)	NEG	NEG	NEG	NEG	POS	NEG	POS	POS	NEG	NEG	NEG
HU21 (Turkey)	NEG	NEG	NEG	NEG	POS	NEG	POS	POS	NEG	NEG	NEG
HU23 (Turkey)	NEG	NEG	NEG	NEG	POS	NEG	POS	POS	NEG	NEG	NEG
HU26 (Turkey)	NEG	NEG	NEG	NEG	POS	NEG	POS	POS	NEG	NEG	NEG
HU4 (Turkey)	NEG	NEG	NEG	NEG	POS	NEG	POS	POS	NEG	NEG	NEG
HU5 (Turkey)	NEG	NEG	NEG	NEG	POS	NEG	POS	POS	NEG	NEG	NEG
HU6 (Turkey)	NEG	NEG	NEG	NEG	POS	NEG	POS	POS	NEG	NEG	NEG
HU7 (Turkey)	NEG	NEG	NEG	NEG	POS	NEG	POS	POS	NEG	NEG	NEG
HU8 (Turkey)	NEG	NEG	NEG	NEG	POS	NEG	POS	POS	NEG	NEG	NEG
HU9 (Turkey)	NEG	NEG	NEG	NEG	POS	NEG	POS	POS	NEG	NEG	NEG
IU1 (Turkey)	NEG	NEG	NEG	NEG	POS	NEG	POS	POS	NEG	NEG	NEG
IU10 (Turkey)	NEG	NEG	NEG	NEG	POS	NEG	POS	POS	NEG	NEG	NEG
IU11 (Turkey)	NEG	NEG	NEG	NEG	POS	NEG	POS	POS	NEG	NEG	NEG
IU12 (Turkey)	NEG	NEG	NEG	NEG	POS	NEG	POS	POS	NEG	NEG	NEG
IU13 (Turkey)	NEG	NEG	NEG	NEG	POS	NEG	POS	POS	NEG	NEG	NEG
IU15 (Turkey)	NEG	NEG	NEG	NEG	POS	NEG	POS	POS	NEG	NEG	NEG
IU18 (Turkey)	NEG	NEG	NEG	NEG	POS	NEG	POS	POS	NEG	NEG	NEG
IU19 (Turkey)	NEG	NEG	NEG	NEG	POS	NEG	POS	POS	NEG	NEG	NEG
IU2 (Turkey)	NEG	NEG	NEG	NEG	POS	NEG	POS	POS	NEG	NEG	NEG
IU4 (Turkey)	NEG	NEG	NEG	NEG	POS	NEG	POS	POS	NEG	NEG	NEG
IU6 (Turkey)	NEG	NEG	NEG	NEG	NEG	NEG	NEG	NEG	NEG	NEG	NEG
IU7 (Turkey)	NEG	NEG	NEG	NEG	POS	NEG	POS	POS	NEG	NEG	NEG
IU9 (Turkey)	NEG	NEG	NEG	NEG	POS	NEG	POS	POS	NEG	NEG	NEG
MU1 (Turkey)	NEG	NEG	NEG	NEG	POS	NEG	POS	POS	NEG	NEG	NEG
MU10 (Turkey)	NEG	NEG	NEG	NEG	POS	NEG	POS	POS	NEG	NEG	NEG
MU20 (Turkey)	NEG	NEG	NEG	NEG	NEG	NEG	NEG	NEG	NEG	NEG	NEG
Mu3 (Turkey)	NEG	NEG	NEG	NEG	POS	NEG	POS	POS	NEG	NEG	NEG
MU5 (Turkey)	NEG	NEG	NEG	NEG	POS	NEG	POS	POS	NEG	NEG	NEG
MU6 (Turkey)	NEG	NEG	NEG	NEG	POS	NEG	POS	POS	NEG	NEG	NEG
MU7 (Turkey)	NEG	NEG	NEG	NEG	POS	NEG	POS	POS	NEG	NEG	NEG
TUR27 (Turkey)	NEG	NEG	NEG	NEG	POS	NEG	POS	POS	NEG	NEG	NEG
NCTR 32S	NEG	NEG	NEG	NEG	POS	NEG	POS	POS	NEG	NEG	NEG
SA02_A4 = Russia-06_0085_Moscow	NEG	NEG	NEG	NEG	POS	NEG	POS	POS	NEG	NEG	NEG
	NEG	NEG	NEG	NEG	POS	POS	POS	POS	NEG	NEG	NEG
Dresden_08V35987 (Turkey)	NEG	NEG	NEG	NEG	POS	POS	POS	POS	NEG	NEG	NEG
Dresden_17ANR577152	NEG	NEG	NEG	NEG	POS	POS	POS	POS	NEG	NEG	NEG
Dresden_17ANR580374 (Makedonia)	NEG	NEG	NEG	NEG	POS	POS	POS	POS	NEG	NEG	NEG
Hong Kong_69-II	NEG	NEG	NEG	NEG	POS	POS	POS	POS	NEG	NEG	NEG
Hong Kong_93	NEG	NEG	NEG	NEG	POS	POS	POS	POS	NEG	NEG	POS
Rawalpindi_Kidney Center_03_SK1	NEG	NEG	NEG	NEG	POS	POS	POS	POS	NEG	NEG	NEG
Rawalpindi_Kidney Center_08	NEG	NEG	NEG	NEG	POS	POS	POS	POS	NEG	NEG	NEG
Rawalpindi_Kidney Center_10	NEG	NEG	NEG	NEG	POS	POS	POS	POS	NEG	NEG	NEG
Rawalpindi_Kidney Center_19	NEG	NEG	NEG	NEG	POS	POS	POS	POS	NEG	NEG	NEG
Rawalpindi_Kidney Center_50_SK1	NEG	NEG	NEG	NEG	POS	POS	POS	POS	NEG	NEG	NEG
Romania_Iasi_BC-49_430	NEG	NEG	NEG	NEG	POS	POS	POS	POS	NEG	NEG	NEG
Romania_Iasi_MRSA_284	NEG	NEG	NEG	NEG	POS	POS	POS	POS	NEG	NEG	NEG
Romania_Iasi_MRSA-32_318	NEG	NEG	#ZAHL!	NEG	POS	POS	POS	POS	NEG	NEG	NEG
Romania_Iasi_MRSA-54_430	NEG	NEG	NEG	NEG	POS	POS	POS	POS	NEG	NEG	NEG
Romania_Iasi_SSTI-01_2	NEG	NEG	NEG	NEG	POS	POS	POS	POS	NEG	NEG	NEG
Romania_Iasi_SSTI-10_101	NEG	NEG	NEG	NEG	POS	POS	POS	POS	NEG	NEG	NEG
Romania_Iasi_SSTI-11_106	NEG	NEG	NEG	NEG	POS	POS	POS	POS	NEG	NEG	NEG
Russia-01_0001_SaintPetersburg	NEG	NEG	NEG	NEG	POS	POS	POS	POS	NEG	NEG	NEG
Russia-02_0004_SaintPetersburg	NEG	NEG	NEG	NEG	POS	POS	POS	POS	NEG	NEG	NEG
Russia-03_0057_SaintPetersburg	NEG	NEG	NEG	NEG	POS	POS	POS	POS	NEG	NEG	NEG
Russia-04_0076_Moscow	NEG	NEG	NEG	NEG	POS	POS	POS	POS	NEG	NEG	NEG
Russia-05_0078_Moscow	NEG	NEG	NEG	NEG	POS	POS	POS	POS	NEG	NEG	NEG
Russia-08_0135_Moscow	NEG	NEG	NEG	NEG	POS	POS	POS	POS	NEG	NEG	AMB
Russia-09_0150_Kurgan	NEG	NEG	NEG	NEG	POS	POS	POS	POS	NEG	NEG	NEG
Russia-10_0162_Kurgan	NEG	NEG	NEG	NEG	POS	POS	POS	POS	NEG	NEG	NEG
Russia-14_0184_Moscow	NEG	NEG	NEG	NEG	POS	POS	POS	POS	NEG	NEG	NEG
Russia-15_0232_SaintPetersburg	NEG	NEG	NEG	NEG	POS	POS	POS	POS	NEG	NEG	NEG
Russia-22_0391_Chelyabinsk	NEG	NEG	NEG	NEG	POS	POS	POS	POS	NEG	NEG	NEG
Russia-23_0392_Chelyabinsk	NEG	NEG	NEG	NEG	POS	POS	POS	POS	NEG	NEG	NEG
**>"Eurasian Clade" : IU17**											
IU17 (Turkey)	NEG	NEG	NEG	NEG	POS	NEG	POS	POS	NEG	NEG	NEG
IU20 (Turkey)	NEG	NEG	NEG	NEG	POS	NEG	POS	POS	NEG	NEG	NEG

STRAIN / ISOLATE	MISCELLANEOUS GENES			HYALURONATE LYASE							
	Q2YUB3			hysA1			hysA2				
	Q2YUB3 (RF122)	Q2YUB3 (Swar)	Q2YUB3 (Sepi)	hysA1 (MRSA252)	hysA1 (MRSA252+RF122) and/or hysA2 (cons)	hysA1 (MRSA252+RF122) and/or hysA2 (COL+USA300)	hysA2 (All Other Than MRSA252)	hysA2 (COL+USA300+NCTC8325)	hysA2 (All Other Than COL+USA300+NCTC8325)	hysA2 (All Other Than COL+USA300+NCTC8325)	hysA2 (MRSA252)
	Multidrug resistance transporter			Hyaluronate lyase, variable first / second locus			Hyaluronate lyase, second locus				
**>"Eurasian Clade": Spora**											
Dresden_16ANR581769	NEG	NEG	NEG	NEG	POS	POS	POS	POS	NEG	NEG	NEG
Dresden_17ANR578769	NEG	NEG	NEG	NEG	POS	POS	POS	POS	NEG	NEG	NEG
**>"Eurasian Clade": Kuwa**											
Kuwait_306	NEG	NEG	NEG	NEG	POS	POS	POS	POS	NEG	NEG	POS
Kuwait_301	NEG	NEG	NEG	NEG	POS	POS	POS	POS	NEG	NEG	POS
**>"European Clade": "Gre**											
GRE18 (Greece)	NEG	NEG	NEG	NEG	POS	NEG	POS	POS	NEG	NEG	NEG
GRE317 (Greece)	NEG	NEG	NEG	NEG	POS	NEG	POS	POS	NEG	NEG	NEG
GRE4 (Greece)	NEG	NEG	NEG	NEG	POS	NEG	POS	POS	NEG	NEG	NEG
R35 (USA)	NEG	NEG	NEG	NEG	POS	NEG	POS	POS	NEG	NEG	NEG
Casablanca_HT20060548	NEG	NEG	NEG	NEG	POS	POS	POS	POS	NEG	NEG	NEG
Casablanca_HT20060550	NEG	NEG	NEG	NEG	POS	POS	POS	POS	NEG	NEG	NEG
Dresden_01V34572 (Greece)	NEG	NEG	NEG	NEG	POS	POS	NEG	POS	NEG	NEG	NEG
Dresden_01V36469	NEG	NEG	NEG	NEG	POS	POS	POS	POS	NEG	NEG	NEG
Dresden_01V39123	NEG	NEG	NEG	NEG	POS	POS	POS	POS	NEG	NEG	AMB
Dresden_01V39143	NEG	NEG	NEG	NEG	POS	POS	POS	POS	NEG	NEG	AMB
Greece 1_3680_Harmony	NEG	NEG	NEG	NEG	POS	POS	NEG	POS	NEG	NEG	NEG
**>"European Clade": UK-(**											
ANS46 (Australia)	NEG	NEG	NEG	NEG	POS	NEG	POS	POS	NEG	NEG	NEG
LHH1 (USA)	NEG	NEG	NEG	NEG	POS	NEG	POS	POS	NEG	NEG	NEG
Dublin-DSH_AR01_0-1085	NEG	NEG	NEG	NEG	POS	POS	POS	POS	NEG	NEG	NEG
Dublin-DSH_AR01_0-1118	NEG	NEG	NEG	NEG	POS	POS	POS	POS	NEG	NEG	NEG
Dublin-DSH_AR15_0098	NEG	NEG	NEG	NEG	POS	POS	AMB	POS	NEG	NEG	NEG
Dublin-DSH_AR15_0104	NEG	NEG	NEG	NEG	POS	POS	POS	POS	NEG	NEG	NEG
UK_NCTC11939_UK-1_Harmony	NEG	NEG	NEG	NEG	POS	POS	POS	POS	NEG	NEG	NEG
**>"European Clade": BK2**											
BK2421 (USA)	NEG	NEG	NEG	NEG	POS	NEG	POS	POS	NEG	NEG	NEG
**>"Aussi/NZ Clade", JKD6**											
JKD6009 (Australia)	NEG	NEG	NEG	NEG	POS	NEG	POS	POS	NEG	NEG	NEG
JKD6008 (New Zealand)	NEG	NEG	NEG	NEG	POS	NEG	POS	POS	NEG	NEG	NEG
Perth_2015-S20209	NEG	NEG	NEG	NEG	POS	NEG	POS	POS	NEG	NEG	NEG
Perth_2015-S20305	NEG	NEG	NEG	NEG	POS	NEG	POS	POS	NEG	NEG	NEG
Perth_2015-S20603	NEG	NEG	NEG	NEG	POS	NEG	POS	POS	NEG	NEG	NEG
Perth_2015-S20607	NEG	NEG	NEG	NEG	POS	NEG	POS	POS	NEG	NEG	NEG
Perth_2015-S20608	NEG	NEG	NEG	NEG	POS	NEG	POS	POS	NEG	NEG	NEG
Perth_2015-S20610	NEG	NEG	NEG	NEG	POS	NEG	POS	POS	NEG	NEG	NEG
Perth_2015-S20613	NEG	NEG	NEG	NEG	POS	NEG	POS	POS	NEG	NEG	NEG
Perth_2015-S20617	NEG	NEG	NEG	NEG	POS	NEG	POS	POS	NEG	NEG	NEG
Perth_2015-S20622	NEG	NEG	NEG	NEG	POS	NEG	POS	POS	NEG	NEG	NEG
Perth_2015-S23619	NEG	NEG	NEG	NEG	NEG	NEG	NEG	NEG	NEG	NEG	NEG
Perth_2015-S41210	NEG	NEG	NEG	NEG	POS	NEG	POS	POS	NEG	NEG	NEG
Perth_2015-S51001	NEG	NEG	NEG	NEG	POS	NEG	POS	POS	NEG	NEG	NEG
Perth_2015-S51003	NEG	NEG	NEG	NEG	POS	NEG	POS	POS	NEG	NEG	NEG
Perth_2015-S51006	NEG	NEG	NEG	NEG	POS	NEG	POS	POS	NEG	NEG	NEG
Perth_2015-S51007	NEG	NEG	NEG	NEG	POS	NEG	POS	POS	NEG	NEG	NEG
Perth_2015-S51008	NEG	NEG	NEG	NEG	POS	NEG	POS	POS	NEG	NEG	NEG
Perth_2015-S51014	NEG	NEG	NEG	NEG	POS	NEG	POS	POS	NEG	NEG	NEG
Perth_2015-S51408	NEG	NEG	NEG	NEG	POS	NEG	POS	POS	NEG	NEG	NEG
Perth_2016-S20614	NEG	NEG	NEG	NEG	POS	NEG	POS	POS	NEG	NEG	NEG
Perth_2016-S23614	NEG	NEG	NEG	NEG	NEG	NEG	NEG	NEG	NEG	NEG	NEG
Perth_2016-S42009	NEG	NEG	NEG	NEG	POS	NEG	POS	POS	NEG	NEG	NEG
Perth_2016-S51001	NEG	NEG	NEG	NEG	POS	NEG	POS	POS	NEG	NEG	NEG
Perth_2016-S51403	NEG	NEG	NEG	NEG	POS	NEG	POS	POS	NEG	NEG	NEG
Perth_07 RPA 20	NEG	NEG	NEG	NEG	POS	POS	POS	POS	NEG	NEG	NEG
Perth_08-19088	NEG	NEG	NEG	NEG	POS	POS	POS	POS	NEG	NEG	NEG
Perth_01-15351	NEG	NEG	NEG	NEG	POS	POS	POS	POS	NEG	NEG	NEG
Perth_01-15357	NEG	NEG	NEG	NEG	POS	POS	POS	POS	NEG	NEG	NEG
Perth_01-15419	NEG	NEG	NEG	NEG	POS	POS	POS	POS	NEG	NEG	NEG
Perth_01-16309	NEG	NEG	NEG	NEG	POS	POS	POS	POS	NEG	NEG	NEG
Perth_03 SNP 95	NEG	NEG	NEG	NEG	POS	POS	POS	POS	NEG	NEG	AMB
Perth_2003_AH_47	NEG	NEG	NEG	NEG	POS	POS	POS	POS	NEG	NEG	NEG
**>"Australian/NZ Clade":**											
Perth_2015-S20623	NEG	NEG	NEG	NEG	POS	NEG	POS	POS	NEG	NEG	NEG
Perth_2015-S20826	NEG	NEG	NEG	NEG	POS	NEG	POS	POS	NEG	NEG	NEG
Perth_05 NH 56	NEG	NEG	NEG	NEG	POS	POS	POS	POS	NEG	NEG	NEG
**>CC5-MRSA-III LA MRSA**											
Delbrück 38 isolate from domestic turkey	NEG	NEG	NEG	NEG	POS	NEG	POS	NEG	POS	POS	AMB
**> Staph. pseudintermed**											
KM1381	NEG	NEG	NEG	NEG	NEG	NEG	NEG	NEG	NEG	NEG	NEG
**>"South-East Asian Clad**											
M92 (Canada)	NEG	NEG	NEG	NEG	POS	NEG	POS	POS	NEG	NEG	NEG
CHI59 (China)	NEG	NEG	NEG	NEG	POS	NEG	POS	POS	NEG	NEG	NEG
CHI61 (China)	NEG	NEG	NEG	NEG	POS	NEG	POS	POS	NEG	NEG	NEG
CUHK_HK1997 (China)	NEG	NEG	NEG	NEG	POS	NEG	POS	POS	NEG	NEG	NEG
M996 (China)	NEG	NEG	NEG	NEG	POS	NEG	POS	POS	NEG	NEG	NEG
H211 (Denmark)	NEG	NEG	NEG	NEG	POS	NEG	POS	POS	NEG	NEG	NEG
H216 (Denmark)	NEG	NEG	NEG	NEG	POS	NEG	POS	POS	NEG	NEG	NEG
D71 (Germany)	NEG	NEG	NEG	NEG	POS	NEG	POS	POS	NEG	NEG	NEG
NMR05 (India)	NEG	NEG	NEG	NEG	POS	NEG	POS	POS	NEG	NEG	NEG
MAL11 (Malaysia)	NEG	NEG	NEG	NEG	POS	NEG	POS	POS	NEG	NEG	NEG
MAL9 (Malaysia)	NEG	NEG	NEG	NEG	POS	NEG	POS	POS	NEG	NEG	NEG
Na21 (Sri Lanka)	NEG	NEG	NEG	NEG	POS	NEG	POS	POS	NEG	NEG	NEG
M592 (Syria)	NEG	NEG	NEG	NEG	POS	NEG	POS	POS	NEG	NEG	NEG
H202 (Thailand)	NEG	NEG	NEG	NEG	POS	NEG	POS	POS	NEG	NEG	NEG
S102 (Thailand)	NEG	NEG	NEG	NEG	POS	NEG	POS	POS	NEG	NEG	NEG
S130 (Thailand)	NEG	NEG	NEG	NEG	POS	NEG	POS	POS	NEG	NEG	NEG
S40 (Thailand)	NEG	NEG	NEG	NEG	POS	NEG	POS	POS	NEG	NEG	NEG
S71 (Thailand)	NEG	NEG	NEG	NEG	POS	NEG	POS	POS	NEG	NEG	NEG
S87 (Thailand)	NEG	NEG	NEG	NEG	POS	NEG	POS	POS	NEG	NEG	NEG
S93 (Thailand)	NEG	NEG	NEG	NEG	POS	NEG	POS	POS	NEG	NEG	NEG
US_002 (Thailand)	NEG	NEG	NEG	NEG	POS	NEG	POS	POS	NEG	NEG	NEG
US_017 (Thailand)	NEG	NEG	NEG	NEG	POS	NEG	POS	POS	NEG	NEG	NEG
WAMC6102 (Thailand)	NEG	NEG	NEG	NEG	POS	NEG	POS	POS	NEG	NEG	NEG
NCTC_13626_TW20 (UK)	NEG	NEG	NEG	NEG	POS	NEG	POS	POS	NEG	NEG	NEG
BSAC3021 (UK)	NEG	NEG	NEG	NEG	POS	NEG	POS	POS	NEG	NEG	NEG
BSAC3046 (UK)	NEG	NEG	NEG	NEG	POS	NEG	POS	POS	NEG	NEG	NEG
1193_SAUR (USA)	NEG	NEG	NEG	NEG	POS	NEG	POS	POS	NEG	NEG	NEG
1194_SAUR (USA)	NEG	NEG	NEG	NEG	POS	NEG	POS	POS	NEG	NEG	NEG

STRAIN / ISOLATE	MISCELLANEOUS GENES			HYALURONATE LYASE							
	Q2YUB3			hysA1			hysA2				
	Q2YUB3 (RF122)	Q2YUB3 (Swar)	Q2YUB3 (Sepi)	hysA1 (MRSA252)	hysA1 (MRSA252+RF122) and/or hysA2 (cons)	hysA1 (MRSA252+RF122) and/or hysA2 (COL+USA300)	hysA2 (All Other Than MRSA252)	hysA2 (COL+USA300+NCTC8325)	hysA2 (All Other Than COL+USA300+NCTC8325)	hysA2 (All Other Than COL+USA300+NCTC8325)	hysA2 (MRSA252)
	Multidrug resistance transporter			Hyaluronate lyase, variable first / second locus			Hyaluronate lyase, second locus				
>"South-East Asian Clade": TW20 (Irish AR44), c											
1195_SAUR (USA)	NEG	NEG	NEG	NEG	POS	NEG	POS	POS	NEG	NEG	NEG
FVRH6002 (USA)	NEG	NEG	NEG	NEG	POS	NEG	POS	POS	NEG	NEG	NEG
KINW6048 (USA)	NEG	NEG	NEG	NEG	POS	NEG	POS	POS	NEG	NEG	NEG
LAMC0011 (USA)	NEG	NEG	NEG	NEG	POS	NEG	POS	POS	NEG	NEG	NEG
SJOS6053 (USA)	NEG	NEG	NEG	NEG	POS	NEG	POS	POS	NEG	NEG	NEG
SJOS6072 (USA)	NEG	NEG	NEG	NEG	POS	NEG	POS	POS	NEG	NEG	NEG
UCIM6042 (USA)	NEG	NEG	NEG	NEG	POS	NEG	POS	POS	NEG	NEG	NEG
Perth_2015-S31902	NEG	NEG	NEG	NEG	POS	NEG	POS	POS	NEG	NEG	NEG
Perth_2015-S31904	NEG	NEG	NEG	NEG	POS	NEG	POS	POS	NEG	NEG	NEG
Perth_2015-S31913	NEG	NEG	NEG	NEG	POS	NEG	POS	POS	NEG	NEG	NEG
Perth_2015-S31918	NEG	NEG	NEG	NEG	POS	NEG	NEG	POS	NEG	NEG	NEG
Dublin-DSH_AR44_E1183	NEG	NEG	NEG	NEG	POS	POS	POS	POS	NEG	NEG	NEG
Dublin-DSH_Unfamiliar-3_E1520	NEG	NEG	NEG	NEG	POS	POS	POS	POS	NEG	NEG	NEG
Hong Kong_042	NEG	NEG	NEG	NEG	POS	POS	POS	POS	NEG	NEG	NEG
Hong Kong_101	NEG	NEG	NEG	NEG	POS	POS	POS	POS	NEG	NEG	NEG
Hong Kong_111	NEG	NEG	NEG	NEG	POS	POS	POS	POS	NEG	NEG	AMB
Hong Kong_113	NEG	NEG	NEG	NEG	POS	POS	POS	POS	NEG	NEG	NEG
Hong Kong_115	NEG	NEG	NEG	NEG	POS	POS	POS	POS	NEG	NEG	NEG
Hong Kong_118	NEG	NEG	NEG	NEG	POS	POS	POS	POS	NEG	NEG	NEG
Hong Kong_125	NEG	NEG	NEG	NEG	POS	POS	POS	POS	NEG	NEG	AMB
Hong Kong_129	NEG	NEG	NEG	NEG	POS	POS	POS	POS	NEG	NEG	NEG
Hong Kong_133	NEG	NEG	NEG	NEG	POS	POS	POS	POS	NEG	NEG	NEG
Kuwait_009	NEG	NEG	NEG	NEG	POS	POS	POS	POS	NEG	NEG	NEG
Kuwait_103	NEG	NEG	NEG	NEG	POS	POS	POS	POS	NEG	NEG	POS
Kuwait_107	NEG	NEG	NEG	NEG	POS	POS	POS	POS	NEG	NEG	NEG
Perth_03 AH 88	NEG	NEG	NEG	NEG	POS	POS	POS	POS	NEG	NEG	NEG
Perth_08-16905	NEG	NEG	NEG	NEG	POS	POS	POS	POS	NEG	NEG	NEG
Riyadh_Alfaisal-10_SICU_13_B5672_1117353	NEG	NEG	NEG	NEG	POS	POS	POS	POS	NEG	NEG	NEG
Riyadh_SSTI_17_3508602	NEG	NEG	NEG	NEG	POS	POS	POS	POS	NEG	NEG	NEG
Riyadh-2822088-R	NEG	NEG	NEG	NEG	POS	POS	POS	POS	NEG	NEG	AMB
Trinidad&Tobago_2012_195 (619/12)	NEG	NEG	NEG	NEG	POS	POS	POS	POS	NEG	NEG	NEG
Trinidad&Tobago_2012_370 (1112-52177)	NEG	NEG	NEG	NEG	POS	POS	POS	POS	NEG	NEG	NEG
Trinidad&Tobago_2012_378 (4139)	NEG	NEG	NEG	NEG	POS	POS	POS	POS	NEG	NEG	AMB
Trinidad&Tobago_2013_PA21	NEG	NEG	NEG	NEG	POS	POS	POS	POS	NEG	NEG	NEG
Trinidad&Tobago_2013_PA22	NEG	NEG	NEG	NEG	POS	POS	POS	POS	NEG	NEG	AMB
Trinidad&Tobago_2912_340 (8523/12)	NEG	NEG	NEG	NEG	POS	POS	POS	POS	NEG	NEG	NEG
Trinidad&Tobago_MRSA2010_105	NEG	NEG	NEG	NEG	POS	POS	POS	POS	NEG	NEG	NEG
Trinidad&Tobago_MRSA2010_108	NEG	NEG	NEG	NEG	POS	POS	POS	POS	NEG	NEG	POS
Trinidad&Tobago_MRSA2010_33	NEG	NEG	NEG	NEG	POS	POS	POS	POS	NEG	NEG	NEG
Trinidad&Tobago_MRSA2010_79	NEG	NEG	NEG	NEG	POS	POS	POS	POS	NEG	NEG	NEG
Trinidad&Tobago_MRSA2010_88	NEG	NEG	NEG	NEG	POS	POS	POS	POS	NEG	NEG	NEG
Trinidad&Tobago_MRSA2010_97	NEG	NEG	NEG	NEG	POS	POS	POS	POS	NEG	NEG	NEG
Trinidad&Tobago_SSTI 2012_031	NEG	NEG	NEG	NEG	POS	POS	POS	POS	NEG	NEG	AMB
Uganda-29_17148_250811	NEG	NEG	NEG	NEG	POS	POS	POS	POS	NEG	NEG	NEG
>"South-East Asian Clad											
UCIM6015	NEG	NEG	NEG	NEG	POS	NEG	POS	POS	NEG	NEG	NEG
Hong Kong_121	NEG	NEG	NEG	NEG	POS	POS	POS	POS	NEG	NEG	NEG
>"South-East Asian Clad											
Z172 (Taiwan)	NEG	NEG	NEG	NEG	POS	NEG	POS	POS	NEG	NEG	NEG
Russia-19_0342_Kurgan	NEG	NEG	NEG	NEG	POS	POS	POS	POS	NEG	NEG	NEG
>"South-East Asian Clad											
Perth_01-15392	NEG	NEG	NEG	NEG	POS	POS	POS	POS	NEG	NEG	NEG
>"South-East Asian Clad											
XN108 (China)	NEG	NEG	NEG	NEG	POS	NEG	POS	POS	NEG	NEG	NEG
NMR09 (India)	NEG	NEG	NEG	NEG	POS	NEG	POS	POS	NEG	NEG	NEG
346 (Malaysia)	NEG	NEG	NEG	NEG	POS	NEG	POS	POS	NEG	NEG	NEG
DS_009 (Thailand)	NEG	NEG	NEG	NEG	POS	NEG	POS	POS	NEG	NEG	NEG
18ANR581005 (Ind)	NEG	NEG	NEG	NEG	POS	POS	POS	POS	NEG	NEG	NEG
Bengalaru_ST20141405	NEG	NEG	NEG	NEG	POS	POS	POS	POS	NEG	NEG	NEG
Bengalaru_ST20141406	NEG	NEG	NEG	NEG	POS	POS	POS	POS	NEG	NEG	NEG
Bengalaru_ST20141417	NEG	NEG	NEG	NEG	POS	POS	POS	POS	NEG	NEG	NEG
Hong Kong_107	NEG	NEG	NEG	NEG	POS	POS	POS	POS	NEG	NEG	NEG
Hong Kong_12	NEG	NEG	NEG	NEG	POS	POS	POS	POS	NEG	NEG	POS
Hong Kong_132	NEG	NEG	NEG	NEG	POS	POS	POS	POS	NEG	NEG	NEG
Riyadh-3028763-R	NEG	NEG	NEG	NEG	POS	POS	POS	POS	NEG	NEG	NEG
Trinidad&Tobago_MRSA2010_94	NEG	NEG	NEG	NEG	POS	POS	POS	POS	NEG	NEG	NEG
>"South-East Asian Clad											
M705 (Thailand)	NEG	NEG	NEG	NEG	POS	NEG	POS	POS	NEG	NEG	NEG
S85 (Thailand)	NEG	NEG	NEG	NEG	POS	NEG	POS	POS	NEG	NEG	NEG
Riyadh_Alfaisal-04_281014_S5076	NEG	NEG	NEG	NEG	POS	POS	POS	POS	NEG	NEG	POS
Riyadh_Alfaisal-26_S15145_1104163	NEG	NEG	NEG	NEG	POS	POS	POS	POS	NEG	NEG	NEG
Riyadh-2817437-W	NEG	NEG	NEG	NEG	POS	POS	POS	POS	NEG	NEG	AMB
>"South-East Asian Clad											
PPUKM-775-2009 (Malaysia)	NEG	NEG	NEG	NEG	POS	NEG	POS	POS	NEG	NEG	NEG
Hong Kong_138	NEG	NEG	NEG	NEG	POS	POS	POS	POS	NEG	NEG	NEG
>"South-East Asian Clad											
Kuwait_001	NEG	NEG	NEG	NEG	POS	POS	POS	POS	NEG	NEG	NEG
Kuwait_337	NEG	NEG	NEG	POS	POS	POS	POS	POS	NEG	NEG	NEG
>"South-East Asian Clad											
CUHK_HK2007 (China)	NEG	NEG	NEG	NEG	POS	NEG	POS	POS	NEG	NEG	NEG
NMR02 (India)	NEG	NEG	NEG	NEG	POS	NEG	POS	POS	NEG	NEG	NEG
VB1490 (India)	NEG	NEG	NEG	NEG	POS	NEG	POS	POS	NEG	NEG	NEG
V521 (Korea)	NEG	NEG	NEG	NEG	POS	NEG	POS	POS	NEG	NEG	NEG
HST-077 (Lebanon)	NEG	NEG	NEG	NEG	POS	NEG	POS	POS	NEG	NEG	NEG
MAL1 (Malaysia)	NEG	NEG	NEG	NEG	POS	NEG	POS	POS	NEG	NEG	NEG
MAL3 (Malaysia)	NEG	NEG	NEG	NEG	POS	NEG	POS	POS	NEG	NEG	NEG
PPUKM-261-2009 (Malaysia)	NEG	NEG	NEG	NEG	POS	NEG	POS	POS	NEG	NEG	NEG
PPUKM-332-2009 (Malaysia)	NEG	NEG	NEG	NEG	POS	NEG	POS	POS	NEG	NEG	NEG
S38 (Thailand)	NEG	NEG	NEG	NEG	POS	NEG	POS	POS	NEG	NEG	NEG
S7 (Thailand)	NEG	NEG	NEG	NEG	POS	NEG	POS	POS	NEG	NEG	NEG
US_008 (Thailand)	NEG	NEG	NEG	NEG	POS	NEG	POS	POS	NEG	NEG	NEG
US_030 (Thailand)	NEG	NEG	NEG	NEG	POS	NEG	POS	POS	NEG	NEG	NEG
M170 (Tsunami-related)	NEG	NEG	NEG	NEG	POS	NEG	POS	POS	NEG	NEG	NEG
BSAC697 (UK)	NEG	NEG	NEG	NEG	POS	NEG	POS	POS	NEG	NEG	NEG
IS-189	NEG	NEG	NEG	NEG	POS	NEG	POS	POS	NEG	NEG	NEG
MRGR3	NEG	NEG	NEG	NEG	POS	NEG	POS	POS	NEG	NEG	NEG
smg07	NEG	NEG	NEG	NEG	POS	NEG	POS	POS	NEG	NEG	NEG
AH1-AUS-EMRSA-3	NEG	NEG	NEG	NEG	POS	POS	POS	POS	NEG	NEG	NEG
Bengalaru_ST20141403	NEG	NEG	NEG	NEG	POS	POS	POS	POS	NEG	NEG	NEG
Finland E24_98541_Harmony	NEG	NEG	NEG	NEG	POS	POS	POS	POS	NEG	NEG	NEG
Hong Kong_10	NEG	NEG	NEG	NEG	POS	POS	POS	POS	NEG	NEG	NEG
Hong Kong_100	NEG	NEG	NEG	NEG	POS	POS	POS	POS	NEG	NEG	NEG
Hong Kong_199	NEG	NEG	NEG	NEG	POS	POS	POS	POS	NEG	NEG	POS
Hong Kong_34	NEG	NEG	NEG	NEG	POS	POS	POS	POS	NEG	NEG	AMB
Hong Kong_88	NEG	NEG	NEG	NEG	POS	POS	POS	POS	NEG	NEG	POS
Hong Kong_92	NEG	NEG	NEG	NEG	POS	POS	POS	POS	NEG	NEG	POS
Hong Kong_94	NEG	NEG	NEG	NEG	POS	POS	POS	POS	NEG	NEG	NEG
Kuwait_257	NEG	NEG	NEG	NEG	POS	POS	POS	POS	NEG	NEG	POS
Perth_01-15330	NEG	NEG	NEG	NEG	POS	POS	POS	POS	NEG	NEG	NEG
Perth_01-15349	NEG	NEG	NEG	NEG	POS	POS	POS	POS	NEG	NEG	NEG
Perth_01-16176	NEG	NEG	NEG	NEG	POS	POS	POS	POS	NEG	NEG	NEG
Perth_WBG8379	NEG	NEG	NEG	NEG	POS	POS	POS	POS	NEG	NEG	NEG
Rawalpindi_Kidney Center_56	NEG	NEG	NEG	NEG	POS	POS	POS	POS	NEG	NEG	NEG
Riyadh_SSTI_05_3464614	NEG	NEG	NEG	NEG	POS	POS	POS	POS	NEG	NEG	NEG
Trinidad&Tobago_2012_157	NEG	NEG	NEG	NEG	POS	POS	POS	POS	NEG	NEG	NEG

STRAIN / ISOLATE	MISCELLANEOUS GENES			HYALURONATE LYASE							
	Q2YUB3			hysA1			hysA2				
	Q2YUB3 (RF122)	Q2YUB3 (Swar)	Q2YUB3 (Sepi)	hysA1 (MRSA252)	hysA1 (MRSA252+RF122) and/or hysA2 (cons)	hysA1 (MRSA252+RF122) and/or hysA2 (COL+USA300)	hysA2 (All Other Than MRSA252)	hysA2 (COL+USA300+NCTC8325)	hysA2 (All Other Than COL+USA300+NCTC8325)	hysA2 (All Other Than COL+USA300+NCTC8325)	hysA2 (MRSA252)
	Multidrug resistance transporter			Hyaluronate lyase, variable first / second locus			Hyaluronate lyase, second locus				
>"South-East Asian Clad											
M418	NEG	NEG	NEG	NEG	POS	NEG	POS	POS	NEG	NEG	NEG
>"South-East Asian Clad											
NMR07 (India)	NEG	NEG	NEG	NEG	POS	NEG	POS	POS	NEG	NEG	NEG
NMR08 (India)	NEG	NEG	NEG	NEG	POS	NEG	POS	POS	NEG	NEG	NEG
Bengalaru_ST20121978	NEG	NEG	NEG	NEG	POS	POS	POS	POS	NEG	NEG	NEG
Hong Kong_89	NEG	NEG	NEG	NEG	POS	POS	POS	POS	NEG	NEG	POS
>"South-East Asian Clad											
Perth_2005_AH_5	NEG	NEG	NEG	NEG	POS	POS	POS	POS	NEG	NEG	NEG
>"South-East Asian Clad											
Trinidad&Tobago_MRSA2010_103	NEG	NEG	NEG	NEG	POS	POS	POS	POS	NEG	NEG	NEG
Trinidad&Tobago_2013_PA25	NEG	NEG	NEG	NEG	POS	POS	POS	POS	NEG	NEG	NEG
Trinidad&Tobago_MRSA2010_110	NEG	NEG	NEG	NEG	POS	POS	POS	POS	NEG	NEG	NEG
Trinidad&Tobago_SSTI 2012_111	NEG	NEG	NEG	NEG	POS	POS	POS	POS	NEG	NEG	NEG
Trinidad&Tobago_MRSA2010_158	NEG	NEG	NEG	NEG	POS	POS	POS	POS	NEG	NEG	NEG
>"South-East Asian Clad											
Kuwait_122	NEG	NEG	NEG	NEG	POS	POS	POS	POS	NEG	NEG	NEG
Kuwait_002	NEG	NEG	NEG	NEG	POS	POS	POS	POS	NEG	NEG	NEG
Kuwait_196	NEG	NEG	NEG	NEG	POS	POS	POS	POS	NEG	NEG	NEG
Kuwait_199	NEG	NEG	NEG	NEG	POS	POS	POS	POS	NEG	NEG	AMB
Hong Kong_136	NEG	NEG	NEG	NEG	POS	POS	POS	POS	NEG	NEG	NEG
>"South-East Asian Clad											
P32 (Poland)	NEG	NEG	NEG	NEG	POS	NEG	POS	POS	NEG	NEG	NEG
>"South-East Asian Clad											
Perth_2015-S32313	NEG	NEG	NEG	NEG	POS	NEG	POS	POS	NEG	NEG	NEG
>"hla-negative South-Ea											
DEN907 (Denmark)	NEG	NEG	NEG	NEG	POS	NEG	POS	POS	NEG	NEG	NEG
>"hla-negative South-Ea											
S26 (Thailand)	NEG	NEG	NEG	NEG	POS	NEG	POS	POS	NEG	NEG	NEG
S42 (Thailand)	NEG	NEG	NEG	NEG	POS	NEG	POS	POS	NEG	NEG	NEG
S97 (Thailand)	NEG	NEG	NEG	NEG	POS	NEG	POS	POS	NEG	NEG	NEG
S78 (Thailand)	NEG	NEG	NEG	NEG	POS	NEG	POS	POS	NEG	NEG	NEG
S25 (Thailand)	NEG	NEG	NEG	NEG	POS	NEG	POS	POS	NEG	NEG	NEG
S21 (Thailand)	NEG	NEG	NEG	NEG	POS	NEG	POS	POS	NEG	NEG	NEG
S106 (Thailand)	NEG	NEG	NEG	NEG	POS	NEG	POS	POS	NEG	NEG	NEG
S24 (Thailand)	NEG	NEG	NEG	NEG	POS	NEG	POS	POS	NEG	NEG	NEG
S39 (Thailand)	NEG	NEG	NEG	NEG	POS	NEG	POS	POS	NEG	NEG	NEG
S2 (Thailand)	NEG	NEG	NEG	NEG	POS	NEG	POS	POS	NEG	NEG	NEG
S81 (Thailand)	NEG	NEG	NEG	NEG	POS	NEG	POS	POS	NEG	NEG	NEG
M116 (Vietnam)	NEG	NEG	NEG	NEG	POS	NEG	POS	POS	NEG	NEG	NEG
>"Portuguese Clade": Pc											
FFP103 (Portugal)	NEG	NEG	NEG	NEG	POS	NEG	POS	POS	NEG	NEG	NEG
HDG2 (Portugal)	NEG	NEG	NEG	NEG	POS	NEG	POS	POS	NEG	NEG	NEG
HSA10 (Portugal)	NEG	NEG	NEG	NEG	POS	NEG	POS	POS	NEG	NEG	NEG
HSA11 (Portugal)	NEG	NEG	NEG	NEG	POS	NEG	POS	POS	NEG	NEG	NEG
ICP5011 (Portugal)	NEG	NEG	NEG	NEG	POS	NEG	POS	POS	NEG	NEG	NEG
ICP5014 (Portugal)	NEG	NEG	NEG	NEG	POS	NEG	POS	POS	NEG	NEG	NEG
ICP5062 (Portugal)	NEG	NEG	NEG	NEG	POS	NEG	POS	POS	NEG	NEG	NEG
Strain 1063_ATCC 33592 FDAARGOS_3 (USA)	NEG	NEG	NEG	NEG	POS	NEG	POS	POS	NEG	NEG	NEG
	NEG	NEG	NEG	NEG	POS	POS	POS	POS	NEG	NEG	NEG
Russia-07_0110_Kurgan	NEG	NEG	NEG	NEG	POS	POS	POS	POS	NEG	NEG	NEG
Russia-11_0164_Kurgan	NEG	NEG	NEG	NEG	POS	POS	POS	POS	NEG	NEG	AMB
Russia-20_0367_Kurgan	NEG	NEG	NEG	NEG	POS	POS	POS	POS	NEG	NEG	NEG
Russia-21_0390_Chelyabinsk	NEG	NEG	NEG	NEG	POS	POS	POS	POS	NEG	NEG	NEG
>"South American/ Mid											
AGT1 (Argentina)	NEG	NEG	NEG	NEG	POS	NEG	POS	POS	NEG	NEG	NEG
AGT120 (Argentina)	NEG	NEG	NEG	NEG	POS	NEG	POS	POS	NEG	NEG	NEG
AGT67 (Argentina)	NEG	NEG	NEG	NEG	POS	NEG	POS	POS	NEG	NEG	NEG
AGT9 (Argentina)	NEG	NEG	NEG	NEG	POS	NEG	POS	POS	NEG	NEG	NEG
RA3 (Argentina)	NEG	NEG	NEG	NEG	POS	NEG	POS	POS	NEG	NEG	NEG
RA6 (Argentina)	NEG	NEG	NEG	NEG	POS	NEG	POS	POS	NEG	NEG	NEG
RA7 (Argentina)	NEG	NEG	NEG	NEG	POS	NEG	POS	POS	NEG	NEG	NEG
Be62 (Brazil)	NEG	NEG	NEG	NEG	POS	NEG	POS	POS	NEG	NEG	NEG
Bmb9393 (Brazil)	NEG	NEG	NEG	NEG	POS	NEG	POS	POS	NEG	NEG	NEG
BRA36 (Brazil)	NEG	NEG	NEG	NEG	POS	NEG	POS	POS	NEG	NEG	NEG
BZ48 (Brazil)	NEG	NEG	NEG	NEG	POS	NEG	POS	POS	NEG	NEG	NEG
Gv51 (Brazil)	NEG	NEG	NEG	NEG	POS	NEG	POS	POS	NEG	NEG	NEG
Gv69 (Brazil)	NEG	NEG	NEG	NEG	POS	NEG	POS	POS	NEG	NEG	NEG
Gv88 (Brazil)	NEG	NEG	NEG	NEG	POS	NEG	POS	POS	NEG	NEG	NEG
HC1335 (Brazil)	NEG	NEG	NEG	NEG	POS	NEG	POS	POS	NEG	NEG	NEG
HC1340 (Brazil)	NEG	NEG	NEG	NEG	POS	NEG	POS	POS	NEG	NEG	NEG
HC556 (Brazil)	NEG	NEG	NEG	NEG	POS	NEG	POS	POS	NEG	NEG	NEG
HU25 (Brazil)	NEG	NEG	NEG	NEG	POS	NEG	POS	POS	NEG	NEG	NEG
UB563 (Brazil)	NEG	NEG	NEG	NEG	POS	NEG	POS	POS	NEG	NEG	NEG
2A8 (Czech Republic)	NEG	NEG	NEG	NEG	POS	NEG	POS	POS	NEG	NEG	NEG
M1229 (Denmark)	NEG	NEG	NEG	NEG	POS	NEG	POS	POS	NEG	NEG	NEG
FRICAR (France)	NEG	NEG	NEG	NEG	POS	NEG	POS	POS	NEG	NEG	NEG
D90 (Germany)	NEG	NEG	NEG	NEG	POS	NEG	POS	POS	NEG	NEG	NEG
LIT68 (Lithuania)	NEG	NEG	NEG	NEG	POS	NEG	POS	POS	NEG	NEG	NEG
LIT76 (Lithuania)	NEG	NEG	NEG	NEG	POS	NEG	POS	POS	NEG	NEG	NEG
HGSA142 (Portugal)	NEG	NEG	NEG	NEG	POS	NEG	POS	POS	NEG	NEG	NEG
HGSA9 (Portugal)	NEG	NEG	NEG	NEG	POS	NEG	POS	POS	NEG	NEG	NEG
HSJ216 (Portugal)	NEG	NEG	NEG	NEG	POS	NEG	POS	POS	NEG	NEG	NEG
M278 (Portugal)	NEG	NEG	NEG	NEG	POS	NEG	POS	POS	NEG	NEG	NEG
ES26 (Spain)	NEG	NEG	NEG	NEG	POS	NEG	POS	POS	NEG	NEG	NEG
UK102 (UK)	NEG	NEG	NEG	NEG	POS	NEG	POS	POS	NEG	NEG	NEG
URU34 (Uruguay)	NEG	NEG	NEG	NEG	POS	NEG	POS	POS	NEG	NEG	NEG
IS-125	NEG	NEG	NEG	NEG	POS	NEG	POS	POS	NEG	NEG	NEG
IS-157	NEG	NEG	NEG	NEG	POS	NEG	POS	POS	NEG	NEG	NEG
Ecuador_1	NEG	NEG	NEG	NEG	POS	POS	POS	POS	NEG	NEG	AMB
Ecuador_2	NEG	NEG	NEG	NEG	POS	POS	POS	POS	NEG	NEG	NEG
Dresden_15ANR574263	NEG	NEG	NEG	NEG	POS	POS	POS	POS	NEG	NEG	AMB
UK-EMRSA-11	NEG	NEG	NEG	NEG	POS	POS	POS	POS	NEG	NEG	NEG
>"South American/ Mid											
BRA2 (Brazil)	NEG	NEG	NEG	NEG	POS	NEG	POS	POS	NEG	NEG	NEG
CHL1 (Chile)	NEG	NEG	NEG	NEG	POS	NEG	POS	POS	NEG	NEG	NEG
CHL151 (Chile)	NEG	NEG	NEG	NEG	POS	NEG	POS	POS	NEG	NEG	NEG
UCL378 (Chile)	NEG	NEG	NEG	NEG	POS	NEG	POS	POS	NEG	NEG	NEG
UP72 (Peru)	NEG	NEG	NEG	NEG	POS	NEG	POS	POS	NEG	NEG	NEG
UP81 (Peru)	NEG	NEG	NEG	NEG	POS	NEG	POS	POS	NEG	NEG	NEG
>"South American/ Mid											
UP1073 (Peru)	NEG	NEG	NEG	NEG	POS	NEG	POS	POS	NEG	NEG	NEG

STRAIN / ISOLATE	MISCELLANEOUS GENES			HYALURONATE LYASE							
	Q2YUB3			hysA1			hysA2				
	Q2YUB3 (RF122)	Q2YUB3 (Swar)	Q2YUB3 (Sepi)	hysA1 (MRSA252)	hysA1 (MRSA252+RF122) and/or hysA2 (cons)	hysA1 (MRSA252+RF122) and/or hysA2 (COL+USA300)	hysA2 (All Other Than MRSA252)	hysA2 (COL+USA300+NCTC8325)	hysA2 (All Other Than COL+USA300+NCTC8325)	hysA2 (All Other Than COL+USA300+NCTC8325)	hysA2 (MRSA252)
	Multidrug resistance transporter			Hyaluronate lyase, variable first / second locus			Hyaluronate lyase, second locus				
>"South American/ Mid											
LIT89 (Lithuania)	NEG	NEG	NEG	NEG	POS	NEG	POS	POS	NEG	NEG	NEG
MRSA_PR1 (Malaysia)	NEG	NEG	NEG	NEG	POS	NEG	POS	POS	NEG	NEG	NEG
Riyadh-2888905-R	NEG	NEG	NEG	NEG	POS	POS	POS	POS	NEG	NEG	NEG
Riyadh-2888915-BC	NEG	NEG	NEG	NEG	POS	POS	POS	POS	NEG	NEG	NEG
Riyadh-2793706-R	NEG	NEG	NEG	NEG	POS	POS	POS	POS	NEG	NEG	NEG
>"South American/ Mid											
Riyadh_SSTI_18_3502925	NEG	NEG	NEG	NEG	POS	POS	POS	POS	NEG	NEG	POS
Riyadh-2822825-W	NEG	NEG	NEG	NEG	POS	POS	POS	POS	NEG	NEG	POS
>"South American/ Mid											
UK-EMRSA-9	NEG	NEG	NEG	NEG	POS	POS	POS	POS	NEG	NEG	NEG
>"South American/ Mid											
Lome_HT20020815	NEG	NEG	NEG	NEG	POS	POS	POS	POS	NEG	NEG	NEG
>"South American/ Mid											
NA32 (Denmark)	NEG	NEG	NEG	NEG	POS	NEG	POS	POS	NEG	NEG	NEG
H24 (Egypt)	NEG	NEG	NEG	NEG	POS	NEG	POS	POS	NEG	NEG	NEG
ATCC BAA-39 (=HUSA304) (Hungary)	NEG	NEG	NEG	NEG	POS	NEG	POS	POS	NEG	NEG	NEG
HUSA304 (Hungary)	NEG	NEG	NEG	NEG	POS	NEG	POS	POS	NEG	NEG	NEG
HU106 (Hungary)	NEG	NEG	NEG	NEG	POS	NEG	POS	POS	NEG	NEG	NEG
BSAC27 (UK)	NEG	NEG	NEG	NEG	POS	NEG	POS	POS	NEG	NEG	NEG
NCTC13131, UK-EMRSA-4 (UK)	NEG	NEG	NEG	NEG	POS	NEG	POS	POS	NEG	NEG	NEG
	NEG	NEG	NEG	NEG	POS	POS	POS	POS	NEG	NEG	NEG
Algiers_HT20040080	NEG	NEG	NEG	NEG	POS	POS	POS	POS	NEG	NEG	NEG
Dublin-DSH_AR09_0.0066	NEG	NEG	NEG	NEG	POS	POS	POS	POS	NEG	NEG	NEG
Dublin-DSH_AR09_0-0065	NEG	NEG	NEG	NEG	POS	POS	NEG	POS	NEG	NEG	NEG
Dublin-DSH_Phenotype-III_84	NEG	NEG	NEG	NEG	POS	POS	POS	POS	NEG	NEG	NEG
Hong Kong_130	NEG	NEG	NEG	NEG	POS	POS	POS	POS	NEG	NEG	NEG
Kuwait_018	NEG	NEG	NEG	NEG	POS	POS	POS	POS	NEG	NEG	NEG
Perth_08-17726	NEG	NEG	NEG	NEG	POS	POS	POS	POS	NEG	NEG	NEG
Riyadh_Alfaisal/KKKSUH_86_MRS-14-279	NEG	NEG	NEG	NEG	POS	POS	POS	POS	NEG	NEG	NEG
Riyadh_Alfaisal-04_Z3861_831588	NEG	NEG	NEG	NEG	POS	POS	POS	POS	NEG	NEG	AMB
Riyadh_Alfaisal-30_S15724_1108013	NEG	NEG	NEG	NEG	POS	POS	POS	POS	NEG	NEG	NEG
Riyadh_Alfaisal-6_22A_13_B3992_397721	NEG	NEG	NEG	NEG	POS	POS	POS	POS	NEG	NEG	NEG
Riyadh_SSTI_52_3615482	NEG	NEG	NEG	NEG	POS	POS	POS	POS	NEG	NEG	NEG
Riyadh-2817276-2	NEG	NEG	NEG	NEG	POS	POS	POS	POS	NEG	NEG	AMB
Riyadh-2891670-W	NEG	NEG	NEG	NEG	POS	POS	POS	POS	NEG	NEG	NEG
Riyadh-3006920-W	NEG	NEG	NEG	NEG	POS	POS	POS	POS	NEG	NEG	AMB
Riyadh-R2567782	NEG	NEG	NEG	NEG	POS	POS	POS	POS	NEG	NEG	NEG
Russia-18_0252_Krasnoyarsk_SK2	NEG	NEG	NEG	NEG	POS	POS	POS	POS	NEG	NEG	NEG
UK-EMRSA-7	NEG	NEG	NEG	NEG	POS	POS	POS	POS	NEG	NEG	NEG
>"South American/ Mid											
Dublin-DSH_AR23_0073	NEG	NEG	NEG	NEG	POS	POS	NEG	POS	NEG	NEG	NEG
>Related to "South Ame											
MRSA-OC3 (Russia)	NEG	NEG	NEG	NEG	POS	NEG	POS	POS	NEG	NEG	NEG
Russia-12_0176_Krasnoyarsk	NEG	NEG	NEG	NEG	POS	POS	POS	POS	NEG	NEG	NEG
Russia-13_0180_Krasnoyarsk	NEG	NEG	NEG	NEG	POS	POS	POS	POS	NEG	NEG	AMB
Russia-16_0249_Krasnoyarsk	NEG	NEG	NEG	NEG	POS	POS	POS	POS	NEG	NEG	NEG
Russia-17_0250_Krasnoyarsk	NEG	NEG	NEG	NEG	POS	POS	POS	POS	NEG	NEG	NEG
>Related to "South Ame											
URU110 (Uruguay)	NEG	NEG	NEG	NEG	POS	NEG	POS	POS	NEG	NEG	NEG
>Related to "South Ame											
DS_014 (Thailand)	NEG	NEG	NEG	NEG	POS	NEG	POS	POS	NEG	NEG	NEG
>Unassigned Middle Eas											
Frankfurt_Oder_0490031797 (Libya)	NEG	NEG	NEG	NEG	POS	POS	POS	POS	NEG	NEG	NEG
Kuwait_192	NEG	NEG	NEG	NEG	POS	POS	POS	POS	NEG	NEG	AMB
Riyadh-9	NEG	NEG	NEG	NEG	POS	POS	POS	POS	NEG	NEG	AMB
Russia-24_0407_Moscow	NEG	NEG	NEG	NEG	POS	POS	POS	POS	NEG	NEG	NEG
